# A Double-Barrel Liquid Chromatography-Tandem Mass Spectrometry (LC-MS/MS) System to Quantify 96 Interactomes per Day*[Fn FN2]

**DOI:** 10.1074/mcp.O115.049460

**Published:** 2015-04-17

**Authors:** Fabian Hosp, Richard A. Scheltema, H. Christian Eberl, Nils A. Kulak, Eva C. Keilhauer, Korbinian Mayr, Matthias Mann

**Affiliations:** From the ‡Department of Proteomics and Signal Transduction, Max-Planck Institute of Biochemistry, Am Klopferspitz 18, D-82152 Martinsried, Germany

## Abstract

The field of proteomics has evolved hand-in-hand with technological advances in LC-MS/MS systems, now enabling the analysis of very deep proteomes in a reasonable time. However, most applications do not deal with full cell or tissue proteomes but rather with restricted subproteomes relevant for the research context at hand or resulting from extensive fractionation. At the same time, investigation of many conditions or perturbations puts a strain on measurement capacity. Here, we develop a high-throughput workflow capable of dealing with large numbers of low or medium complexity samples and specifically aim at the analysis of 96-well plates in a single day (15 min per sample). We combine parallel sample processing with a modified liquid chromatography platform driving two analytical columns in tandem, which are coupled to a quadrupole Orbitrap mass spectrometer (Q Exactive HF). The modified LC platform eliminates idle time between measurements, and the high sequencing speed of the Q Exactive HF reduces required measurement time. We apply the pipeline to the yeast chromatin remodeling landscape and demonstrate quantification of 96 pull-downs of chromatin complexes in about 1 day. This is achieved with only 500 μg input material, enabling yeast cultivation in a 96-well format. Our system retrieved known complex-members and the high throughput allowed probing with many bait proteins. Even alternative complex compositions were detectable in these very short gradients. Thus, sample throughput, sensitivity and LC/MS-MS duty cycle are improved severalfold compared with established workflows. The pipeline can be extended to different types of interaction studies and to other medium complexity proteomes.

Shotgun proteomics is concerned with the identification and quantification of proteins (1–3). Prior to analysis, the proteins are digested into peptides, resulting in highly complex mixtures. To deal with this complexity, the peptides are separated by liquid chromatography followed by online analysis with mass spectrometry (MS), today facilitating the characterization of almost complete cell line proteomes in a short time (3–5). In addition to the characterization of entire proteomes, there is also a great demand for analyzing low or medium complexity samples. Given the trend toward a systems biology view, relatively larges sets of samples often have to be measured. One such category of lower complexity protein mixtures occurs in the determination of physical interaction partners of a protein of interest, which requires the identification and quantification of the proteins “pulled-down” or immunoprecipitated via a bait protein. Protein interactions are essential for almost all biological processes and orchestrate a cell's behavior by regulating enzymes, forming macromolecular assemblies and functionalizing multiprotein complexes that are capable of more complex behavior than the sum of their parts. The human genome has almost 20,000 protein encoding genes, and it has been estimated that 80% of the proteins engage in complex interactions and that 130,000 to 650,000 protein interactions can take place in a human cell (6, 7). These numbers demonstrate a clear need for systematic and high-throughput mapping of protein–protein interactions (PPIs) to understand these complexes.

The introduction of generic methods to detect PPIs, such as the yeast two-hybrid screen (Y2H) (8) or affinity purification combined with mass spectrometry (AP-MS)^1^ (9), have revolutionized the protein interactomics field. AP-MS in particular has emerged as an important tool to catalogue interactions with the aim of better understanding basic biochemical mechanisms in many different organisms (10–17). It can be performed under near-physiological conditions and is capable of identifying functional protein complexes (18). In addition, the combination of affinity purification with quantitative mass spectrometry has greatly improved the discrimination of true interactors from unspecific background binders, a long-standing challenge in the AP-MS field (19–21). Nowadays, quantitative AP-MS is employed to address many different biological questions, such as detection of dynamic changes in PPIs upon perturbation (22–25) or the impact of posttranslational signaling on PPIs (26, 27). Recent developments even make it possible to provide abundances and stoichiometry information of the bait and prey proteins under study, combined with quantitative data from very deep cellular proteomes. Furthermore, sample preparation in AP-MS can now be performed in high-throughput formats capable of producing hundreds of samples per day. With such throughput in sample generation, the LC-MS/MS part of the AP-MS pipeline has become a major bottleneck for large studies, limiting throughput to a small fraction of the available samples. In principle, this limitation could be circumvented by multiplexing analysis via isotope-labeling strategies (28, 29) or by drastically reducing the measurement time per sample (30–32). The former strategy requires exquisite control of the processing steps and has not been widely implemented yet. The latter strategy depends on mass spectrometers with sufficiently high sequencing speed to deal with the pull-down in a very short time. Since its introduction about 10 years ago (33), the Orbitrap mass spectrometer has featured ever-faster sequencing capabilities, with the Q Exactive HF now reaching a peptide sequencing speed of up to 17 Hz (34). This should now make it feasible to substantially lower the amount of time spent per measurement.

Although very short LC-MS/MS runs can in principle be used for high-throughput analyses, they usually lead to a drop in LC-MS duty cycle. This is because each sample needs initial washing, loading, and equilibration steps, independent of gradient time, which takes a substantial percentage for most LC setups - typically at least 15–20 min. To achieve a more efficient LC-MS duty cycle, while maintaining high sensitivity, a second analytical column can be introduced. This enables the parallelization of several steps related to sample loading and to the LC operating steps, including valve switching. Such dual analytical column or “double-barrel: setups have been described for various applications and platforms (30, 35–39).

Starting from the reported performance and throughput of workflows that are standard today (16, 21, 40–42), we asked if it would be possible to obtain a severalfold increase in both sample throughput and sensitivity, as well as a considerable reduction in overall wet lab costs and working time. Specifically, our goal was to quantify 96 medium complexity samples in a single day. Such a number of samples can be processed with a 96-well plate, which currently is the format of choice for highly parallelized sample preparation workflows, often with a high degree of automation. We investigated which advances were needed in sample preparation, liquid chromatography, and mass spectrometry. Based on our findings, we developed a parallelized platform for high-throughput sample preparation and LC-MS/MS analysis, which we applied to pull-down samples from the yeast chromatin remodeling landscape. The extent of retrieval of known complex members served as a quality control of the developed pipeline.

## EXPERIMENTAL PROCEDURES

### 

#### 

##### Preparation of Yeast Lysates

GFP-tagged yeast strains from the *Saccharomyces cerevisiae* GFP Clone Collection (43), the parental strain BY4741 and the control strain pHis3-GFP (21) were cultured in YPD liquid medium in 96-deep well plates (Sarstedt, Nümbrecht, Germany) at standard conditions. We used 32 distinct yeast strains in biological triplicates, resulting in 96 experimental samples. Yeast cells were grown until they reached an Optical Density_600 nm_ of around 1, followed by harvesting culture volumes equaling 2 ODs per well. Yeast cell pellets were dissolved in 300 μl lysis buffer (150 mm NaCl, 50 mm Tris-HCl (pH 8.0), 1 mm MgCl_2_, 5% glycerol, 1% IGEPAL CA-630 (Sigma-Aldrich, Schnelldorf, Germany), complete protease inhibitors (Roche, Mannheim, Germany), 1% benzonase (Merck, Darmstadt, Germany)), transferred into FastPrep tubes (MP Biomedicals, Eschwege, Germany) containing 1 mm silica spheres (lysing matrix C, MP Biomedicals), and lysed in a FastPrep24 instrument (MP Biomedicals) for 6 × 1 min at maximum speed. Lysates were cleared by centrifugation at 16,100 × *g* for 10 min at 4 °C.

##### Affinity Purification

Each well of a GFP-multiTrap plate (ChromoTek, Martinsried, Germany) was washed three times with 200 μl buffer 1 (150 mm NaCl, 50 mm Tris-HCl, pH 8.0) and then incubated with the cleared yeast cell lysate (500 μg total protein extract) with gentle shaking at 100 rpm for 60 min at 4 °C. Next, each well was washed twice with 200 μl buffer 2 (150 mm NaCl, 50 mm Tris-HCl (pH 8.0), 0.25% IGEPAL CA-630) and four times with 200 μl buffer 1 before incubation with 25 μl elution buffer (2 m urea, 20 mm Tris-HCl (pH 8.0), 1 mm DTT, 100 ng sequence-grade modified trypsin (Promega, Madison, WI, USA) at room temperature for 90 min. Subsequently, the resulting peptides were alkylated with 25 μl alkylation buffer (2 m urea, 20 mm Tris HCl (pH 8.0), 5 mm iodoacetamide) and finally washed once with 50 μl urea buffer (2 m urea, 20 mm Tris HCl, pH 8.0) for 10 min, respectively. The supernatants from the elution, alkylation and washing step were collected after each step and combined in a clean 96-well plate. This plate was incubated overnight at room temperature to ensure a complete digest. The next morning, the digest was stopped by addition of 10 μl 10% TFA per well. The acidified peptides were purified on StageTips (44) containing two layers of Poly(styrenedivinylbenzene)-Reversed-Phase Sulfonate (Empore 2241, 3 m, Neuss, Germany) material to desalt and purify the peptides. Samples were eluted from the StageTips with 60 μl elution buffer (80% acetonitrile, 1% ammonium hydroxide) and evaporated in a SpeedVac concentrator for 30 min. The remaining peptide solution volume was adjusted to 4 μl with buffer A* (2% ACN, 0.1% formic acid).

##### LC-MS/MS Analysis

Online chromatography was performed with a modified Thermo EASY-nLC 1000 UHPLC system (Thermo Fisher Scientific, Bremen, Germany) coupled online to the Q Exactive HF instrument with a nano-electrospray ion source (Thermo Fisher Scientific). Two analytical columns (15 cm long, 75 μm inner diameter) were packed in-house with ReproSil-Pur C_18_ AQ 1.9 μm reversed phase resin (Dr. Maisch GmbH, Ammerbuch, Germany) in buffer A (0.5% formic acid) and matched with regard to back-pressure to ensure intercolumn reproducibility. During online analysis, the analytical columns were placed in a modified column heater (Sonation GmbH, Biberach, Germany) regulated to a temperature of 55 °C. Modifications to both systems are described in RESULTS. Peptides were loaded onto the analytical columns with buffer A at a back pressure of 650 bar (generally resulting in a flow rate of 500 nL/min) and separated with two distinct linear gradients of 8–30% buffer B (80% ACN and 0.5% formic acid) at a flow rate of 450 nL/min controlled by IntelliFlow technology over 10 min and 22 min, respectively (generally at a back pressure of around 500 bar). Online quality control was performed with SprayQc (45), which was extended with an additional plugin to support a high-voltage switch controlling the spray voltage for the analytical columns (RESULTS). MS data were acquired with a Q Exactive Plus (27 min gradients) and a Q Exactive HF (14 min gradients) instrument, as the latter has been found to be up to twice as fast (34) and thus capable of dealing with the fast chromatography of the 14 min gradient. The instruments were programmed with a data-dependent top 5 and top 10 method, respectively, dynamically choosing the most abundant not yet sequenced precursor ions from the survey scans (300–1,650 Th). Instruments were controlled using Tune 2.5 and Xcalibur 3.0.63. At a maximum ion inject time of 45 ms for both instruments, the cycle time was ∼800 ms, sufficient for generating a median of 16 data points (14 min) or 25 data points (27 min) over the observed elution peaks (RESULTS). Further settings were chosen according to their previously determined optimal values (34). Sequencing was done with higher-energy collisional dissociation fragmentation with a target value of 1e5 ions determined with predictive automatic gain control, for which the isolation of precursors was performed with a window of 1.4 Th. Survey scans were acquired at a resolution of 70,000 and 60,000, respectively, at *m/z* 200 and the resolution for HCD spectra was set to 17,500 and 15,000, respectively, at *m/z* 200. Normalized collision energy was set to 27 and the “underfill ratio,” specifying the minimum percentage of the target ion value likely to be reached at maximum fill time was defined as 10% (27 min) and 40% (14 min). The elevated sequencing threshold ensured that, with the reduced complexity of samples, the fragmentation scans are of higher quality. Furthermore, the S-lens radio frequency level was set to 60, which gave optimal transmission of the *m/z* region occupied by the peptides from our digest (34). We excluded precursor ions with unassigned, single, or five and higher charge states from fragmentation selection.

##### Data Analysis

All data were analyzed with the MaxQuant proteomics data analysis workflow version 1.4.3.14 (46). The false discovery rate (FDR) cut off was set to 1% for protein and peptide spectrum matches. Peptides were required to have a minimum length of seven amino acids and a maximum mass of 4,600 Da. MaxQuant was used to score fragmentation scans for identification based on a search with an initial allowed mass deviation of the precursor ion of a maximum of 4.5 ppm after time-dependent mass calibration. The allowed fragment mass deviation was 20 ppm. Fragmentation spectra were identified using the UniprotKB *S. cerevisiae* database (based on 2014–07 release; 6,643 entries) combined with 262 common contaminants by the integrated Andromeda search engine (47). Enzyme specificity was set as C-terminal to arginine and lysine, also allowing cleavage before proline, and a maximum of two missed cleavages. Carbamidomethylation of cysteine was set as fixed modification and N-terminal protein acetylation and methionine oxidation as variable modifications. Both “match between runs,” with a maximum time difference of 30 s, and label-free quantification (LFQ) with standard settings, were enabled (48). Additional metadata stored in the RAW files (*e.g.* ion inject time, noise level, etc.) were extracted using MSFileReader (Thermo Scientific) with in-house-developed tools.

Further data analysis with the goal of assigning the interactors was performed with the R scripting and statistical environment (49) using ggplot (50) for data visualization. Briefly, LFQ intensity values were base10 logarithmized, resulting in a normal distribution. Missing values were imputed by randomly selecting from a normal distribution centered on the lower edge of the intensity values (for this normal distribution the shift was set to 1.8 standard deviations from the mean and the width to 0.3 standard deviations; see histograms describing placement in Figs. S8 and S9). Proteins were excluded in subsequent steps for baits with less than two valid values in the triplicate for the bait (mostly presented as significantly depleted proteins due to the imputed character of the intensity values). The fold enrichment was calculated as the mean ratio between the bait measurements and the proteome measurements of the parental strain (conforming to the mean used in the consequent *t* test). For the fold enrichment, the standard error of the mean was additionally determined. Permutation-based FDR-controlled *t* test *p* values were calculated for each protein between the bait triplicate and the parental strain triplicate (employing 250 permutations). The *p* value was adjusted using a scaling factor s0 with a value of 1 prior to FDR control, which magnifies the importance of the difference of the mean (51). Furthermore, the correlation of each protein's LFQ intensity profile (consisting of all the measured intensity values for that protein) to the LFQ intensity profile of the bait was calculated (21), and the resulting correlation *p* values were adjusted to 1% FDR using the Benjamini and Hochberg procedure. Interactor classes were assigned based on the following rules: (A) only <1% FDR *t* test significance, (A+) both <1% FDR *t* test significance, and <1% FDR correlation significance, (B+) both <5% FDR *t* test significance and <1% FDR correlation significance and (B) only <5% FDR *t* test significance. Known interactors from the *Saccharomyces* Genome Database (www.yeastgenome.org) mainly fell in classes A+, A, and B+. Therefore, we conducted follow-up analyses solely on these classes. For each significant outlier, we also introduced a single significance value, based on the s0 scaling introduced in the *t* test, which combines the enrichment value and the *t* test statistic. This is calculated as the distance in log-space from the origin. The higher this value, the better the data quality and experimental success of that particular interactor. Stoichiometry information was determined in two ways. The first, termed interaction stoichiometry, is the ratio between the calculated intensity-based absolute quantification values (determining the copy numbers from the acquired mass spectrometry data) of the interactors to the bait (52). The second, termed abundance stoichiometry, is the ratio between the normal cellular copy numbers of the interactors to the bait.

## RESULTS

### 

#### 

##### Reducing the LC-MS/MS Analysis Time

**Fig. 1.**
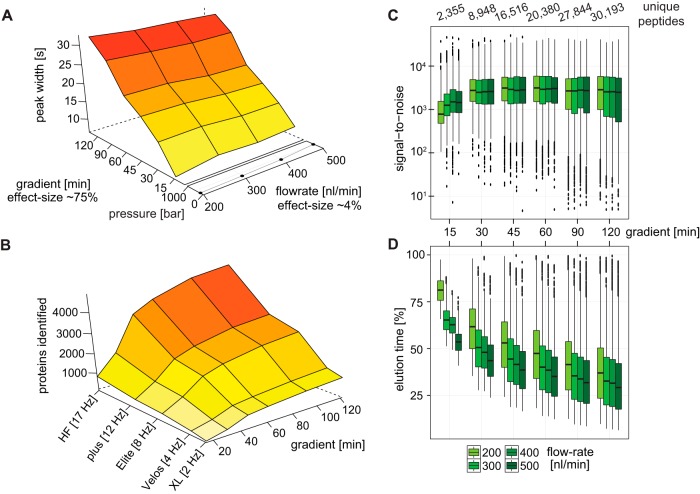
**Chromatography optimization for very short gradients**. (*A*) Peak-width as a function of gradient length and flow rate. Effect size is the calculation of the reduction compared with the largest change in peak-width. (*B*) Extrapolation of protein identifications as a function of gradient length and scan speed of various MS platforms (Q Exactive HF and plus, Orbitrap Elite, and Velos, LTQ Orbitrap XL, respectively). (*C*) Effect of flow rate on the signal-to-noise for a set of 750 unique isotope patterns identified in all measurements and spread out over the entire gradient. (*D*) Elution time shift induced by higher flow rates, normalized to the gradient length.

First, we aimed to establish optimal conditions for reducing the LC gradient length. Both the flow rate and gradient starting percentage require adaptations to ensure that the signal of each peptide does not degrade and to maximize the spread of peptides over the gradient. To achieve this, we tested the effect of flow rate (ranging from 200 to 500 nl/min) and gradient length (from 15 to 120 min) on the chromatographic peak-width with a standard HeLa digest on the Q Exactive HF (34). By far, the largest effect on peak width was shortening the gradient length as this provided a reduction of ∼75% on the width, while the flow rate reduced it only by ∼4% (Fig. 1*A*). With regard to overall proteome depth, we were able to identify about 740 proteins with a standard HeLa digest using the shortest gradient length of 15 min with the Q Exactive HF (Fig. 1*B*). Hence, the complexity of protein samples should not exceed such a number when high sample throughput is envisioned. We also determined protein identifications for lower sequencing speed (Fig. 1*B*). Notably, even platforms with lower sequencing speed like the Orbitrap XL identified about 1,000 proteins with a 120 min gradient, suggesting that already this machine generation had the potential to identify all proteins of a lower complexity sample given sufficiently long gradients.

Higher flow rates could have a detrimental effect on the signal-to-noise due to the higher dilution of peptides in the buffer, which we investigated by extracting the signal-to-noise values for a set of 750 isotope patterns identified in all the runs and spread out over the full retention time range. For the longest gradient length of 120 min, we observe a slight decrease in signal-to-noise for the higher flow rates, whereas unexpectedly higher flow rates partially improve the signal-to-noise for the shortest gradient. For the intermediate gradient lengths, the flow rate does not appreciably affect the signal-to-noise ratio. Between the two shortest gradients of 30 and 15 min, we observe a drop in signal-to-noise, which we attribute to imprecision of the buffer delivery by the LC (Fig. 1*C*). Given that it takes time for the buffer mixture to arrive from the mixing T connection to the tip of the analytical column, and therefore for the peptides to elute, the shorter gradients suffer in terms of gradient occupancy (percentage of the gradient occupied by peptides) when using lower flow rates. This is mostly improved by forcing the peptides to elute earlier with higher flow rates. For the shortest gradient lengths, we were able to move the start of peptide elution from 60% in the gradient (at 9 min) to 40% in the gradient (at 6 min), improving the spread of the peptides over the complete gradient and providing better chromatographic resolution. For the 30 min gradient, the first elution was moved from 10 min (35% of the gradient time) to 7 min (25%) (Fig. 1*D*).

Based on these findings, we determined the optimal gradient time to be 27 min with a flow rate of 450 nl/min, which kept the backpressure of the LC pumps at an acceptable level of around 500 bar. This, however, still results in 2 days of measurements for 96 samples. The 12 min gradient at the same flow rate necessary for exactly 24 h of measurement for the same number of samples is expected to have reduced chromatographic performance compared with the 27 min gradient. This period is also too short to transfer the peptides onto the analytical column in parallel. We therefore increased the gradient time to 14 min and activated the loading pump during the intersample preparation time, which reliably loaded all the peptides onto the analytical column. Additionally, we increased the starting acetonitrile percentage of the gradient from 2% to 8% (EXPERIMENTAL PROCEDURES) to start the peptide elution at an earlier point of the gradient. Collectively, this resulted in a time frame for peptide elution of 8 min and 18 min, representing 60 and 75%, respectively, of the total measurement time for the 14 and 27 min gradients. At these conditions, the median peak-width (base-to-base) was 6 s (14 min) and 11 s (27 min), respectively.

##### Double-Barrel Chromatography on the EASY-nLC

**Fig. 2.**
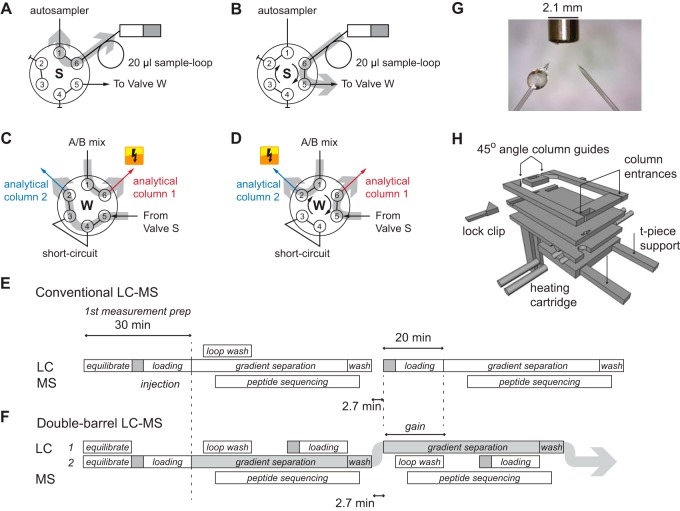
**Parallel UHPLC operation with two analytical columns.** (*A*) In this position of valve S, the sample pump can fill the sample loop. (*B*) By switching valve S, the contents of the sample loop can be loaded onto one of the analytical columns. (*C*) In this position of valve W, the analytical column 1 can be eluted with the mobile phase, while analytical column 2 is loaded. (*D*) By switching the position of valve W, this behavior is inverted. (*E*) In the conventional setup, the mass spectrometer is not sequencing while the HPLC is loading a new sample. The light gray arrow indicates where the mobile phase is active. (*F*) With the double-barrel setup, this idle-time is circumvented, enabling almost continuous operation. (*G*) Positioning of the analytical columns in reference to the inlet of the mass spectrometer. (*H*) Redesign of the column oven for two analytical columns.

Next, we set out to develop a double-barrel chromatography system in order to reduce the idling time of the mass spectrometer during loading of the peptides to the LC column. Unfortunately, no such setup has been described for the Thermo EASY-nLC 1000 UHPLC systems (Thermo Fisher Scientific) that we employ and that are widely used with the Orbitrap-family of mass spectrometers. To address this, we modified the liquid pathway of the EASY-nLC 1000 UHPLC system (Figs. 2*A*-2*D*). In brief, we placed the sample loop directly between the pump S and valve S, allowing the system to utilize pump S as both the sample pickup as well as the sample-loading pump (in the original setup, pump A is used as sample-loading pump). The valve S is connected to valve W (in the original setup this valve is connected to a waste line used for rapid evacuation of the buffers from the lines), which connects to the buffer A and B mixing-T connection and the two analytical columns through standard sample lines. This setup allows loading of one sample onto one of the analytical columns while the other is eluted.

To make use of this new liquid pathway and to drive two analytical columns in parallel, we also modified the “business logic” controlling the UHPLC system. The normally sequential steps in the analysis process (Fig. 2*E*) were altered to work in parallel with each other (Fig. 2*F*). As soon as the preparation for the currently active analytical column has finished, the initiation phase and the valve W has switched to elute the loaded peptides, the inactive analytical column is prepared in parallel for the next sample. This is done in three consecutive steps: First, the sample loop is washed, then the new sample is loaded into the sample loop, and finally the sample is loaded from the sample loop onto the analytical column. With the above described arrangement of the pumps and valves, these operations can be performed independently for each of the two analytical columns. The intermeasurement time for the double-barrel system was clocked at a maximum of 160 s (Figs. 2*E* and 2*F*), which cannot be further reduced on this particular system due to the necessity of refilling the syringe-based pumps and bringing them back up to pressure (Supplemental Fig. S1).

Finally, we modified our standard analytical column heater (33) to accommodate the two analytical columns. The two columns are now pointing sideways toward the mass spectrometer inlet at a fixed angle of 45 degrees at a distance of roughly 2 mm from each other at the tip ends (equaling the width of the heated capillary mounted on Orbitrap platforms; Fig. 2*G*). As we utilize a fixed setup for the analytical columns, we cannot supply the spray voltage in parallel (Fig. 2*H*). To shift the voltage between the analytical columns, we additionally developed a high-voltage switch capable of supplying electricity to a single analytical column, controllable through a universal serial bus connection (Supplemental Fig. S2). A plugin module that we developed for the SprayQc environment (45) monitors the current position of the valve W and switches the spray voltage to the eluting analytical column according to a user-definable setting.

##### A Parallel Workflow for Analyzing 96 Pull-Down Samples within a Single Day

**Fig. 3.**
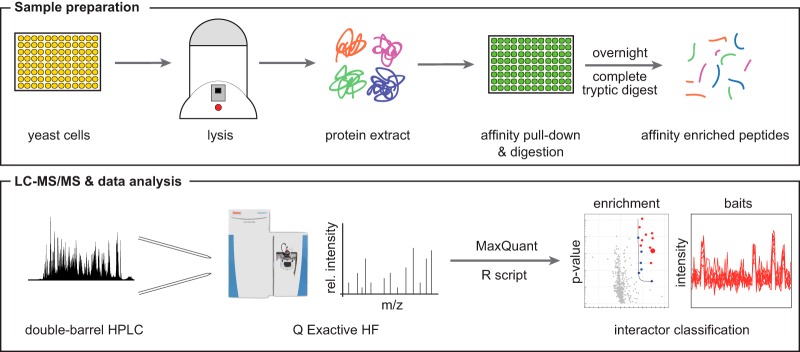
**Workflow of the high-throughput LC-MS/MS protein interaction analysis pipeline.** Both culturing of yeast cells and affinity purification are performed in 96-well plate format, thus parallelizing sample preparation and minimizing handling errors. LC-MS/MS analysis of 96 pull-down samples in 1 day is achieved through a double-barrel chromatography setup and the increased sequencing speed of the Q Exactive HF mass spectrometer.

A high-throughput platform should be able to prepare samples in a parallelized format and subsequently measure all of them within a very short time period. Here, we developed an analysis pipeline for pull-down samples that is capable of achieving this goal on pull-down samples (Fig. 3). To facilitate a streamlined workflow necessary for achieving high-throughput processing of pull-down samples, we used GFP-tagged yeast strains originating from the yeast GFP clone collection (43). Further improvements were gained by combining both the cultivation of the yeast and the pull-downs in a 96-well format. Each well yields ∼50 million yeast cells, equal to 500 μg of protein lysate, which turned out to be sufficient for the pull-down experiments.

##### Mass Spectrometry Platform Performance on Pull-Down Samples

Using the transcriptional adapter protein ADA2 as a bait, we compared the performance of the Q Exactive HF to that of the LTQ-Orbitrap XL, an instrument introduced about 9 years ago with a sequencing speed of 2 Hz that is frequently used for pull-down analyses. Notably, both instruments were able to identify all known members of the reconstituted ADA2 complex within the commonly used measurement time of 2 h (Supplemental Fig. S3*A*). This suggests that protein interaction data acquired with older Orbitrap generations over the last 10 years would generally gain little by remeasurement as long as extended LC-MS/MS gradients have been used. However, we note that the protein sequence coverage and, consequently, enrichment of the preys (calculated by dividing the MaxLFQ intensity of the interactors by the median of all MaxLFQ intensities) was somewhat improved with the Q Exactive HF, making the setup slightly more sensitive in detecting interactors (Supplemental Fig. S3*B*). Clearly, these gradient times are not making effective use of the superior sequencing speed of the Q Exactive HF. By lowering the measurement time to as low as 15 min, the identification performance of the older platform started to suffer while that of the Q Exactive HF still allowed capturing all the expected interactors (Supplemental Fig. S3*A*). The major difference between the systems was in the sequence coverage per protein, which for the Q Exactive HF remains constant up to 30 min and slightly degrades at 15 min, while it degrades dramatically for the Orbitrap XL (Supplemental Fig. S3*C*). The decreased sequence coverage negatively impacts the ability to accurately quantify proteins as label-free quantification improves with the number of peptides associated to a given protein (48). This is reflected in the measured enrichment ratios, which for the Orbitrap XL made the bait interactors nearly indistinguishable from the background, while for the Q Exactive HF it remained superior even at 30 min when comparing to 2 h (Supplemental Fig. S3*B*). Overall, as expected, the Q Exactive HF outperformed the Orbitrap XL for all measurement times tested in terms of prey enrichment, sequence coverage, and isotopic features (Supplemental Fig. S3*B*-S3*D*). While we observed a decrease in obtained sequence information in the 15 min Q Exactive HF methods, these very short runs still yielded sufficiently high sequence coverage to identify the members of the complex under investigation. In conclusion, these results show that mass spectrometers with relatively low sequencing speed can perform equivalently at long gradients for protein interaction studies, whereas very high sequencing speeds are required for high-throughput identification.

##### Reproducibility of the Data Acquisition System

**Fig. 4.**
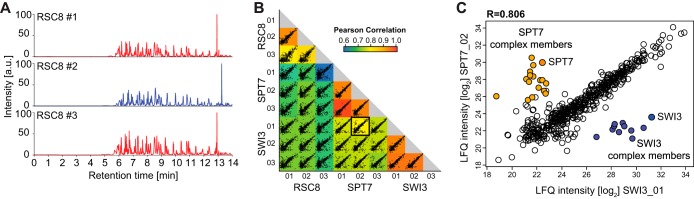
**Double-barrel chromatography with 14 min gradients on three pull-downs.** (*A*) Base-peak chromatogram of a biological triplicate RSC8 pull-down run on the double-barrel LC-MS/MS setup. Chromatography in all cases is very reproducible. (*B*) Comparison of RSC8, SPT7 and SWI3 pull-downs; all measured in triplicates. The matrix of 36 correlation plots reveals high correlations between MaxLFQ intensities within triplicates. (*C*) Zoom into SPT7_02 *versus* the SWI3_01 correlation plot. While most proteins were detected with very similar MaxLFQ intensities, the two outlier populations marked in orange (SPT7) and blue (SWI3) represent the different complex members of the distinct protein complexes.

To investigate the reproducibility of protein quantification between different measurements, we acquired PPI data for the yeast chromatin remodelers RSC8, SPT7, and SWI3 with our workflow. Visual inspection of the chromatograms for the RSC8 pull-down, measured in triplicates, already shows a high degree of technical reproducibility for the double barrel system with back pressure matched analytical columns (Fig. 4*A*). In modern PPI experiments, the number of background binders can be in the thousands as opposed to only a few true interactors. We take advantage of these unspecific binders to estimate reproducibility by calculating the correlation between each pair of the measurements where only the generally small number of true interactors degrade the correlation (21). Most of the detected unspecific binders were indeed reproducibly quantified in all three samples. There was one exception with a slightly reduced Pearson correlation coefficient for the RSC8 pull-down (Fig. 4*B*), for which we concluded based on the large number of imputed values that the enrichment was not completely successful. A small outlier population observed for each bait protein indeed represented the expected interaction partners (Fig. 4*C* and Supplemental Fig. S4). Collectively, these results indicate that our double-barrel setup can be operated with very low MS idling time between two independent measurements and achieves high reproducibility at the same time.

##### PPI Data Quality from Very Short Gradients

To identify preys of a given bait protein, we classified all interactors into four distinct classes essentially as described (21) and improved on that concept by making it completely data driven (EXPERIMENTAL PROCEDURES). Distinction of specific from unspecific binders was achieved by a permutation-based false-discovery rate approach operating on a *t* test and enrichment with two distinct stringencies (EXPERIMENTAL PROCEDURES; Supplemental Fig. S5*A*). Proteins passing the stringent cutoff represent highly enriched interactors, whereas proteins only passing the less stringent cutoff are characterized as mildly enriched interactors. All other proteins were considered to be unspecific binders. In addition, we used Benjamini–Hochberg-corrected intensity profile correlation of potential interactors compared with the bait protein to minimize false-positive identifications of mildly enriched interactors (EXPERIMENTAL PROCEDURES; Supplemental Figs. S5*E* and S5*F*) (21). With these criteria, interactors were grouped into confidence classes A+, A, B+, and B (Supplemental Figs. S4*C* and S4*G*). Absolute quantification data from whole yeast proteome experiments (53) allowed us to also estimate interaction and abundance stoichiometries for every protein complex under investigation (Supplemental Fig. S5*D*).

To assess the quality control of both the LC-MS/MS measurements and the subsequent interactor classification given our large throughput, we employed three distinct layers. The first layer consists of the real-time validation provided by SprayQc (45). Besides the logic for the voltage switch, this software implements automatic warnings via E-mail to the operator for a large number of components involved in the measurement and reports meta-data for these components (EXPERIMENTAL PROCEDURES). The second layer consists of verification of the sample preparation and LC-MS/MS measurement success by the number of identified proteins per measurement. Given the preponderance of background proteins, this value should be roughly equal for all pull-downs. The histograms displaying the imputed values provide a simple visual guide in the form of the peaks for the imputed proteins (EXPERIMENTAL PROCEDURES). The third layer is the data-driven determination of what constitutes a successful pull-down experiment. For this, we used the information from the volcano plots, specifically the significance value as described (EXPERIMENTAL PROCEDURES). For all the pull-downs, we combine this value for all the baits to determine a valid range for the baits. Anything falling outside this range is flagged as potentially unreliable.

##### A Snapshot of the S. cerevisiae Chromatin Remodeling Landscape

**Fig. 5.**
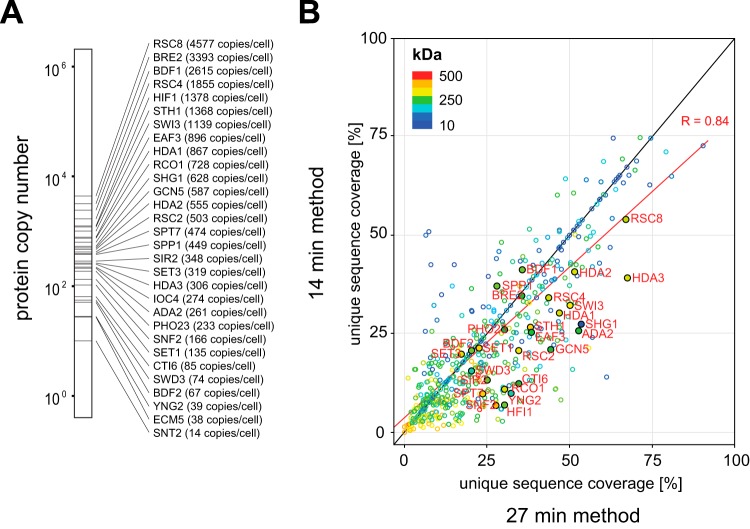
**Bait and prey characteristics comparing 14 *versus* 27 min gradient methods.** (*A*) The 30 bait proteins selected for the pull-down experiments span several orders of protein expression abundance in *S. cerevisiae*, including several very low abundant proteins (<100 copies per cell). (*B*) Unique sequence coverage for all identified proteins decreases for the 14 min method compared with the 27 min method. Bait proteins are labeled in red.

The data obtained from our very short LC-MS/MS measurements operated with double barrel chromatography demonstrated that AP-MS screens of sufficient quality can be performed in a high-throughput format (Fig. 4). To investigate our workflow on a set of protein complexes involved in a particular biological pathway, we selected 30 distinct bait proteins that are part of the yeast chromatin remodeling landscape. In addition, we also used a GFP-expressing control and the haploid parental strain (EXPERIMENTAL PROCEDURES). Our bait selection spans three orders of expression abundance over the whole yeast proteome (Fig. 5*A*) and includes several baits with very low abundance (<100 copies per cell). We found that the protein input amount of 500 μg, which is much lower than that traditionally used, was sufficient to identify the bait proteins and to retrieve known interactors, even for lowest expressed bait proteins (Supplementary Material_14min and Supplementary Material_27min). Additionally, where possible, we selected multiple baits per protein complex in an attempt to characterize the complex as thoroughly as possible. This collection covers 21 distinct protein complexes subdivided into four enzyme classes: histone acetyltranferase, chromatin remodeling, histone methyltransferase, and histone deacetylase complexes. For the 32 distinct yeast strains, we performed pull-down experiments in biological triplicates, resulting in 96 samples. Each of these pull-down samples was measured with both the 14 and the 27 min LC-MS/MS methods, respectively. Together, the interactomes of 96 pull-down samples were measured in either 47.5 h (27 min method) or 26.7 h (14 min method) of start-to-end complete measurement time, including all overhead. As expected, we found that the sequence coverage of bait proteins and specific interactors was reduced for almost every protein in the 14 compared with the 27 min method (Fig. 5*B*). Nevertheless, the sequence information acquired in the 14 min runs was still sufficient to identify enriched baits and their corresponding preys. We did not experience problems with regard to the bioinformatic enrichment value based on the LFQ intensities, as had been the case for the short gradients on the older platforms (see Supplemental Figs. S6 and S7, Supplementary Material_14min and Supplementary Material_27min).

**Fig. 6.**
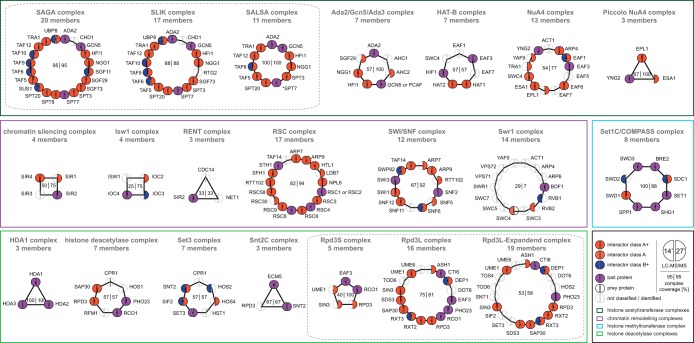
**Topology network of all interactors.** Global overview of the measured complexes and the success-rate achieved with the 14 *versus* 27 min gradients. Each protein is depicted as a circle, where the left half corresponds to the 14 min and the right half to the 27 min run results. Color coding refers to the different interactor classes and selected bait proteins. Numbers in the center of each complex represent the percentile coverage of the total complex composition as identified by 14 min (left) or 27 min (right) runs. Colored rectangles group the complexes into their distinct biological functionalities.

To provide an overview of our identified PPIs, we created a topology network of all interactors assigned to one of the defined prey classifications. While the overall interactor class ranking was slightly reduced, we found only small variations in the final complex coverage even though the LC-MS/MS gradient was nearly halved when comparing the 14 to the 27 min method (Fig. 6). Out of 21 protein complexes analyzed, both run times performed equally well in nine cases, whereas the 27 min outperforms the 14 min in ten cases. Conversely, the 14 min runs were better in two cases. The 27 min method allowed a high retrieval of known interactors even for several very low abundant baits with less than 100 copies per cells. While the 14 min method identified less preys of baits with very low abundance, its superior speed allowed throughput of the same sample set in almost half the time.

Remarkably, we could even validate the presence of two different RSC nucleosome-remodeling complexes. The RSC complex is present in two distinct isoforms with distinct roles in the DNA damage response, as defined by the presence of either RSC1 or RSC2 (54, 55). While performing pull-downs on either RSC4 or RSC8, we identified both RSC1 and RSC2 as interactors, demonstrating that RSC4 and RSC8 are part of both RSC complex isoforms (Supplementary Material_27min). In contrast, pulling down RSC2 only resulted in RSC2 but not RSC1 as complex members. These results demonstrate that our workflow is capable of identifying distinct complex compositions in a rapid manner.

##### Discussion and Outlook

In this study, we have described advances for analyzing up to 96 proteomes with lower complexity in about 1 day of LC-MS/MS data acquisition, including all overhead. Our interaction workflow employs parallelized sample generation in a 96-well format together with a modified LC setup and mass spectrometers with very high sequencing speed. With this combination, we demonstrated a severalfold increase in sample processing throughput and sensitivity, as well as in the LC-MS duty cycle.

Including the preceding yeast cultivation and sample preparation steps, processing of 96 pull-down experiments can be achieved within 48 h. However, several 96 samples could be handled in parallel, allowing nesting upstream sample preparation and downstream LC-MS/MS analysis. This in principle would allow a sustained workflow with a capacity of 96 distinct samples per day. The data presented here were acquired following manual sample preparation. However, the majority of sample preparation steps in our workflow only require liquid handling and are thus easily automated using robotic sample preparation systems.

LC-MS/MS data acquisition within 14 min per sample pushes both the LC and MS systems to their current limits. Consequently, the 14 min runs yielded reduced chromatographic quality compared with the 27 min runs. Although this was still sufficient to yield almost the same complex coverage, the 14 min runs did result in lower sequence coverage for both bait and prey proteins (Fig 5*B*). This adversely affects analyses and more importantly reduces the enrichment values, making it harder to pinpoint interactors (Fig 2B). Potential optimization could be obtained in an improved experimental design. In this study, we focused on the reproducibility of the complete workflow and chose to perform all steps and measurements in a consecutive series of steps. However, randomizing the measurements, while ensuring that all the replicates of one particular pull-down are always run on the same column, should further improve higher data quality and statistical significance for the interaction determination.

The implementation of double-barrel systems opens up interesting possibilities. On the technological side, it enables automatic detection of a break down in one of the columns due to clogging and reacting to this by using the other column, instead of stopping further analysis. To detect this situation, the software tracks the amount of pressure during the gradient and the flow rate achieved during loading. When the pressure during the gradient or the flow rate during loading exceed critical parameters the system automatically stops operations on this particular column. Further operation is then continued as a single-barrel system. This simple mechanism has the potential to drastically extend the effective up-time and enable almost 24/7 operation of the mass spectrometer. A second technological possibility is the automatic determination of the optimal time for sample loading. The flow rate achieved during loading of the previous sample on the particular analytical column can be used to estimate the required loading time for the current sample. The software then automatically determines the delay required before loading the sample, for instance with a 10 min overhead to ensure that the sample is completely loaded irrespective of fluctuations in the flow rate. This is particularly important for double-barrel-based LC setups as during long gradients it is conceivable that it would be detrimental for the sample to be loaded at the start of the gradient of the other analytical column and then remain at the elevated temperature conditions of the analytical column heater. Third, the described setup could be further extended by using two completely independent UHPLC systems. Even though such a concept is not straightforward to implement on our current system due to software-related issues, the extra redundancy of hardware components would enable troubleshooting of an erroneous UHPLC while the other system maintains measuring. In this way, genuine 24/7 operation of LC-MS/MS data acquisition would be feasible.

Recently, we have reported a high-performance affinity enrichment-mass spectrometry method (21) that uses accurate quantitation of background and unspecific binders for retrieval of true protein complexes. We propose to combine both strategies to allow both the confident retrieval of binding partners and a high throughput. This should be a powerful strategy, especially when a high sequence coverage is not essential (56). Moreover, our results also show that AP-MS can be performed with protein input amounts as low as 500 μg per pull-down and probably much lower in the future, which is considerably less than previously described (21, 42). This increase in sensitivity strongly promotes parallelization and thus throughput efforts. Currently, our pipeline permits a maximum throughput of 96 samples in about 1 day. Employment of other quantification strategies with higher multiplexing, such as TMT labeling for instance, would drastically increase throughput even further.

While we have demonstrated the workflow for protein–protein interactions, our pipeline is generic and can be extended to any kind of protein-based interaction studies in which there is an effective immobilization of the bait material as affinity matrix. We envision other baits such as peptides, DNA, RNA, lipids, or small molecules will greatly facilitate large-scale screening and elucidate drug targets, changes in protein complex formation upon perturbation, and the intertwined relationship between proteins and DNA or RNA.

Finally, the advances described here for the LC-MS/MS part of the workflow can also be extended to the analysis of whole proteomes. For example, biochemical fractionation of whole cell lysates is a routine procedure in mass-spectrometry-based proteomics as it enables much deeper characterization (57, 58). The concomitant increase in LC-MS/MS measurement time caused by the larger number of fractions could be mitigated by using our optimized LC-MS/MS setup. Here, we demonstrated that our very short gradients of 15 min are still able to identify about 700 proteins in a standard HeLa digest (Fig. 1*B*). If such a complexity is not exceeded, high-throughput analysis can be performed even for fractionated whole proteomes of cell lines, small model organisms, or clinical samples. Finally, given the exponential progress in proteomics related technology, it should only be a matter of time until entire proteomes can be measured in minutes.

## Supplementary Material

Supplemental Data

## Footnotes

^1^ The abbreviations used are:

AP-MS: affinity purification mass spectrometry
FDR: false discovery rate
GFP: green fluorescent protein
MaxLFQ: MaxQuant label-free quantification
PPI: protein–protein interaction
TMT: tandem mass tag
Y2H: yeast two-hybrid.
