# Supplementary material for: A Double-Barrel Liquid Chromatography-Tandem Mass Spectrometry (LC-MS/MS) System to Quantify 96 Interactomes per Day
Source: Mol Cell Proteomics. 2015 Apr 17;14(7):2030–41. doi: 10.1074/mcp.O115.049460 (PMC4587330; doi:10.1074/mcp.O115.049460)
Supplement: Supplemental Data [file supp_14_7_2030__index.html]

A double-barrel LC-MS/MS system to quantify 96 interactomes per day — A Double-Barrel Liquid Chromatography-Tandem Mass Spectrometry (LC-MS/MS) System to Quantify 96 Interactomes per Day — Analyzing 96 Low-Complexity Proteomes per Day — Supplemental Data 

# A Double-Barrel Liquid Chromatography-Tandem Mass Spectrometry (LC-MS/MS) System to Quantify 96 Interactomes per Day

## Supplemental Data

**Files in this Data Supplement:**

- High throughput PPIs\_Supplemental Material\_and Figures - Suppl figures 1-9
- Supplementary Material\_14min - Data analysis for 14 min gradient pull downs
- Supplementary Material\_27min - Data analysis for 27min gradient pull downs
- single\_hit\_wonders\_14min - Spectra for single peptide protein identification in 14 min gradients
- single\_hit\_wonders\_27min - Spectra for single peptide protein identification in 27 min gradients
- Supplementary data ppis - Classification of all interactors
