# Supplementary material for: A Double-Barrel Liquid Chromatography-Tandem Mass Spectrometry (LC-MS/MS) System to Quantify 96 Interactomes per Day
Source: Mol Cell Proteomics. 2015 Apr 17;14(7):2030–41. doi: 10.1074/mcp.O115.049460 (PMC4587330; doi:10.1074/mcp.O115.049460)
Supplement: Supplemental Data [file supp_O115.049460_mcp.O115.049460-1.pdf]

Supplementary materials for A double-barrel LC-MS/MS system to quantify 96 interactomes per day by Fabian Hosp et al.

Suppl. Figures:

Figure S1: Life time of a 14 min gradient

Figure S2: Voltage switch

Figure S3: Optimization of LC-MS/MS data acquisition parameters

Figure S4: Pull-down reproducibility

Figure S5: Pull-down analysis overview

Figure S6: Bait protein sequence coverage pull-downs

Figure S7: Unsupervised hierarchical clustering for 14 and 27 min methods

Figure S8: Retaining chromatographic resolving power

Figure S9: Xcalibur queue support

Figure S1

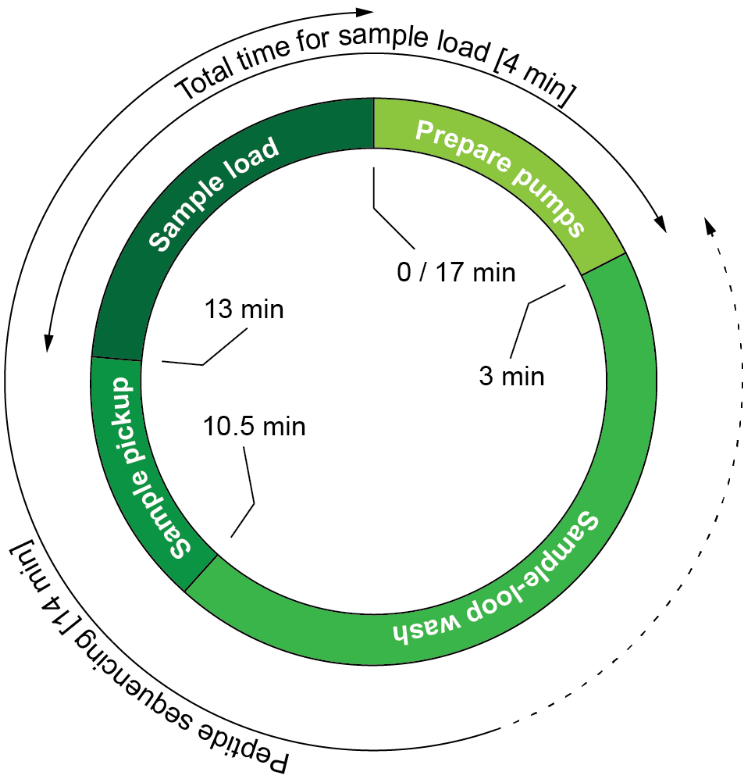

Liquid chromatography lifetime of a 14 minute gradient. At the start of the measurement pumps A and B reload with buffer and built up their pressure to the target pressure (for our experiments around 400 bar) (**Prepare pumps**). In the double barrel system the pressure is built up on the now inactive column used for the previous measurement preventing misaligned buffer from entering the active column. After 3 min the flow-path is switched to the active column and elution starts (outer ring '**Peptide sequencing [14 min]**' – the dotted line indicates the time of around 6 min where the peptides are not yet eluting). In parallel the system starts with **Sample-loop wash** with pump S, which ensures that the sample loop is cleaned of any remains of the previous sample (flush out with 1x buffer B and 1x buffer A; refill pump S to a volume at which the sample can still be put in the sample loop and consequently be loaded onto the analytical column). At **Sample pickup** the pump S is allowed to settle with the needle still in buffer A to prevent air in the pump for 1 min. After this 2 μl of sample is picked up at a speed of 10 μl / min, followed by a plug of 1 μl of air and 10 μl of buffer A to account for the volume of the needle. At **Sample load** the valve S is switched and pump S is pushing the sample from the sample loop at a pressure of maximum 550 bar (to prevent excessive droplet formation on the loading column). This process is extended beyond the end of the gradient to the stage **Prepare pumps** for the next round of measurements; a stage which is active on the now eluted and inactive analytical column. This yields a total loading time of 7 min, which is sufficient to get 4 μl of buffer A from pump S over the sample loop and to consequently to get the sample on the analytical column.

Figure S2

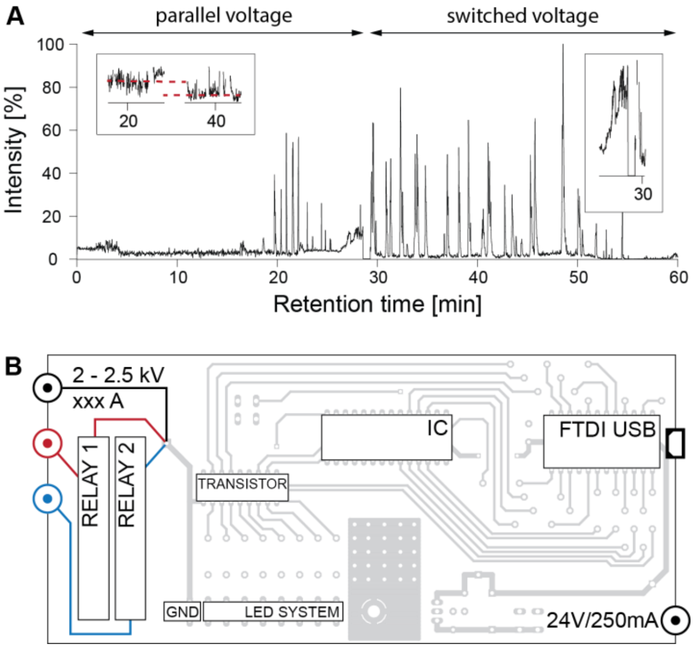

A high voltage switch is required to prevent excessive background from the loading analytical column. A. BSA run where the spray voltage is applied to both the analytical columns up to 30 min; from 30 min on the voltage is applied to the eluting column (switched voltage). During parallel voltage the basepeak chromatogram displays twice the background compared to the switched voltage (top left panel). When the loading of the not-eluting column starts at approximately 25 min, a pronounced increase of the background can be observed drowning all but the most intense peaks (top right panel). **B.** Wiring diagram representation of the high voltage switch with the most important components highlighted. The device operates on an external power source of 24V (250mA) to operate the switching required to transfer the spray voltage between the two columns (black connection at the bottom-right). The spray voltage from the mass spectrometer is fed into the device via the top-left black connection to the high voltage RELAY 1 and 2 (capable of operating up to 15kV). The state of RELAY 1 and 2 is controlled by the IC unit through the high voltage TRANSISTOR for which a dedicated embedded software module has been developed. In order to physically notify the operator about the state of RELAY 1 and 2, a LED SYSTEM has been installed with the appropriate coloring (i.e. red for RELAY 1 and blue for RELAY 2). The whole device can be externally controlled by a computer through the FTDI USB module.

Figure S3

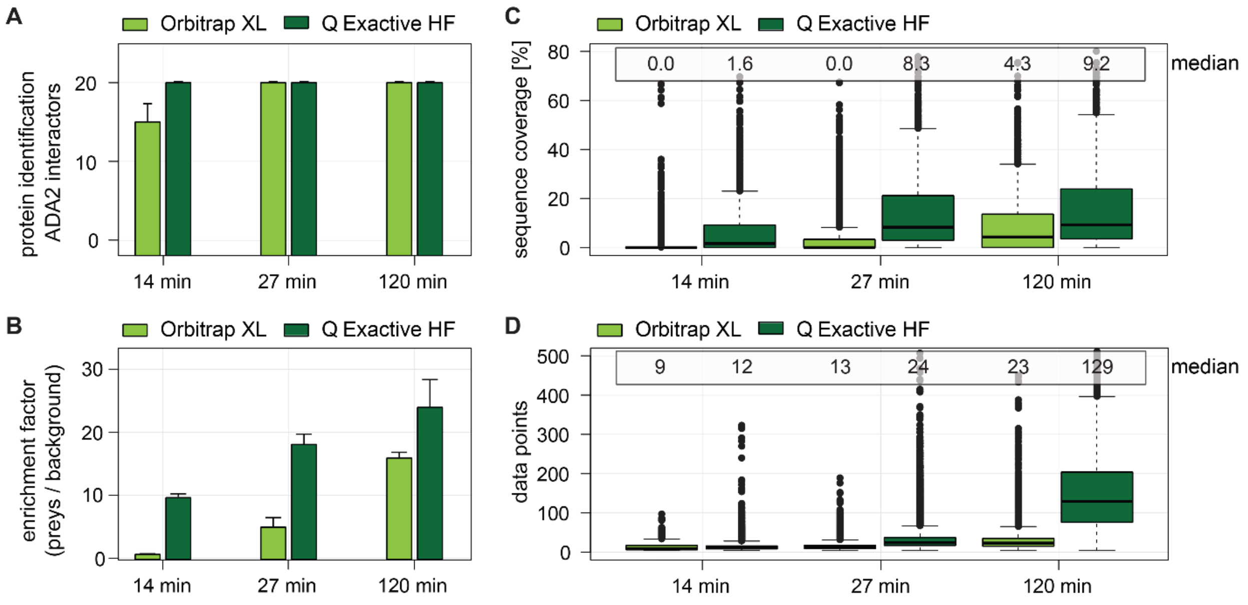

Optimization of LC-MS/MS data acquisition parameters. A. Recovery of known ADA2 interactors. Orbitrap performance drops at 15 min run-time. **B.** Q Exactive HF yields much higher enrichment factors. The enrichment is calculated by dividing the median MaxLFQ intensity of all known preys versus the median of all background proteins identified in the same samples. **C.** Sequence coverage boxplots of all identified proteins, the Q Exactive HF outperforms the Orbitrap XL for all LC-MS/MS acquisition times. **D.** Boxplots of the number of data points recorded per peak over all acquisition times. Again, the Q Exactive HF is significantly better than the older instrument type

Figure S4

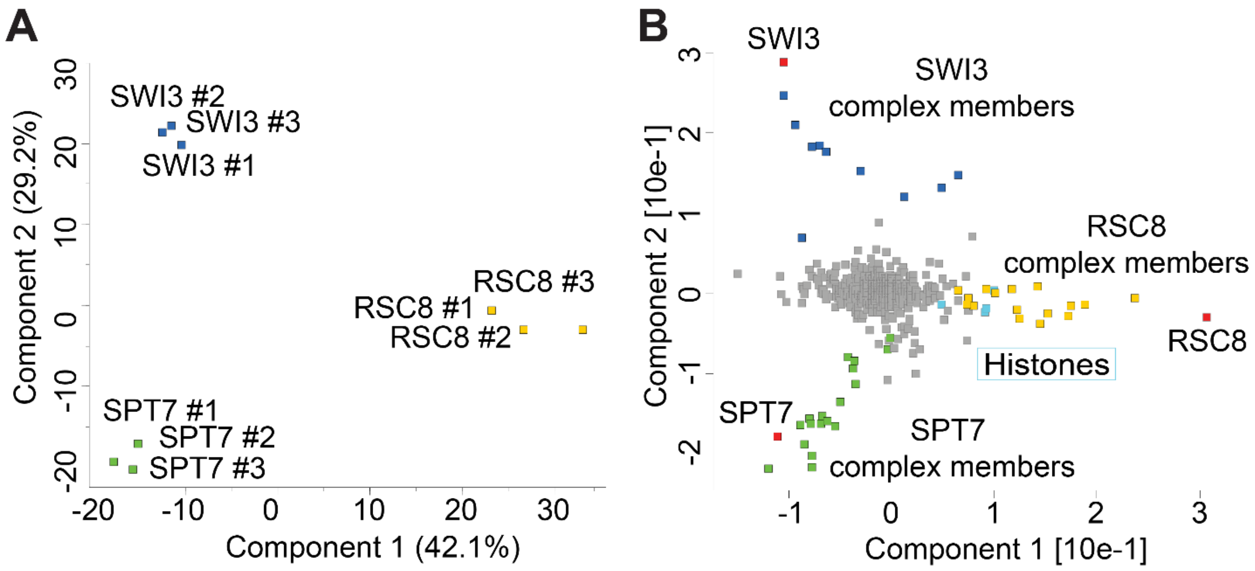

Pull-down reproducibility. A. Principal Component Analysis of 3 pull-downs run on the double-barrel LC-MS setup. **B.** All biological replicates cluster close together. In the loading plot (right) it is clearly visible that the complex members are responsible for the separation observed in the PCA

Figure S5

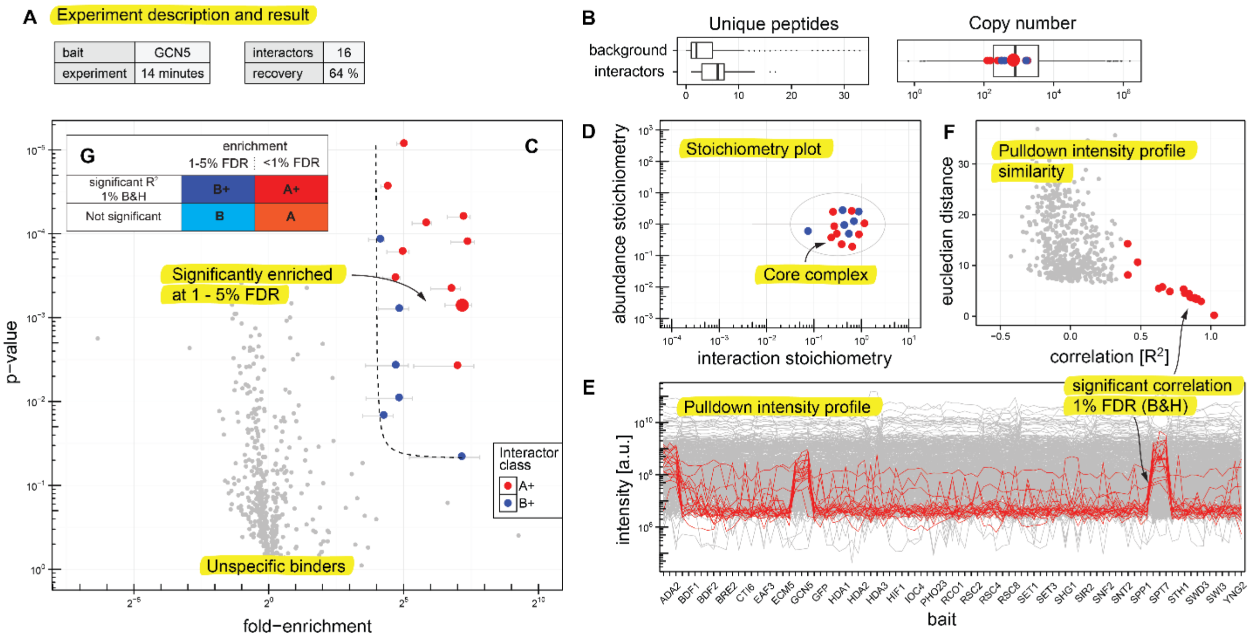

Pull-down analysis overview provided for each individual pull-down experiment. A. A short description of the experiment (incl. pull-down and gradient time) and the result of the pull-down. The recovery is calculated based on a list of known interactors extracted from the CORUM database. **B.** The difference in unique peptides is an indicator for the successful enrichment of the interactors. The copy number plot indicates the copy numbers of the bait and the identified interactors in the copy number distribution of the entire yeast proteome. **C.** Significance of enrichment calculated with a t-test (s0=1). Permutation-based FDR control is applied on the adjusted p-value (250 permutations) and the proteins selected at <1% and 1-5% FDR. Unspecific binders cluster around fold-enrichment of 1 (i.e. no enrichment) and are reported in grey. **D.** The stoichiometry plot provides information about the strength of the detected interactions. Abundance stoichiometry is calculated by dividing the yeast copy-number of the interactor by that of the bait; indicates whether the bait and interactors have been found to be close in copy number in a normal yeast cell. The interaction stoichiometry is calculated by dividing the absolute protein abundance of each interactor by that of the bait (Abundance is calculated as iBAQ values; the median iBAQ value of all proteins in each experiment is subtracted to account for unspecific binding of each protein in that particular experiment). The interaction stoichiometry indicates the strength of the interaction between bait and interactor. The circle in the stoichiometry plot has been experimentally determined to be the boundary of strong interactions likely forming the core complex (Hein et al). **E.** Display of the intensity profiles of all the detected proteins. The profiles of the proteins significantly correlating (1% FDR corrected with Benjamini & Hochberg) with that of the bait are marked in red. Good correlation is indicative of true interaction. The intensity profile plot can also reveal relationships between complexes, like shared subunits (Keilhauer et al). **F.** For each detected protein, the correlation of the intensity profile to the profile of the bait is plotted against the Euclidian distance of the intensity profile to the profile of the bait. The significantly (1% FDR corrected with Benjamini & Hochberg) correlating proteins are marked in red. **G.** Combining the enrichment significance with the correlation significance we define four distinct classes: A+, <1% FDR enrichment & <1% FDR correlation; A, <1% FDR enrichment; B+, 1-5% FDR enrichment & <1% FDR correlation; B, only 1-5% FDR enrichment. From comparisons to known data we found class B to mainly contain false positives, and consequently excluded these from further analysis.

Figure S6

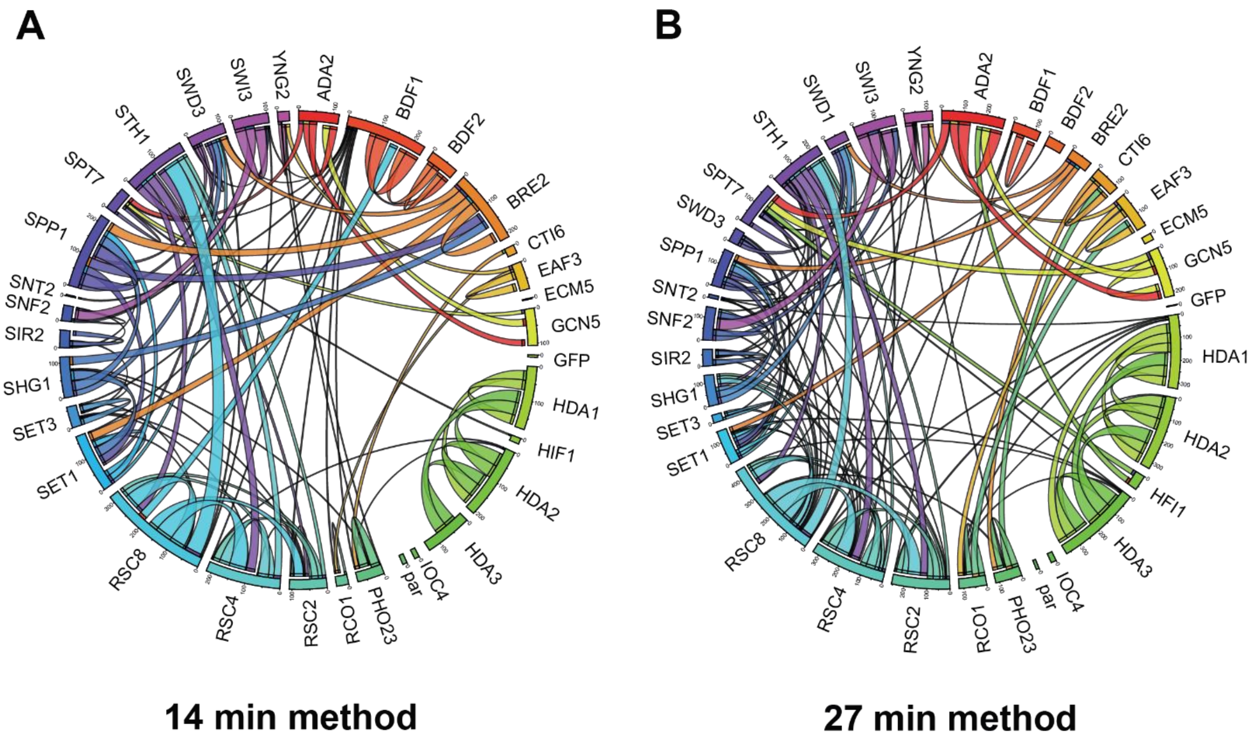

Sequence coverage of bait proteins. Circular plots representing the recurrent sequencing of bait proteins in pull-downs of distinct baits and thus their connectivity across different complexes. Outgoing ribbons (touching the circularly arranged stacked bars) indicate identification of the respective bait protein in another pull-down experiment (ribbon not touching the stacked bars). The length of the circularly arranged stacked bars is the relative contribution of both incoming and outgoing ribbons for each bait protein. **A.** Bait sequence coverage from pull-downs generated with the 14 min LC-MS/MS analysis. **B.** From pull-downs generated with the 27 min LC-MS/MS analysis.

Figure S7

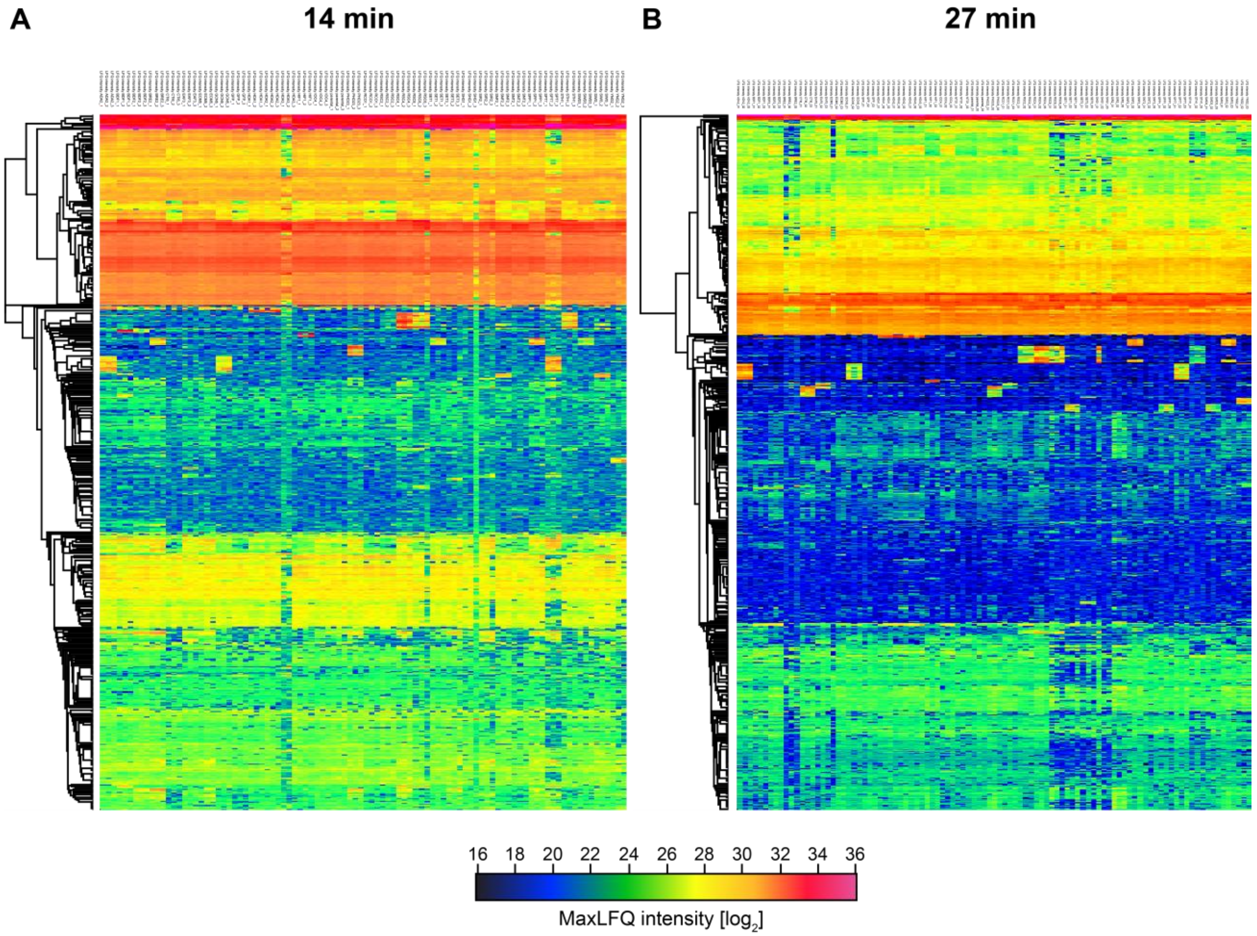

Unsupervised hierarchical clustering of all identified proteins from the pull-down experiments. A. Heatmap plot of proteins identified from pull-downs generated with the 14 min LC-MS/MS analysis. **B.** From pull-downs generated with the 27 min LC-MS/MS analysis. Color coding is relative to the log2-transformed MaxLFQ intensity. Whereas unspecific protein binders show a continuous distribution, specific interactors form separated clusters indicative of distinct protein complexes.

Figure S8

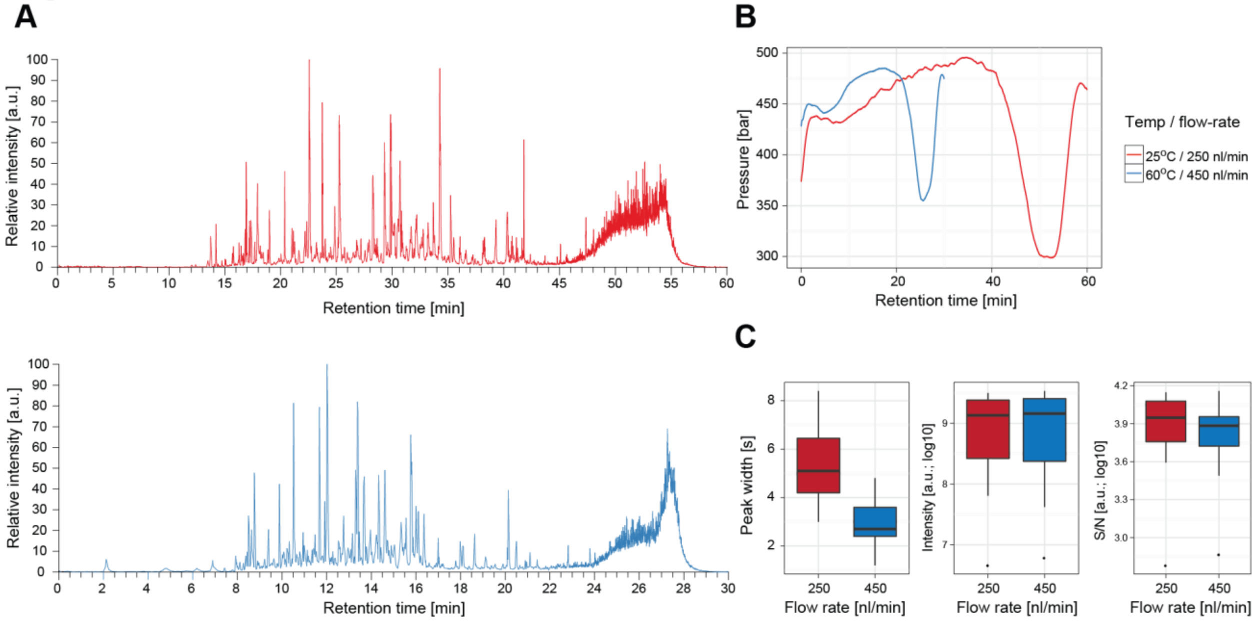

Retaining chromatographic resolving power and sensitivity while reducing the gradient length to half on a BSA digest on a 50 cm analytical column. A. For half the chromatographic time the peak-width is automatically reduced to half providing the potential for the same chromatographic resolving power (see also Fig. 2 for a discussion). In order to spread the peptides over the same area of the gradient the flow-rate needs to be increased for the shorter gradient, in this case from 250 nl/min to 450 nl/min. **B.** By increasing the temperature in the column oven the back pressure can be kept at the same level for both flow-rates. Here we found that operating at 25°C for a flow-rate of 250 nl/min results in the same back pressure as operating at 60°C for a flow-rate of 450 nl/min. **C.** For the set of BSA peptides, the peak-width is indeed reduced to half from 5 to 2.5 s. The summed intensity of the peaks is unaffected, and more importantly the signal-to-noise is only very slightly affected. With the aforementioned adjustments the short gradient results in the same chromatographic resolving power and sensitivity.

Figure S9

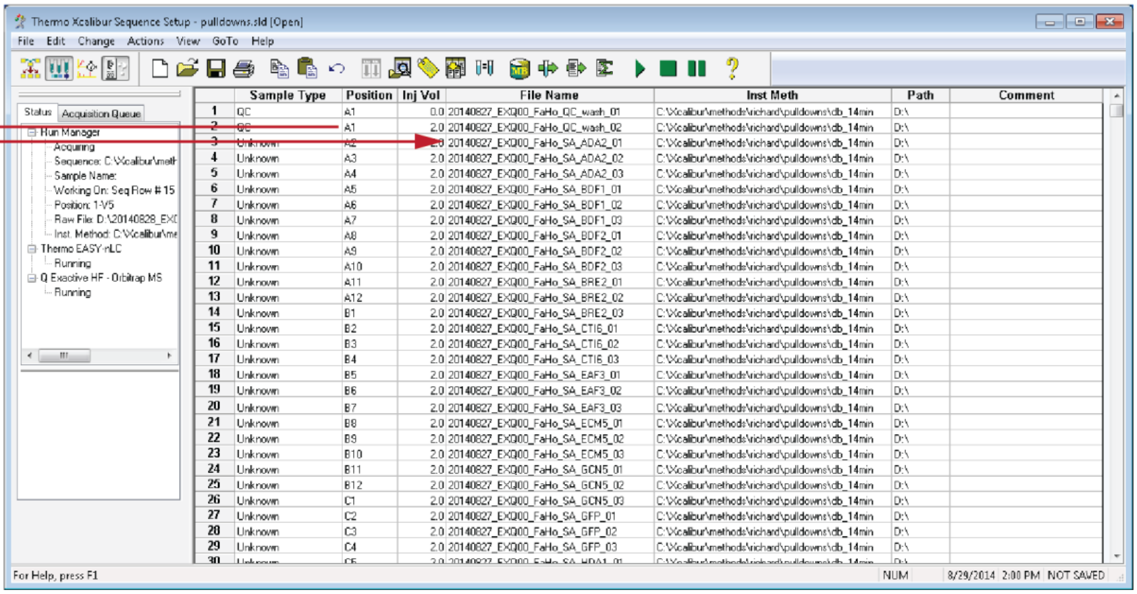

Xcalibur queue supporting the double barrel setup. Even though Xcalibur has no native support for two analytical columns we can still use the queue by moving each injection position up one row. As the inactive column is loaded during the elution of the active column, the vial position on row up corresponds to the loading position for the next run. During our tests we have found it easiest to create the queue, export if to CSV (from the file menu), shift all rows of the column Position up by one in Excel and re-importing the queue from the CSV file.
