# Supplementary material for: A Double-Barrel Liquid Chromatography-Tandem Mass Spectrometry (LC-MS/MS) System to Quantify 96 Interactomes per Day
Source: Mol Cell Proteomics. 2015 Apr 17;14(7):2030–41. doi: 10.1074/mcp.O115.049460 (PMC4587330; doi:10.1074/mcp.O115.049460)
Supplement: Supplemental Data [file supp_O115.049460_mcp.O115.049460-2.pdf]

Ultra−high Throughput LC−MS/MS Protein Interaction Analysis – Processing 96 Pull−downs in a Single Day

Experiment: 14 minutes

F. Hosp*, R.A. Scheltema*, H.C. Eberl*, N.A. Kulak*, E.C. Keilhauer, K. Mayr and M. Mann

Missing values

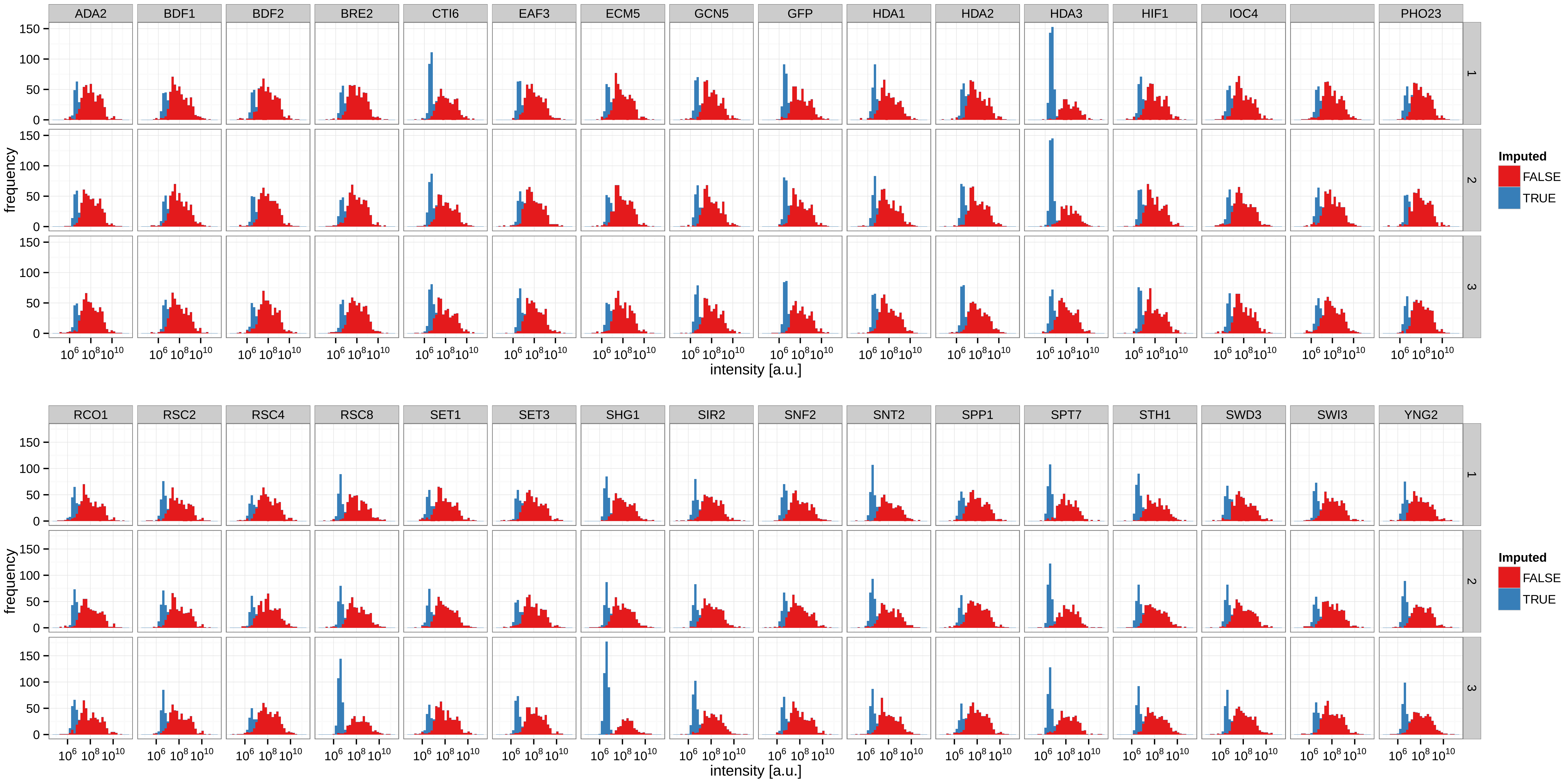

**bait**	ADA2		**interactors**	29
**experiment**	14 minutes		**recovery**	73 %

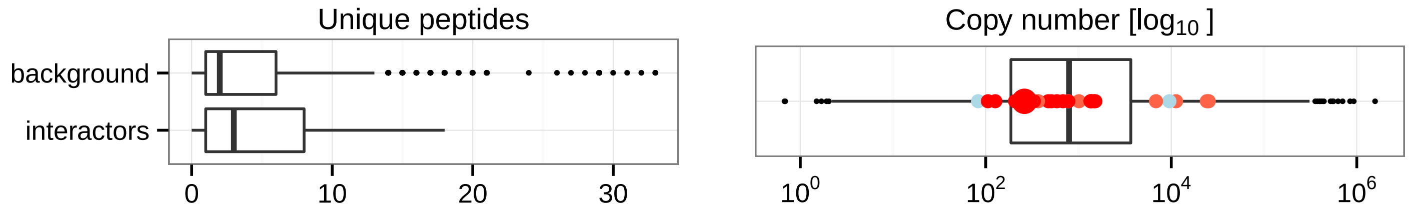

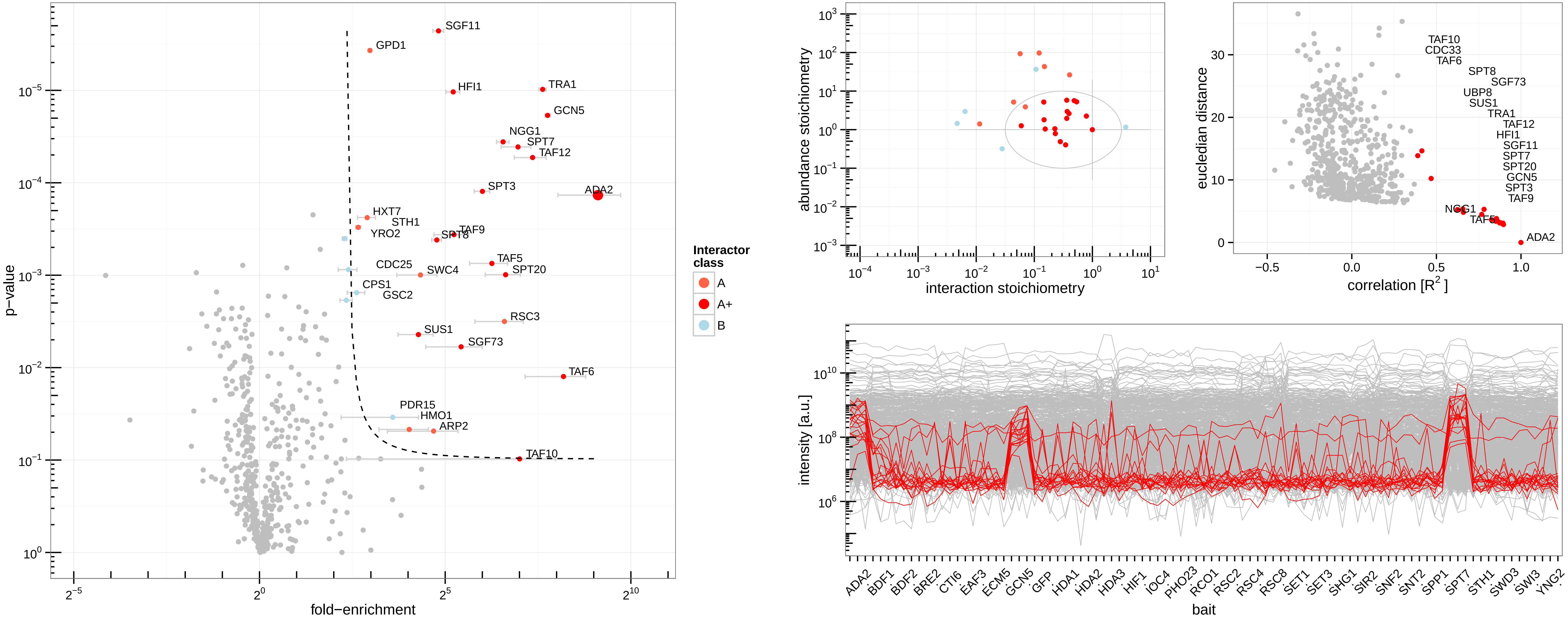

	ADA2	ARP2	GCN5	GPD1	HFI1	HMO1	HXT7	NGG1	RSC3	SGF11	SGF73	SPT20	SPT3	SPT7	SPT8	STH1	SUS1	SWC4	TAF10	TAF12	TAF5	TAF6	TAF9	TRA1
significance	9.0	3.9	8.2	5.8	6.5	3.3	4.1	7.1	6.1	6.8	5.0	6.4	6.3	7.4	5.1	3.9	4.0	4.5	6.1	7.7	6.1	7.4	5.5	8.3
interactor class	A+	A	A+	A	A+	A	A	A+	A	A+	A+	A+	A+	A+	A+	A	A+	A	A+	A+	A+	A+	A+	A+

**bait**	BDF1
**experiment**	14 minutes

**interactors**	38
**recovery**	31 %

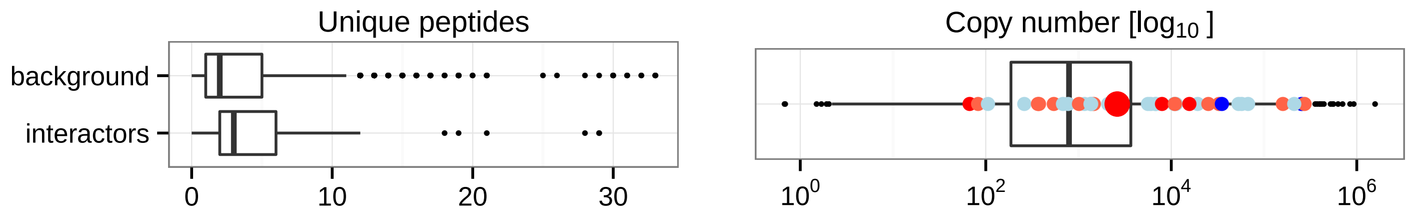

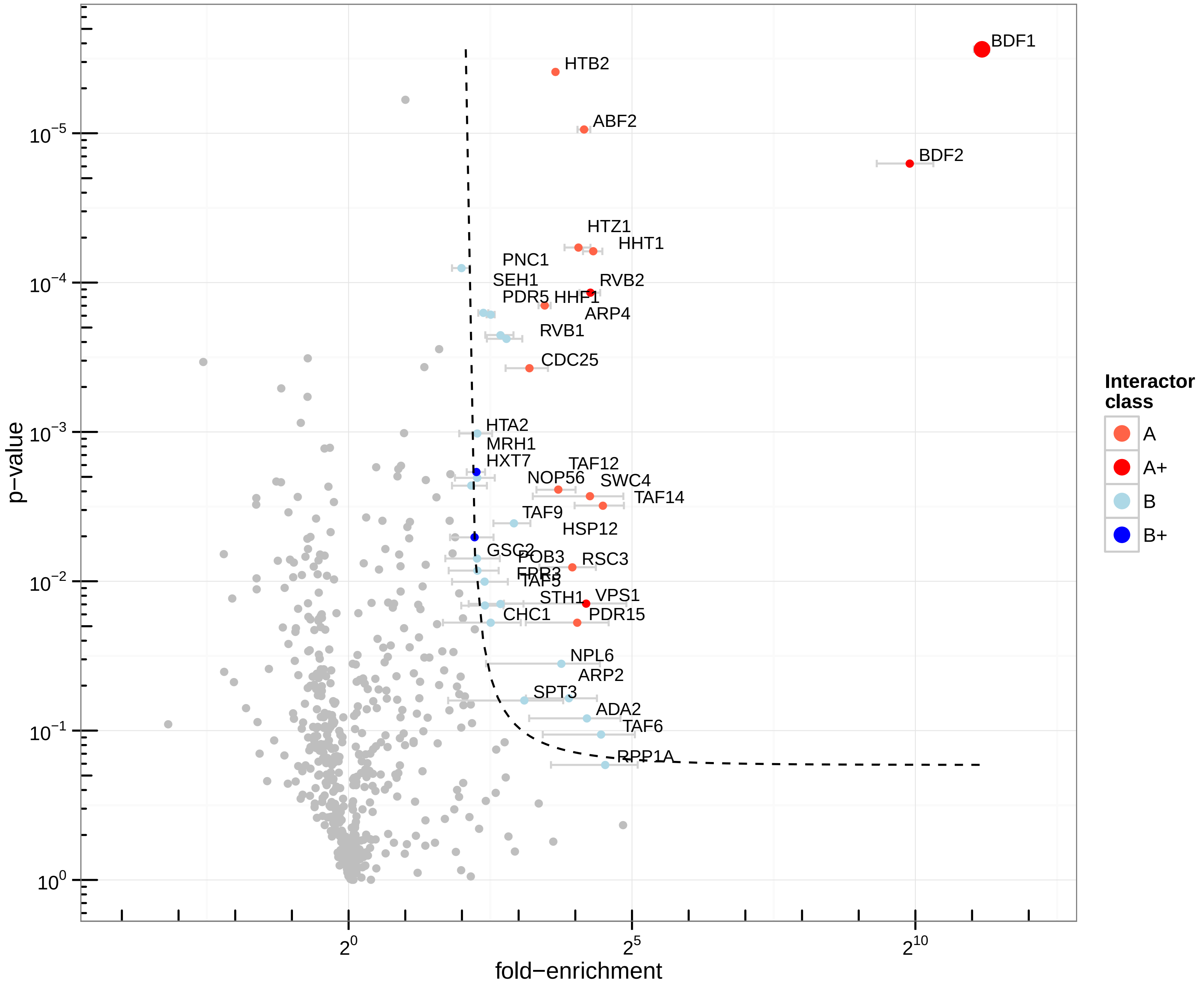

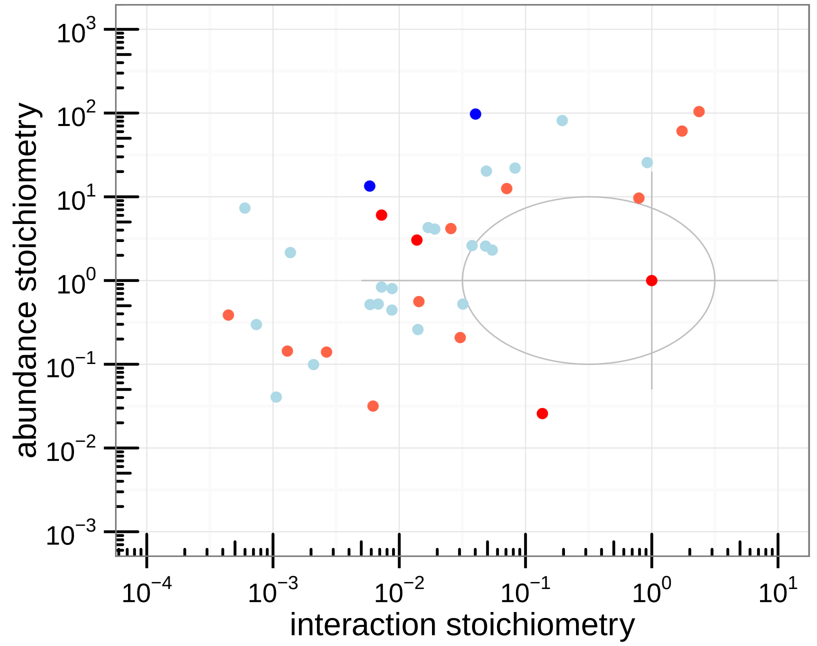

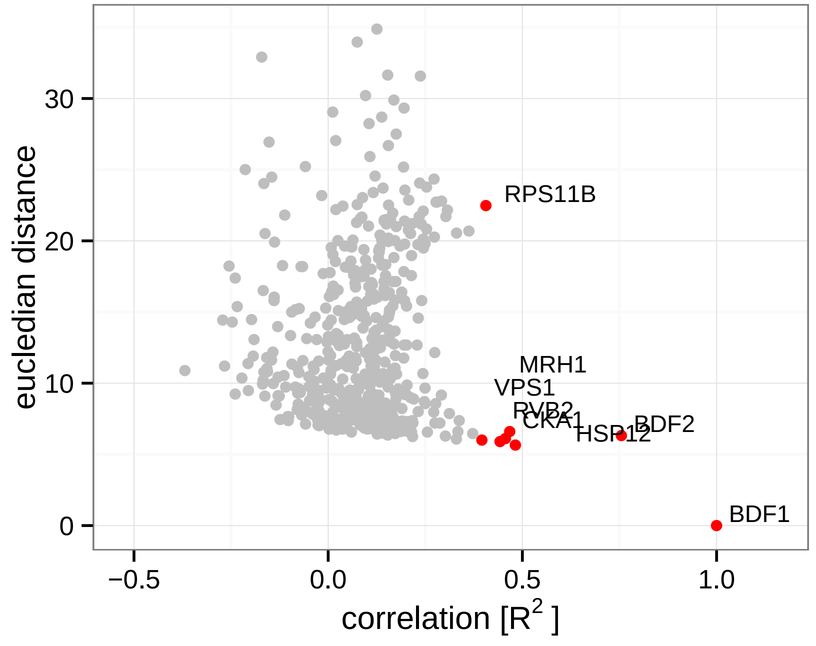

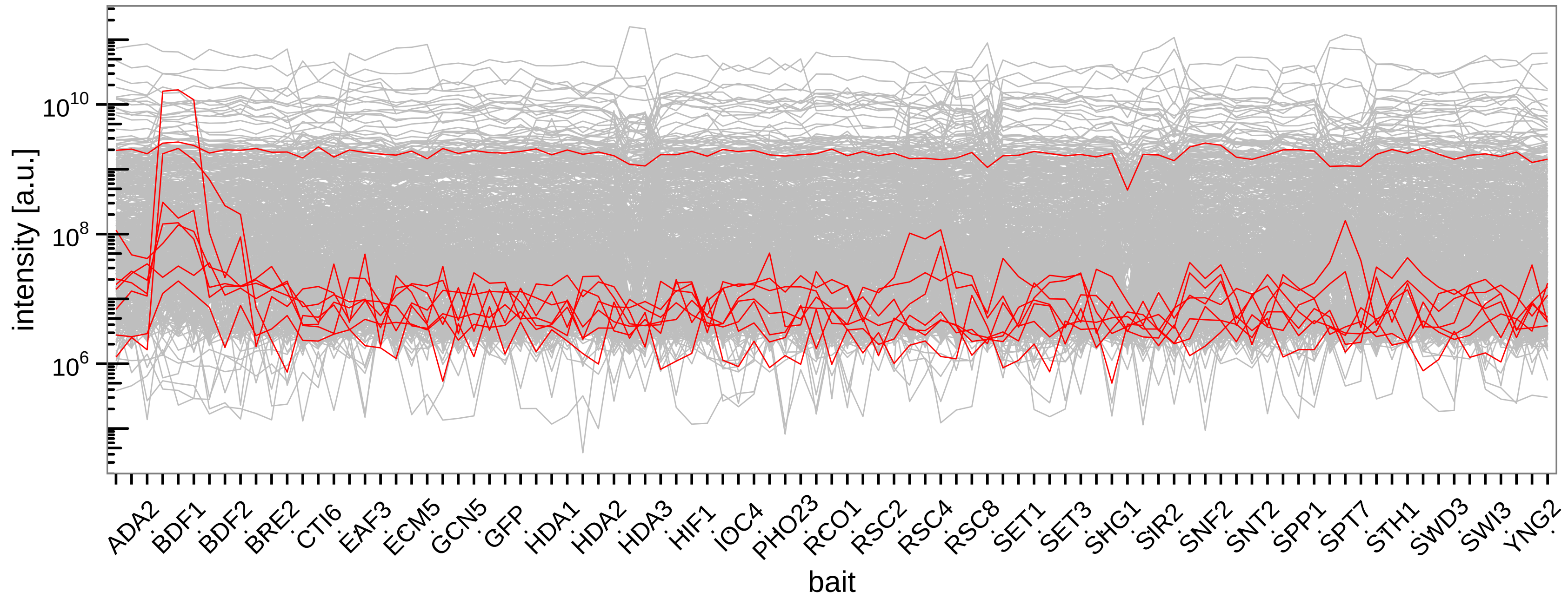

	ABF2	BDF1	BDF2	CDC25	HHF1	HHT1	HSP12	HTB2	HTZ1	MRH1	PDR15	RSC3	RVB2	SWC4	TAF12	TAF14	VPS1
significance	5.9	12.0	10.0	4.1	4.6	5.4	2.6	6.0	5.2	3.0	3.5	3.6	5.1	4.2	3.8	4.3	3.7
interactor class	A	A+	A+	A	A	A	B+	A	A	B+	A	A	A+	A	A	A	A+

**bait**	BDF2
**experiment**	14 minutes

**interactors**	1
**recovery**	0 %

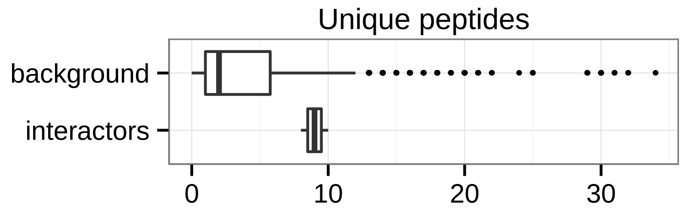

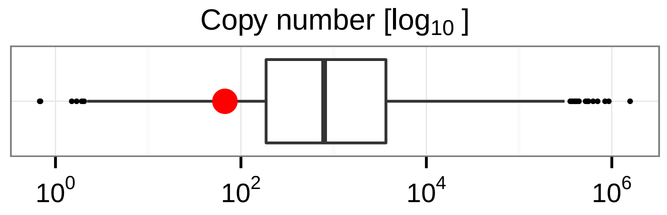

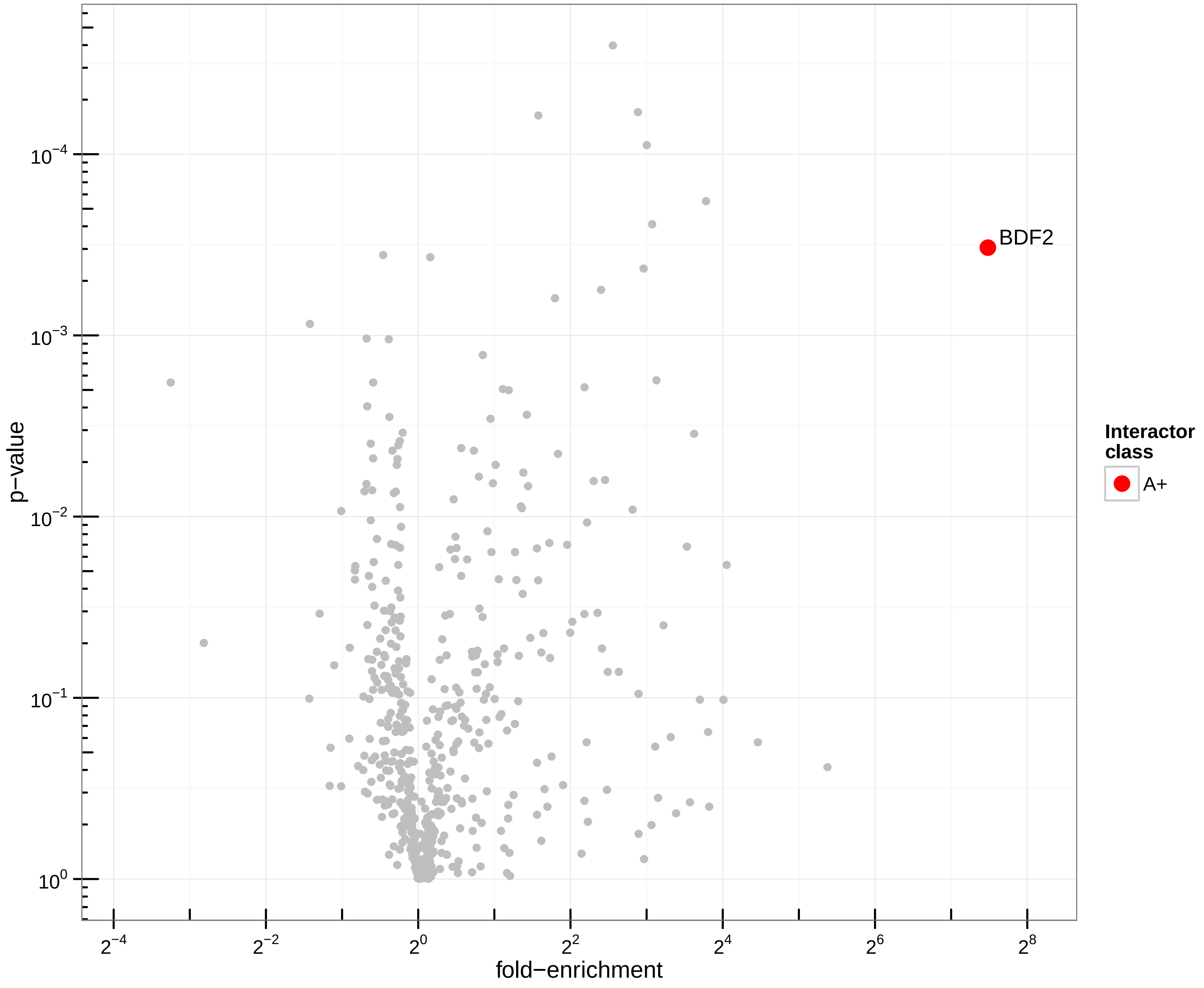

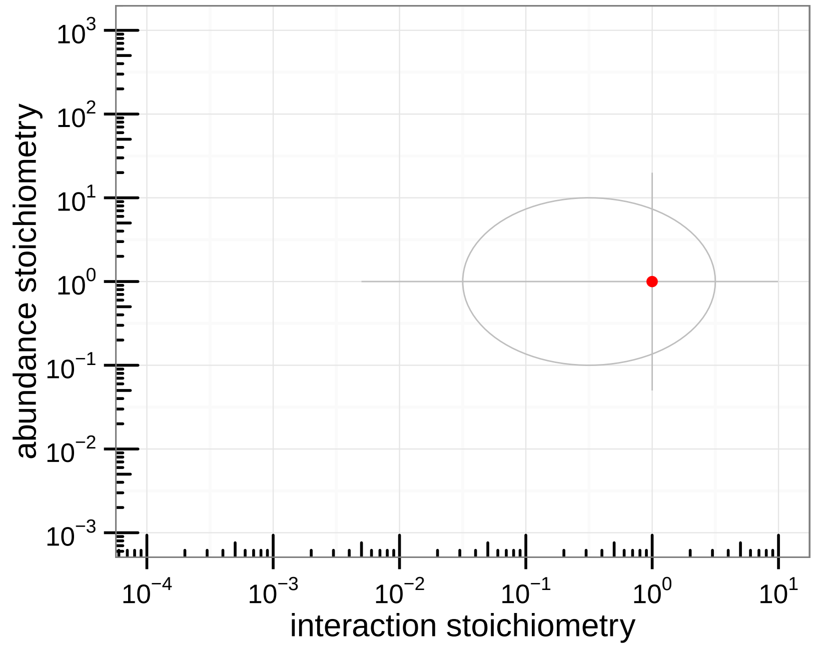

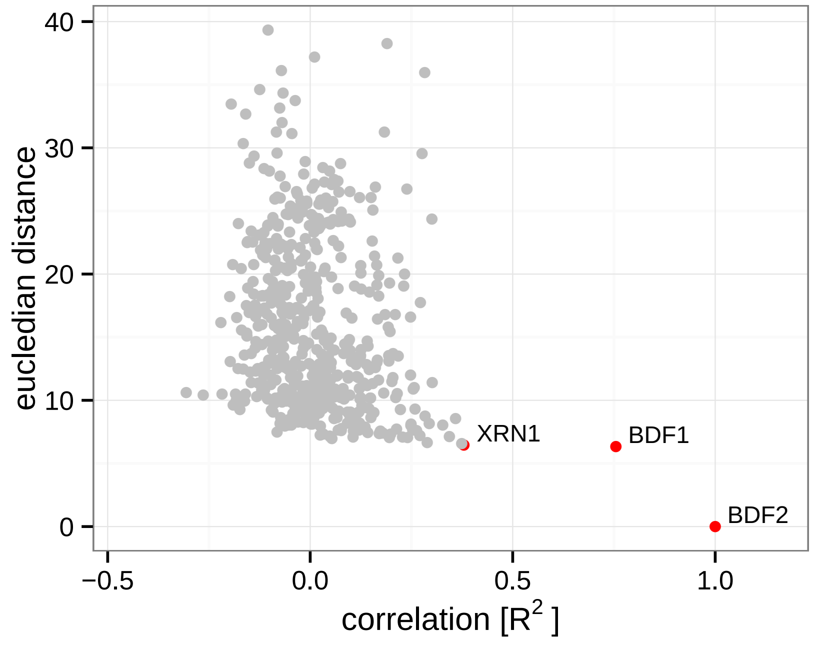

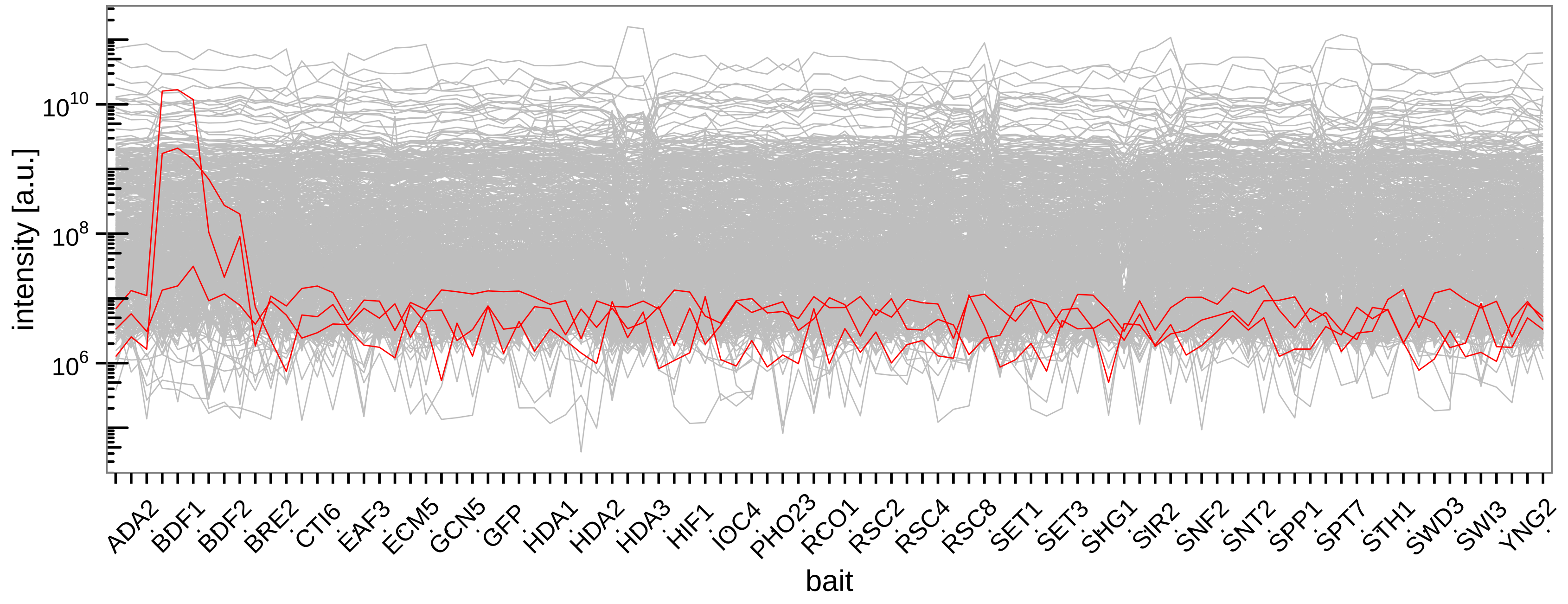

	BDF2
significance	7.4
interactor class	A+

**bait**	BRE2		**interactors**	43
**experiment**	14 minutes		**recovery**	100 %

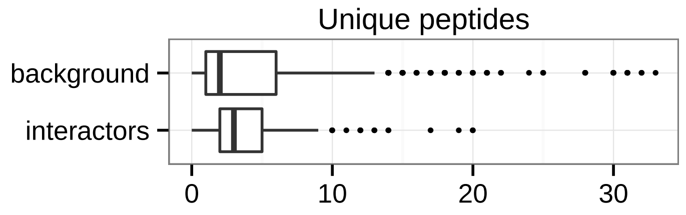

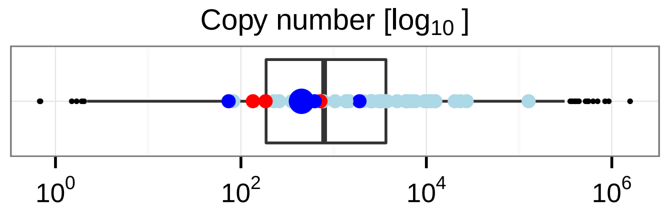

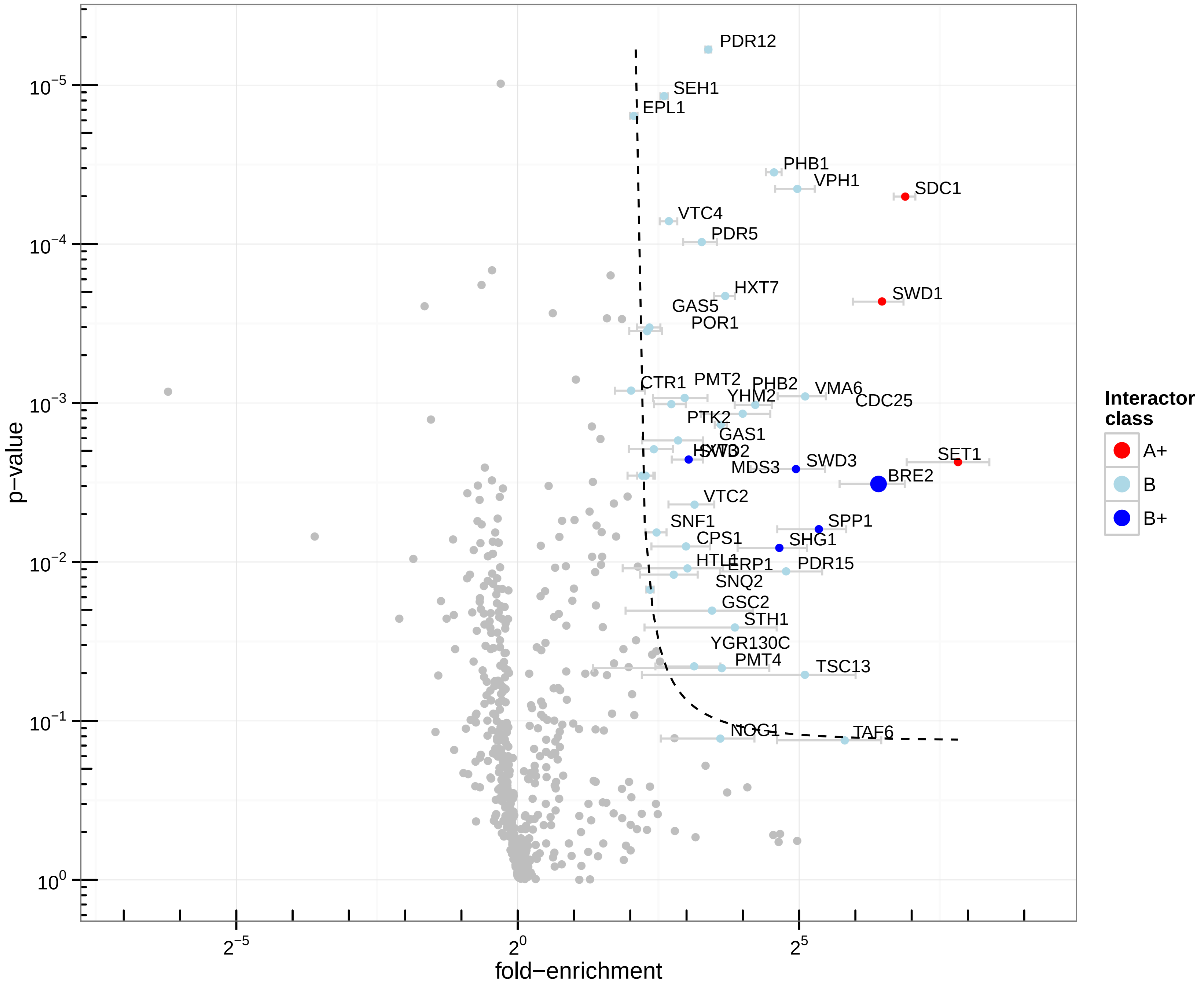

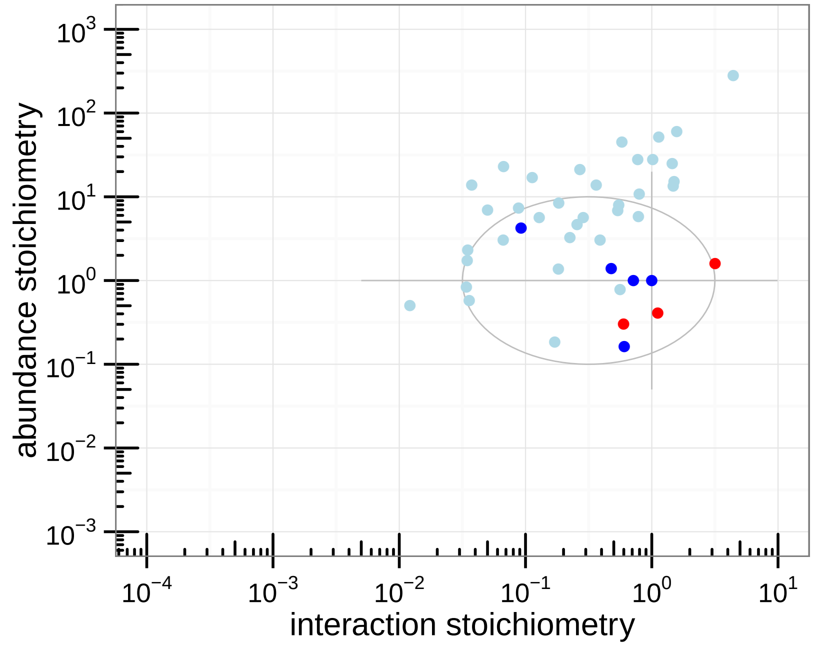

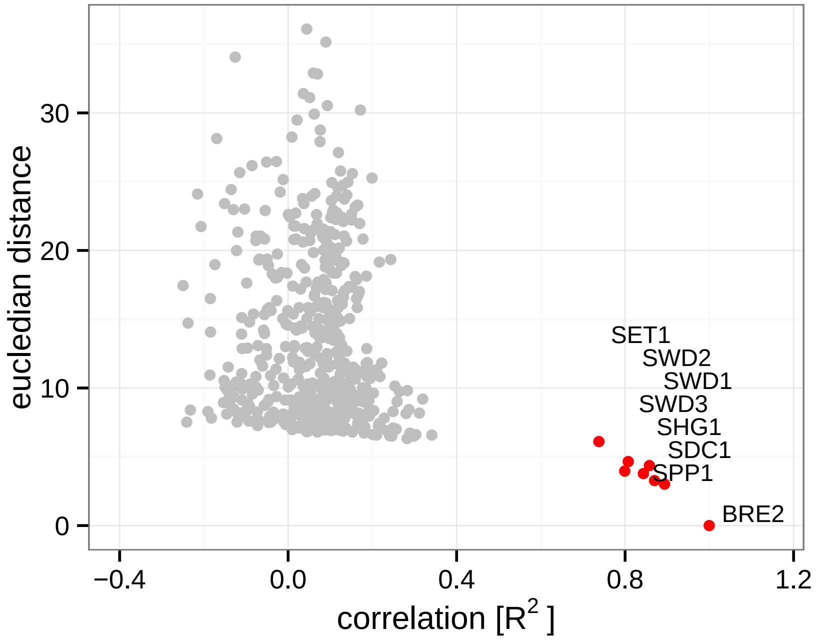

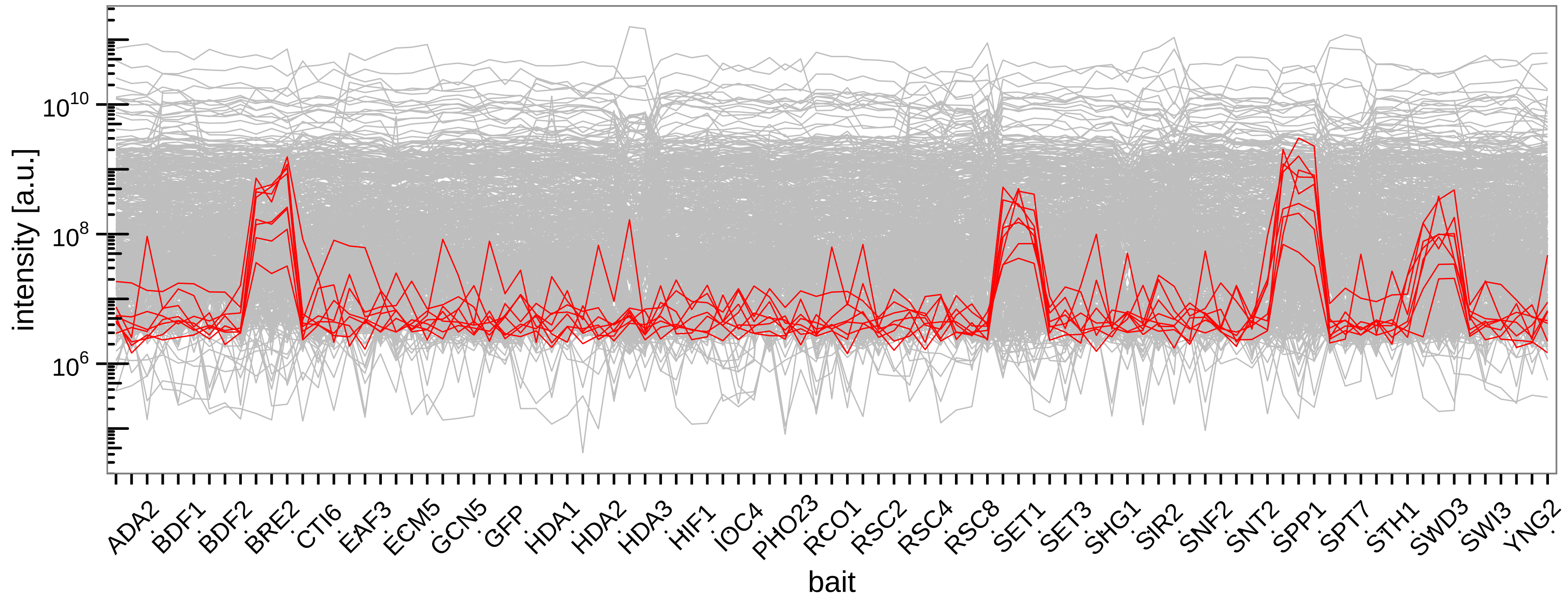

	BRE2	SDC1	SET1	SHG1	SPP1	SWD1	SWD2	SWD3
significance	6.0	7.3	7.3	4.2	4.9	6.6	3.3	4.7
interactor class	B+	A+	A+	B+	B+	A+	B+	B+

**bait**	CTI6
**experiment**	14 minutes

**interactors**	2
**recovery**	0 %

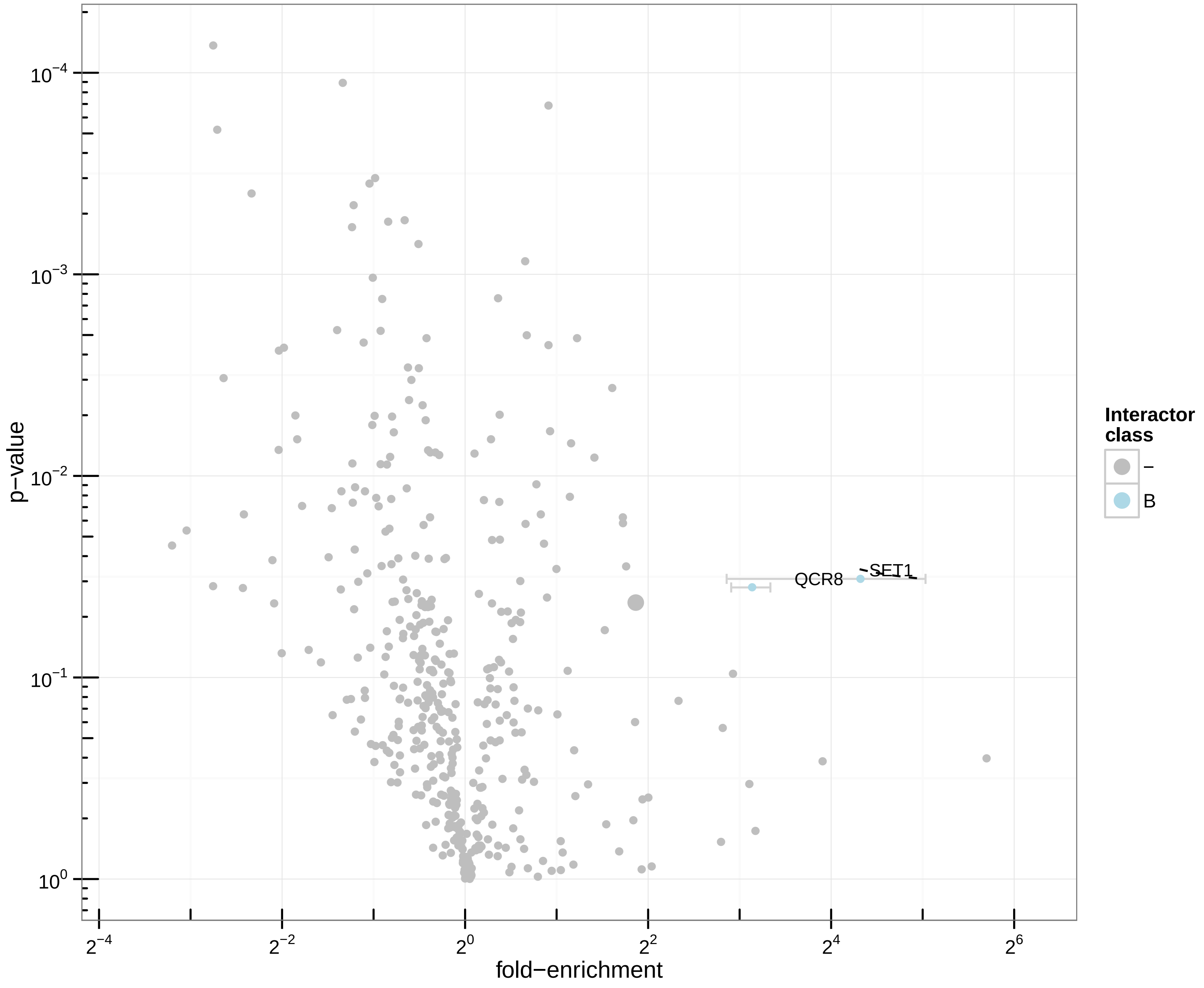

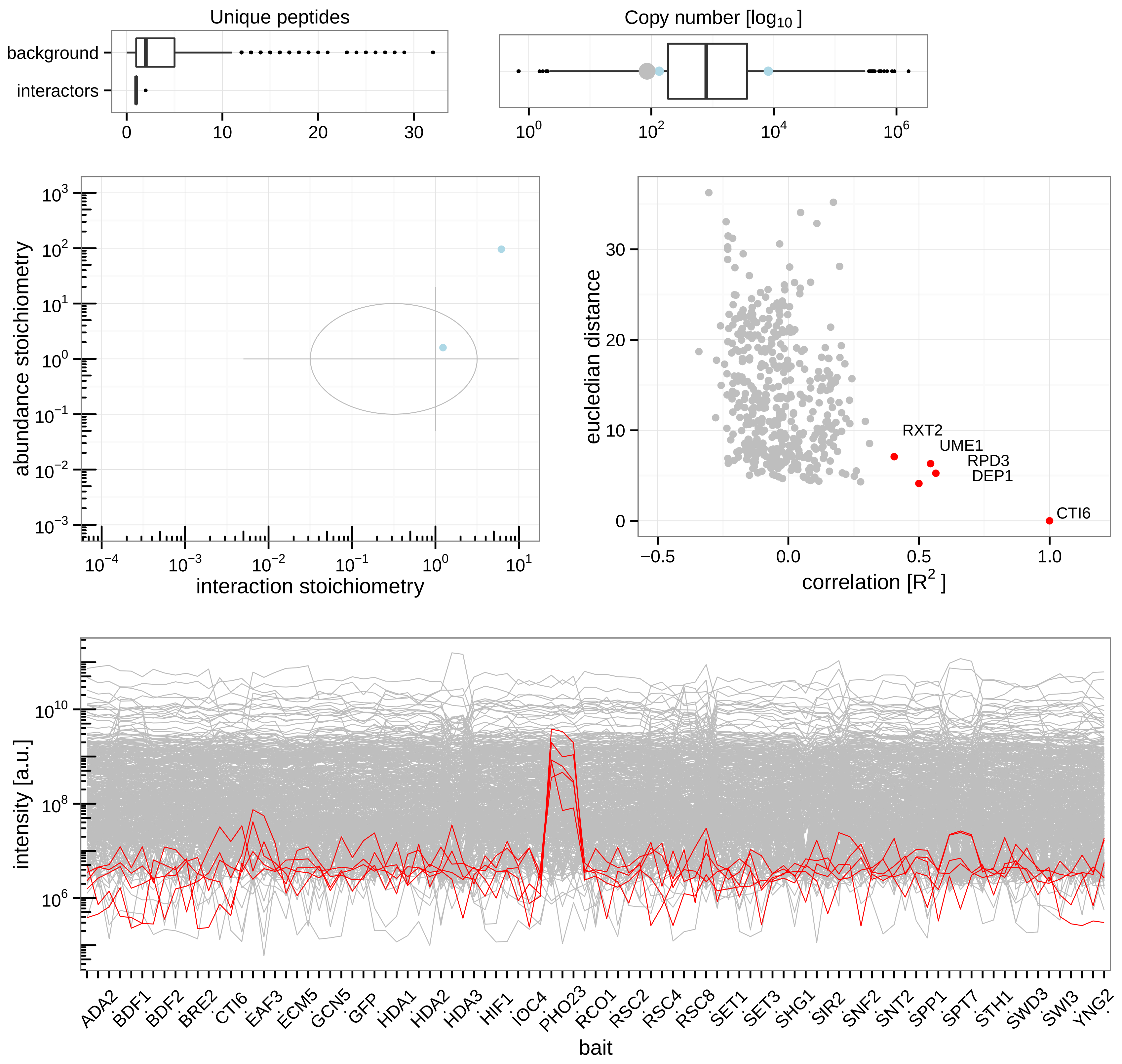

significance

interactor class

**bait**	EAF3
**experiment**	14 minutes

**interactors**	3
**recovery**	3 %

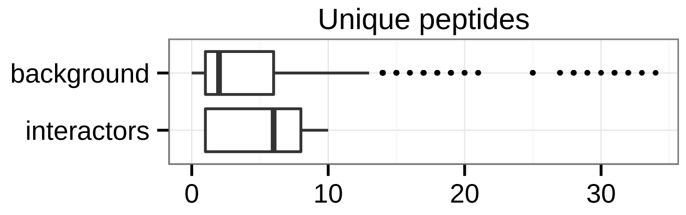

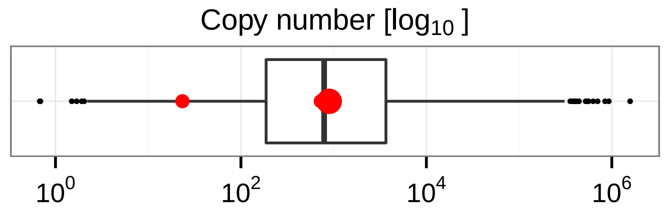

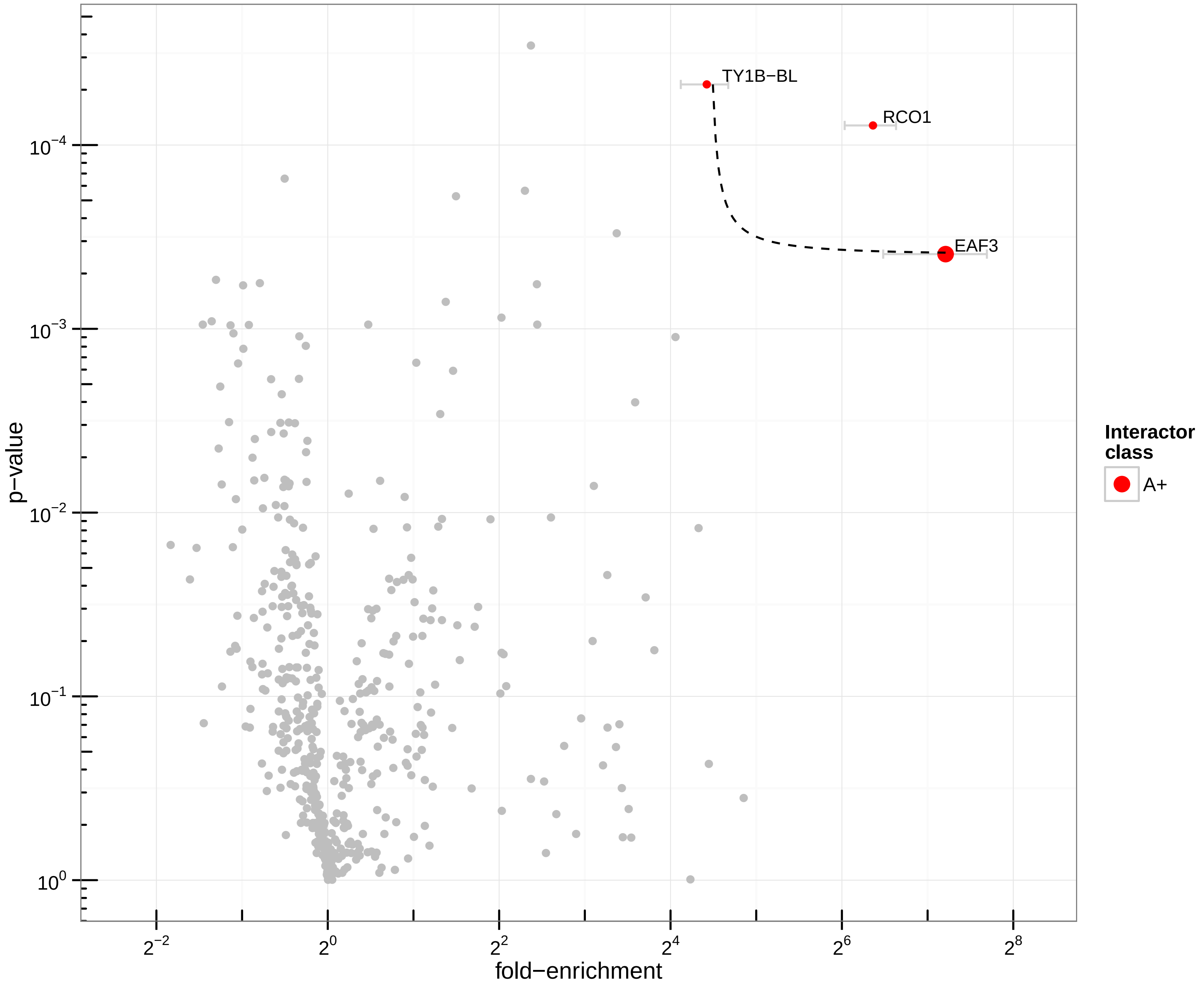

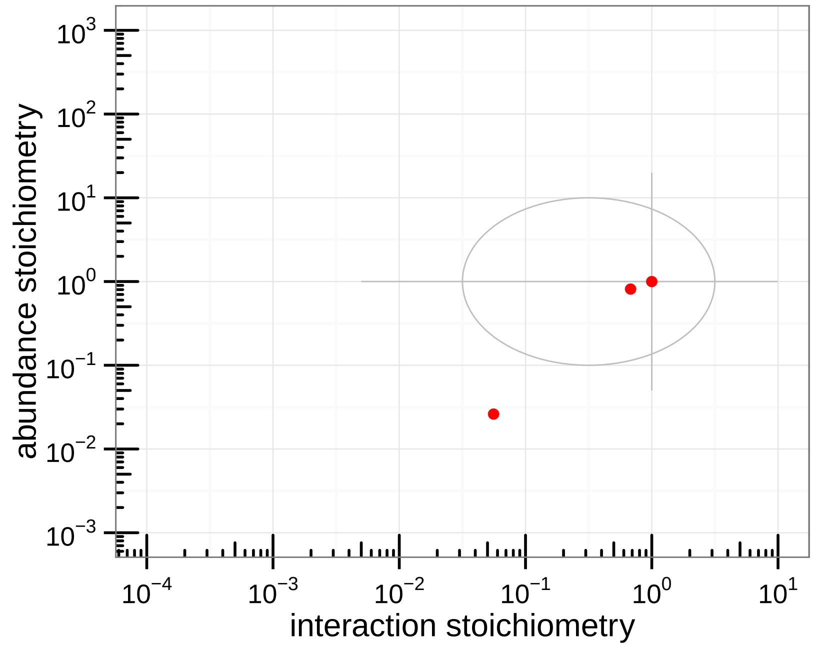

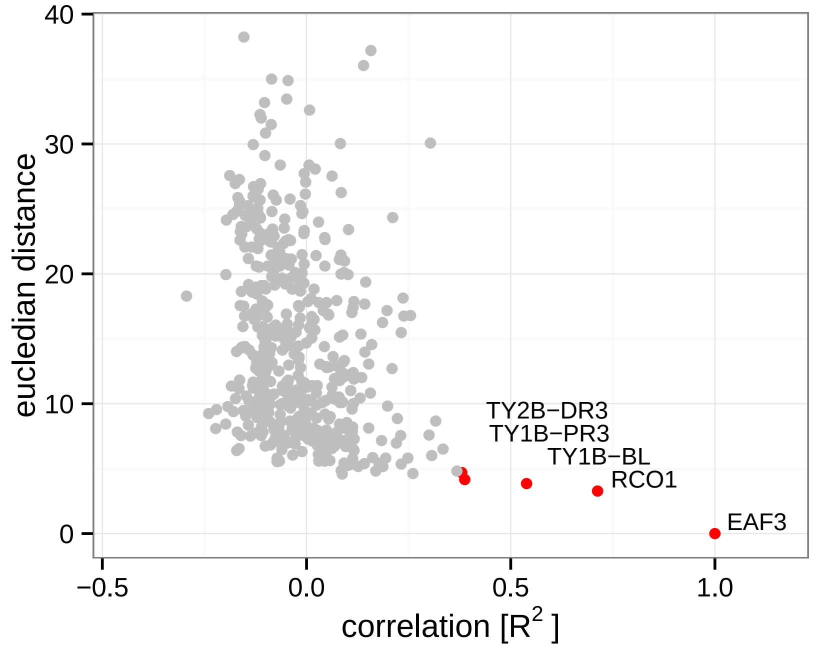

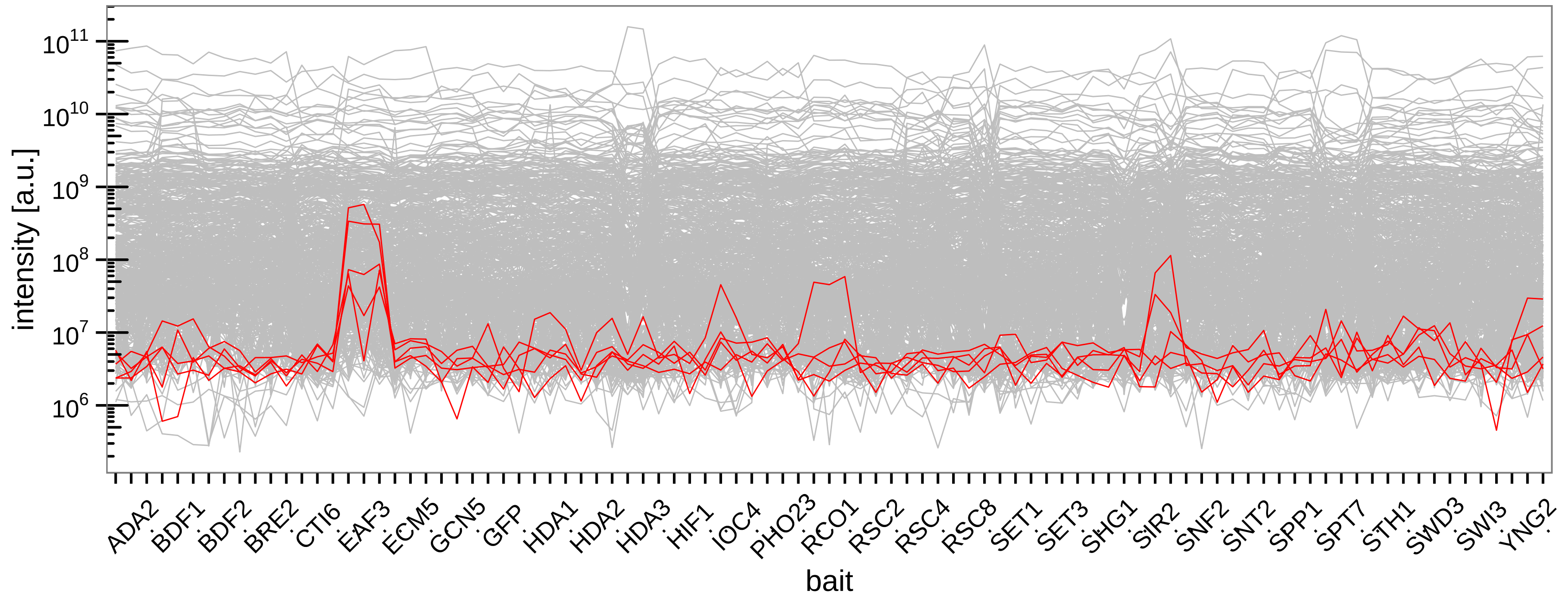

	EAF3	RCO1	TY1B−BL
significance	7.1	6.8	5.5
interactor class	A+	A+	A+

**bait**	ECM5
**experiment**	14 minutes

**interactors**	0
**recovery**	0 %

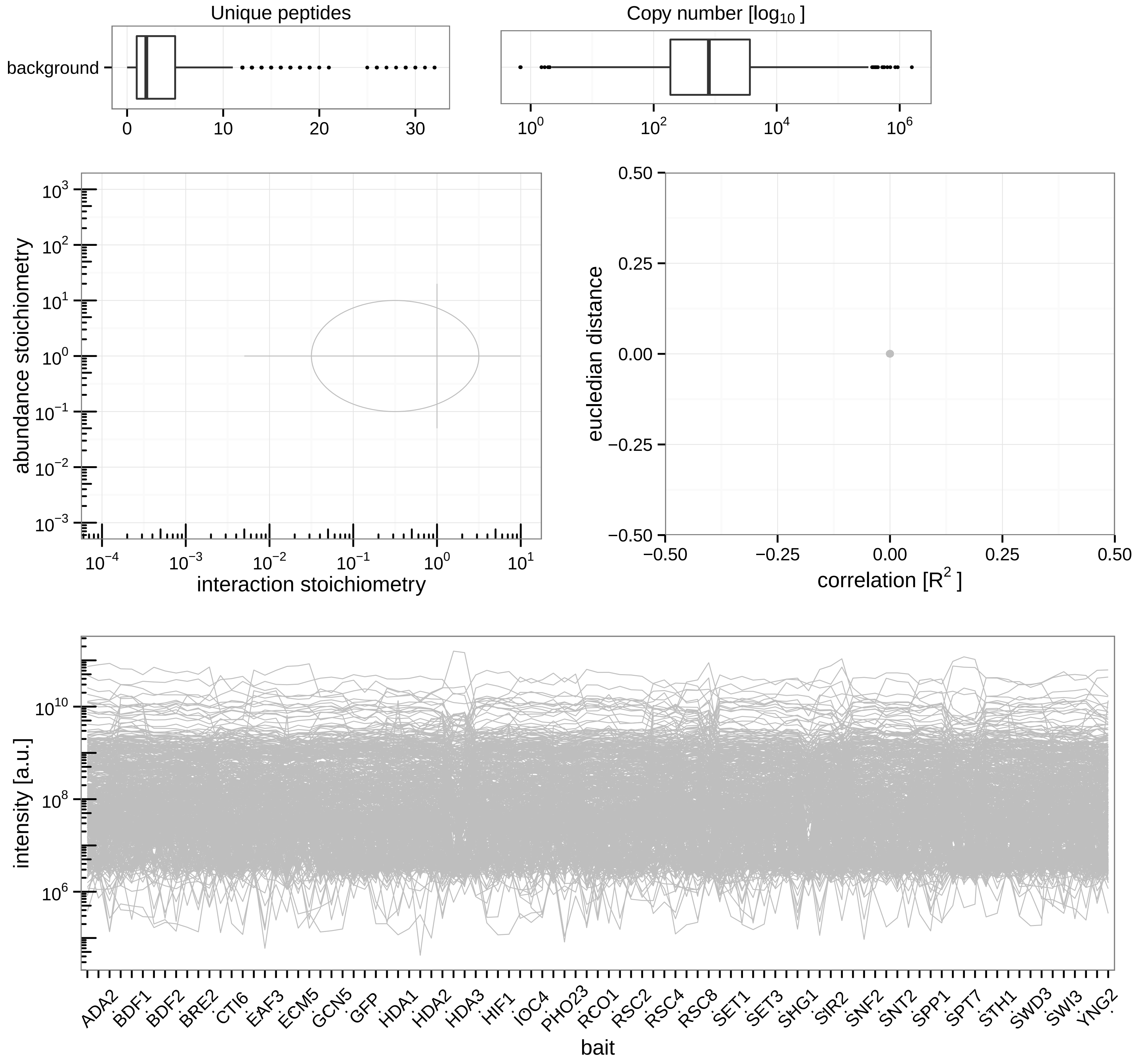

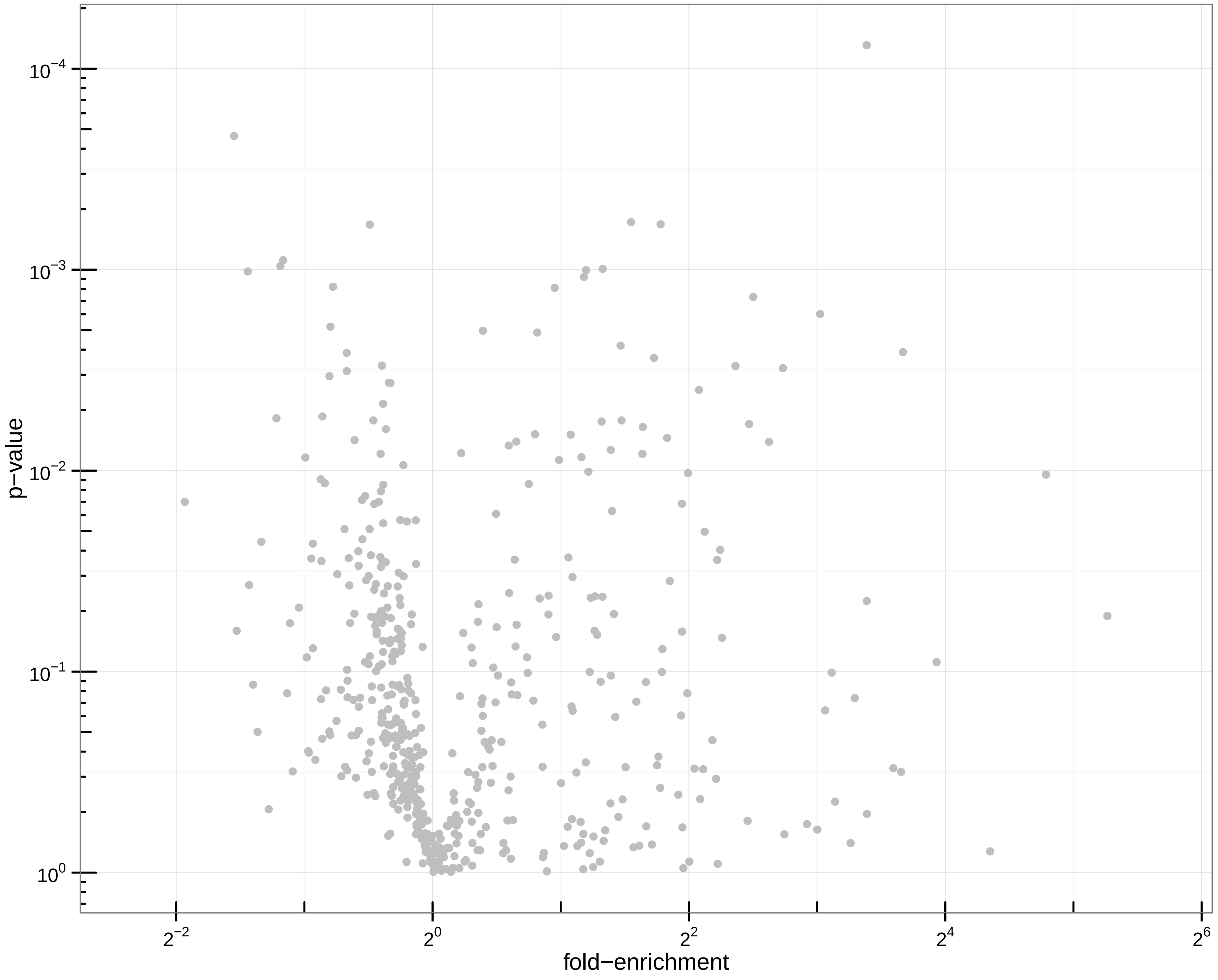

significance

interactor class

bait	GCN5
experiment	14 minutes

interactors	16
recovery	67 %

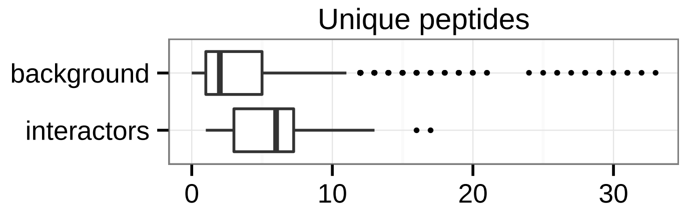

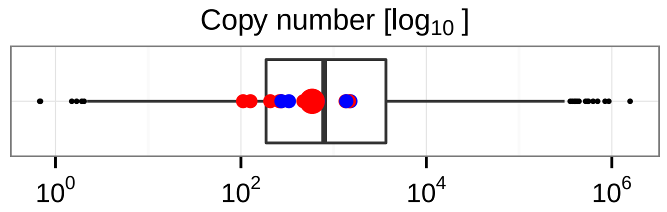

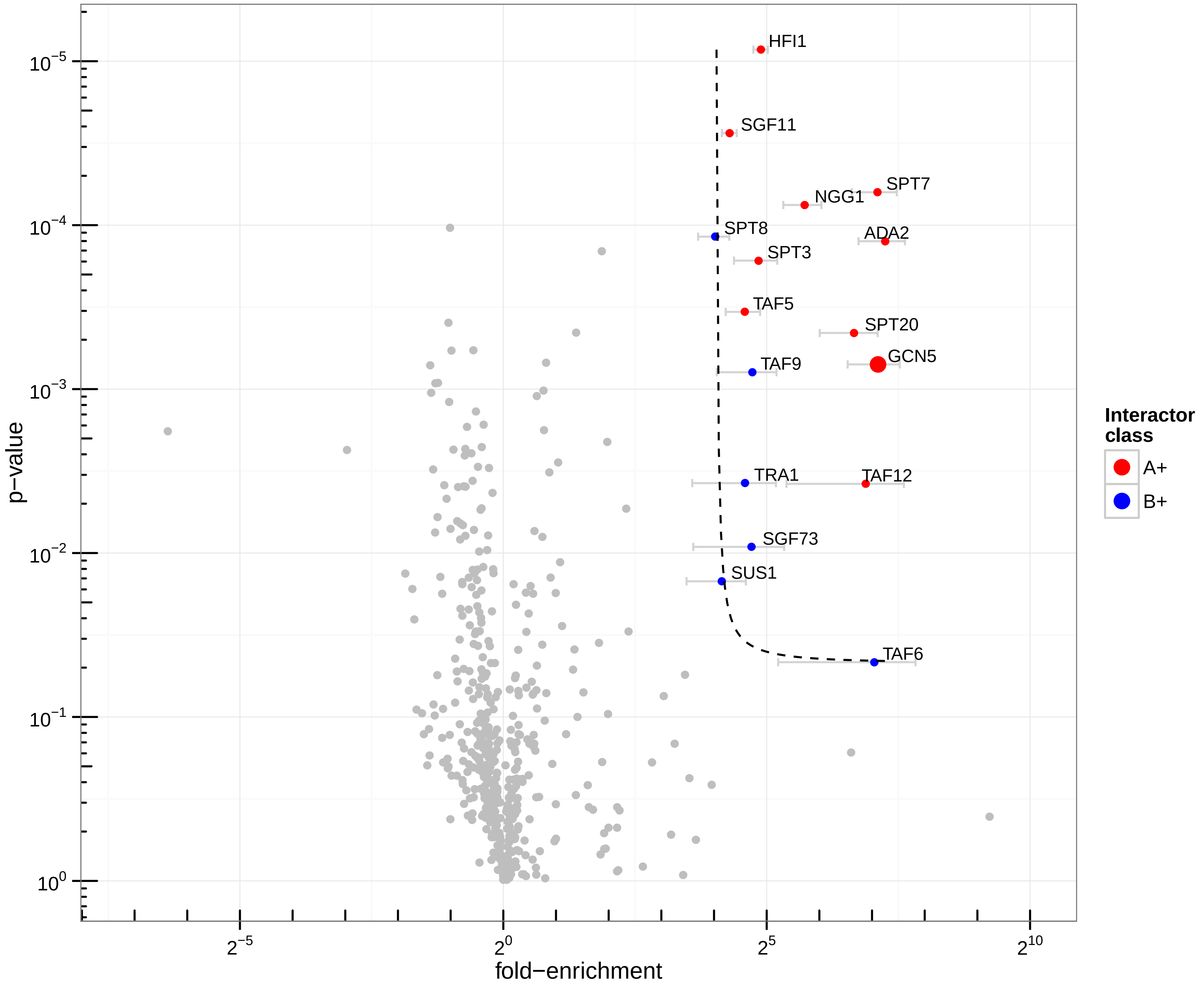

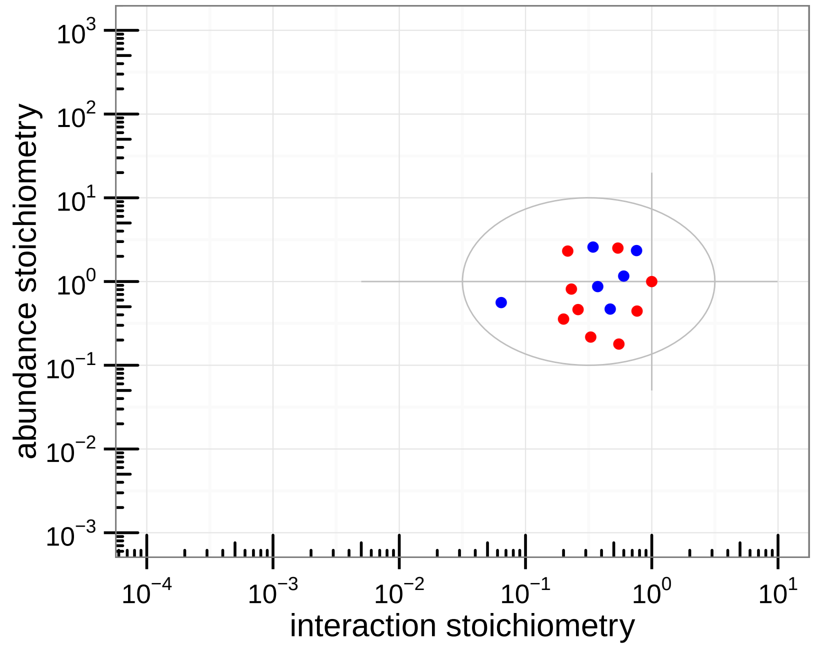

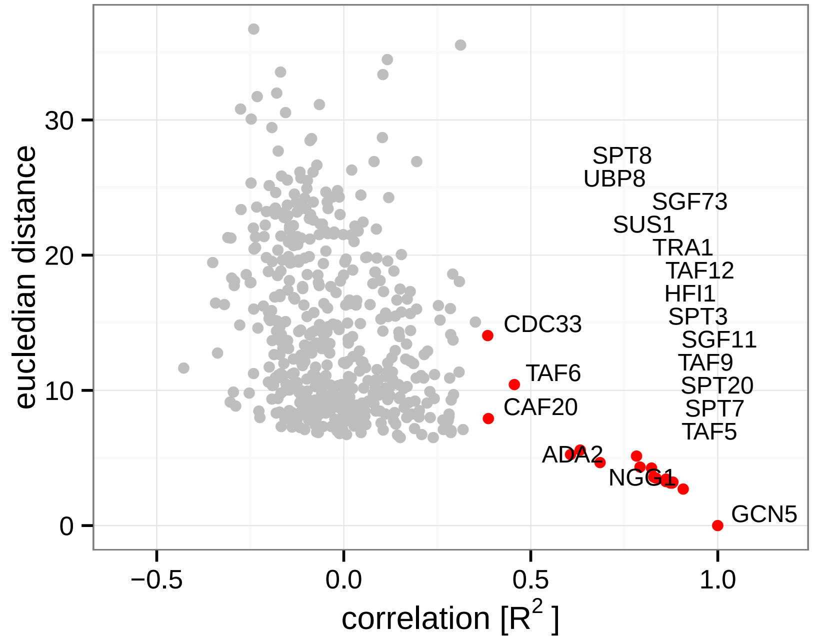

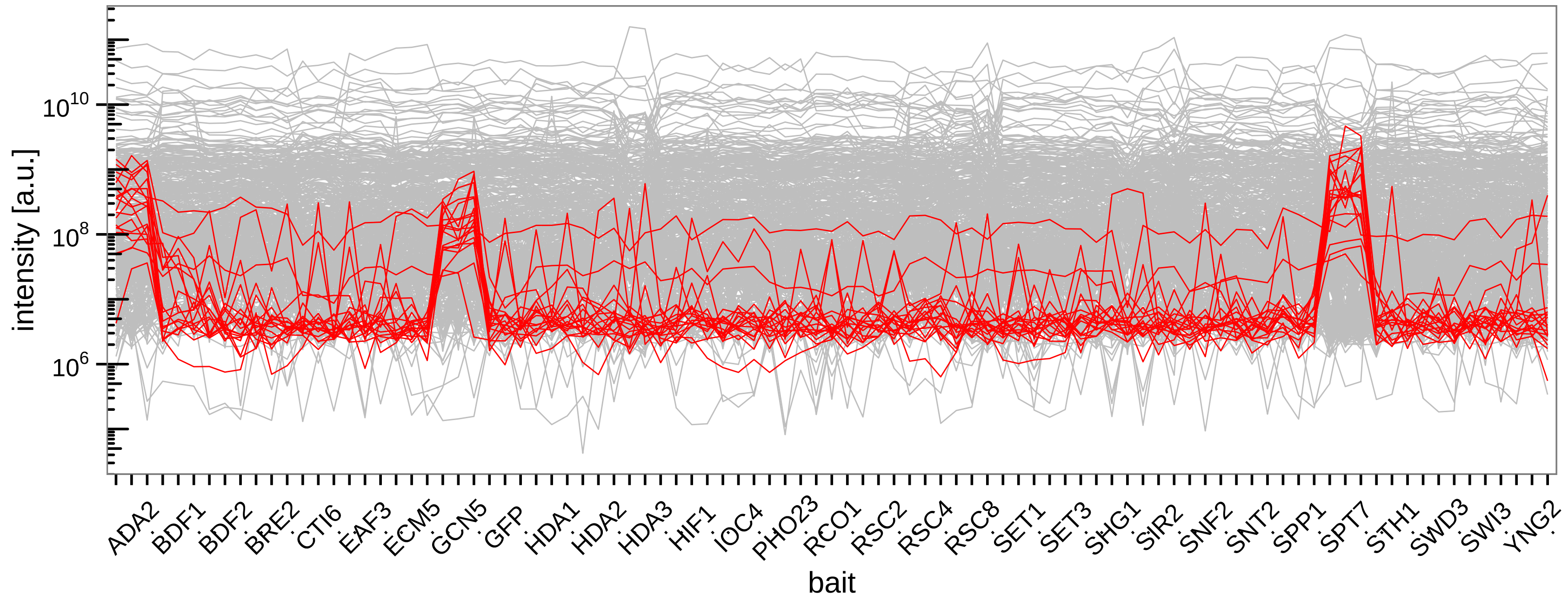

	ADA2	GCN5	HFI1	NGG1	SGF11	SGF73	SPT20	SPT3	SPT7	SPT8	SUS1	TAF12	TAF5	TAF6	TAF9	TRA1
significance	7.4	6.9	6.4	6.3	5.6	4.2	6.6	5.4	7.4	5.0	3.6	6.4	5.0	6.2	4.9	4.3
interactor class	A+	A+	A+	A+	A+	B+	A+	A+	A+	B+	B+	A+	A+	B+	B+	B+

**bait**	GFP
**experiment**	14 minutes

**interactors**	1
**recovery**	NaN %

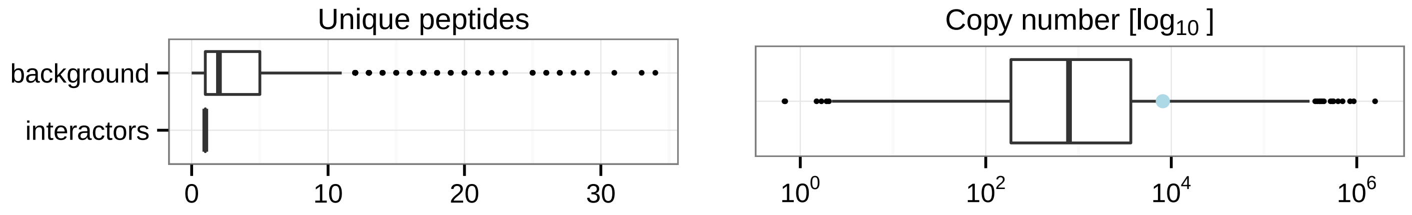

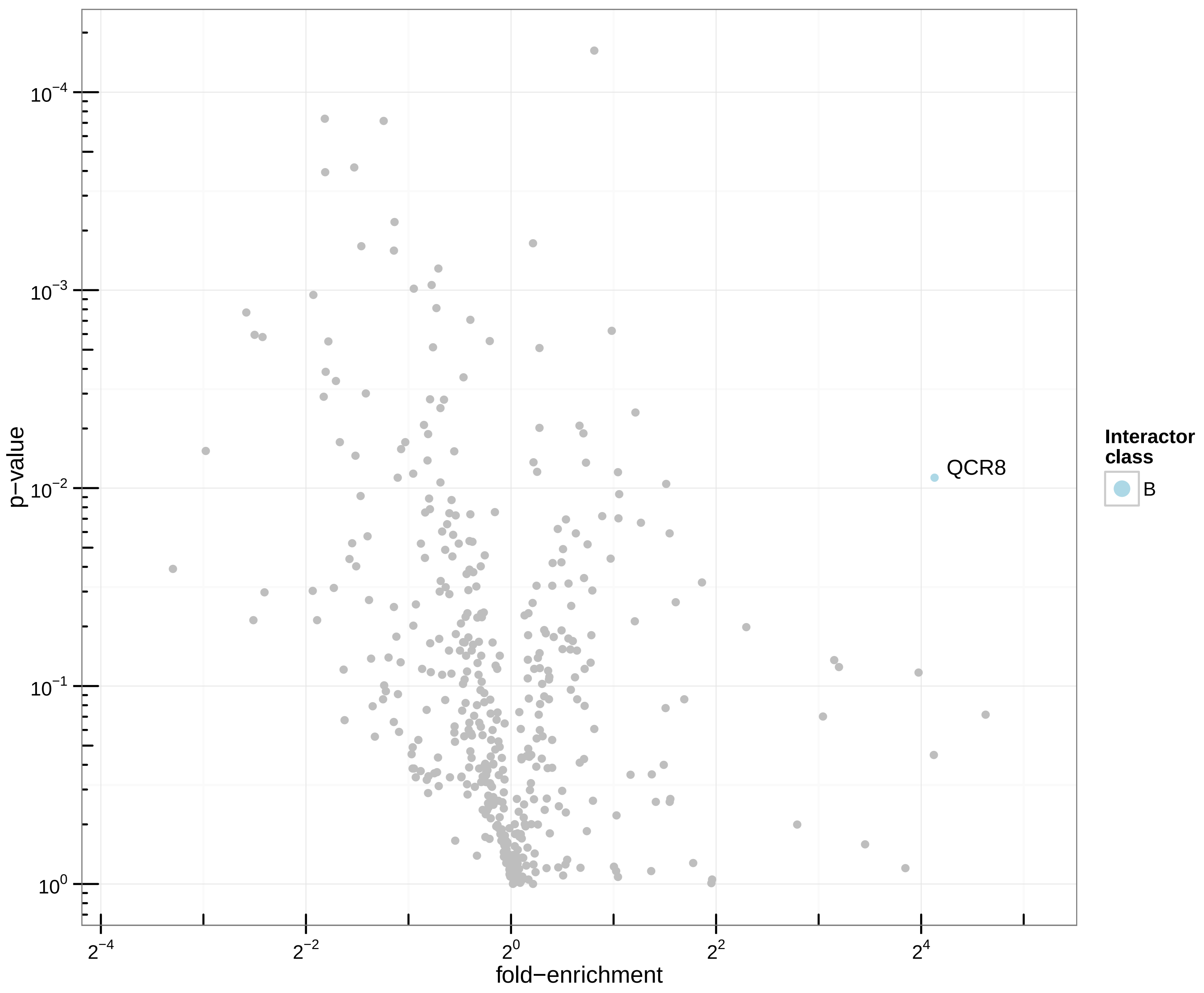

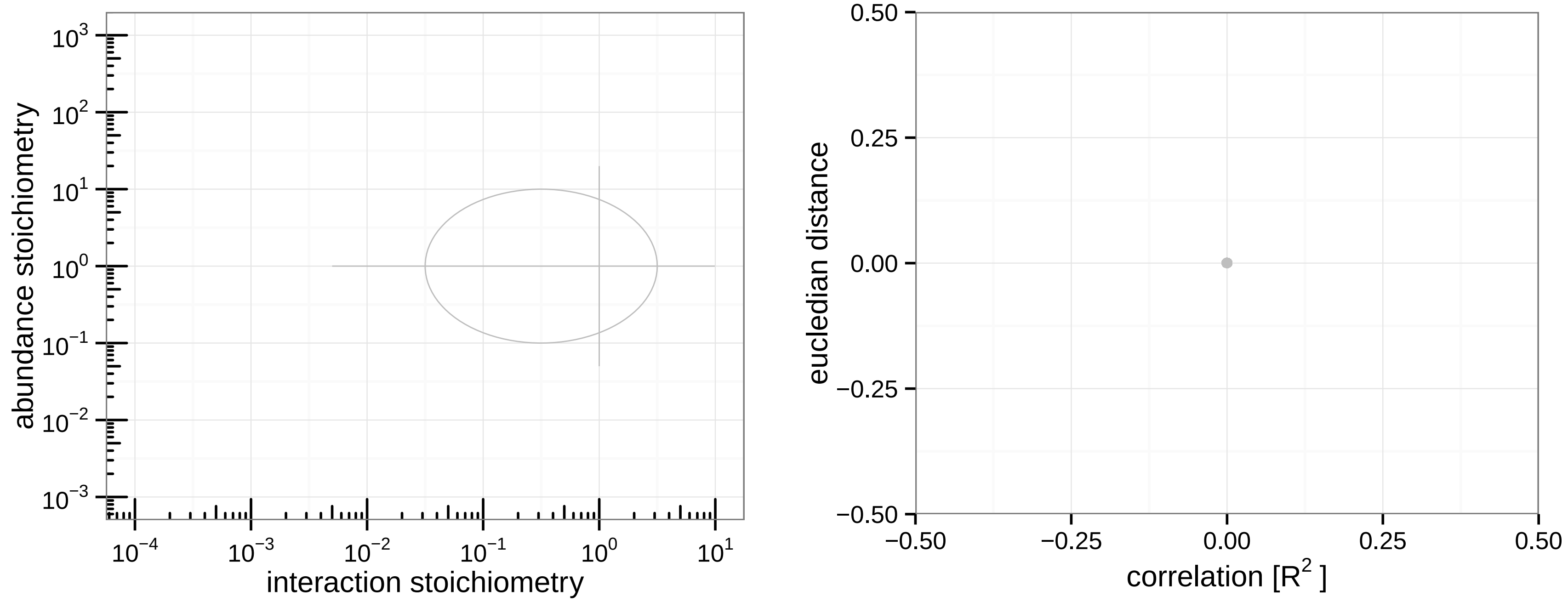

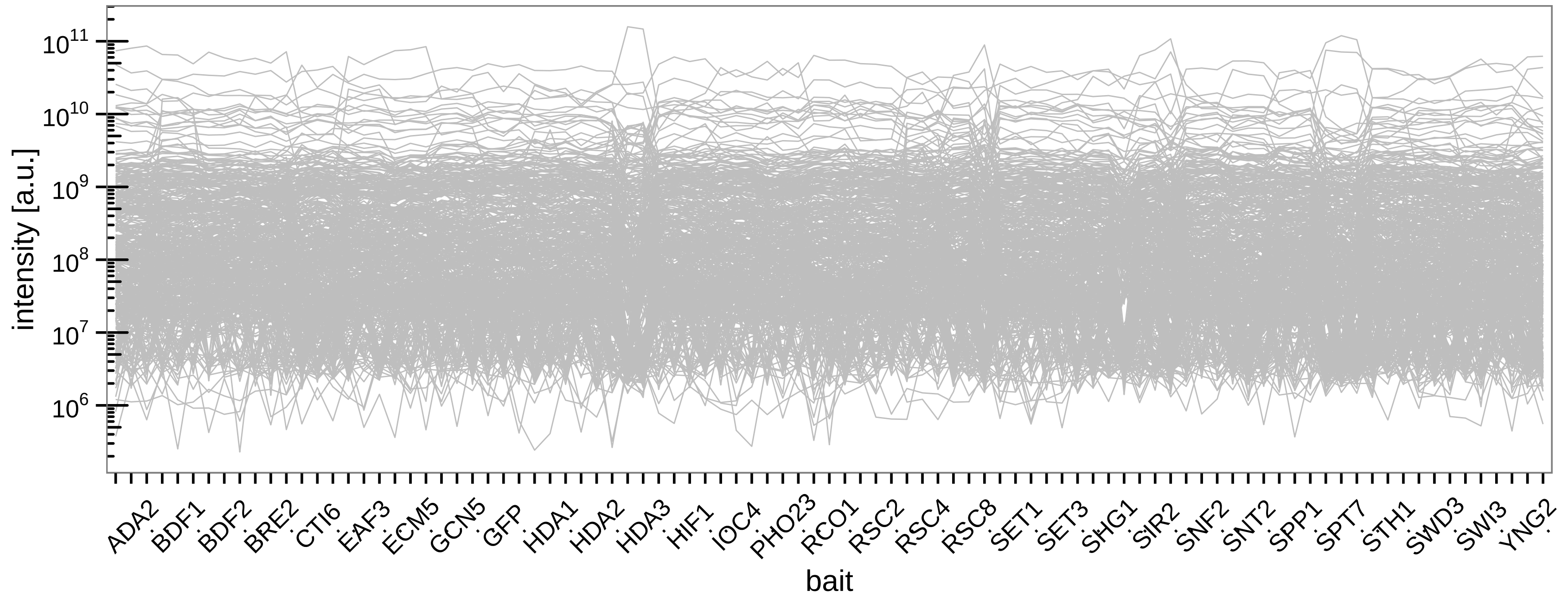

significance

interactor class

**bait**	HDA1
**experiment**	14 minutes

**interactors**	3
**recovery**	100 %

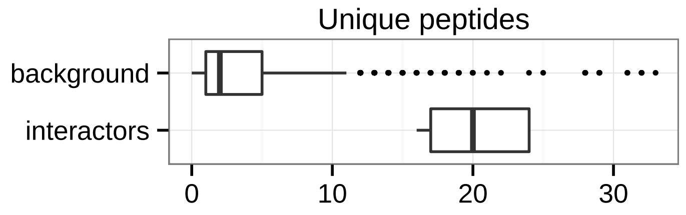

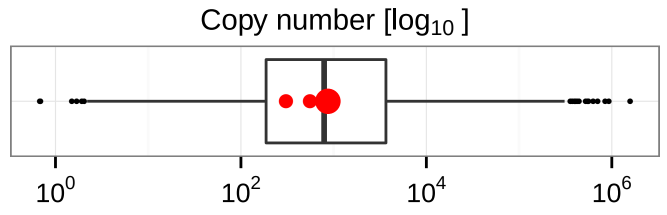

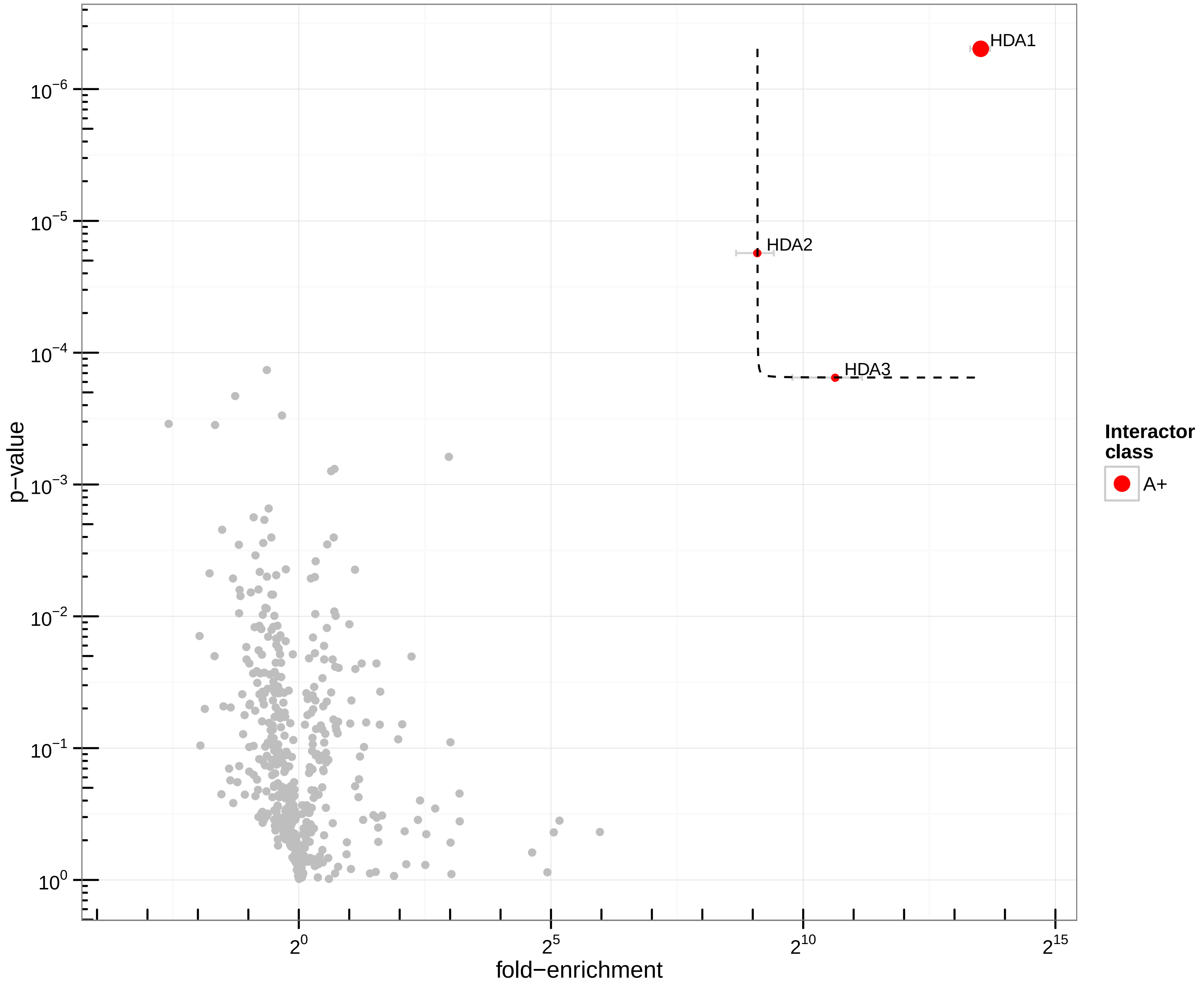

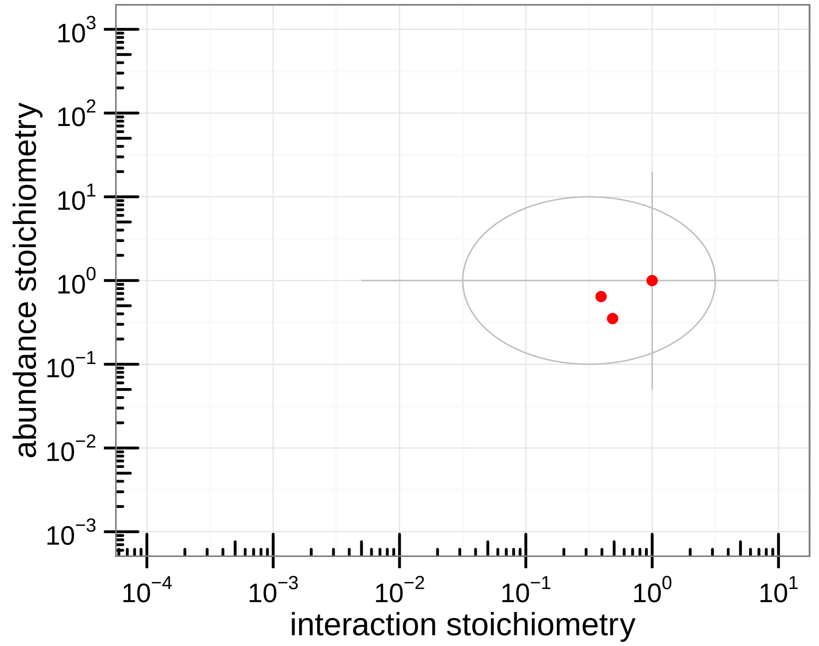

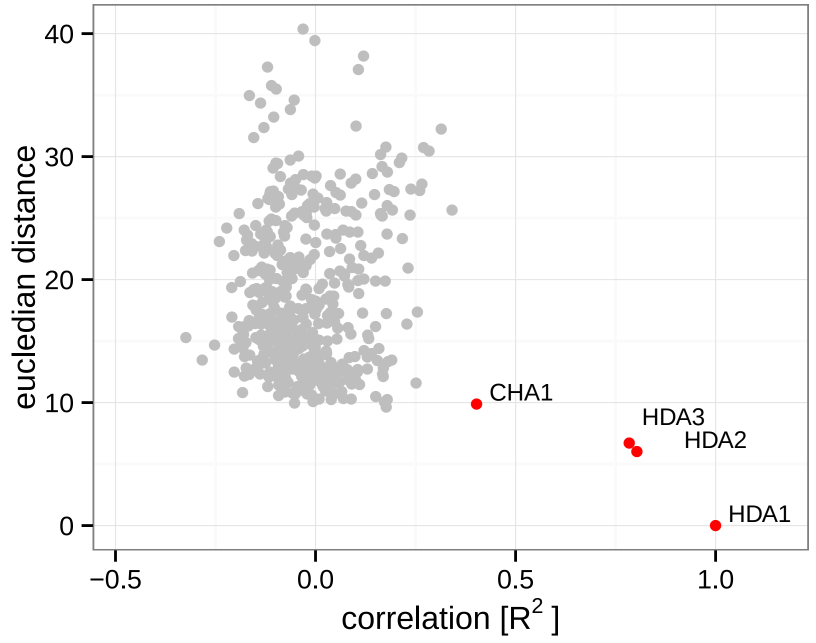

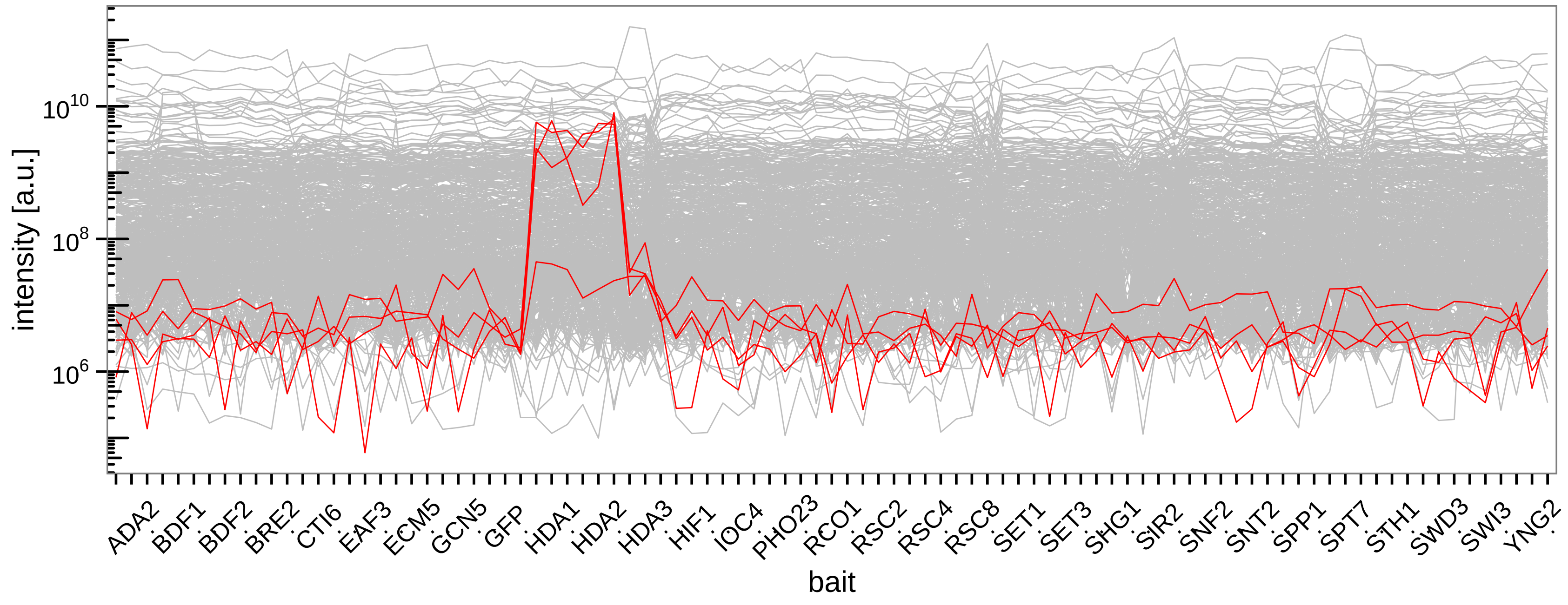

	HDA1	HDA2	HDA3
significance	14.0	9.4	10.0
interactor class	A+	A+	A+

**bait**	HDA2
**experiment**	14 minutes

**interactors**	3
**recovery**	100 %

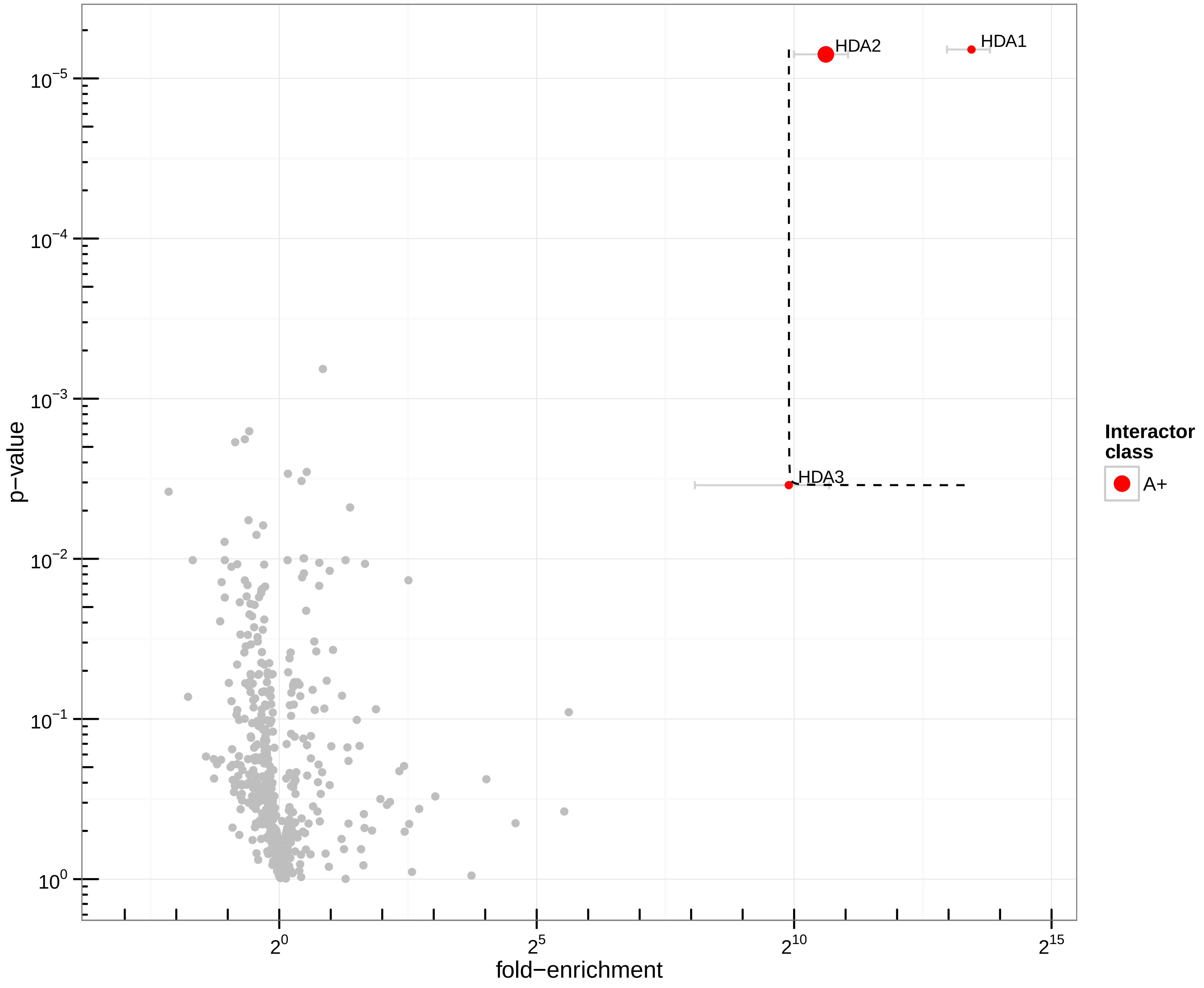

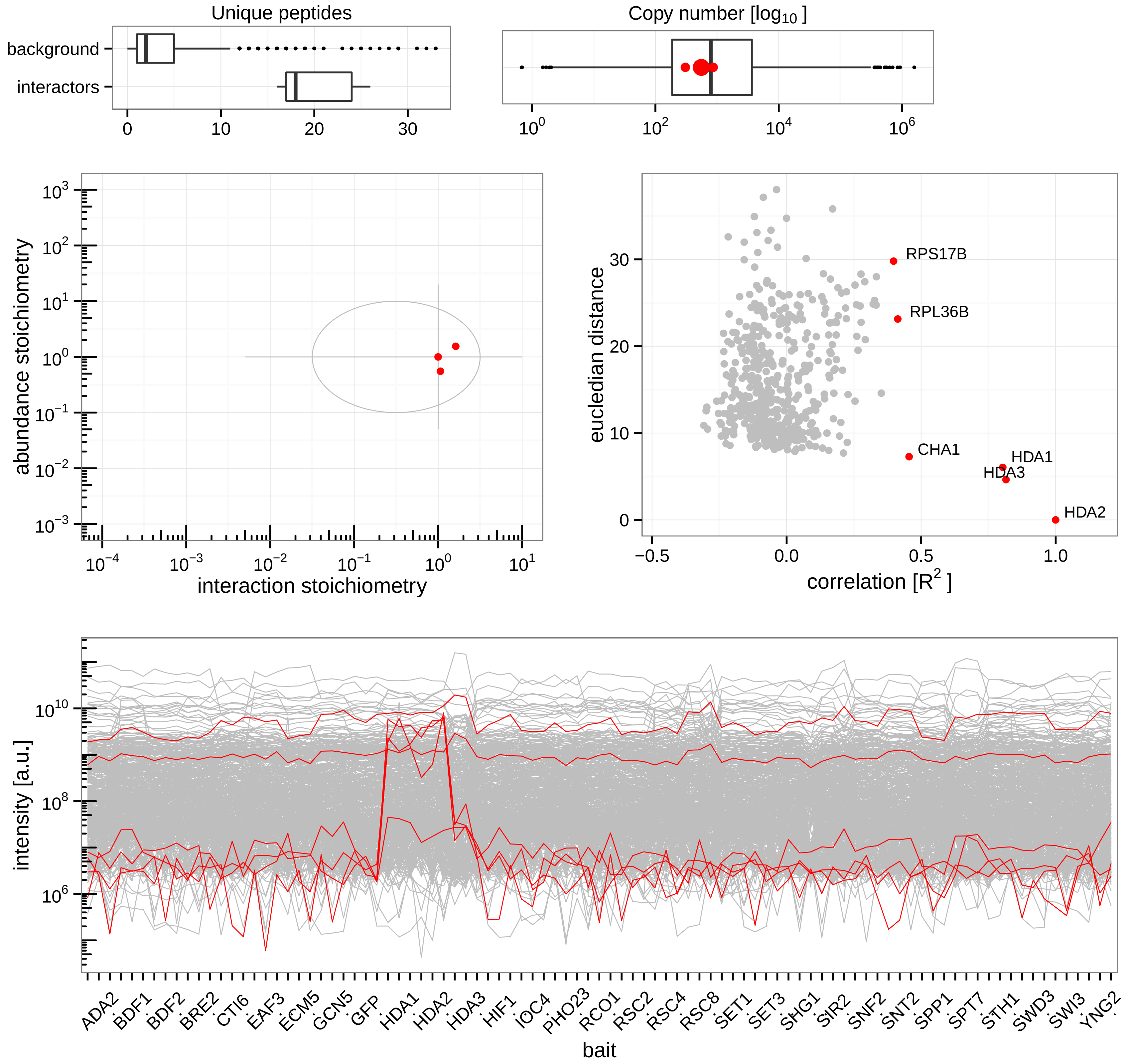

	HDA1	HDA2	HDA3
significance	13.0	11.0	9.2
interactor class	A+	A+	A+

bait	HDA3
experiment	14 minutes

interactors	1
recovery	50 %

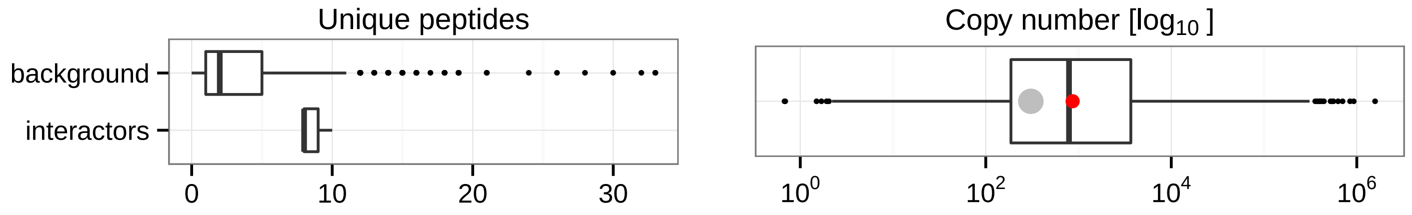

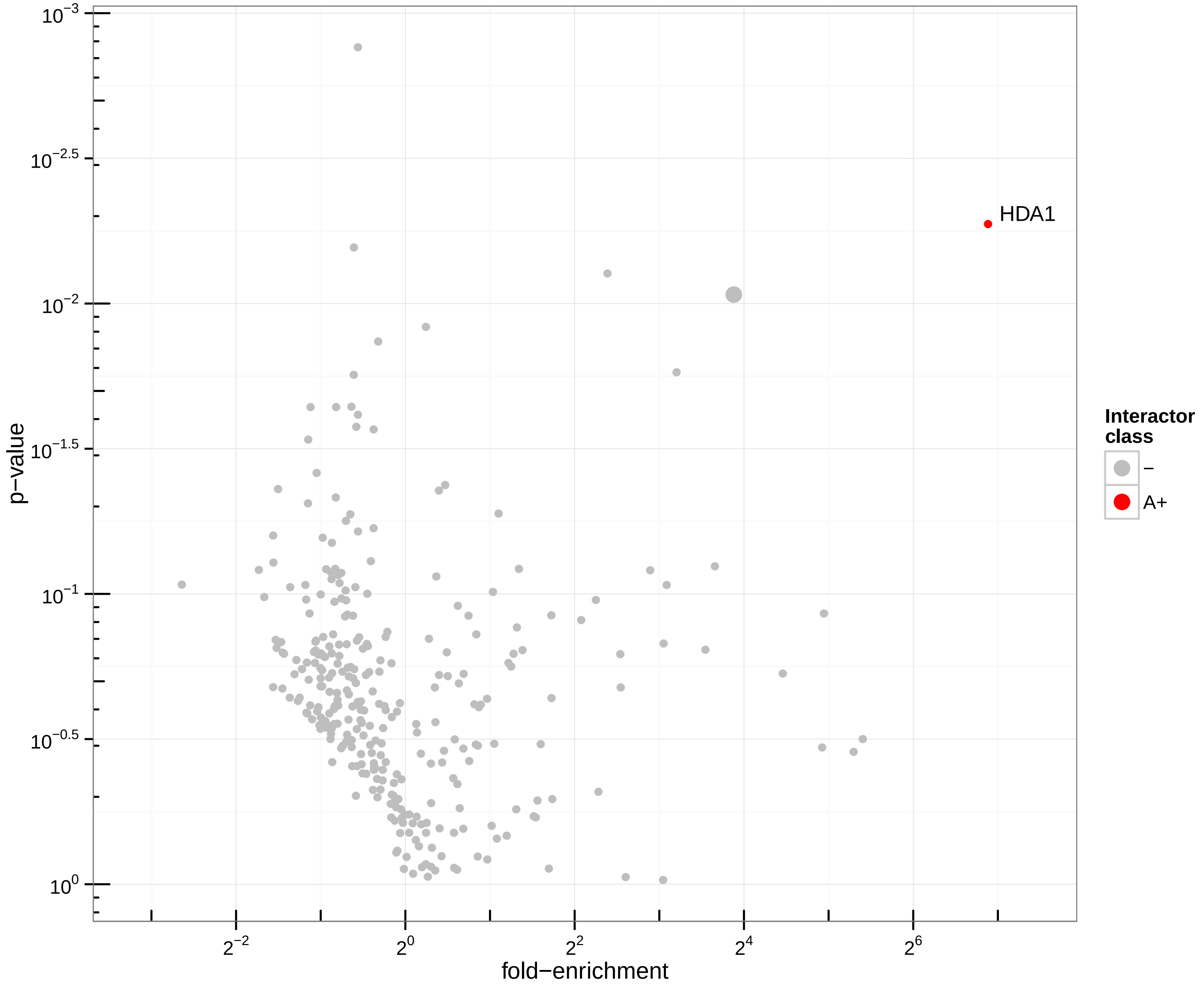

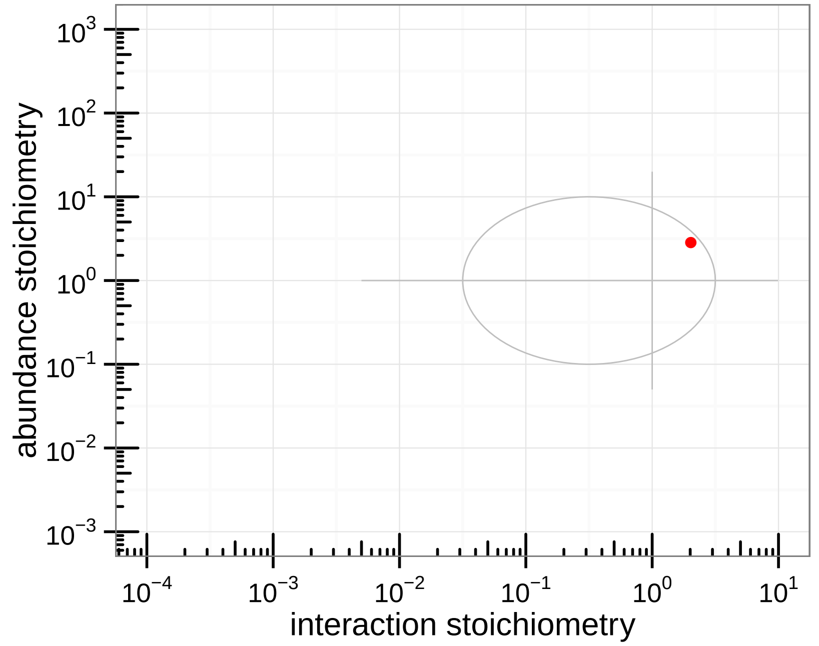

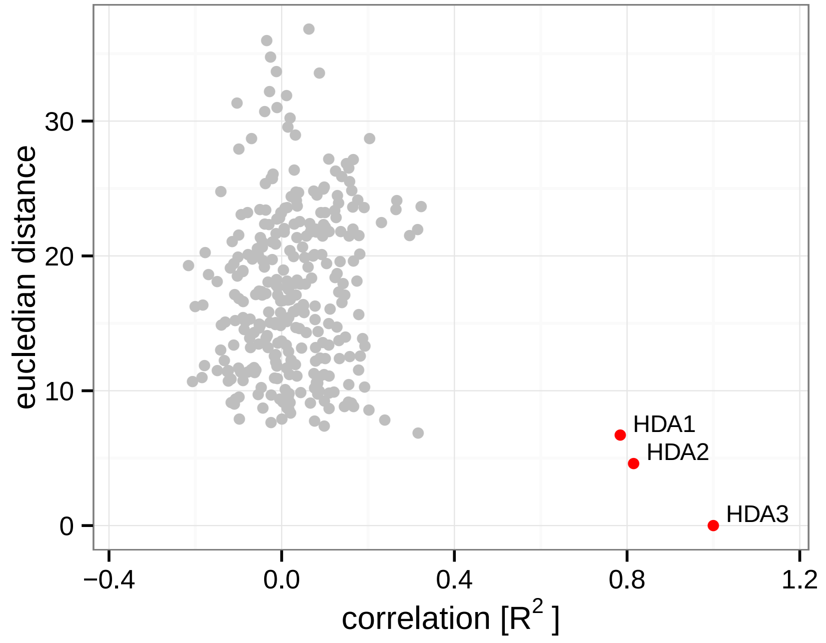

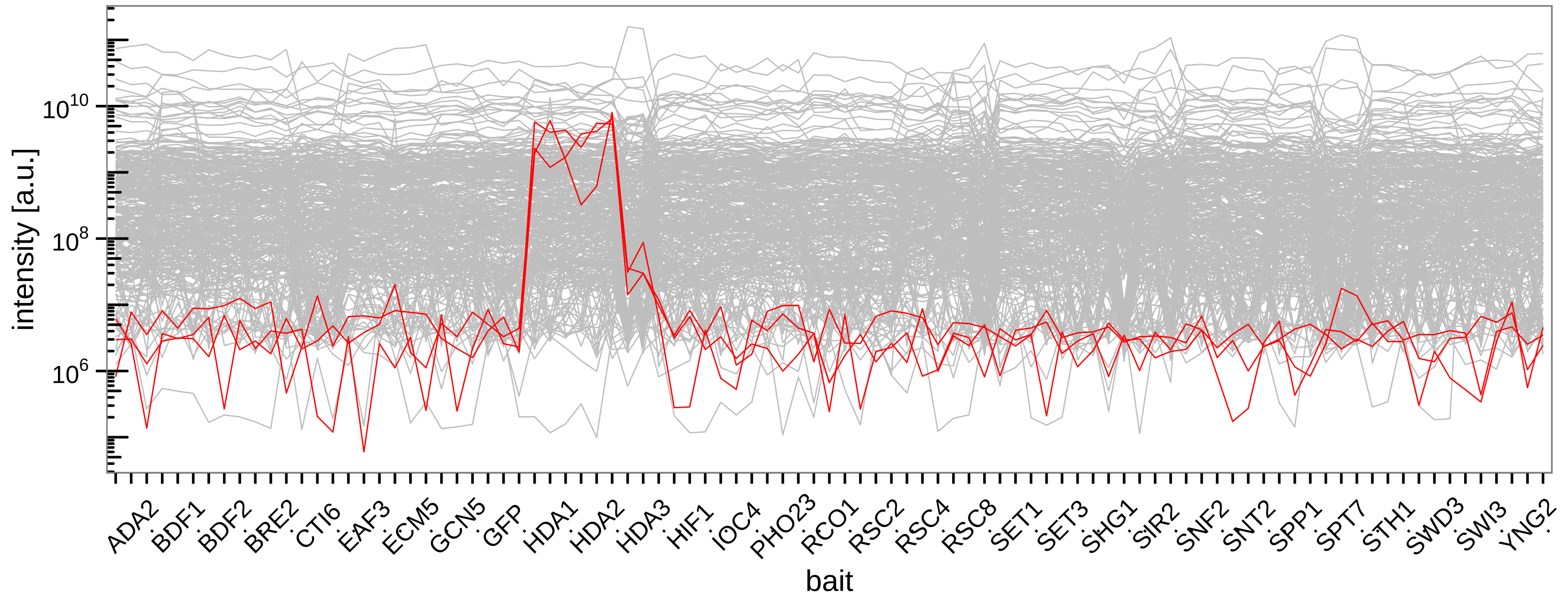

	HDA1
significance	6.3
interactor class	A+

**bait**	HIF1
**experiment**	14 minutes

**interactors**	3
**recovery**	33 %

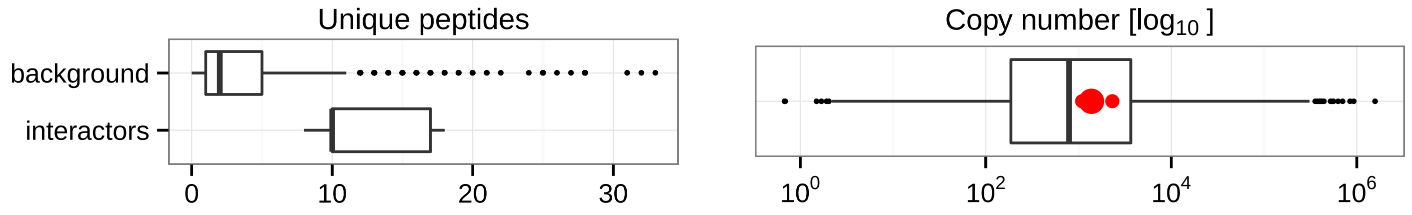

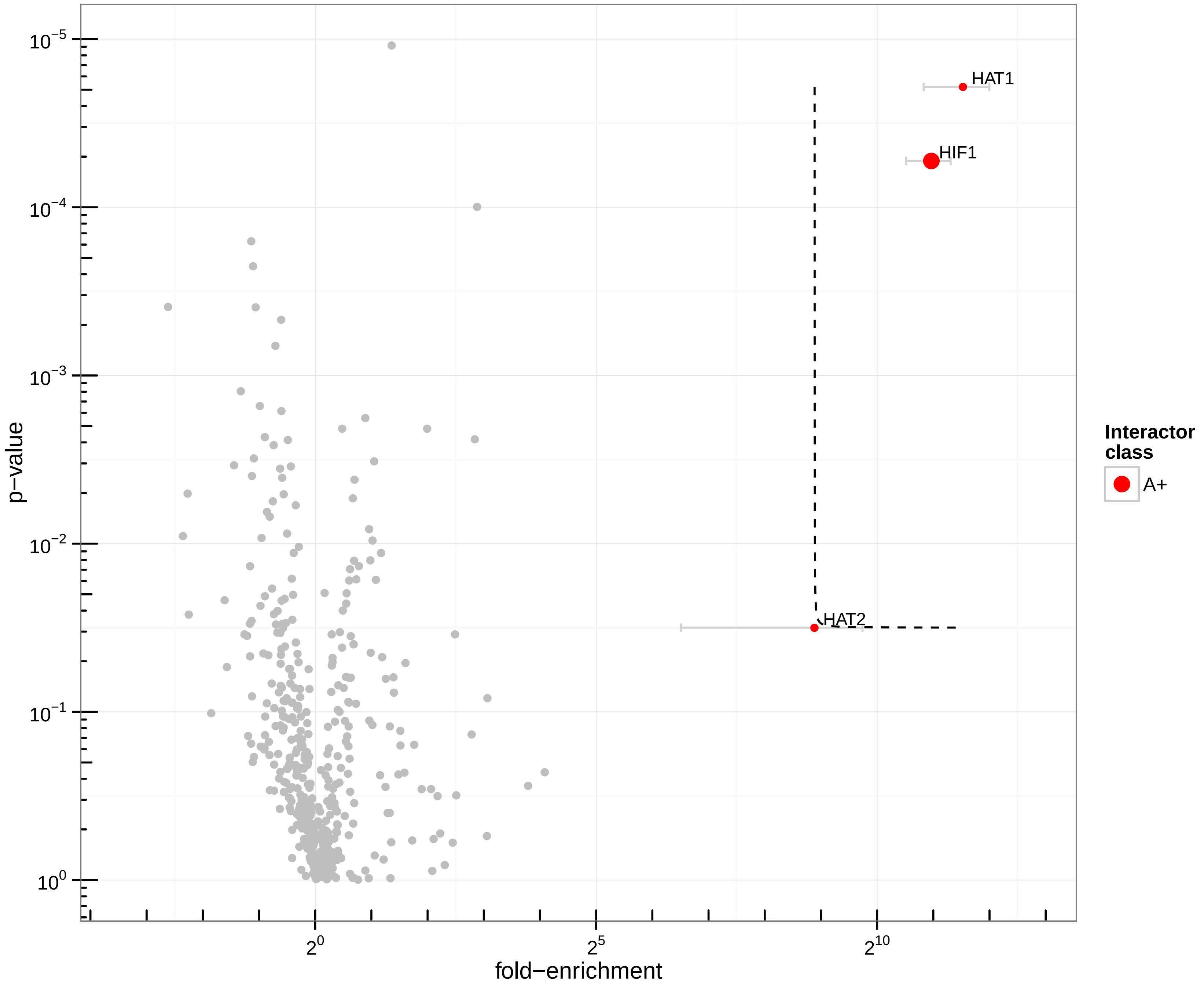

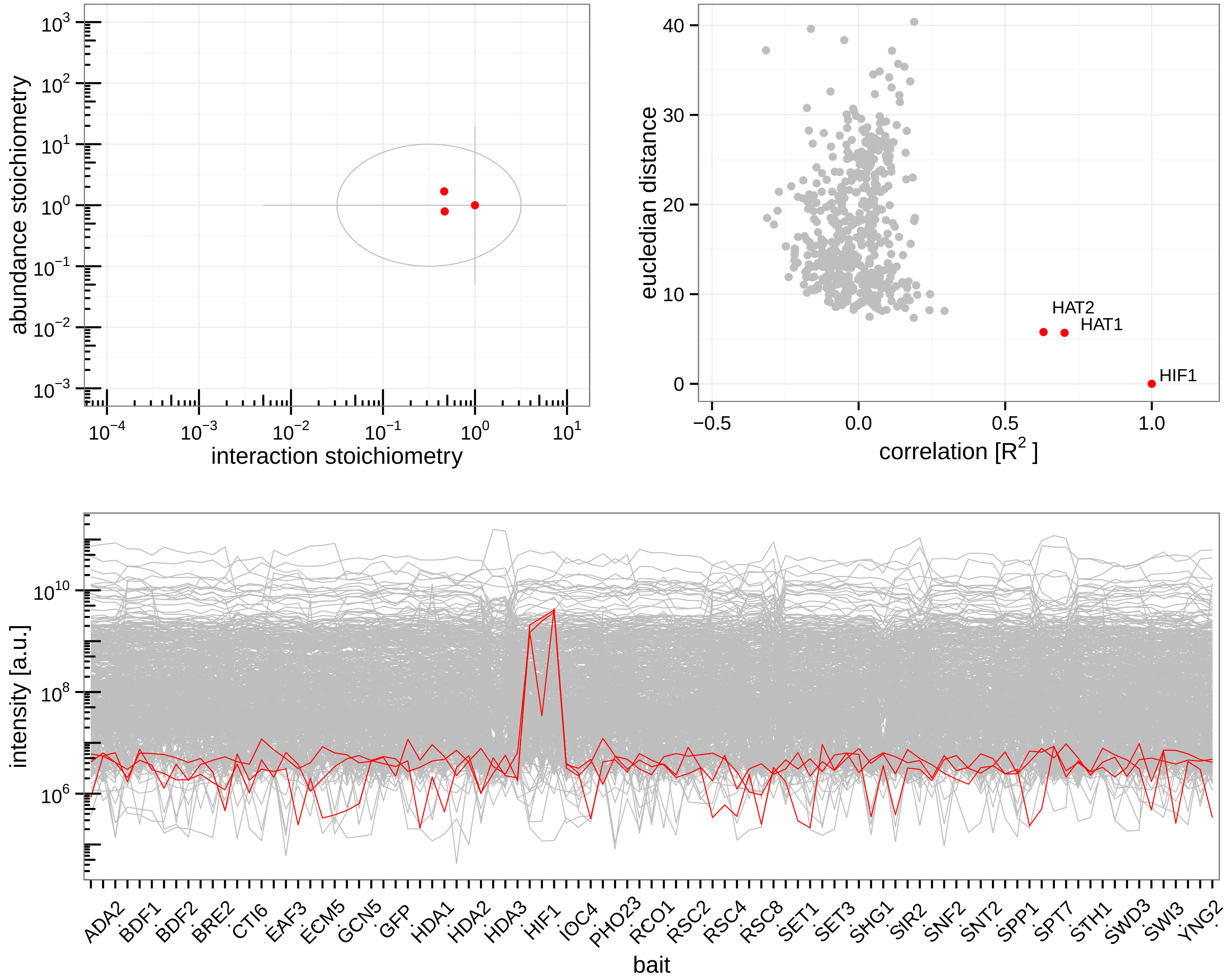

	HAT1	HAT2	HIF1
significance	12	8	11
interactor class	A+	A+	A+

**bait**	IOC4
**experiment**	14 minutes

**interactors**	0
**recovery**	0 %

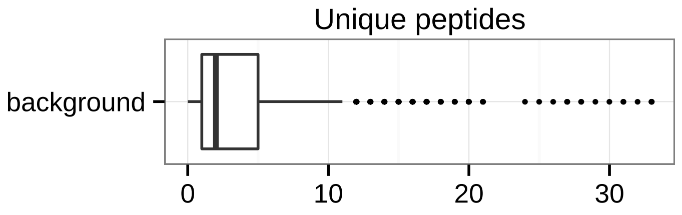

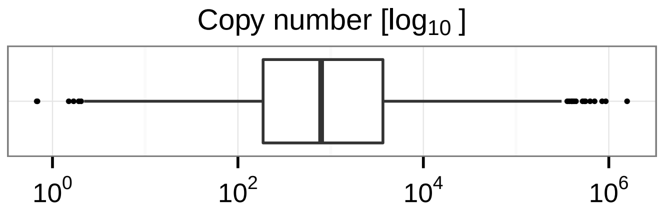

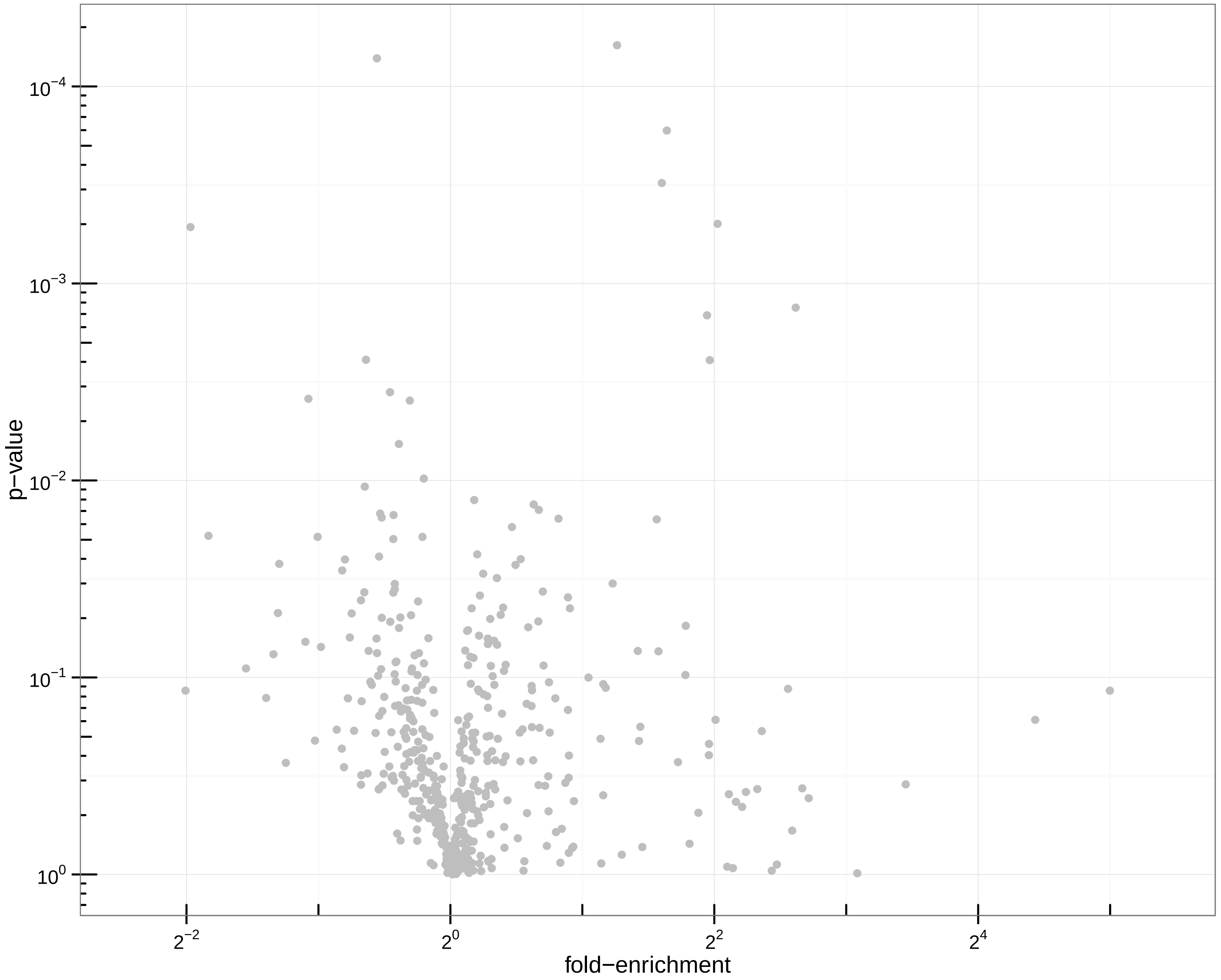

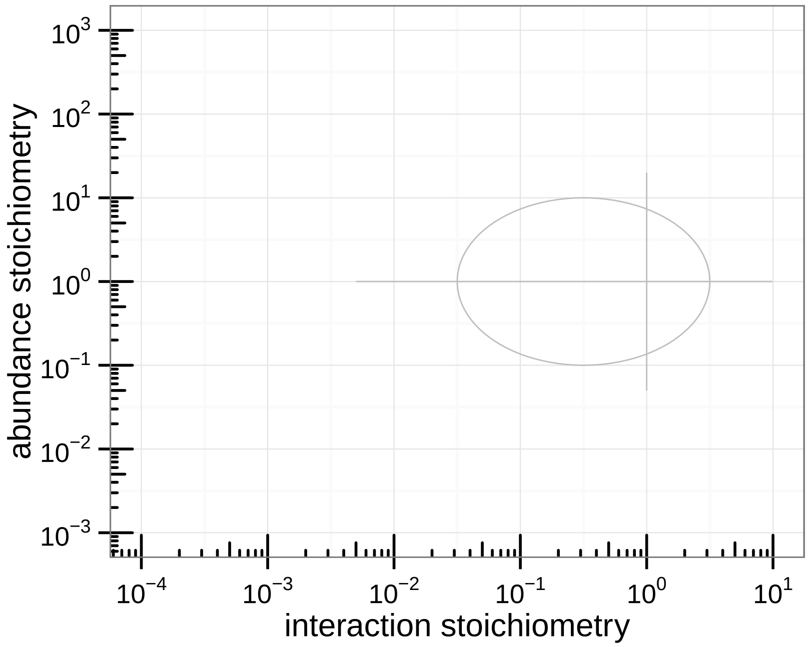

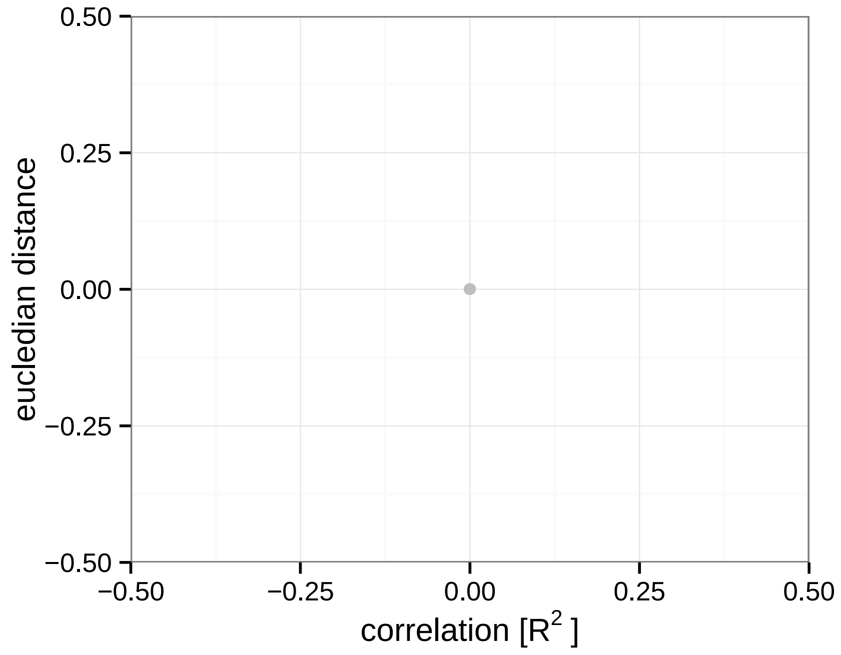

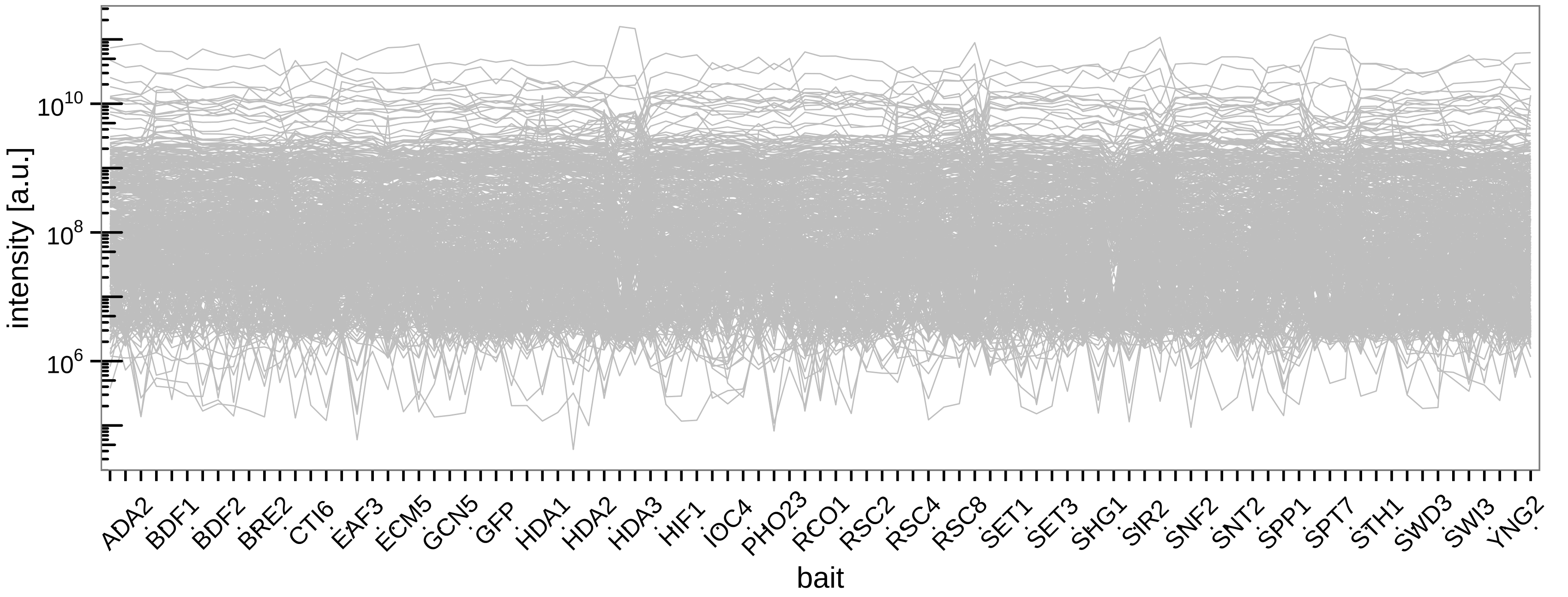

significance

interactor class

**bait**	PHO23
**experiment**	14 minutes

**interactors**	18
**recovery**	38 %

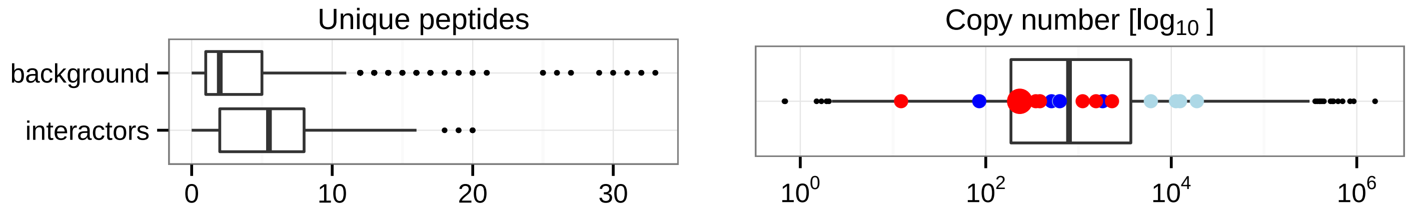

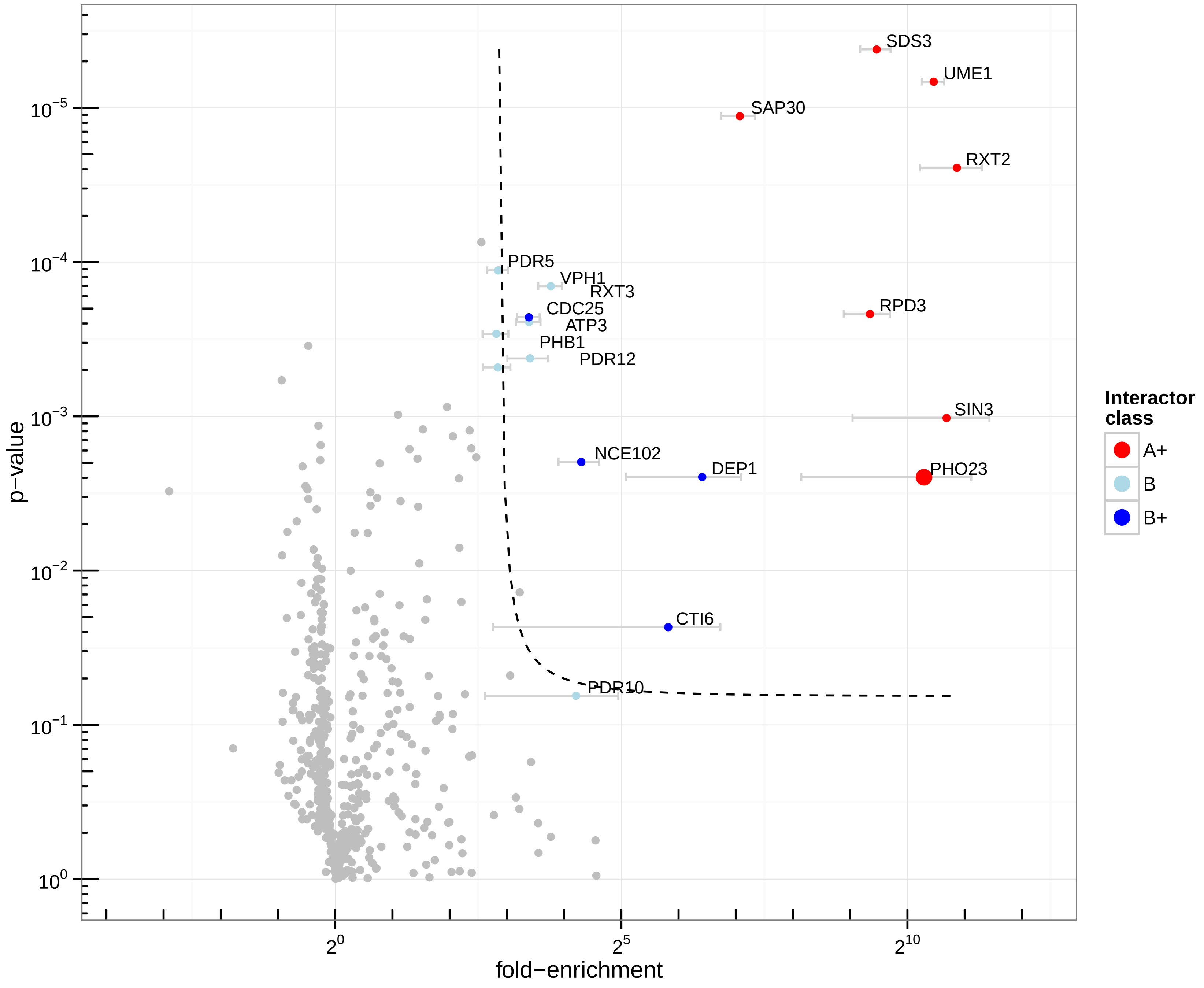

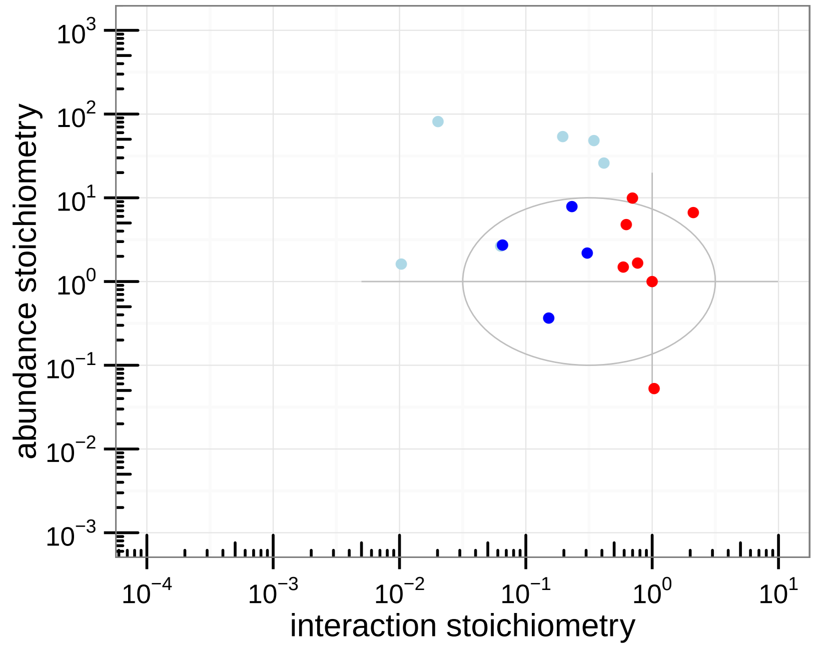

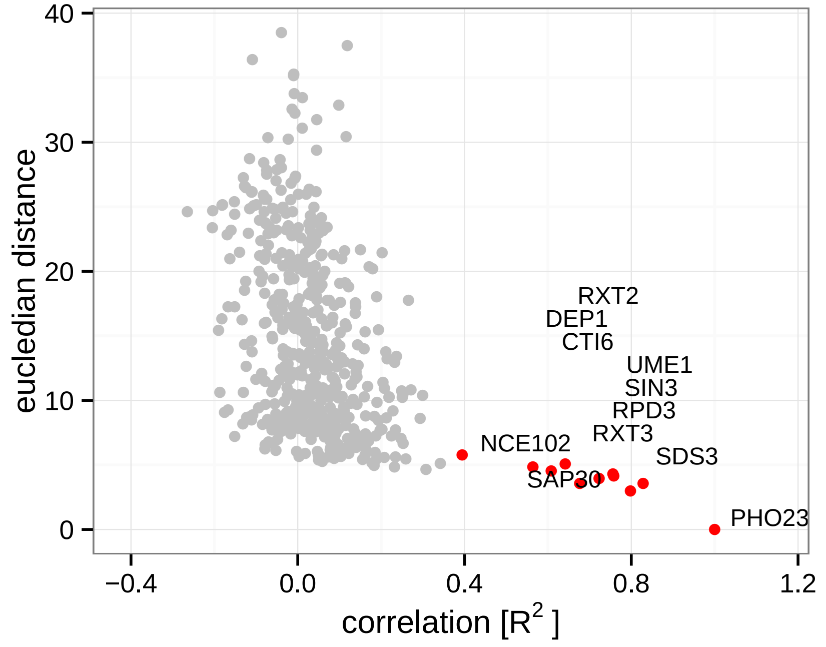

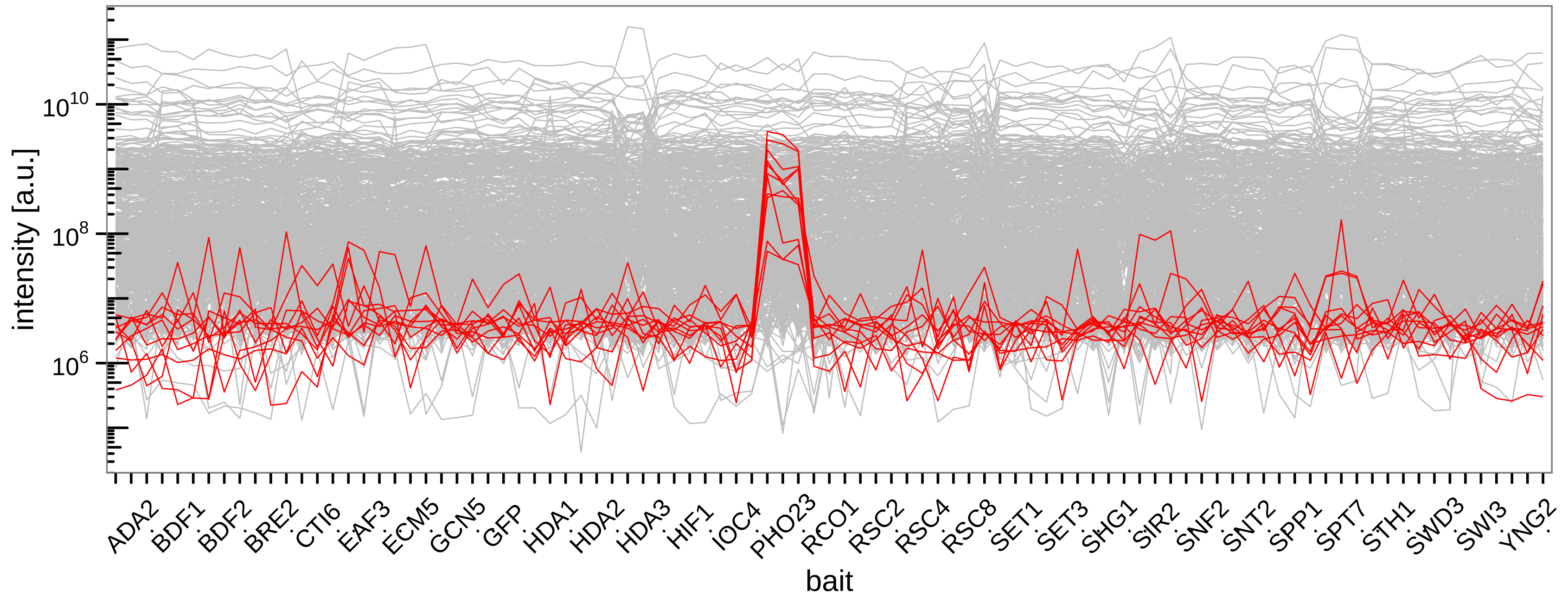

	CTI6	DEP1	NCE102	PHO23	RPD3	RXT2	RXT3	SAP30	SDS3	SIN3	UME1
significance	5.1	6.0	4.3	9.6	9.1	11.0	4.4	7.8	10.0	10.0	11.0
interactor class	B+	B+	B+	A+	A+	A+	B+	A+	A+	A+	A+

**bait**	RCO1
**experiment**	14 minutes

**interactors**	0
**recovery**	0 %

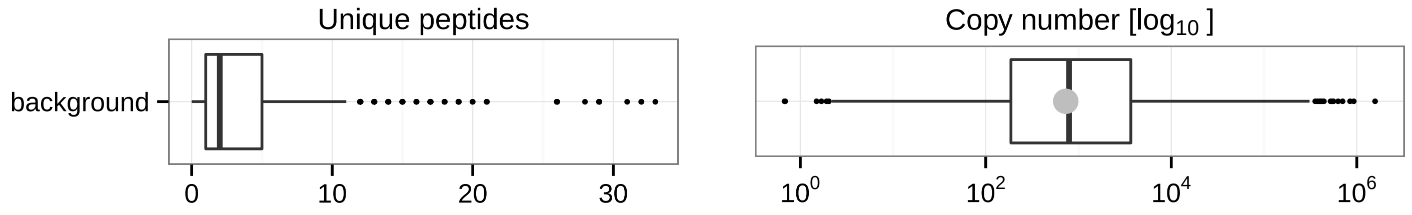

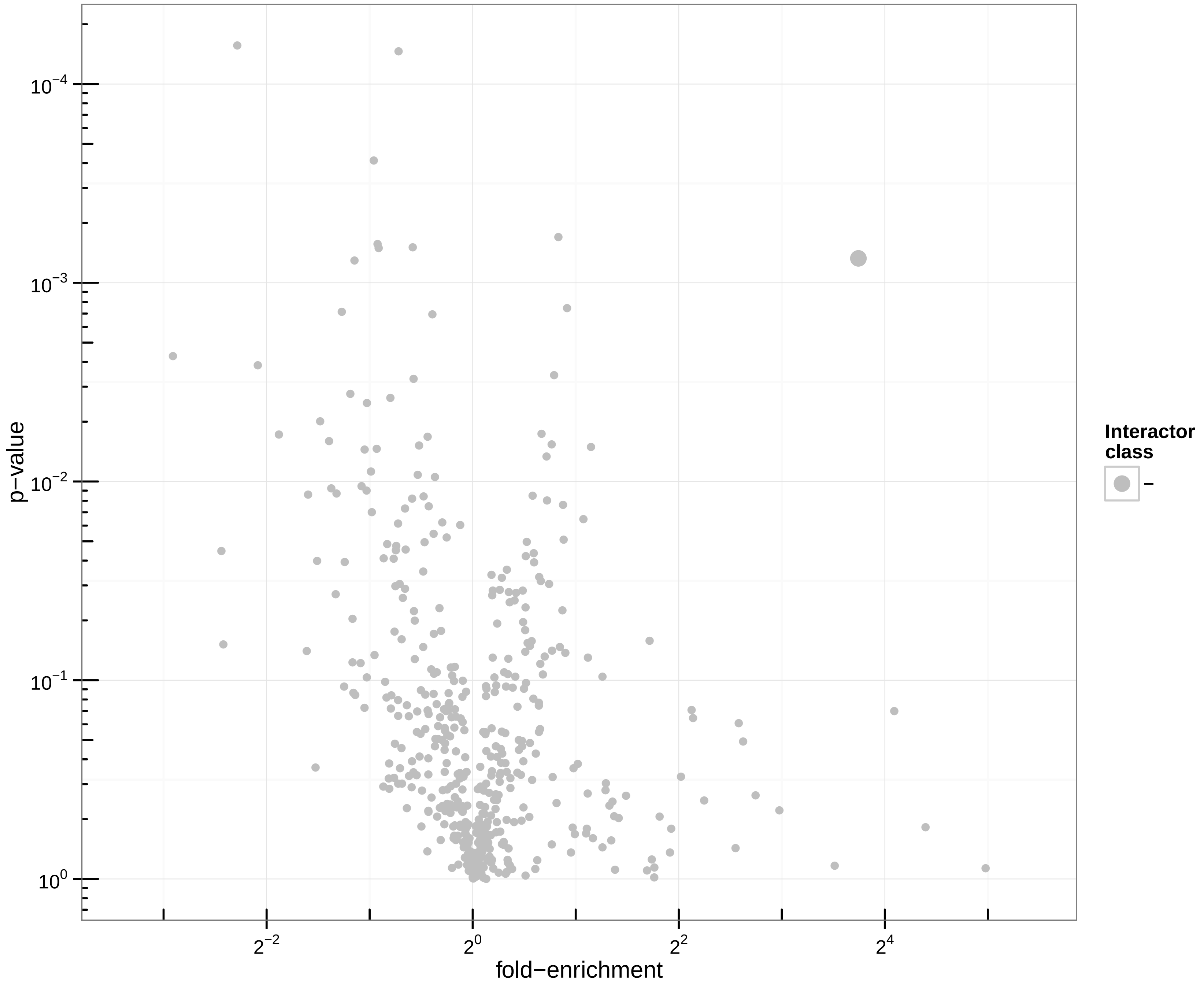

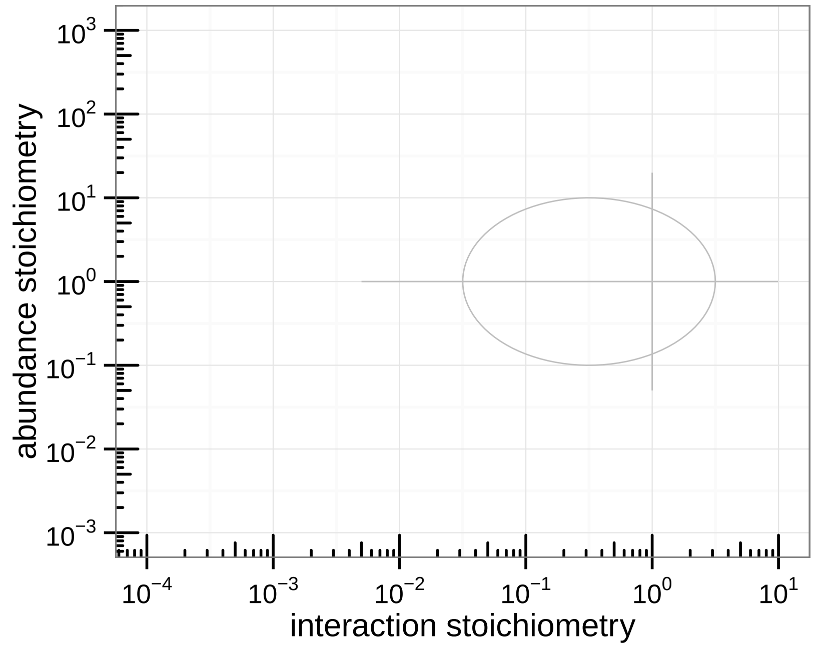

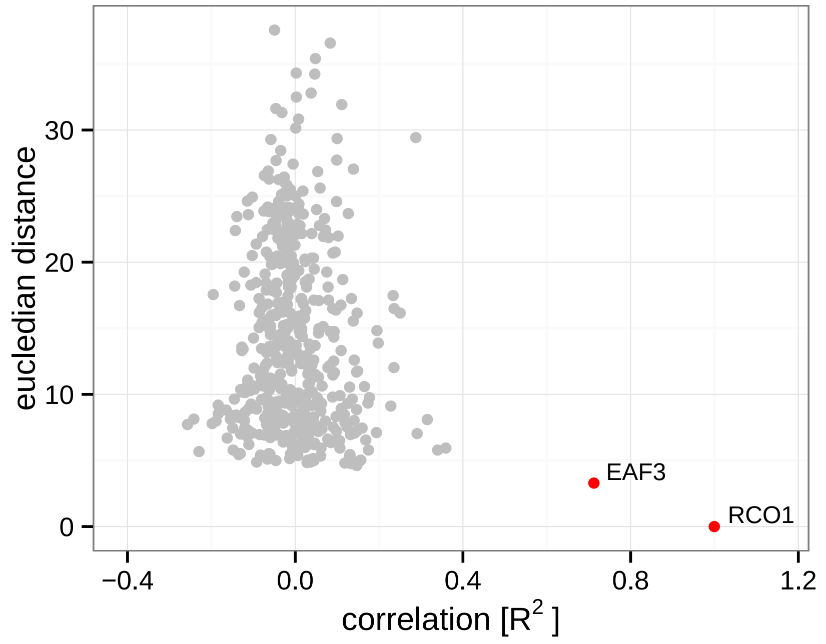

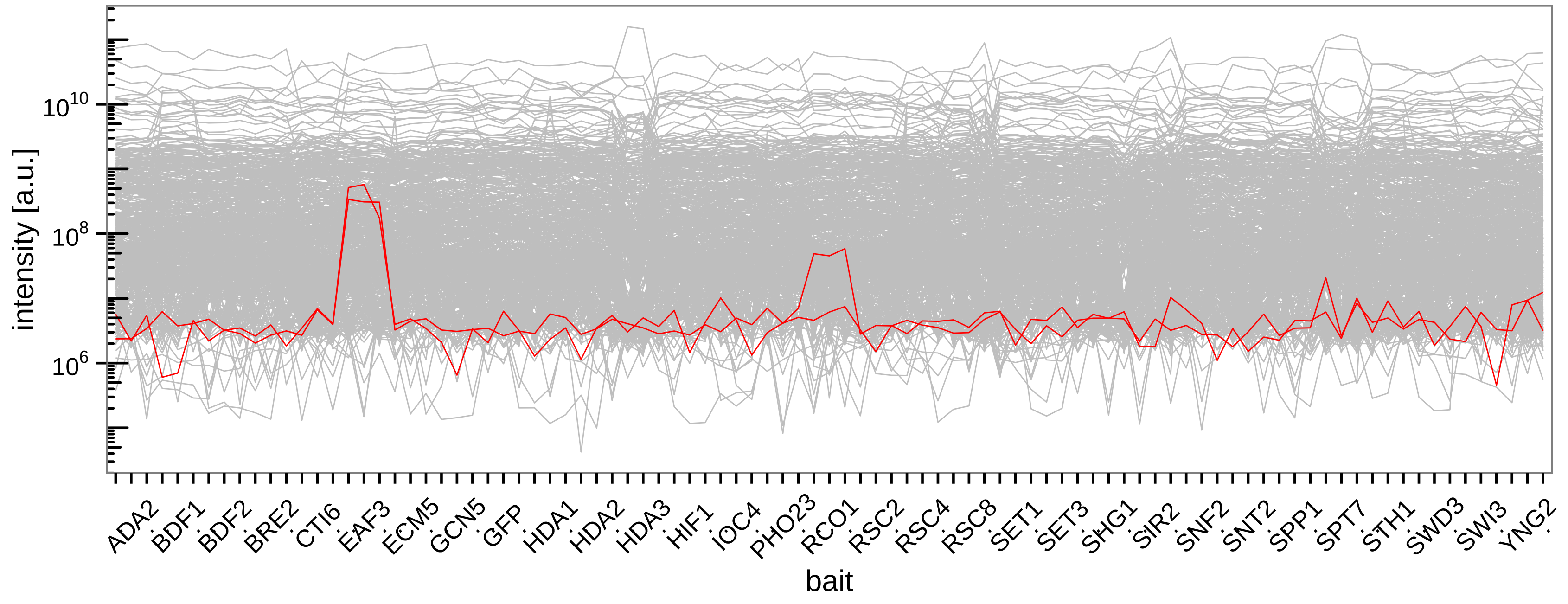

significance

interactor class

bait	RSC2
experiment	14 minutes

interactors	0
recovery	0 %

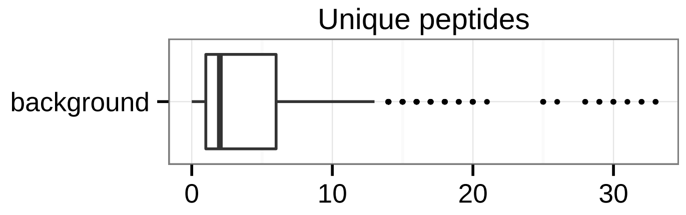

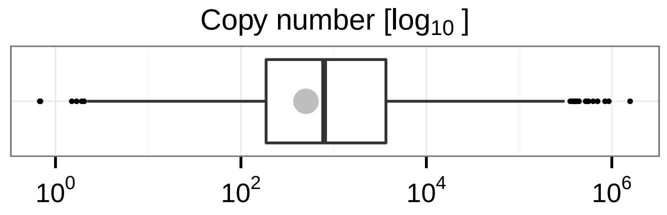

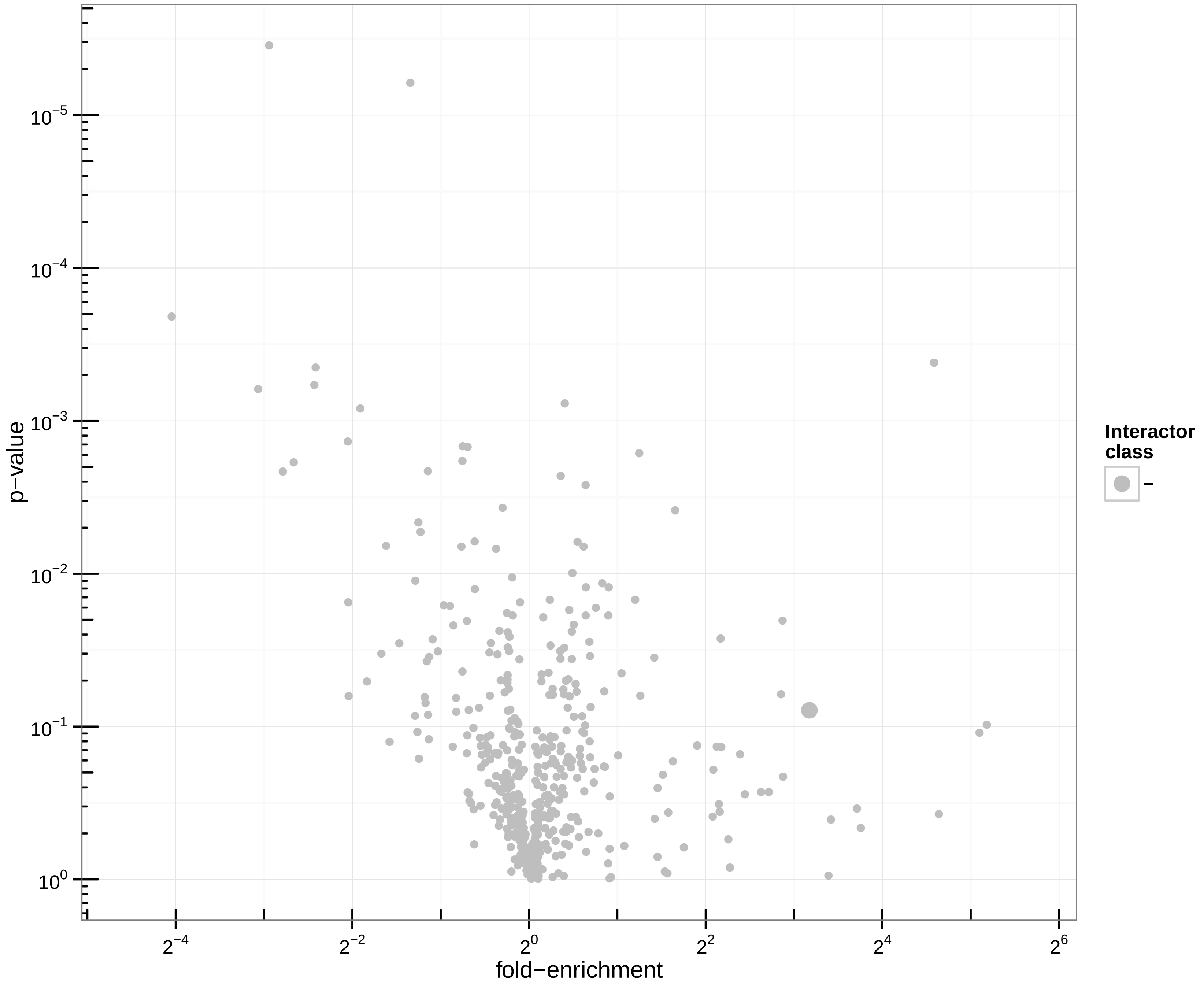

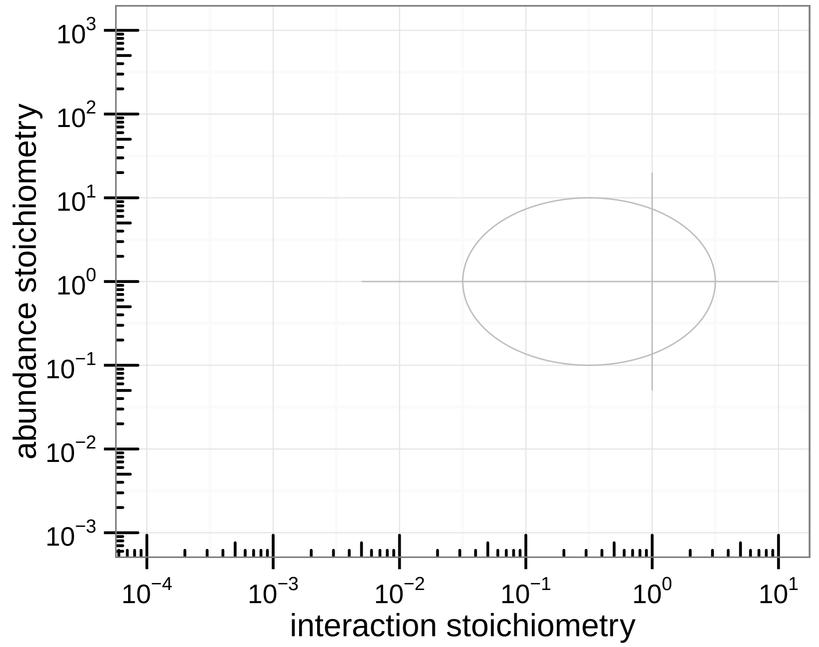

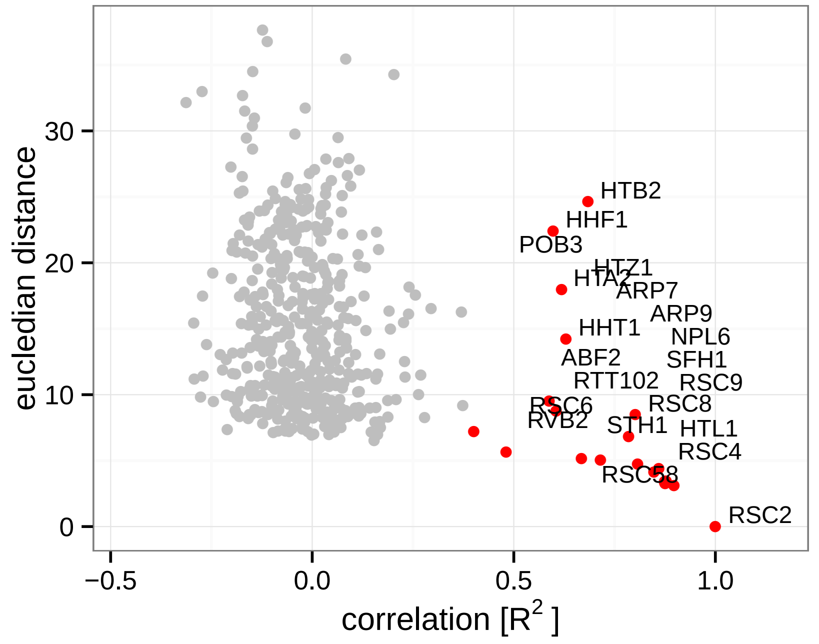

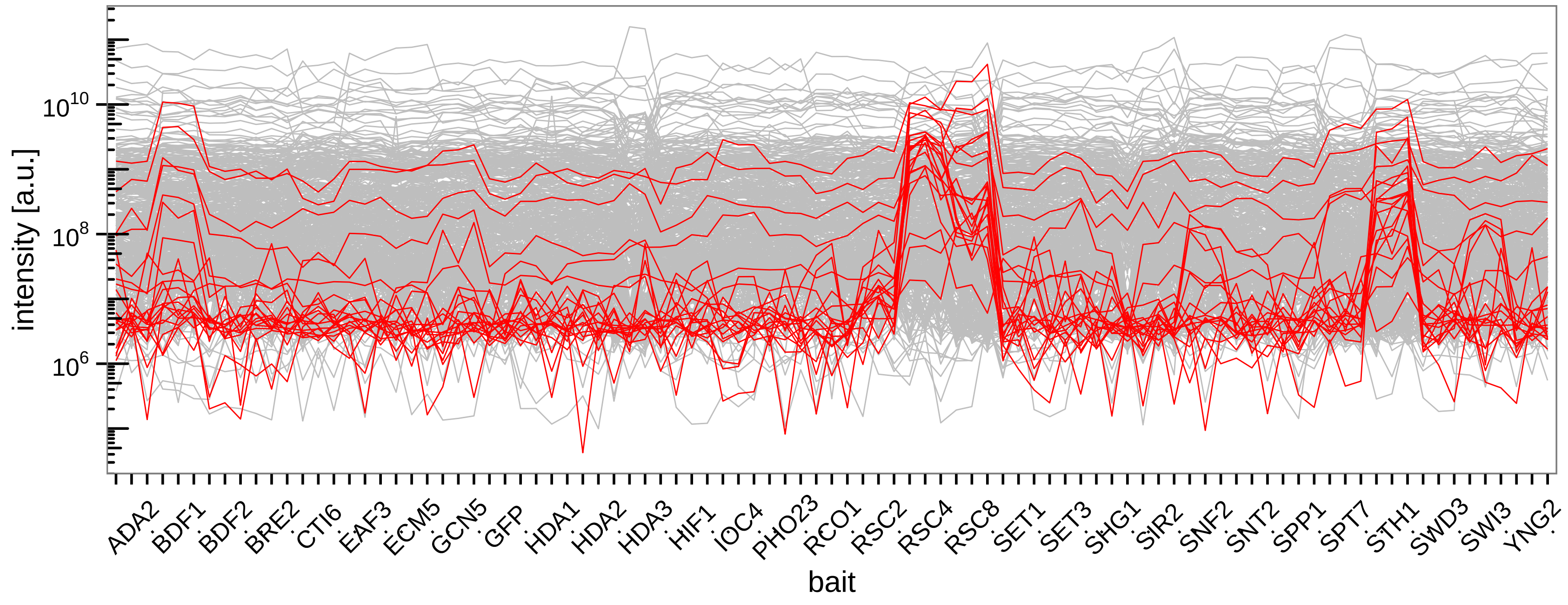

significance

interactor class

bait	RSC4
experiment	14 minutes

interactors	33
recovery	82 %

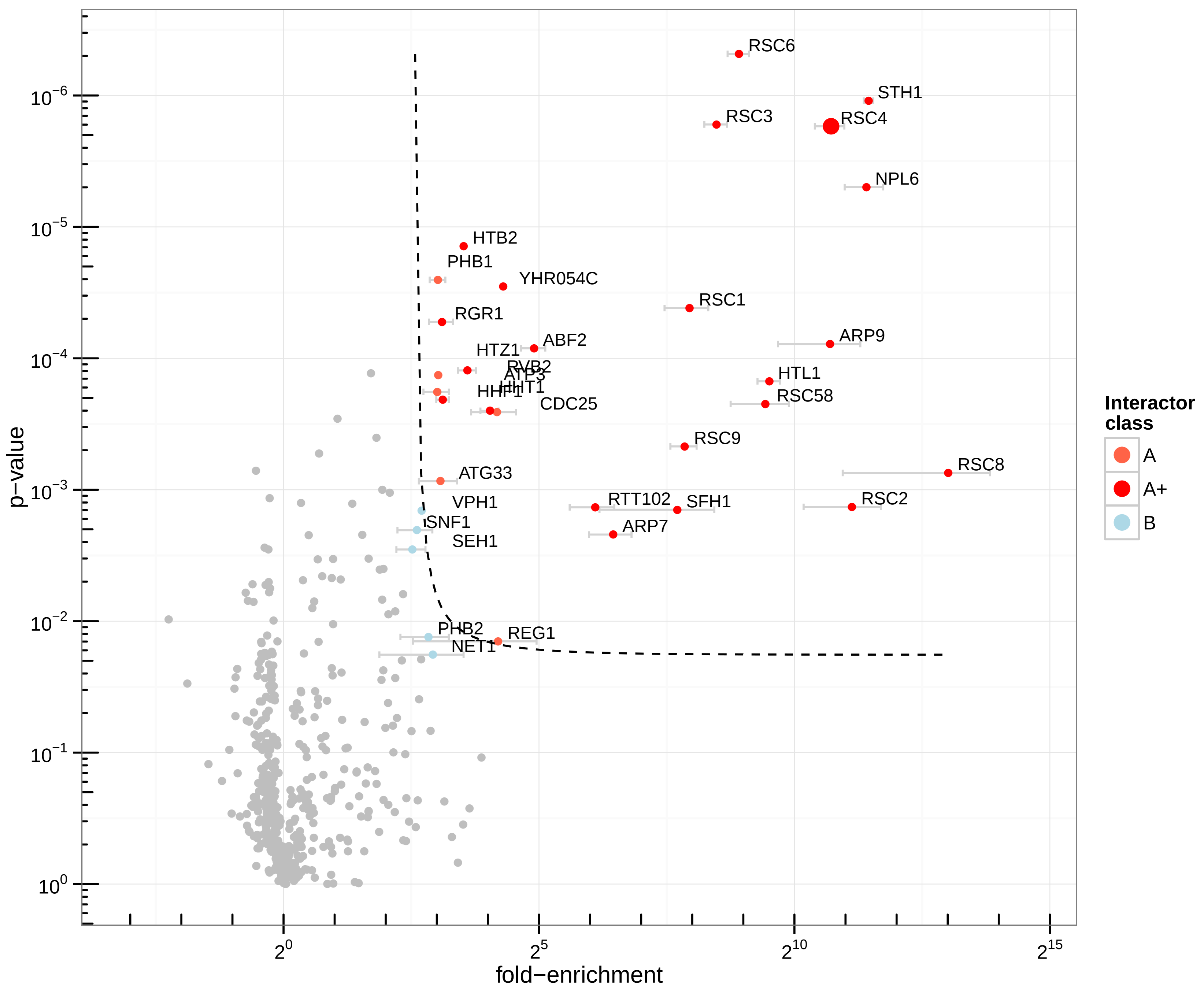

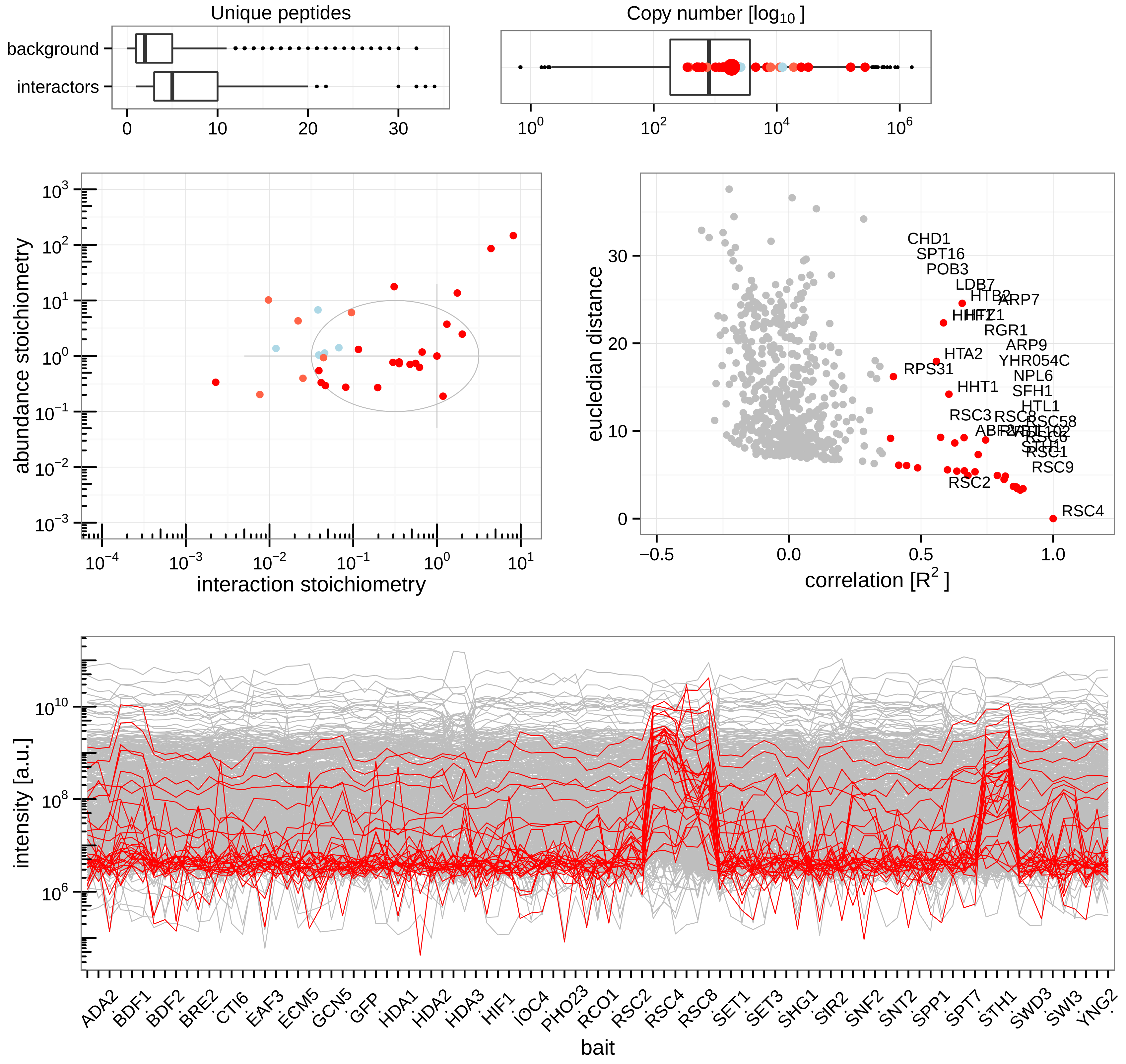

	ABF2	ARP7	ARP9	ATG33	ATP3	CDC25	HHF1	HHT1	HTB2	HTL1	HTZ1	NPL6	PHB1	REG1	RGR1	RSC1	RSC2	RSC3	RSC4	RSC58	RSC6	RSC8	RSC9	RTT102	RVB2	SFH1	STH1	YHR054C
significance	5.6	6.1	11.0	3.7	4.2	4.8	4.3	4.7	5.5	9.3	4.7	12.0	5.0	3.7	4.8	8.2	11.0	9.4	11.0	9.2	10.0	12.0	7.6	5.9	4.4	7.3	12.0	5.6
interactor class	A+	A+	A+	A	A	A	A+	A+	A+	A+	A+	A+	A	A	A+	A+	A+	A+	A+	A+	A+	A+	A+	A+	A	A+	A+	A+

**bait**	RSC8
**experiment**	14 minutes

**interactors**	25
**recovery**	82 %

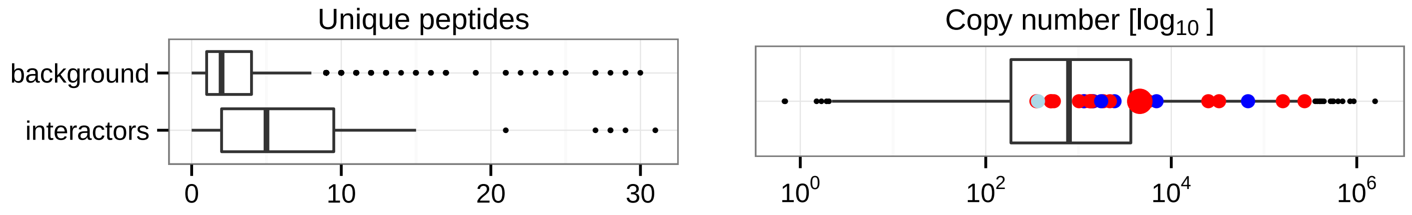

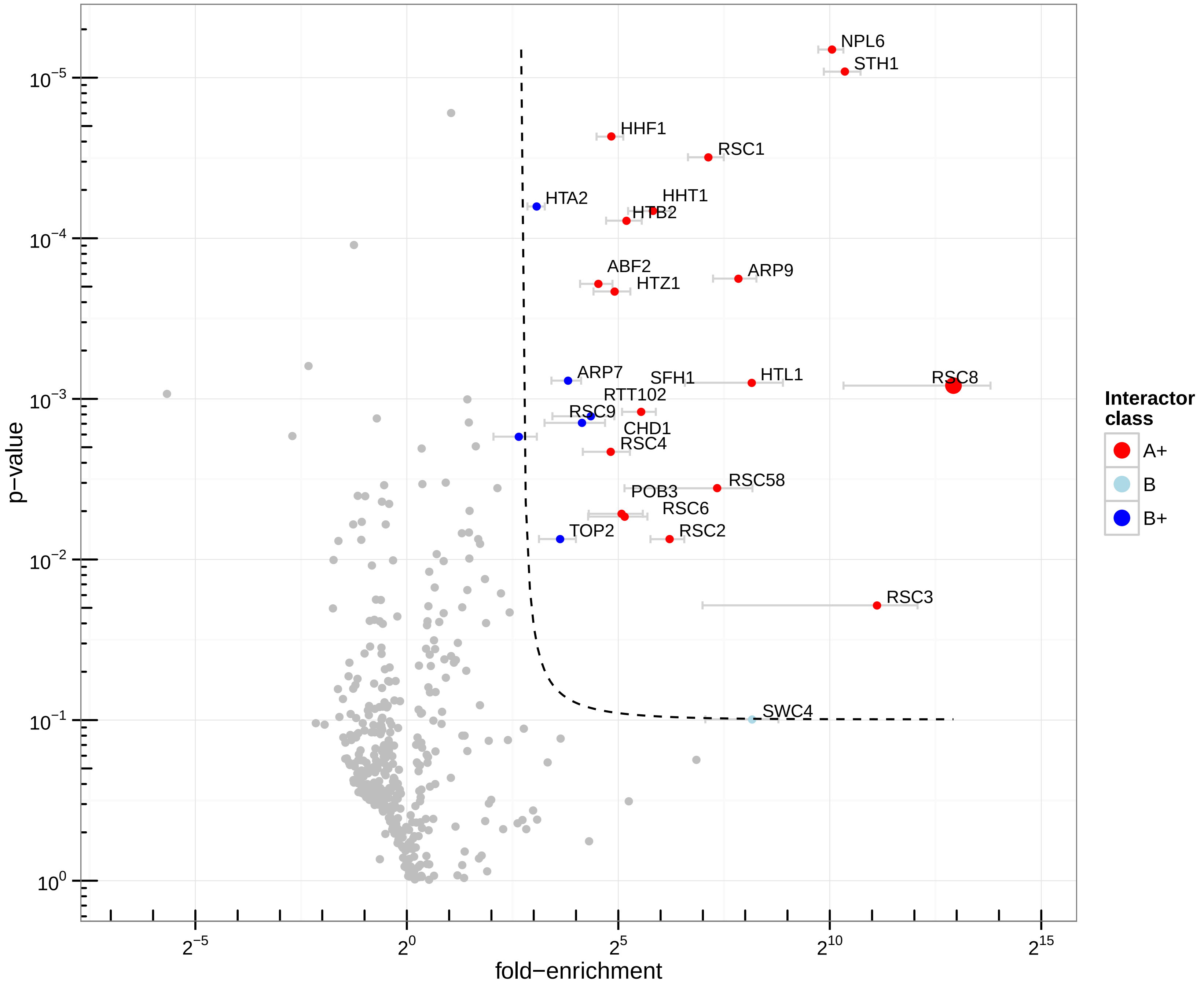

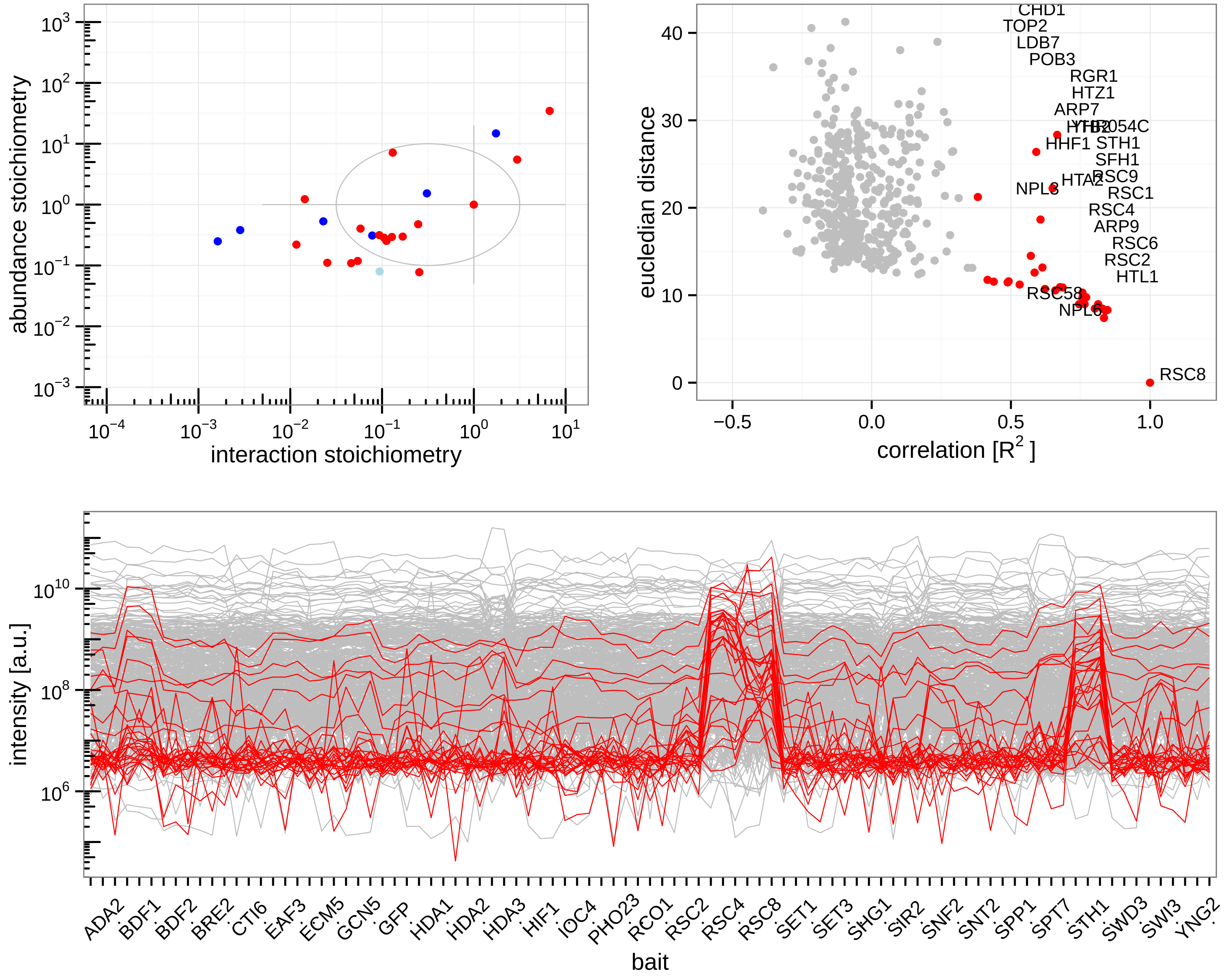

	ABF2	ARP7	ARP9	CHD1	HHF1	HHT1	HTA2	HTB2	HTL1	HTZ1	NPL6	POB3	RSC1	RSC2	RSC3	RSC4	RSC58	RSC6	RSC8	RSC9	RTT102	SFH1	STH1	TOP2
significance	5.1	4.2	7.8	3.2	6.0	6.4	4.7	5.9	7.8	5.4	10.0	4.7	7.6	5.6	10.0	4.7	6.8	4.7	12.0	4.2	4.4	5.4	11.0	3.4
interactor class	A+	B+	A+	B+	A+	A+	B+	A+	A+	A+	A+	A+	A+	A+	A+	A+	A+	A+	A+	B+	B+	A+	A+	B+

**bait**	SET1
**experiment**	14 minutes

**interactors**	9
**recovery**	100 %

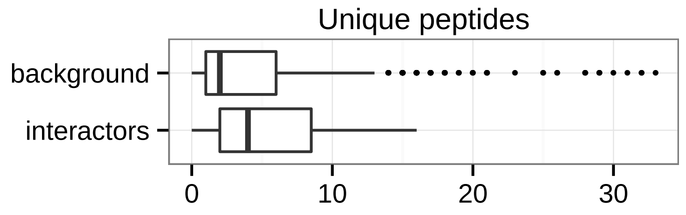

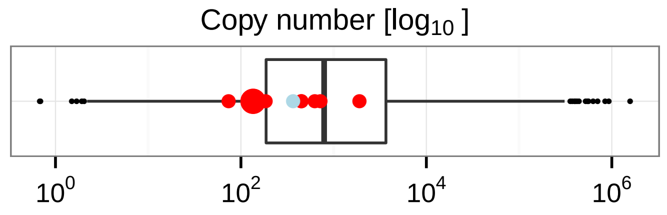

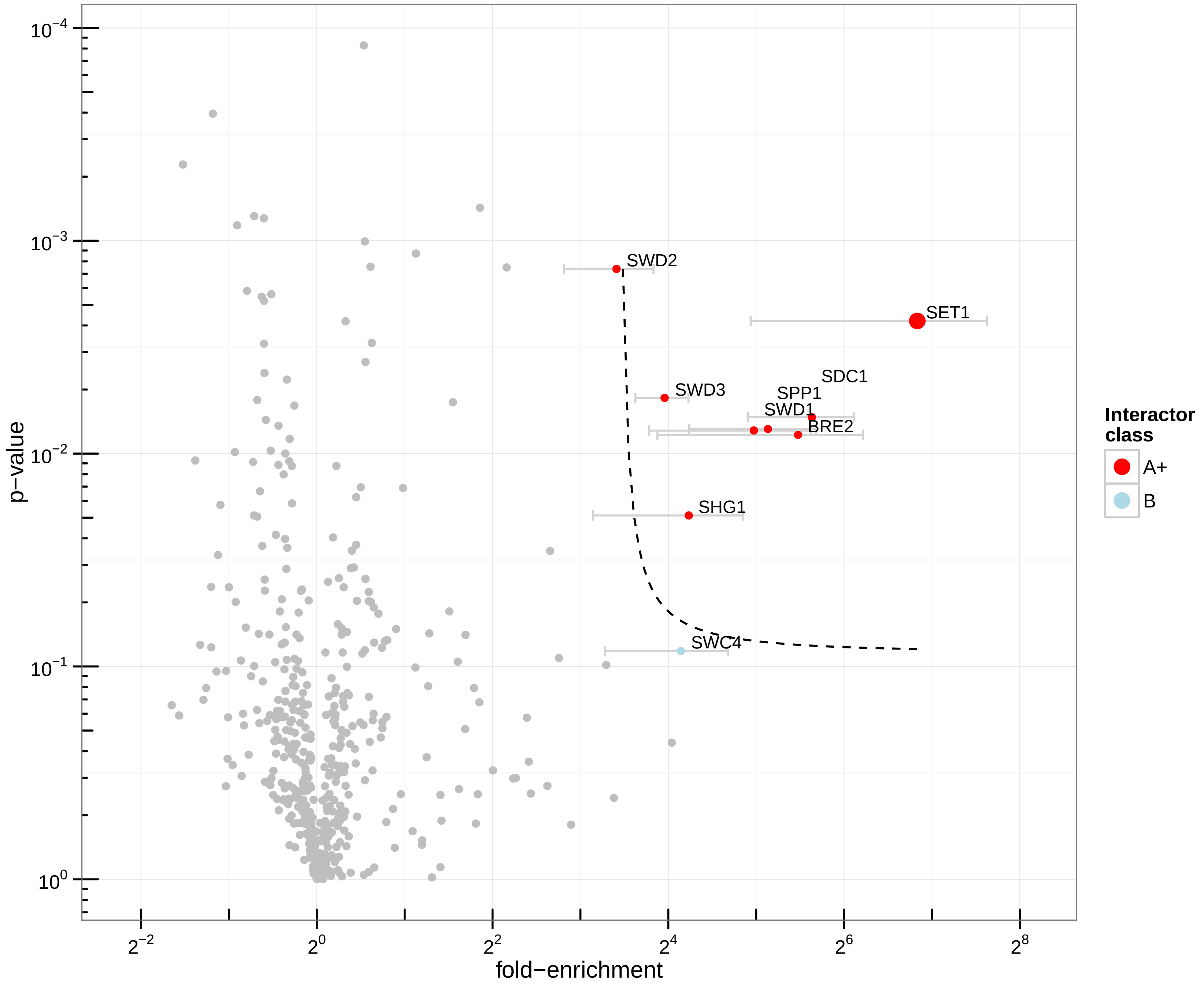

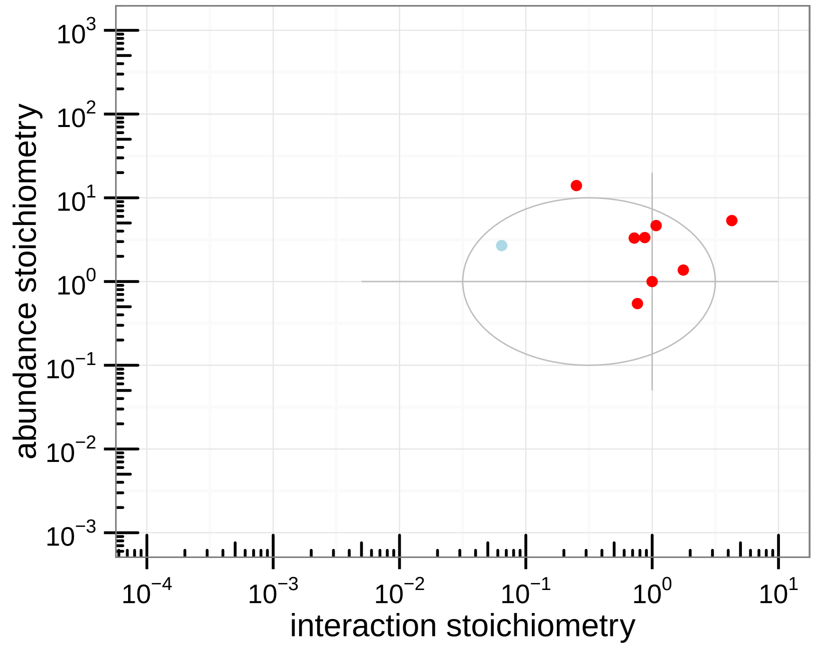

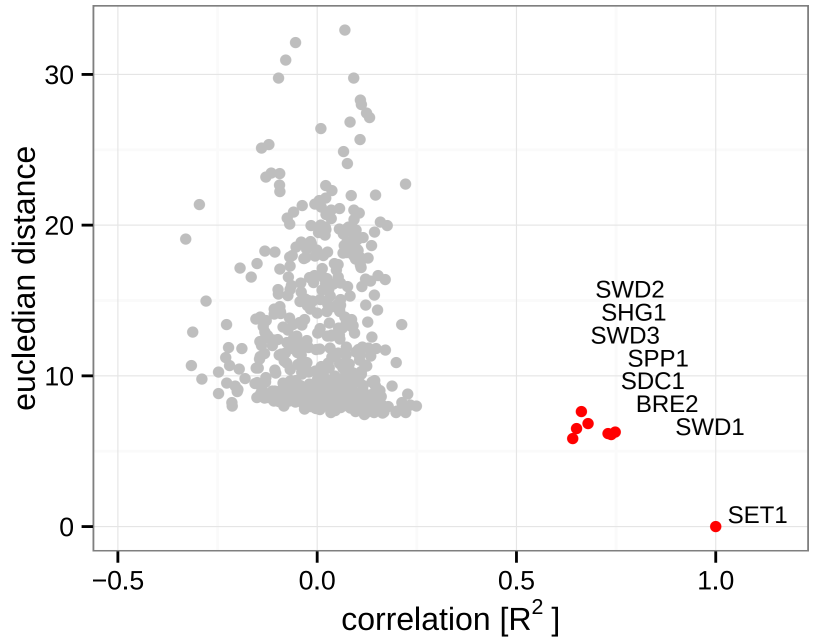

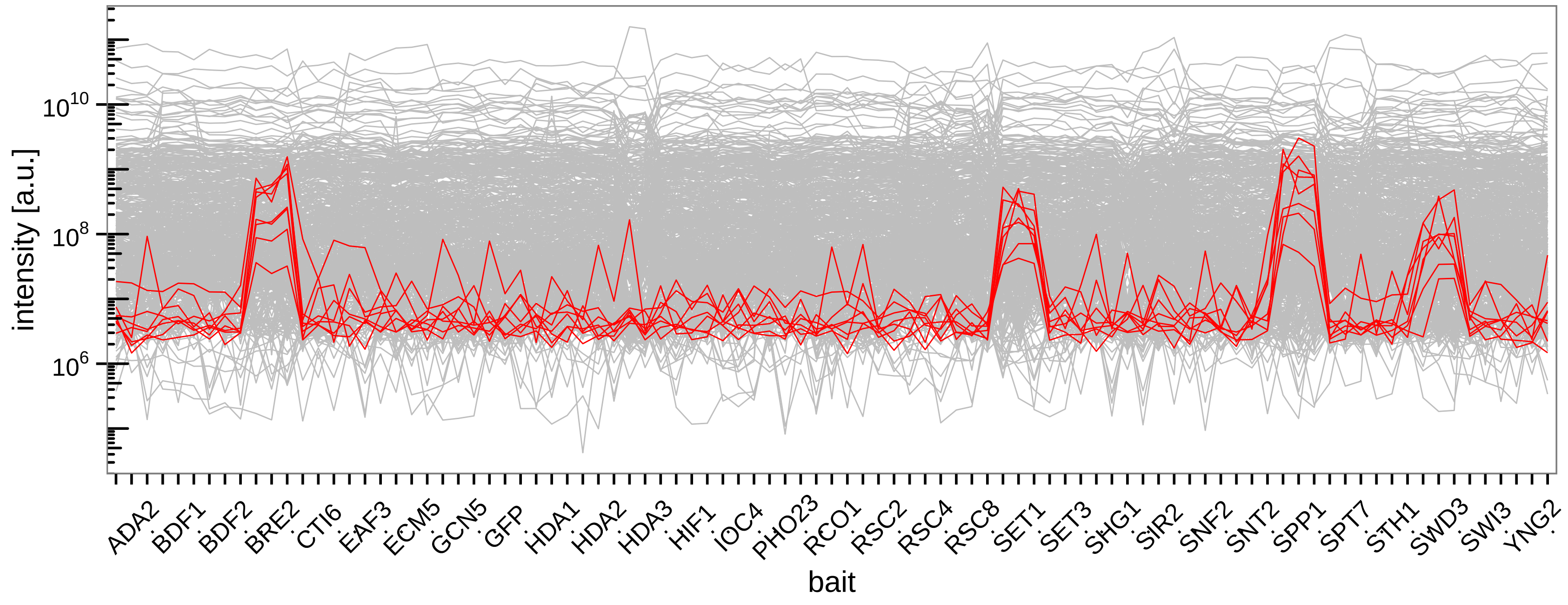

	BRE2	SDC1	SET1	SHG1	SPP1	SWD1	SWD2	SWD3
significance	4.9	5.1	6.4	3.7	4.6	4.5	3.7	3.7
interactor class	A+	A+	A+	A+	A+	A+	A+	A+

**bait**	SET3
**experiment**	14 minutes

**interactors**	4
**recovery**	18 %

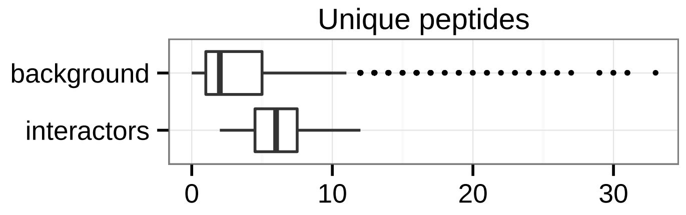

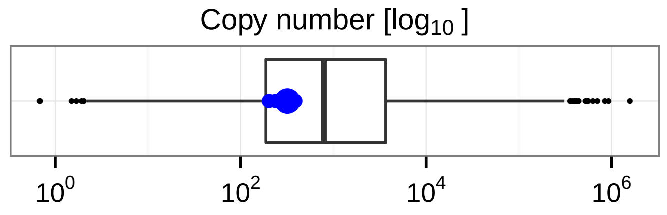

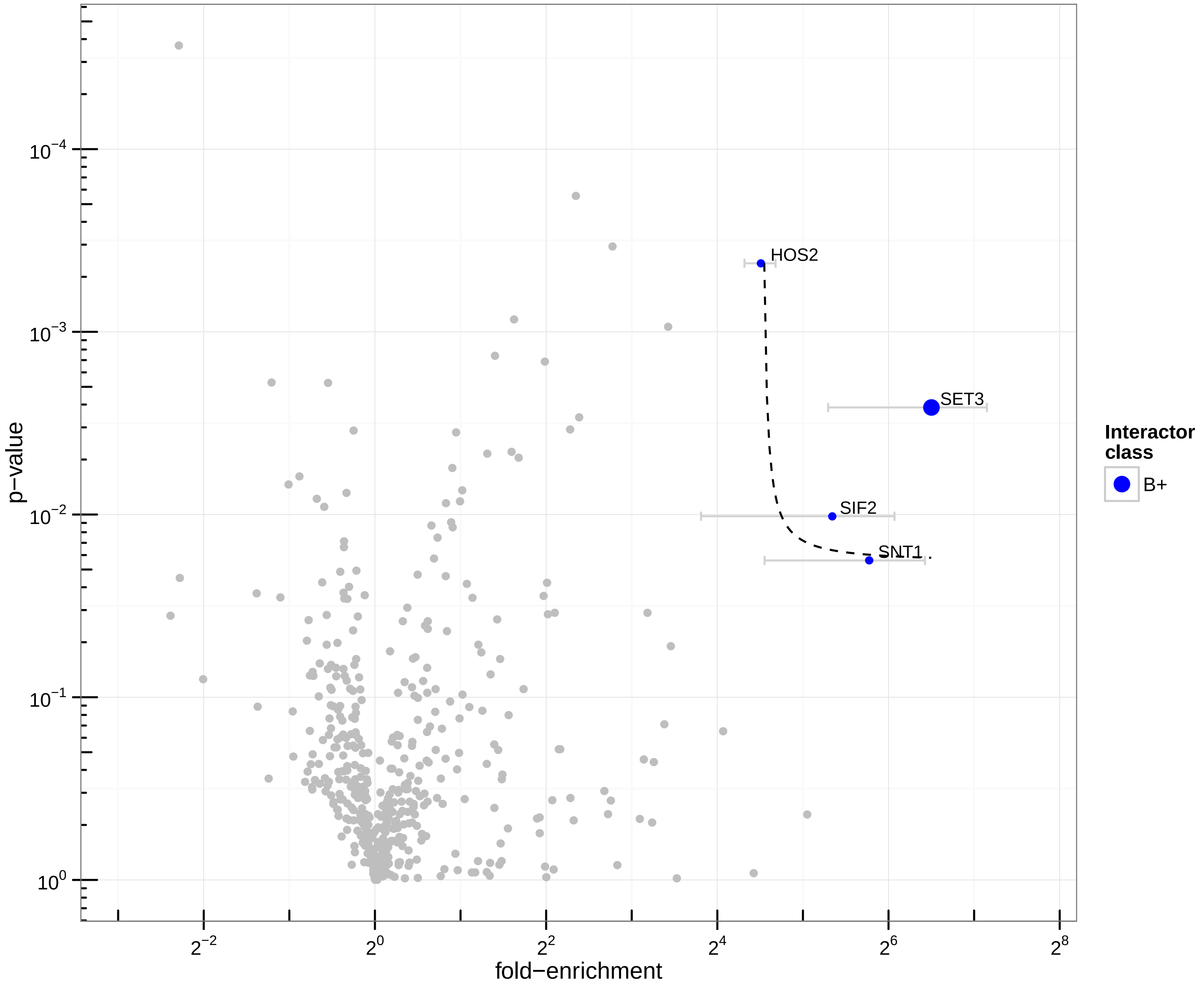

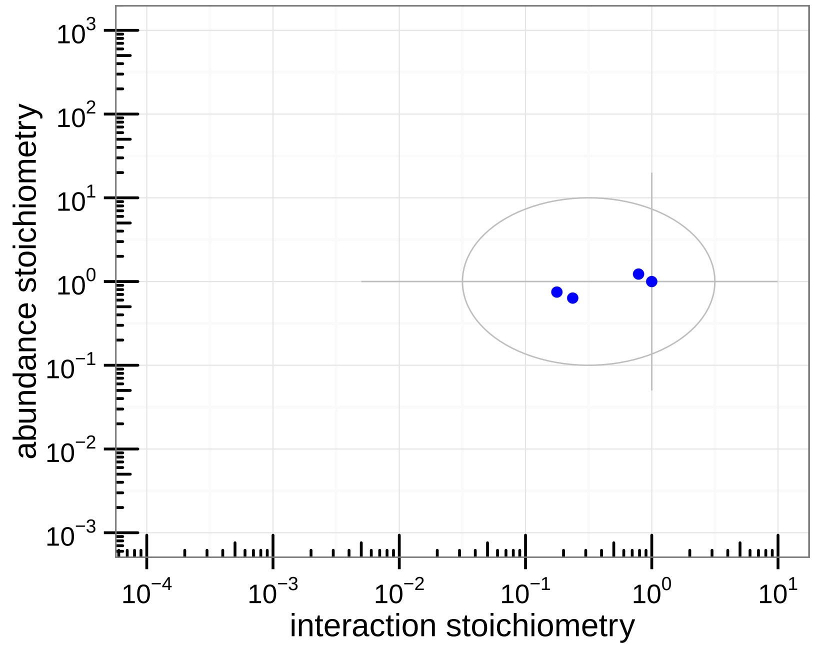

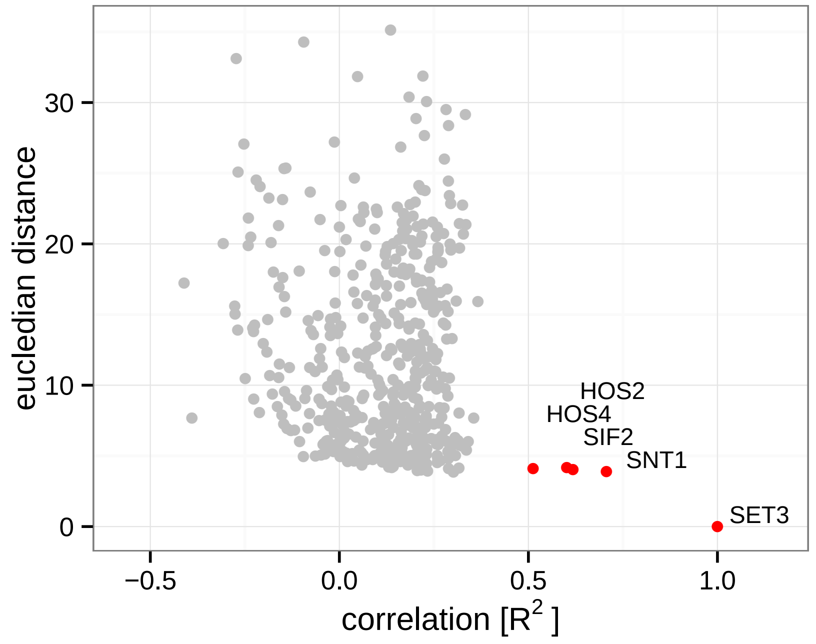

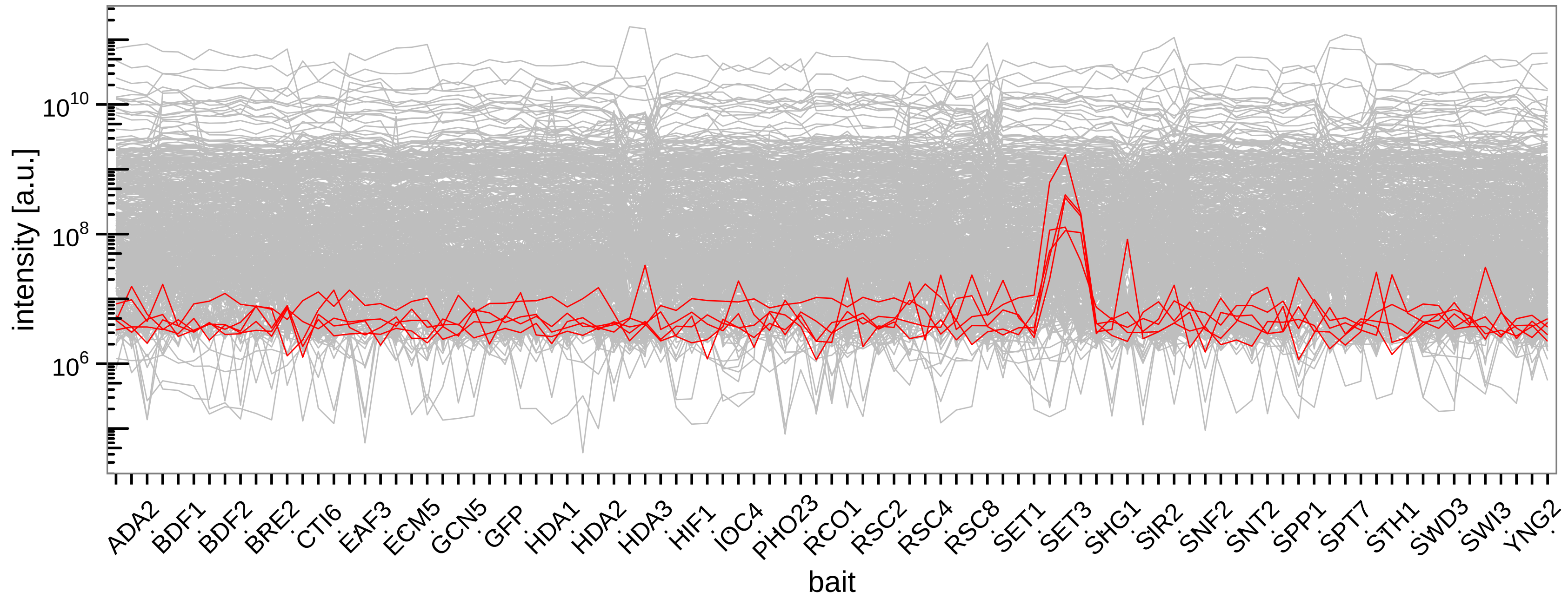

	HOS2	SET3	SIF2	SNT1
significance	4.9	6.1	4.8	5.1
interactor class	B+	B+	B+	B+

**bait**	SHG1	**interactors**	0
**experiment**	14 minutes	**recovery**	0 %

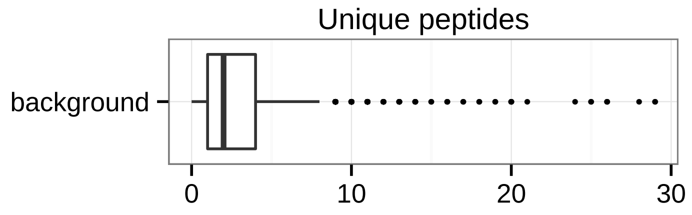

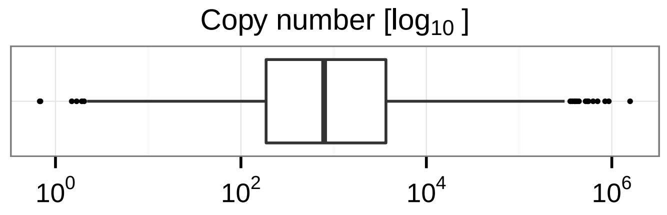

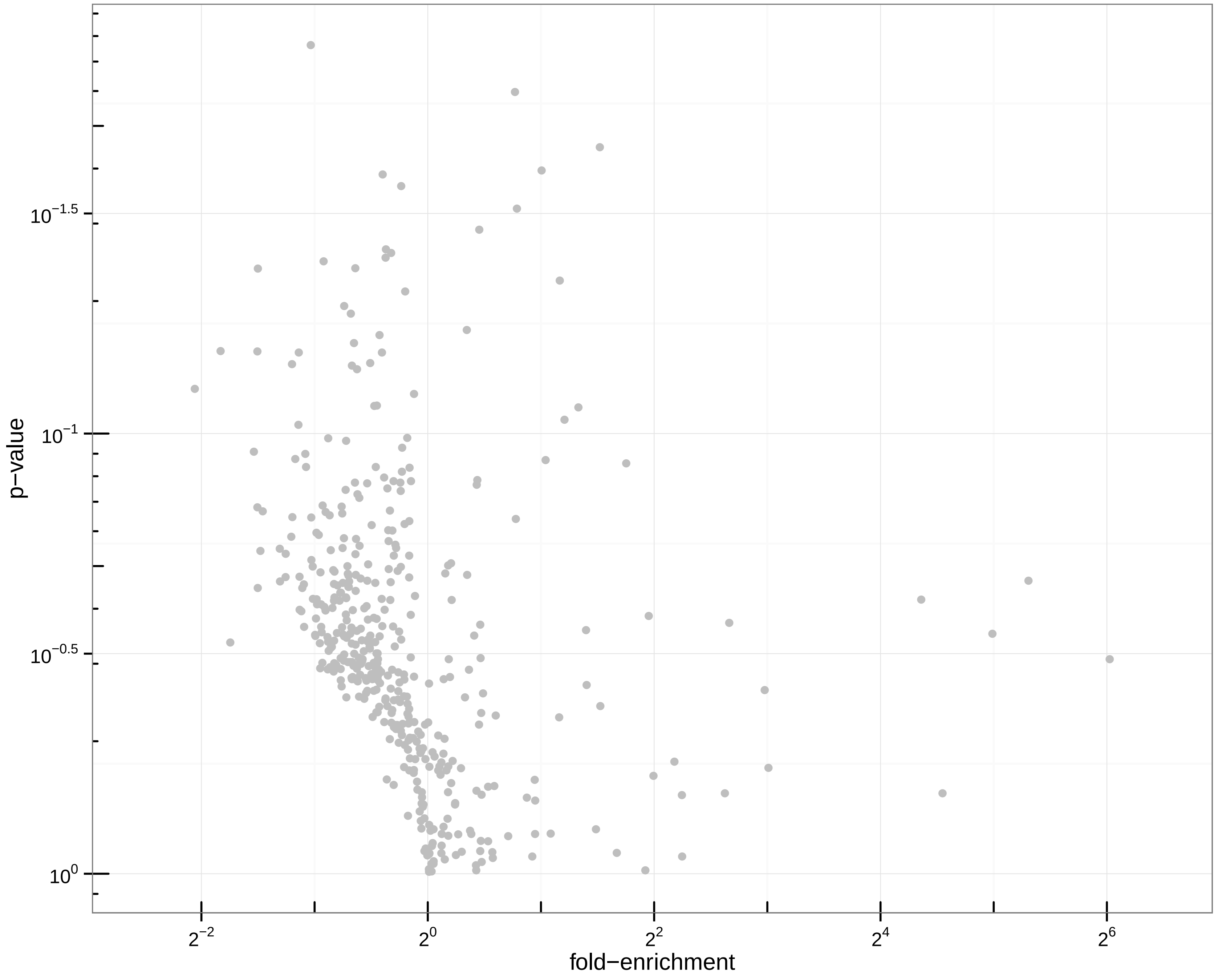

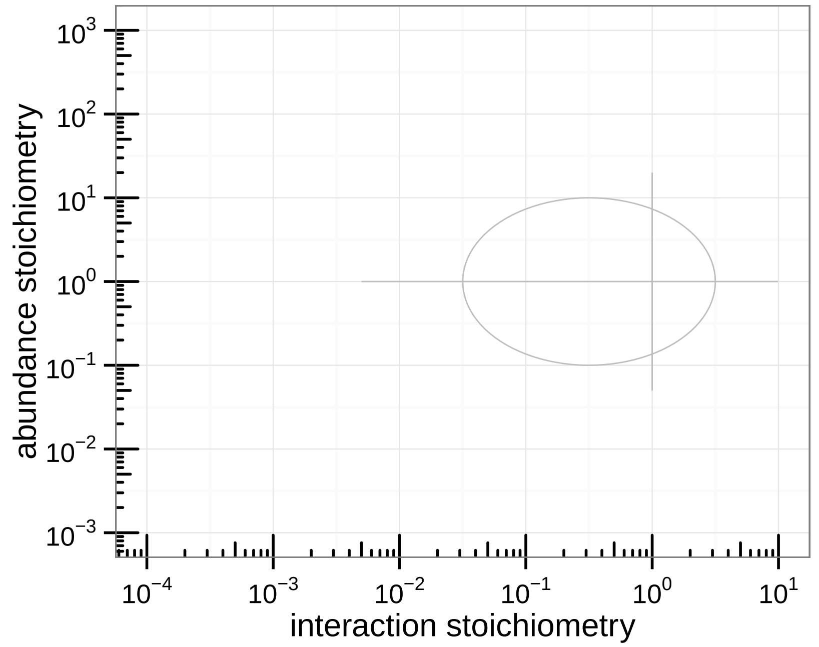

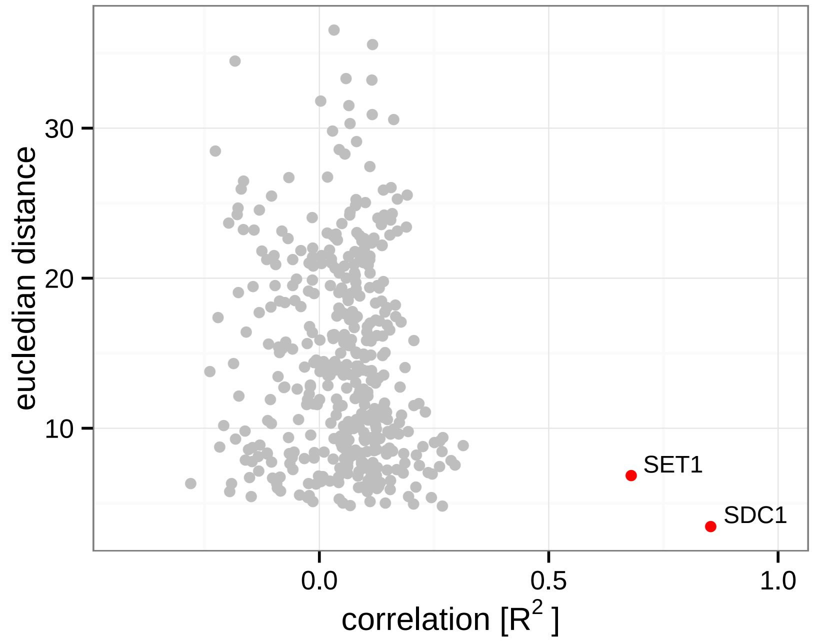

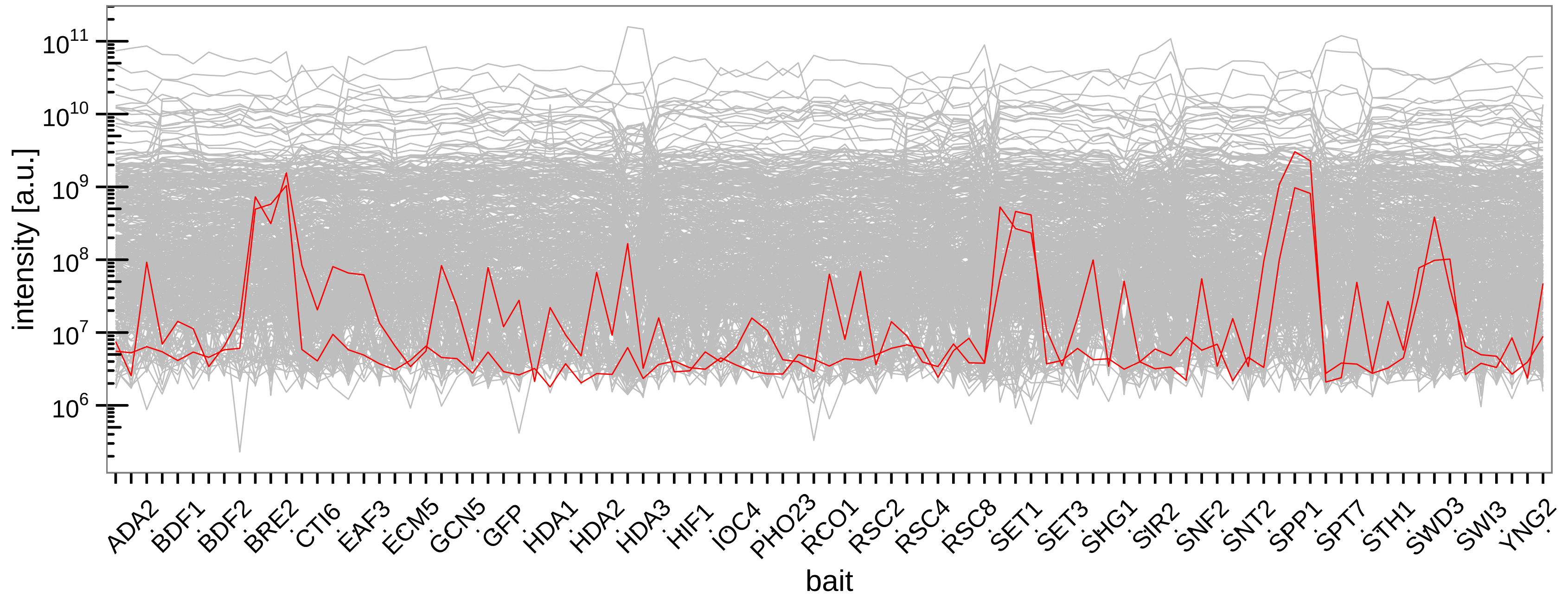

significance

interactor class

**bait**	SIR2
**experiment**	14 minutes

**interactors**	3
**recovery**	20 %

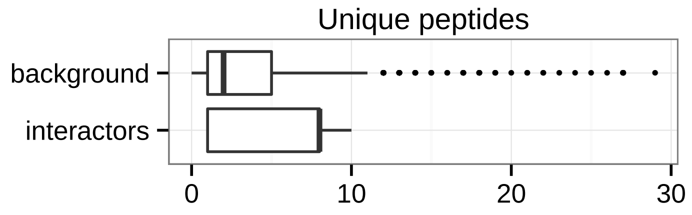

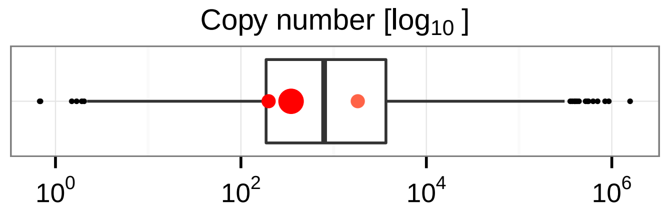

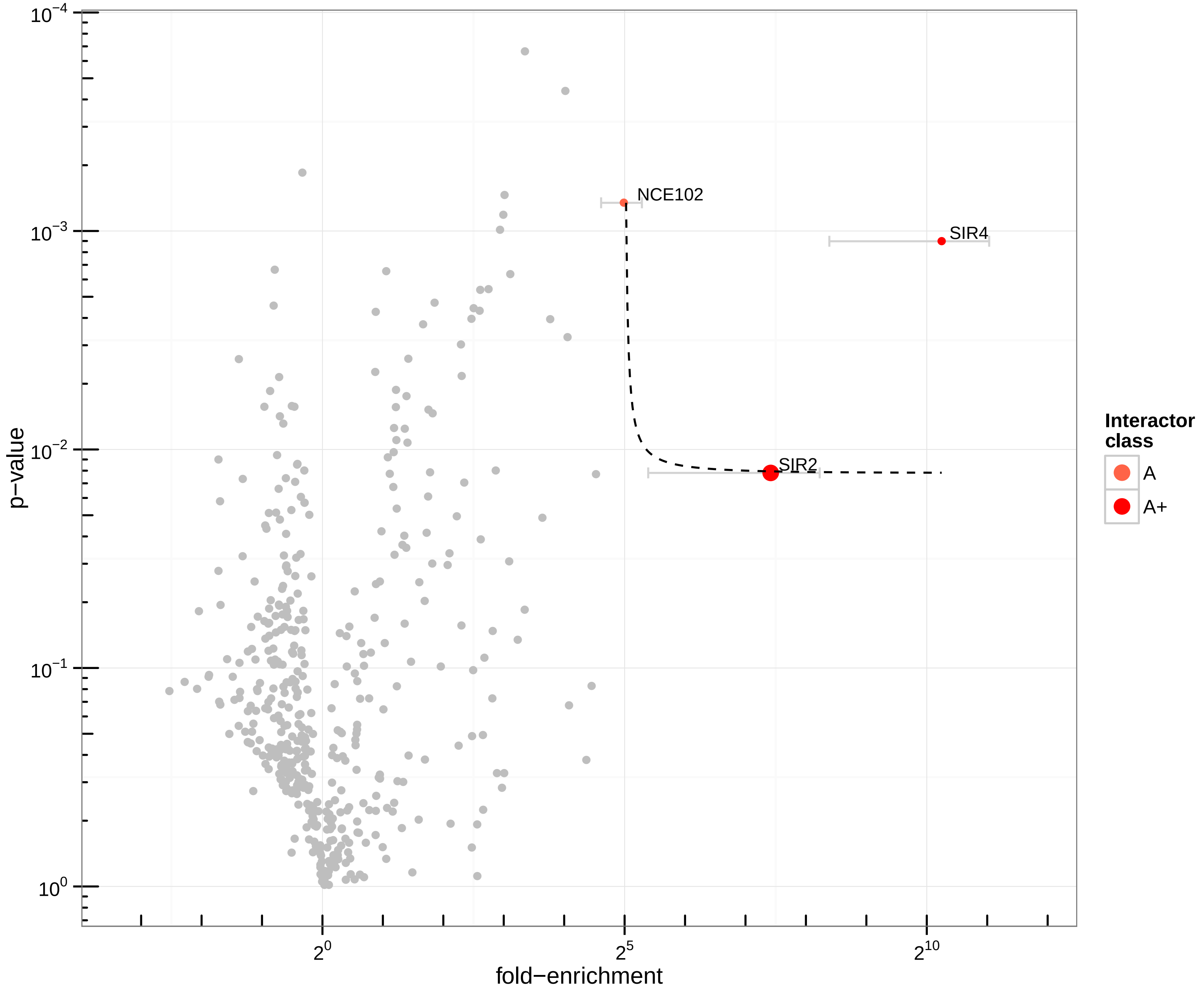

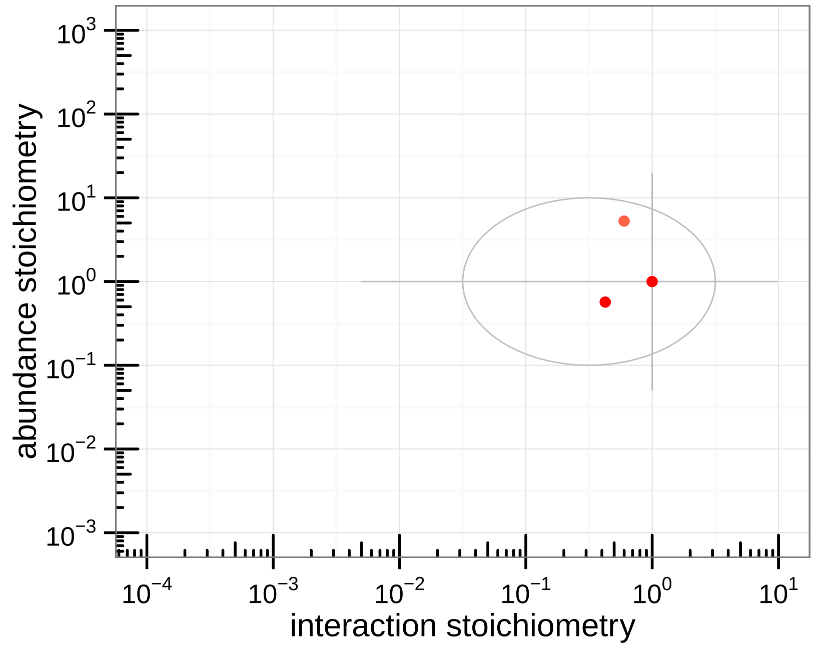

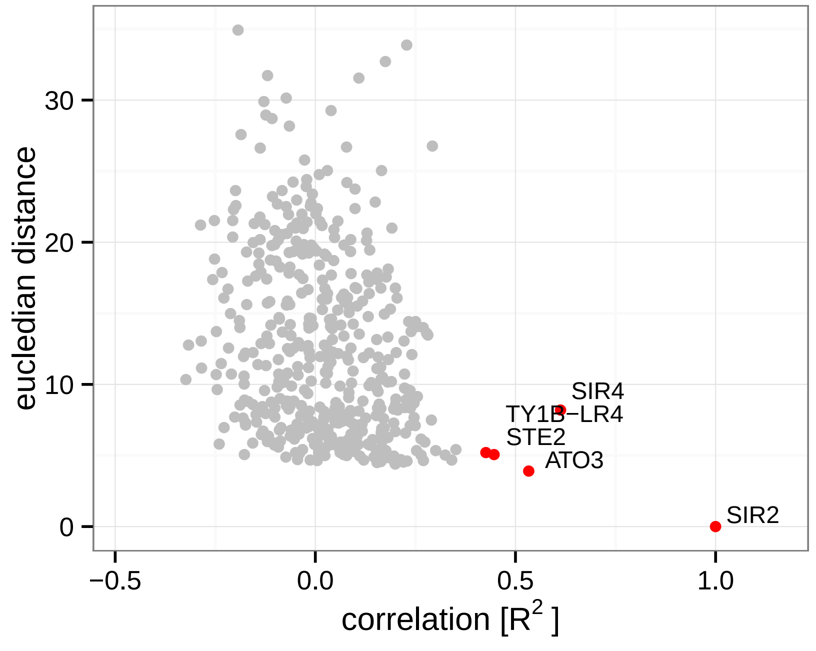

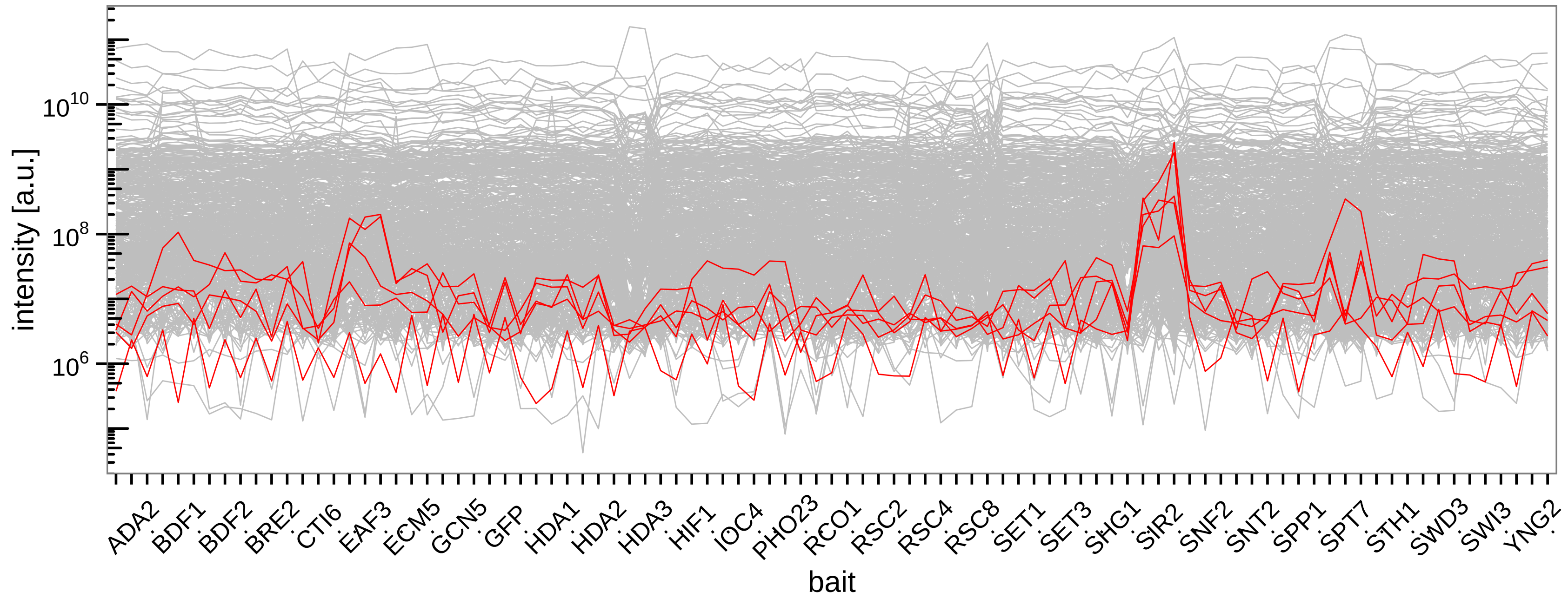

	NCE102	SIR2	SIR4
significance	5.1	6.7	9.7
interactor class	A	A+	A+

**bait**	SNF2
**experiment**	14 minutes

**interactors**	6
**recovery**	64 %

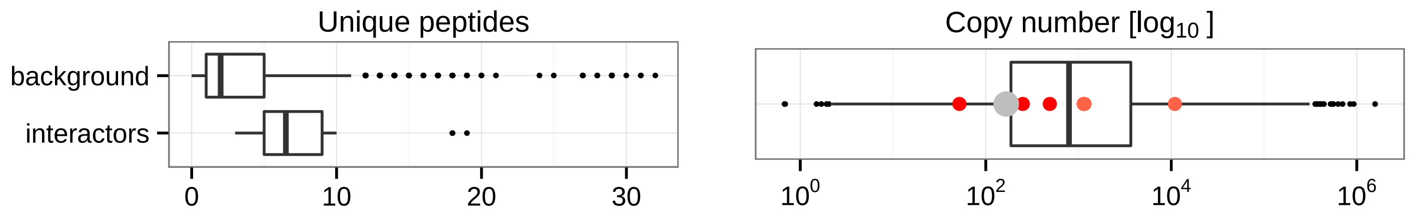

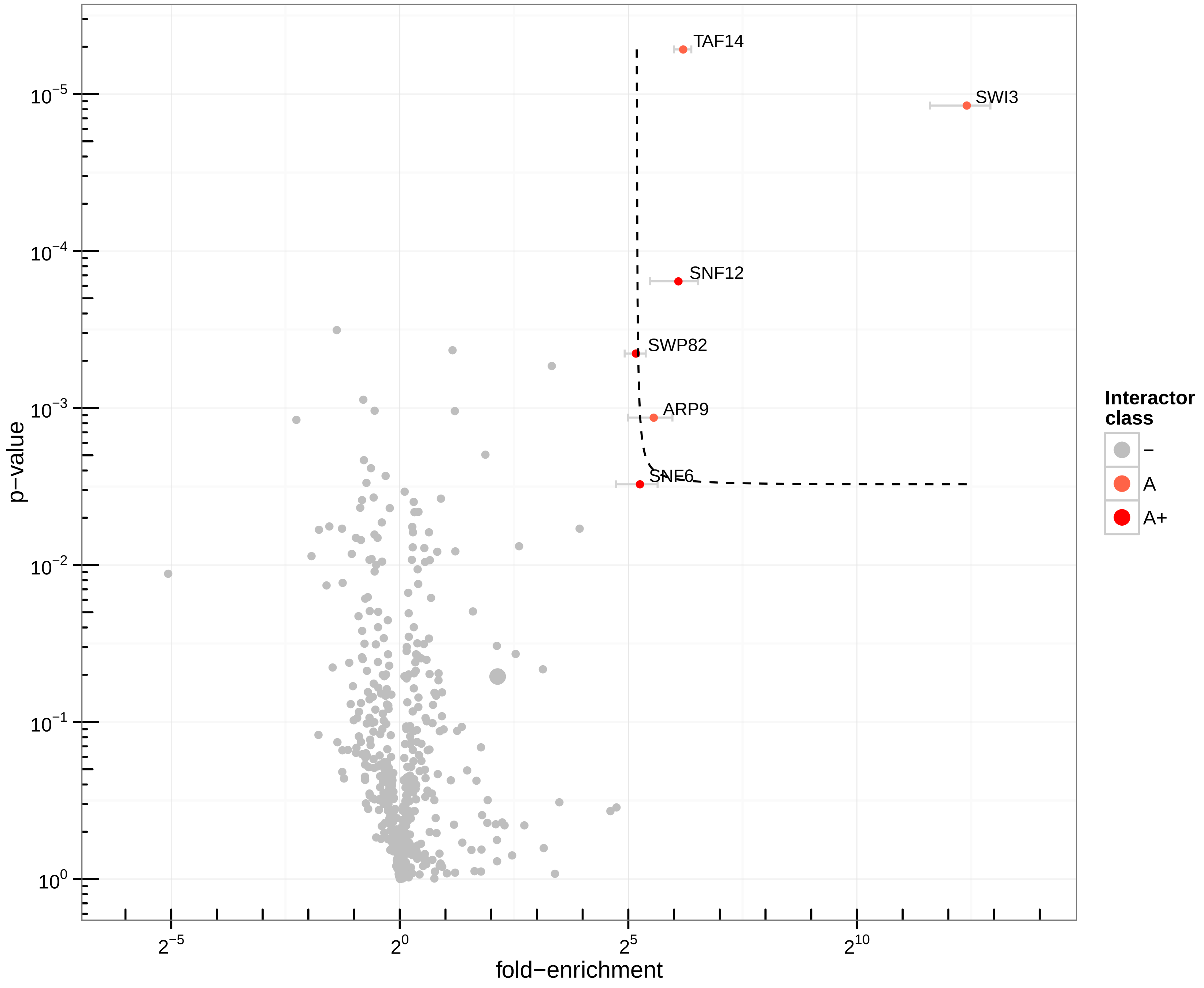

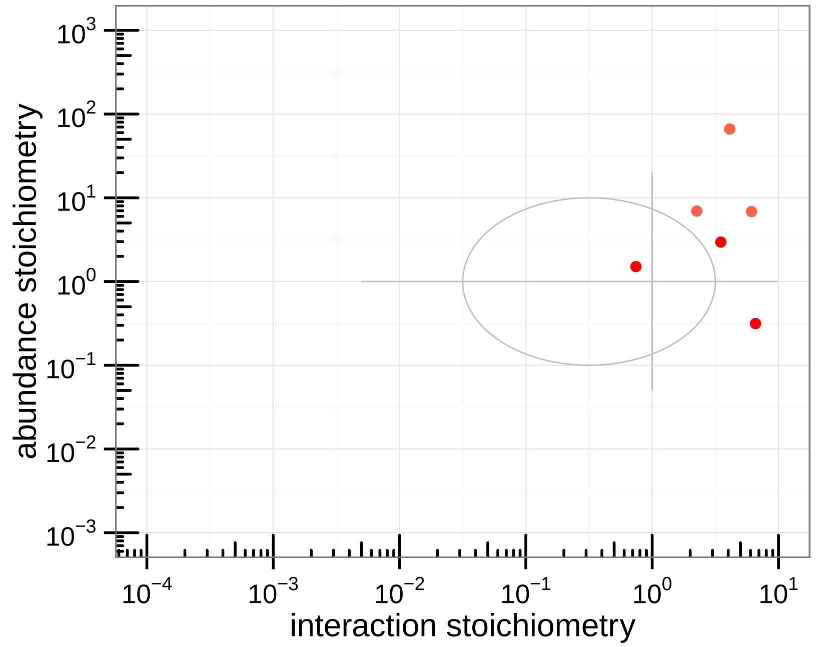

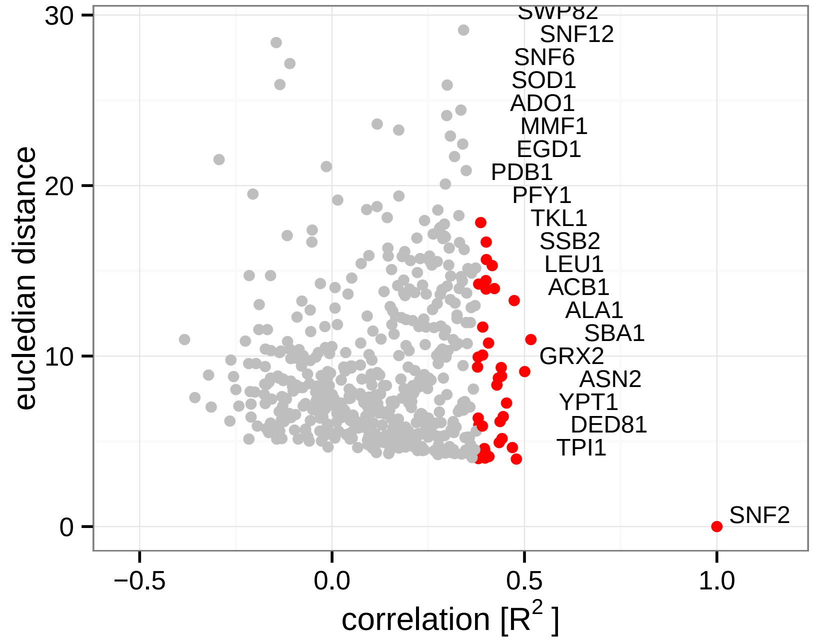

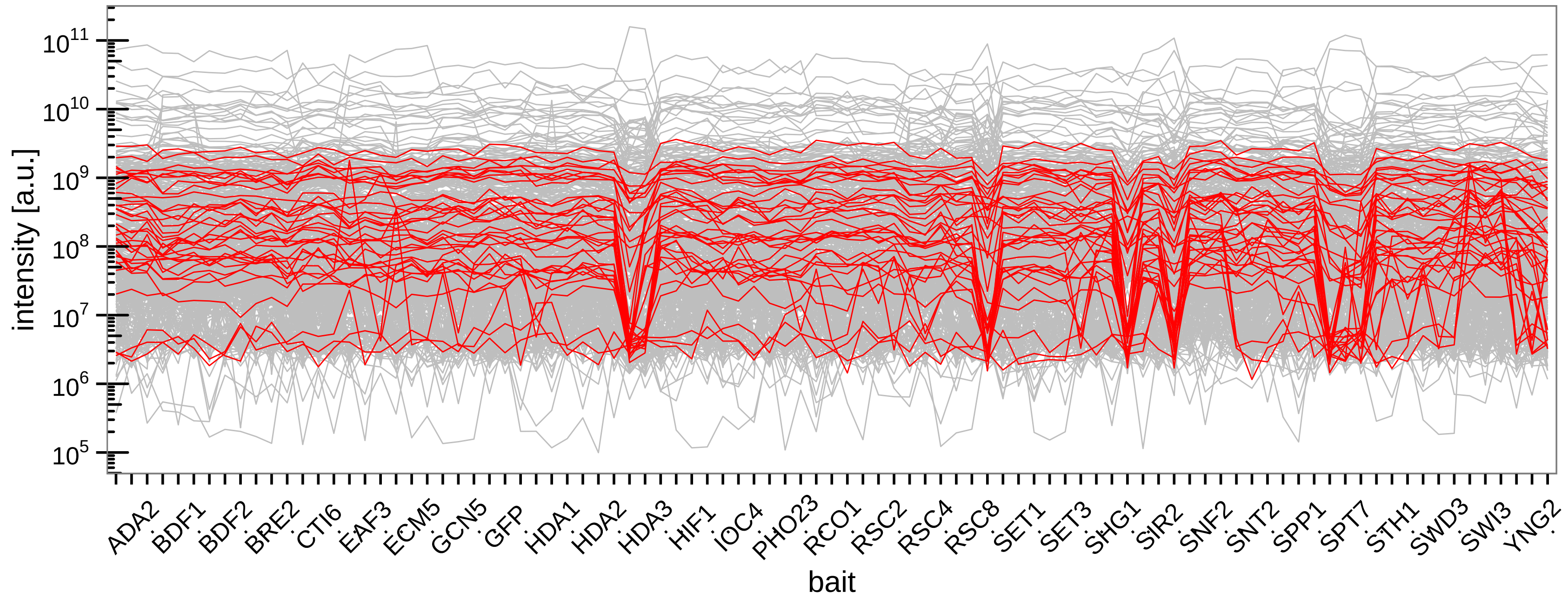

	ARP9	SNF12	SNF6	SWI3	SWP82	TAF14
significance	5.4	6.4	4.9	12.0	5.3	7.4
interactor class	A	A+	A+	A	A+	A

**bait**	SNT2
**experiment**	14 minutes

**interactors**	0
**recovery**	0 %

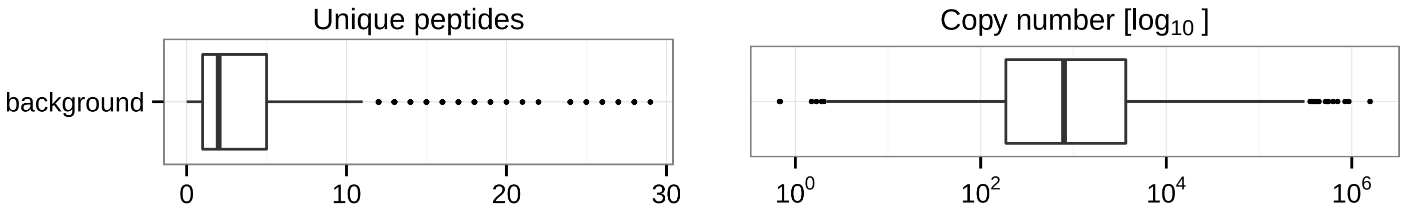

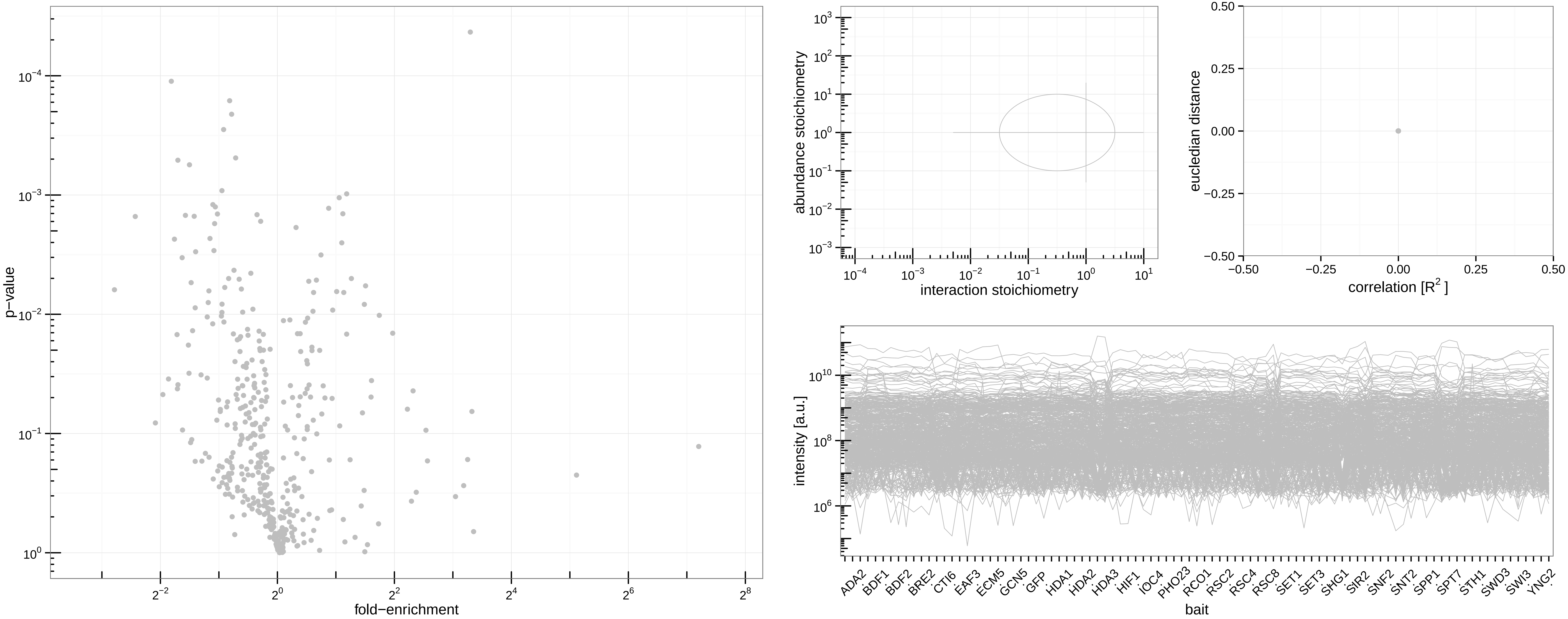

significance

interactor class

**bait**	SPP1		**interactors**	8
**experiment**	14 minutes		**recovery**	100 %

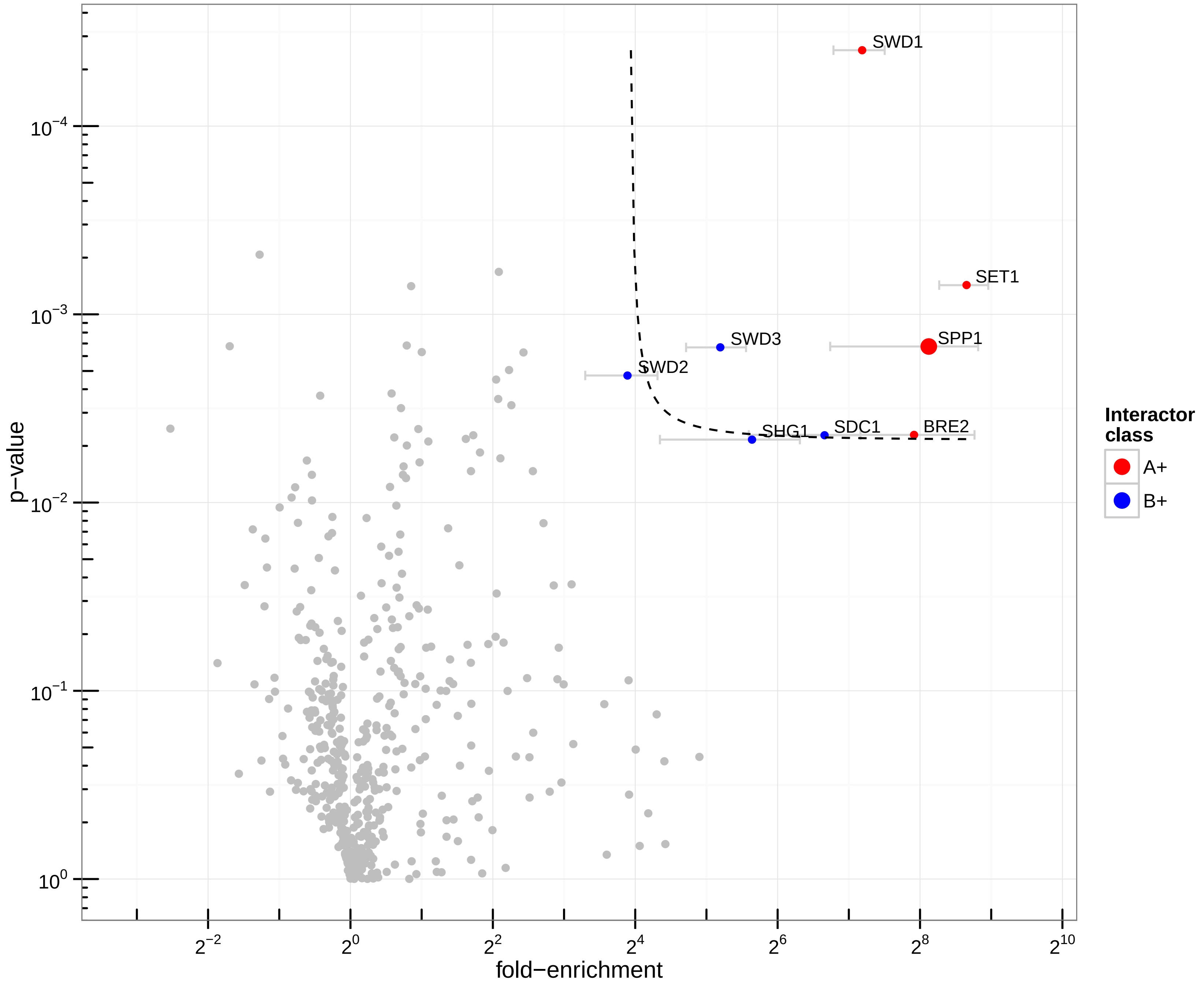

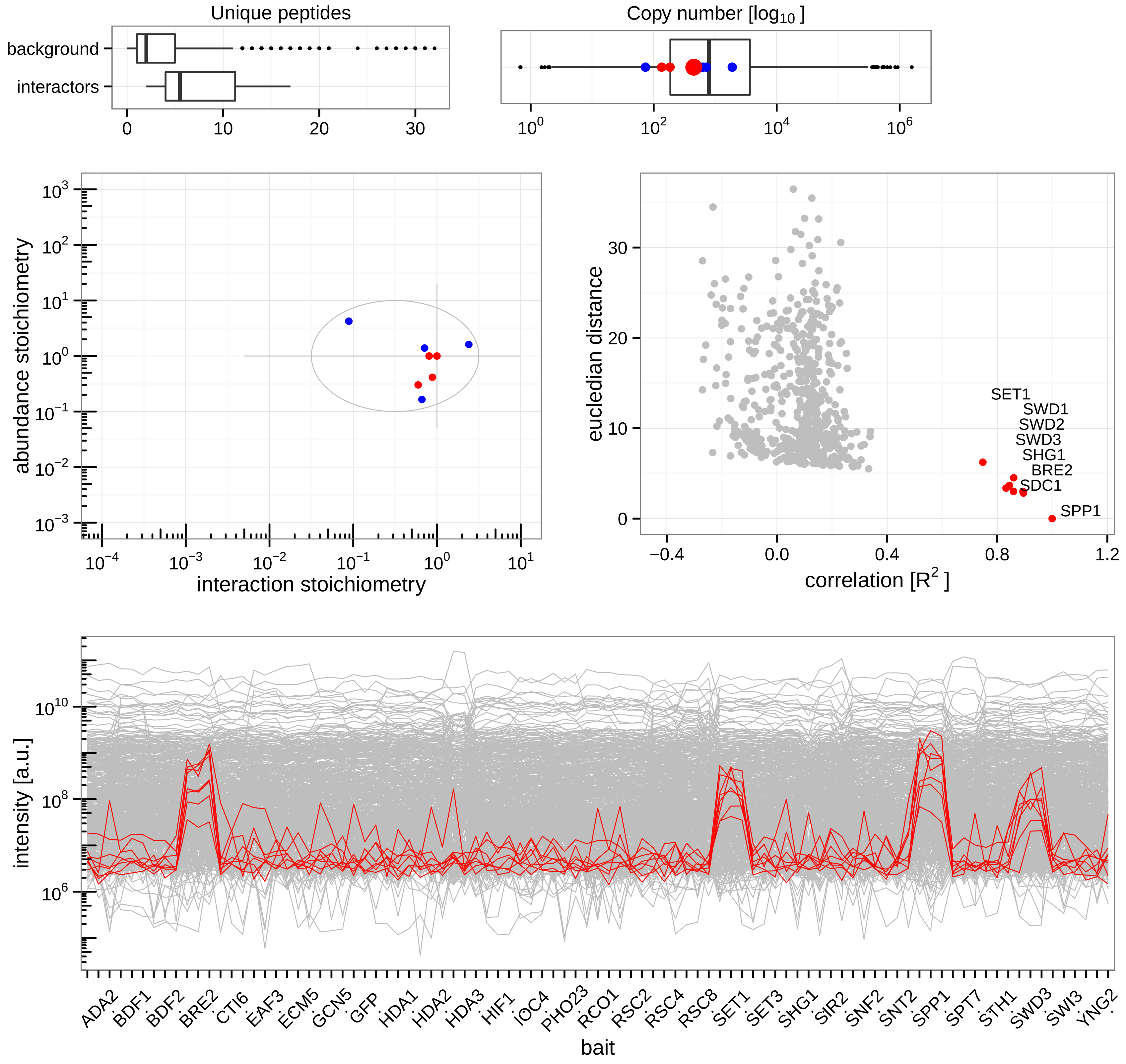

	BRE2	SDC1	SET1	SHG1	SPP1	SWD1	SWD2	SWD3
significance	7.3	6.1	8.3	5.2	7.7	7.6	3.9	5.1
interactor class	A+	B+	A+	B+	A+	A+	B+	B+

bait	SPT7
experiment	14 minutes

interactors	64
recovery	74 %

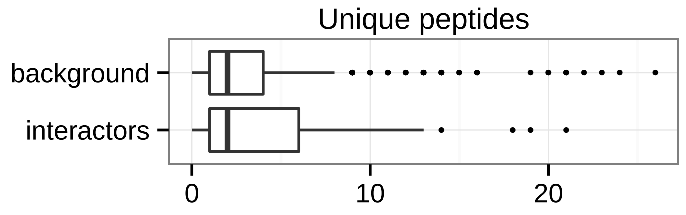

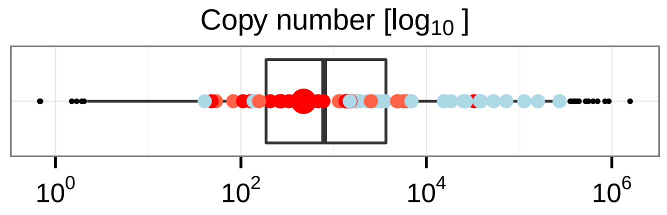

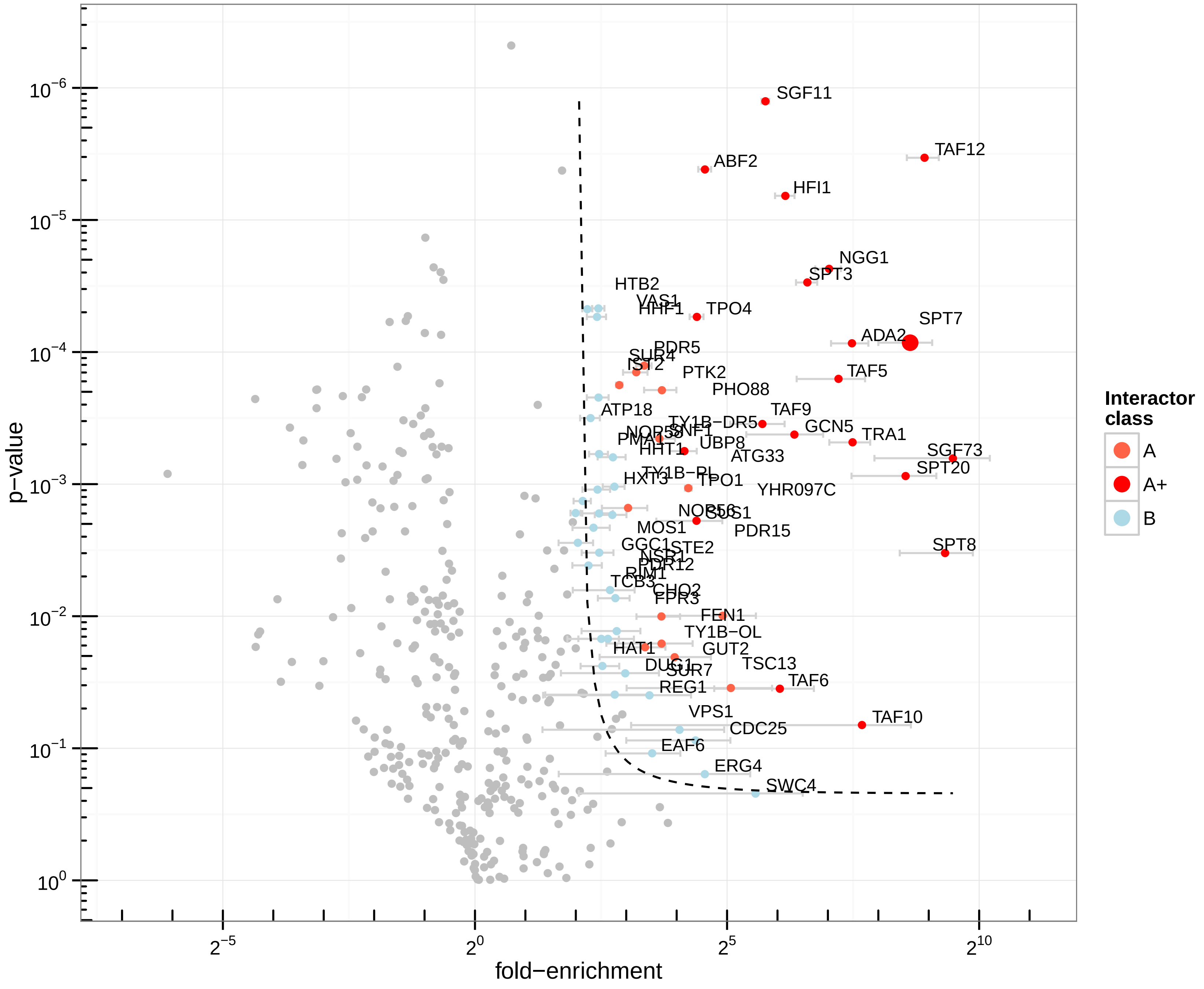

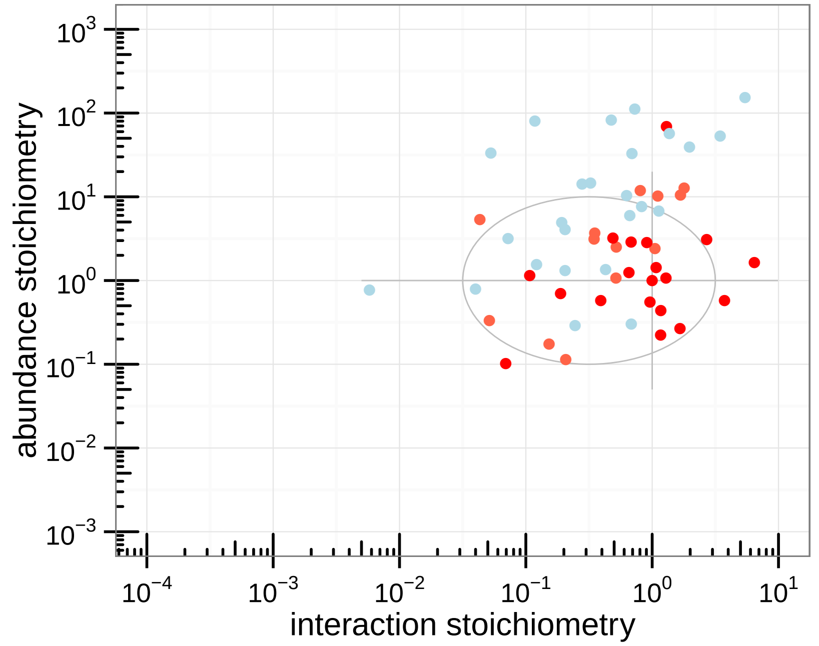

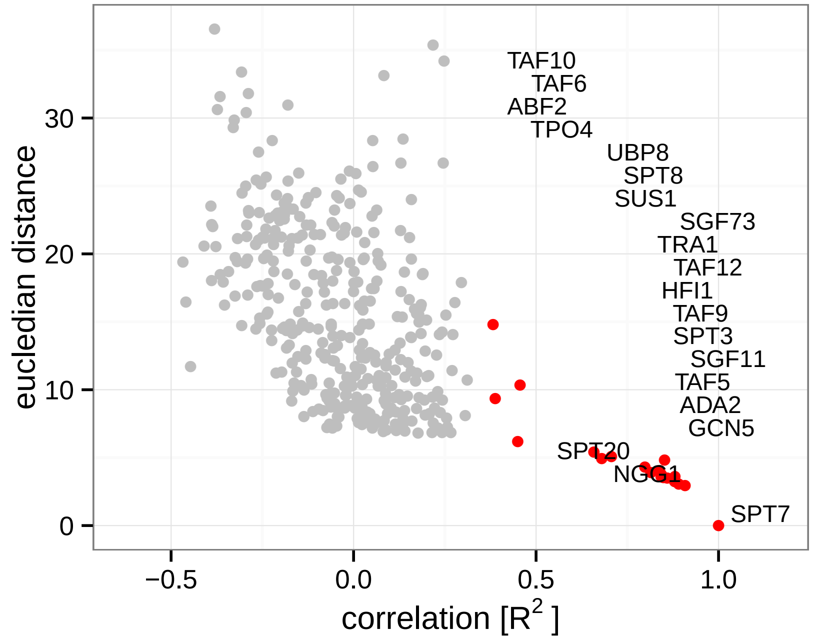

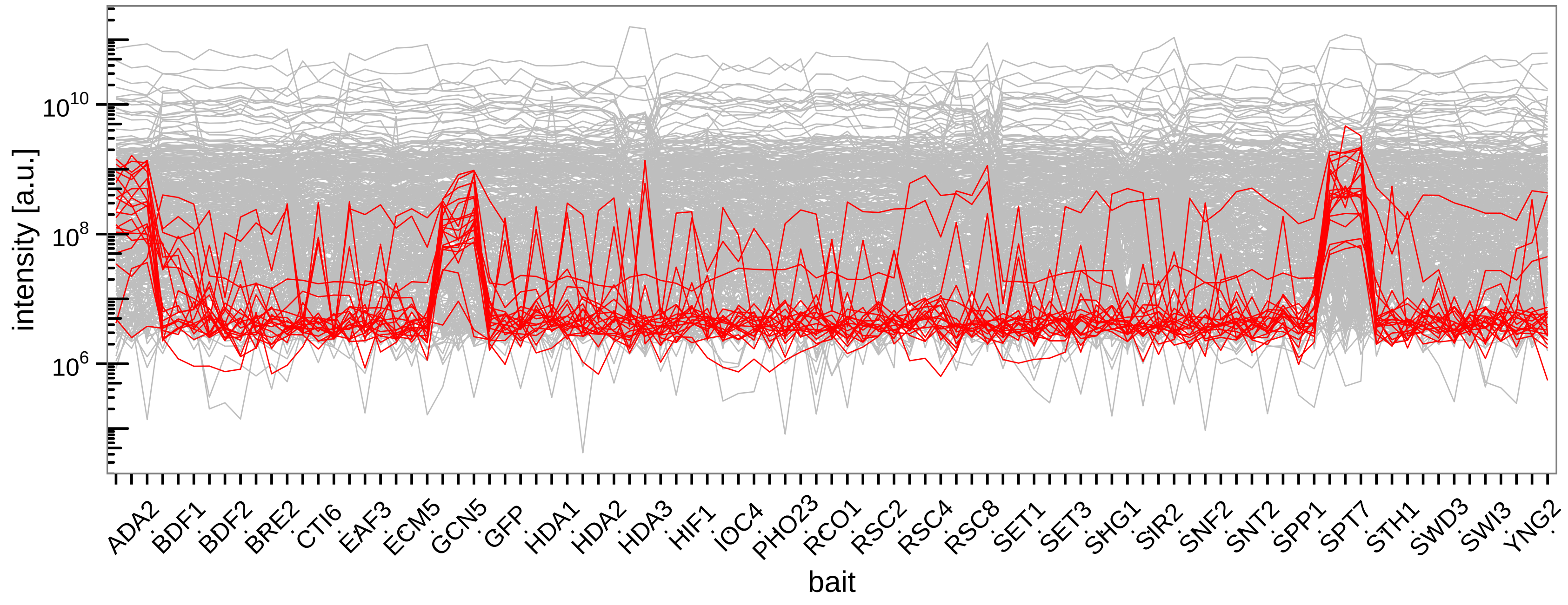

	ABF2	ADA2	ATG33	FEN1	GCN5	GUT2	HFI1	IST2	NGG1	PDR15	PDR5	PTK2	SGF11	SGF73	SNF1	SPT20
significance	6.5	7.6	3.5	3.2	6.3	3.4	7.3	4.4	7.6	4.4	4.6	4.6	7.6	9.1	4.3	8.1
interactor class	A+	A+	A	A	A+	A	A+	A	A+	A	A	A	A+	A+	A	A+

	SPT3	SPT7	SPT8	STE2	SUR4	SUS1	TAF10	TAF12	TAF5	TAF6	TAF9	TPO1	TPO4	TRA1	TSC13	TY1B−OL	UBP8
significance	7.2	8.6	8.7	3.4	4.2	4.4	6.8	9.6	7.3	5.3	5.8	4.4	5.5	7.3	4.3	3.0	4.5
interactor class	A+	A+	A+	A	A	A+	A+	A+	A+	A+	A+	A	A+	A+	A	A	A+

**bait**	STH1
**experiment**	14 minutes

**interactors**	22
**recovery**	82 %

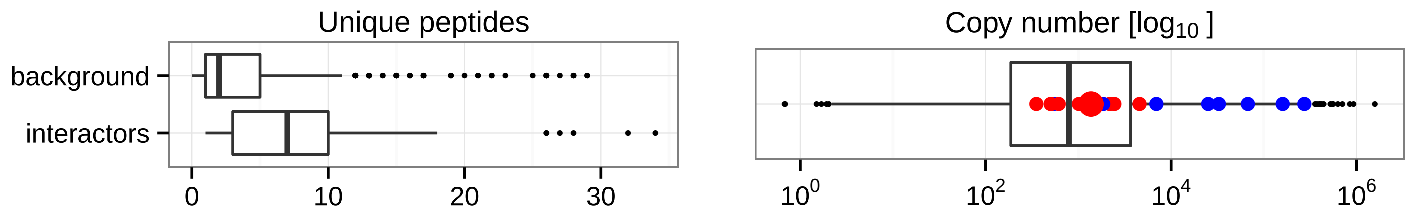

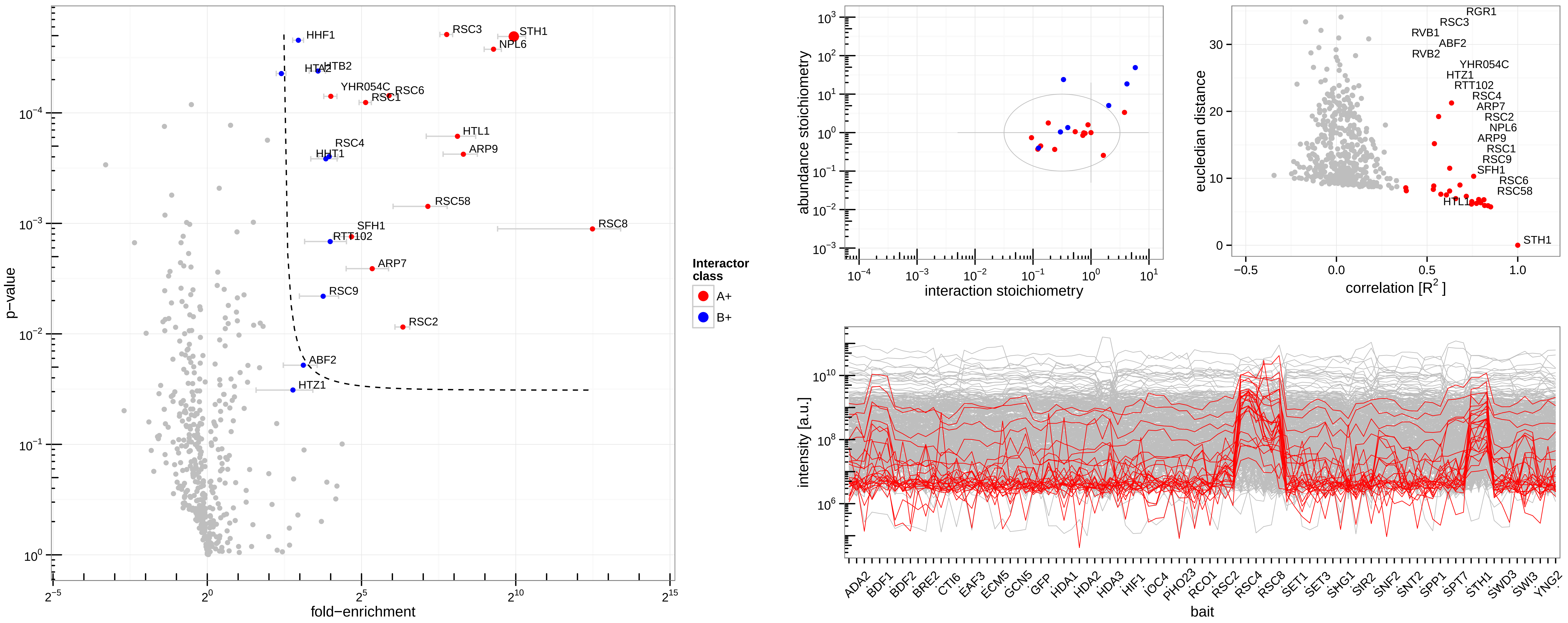

	ABF2	ARP7	ARP9	HHF1	HHT1	HTA2	HTB2	HTL1	HTZ1	NPL6	RSC1	RSC2	RSC3	RSC4	RSC58	RSC6	RSC8	RSC9	RTT102	SFH1	STH1	YHR054C
significance	2.7	5.1	8.2	5.1	4.6	4.6	5.1	8.1	2.3	9.5	5.8	5.7	8.2	4.7	6.9	6.4	12.0	3.6	4.1	4.7	10.0	5.1
interactor class	B+	A+	A+	B+	B+	B+	B+	A+	B+	A+	A+	A+	A+	B+	A+	A+	A+	B+	B+	A+	A+	A+

**bait**	SWD3
**experiment**	14 minutes

**interactors**	4
**recovery**	43 %

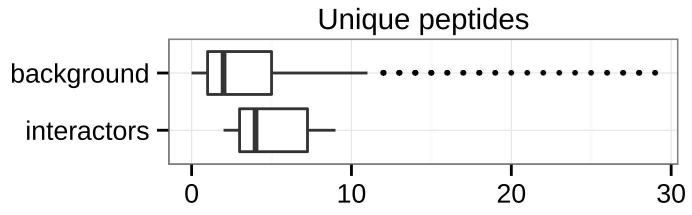

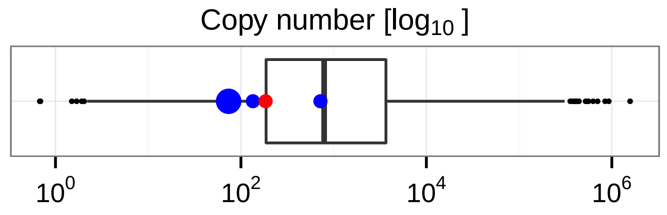

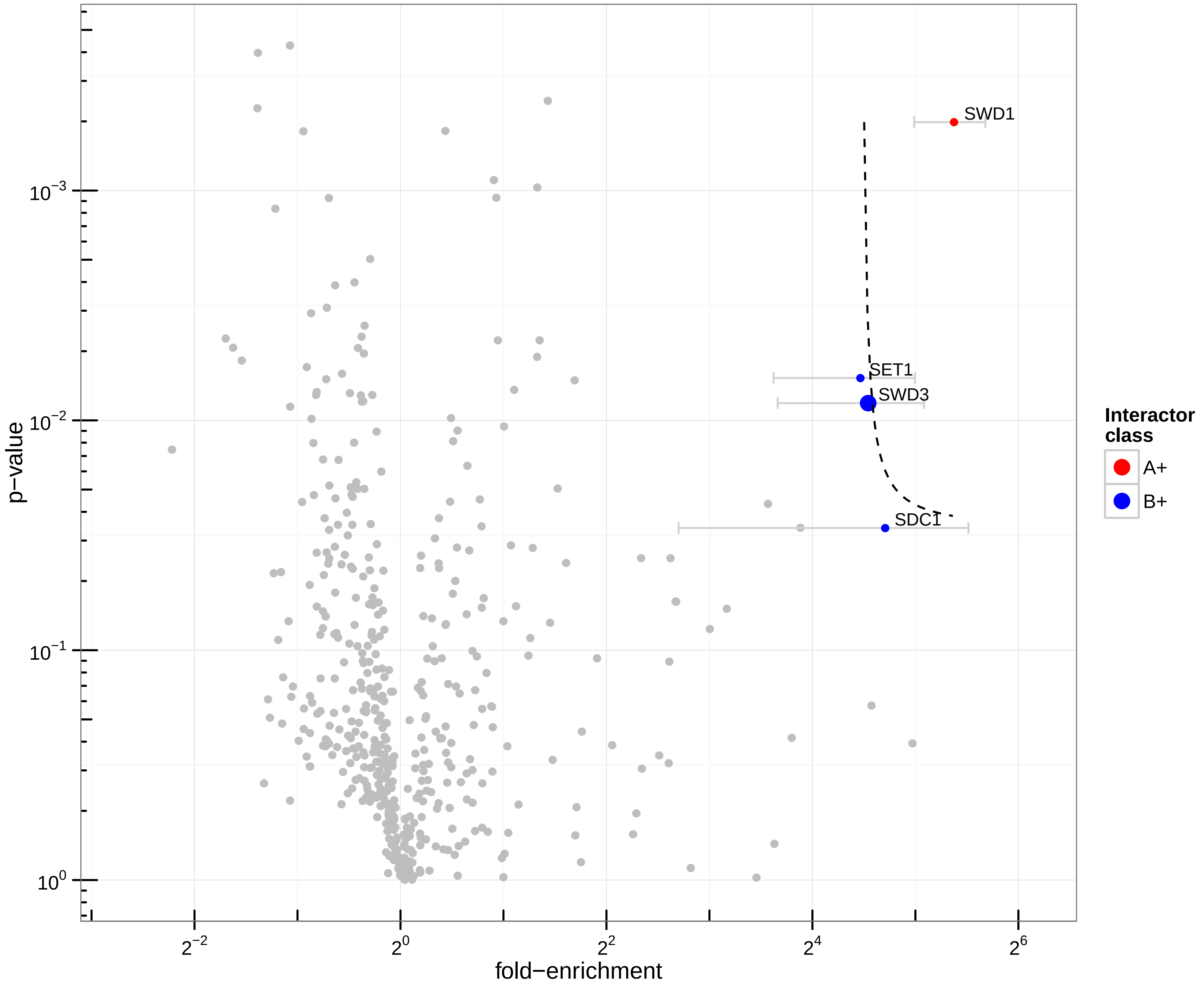

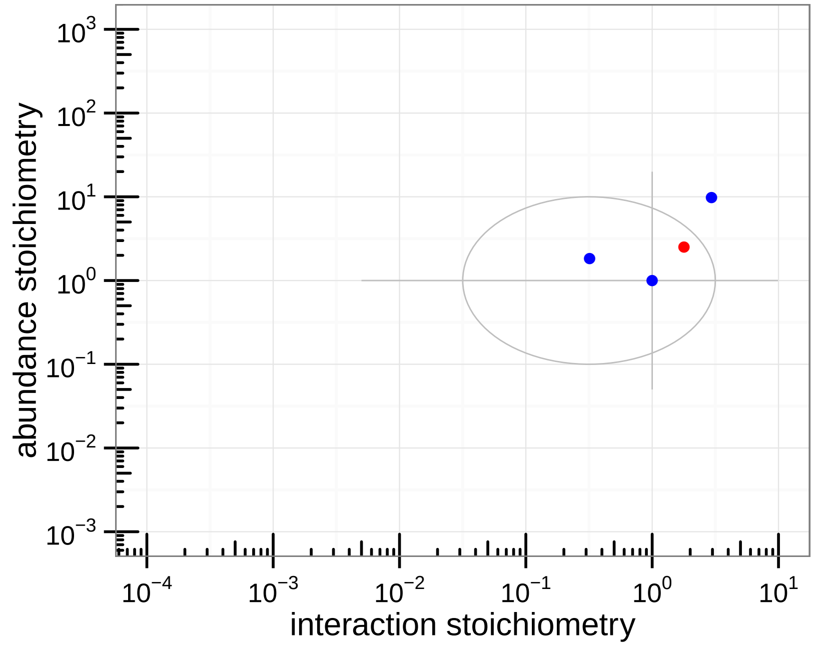

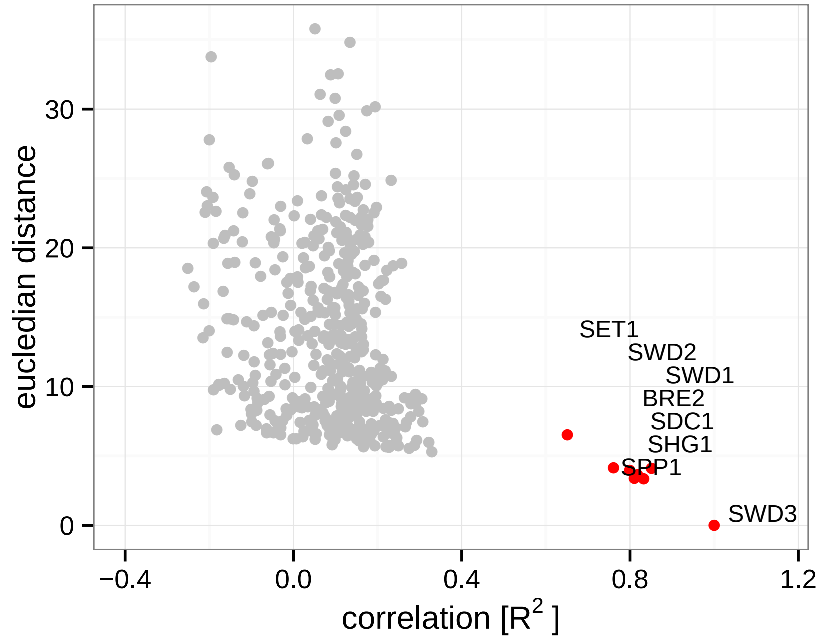

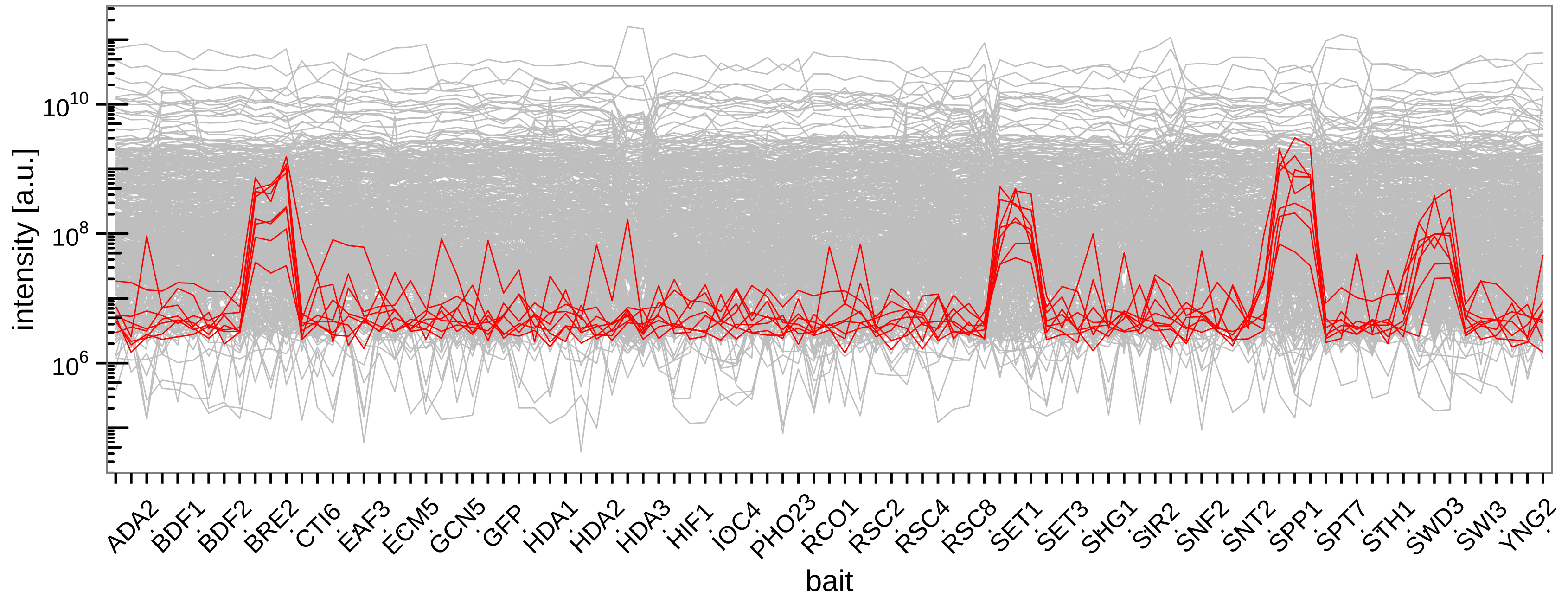

	SDC1	SET1	SWD1	SWD3
significance	4.0	4.1	5.5	4.1
interactor class	B+	B+	A+	B+

**bait**	SWI3
**experiment**	14 minutes

**interactors**	10
**recovery**	64 %

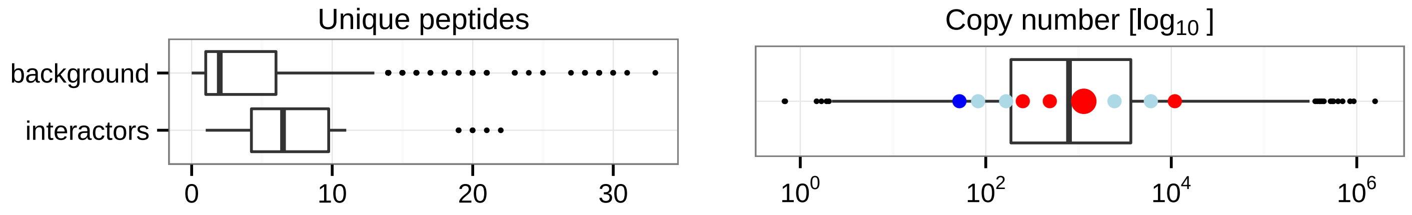

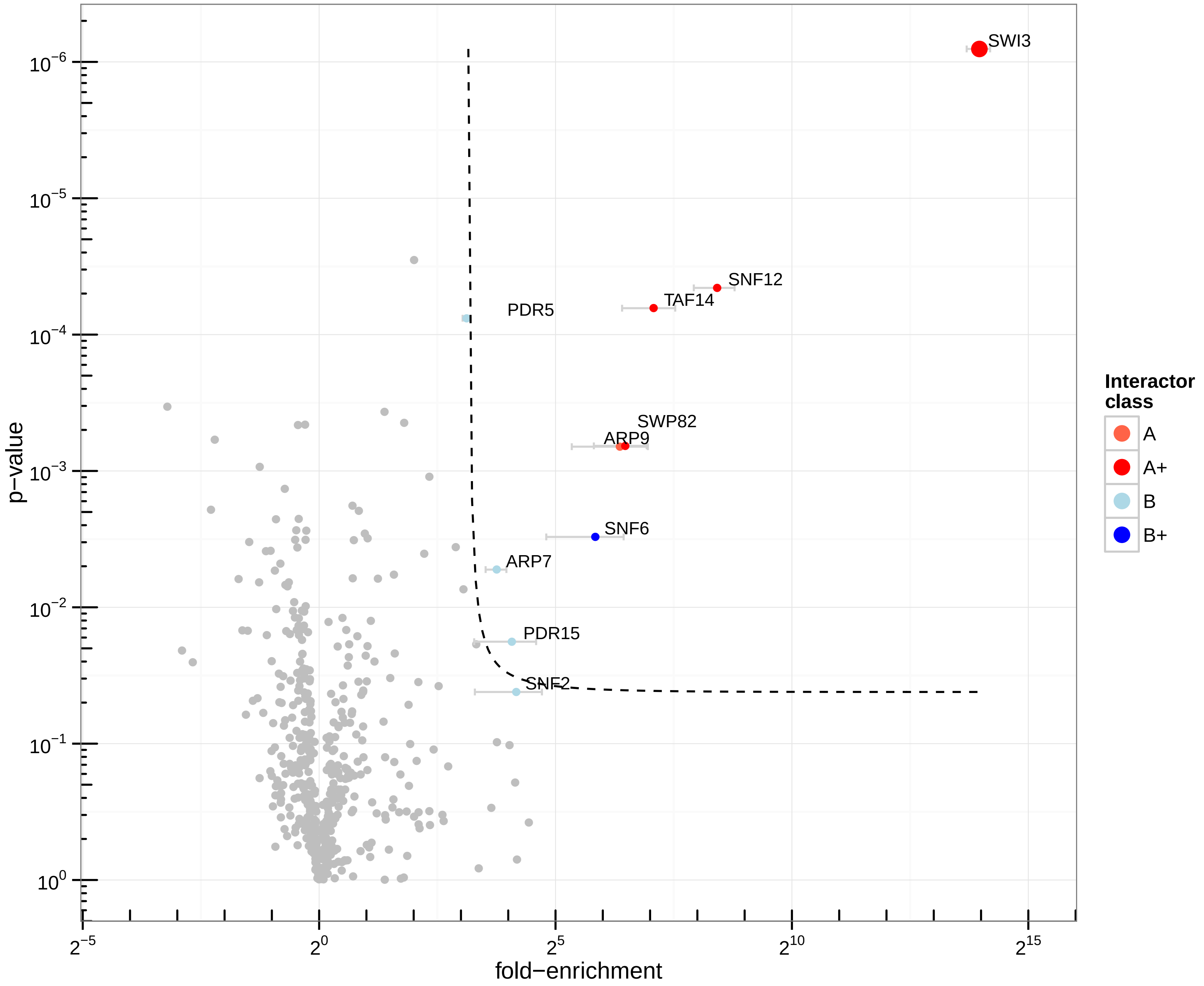

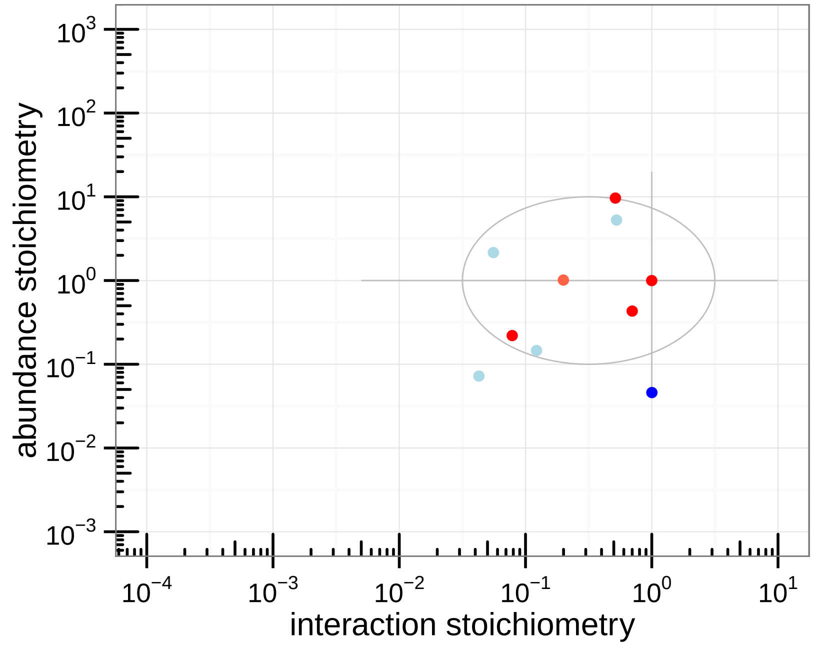

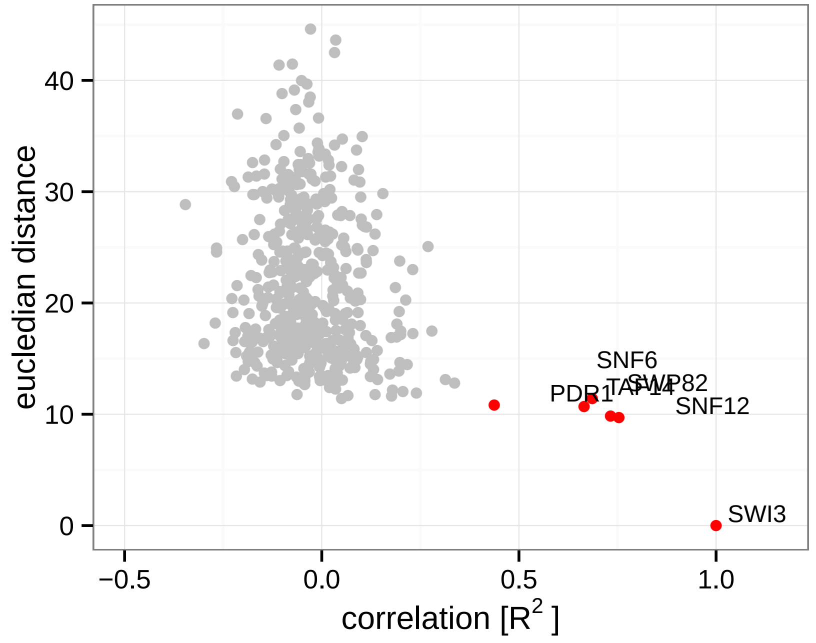

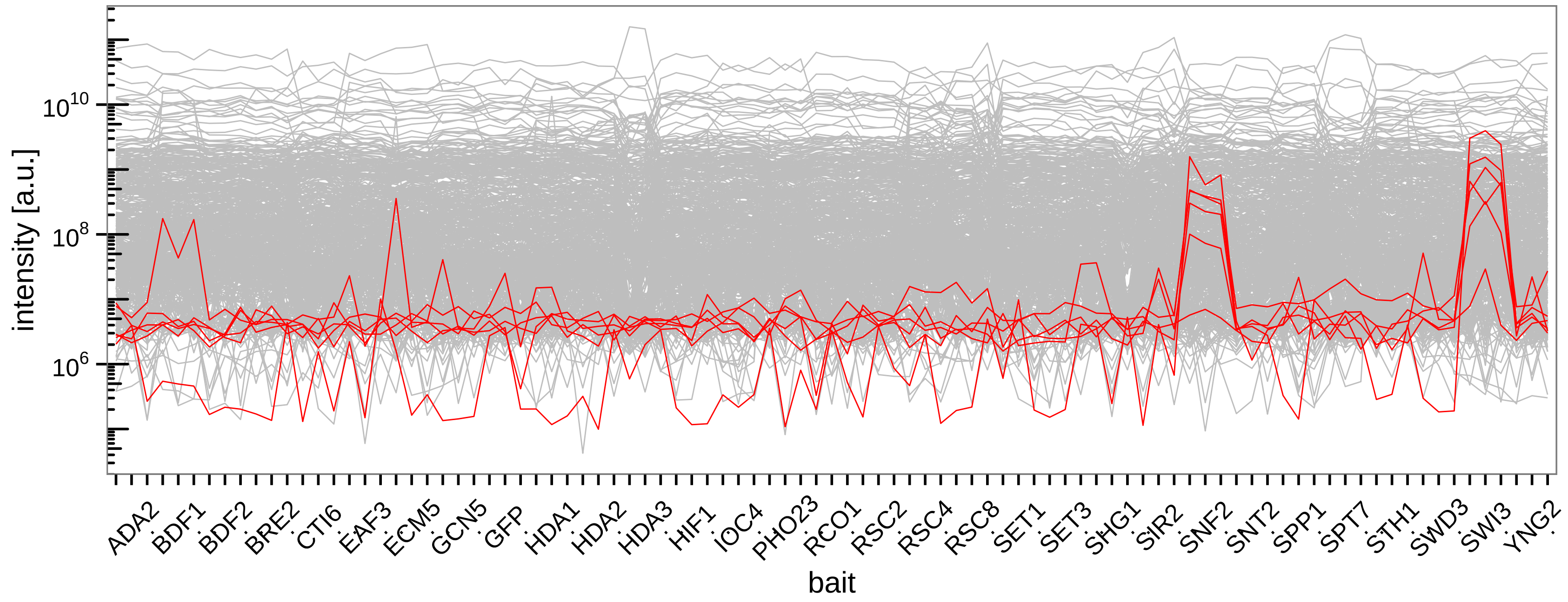

	ARP9	SNF12	SNF6	SWI3	SWP82	TAF14
significance	6.2	8.6	5.5	14.0	6.3	7.4
interactor class	A	A+	B+	A+	A+	A+

**bait**	YNG2	**interactors**	13
**experiment**	14 minutes	**recovery**	54 %

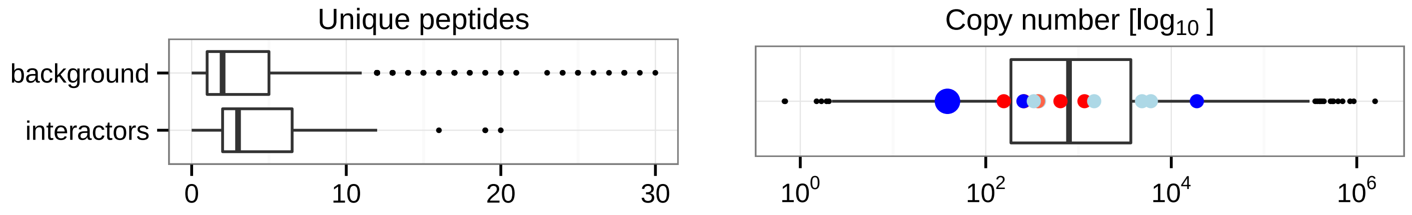

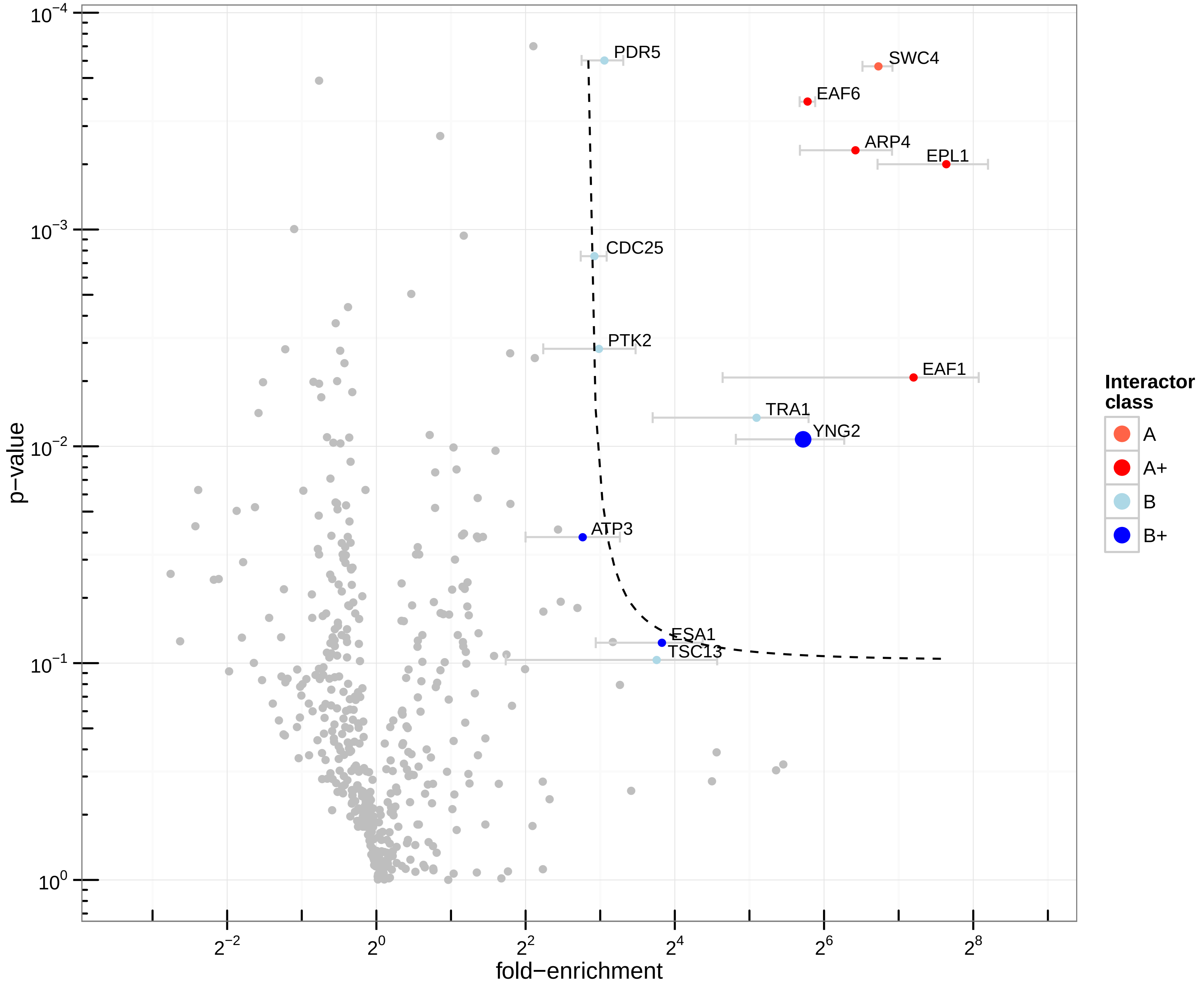

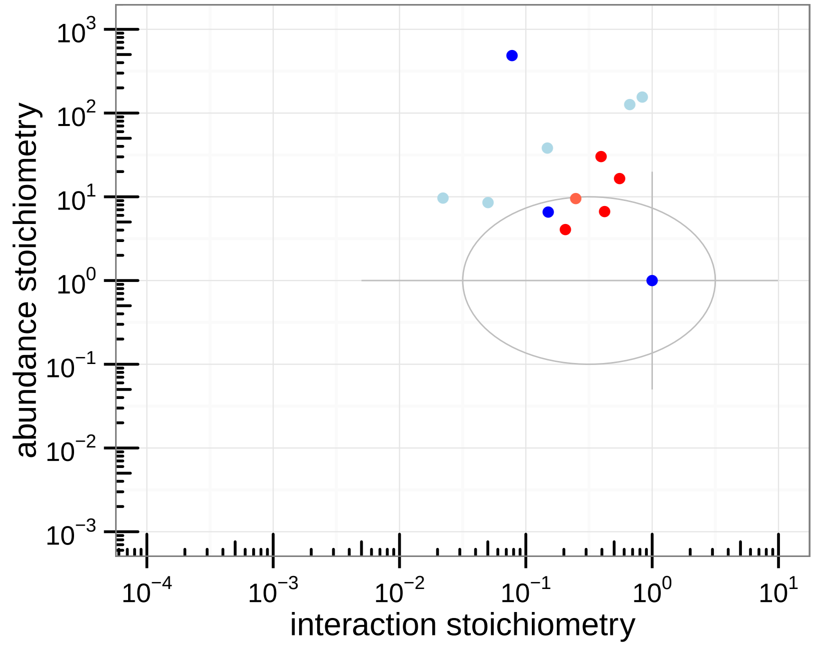

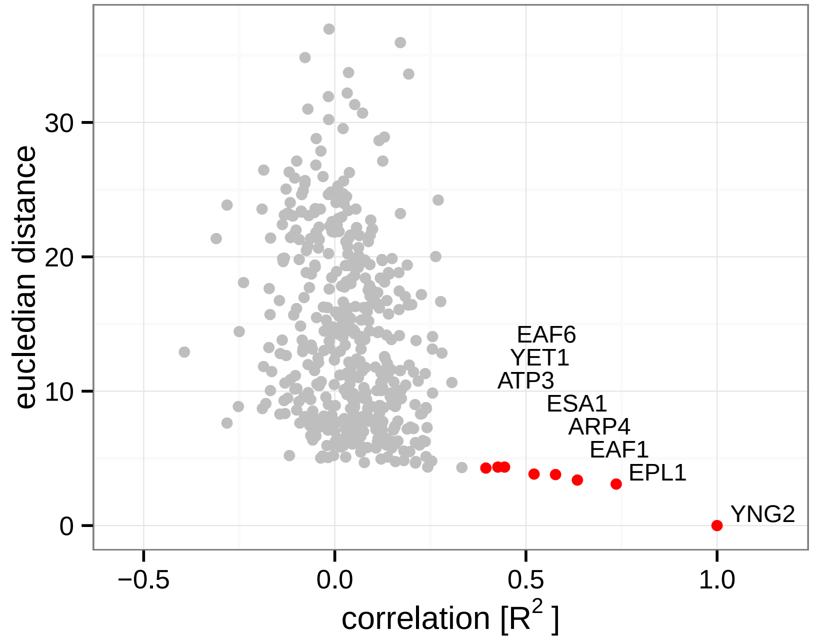

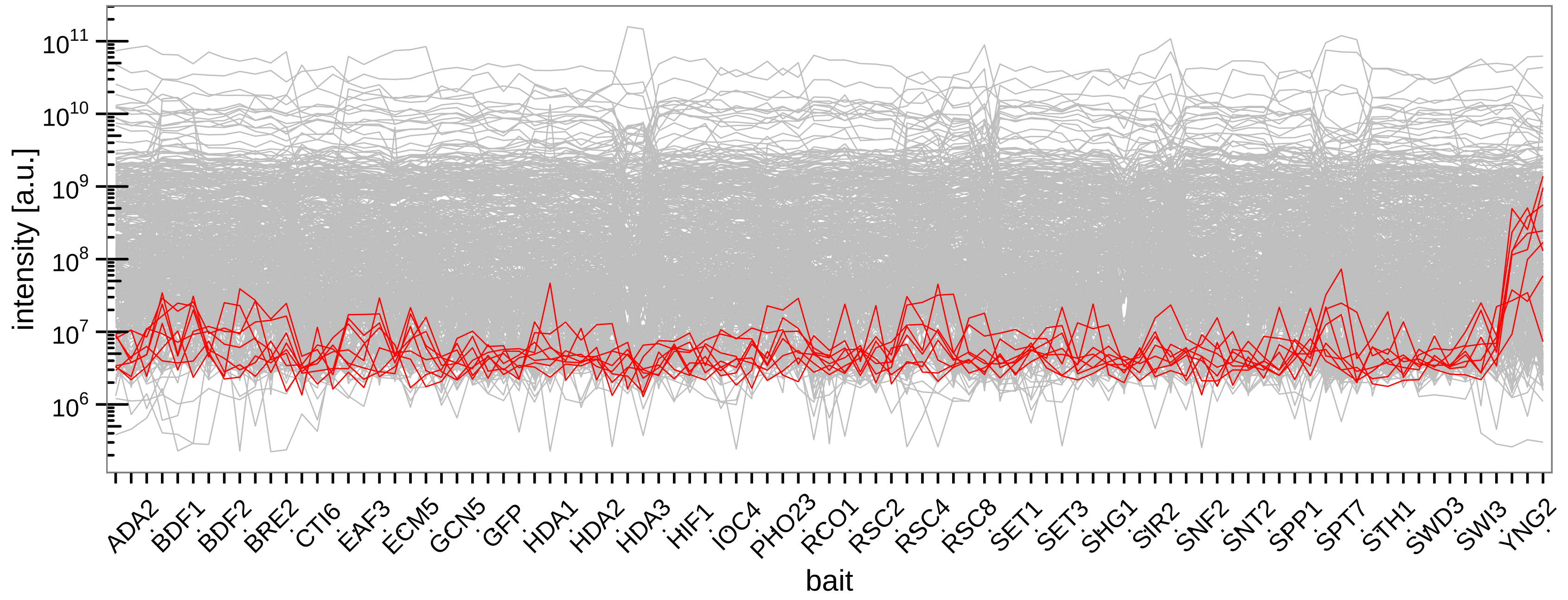

	ARP4	ATP3	EAF1	EAF6	EPL1	ESA1	SWC4	YNG2
significance	6.4	2.4	6.6	6.0	7.4	3.0	6.8	5.1
interactor class	A+	B+	A+	A+	A+	B+	A	B+
