# Supplementary material for: A Double-Barrel Liquid Chromatography-Tandem Mass Spectrometry (LC-MS/MS) System to Quantify 96 Interactomes per Day
Source: Mol Cell Proteomics. 2015 Apr 17;14(7):2030–41. doi: 10.1074/mcp.O115.049460 (PMC4587330; doi:10.1074/mcp.O115.049460)
Supplement: Supplemental Data [file supp_O115.049460_mcp.O115.049460-3.pdf]

Ultra−high Throughput LC−MS/MS Protein Interaction Analysis – Processing 96 Pull−downs in a Single Day

Experiment: 27 minutes

F. Hosp*, R.A. Scheltema*, H.C. Eberl*, N.A. Kulak*, E.C. Keilhauer, K. Mayr and M. Mann

Missing values

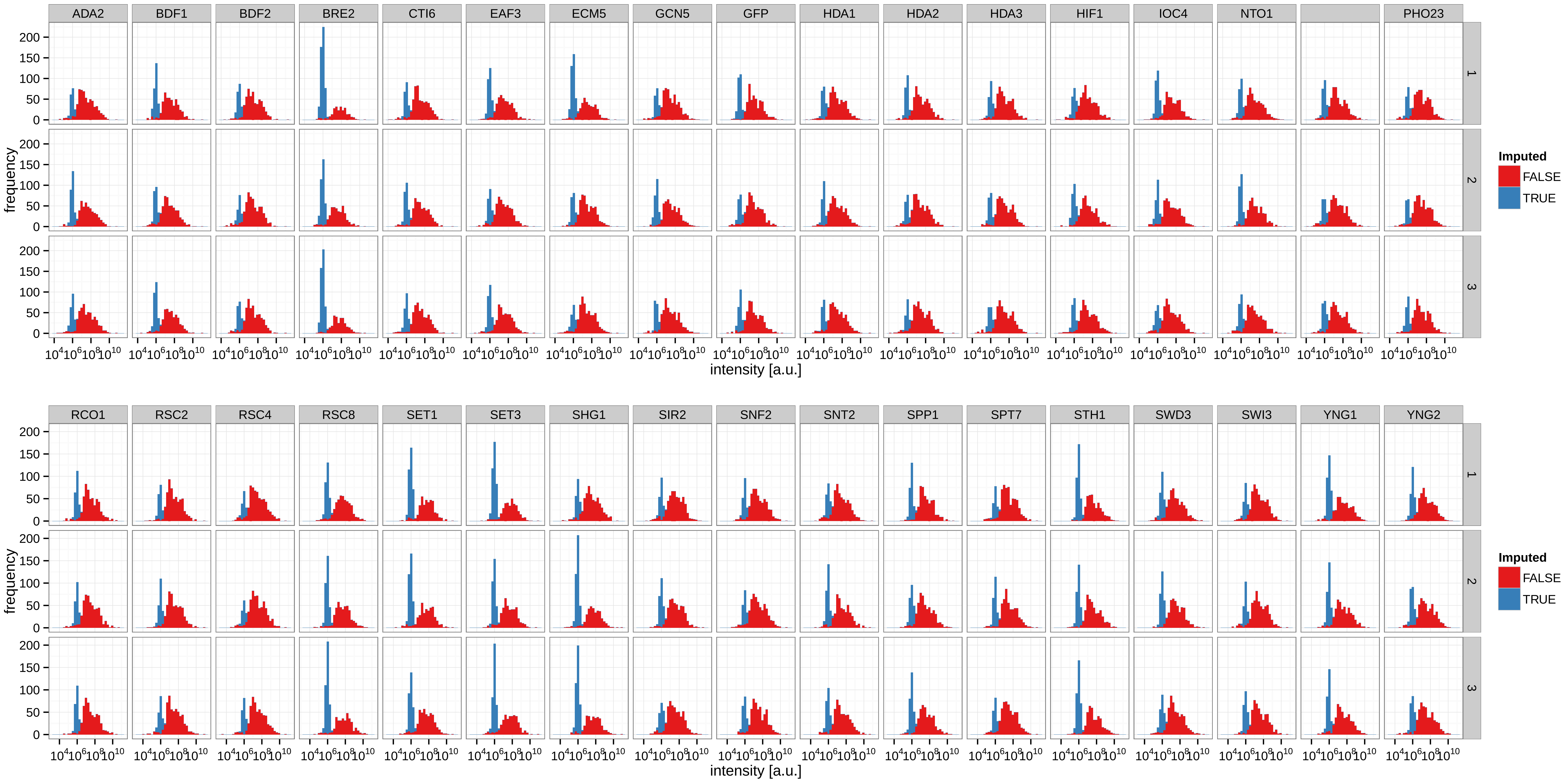

**bait**	ADA2
**experiment**	27 minutes

**interactors**	26
**recovery**	91 %

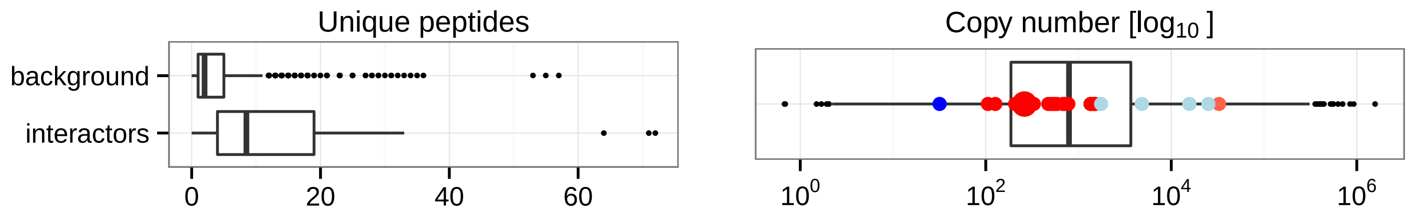

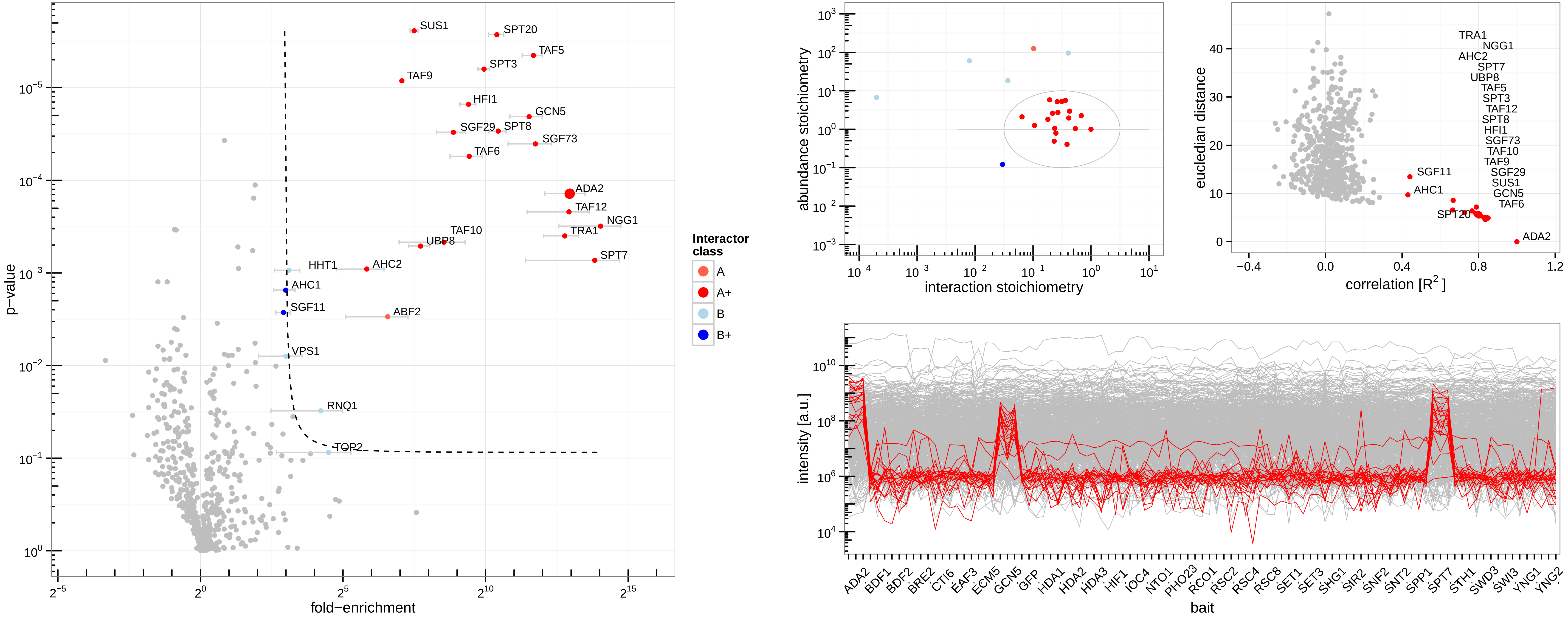

	ABF2	ADA2	AHC1	AHC2	GCN5	HFI1	NGG1	SGF11	SGF29	SGF73	SPT20	SPT3	SPT7	SPT8	SUS1	TAF10	TAF12	TAF5	TAF6	TAF9	TRA1	UBP8
significance	6.1	13.0	3.4	5.7	12.0	9.7	13.0	3.2	9.1	12.0	11.0	10.0	13.0	10.0	8.6	8.2	12.0	12.0	9.4	7.9	12.0	7.5
interactor class	A	A+	B+	A+	A+	A+	A+	B+	A+	A+	A+	A+	A+	A+	A+	A+	A+	A+	A+	A+	A+	A+

**bait**	BDF1		**interactors**	5
**experiment**	27 minutes		**recovery**	0 %

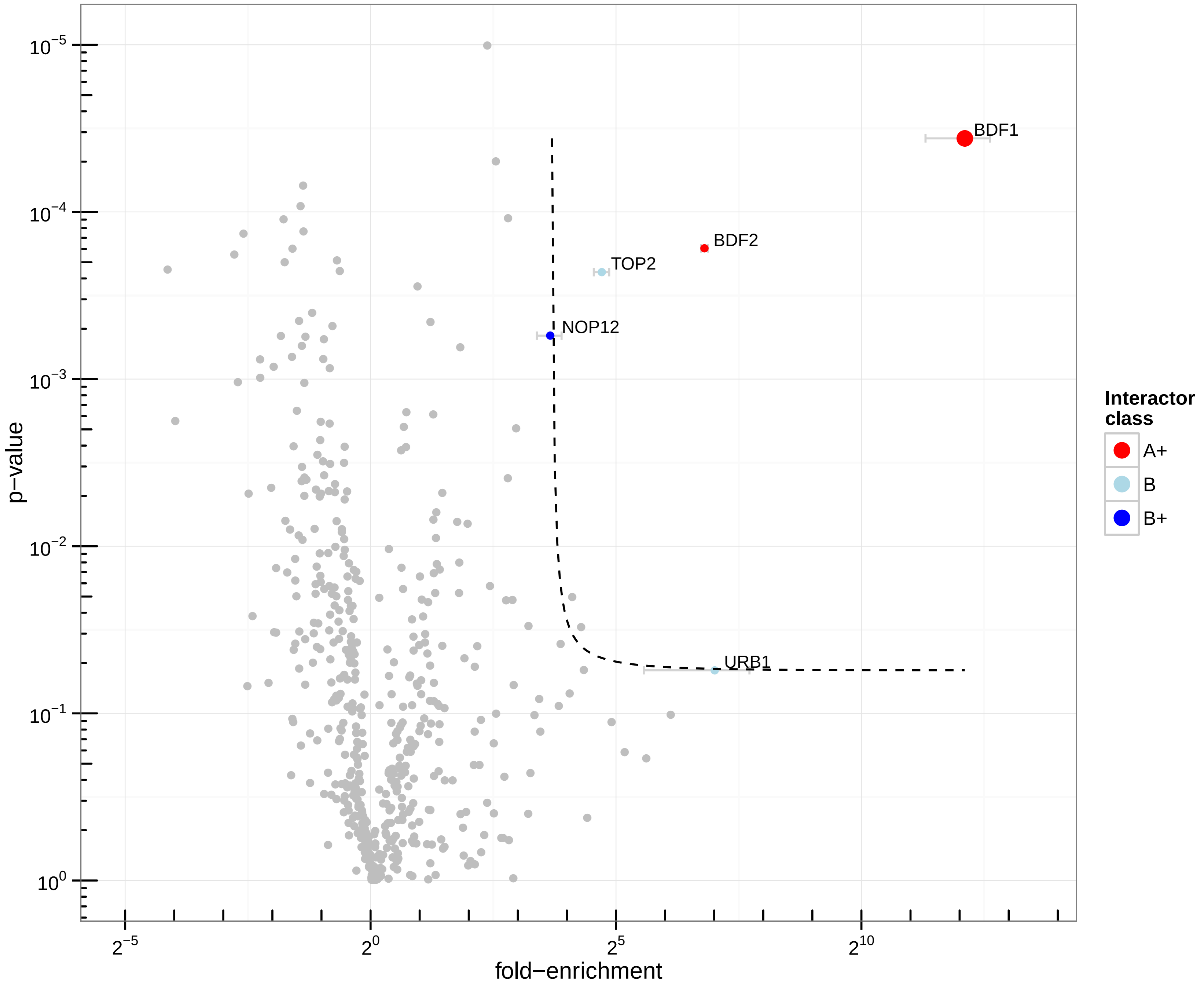

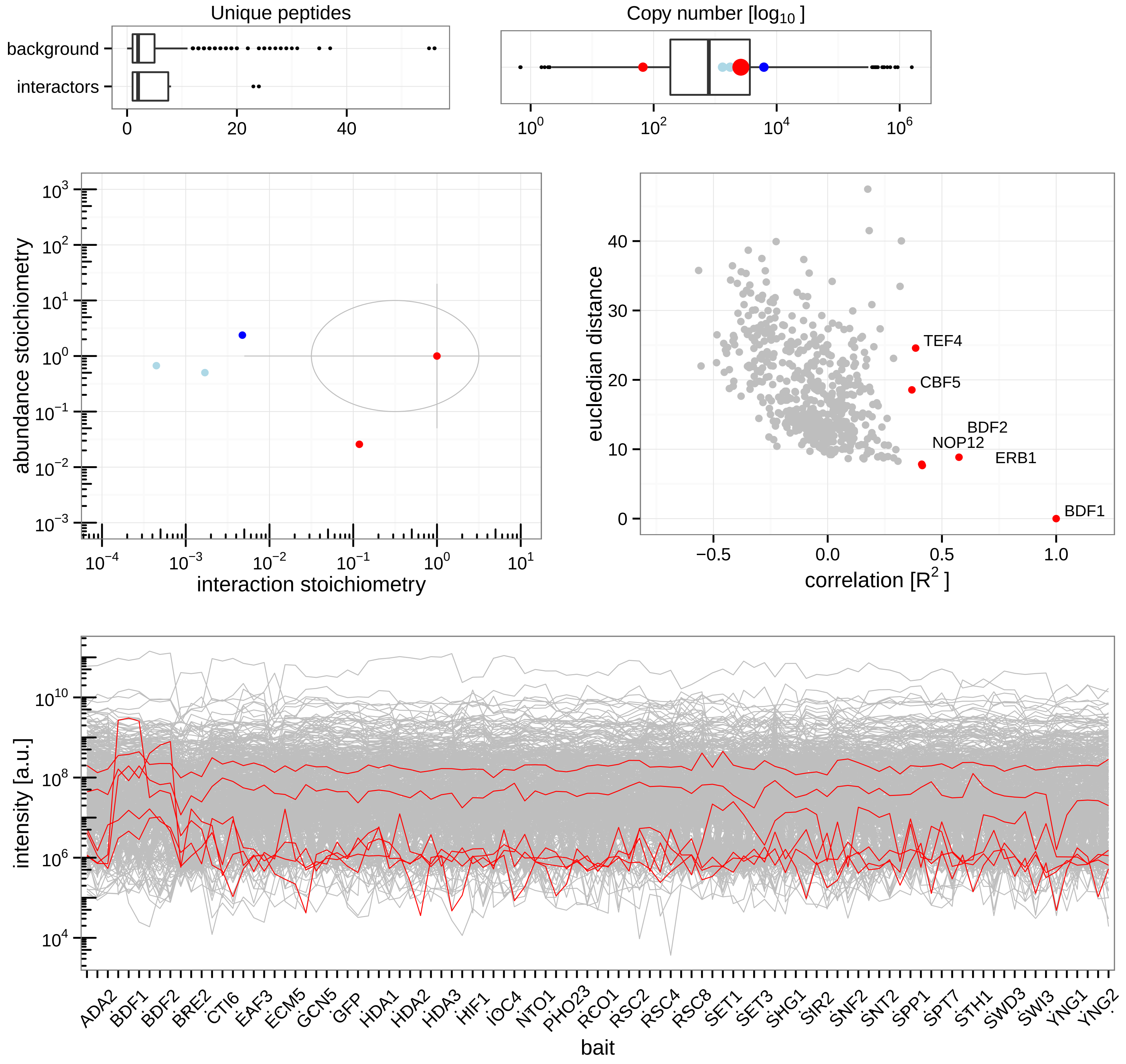

	BDF1	BDF2	NOP12
significance	12.0	6.9	4.2
interactor class	A+	A+	B+

**bait**	BDF2
**experiment**	27 minutes

**interactors**	18
**recovery**	0 %

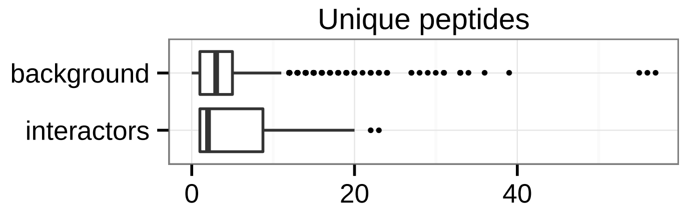

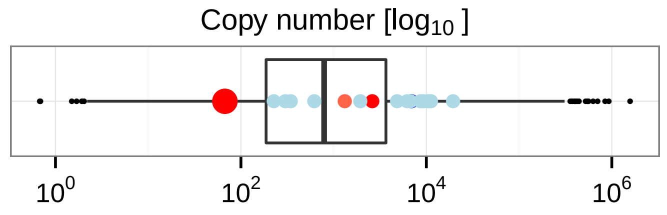

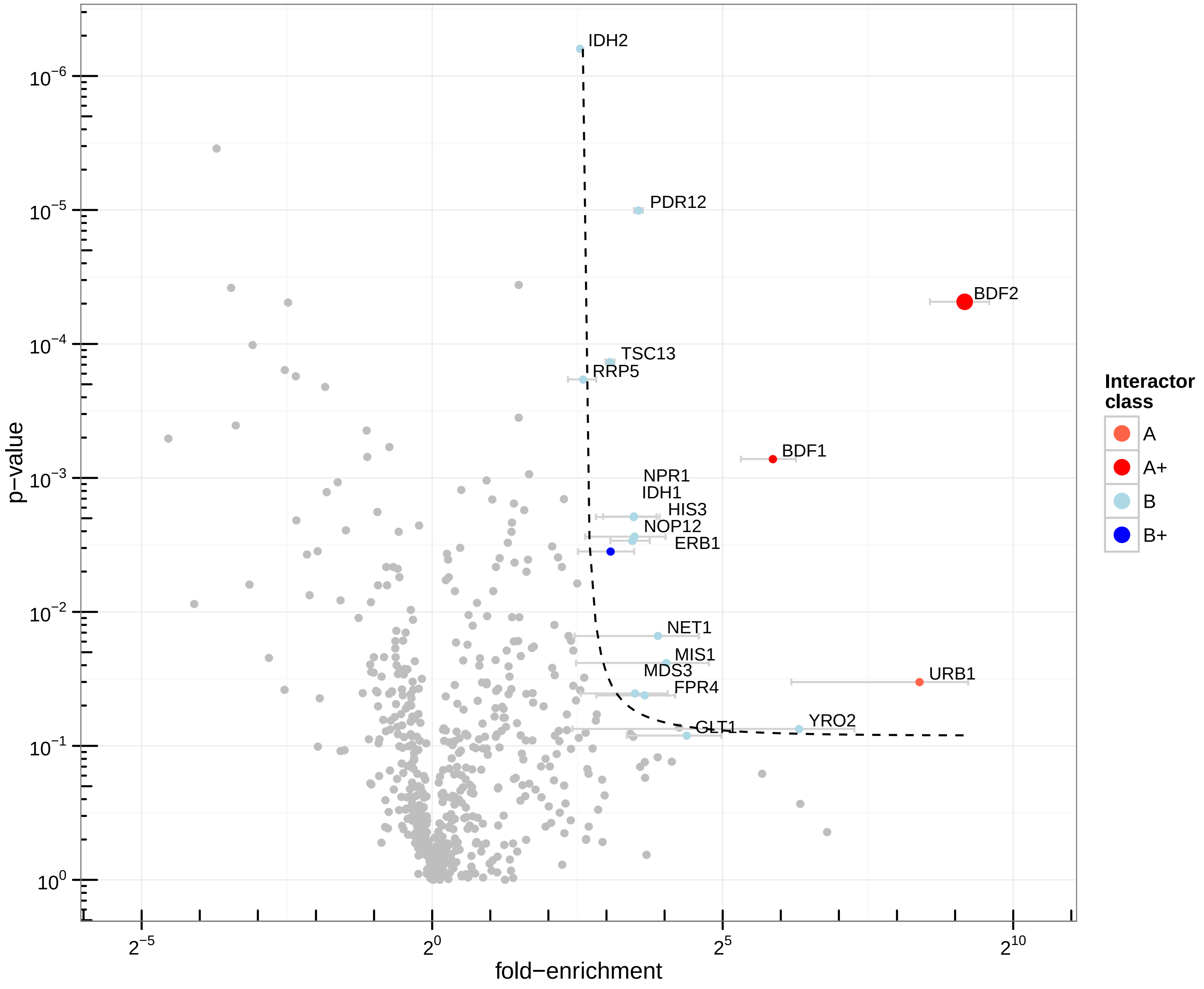

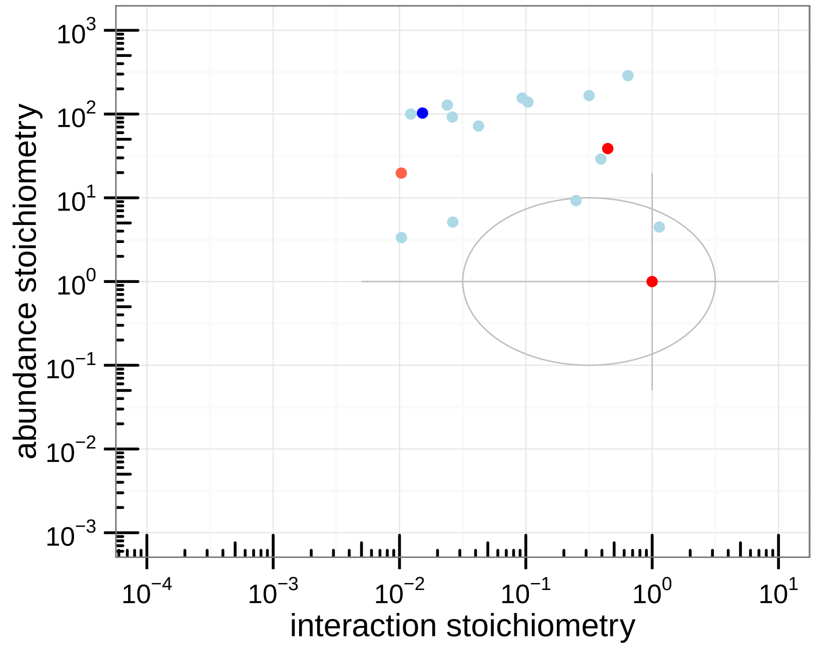

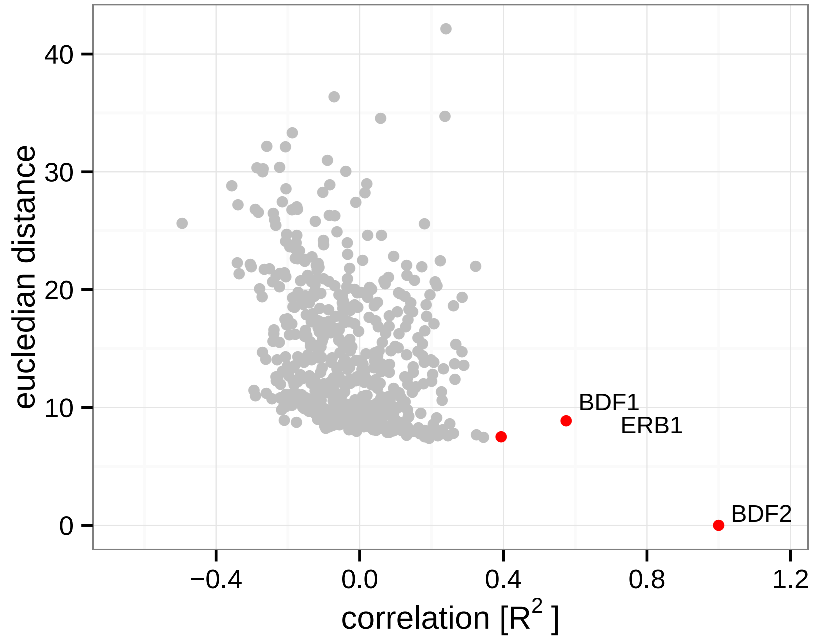

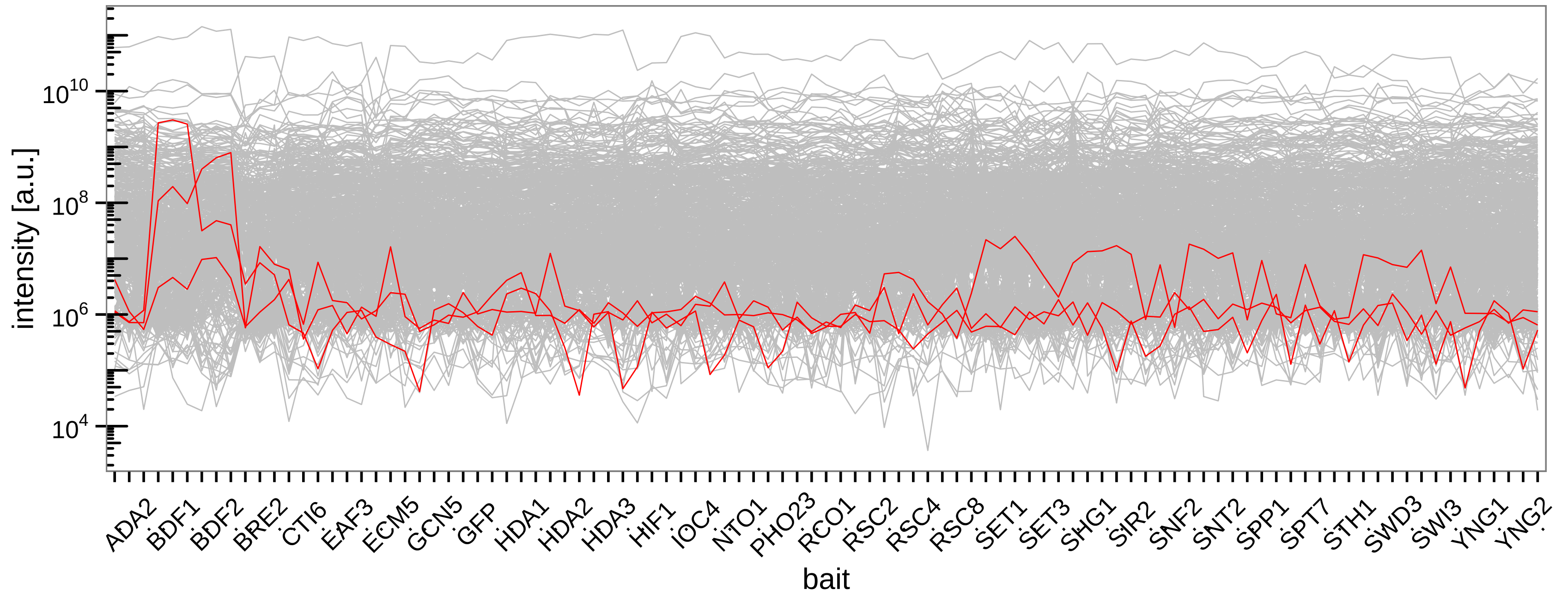

	BDF1	BDF2	ERB1	URB1
significance	5.8	9.2	3.2	7.5
interactor class	A+	A+	B+	A

bait	BRE2
experiment	27 minutes

interactors	8
recovery	0 %

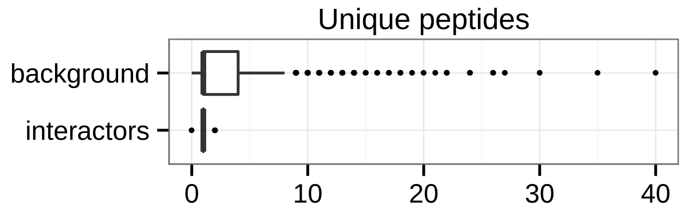

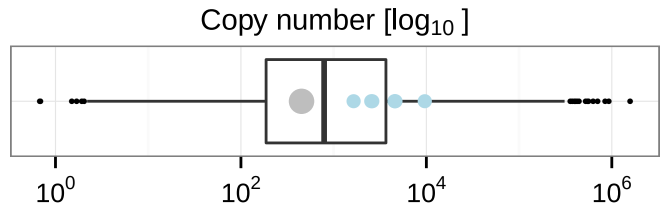

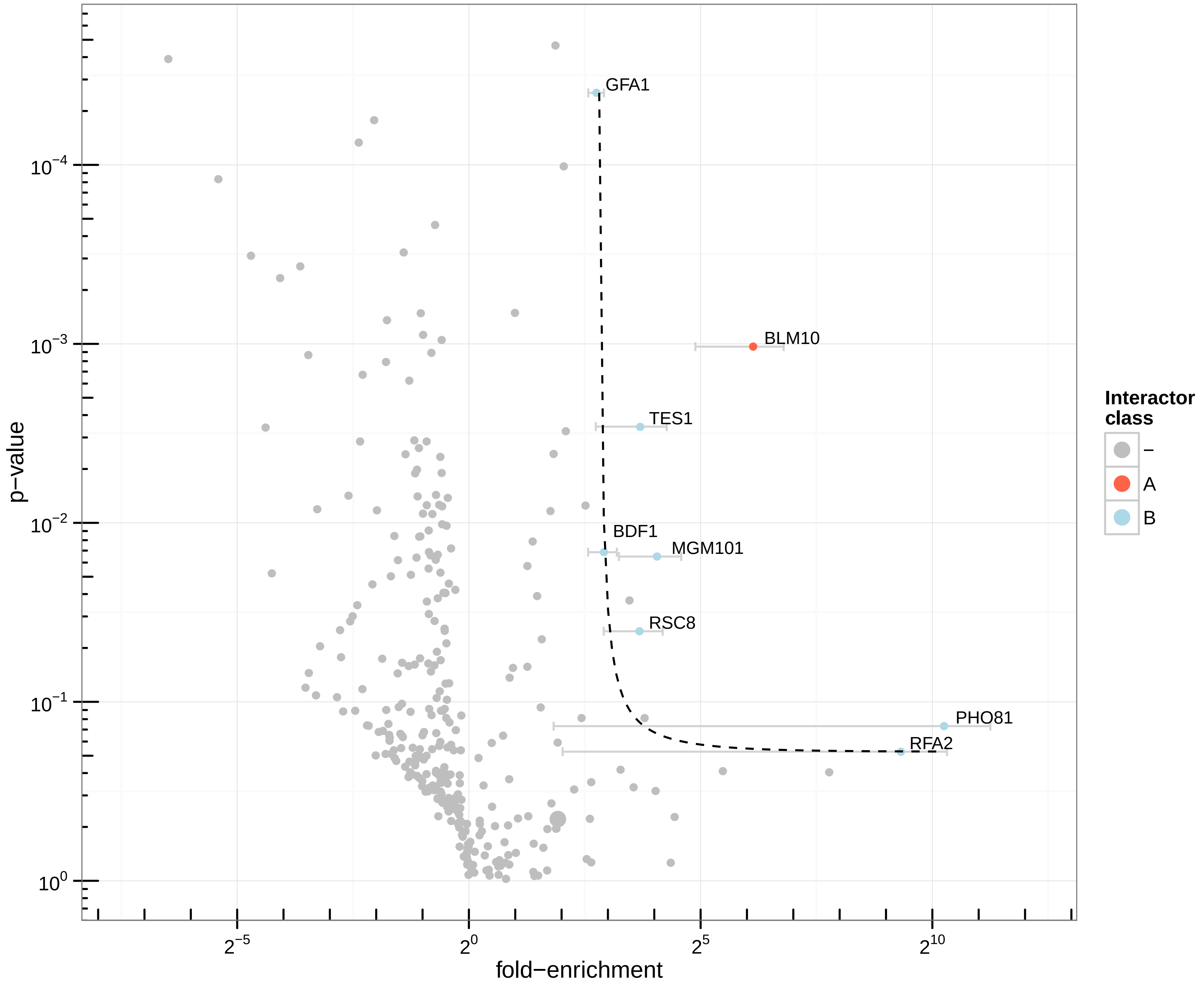

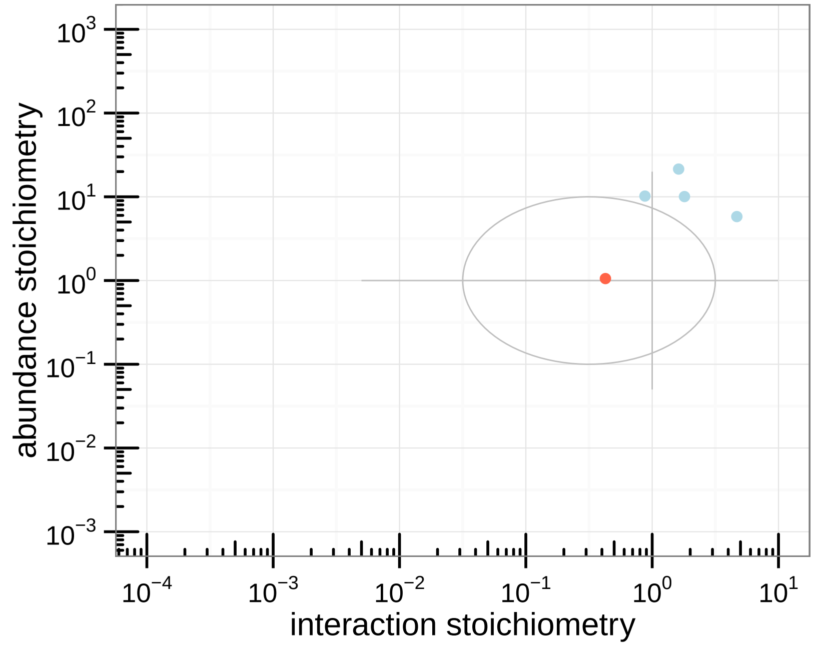

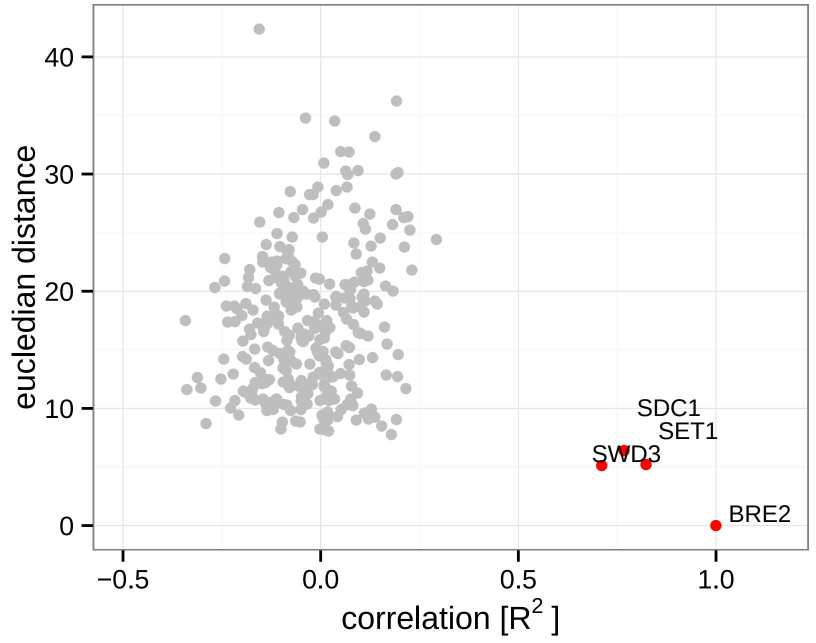

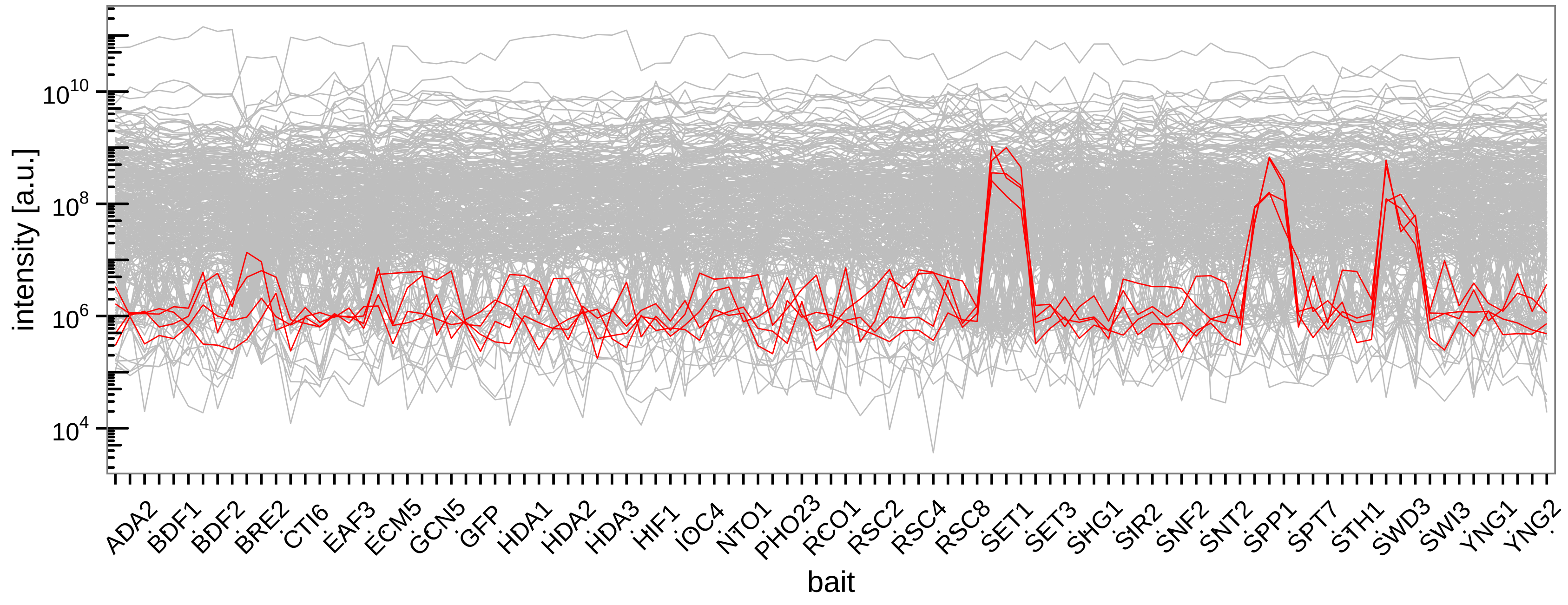

	BLM10
significance	5.9
interactor class	A

**bait**	CTI6
**experiment**	27 minutes

**interactors**	20
**recovery**	48 %

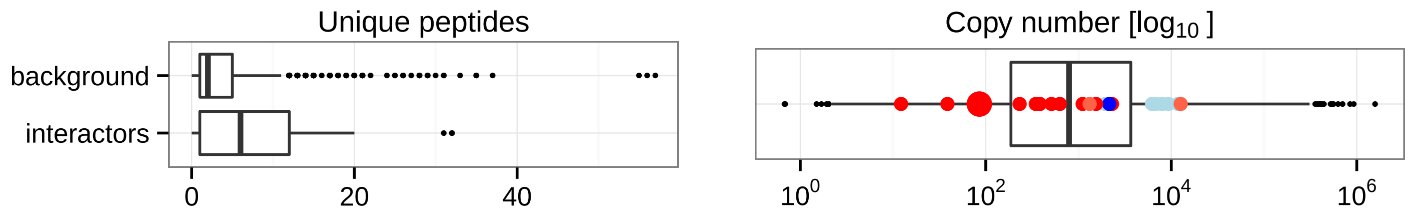

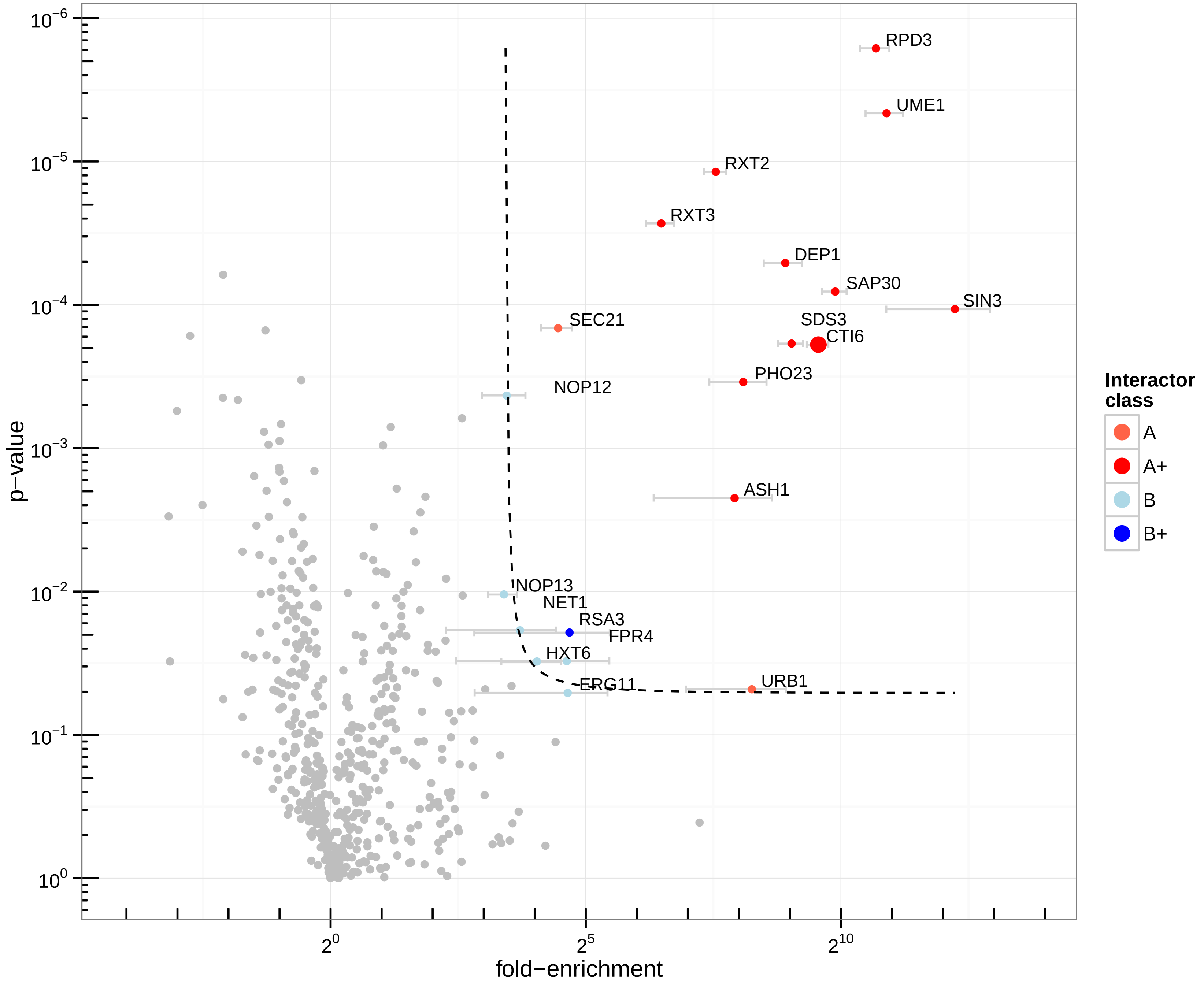

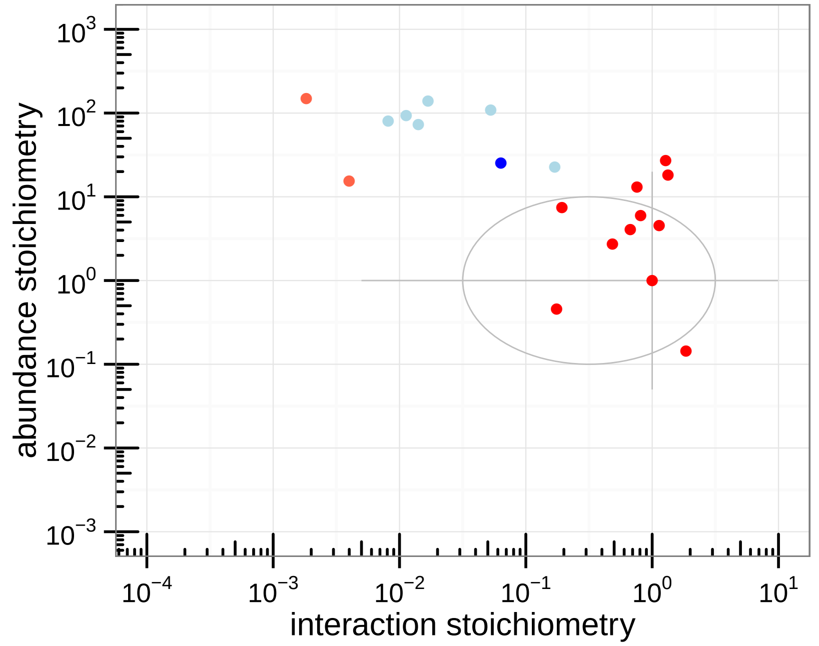

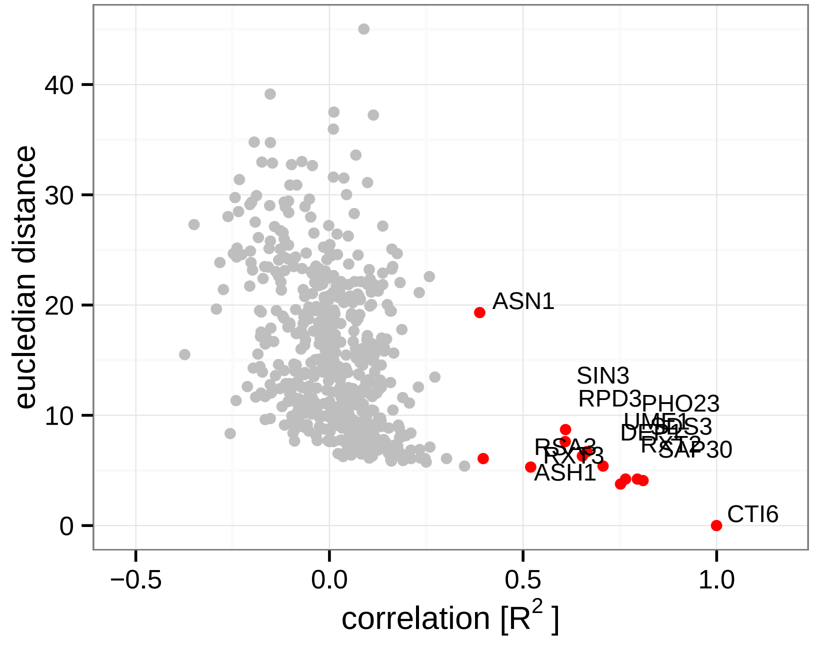

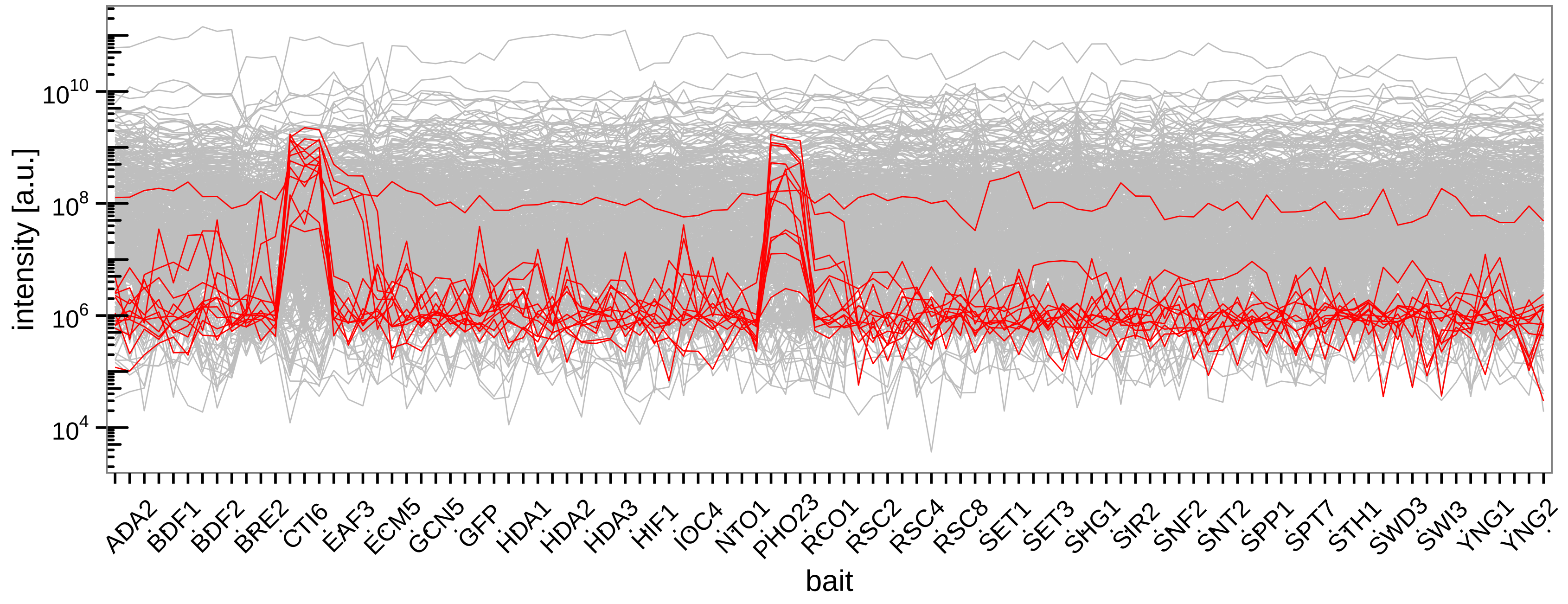

	ASH1	CTI6	DEP1	PHO23	RPD3	RSA3	RXT2	RXT3	SAP30	SDS3	SEC21	SIN3	UME1	URB1
significance	7.4	9.3	9.0	7.9	11.0	4.1	8.2	7.1	9.8	8.9	5.2	12.0	11.0	7.4
interactor class	A+	A+	A+	A+	A+	B+	A+	A+	A+	A+	A	A+	A+	A

**bait**	EAF3
**experiment**	27 minutes

**interactors**	5
**recovery**	12 %

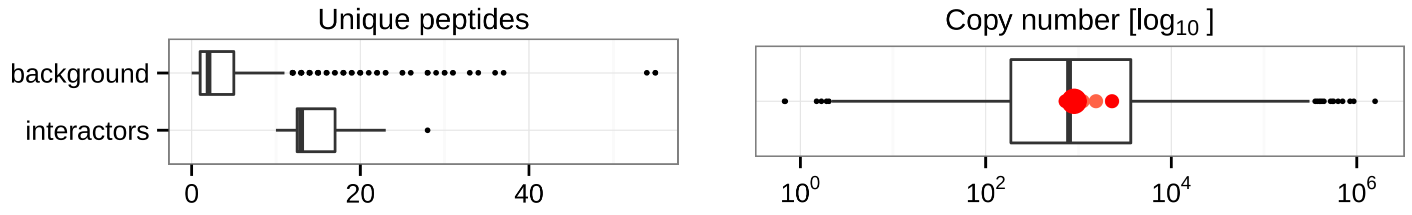

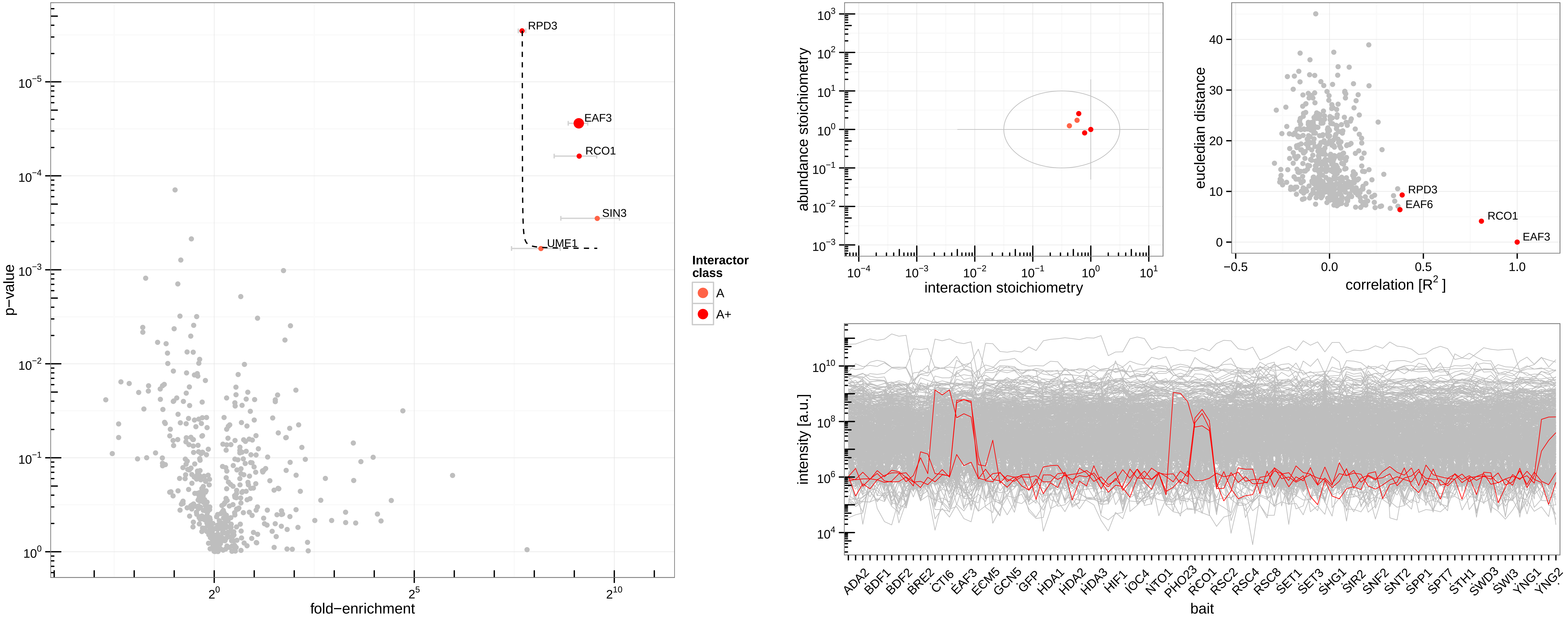

	EAF3	RCO1	RPD3	SIN3	UME1
significance	9.3	9.2	8.7	9.3	7.9
interactor class	A+	A+	A+	A	A

bait	ECM5
experiment	27 minutes

interactors	0
recovery	0 %

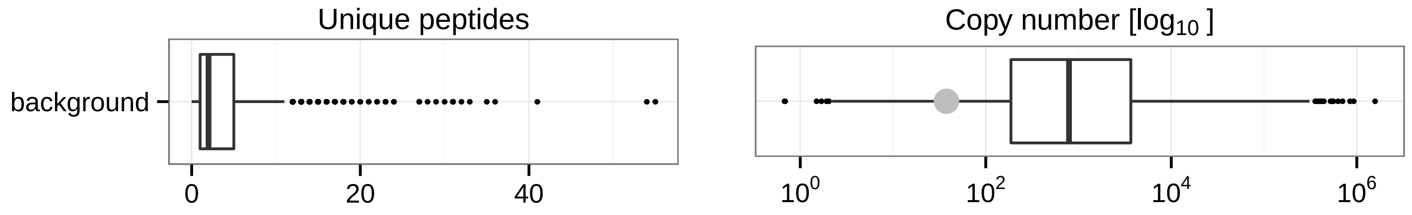

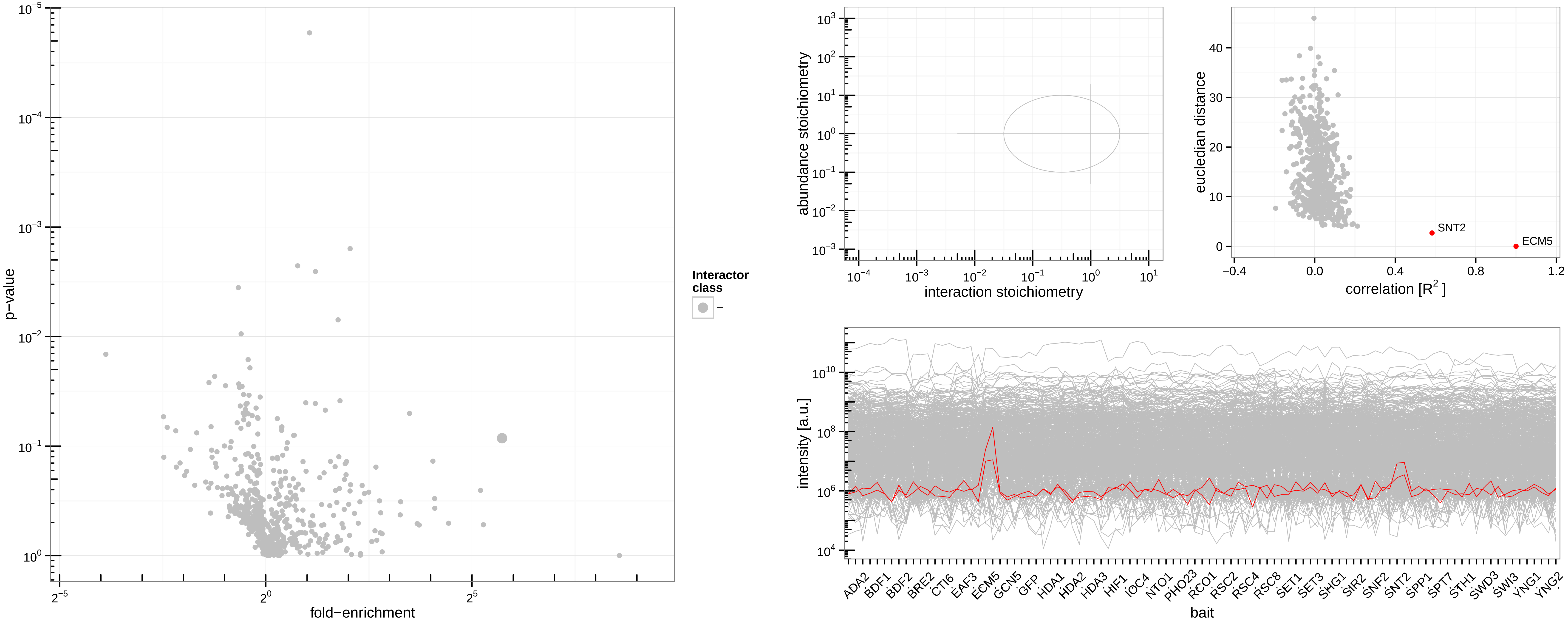

significance

interactor class

**bait**	GCN5
**experiment**	27 minutes

**interactors**	17
**recovery**	71 %

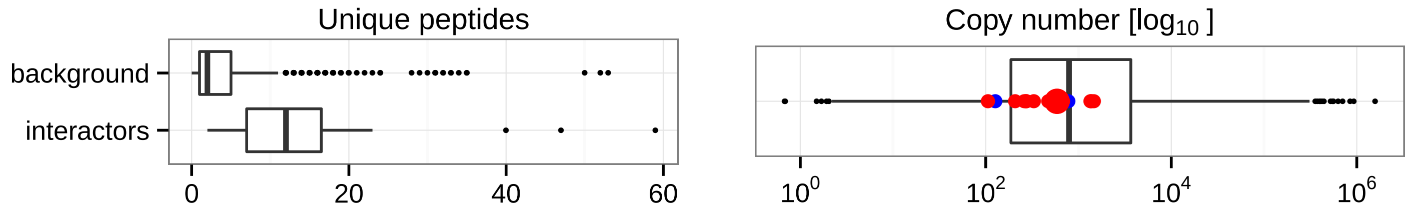

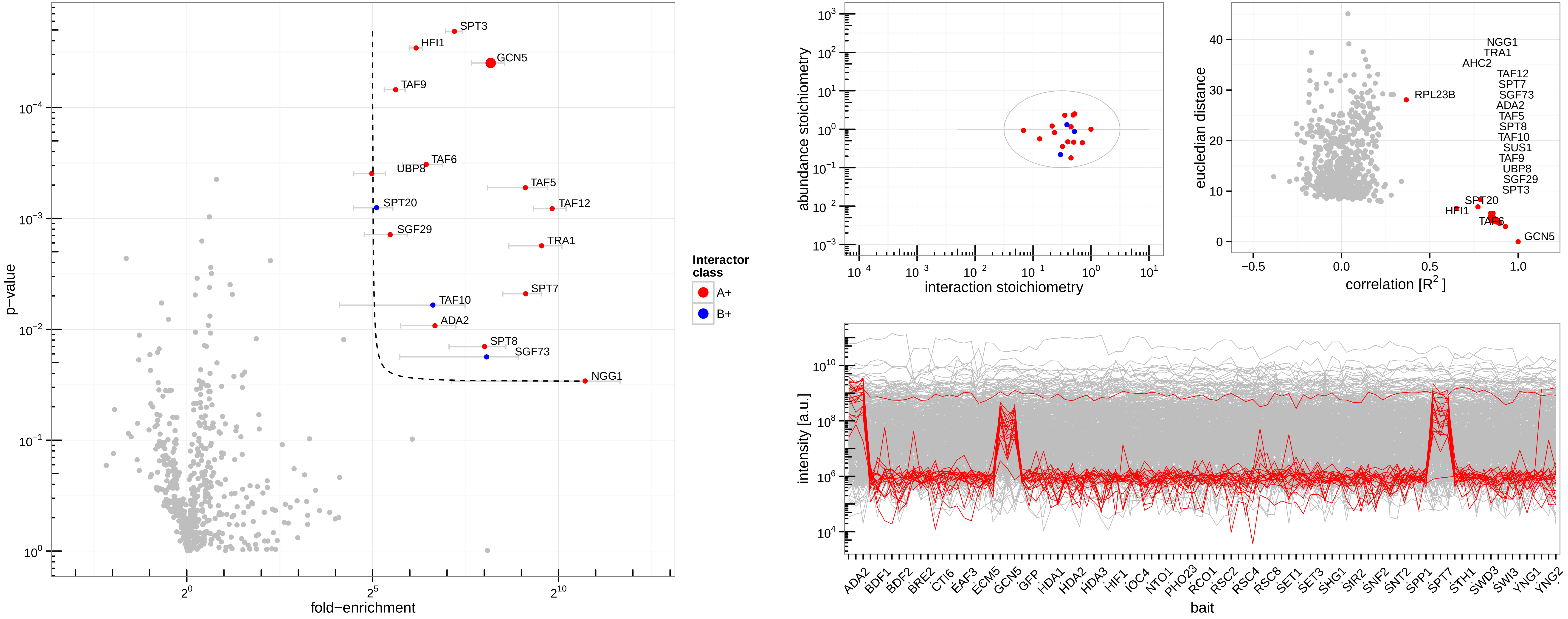

	ADA2	GCN5	HFI1	NGG1	SGF29	SGF73	SPT20	SPT3	SPT7	SPT8	TAF10	TAF12	TAF5	TAF6	TAF9	TRA1	UBP8
significance	6.0	8.4	6.9	9.8	5.3	7.3	5.1	7.8	8.4	7.3	6.0	9.4	8.7	6.5	6.2	9.0	5.2
interactor class	A+	A+	A+	A+	A+	B+	B+	A+	A+	A+	B+	A+	A+	A+	A+	A+	A+

**bait**	GFP
**experiment**	27 minutes

**interactors**	0
**recovery**	NaN %

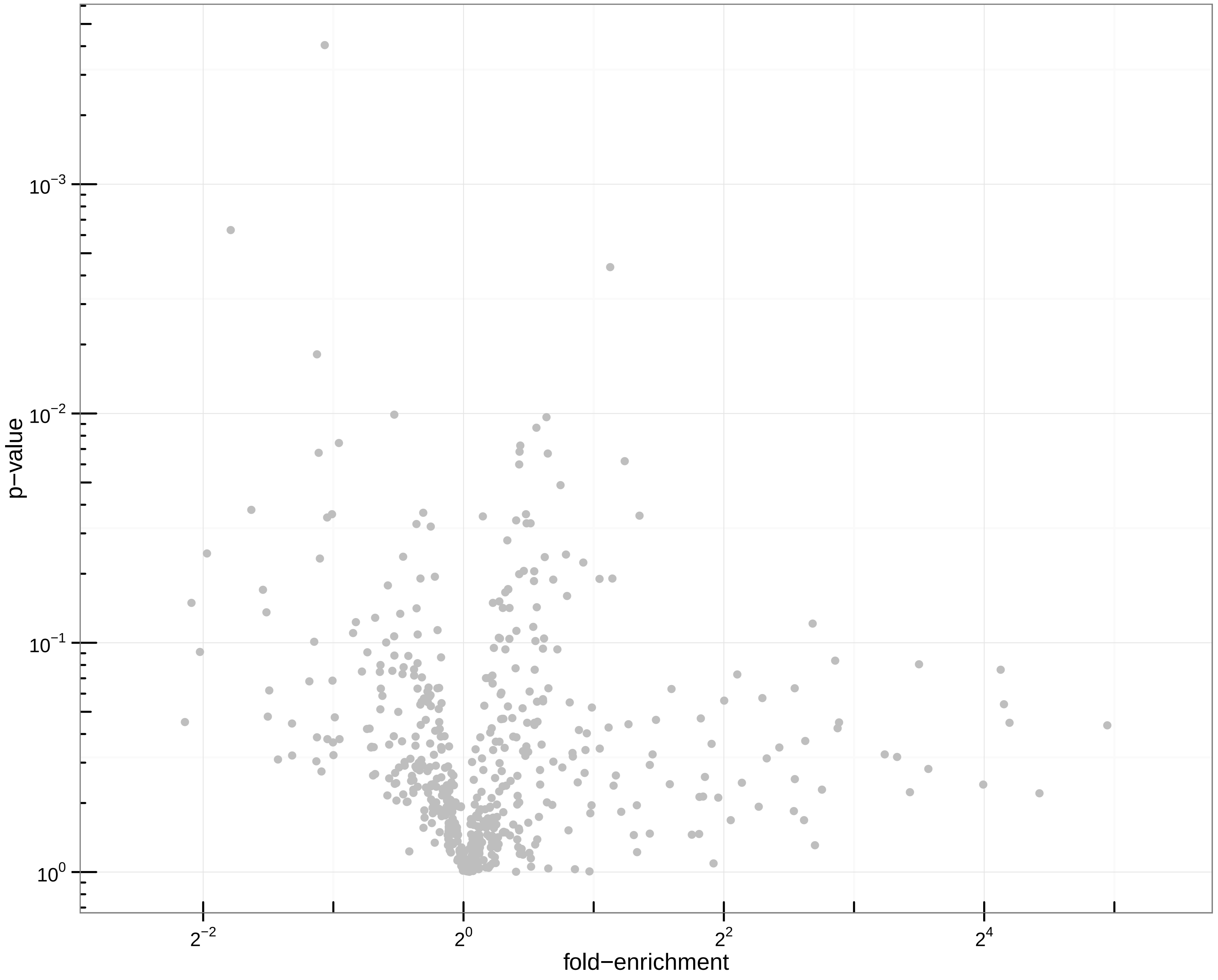

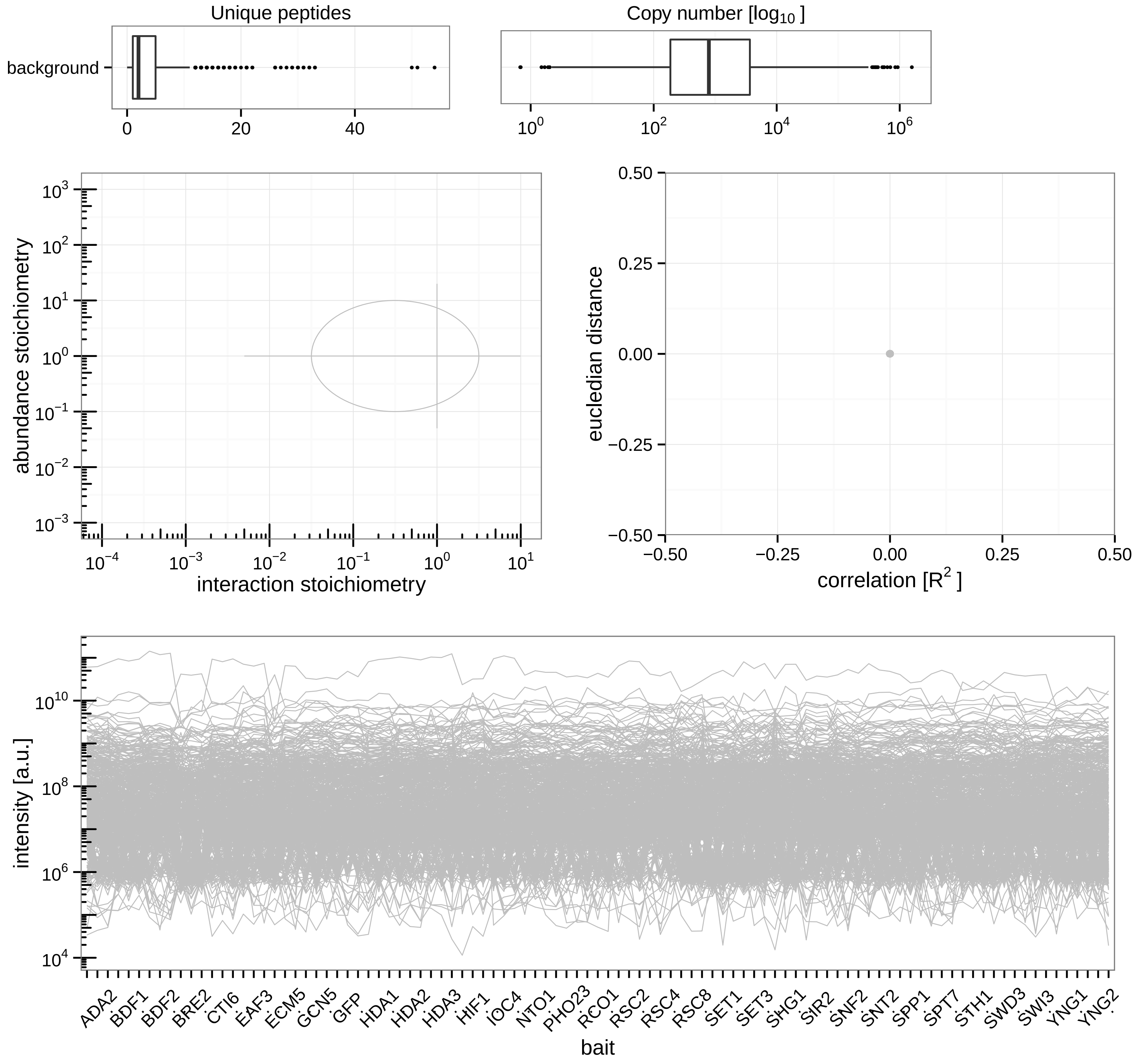

significance

interactor class

**bait**	HDA1		**interactors**	3
**experiment**	27 minutes		**recovery**	100 %

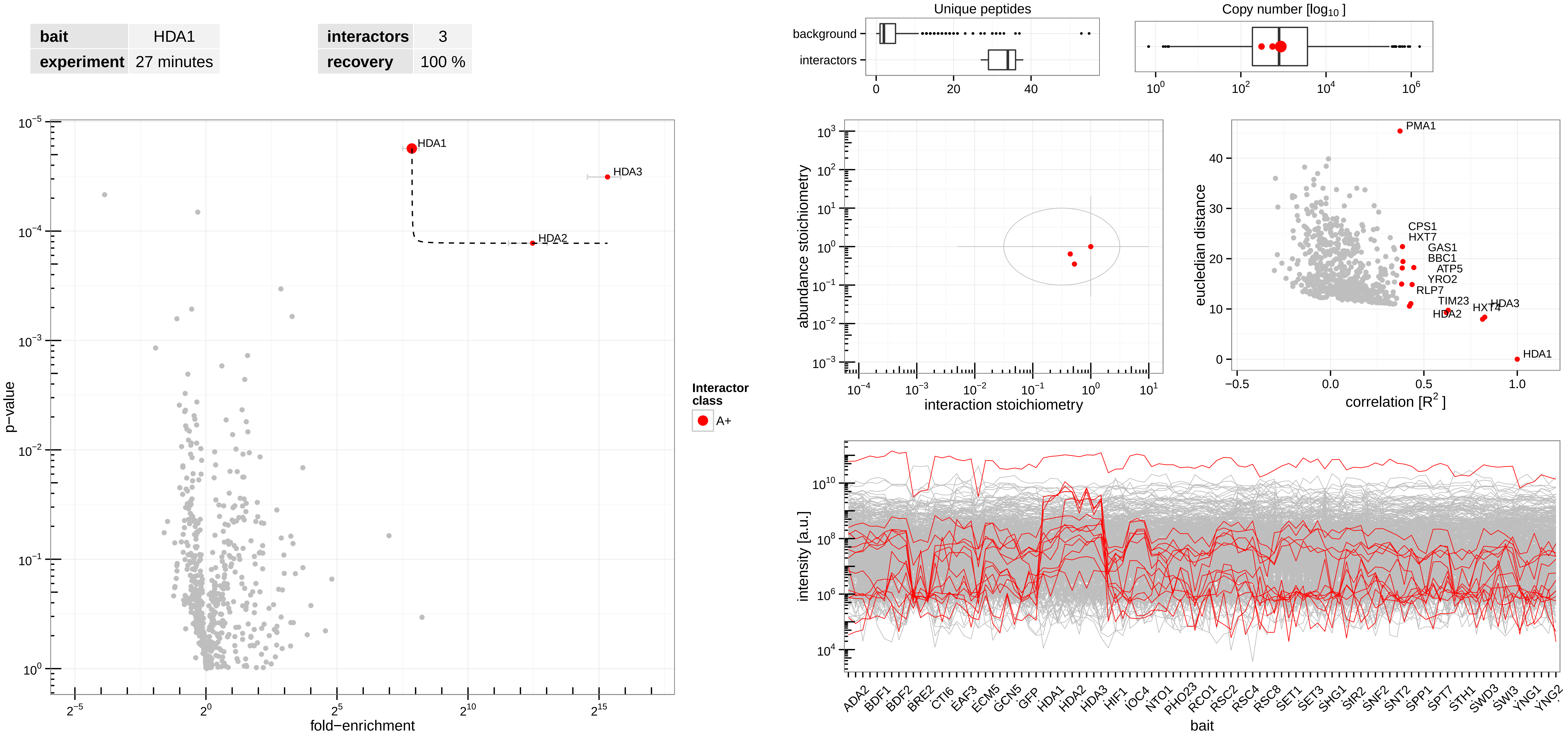

	HDA1	HDA2	HDA3
significance	8.3	12.0	15.0
interactor class	A+	A+	A+

**bait**	HDA2
**experiment**	27 minutes

**interactors**	6
**recovery**	100 %

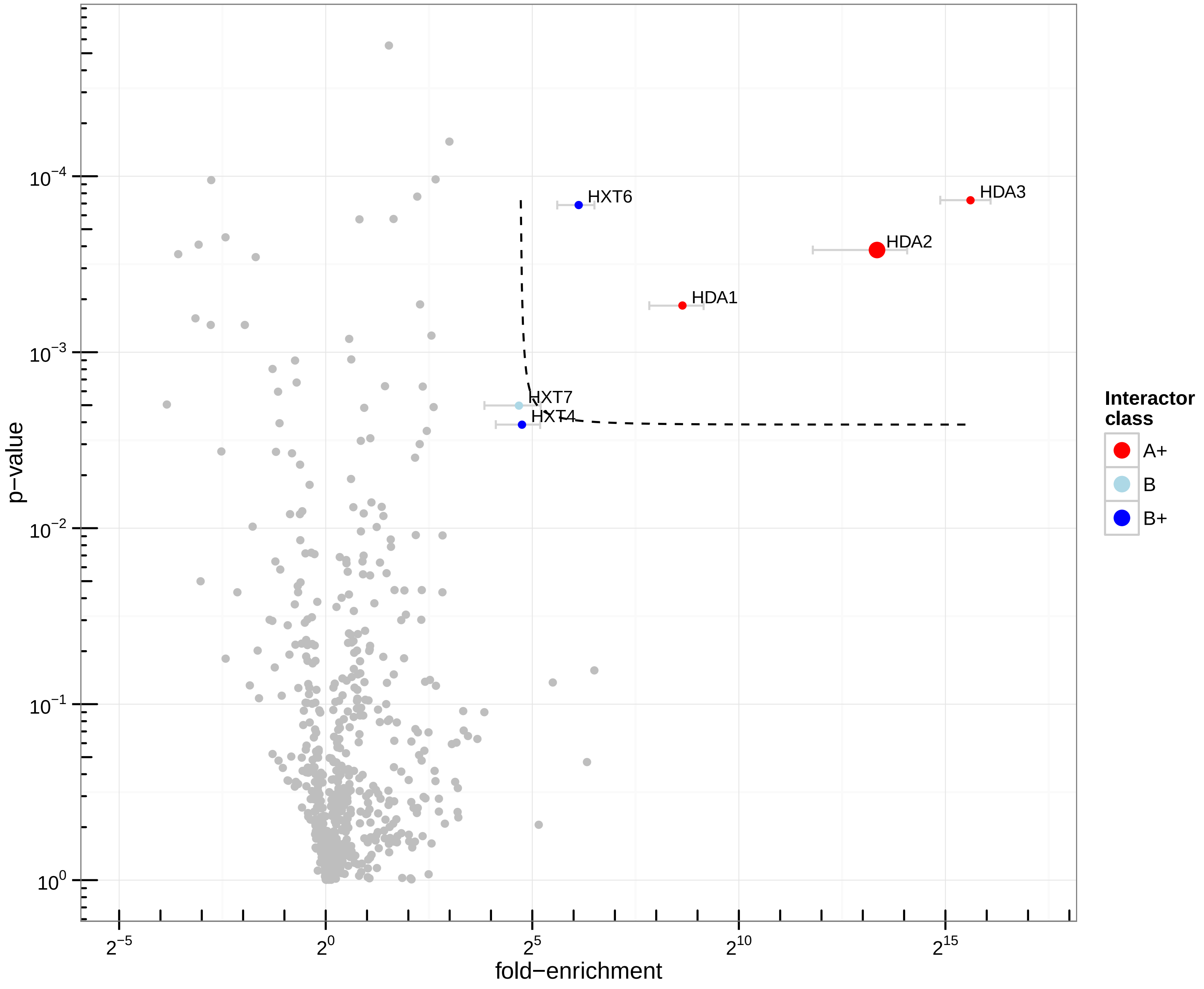

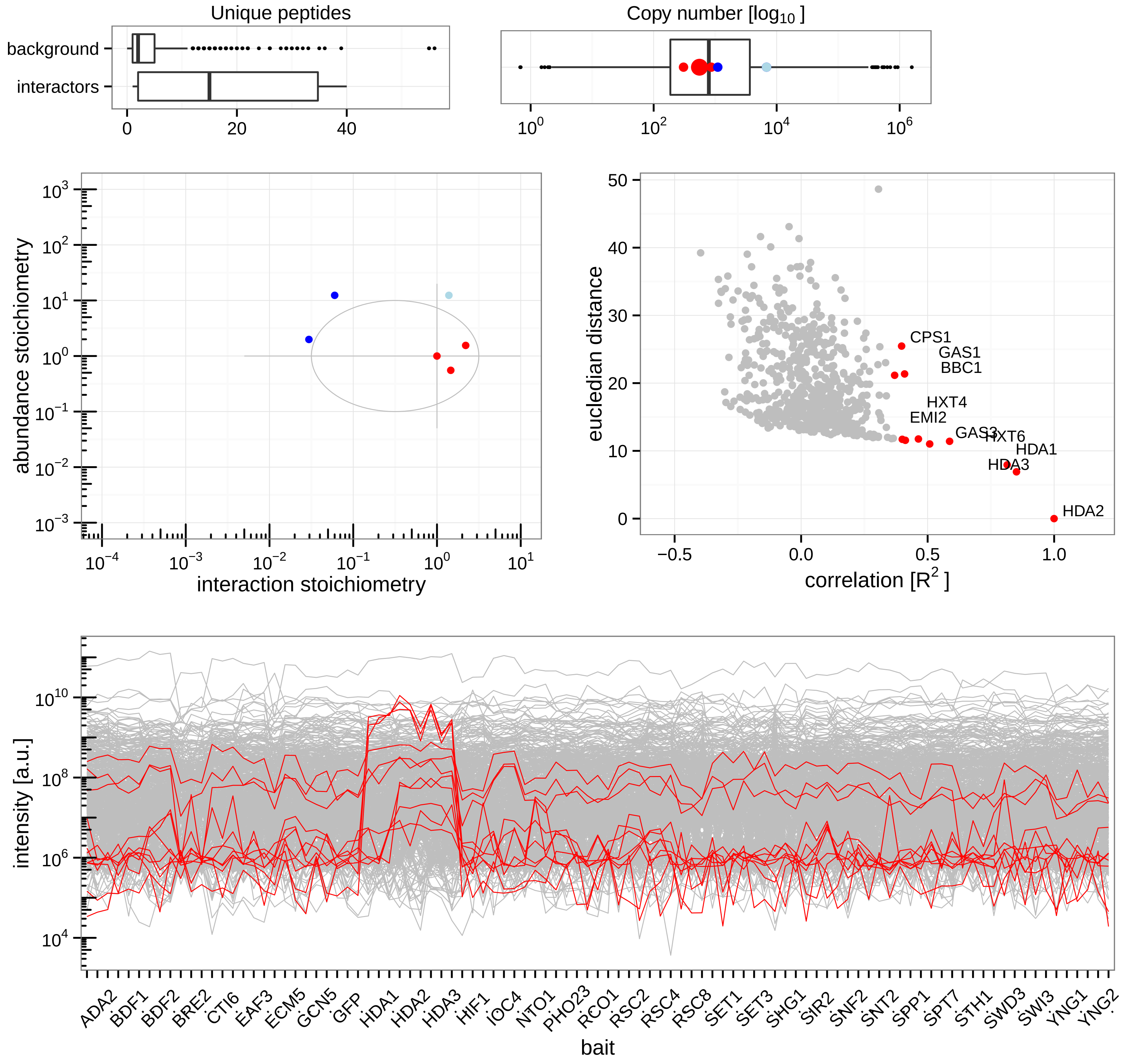

	HDA1	HDA2	HDA3	HXT4	HXT6
significance	8.3	13.0	15.0	4.6	6.4
interactor class	A+	A+	A+	B+	B+

**bait**	HDA3		**interactors**	6
**experiment**	27 minutes		**recovery**	100 %

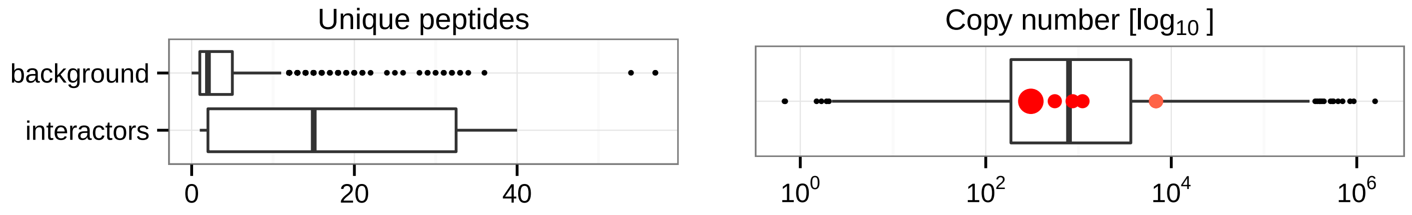

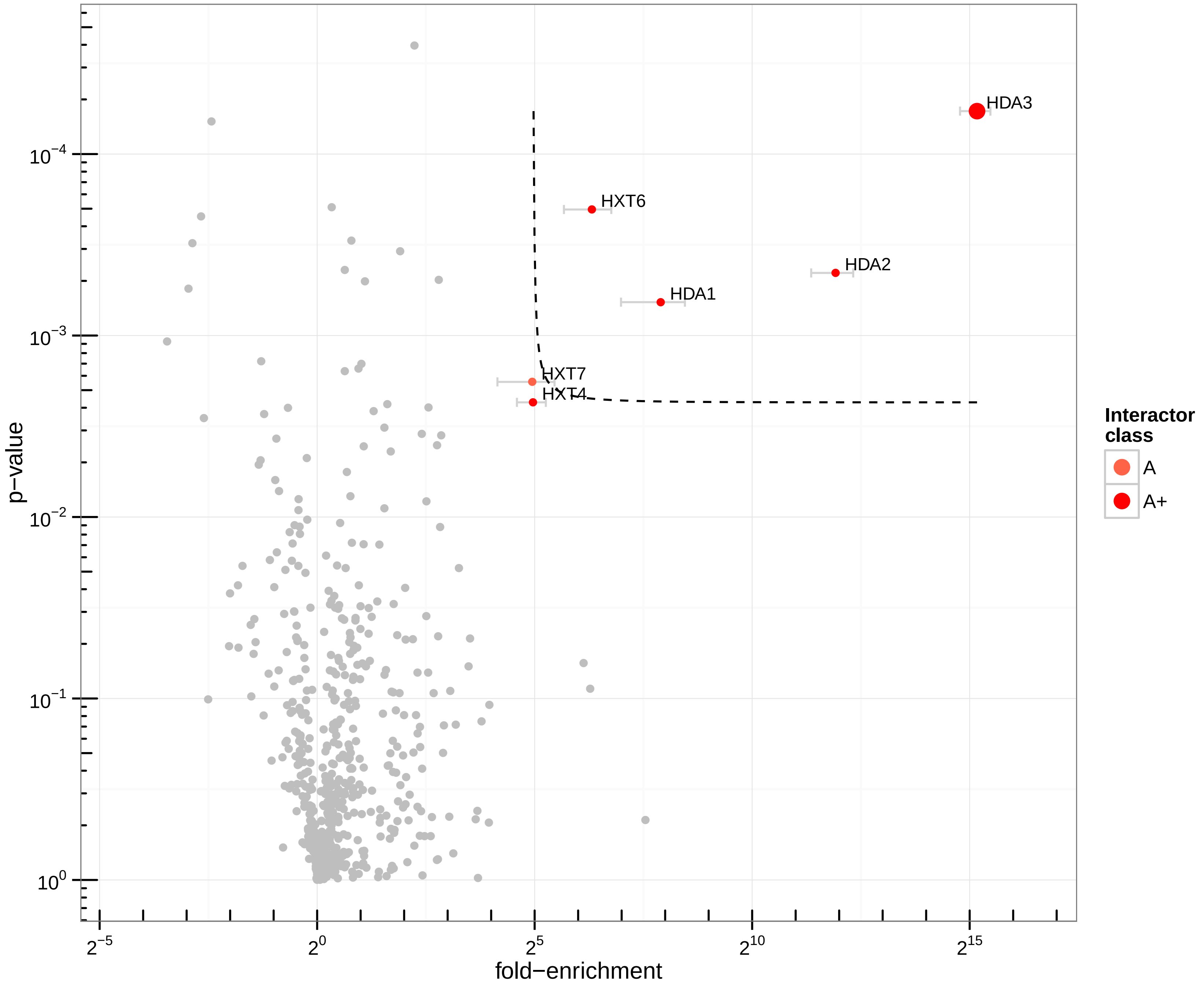

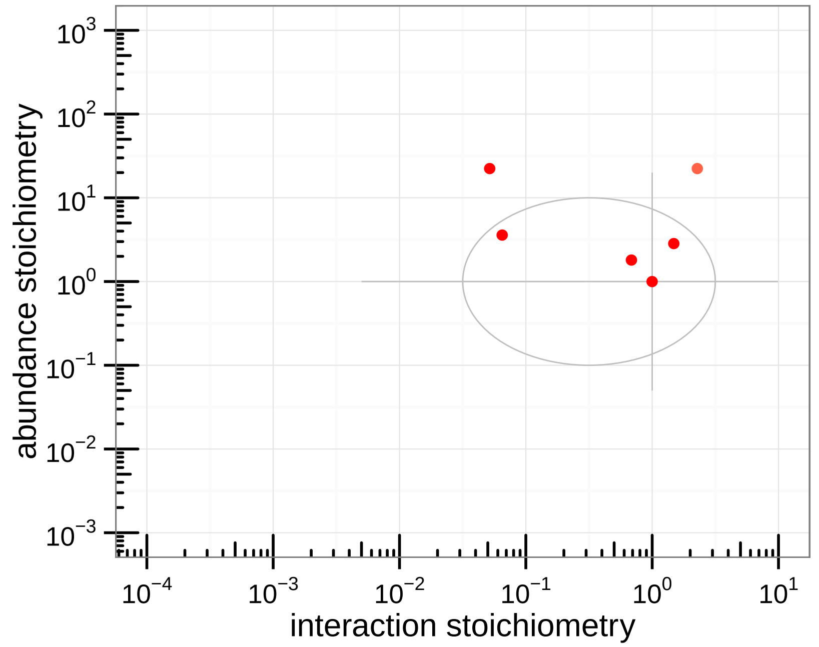

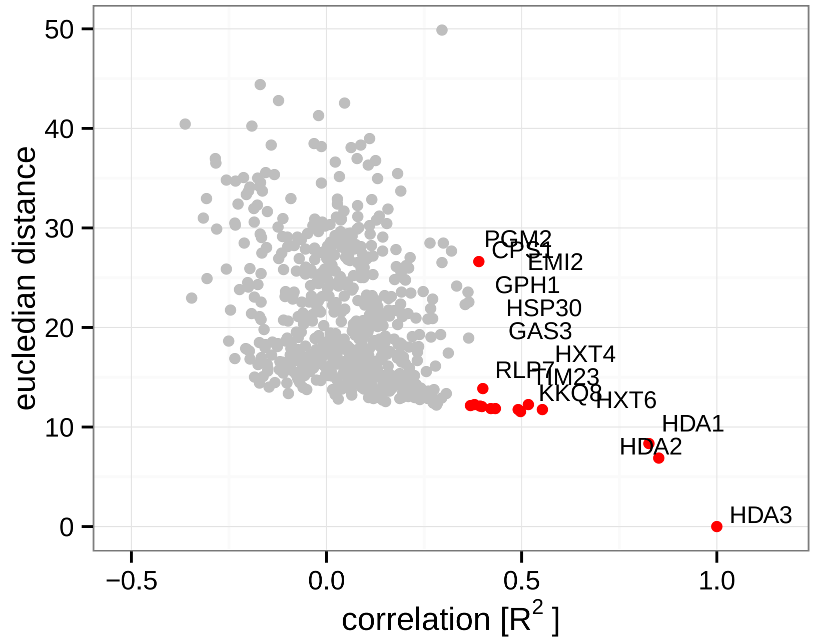

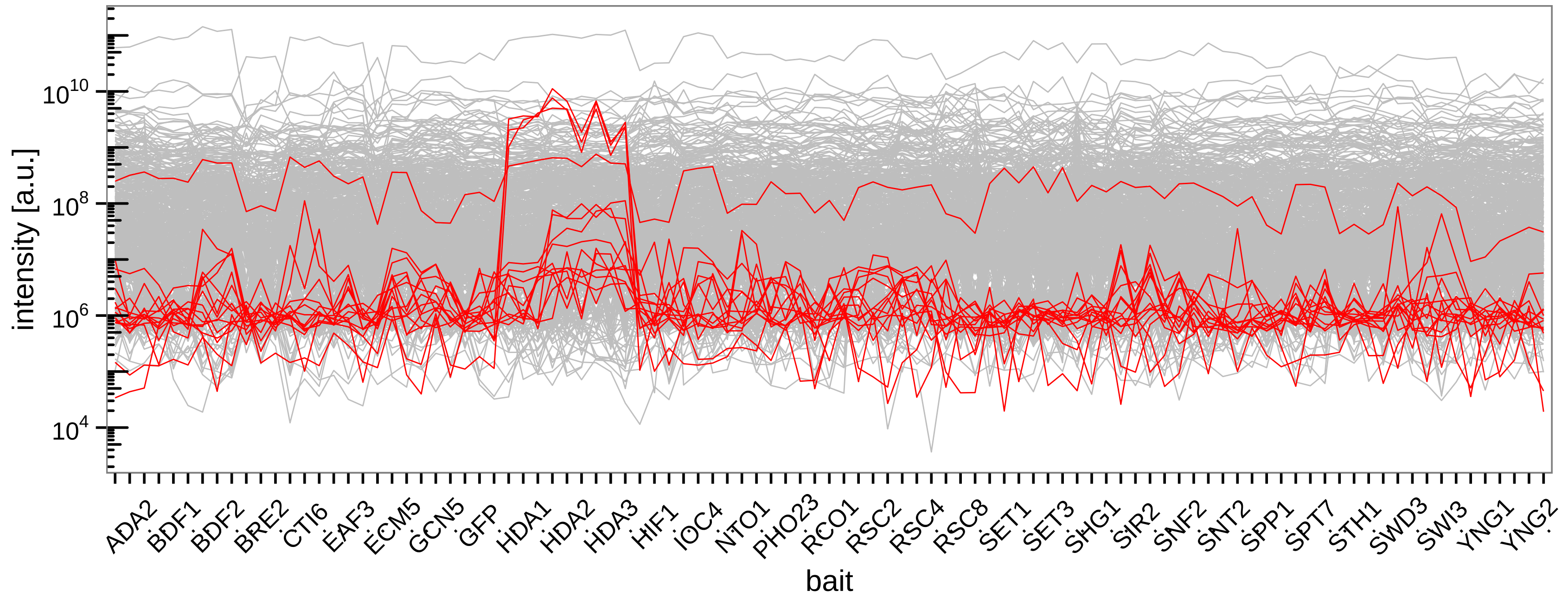

	HDA1	HDA2	HDA3	HXT4	HXT6	HXT7
significance	7.6	11.0	15.0	4.8	6.5	4.8
interactor class	A+	A+	A+	A+	A+	A

**bait**	HIF1
**experiment**	27 minutes

**interactors**	5
**recovery**	33 %

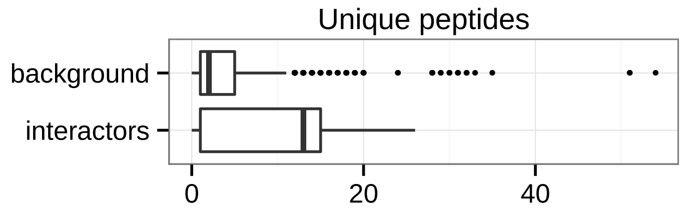

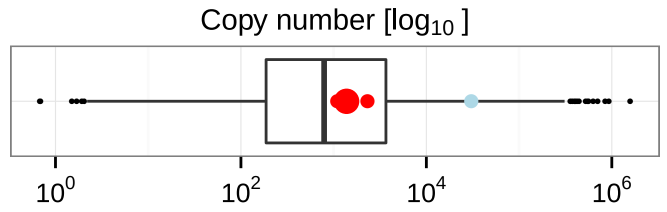

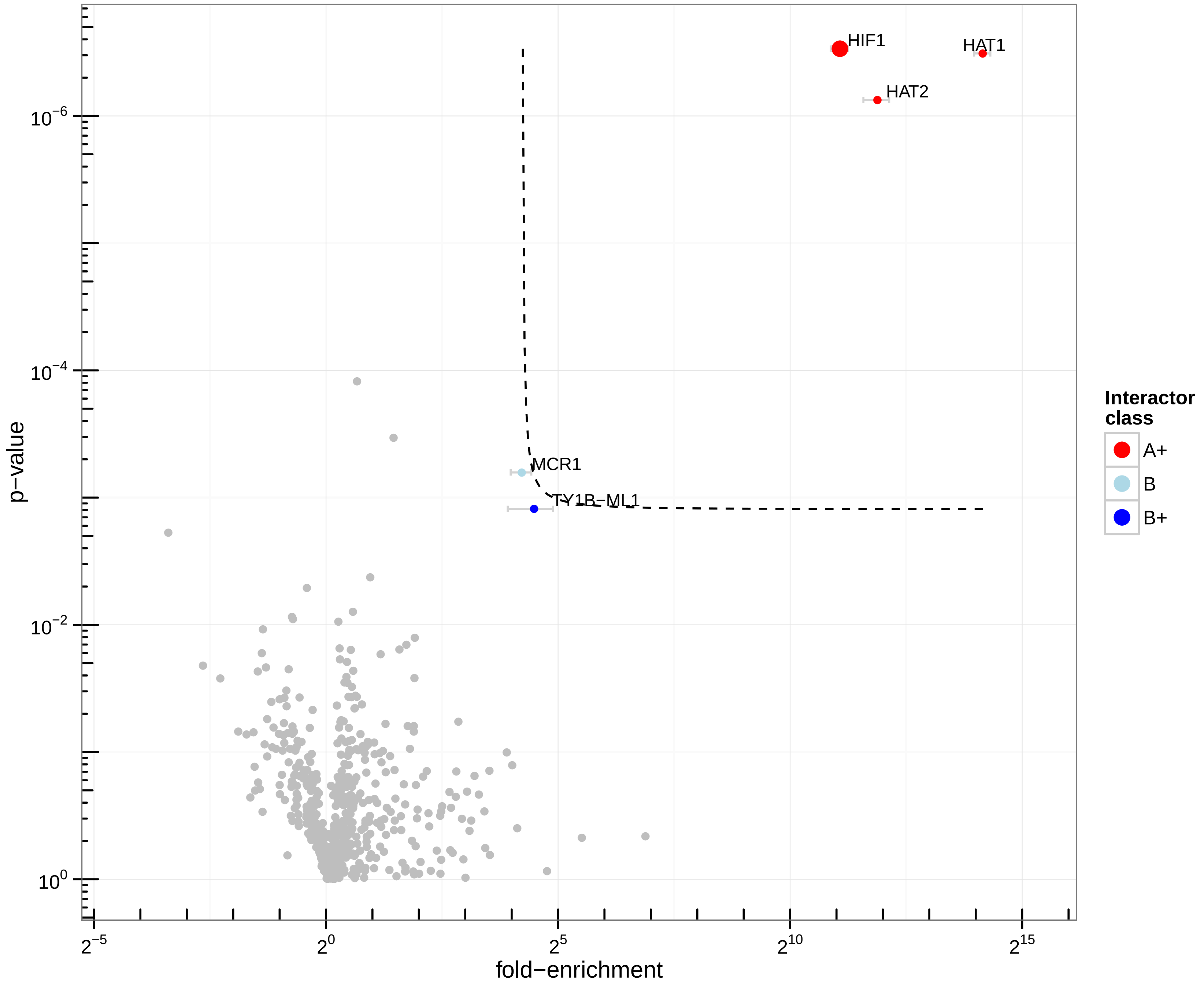

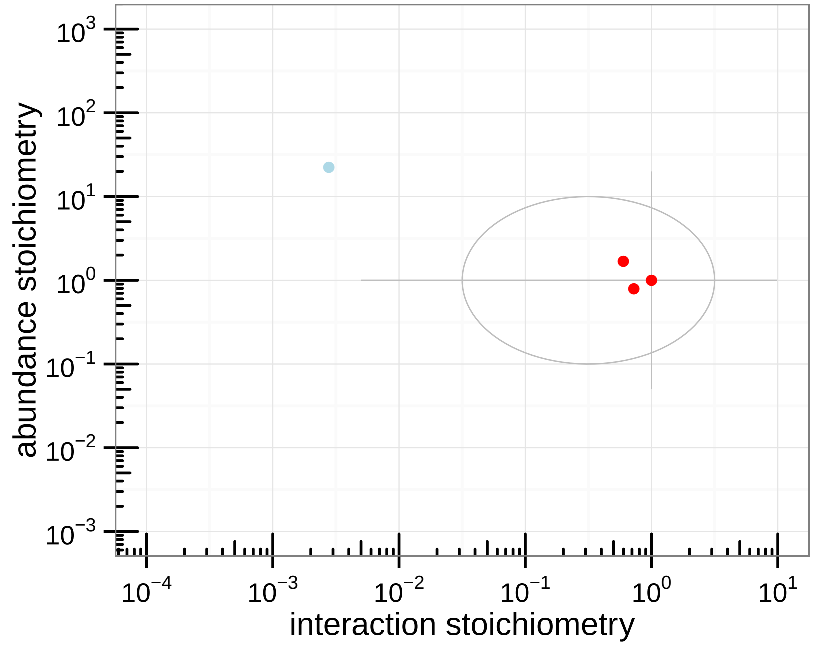

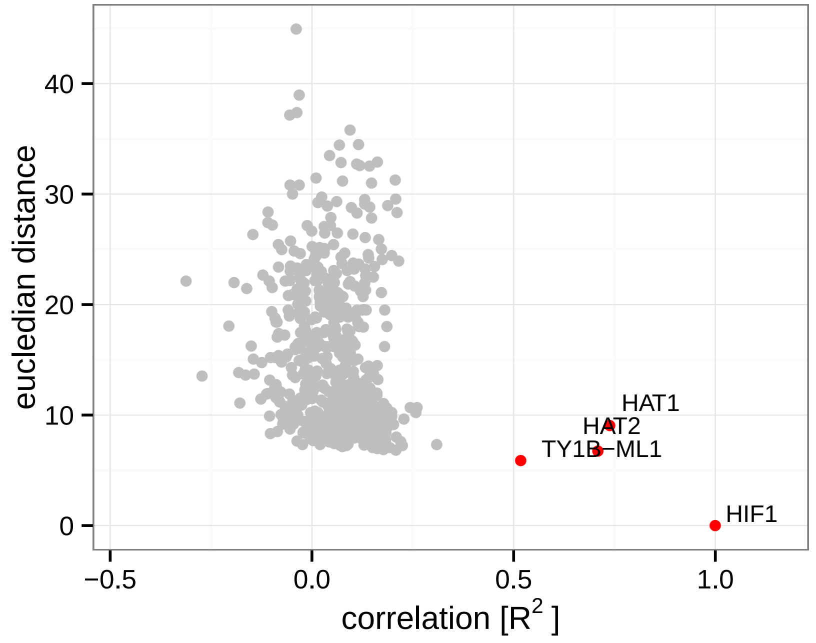

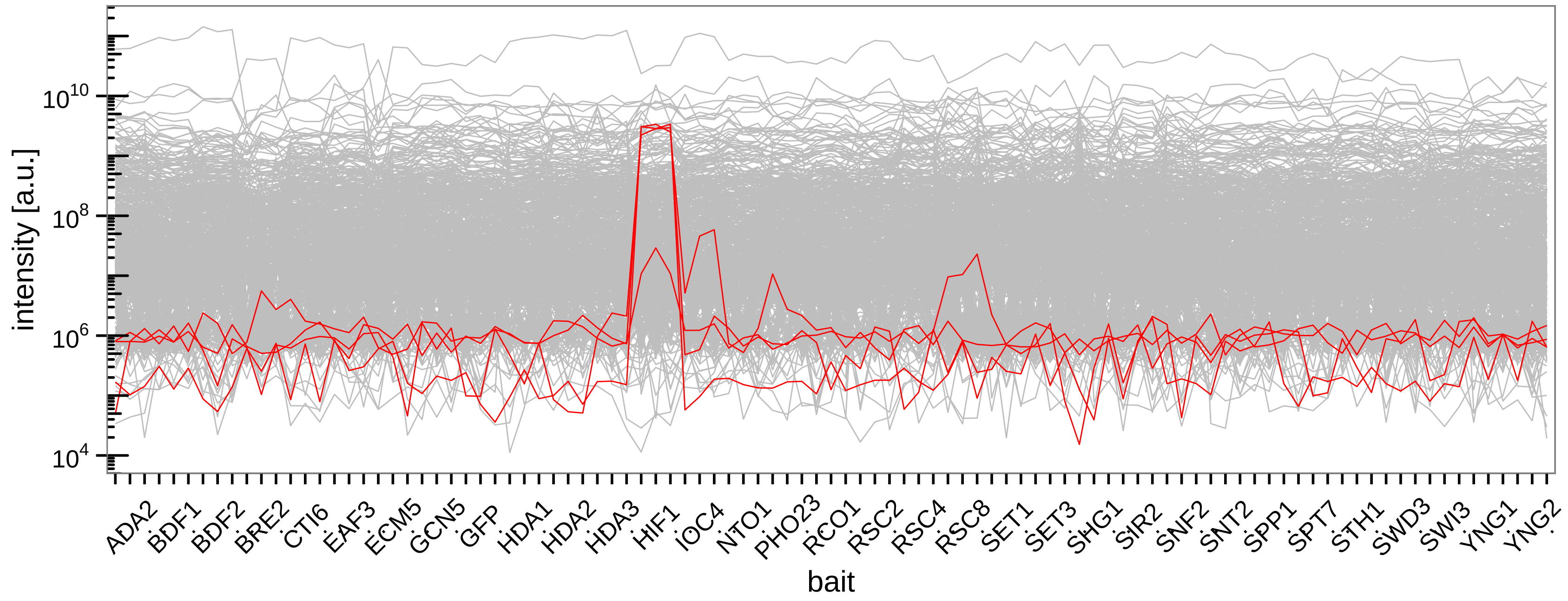

	HAT1	HAT2	HIF1	TY1B−ML1
significance	15.0	12.0	12.0	4.5
interactor class	A+	A+	A+	B+

**bait**	IOC4
**experiment**	27 minutes

**interactors**	0
**recovery**	0 %

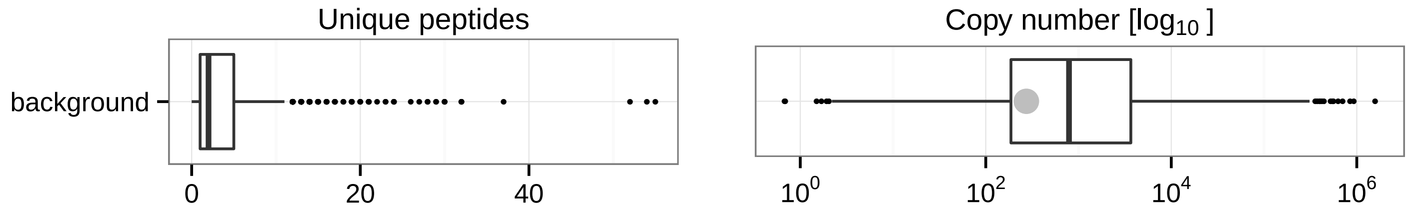

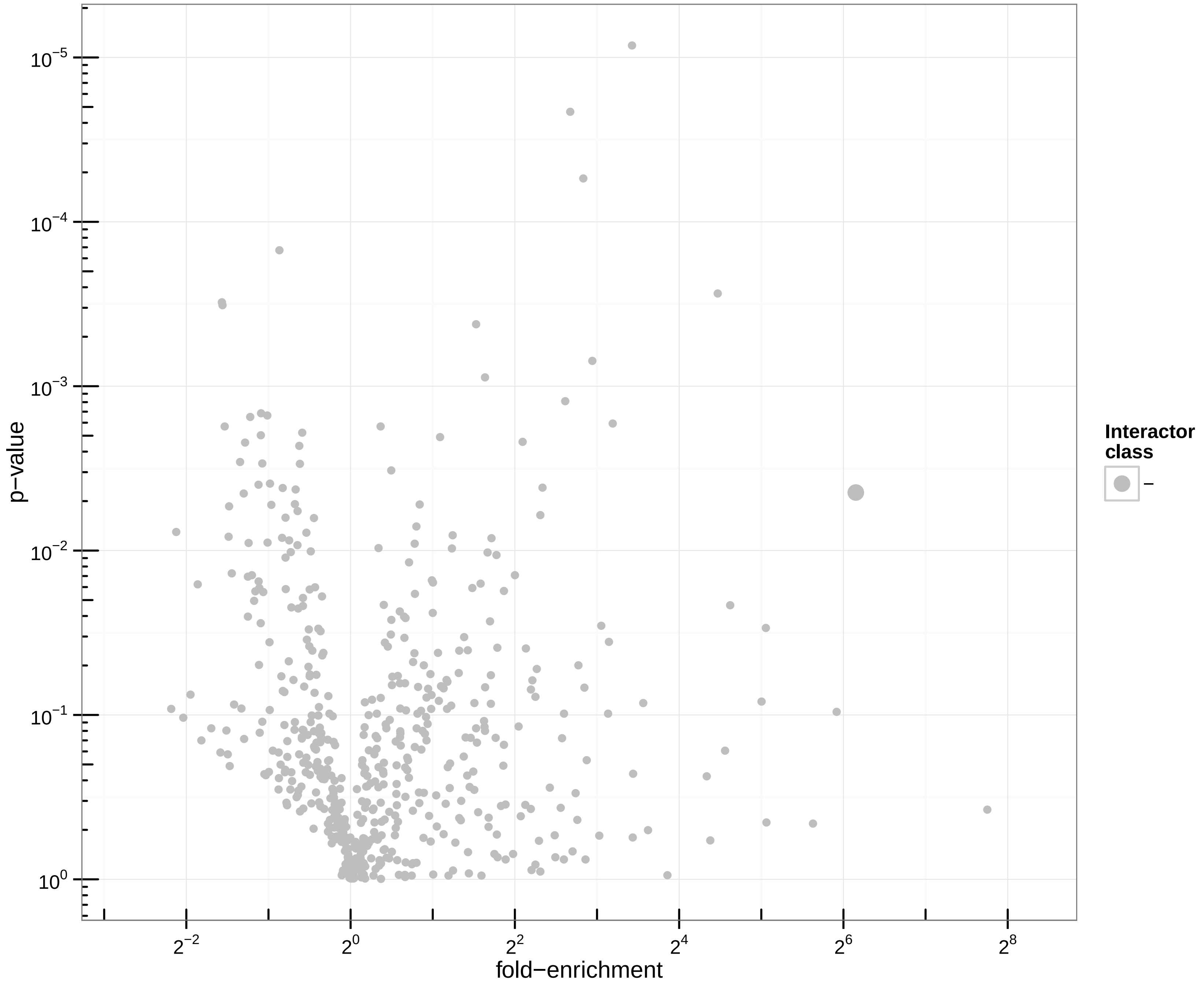

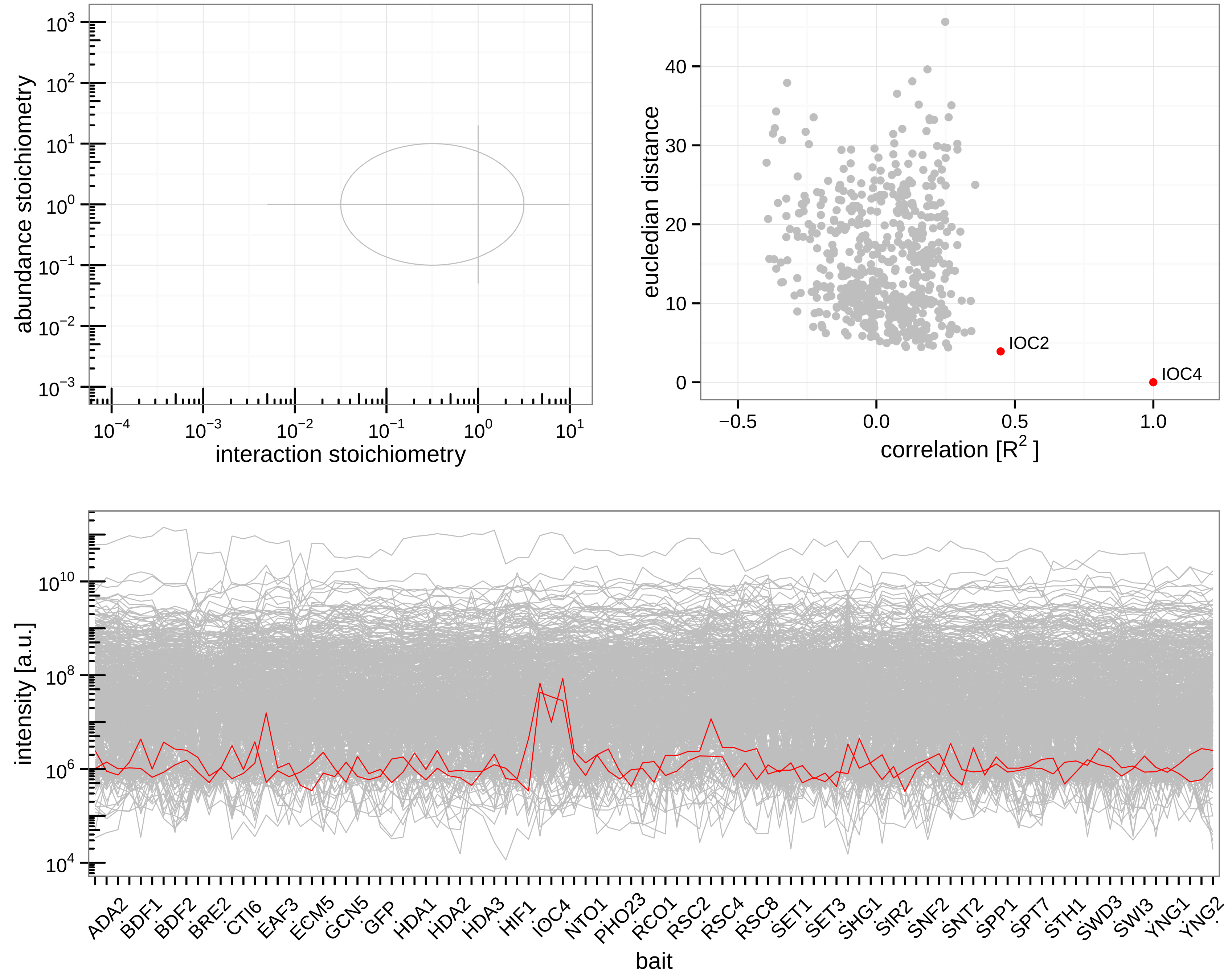

significance

interactor class

**bait**	NTO1
**experiment**	27 minutes

**interactors**	0
**recovery**	NaN %

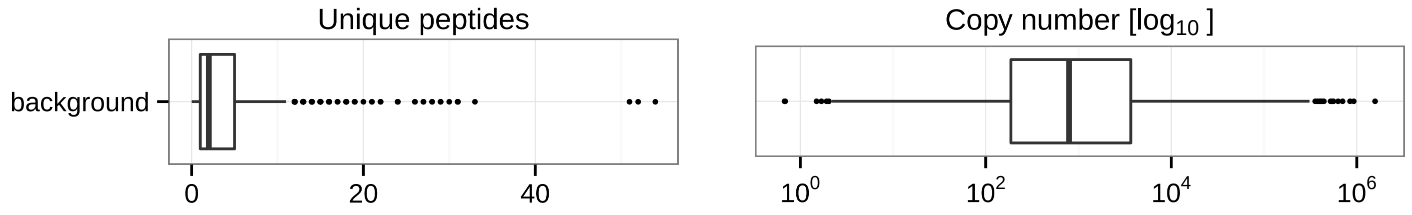

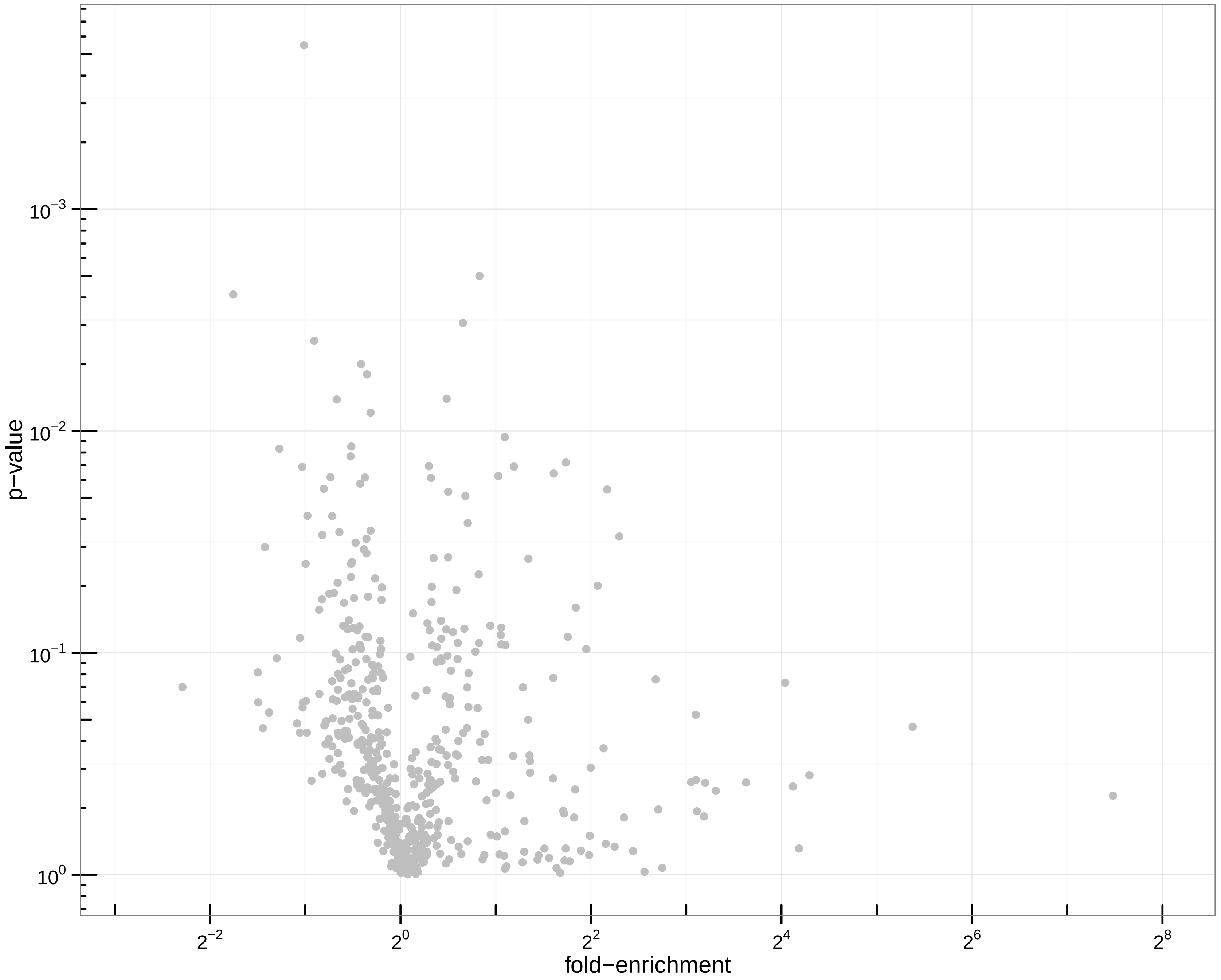

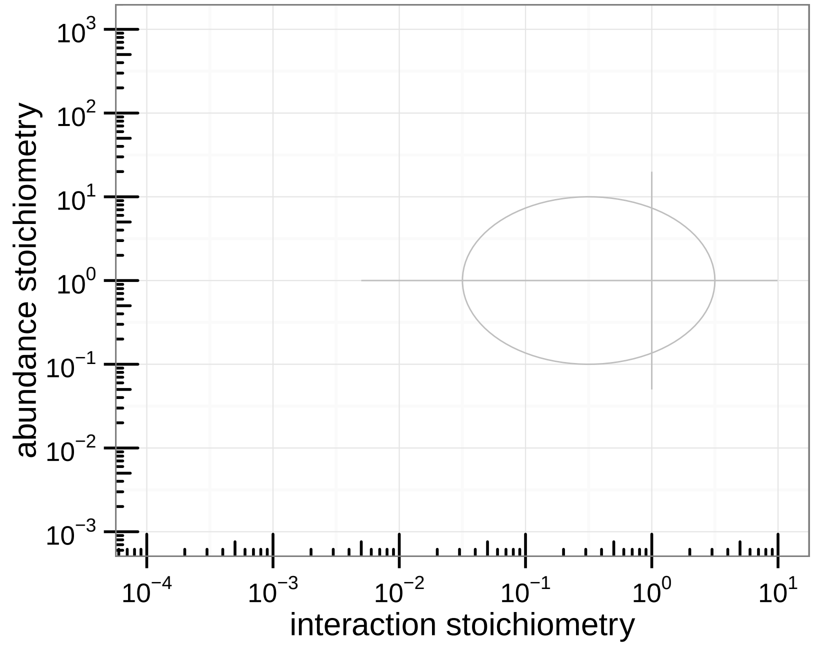

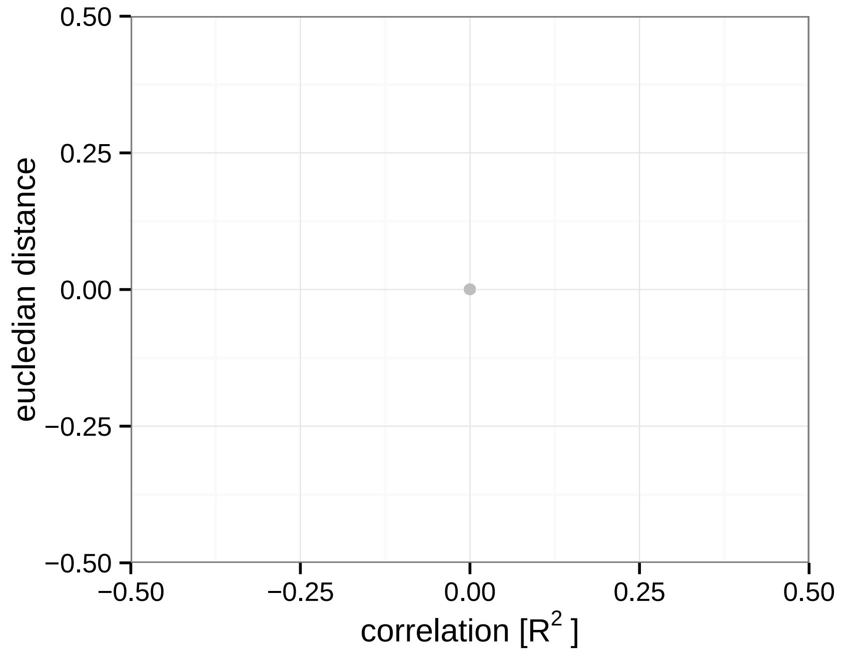

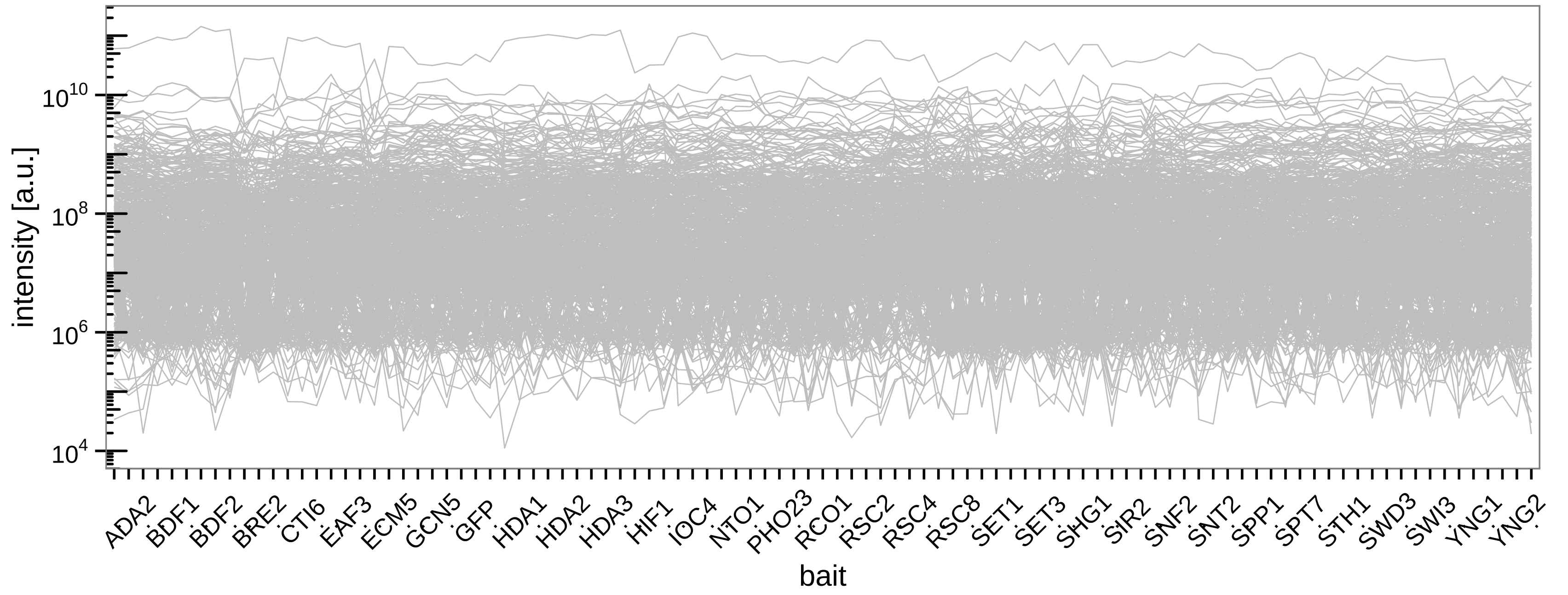

significance
interactor class

**bait**	PHO23
**experiment**	27 minutes

**interactors**	10
**recovery**	38 %

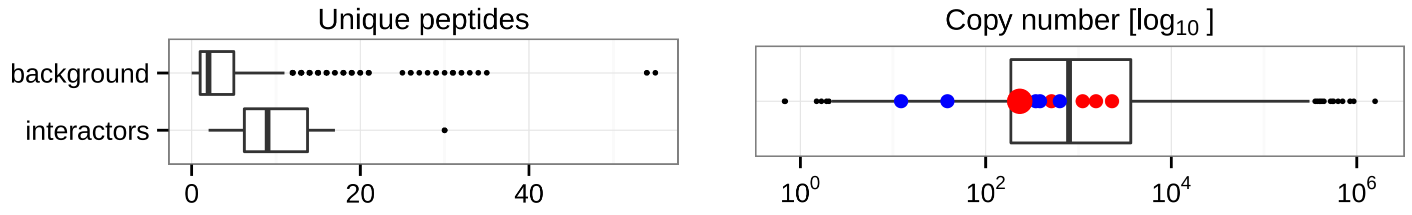

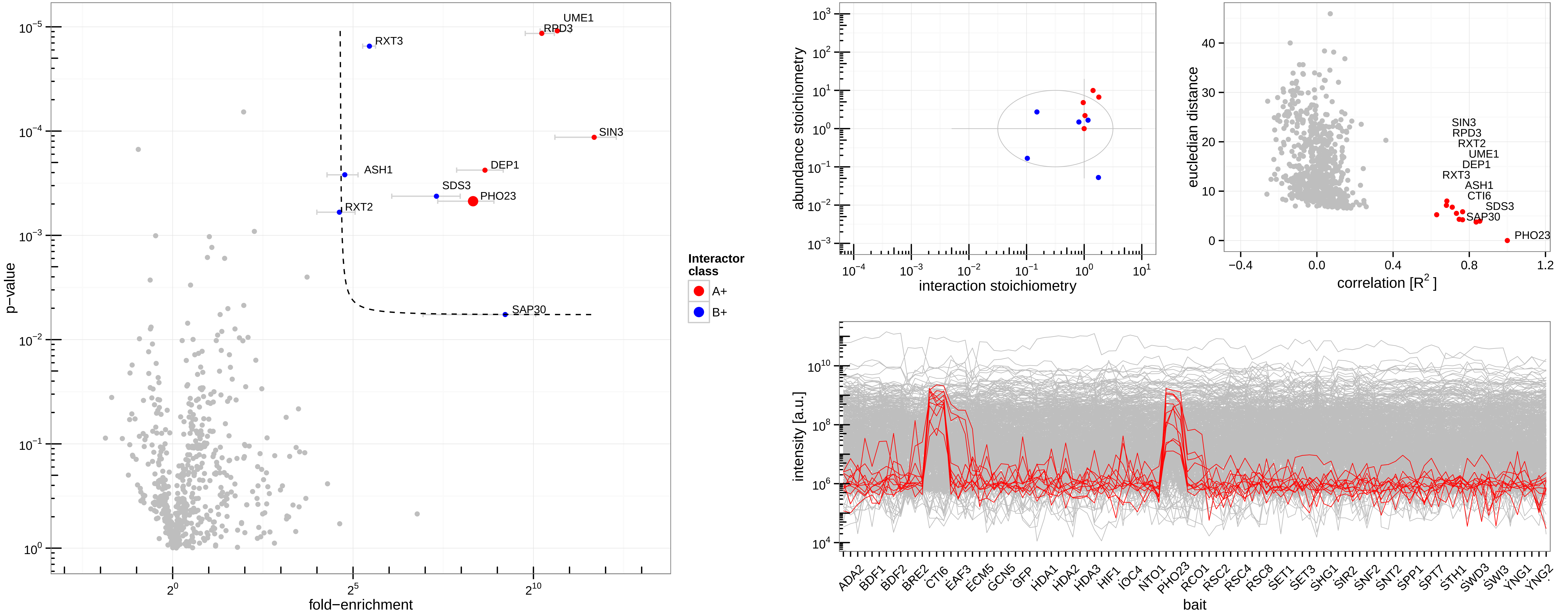

	ASH1	DEP1	PHO23	RPD3	RXT2	RXT3	SAP30	SDS3	SIN3	UME1
significance	5.2	8.5	8.0	10.0	4.8	6.6	8.5	7.2	11.0	11.0
interactor class	B+	A+	A+	A+	B+	B+	B+	B+	A+	A+

**bait**	RCO1
**experiment**	27 minutes

**interactors**	3
**recovery**	11 %

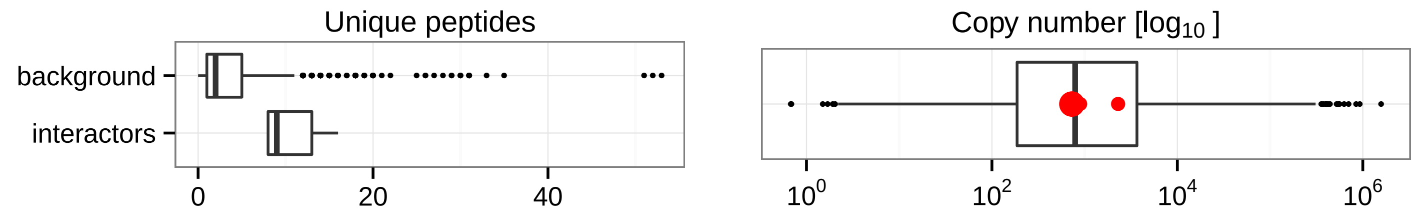

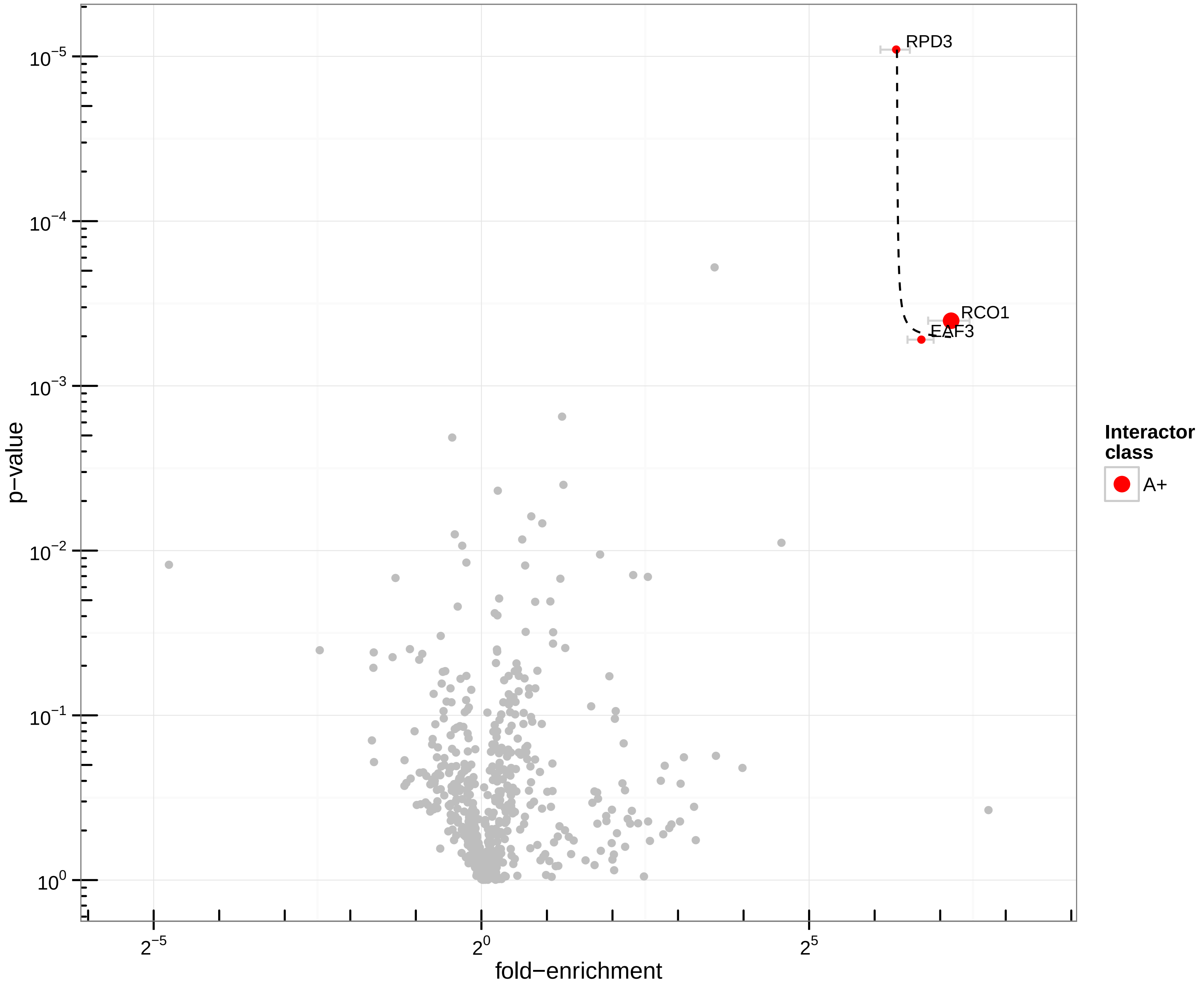

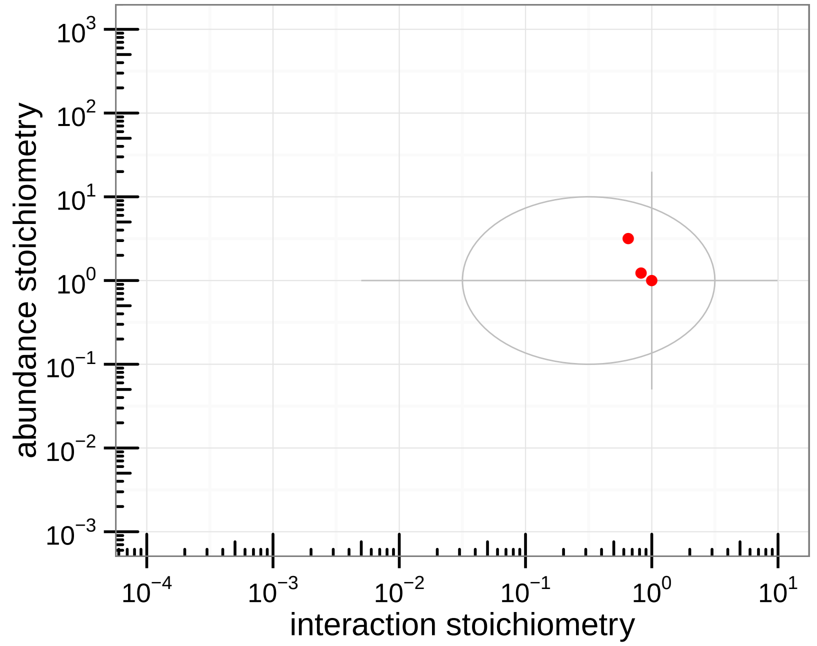

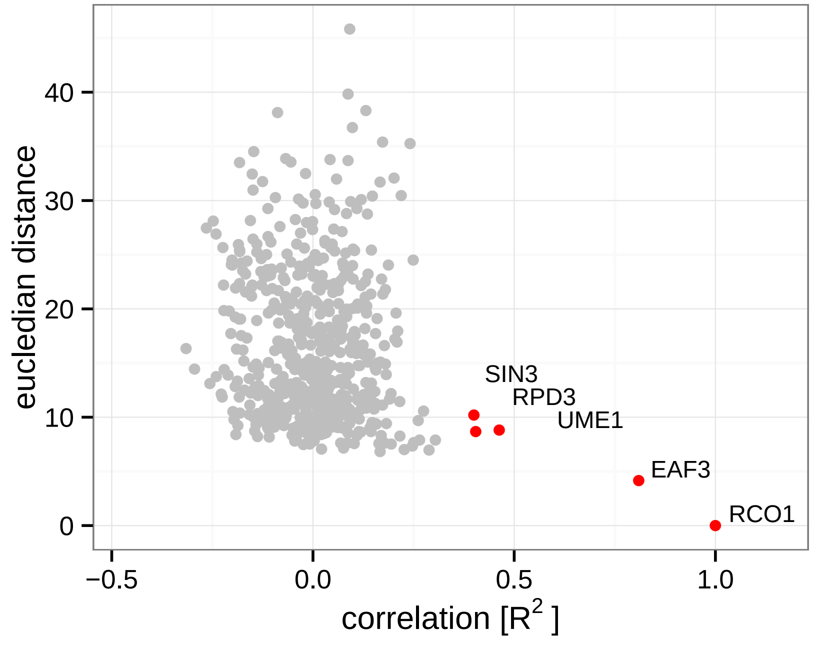

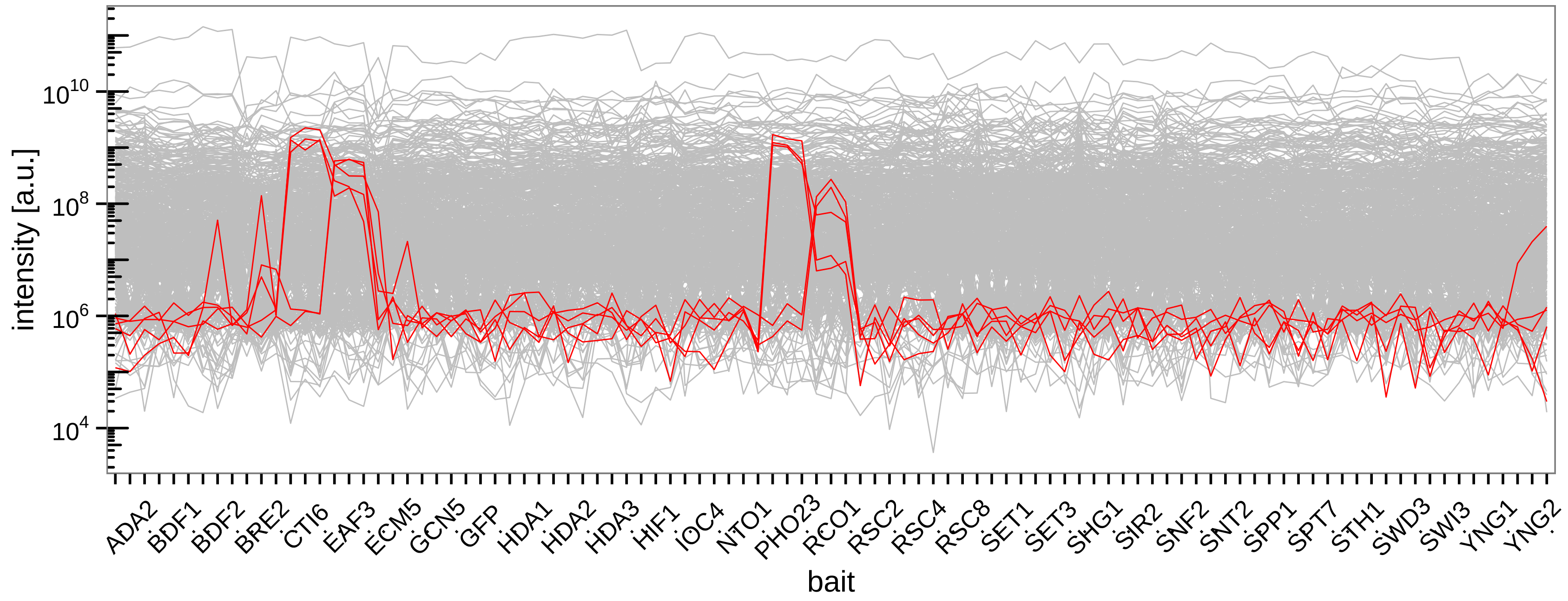

	EAF3	RCO1	RPD3
significance	6.6	7.0	7.3
interactor class	A+	A+	A+

**bait**	RSC2		**interactors**	25
**experiment**	27 minutes		**recovery**	88 %

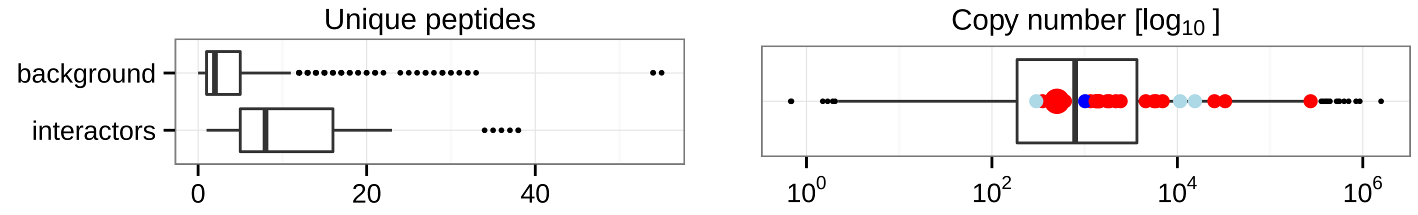

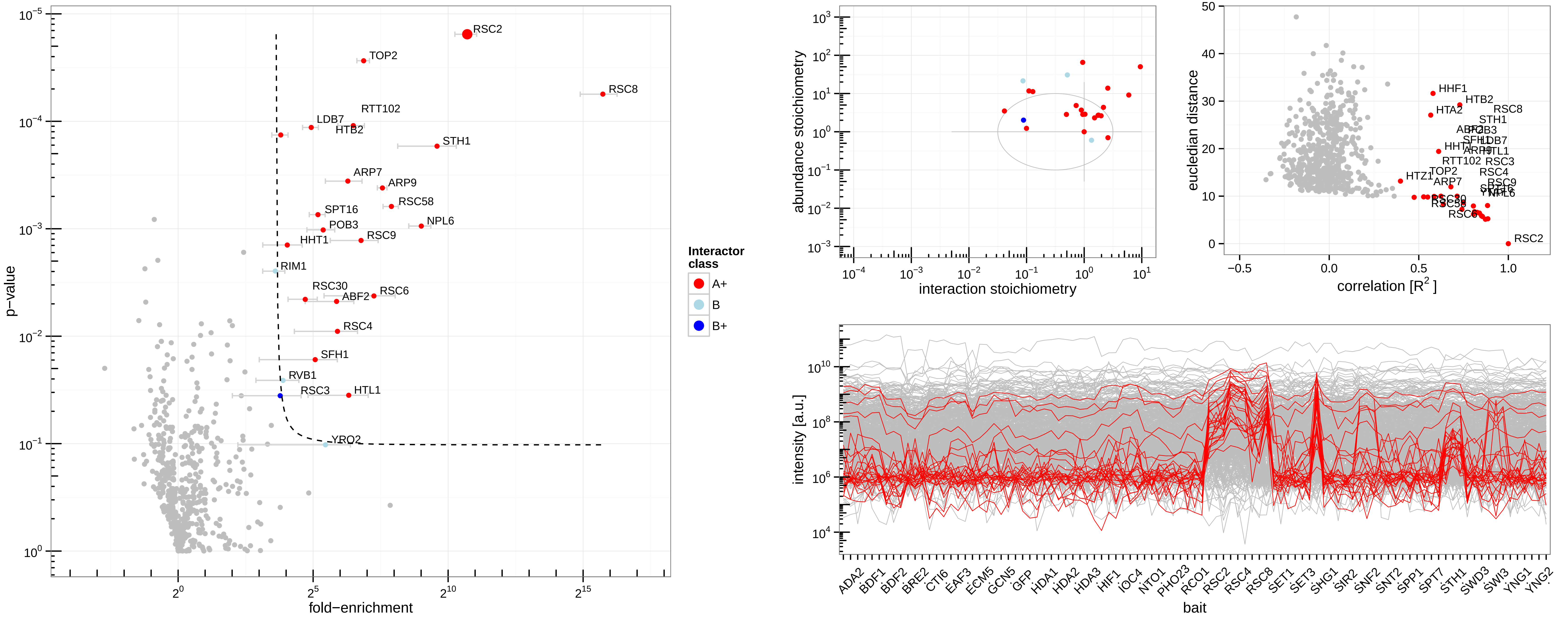

	ABF2	ARP7	ARP9	HHT1	HTB2	HTL1	LDB7	NPL6	POB3	RSC2	RSC3	RSC30	RSC4	RSC58	RSC6	RSC8	RSC9	RTT102	SFH1	SPT16	STH1	TOP2
significance	5.4	6.3	7.4	4.2	4.8	5.5	5.6	8.6	5.3	11.0	3.1	4.4	5.3	7.6	6.7	15.0	6.5	6.8	4.5	5.2	9.4	7.4
interactor class	A+	A+	A+	A+	A+	A+	A+	A+	A+	A+	B+	A+	A+	A+	A+	A+	A+	A+	A+	A+	A+	A+

**bait**	RSC4
**experiment**	27 minutes

**interactors**	26
**recovery**	94 %

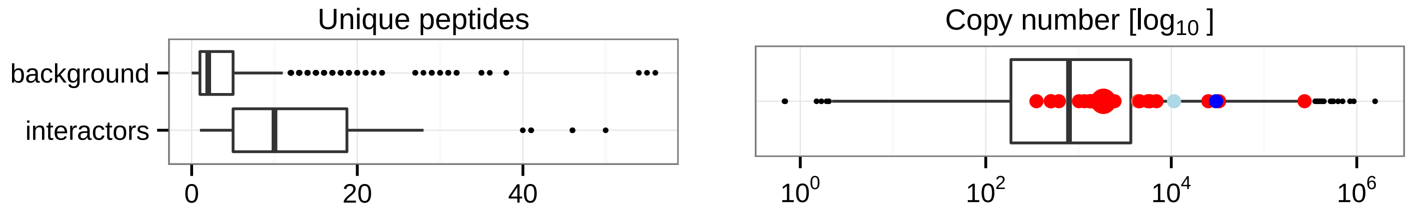

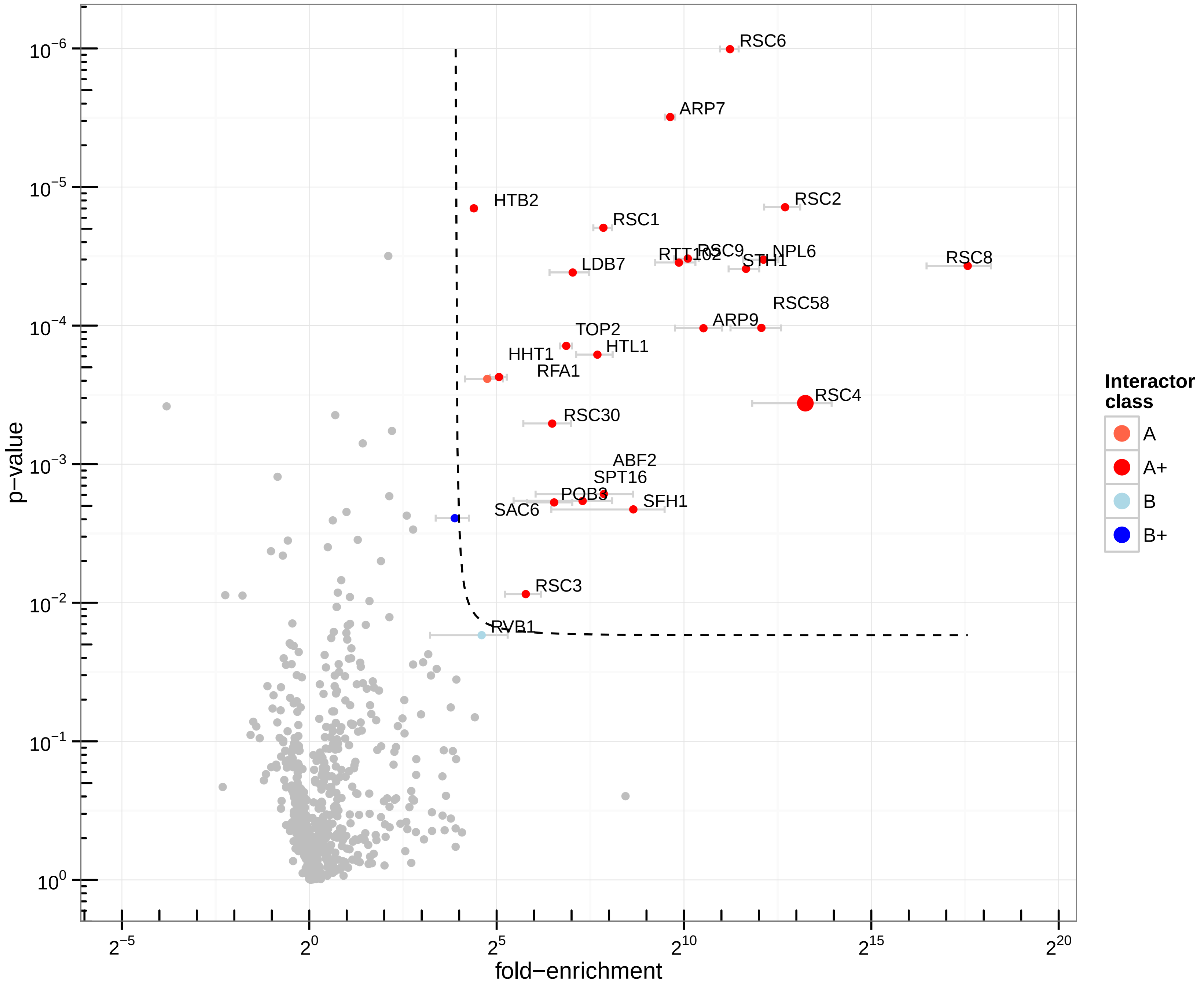

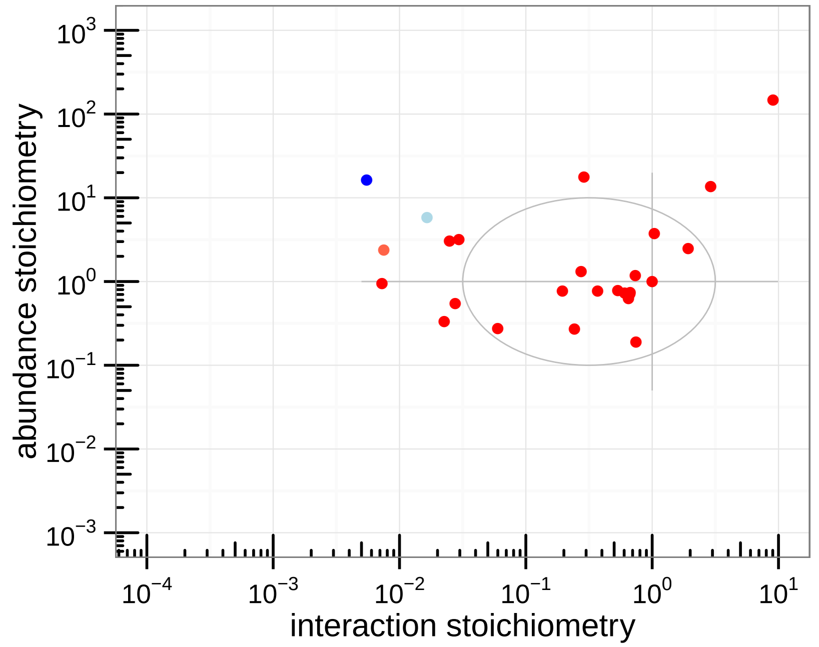

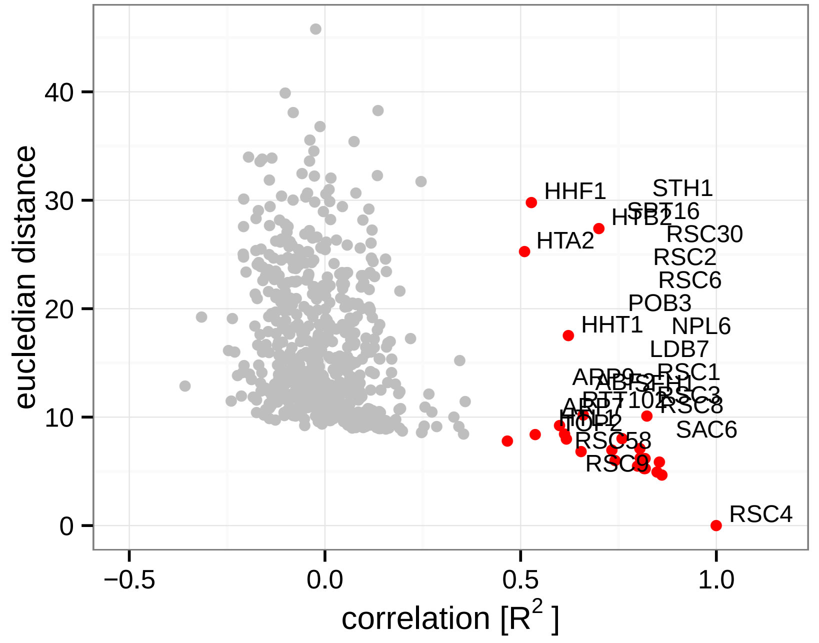

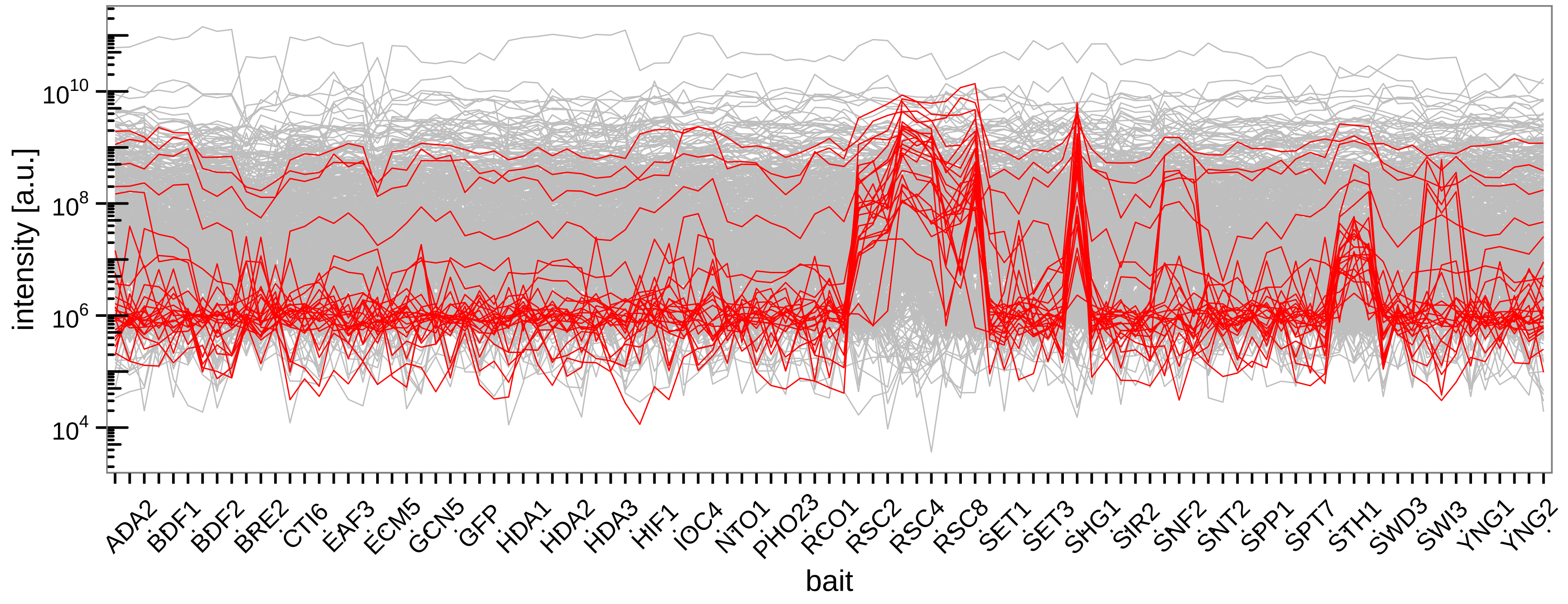

	ABF2	ARP7	ARP9	HHT1	HTB2	HTL1	LDB7	NPL6	POB3	RFA1	RSC1	RSC2	RSC3	RSC30	RSC4	RSC58	RSC6	RSC8	RSC9	RTT102	SAC6	SFH1	SPT16	STH1	TOP2
significance	7.4	10.0	10.0	5.4	5.9	7.7	7.5	12.0	6.2	5.2	8.3	13.0	5.2	6.4	13.0	12.0	12.0	17.0	10.0	9.9	3.9	8.1	6.9	12.0	7.0
interactor class	A+	A+	A+	A+	A+	A+	A+	A+	A+	A	A+	A+	A+	A+	A+	A+	A+	A+	A+	A+	B+	A+	A+	A+	A+

bait	RSC8
experiment	27 minutes

interactors	23
recovery	82 %

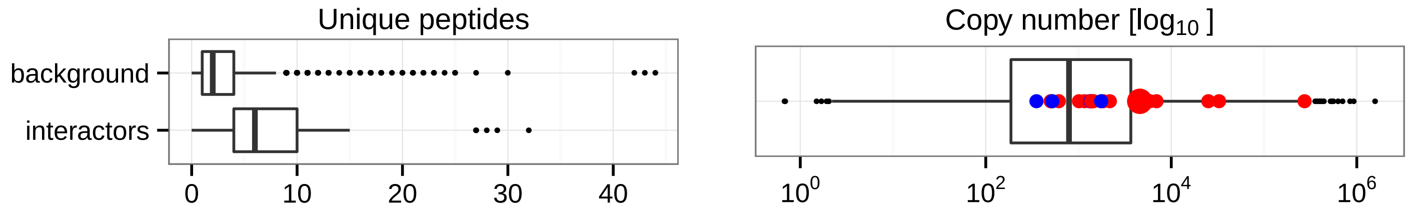

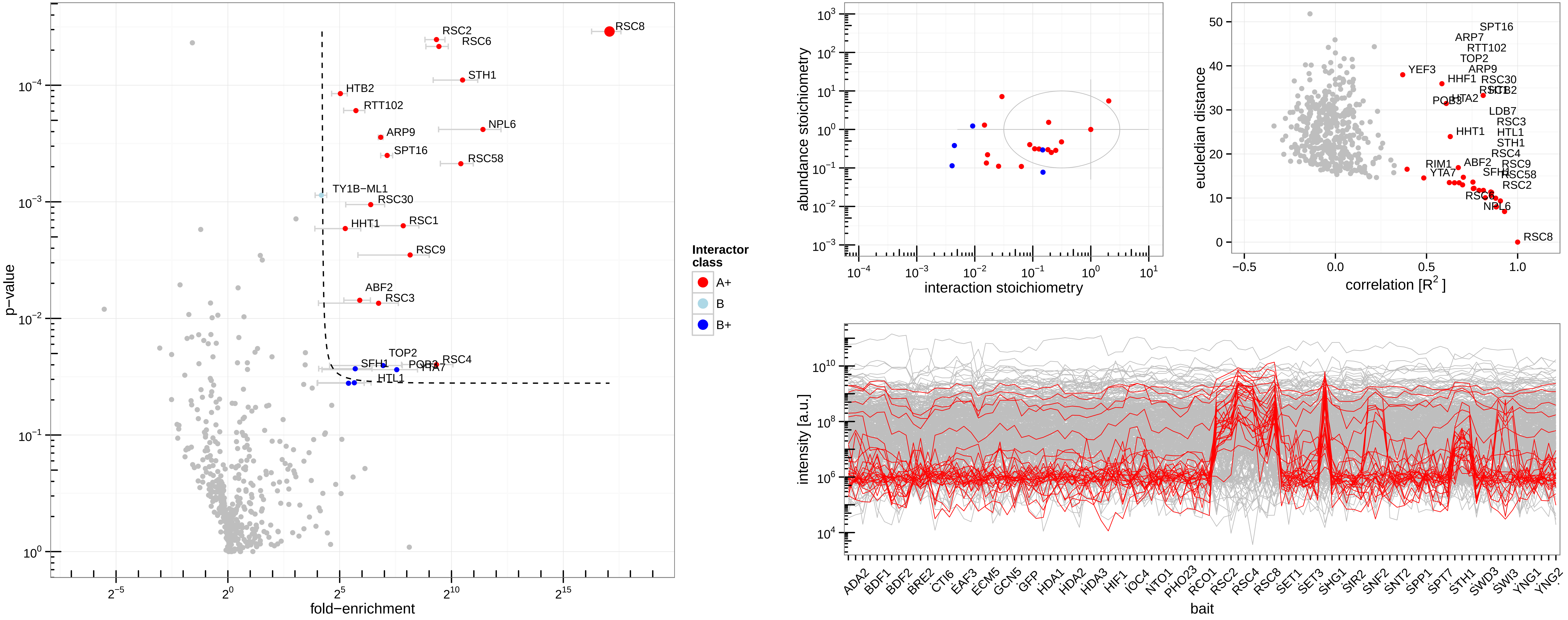

	ABF2	ARP9	HHT1	HTB2	HTL1	NPL6	POB3	RSC1	RSC2	RSC3	RSC30	RSC4	RSC58	RSC6	RSC8	RSC9	RTT102	SFH1	SPT16	STH1	TOP2	YTA7
significance	5.4	6.8	5.1	5.6	4.6	11.0	6.7	7.4	9.4	6.1	6.2	8.5	10.0	9.5	17.0	7.6	6.1	5.0	7.0	10.0	6.2	4.9
interactor class	A+	A+	A+	A+	B+	A+	B+	A+	A+	A+	A+	A+	A+	A+	A+	A+	A+	B+	A+	A+	B+	B+

**bait**	SET1
**experiment**	27 minutes

**interactors**	12
**recovery**	86 %

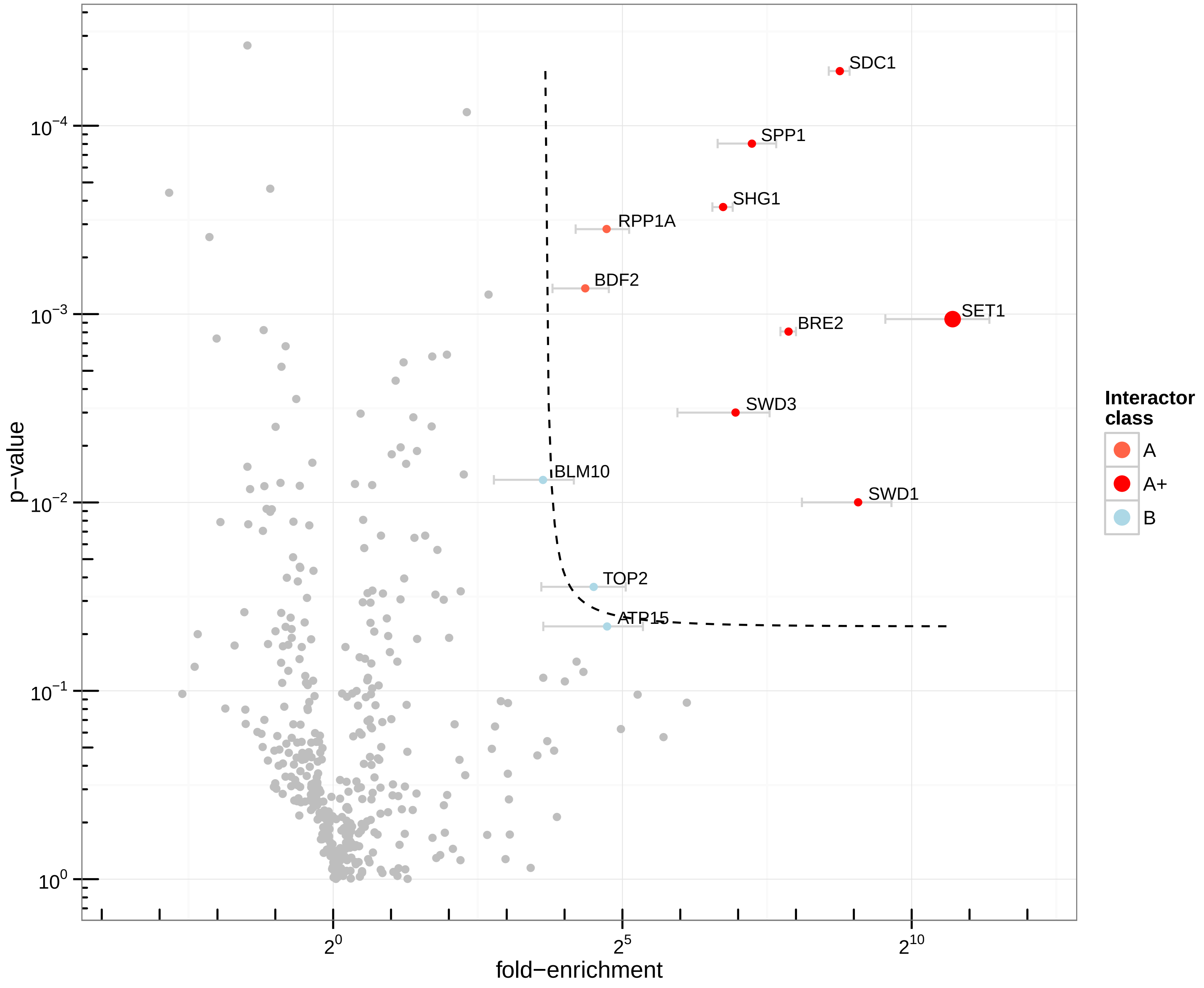

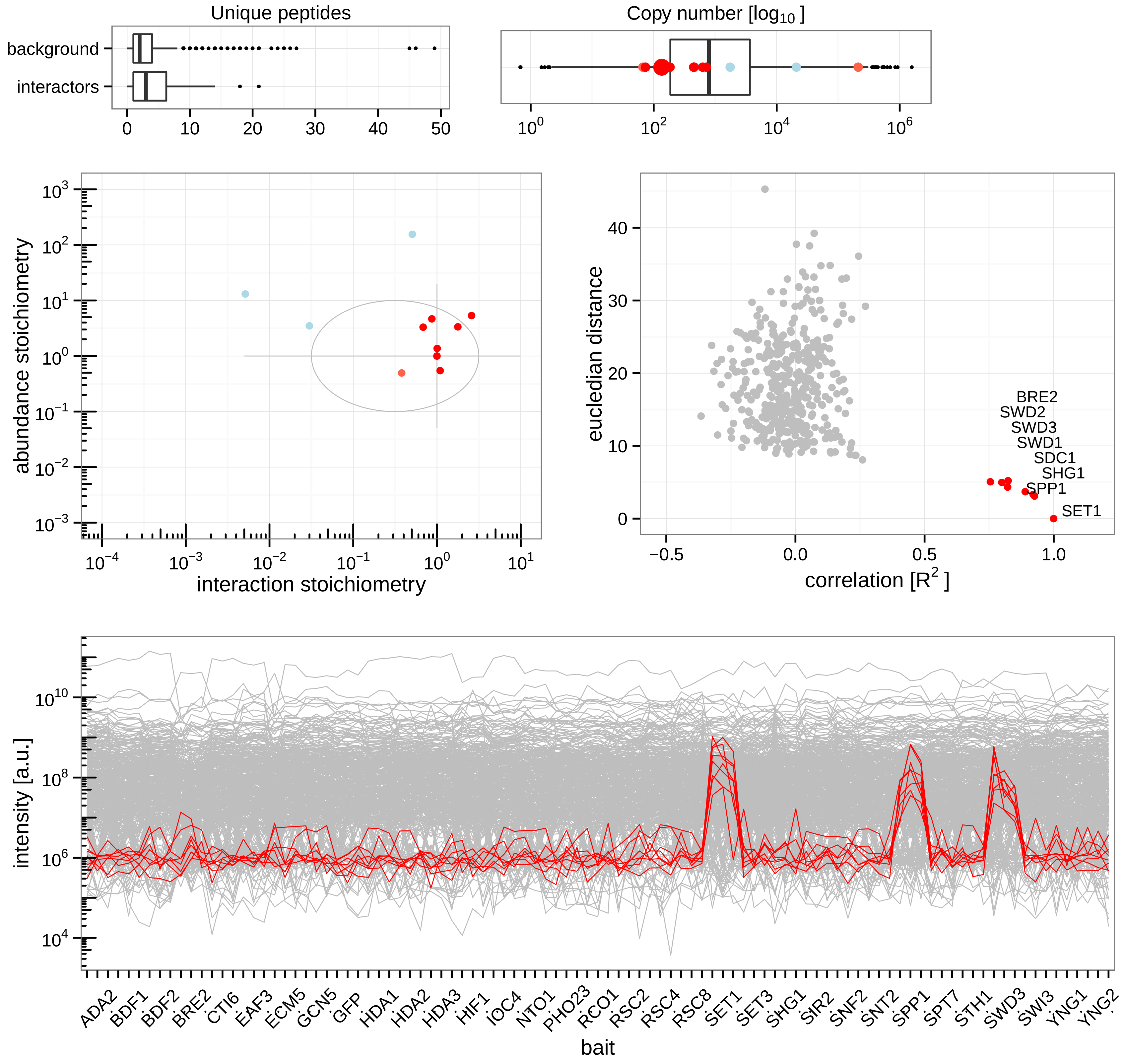

	BDF2	BRE2	RPP1A	SDC1	SET1	SHG1	SPP1	SWD1	SWD3
significance	4.6	7.5	5.1	8.9	10.0	6.8	7.4	8.3	6.5
interactor class	A	A+	A	A+	A+	A+	A+	A+	A+

**bait**	SET3
**experiment**	27 minutes

**interactors**	4
**recovery**	18 %

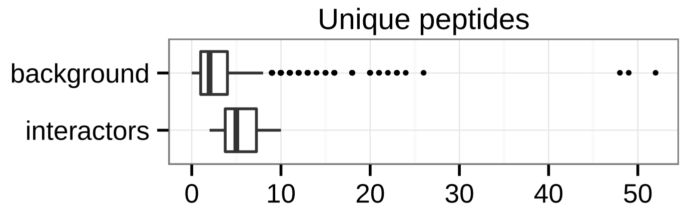

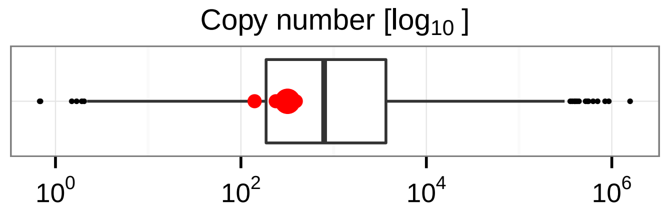

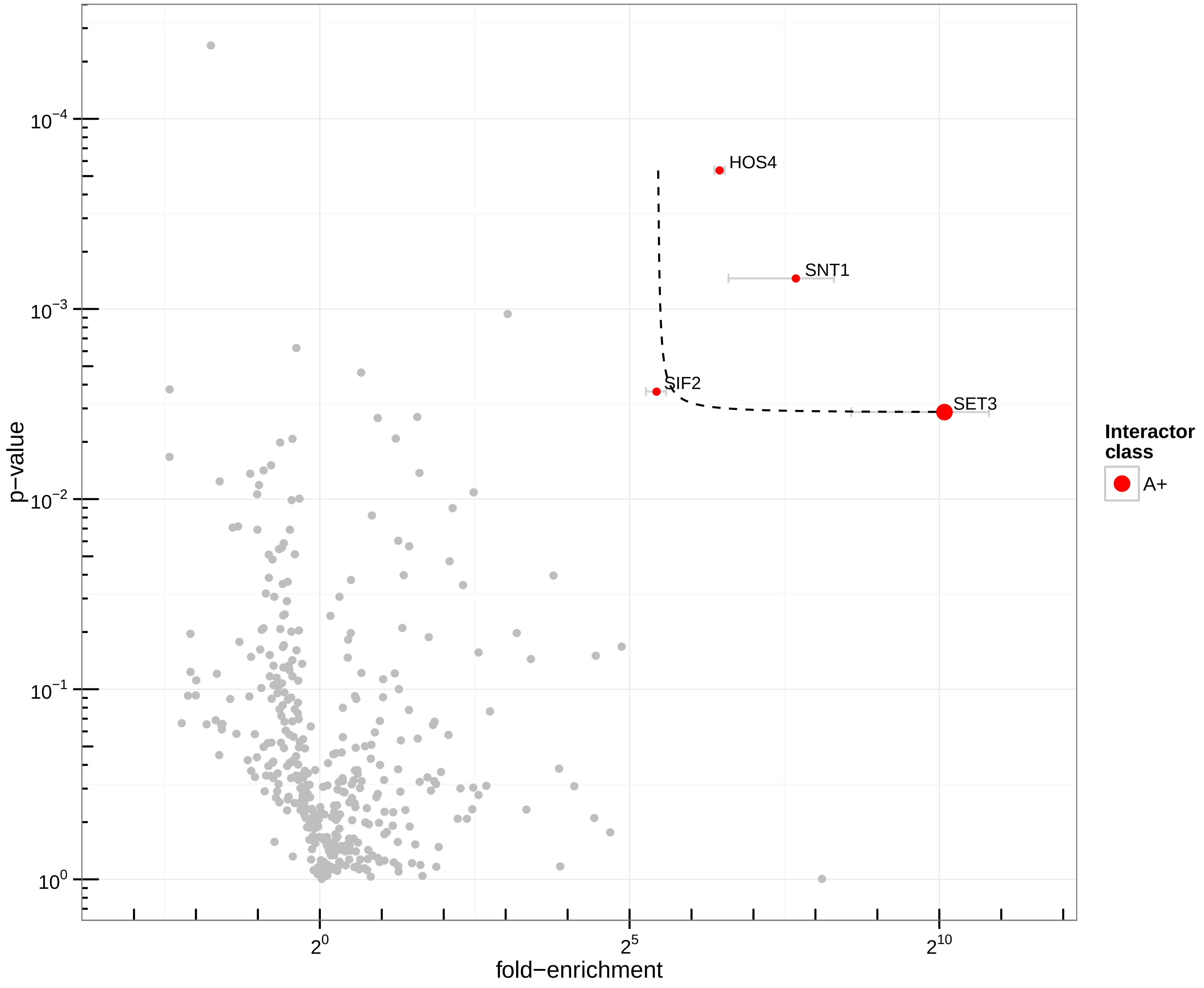

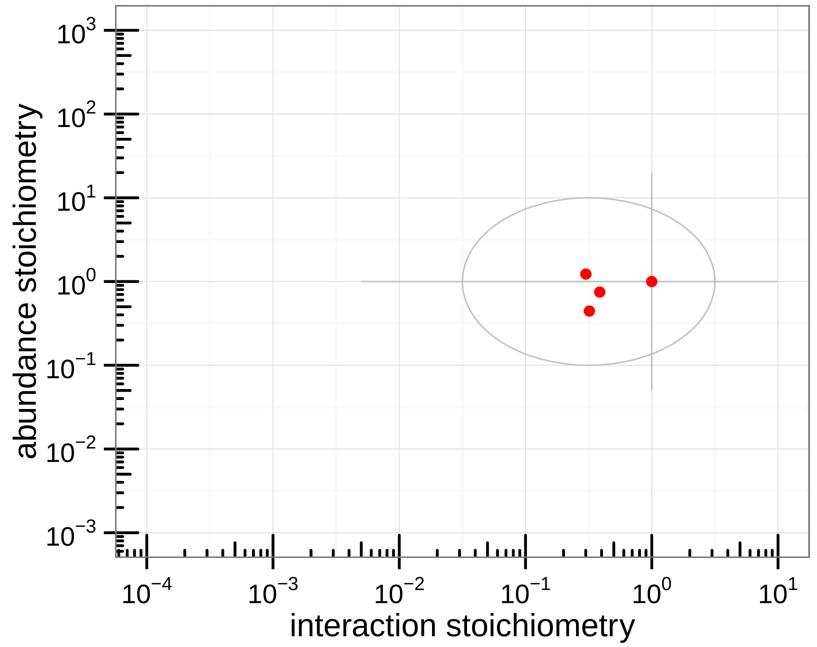

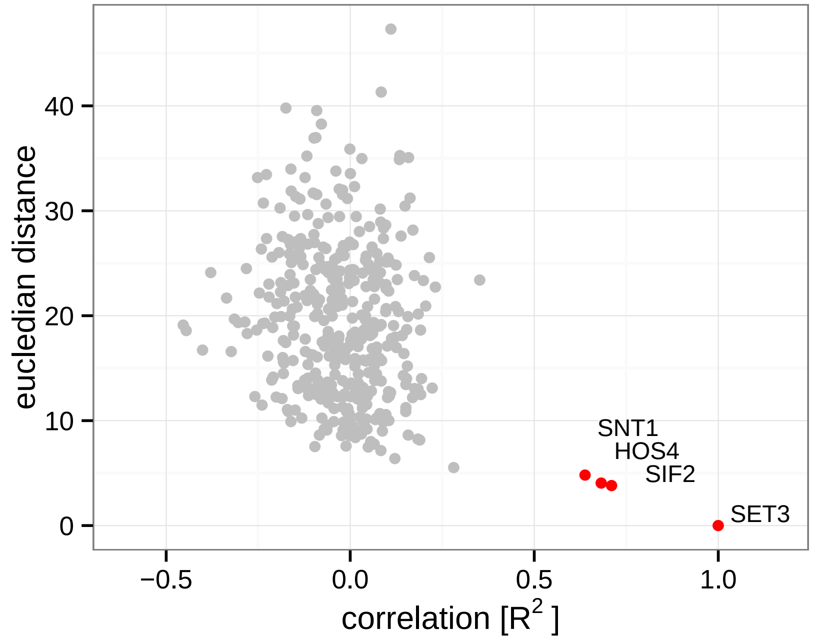

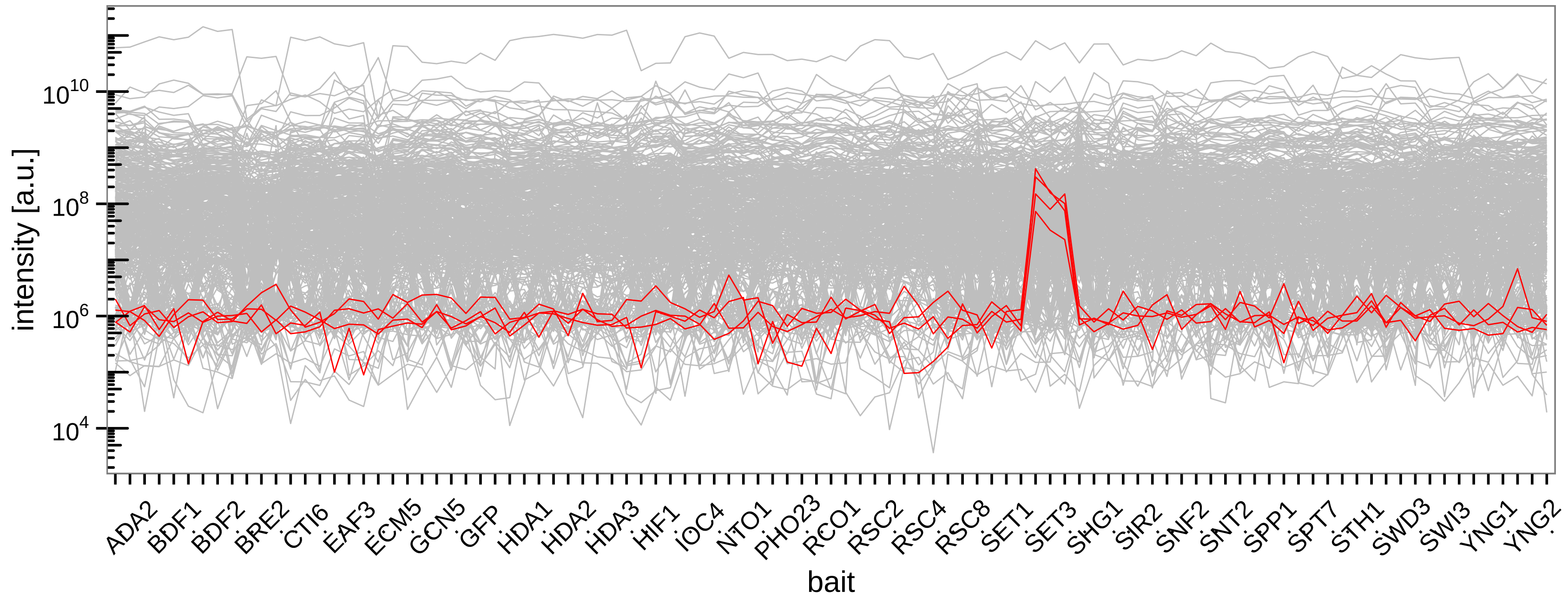

	HOS4	SET3	SIF2	SNT1
significance	6.6	9.4	5.1	7.4
interactor class	A+	A+	A+	A+

**bait**	SHG1
**experiment**	27 minutes

**interactors**	1
**recovery**	0 %

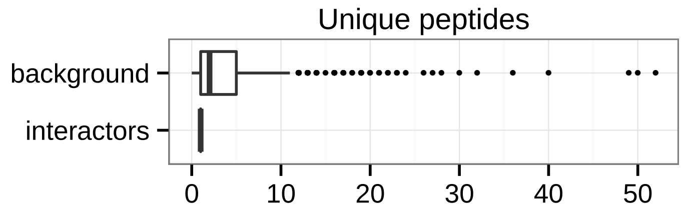

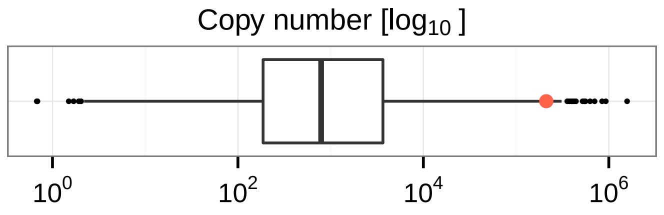

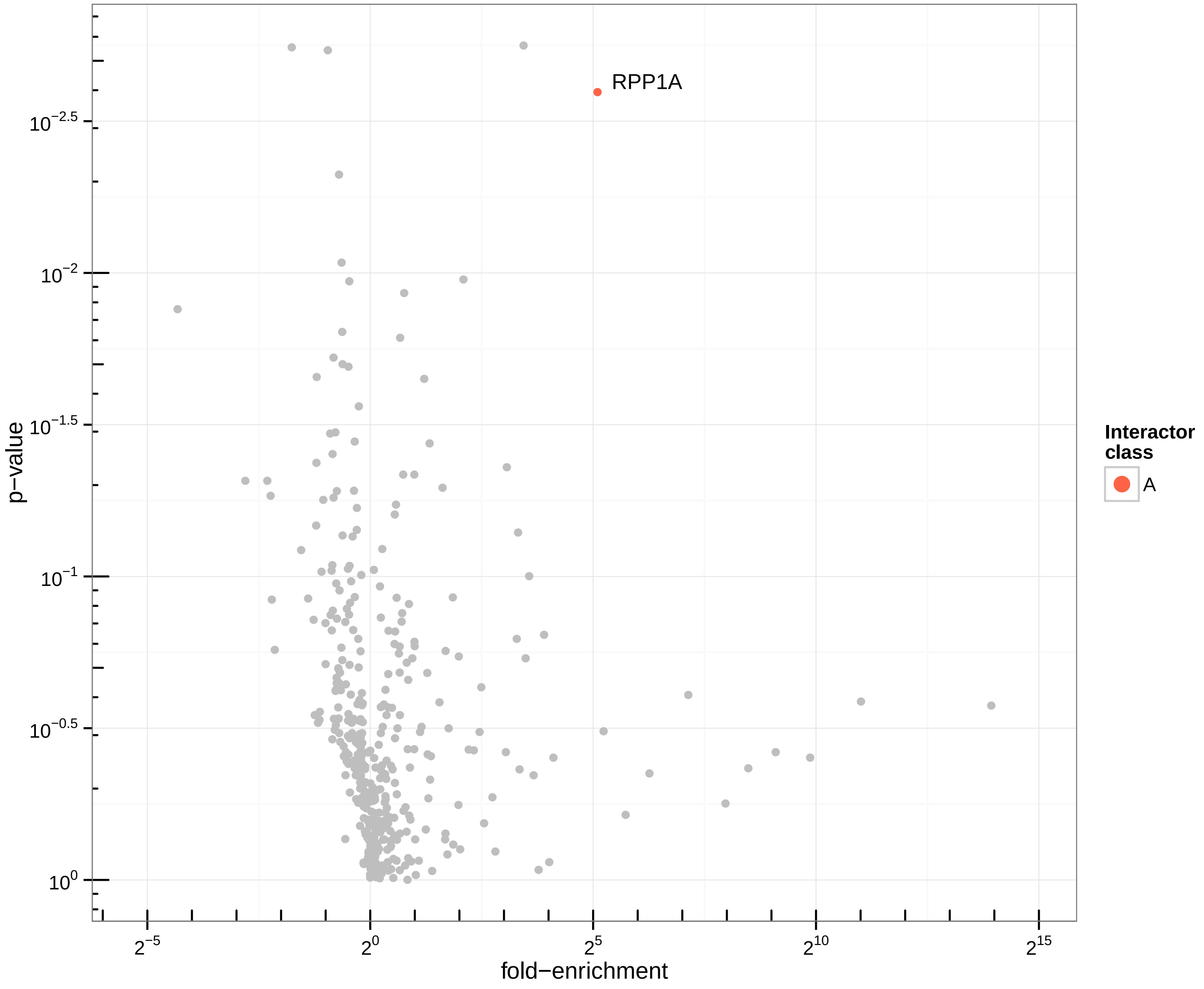

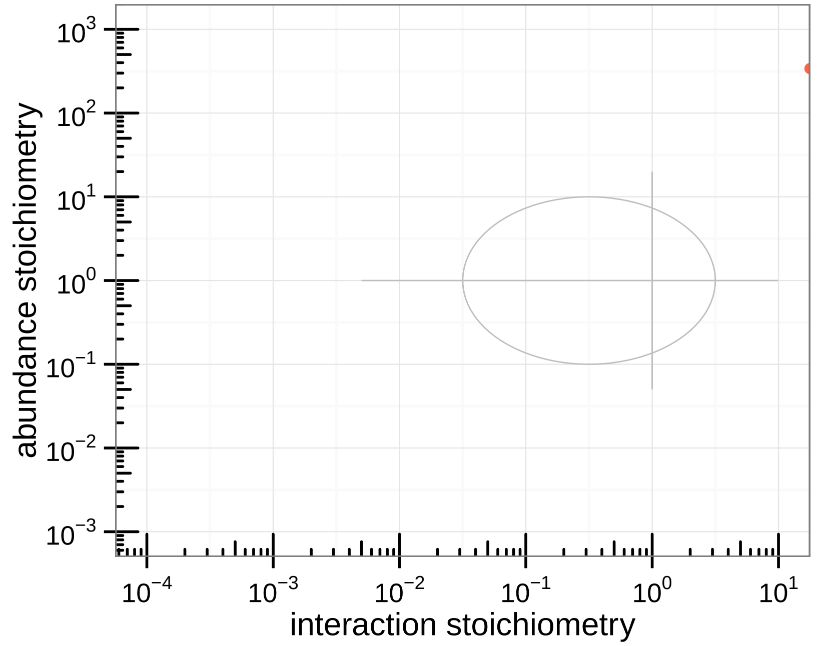

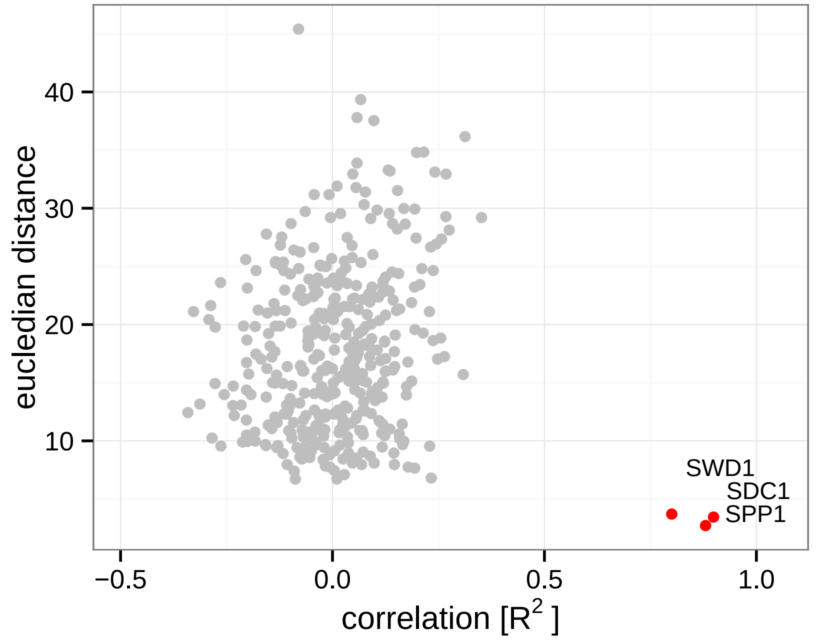

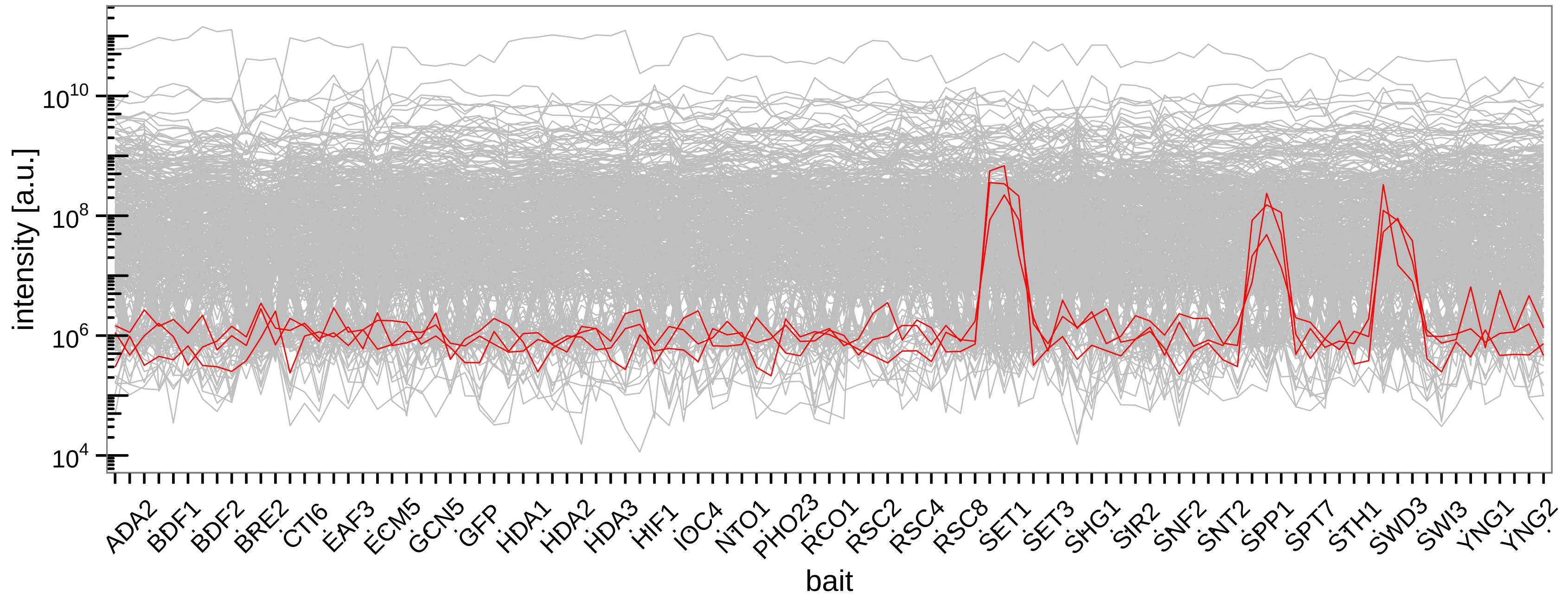

	RPP1A
significance	4.9
interactor class	A

**bait**	SIR2
**experiment**	27 minutes

**interactors**	2
**recovery**	20 %

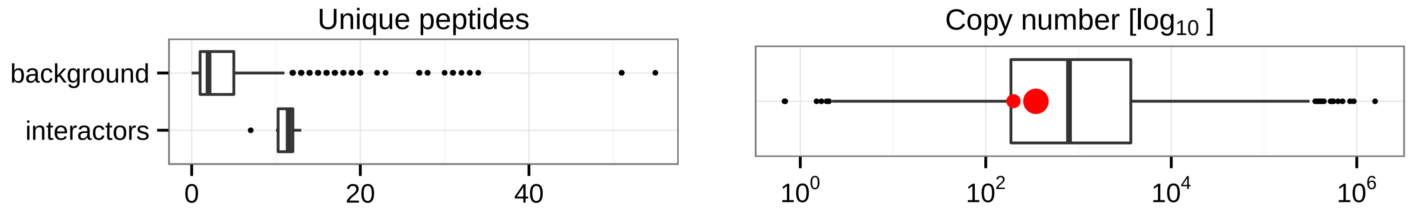

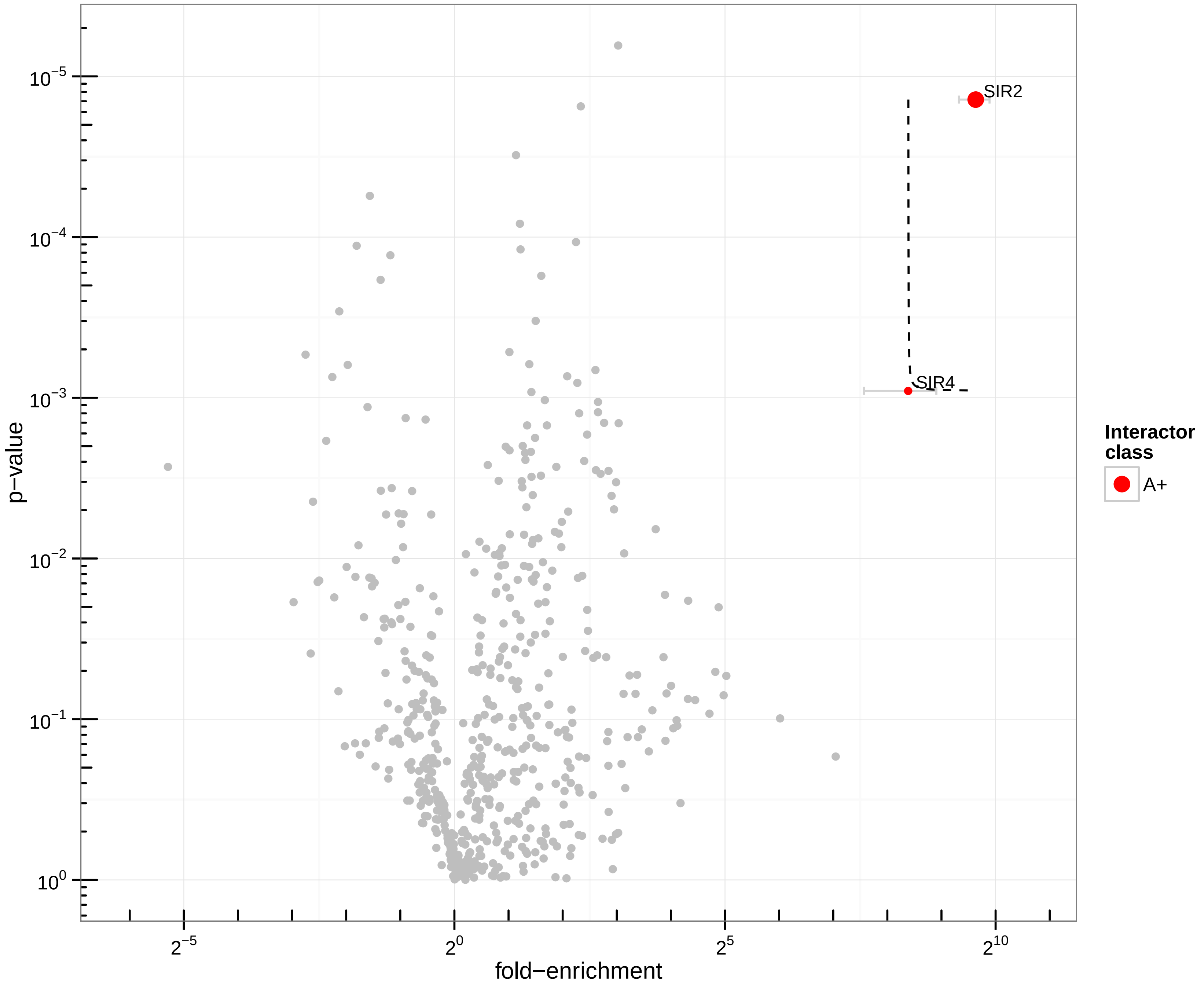

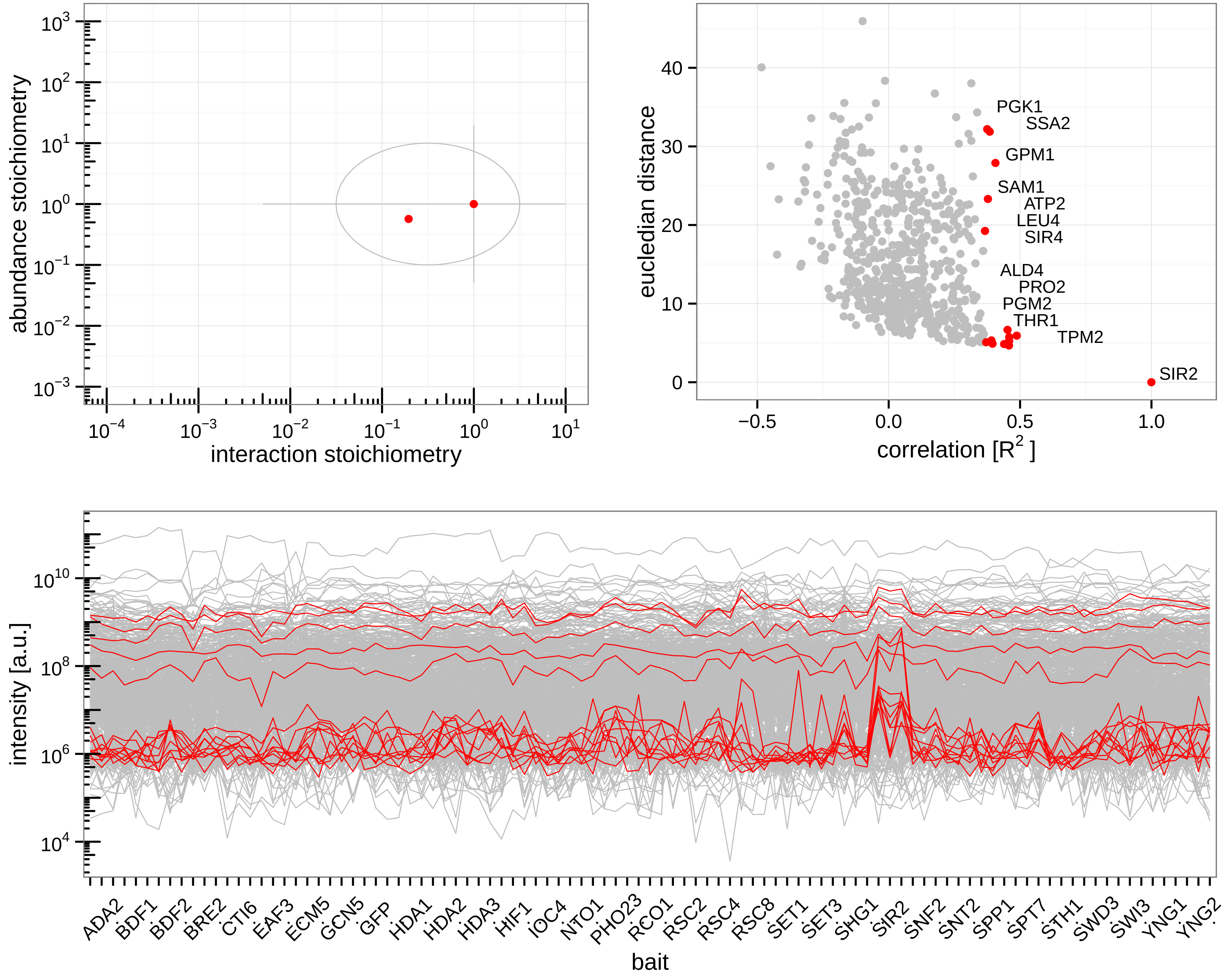

	SIR2	SIR4
significance	9.9	8.0
interactor class	A+	A+

bait	SNF2
experiment	27 minutes

interactors	12
recovery	91 %

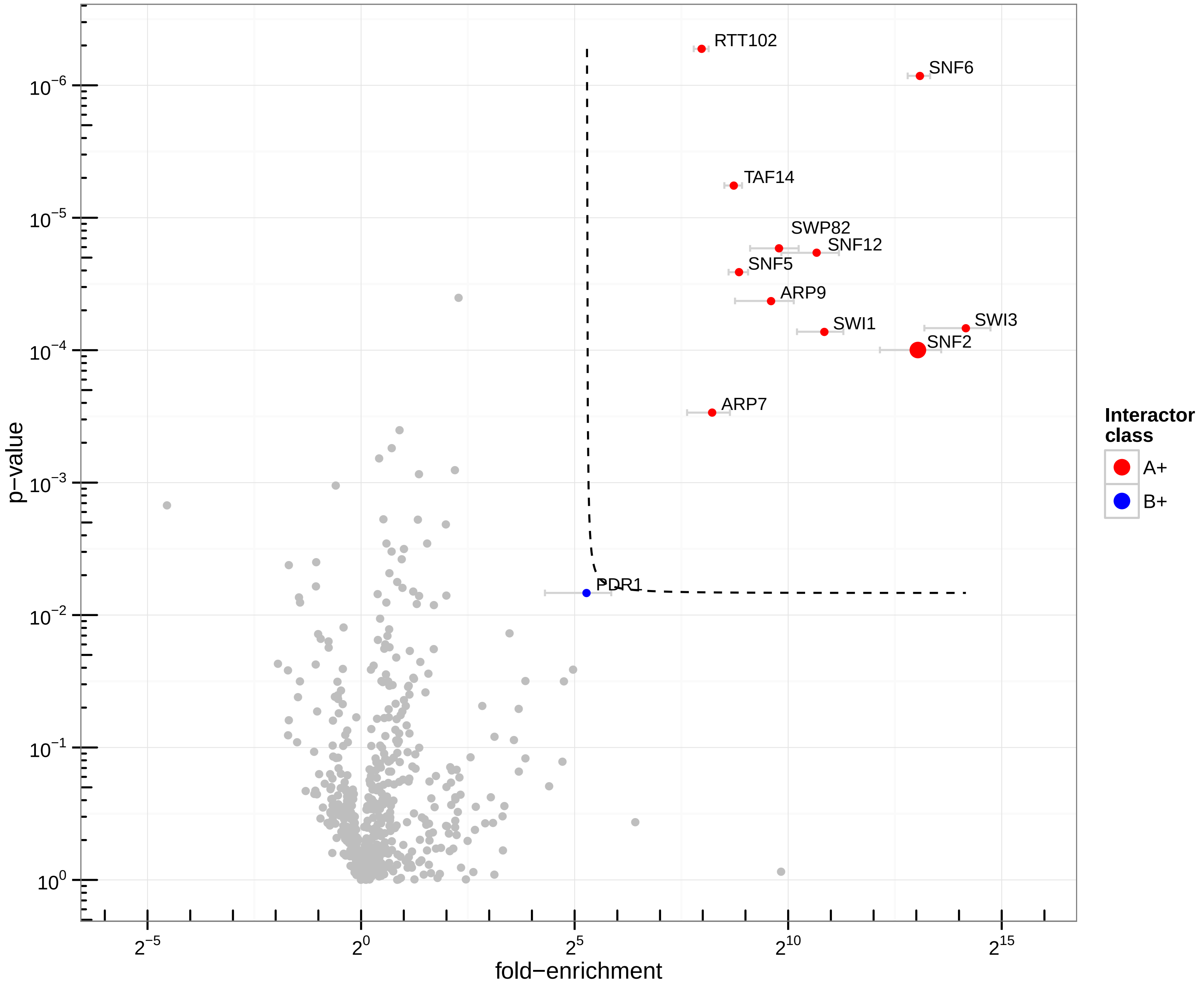

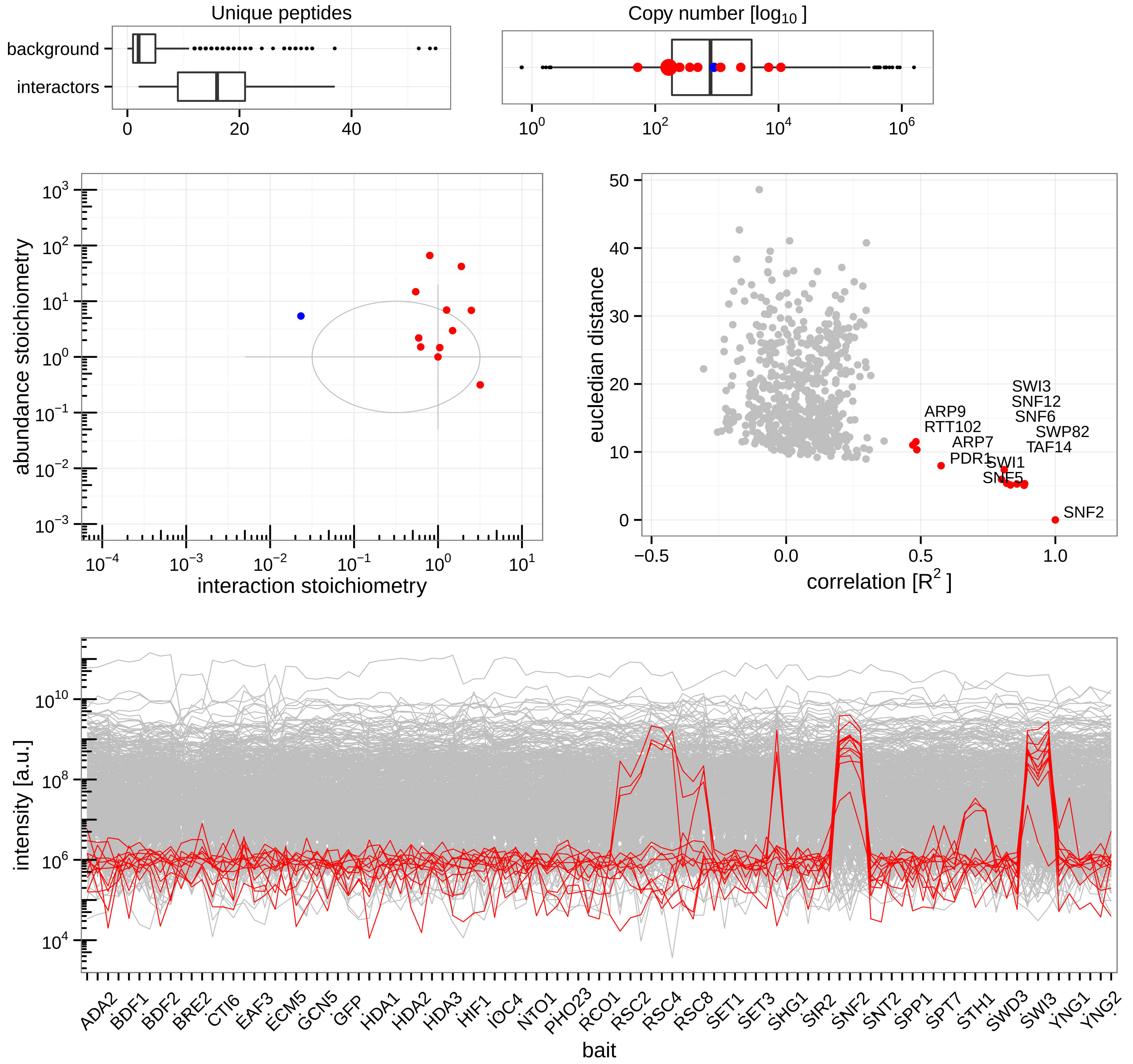

	ARP7	ARP9	PDR1	RTT102	SNF12	SNF2	SNF5	SNF6	SWI1	SWI3	SWP82	TAF14
significance	8.0	9.6	4.8	9.4	11.0	13.0	9.1	14.0	11.0	14.0	10.0	9.3
interactor class	A+	A+	B+	A+	A+	A+	A+	A+	A+	A+	A+	A+

**bait**	SNT2
**experiment**	27 minutes

**interactors**	0
**recovery**	0 %

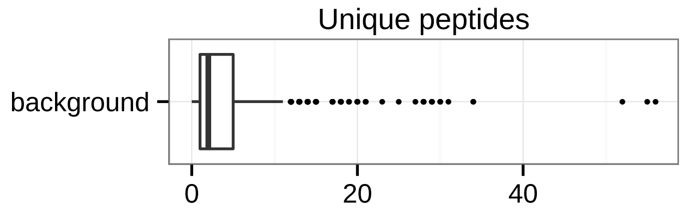

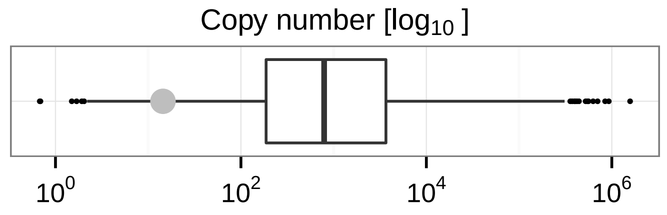

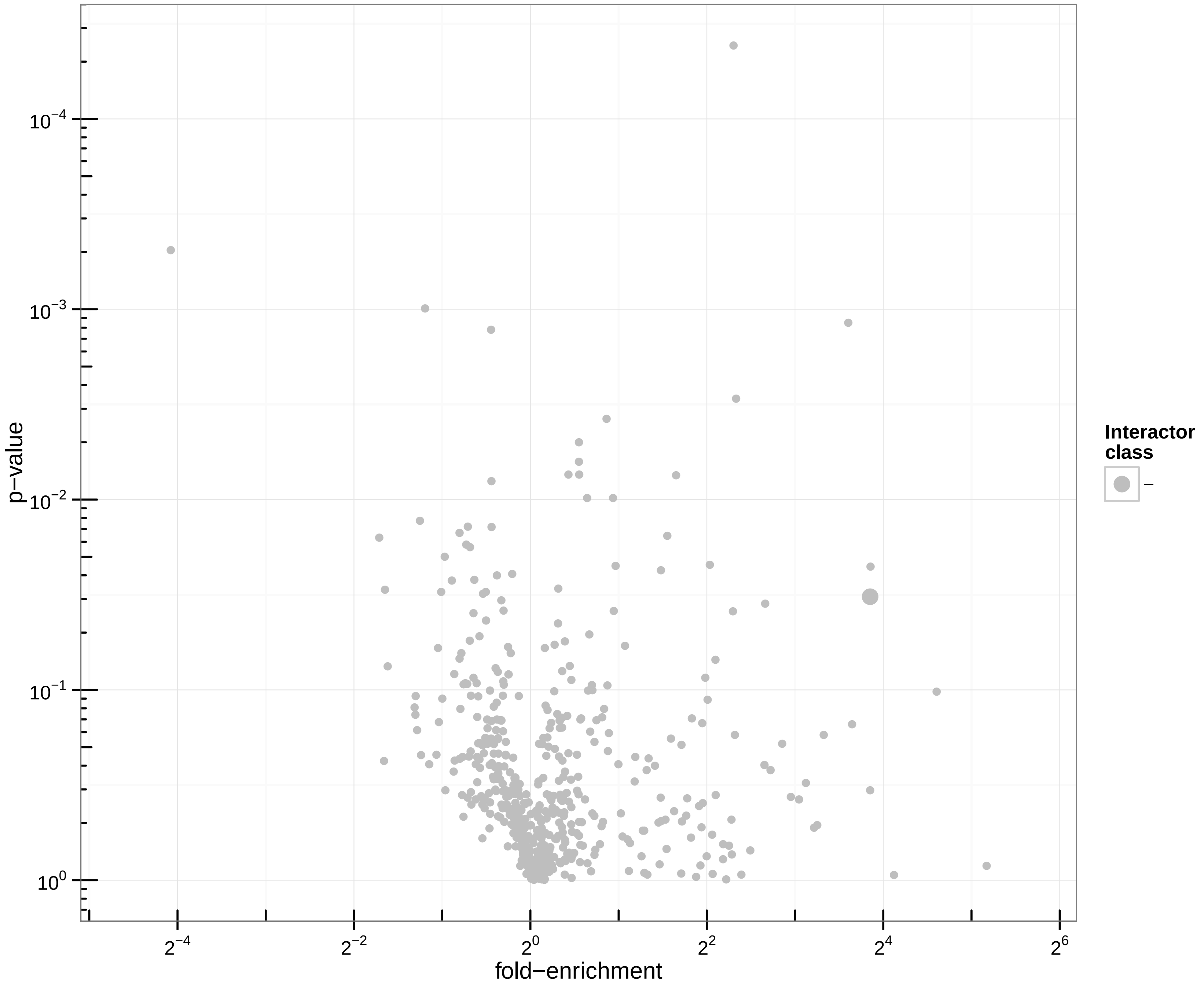

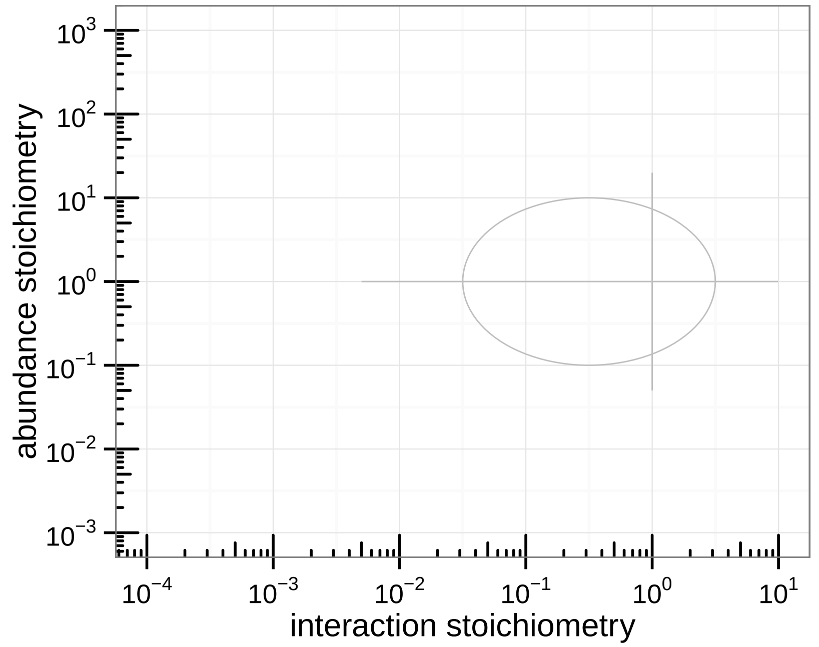

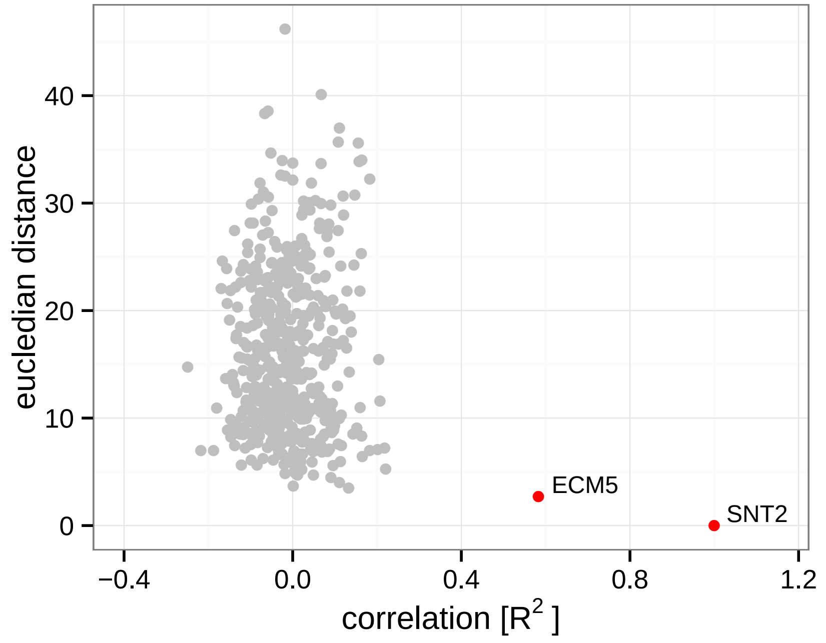

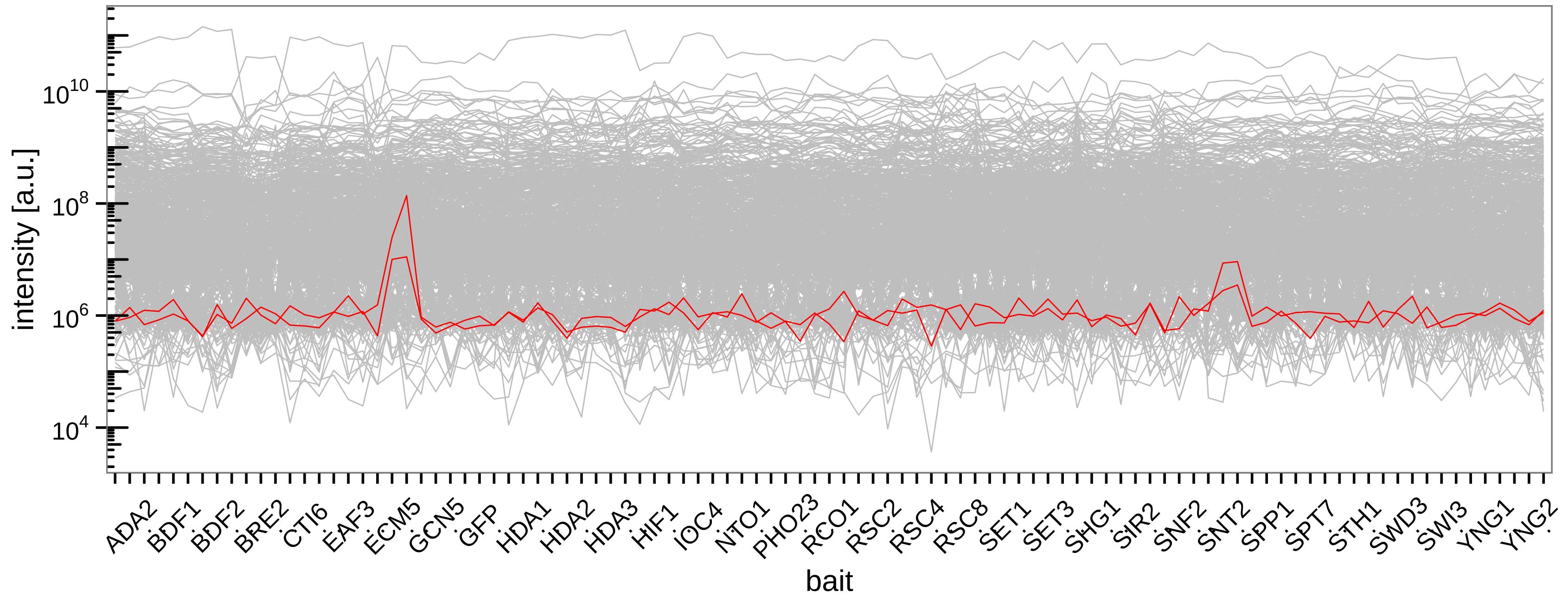

significance

interactor class

**bait**	SPP1
**experiment**	27 minutes

**interactors**	8
**recovery**	100 %

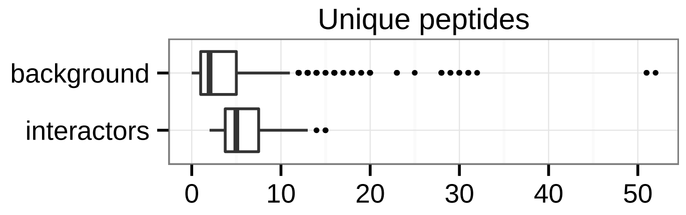

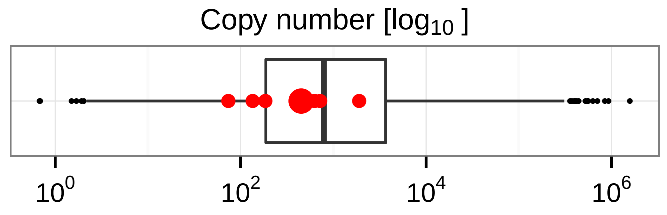

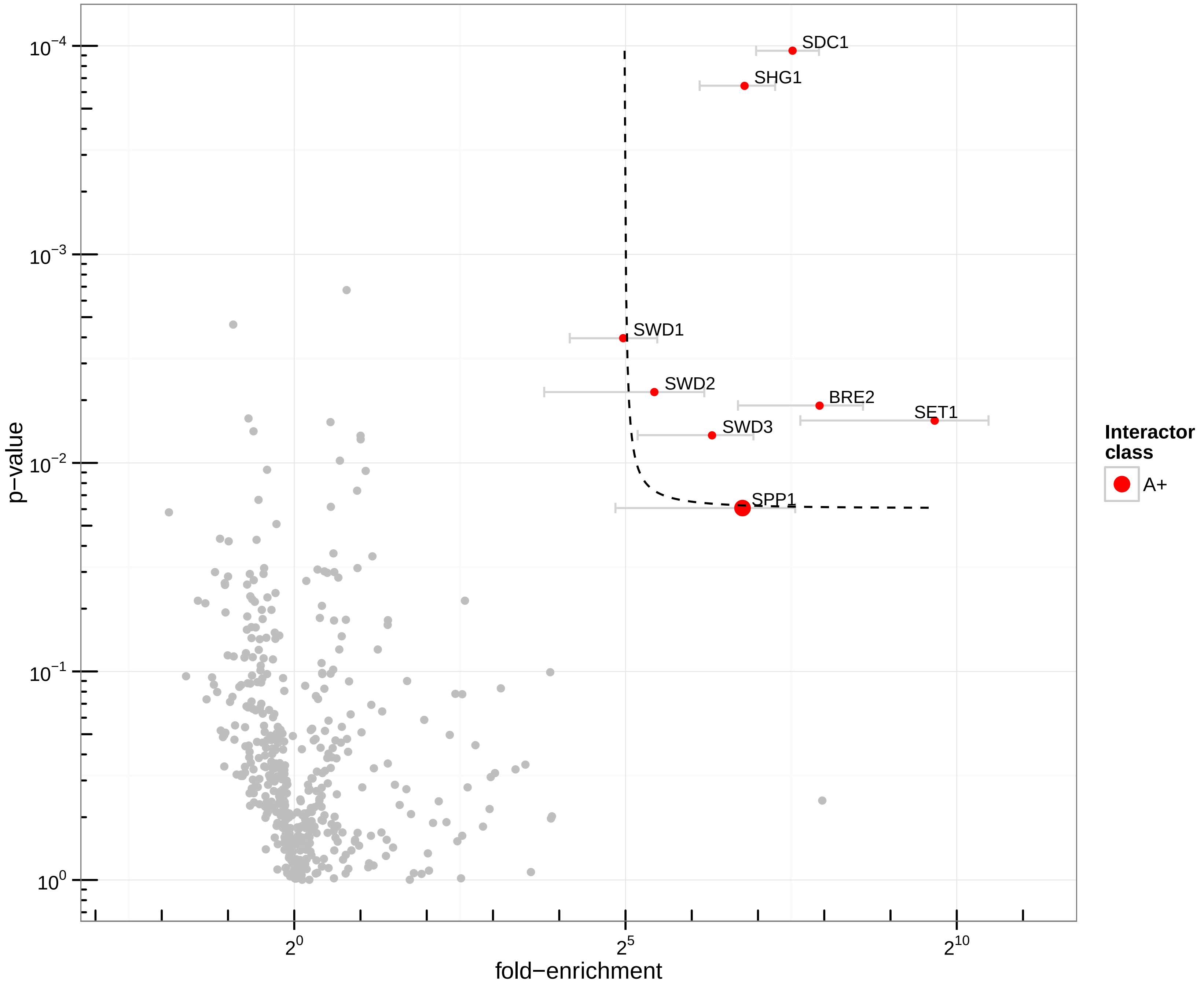

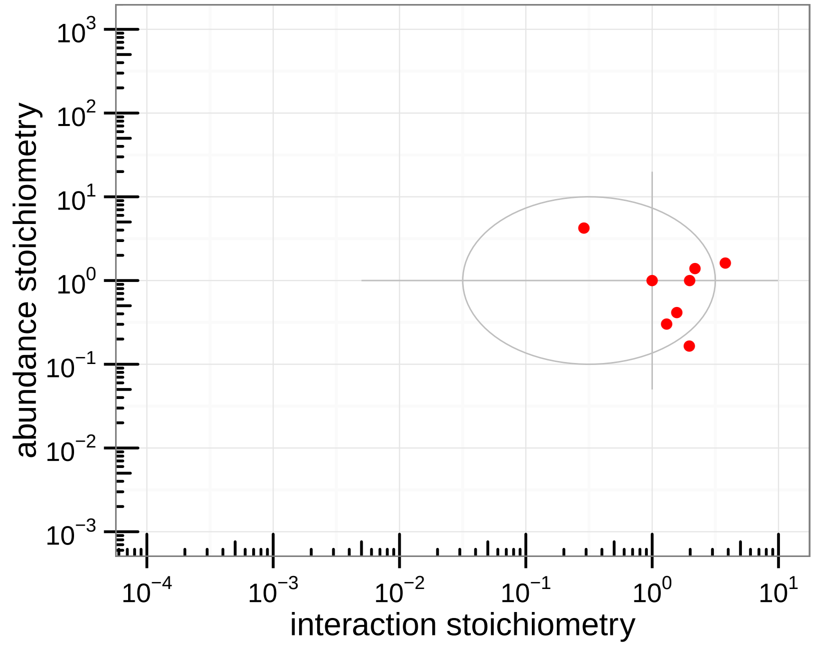

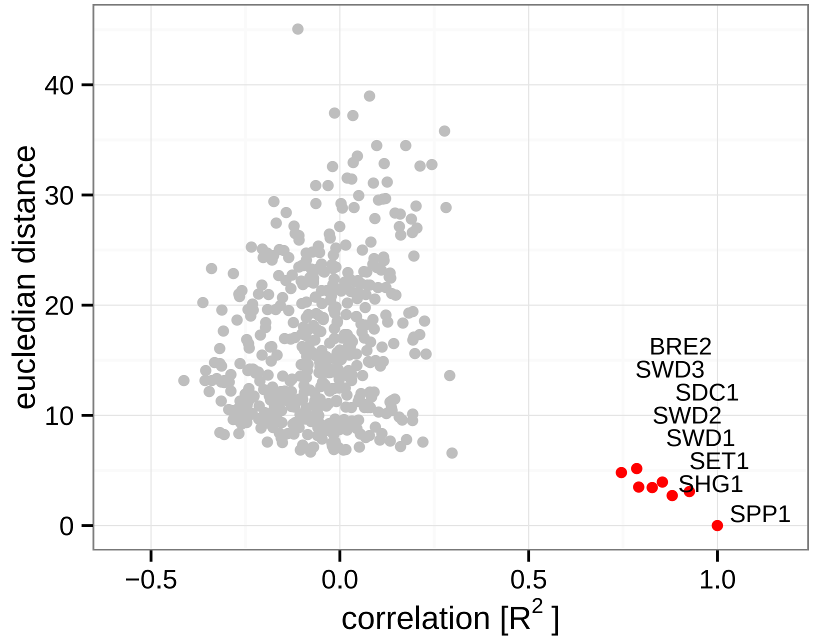

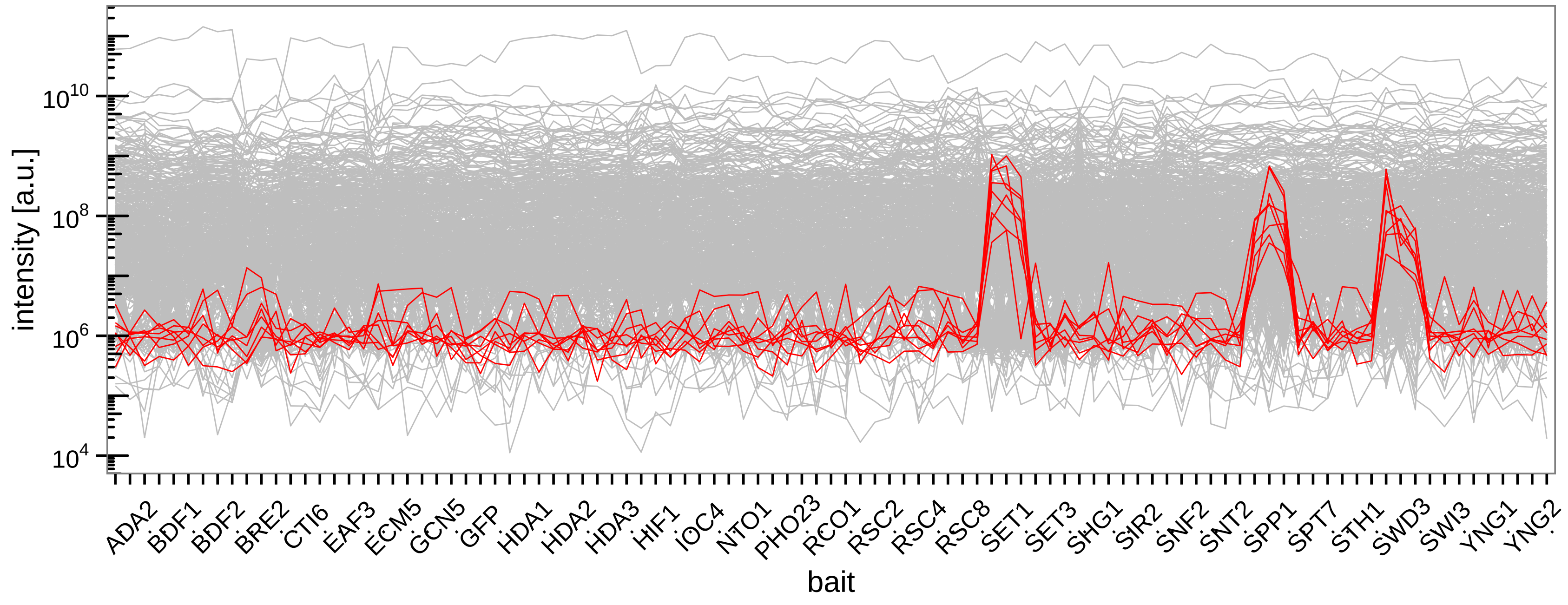

	BRE2	SDC1	SET1	SHG1	SPP1	SWD1	SWD2	SWD3
significance	7.3	7.6	8.9	6.9	6.0	4.7	5.0	5.7
interactor class	A+	A+	A+	A+	A+	A+	A+	A+

**bait**	SPT7	**interactors**	18
**experiment**	27 minutes	**recovery**	74 %

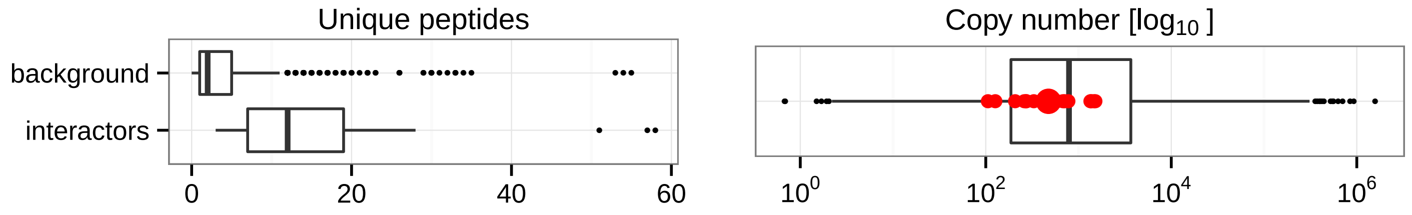

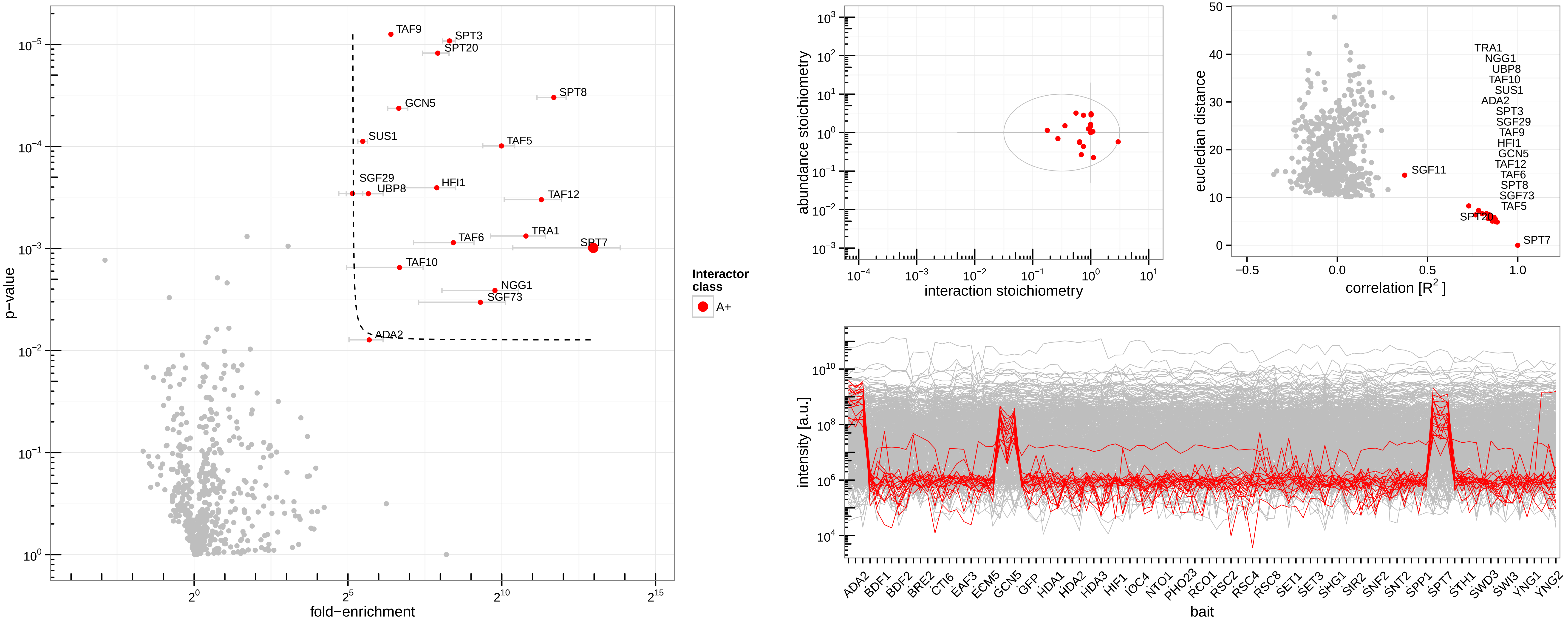

	ADA2	GCN5	HFI1	NGG1	SGF29	SGF73	SPT20	SPT3	SPT7	SPT8	SUS1	TAF10	TAF12	TAF5	TAF6	TAF9	TRA1	UBP8
significance	5.1	7.1	7.8	9.2	5.4	8.7	8.5	8.9	12.0	12.0	6.0	6.3	11.0	9.8	8.0	7.4	10.0	5.9
interactor class	A+	A+	A+	A+	A+	A+	A+	A+	A+	A+	A+	A+	A+	A+	A+	A+	A+	A+

**bait**	STH1
**experiment**	27 minutes

**interactors**	18
**recovery**	71 %

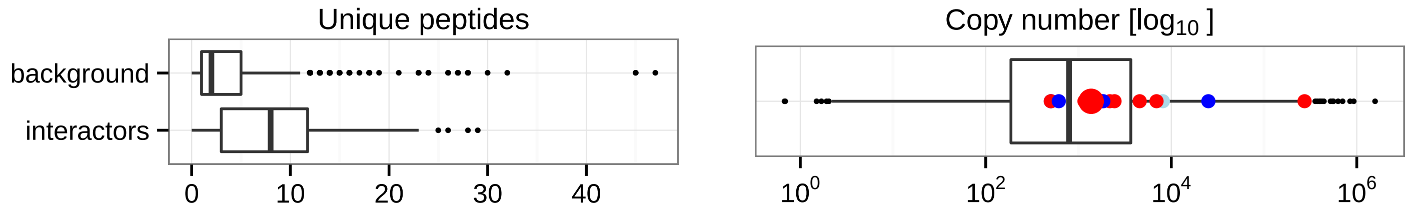

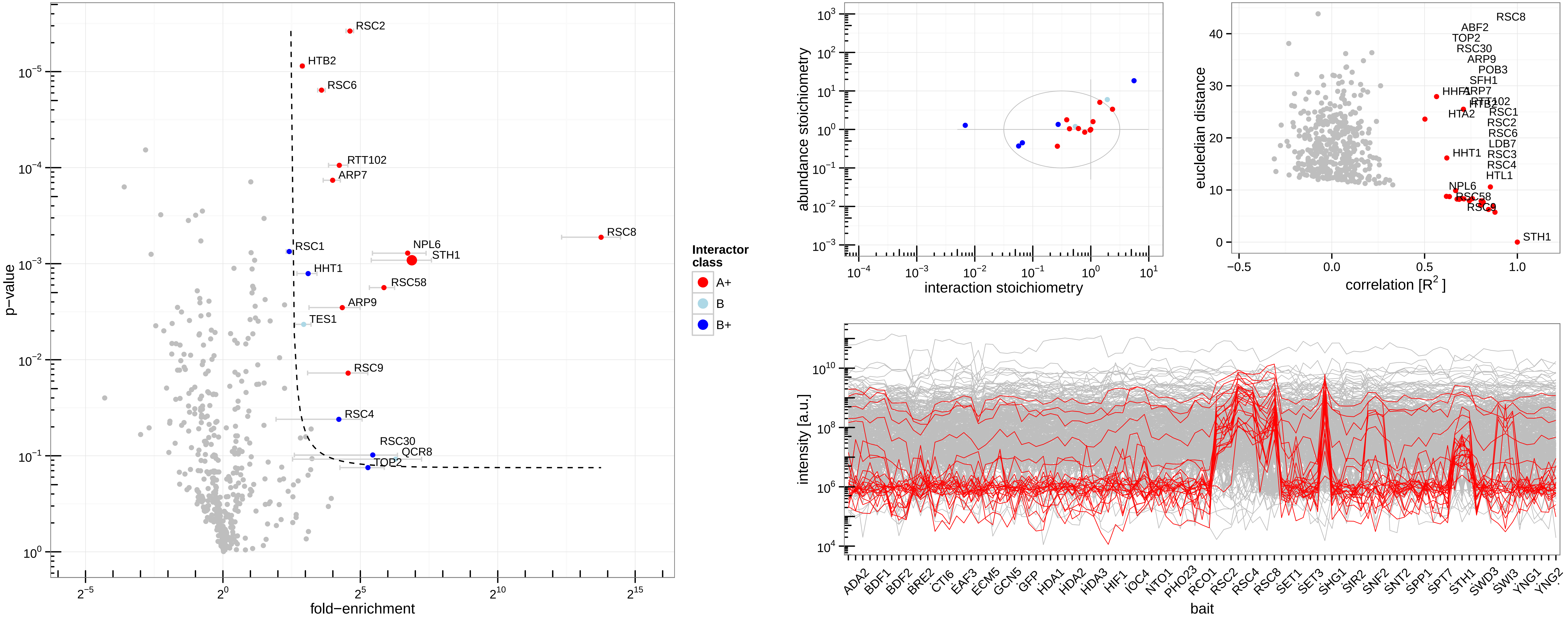

	ARP7	ARP9	HHT1	HTB2	NPL6	RSC1	RSC2	RSC30	RSC4	RSC58	RSC6	RSC8	RSC9	RTT102	STH1	TOP2
significance	4.9	4.2	3.6	5.4	6.5	3.4	6.5	4.6	3.5	5.6	5.5	13.0	4.0	5.2	6.6	4.4
interactor class	A+	A+	B+	A+	A+	B+	A+	B+	B+	A+	A+	A+	A+	A+	A+	B+

**bait**	SWD3
**experiment**	27 minutes

**interactors**	8
**recovery**	100 %

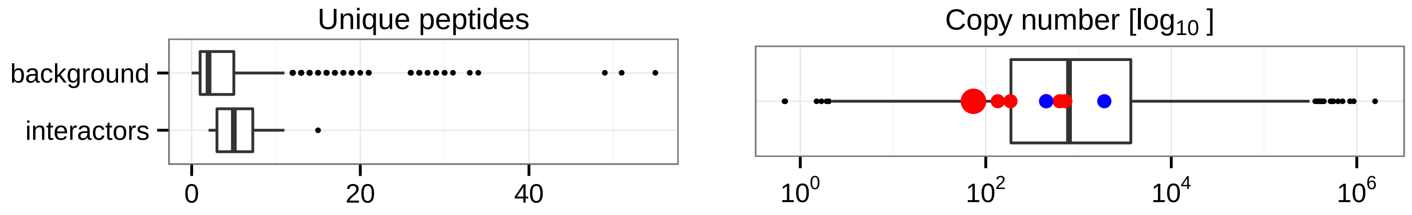

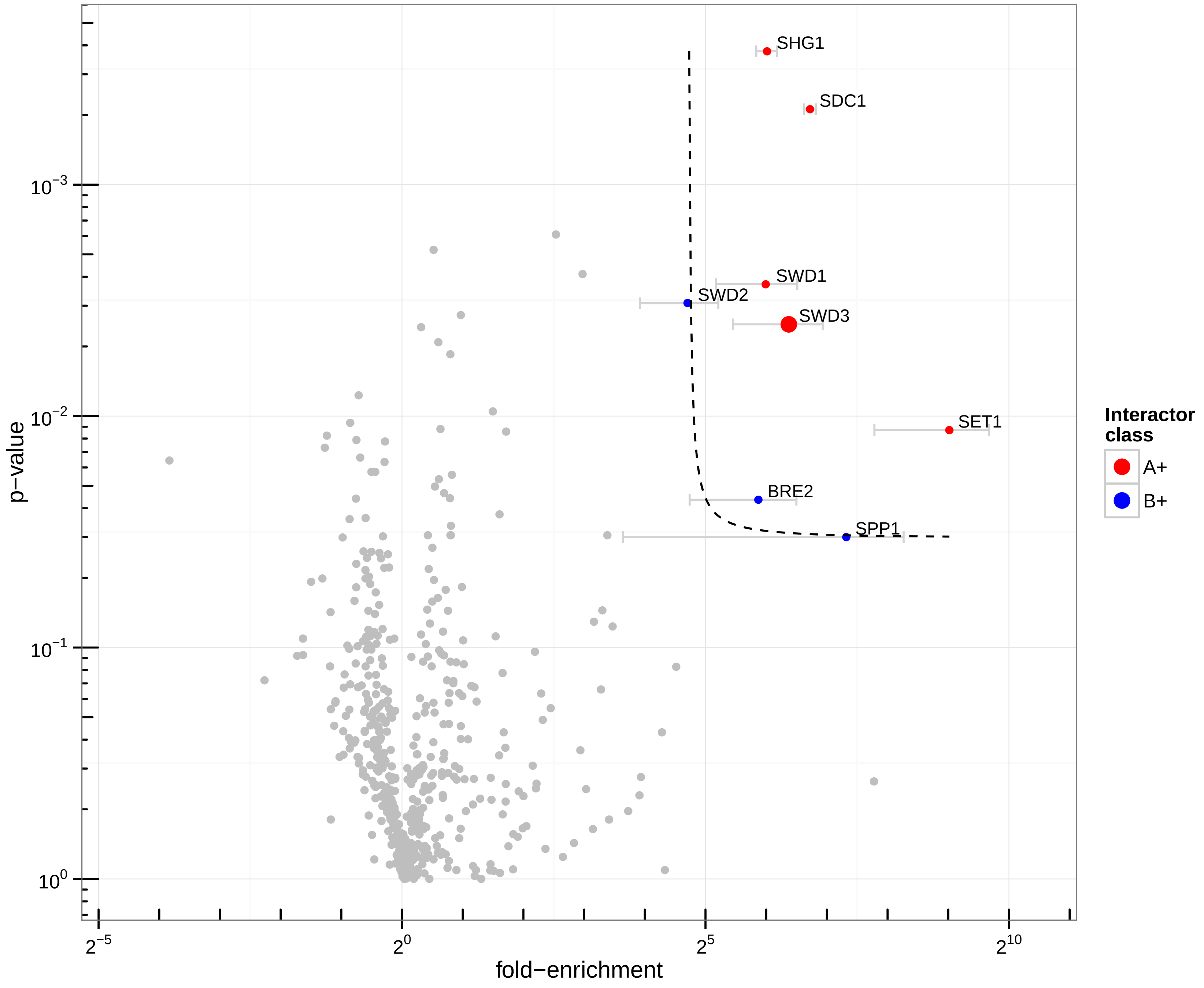

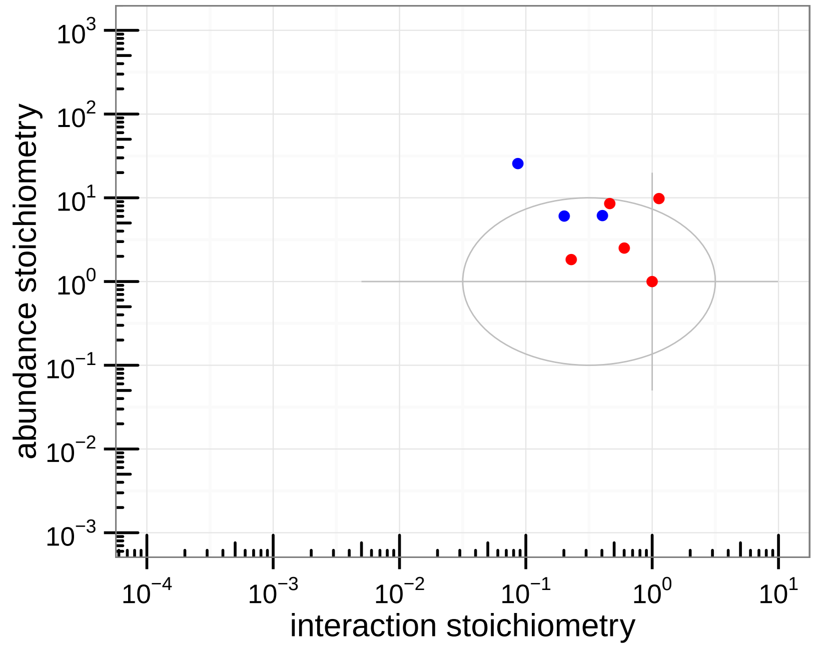

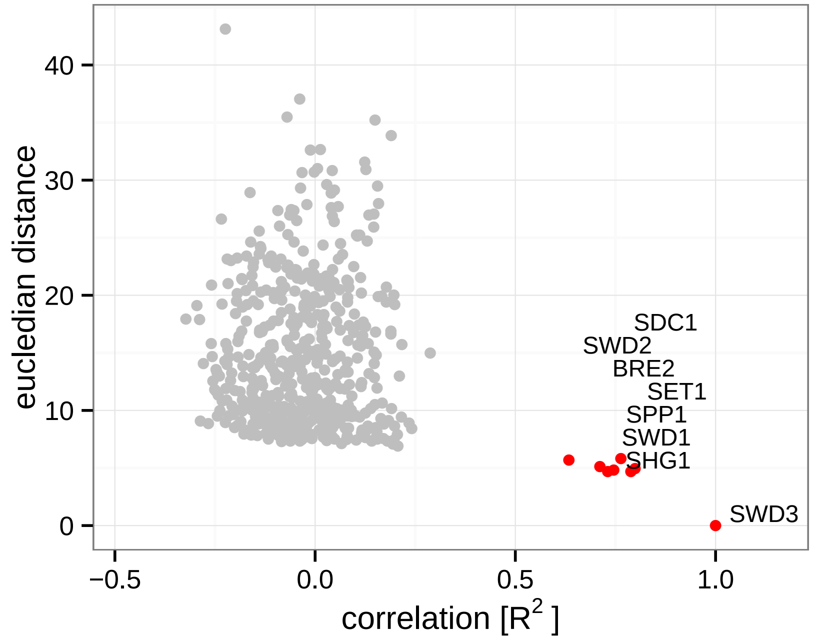

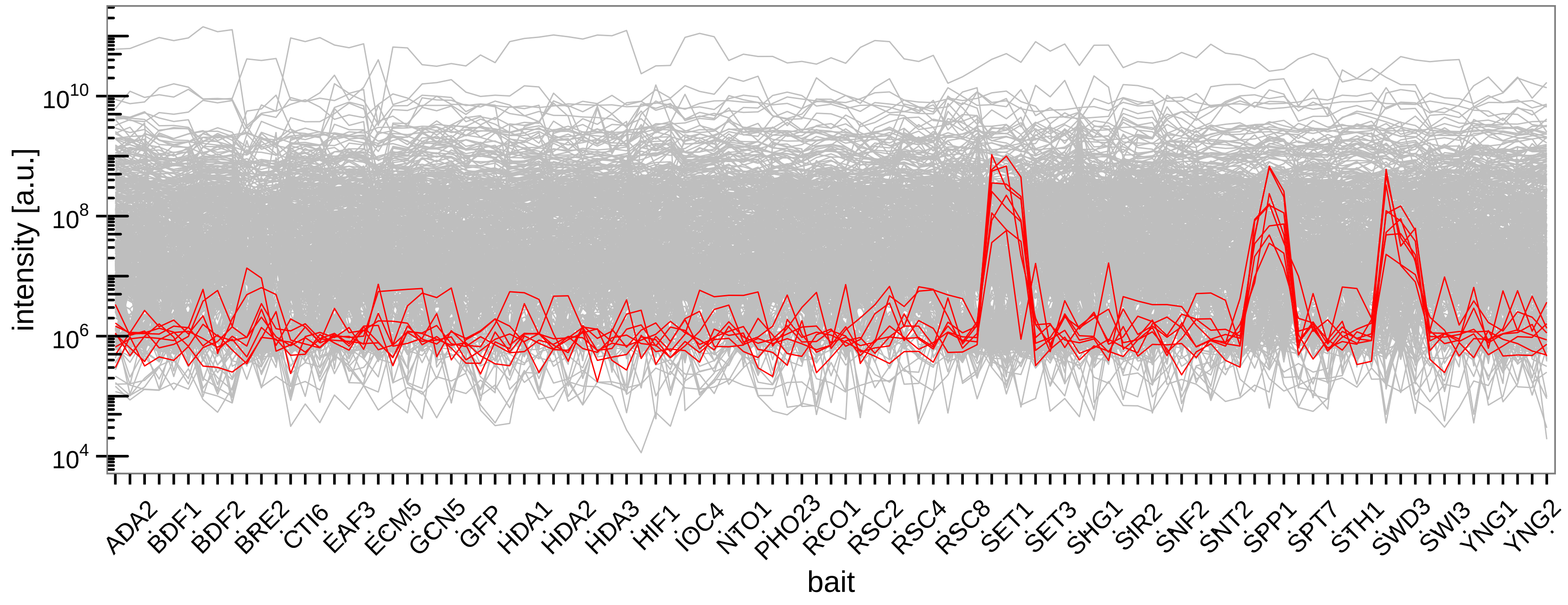

	BRE2	SDC1	SET1	SHG1	SPP1	SWD1	SWD2	SWD3
significance	5.1	6.6	8.2	6.2	6.5	5.6	4.5	5.9
interactor class	B+	A+	A+	A+	B+	A+	B+	A+

**bait**	SWI3
**experiment**	27 minutes

**interactors**	11
**recovery**	91 %

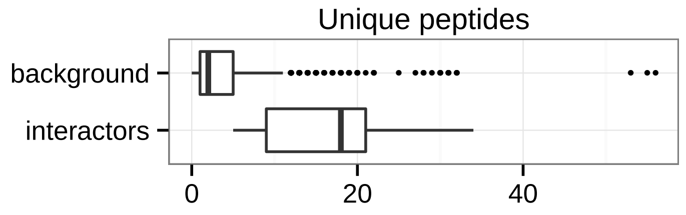

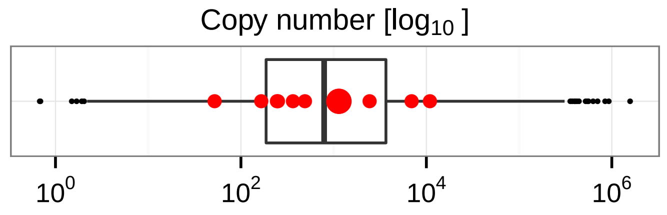

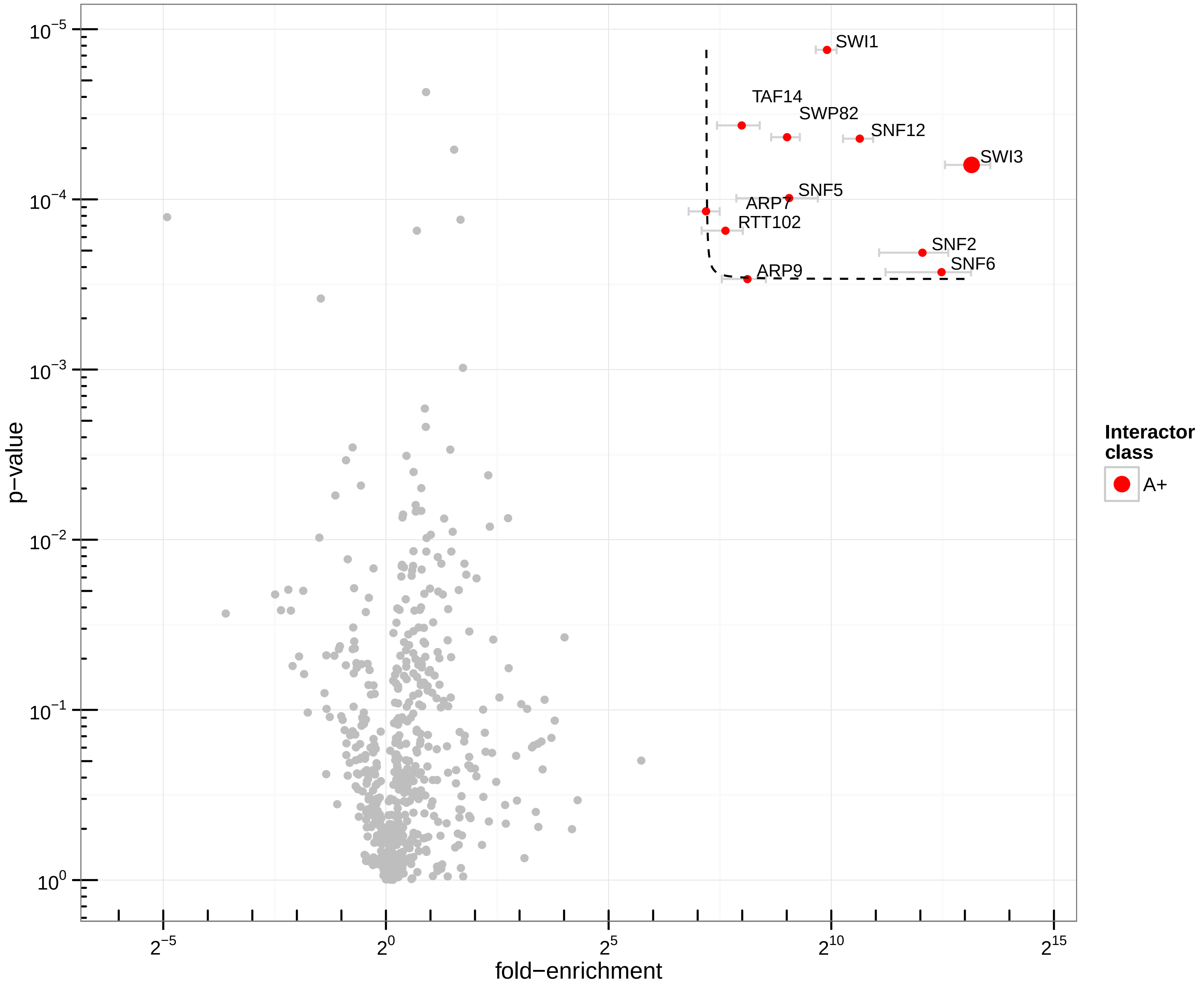

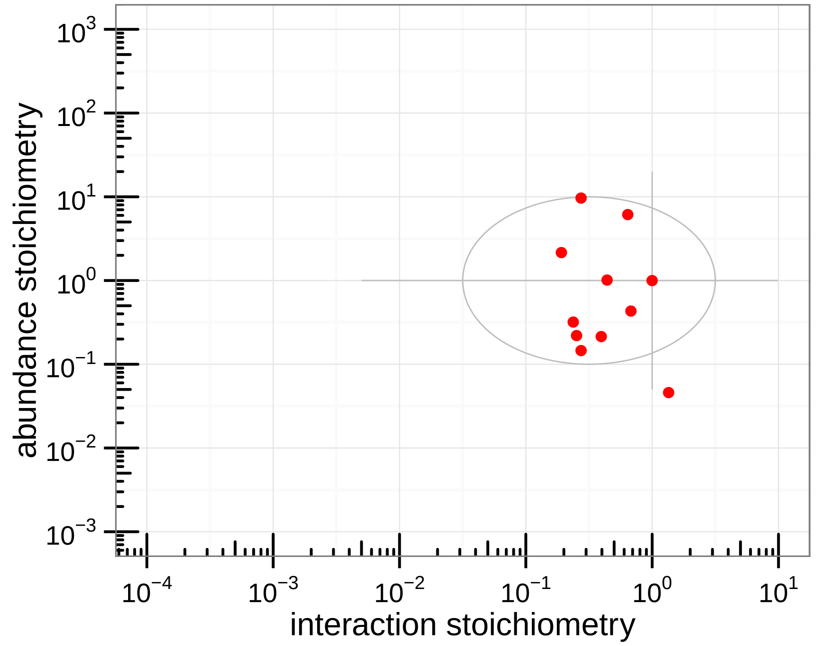

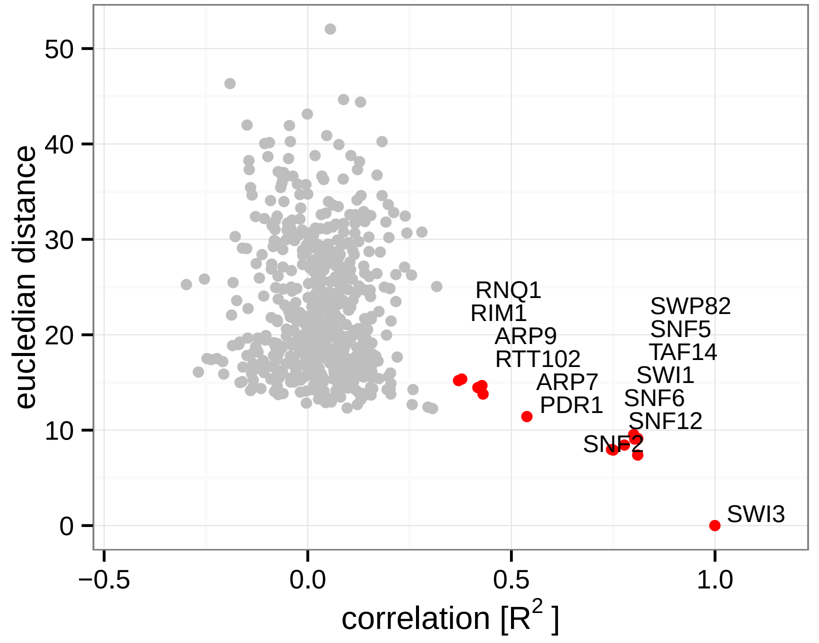

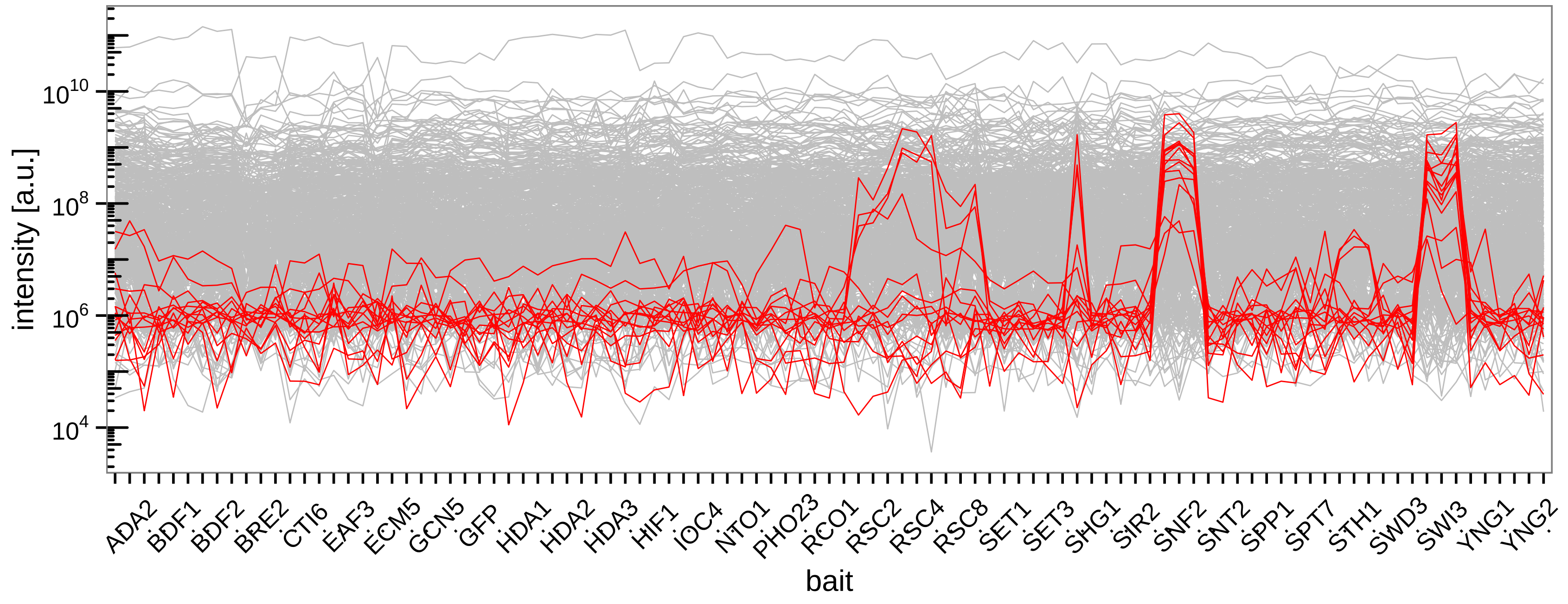

	ARP7	ARP9	RTT102	SNF12	SNF2	SNF5	SNF6	SWI1	SWI3	SWP82	TAF14
significance	7.3	7.9	7.6	11.0	12.0	9.0	12.0	10.0	13.0	9.1	8.3
interactor class	A+	A+	A+	A+	A+	A+	A+	A+	A+	A+	A+

**bait**	YNG1
**experiment**	27 minutes

**interactors**	5
**recovery**	NaN %

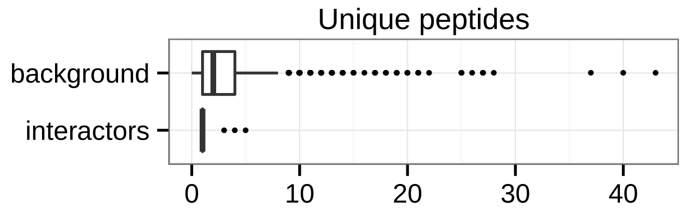

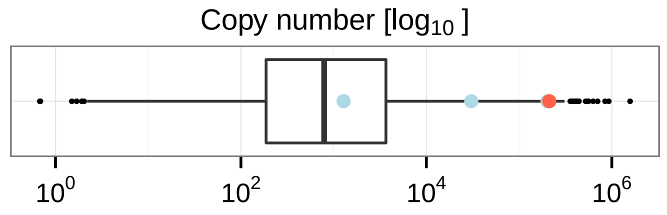

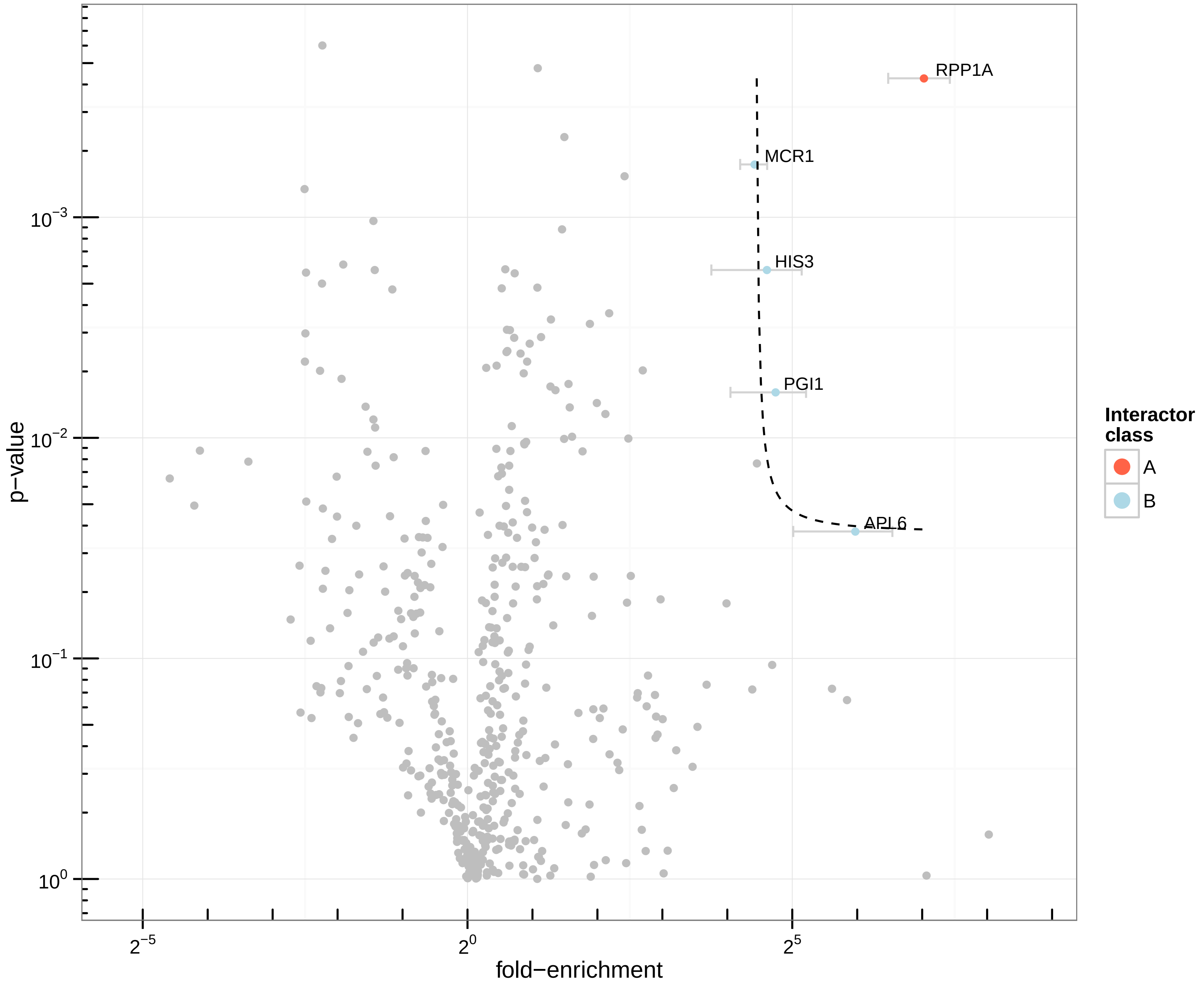

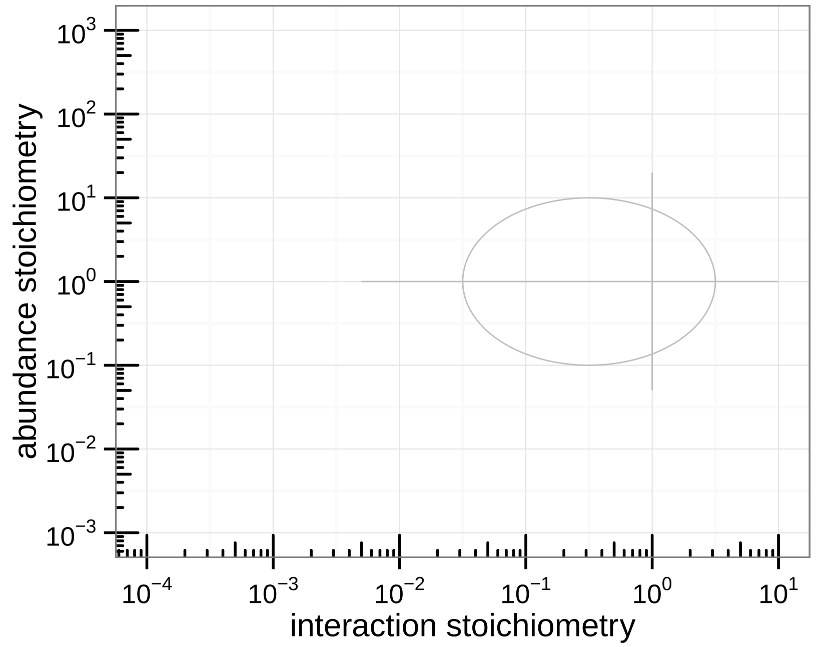

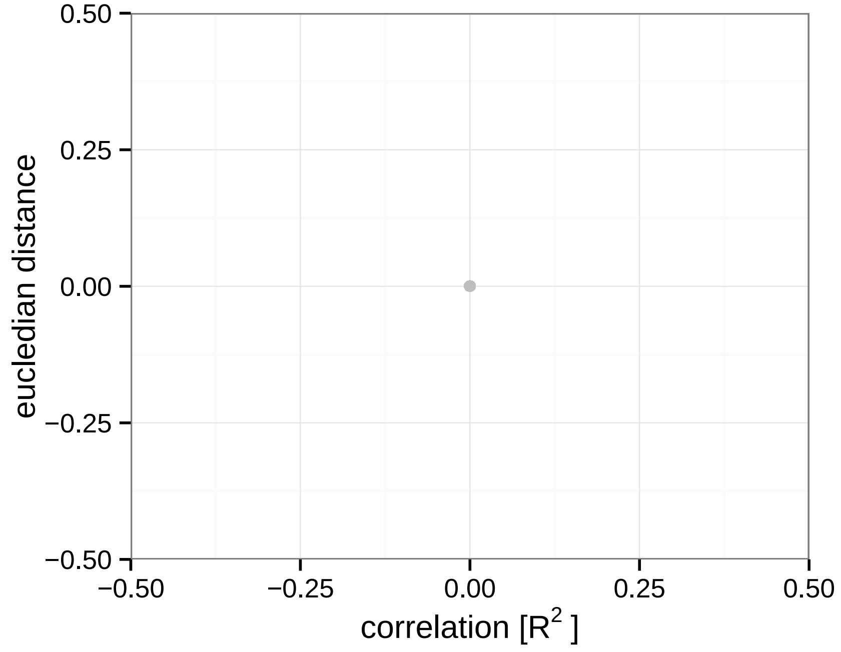

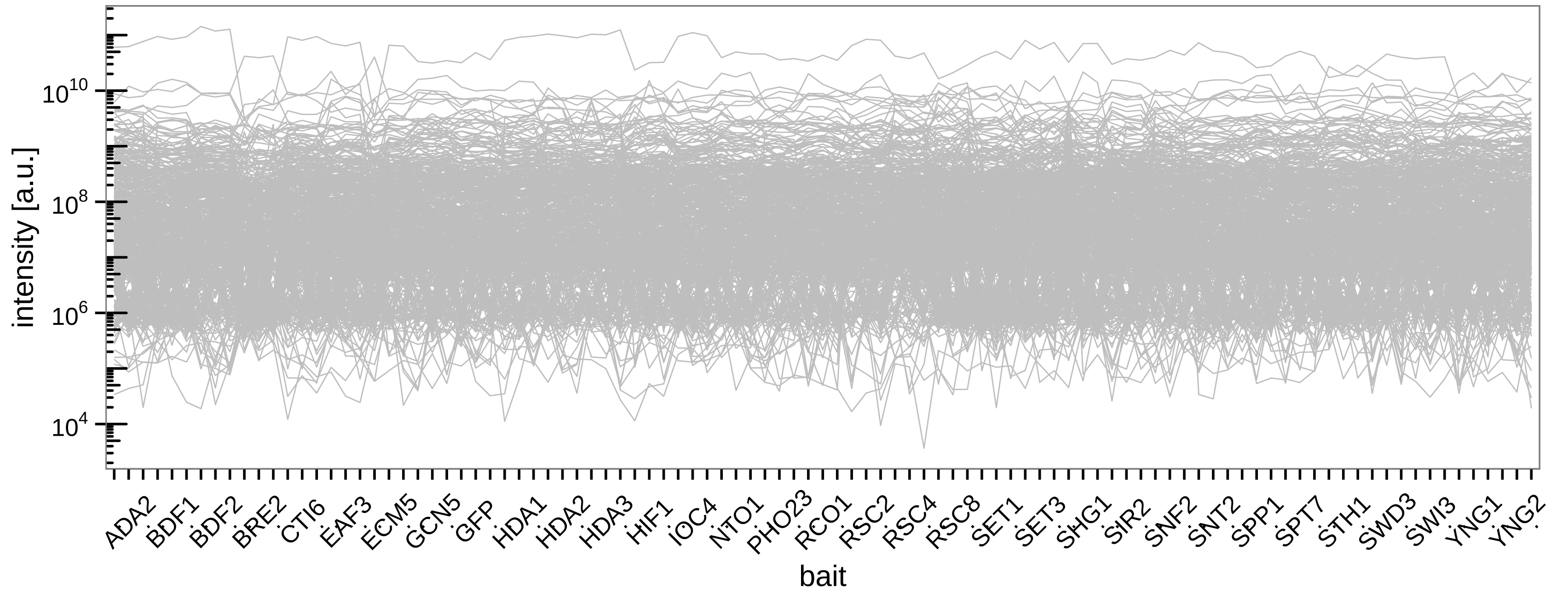

	RPP1A
significance	7
interactor class	A

**bait**	YNG2
**experiment**	27 minutes

**interactors**	15
**recovery**	69 %

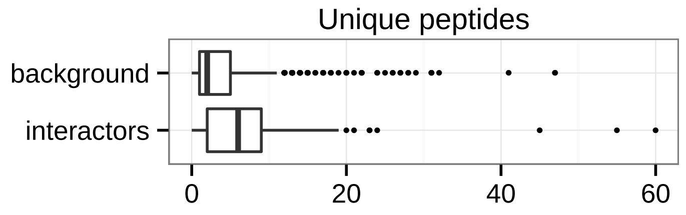

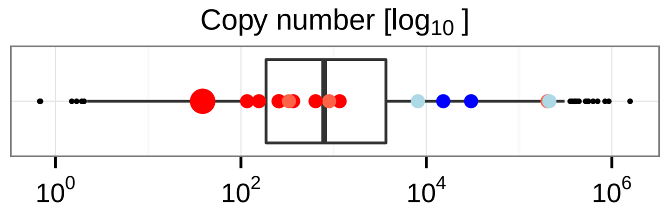

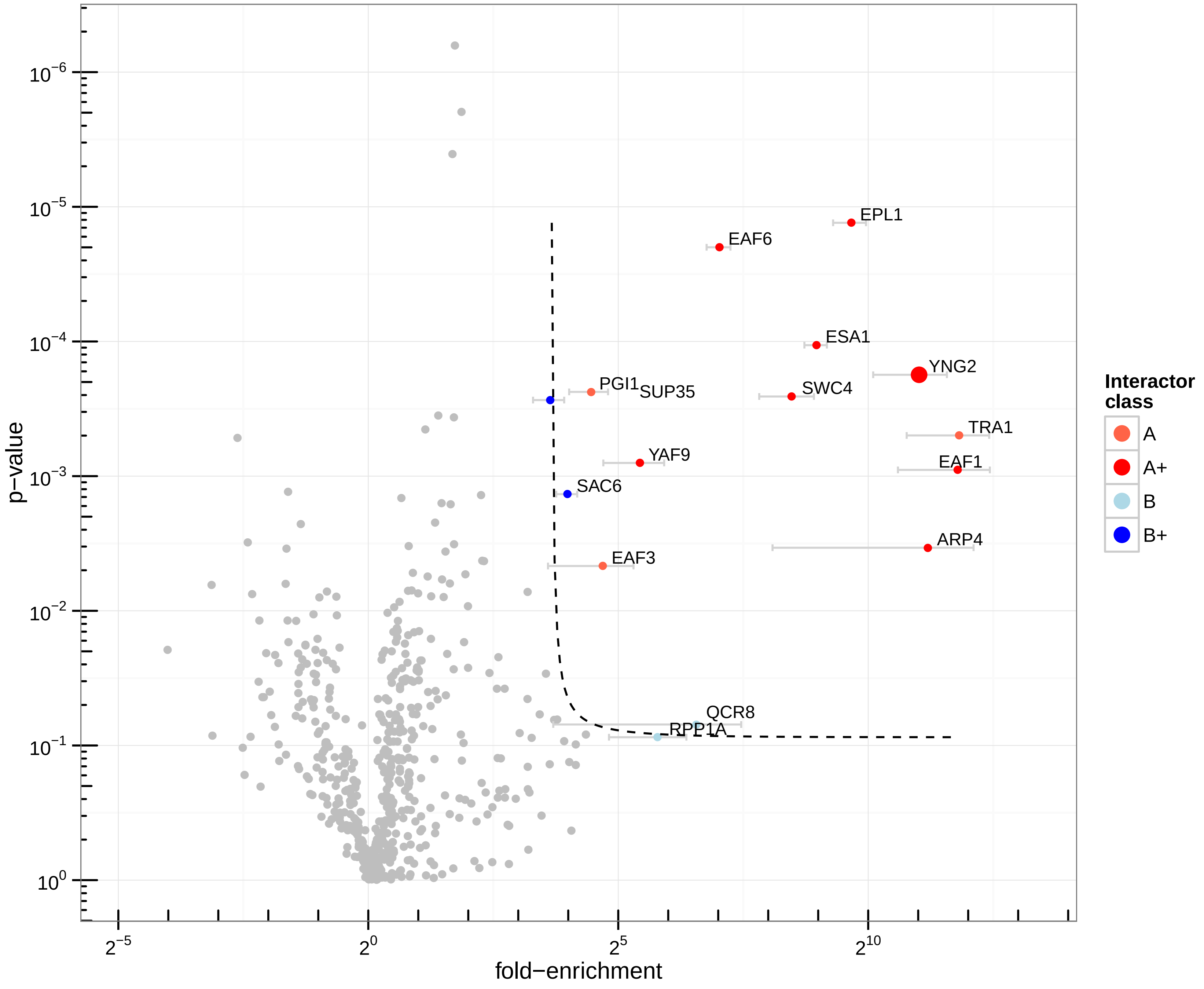

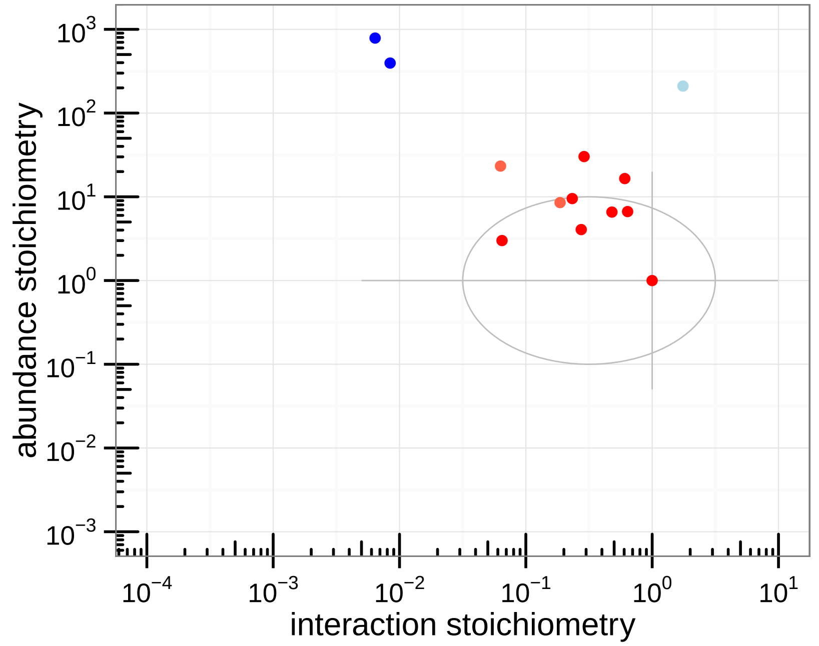

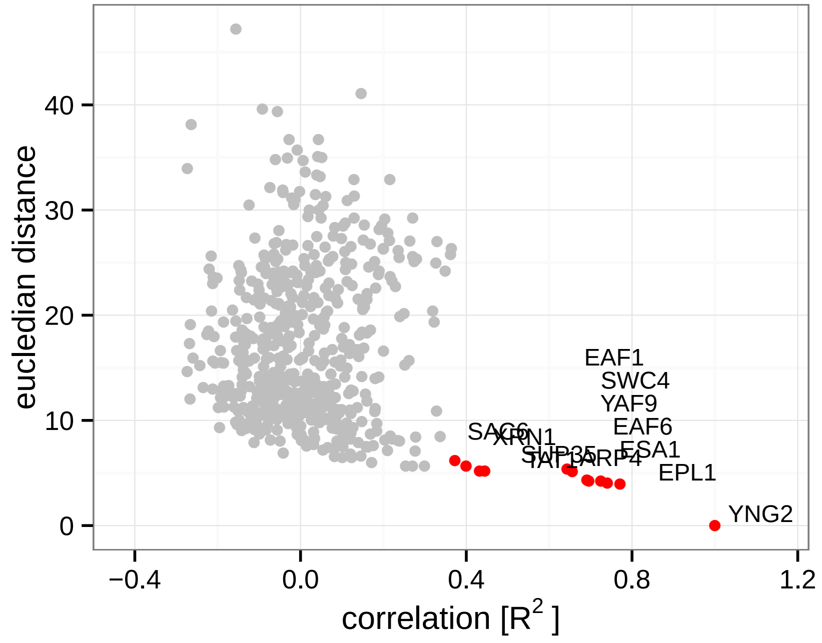

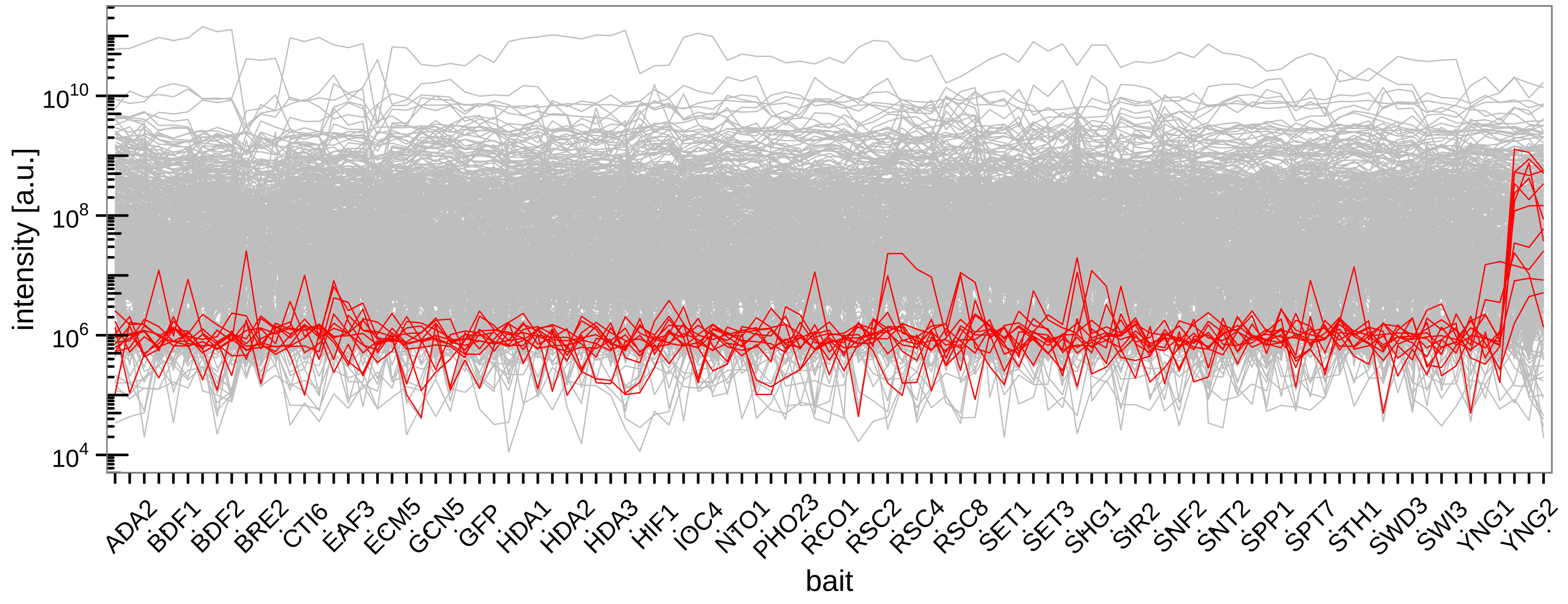

	ARP4	EAF1	EAF3	EAF6	EPL1	ESA1	PGI1	SAC6	SUP35	SWC4	TRA1	YAF9	YNG2
significance	10.0	11.0	4.4	7.6	9.9	8.9	5.0	4.1	4.4	8.3	11.0	5.4	11.0
interactor class	A+	A+	A	A+	A+	A+	A	B+	B+	A+	A	A+	A+
