# Supplementary material for: A Double-Barrel Liquid Chromatography-Tandem Mass Spectrometry (LC-MS/MS) System to Quantify 96 Interactomes per Day
Source: Mol Cell Proteomics. 2015 Apr 17;14(7):2030–41. doi: 10.1074/mcp.O115.049460 (PMC4587330; doi:10.1074/mcp.O115.049460)

Raw file	Scan	Method	Score	m/z	Gene names
20140827_EXQ00_FaHo_SA_ADA2_01	2984	FTMS; HCD	155.42	505.25	RPL17A;RPL17B

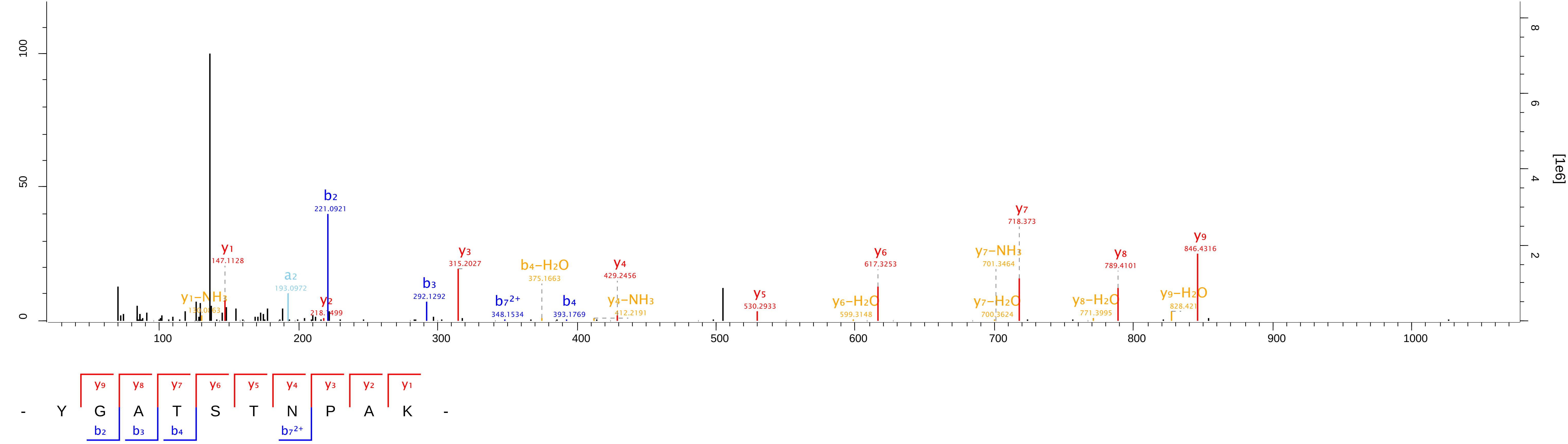

Raw file	Scan	Method	Score	m/z	Gene names
20140827_EXQ00_FaHo_SA_ADA2_0	4010	FTMS; HCD	136.11	415.74	TY1B-LR3;TY1A-PL;TY1A-LR2;TY1A-ER1;TY1A-DR6;TY1B-LR4;TY1B-LR2;TY1B-PL;TY1B-ER1;TY1B-PR3;TY1A-PR1;TY1A-A;TY1A-DR4;TY1B-H;TY1B-GR2;TY1B-MR2;TY1B-ER2;TY1B-OR;TY1B-BR;TY1B-DR1;TY1B-NL2;TY1B-

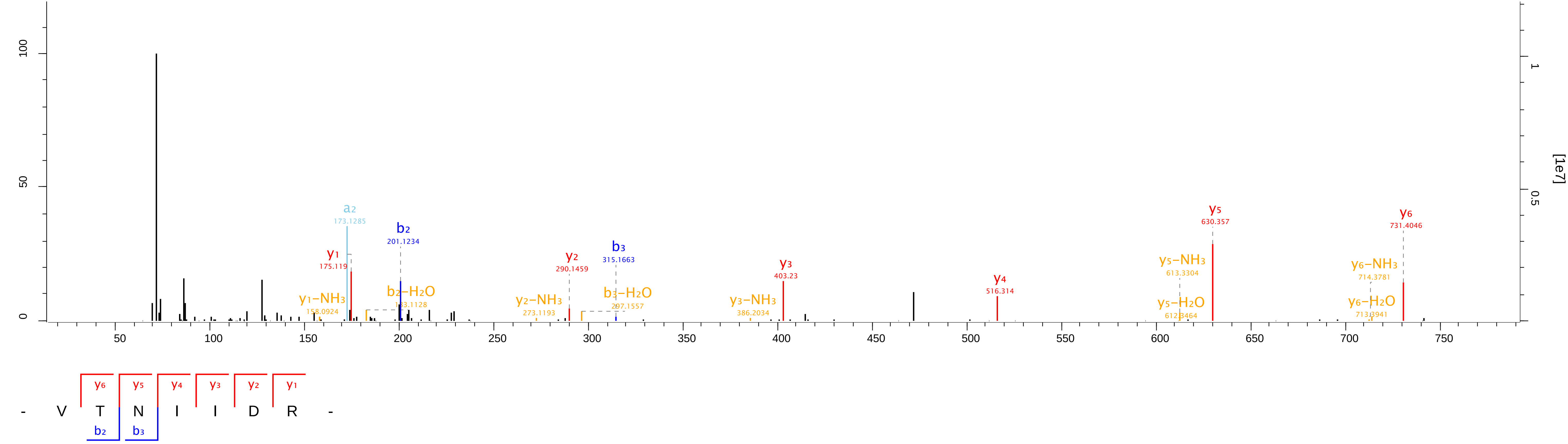

Raw file	Scan	Method	Score	m/z	Gene names
20140827_EXQ00_FaHo_SA_ADA2_02	6574	FTMS; HCD	86.01	656.33	AHC2

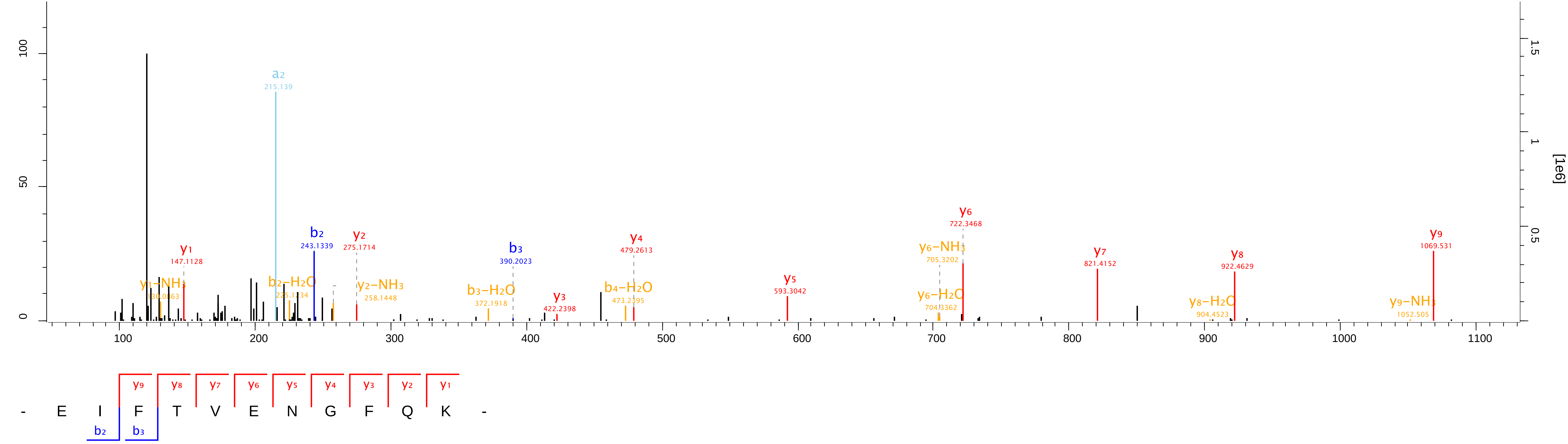

Raw file	Scan	Method	Score	m/z	Gene names
20140827_EXQ00_FaHo_SA_ADA2_02	7158	FTMS; HCD	156.96	885.94	UBP8

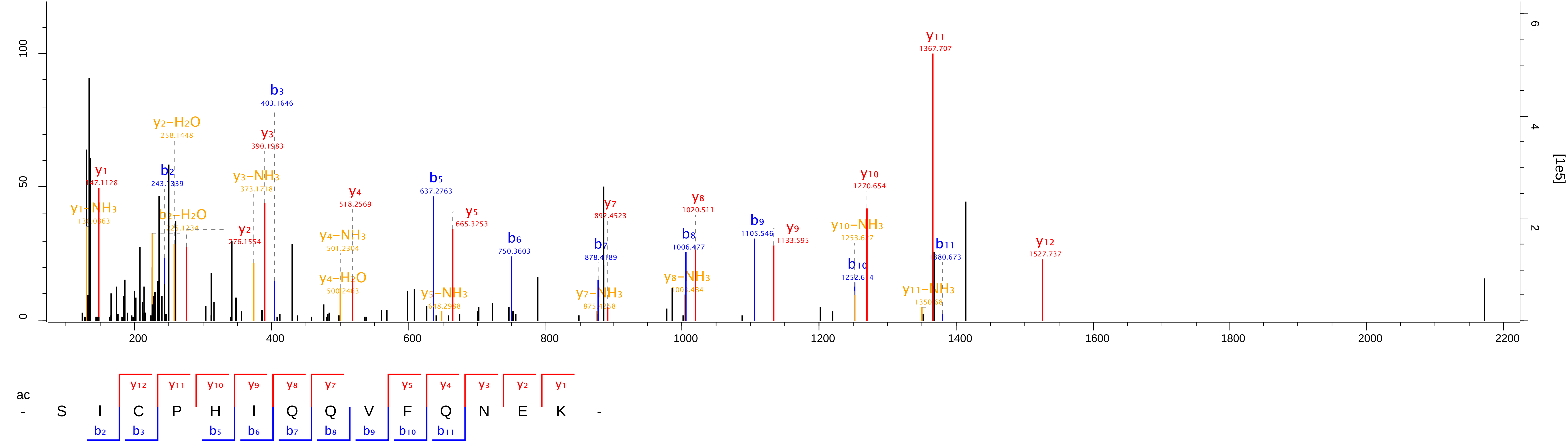

Raw file	Scan	Method	Score	m/z	Gene names
20140827_EXQ00_FaHo_SA_BDF1_01	3311	FTMS; HCD	180.97	662.32	RPL7A;RPL7B

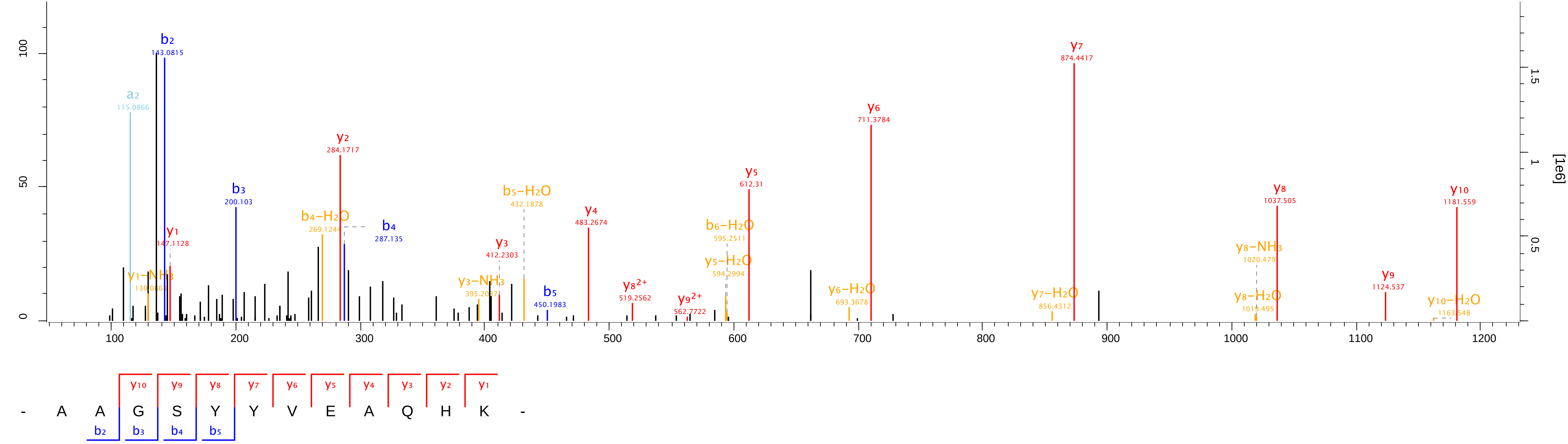

Raw file	Scan	Method	Score	m/z	Gene names
20140827_EXQ00_FaHo_SA_BDF1_01	4316	FTMS; HCD	27.22	640.31	TY1B-LR3;TY1B-OL;TY1B-LR4;TY1B-LR2;TY1B-PL;TY1B-ER1;TY1B-PR3;TY1B-GR2;TY1B-MR2;TY1B-OR;TY1B-DR1;TY1B-A

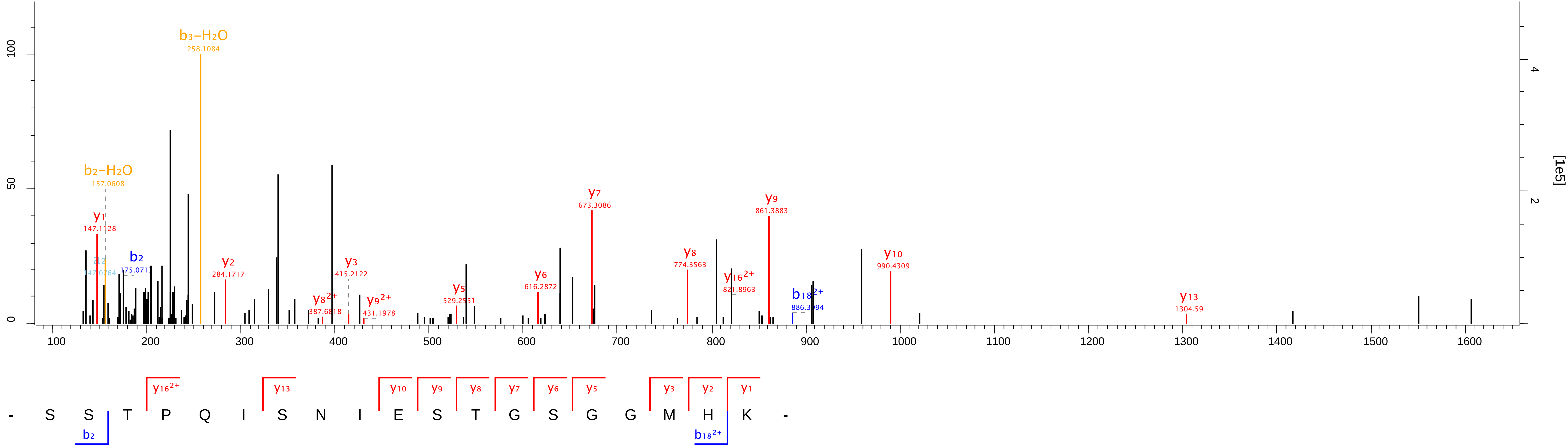

Raw file	Scan	Method	Score	m/z	Gene names
20140827_EXQ00_FaHo_SA_BDF1_01	5409	FTMS; HCD	119.21	443.77	RPL36A;RPL36B

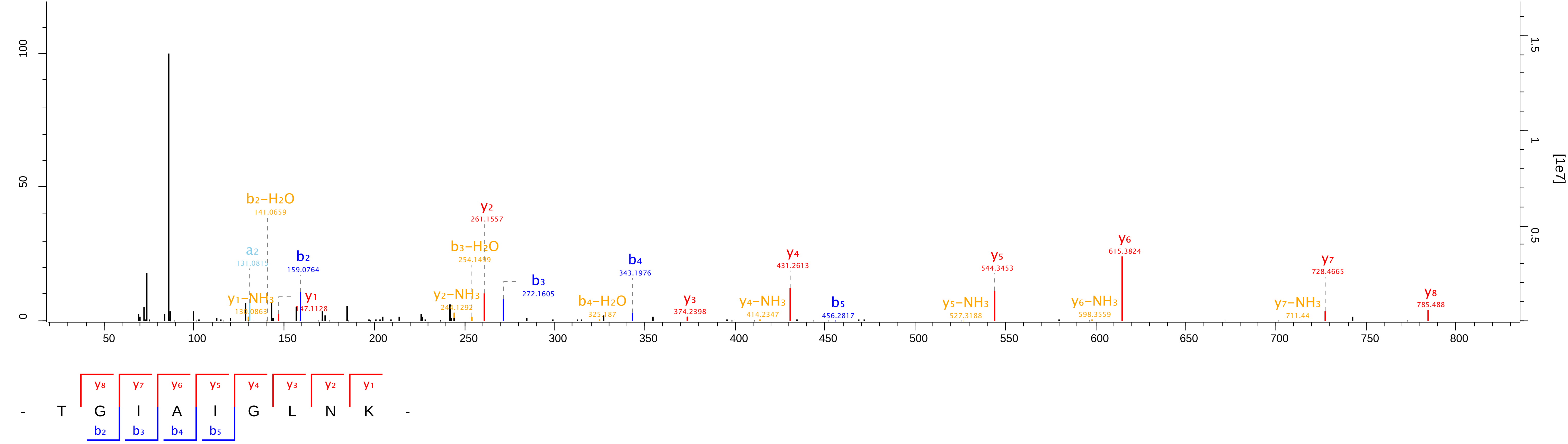

Raw file	Scan	Method	Score	m/z	Gene names
20140827_EXQ00_FaHo_SA_BDF1_01	7042	FTMS; HCD	67.25	614.35	FPR4

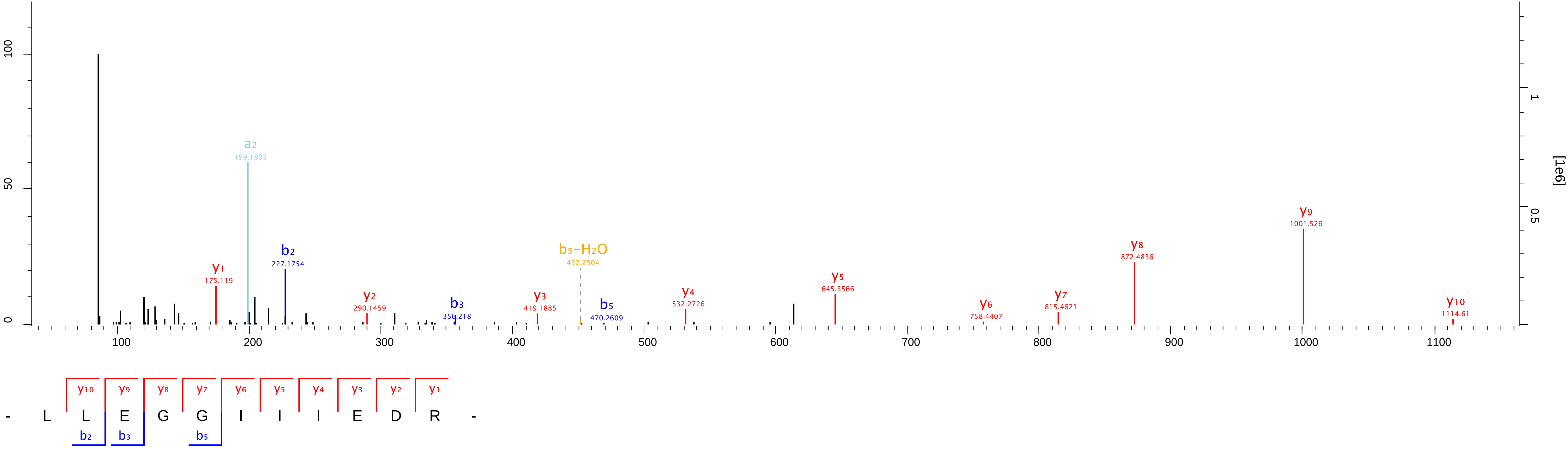

Raw file	Scan	Method	Score	m/z	Gene names
20140827_EXQ00_FaHo_SA_BDF1_01	7830	FTMS; HCD	72.9	731.38	TOP2

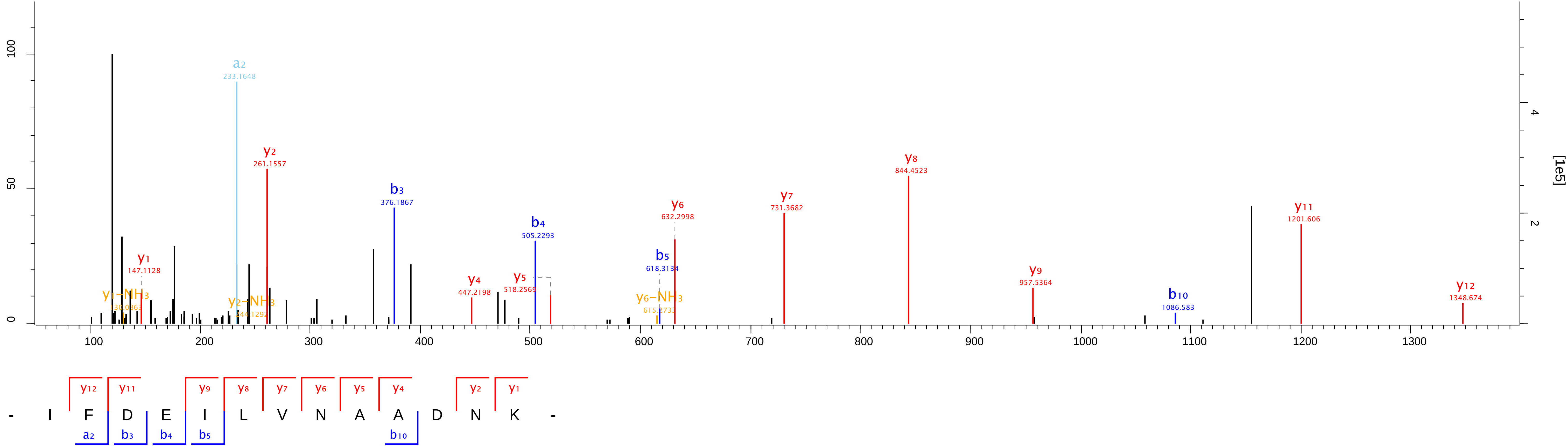

Raw file	Scan	Method	Score	m/z	Gene names
20140827_EXQ00_FaHo_SA_BDF1_01	8367	FTMS; HCD	93.23	900.48	FPR4;FPR3

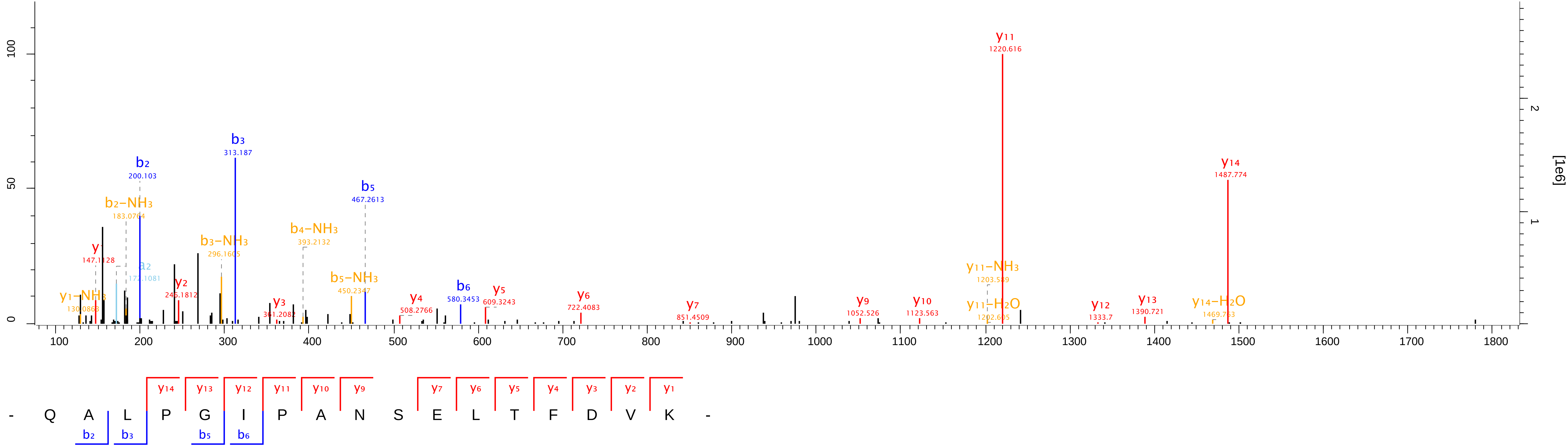

Raw file	Scan	Method	Score	m/z	Gene names
20140827_EXQ00_FaHo_SA_BDF1_02	3249	FTMS; HCD	105.98	494.76	RPL7B

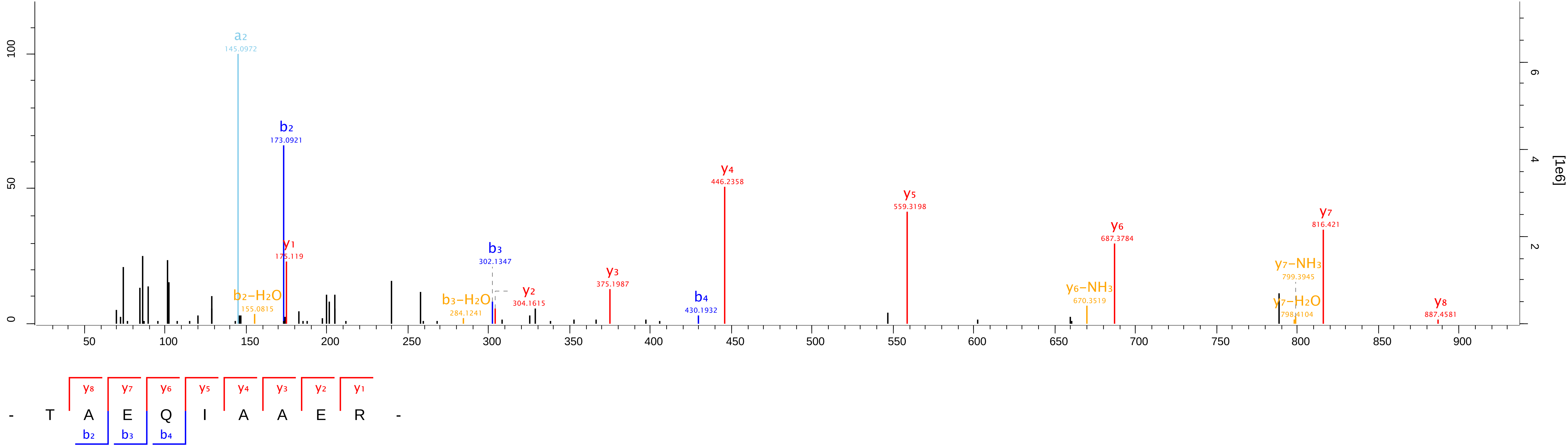

Raw file	Scan	Method	Score	m/z	Gene names
20140827_EXQ00_FaHo_SA_BDF1_02	3922	FTMS; HCD	66.02	663.31	TY1B-LR4

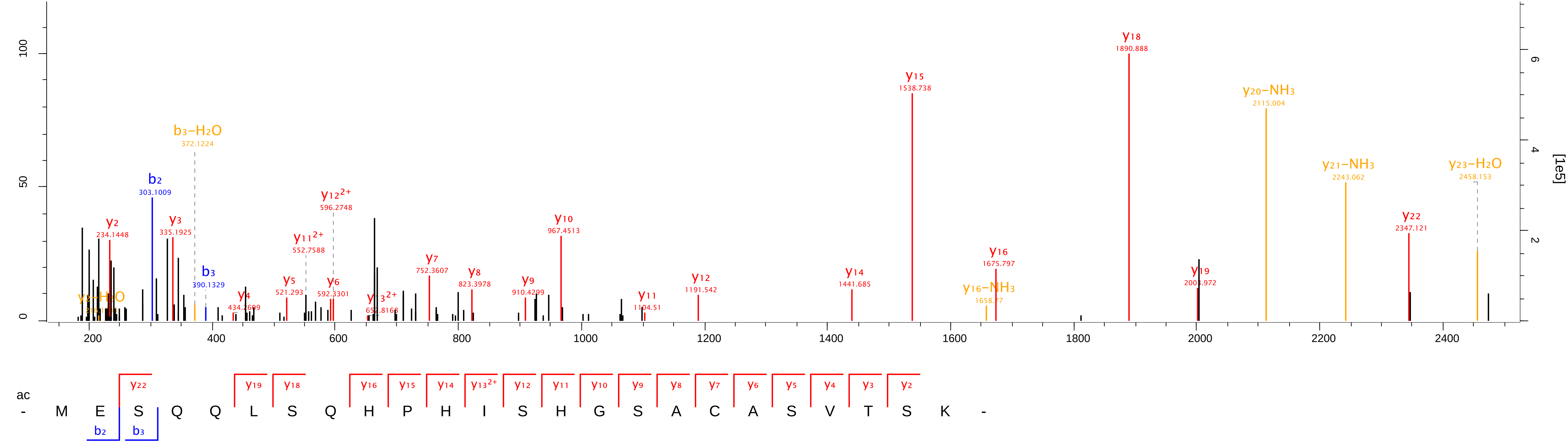

Raw file	Scan	Method	Score	m/z	Gene names
20140827_EXQ00_FaHo_SA_BDF1_02	4559	FTMS; HCD	188.48	748.91	RPL17A;RPL17B

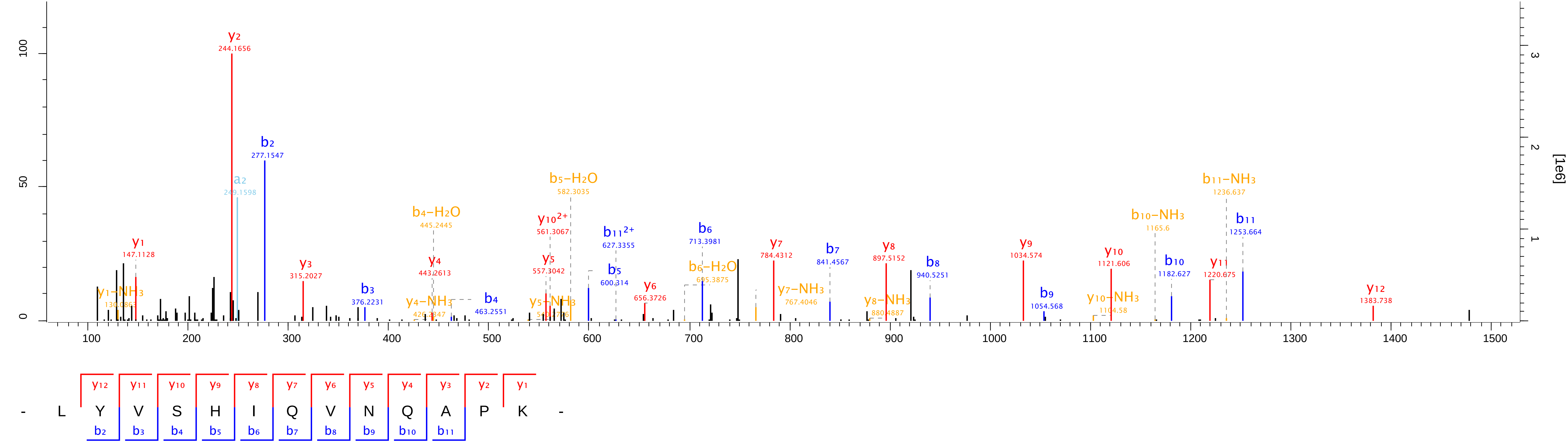

Raw file	Scan	Method	Score	m/z	Gene names
20140827_EXQ00_FaHo_SA_BDF1_03	2631	FTMS; HCD	128.86	366.7	RPS0A

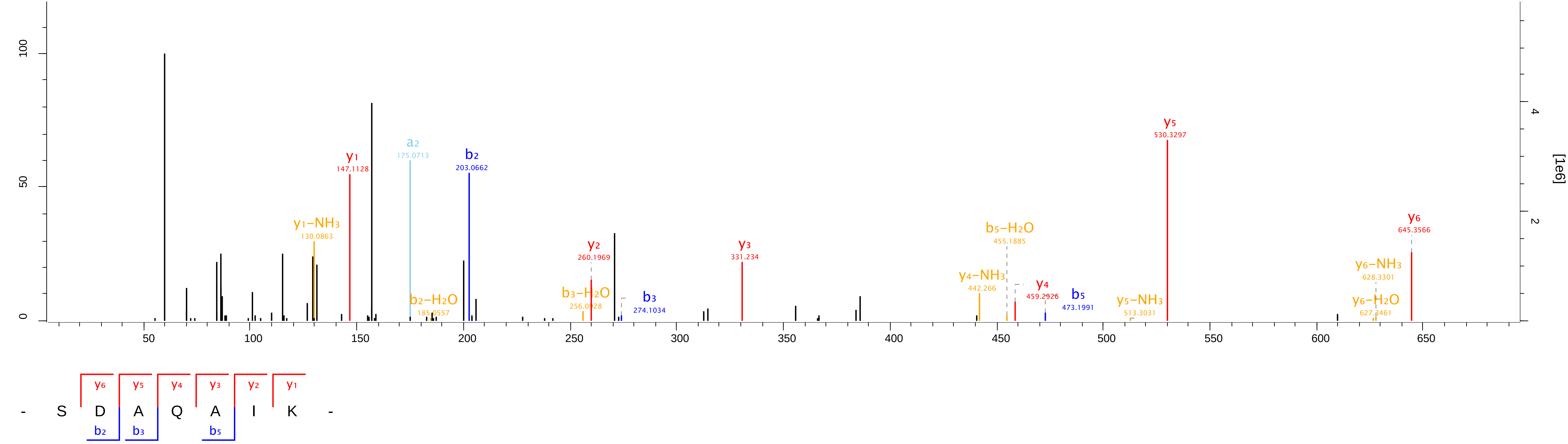

Raw file	Scan	Method	Score	m/z	Gene names
20140827_EXQ00_FaHo_SA_BDF1_03	3754	FTMS; HCD	144.65	505.78	RPL4B;RPL4A

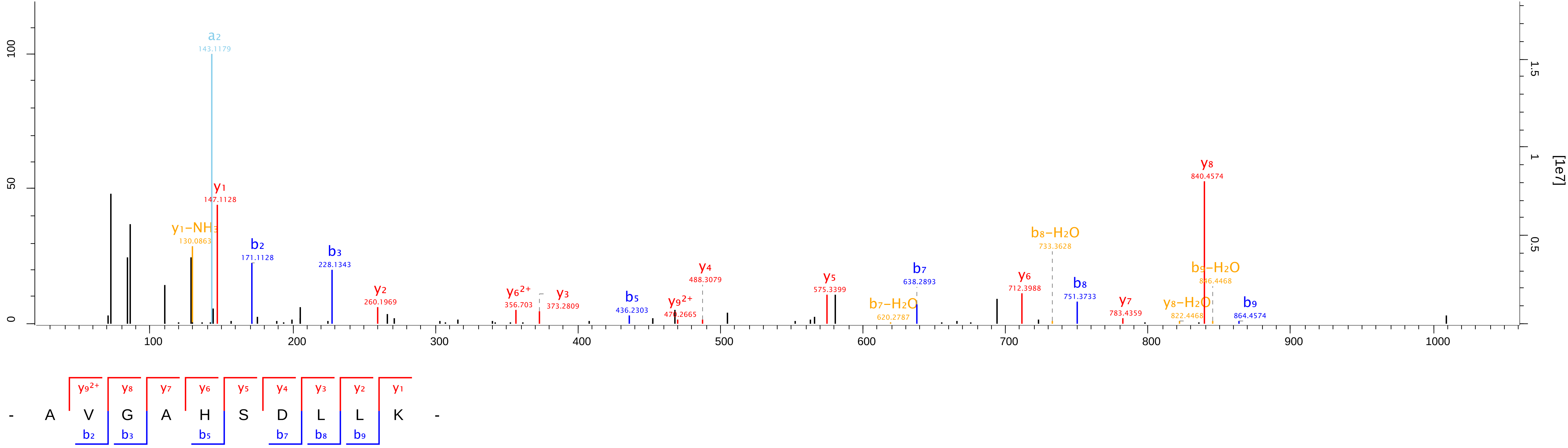

Raw file	Scan	Method	Score	m/z	Gene names
20140827_EXQ00_FaHo_SA_BDF1_03	5978	FTMS; HCD	131.06	673.84	RPL36A;RPL36B

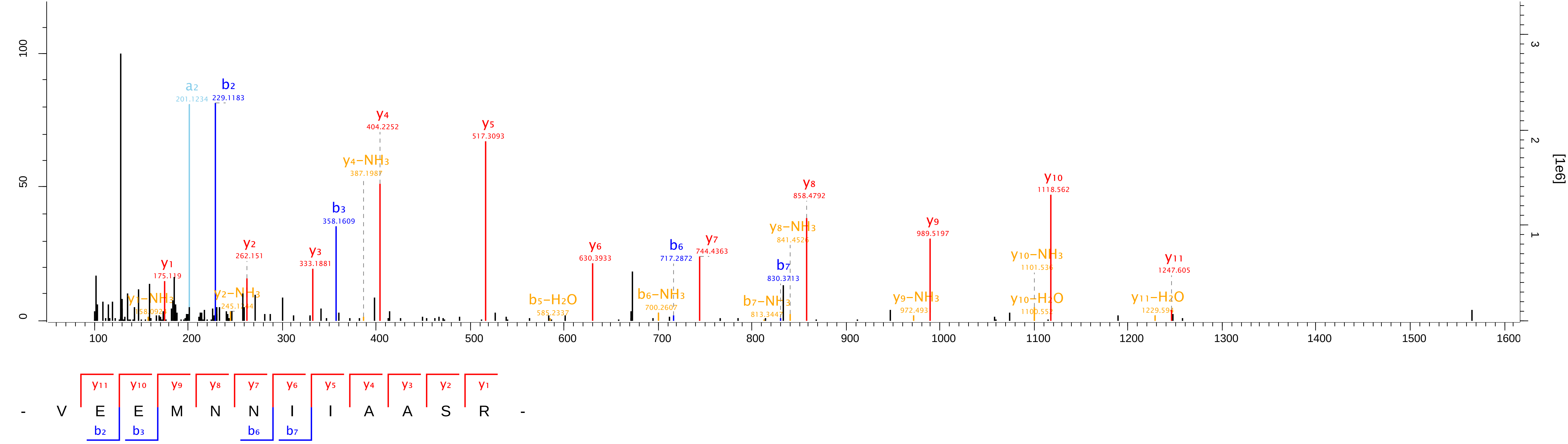

Raw file	Scan	Method	Score	m/z	Gene names
20140827_EXQ00_FaHo_SA_BDF1_03	6058	FTMS; HCD	110.08	661.35	ERV29

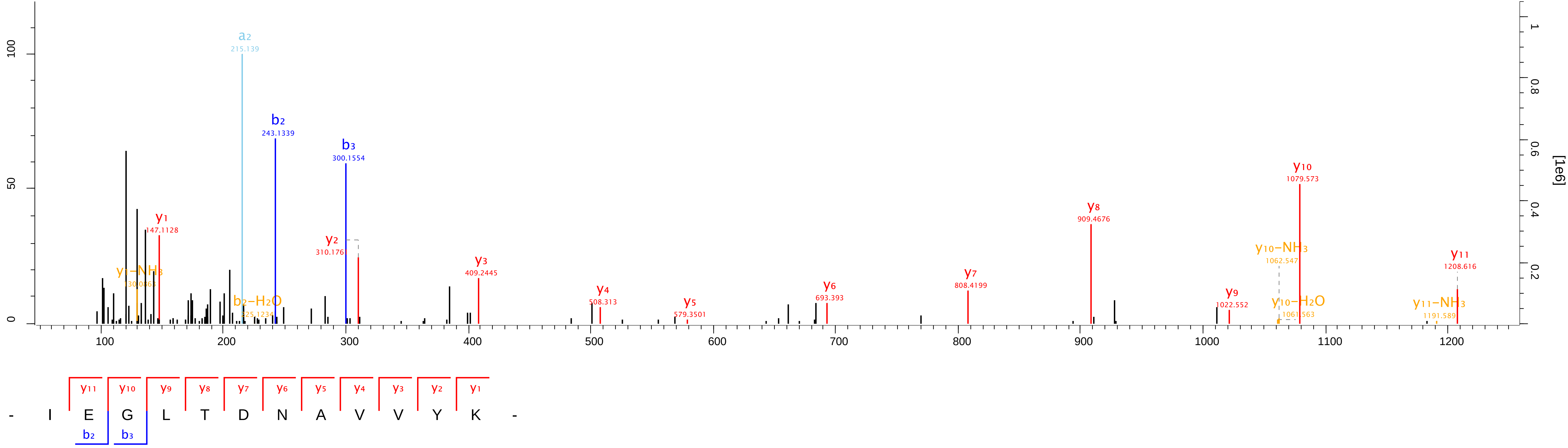

Raw file	Scan	Method	Score	m/z	Gene names
20140827_EXQ00_FaHo_SA_BDF1_03	7585	FTMS; HCD	84.75	766.42	RPT6

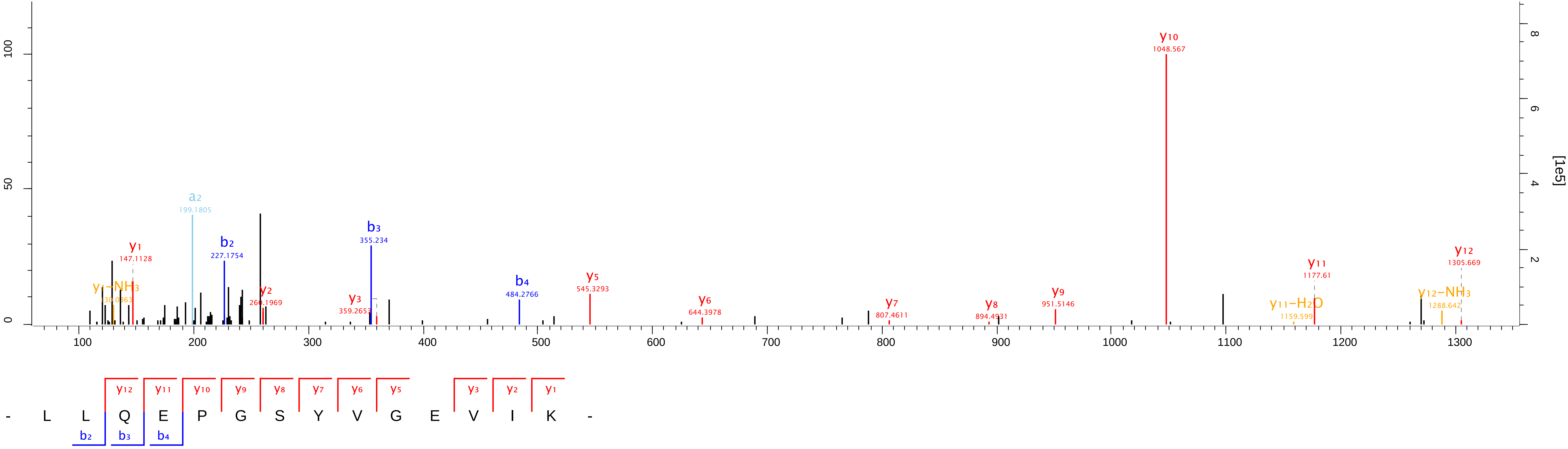

Raw file	Scan	Method	Score	m/z	Gene names
20140827_EXQ00_FaHo_SA_BDF1_03	8286	FTMS; HCD	100.11	602.99	RPL9A;RPL9B

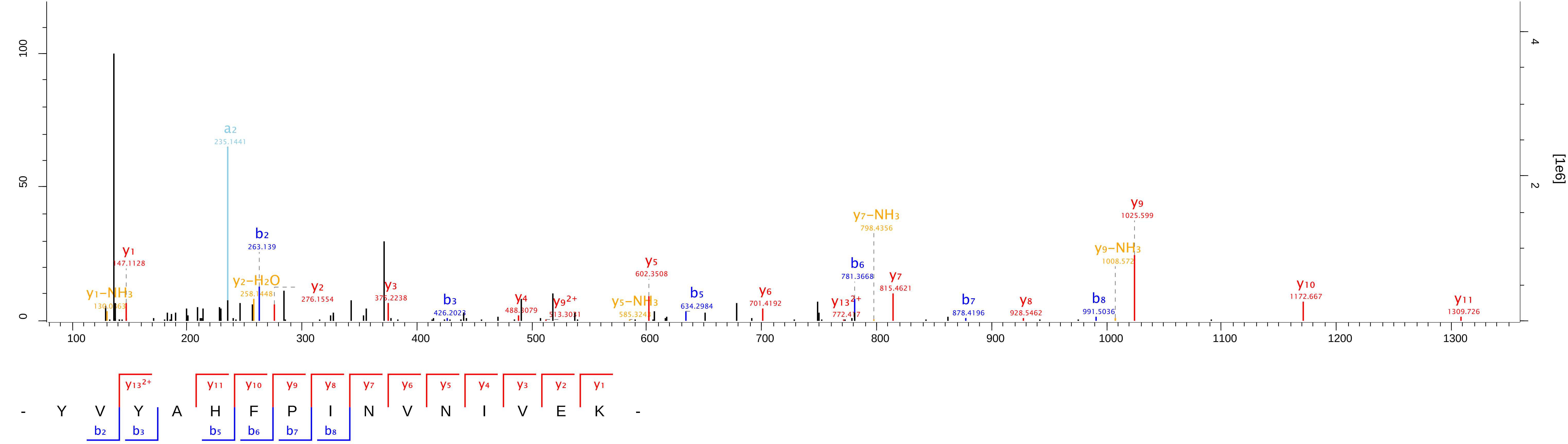

Raw file	Scan	Method	Score	m/z	Gene names
20140827_EXQ00_FaHo_SA_BDF1_03	8802	FTMS; HCD	64.59	871.48	RPL4B;RPL4A

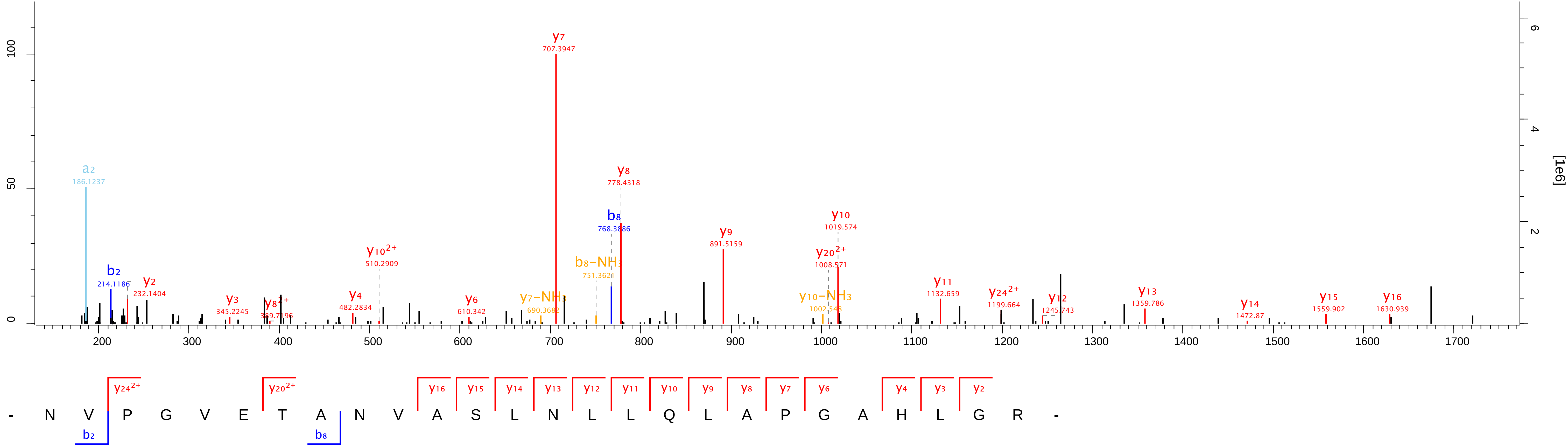

Raw file	Scan	Method	Score	m/z	Gene names
20140827_EXQ00_FaHo_SA_BDF1_03	8929	FTMS; HCD	97.08	1138.1	RPL21A;RPL21B

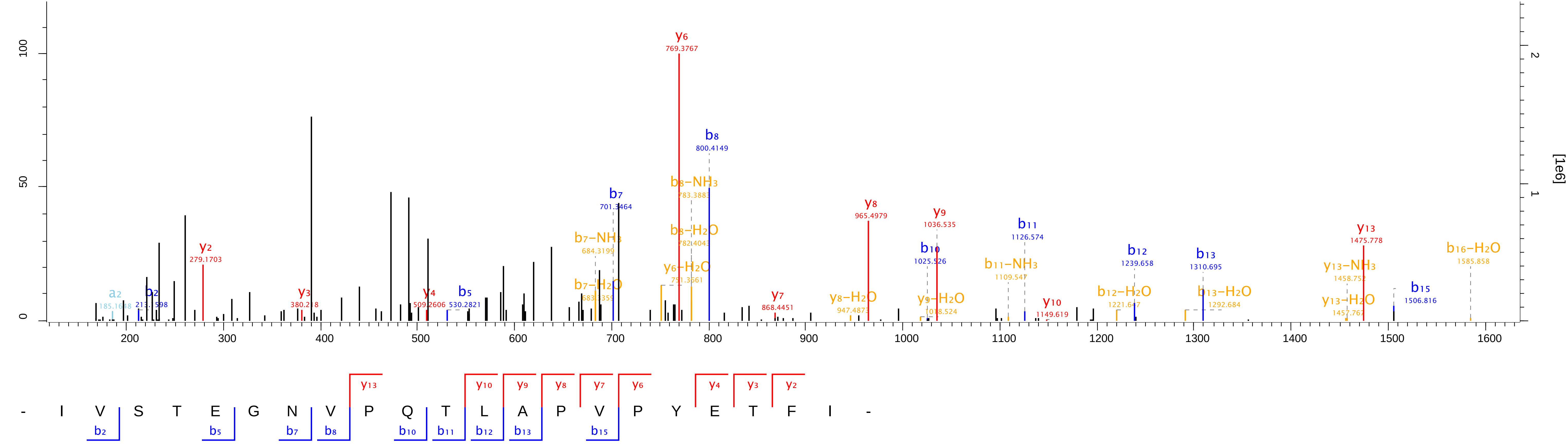

Raw file	Scan	Method	Score	m/z	Gene names
20140827_EXQ00_FaHo_SA_BDF2_01	3821	FTMS; HCD	94.26	408.22	RPS14A

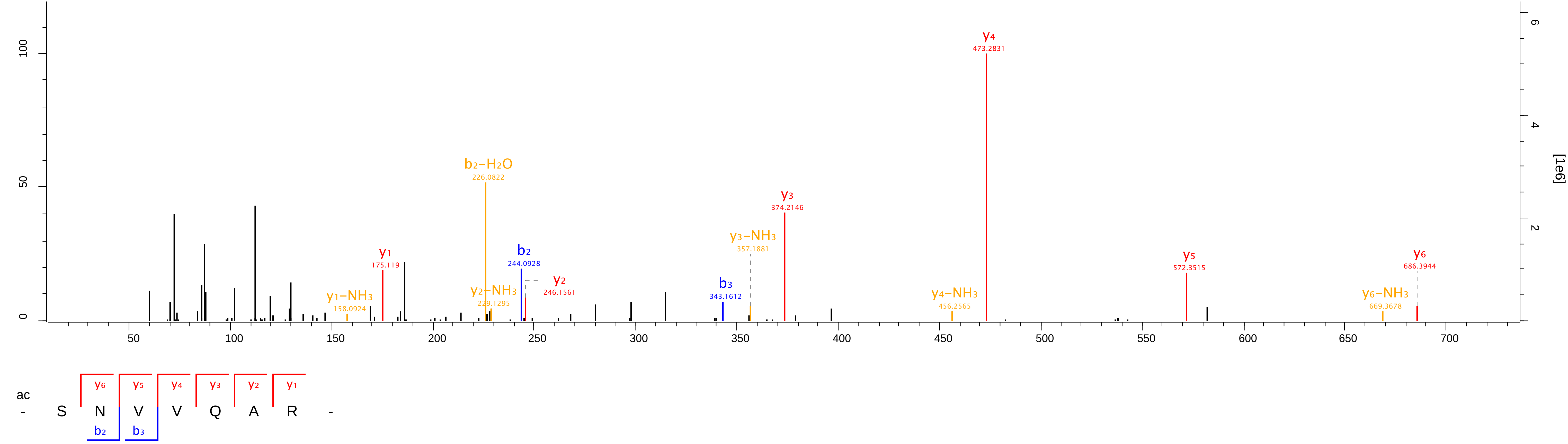

Raw file	Scan	Method	Score	m/z	Gene names
20140827_EXQ00_FaHo_SA_BDF2_01	4932	FTMS; HCD	80.74	572.97	RPL4A

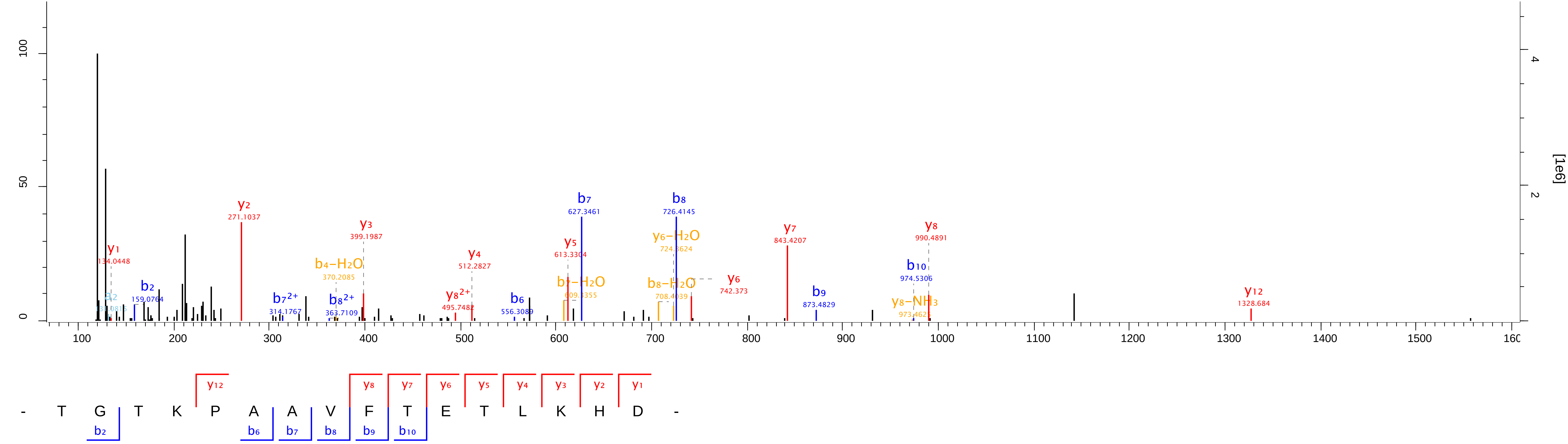

Raw file	Scan	Method	Score	m/z	Gene names
20140827_EXQ00_FaHo_SA_BDF2_01	5716	FTMS; HCD	80.74	867.43	UBP3

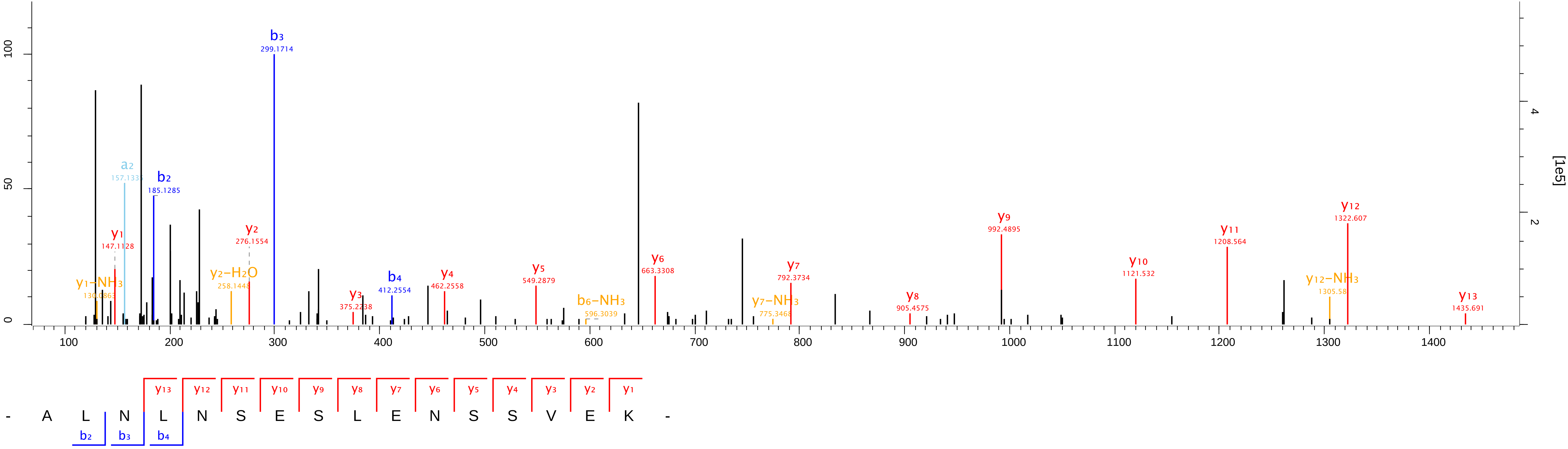

Raw file	Scan	Method	Score	m/z	Gene names
20140827_EXQ00_FaHo_SA_BDF2_01	7228	FTMS; HCD	64.1	726.39	RLP24

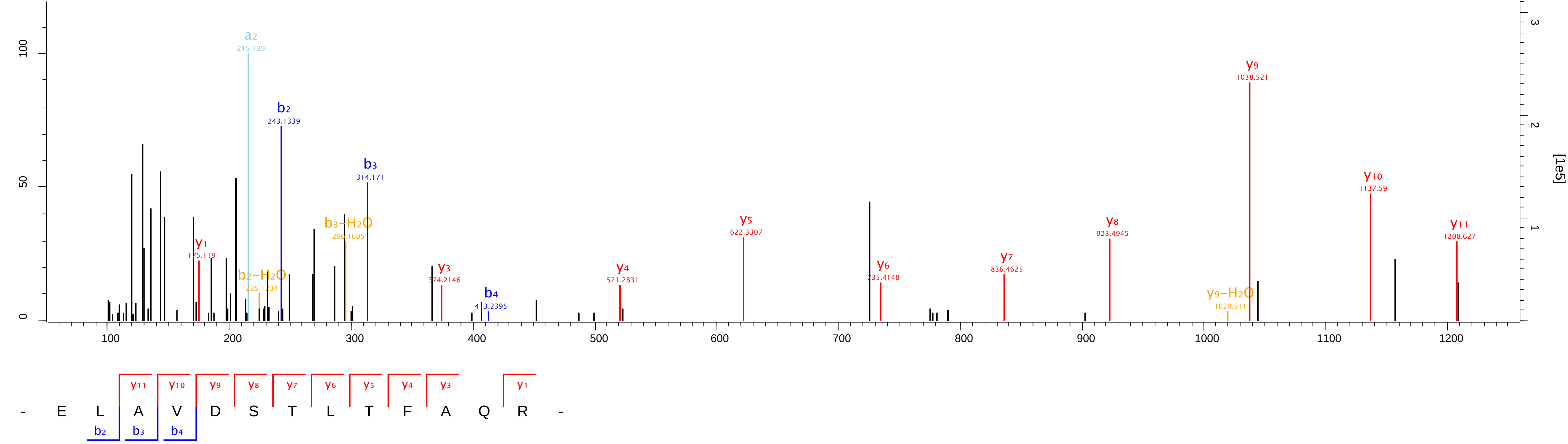

Raw file	Scan	Method	Score	m/z	Gene names
20140827_EXQ00_FaHo_SA_BDF2_02	3425	FTMS; HCD	113.7	386.72	PDR15

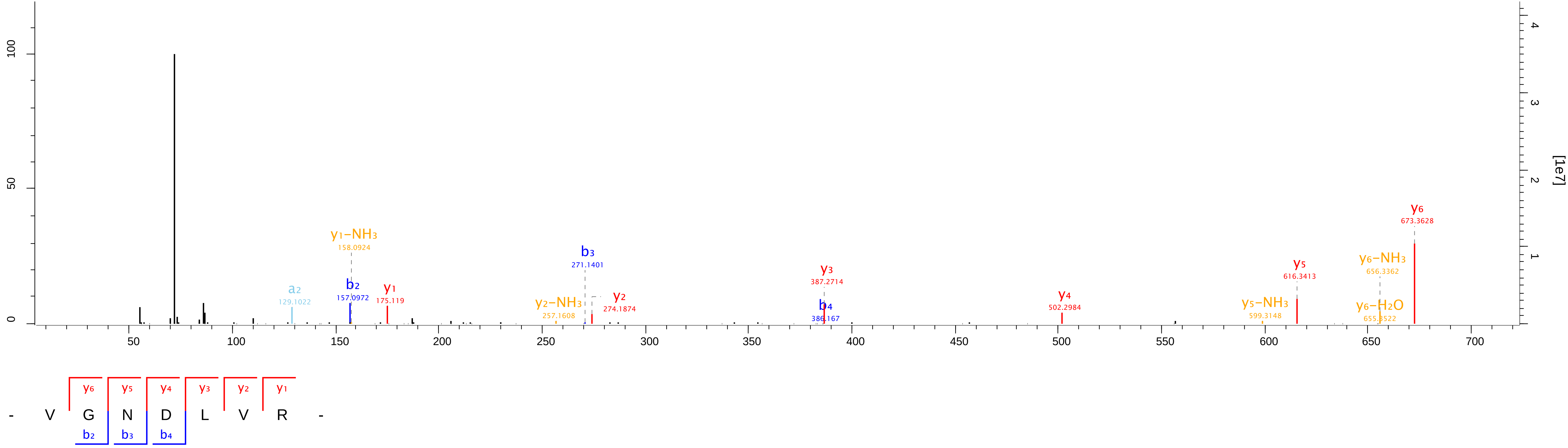

Raw file	Scan	Method	Score	m/z	Gene names
20140827_EXQ00_FaHo_SA_BDF2_02	4951	FTMS; HCD	143.96	650.83	RPL17A

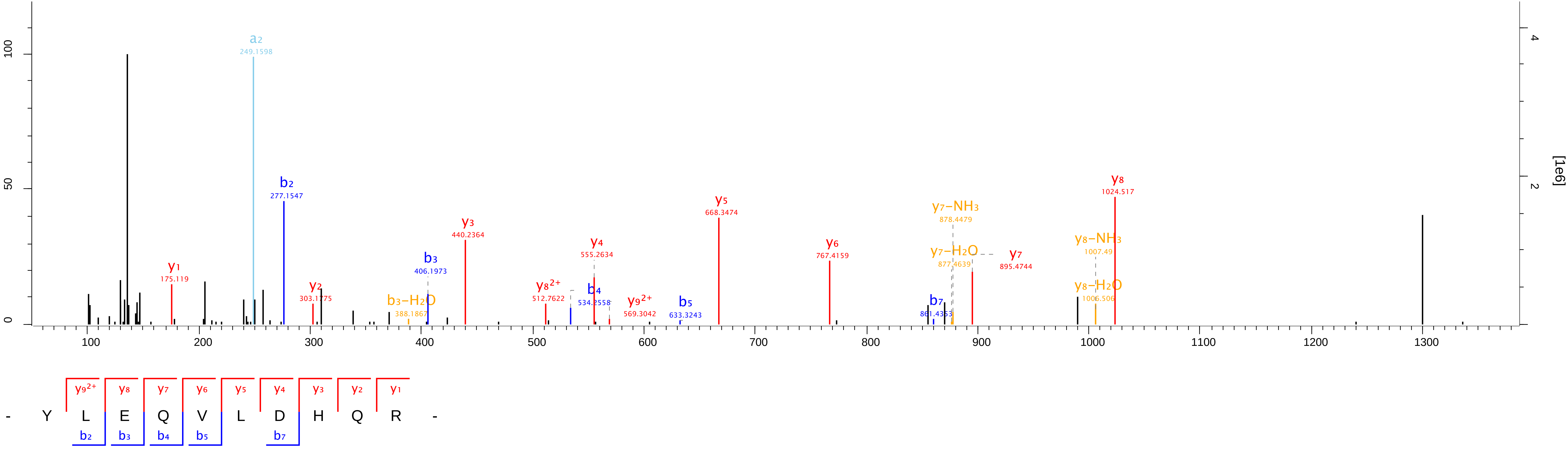

Raw file	Scan	Method	Score	m/z	Gene names
20140827_EXQ00_FaHo_SA_BDF2_02	5445	FTMS; HCD	67.1	538.61	MDS3

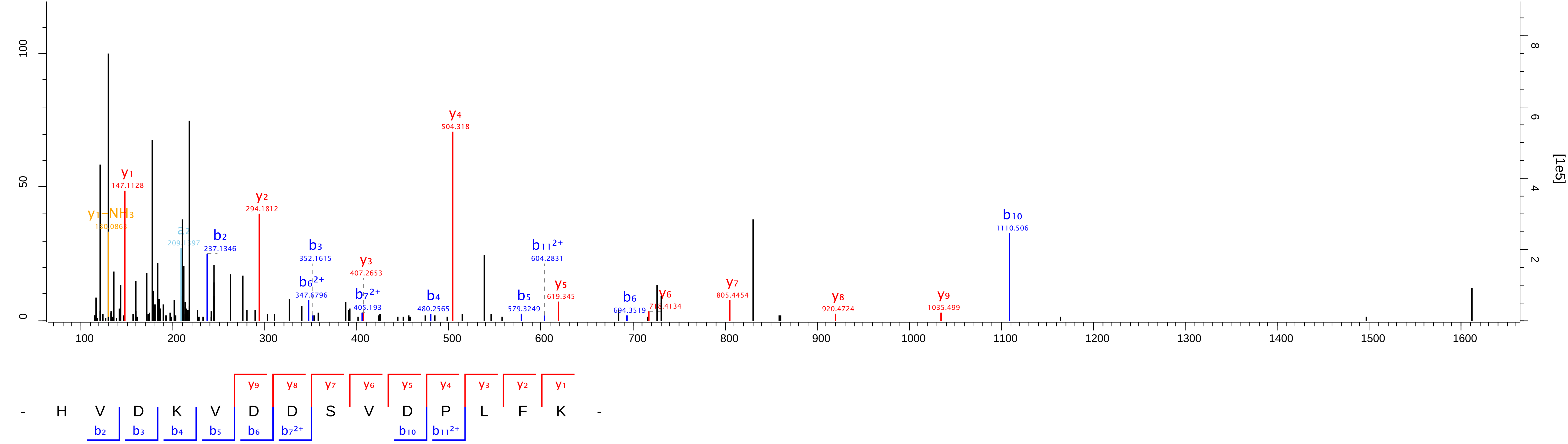

Raw file	Scan	Method	Score	m/z	Gene names
20140827_EXQ00_FaHo_SA_BDF2_02	7184	FTMS; HCD	82.42	624.35	ATP5

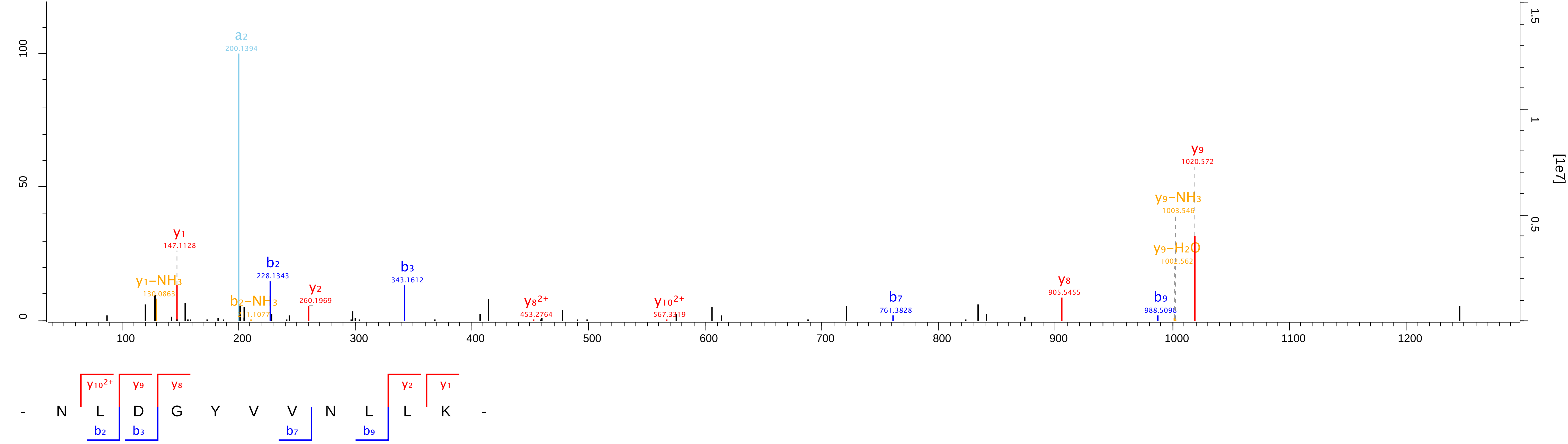

Raw file	Scan	Method	Score	m/z	Gene names
20140827_EXQ00_FaHo_SA_BDF2_02	7374	FTMS; HCD	42.63	731.04	ATP7

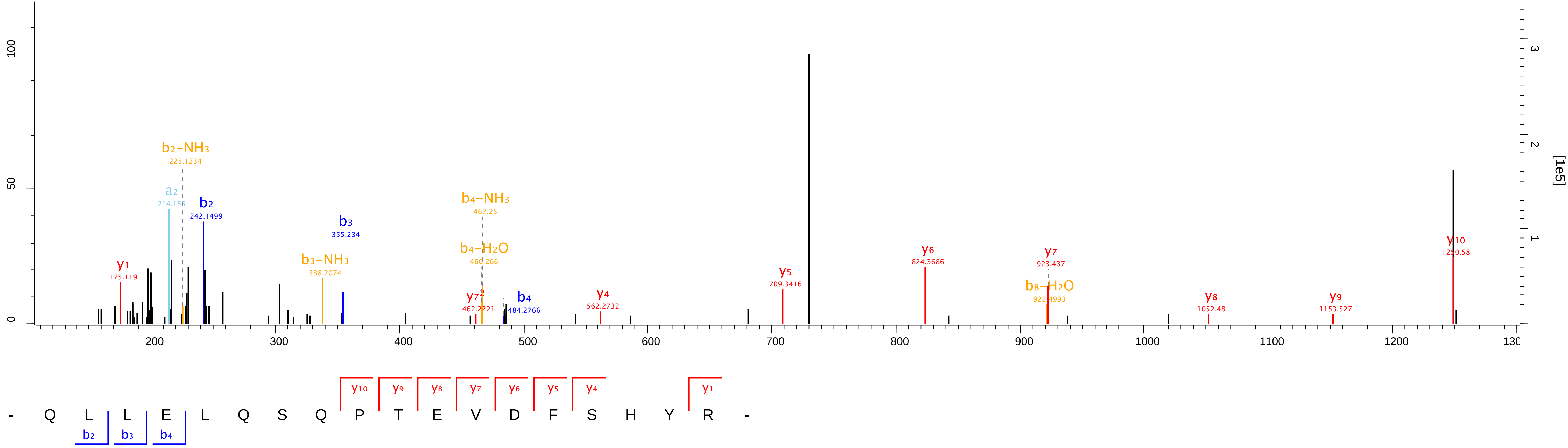

Raw file	Scan	Method	Score	m/z	Gene names
20140827_EXQ00_FaHo_SA_BDF2_03	4159	FTMS; HCD	310.49	1209.06	TY1B-LR3;TY1A-PL;TY1A-LR2;TY1A-ER1;TY1A-DR6;TY1B-OL;TY1B-LR4;TY1B-LR2;TY1B-PL;TY1B-ER1;TY1B-PR3;TY1A-PR1;TY1A-A;TY1A-DR4

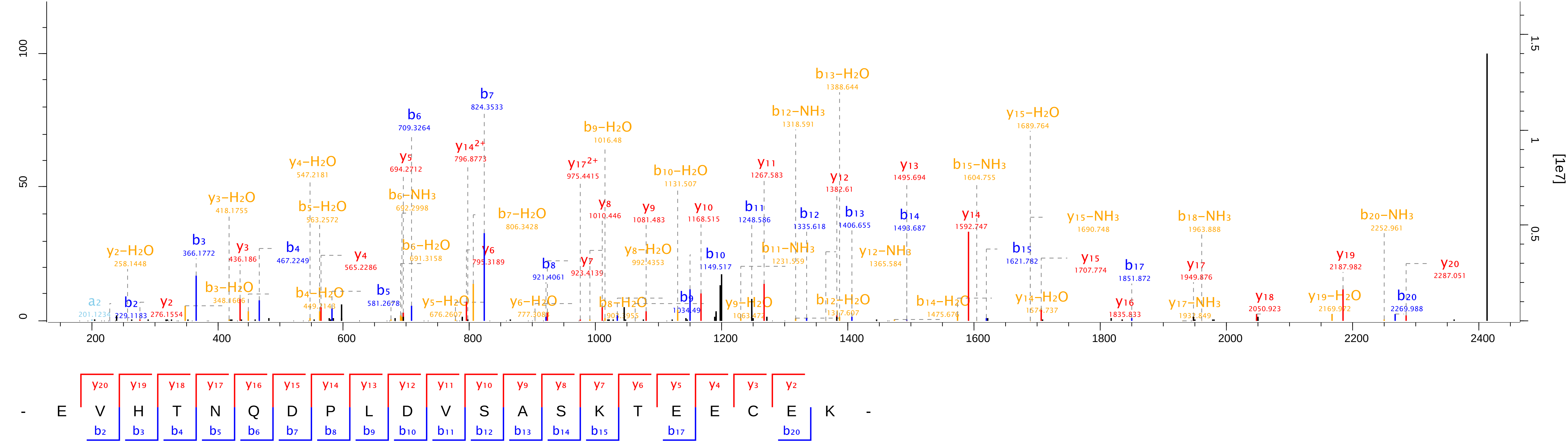

Raw file	Scan	Method	Score	m/z	Gene names
20140827_EXQ00_FaHo_SA_BDF2_03	5772	FTMS; HCD	99.14	429.75	RPL21A;RPL21B

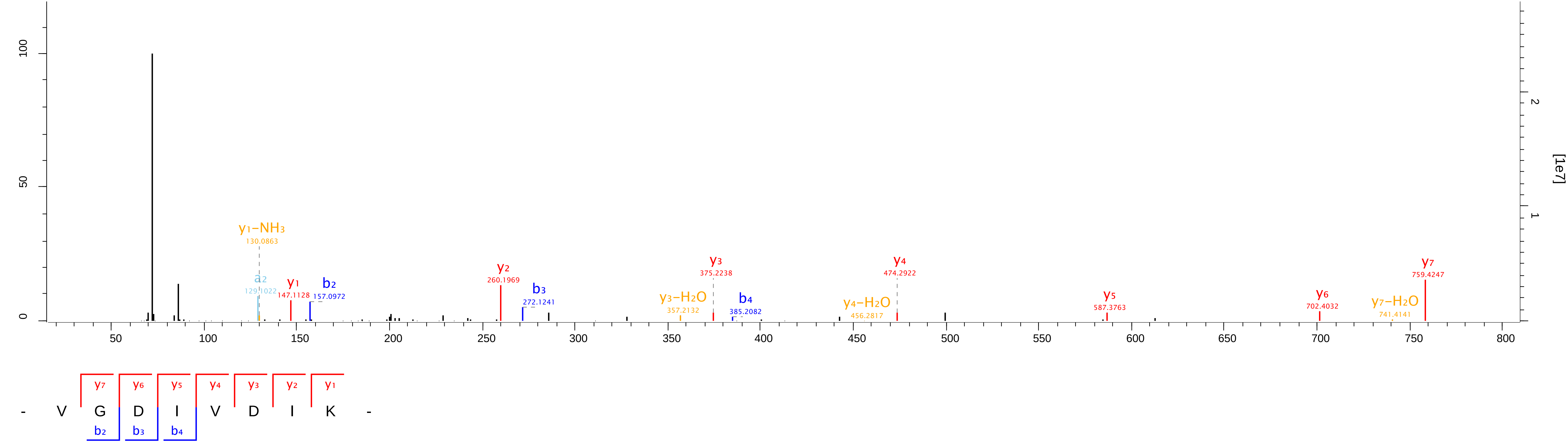

Raw file	Scan	Method	Score	m/z	Gene names
20140827_EXQ00_FaHo_SA_BDF2_03	8614	FTMS; HCD	166.6	1088.59	RPL9B

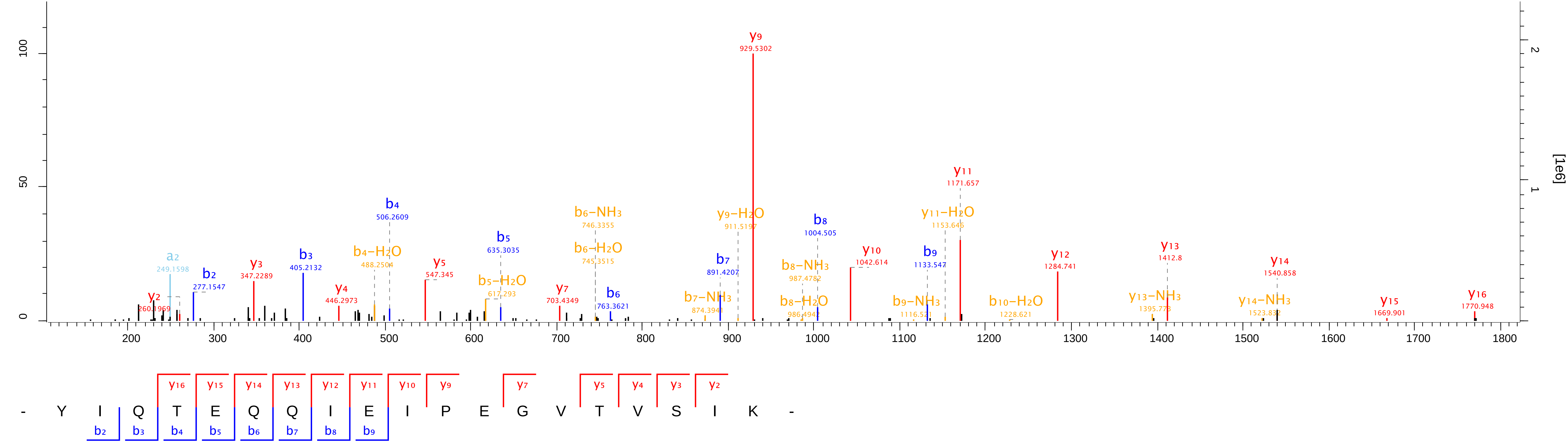

Raw file	Scan	Method	Score	m/z	Gene names
20140827_EXQ00_FaHo_SA_BRE2_01	4127	FTMS; HCD	7.94	413.2	ENO2

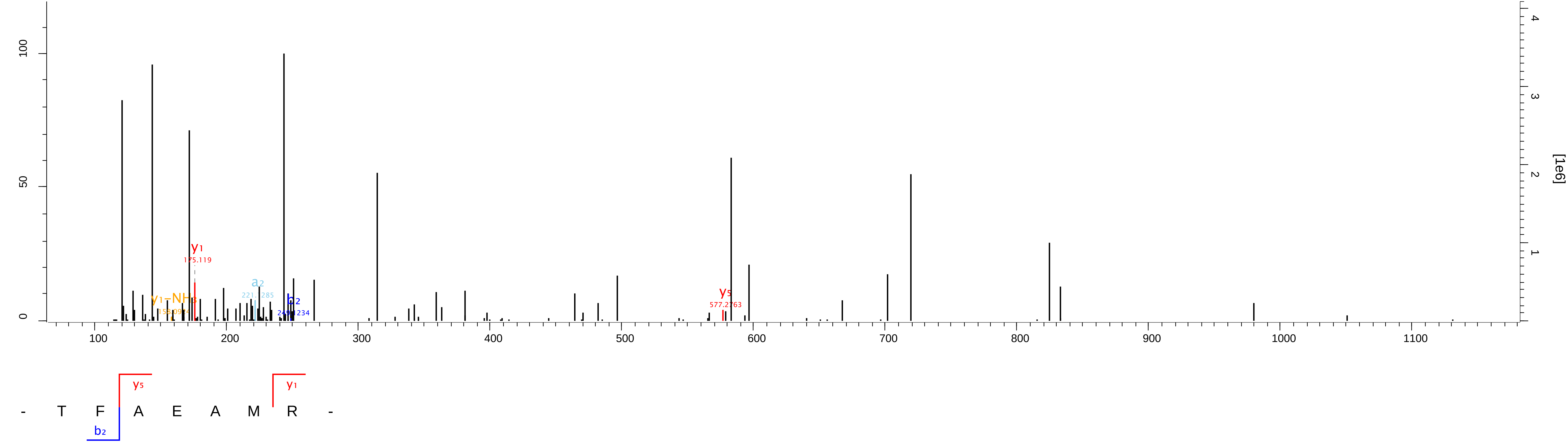

Raw file	Scan	Method	Score	m/z	Gene names
20140827_EXQ00_FaHo_SA_BRE2_01	4213	FTMS; HCD	63.62	568.79	VMA5

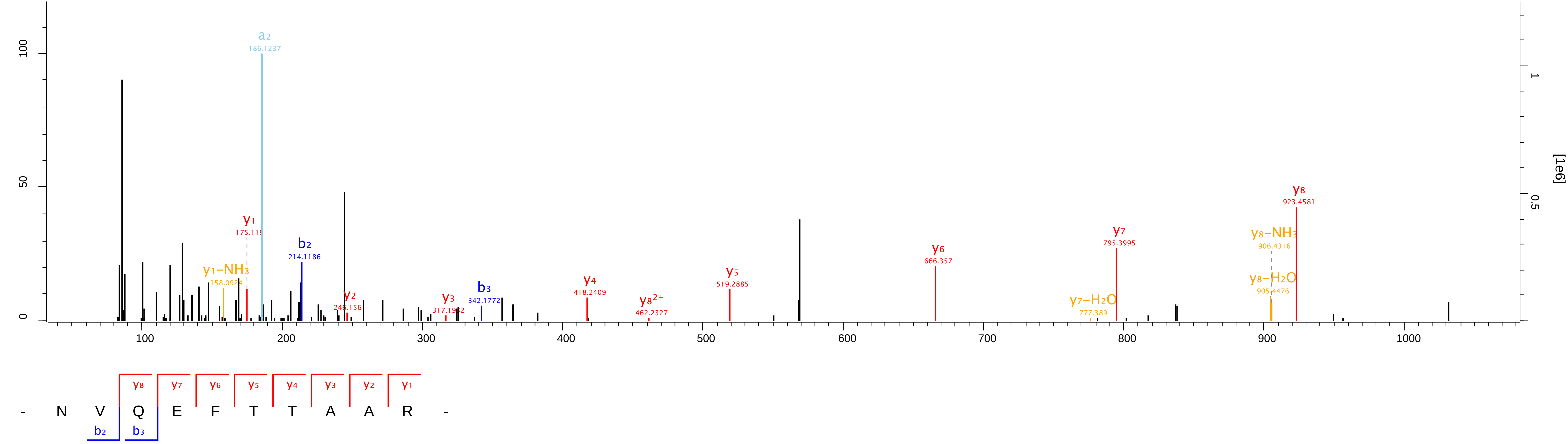

Raw file	Scan	Method	Score	m/z	Gene names
20140827_EXQ00_FaHo_SA_BRE2_01	4500	FTMS; HCD	95.42	652.83	HMF1

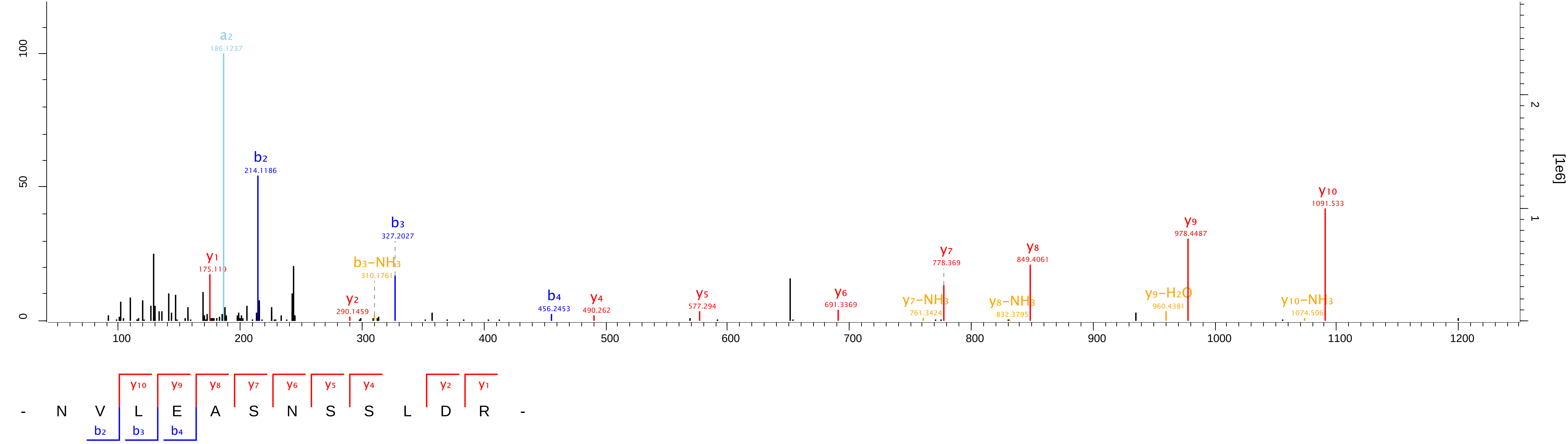

Raw file	Scan	Method	Score	m/z	Gene names
20140827_EXQ00_FaHo_SA_BRE2_02	5234	FTMS; HCD	208.39	773.41	RPL36A;RPL36B

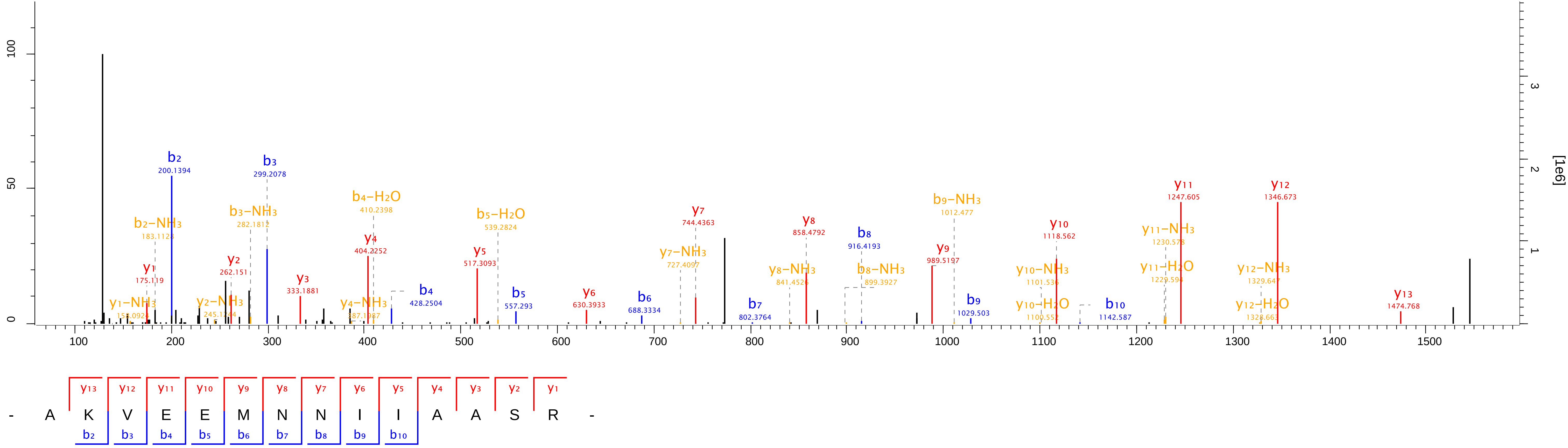

Raw file	Scan	Method	Score	m/z	Gene names
20140827_EXQ00_FaHo_SA_BRE2_02	6351	FTMS; HCD	68.41	896.44	CYT1

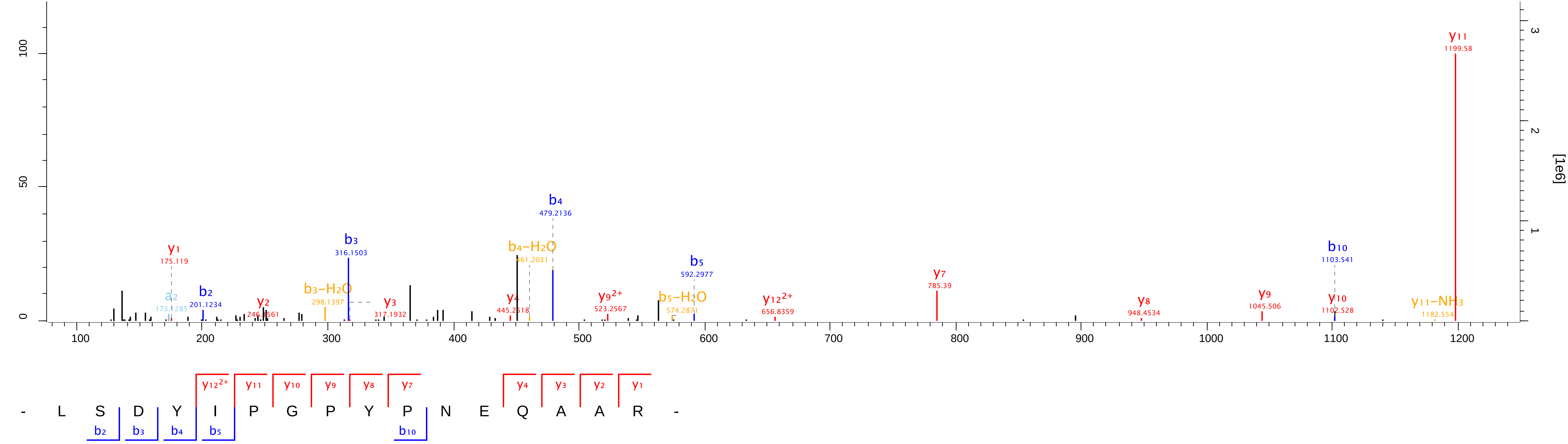

Raw file	Scan	Method	Score	m/z	Gene names
20140827_EXQ00_FaHo_SA_BRE2_02	6442	FTMS; HCD	117.93	687.35	ERV25

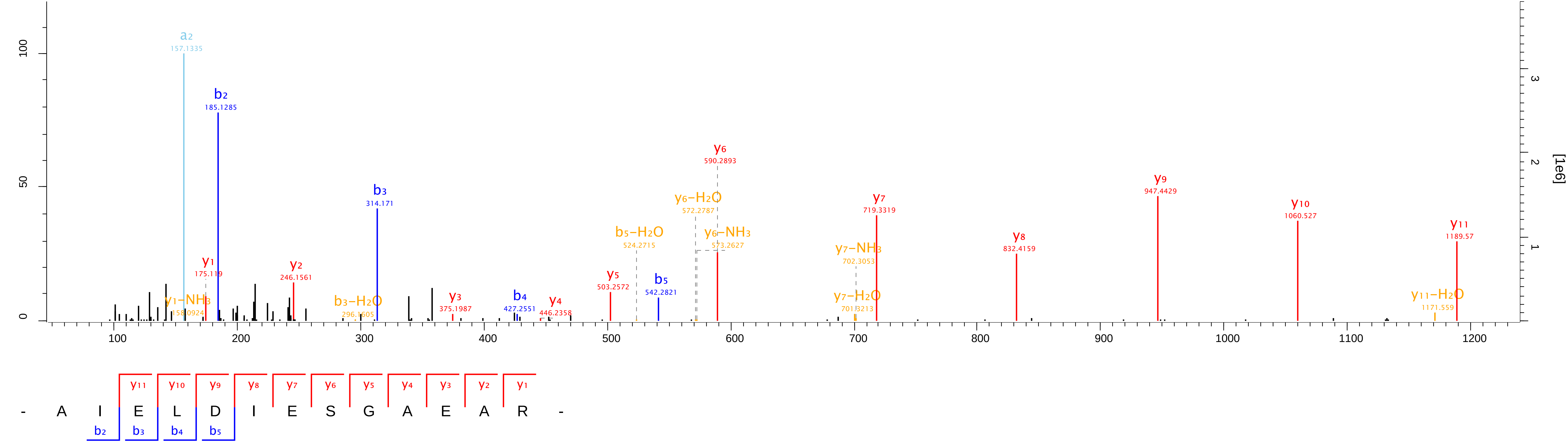

Raw file	Scan	Method	Score	m/z	Gene names
20140827_EXQ00_FaHo_SA_BRE2_02	8525	FTMS; HCD	69.98	900.45	NPT1

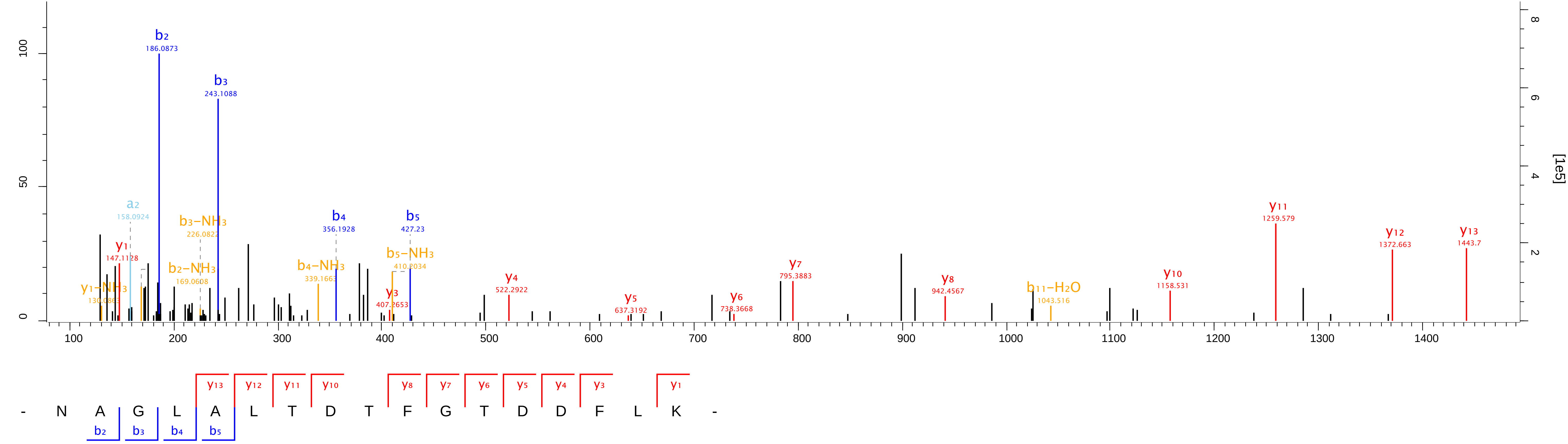

Raw file	Scan	Method	Score	m/z	Gene names
20140827_EXQ00_FaHo_SA_BRE2_03	4027	FTMS; HCD	70.89	668.55	ERV46

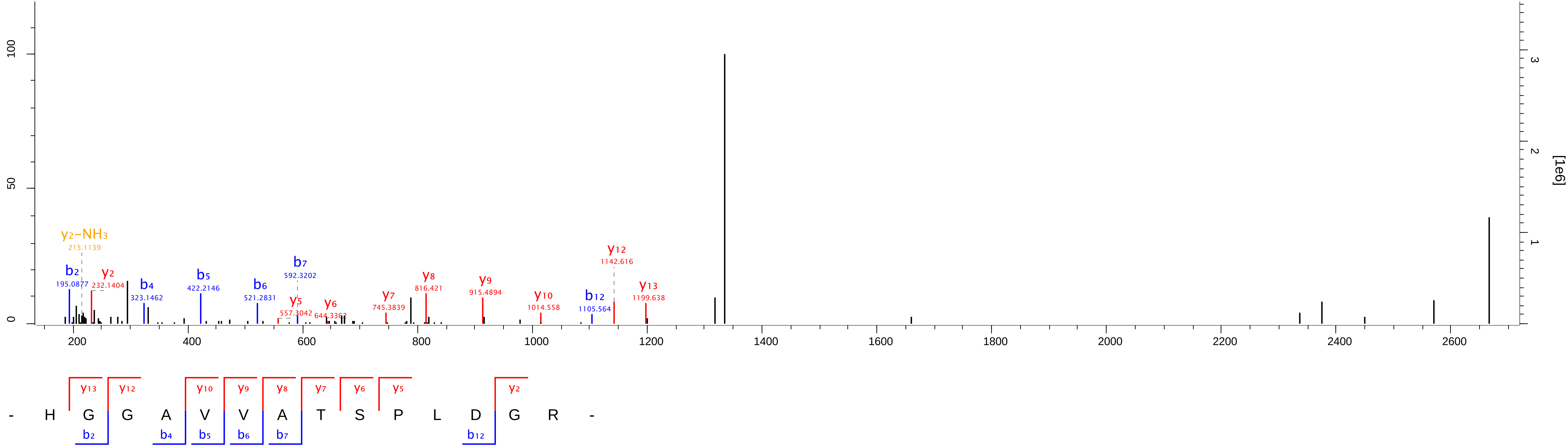

Raw file	Scan	Method	Score	m/z	Gene names
20140827_EXQ00_FaHo_SA_BRE2_03	6024	FTMS; HCD	87.32	523.26	PDR5;PDR15

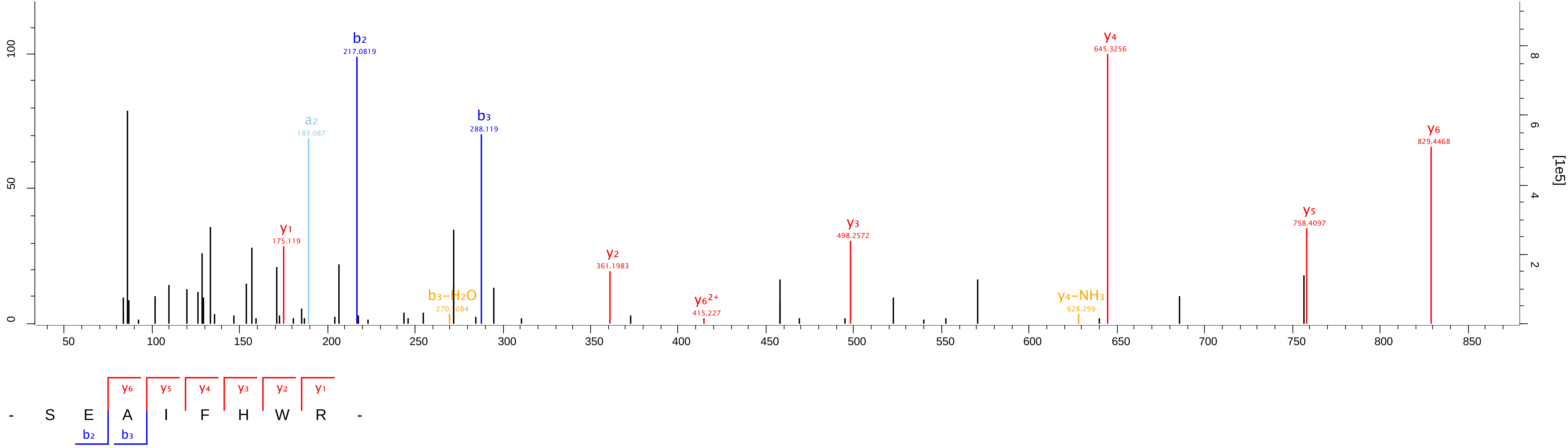

Raw file	Scan	Method	Score	m/z	Gene names
20140827_EXQ00_FaHo_SA_BRE2_03	6097	FTMS; HCD	86.88	442.25	AAC1

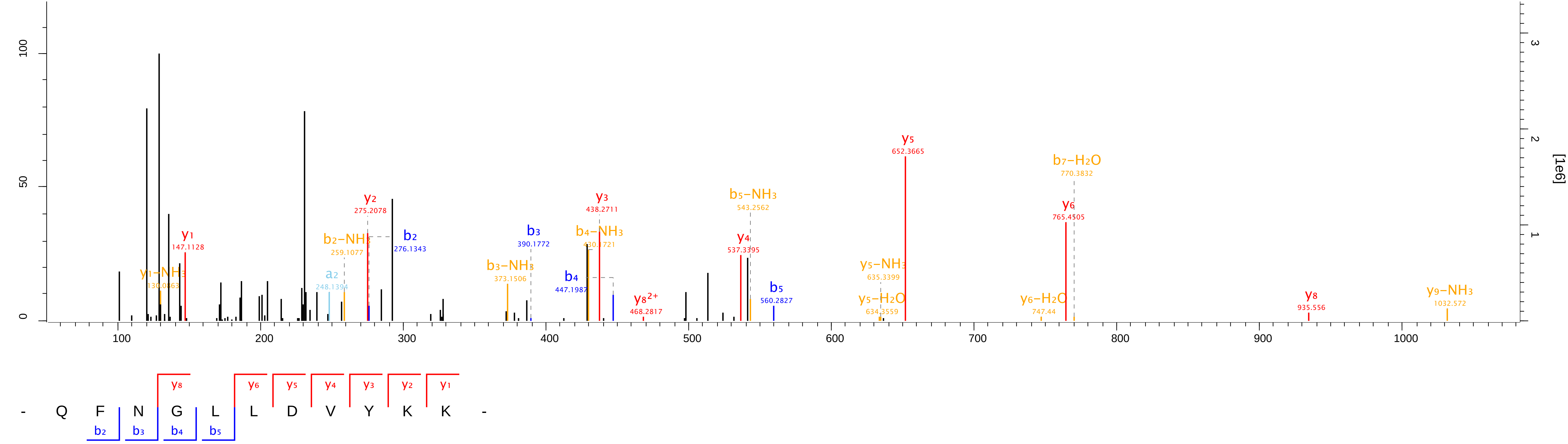

Raw file	Scan	Method	Score	m/z	Gene names
20140827_EXQ00_FaHo_SA_BRE2_03	8347	FTMS; HCD	124.6	646.85	PDR5;PDR15

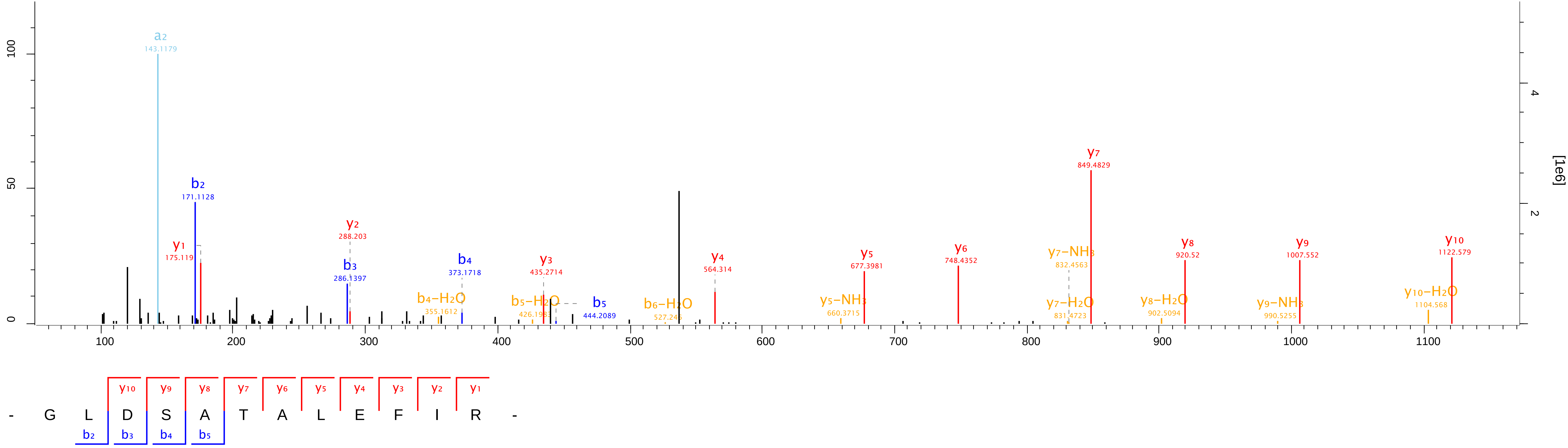

Raw file	Scan	Method	Score	m/z	Gene names
20140827_EXQ00_FaHo_SA_CTI6_03	1654	FTMS; HCD	74.42	359.2	RPL21A;RPL21B

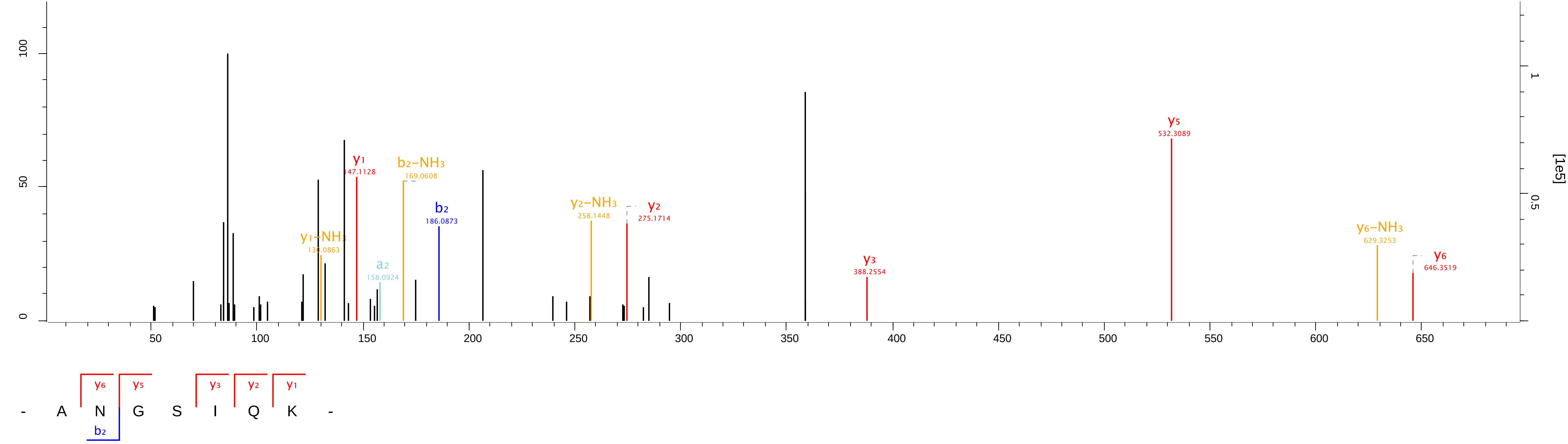

Raw file	Scan	Method	Score	m/z
20140827_EXQ00_FaHo_SA_CTI6_03	2759	FTMS; HCD	114.02	367.21

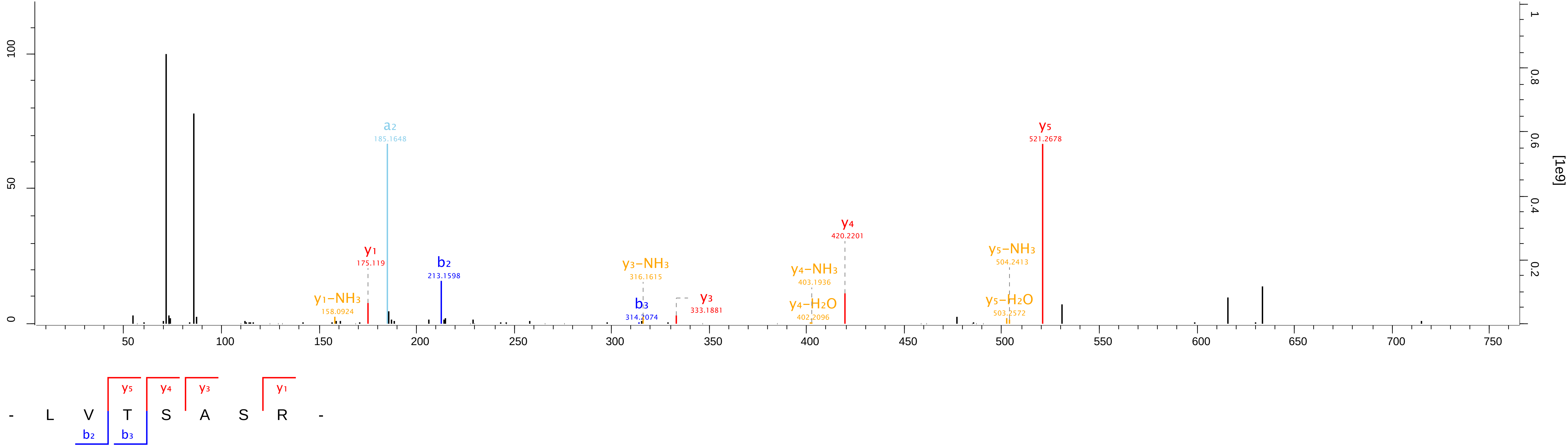

Raw file	Scan	Method	Score	m/z	Gene names
20140827_EXQ00_FaHo_SA_CTI6_03	3274	FTMS; HCD	81.66	653.33	TPM1

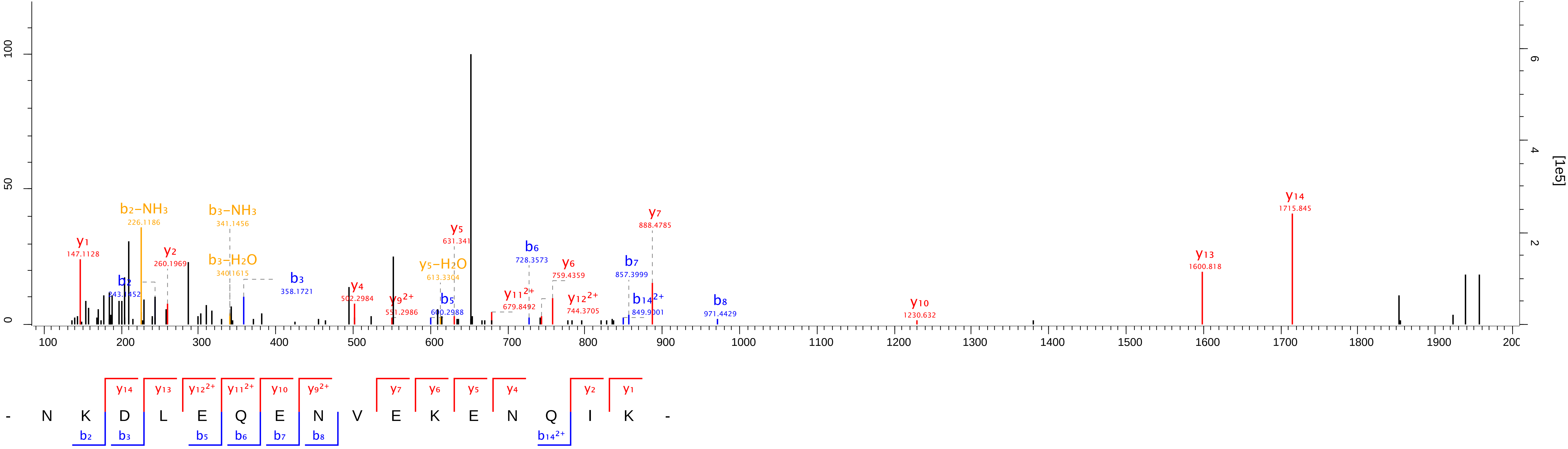

Raw file	Scan	Method	Score	m/z	Gene names
20140827_EXQ00_FaHo_SA_EAF3_01	3548	FTMS; HCD	70.29	701.83	TY1B-LR3;TY1A-PL;TY1A-LR2;TY1A-ER1;TY1A-DR6;TY1B-OL;TY1B-LR4;TY1B-LR2;TY1B-PL;TY1B-ER1;TY1B-PR3;TY1A-PR1;TY1A-A;TY1A-DR4

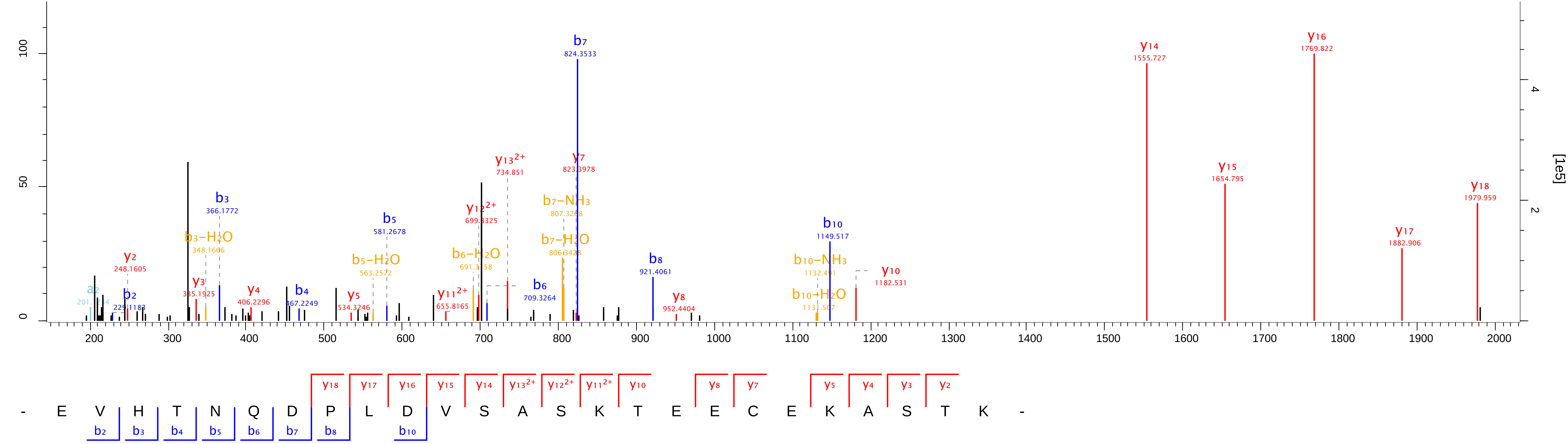

Raw file	Scan	Method	Score	m/z	Gene names
20140827_EXQ00_FaHo_SA_EAF3_01	3957	FTMS; HCD	89.23	507.26	TY1B-LR3;TY1B-OL;TY1B-LR4;TY1B-LR2;TY1B-PL;TY1B-ER1;TY1B-PR3;TY1B-H;TY1B-GR2;TY1B-MR2;TY1B-ER2;TY1B-OR;TY1B-BR;TY1B-DR1;TY1B-NL2;TY1B-LR1;TY1B-DR3;TY1B-NL1;TY1B-A;TY1B-BL;TY1B-MR1

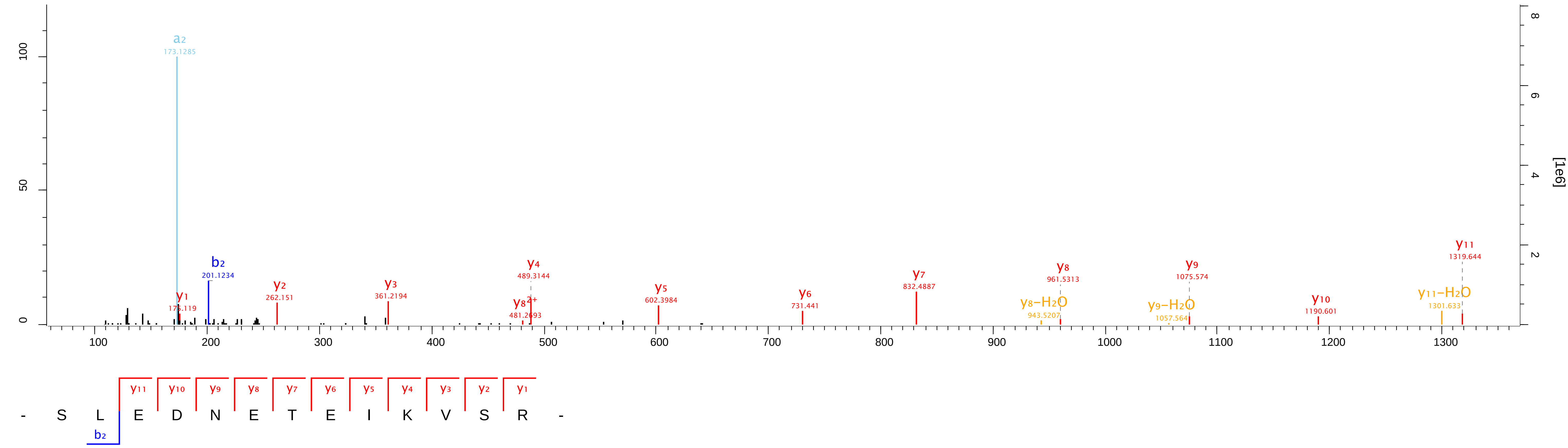

Raw file	Scan	Method	Score	m/z	Gene names
20140827_EXQ00_FaHo_SA_EAF3_01	4134	FTMS; HCD	79.65	778.85	TY1B-LR3;TY1B-OL;TY1B-LR4;TY1B-LR2;TY1B-PL;TY1B-ER1;TY1B-PR3;TY1B-H;TY1B-GR2;TY1B-MR2;TY1B-ER2;TY1B-OR;TY1B-BR;TY1B-DR1;TY1B-NL2;TY1B-LR1;TY1B-DR3;TY1B-NL1;TY1B-A;TY1B-BL;TY1B-MR1

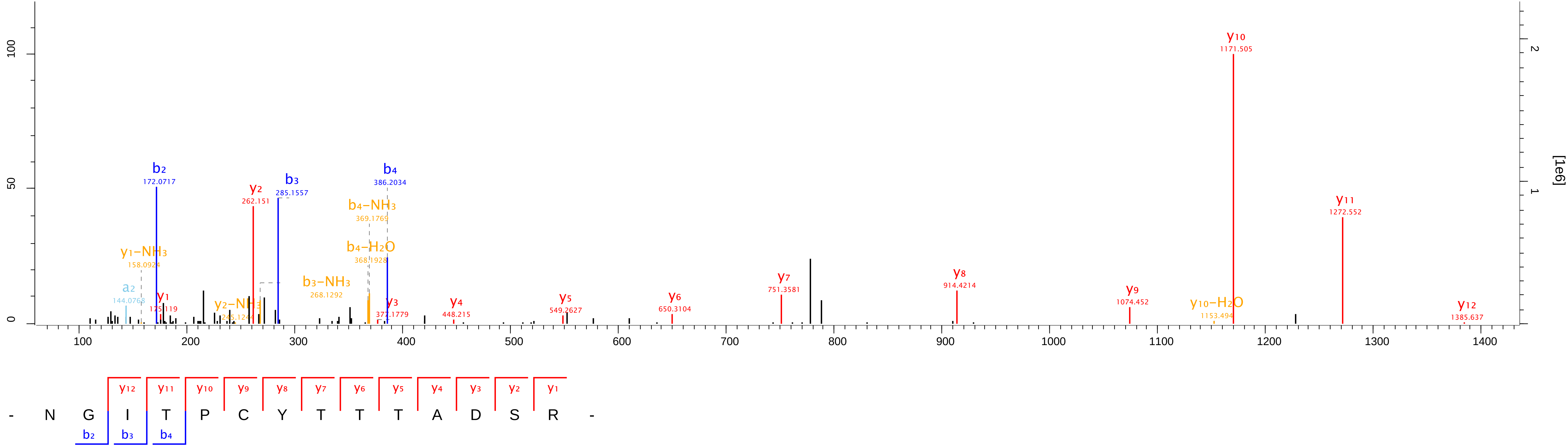

Raw file	Scan	Method	Score	m/z	Gene names
20140827_EXQ00_FaHo_SA_EAF3_01	4562	FTMS; HCD	94.66	432.79	TY1B-OL;TY1B-LR4;TY1B-LR2;TY1B-PL;TY1B-ER1;TY1B-PR3;TY1B-H;TY1B-GR2;TY1B-MR2;TY1B-ER2;TY1B-OR;TY1B-BR;TY1B-DR1;TY1B-NL2;TY1B-LR1;TY1B-DR3;TY1B-NL1;TY1B-A;TY1B-BL;TY1B-MR1

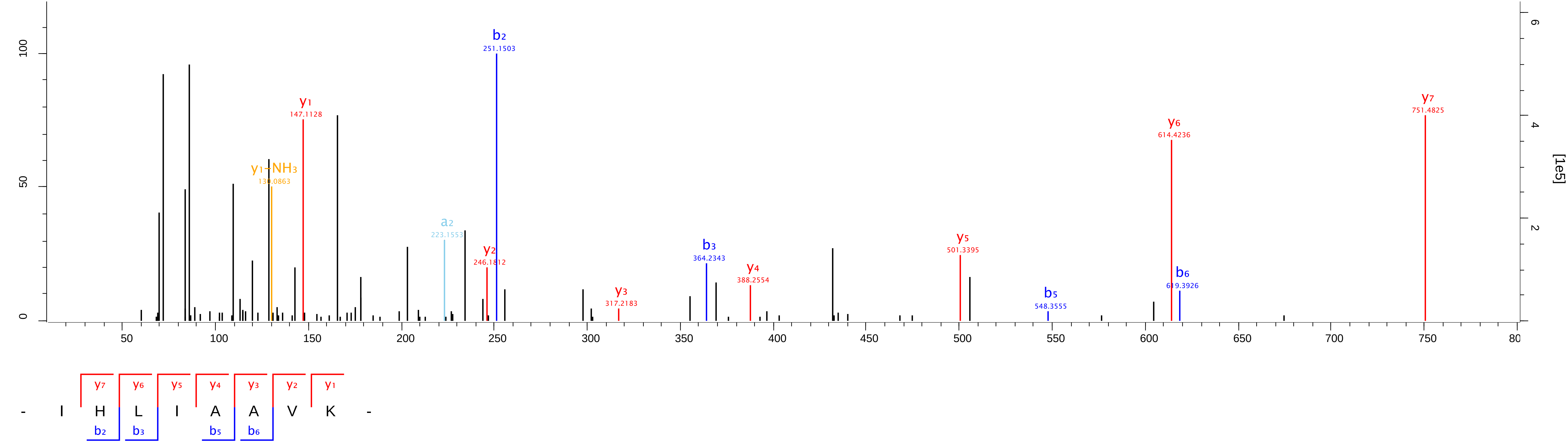

Raw file	Scan	Method	Score	m/z	Gene names
20140827_EXQ00_FaHo_SA_EAF3_01	4781	FTMS; HCD	91.66	581.64	RPS14B;RPS14A

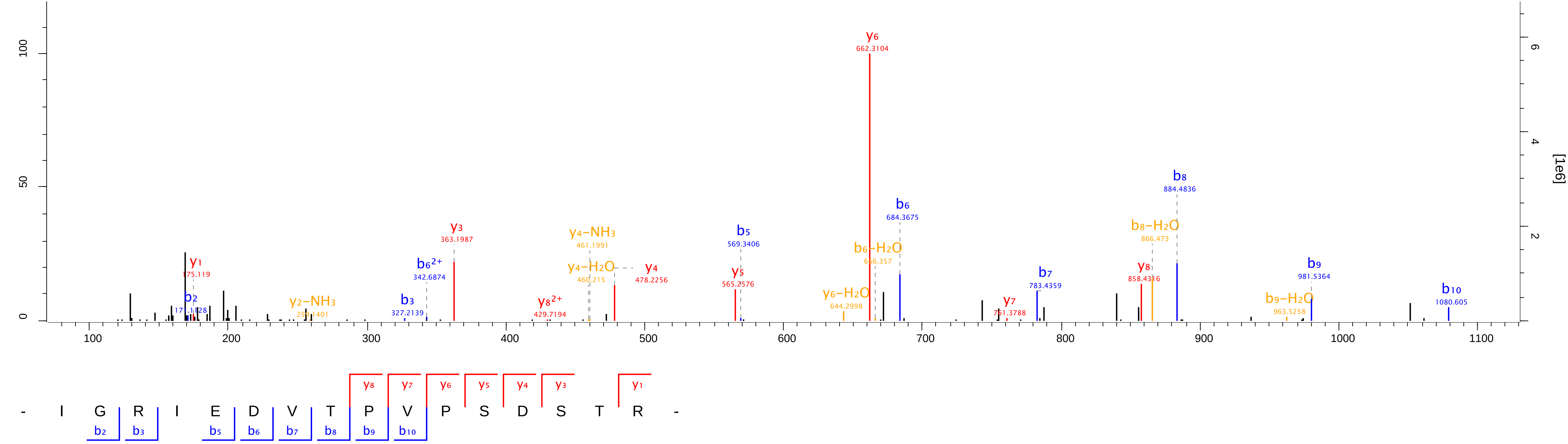

Raw file	Scan	Method	Score	m/z	Gene names
20140827_EXQ00_FaHo_SA_E	497[illegible]	FTMS;	137.1	447.7	TY1B-LR3;TY1B-OL;TY1B-LR4;TY1B-LR2;TY1B-PL;TY1B-ER1;TY1B-PR3;TY1B-H;TY1B-GR2;TY1B-MR2;TY1B-ER2;TY1B-DR1;TY1B-LR1;TY1B-DR3;TY1B-NL1;TY1B-A;TY1B-BL;TY1B-MR1;TY2B-B;TY2B-GR1;TY2B-OR2;TY2B-DR2;TY2B-GR

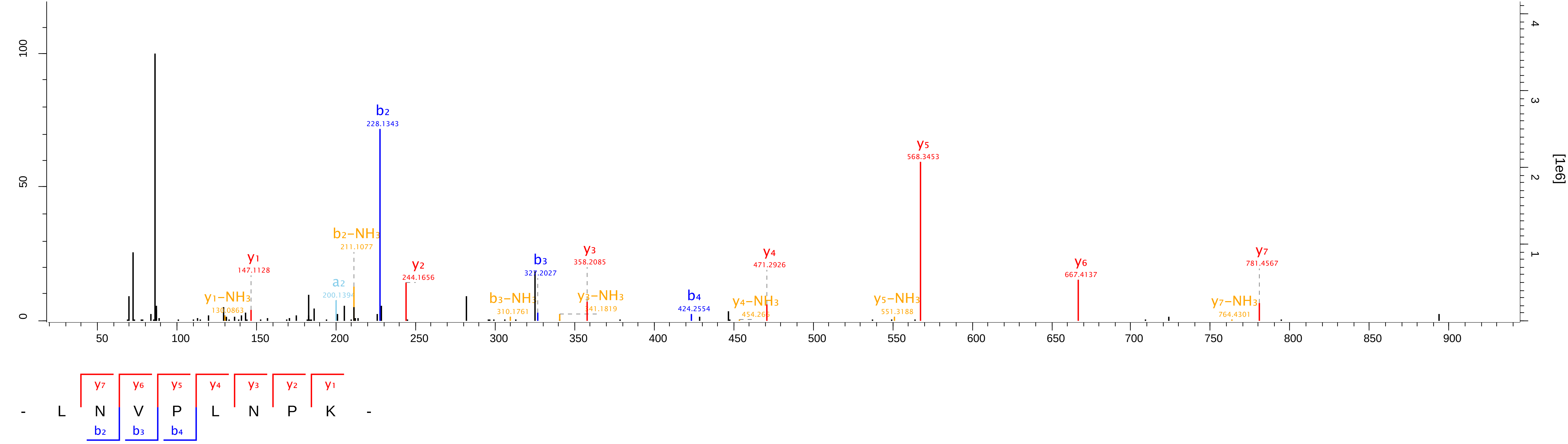

Raw file	Scan	Method	Score	m/z	Gene names
20140827_EXQ00_FaHo_SA_EAF3_01	6070	FTMS; HCD	77.22	649.97	ATO3

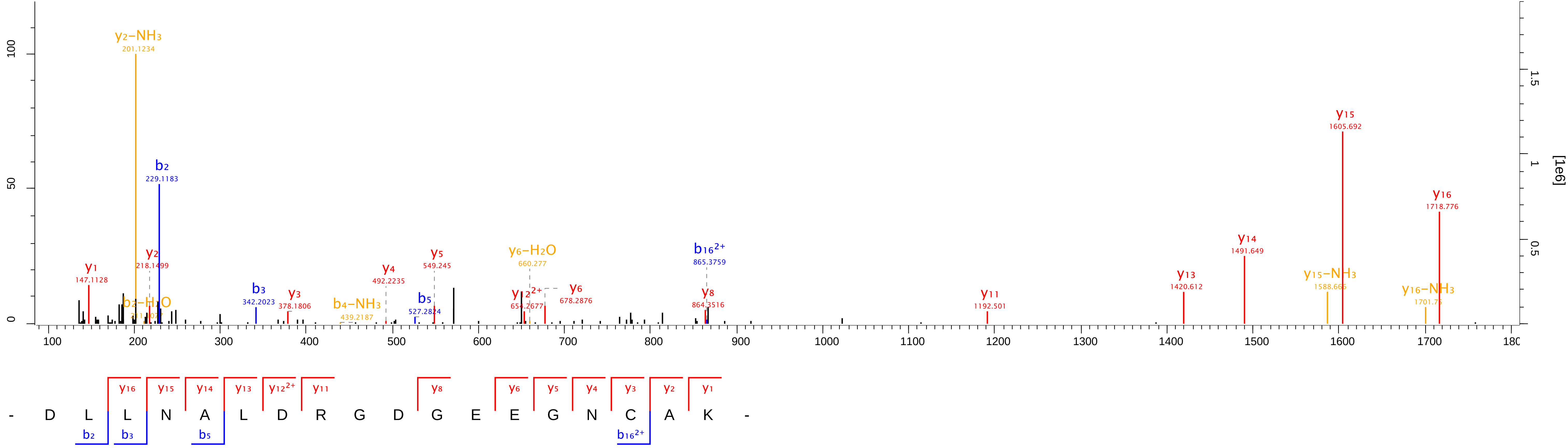

Raw file	Scan	Method	Score	m/z	Gene names
20140827_EXQ00_FaHo_SA_EAF3_01	6822	FTMS; HCD	121.68	660.37	TY1B-OL;TY1B-LR2;TY1B-PL;TY1B-ER1;TY1B-PR3;TY1B-H;TY1B-MR2;TY1B-ER2;TY1B-OR;TY1B-BR;TY1B-LR1;TY1B-NL1;TY1B-A;TY1B-BL;TY1B-MR1;TY2B-DR3

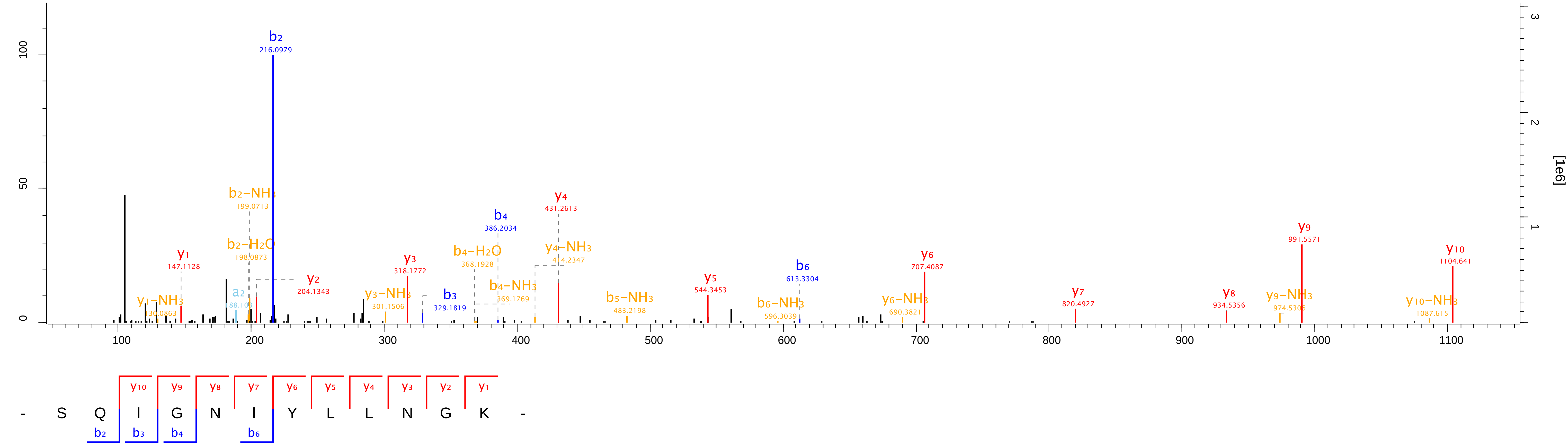

Raw file	Scan	Method	Score	m/z	Gene names
20140827_EXQ00_FaHo_SA_EAF3_01	8011	FTMS; HCD	79.84	996.51	RPL7A;RPL7B

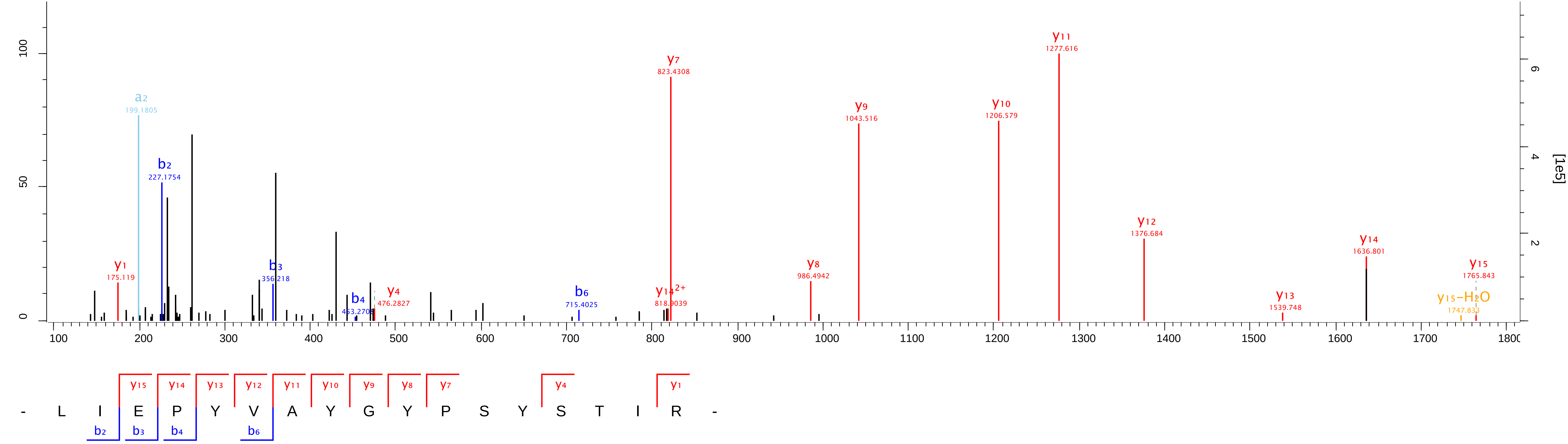

Raw file	Scan	Method	Score	m/z	Gene names
20140827_EXQ00_FaHo_SA_EAF3_02	5308	FTMS; HCD	164.27	888.07	TY1B-H;TY1B-MR2;TY1B-ER2;TY1B-OR;TY1B-BR;TY1B-DR1;TY1B-LR1;TY1B-DR3

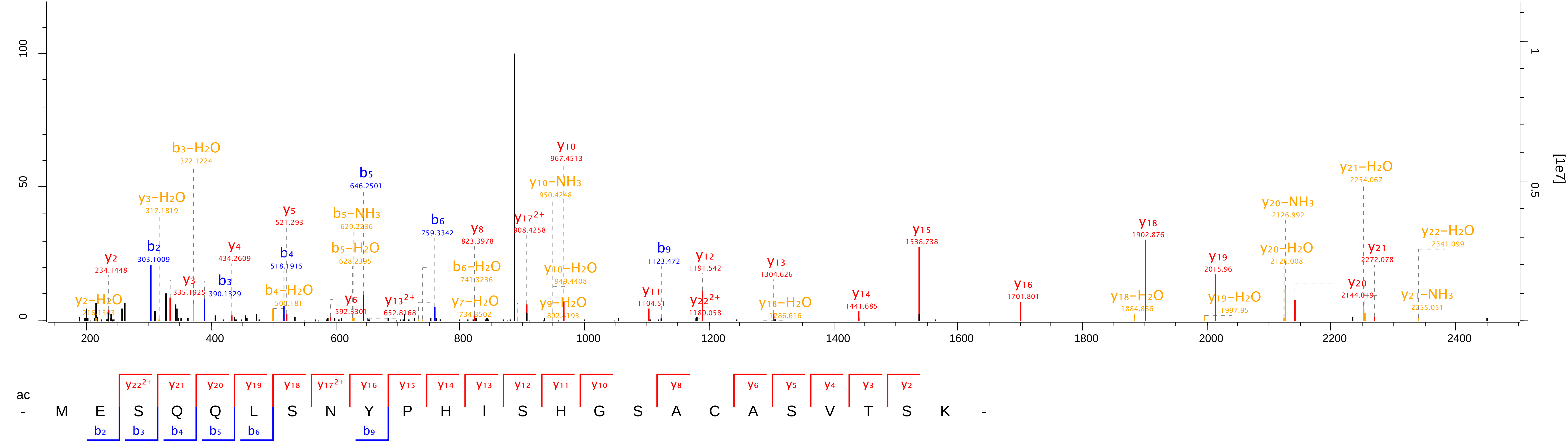

Raw file	Scan	Method	Score	m/z	Gene names
20140827_EXQ00_FaHo_SA_EAF3_02	7798	FTMS; HCD	72.99	815.09	TY1B-LR3;TY1B-OL;TY1B-LR4;TY1B-LR2;TY1B-PL;TY1B-ER1;TY1B-PR3;TY1B-H;TY1B-GR2;TY1B-MR2;TY1B-ER2;TY1B-OR;TY1B-BR;TY1B-DR1;TY1B-NL2;TY1B-LR1;TY1B-DR3;TY1B-NL1;TY1B-BL;TY1B-MR1

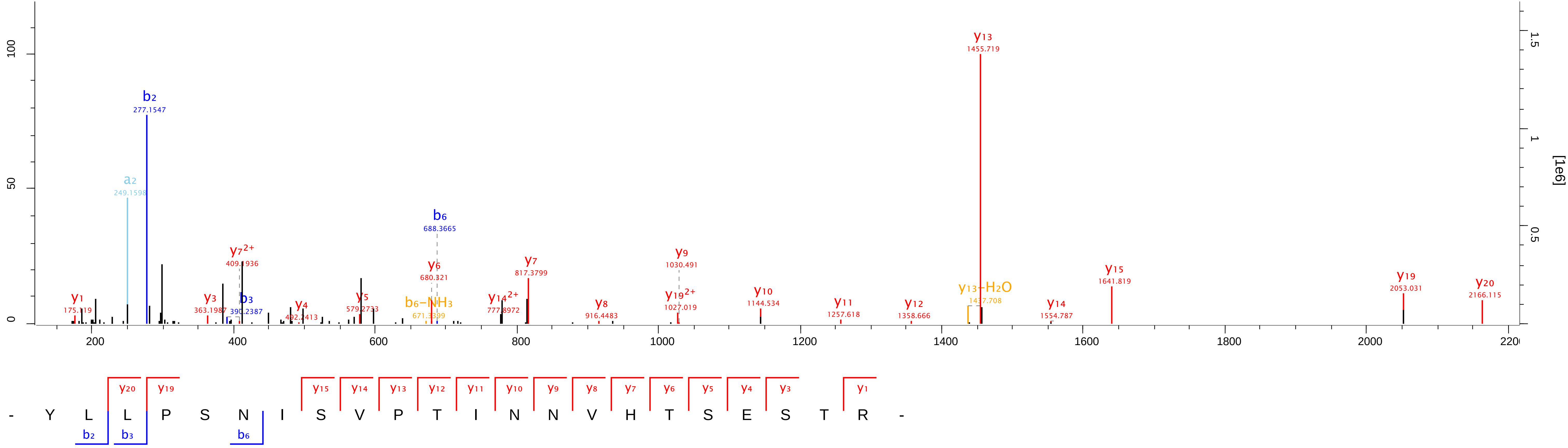

Raw file	Scan	Method	Score	m/z	Gene names
20140827_EXQ00_FaHo_SA_EAF3_03	2752	FTMS; HCD	65.3	504.24	TY1B-OL;TY1B-LR4;TY1B-LR2;TY1B-PL;TY1B-ER1;TY1B-H;TY1B-GR2;TY1B-MR2;TY1B-ER2;TY1B-OR;TY1B-BR;TY1B-DR1;TY1B-NL2;TY1B-LR1;TY1B-DR3;TY1B-NL1;TY1B-A;TY1B-BL;TY1B-MR1

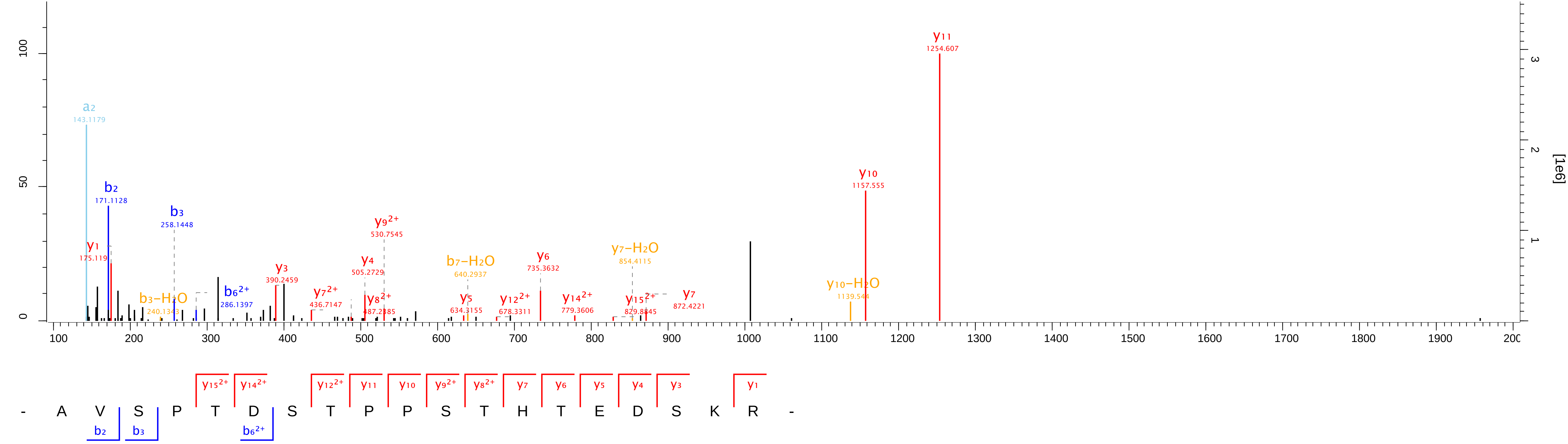

Raw file	Scan	Method	Score	m/z	Gene names
20140827_EXQ00_FaHo_SA_EAF3_03	3538	FTMS; HCD	163.84	414.75	RPL9A;RPL9B

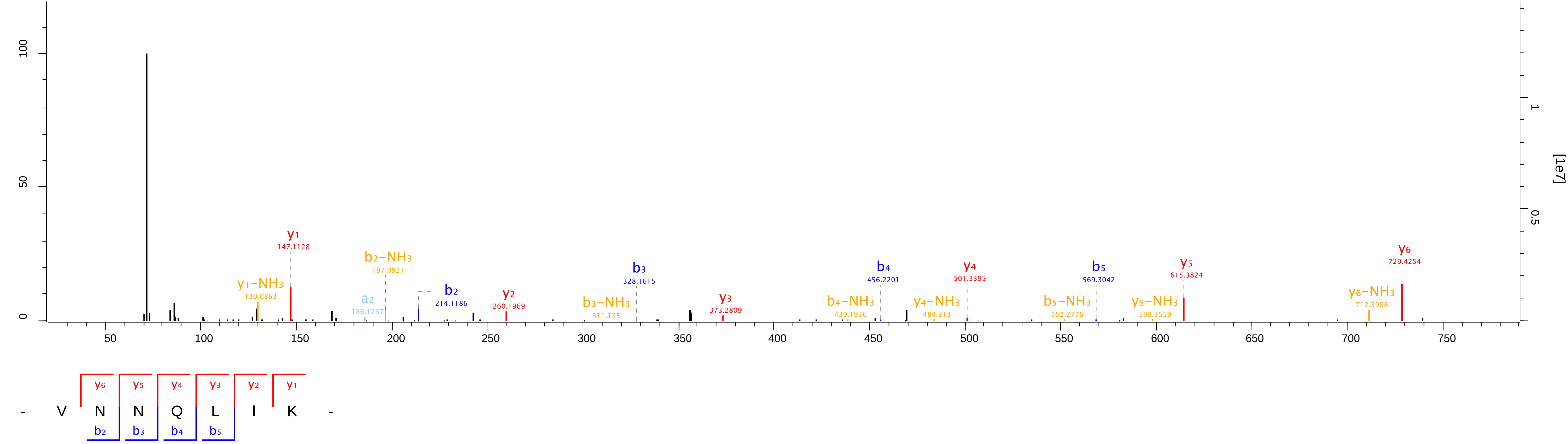

Raw file	Scan	Method	Score	m/z	Gene names
20140827_EXQ00_FaHo_SA_EAF3_03	3721	FTMS; HCD	72.2	589.28	TY1B-LR3;TY1B-OL;TY1B-LR4;TY1B-LR2;TY1B-PL;TY1B-ER1;TY1B-PR3;TY1B-H;TY1B-GR2;TY1B-MR2;TY1B-ER2;TY1B-OR;TY1B-BR;TY1B-DR1;TY1B-NL2;TY1B-LR1;TY1B-DR3;TY1B-NL1;TY1B-A;TY1B-BL;TY1B-MR1

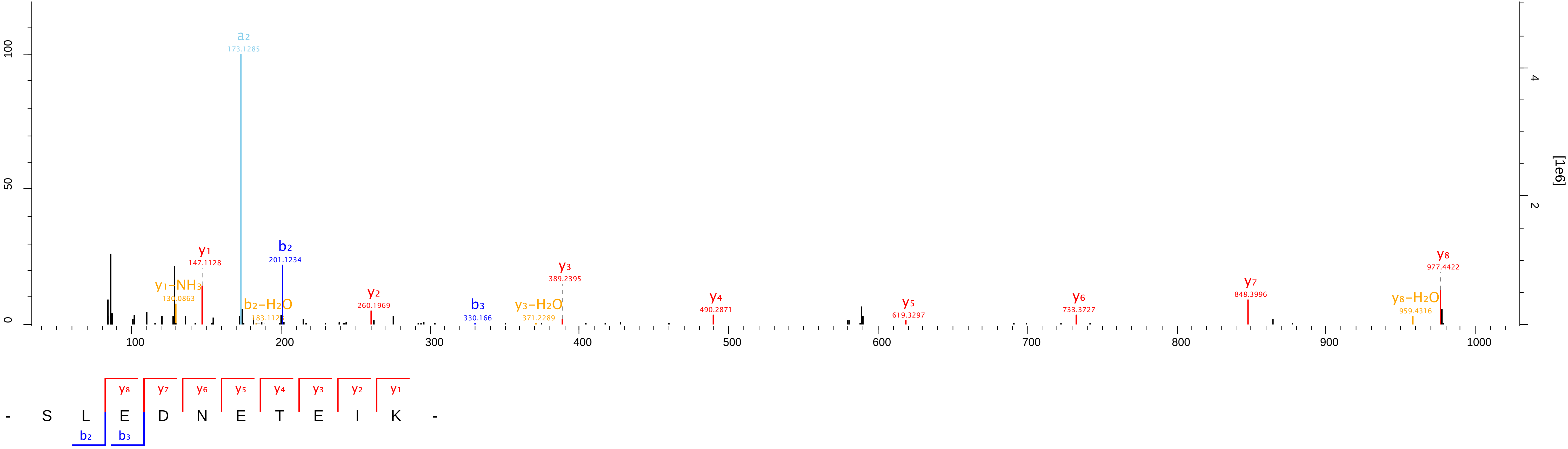

Raw file	Scan	Method	Score	m/z	Gene names
20140827_EXQ00_FaHo_SA_EAF3_03	4506	FTMS; HCD	97.47	561.28	TY1B-LR3;TY1B-OL;TY1B-LR4;TY1B-LR2;TY1B-PL;TY1B-ER1;TY1B-PR3;TY1B-H;TY1B-GR2;TY1B-MR2;TY1B-ER2;TY1B-OR;TY1B-BR;TY1B-LR1;TY1B-NL1;TY1B-BL;TY1B-MR1

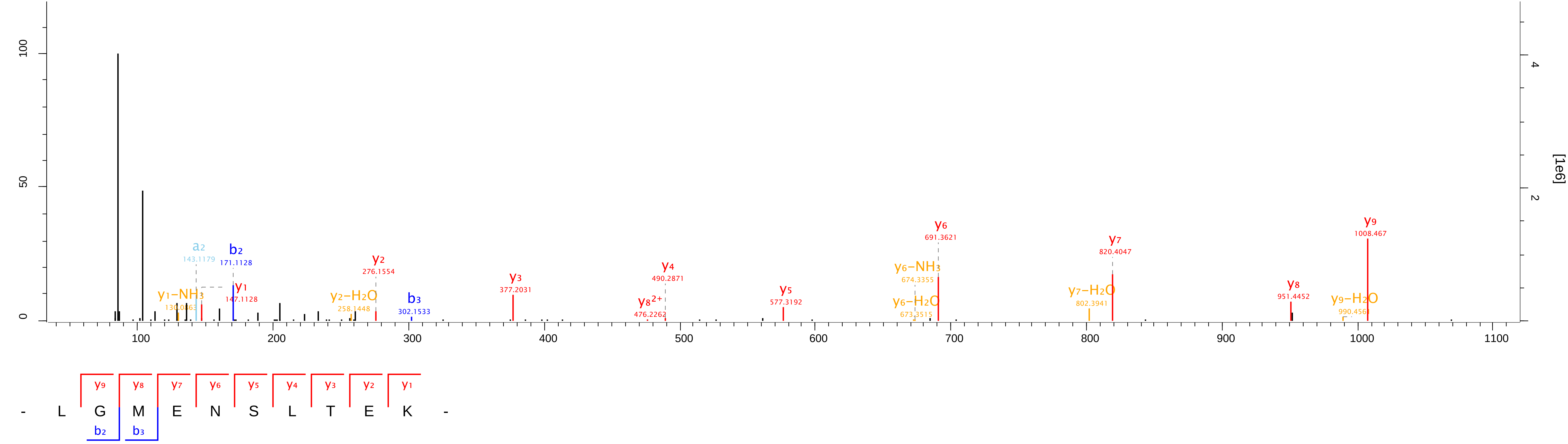

Raw file	Scan	Method	Score	m/z	Gene names
20140827_EXQ00_FaHo_SA_EAF3_03	4950	FTMS; HCD	62.66	381.24	TY1B-LR3;TY1B-OL;TY1B-LR4;TY1B-LR2;TY1B-PL;TY1B-ER1;TY1B-GR2;TY1B-DR1;TY1B-NL2;TY1B-DR3;TY1B-A;TY2B-B;TY2B-GR1;TY2B-OR2;TY2B-DR2;TY2B-GR2;TY2B-F;TY2B-C;TY2B-OR1;TY2B-DR1;TY2B-DR3;TY2B

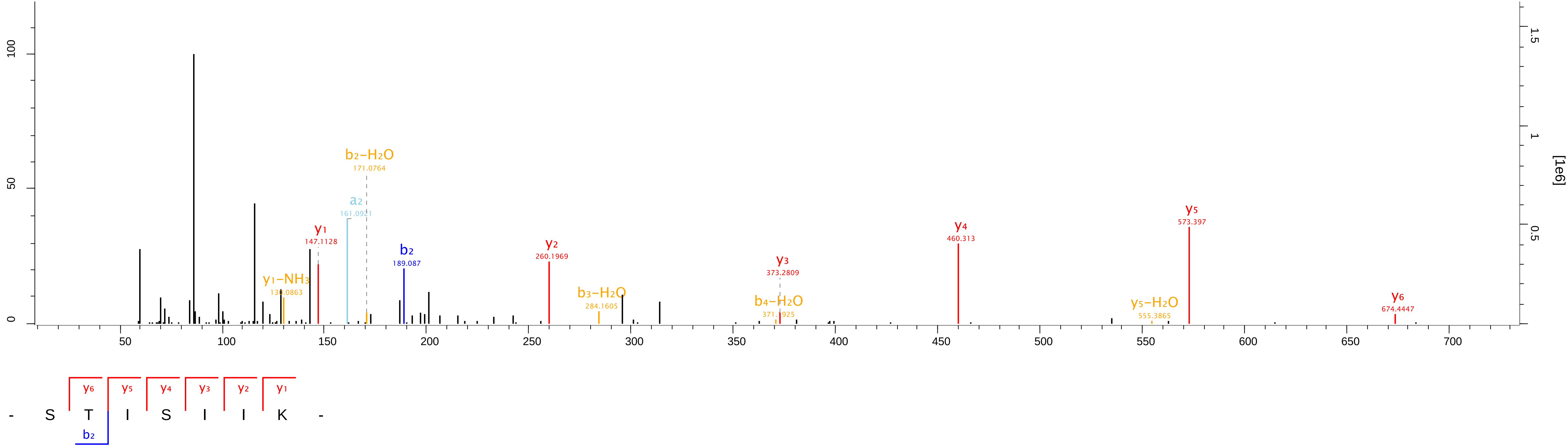

Raw file	Scan	Method	Score	m/z	Gene names
20140827_EXQ00_FaHo_SA_EAF3_03	5477	FTMS; HCD	99.07	816.41	TY1B-BL;TY1B-MR1;TY1A-DR2;TY1A-MR1

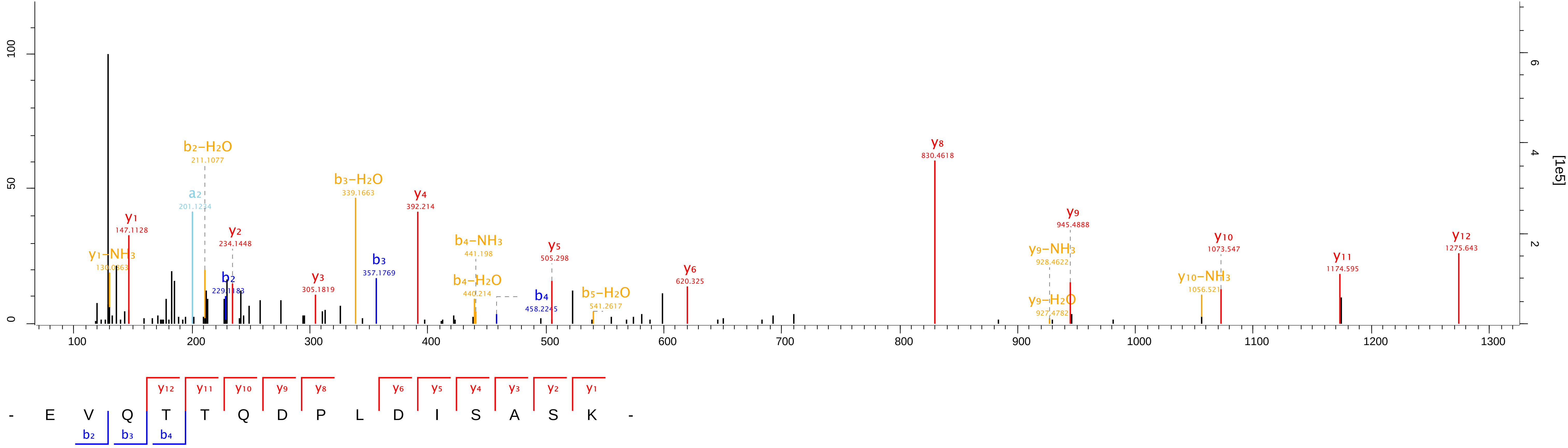

Raw file	Scan	Method	Score	m/z	Gene names
20140827_EXQ00_FaHo_SA_EAF3_03	5763	FTMS; HCD	102.69	887.08	TY1B-BL

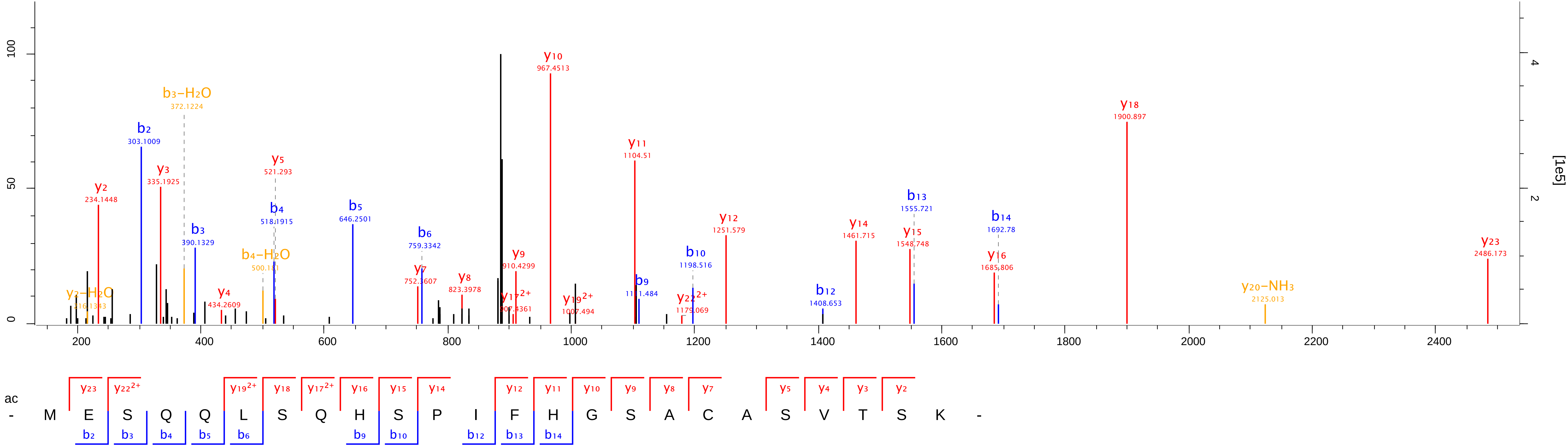

Raw file	Scan	Method	Score	m/z	Gene names
20140827_EXQ00_FaHo_SA_EAF3_03	5868	FTMS; HCD	129.08	1327.11	TY1B-OL

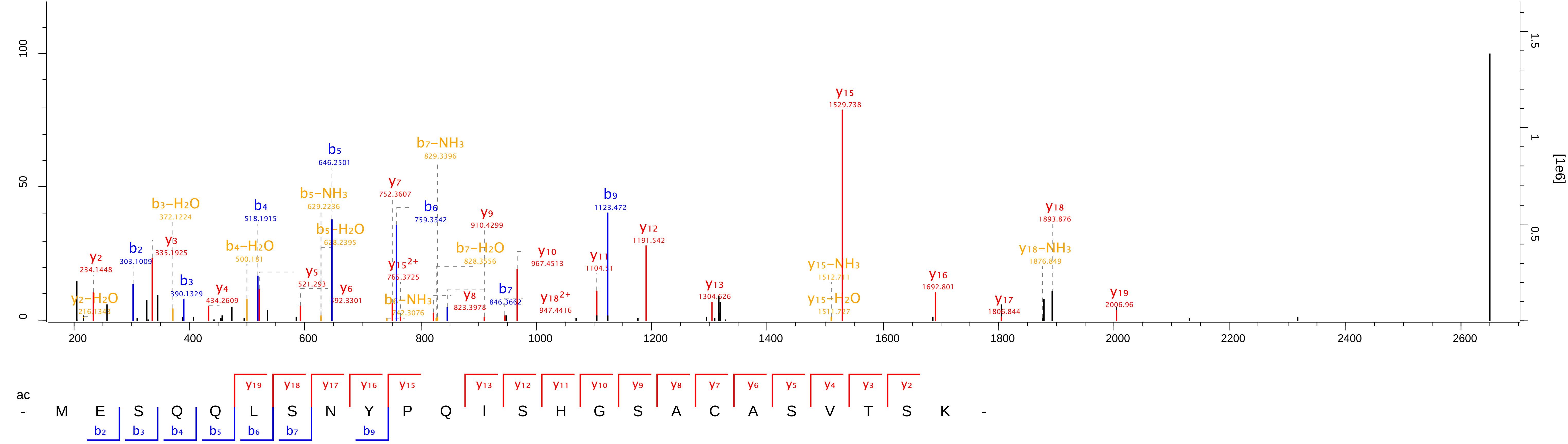

Raw file	Scan	Method	Score	m/z	Gene names
20140827_EXQ00_FaHo_SA_EAF3_03	5923	FTMS; HCD	80.63	894.94	TY1B-LR3;TY1B-OL;TY1B-LR4;TY1B-LR2;TY1B-PL;TY1B-ER1;TY1B-PR3;TY1B-H;TY1B-GR2;TY1B-DR1;TY1B-NL2;TY1B-DR3;TY1B-A;TY2B-B;TY2B-GR1;TY2B-OR2;TY2B-DR2;TY2B-GR2;TY2B-F;TY2B-C;TY2B-OR1;TY2B-DR1

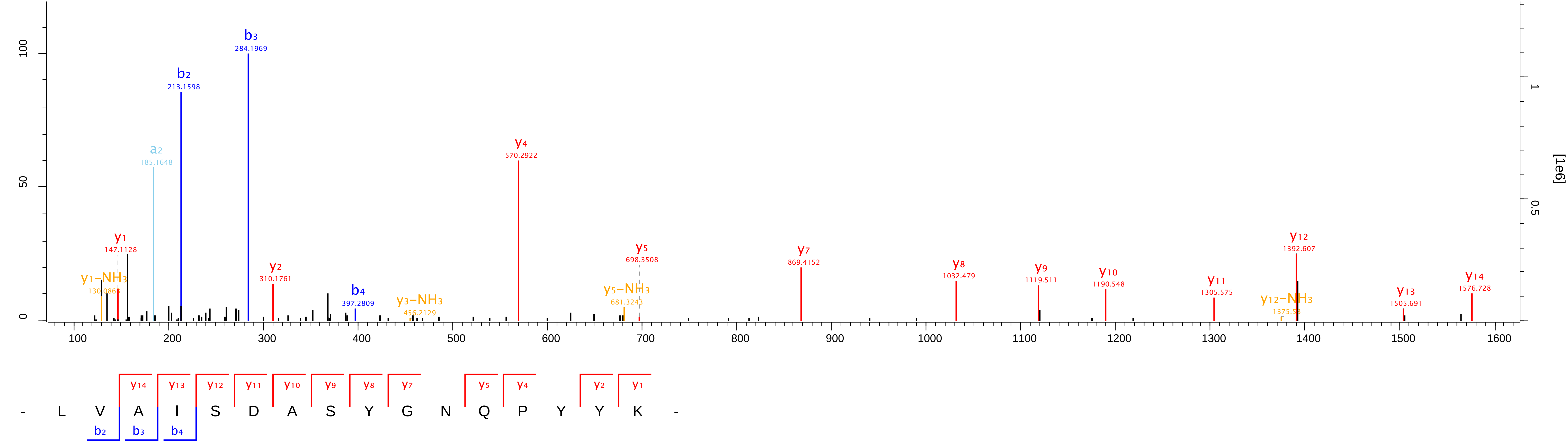

Raw file	Scan	Method	Score	m/z	Gene names
20140827_EXQ00_FaHo_SA_EAF3_03	6294	FTMS; HCD	90.69	871.4	TY1B-PR3

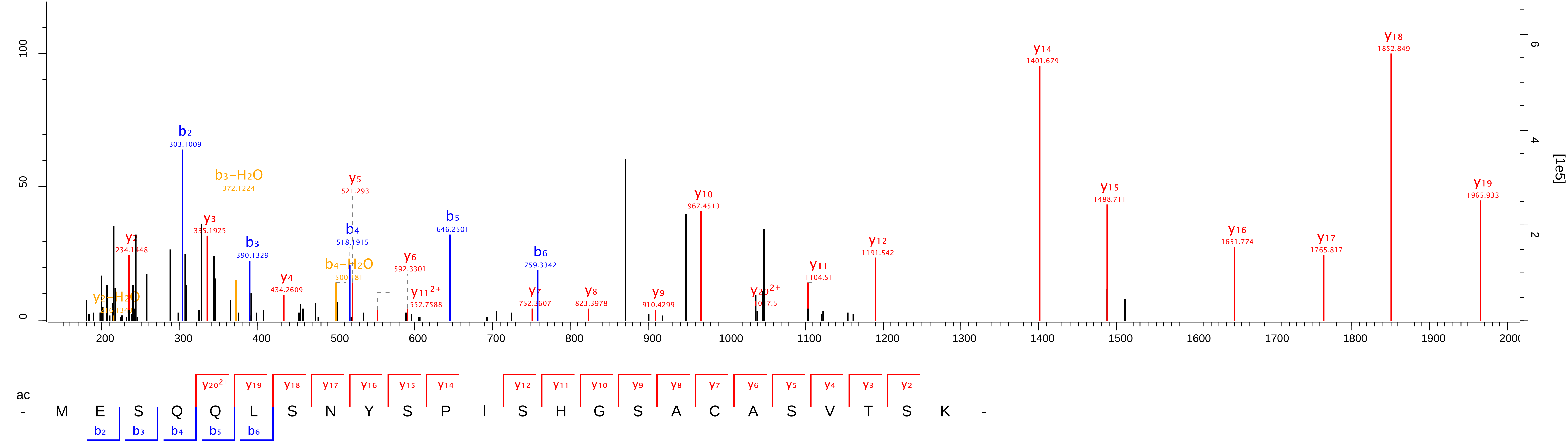

Raw file	Scan	Method	Score	m/z	Gene names
20140827_EXQ00_FaHo_SA_EAF3_03	7268	FTMS; HCD	88.6	596.31	PDR5;PDR15;PDR10

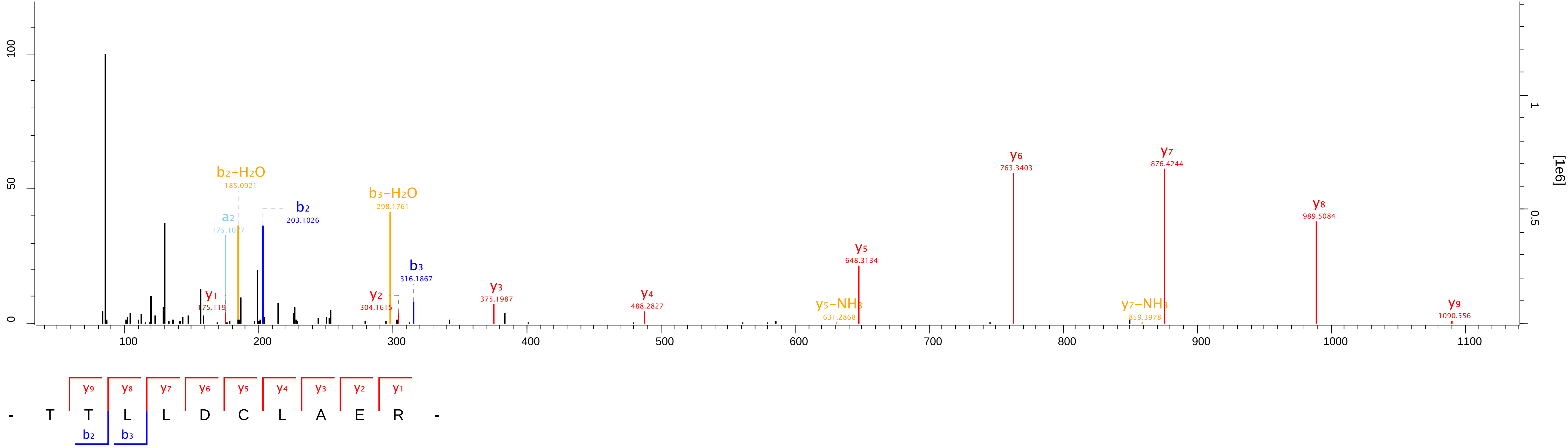

Raw file	Scan	Method	Score	m/z	Gene names
20140827_EXQ00_FaHo_SA_EAF3_03	7960	FTMS; HCD	97.24	880.47	TY1B-NL1;TY1B-BL;TY1B-MR1;TY1A-DR2;TY1A-MR1

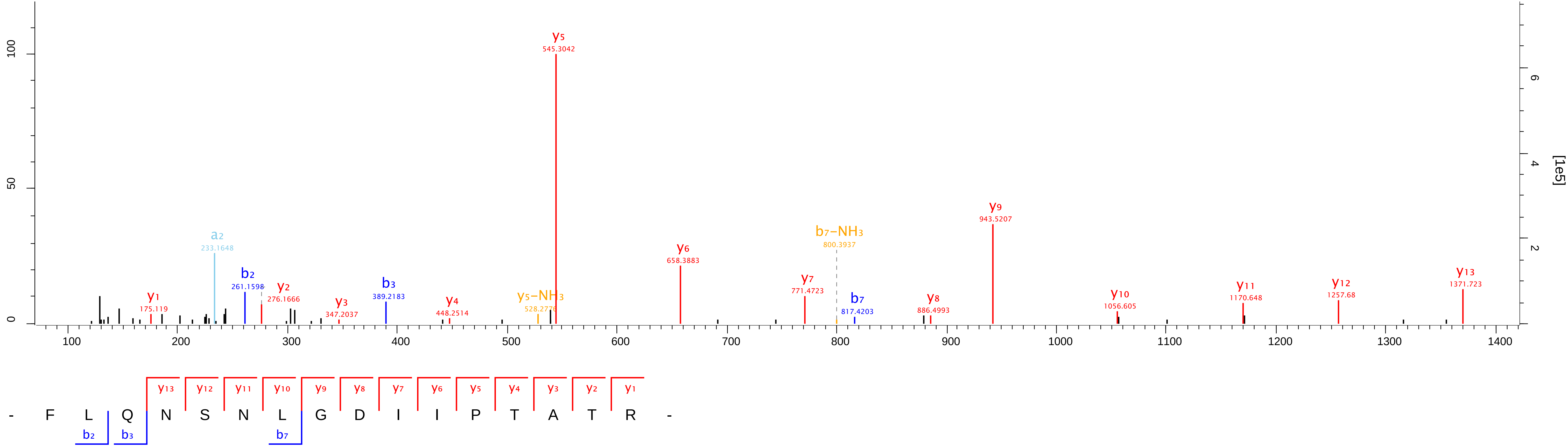

Raw file	Scan	Method	Score	m/z	Gene names
20140827_EXQ00_FaHo_SA_EAF3_(	8092	FTMS; HCD	165.3	1125.52	TY1B-LR3;TY1A-PL;TY1A-LR2;TY1A-ER1;TY1A-DR6;TY1B-OL;TY1B-LR4;TY1B-LR2;TY1B-PL;TY1B-ER1;TY1B-PR3;TY1A-PR1;TY1A-A;TY1A-DR4;TY1B-H;TY1B-GR2;TY1B-MR2;TY1B-ER2;TY1B-OR;TY1B-BR;TY1B-DR1;TY1B-NL

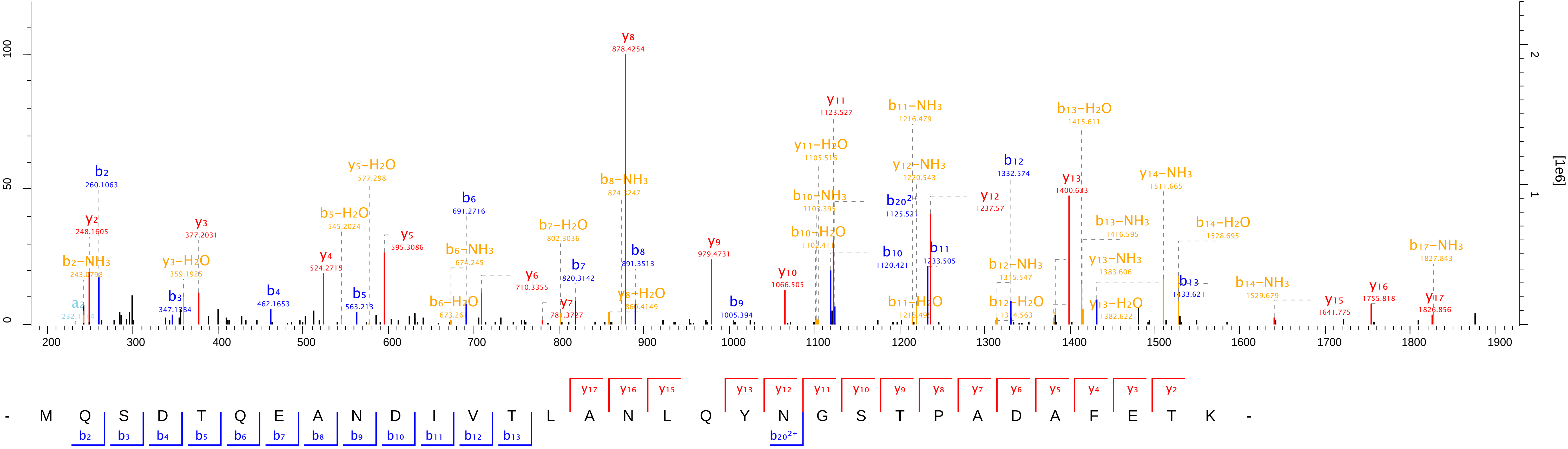

Raw file	Scan	Method	Score	m/z	Gene names
20140827_EXQ00_FaHo_SA_ECM5_01	6608	FTMS; HCD	69.34	625.83	PDR5;PDR10

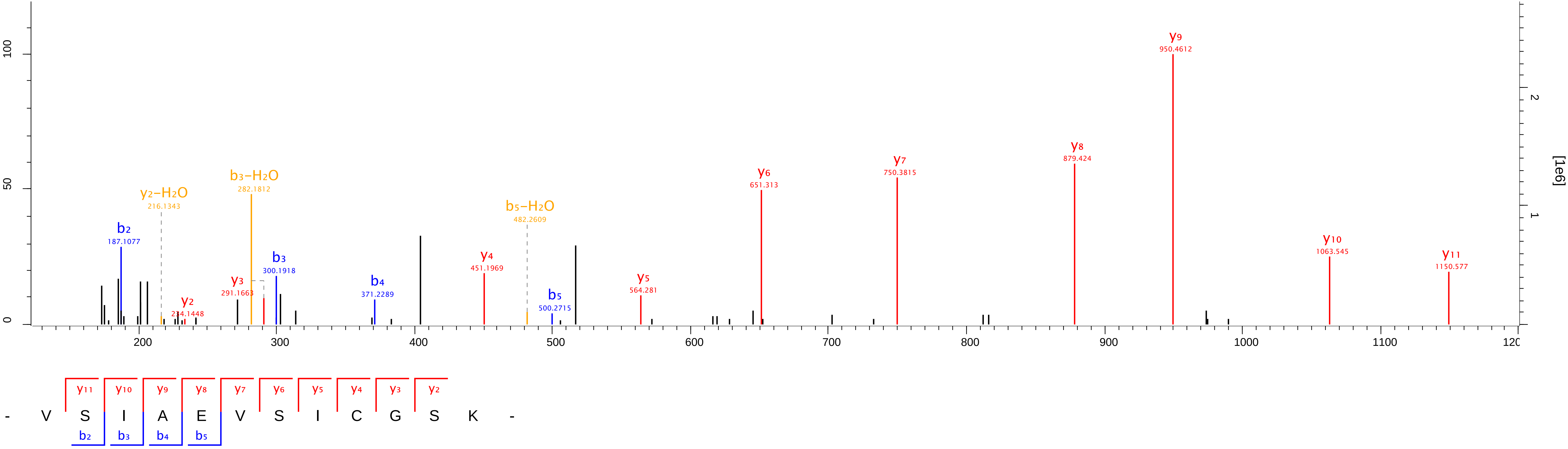

Raw file	Scan	Method	Score	m/z	Gene names
20140827_EXQ00_FaHo_SA_ECM5_01	8895	FTMS; HCD	96.46	724.89	GRX1

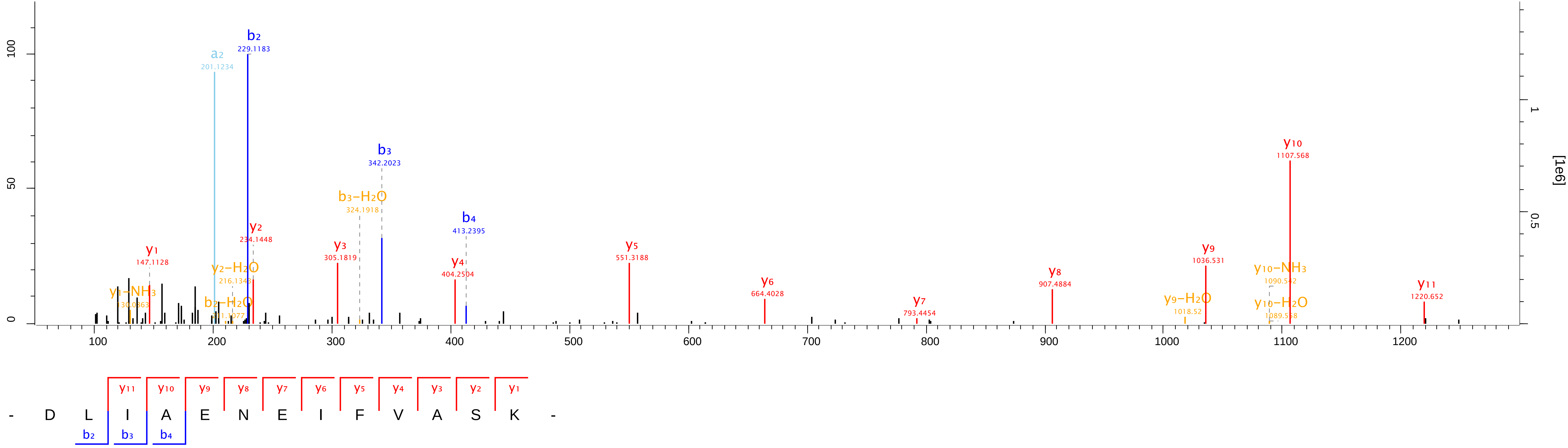

Raw file	Scan	Method	Score	m/z	Gene names
20140827_EXQ00_FaHo_SA_ECM5_02	2683	FTMS; HCD	105.46	549.29	TSC13

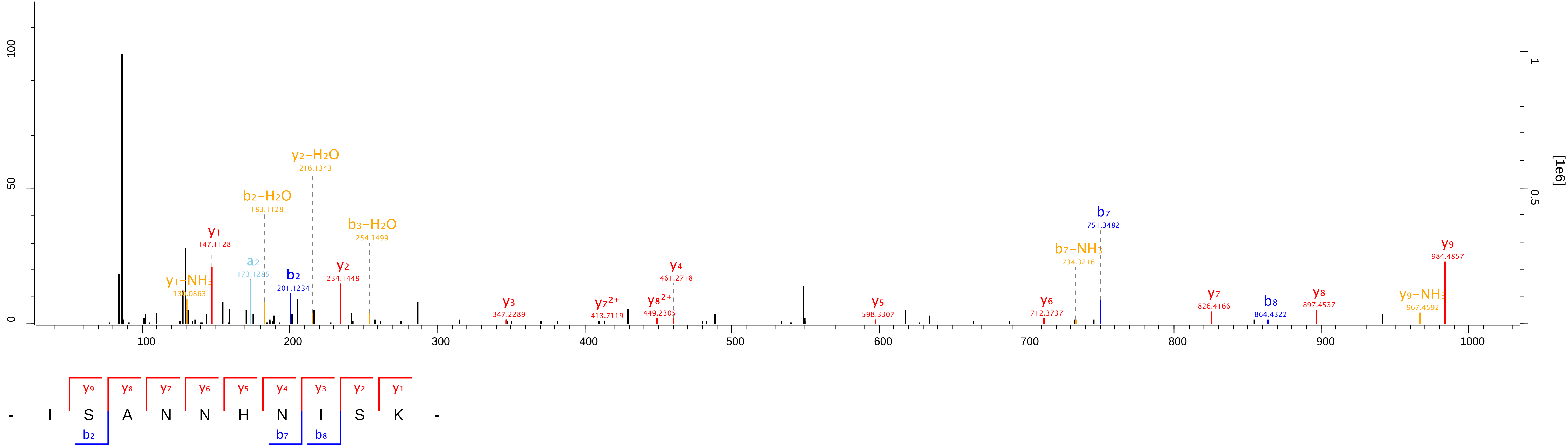

Raw file	Scan	Method	Score	m/z	Gene names
20140827_EXQ00_FaHo_SA_ECM5_02	3206	FTMS; HCD	141.73	482.22	RPL4B;RPL4A

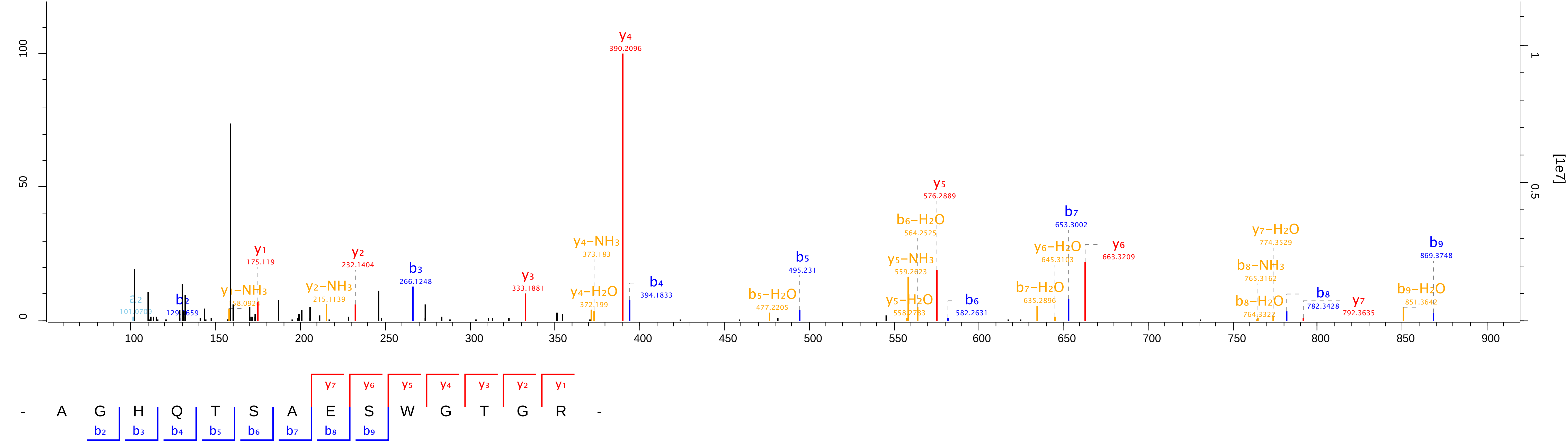

Raw file	Scan	Method	Score	m/z	Gene names
20140827_EXQ00_FaHo_SA_ECM5_02	7828	FTMS; HCD	0.71	701.6	MPA43

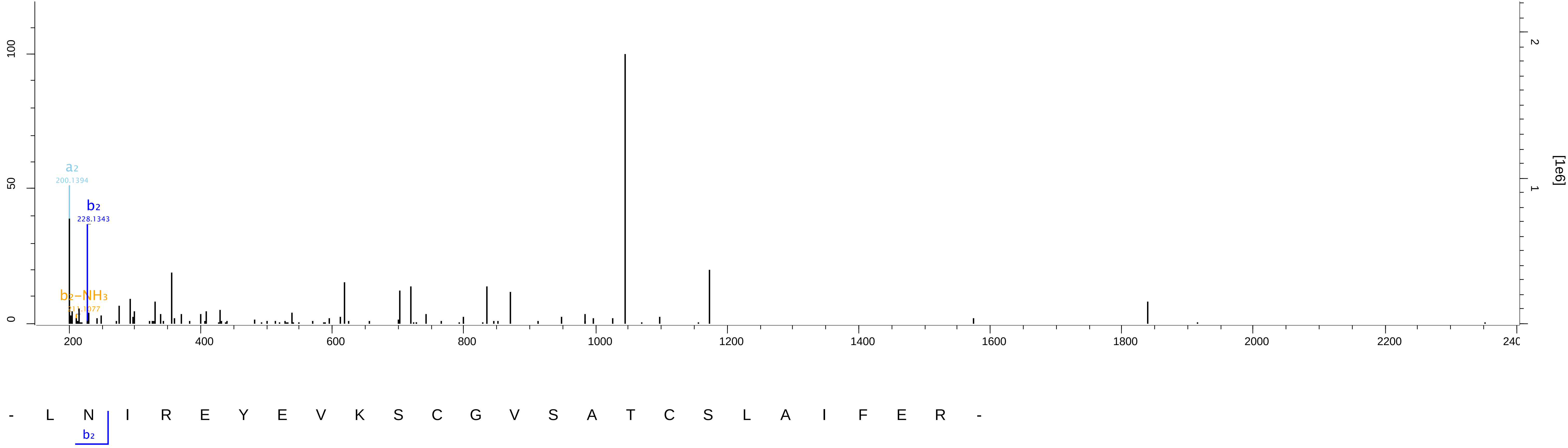

Raw file	Scan	Method	Score	m/z	Gene names
20140827_EXQ00_FaHo_SA_ECM5_03	3094	FTMS; HCD	57.28	414.75	RPL24A;RPL24B

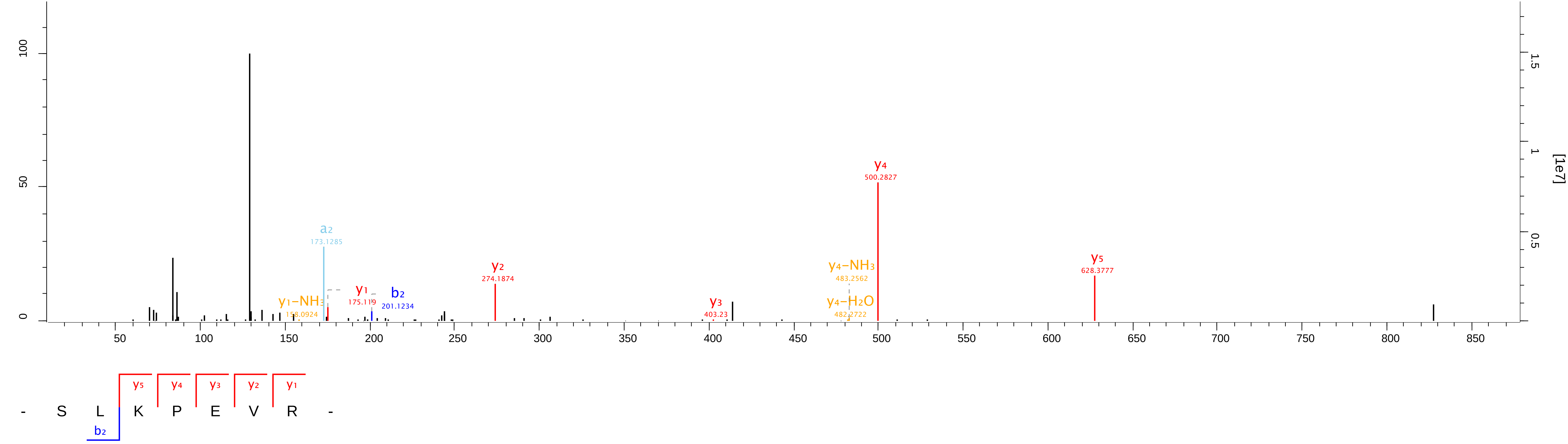

Raw file	Scan	Method	Score	m/z	Gene names
20140827_EXQ00_FaHo_SA_ECM5_03	3380	FTMS; HCD	105.52	486.76	RPL36B

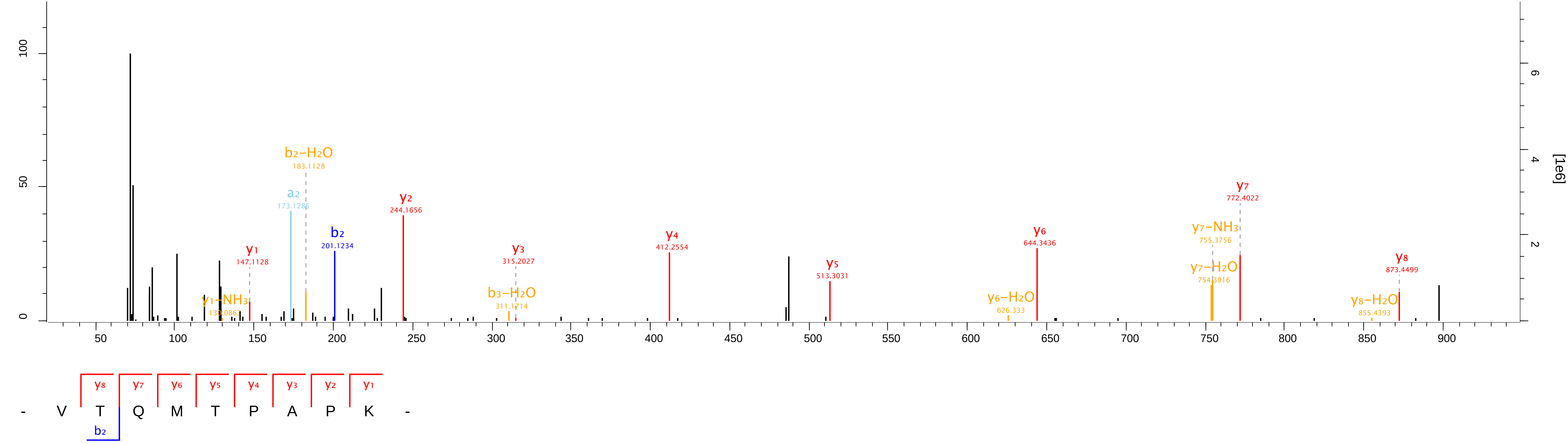

Raw file	Scan	Method	Score	m/z	Gene names
20140827_EXQ00_FaHo_SA_ECM5_(	5387	FTMS; HCD	142.64	495.77	TY1B-LR3;TY1A-PL;TY1A-LR2;TY1A-ER1;TY1A-DR6;TY1B-OL;TY1B-LR4;TY1B-LR2;TY1B-PL;TY1B-ER1;TY1B-PR3;TY1A-PR1;TY1A-A;TY1A-DR4;TY1B-H;TY1B-GR2;TY1B-MR2;TY1B-ER2;TY1B-OR;TY1B-BR;TY1B-DR1;TY1B-NL

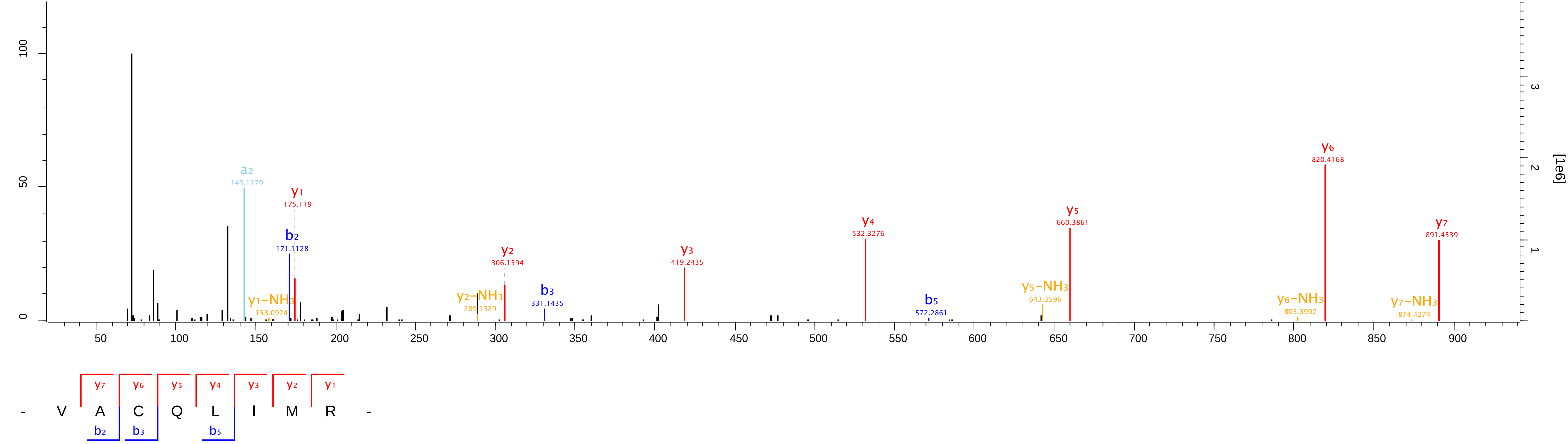

Raw file	Scan	Method	Score	m/z	Gene names
20140827_EXQ00_FaHo_SA_ECM5_03	7295	FTMS; HCD	92.04	775.38	PGA2

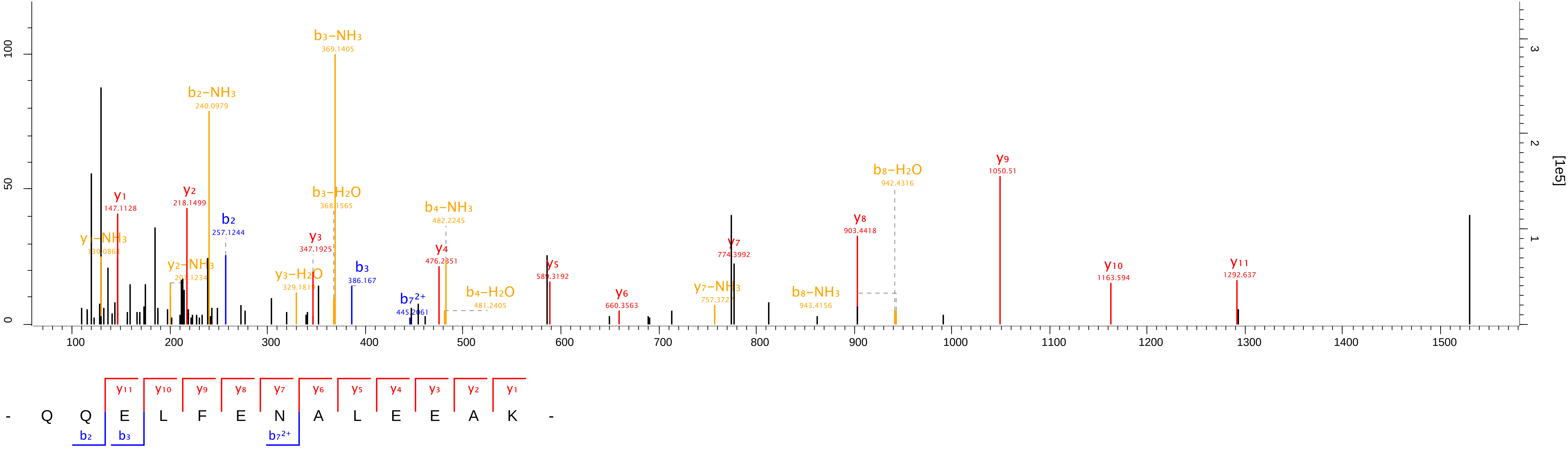

Raw file	Scan	Method	Score	m/z	Gene names
20140827_EXQ00_FaHo_SA_ECM5_03	7349	FTMS; HCD	74.08	593.33	NCE102

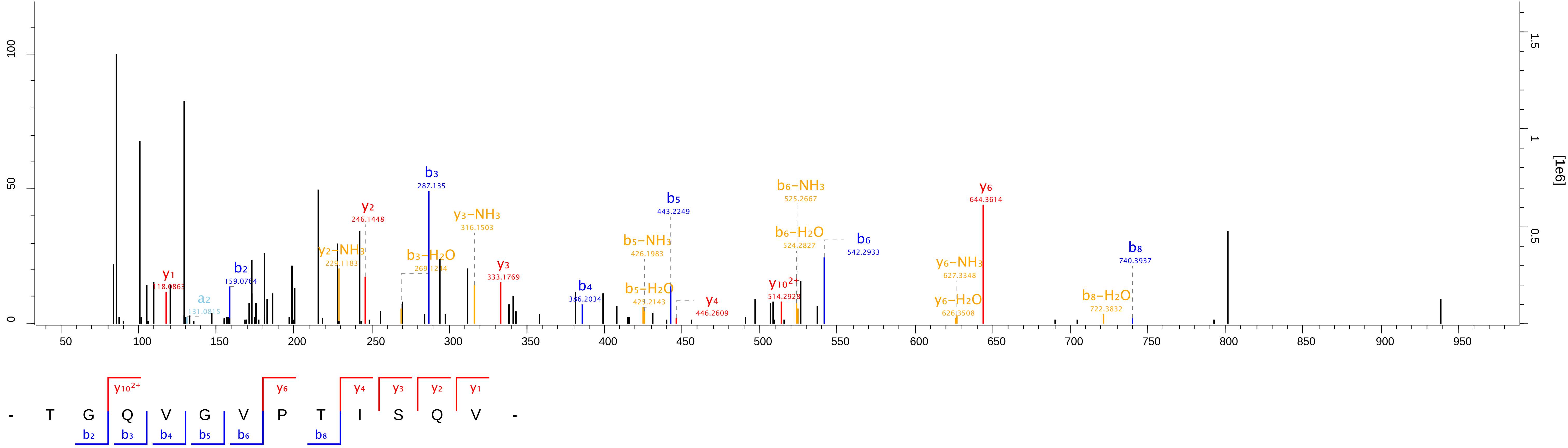

Raw file	Scan	Method	Score	m/z	Gene names
20140827_EXQ00_FaHo_SA_ECM5_03	7535	FTMS; HCD	51.77	580.31	NMA1

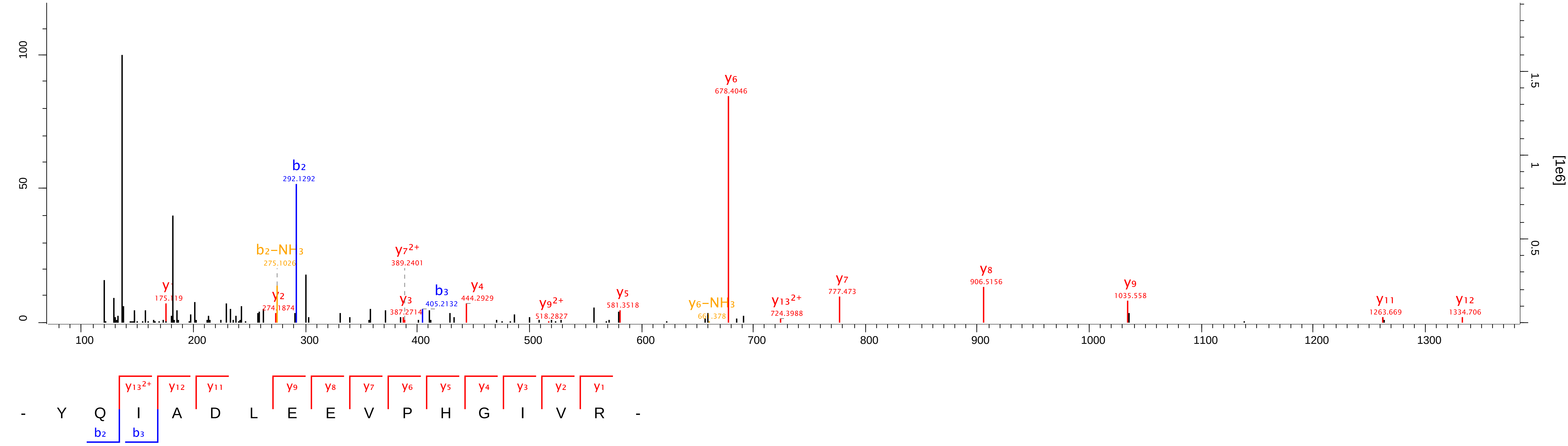

Raw file	Scan	Method	Score	m/z	Gene names
20140827_EXQ00_FaHo_SA_ECM5_03	8316	FTMS; HCD	79.87	715.9	DCW1

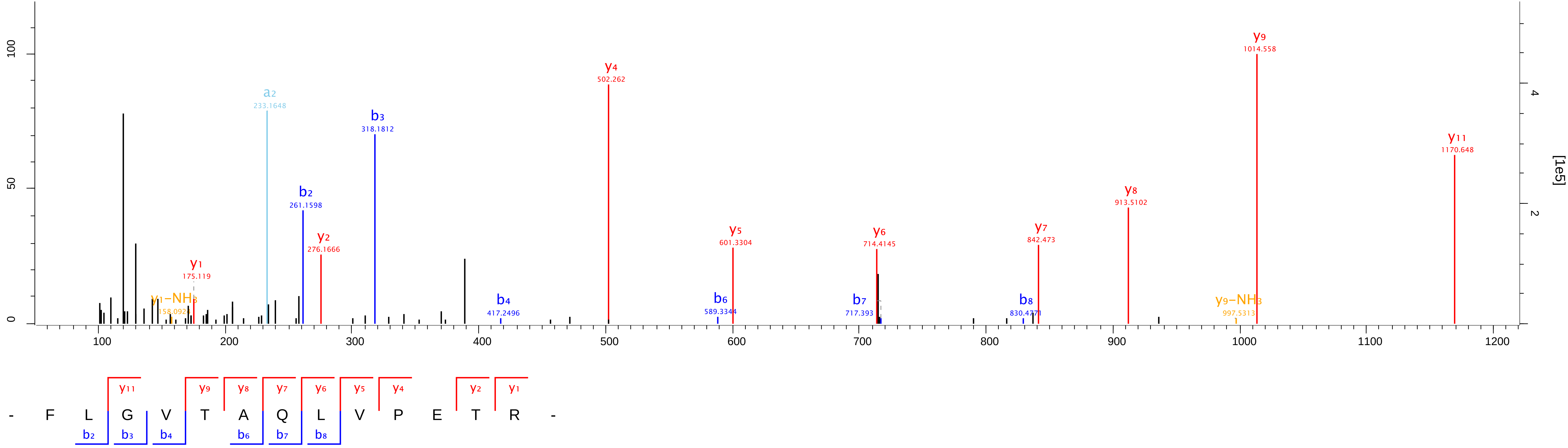

Raw file	Scan	Method	Score	m/z	Gene names
20140827_EXQ00_FaHo_SA_GCN5_01	3483	FTMS; HCD	115.92	635.34	RPS0A;RPS0B

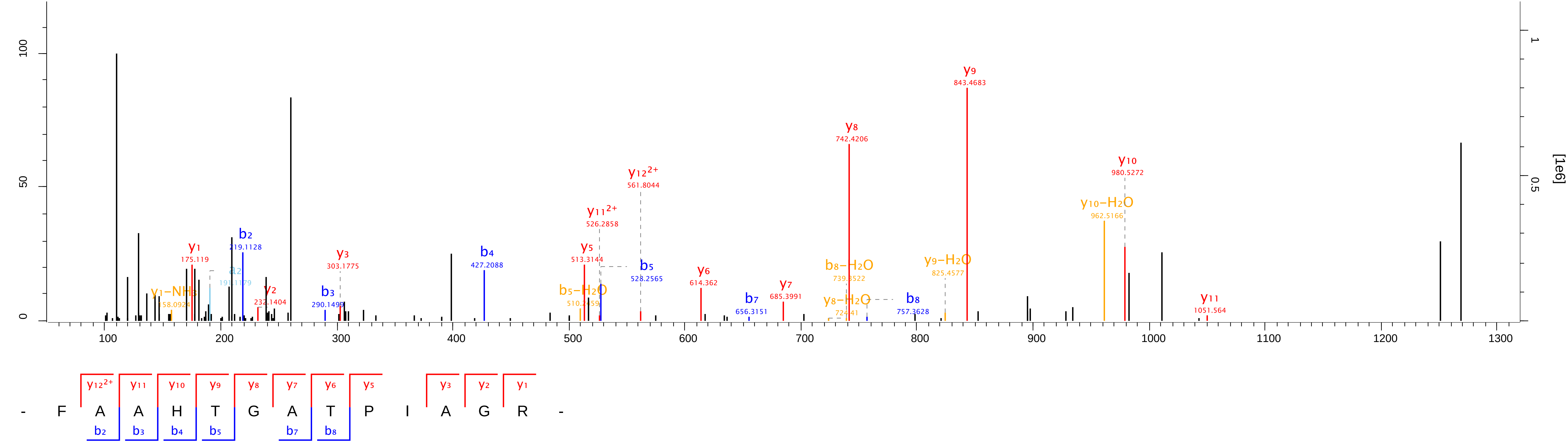

Raw file	Scan	Method	Score	m/z	Gene names
20140827_EXQ00_FaHo_SA_GCN5_02	4159	FTMS; HCD	54.2	593.3	ACT1

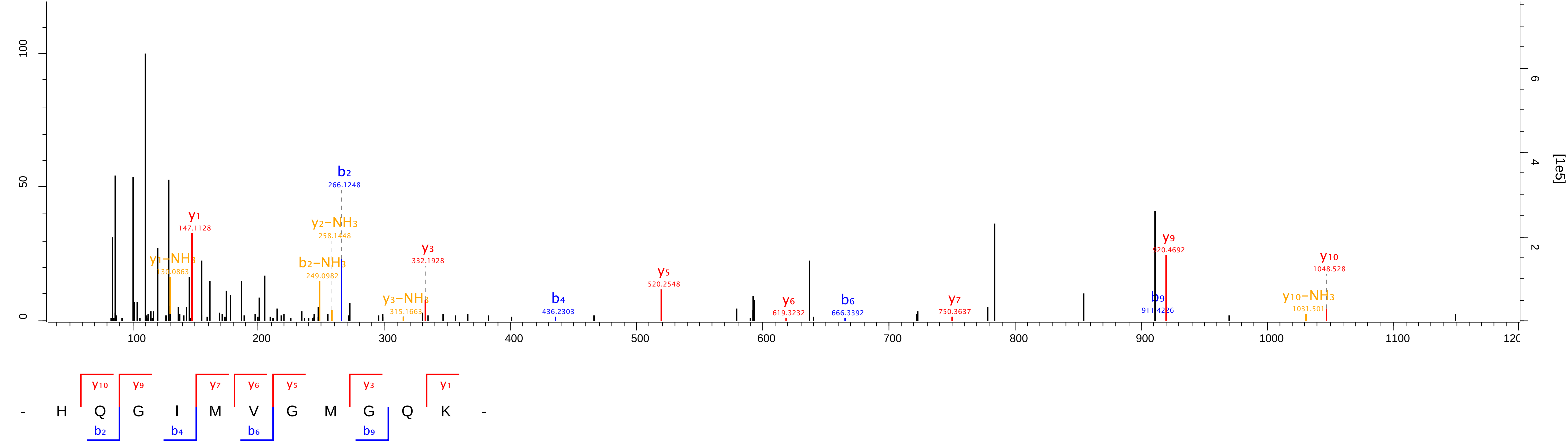

Raw file	Scan	Method	Score	m/z	Gene names
20140827_EXQ00_FaHo_SA_GCN5_03	3285	FTMS; HCD	47.71	383.95	HPC2

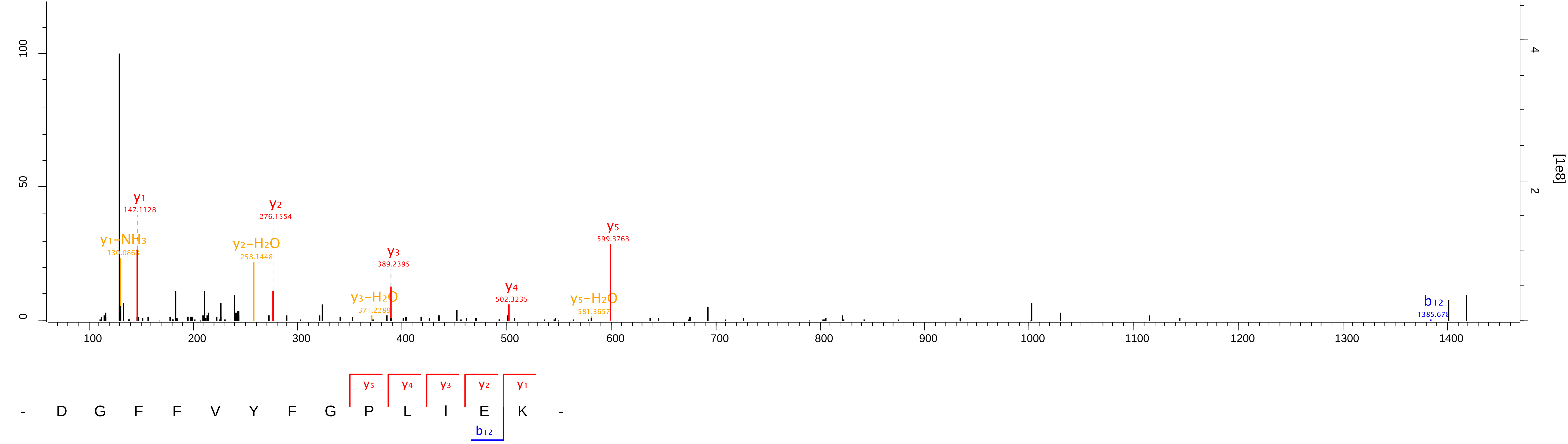

Raw file	Scan	Method	Score	m/z	Gene names
20140827_EXQ00_FaHo_SA_GFP_03	2691	FTMS; HCD	95.36	320.5	QCR8

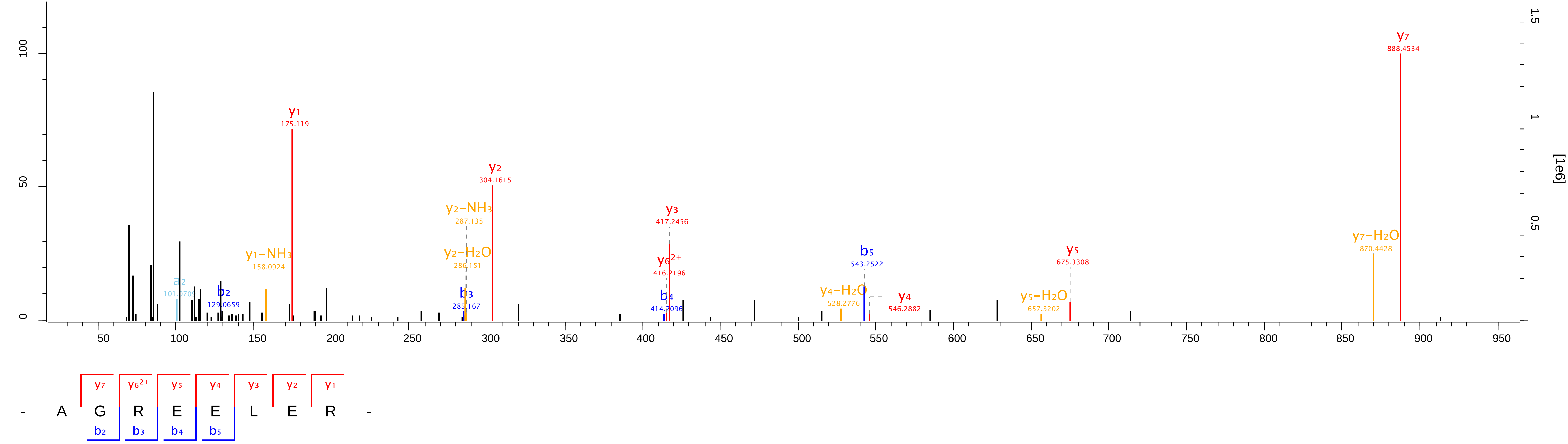

Raw file	Scan	Method	Score	m/z	Gene names
20140827_EXQ00_FaHo_SA_GFP_03	4324	FTMS; HCD	104.05	409.23	RPL21A

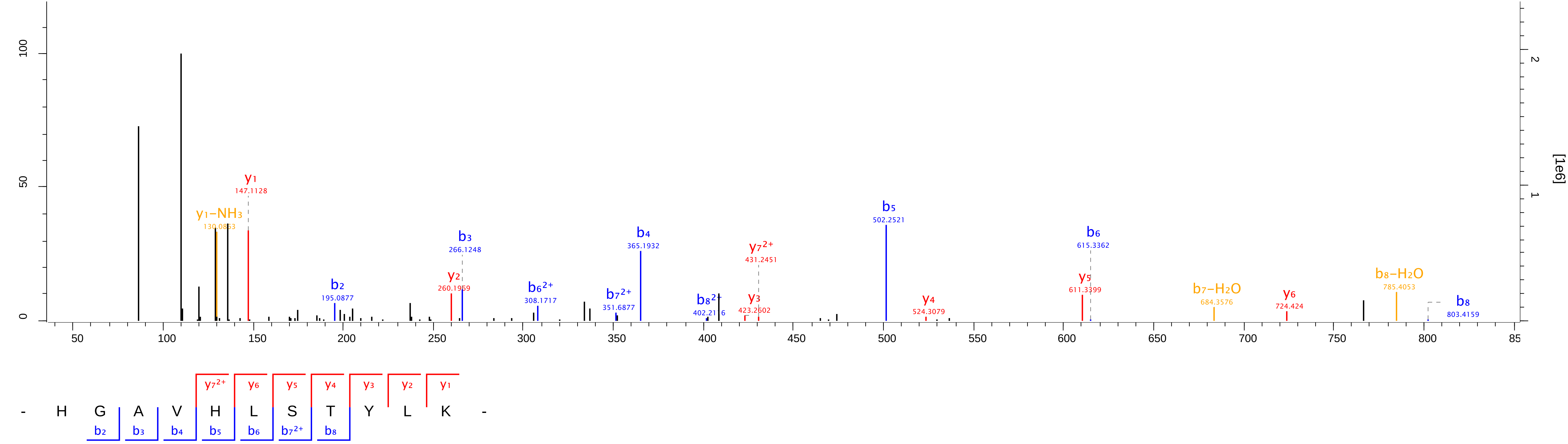

Raw file	Scan	Method	Score	m/z	Gene names
20140827_EXQ00_FaHo_SA_GFP_03	7702	FTMS; HCD	147.67	862.44	RPS14B;RPS14A

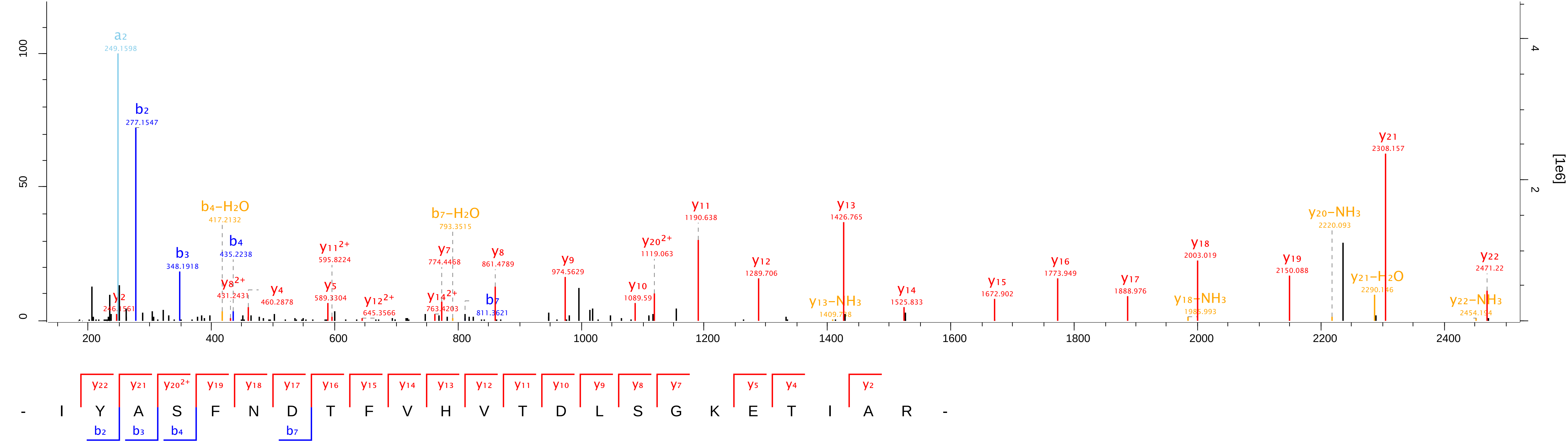

Raw file	Scan	Method	Score	m/z	Gene names
20140827_EXQ00_FaHo_SA_HDA1_02	5650	FTMS; HCD	52.07	613.82	RPL7A;RPL7B

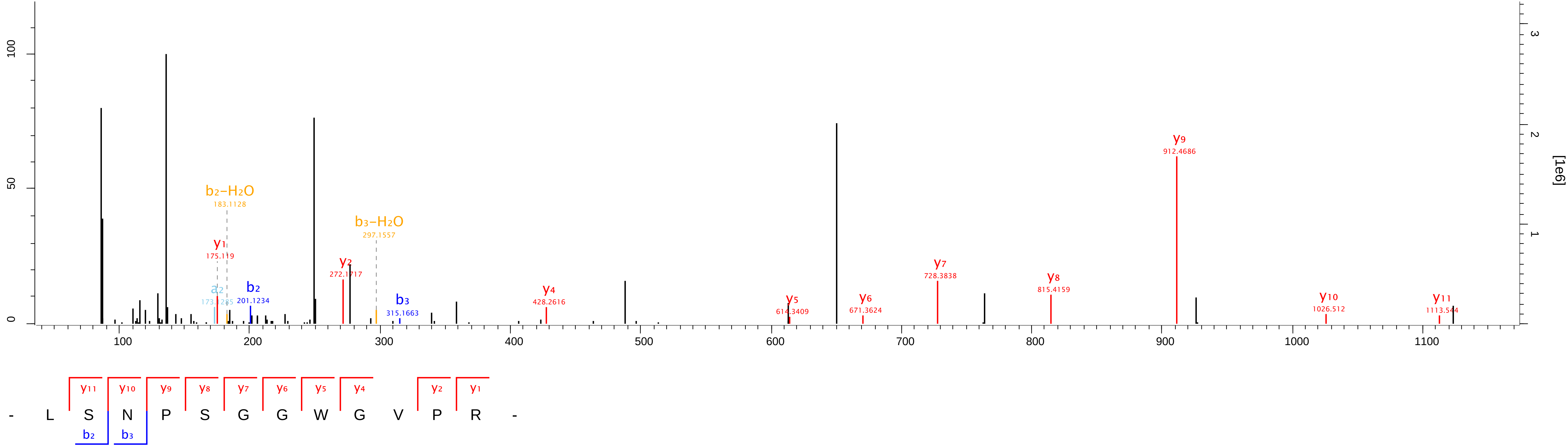

Raw file	Scan	Method	Score	m/z	Gene names
20140827_EXQ00_FaHo_SA_HDA1_02	5895	FTMS; HCD	95.53	457.26	VTC1

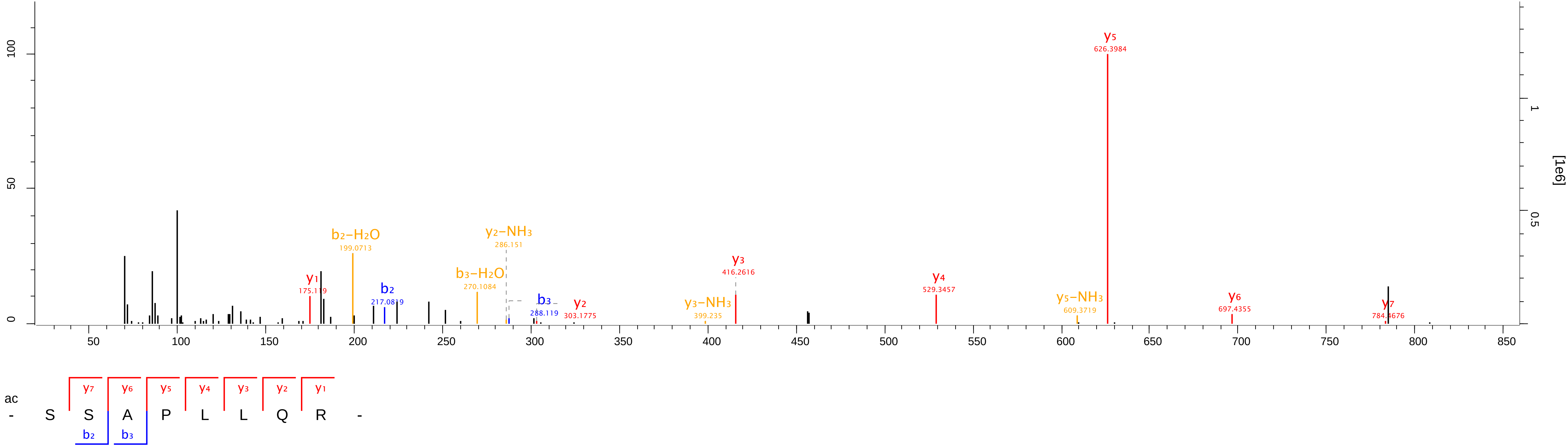

Raw file	Scan	Method	Score	m/z	Gene names
20140827_EXQ00_FaHo_SA_HDA1_02	6389	FTMS; HCD	79.69	602.8	RPS14B;RPS14A

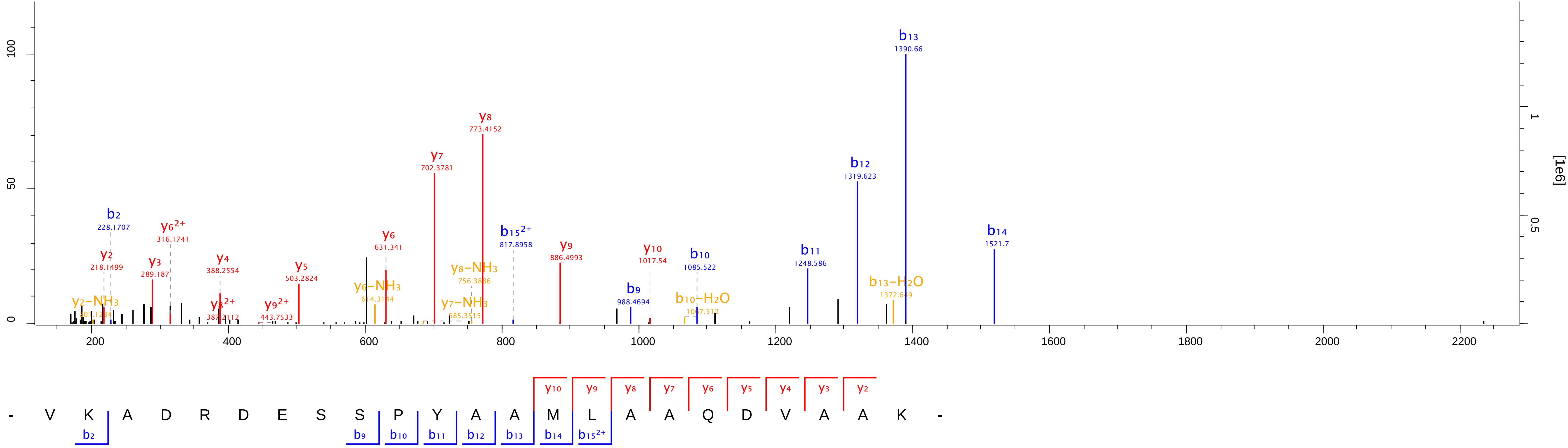

Raw file	Scan	Method	Score	m/z	Gene names
20140827_EXQ00_FaHo_SA_HDA1_02	8211	FTMS; HCD	141.13	672.34	RPS14B;RPS14A

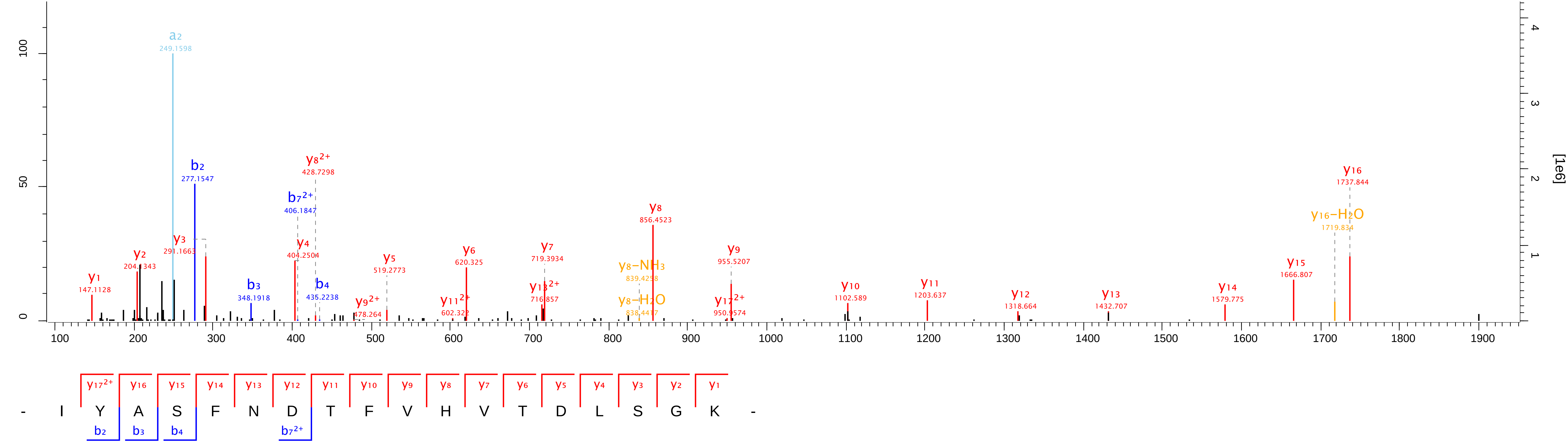

Raw file	Scan	Method	Score	m/z	Gene names
20140827_EXQ00_FaHo_SA_HDA1_03	7211	FTMS; HCD	217.48	726.39	RPL9A;RPL9B

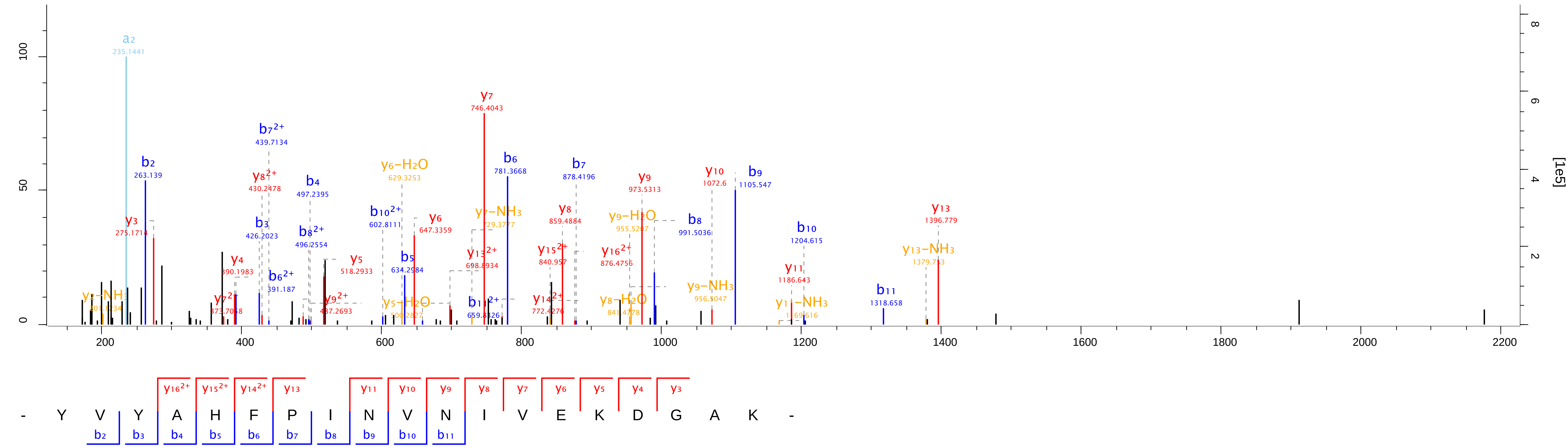

Raw file	Scan	Method	Score	m/z	Gene names
20140827_EXQ00_FaHo_SA_HDA2_01	3032	FTMS; HCD	158.79	487.75	RPL7A

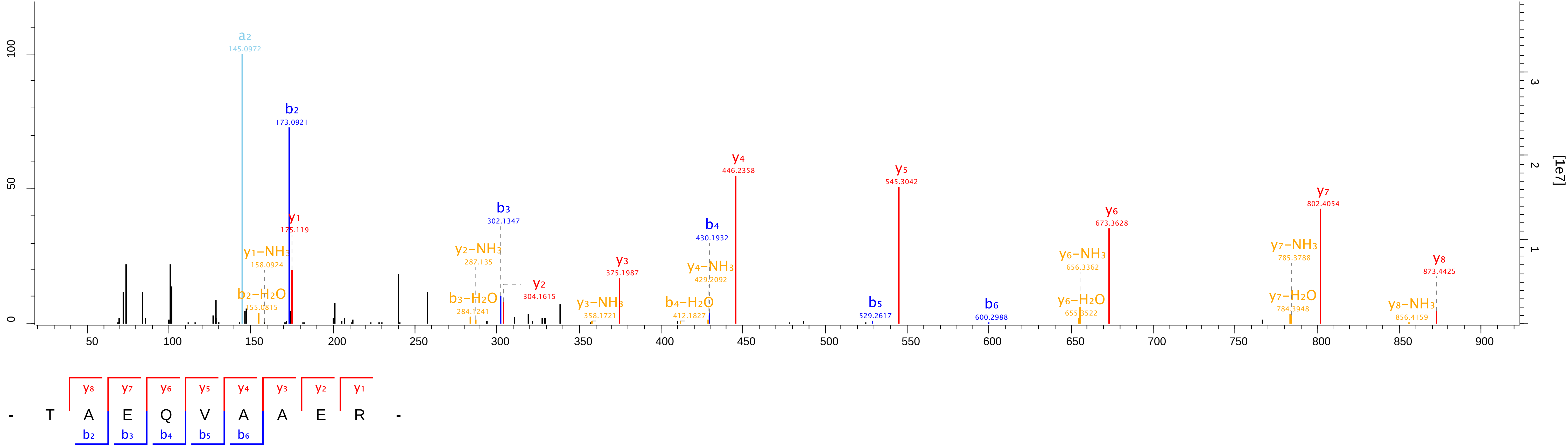

Raw file	Scan	Method	Score	m/z	Gene names
20140827_EXQ00_FaHo_SA_HDA2_03	4079	FTMS; HCD	81.02	613.79	CDC42

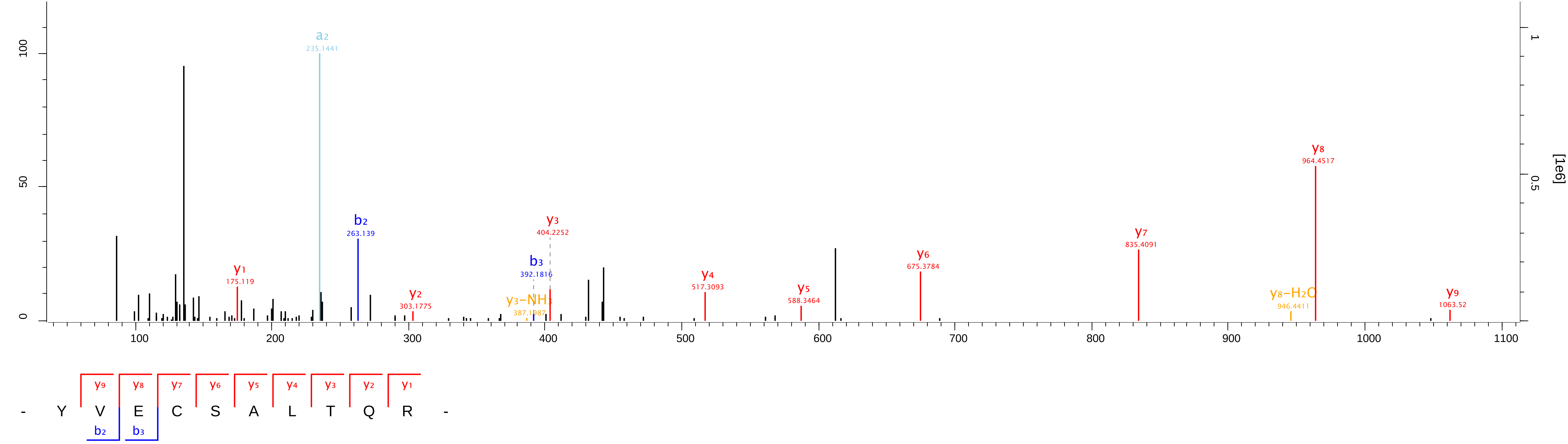

Raw file	Scan	Method	Score	m/z	Gene names
20140827_EXQ00_FaHo_SA_HDA2_03	7883	FTMS; HCD	61.34	749.44	CRM1

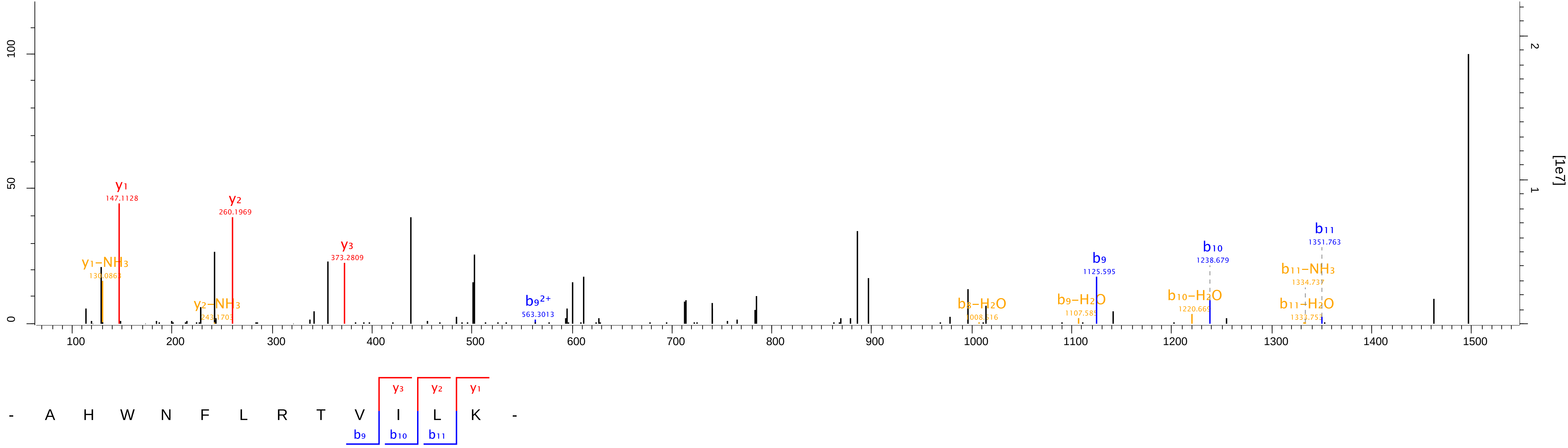

Raw file	Scan	Method	Score	m/z	Gene names
20140827_EXQ00_FaHo_SA_HDA3_03	5343	FTMS; HCD	50.09	579.62	YKT6

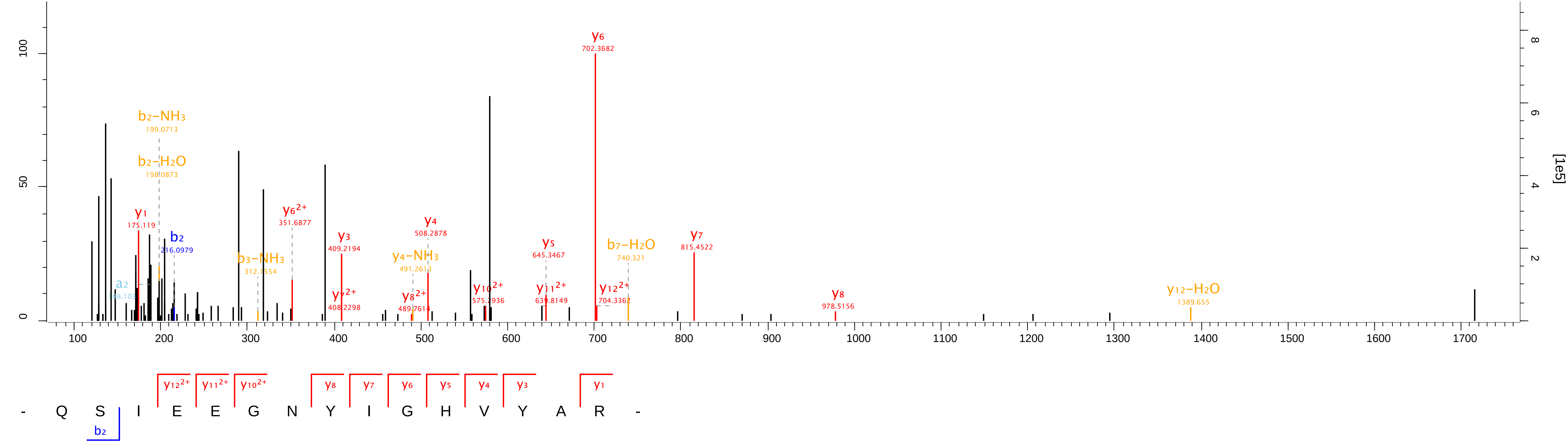

Raw file	Scan	Method	Score	m/z	Gene names
20140827_EXQ00_FaHo_SA_HDA3_03	6008	FTMS; HCD	85.16	466.75	TRX2;TRX1

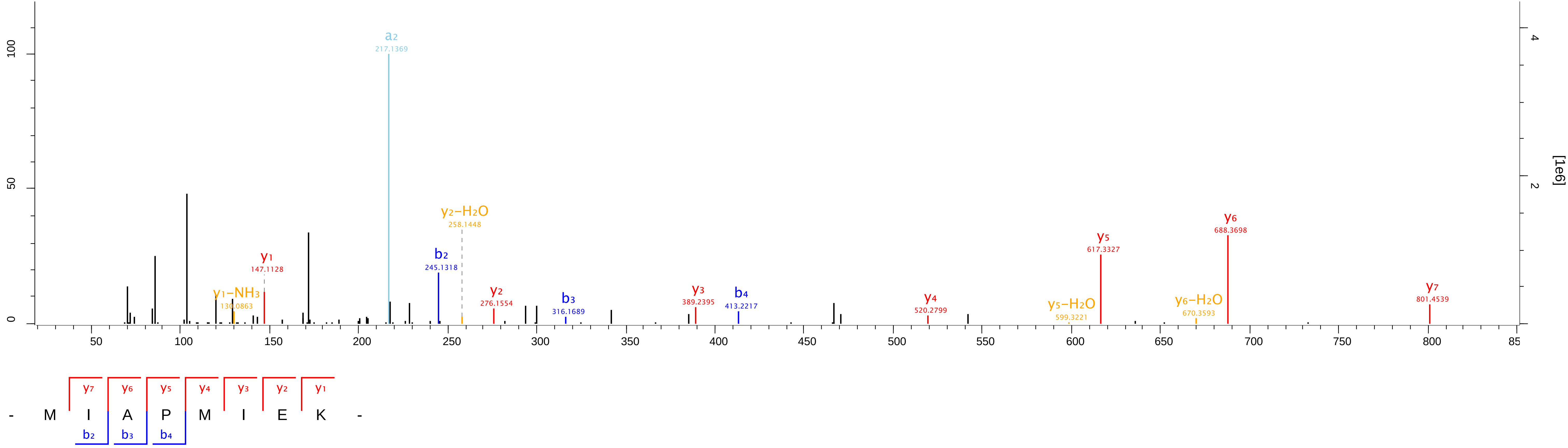

Raw file	Scan	Method	Score	m/z	Gene names
20140827_EXQ00_FaHo_SA_HDA3_03	7547	FTMS; HCD	68.54	594.35	ARG4

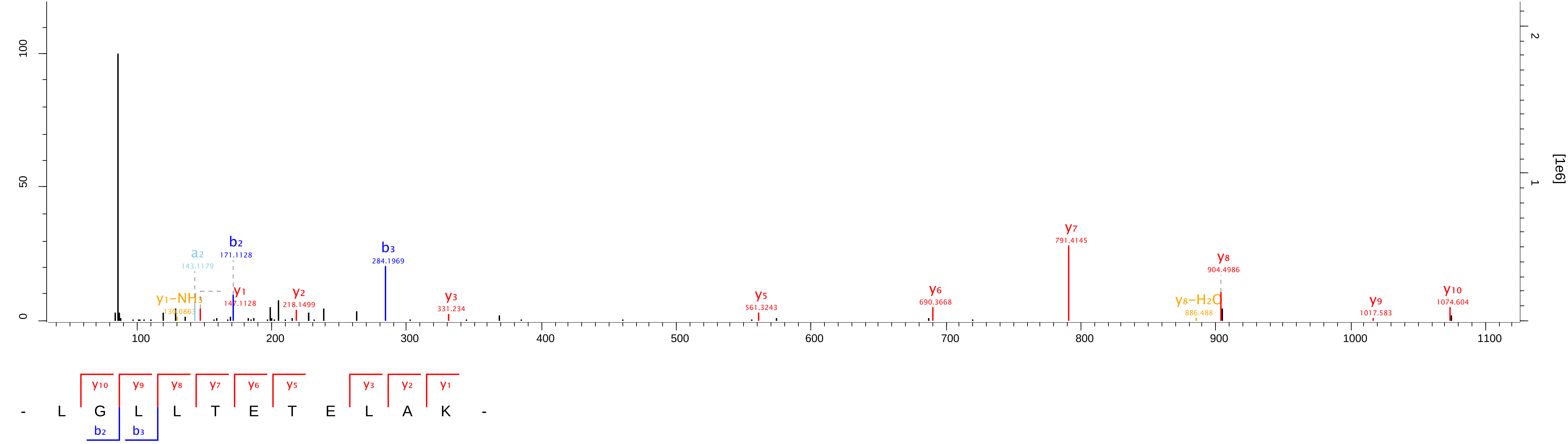

Raw file	Scan	Method	Score	m/z
20140827_EXQ00_FaHo_SA_HDA3_03	7592	FTMS; HCD	112.42	781.4

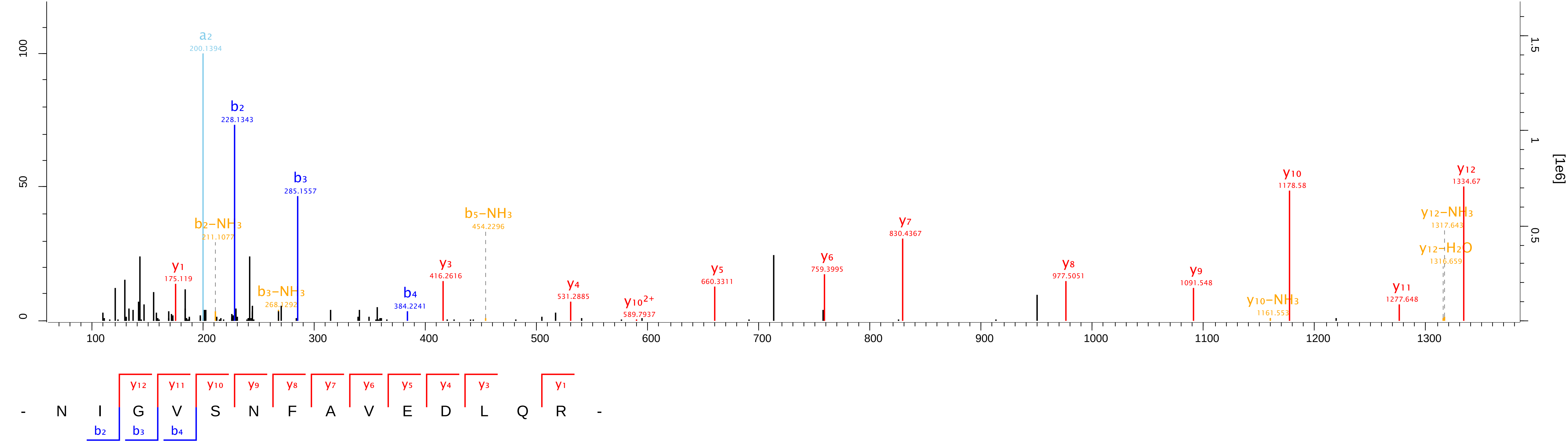

Raw file	Scan	Method	Score	m/z	Gene names
20140827_EXQ00_FaHo_SA_HIF1_01	5817	FTMS; HCD	123.67	689.85	HNT1

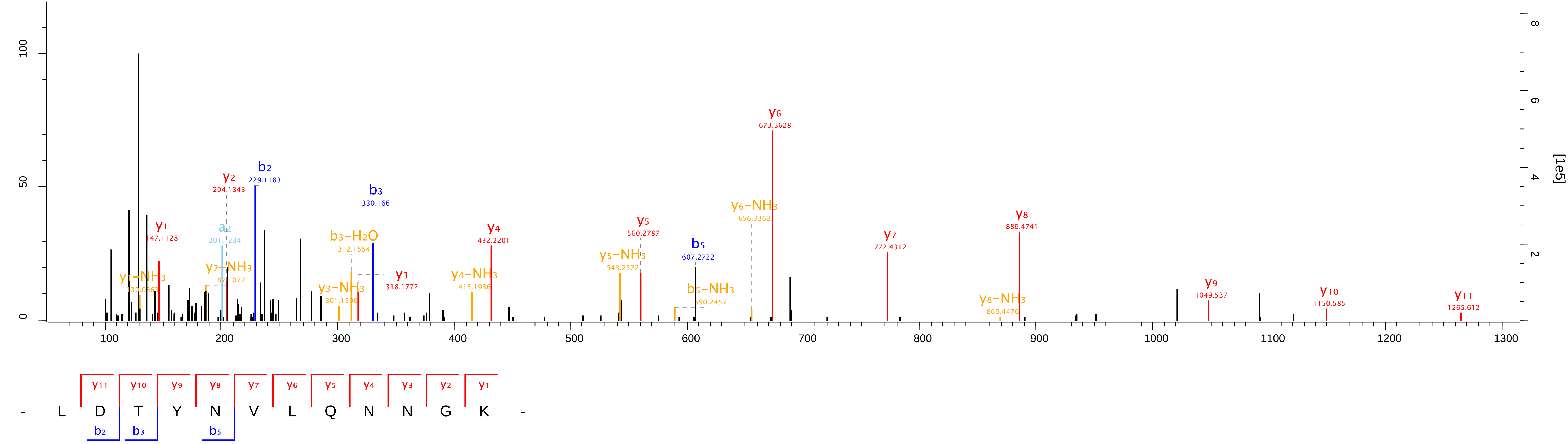

Raw file	Scan	Method	Score	m/z	Gene names
20140827_EXQ00_FaHo_SA_HIF1_01	6493	FTMS; HCD	59.67	800.42	SAM4

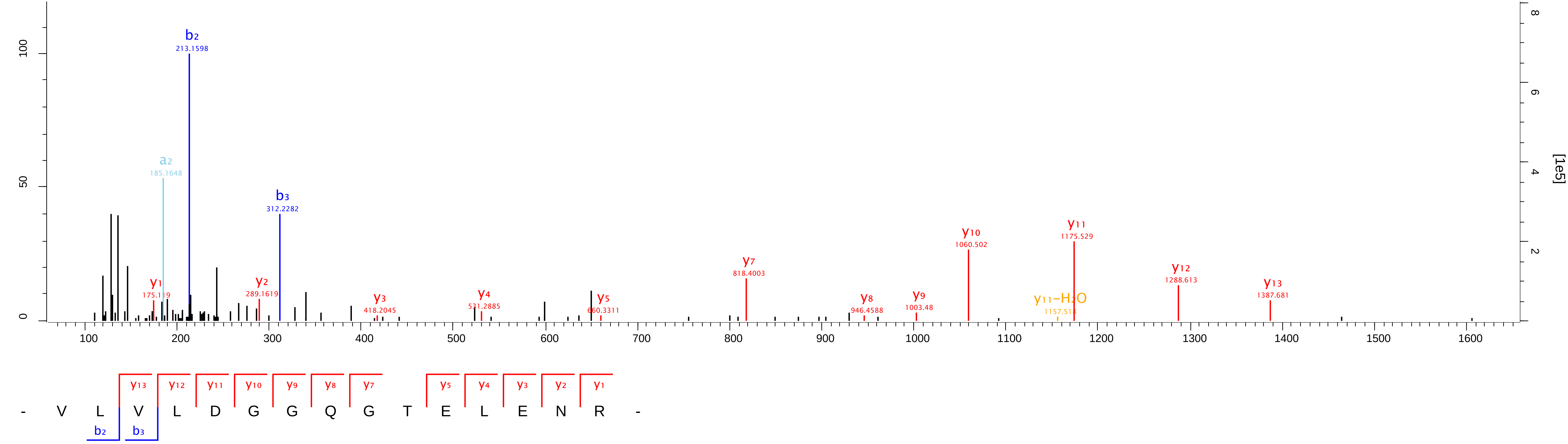

Raw file	Scan	Method	Score	m/z	Gene names
20140827_EXQ00_FaHo_SA_HIF1_01	7248	FTMS; HCD	97.73	537.31	ARF1;ARF2

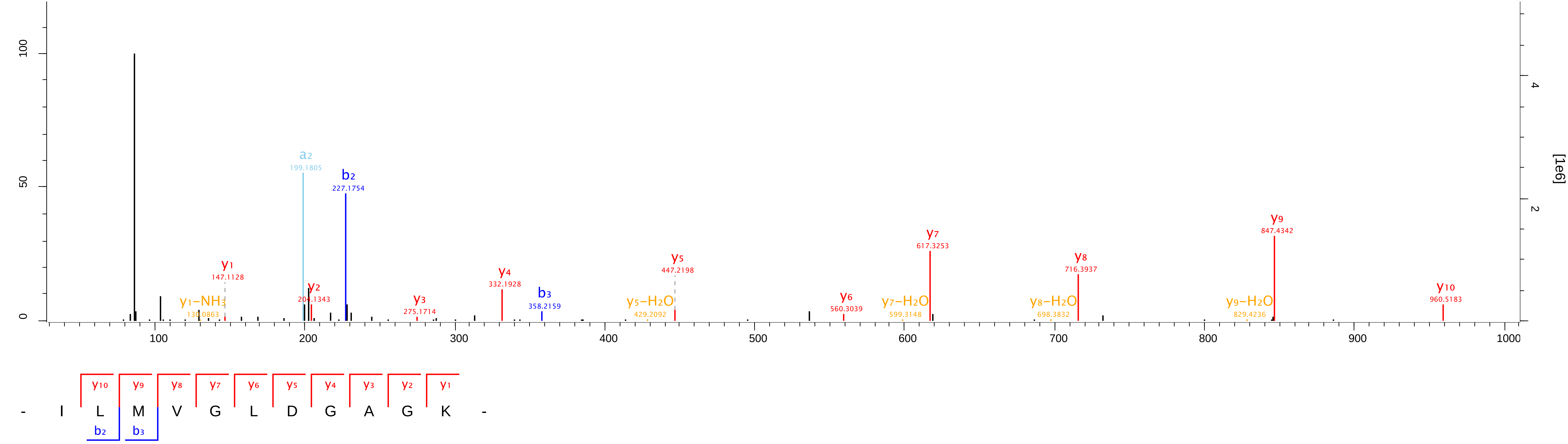

Raw file	Scan	Method	Score	m/z	Gene names
20140827_EXQ00_FaHo_SA_HIF1_02	4854	FTMS; HCD	133.23	490.26	ACB1

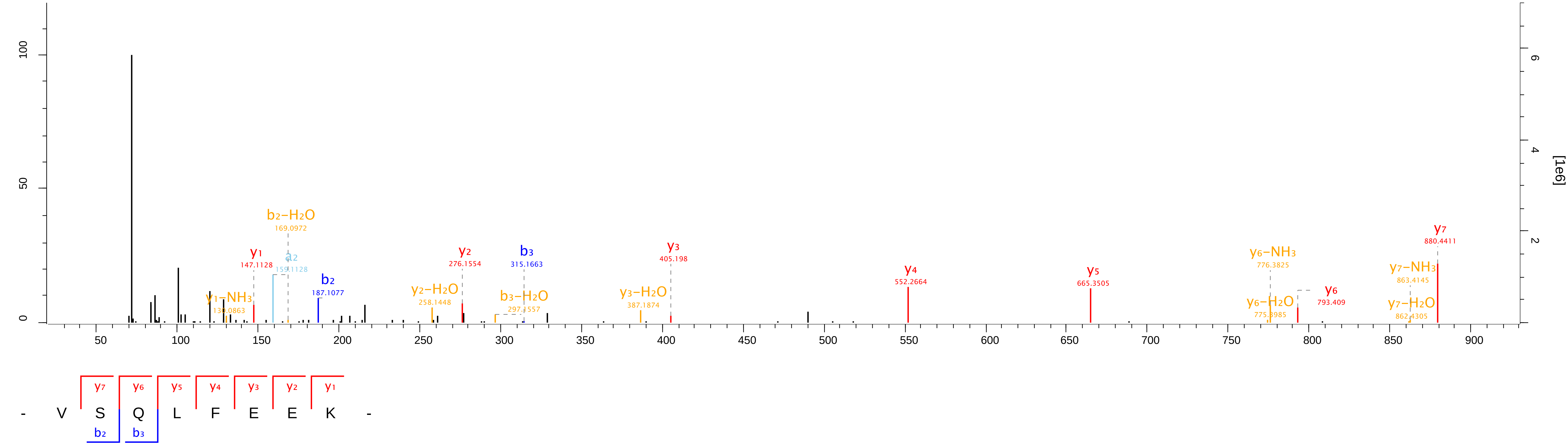

Raw file	Scan	Method	Score	m/z	Gene names
20140827_EXQ00_FaHo_SA_HIF1_02	8389	FTMS; HCD	55.75	883	RPS0A;RPS0B

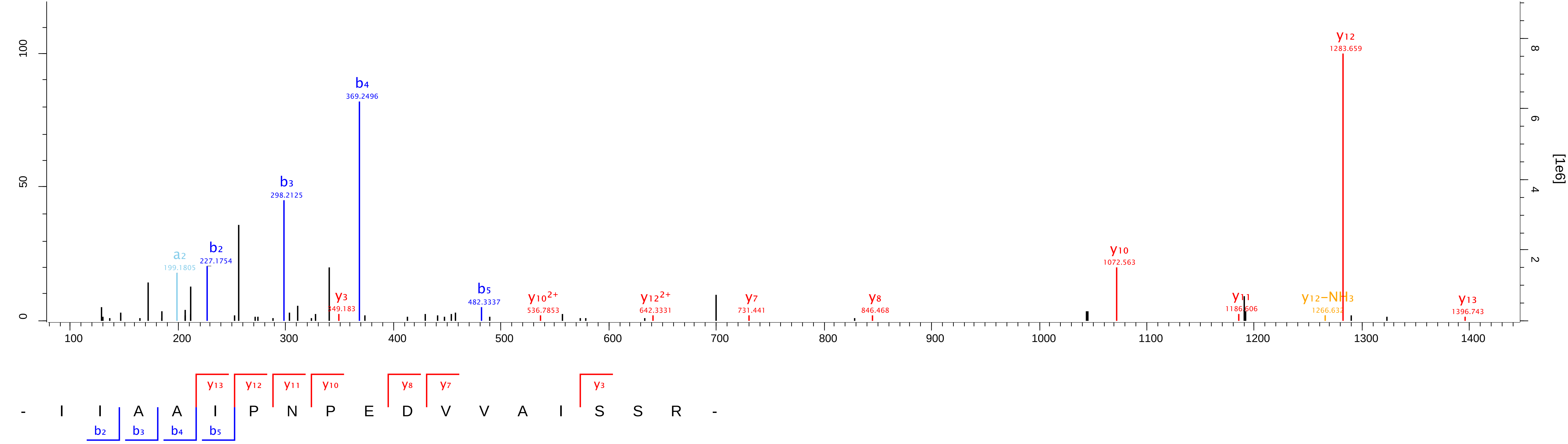

Raw file	Scan	Method	Score	m/z	Gene names
20140827_EXQ00_FaHo_SA_IOC4_(	6157	FTMS; HCD	124.83	479.79	TY2B-B;TY2A-OR1;TY2A-OR2;TY2A-B;TY2A-DR2;TY2A-LR2;TY2A-GR2;TY2A-F;TY2B-GR1;TY2B-OR2;TY2B-DR2;TY2B-GR2;TY2B-F;TY2B-LR2;TY2A-LR1;TY2A-DR1;TY2B-C;TY2A-C;TY2B-OR1;TY2B-DR1;TY2B-DR3;TY2A-DR3

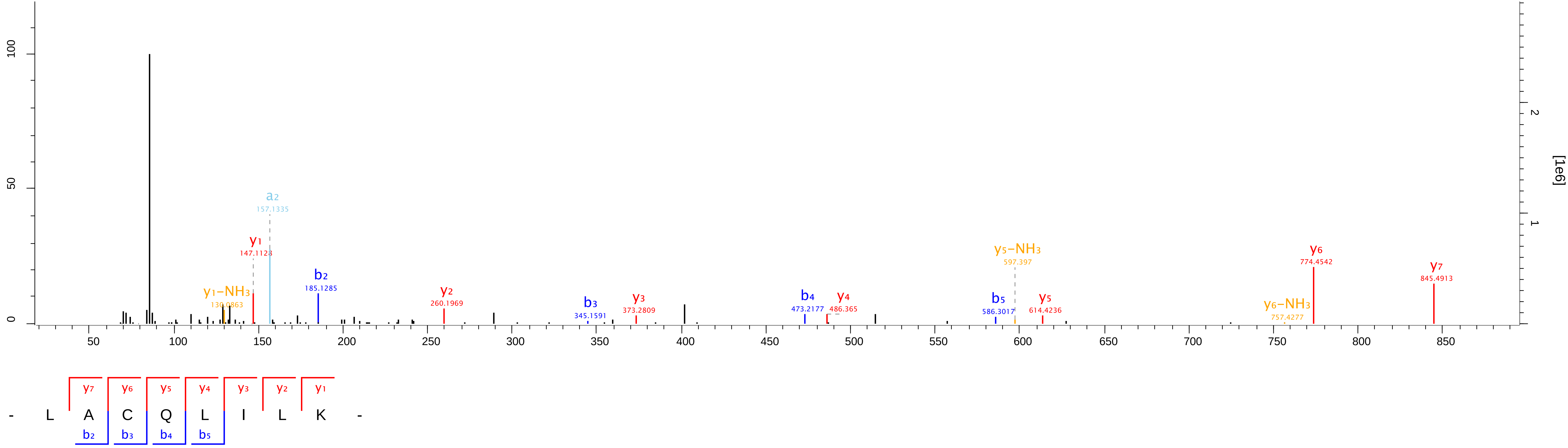

Raw file	Scan	Method	Score	m/z	Gene names
20140827_EXQ00_FaHo_SA_IOC4_02	8333	FTMS; HCD	128.98	913.77	TRX2

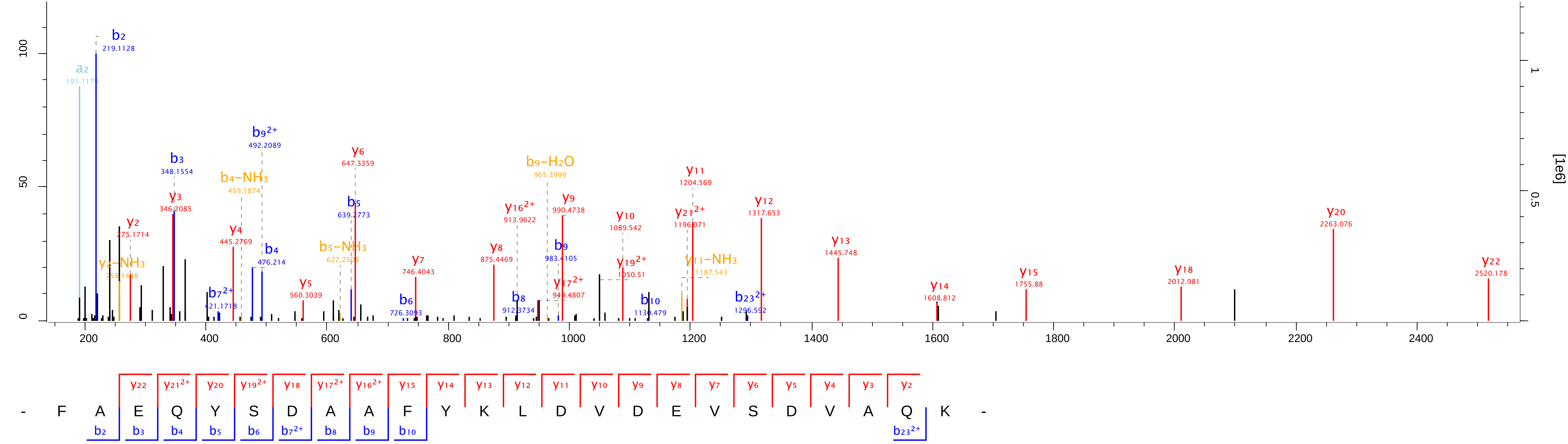

Raw file	Scan	Method	Score	m/z	Gene names
20140827_EXQ00_FaHo_SA_IOC4_03	8458	FTMS; HCD	29.74	656.84	MRP2

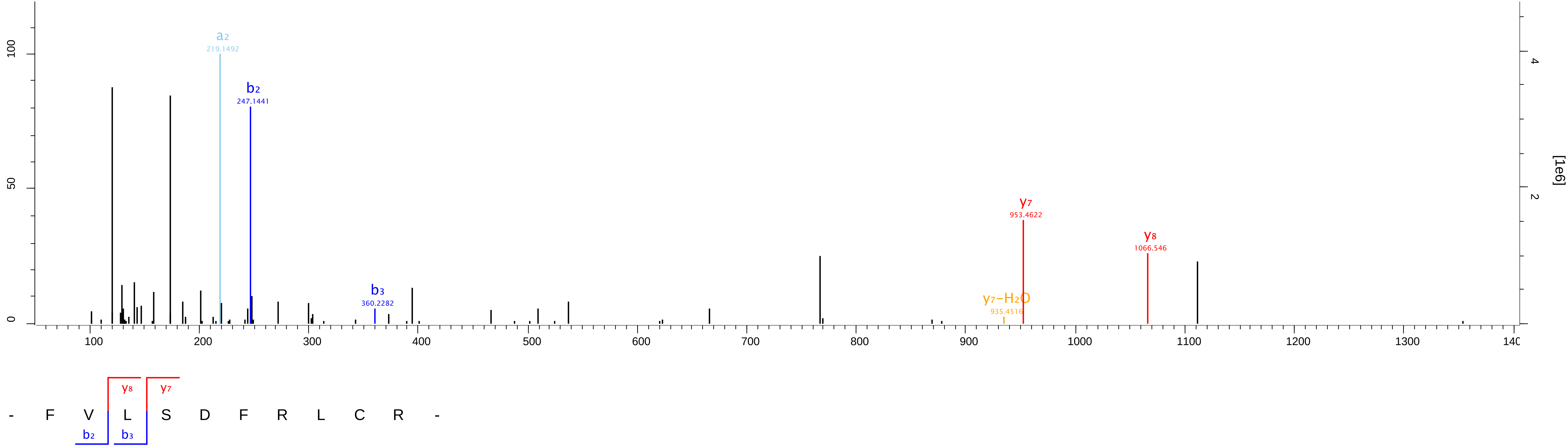

Raw file	Scan	Method	Score	m/z	Gene names
20140827_EXQ00_FaHo_SA_parental_01	4231	FTMS; HCD	98.05	386.24	RPL7A;RPL7B

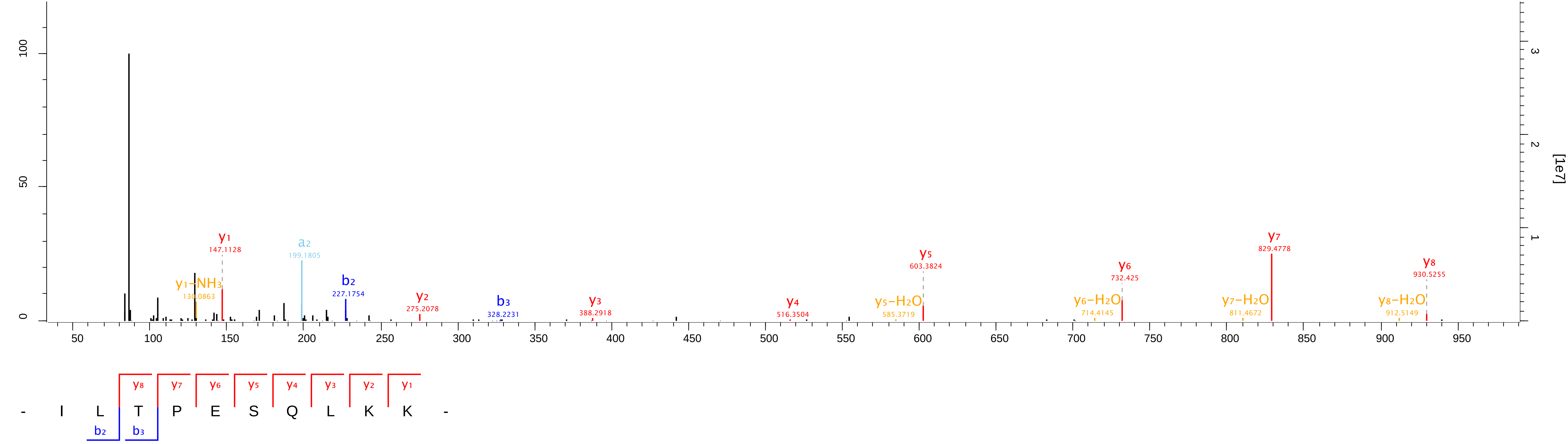

Raw file	Scan	Method	Score	m/z	Gene names
20140827_EXQ00_FaHo_SA_parental_01	4836	FTMS; HCD	68.54	659.78	YPR148C

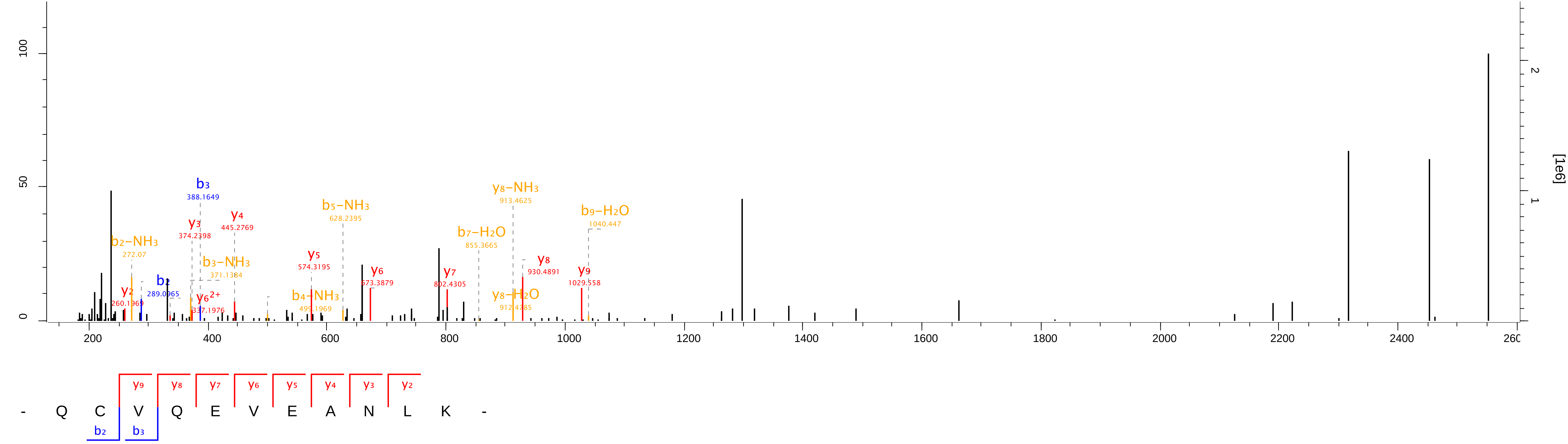

Raw file	Scan	Method	Score	m/z	Gene names
20140827_EXQ00_FaHo_SA_parental_01	5617	FTMS; HCD	71.5	456.78	RPS0A;RPS0B

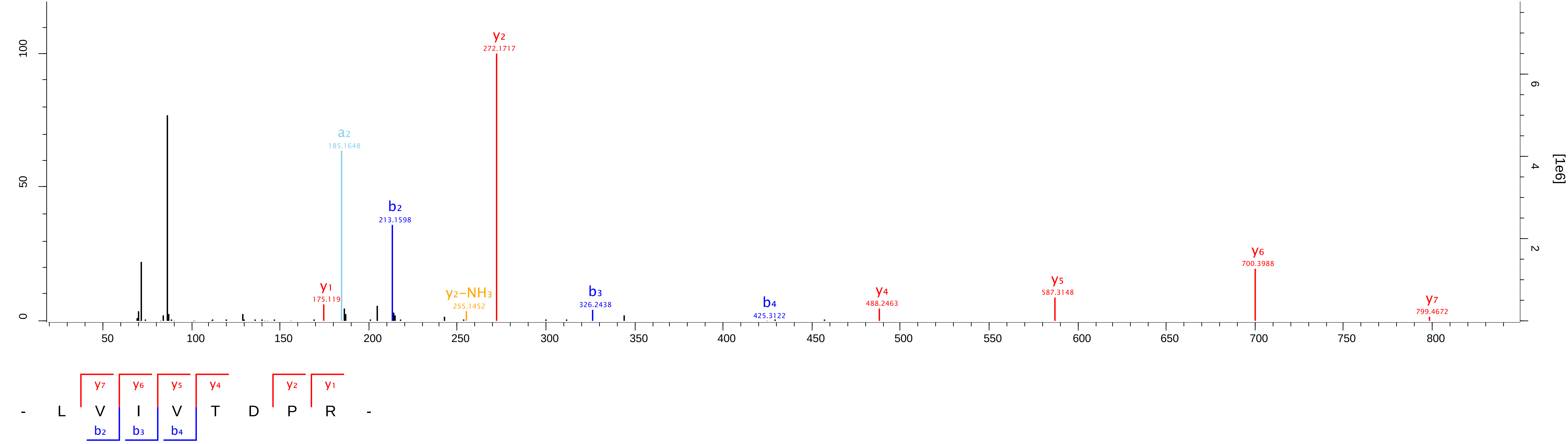

Raw file	Scan	Method	Score	m/z	Gene names
20140827_EXQ00_FaHo_SA_parental_02	3578	FTMS; HCD	131.04	379.72	RPS0B

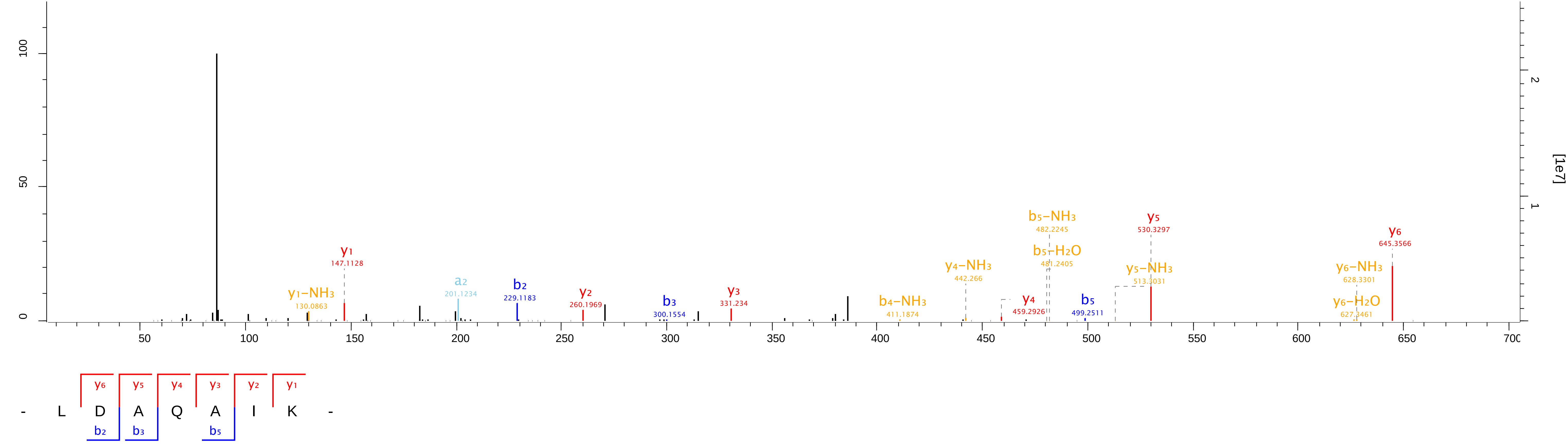

Raw file	Scan	Method	Score	m/z	Gene names
20140827_EXQ00_FaHo_SA_parental_02	6610	FTMS; HCD	170.11	923.49	RPL4B;RPL4A

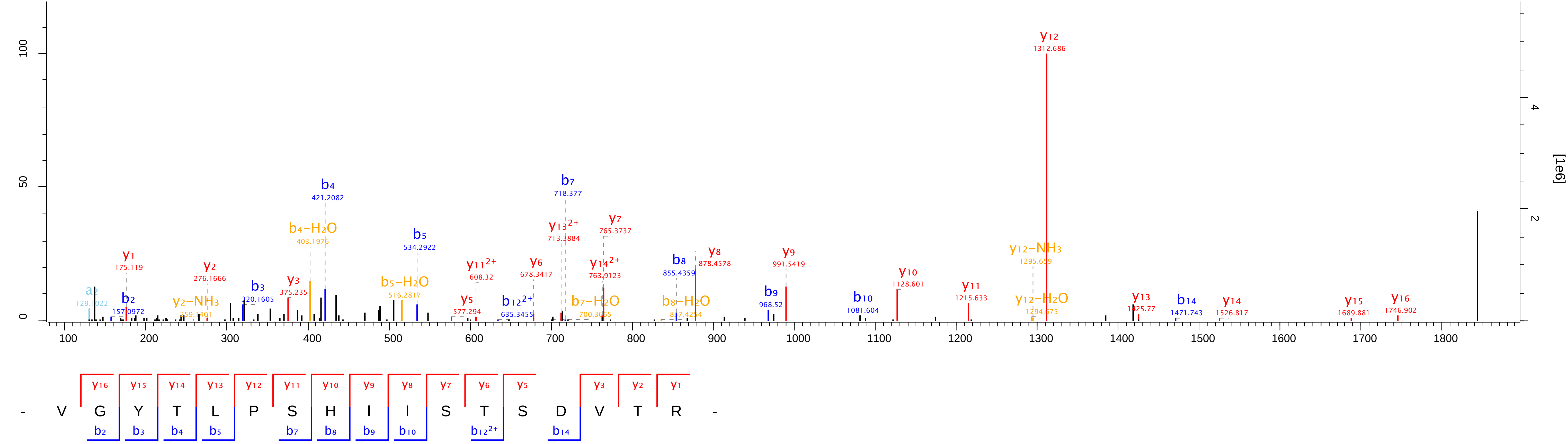

Raw file	Scan	Method	Score	m/z	Gene names
20140827_EXQ00_FaHo_SA_parental_02	8481	FTMS; HCD	99.54	1140.57	RPS0A;RPS0B

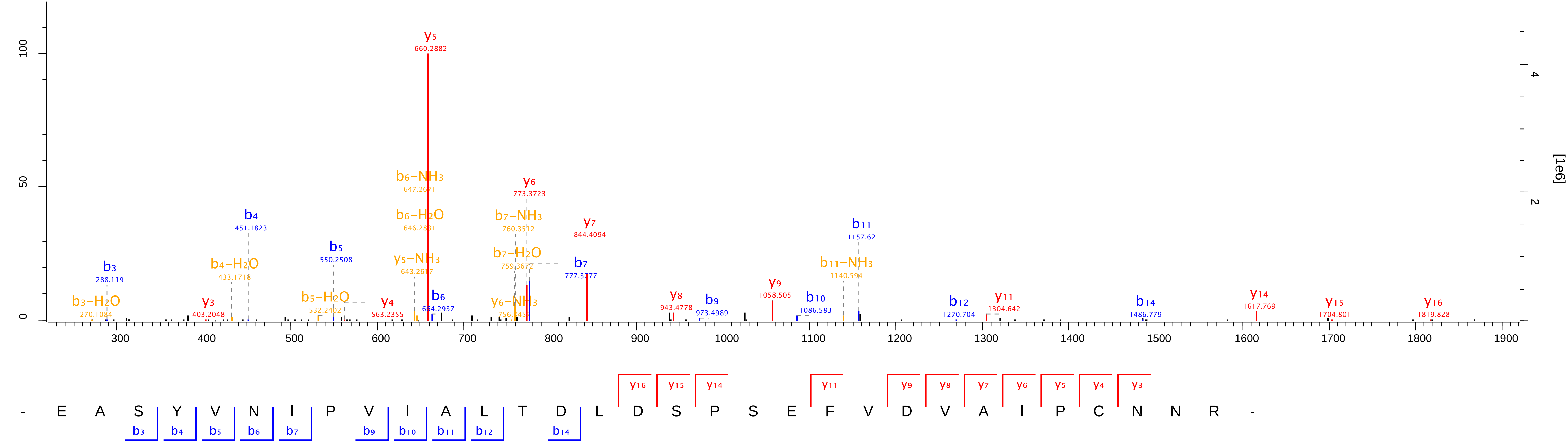

Raw file	Scan	Method	Score	m/z	Gene names
20140827_EXQ00_FaHo_SA_parental_03	3487	FTMS; HCD	149.17	487.78	RPL24A;RPL24B

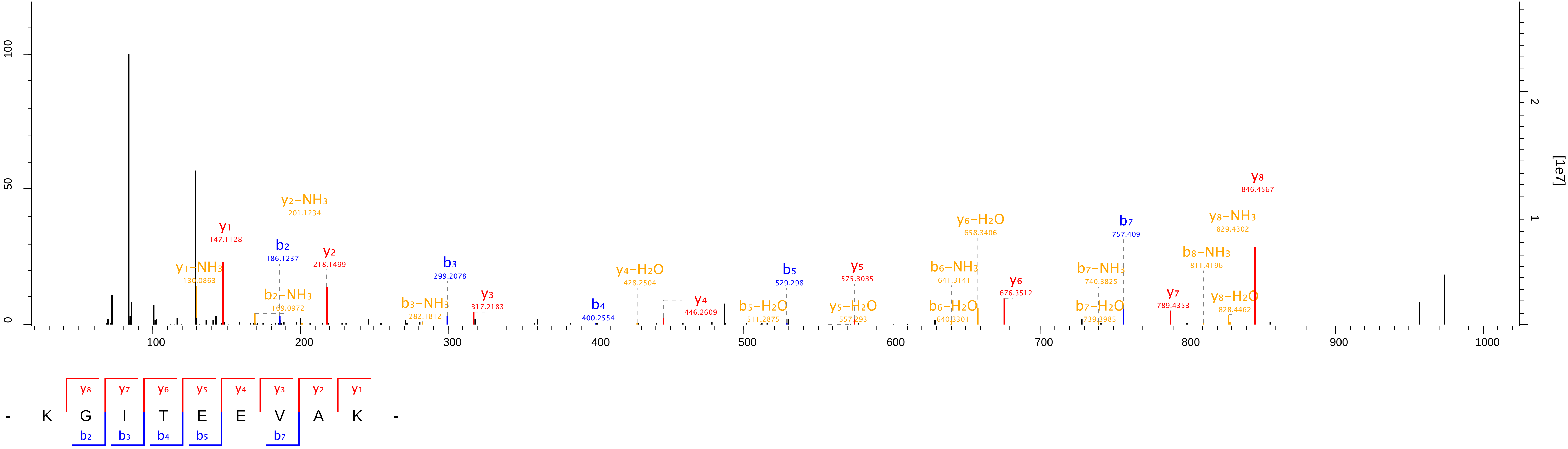

Raw file	Scan	Method	Score	m/z	Gene names
20140827_EXQ00_FaHo_SA_parental_03	7369	FTMS; HCD	75.02	823.94	NAP1

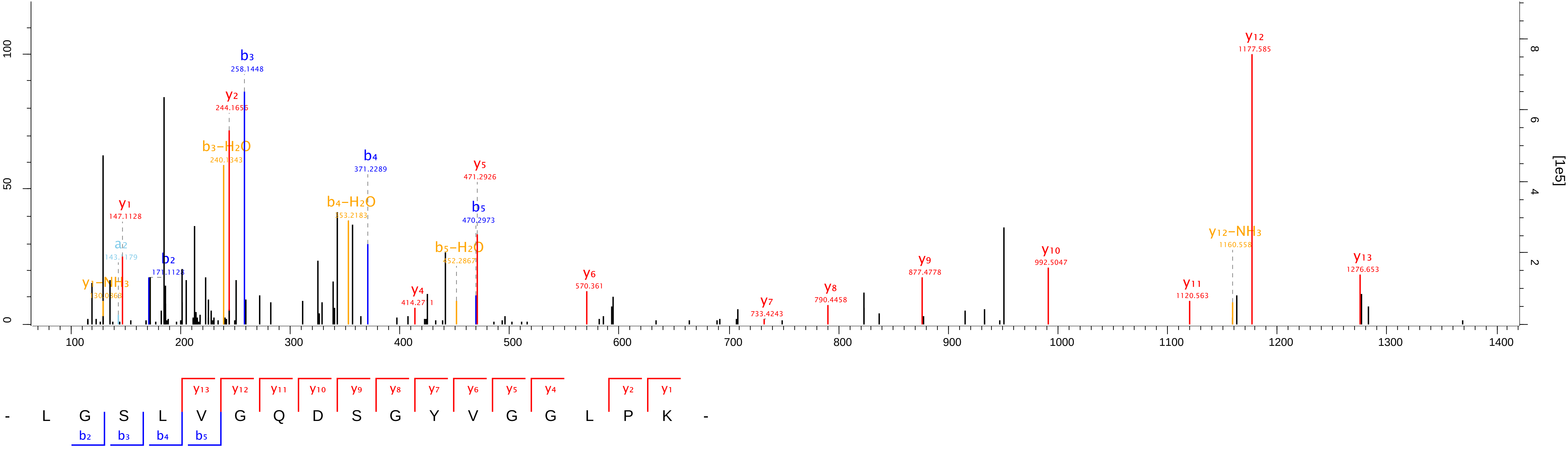

Raw file	Scan	Method	Score	m/z	Gene names
20140827_EXQ00_FaHo_SA_PHO23_(	3583	FTMS;	256.91	625.83	TY1B-LR3;TY1A-PL;TY1A-LR2;TY1A-ER1;TY1A-DR6;TY1B-OL;TY1B-LR4;TY1B-LR2;TY1B-PL;TY1B-ER1;TY1B-PR3;TY1A-PR1;TY1A-A;TY1A-DR4;TY1B-H;TY1B-GR2;TY1B-MR2;TY1B-ER2;TY1B-OR;TY1B-BR;TY1B-DR1;TY1B-NI

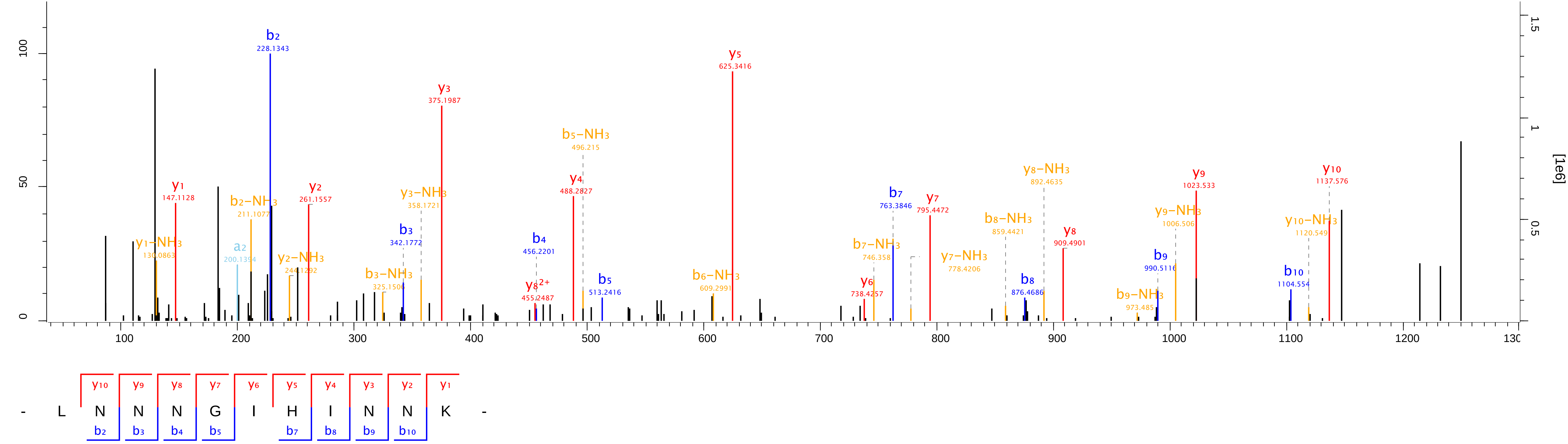

Raw file	Scan	Method	Score	m/z	Gene names
20140827_EXQ00_FaHo_SA_PHO23_02	5468	FTMS; HCD	111.57	812.42	TY1A-PL;TY1A-LR2;TY1A-ER1;TY1A-DR6

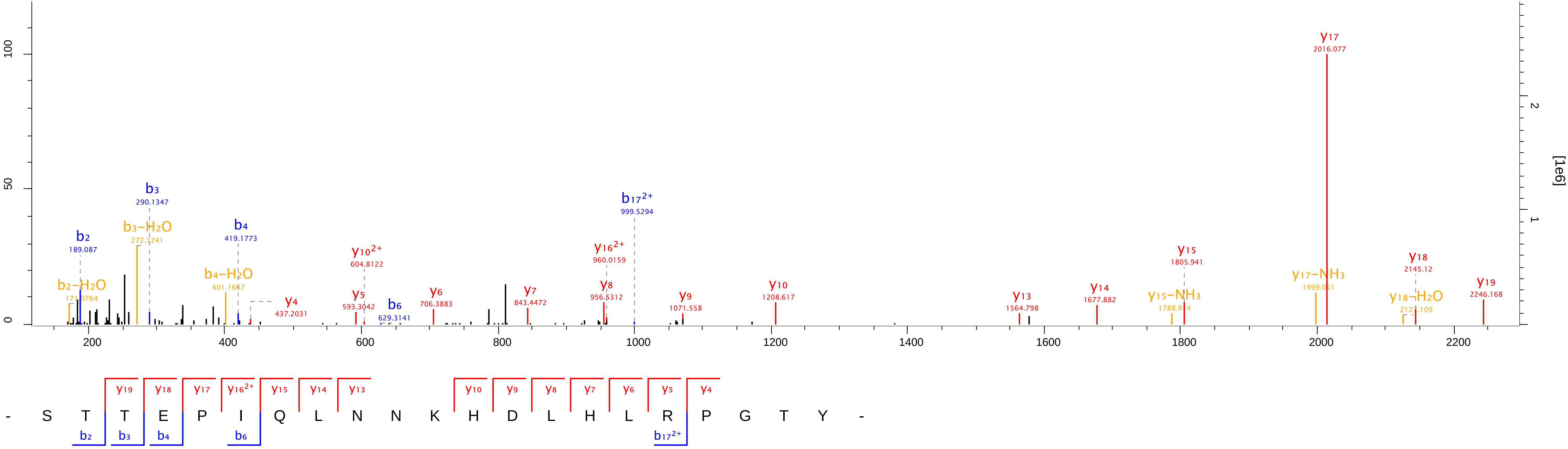

Raw file	Scan	Method	Score	m/z	Gene names
20140827_EXQ00_FaHo_SA_PHO23_02	5553	FTMS; HCD	96.23	572.82	RXT3

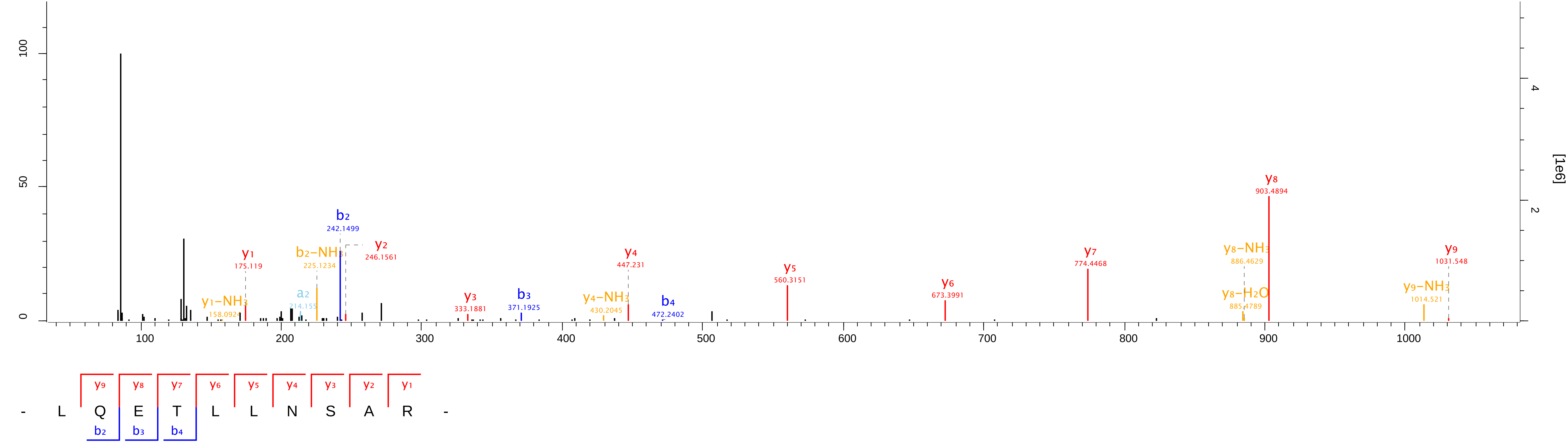

Raw file	Scan	Method	Score	m/z	Gene names
20140827_EXQ00_FaHo_SA_PHO23_03	2902	FTMS; HCD	54.19	352.19	IDH1

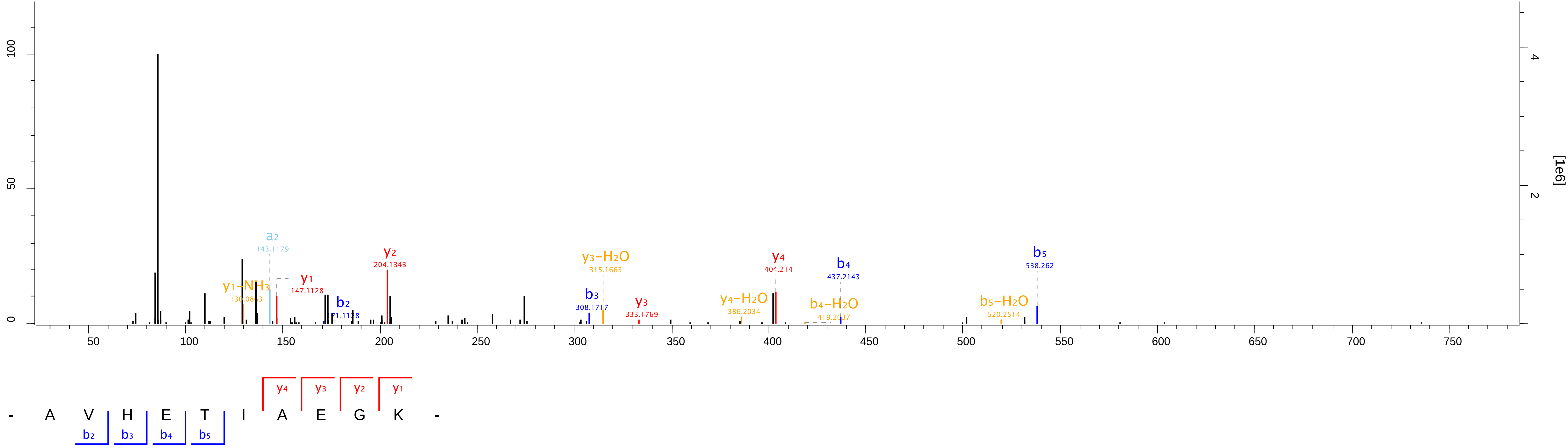

Raw file	Scan	Method	Score	m/z	Gene names
20140827_EXQ00_FaHo_SA_RCO1_01	5649	FTMS; HCD	118.76	437.89	RPL24A

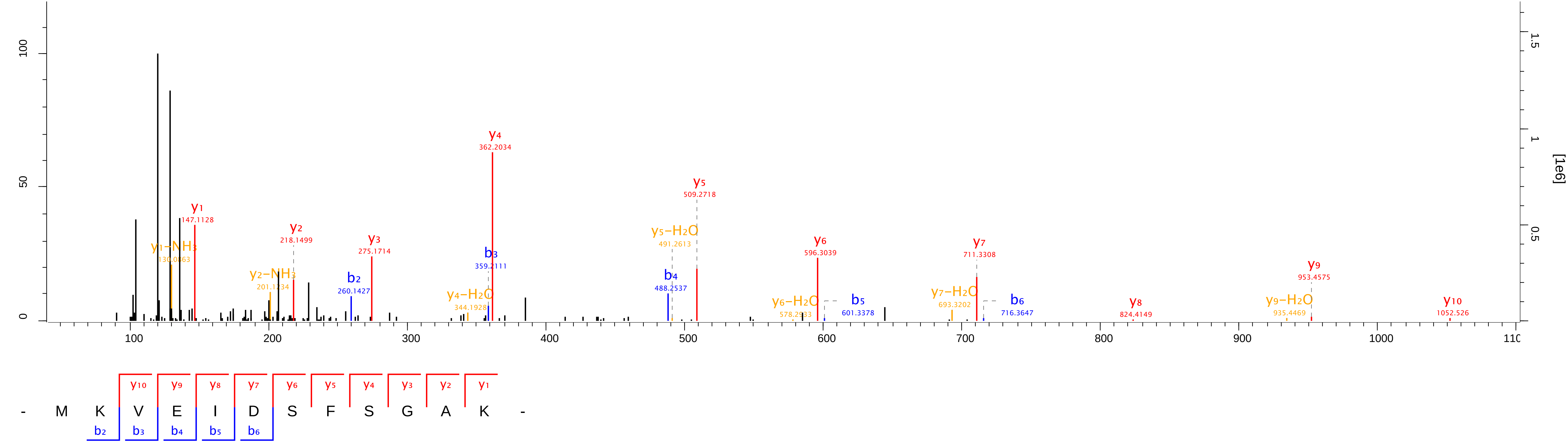

Raw file	Scan	Method	Score	m/z	Gene names
20140827_EXQ00_FaHo_SA_RCO1_02	3599	FTMS; HCD	117.16	390.7	RPL17A;RPL17B

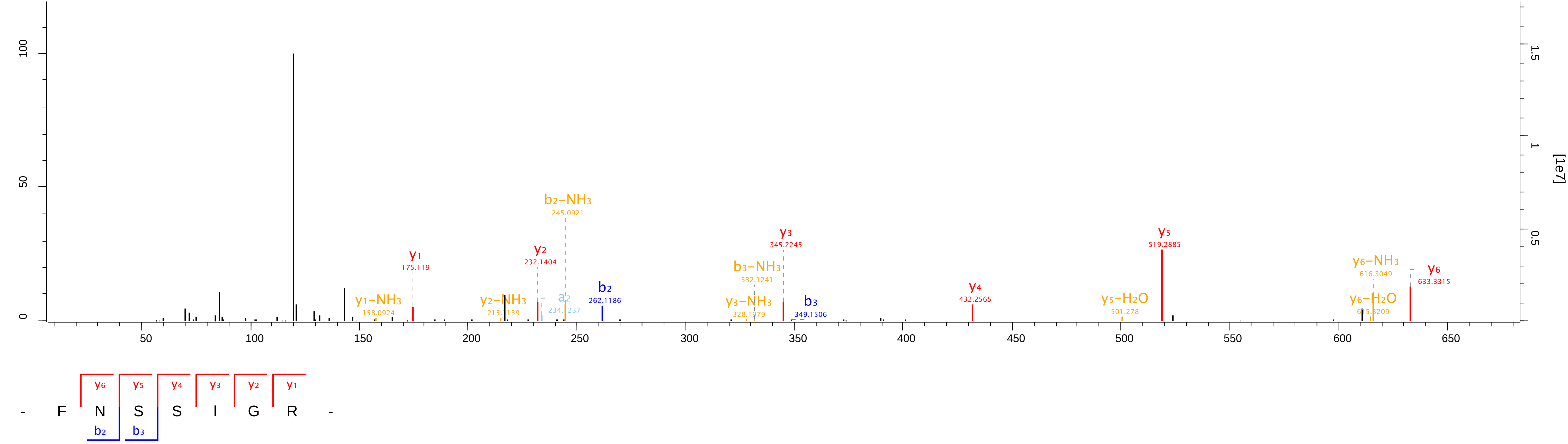

Raw file	Scan	Method	Score	m/z	Gene names
20140827_EXQ00_FaHo_SA_RCO1_02	7864	FTMS; HCD	147.33	639.34	RPL9A;RPL9B

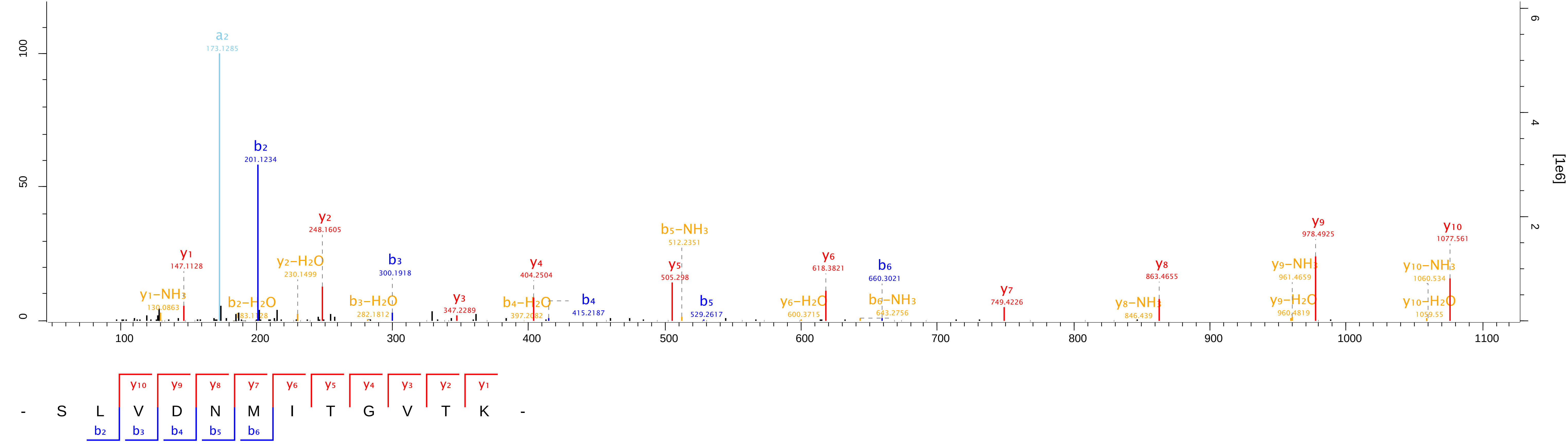

Raw file	Scan	Method	Score	m/z	Gene names
20140827_EXQ00_FaHo_SA_RCO1_02	8716	FTMS; HCD	155.87	1389.73	RPS0A;RPS0B

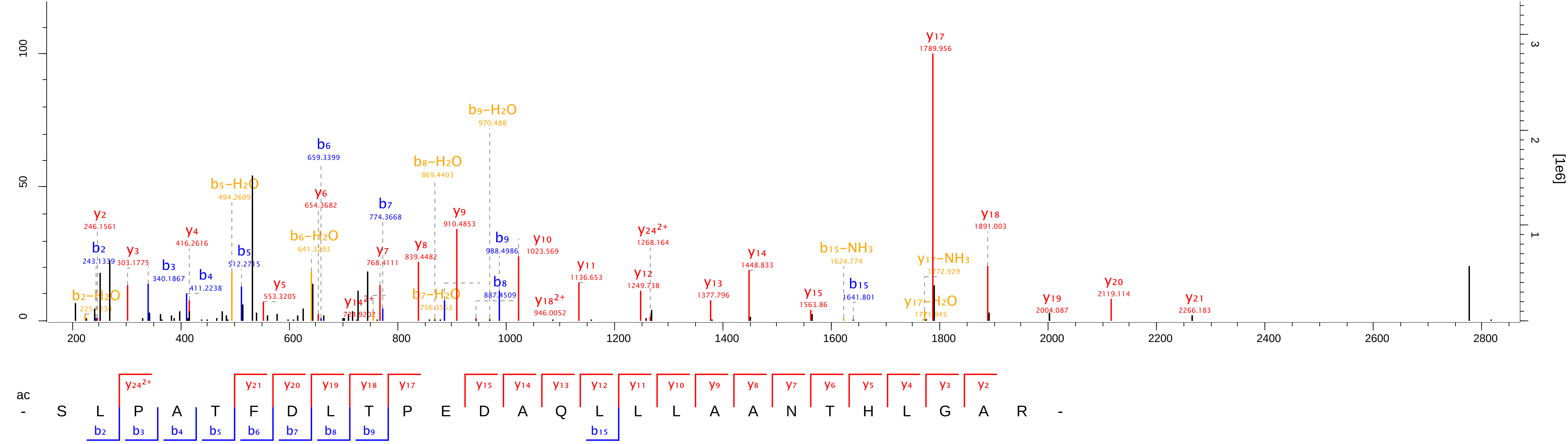

Raw file	Scan	Method	Score	m/z	Gene names
20140827_EXQ00_FaHo_SA_RCO1_03	6831	FTMS; HCD	204.82	822.94	RPL17A;RPL17B

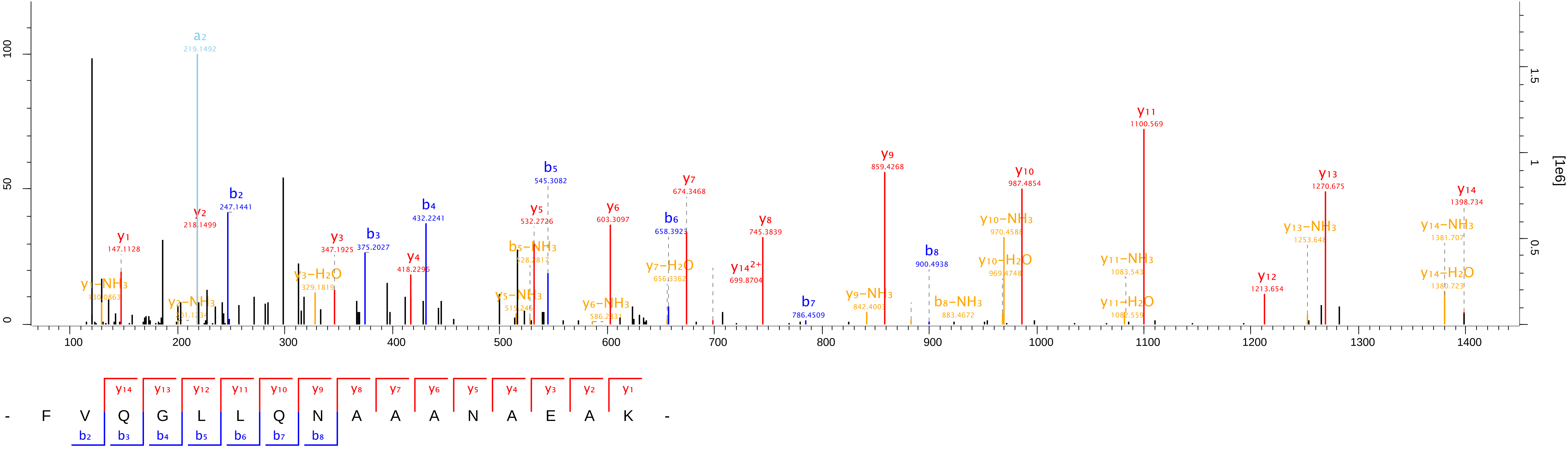

Raw file	Scan	Method	Score	m/z	Gene names
20140827_EXQ00_FaHo_SA_RSC2_02	3751	FTMS; HCD	92.47	351.21	RPL4B;RPL4A

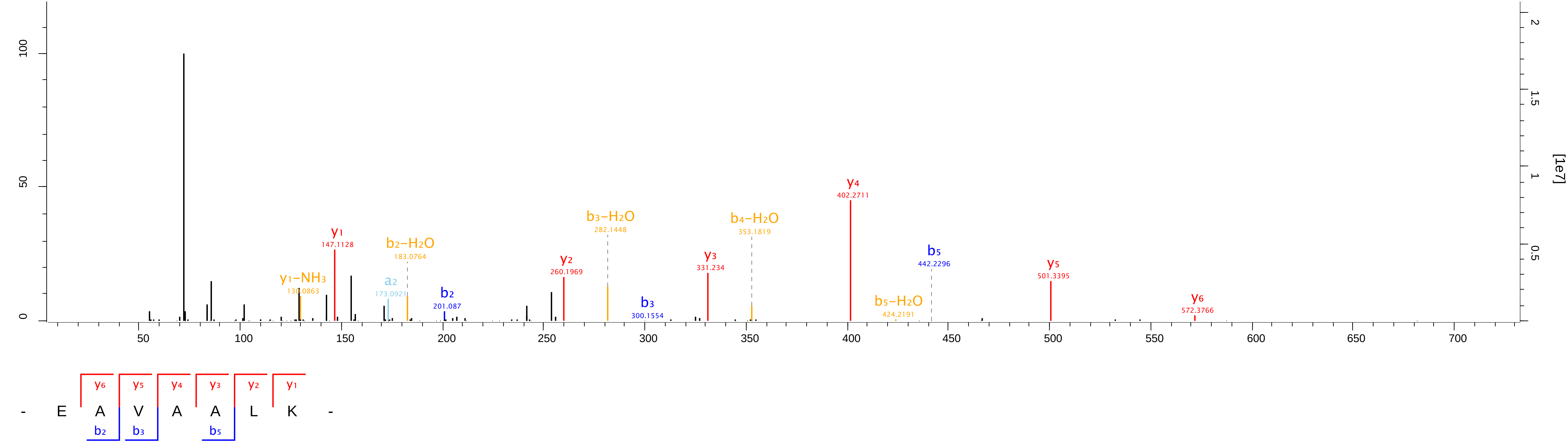

Raw file	Scan	Method	Score	m/z	Gene names
20140827_EXQ00_FaHo_SA_RSC4_01	2455	FTMS; HCD	56.63	375.52	AKL1

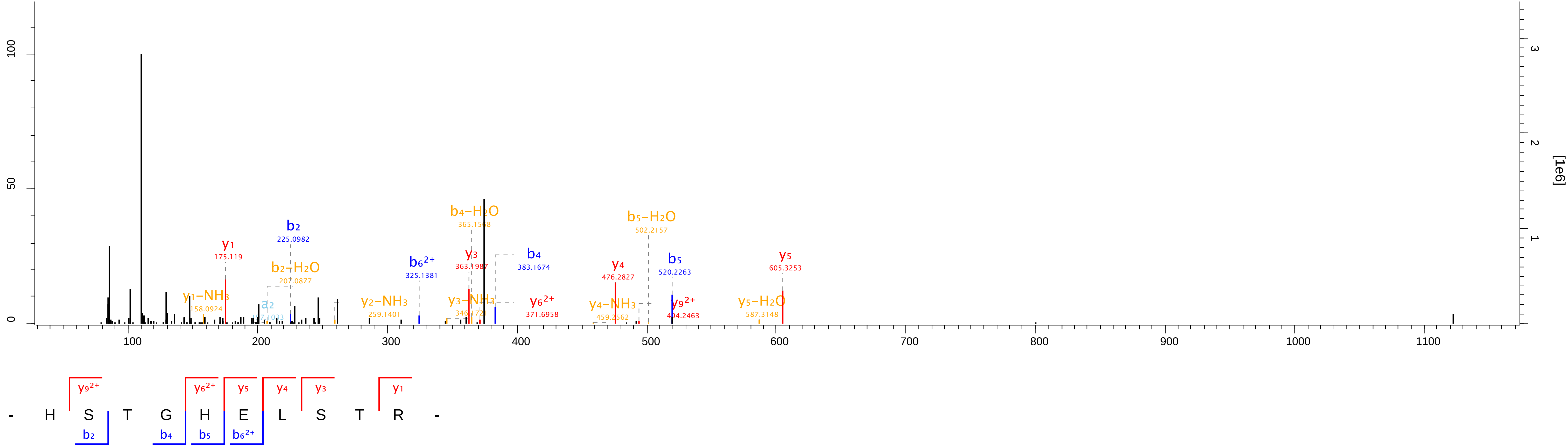

Raw file	Scan	Method	Score	m/z	Gene names
20140827_EXQ00_FaHo_SA_RSC4_02	5492	FTMS; HCD	58.98	919.43	CBP3

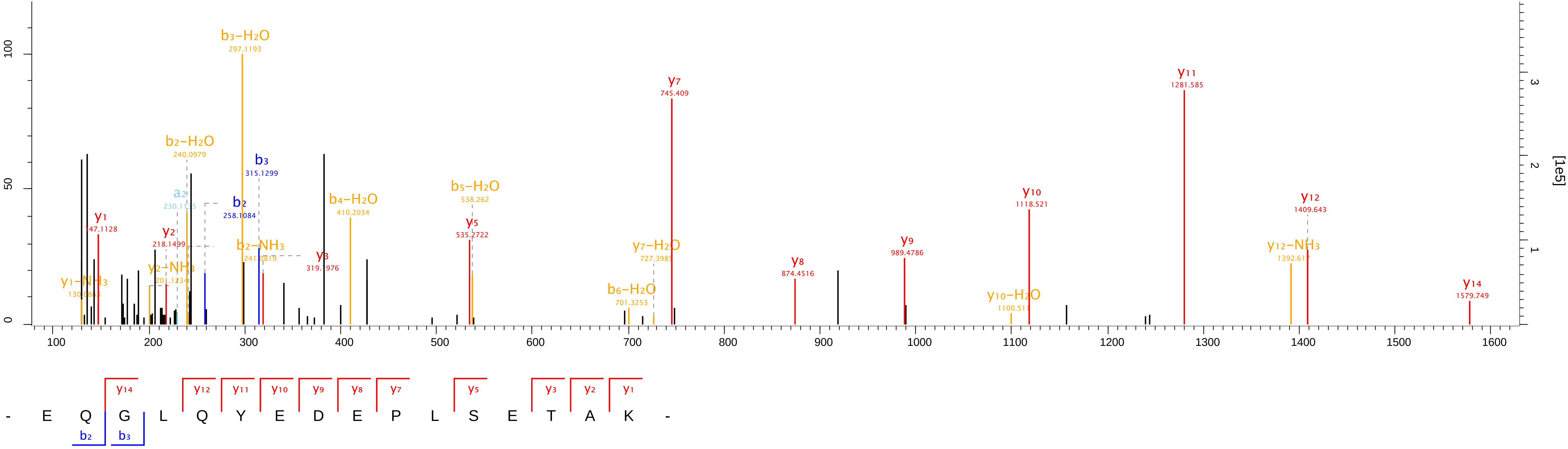

Raw file	Scan	Method	Score	m/z	Gene names
20140827_EXQ00_FaHo_SA_RSC4_02	7278	FTMS; HCD	79.92	859.67	LDB7

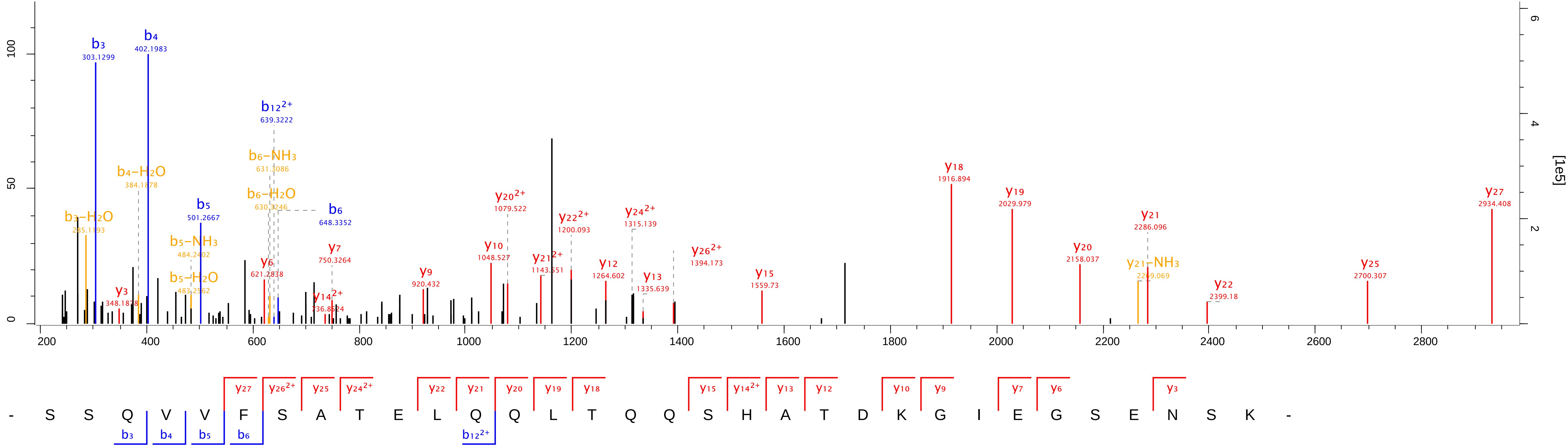

Raw file	Scan	Method	Score	m/z	Gene names
20140827_EXQ00_FaHo_SA_RSC4_02	7446	FTMS; HCD	114.5	478.31	RGR1

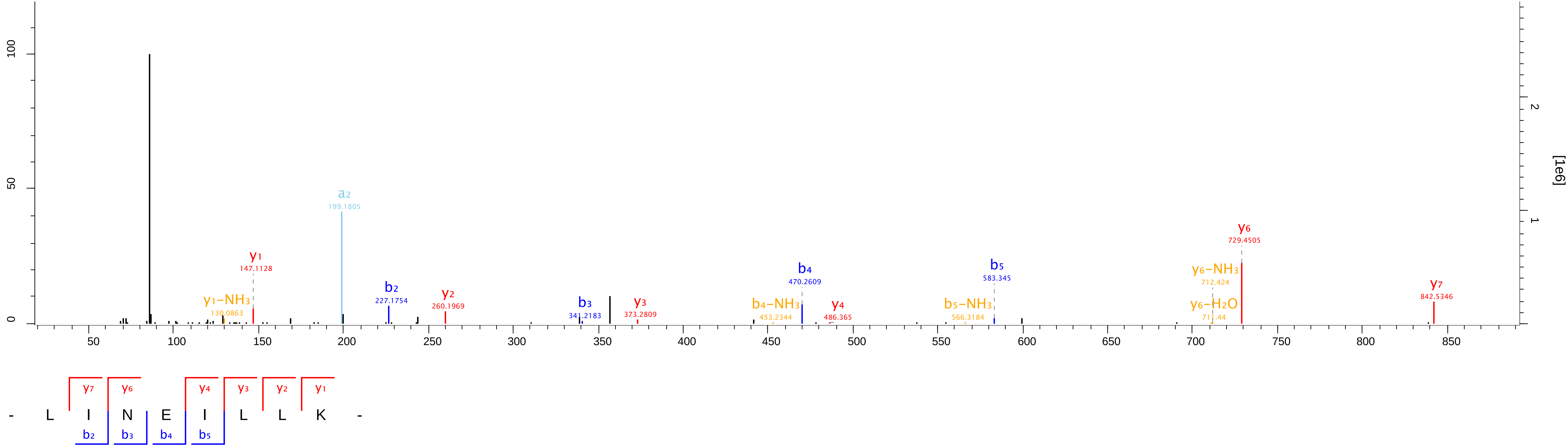

Raw file	Scan	Method	Score	m/z	Gene names
20140827_EXQ00_FaHo_SA_RSC4_03	7566	FTMS; HCD	184.87	777.93	RPL7A;RPL7B

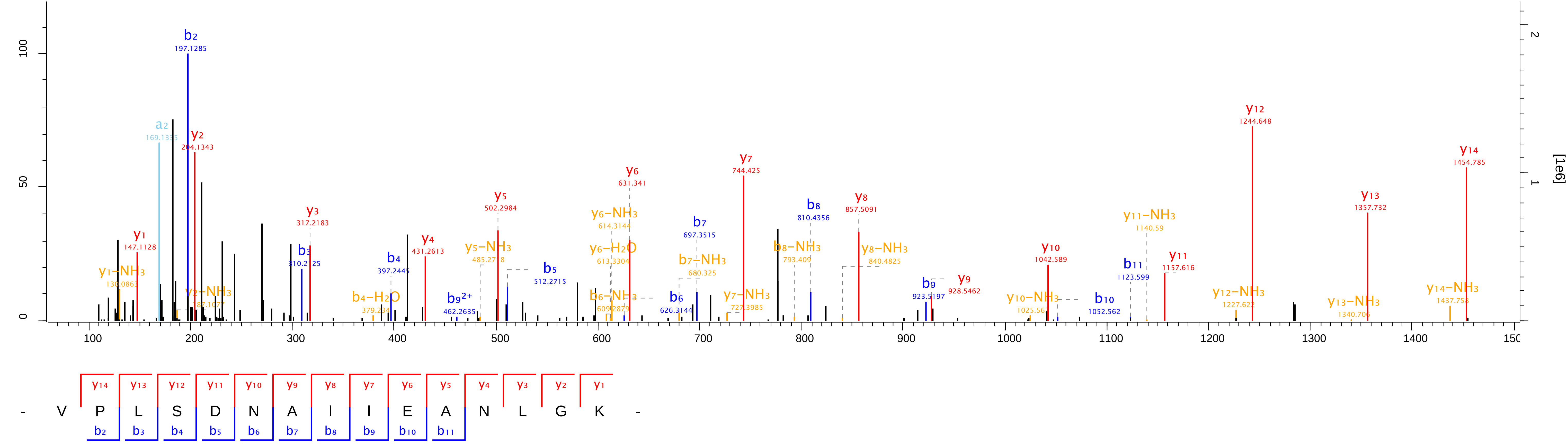

Raw file	Scan	Method	Score	m/z	Gene names
20140827_EXQ00_FaHo_SA_RSC4_03	8051	FTMS; HCD	67.4	1009.5	RPC10

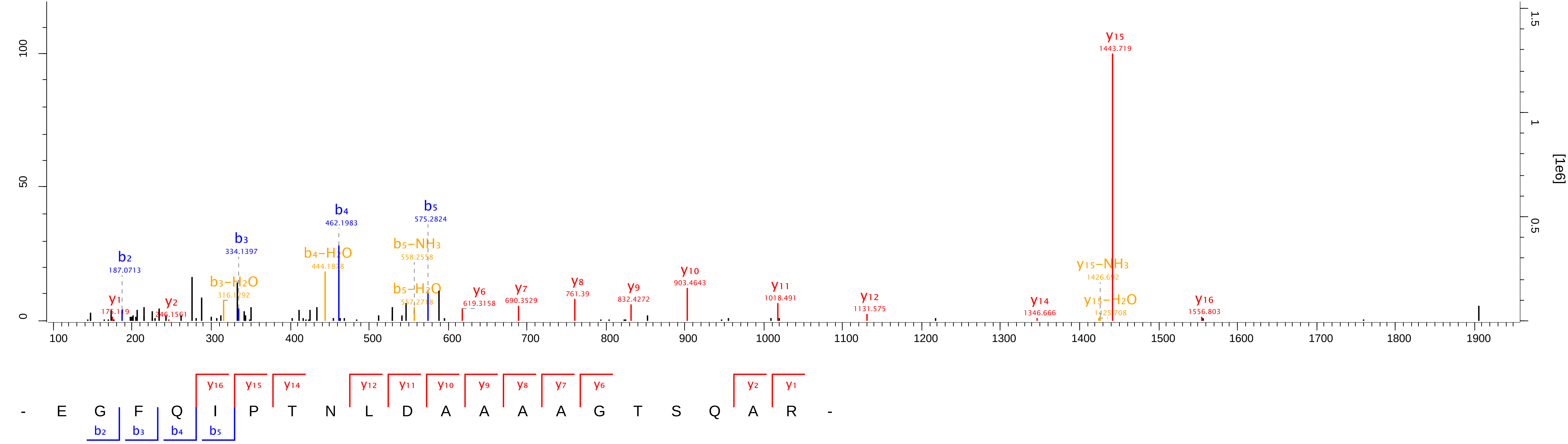

Raw file	Scan	Method	Score	m/z	Gene names
20140827_EXQ00_FaHo_SA_RSC4_03	8383	FTMS; HCD	70.41	808.94	ILV1

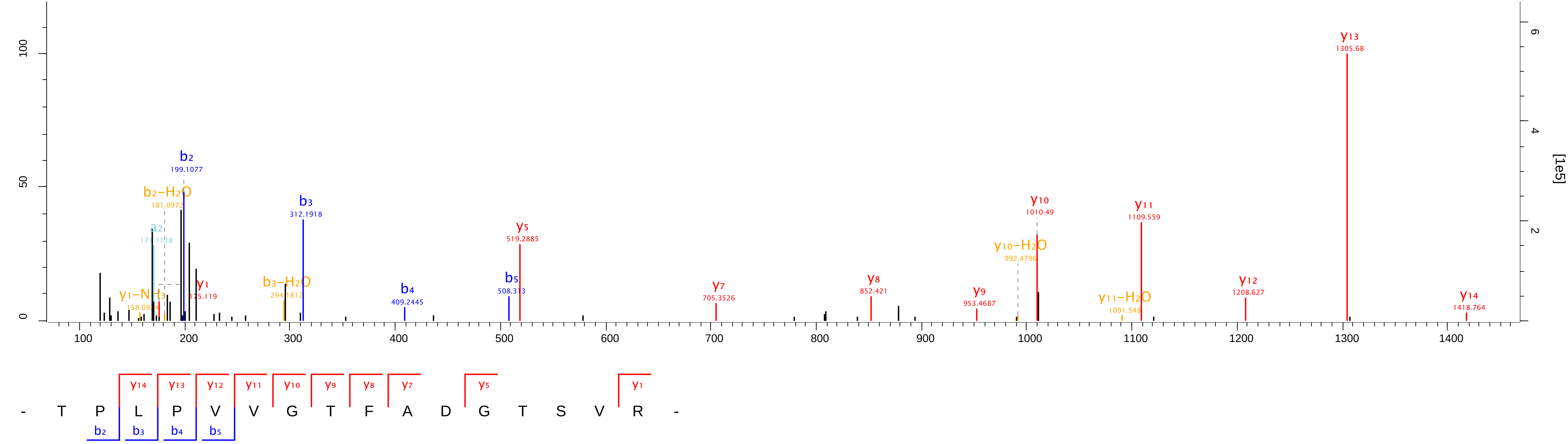

Raw file	Scan	Method	Score	m/z	Gene names
20140827_EXQ00_FaHo_SA_RSC8_02	7106	FTMS; HCD	58.89	826.43	CHD1

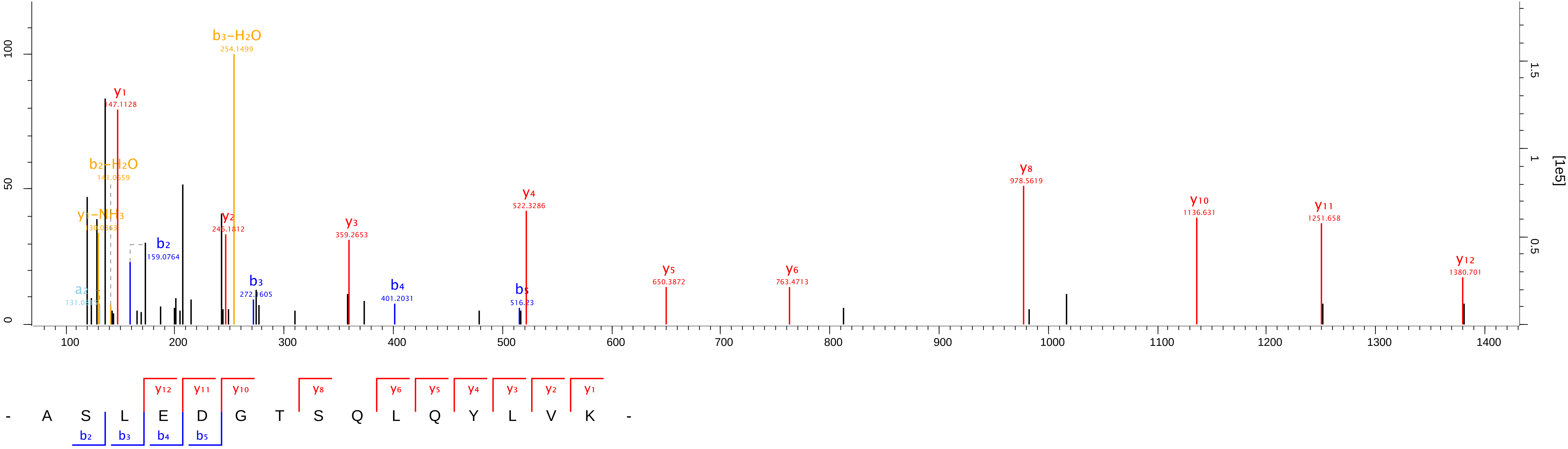

Raw file	Scan	Method	Score	m/z	Gene names
20140827_EXQ00_FaHo_SA_SET1_01	2621	FTMS; HCD	182.93	589.27	TY1B-LR3;TY1A-PL;TY1A-LR2;TY1A-ER1;TY1A-DR6;TY1B-OL;TY1B-LR4;TY1B-LR2;TY1B-PL;TY1B-ER1;TY1B-PR3

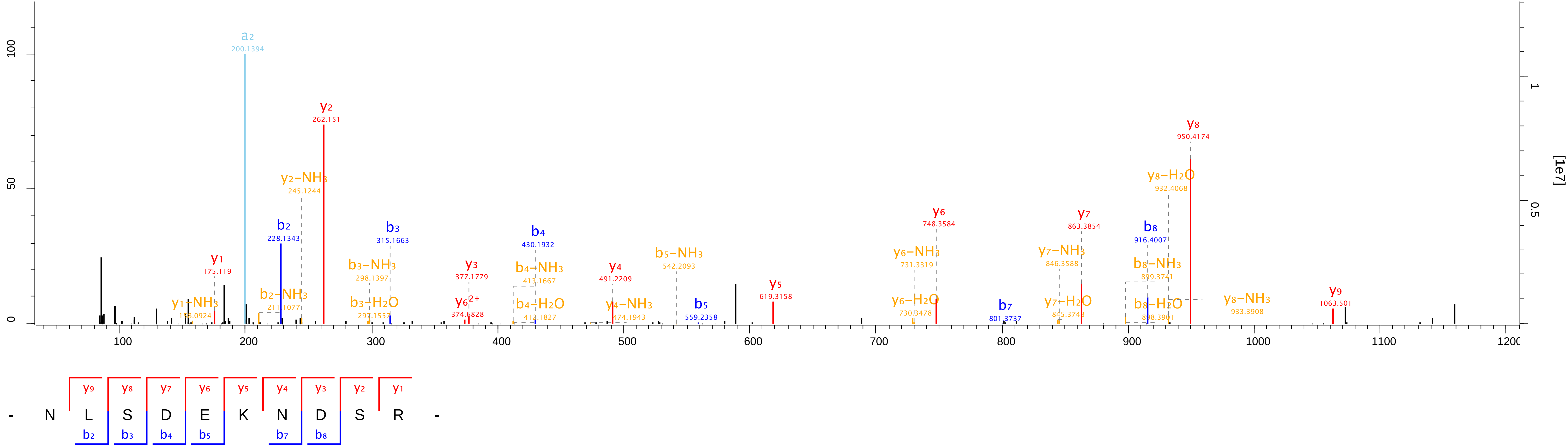

Raw file	Scan	Method	Score	m/z	Gene names
20140827_EXQ00_FaHo_SA_SET1_01	3363	FTMS; HCD	102.53	465.78	RPL4B;RPL4A

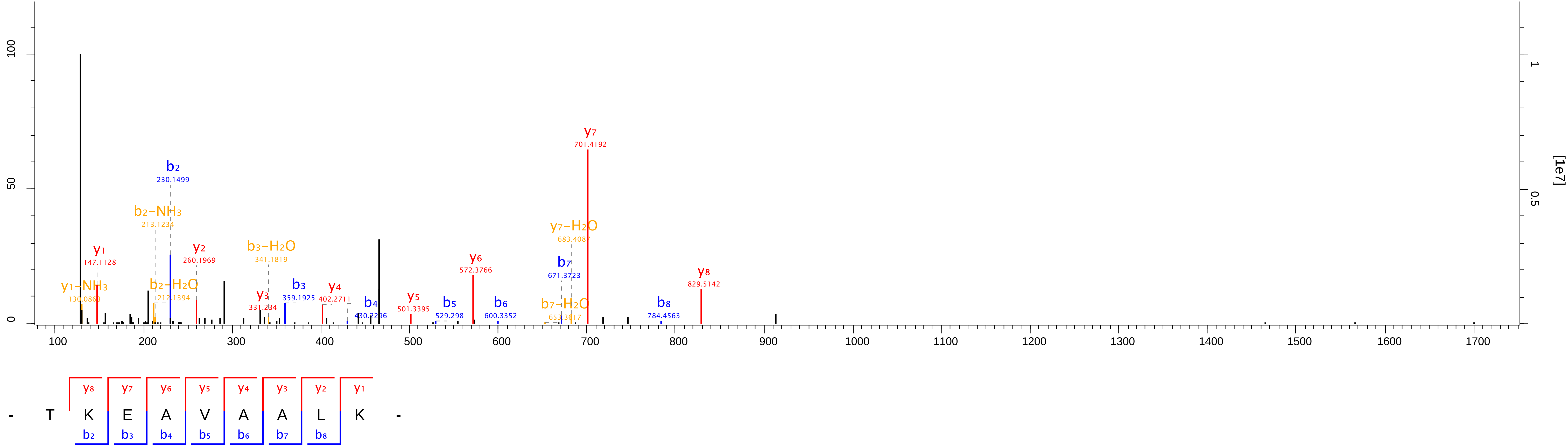

Raw file	Scan	Method	Score	m/z	Gene names
20140827_EXQ00_FaHo_SA_SET1_02	2231	FTMS; HCD	71.08	504.75	YDL121C

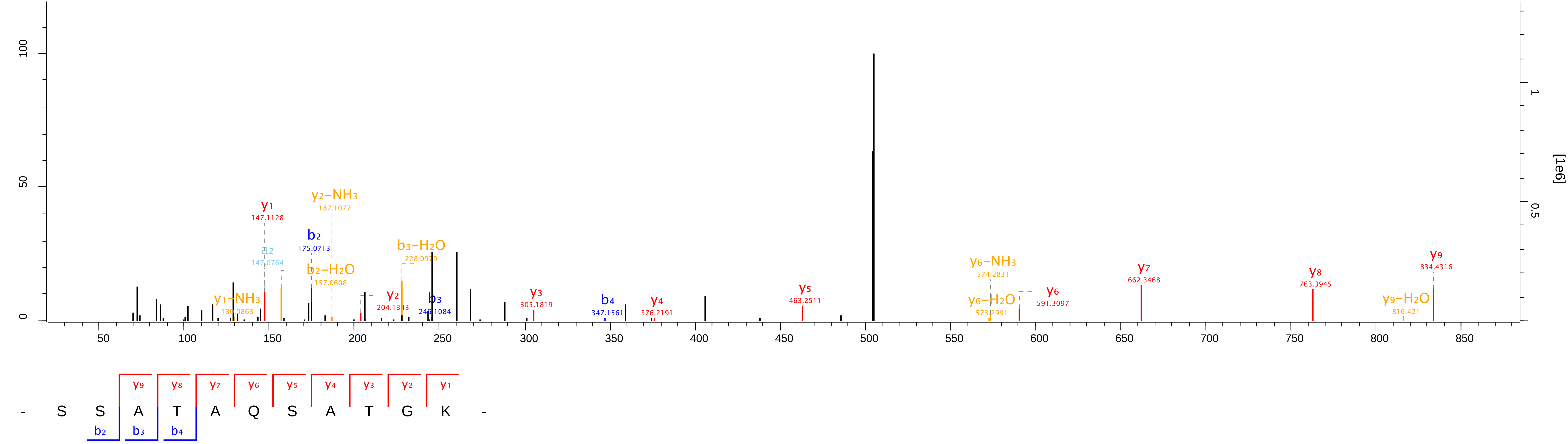

Raw file	Scan	Method	Score	m/z	Gene names
20140827_EXQ00_FaHo_SA_SET1_02	4701	FTMS; HCD	116.25	510.24	ARF1;ARF2

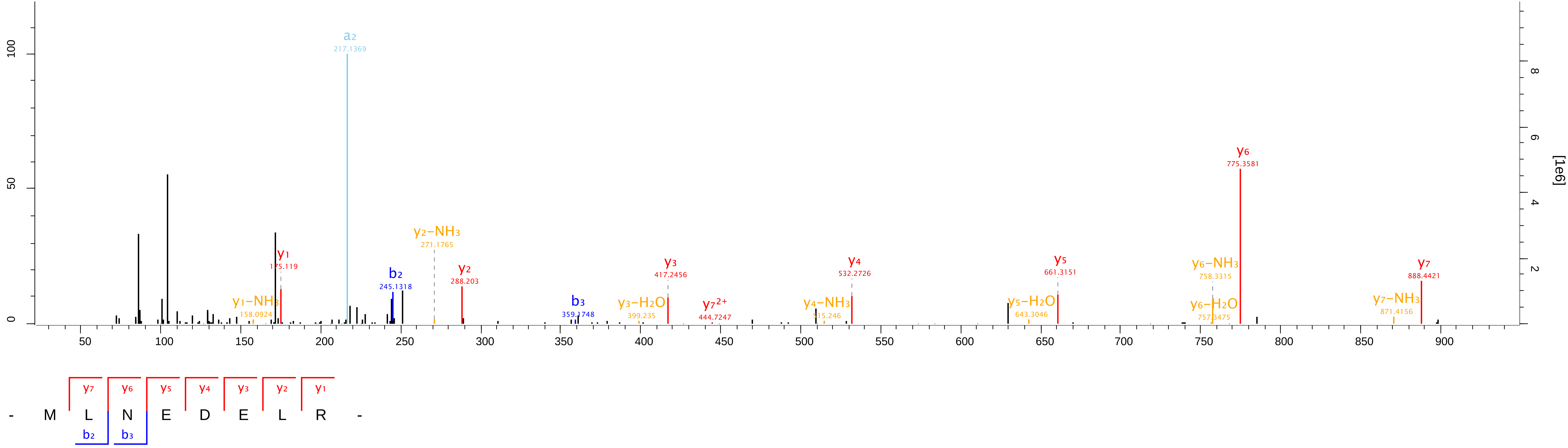

Raw file	Scan	Method	Score	m/z	Gene names
20140827_EXQ00_FaHo_SA_SET1_02	4743	FTMS; HCD	99.14	458.78	RPL21A;RPL21B

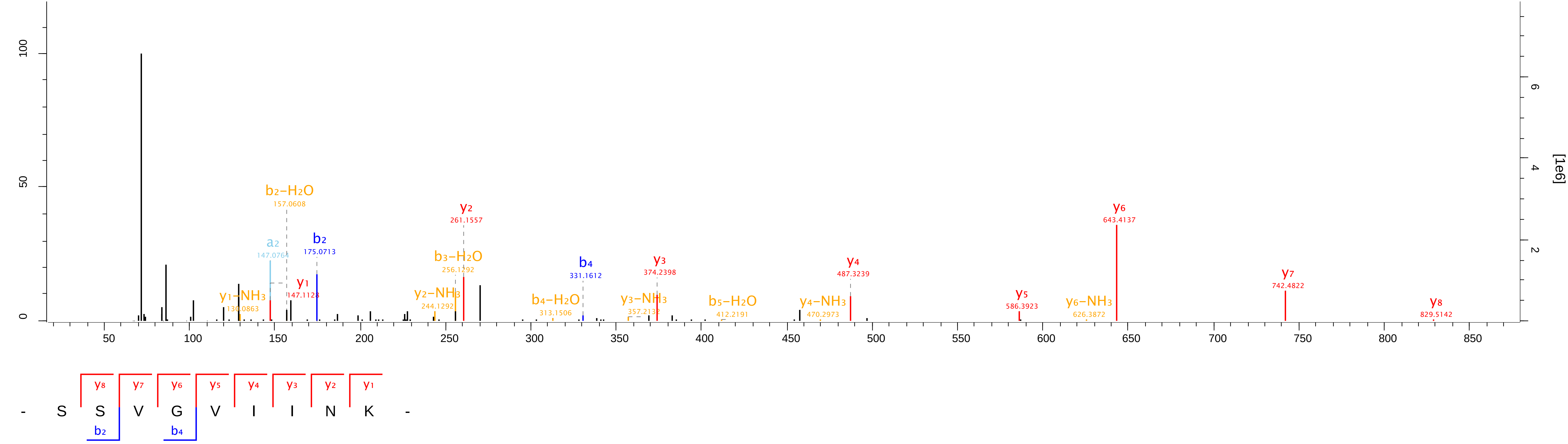

Raw file	Scan	Method	Score	m/z	Gene names
20140827_EXQ00_FaHo_SA_SET1_02	5179	FTMS; HCD	68	674.83	SSP120

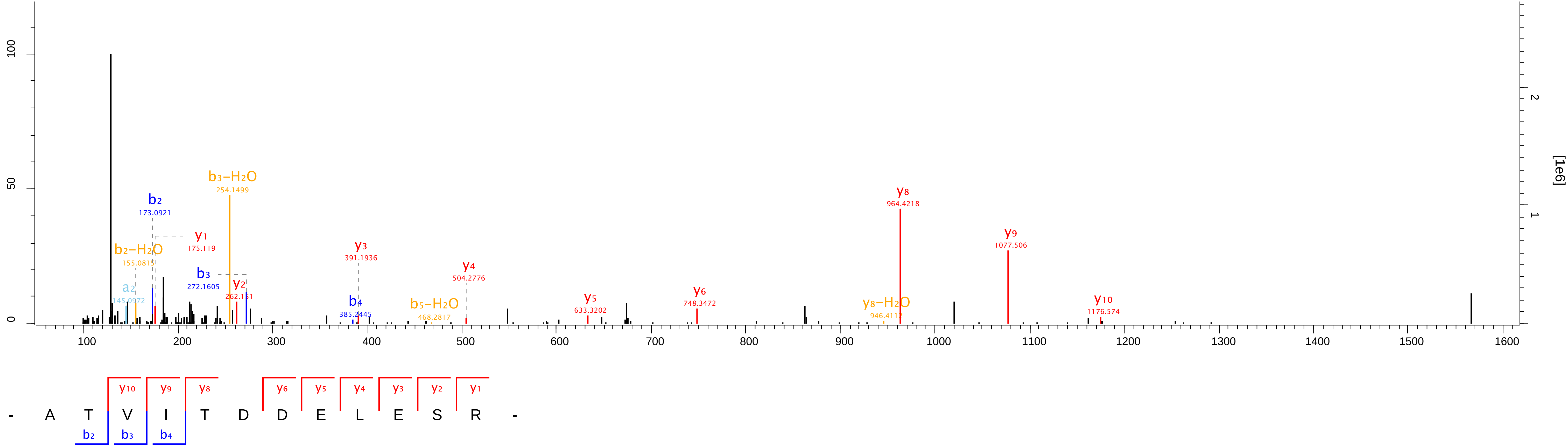

Raw file	Scan	Method	Score	m/z	Gene names
20140827_EXQ00_FaHo_SA_SET1_02	5590	FTMS; HCD	71.03	606.84	CPR6

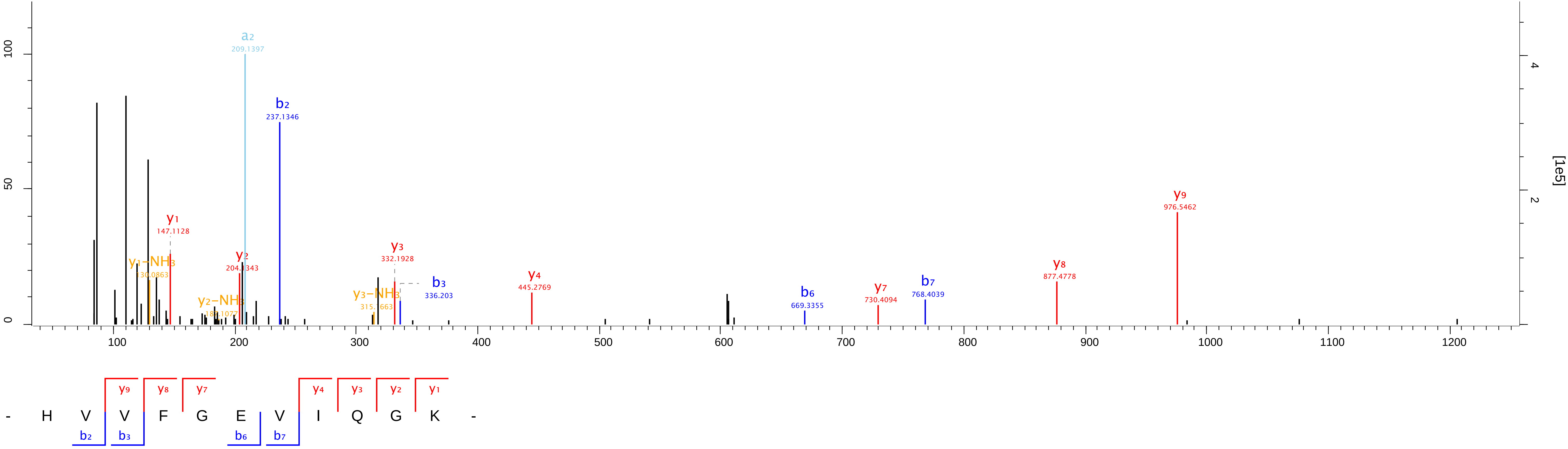

Raw file	Scan	Method	Score	m/z	Gene names
20140827_EXQ00_FaHo_SA_SET1_02	5601	FTMS; HCD	61.26	783.87	TOM40

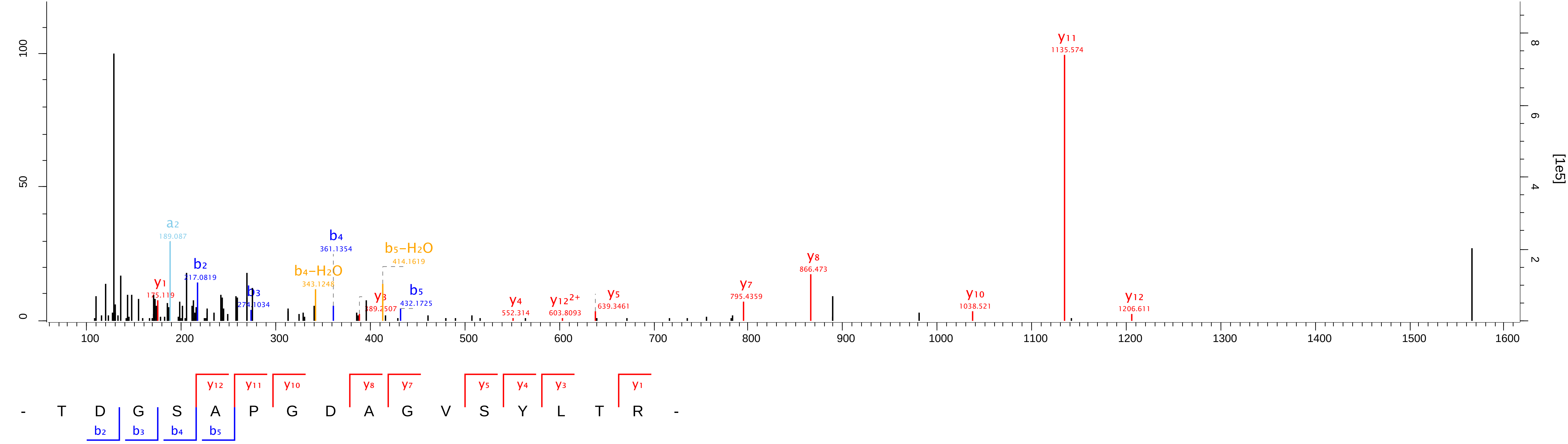

Raw file	Scan	Method	Score	m/z	Gene names
20140827_EXQ00_FaHo_SA_SET1_02	7268	FTMS; HCD	59.91	515.28	TUB1

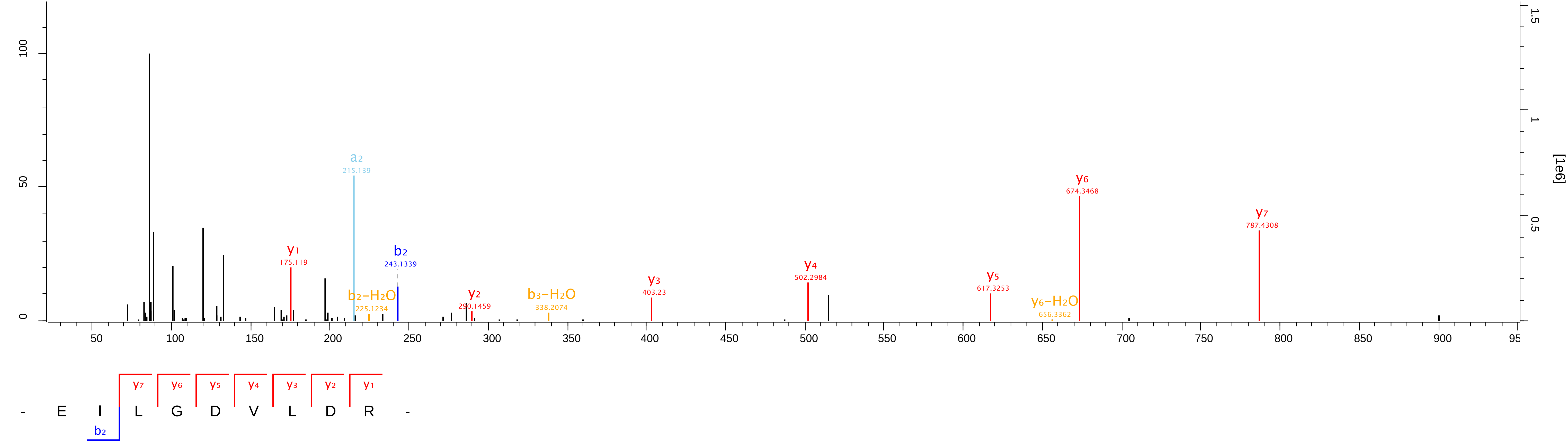

Raw file	Scan	Method	Score	m/z	Gene names
20140827_EXQ00_FaHo_SA_SET1_02	8537	FTMS; HCD	132.64	941.03	RPL4B;RPL4A

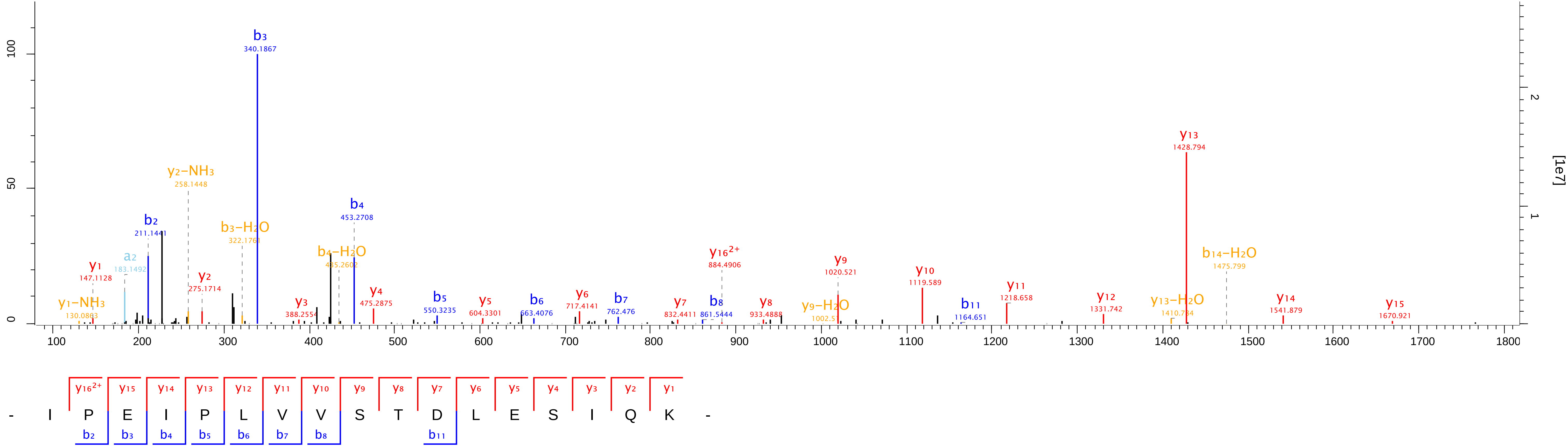

Raw file	Scan	Method	Score	m/z	Gene names
20140827_EXQ00_FaHo_SA_SET1_03	4649	FTMS; HCD	90.71	457.28	RPL21A;RPL21B

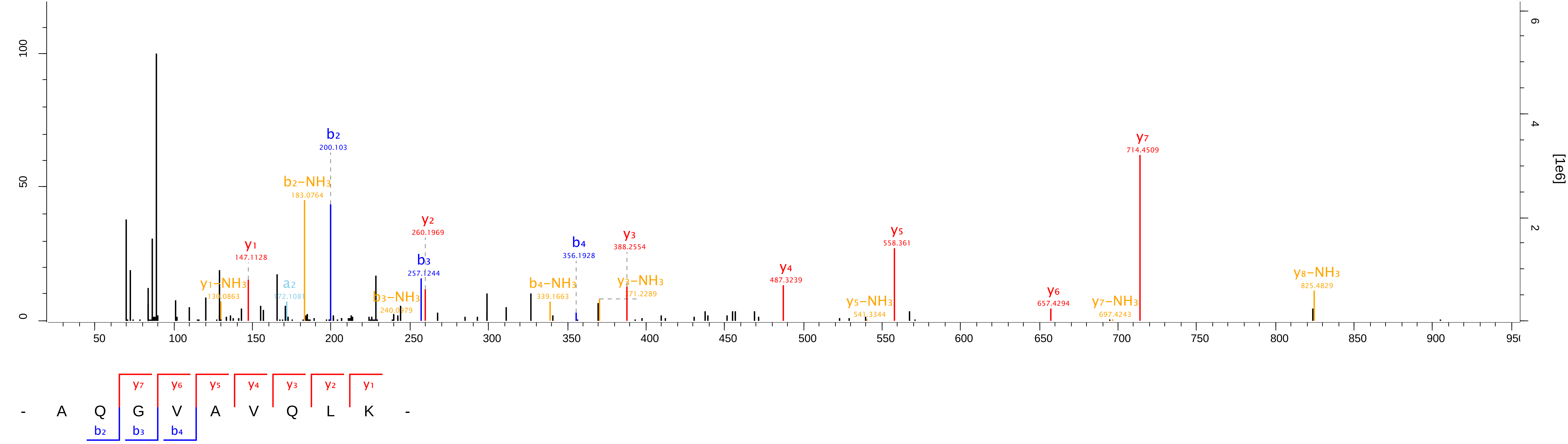

Raw file	Scan	Method	Score	m/z	Gene names
20140827_EXQ00_FaHo_SA_SET1_03	7865	FTMS; HCD	73.28	617.27	TUB1;TUB3

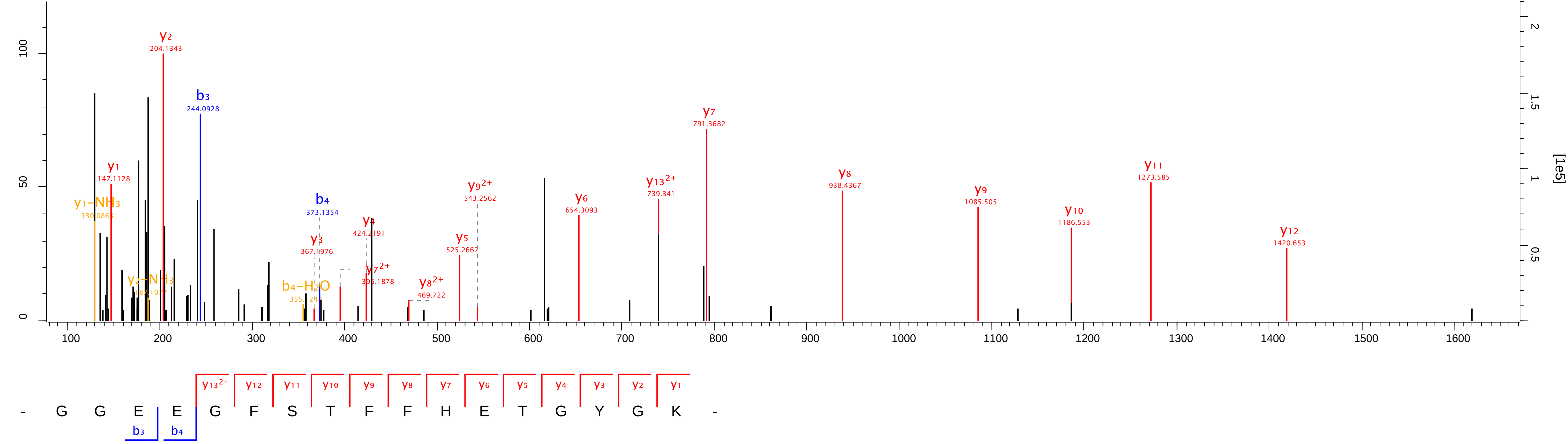

Raw file	Scan	Method	Score	m/z	Gene names
20140827_EXQ00_FaHo_SA_SET3_01	5382	FTMS; HCD	64.82	545.29	CCT2

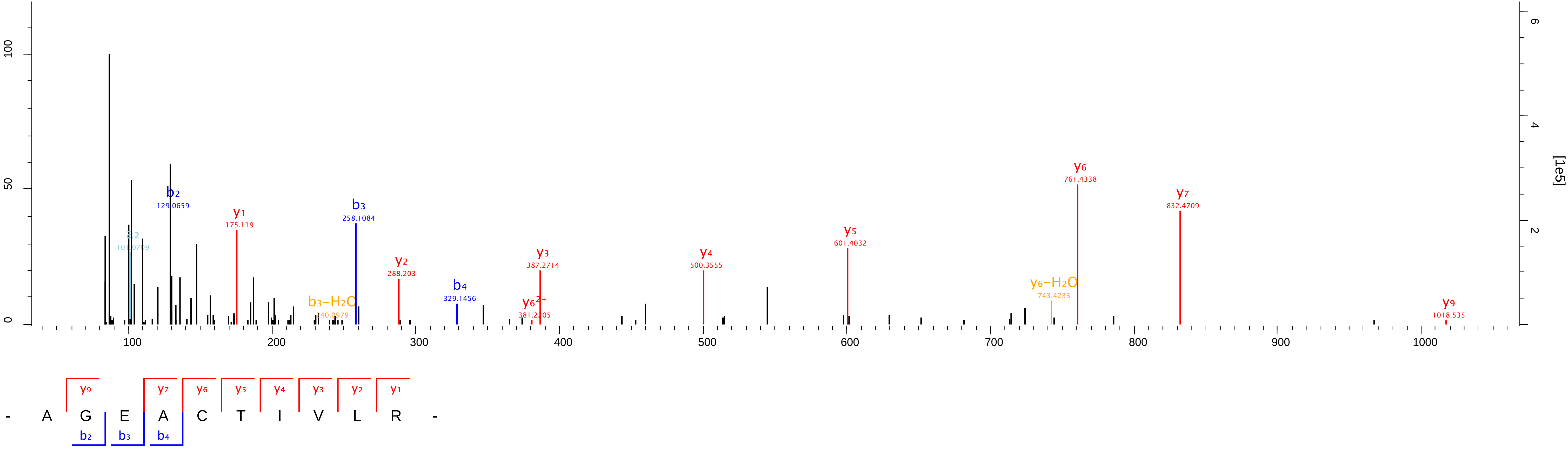

Raw file	Scan	Method	Score	m/z	Gene names
20140827_EXQ00_FaHo_SA_SET3_01	5993	FTMS; HCD	92.87	669.84	ARP3

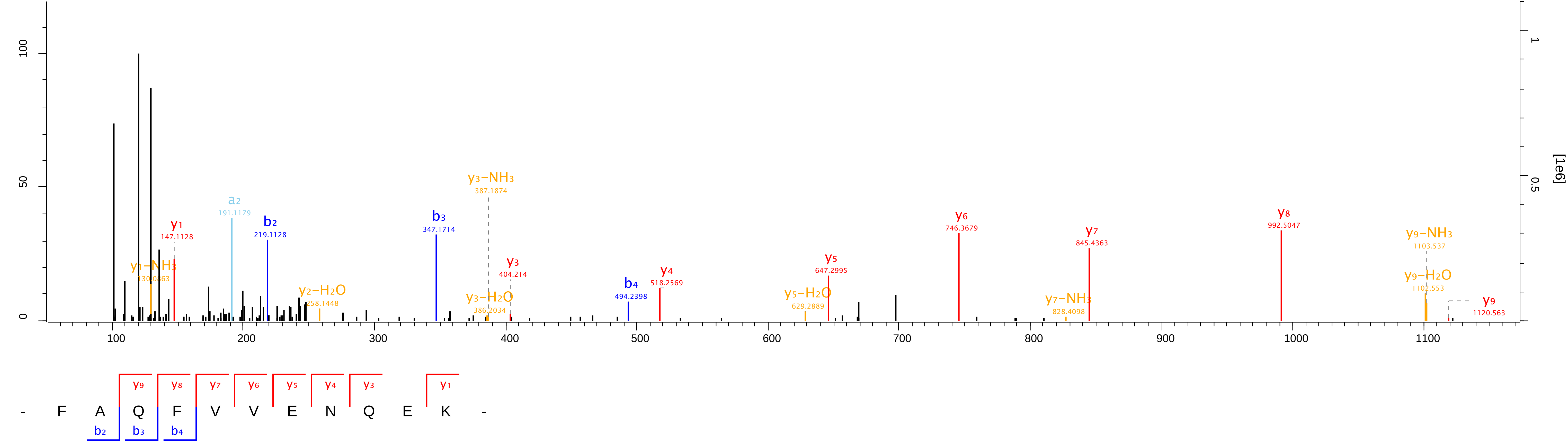

Raw file	Scan	Method	Score	m/z	Gene names
20140827_EXQ00_FaHo_SA_SET3_01	6552	FTMS; HCD	103.91	394.25	RPL7A;RPL7B

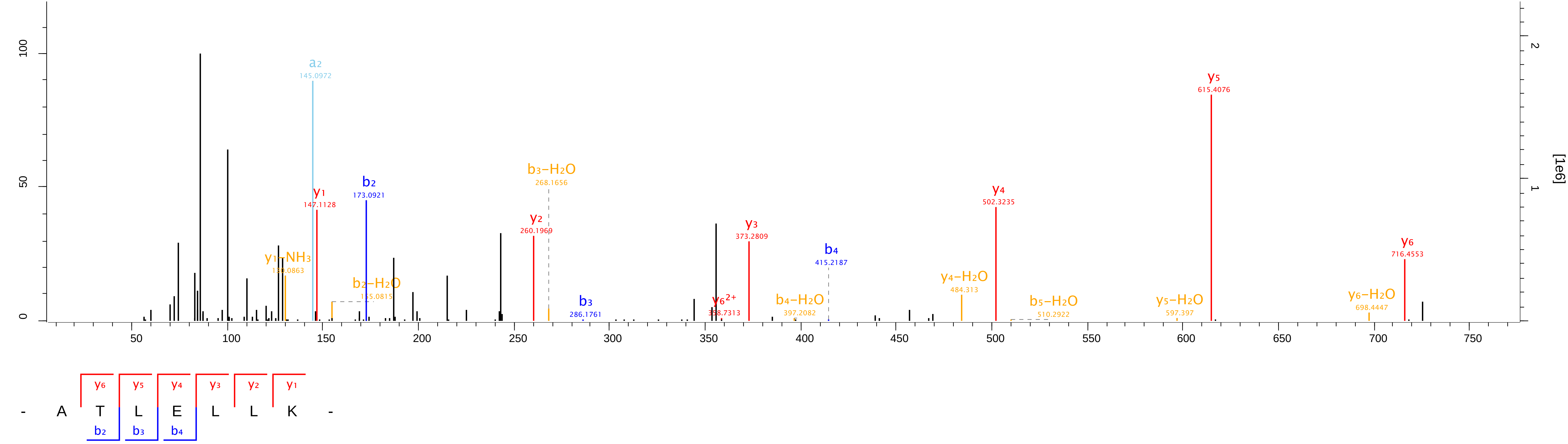

Raw file	Scan	Method	Score	m/z	Gene names
20140827_EXQ00_FaHo_SA_SET3_01	7387	FTMS; HCD	107.65	737.9	RPL4B;RPL4A

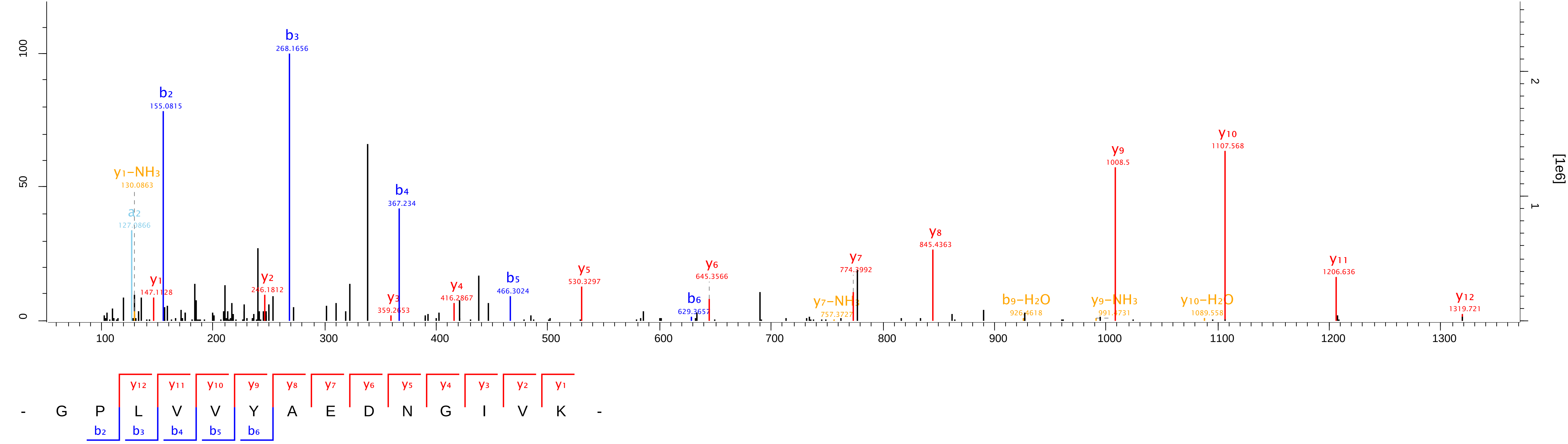

Raw file	Scan	Method	Score	m/z	Gene names
20140827_EXQ00_FaHo_SA_SHG1_01	3993	FTMS; HCD	77.19	515.27	RPS14B;RPS14A

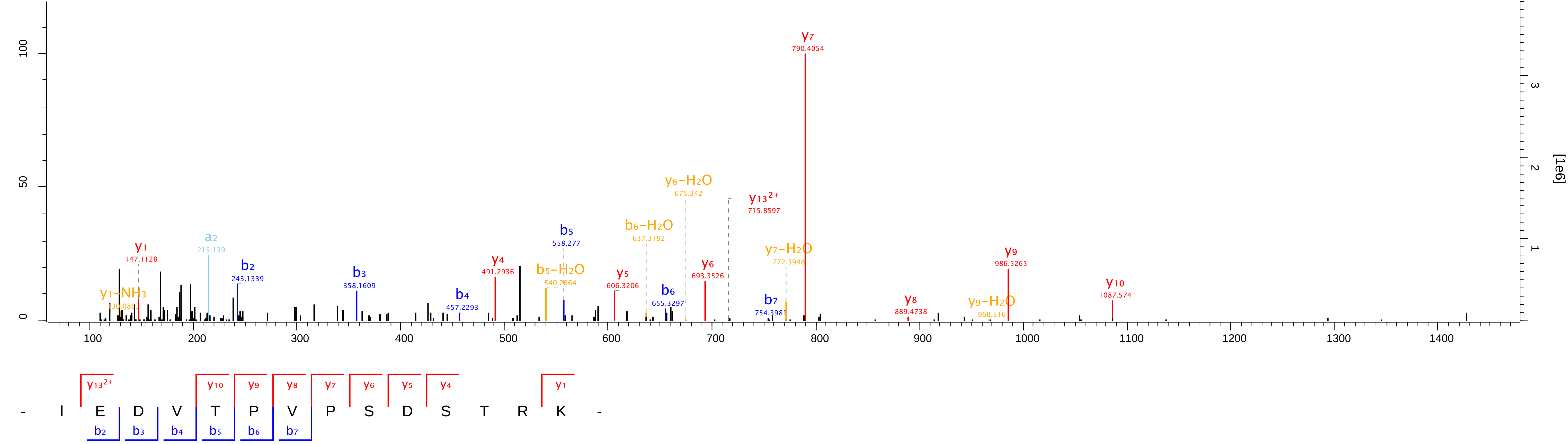

Raw file	Scan	Method	Score	m/z	Gene names
20140827_EXQ00_FaHo_SA_SHG1_01	8310	FTMS; HCD	27.13	596.33	RPL7A;RPL7B

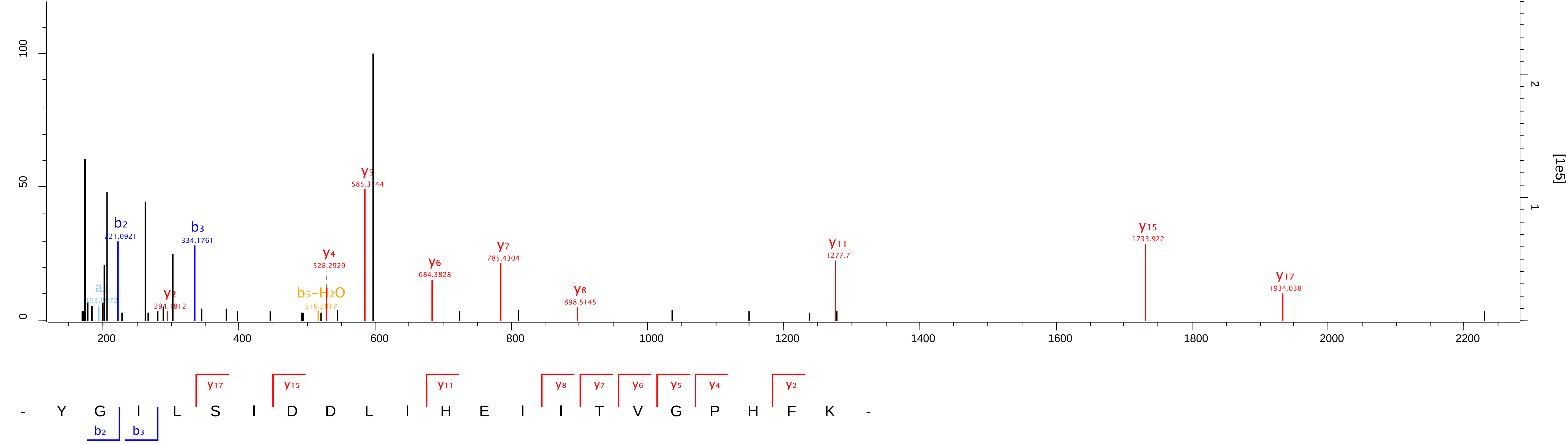

Raw file	Scan	Method	Score	m/z	Gene names
20140827_EXQ00_FaHo_SA_SHG1_02	2962	FTMS; HCD	66.27	494.26	RPS16B;RPS16A

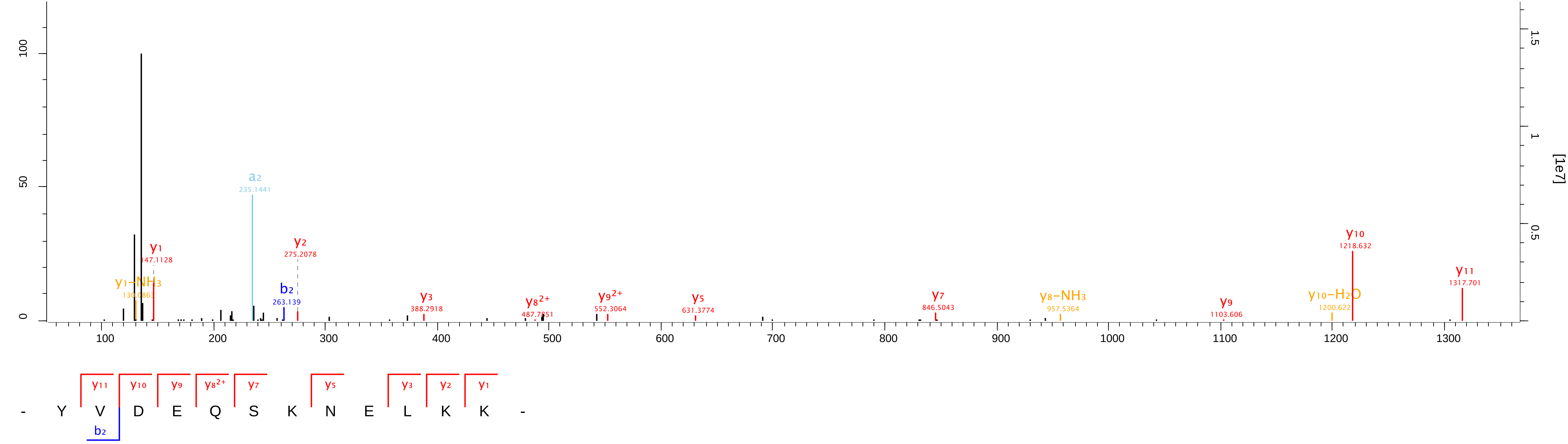

Raw file	Scan	Method	Score	m/z	Gene names
20140827_EXQ00_FaHo_SA_SIR2_01	5060	FTMS; HCD	1.19	803.38	PDR10

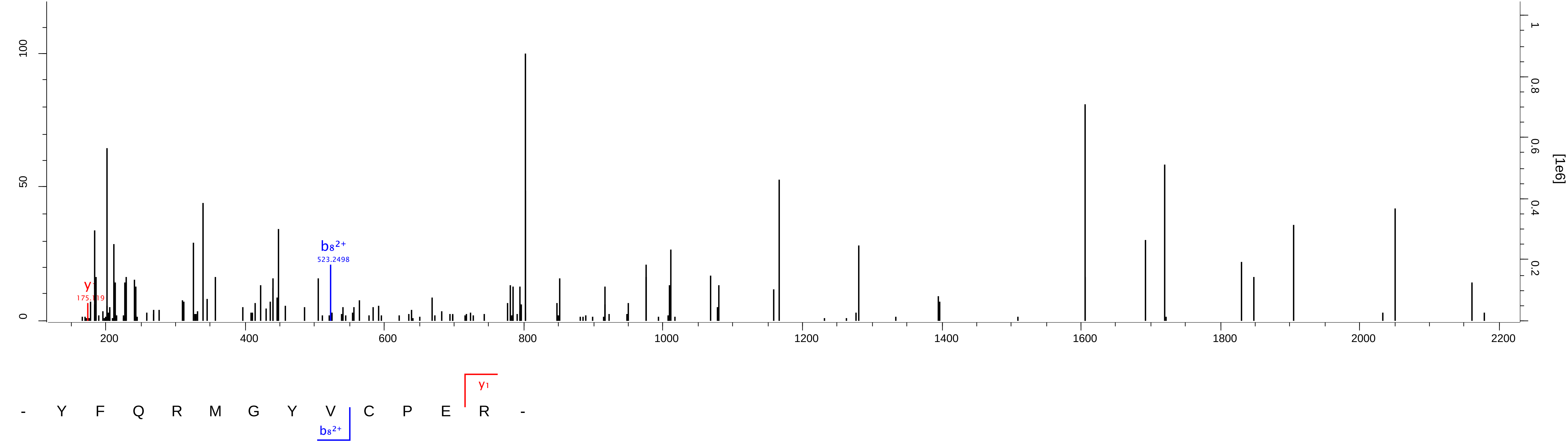

Raw file	Scan	Method	Score	m/z	Gene names
20140827_EXQ00_FaHo_SA_SIR2_02	258	FTMS; HCD	58.92	423.21	TY1A-PR1;TY1A-A;TY1A-DR4;TY1B-H;TY1B-GR2;TY1B-MR2;TY1B-ER2;TY1B-OR;TY1B-BR;TY1B-DR1;TY1B-NL2;TY1B-LR1;TY1B-NL1

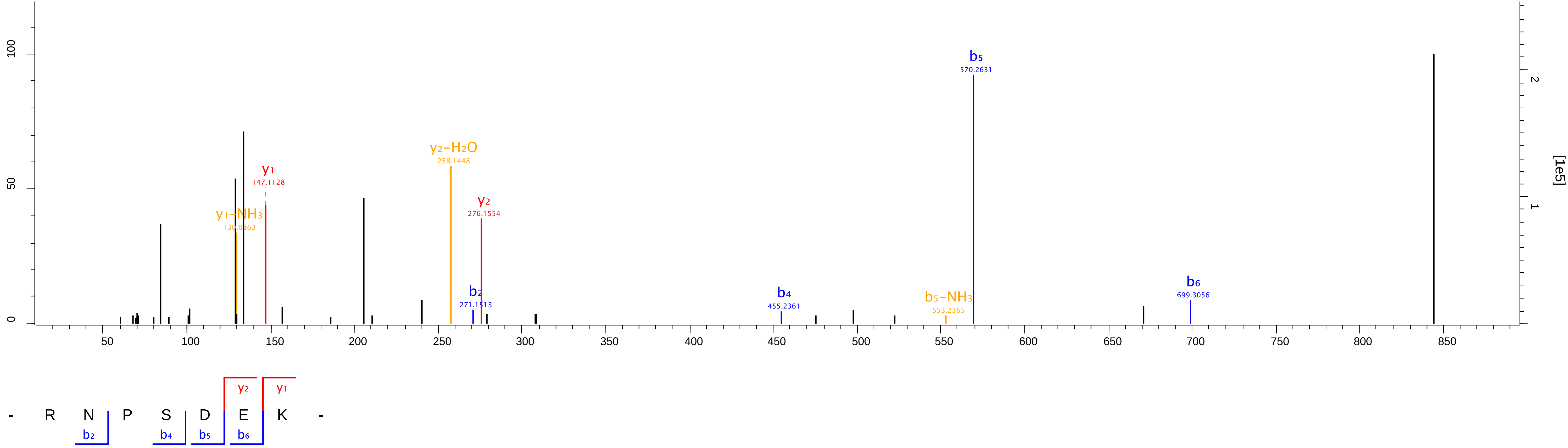

Raw file	Scan	Method	Score	m/z	Gene names
20140827_EXQ00_FaHo_SA_SIR2_(	2143	FTMS; HCD	66.92	464.75	TY2B-B;TY2A-OR1;TY2A-OR2;TY2A-B;TY2A-DR2;TY2A-LR2;TY2A-GR2;TY2A-F;TY2B-GR1;TY2B-OR2;TY2B-DR2;TY2B-GR2;TY2B-F;TY2B-LR2;TY2A-LR1;TY2A-DR1;TY2B-C;TY2A-C;TY2B-OR1;TY2B-DR1;TY2B-DR3;TY2A-DR3;

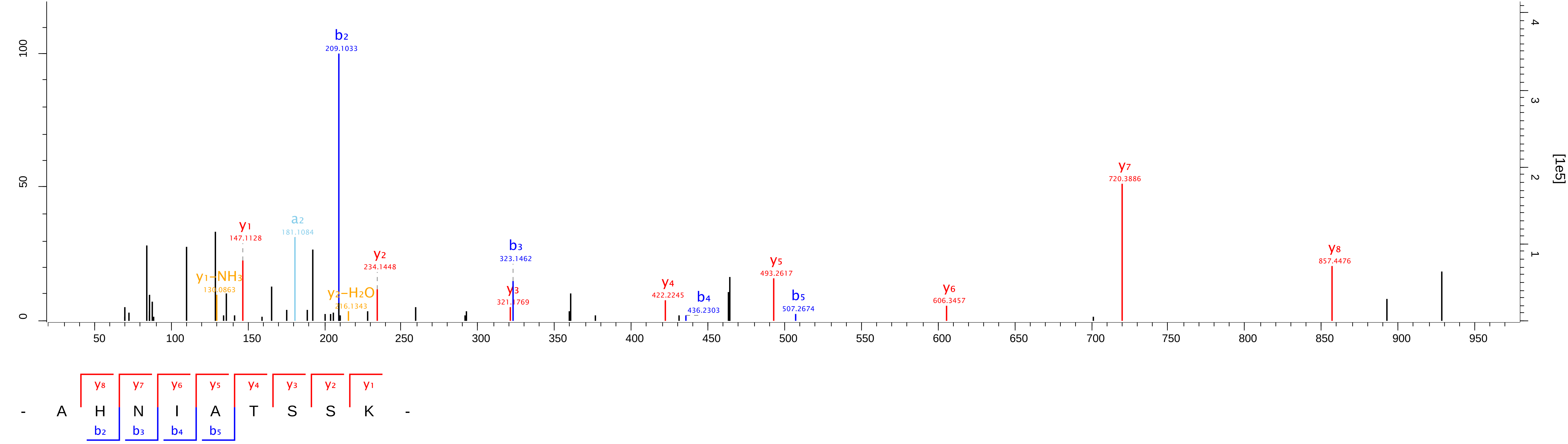

Raw file	Scan	Method	Score	m/z	Gene names
20140827_EXQ00_FaHo_SA_SIR2_0;	2936	FTMS; HCD	209.74	1037.97	TY1B-LR3;TY1A-PL;TY1A-LR2;TY1A-ER1;TY1A-DR6;TY1B-OL;TY1B-LR4;TY1B-LR2;TY1B-PL;TY1B-ER1;TY1B-PR3;TY1A-PR1;TY1A-A;TY1A-DR4;TY1B-H;TY1B-GR2;TY1B-MR2;TY1B-ER2;TY1B-OR;TY1B-BR;TY1B-DR1;TY1B-I

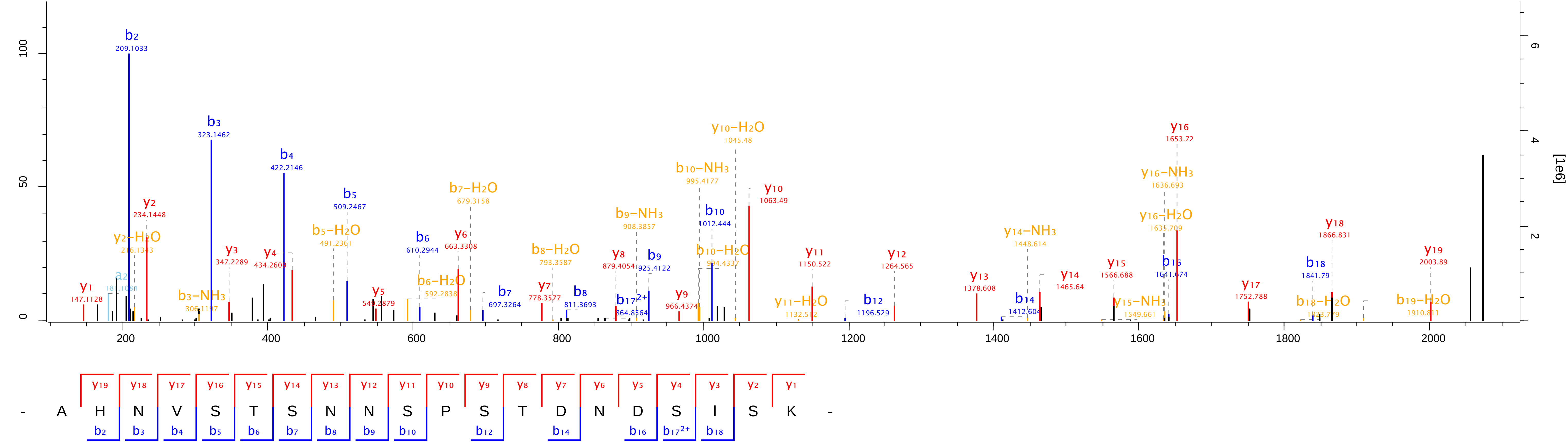

Raw file	Scan	Method	Score	m/z	Gene names
20140827_EXQ00_FaHo_SA_SIR2_02	3971	FTMS; HCD	123.02	559.25	SSA1;SSA2;SSA3

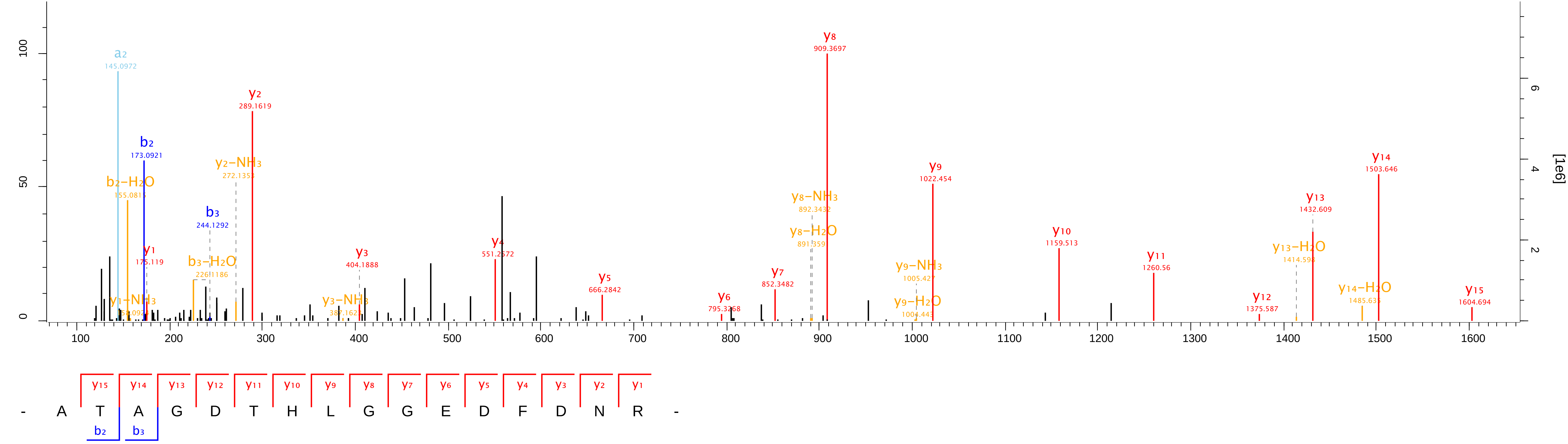

Raw file	Scan	Method	Score	m/z	Gene names
20140827_EXQ00_FaHo_SA_SIR2_02	4109	FTMS; HCD	120.45	605.83	TY1B-NL1;TY1B-BL;TY1B-MR1;TY1A-MR1

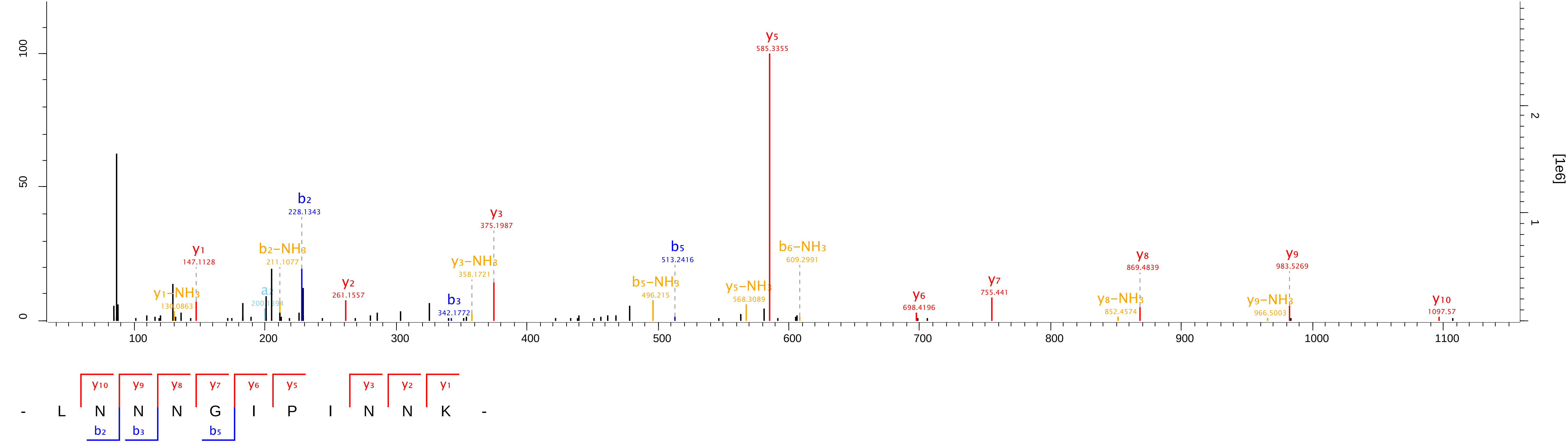

Raw file	Scan	Method	Score	m/z	Gene names
20140827_EXQ00_FaHo_SA_SIR2_02	4239	FTMS; HCD	50.13	446.71	TY1B-LR3;TY1B-OL;TY1B-LR4;TY1B-LR2;TY1B-PL;TY1B-ER1;TY1B-PR3;TY1B-H;TY1B-GR2;TY1B-MR2;TY1B-ER2;TY1B-OR;TY1B-BR;TY1B-DR1;TY1B-NL2;TY1B-LR1;TY1B-DR3;TY1B-NL1;TY1B-A;TY1B-BL;TY1B-MR1

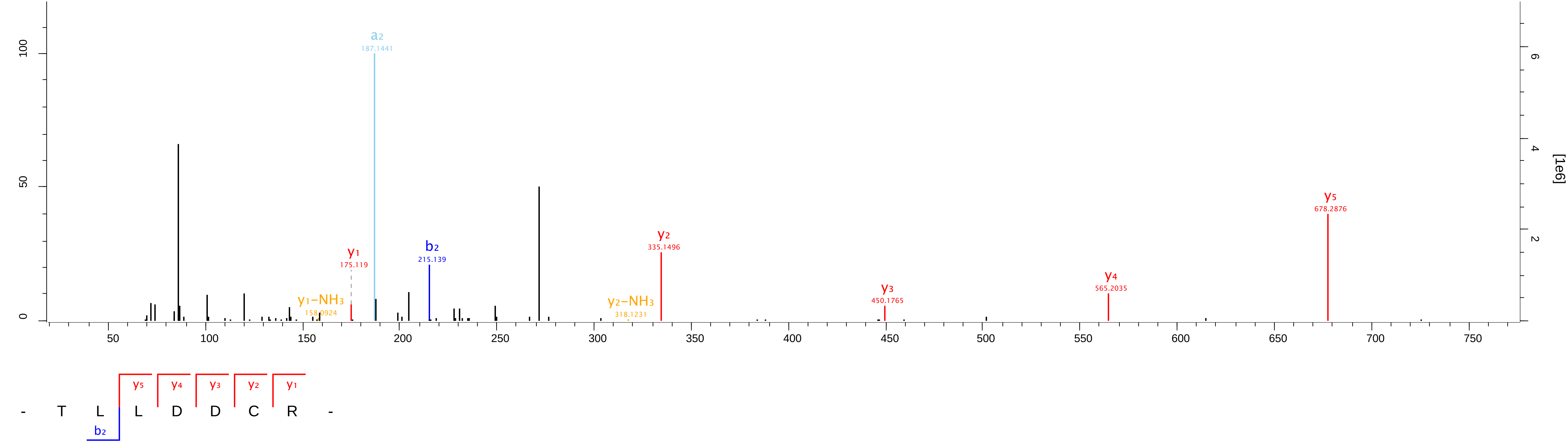

Raw file	Scan	Method	Score	m/z	Gene names
20140827_EXQ00_FaHo_SA_SIR2_(	4299	FTMS; HCD	106.4	622.83	TY1B-LR3;TY1A-PL;TY1A-LR2;TY1A-ER1;TY1A-DR6;TY1B-OL;TY1B-LR4;TY1B-LR2;TY1B-PL;TY1B-ER1;TY1B-PR3;TY1A-PR1;TY1A-A;TY1A-DR4;TY1B-H;TY1B-GR2;TY1B-MR2;TY1B-ER2;TY1B-OR;TY1B-BR;TY1B-DR1;TY1B-NL2

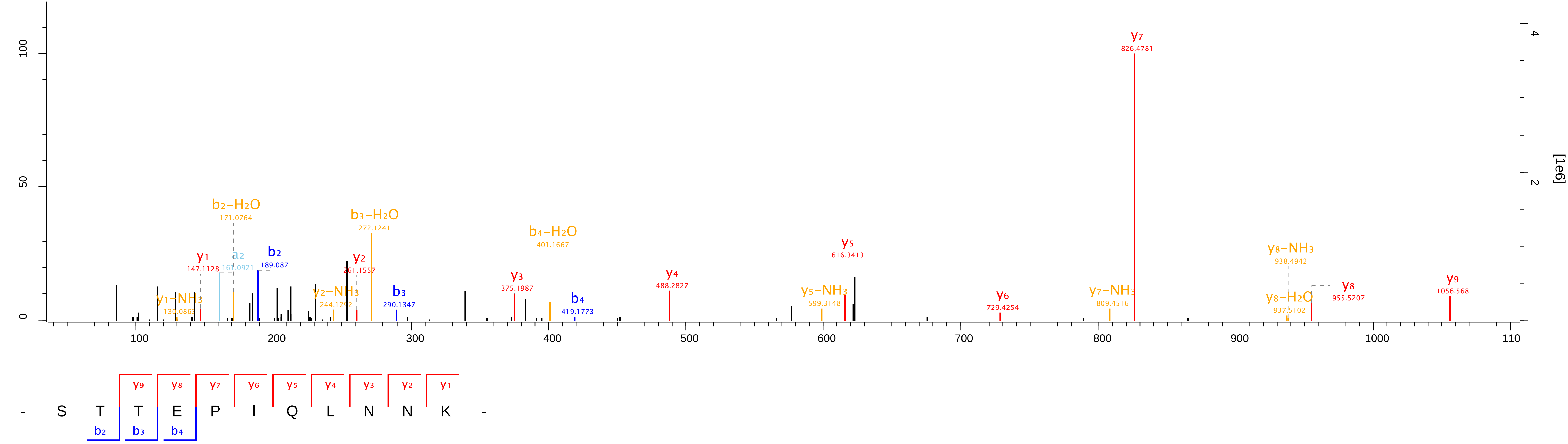

Raw file	Scan	Method	Score	m/z	Gene names
20140827_EXQ00_FaHo_SA_SIR2_02	5452	FTMS; HCD	89.55	512.76	TY1B-NL1;TY1B-BL;TY1B-MR1;TY1A-DR2;TY1A-MR1

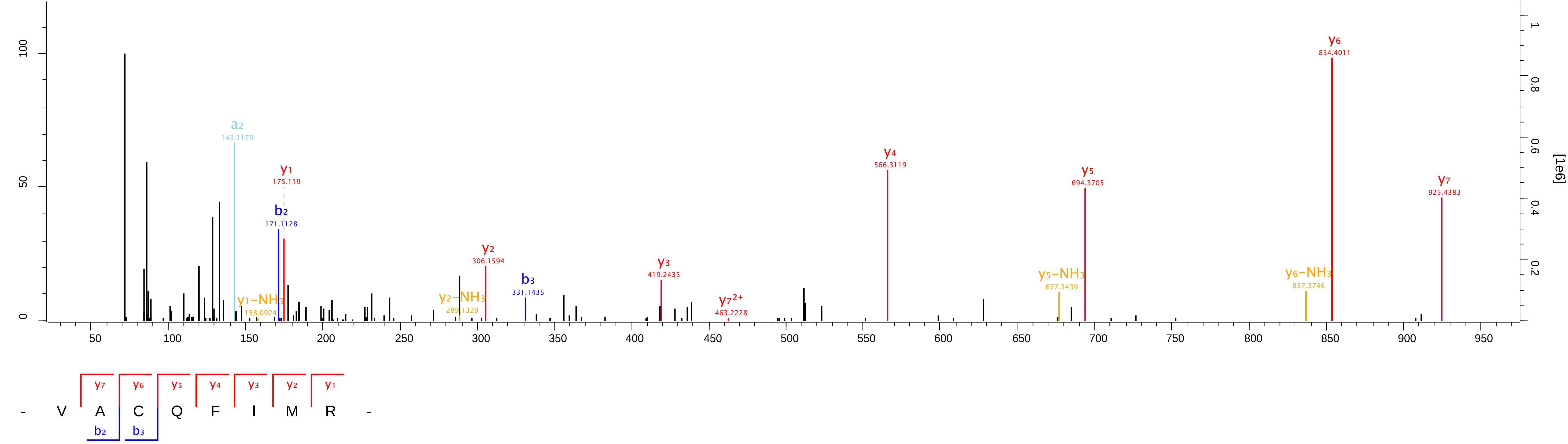

Raw file	Scan	Method	Score	m/z	Gene names
20140827_EXQ00_FaHo_SA_SIR2_02	5601	FTMS; HCD	105.38	1065.02	TY1B-LR3;TY1B-LR4;TY1B-LR2;TY1B-PL;TY1B-ER1;TY1B-PR3;TY1B-MR2;TY1B-ER2;TY1B-OR;TY1B-BR;TY1B-NL2;TY1B-NL1;TY1B-BL;TY1B-MR1

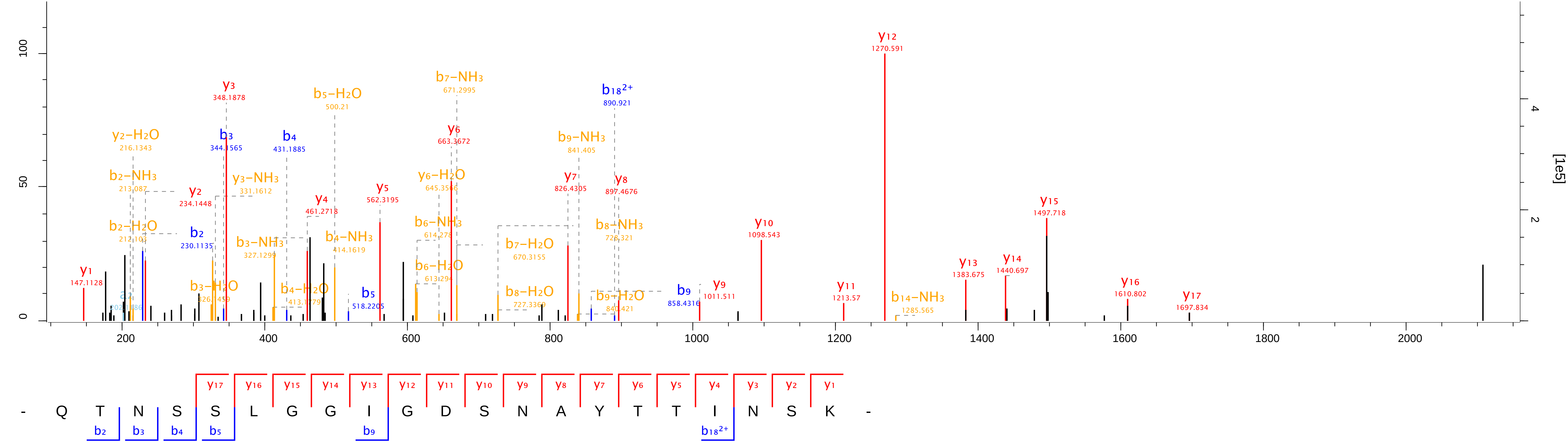

Raw file	Scan	Method	Score	m/z	Gene names
20140827_EXQ00_FaHo_SA_SIR2_(	2290	FTMS; HCD	152.37	520.27	TY1B-LR3;TY1A-PL;TY1A-LR2;TY1A-ER1;TY1A-DR6;TY1B-OL;TY1B-LR4;TY1B-LR2;TY1B-PL;TY1B-ER1;TY1B-PR3;TY1A-PR1;TY1A-A;TY1A-DR4;TY1B-H;TY1B-GR2;TY1B-MR2;TY1B-ER2;TY1B-OR;TY1B-BR;TY1B-DR1;TY1B-NL2

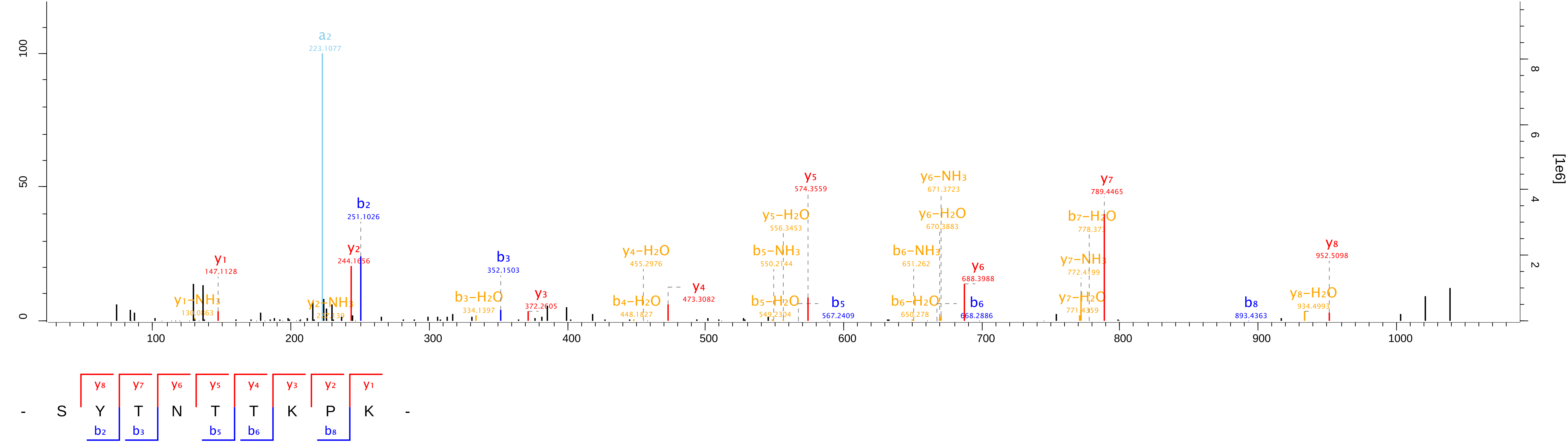

Raw file	Scan	Method	Score	m/z	Gene names
20140827_EXQ00_FaHo_SA_SIR2_03	5595	FTMS; HCD	135.1	726.89	TY1B-LR3;TY1B-OL;TY1B-LR4;TY1B-LR2;TY1B-PL;TY1B-ER1;TY1B-PR3;TY1B-H;TY1B-GR2;TY1B-MR2;TY1B-ER2;TY1B-OR;TY1B-BR;TY1B-DR1;TY1B-NL2;TY1B-LR1;TY1B-DR3;TY1B-NL1;TY1B-A;TY1B-BL;TY1B-MR1

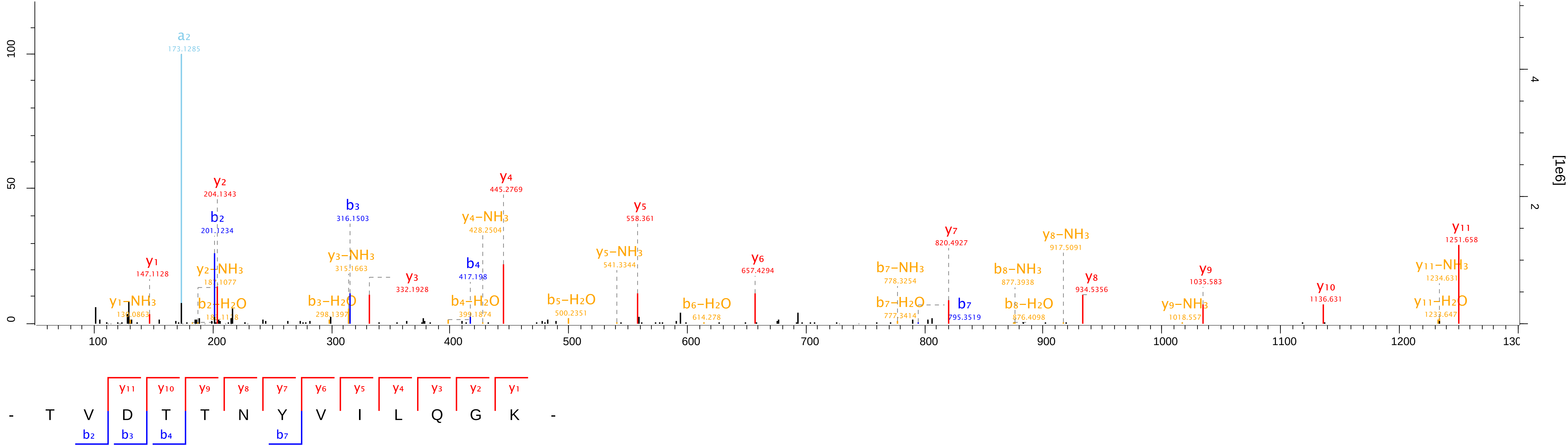

Raw file	Scan	Method	Score	m/z	Gene names
20140827_EXQ00_FaHo_SA_SIR2_03	5676	FTMS; HCD	112.96	821.42	TY1B-LR3;TY1B-OL;TY1B-LR4;TY1B-LR2;TY1B-PL;TY1B-ER1;TY1B-PR3;TY1B-H;TY1B-GR2;TY1B-MR2;TY1B-ER2;TY1B-OR;TY1B-BR;TY1B-DR1;TY1B-LR1;TY1B-DR3;TY1B-A;TY1B-BL;TY1B-MR1

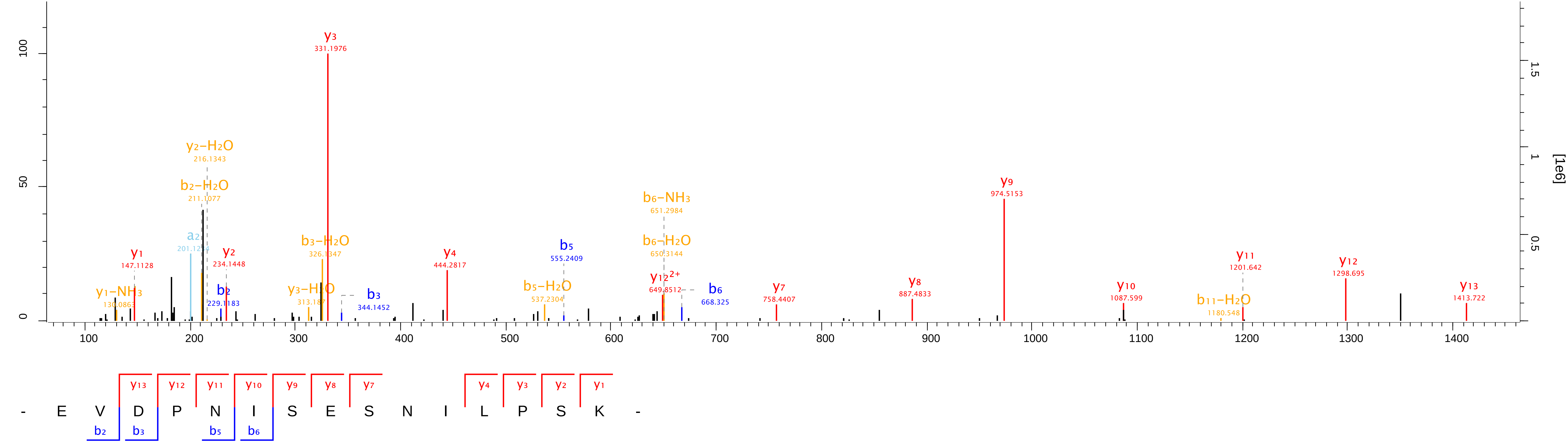

Raw file	Scan	Method	Score	m/z	Gene names
20140827_EXQ00_FaHo_SA_SNF2_01	6877	FTMS; HCD	96.02	727.68	RPS14B;RPS14A

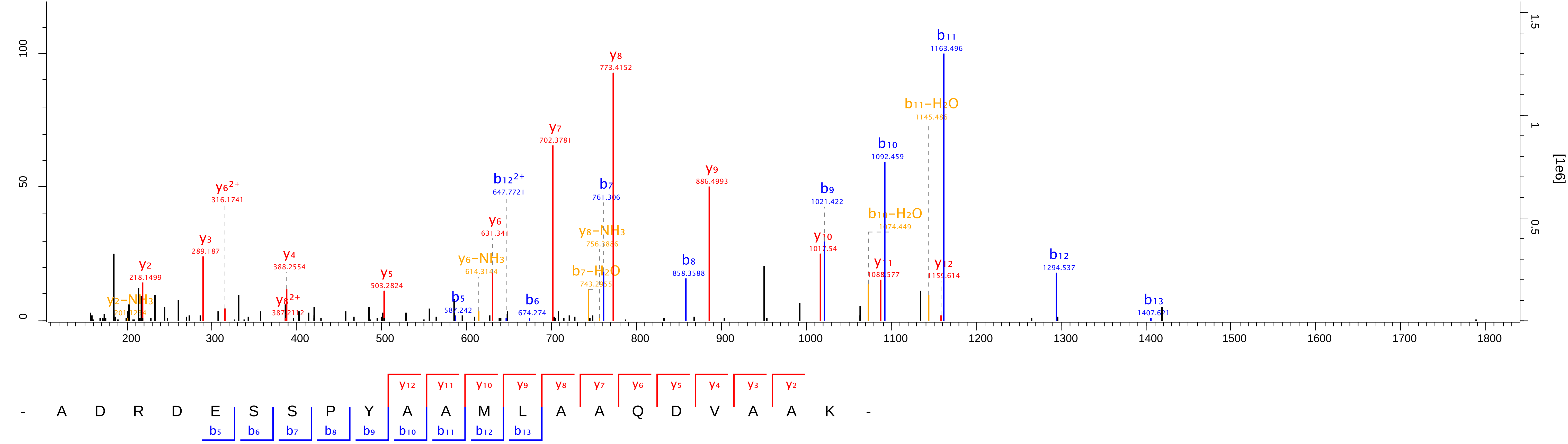

Raw file	Scan	Method	Score	m/z	Gene names
20140827_EXQ00_FaHo_SA_SNF2_02	2995	FTMS; HCD	61.34	422.21	GUK1

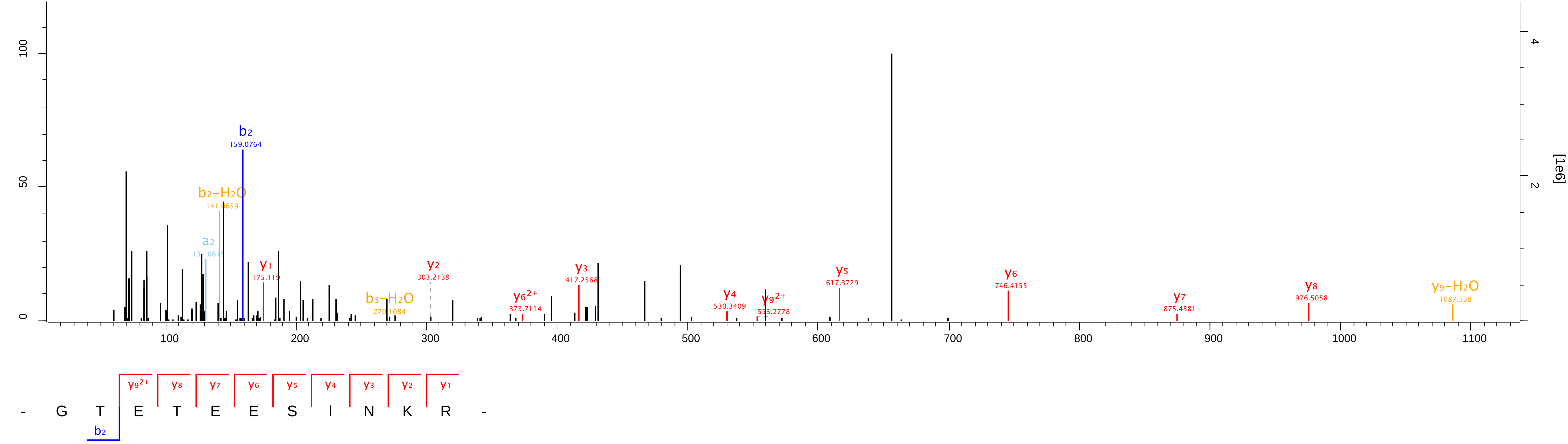

Raw file	Scan	Method	Score	m/z	Gene names
20140827_EXQ00_FaHo_SA_SNF2_02	5411	FTMS; HCD	179.1	546.77	RPS14B;RPS14A

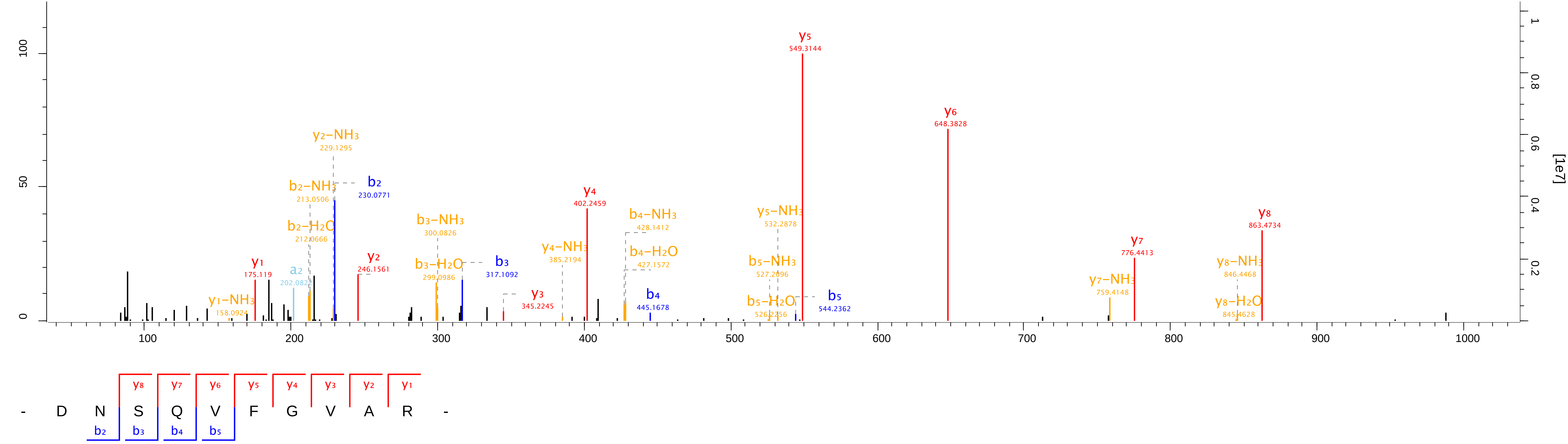

Raw file	Scan	Method	Score	m/z	Gene names
20140827_EXQ00_FaHo_SA_SNF2_03	3980	FTMS; HCD	132.08	423.73	RPL24A;RPL24B

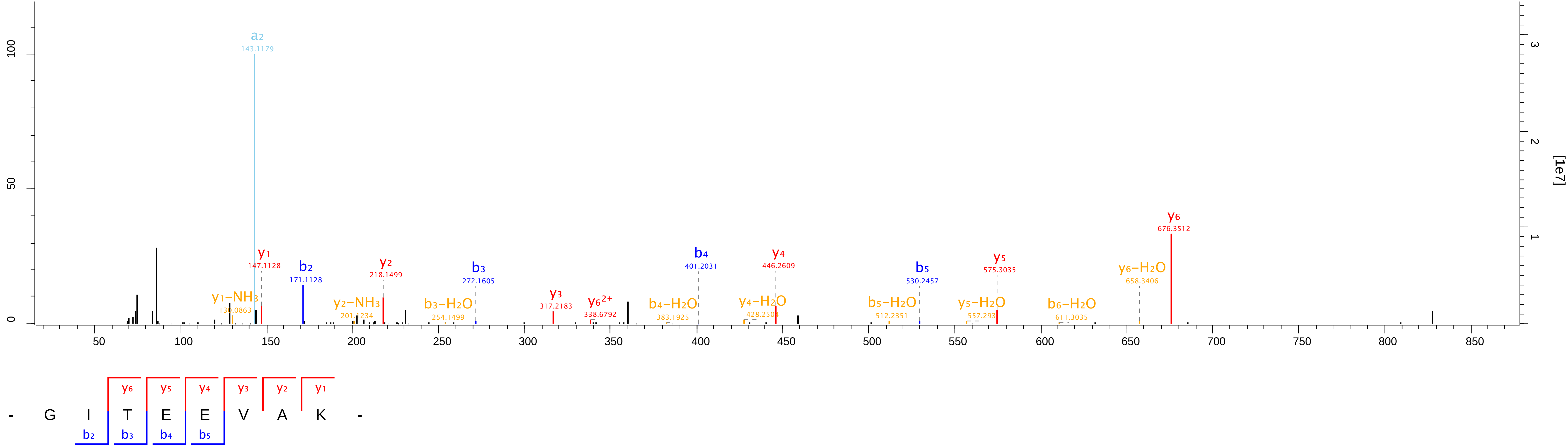

Raw file	Scan	Method	Score	m/z	Gene names
20140827_EXQ00_FaHo_SA_SNF2_03	5283	FTMS; HCD	61.78	514.8	RPL7A;RPL7B

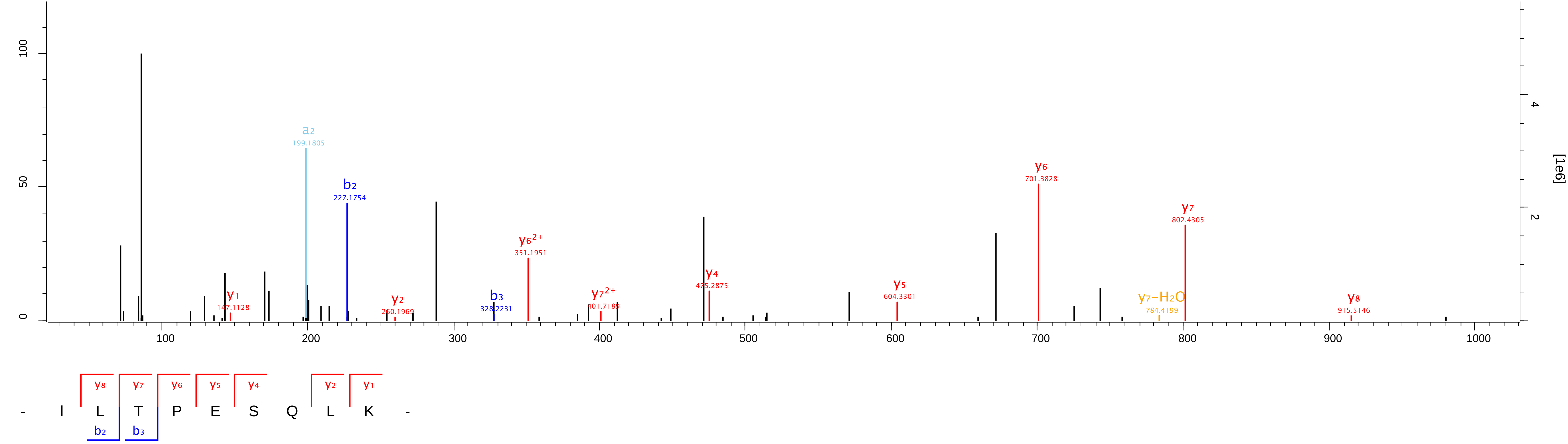

Raw file	Scan	Method	Score	m/z	Gene names
20140827_EXQ00_FaHo_SA_SNF2_03	5852	FTMS; HCD	146.11	567.79	RPL36A;RPL36B

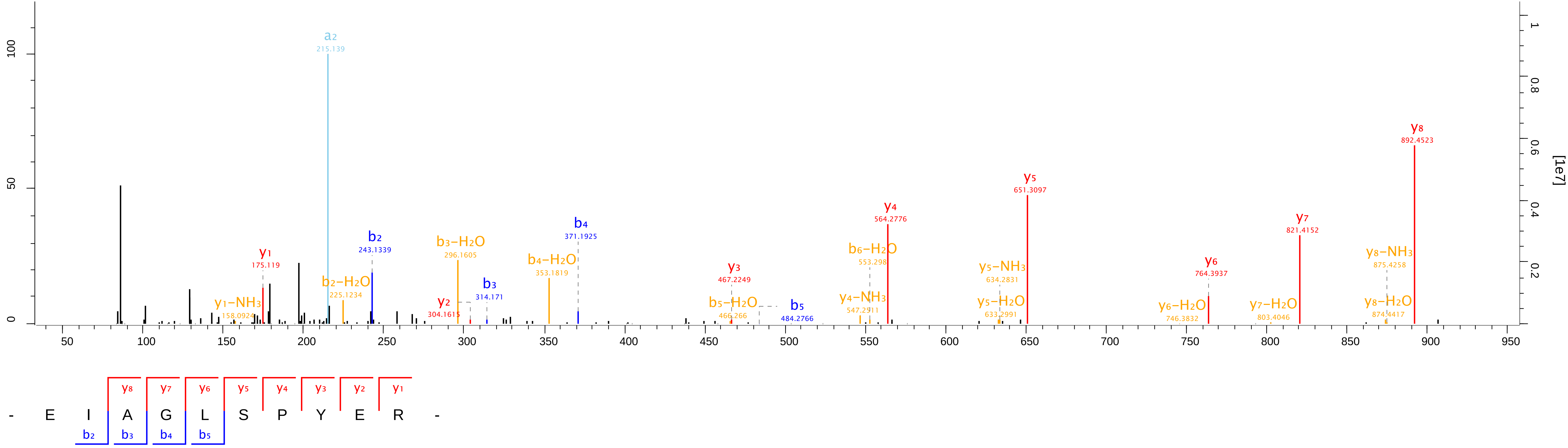

Raw file	Scan	Method	Score	m/z	Gene names
20140827_EXQ00_FaHo_SA_SNF2_03	6827	FTMS; HCD	166.92	816.89	ARF1;ARF2

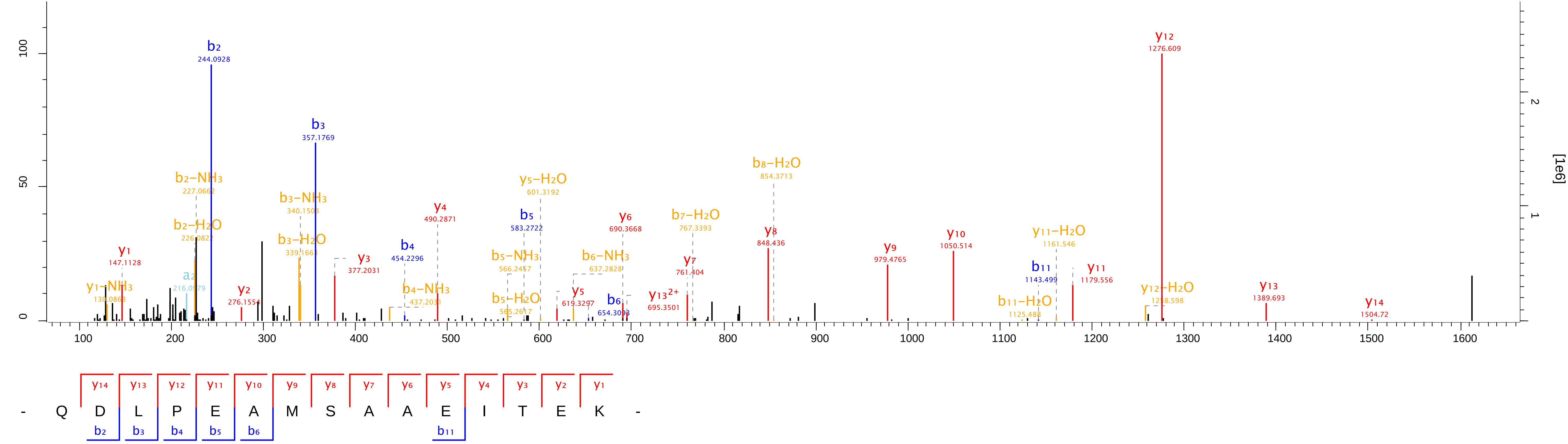

Raw file	Scan	Method	Score	m/z	Gene names
20140827_EXQ00_FaHo_SA_SPP1_01	3643	FTMS; HCD	158.58	786.87	RPL7A;RPL7B

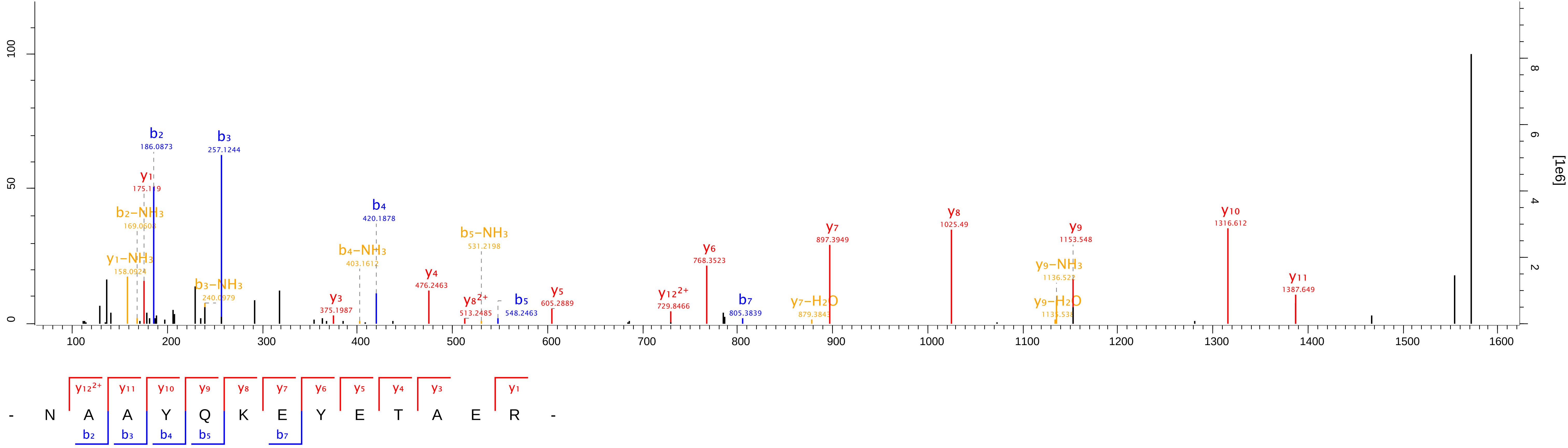

Raw file	Scan	Method	Score	m/z	Gene names
20140827_EXQ00_FaHo_SA_SPP1_01	5229	FTMS; HCD	160.18	643.83	RPL17B

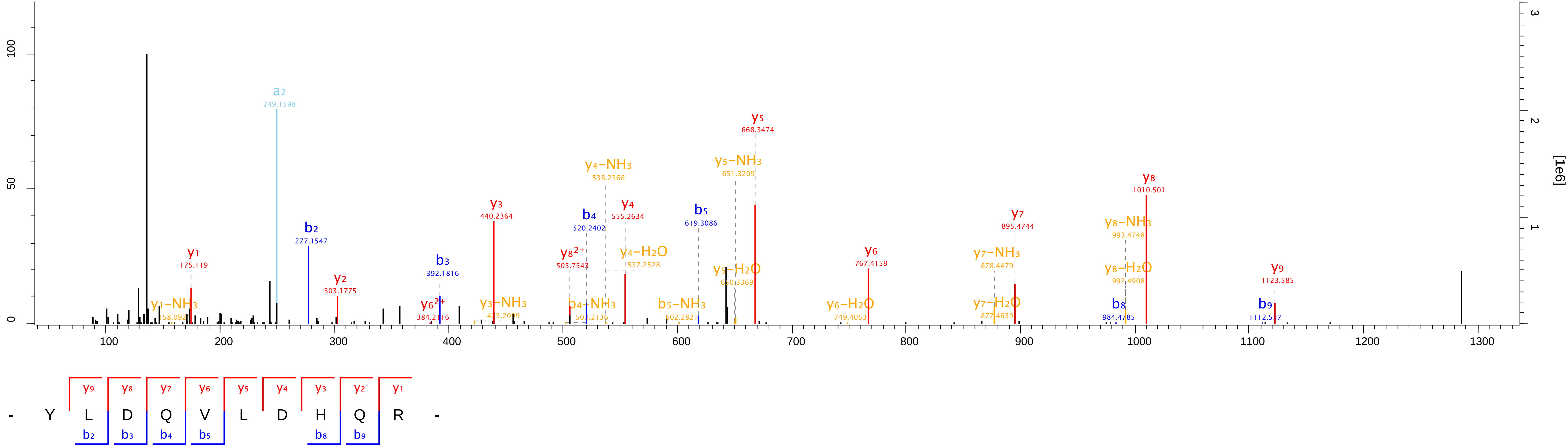

Raw file	Scan	Method	Score	m/z	Gene names
20140827_EXQ00_FaHo_SA_SPP1_01	6414	FTMS; HCD	118.51	808.85	CAF20

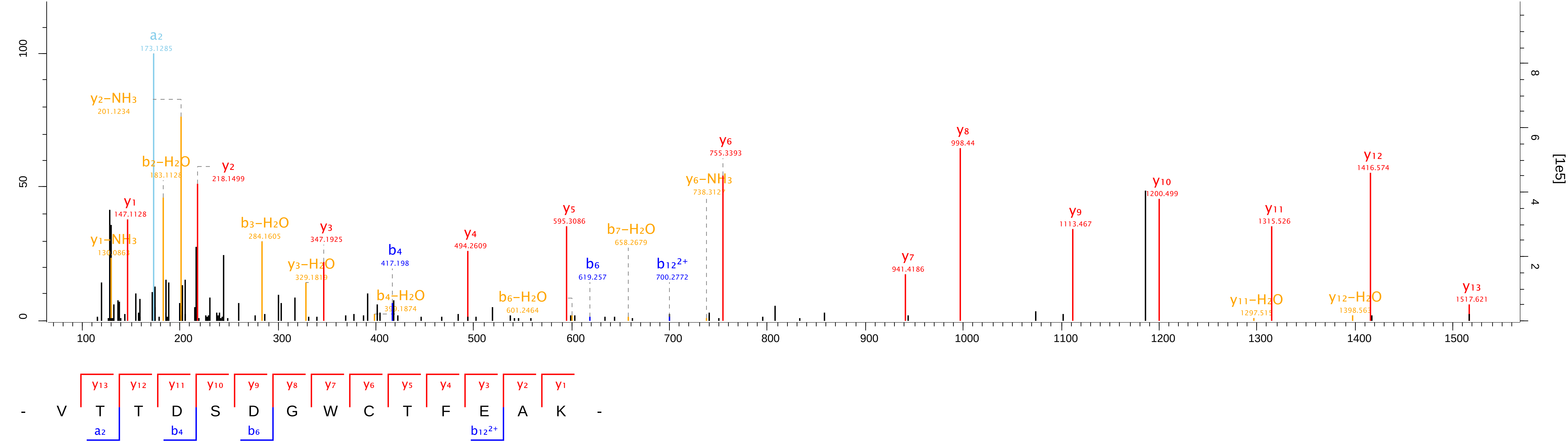

Raw file	Scan	Method	Score	m/z	Gene names
20140827_EXQ00_FaHo_SA_SPP1_01	6441	FTMS; HCD	66.25	544.3	RPL4B;RPL4A

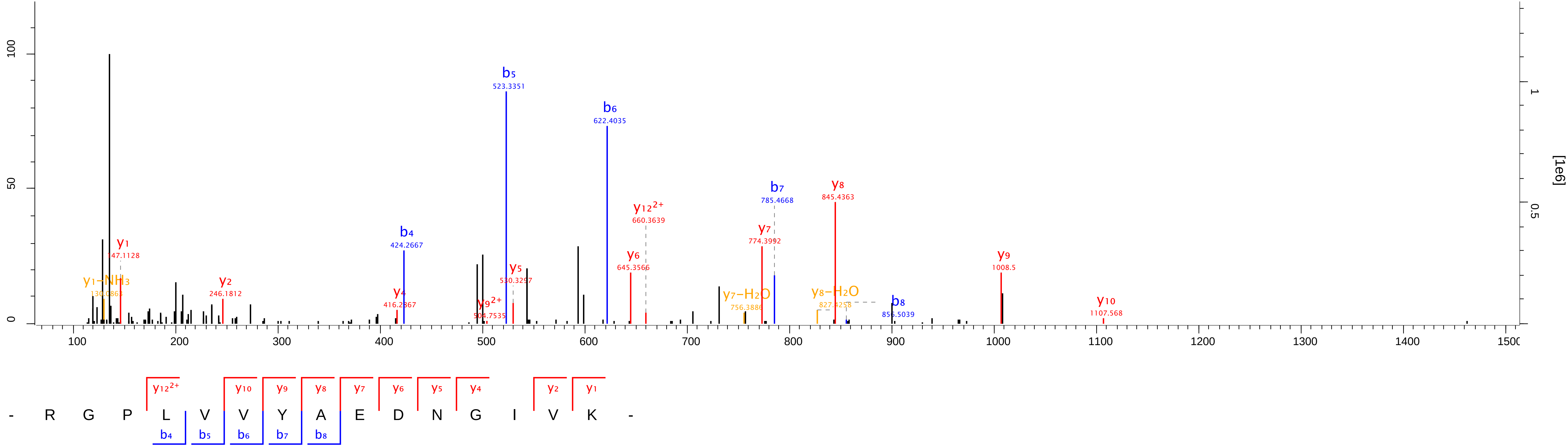

Raw file	Scan	Method	Score	m/z	Gene names
20140827_EXQ00_FaHo_SA_SPP1_02	4370	FTMS; HCD	103.83	433.24	RPL7A;RPL7B

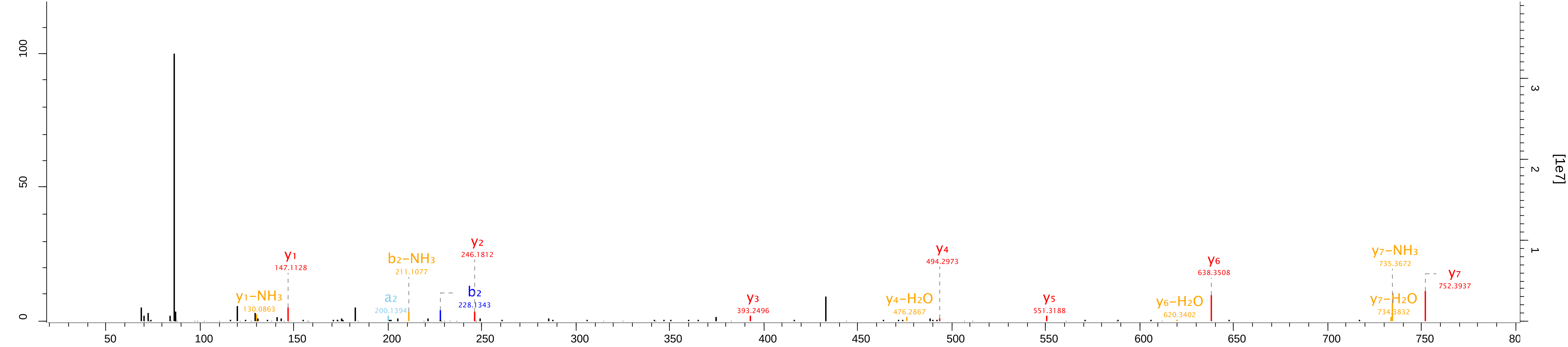

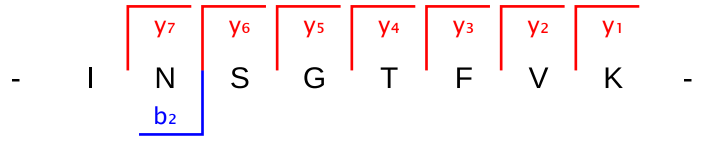

Raw file	Scan	Method	Score	m/z	Gene names
20140827_EXQ00_FaHo_SA_SPP1_02	4436	FTMS; HCD	125.57	490.77	RPL21A;RPL21B

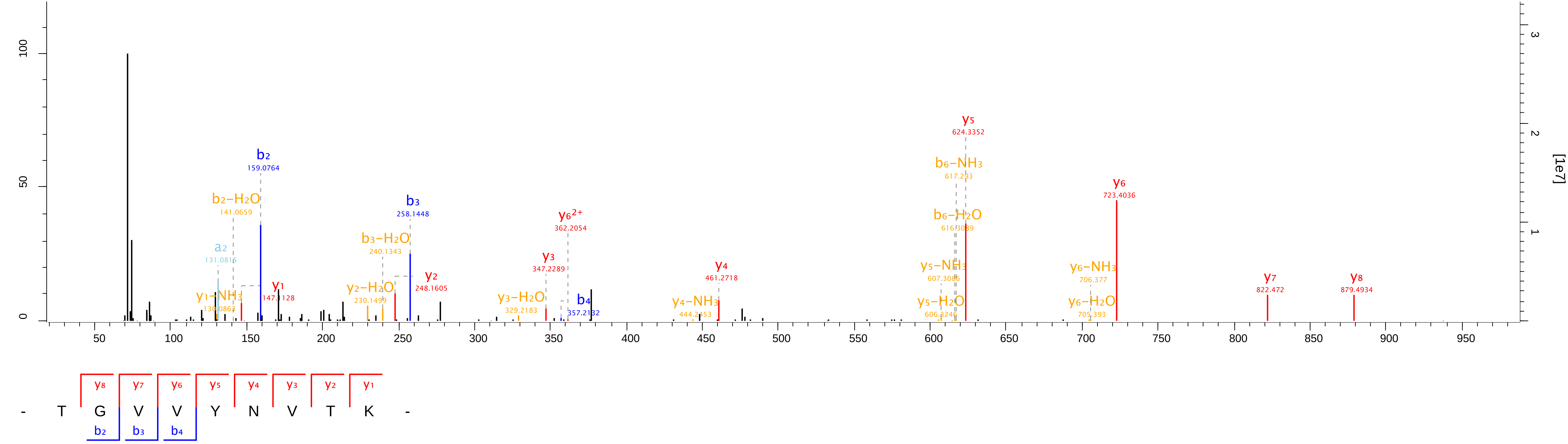

Raw file	Scan	Method	Score	m/z	Gene names
20140827_EXQ00_FaHo_SA_SPP1_02	8295	FTMS; HCD	121.5	885.76	ATG33

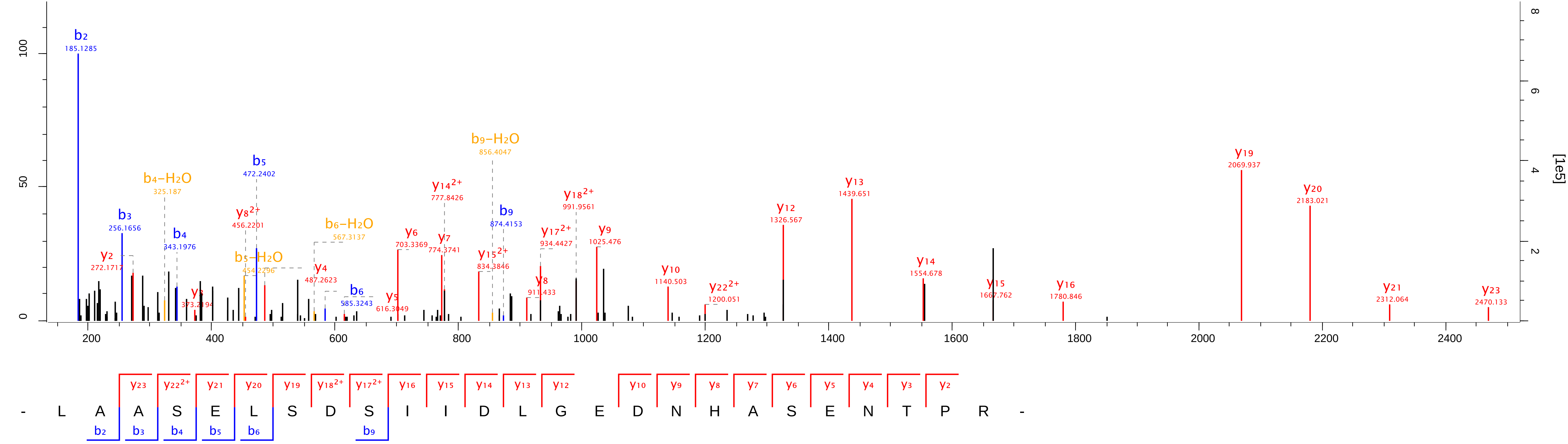

Raw file	Scan	Method	Score	m/z	Gene names
20140827_EXQ00_FaHo_SA_SPP1_03	4347	FTMS; HCD	73.23	470.28	TRX2;TRX1

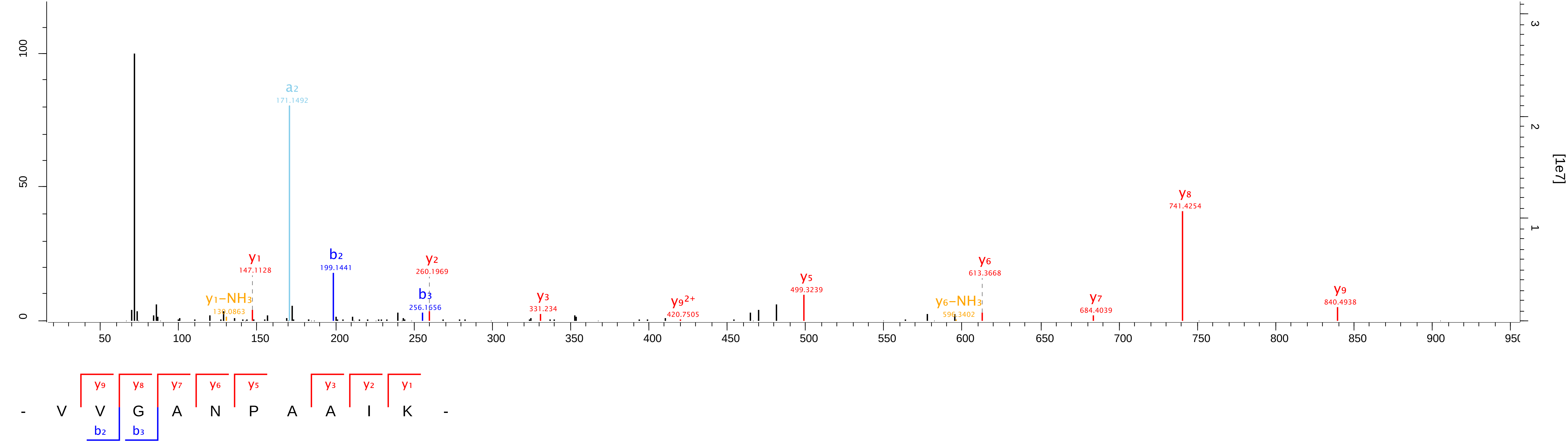

Raw file	Scan	Method	Score	m/z	Gene names
20140827_EXQ00_FaHo_SA_SPP1_03	5158	FTMS; HCD	98.25	761.38	RDL1

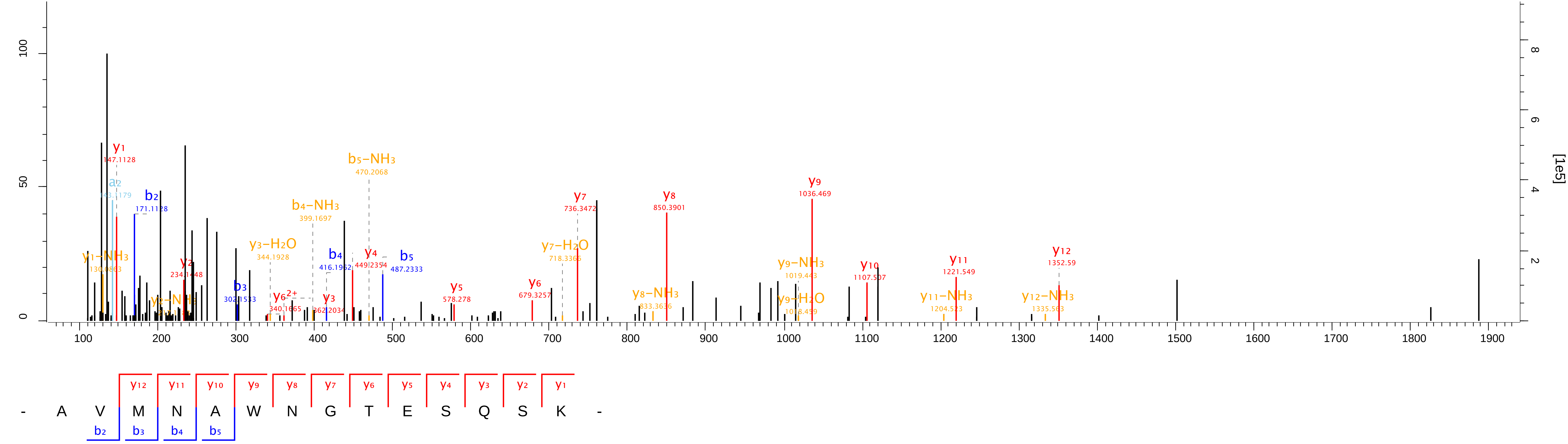

Raw file	Scan	Method	Score	m/z	Gene names
20140827_EXQ00_FaHo_SA_SPP1_03	5802	FTMS; HCD	120.84	703.34	NHP6B

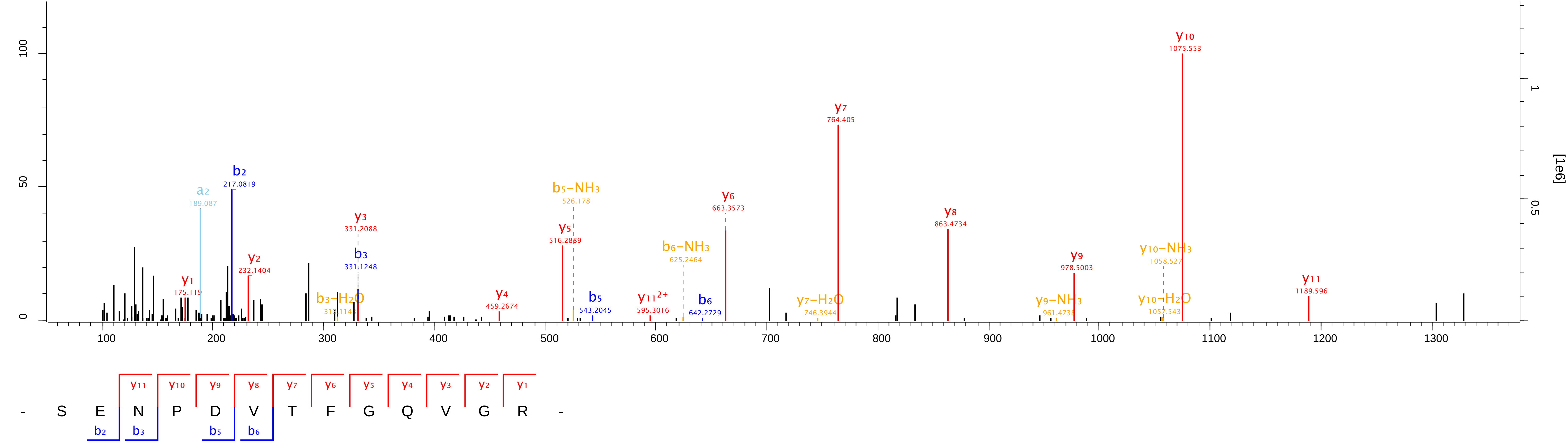

Raw file	Scan	Method	Score	m/z	Gene names
20140827_EXQ00_FaHo_SA_SPP1_03	7170	FTMS; HCD	72.32	840.41	ARP2

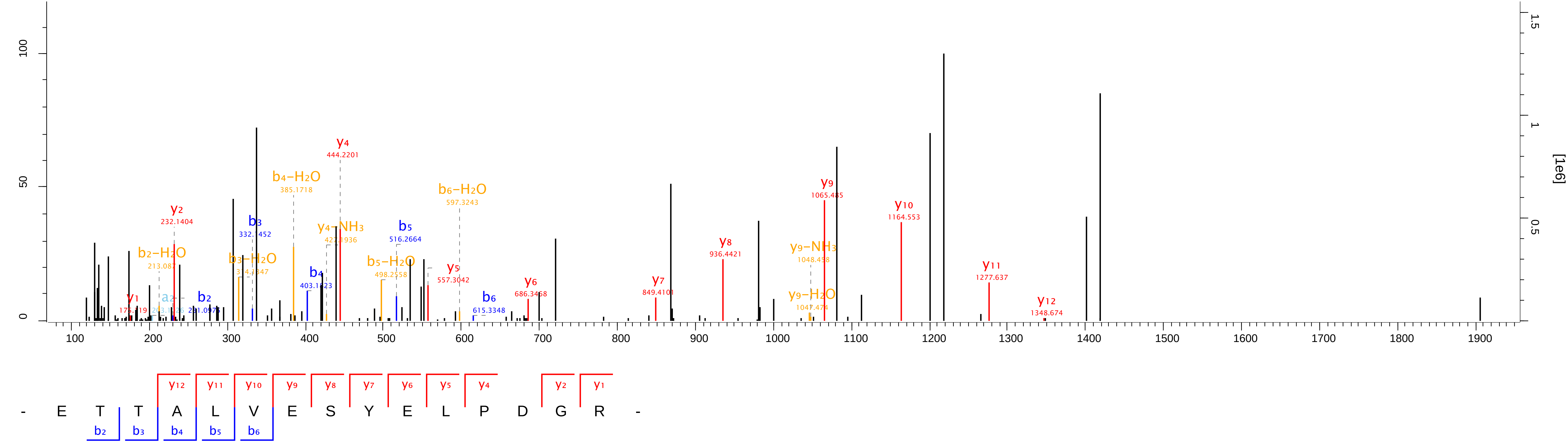

Raw file	Scan	Method	Score	m/z	Gene names
20140827_EXQ00_FaHo_SA_SPP1_03	8286	FTMS; HCD	60.66	1036.93	RPL22A

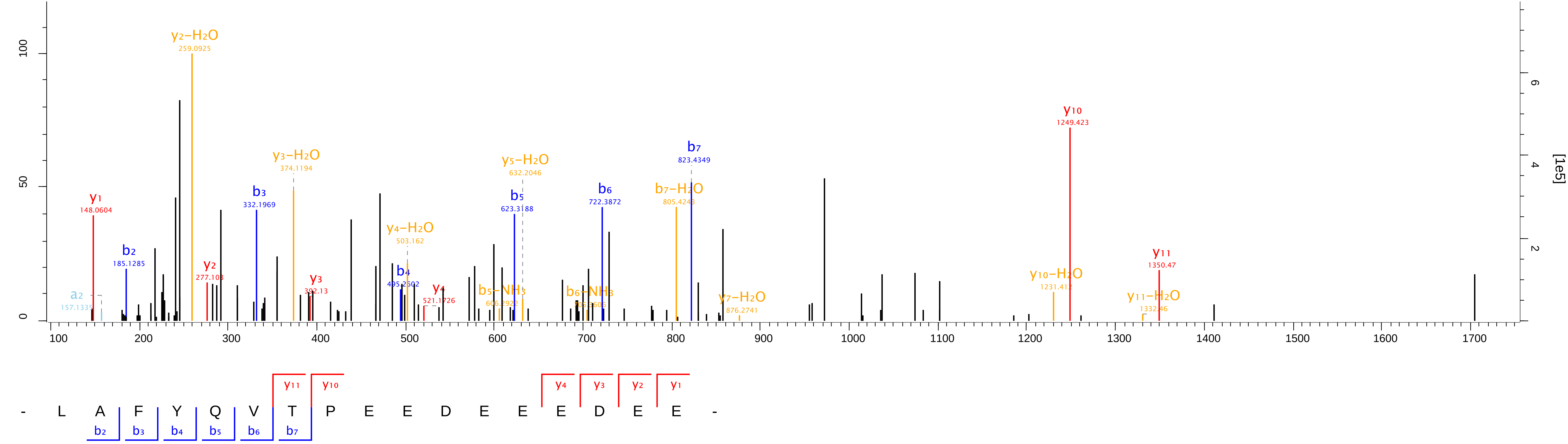

Raw file	Scan	Method	Score	m/z	Gene names
20140827_EXQ00_FaHo_SA_SPP1_03	8342	FTMS; HCD	180.11	789.89	ARF2

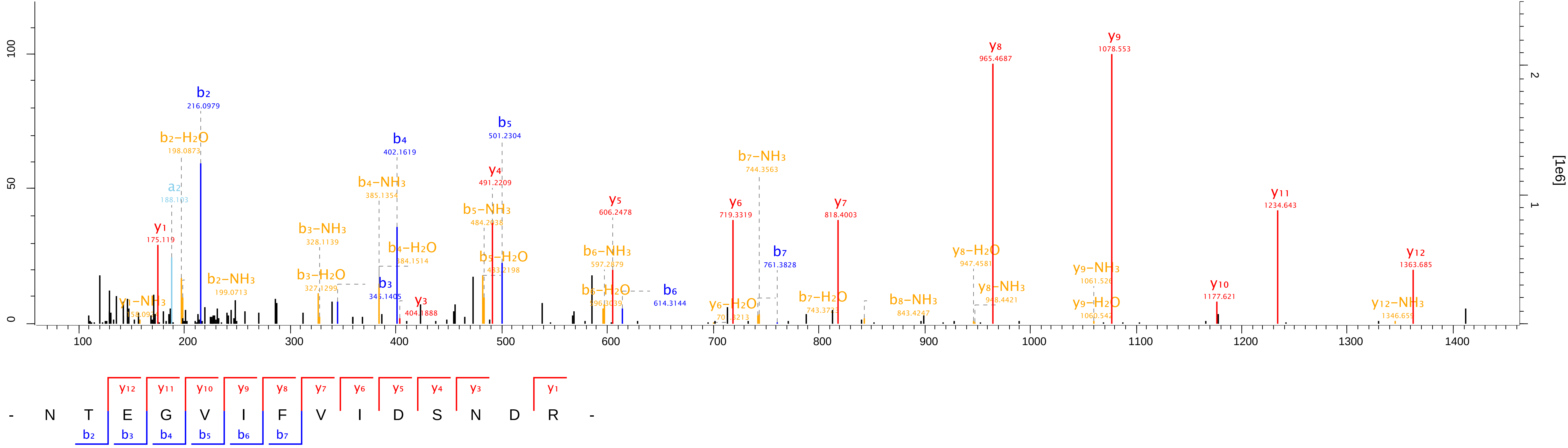

Raw file	Scan	Method	Score	m/z	Gene names
20140827_EXQ00_FaHo_SA_SPT7_01	4380	FTMS; HCD	146.36	530.6	PDR5;PDR15;PDR10

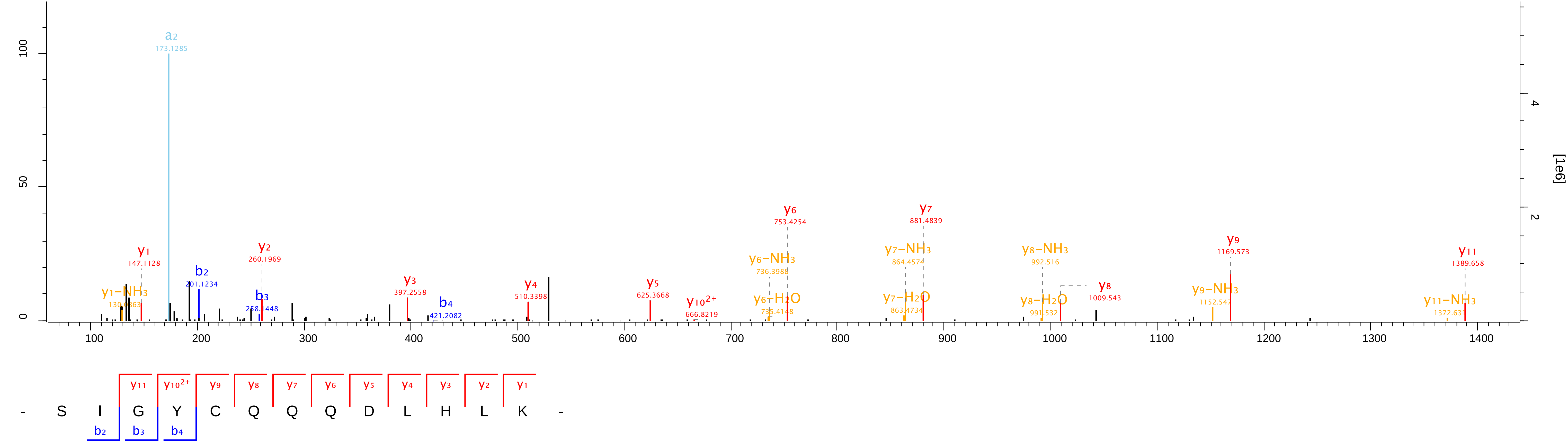

Raw file	Scan	Method	Score	m/z	Gene names
20140827_EXQ00_FaHo_SA_SPT7_02	2617	FTMS; HCD	107.11	591.8	TY1B-H;TY1B-GR2;TY1B-MR2;TY1B-ER2;TY1B-OR;TY1B-BR;TY1B-DR1;TY1B-NL2;TY1B-LR1;TY1B-DR3;TY1B-NL1

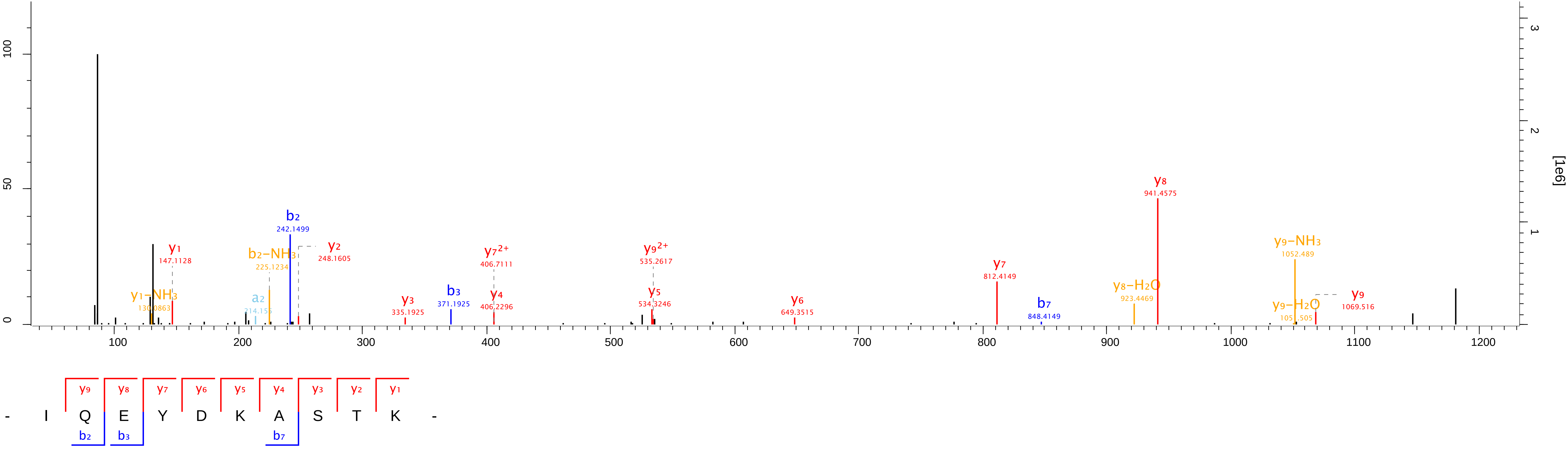

Raw file	Scan	Method	Score	m/z	Gene names
20140827_EXQ00_FaHo_SA_SPT7_02	3250	FTMS; HCD	90.15	584.29	FMP10

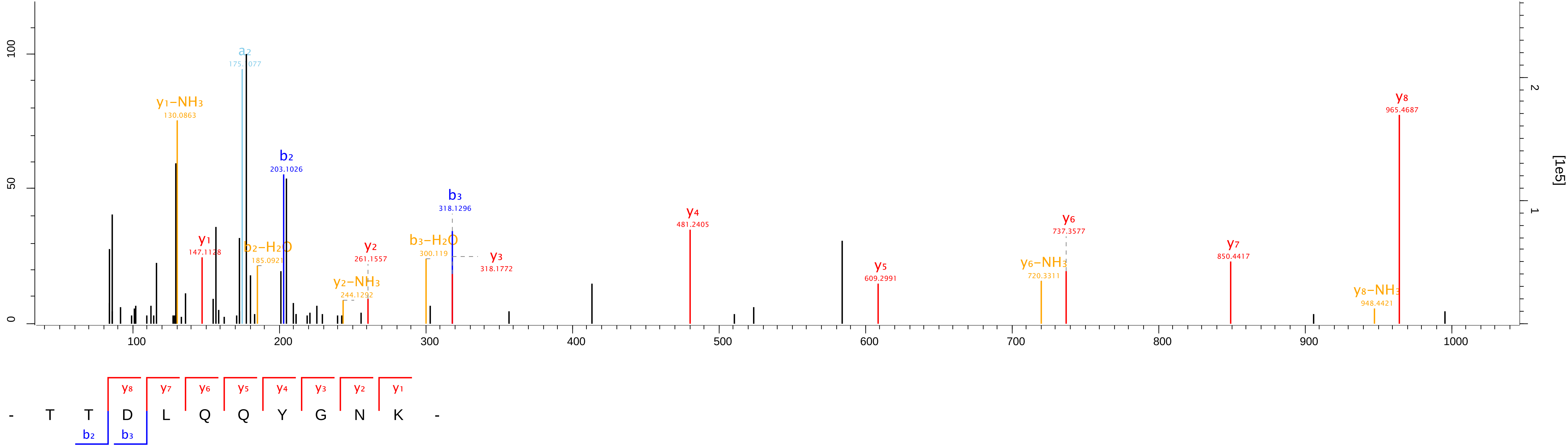

Raw file	Scan	Method	Score	m/z	Gene names
20140827_EXQ00_FaHo_SA_SPT7_02	3647	FTMS; HCD	81.3	403.71	PDR12;PDR10;SNQ2

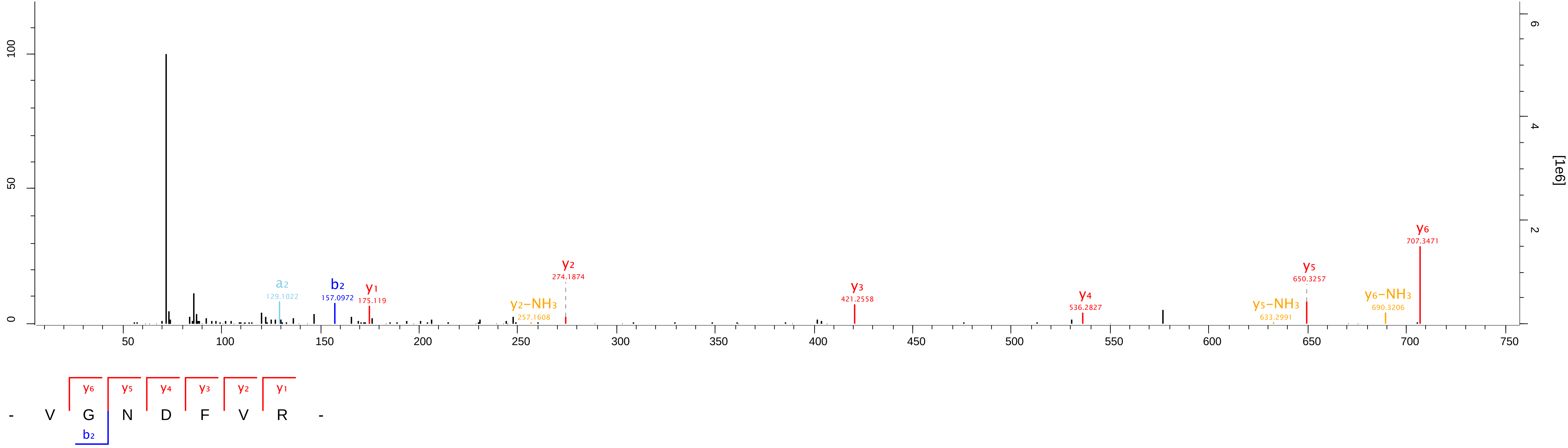

Raw file	Scan	Method	Score	m/z	Gene names
20140827_EXQ00_FaHo_SA_SPT7_02	6517	FTMS; HCD	90.71	748.9	TPO4

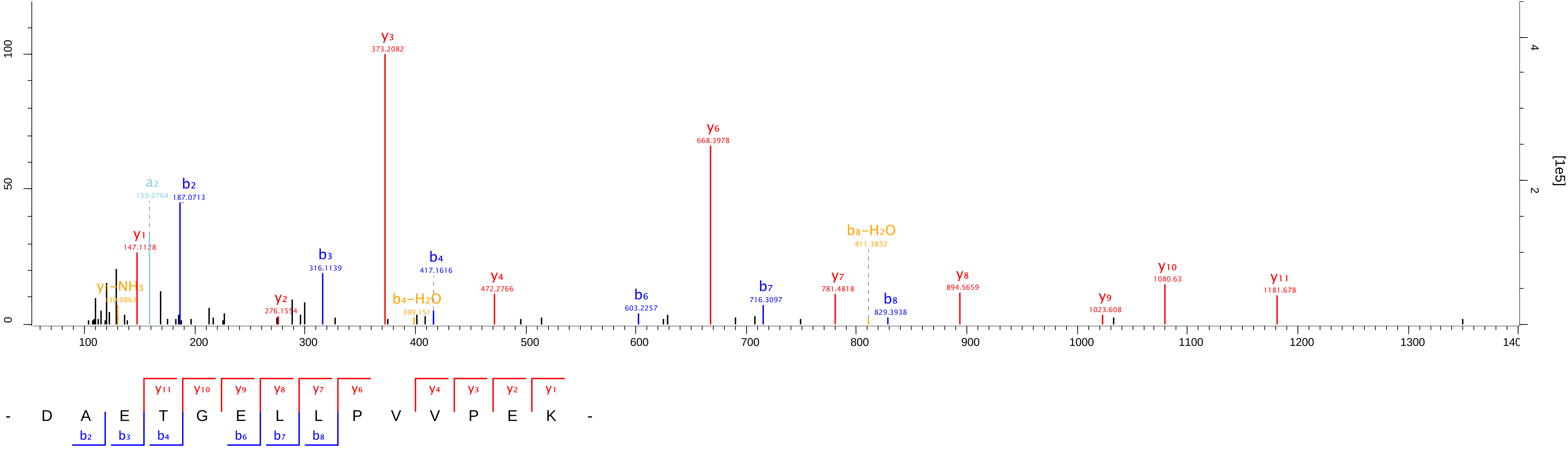

Raw file	Scan	Method	Score	m/z	Gene names
20140827_EXQ00_FaHo_SA_STH1_01	4465	FTMS; HCD	169.12	480.76	RPL9A;RPL9B

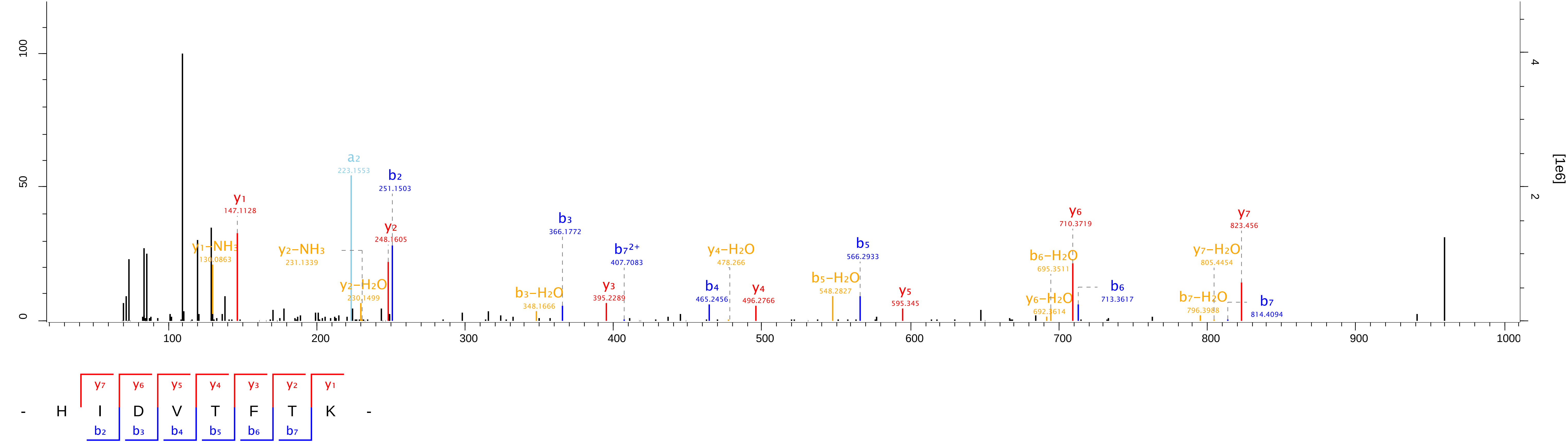

Raw file	Scan Method	Score	m/z	Gene names
20140827_EXQ00_FaHo_SA_S[illegible]	338[illegible] FTMS;	72.14	377.1	TY1B-LR3;TY1A-PL;TY1A-LR2;TY1A-ER1;TY1A-DR6;TY1B-OL;TY1B-LR4;TY1B-LR2;TY1B-PL;TY1B-ER1;TY1B-PR3;TY1A-PR1;TY1A-A;TY1A-DR4;TY1B-H;TY1B-GR2;TY1B-MR2;TY1B-ER2;TY1B-OR;TY1B-BR;TY1B-DR1;TY1B-NL2;TY1B-LR[illegible]

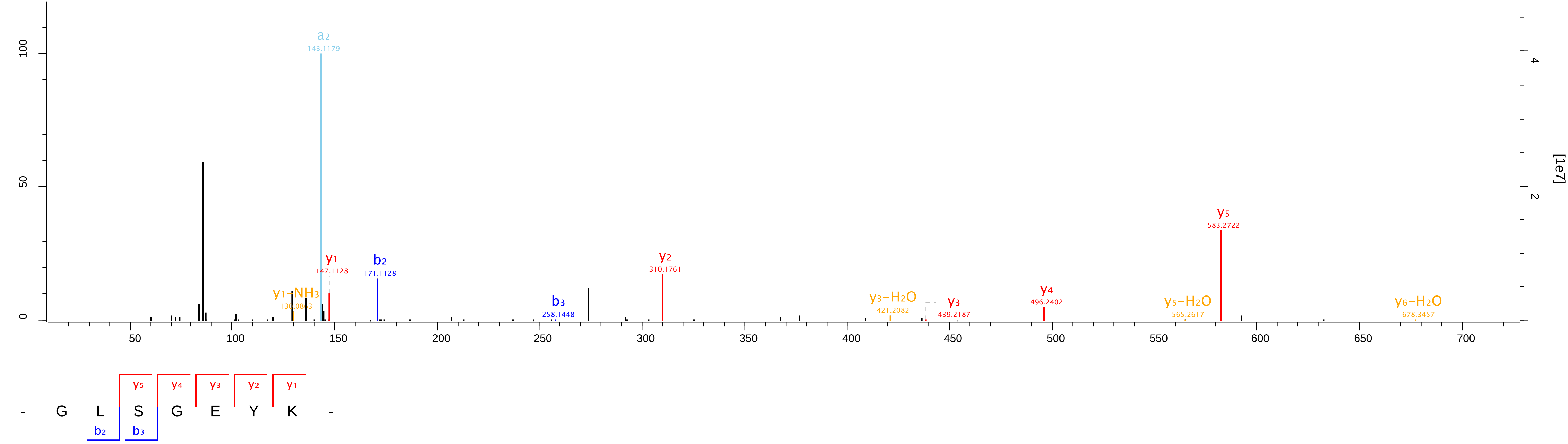

Raw file	Scan	Method	Score	m/z	Gene names
20140827_EXQ00_FaHo_SA_STH1_03	2099	FTMS; HCD	48.95	439.88	TY1A-PR1;TY1A-A;TY1A-DR4;TY1B-H;TY1B-GR2;TY1B-MR2;TY1B-ER2;TY1B-OR;TY1B-BR;TY1B-DR1;TY1B-NL2;TY1B-LR1

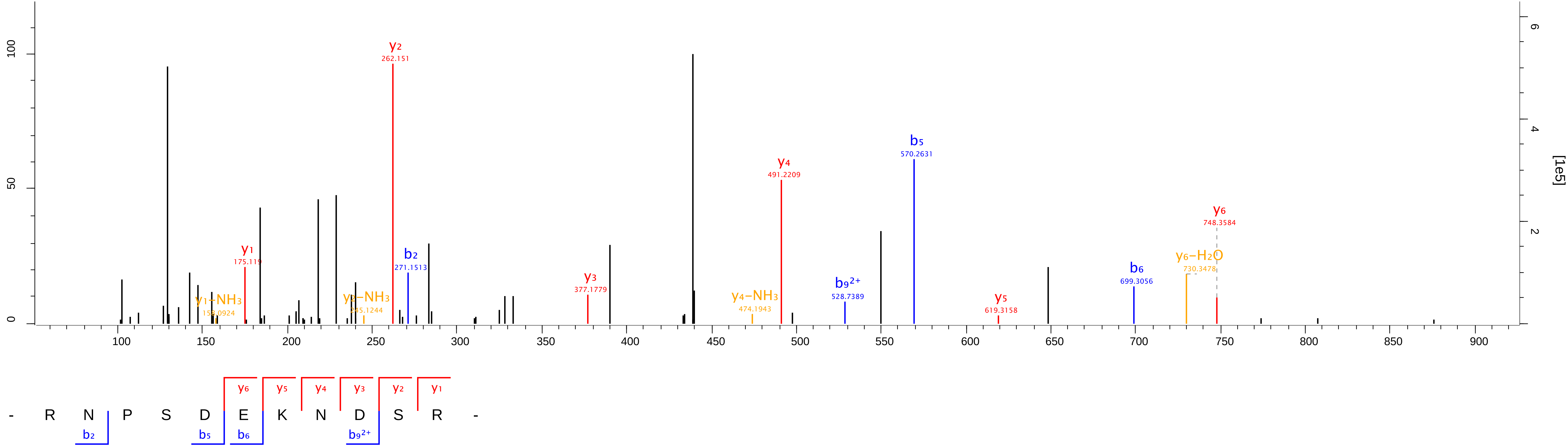

Raw file	Scan	Method	Score	m/z	Gene names
20140827_EXQ00_FaHo_SA_STH1_03	6449	FTMS; HCD	153.32	731.37	RPL17A;RPL17B

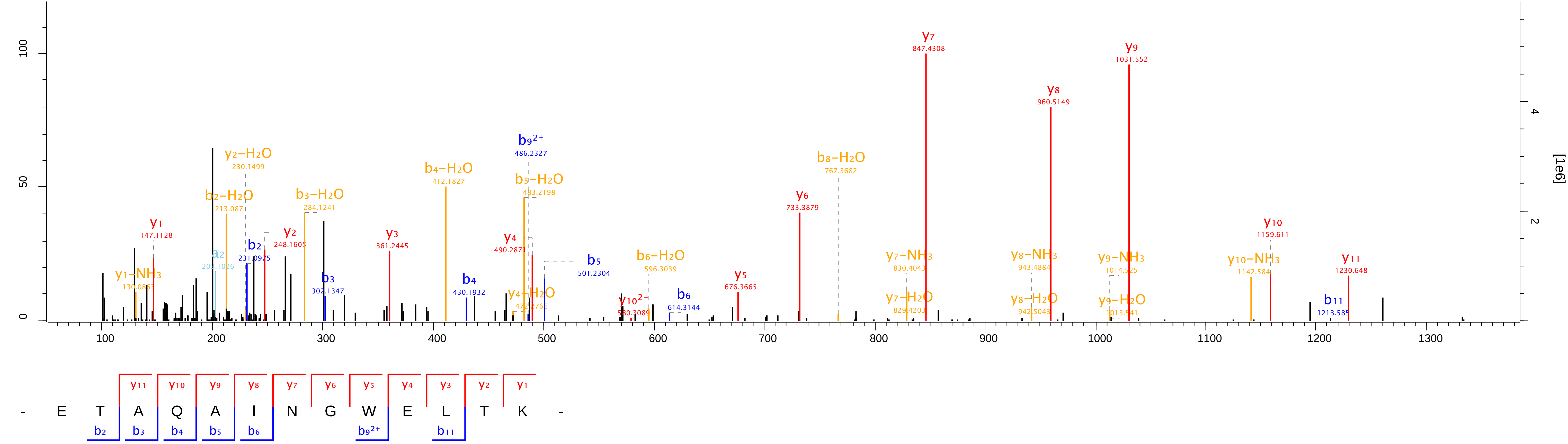

Raw file	Scan	Method	Score	m/z	Gene names
20140827_EXQ00_FaHo_SA_SWD3.	4322	FTMS;	319.74	820.4	TY1B-LR3;TY1A-PL;TY1A-LR2;TY1A-ER1;TY1A-DR6;TY1B-OL;TY1B-LR4;TY1B-LR2;TY1B-PL;TY1B-ER1;TY1B-PR3;TY1A-PR1;TY1A-A;TY1A-DR4;TY1B-H;TY1B-GR2;TY1B-MR2;TY1B-ER2;TY1B-OR;TY1B-BR;TY1B-DR1;TY1B-NL2;T

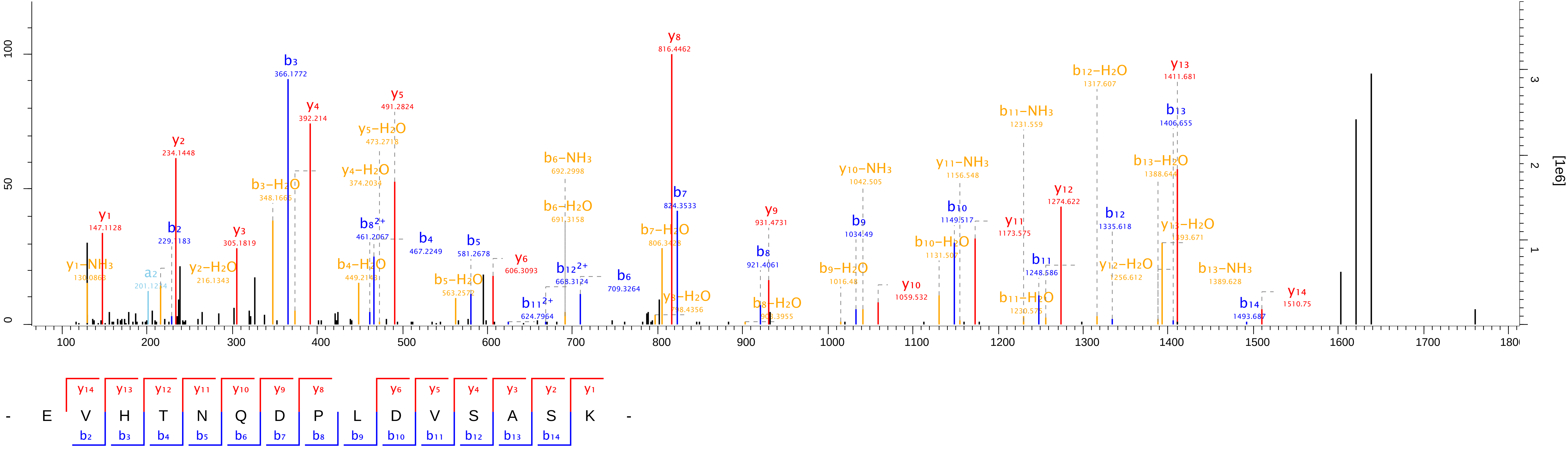

Raw file	Scan	Method	Score	m/z	Gene names
20140827_EXQ00_FaHo_SA_SWD3_02	4883	FTMS; HCD	204.7	867.4	TY1A-PL;TY1A-LR2;TY1A-ER1;TY1A-DR6;TY1B-LR2;TY1B-PL;TY1B-ER1;TY1A-PR1;TY1A-A;TY1A-DR4;TY1B-NL2;TY1B-NL1;TY1B-MR1;TY1A-DR2;TY1A-MR1

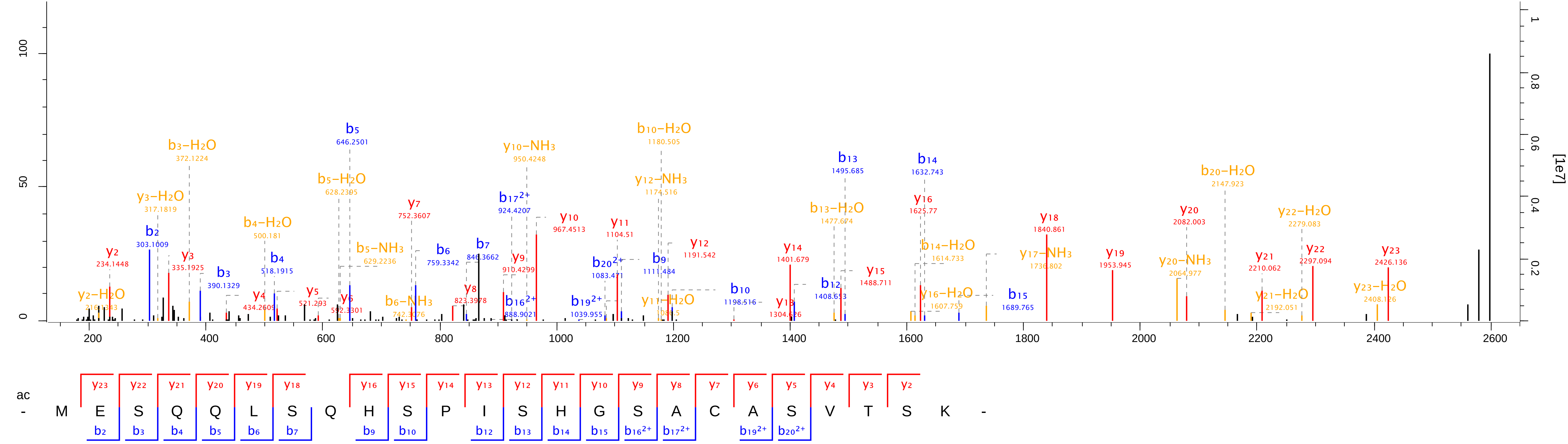

Raw file	Scan	Method	Score	m/z	Gene names
20140827_EXQ00_FaHo_SA_SWD3_03	3940	FTMS; HCD	141.65	670.87	RPS14B;RPS14A

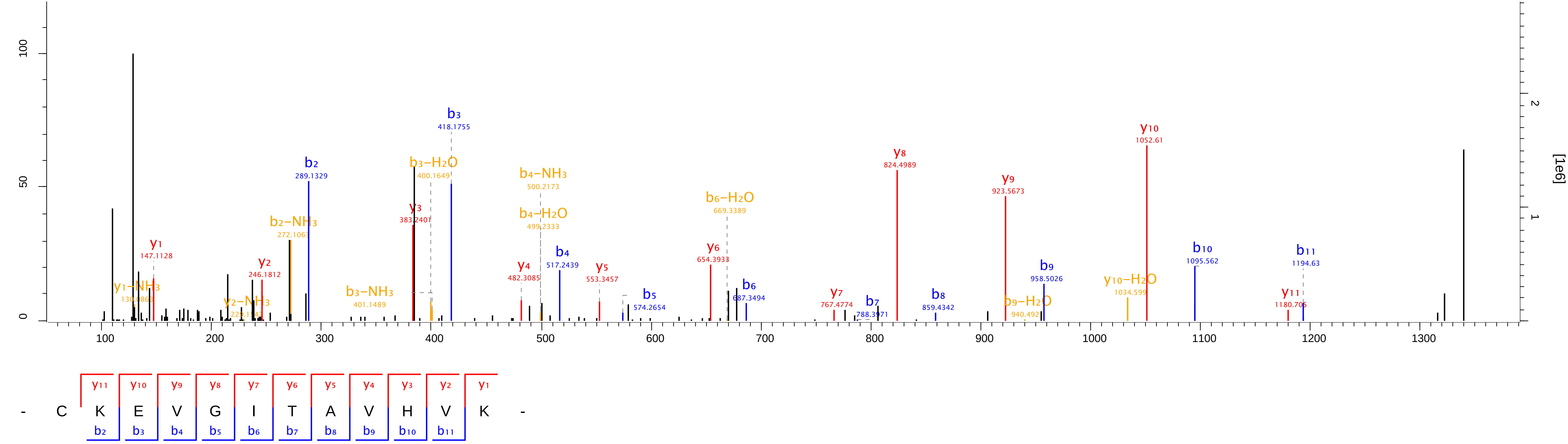

Raw file	Scan	Method	Score	m/z	Gene names
20140827_EXQ00_FaHo_SA_SWD3_03	6140	FTMS; HCD	125.74	589.81	RPL9A;RPL9B

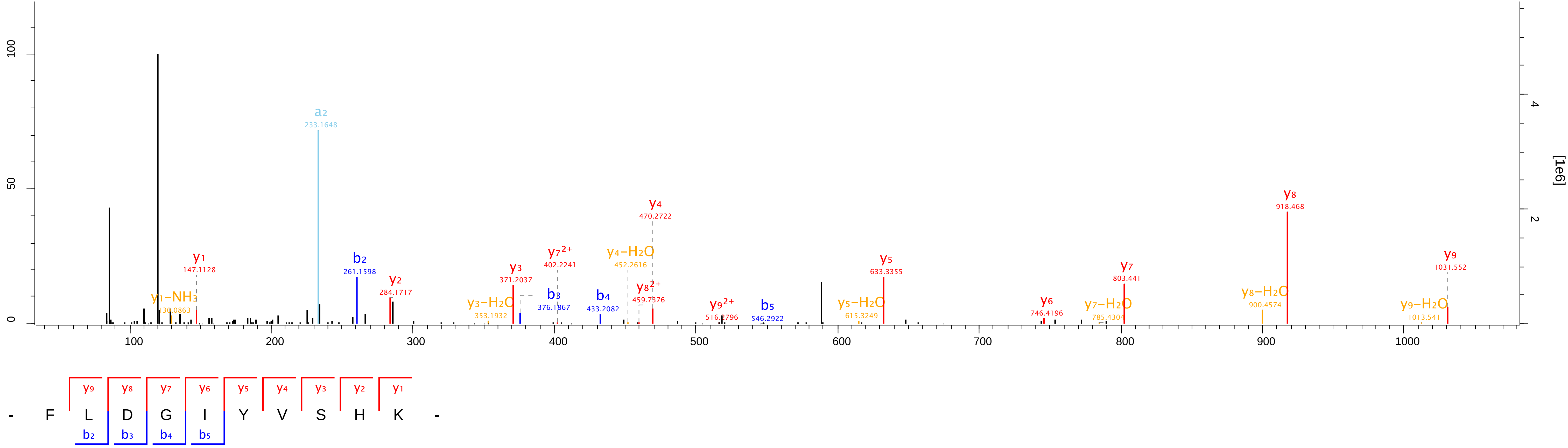

Raw file	Scan	Method	Score	m/z	Gene names
20140827_EXQ00_FaHo_SA_SWI3_01	3461	FTMS; HCD	98.23	448.23	RPL4B;RPL4A

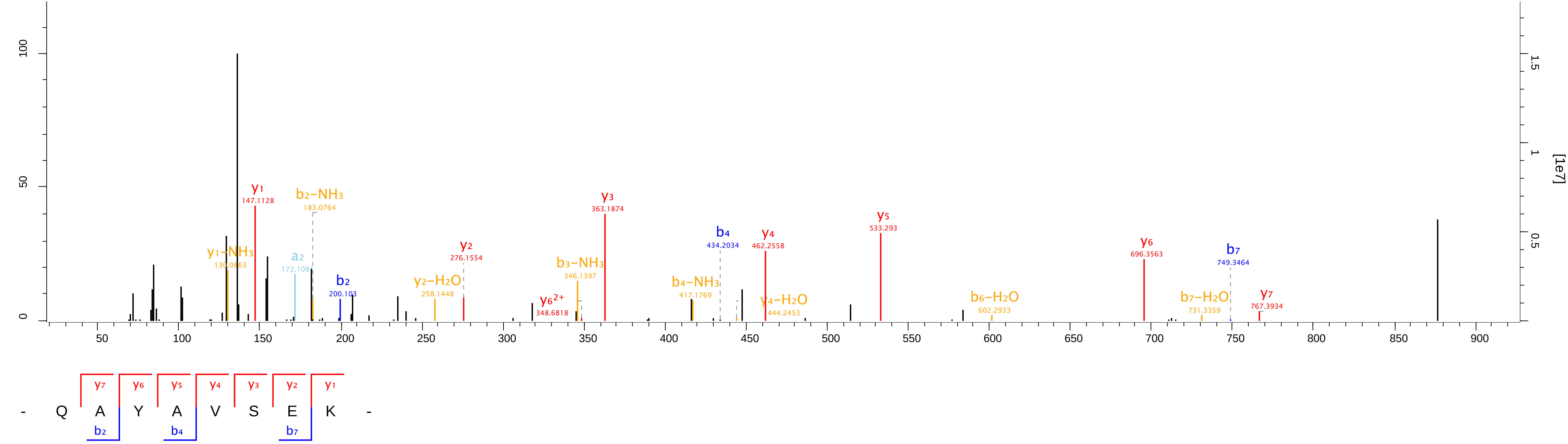

Raw file	Scan	Method	Score	m/z	Gene names
20140827_EXQ00_FaHo_SA_SWI3_01	3762	FTMS; HCD	77.28	411.23	RPL9A;RPL9B

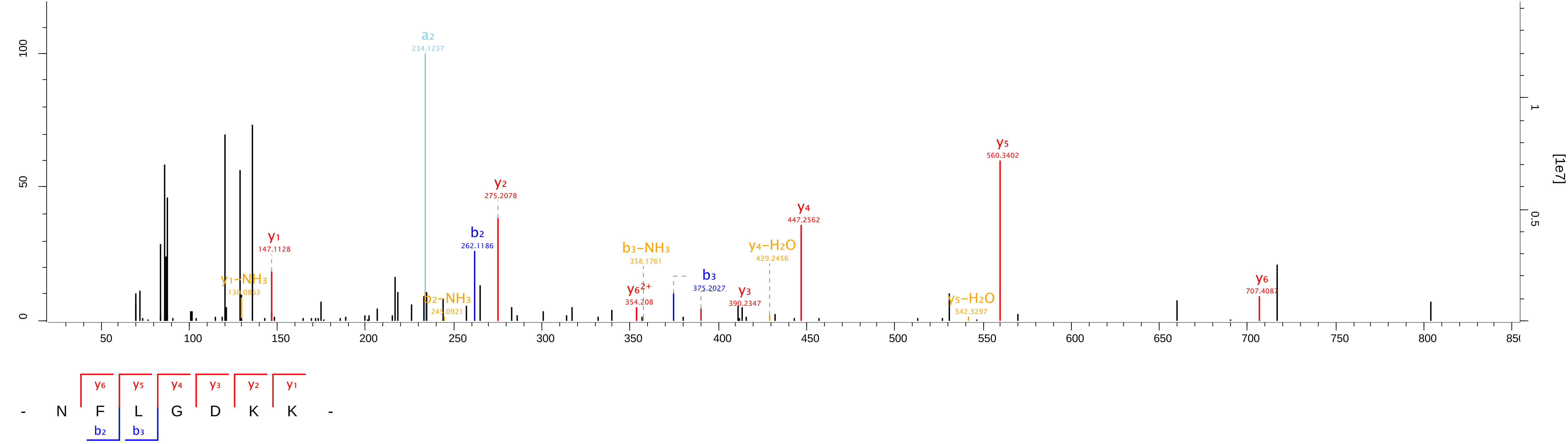

Raw file	Scan	Method	Score	m/z	Gene names
20140827_EXQ00_FaHo_SA_SWI3_01	4734	FTMS; HCD	67.23	672.84	ATP3

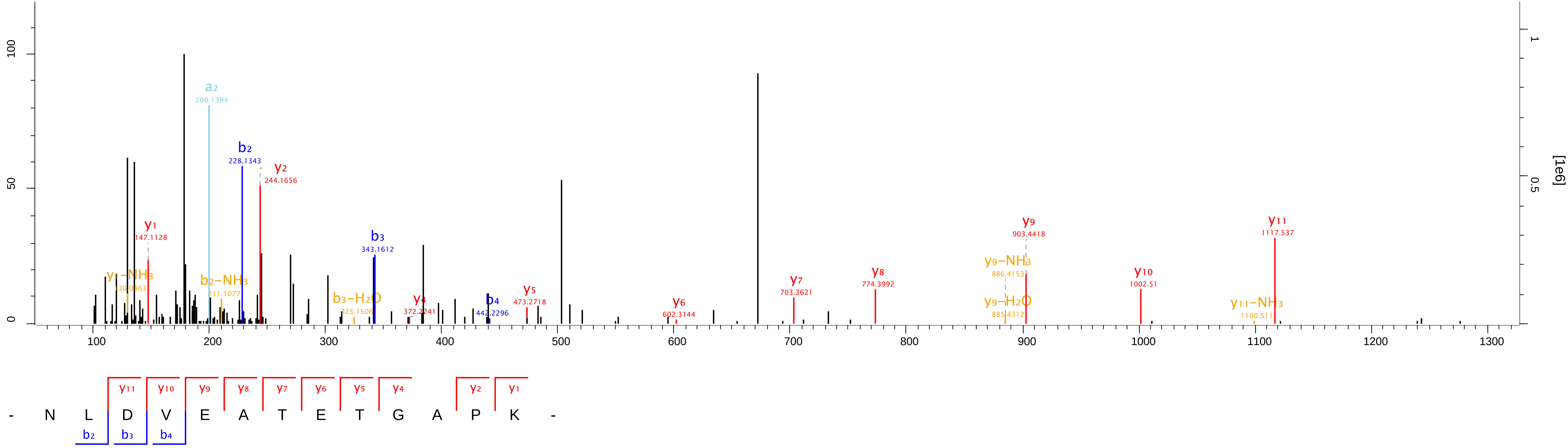

Raw file	Scan	Method	Score	m/z	Gene names
20140827_EXQ00_FaHo_SA_SWI3_01	5969	FTMS; HCD	136.57	753.88	RPL4B;RPL4A

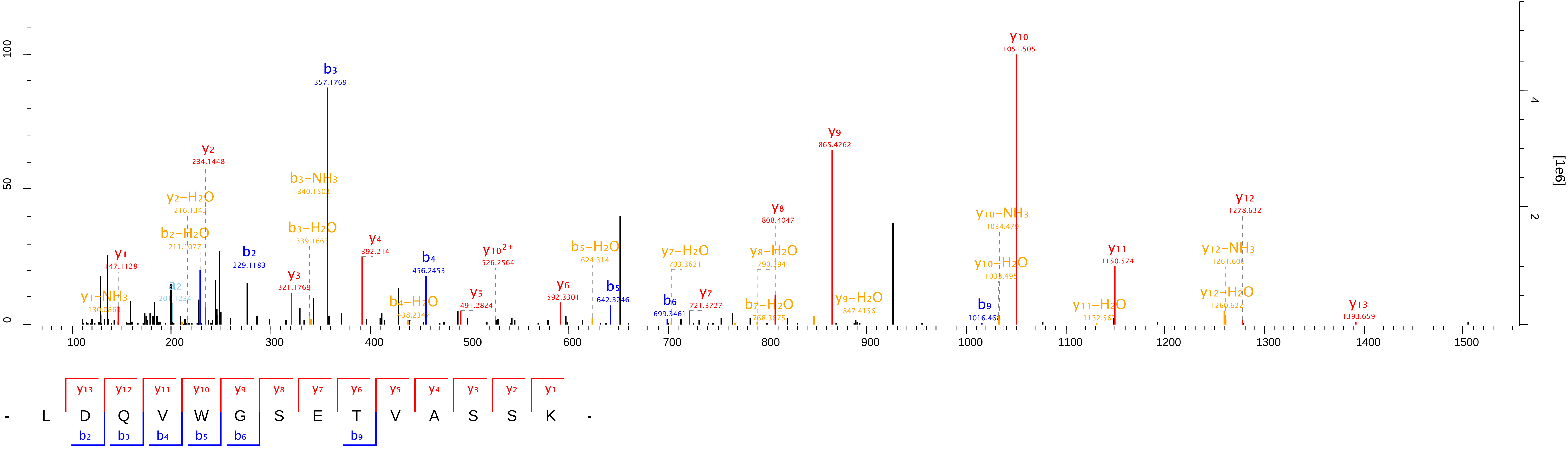

Raw file	Scan	Method	Score	m/z	Gene names
20140827_EXQ00_FaHo_SA_SWI3_02	3187	FTMS; HCD	85.55	676.31	RPB8

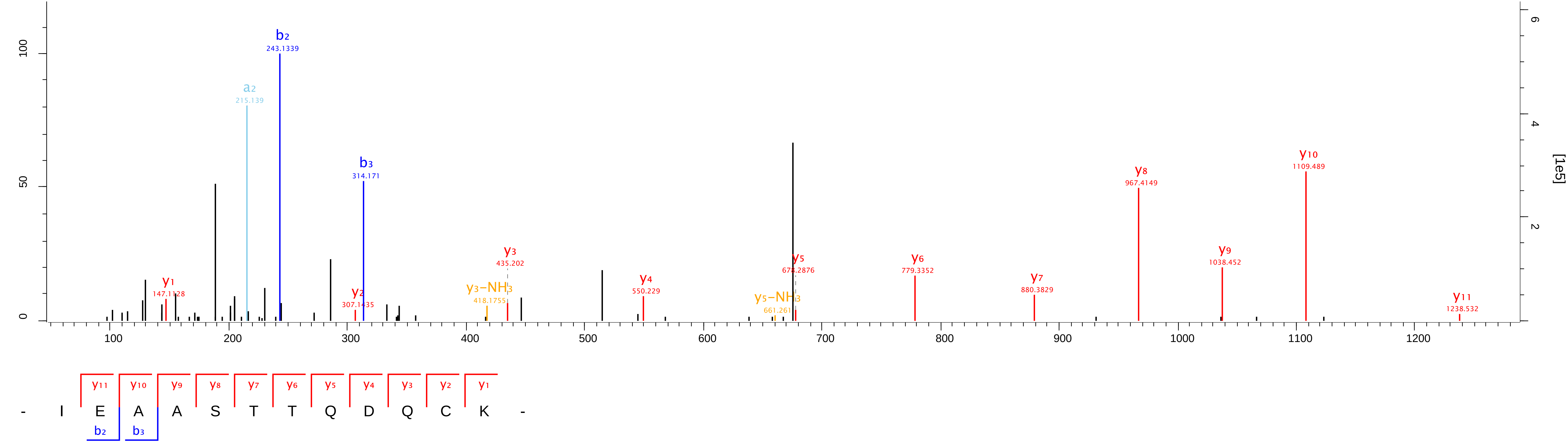

Raw file	Scan	Method	Score	m/z	Gene names
20140827_EXQ00_FaHo_SA_SWI3_02	4798	FTMS; HCD	81.78	440.24	PDR5;PDR15

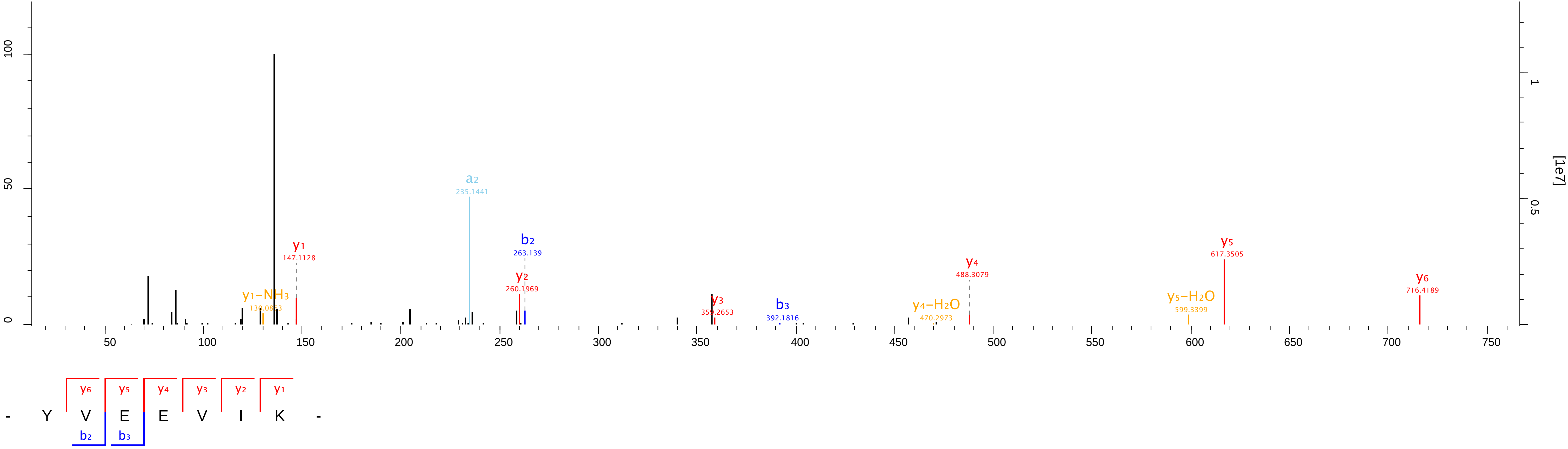

Raw file	Scan	Method	Score	m/z	Gene names
20140827_EXQ00_FaHo_SA_SWI3_02	4888	FTMS; HCD	143.98	708.35	RPS14B;RPS14A

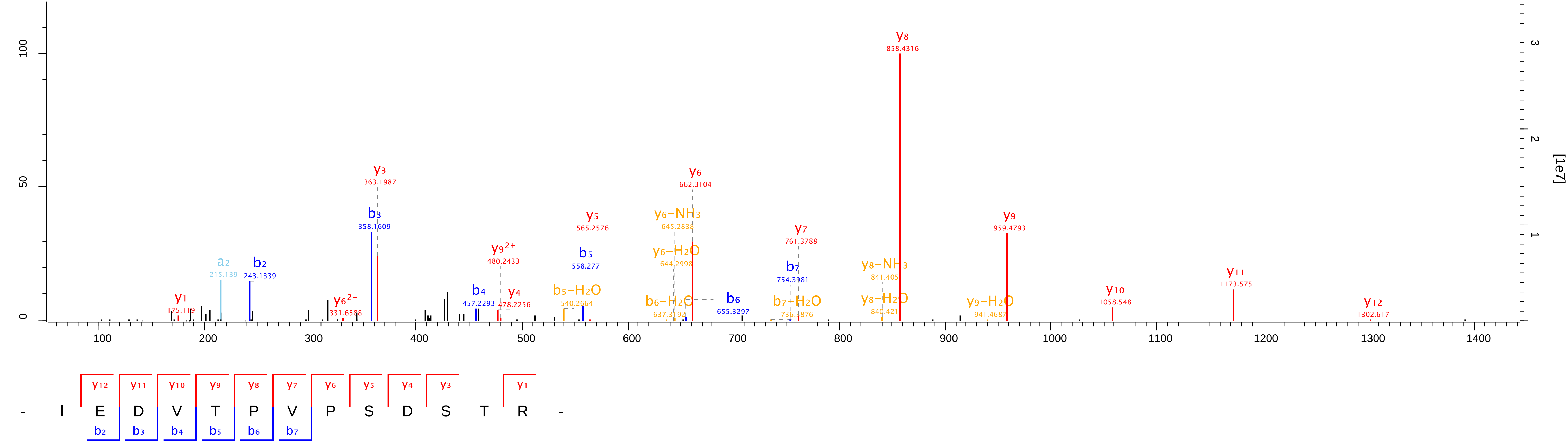

Raw file	Scan	Method	Score	m/z	Gene names
20140827_EXQ00_FaHo_SA_SWI3_02	6041	FTMS; HCD	214.16	901.14	RPL17A;RPL17B

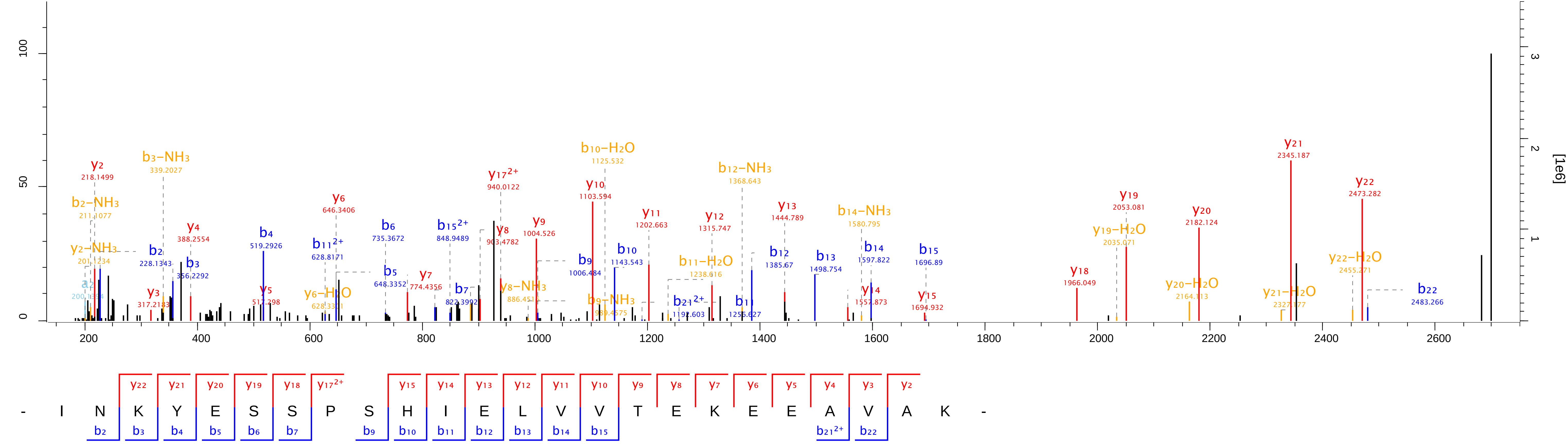

Raw file	Scan	Method	Score	m/z	Gene names
20140827_EXQ00_FaHo_SA_SWI3_02	8547	FTMS; HCD	64.82	576.3	APT1

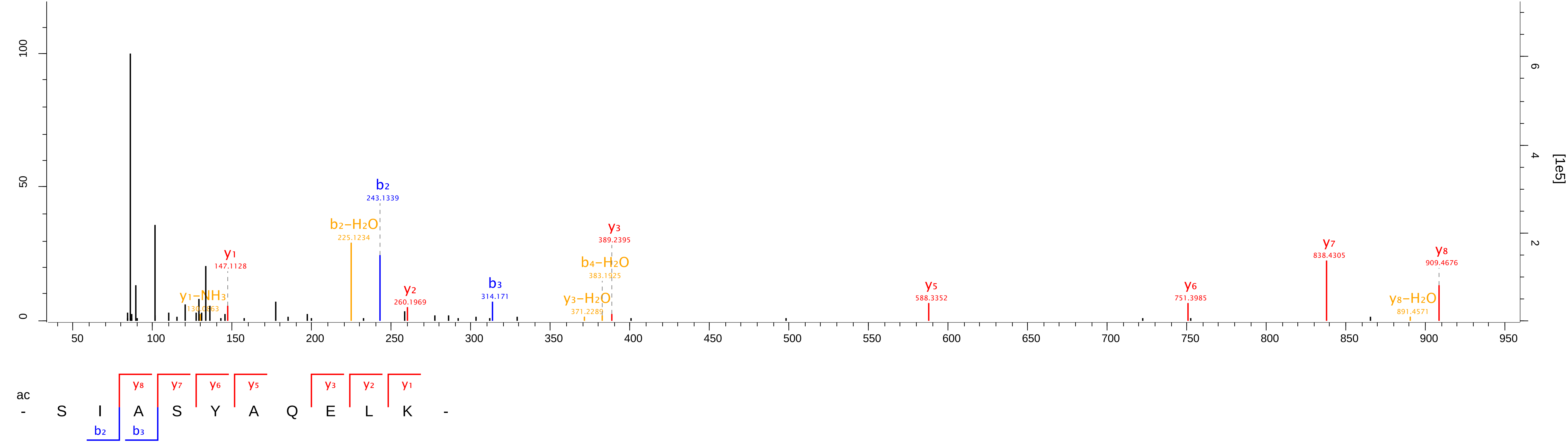

Raw file	Scan	Method	Score	m/z	Gene names
20140827_EXQ00_FaHo_SA_SWI3_03	8116	FTMS; HCD	73.88	702.35	RPS0A;RPS0B

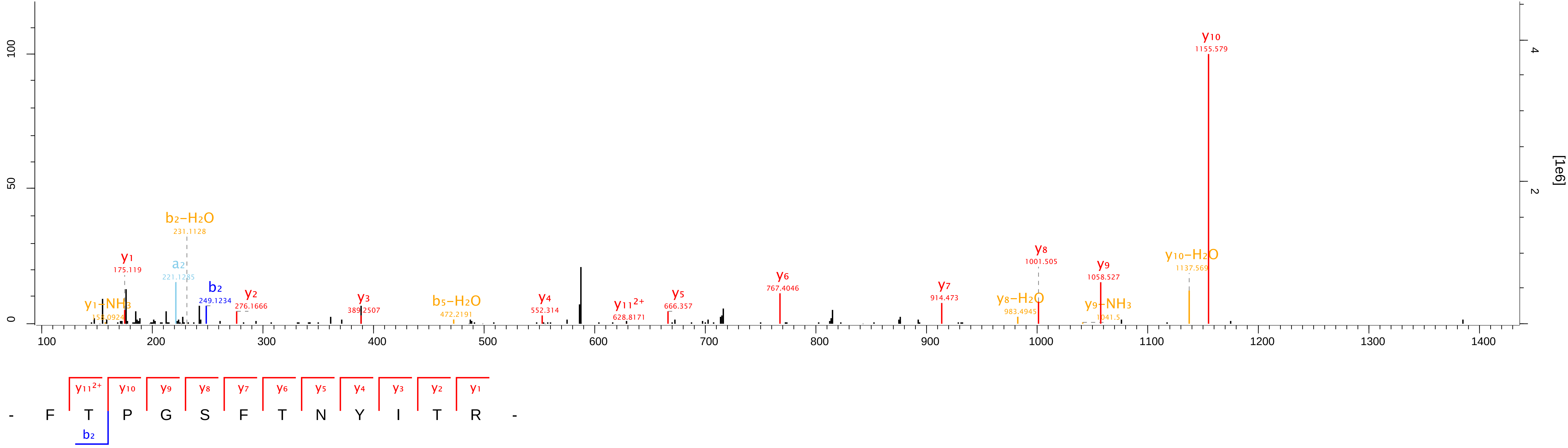

Raw file	Scan	Method	Score	m/z
20140827_EXQ00_FaHo_SA_YNG2_01	4028	FTMS; HCD	35.15	380.17

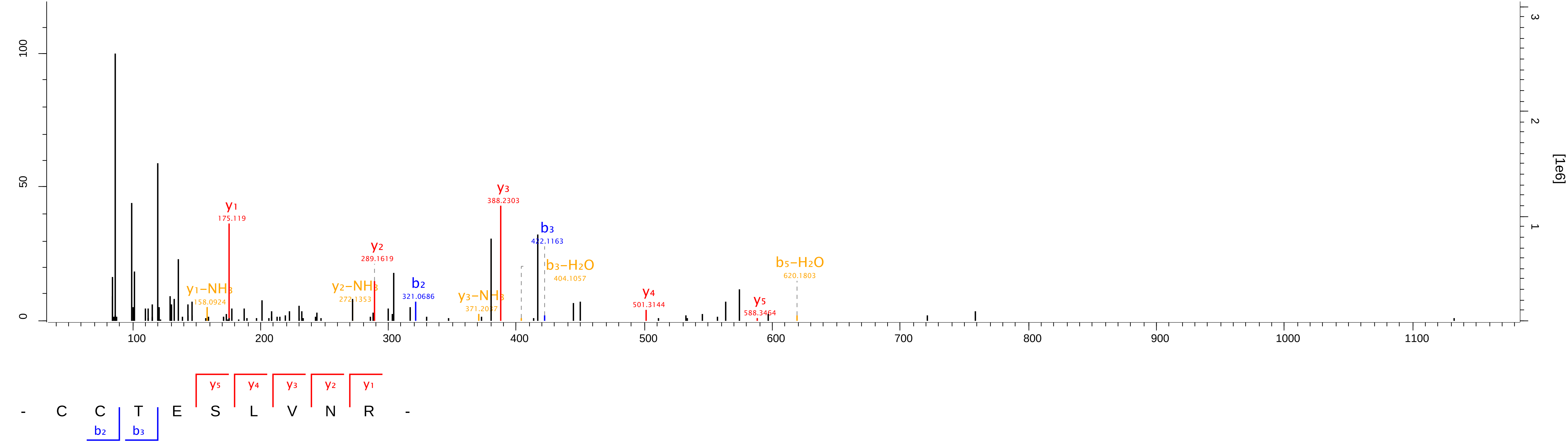

Raw file	Scan	Method	Score	m/z	Gene names
20140827_EXQ00_FaHo_SA_YNG2_01	5103	FTMS; HCD	147.2	599.76	PDR5;PDR15;PDR10

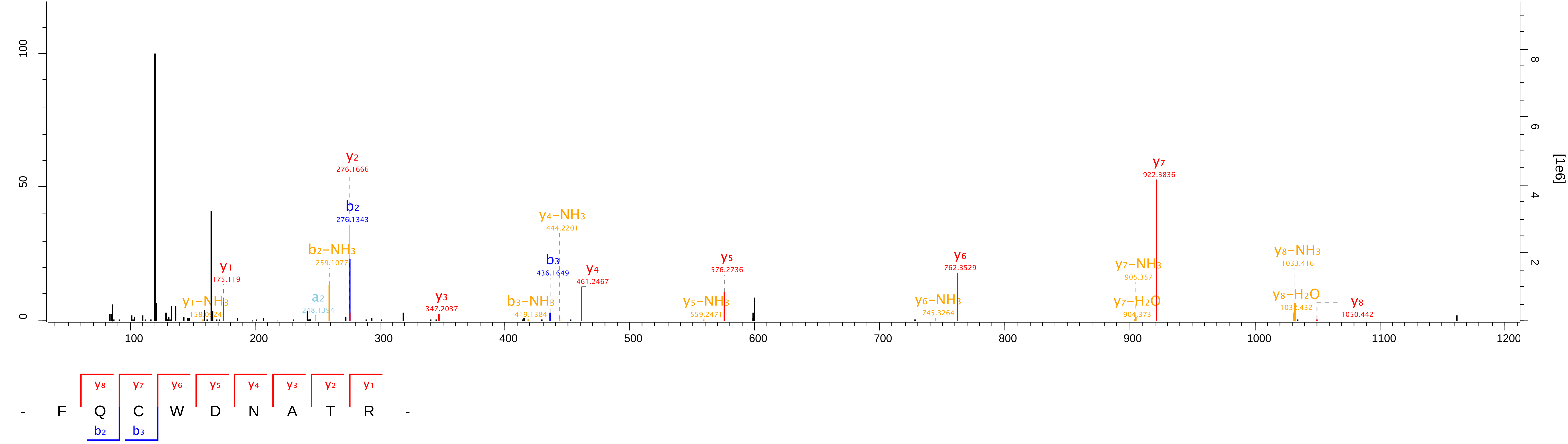

Raw file	Scan	Method	Score	m/z	Gene names
20140827_EXQ00_FaHo_SA_YNG2_01	5622	FTMS; HCD	80.74	562.97	RPL4B

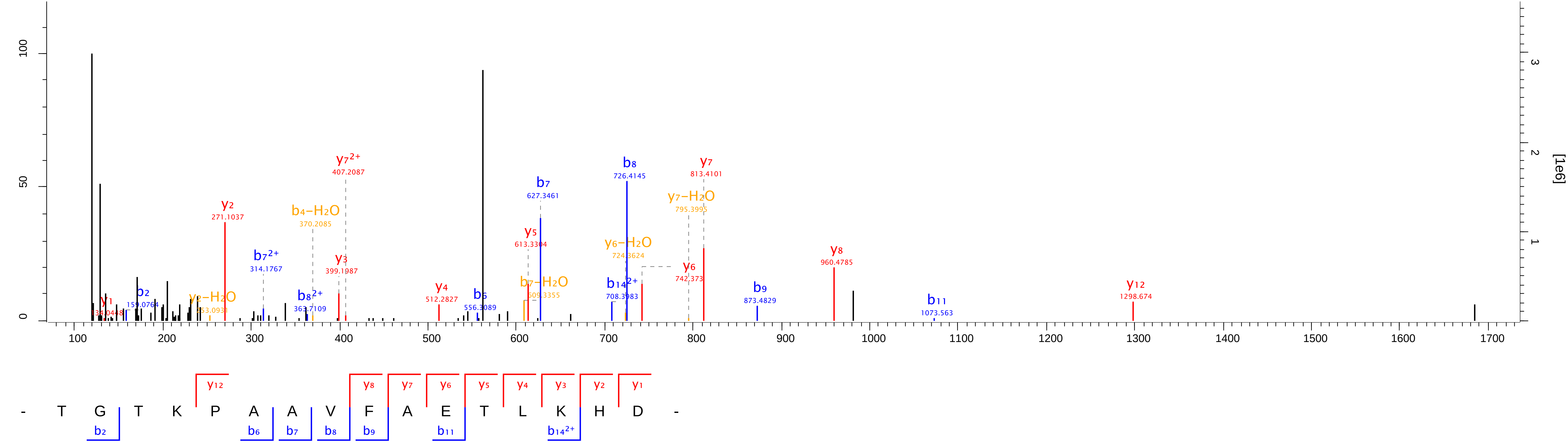

Raw file	Scan	Method	Score	m/z	Gene names
20140827_EXQ00_FaHo_SA_YNG2_01	6447	FTMS; HCD	195.03	705.12	RPS0A;RPS0B

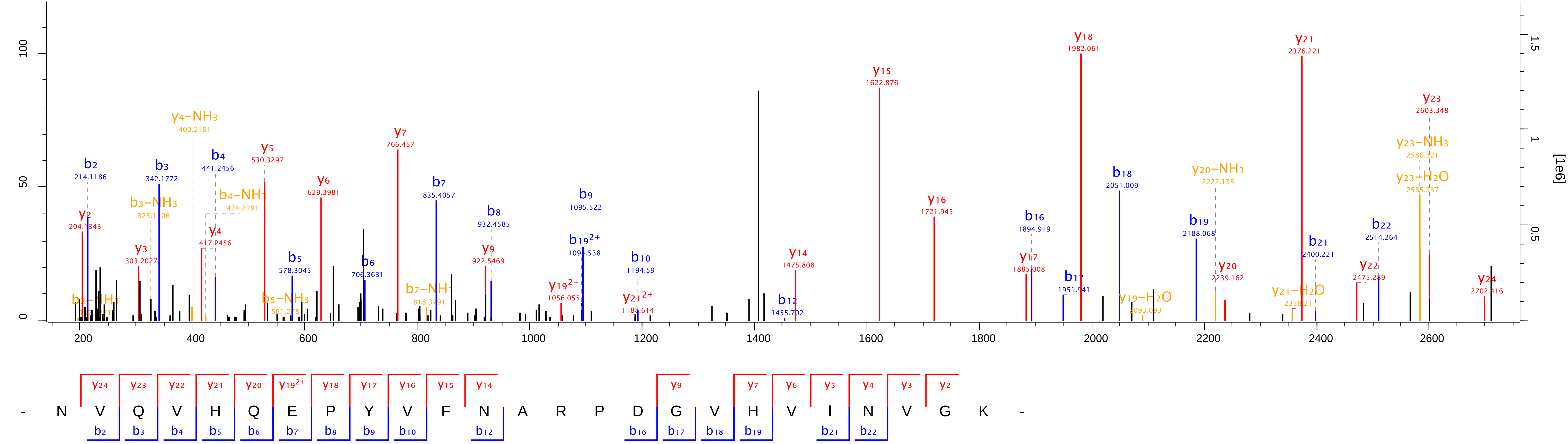

Raw file	Scan	Method	Score	m/z	Gene names
20140827_EXQ00_FaHo_SA_YN	7934	FTMS;	116.2	714.8	TY1B-LR3;TY1A-PL;TY1A-LR2;TY1A-ER1;TY1A-DR6;TY1B-OL;TY1B-LR4;TY1B-LR2;TY1B-PL;TY1B-ER1;TY1B-PR3;TY1A-PR1;TY1A-A;TY1A-DR4;TY1B-H;TY1B-GR2;TY1B-MR2;TY1B-ER2;TY1B-OR;TY1B-BR;TY1B-DR1;TY1B-NL2;TY1B-

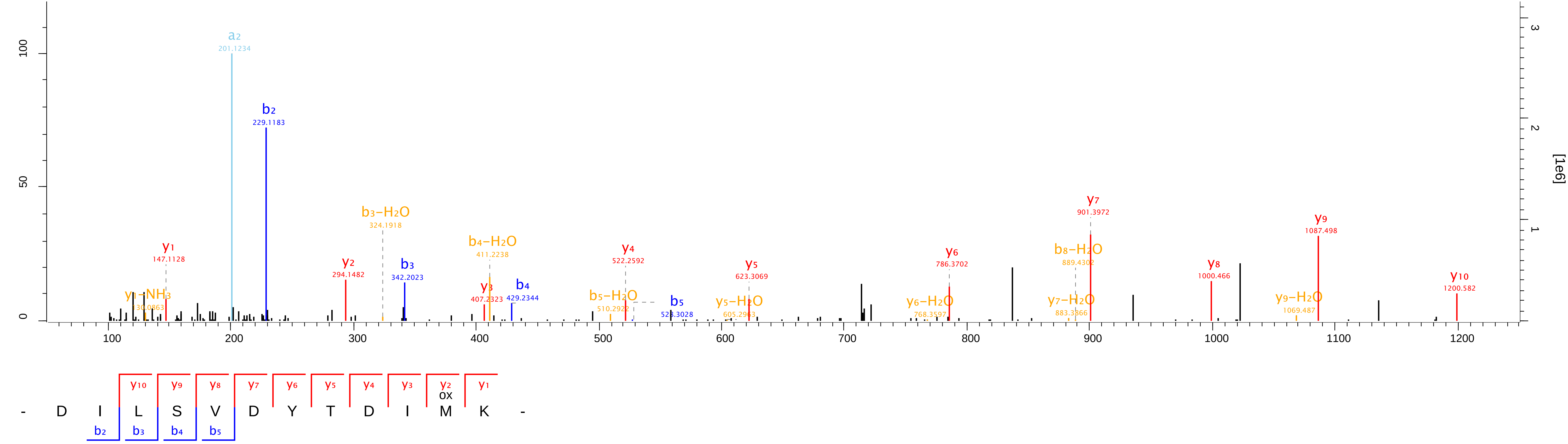

Raw file	Scan	Method	Score	m/z	Gene names
20140827_EXQ00_FaHo_SA_YNG2_02	3421	FTMS; HCD	124.46	784.38	SUR4

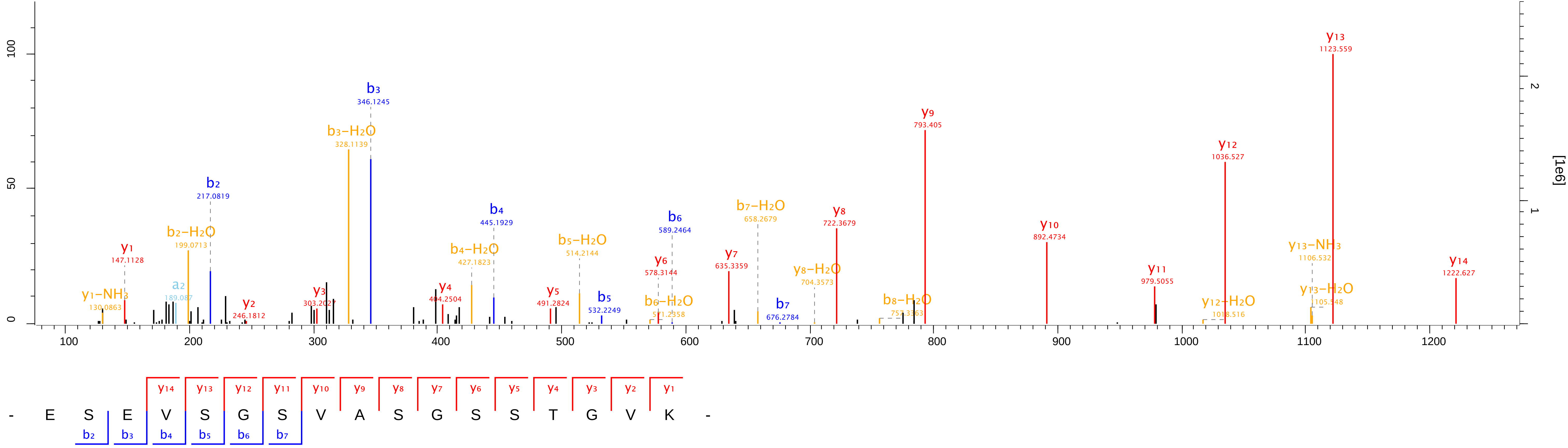

Raw file	Scan Method	Score	m/z	Gene names
20140827_EXQ00_FaHo_SA_YI612(	FTMS;	70.94	550.3	TY1B-OL;TY1B-LR4;TY1B-LR2;TY1B-PL;TY1B-ER1;TY1B-H;TY1B-GR2;TY1B-MR2;TY1B-ER2;TY1B-OR;TY1B-BR;TY1B-DR1;TY1B-NL2;TY1B-LR1;TY1B-DR3;TY1B-NL1;TY1B-BL;TY1B-MR1;TY2B-B;TY2B-GR1;TY2B-OR2;TY2B-DR2;TY2B-GR

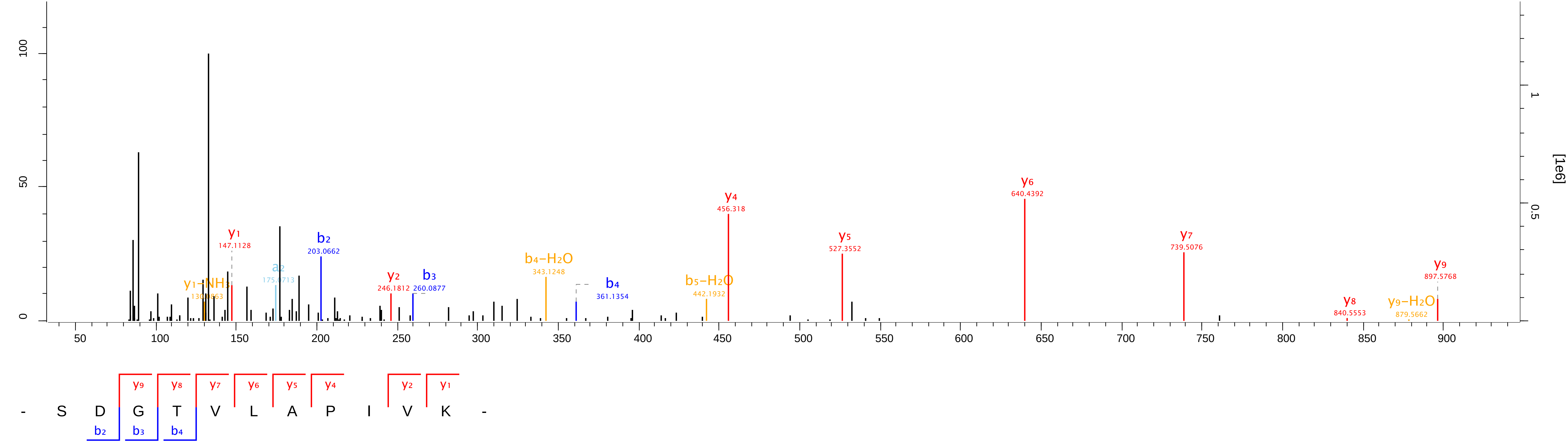

Raw file	Scan	Method	Score	m/z	Gene names
20140827_EXQ00_FaHo_SA_YNG2_03	2422	FTMS; HCD	49.65	413.69	TY1B-LR3;TY1B-OL;TY1B-LR4;TY1B-LR2;TY1B-PL;TY1B-ER1;TY1B-PR3;TY1B-H;TY1B-GR2;TY1B-MR2;TY1B-ER2;TY1B-OR;TY1B-BR;TY1B-DR1;TY1B-NL2;TY1B-LR1;TY1B-DR3;TY1B-NL1;TY1B-A;TY1B-BL;TY1B-MR1

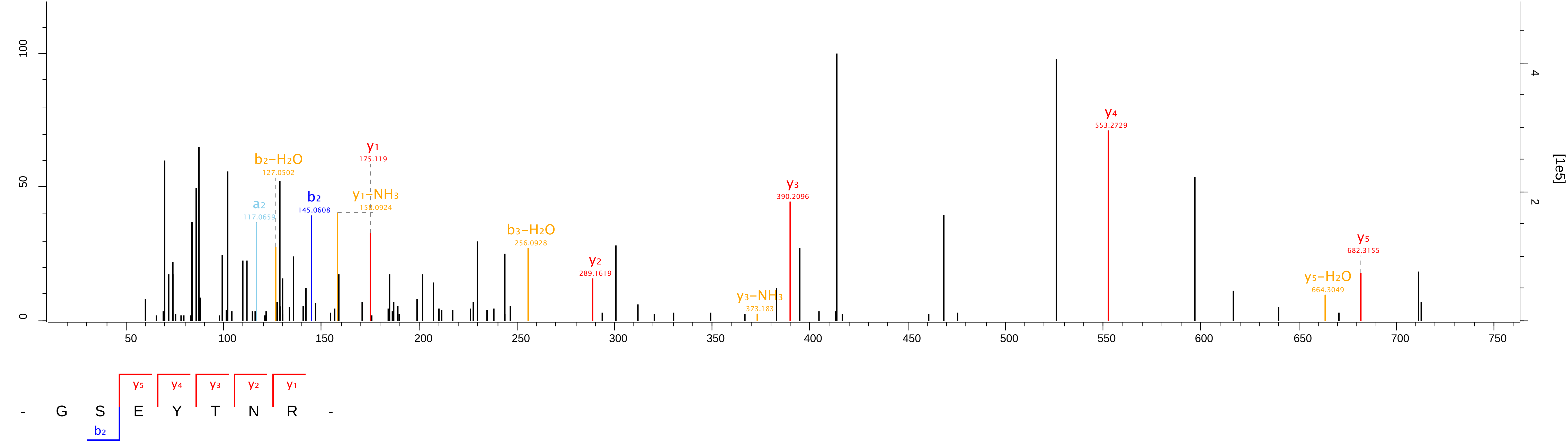

Raw file	Scan	Method	Score	m/z	Gene names
20140827_EXQ00_FaHo_SA_YNG2_03	3683	FTMS; HCD	157.3	660.8	TY1B-LR3;TY1B-GR2

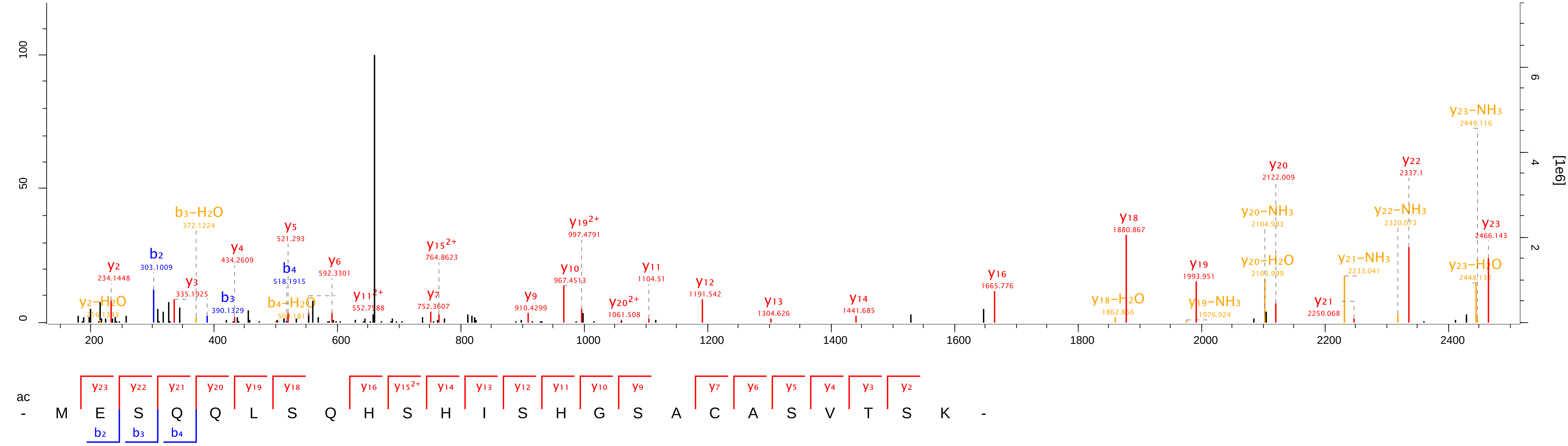

Raw file	Scan	Method	Score	m/z	Gene names
20140827_EXQ00_FaHo_SA_YNG2_03	4221	FTMS; HCD	85.81	526.81	RPS14B;RPS14A

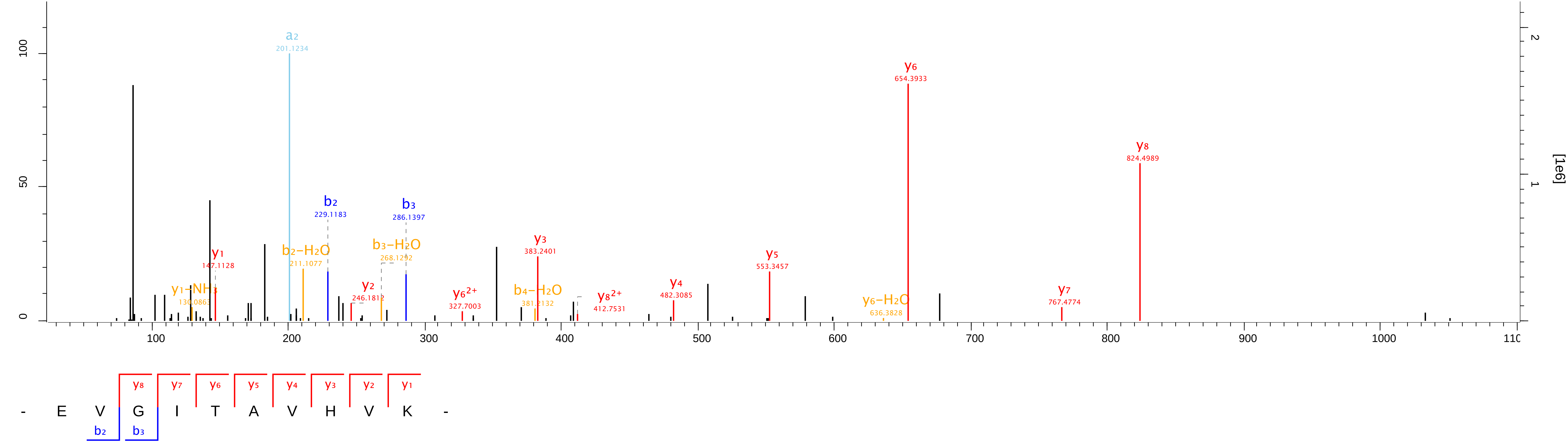

Raw file	Scan	Method	Score	m/z	Gene names
20140827_EXQ00_FaHo_SA_YNG2_03	5488	FTMS; HCD	99.69	508.29	YET1

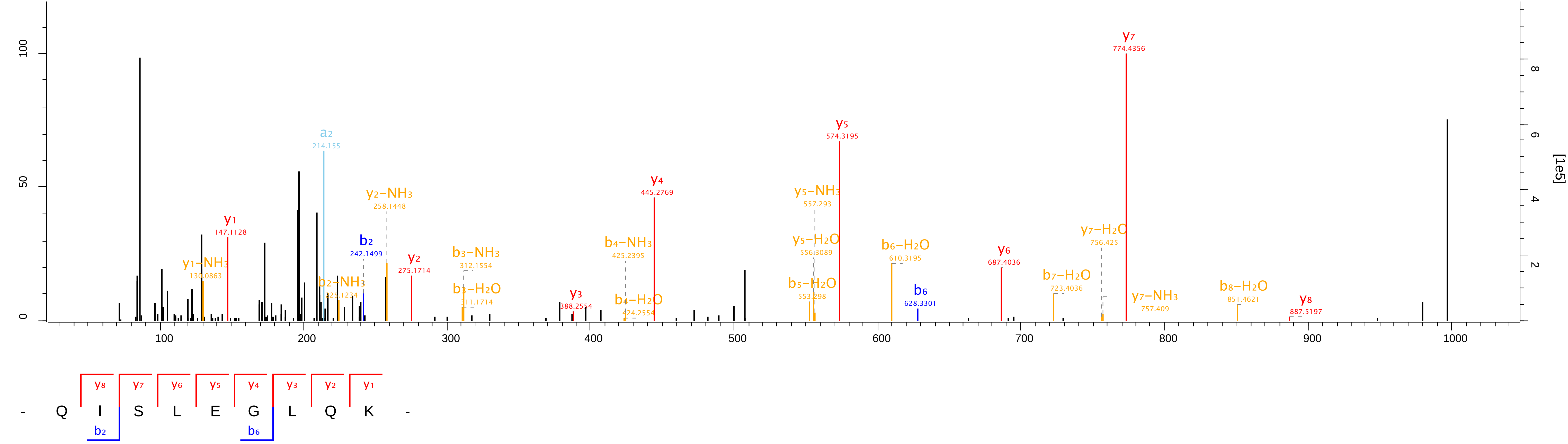

Supplement: Supplemental Data [file supp_O115.049460_mcp.O115.049460-5.pdf]
