# Supplementary material for: A Double-Barrel Liquid Chromatography-Tandem Mass Spectrometry (LC-MS/MS) System to Quantify 96 Interactomes per Day
Source: Mol Cell Proteomics. 2015 Apr 17;14(7):2030–41. doi: 10.1074/mcp.O115.049460 (PMC4587330; doi:10.1074/mcp.O115.049460)

Raw file	Scan	Method	Score	m/z	Gene names
20140602_QEp4_FaHo_SA_ADA2_02	1754	FTMS; HCD	126.28	448.25	RPL13B;RPL13A

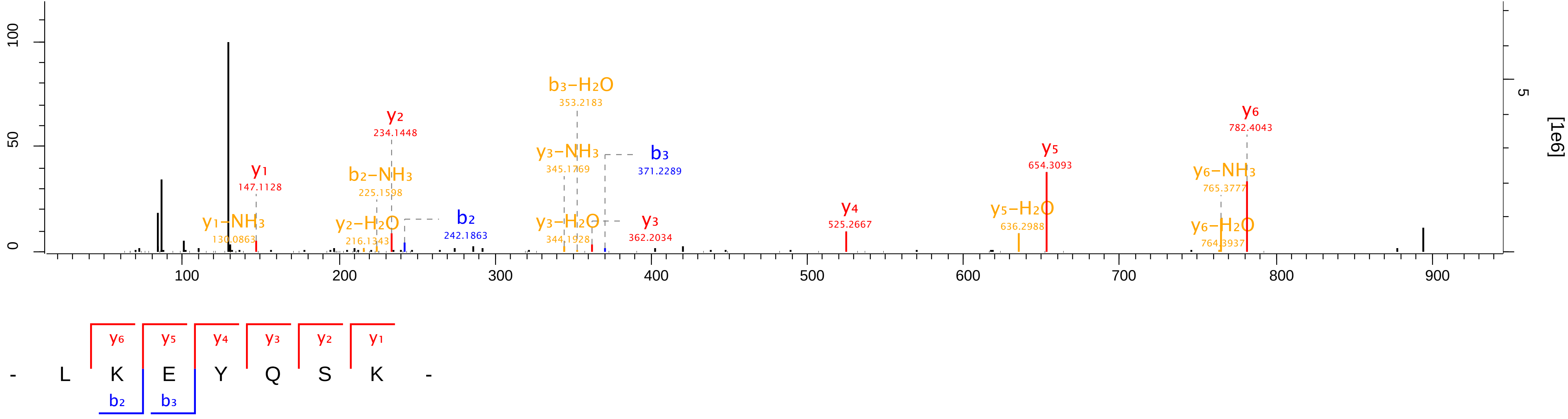

Raw file	Scan	Method	Score	m/z	Gene names
20140602_QEp4_FaHo_SA_ADA2_02	9841	FTMS; HCD	84.48	657.35	RPL4B;RPL4A

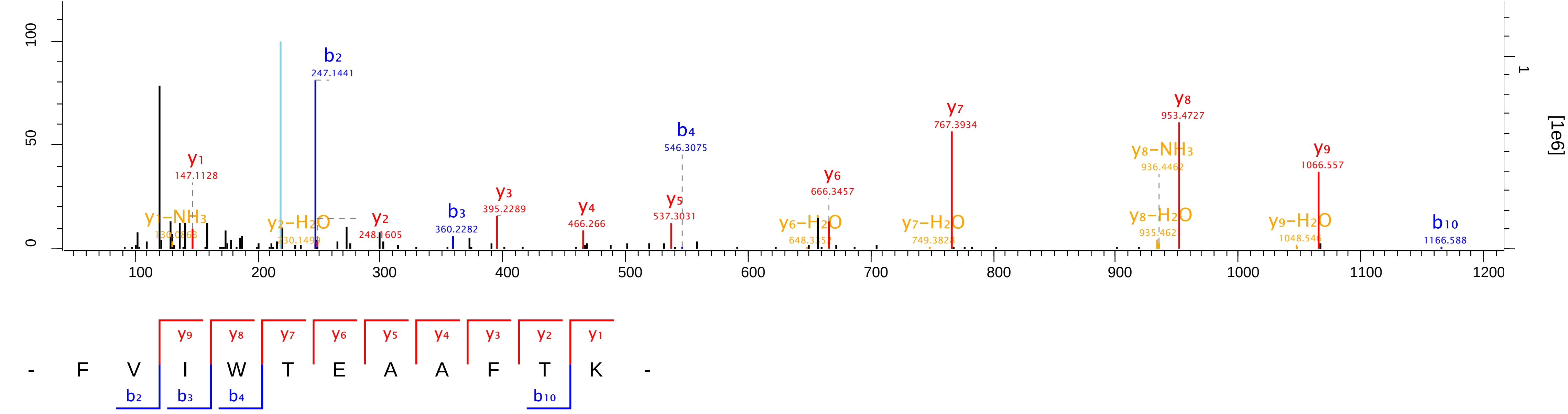

Raw file	Scan	Method	Score	m/z	Gene names
20140602_QEp4_FaHo_SA_BDF1_01	9862	FTMS; HCD	0.19	729.63	CSL4

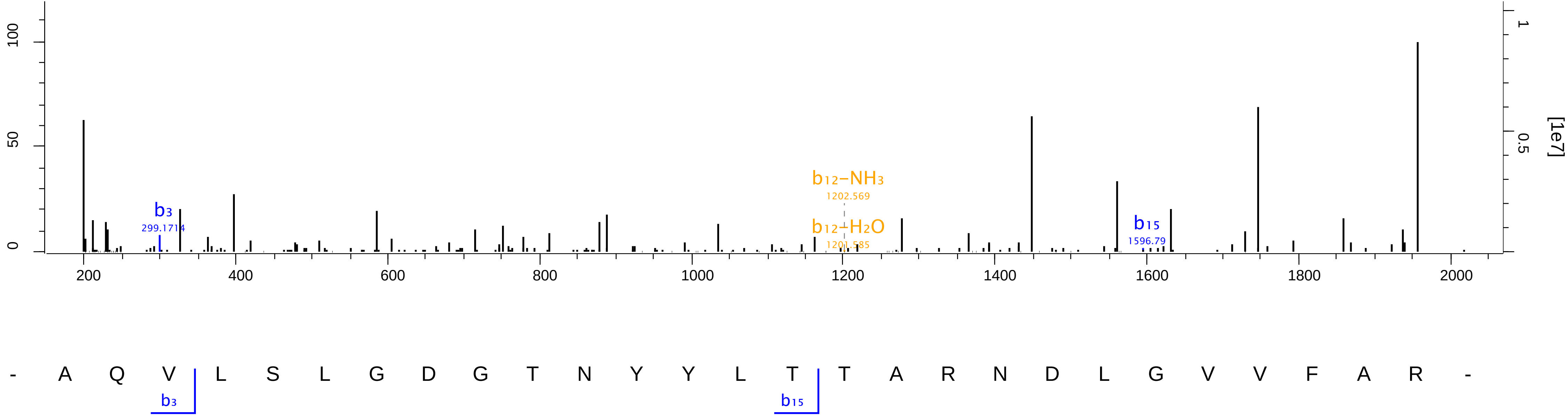

Raw file	Scan	Method	Score	m/z	Gene names
20140602_QEp4_FaHo_SA_BDF1_02	8357	FTMS; HCD	48.22	637.67	EIS1

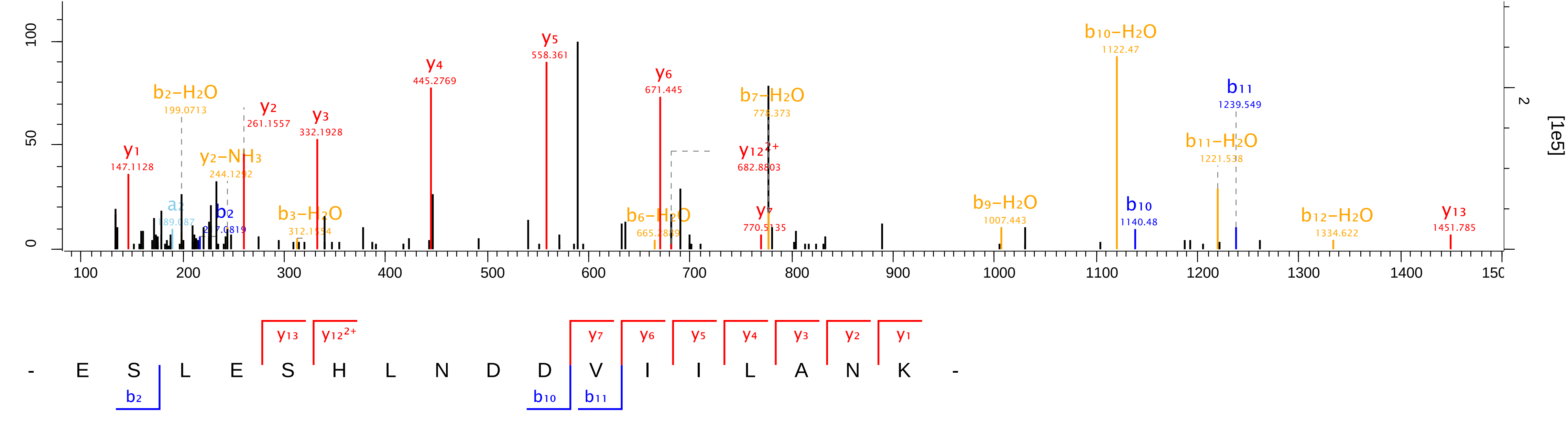

Raw file	Scar	Method	Score	m/z	Gene names
20140602_QEp4_FaHo_SA	992	F FTMS;	178	1045.·	TY1B-ML1;TY1B-BR;TY1B-H;TY1B-MR2;TY1B-OR;TY1B-DR1;TY1B-NL2;TY1B-PR2;TY1B-DR5;TY1B-PR1;TY1B-JR2;TY1B-NL1;TY1B-OL;TY1B-LR4;TY1B-ML2;TY1B-DR3;

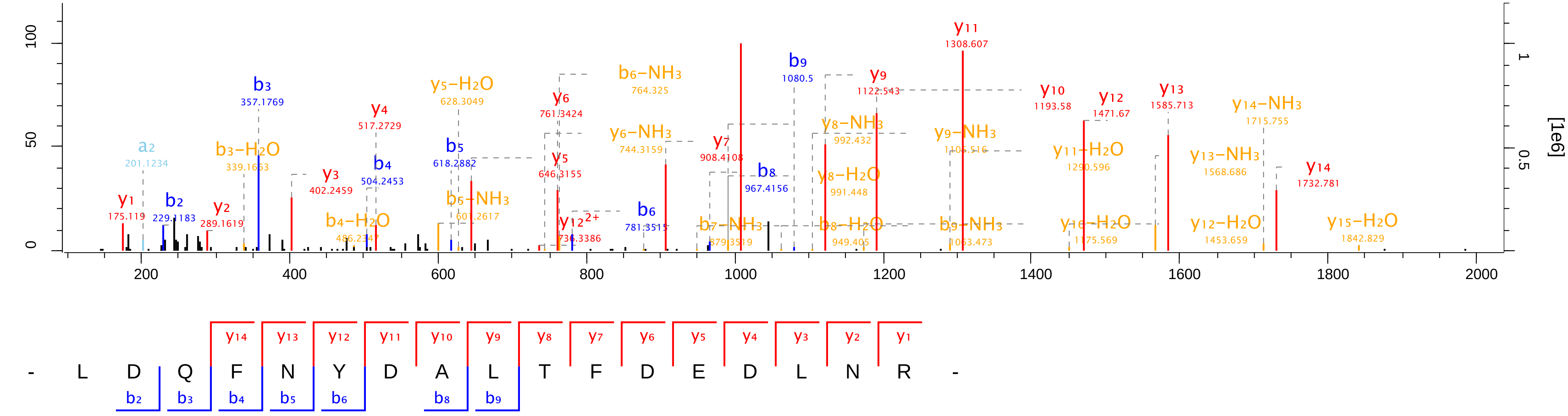

Raw file	Scan	Method	Score	m/z	Gene names
20140602_QEp4_FaHo_SA_BDF2_01	5546	FTMS; HCD	151.29	421.77	PRR1

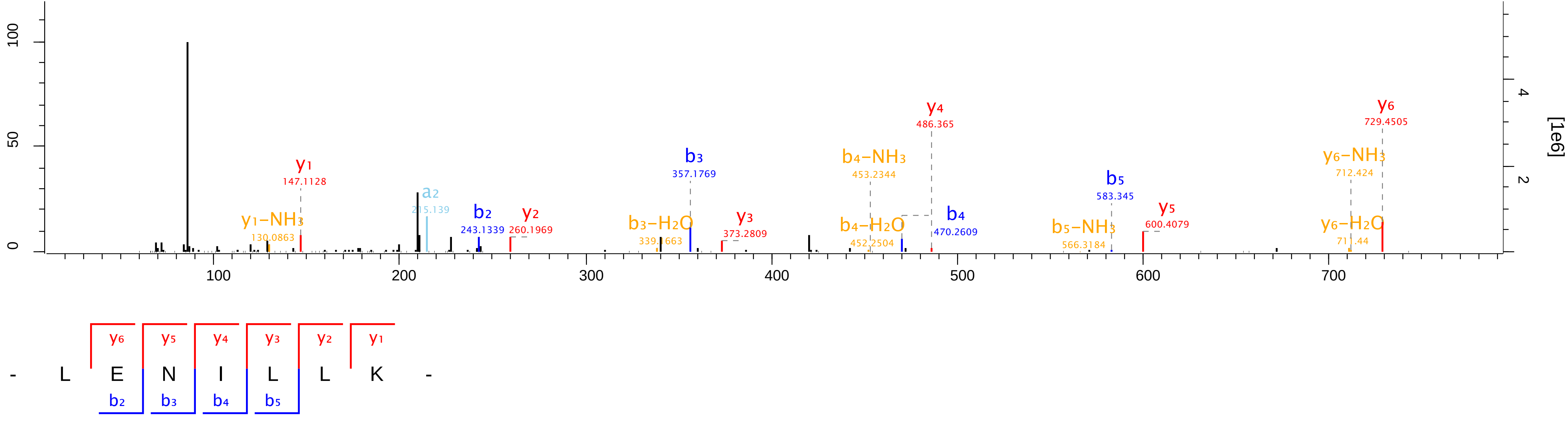

Raw file	Scan	Method	Score	m/z	Gene names
20140602_QEp4_FaHo_SA_BDF2_01	8265	FTMS; HCD	127.02	700.89	NOP12

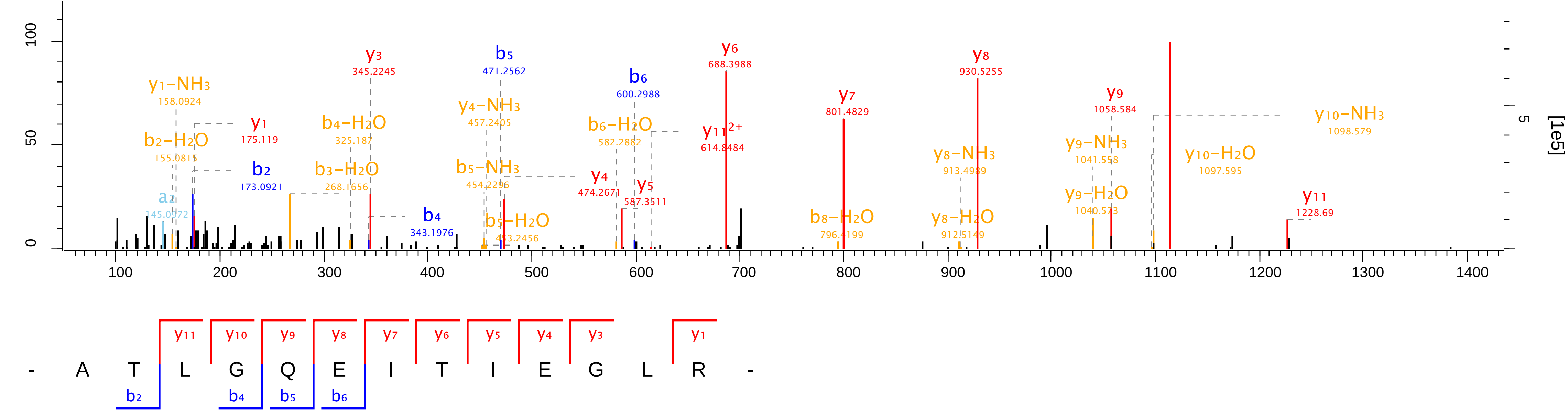

Raw file	Scan	Method	Score	m/z	Gene names
20140602_QEp4_FaHo_SA_BDF2_02	4095	FTMS; HCD	178.94	480.76	RPL9A;RPL9B

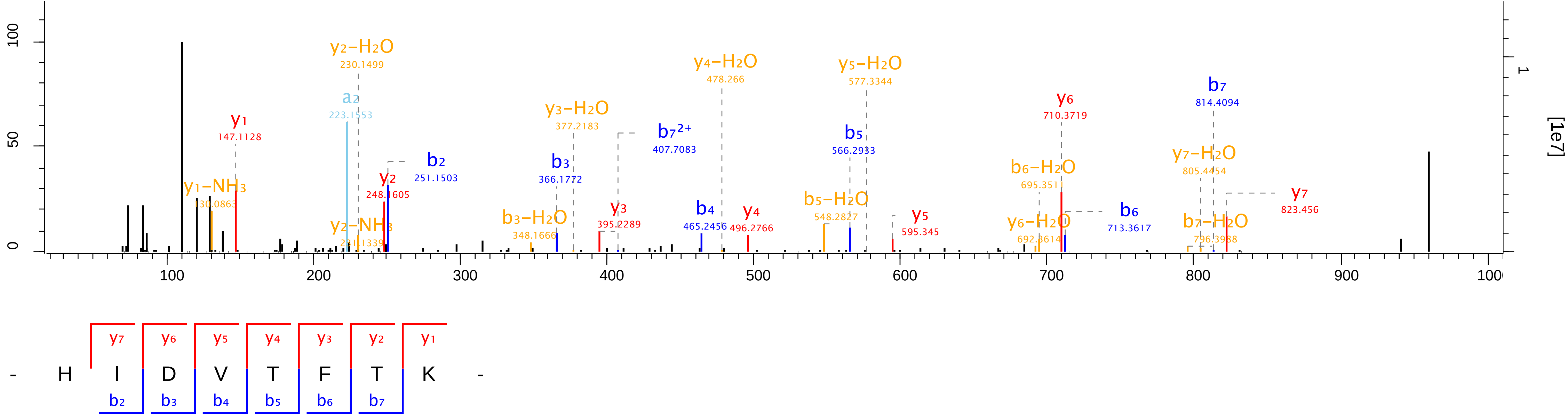

Raw file	Scan	Method	Score	m/z	Gene names
20140602_QEp4_FaHo_SA_BDF2_02	8104	FTMS; HCD	144.29	548.29	RPA135

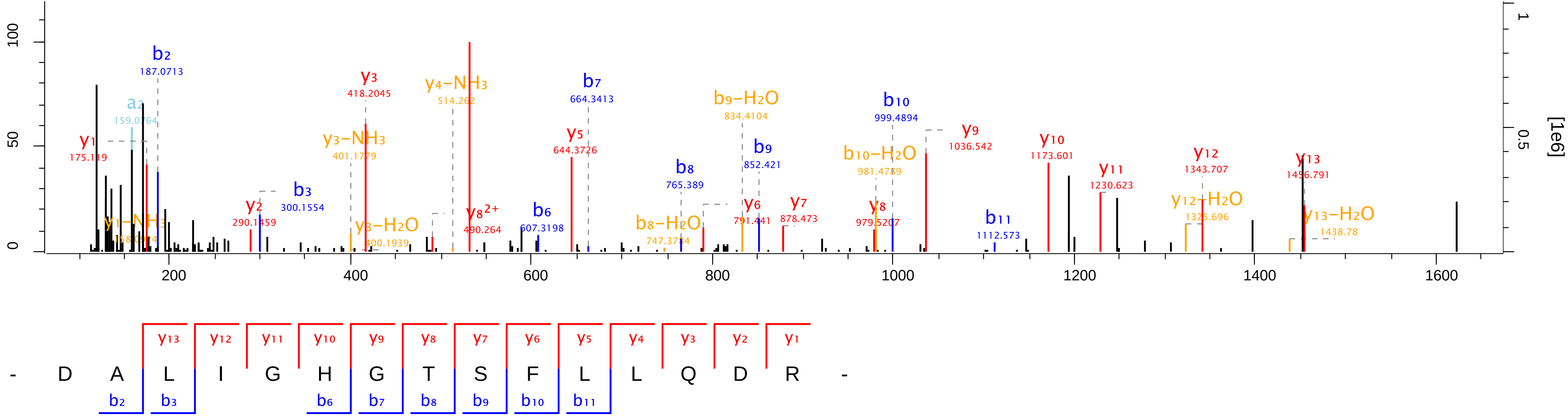

Raw file	Scan	Method	Score	m/z	Gene names
20140602_QEp4_FaHo_SA_BDF2_02	8499	FTMS; HCD	109.44	544.79	KTR3

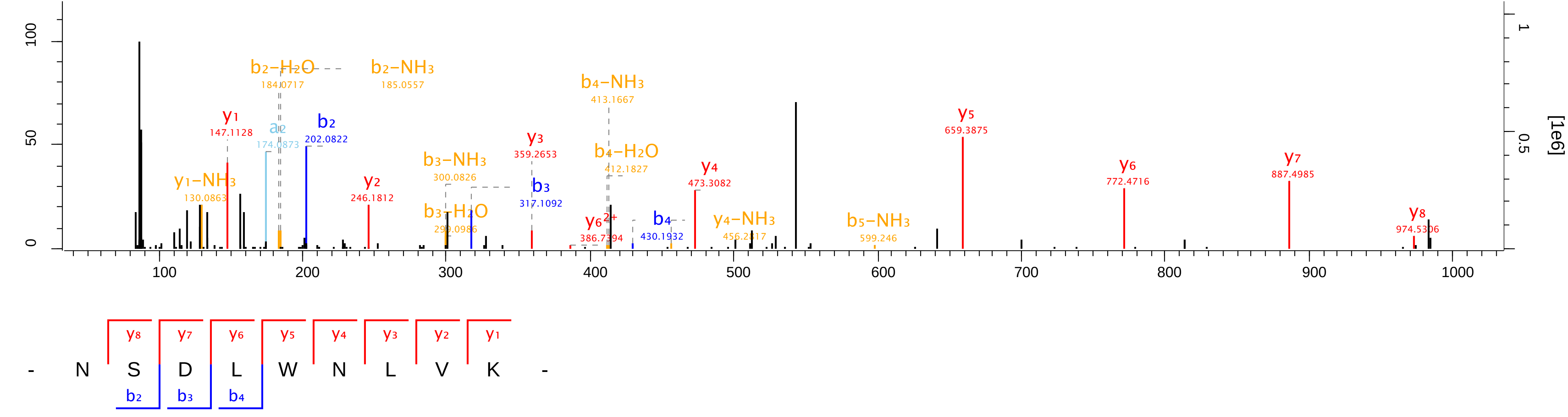

Raw file	Scan	Method	Score	m/z	Gene names
20140602_QEp4_FaHo_SA_BDF2_02	9741	FTMS; HCD	124.99	696.36	LRO1

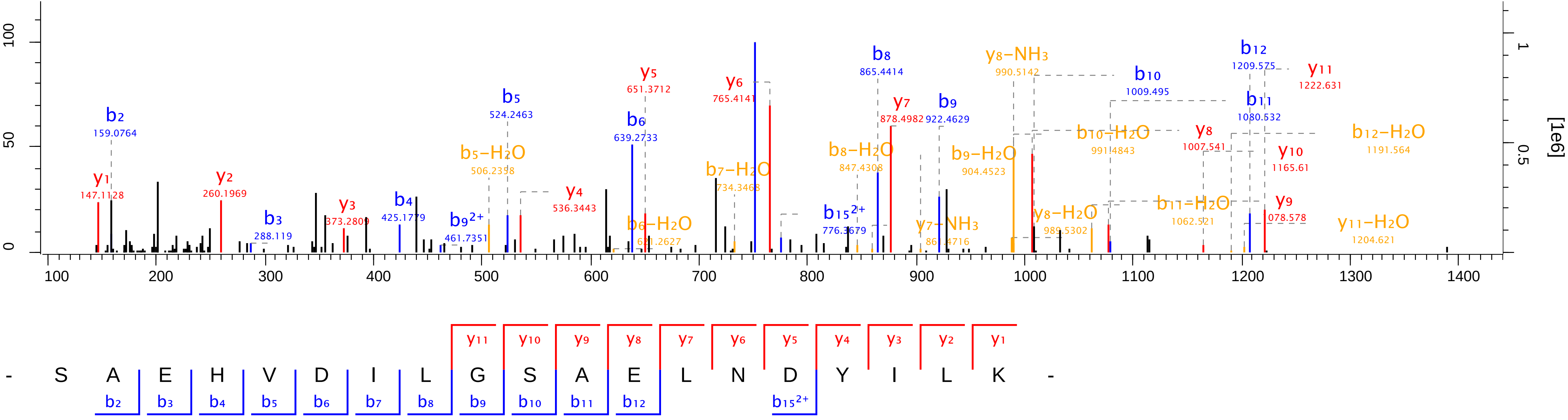

Raw file	Scan	Method	Score	m/z	Gene names
20140602_QEp4_FaHo_SA_BDF2_03	6289	FTMS; HCD	84.5	433.25	RPL13B;RPL13A

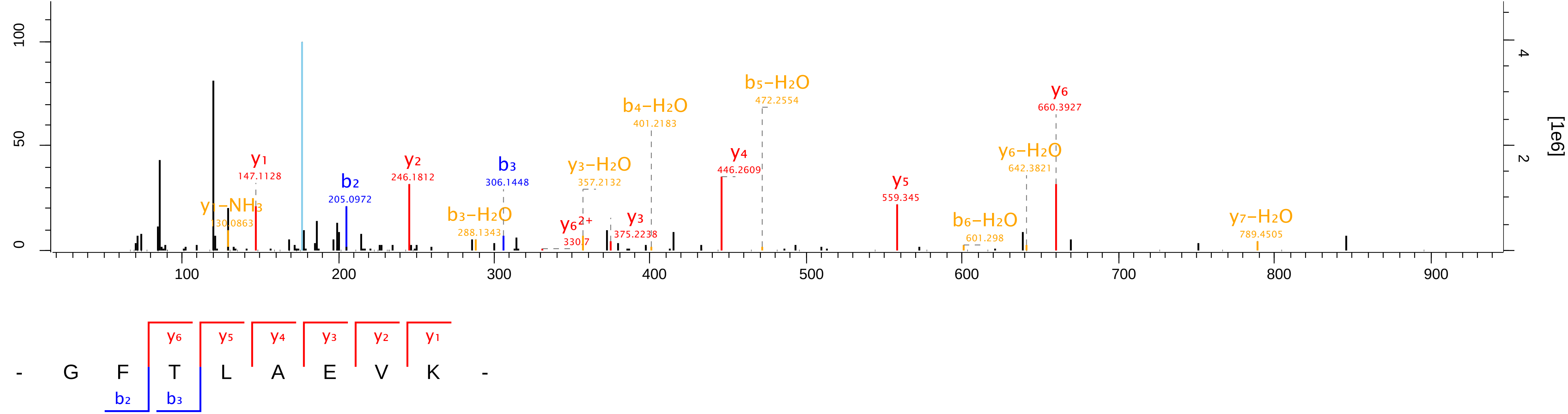

Raw file	Scan	Method	Score	m/z	Gene names
20140602_QEp4_FaHo_SA_BDF2_03	9040	FTMS; HCD	228.15	862.44	RPS14B;RPS14A

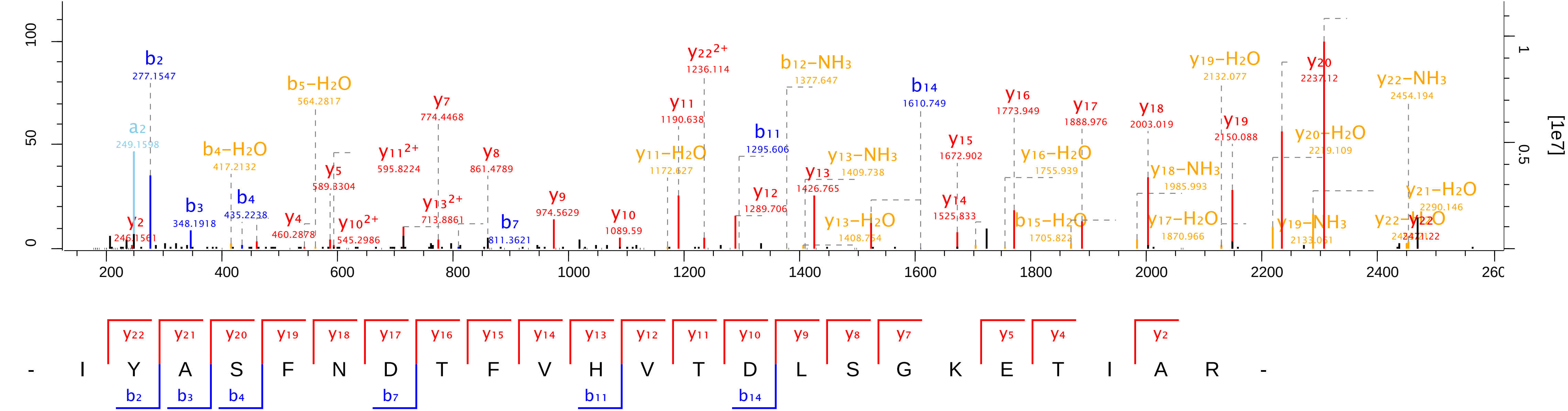

Raw file	Scan	Method	Score	m/z	Gene names
20140602_QEp4_FaHo_SA_CTI6_01	4213	FTMS; HCD	68.58	468.26	RPS14B;RPS14A

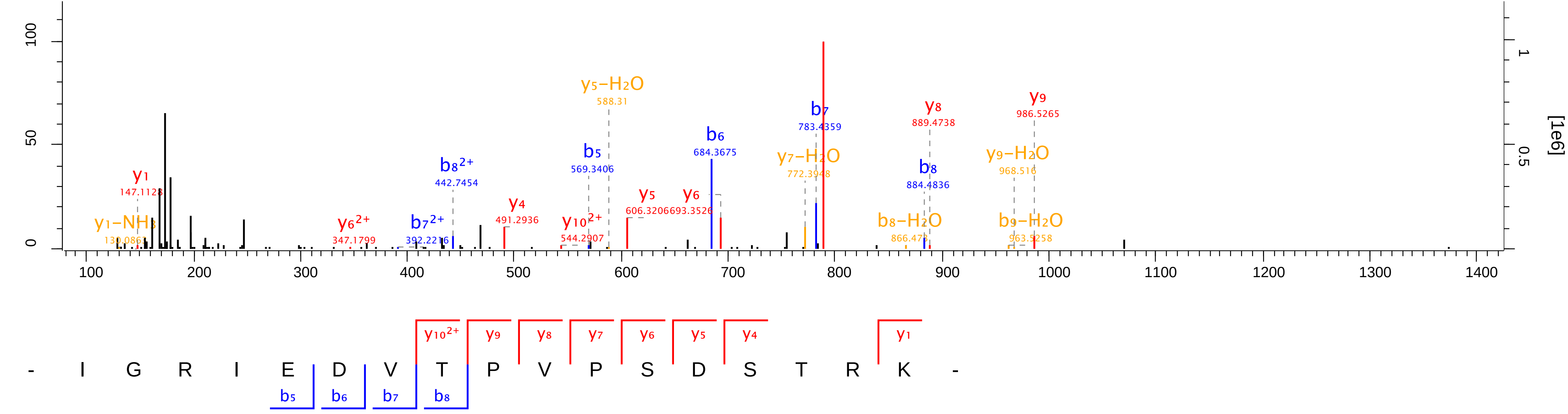

Raw file	Scan	Method	Score	m/z	Gene names
20140602_QEp4_FaHo_SA_CTI6_01	5974	FTMS; HCD	113.44	589.82	RPL9A;RPL9B

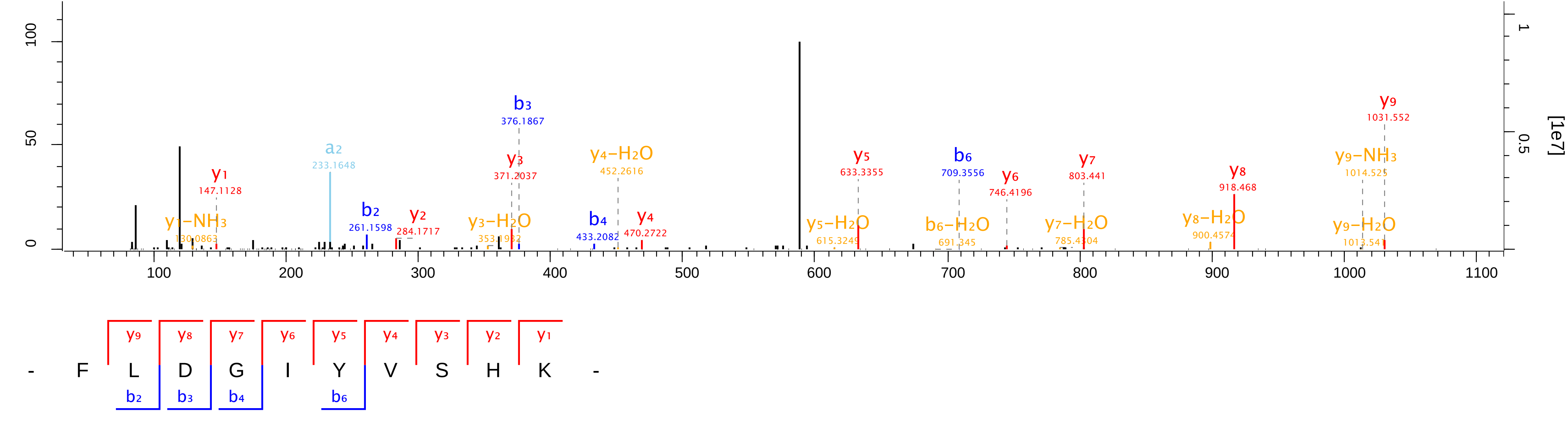

Raw file	Scan	Method	Score	m/z	Gene names
20140602_QEp4_FaHo_SA_CTI6_01	8912	FTMS; HCD	138.75	688.03	ERG11

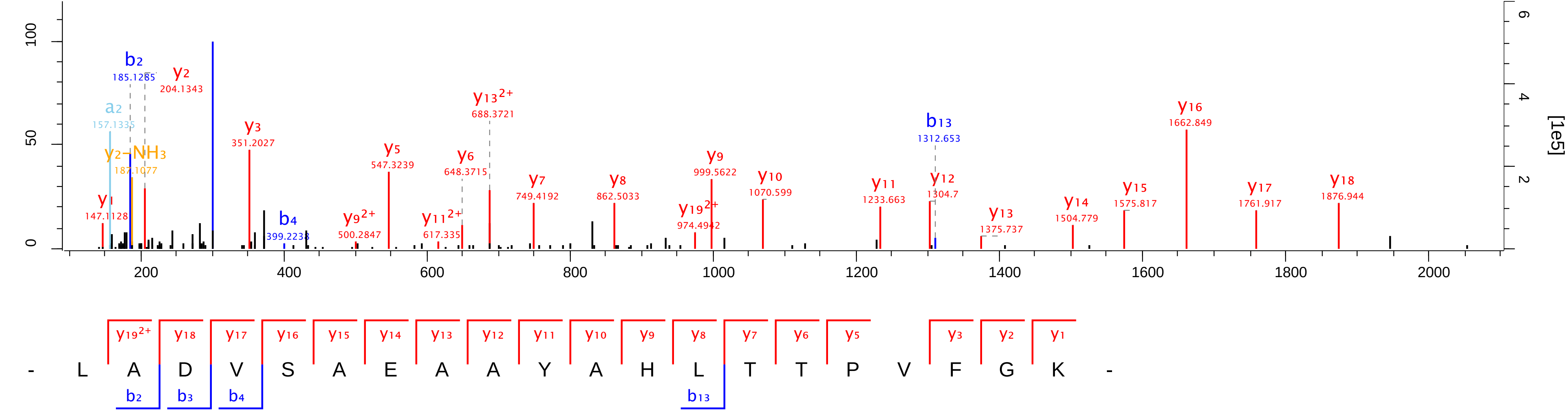

Raw file	Scai	Method	Score	m/z	Gene names
20140602_QEp4_FaHo_	286	FTMS;2	265.:	625.:	TY1B-ML1;TY1B-BR;TY1A-PR1;TY1A-A;TY1A-DR4;TY1B-H;TY1B-MR2;TY1B-OR;TY1B-DR1;TY1B-NL2;TY1B-PR2;TY1B-DR5;TY1B-PR1;TY1B-JR2;TY1A-PL;TY1A-LR2;TY1A-E

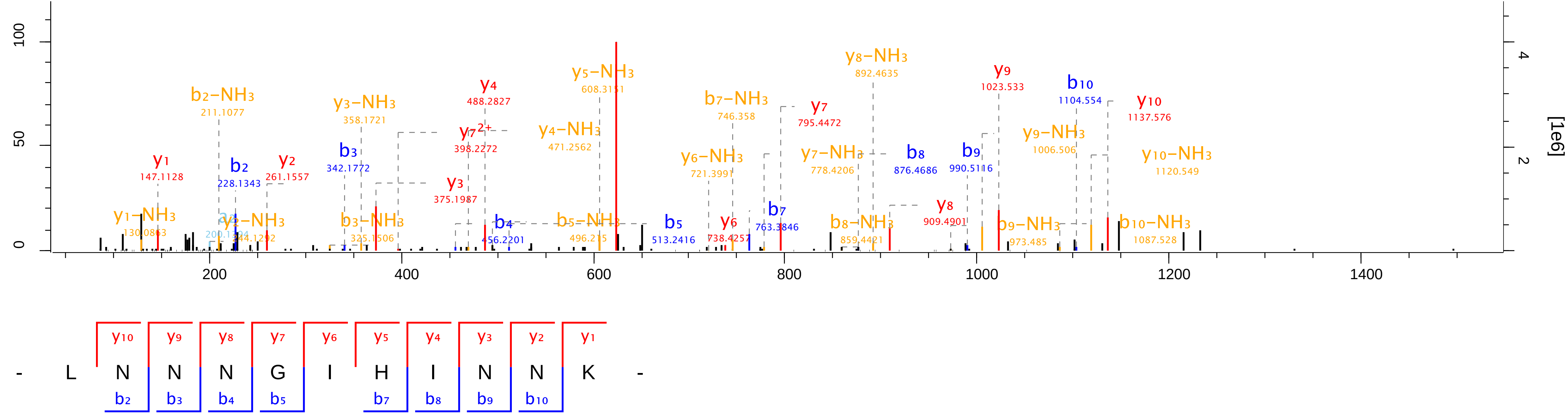

Raw file	Scan	Method	Score	m/z	Gene names
20140602_QEp4_FaHo_SA_CTI6_03	3966	FTMS; HCD	76.36	538.94	FLC3

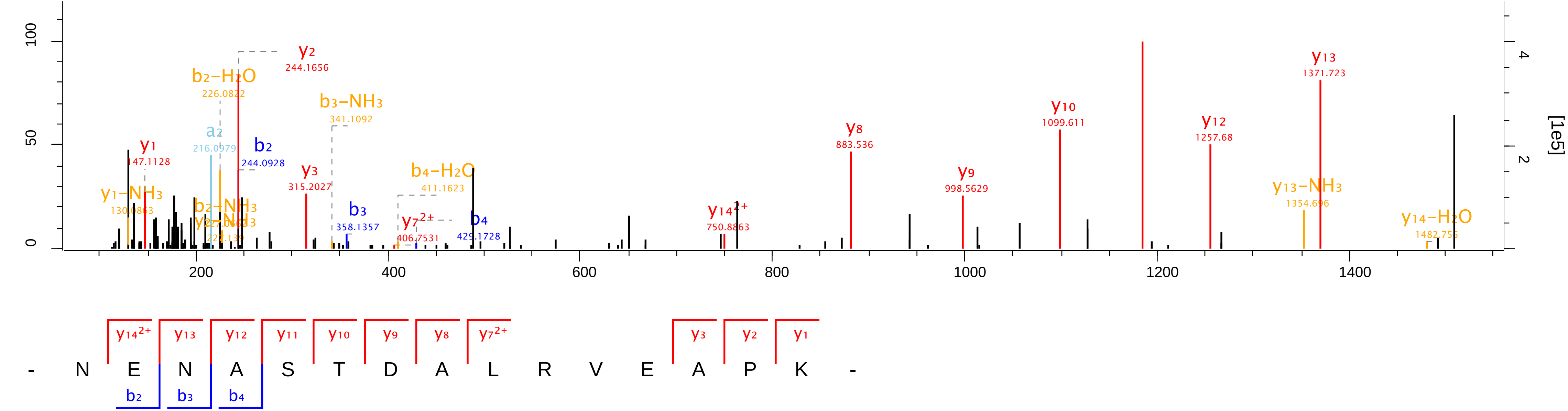

Raw file	Scan	Method	Score	m/z	Gene names
20140602_QEp4_FaHo_SA_CTI6_03	5196	FTMS; HCD	66.99	475.59	NOP13

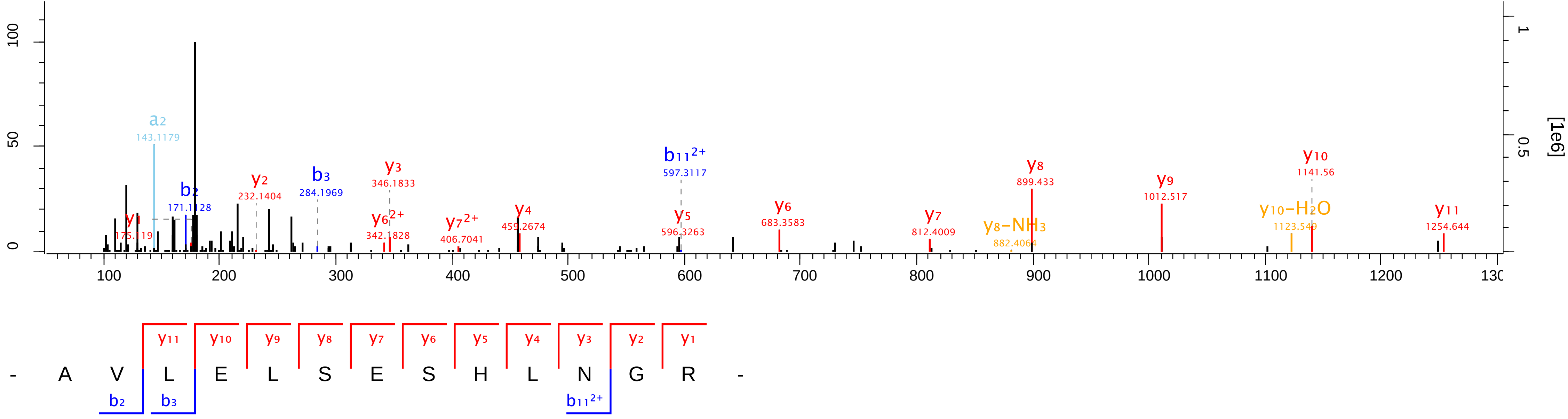

Raw file	Scan	Method	Score	m/z	Gene names
20140602_QEp4_FaHo_SA_CTI6_03	9108	FTMS; HCD	91.62	623.36	PUF6

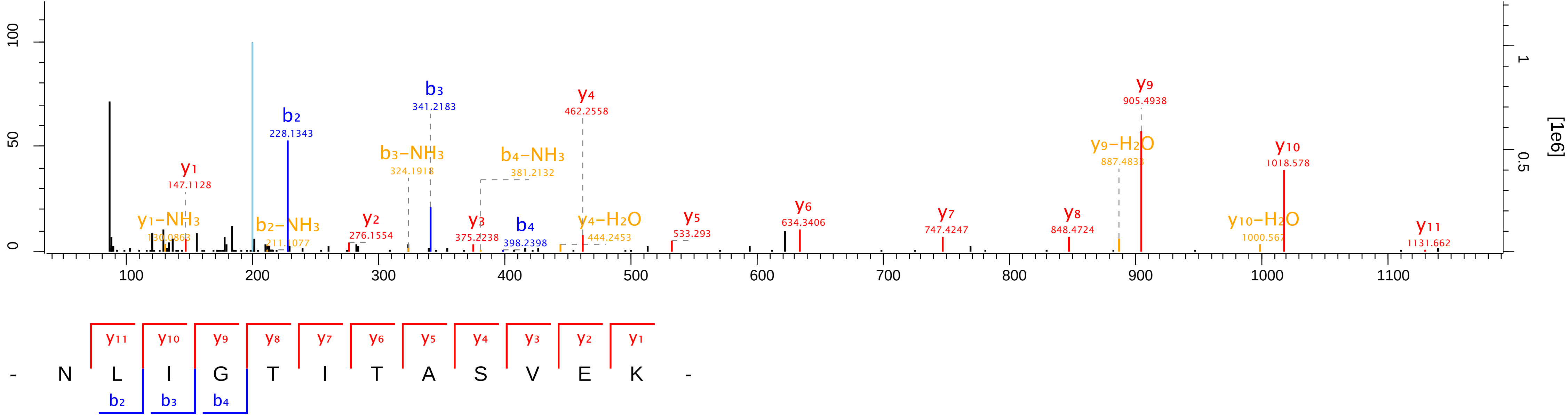

Raw file	Scan	Method	Score	m/z	Gene names
20140602_QEp4_FaHo_SA_EAF3_01	3197	FTMS; HCD	138.85	415.25	TY1B-OL

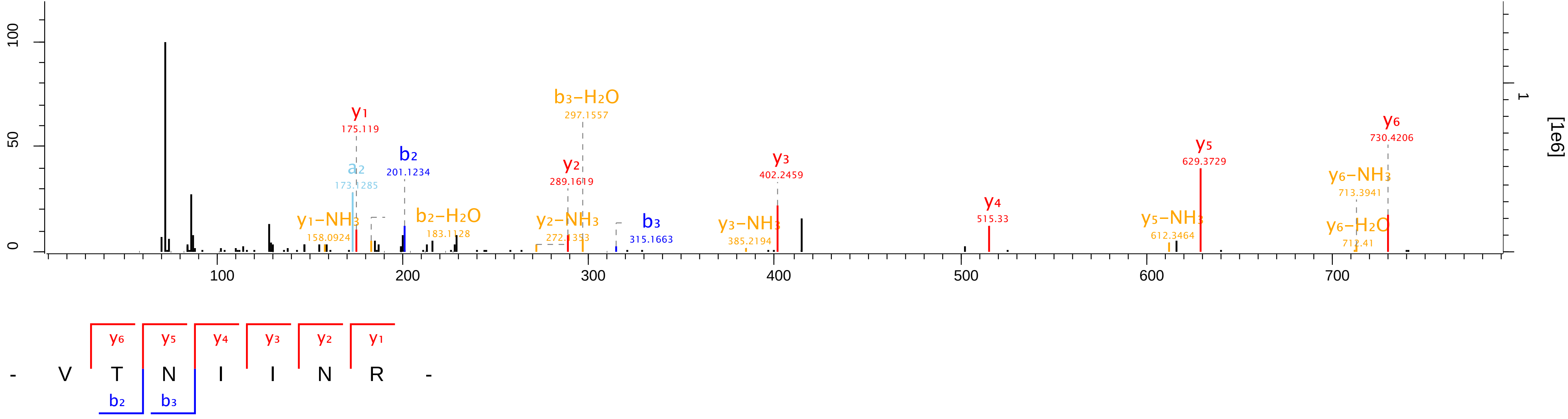

Raw file	Scan Method	Score	m/z	Gene names
20140602_QEp4_FaHo_SA_E 539[illegible]	FTMS;	53.5[illegible]	590.9	TY1B-ML1;TY1B-BR;TY1B-H;TY1B-MR2;TY1B-OR;TY1B-DR1;TY1B-PR2;TY1B-DR5;TY1B-PR1;TY1B-JR2;TY1B-OL;TY1B-LR4;TY1B-ML2;TY1B-DR3;TY1B-PR3;TY1B-P

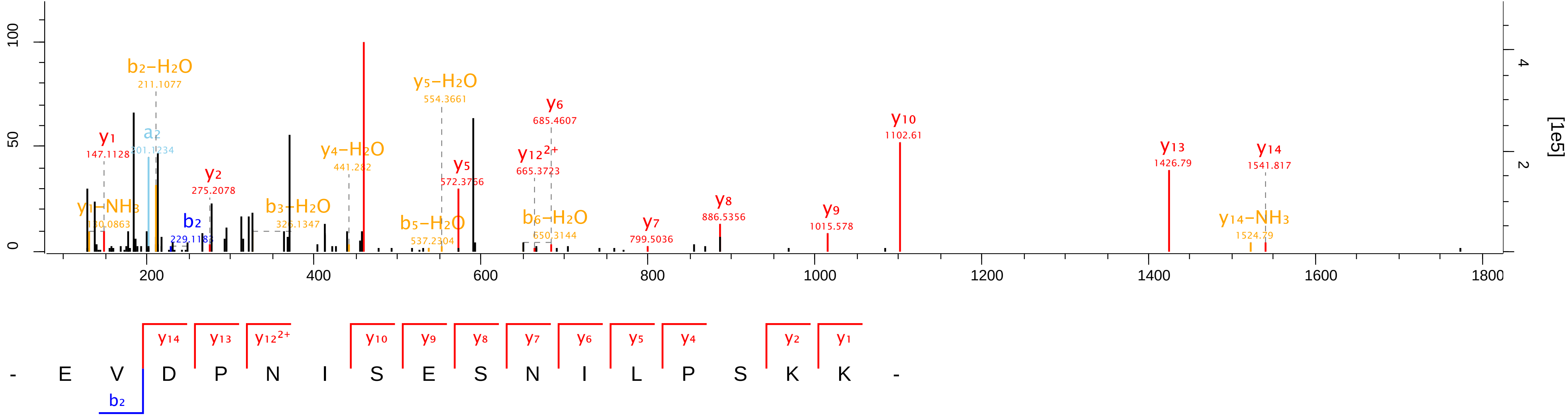

Raw file	Scar	Method	Score	m/z	Gene names
20140602_QEp4_FaHo_S	23E	FTMS;:	411.:	1038.	TY1B-ML1;TY1B-BR;TY1A-PR1;TY1A-A;TY1A-DR4;TY1B-H;TY1B-MR2;TY1B-OR;TY1B-DR1;TY1B-NL2;TY1B-PR2;TY1B-DR5;TY1B-PR1;TY1B-JR2;TY1A-PL;TY1A-LR2;TY1A

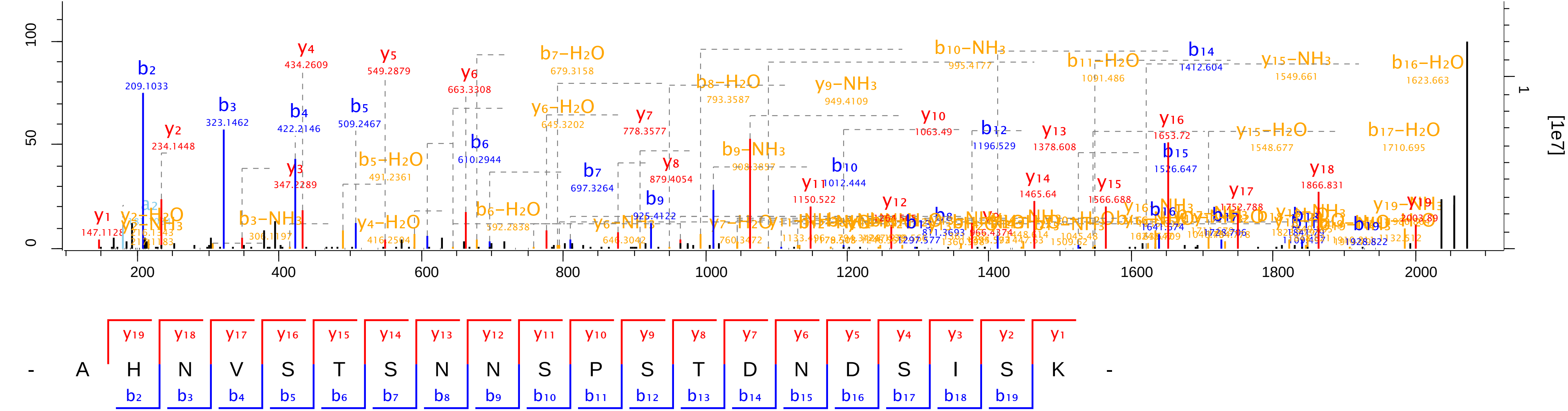

Raw file	Scar	Method	Score	m/z	Gene names
20140602_QEp4_FaHo_SA_	260	FTMS;	55.6	308.4	TY1B-ML1;TY1B-BR;TY1B-OR;TY1B-DR1;TY1B-NL2;TY1B-PR2;TY1B-DR5;TY1B-PR1;TY1B-JR2;TY1B-NL1;TY1B-OL;TY1B-LR4;TY1B-ML2;TY1B-DR3;TY1B-PR3;TY1B-PL;

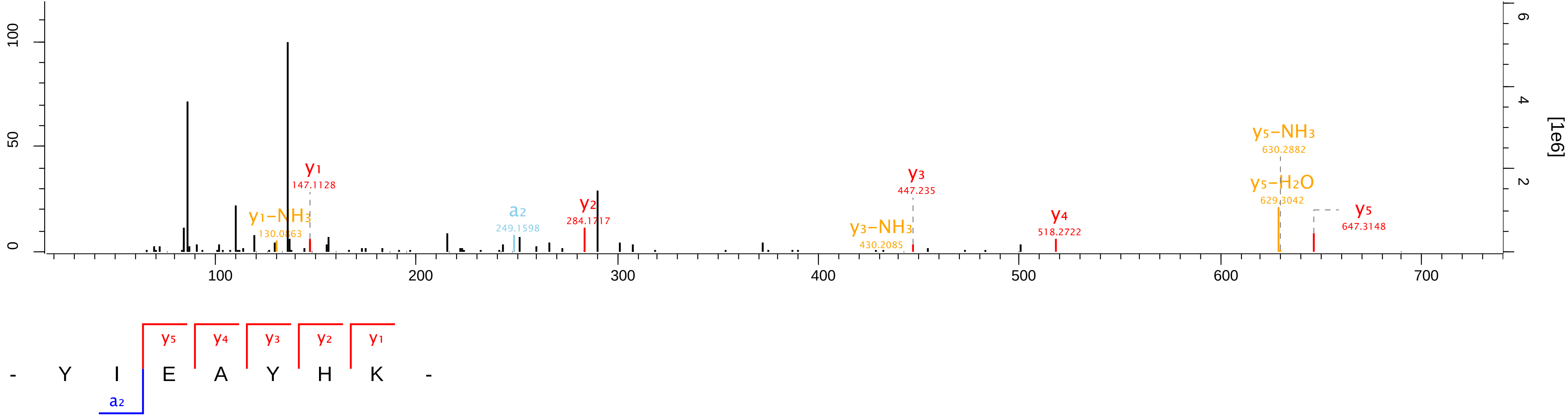

Raw file	Scan	Method	Score	m/z	Gene names
20140602_QEp4_FaHo_SA_EAF3_02	3519	FTMS; HCD	115.7	403.54	TY1A-PL;TY1A-LR2;TY1A-ER1;TY1A-DR6;TY1A-DR2

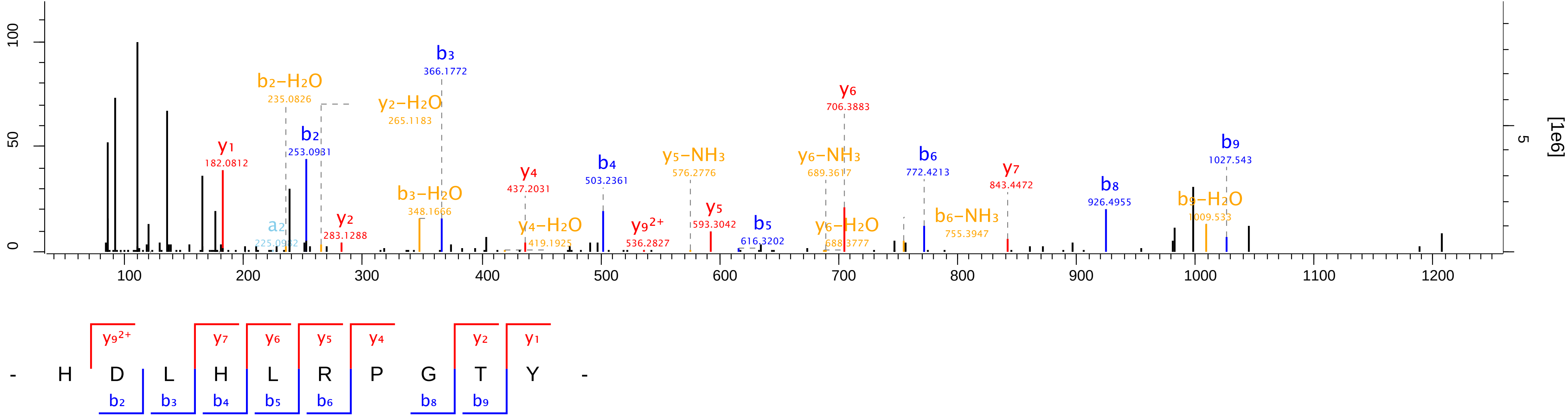

Raw file	Scan	Method	Score	m/z	Gene names
20140602_QEp4_FaHo_SA_EAF3_0	3637	FTMS; HCD	110.74	645.3	TY1B-ML1;TY1B-MR2;TY1B-OR;TY1B-DR1;TY1B-PR2;TY1B-DR5;TY1B-PR1;TY1B-JR2;TY1B-OL;TY1B-LR4;TY1B-ML2;TY1B-PR3;TY1B-PL;TY1B-LR2;TY1B-

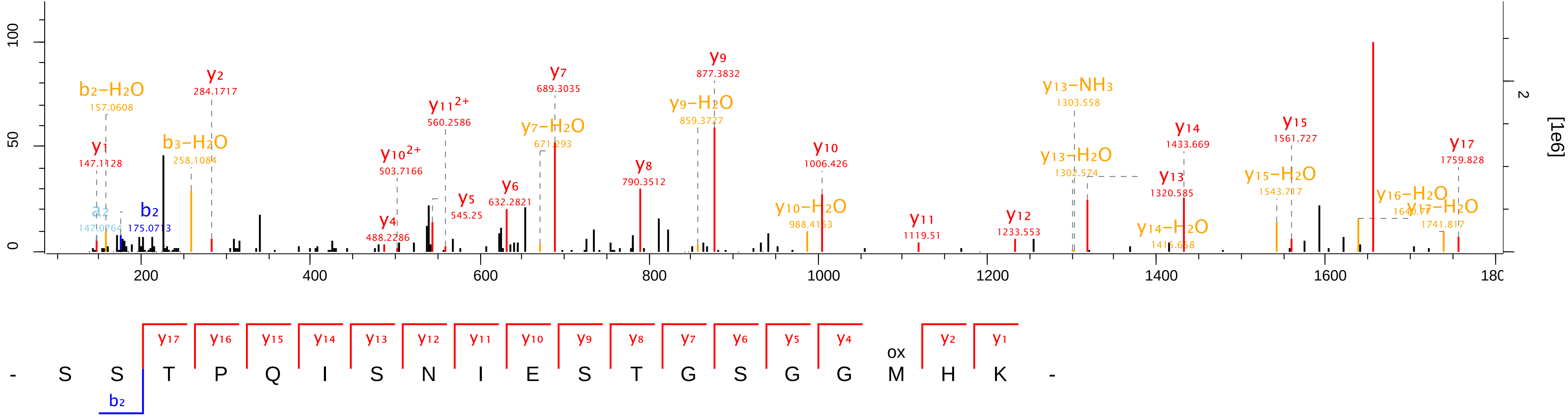

Raw file	Scan	Method	Score	m/z	Gene names
20140602_QEp4_FaHo_SA_EAF3_02	3709	FTMS; HCD	97.81	427.55	TY1A-PR1;TY1A-A;TY1A-DR4

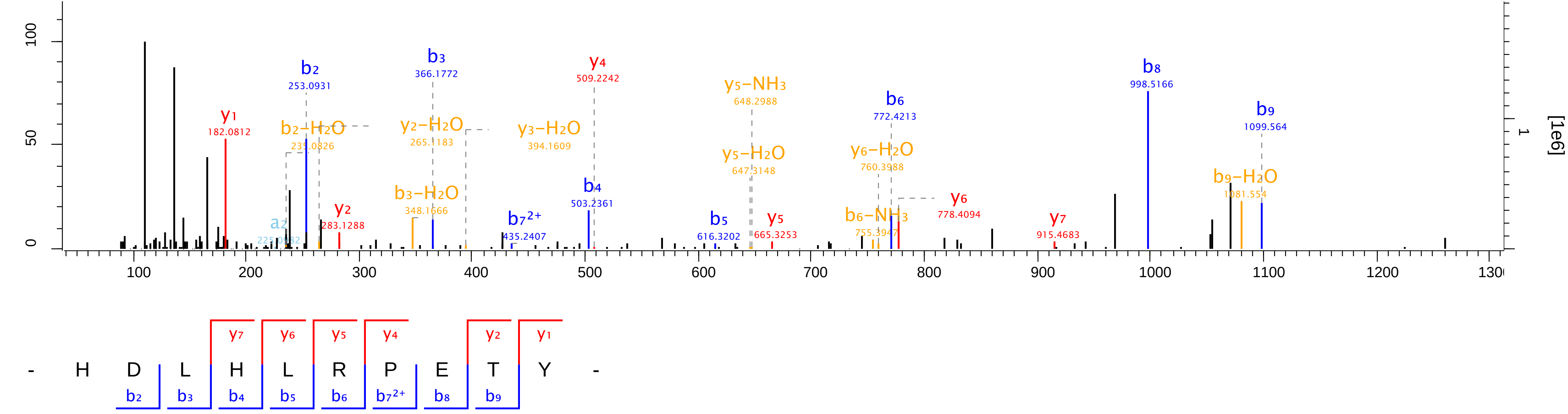

Raw file	Scan	Method	Score	m/z	Gene names
20140602_QEp4_FaHo_SA_	397[illegible]	FTMS;	98.4[illegible]	422.2	TY1B-ML1;TY1B-BR;TY1B-MR2;TY1B-OR;TY1B-DR1;TY1B-NL2;TY1B-PR2;TY1B-DR5;TY1B-PR1;TY1B-JR2;TY1B-NL1;TY1B-OL;TY1B-LR4;TY1B-ML2;TY1B-DR3;TY1B-P

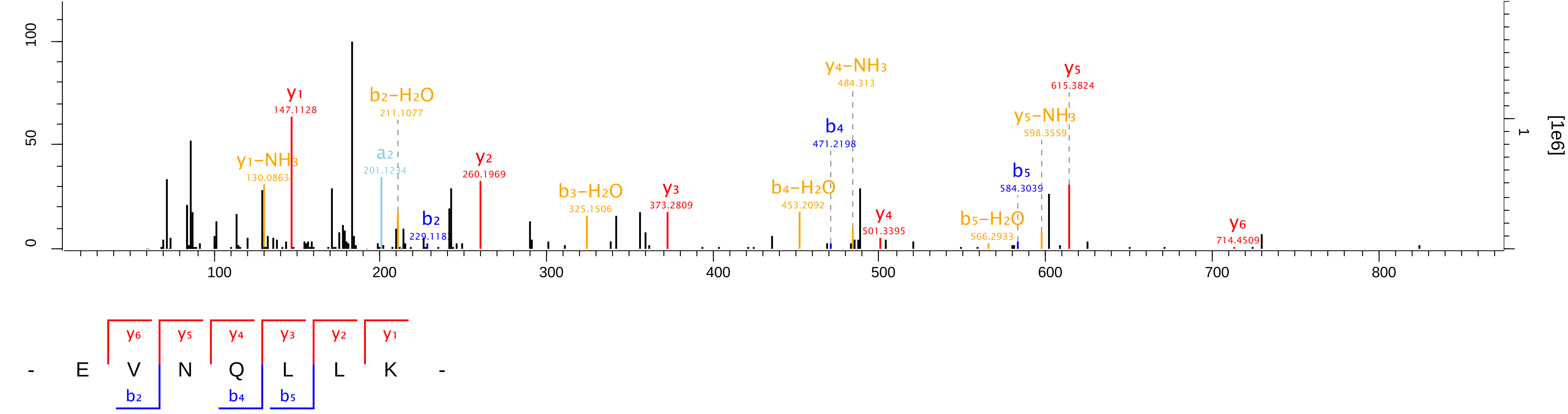

Raw file | Sca | Methc | Sco | m/z | Gene names

20140602_QEp4_Fa 42 A 63 389 TY1B-ML1;TY1B-BR;TY1B-H;TY1B-MR2;TY1B-OR;TY1B-DR1;TY1B-NL2;TY1B-PR2;TY1B-DR5;TY1B-PR1;TY1B-JR2;TY1B-NL1;TY2B-C;TY1B-OL;TY1B-LR4;TY1B-ML2;TY1B-DR3;TY1B

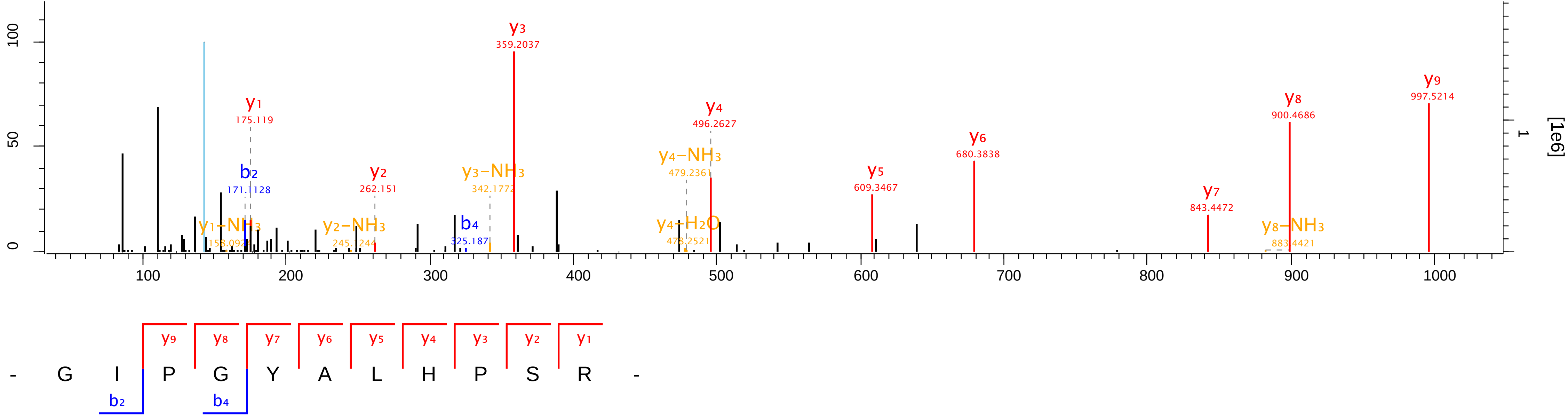

Raw file	Scan	Method	Score	m/z	Gene names
20140602_QEp4_FaHo_SA_EAF	4562	FTMS;	94.02	492.9	TY1B-ML1;TY1B-BR;TY1B-H;TY1B-MR2;TY1B-OR;TY1B-PR2;TY1B-DR5;TY1B-PR1;TY1B-JR2;TY1B-NL1;TY1B-OL;TY1B-LR4;TY1B-ML2;TY1B-PR3;TY1B-PL;TY1B-

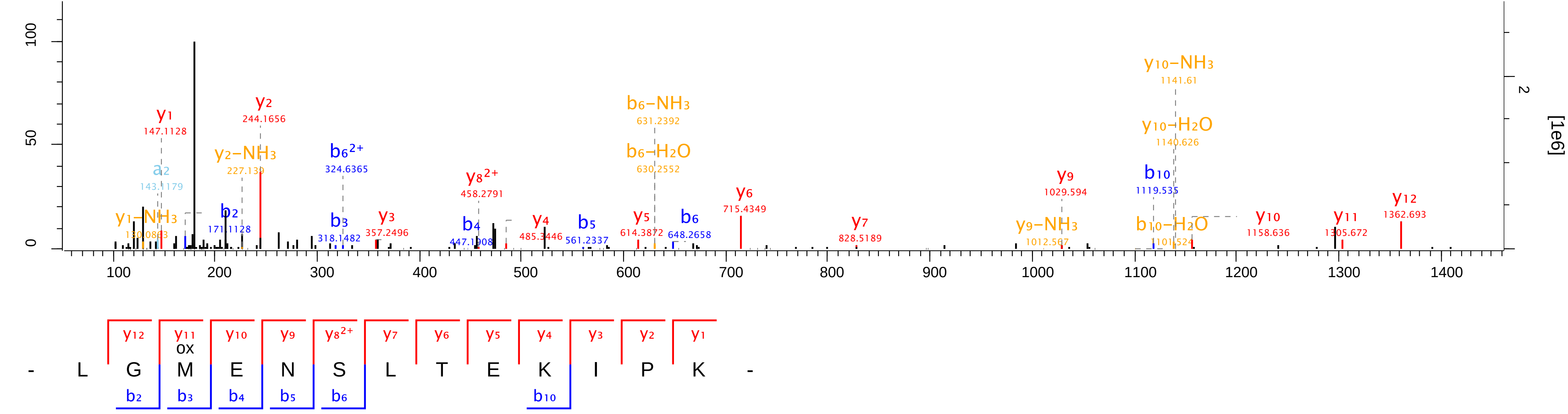

Raw file	Scan	Method	Score	m/z	Gene names
20140602_QEp4_FaHo_SA_	589	FTMS;	120.6	364.8	TY1B-ML1;TY1B-BR;TY1B-H;TY1B-MR2;TY1B-OR;TY1B-DR1;TY1B-NL2;TY1B-PR2;TY1B-DR5;TY1B-PR1;TY1B-JR2;TY1B-NL1;TY1B-OL;TY1B-LR4;TY1B-ML2;TY1B-DR3

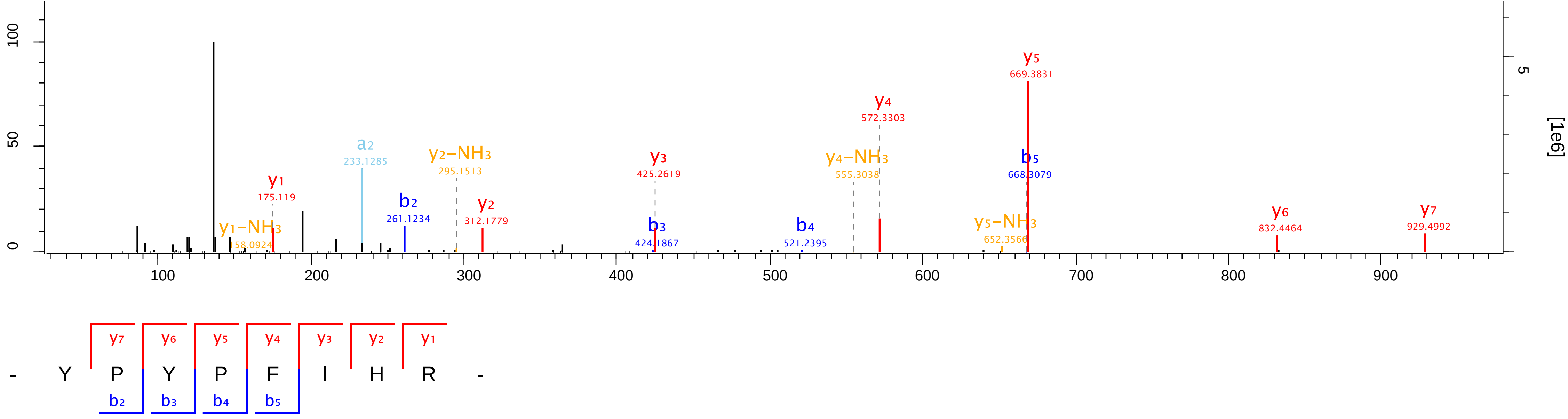

Raw file	Scan	Method	Score	m/z	Gene names
20140602_QEp4_FaHo_SA_EAF3	5992	FTMS;	137.1	479.7	TY2B-C;TY2B-B;TY2B-GR2;TY2B-F;TY2B-GR1;TY2A-GR1;TY2B-OR1;TY2A-DR2;TY2B-DR1;TY2B-DR3;TY2B-LR1;TY2B-OR2;TY2A-OR1;TY2A-LR2;TY2A-OR2;

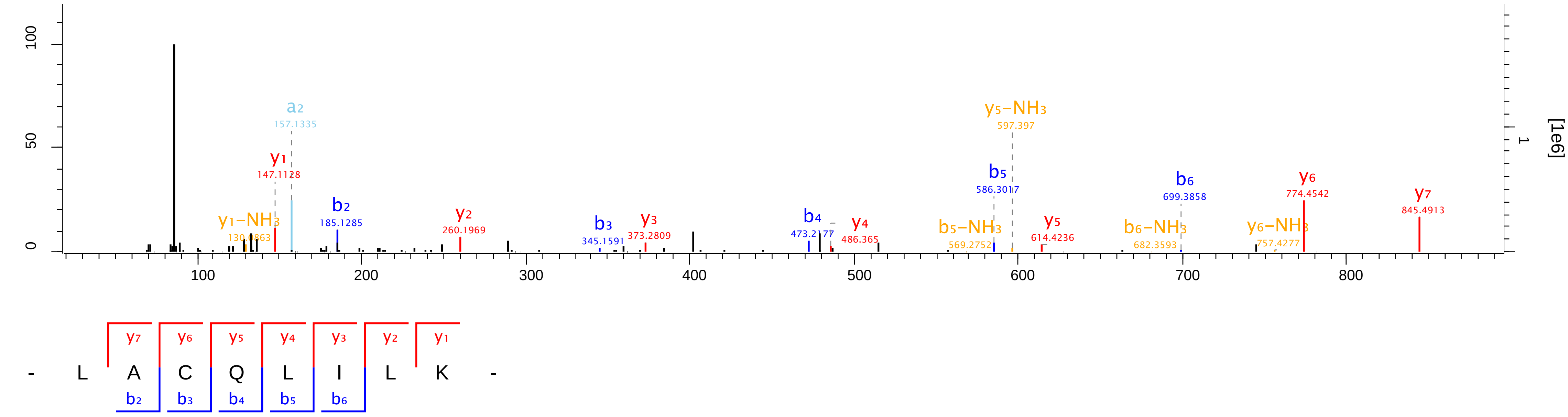

Raw file	Scan Method	Score	m/z	Gene names
20140602_QEp4_FaHo_SA_EA	6180 FTMS;	54.27	431.5	TY1B-ML1;TY1B-BR;TY1B-H;TY1B-MR2;TY1B-OR;TY1B-DR1;TY1B-NL2;TY1B-PR2;TY1B-DR5;TY1B-PR1;TY1B-JR2;TY1B-NL1;TY1B-OL;TY1B-LR4;TY1B-ML2;TY1B-

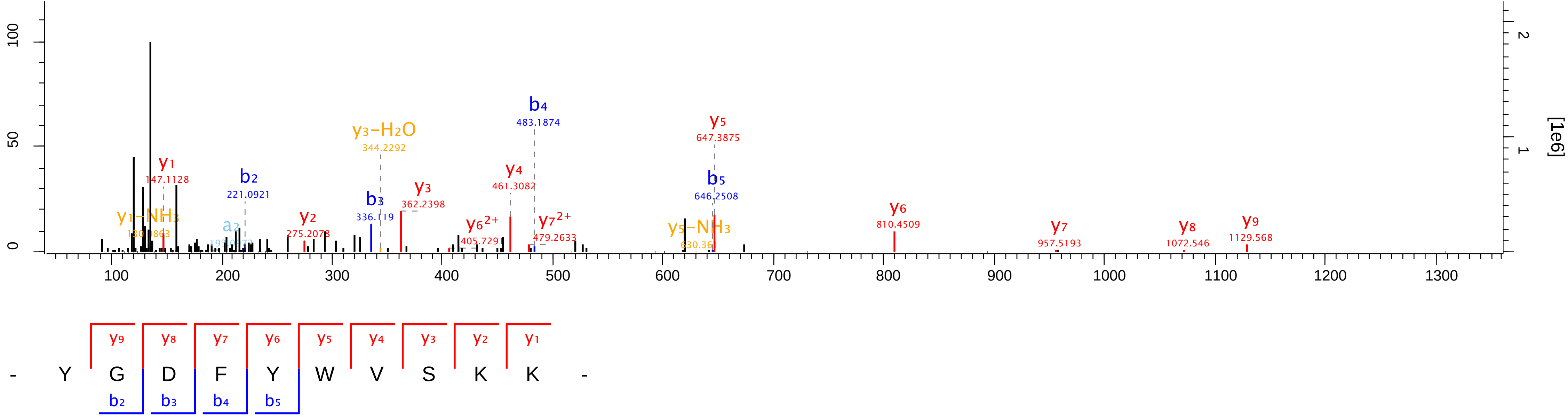

Raw file	Scan Method	Score	m/z	Gene names
20140602_QEp4_FaHo_SA_E 706	FTMS;	107.5	614.8	TY1B-PR1;TY1B-JR2;TY2B-C;TY1B-OL;TY1B-LR4;TY1B-ML2;TY1B-DR3;TY1B-PR3;TY1B-PL;TY1B-LR2;TY1B-DR6;TY1B-A;TY2B-B;TY2B-GR2;TY2B-F;TY2B-GR1;TY2

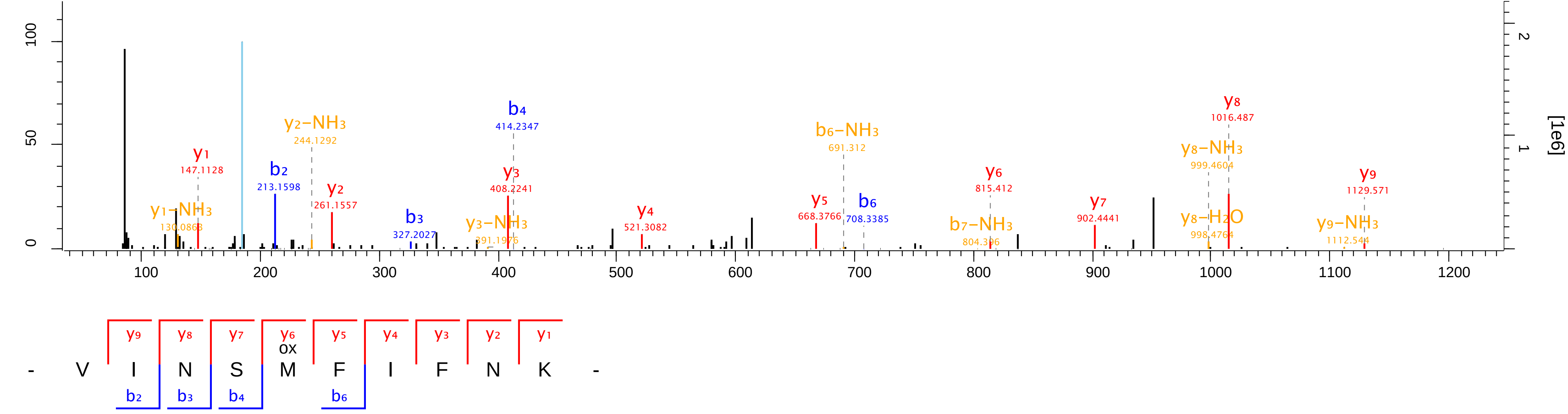

Raw file	Scan Method	Score	m/z	Gene names
20140602_QEp4_FaHo_SA_EA 798[illegible]	FTMS;	114.5[illegible]	726.0[illegible]	TY1B-DR1;TY1B-NL2;TY1B-PR1;TY2B-C;TY1B-OL;TY1B-LR4;TY1B-ML2;TY1B-DR3;TY1B-PL;TY1B-LR2;TY1B-DR6;TY1B-A;TY2B-B;TY2B-GR2;TY2B-F;TY2B-GR1;T[illegible]

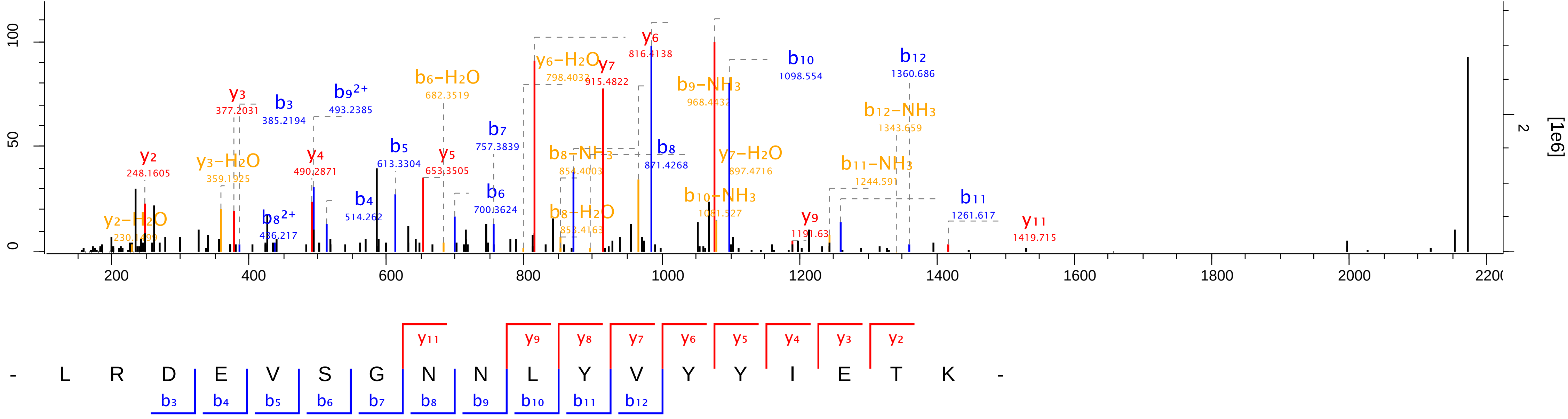

Raw file	Sca	Methc	Sco	m/z	Gene names
20140602_QEp4_Fa	83[illegible]	A	88[illegible]	592	TY1B-ML1;TY1B-BR;TY1B-H;TY1B-MR2;TY1B-OR;TY1B-DR1;TY1B-NL2;TY1B-PR2;TY1B-DR5;TY1B-PR1;TY1B-JR2;TY1B-NL1;TY2B-C;TY1B-OL;TY1B-LR4;TY1B-ML2;TY1B-DR3;TY1B[illegible]

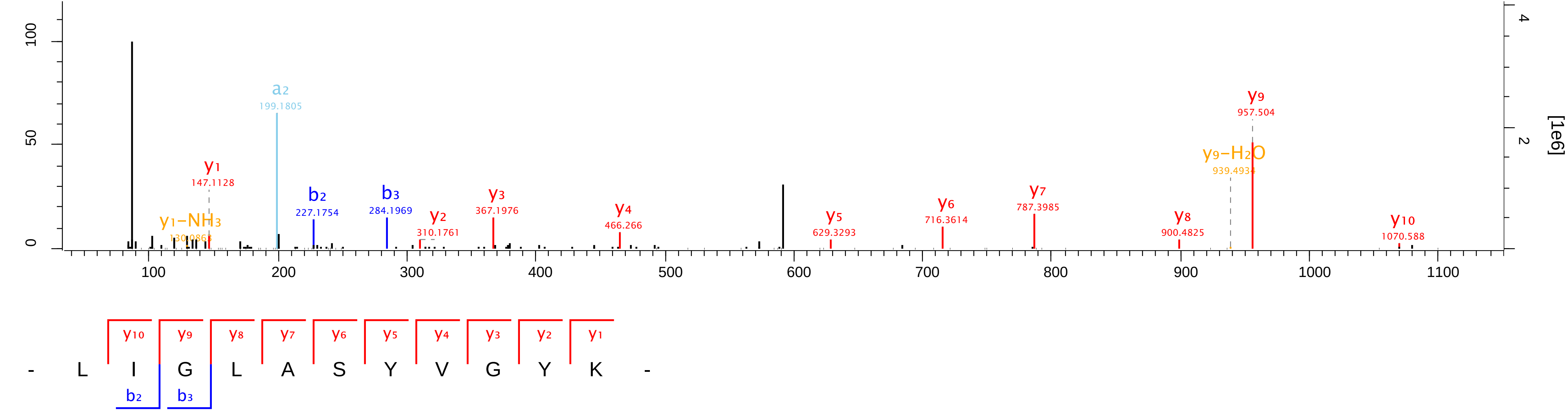

Raw file	Scan	Method	Score	m/z	Gene names
20140602_QEp4_FaHo_SA_	219[illegible]	FTMS;	183.7	503.9	TY1B-ML1;TY1B-BR;TY1B-H;TY1B-MR2;TY1B-OR;TY1B-DR1;TY1B-NL2;TY1B-PR2;TY1B-DR5;TY1B-PR1;TY1B-JR2;TY1B-NL1;TY1B-OL;TY1B-LR4;TY1B-ML2;TY1B-DR

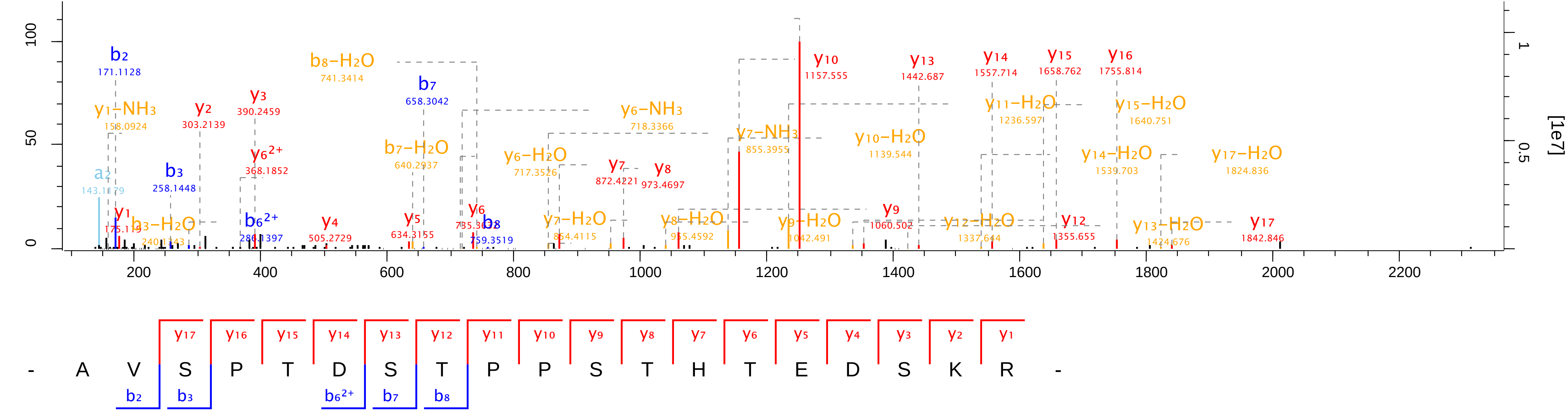

Raw file	Scan	Method	Score	m/z	Gene names
20140602_QEp4_FaHo_SA_E	268	FTMS;	74.61	409.2	TY1B-ML1;TY1B-BR;TY1B-H;TY1B-MR2;TY1B-OR;TY1B-DR1;TY1B-NL2;TY1B-PR2;TY1B-DR5;TY1B-PR1;TY1B-JR2;TY1B-NL1;TY1B-OL;TY1B-LR4;TY1B-ML2;TY1B-DI

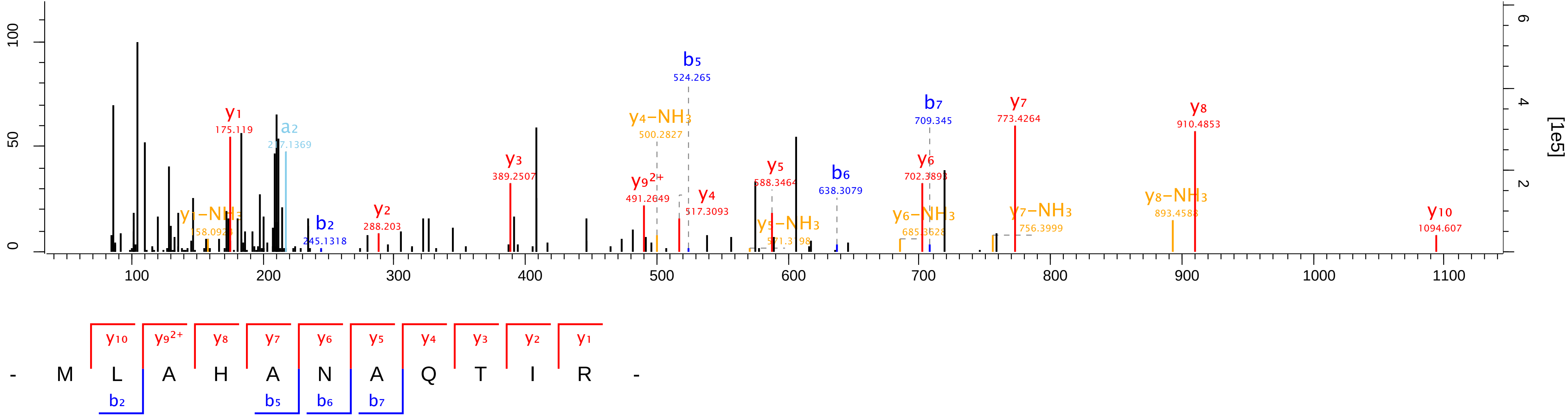

Raw file	Scan	Method	Score	m/z	Gene names
20140602_QEp4_FaHo_SA_EAF3_03	3033	FTMS; HCD	183.01	667.31	TY1B-LR4

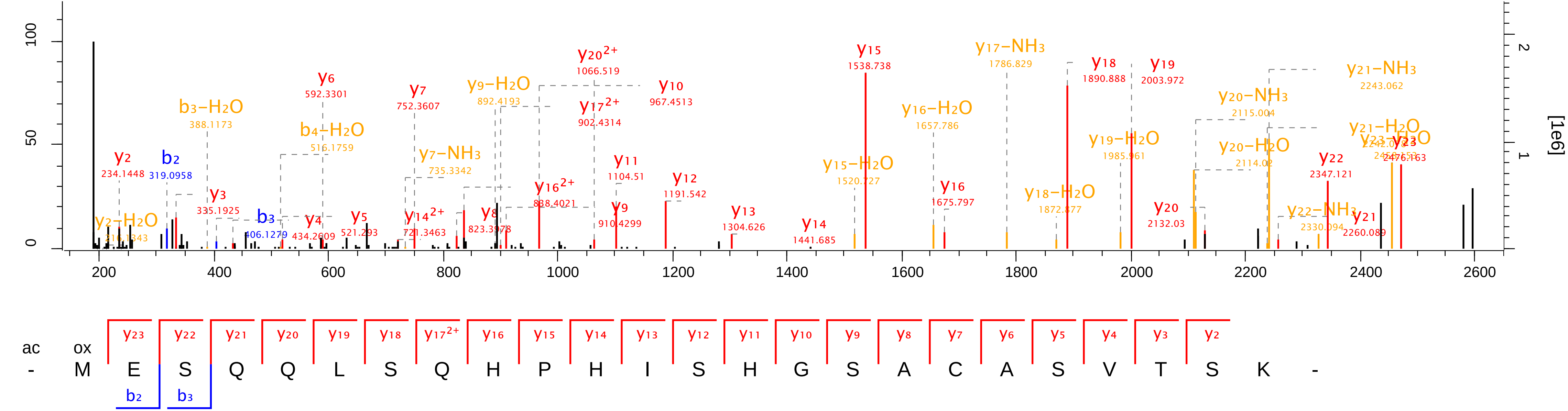

Raw file	Scar	Method	Score	m/z	Gene names
20140602_QEp4_FaHo_SA_	376	FTMS;	40.0	491.5	TY1B-ML1;TY1B-BR;TY1B-H;TY1B-MR2;TY1B-OR;TY1B-DR1;TY1B-NL2;TY1B-PR2;TY1B-DR5;TY1B-PR1;TY1B-JR2;TY1B-NL1;TY1B-OL;TY1B-LR4;TY1B-ML2;TY1B-DR3;

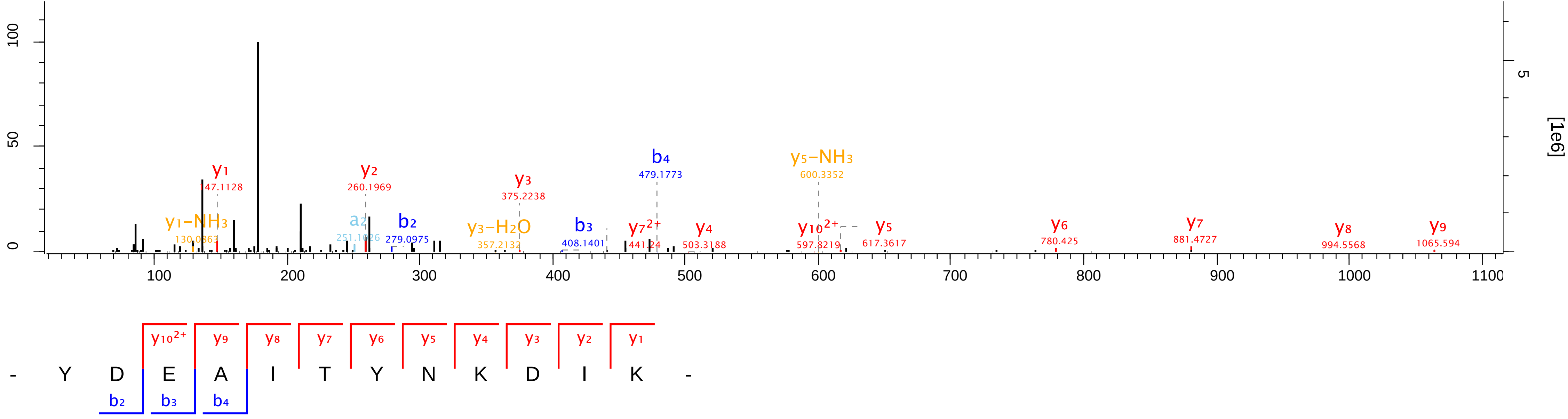

Raw file	Scan	Method	Score	m/z	Gene names
20140602_QEp4_FaHo_S	62[illegible]	FTMS;	179.[illegible]	894.[illegible]	TY1B-ML1;TY1B-H;TY1B-DR1;TY1B-NL2;TY1B-PR2;TY1B-DR5;TY1B-PR1;TY2B-C;TY1B-OL;TY1B-LR4;TY1B-ML2;TY1B-DR3;TY1B-PR3;TY1B-PL;TY1B-LR2;TY1B-DR6;TY1E

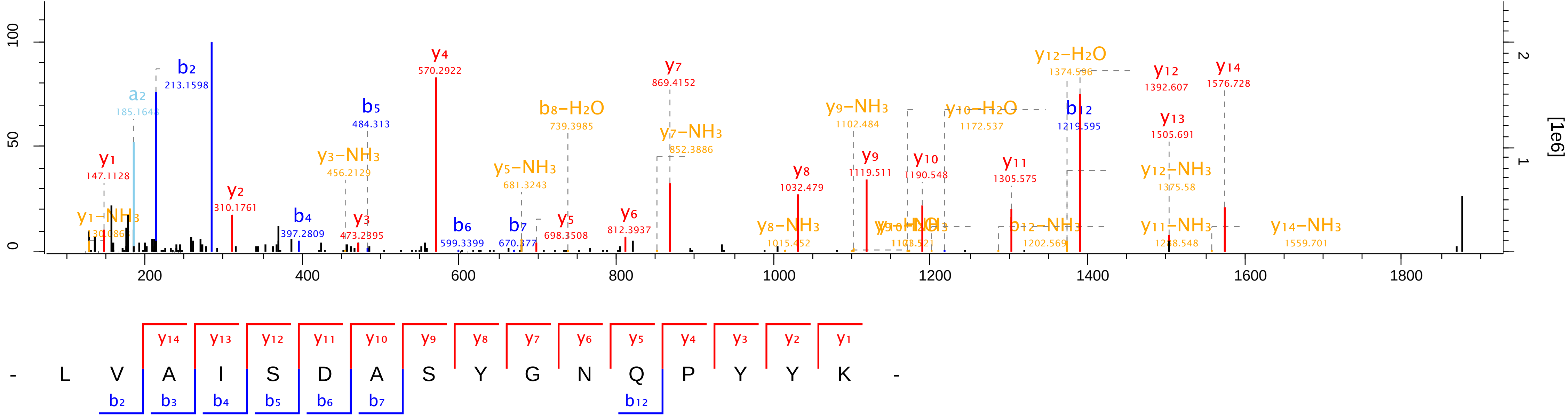

Raw file	Scan	Method	Score	m/z	Gene names
20140602_QEp4_FaHo_SA_EAF3_0	9689	FTMS; HCD	132.39	788.15	TY1B-ML1;TY1B-BR;TY1B-H;TY1B-MR2;TY1B-OR;TY1B-NL2;TY1B-PR2;TY1B-PR1;TY1B-JR2;TY1B-NL1;TY1B-OL;TY1B-ML2;TY1B-PR3;TY1B-BL;TY1B-M

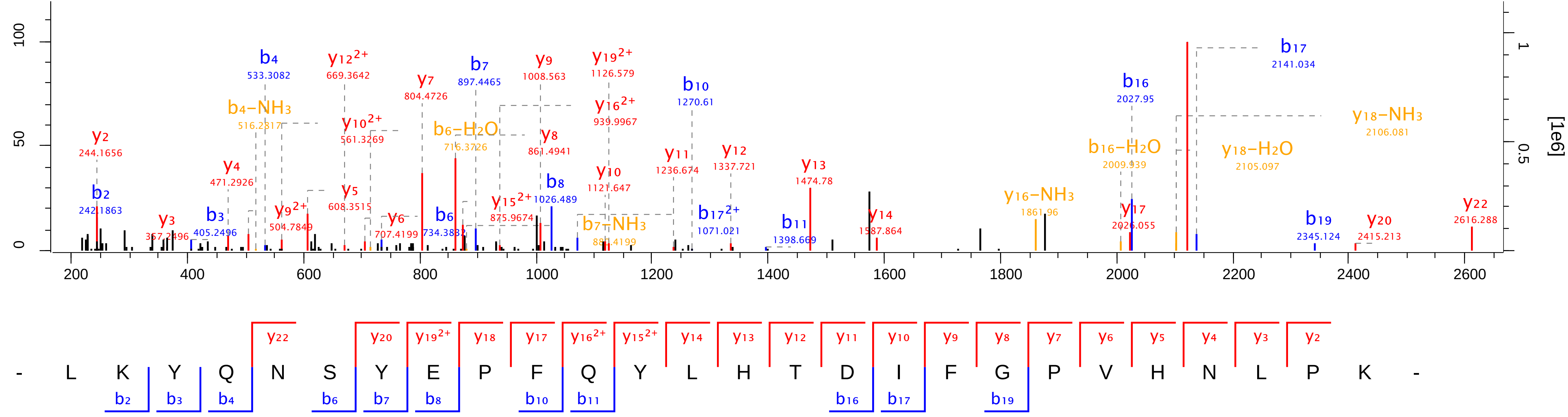

Raw file	Scan	Method	Score	m/z	Gene names
20140602_QEp4_FaHo_SA_ECM5_03	5374	FTMS; HCD	116.03	437.89	RPL24A

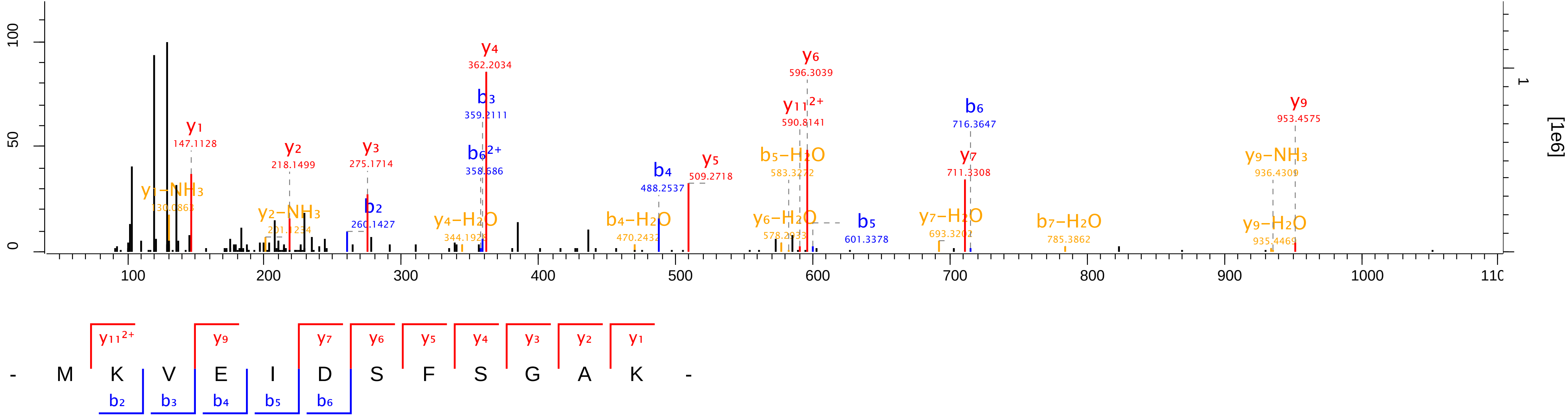

Raw file	Scar Method	Score	m/z	Gene names
20140602_QEp4_FaHo_SA_62	M FTMS;	196.1	726.3	TY1B-ML1;TY1B-BR;TY1B-H;TY1B-MR2;TY1B-OR;TY1B-DR1;TY1B-NL2;TY1B-PR2;TY1B-DR5;TY1B-PR1;TY1B-JR2;TY1B-NL1;TY1B-OL;TY1B-LR4;TY1B-ML2;TY1B-DR3;

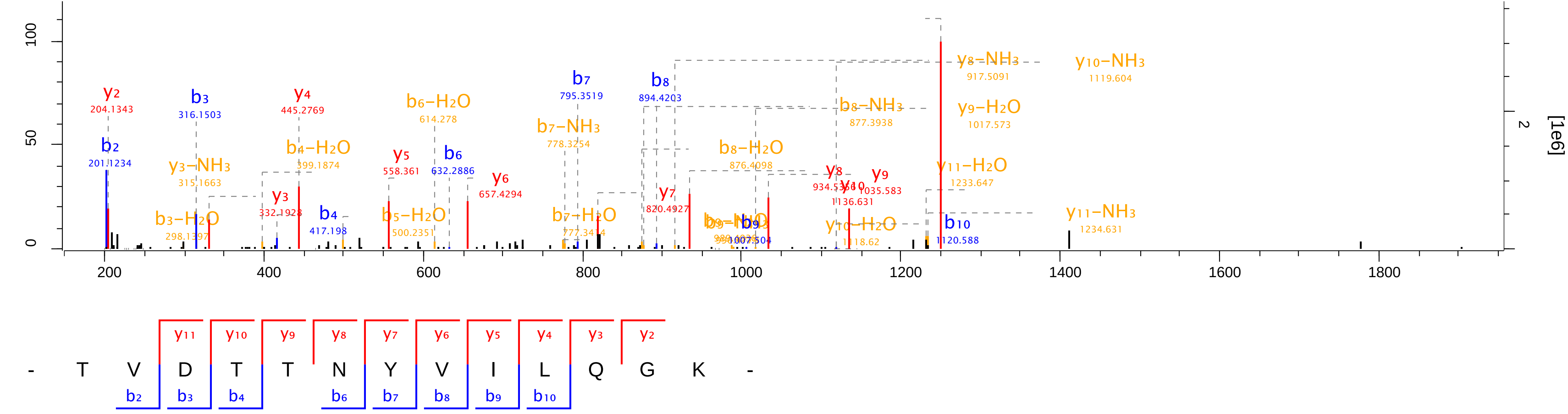

Raw file	Scan	Method	Score	m/z	Gene names
20140602_QEp4_FaHo_SA_ECM5_03	9565	FTMS; HCD	171.21	797.39	ARF1;ARF2

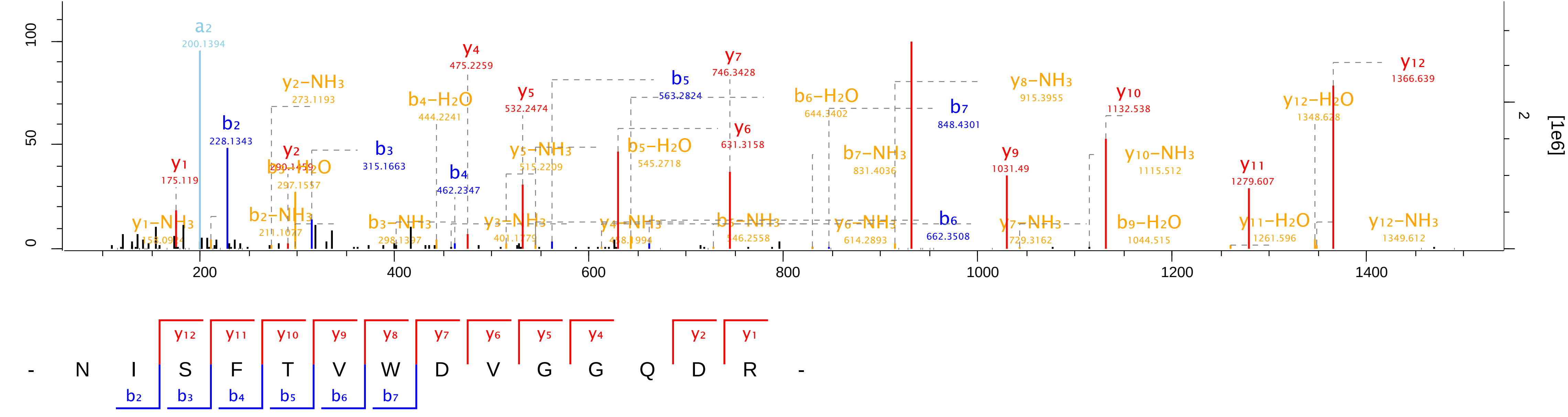

Raw file	Scan	Method	Score	m/z
20140602_QEp4_FaHo_SA_GCN5_01	4728	FTMS; HCD	14.16	495.25

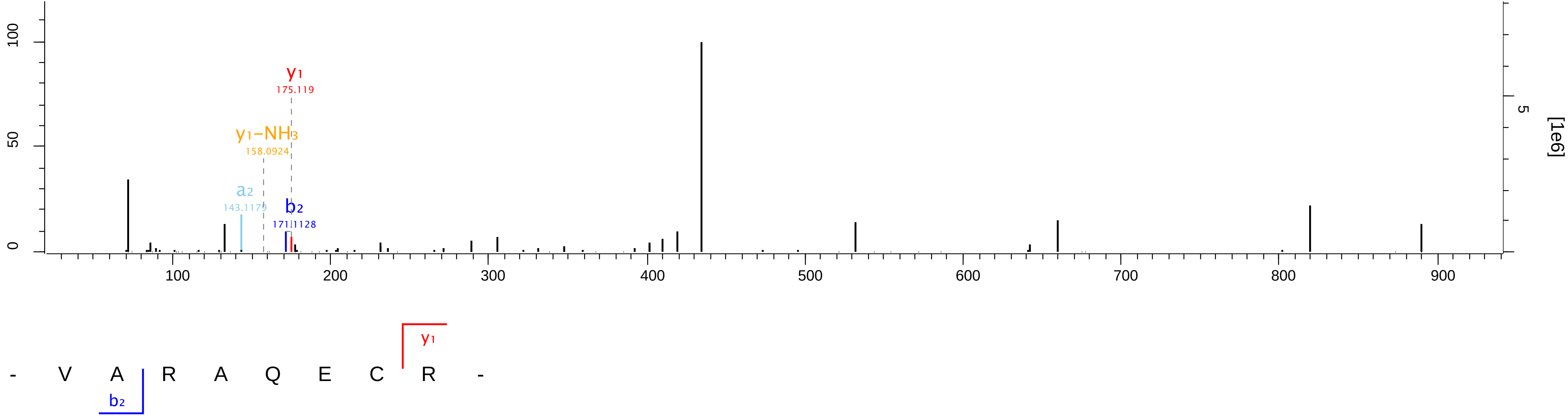

Raw file	Scan	Method	Score	m/z	Gene names
20140602_QEp4_FaHo_SA_GCN5_01	6510	FTMS; HCD	207.04	731.38	RPL17A;RPL17B

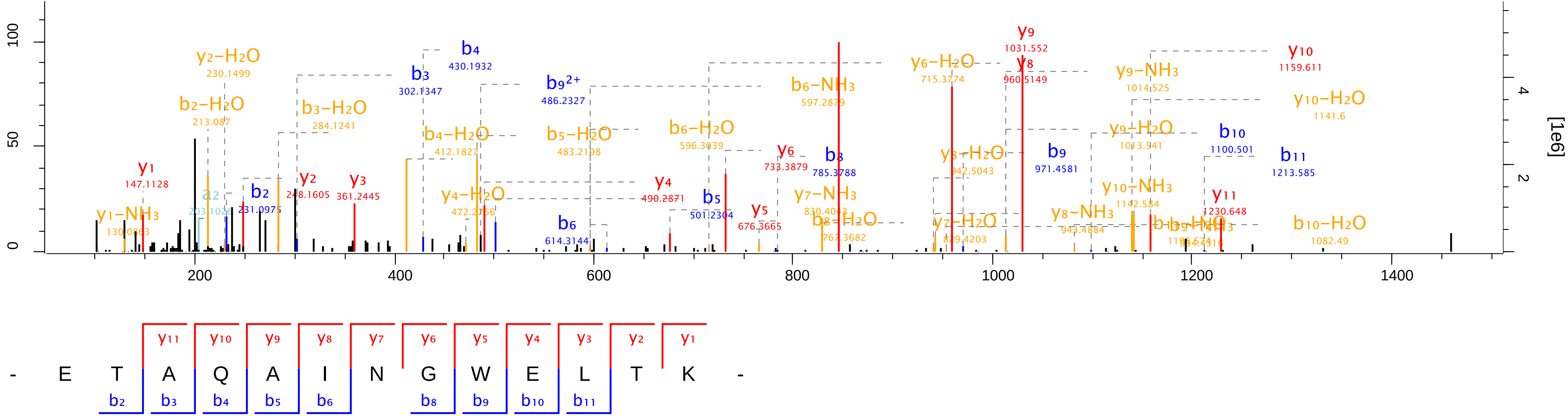

Raw file	Scan	Method	Score	m/z	Gene names
20140602_QEp4_FaHo_SA_(	866	FTMS;	150.9	815.0	TY1B-ML1;TY1B-BR;TY1B-H;TY1B-MR2;TY1B-OR;TY1B-DR1;TY1B-NL2;TY1B-PR2;TY1B-DR5;TY1B-PR1;TY1B-JR2;TY1B-NL1;TY1B-OL;TY1B-LR4;TY1B-ML2;TY1B-DR

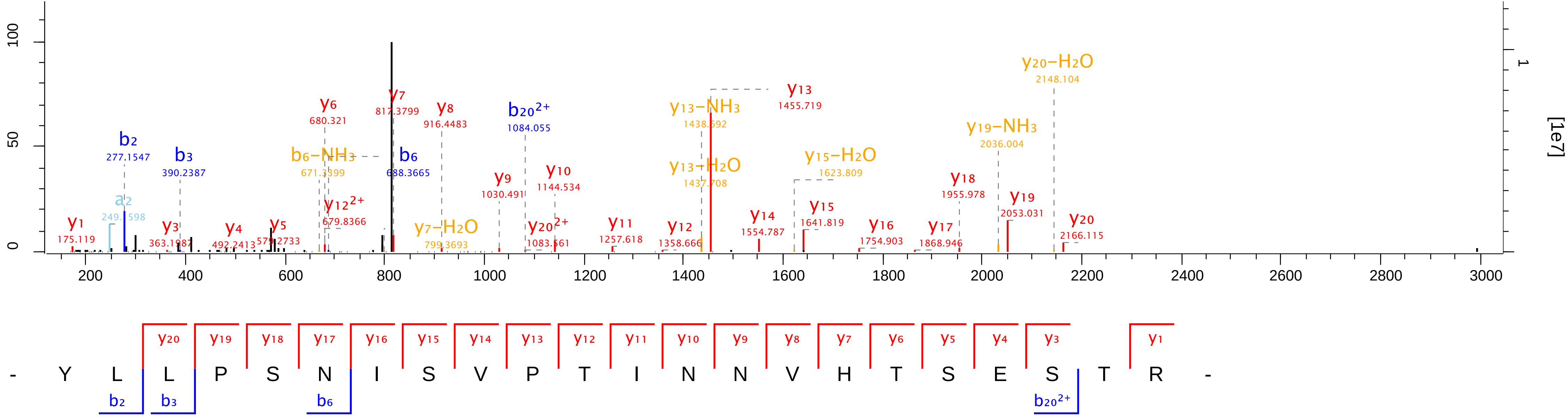

Raw file	Scan	Method	Score	m/z	Gene names
20140602_QEp4_FaHo_SA_GCN5_01	8932	FTMS; HCD	313.37	1008.01	RPS14B;RPS14A

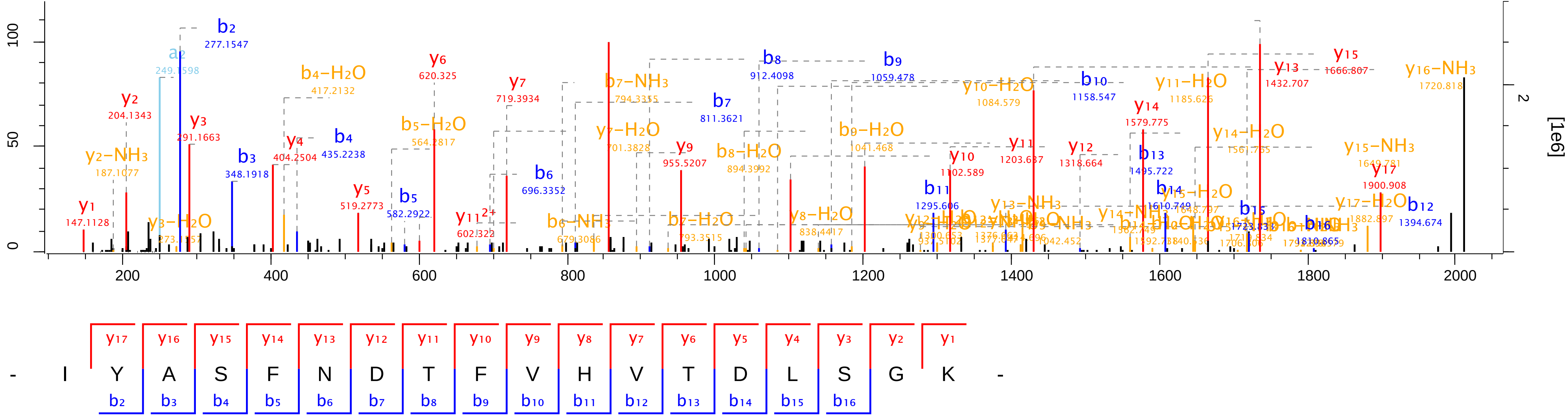

Raw file	Scan	Method	Score	m/z	Gene names
20140602_QEp4_FaHo_SA_GCN5_01	9012	FTMS; HCD	183.03	606.87	RPL14B;RPL14A

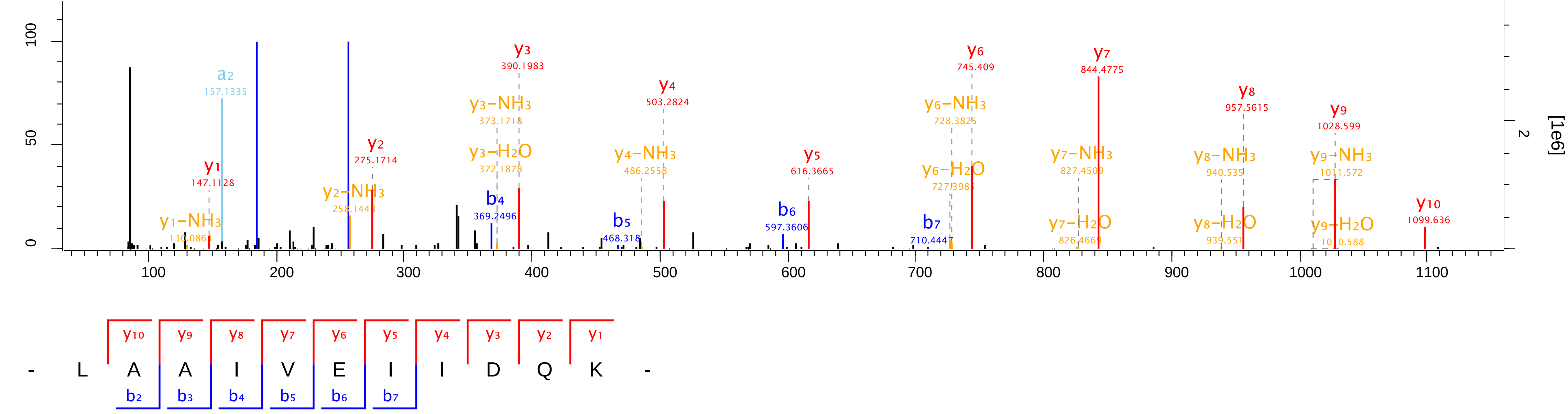

Raw file	Scan	Method	Score	m/z	Gene names
20140602_QEp4_FaHo	101(	FTMS{	320.	1130.	TY1B-ML1;TY1B-BR;TY1A-PR1;TY1A-A;TY1A-DR4;TY1B-H;TY1B-MR2;TY1B-OR;TY1B-DR1;TY1B-NL2;TY1B-PR2;TY1B-DR5;TY1B-PR1;TY1B-JR2;TY1A-PL;TY1A-LR2;TY1A-I

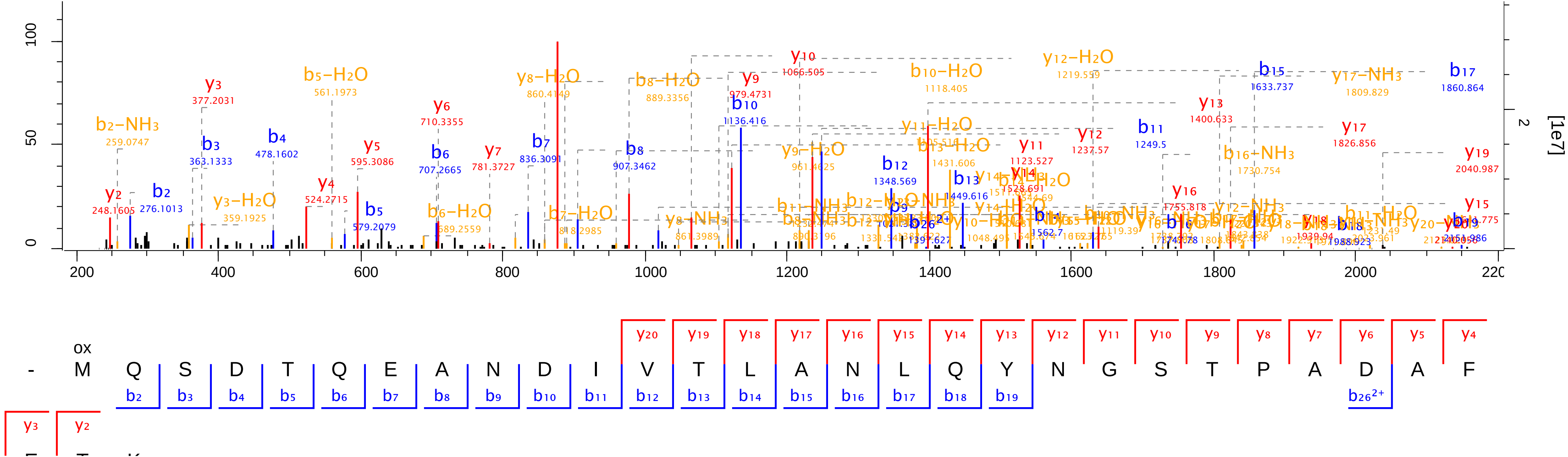

Raw file	Scan	Method	Score	m/z	Gene names
20140602_QEp4_FaHo_SA_GFP_01	10112	FTMS; HCD	127.64	985.51	RPL13B

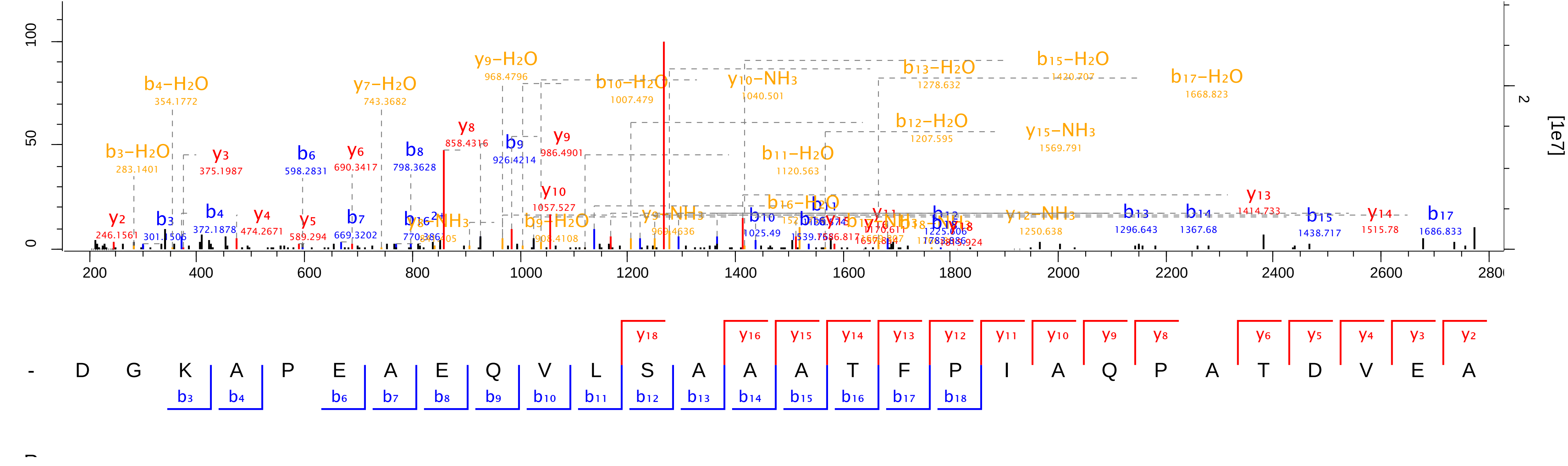

Raw file	Scan	Method	Score	m/z	Gene names
20140602_QEp4_FaHo_SA_GFP_02	3638	FTMS; HCD	157.99	482.76	RPL13B;RPL13A

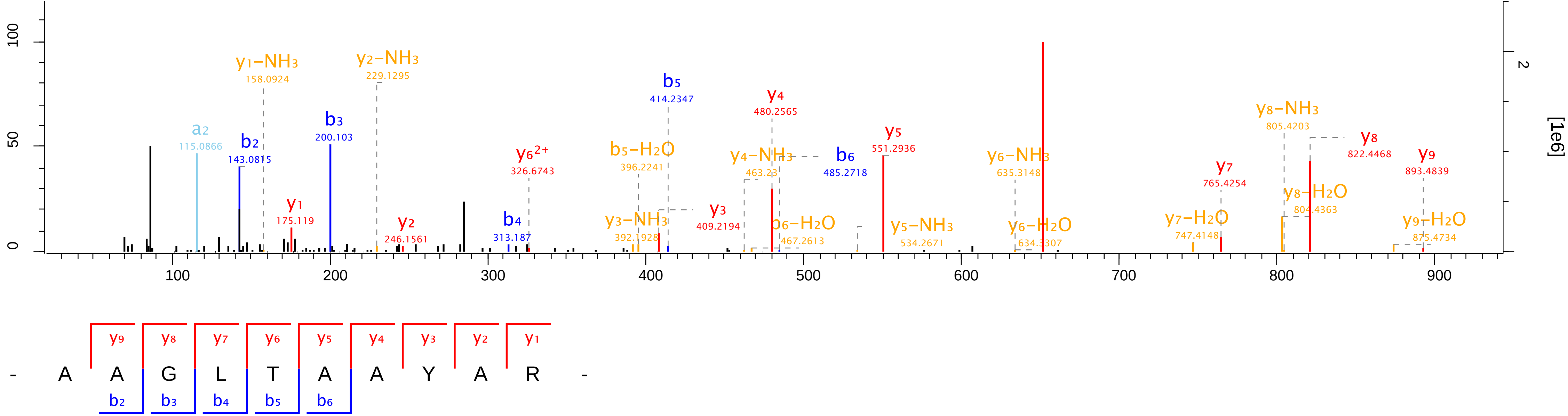

Raw file	Scan	Method	Score	m/z	Gene names
20140602_QEp4_FaHo_SA_GFP_02	4003	FTMS; HCD	197.36	708.35	RPS14B;RPS14A

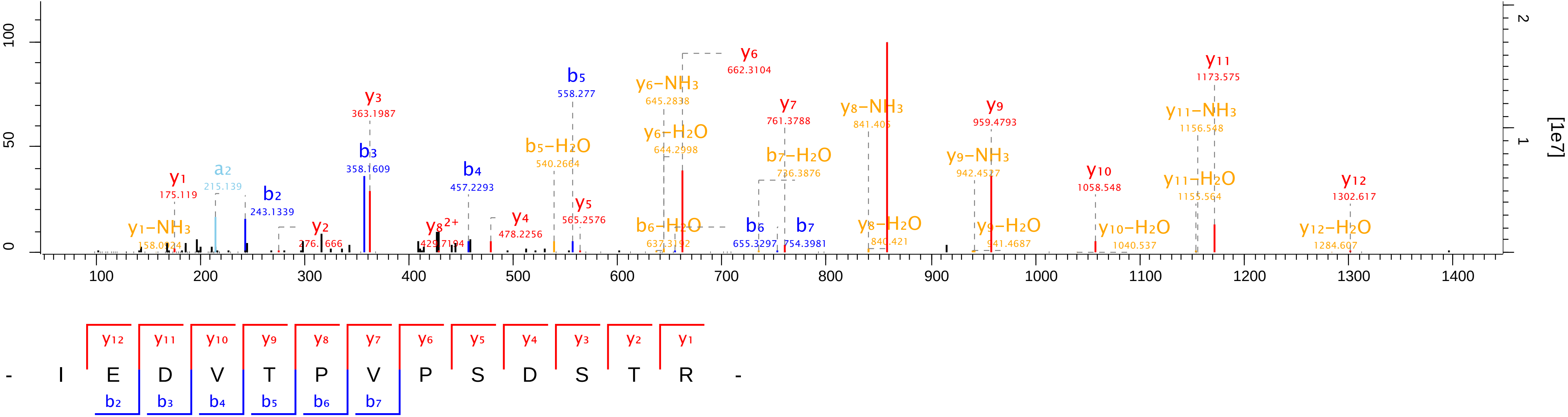

Raw file	Scan	Method	Score	m/z	Gene names
20140602_QEp4_FaHo_SA_HDA1	1475	FTMS;	74.84	464.75	TY2B-C;TY2B-B;TY2B-GR2;TY2B-F;TY2B-GR1;TY2A-GR1;TY2B-OR1;TY2A-DR2;TY2B-DR1;TY2B-DR3;TY2B-LR1;TY2B-OR2;TY2A-OR1;TY2A-LR2;TY2A-OR2;

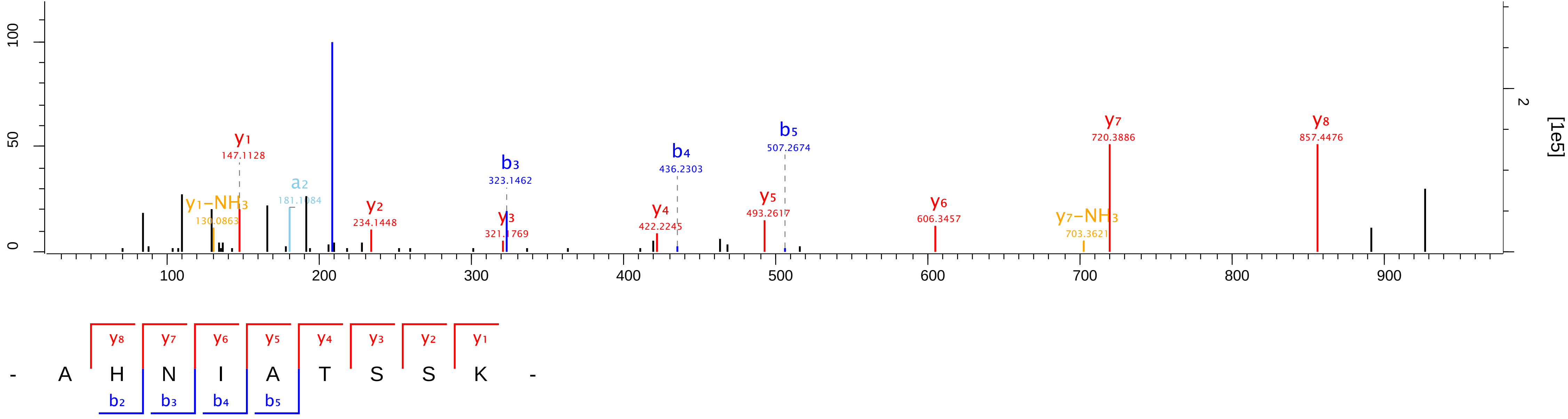

Raw file	Scan	Method	Score	m/z	Gene names
20140602_QEp4_FaHo_SA_HDA1_03	7657	FTMS; HCD	87.18	624.36	ATP5

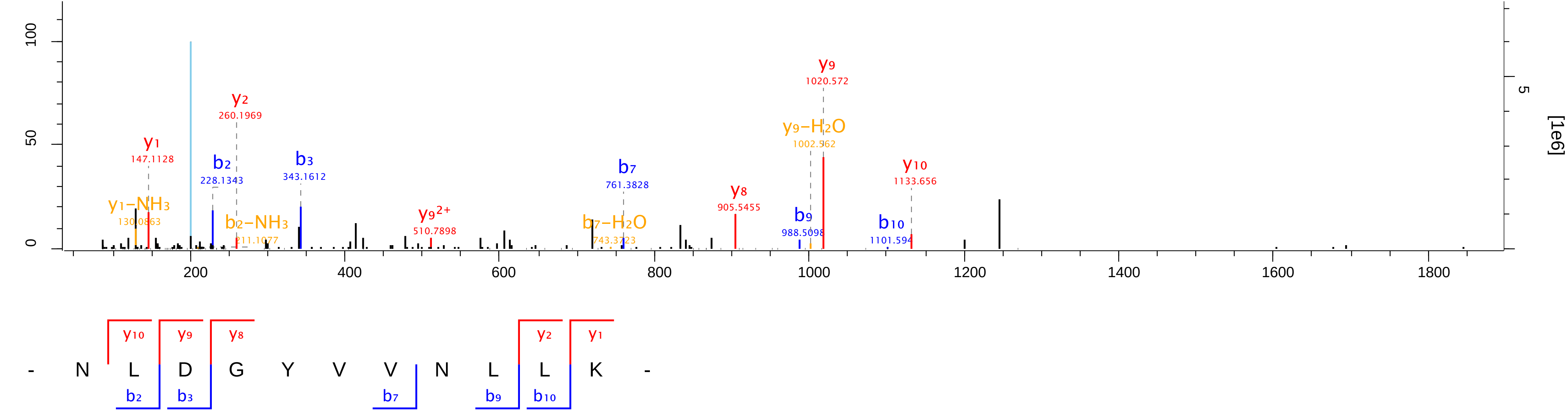

Raw file	Sca	Metho	Scor	m/z	Gene names
20140602_QEp4_Fal	9[illegible]	D	12[illegible]	55[illegible]3	TY1B-ML1;TY1B-BR;TY1B-H;TY1B-MR2;TY1B-OR;TY1B-DR1;TY1B-NL2;TY1B-PR2;TY1B-DR5;TY1B-PR1;TY1B-JR2;TY1B-NL1;TY2B-C;TY1B-OL;TY1B-LR4;TY1B-ML2;TY1B-DR3;TY1

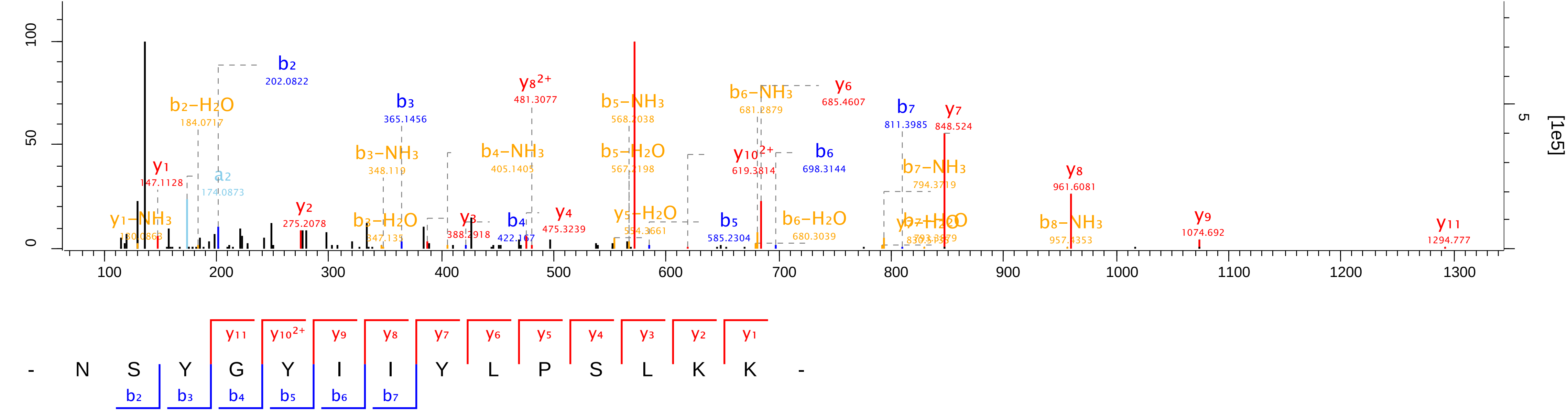

Raw file	Scan	Method	Score	m/z	Gene names
20140602_QEp4_FaHo_SA_HDA2_01	5949	FTMS; HCD	94.47	545.3	ARF1;ARF2

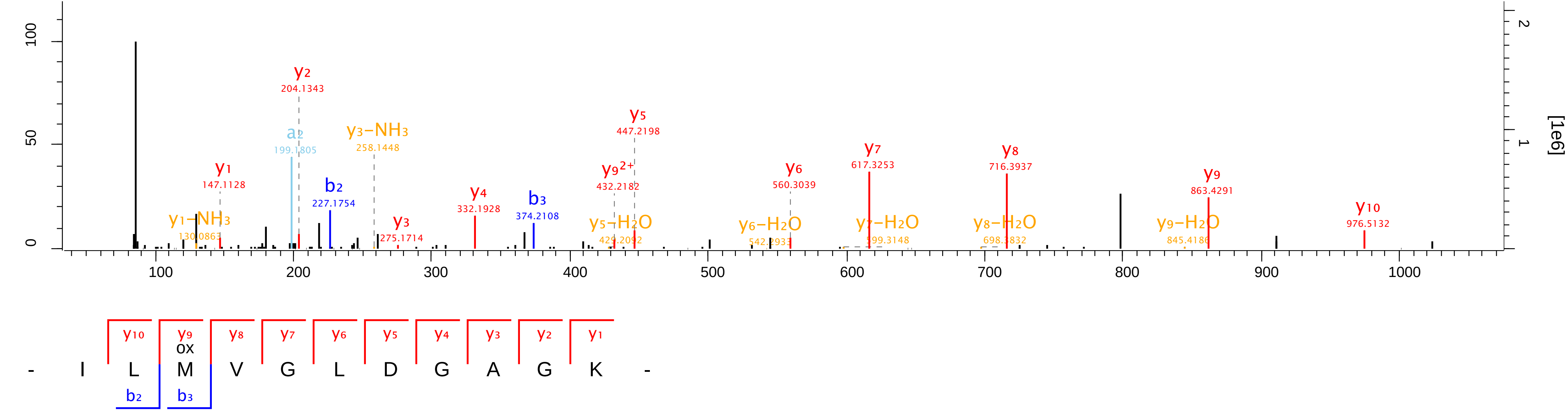

Raw file	Scan	Method	Score	m/z	Gene names
20140602_QEp4_FaHo_SA_HDA2_02	5927	FTMS; HCD	55.45	757.04	HXT6;HXT7

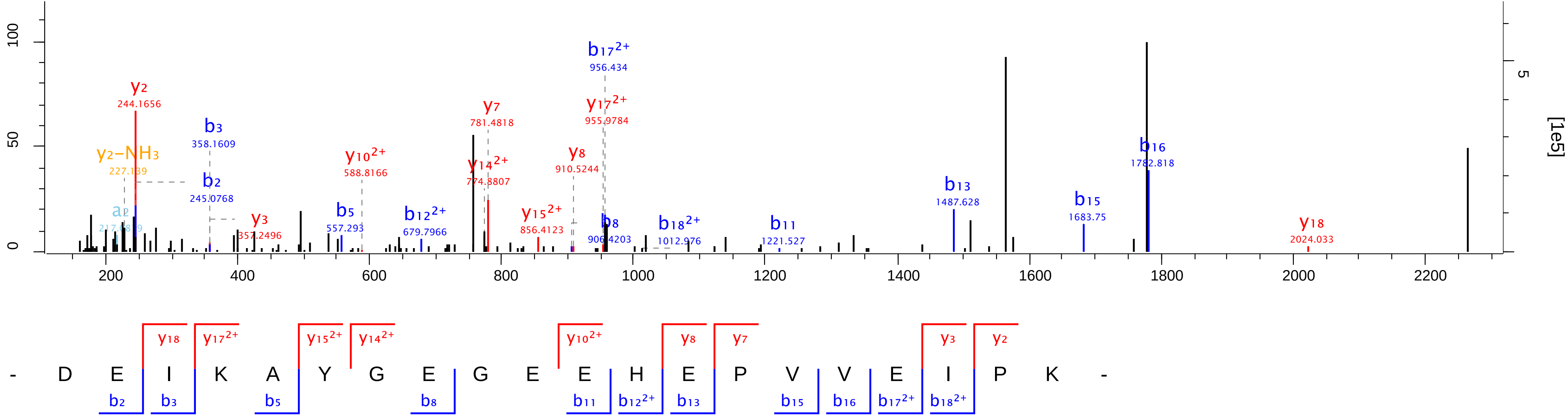

Raw file	Scan	Method	Score	m/z	Gene names
20140602_QEp4_FaHo_SA_HDA2_02	7570	FTMS; HCD	209.36	919.7	HXT6;HXT7

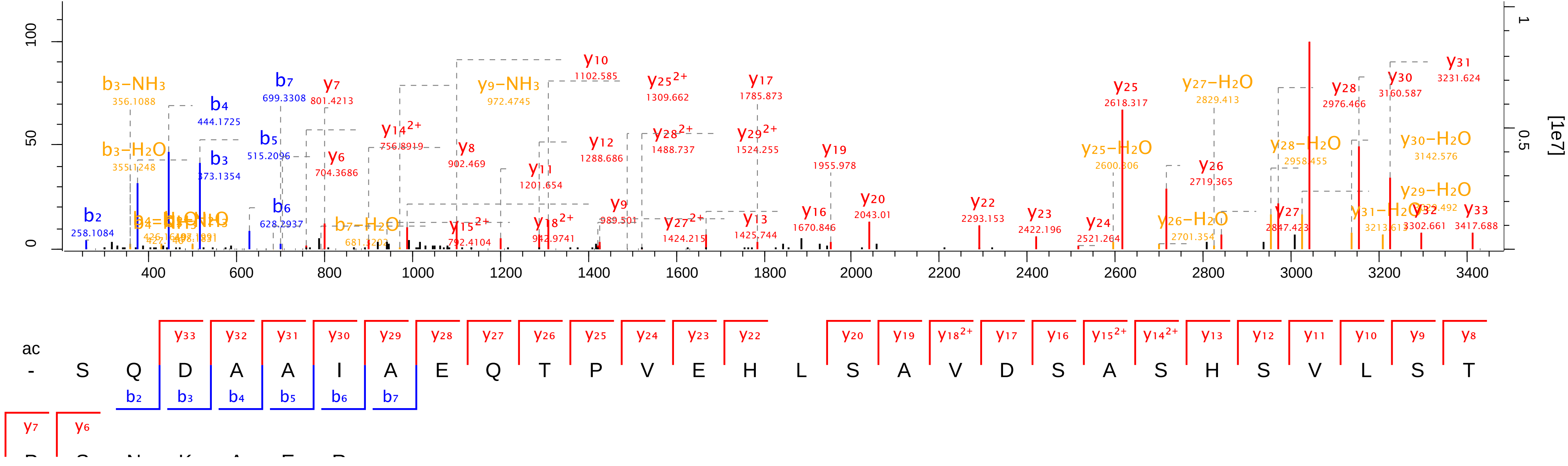

Raw file Sca Metho Scor m/z Gene names

20140602_QEp4_FaH 80S; FTMS; A 144 714 TY1B-ML1;TY1B-BR;TY1A-PR1;TY1A-A;TY1A-DR4;TY1B-H;TY1B-MR2;TY1B-OR;TY1B-DR1;TY1B-NL2;TY1B-PR2;TY1B-DR5;TY1B-PR1;TY1B-JR2;TY1A-PL;TY1A-LR2;TY1A-ER1;TY

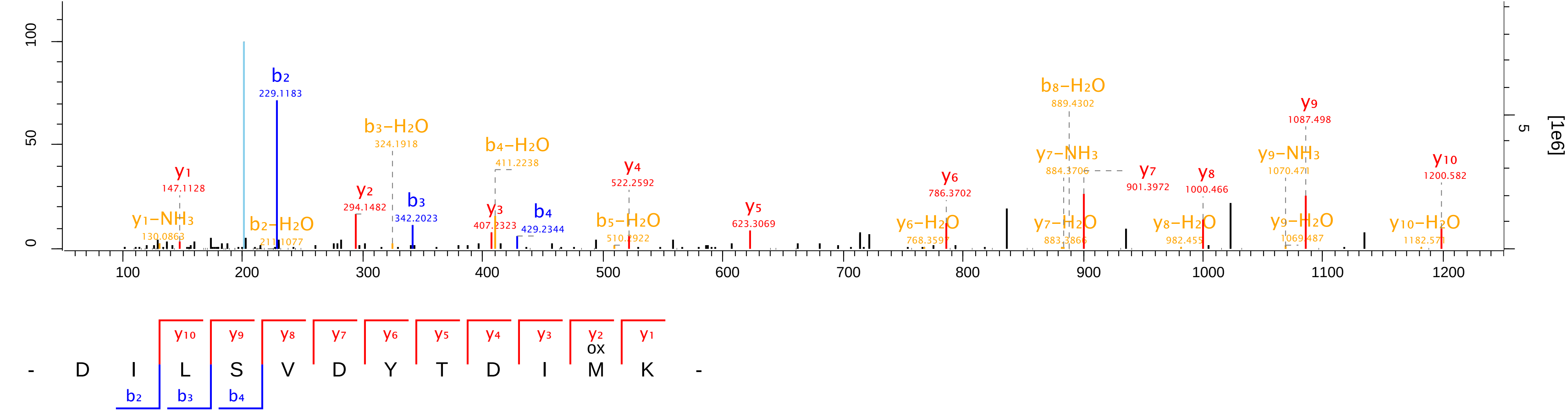

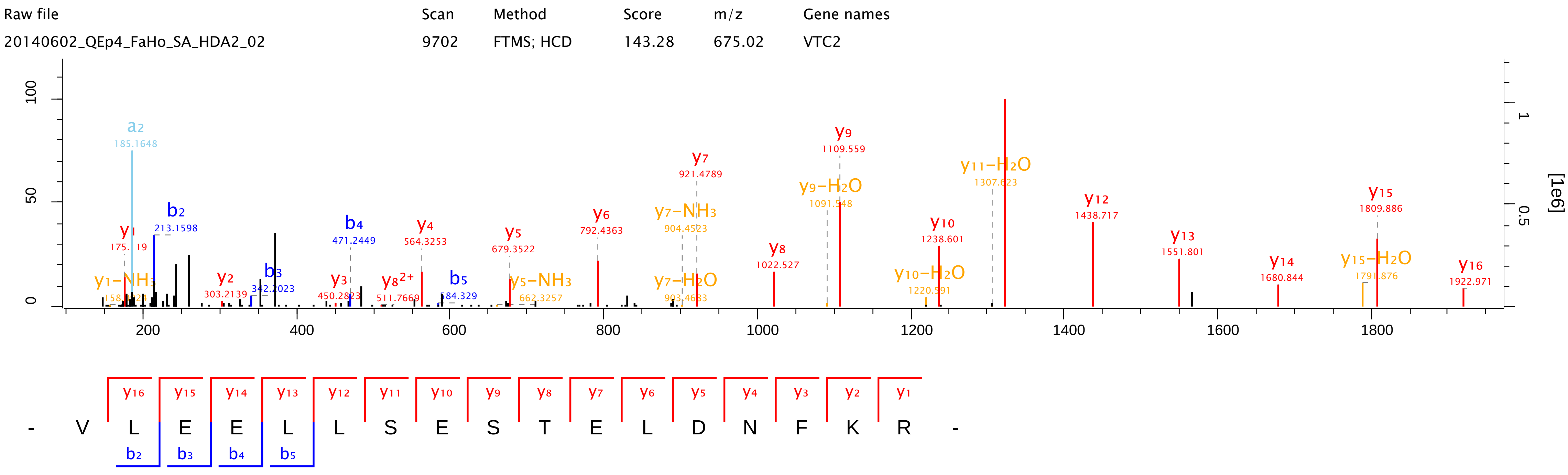
Raw file
20140602_QEp4_FaHo_SA_HDA2_02
Scan
9702
Method
FTMS; HCD
Score
143.28
m/z
675.02
Gene names
VTC2
- V L E E L L S E S T E L D N F K R -

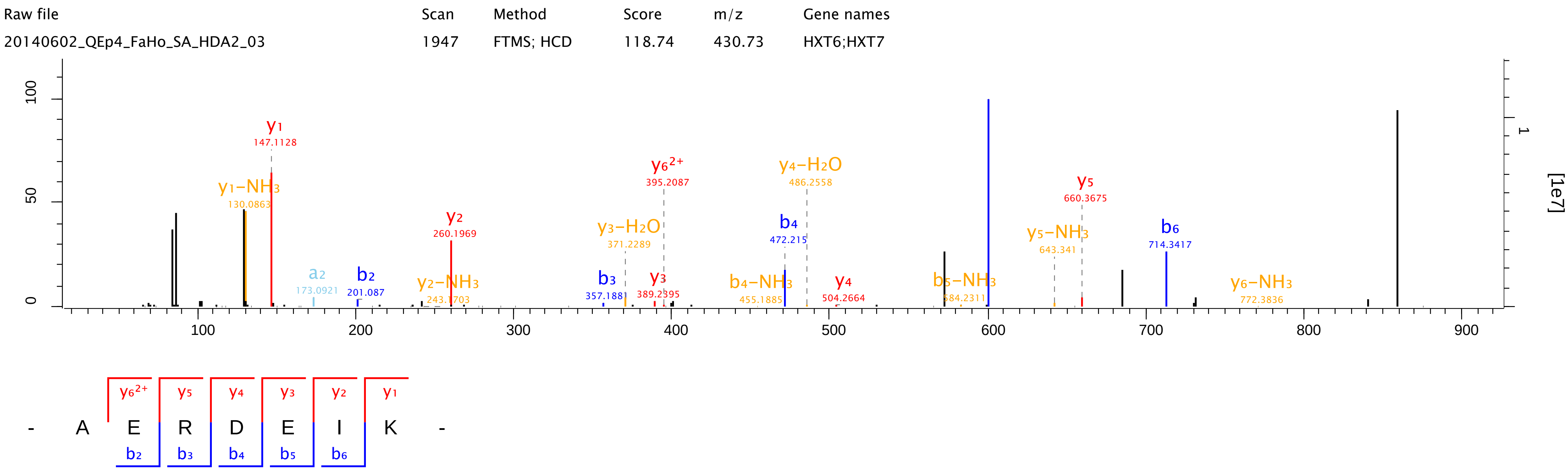

Raw file
20140602_QEp4_FaHo_SA_HDA2_03
Scan
1947
Method
FTMS; HCD
Score
118.74
m/z
430.73
Gene names
HXT6;HXT7
y1 147.1128
y1−NH3 130.0863
a2 173.0921
b2 201.087
y2 260.1969
y2−NH3 243.1703
b3 357.1881
y3−H2O 371.2289
y6 2+ 395.2087
y3 389.2395
b4−NH3 455.1885
b4 472.215
y4−H2O 486.2558
y4 504.2664
b5−NH3 584.2311
y5−NH3 643.341
y5 660.3675
b6 714.3417
y6−NH3 772.3836
100
50
0
100
200
300
400
500
600
700
800
900
1
[1e7]
- A E R D E I K -
y6 2+ y5 y4 y3 y2 y1
b2 b3 b4 b5 b6

Raw file	Scan	Method	Score	m/z	Gene names
20140602_QEp4_FaHo_SA_HDA2_03	2338	FTMS; HCD	111.65	359.71	HXT6;HXT7

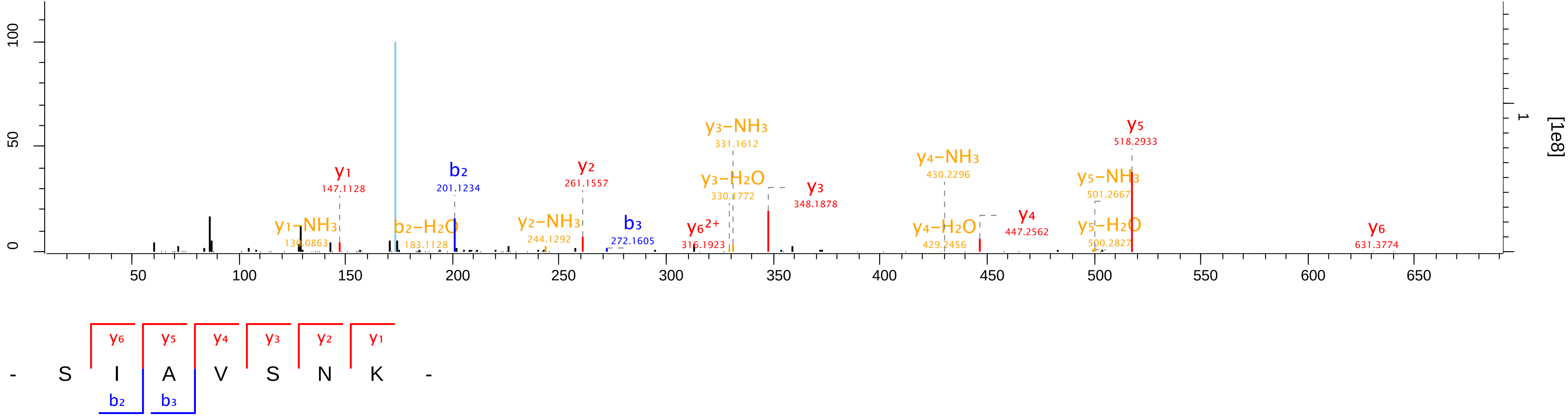

Raw file	Scan	Method	Score	m/z	Gene names
20140602_QEp4_FaHo_SA_HDA2_03	2819	FTMS; HCD	84.23	411.23	RPL9A;RPL9B

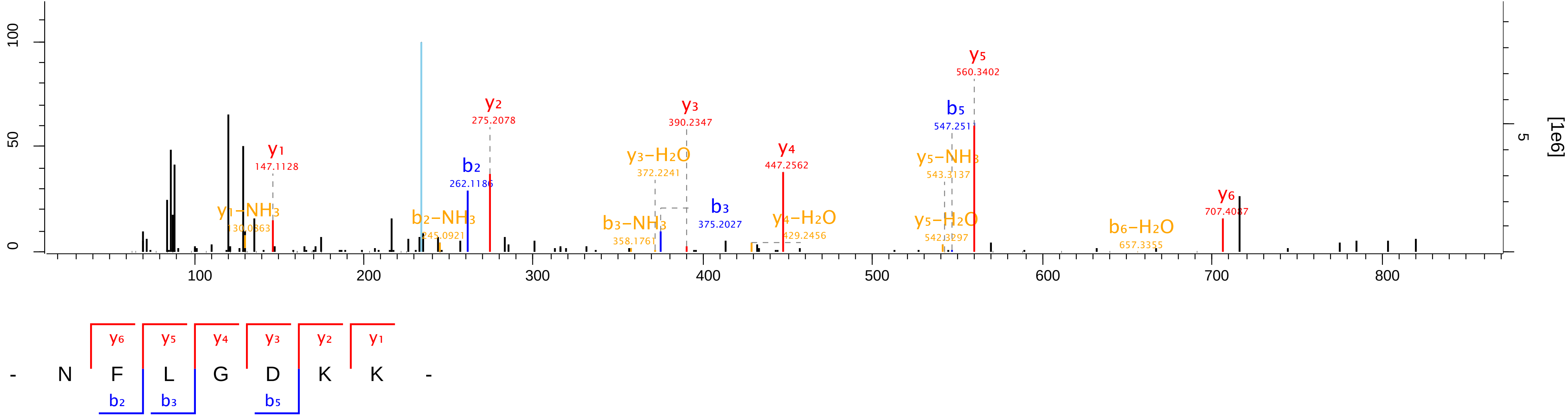

Raw file	Scan	Method	Score	m/z	Gene names
20140602_QEp4_FaHo_SA_HDA2_03	3197	FTMS; HCD	195.79	492.28	CMD1

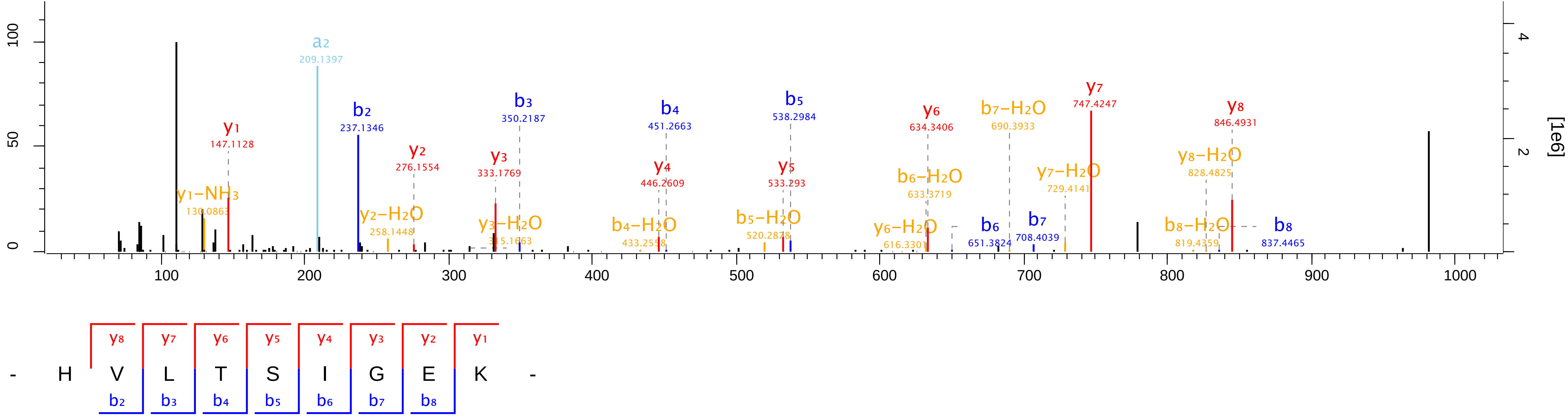

Raw file	Scan	Method	Score	m/z	Gene names
20140602_QEp4_FaHo_SA_HDA2_03	3305	FTMS; HCD	125.47	449.22	HXT6;HXT7

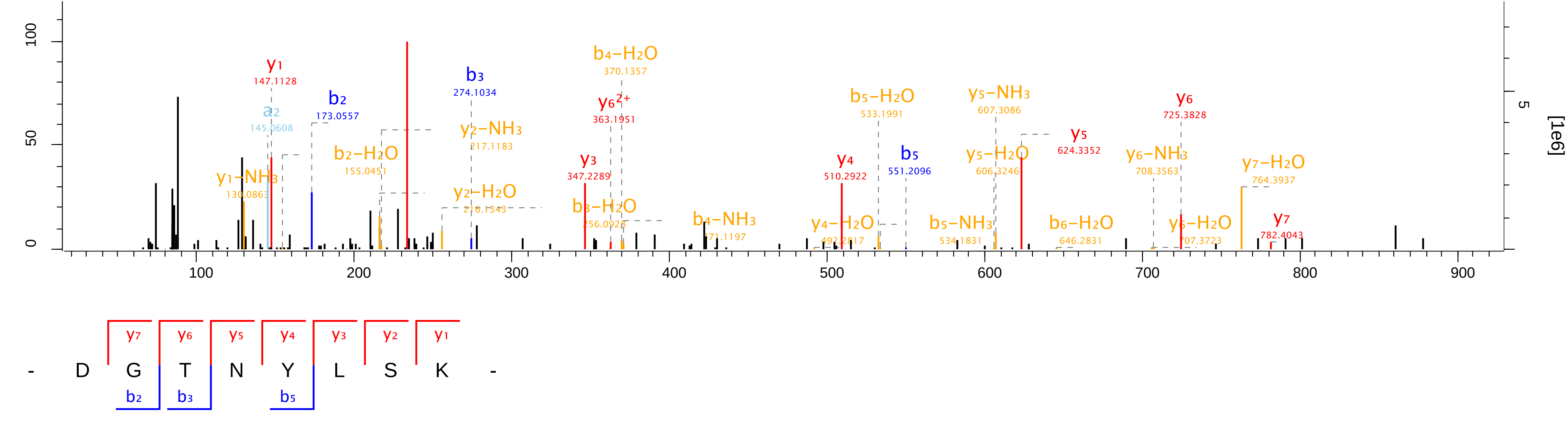

Raw file	Scan	Method	Score	m/z	Gene names
20140602_QEp4_FaHo_SA_HDA2_03	3410	FTMS; HCD	85.36	390.22	HXT6;HXT7

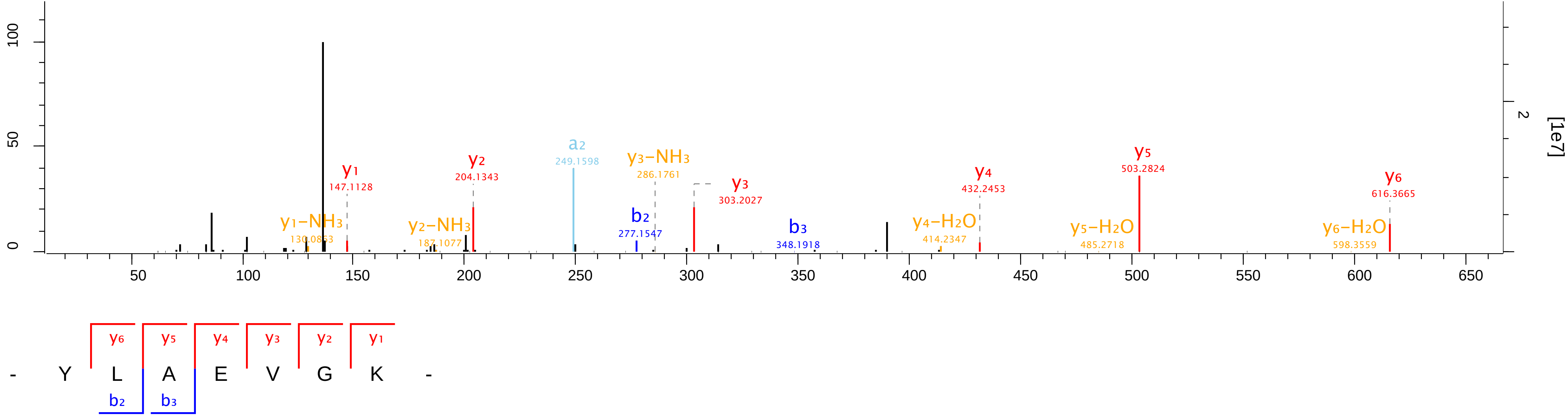

Raw file	Scan	Method	Score	m/z	Gene names
20140602_QEp4_FaHo_SA_HDA2_03	5308	FTMS; HCD	88.56	594.96	HXT6;HXT7

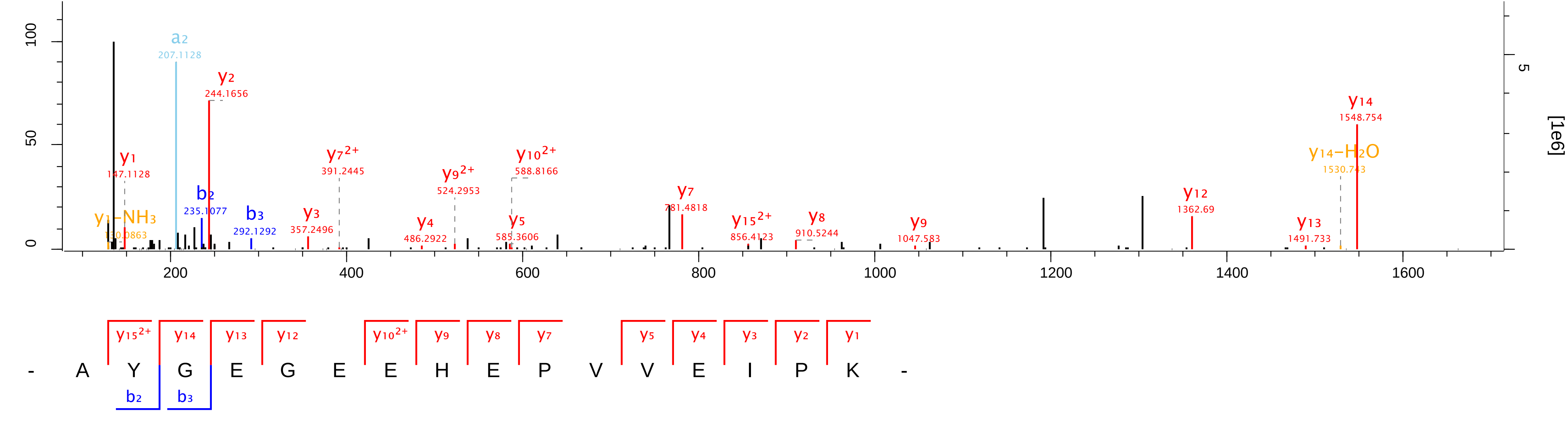

Raw file	Scan	Method	Score	m/z	Gene names
20140602_QEp4_FaHo_SA_HDA2_03	5821	FTMS; HCD	72.36	702	SHM1

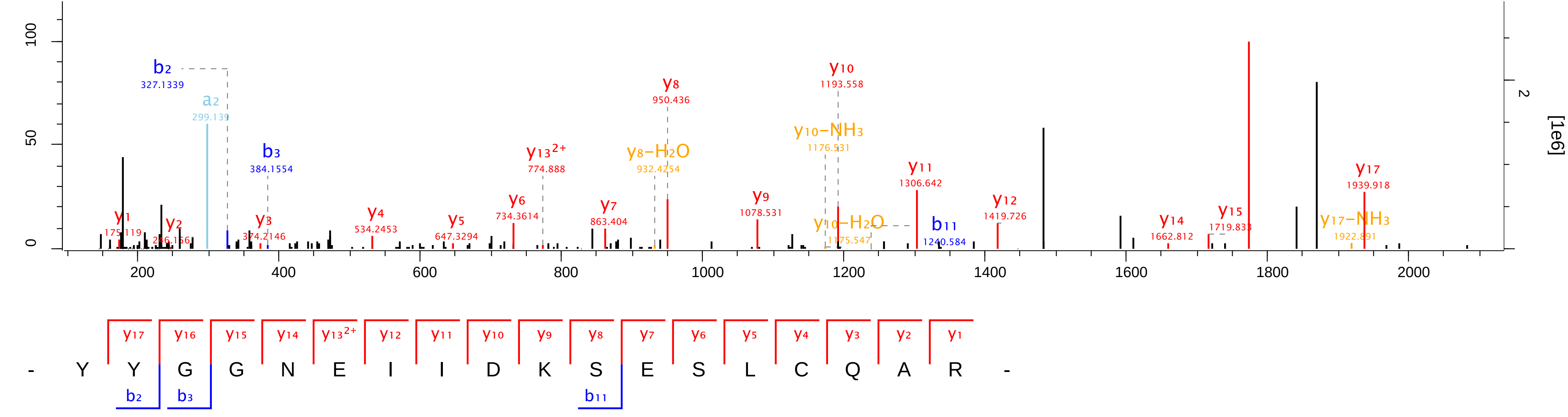

Raw file	Scan	Method	Score	m/z	Gene names
20140602_QEp4_FaHo_SA_HDA2_03	5869	FTMS; HCD	217.19	545.96	PST2

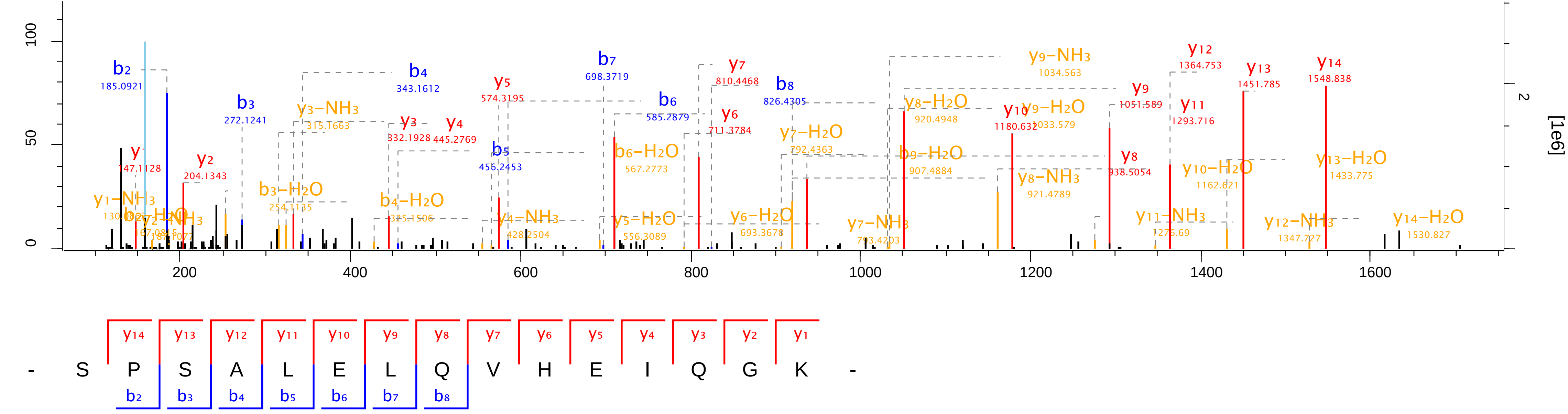

Raw file	Scan	Method	Score	m/z	Gene names
20140602_QEp4_FaHo_SA_HDA2_03	7364	FTMS; HCD	152.85	833.22	HXT6;HXT7

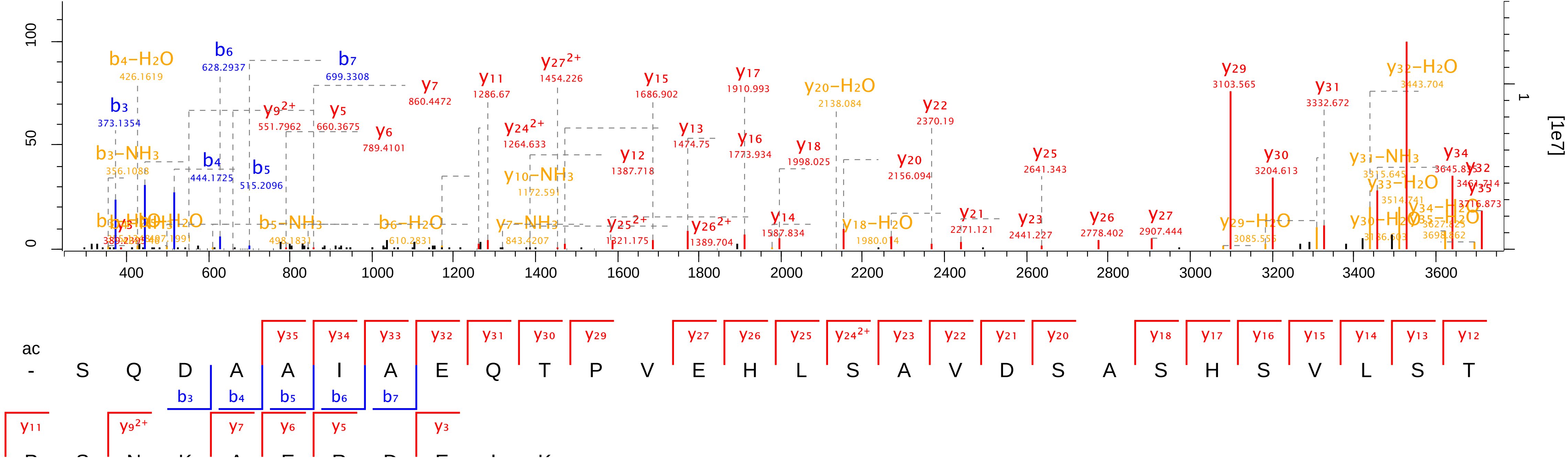

Raw file	Scan	Method	Score	m/z	Gene names
20140602_QEp4_FaHo_SA_HDA2_03	8140	FTMS; HCD	281.13	1107.21	HXT6;HXT7

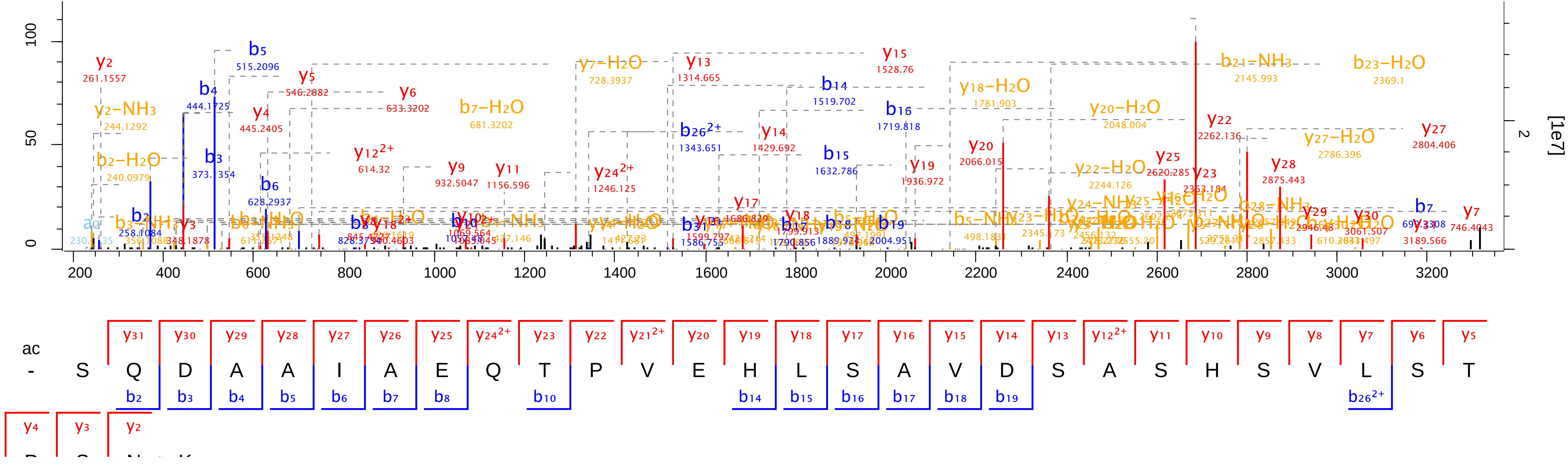

Raw file	Scan	Method	Score	m/z	Gene names
20140602_QEp4_FaHo_SA_HDA2_03	8909	FTMS; HCD	206.66	733.87	HXT6;HXT7

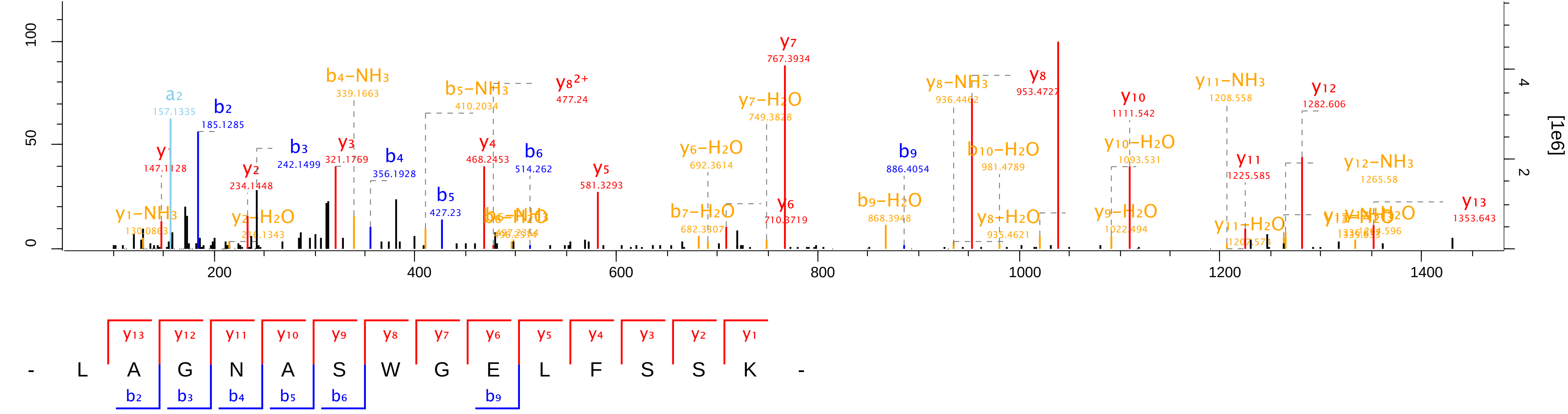

Raw file	Scan	Method	Score	m/z	Gene names
20140602_QEp4_FaHo_SA_HDA2_03	10020	FTMS; HCD	129.05	882.96	RPS0B;RPS0A

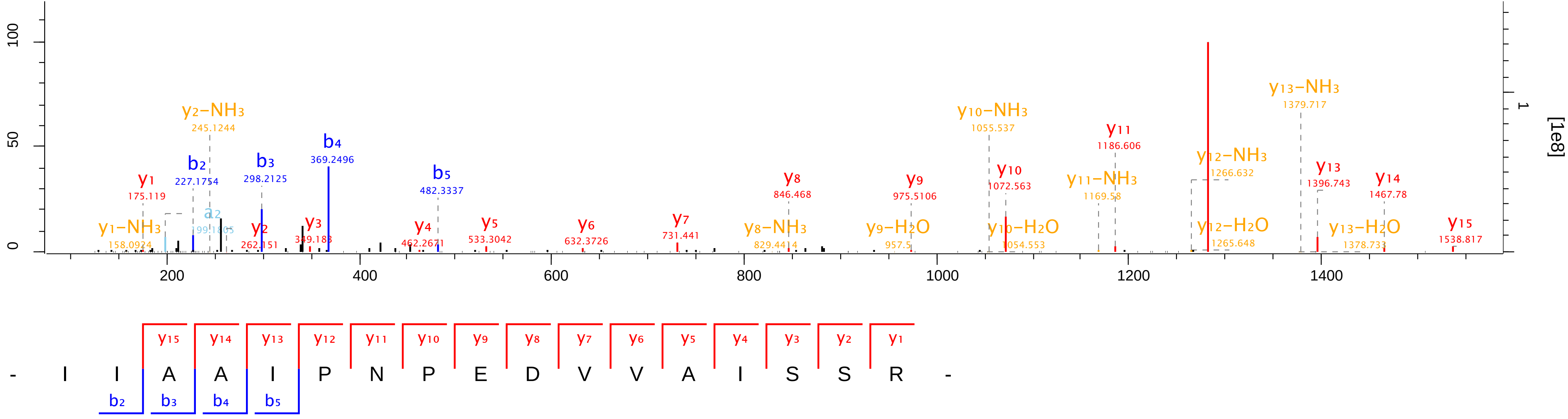

Raw file	Scan	Method	Score	m/z	Gene names
20140602_QEp4_FaHo_SA_HDA2_03	10198	FTMS; HCD	215.32	1156.11	HXT6;HXT7

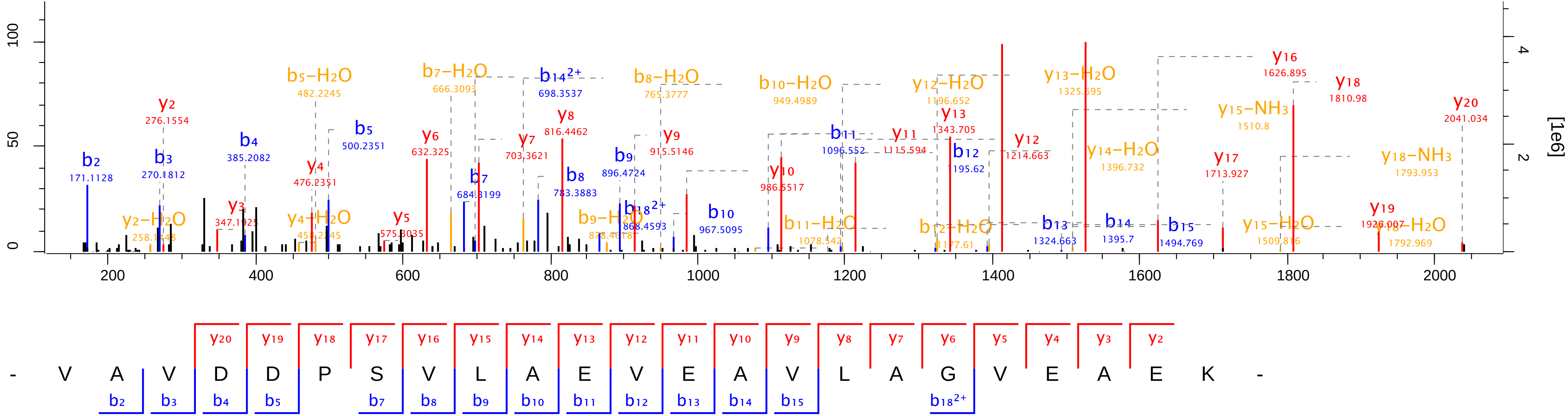

Raw file	Scan	Method	Score	m/z	Gene names
20140602_QEp4_FaHo_SA_HDA3_01	5095	FTMS; HCD	106.1	846.07	HSP30

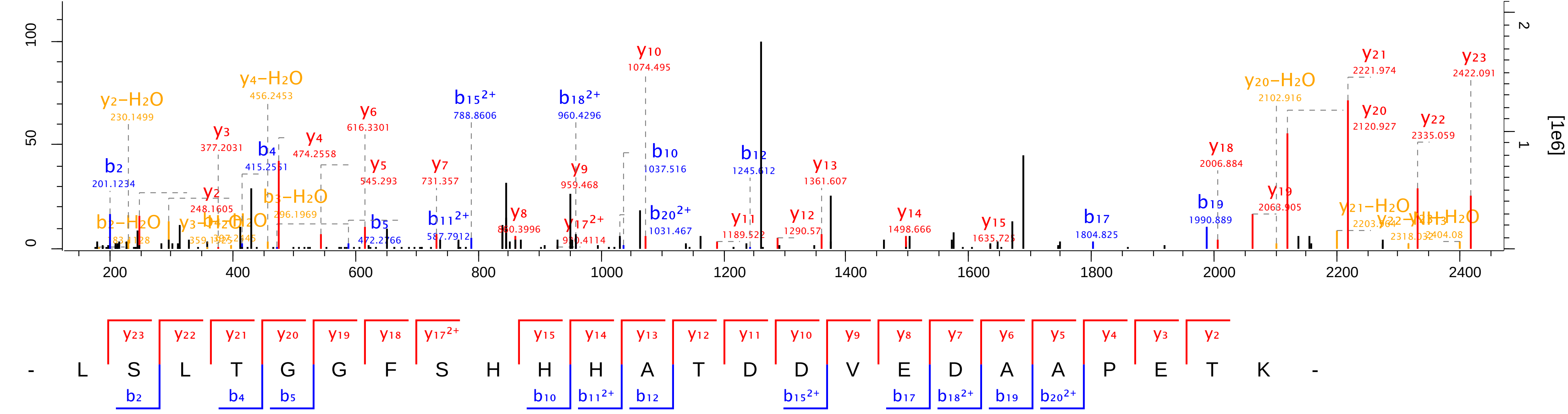

Raw file	Scan	Method	Score	m/z	Gene names
20140602_QEp4_FaHo_SA_HDA3_01	5269	FTMS; HCD	70.68	434.57	GPH1

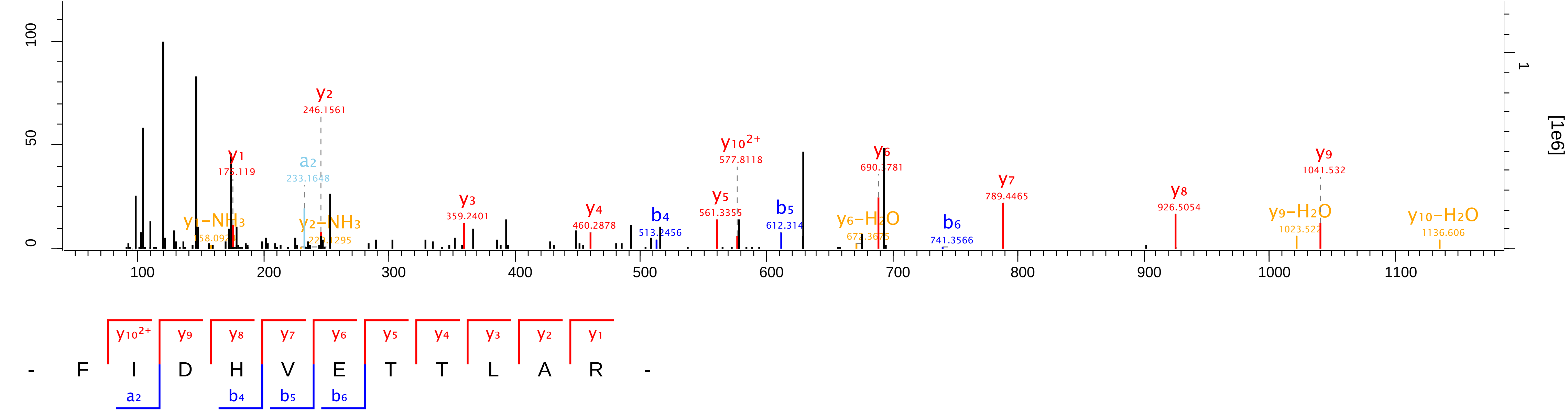

Raw file	Scan	Method	Score	m/z	Gene names
20140602_QEp4_FaHo_SA_HDA3_02	4577	FTMS; HCD	133.86	689.98	HXT6

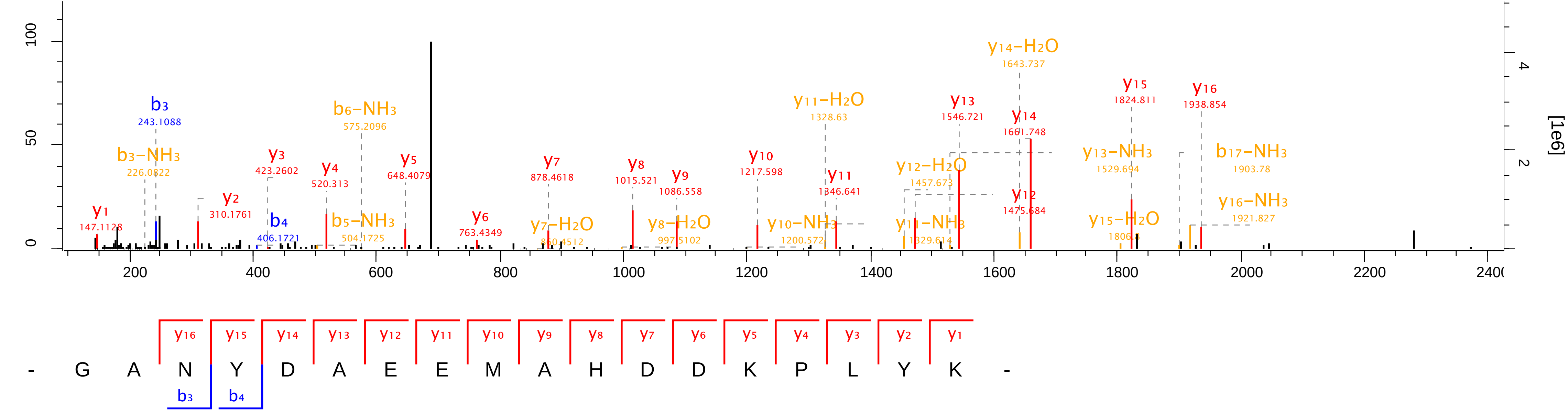

Raw file	Scan	Method	Score	m/z	Gene names
20140602_QEp4_FaHo_SA_HDA3_02	5020	FTMS; HCD	142.12	675.87	HXT6;HXT7

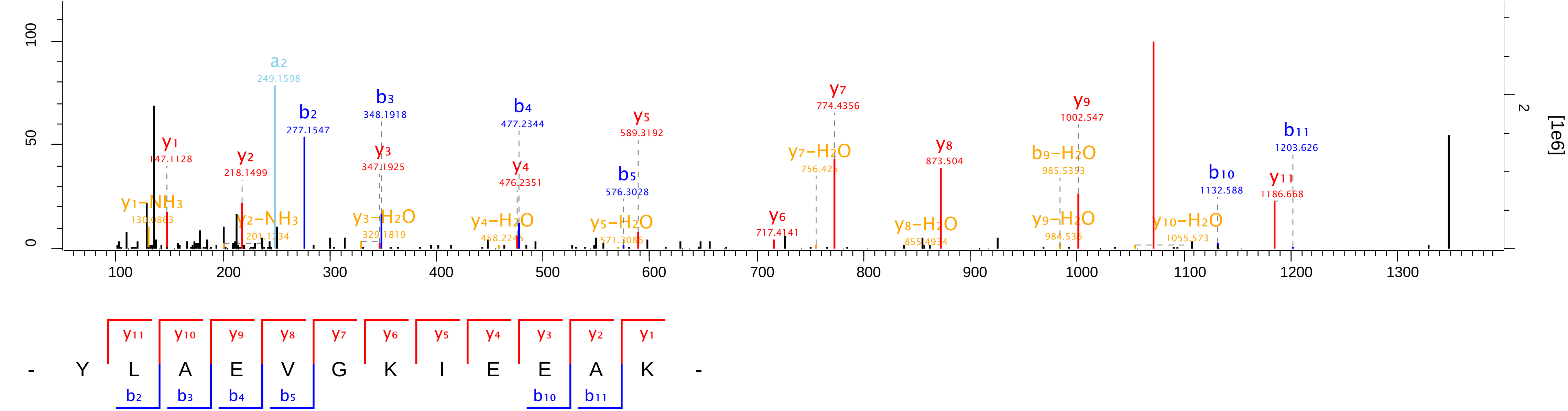

Raw file	Scan	Method	Score	m/z	Gene names
20140602_QEp4_FaHo_SA_HDA3_02	9252	FTMS; HCD	50.3	634.31	HUA1

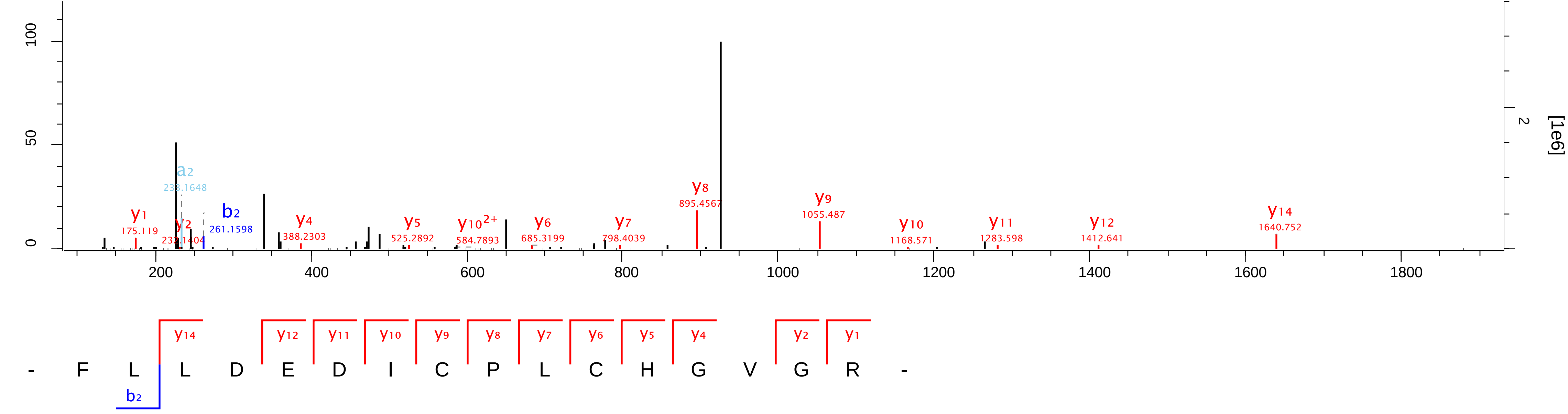

Raw file	Scan	Method	Score	m/z	Gene names
20140602_QEp4_FaHo_SA_HDA3_03	1935	FTMS; HCD	135.6	366.7	RPS0A

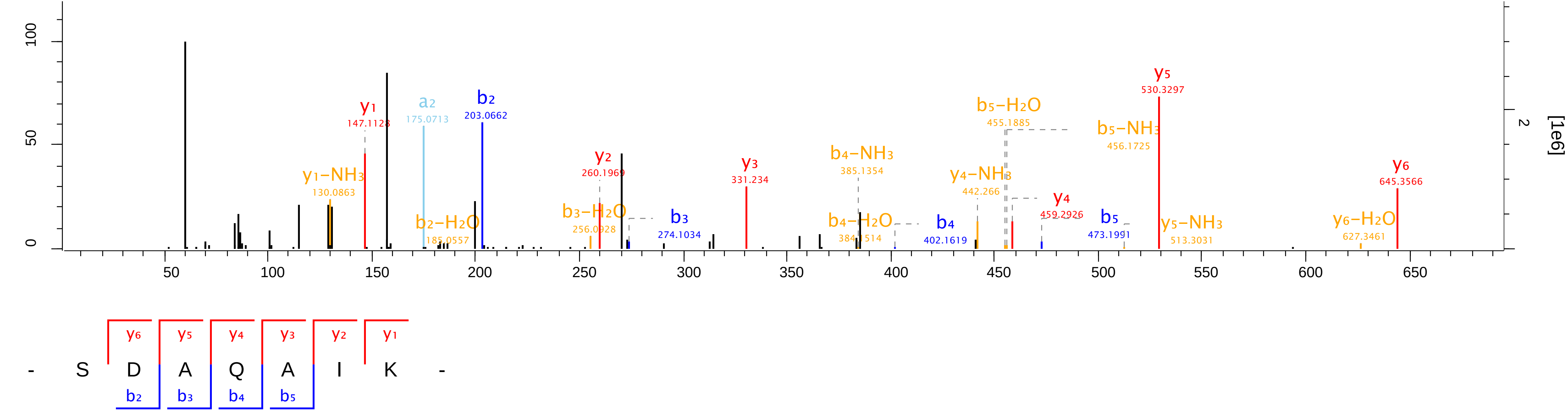

Raw file	Scan	Method	Score	m/z	Gene names
20140602_QEp4_FaHo_SA_HDA3_03	2243	FTMS; HCD	144.57	581.8	HXT6;HXT7

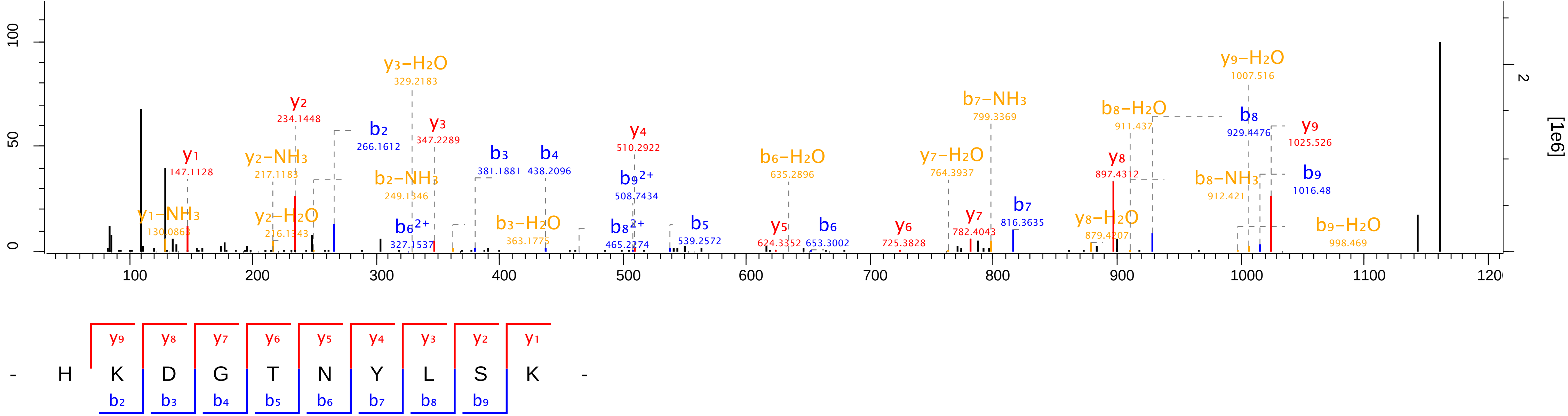

Raw file	Scan	Method	Score	m/z	Gene names
20140602_QEp4_FaHo_SA_HDA3_03	5116	FTMS; HCD	74.99	484.26	EMI2

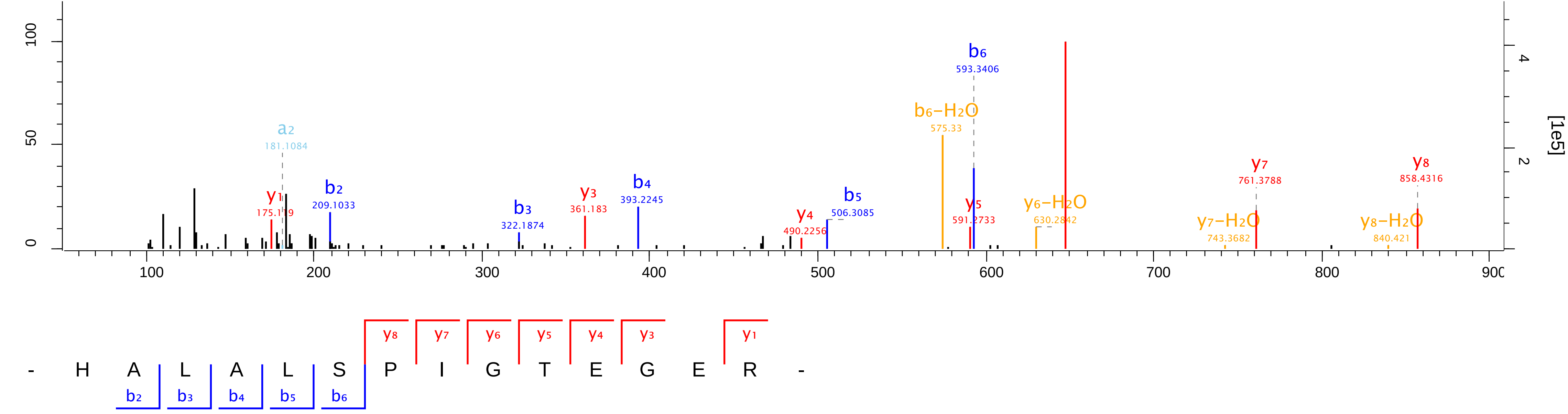

Raw file	Scan	Method	Score	m/z	Gene names
20140602_QEp4_FaHo_SA_HDA3_03	6959	FTMS; HCD	85.94	749.71	GAS3

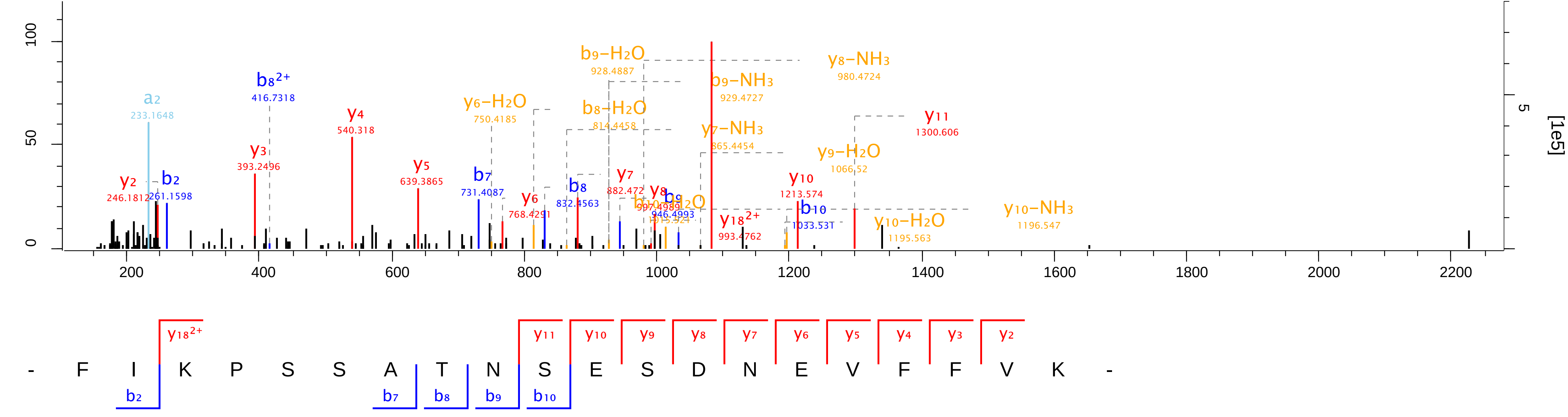

Raw file	Scan	Method	Score	m/z	Gene names
20140602_QEp4_FaHo_SA_HDA3_03	6990	FTMS; HCD	73.8	603.32	YIL108W

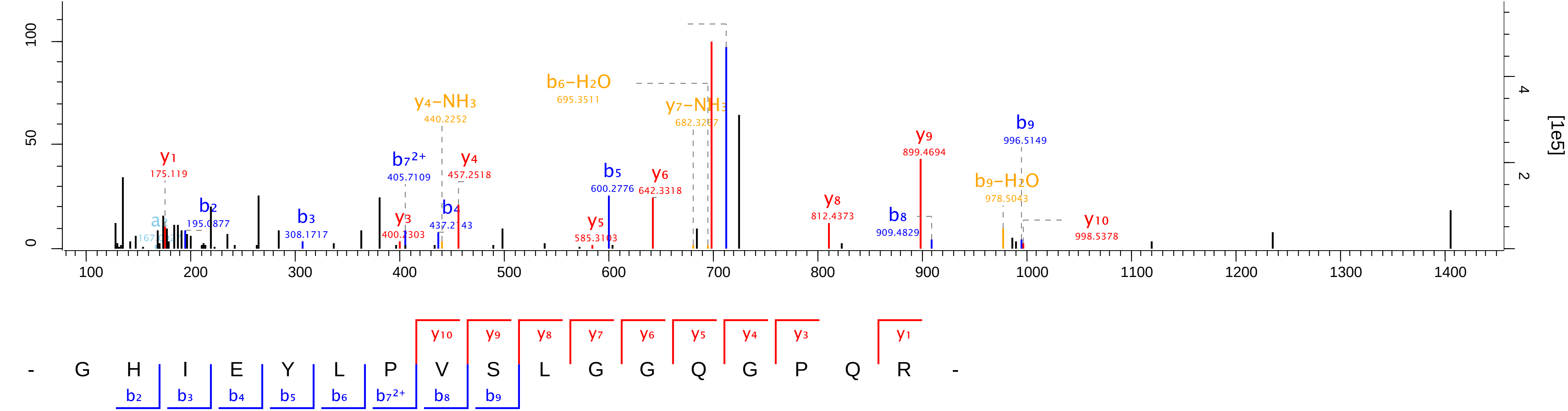

Raw file	Scan	Method	Score	m/z	Gene names
20140602_QEp4_FaHo_SA_HDA3_03	9521	FTMS; HCD	87.18	583.33	ERV46

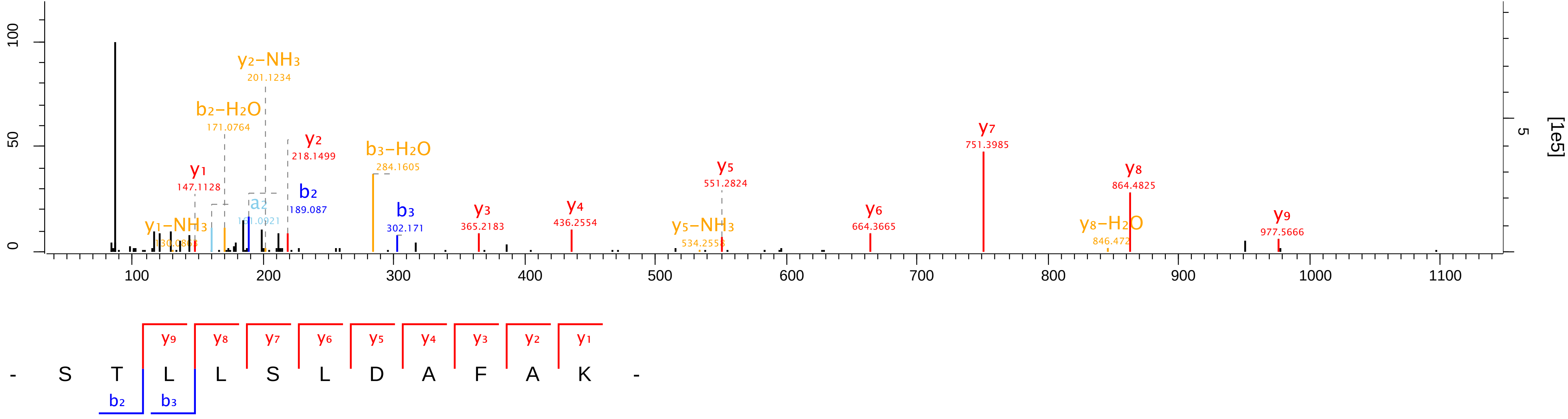

Raw file	Scan	Method	Score	m/z	Gene names
20140602_QEp4_FaHo_SA_HDA3_03	9694	FTMS; HCD	90.46	791.75	HEM15

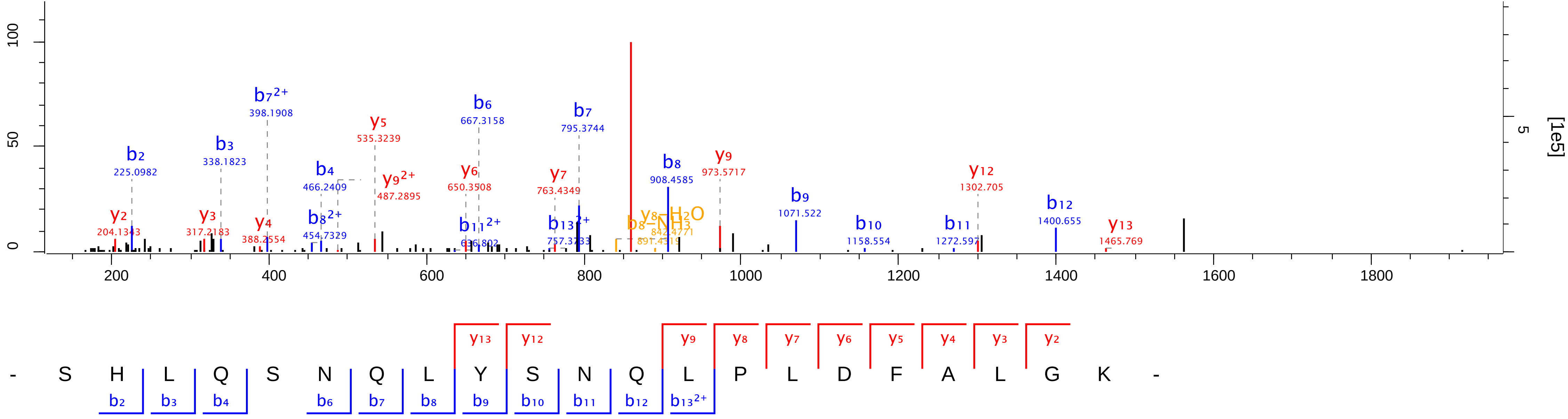

Raw file	Scan	Method	Score	m/z	Gene names
20140602_QEp4_FaHo_SA_HIF1_01	3543	FTMS; HCD	118.03	578.85	RPL7A;RPL7B

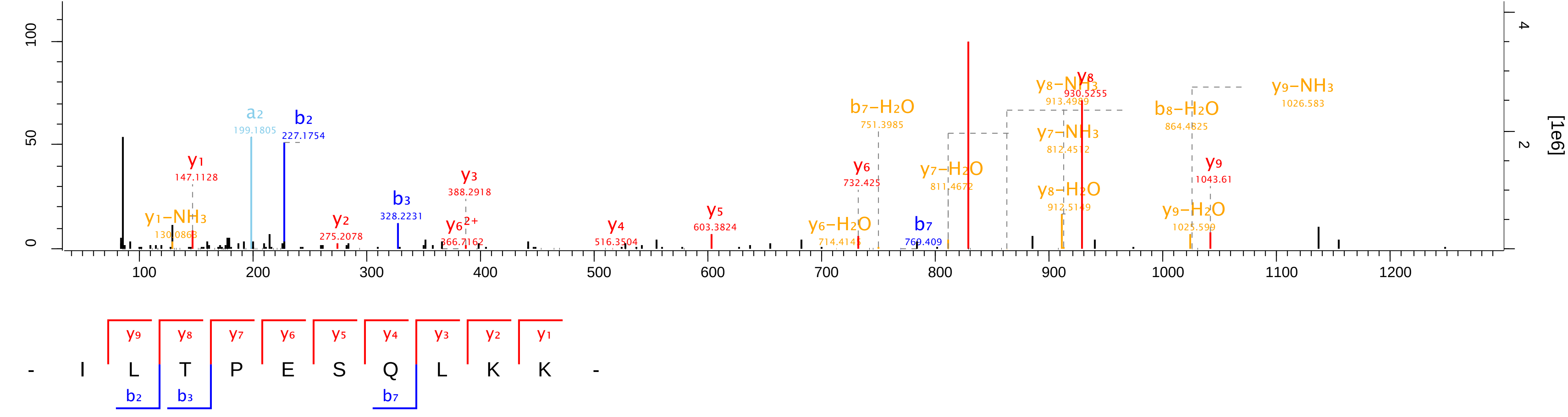

Raw file	Scan	Method	Score	m/z	Gene names
20140602_QEp4_FaHo_SA_HIF1_01	8440	FTMS; HCD	220.85	789.89	ARF2

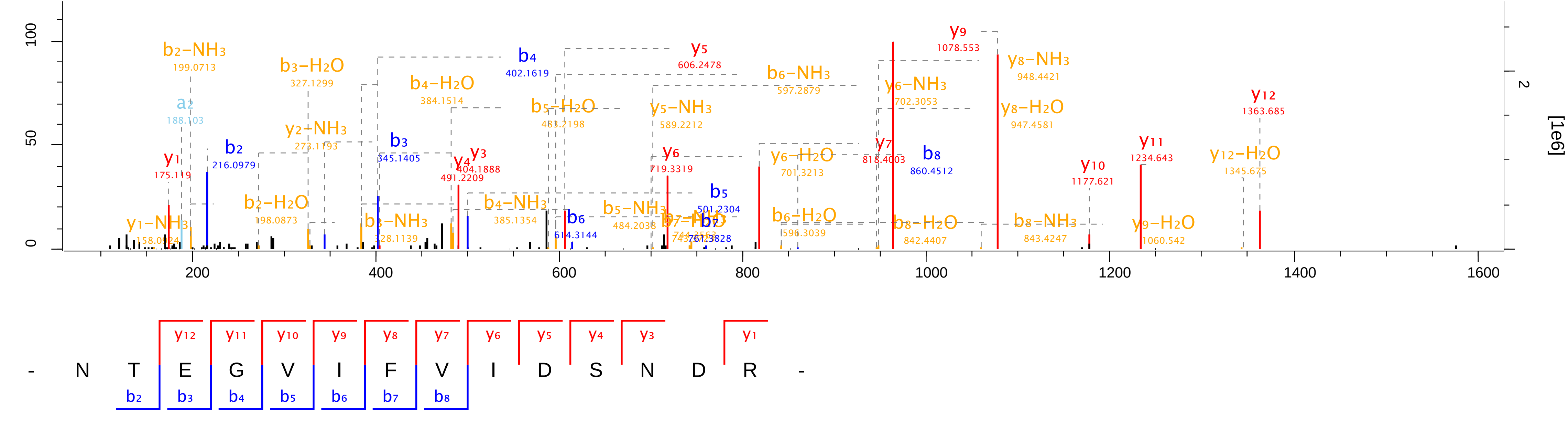

Raw file	Scan	Method	Score	m/z	Gene names
20140602_QEp4_FaHo_SA_HIF1_02	5313	FTMS; HCD	164.79	885.73	TY1B-ML1

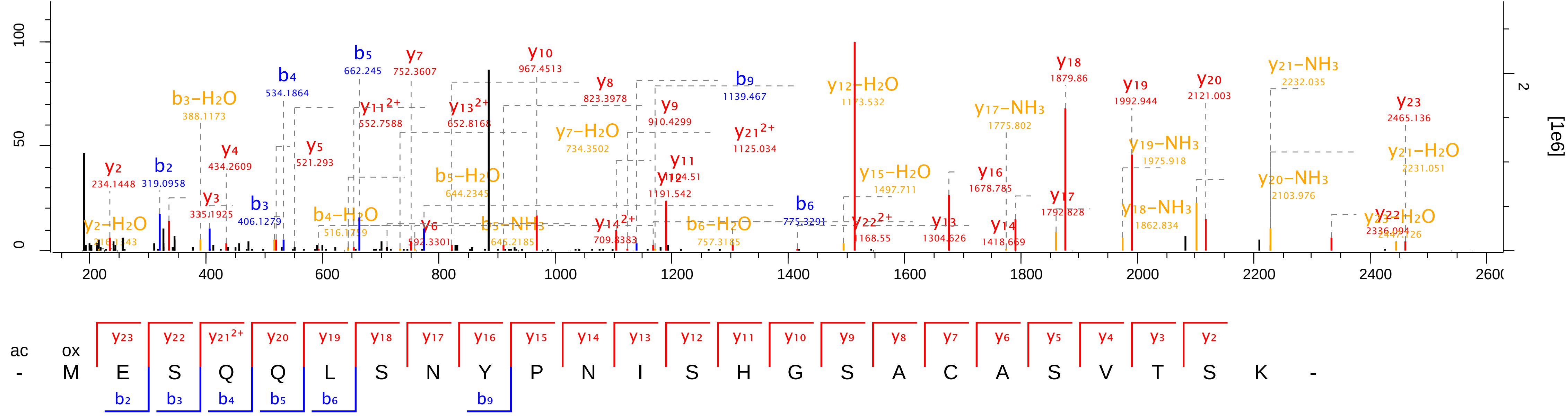

Raw file	Scan	Method	Score	m/z	Gene names
20140602_QEp4_FaHo_SA_HIF1_	5380	FTMS;	44.64	735.37	TY1B-ML1;TY1B-MR2;TY1B-OR;TY1B-DR1;TY1B-PR2;TY1B-DR5;TY1B-PR1;TY1B-JR2;TY1B-OL;TY1B-LR4;TY1B-ML2;TY1B-DR3;TY1B-PR3;TY1B-PL;TY1B-LI

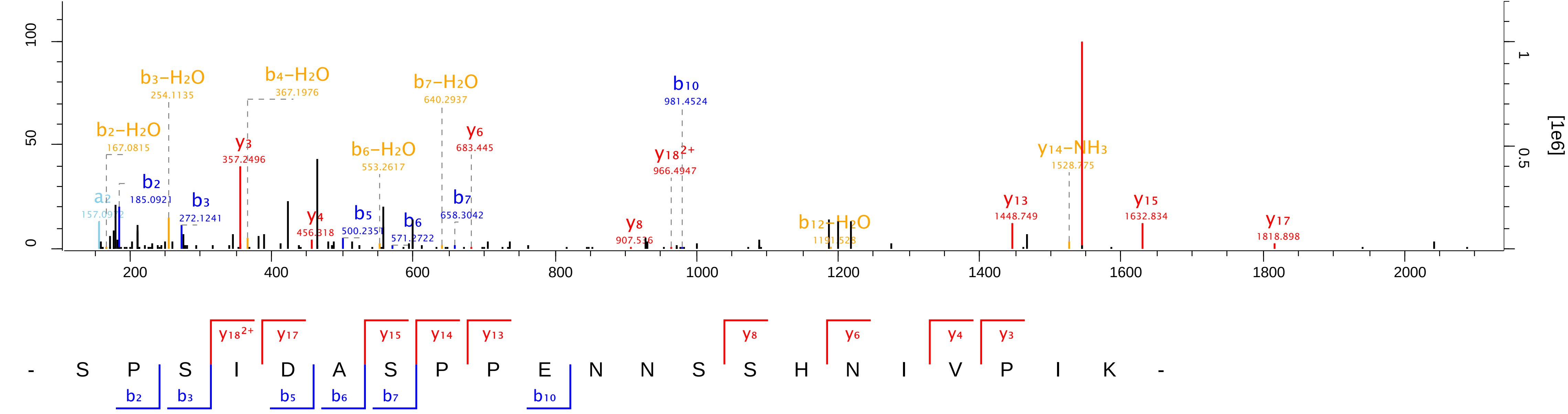

Raw file	Scan	Method	Score	m/z	Gene names
20140602_QEp4_FaHo_SA_HIF1_03	2444	FTMS; HCD	98.58	448.23	RPL4B;RPL4A

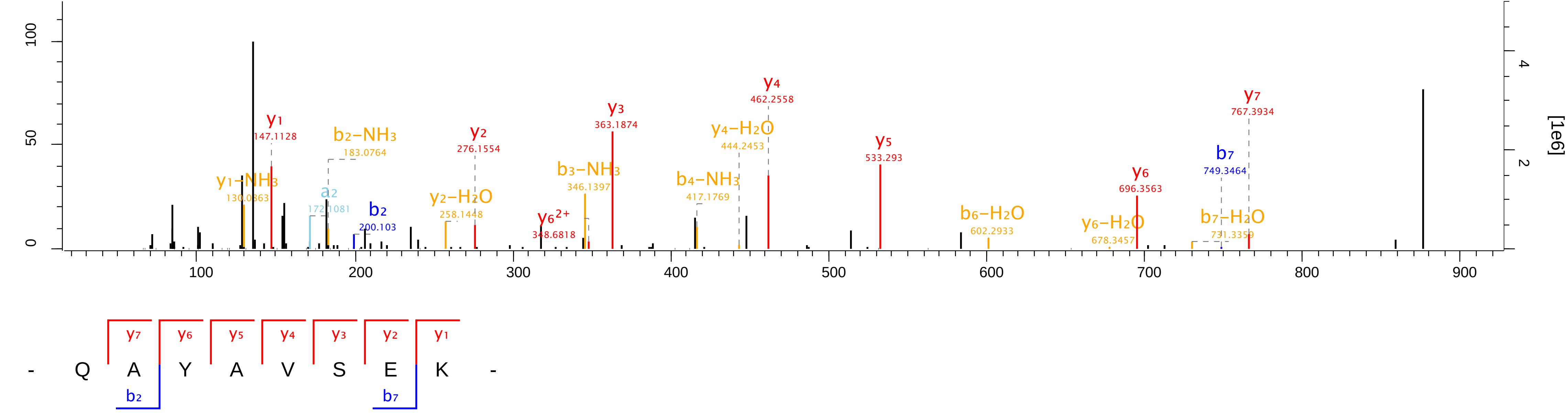

Raw file	Scan	Method	Score	m/z	Gene names
20140602_QEp4_FaHo_SA_HIF1_03	7284	FTMS; HCD	121.99	466.29	NTF2

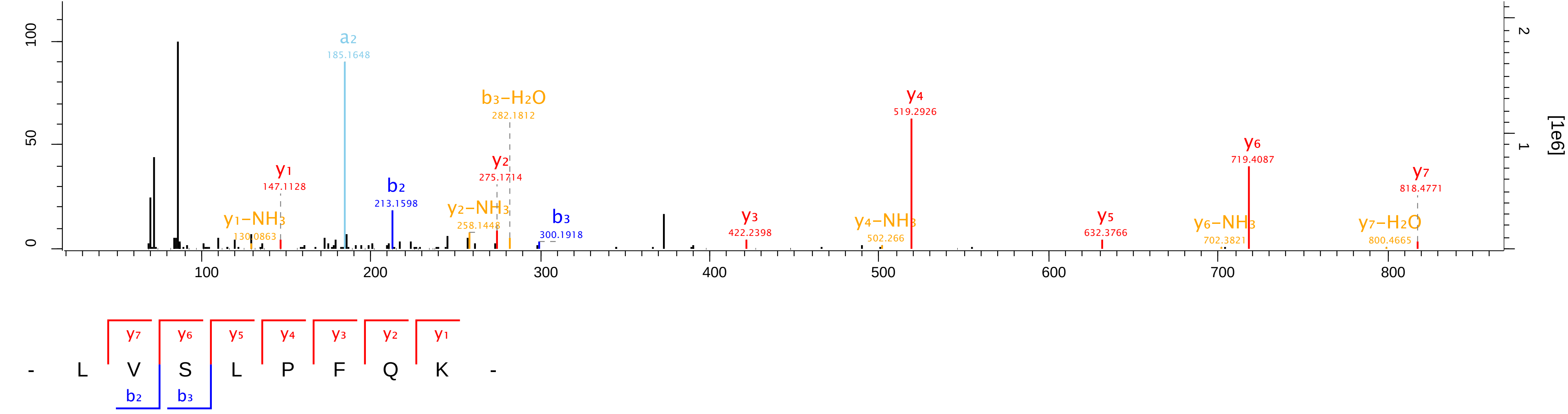

Raw file	Scan	Method	Score	m/z	Gene names
20140602_QEp4_FaHo_SA_IOC4_03	3370	FTMS; HCD	97.97	320.19	RPL31A;RPL31B

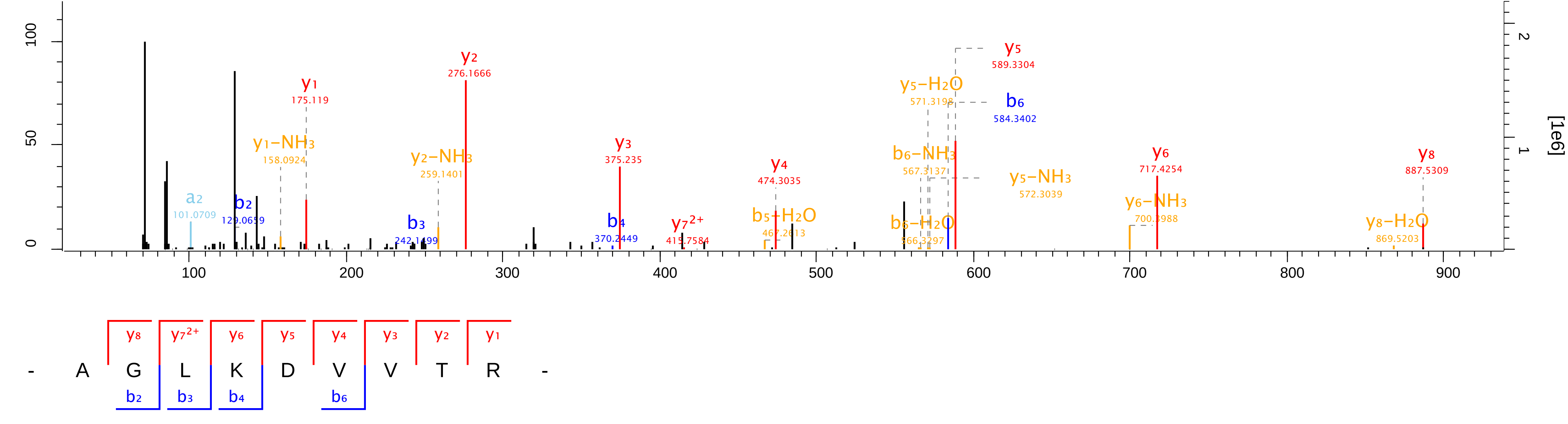

Raw file	Scan	Method	Score	m/z	Gene names
20140602_QEp4_FaHo_SA_IOC4_03	4928	FTMS; HCD	198.07	461.74	RPL14B;RPL14A

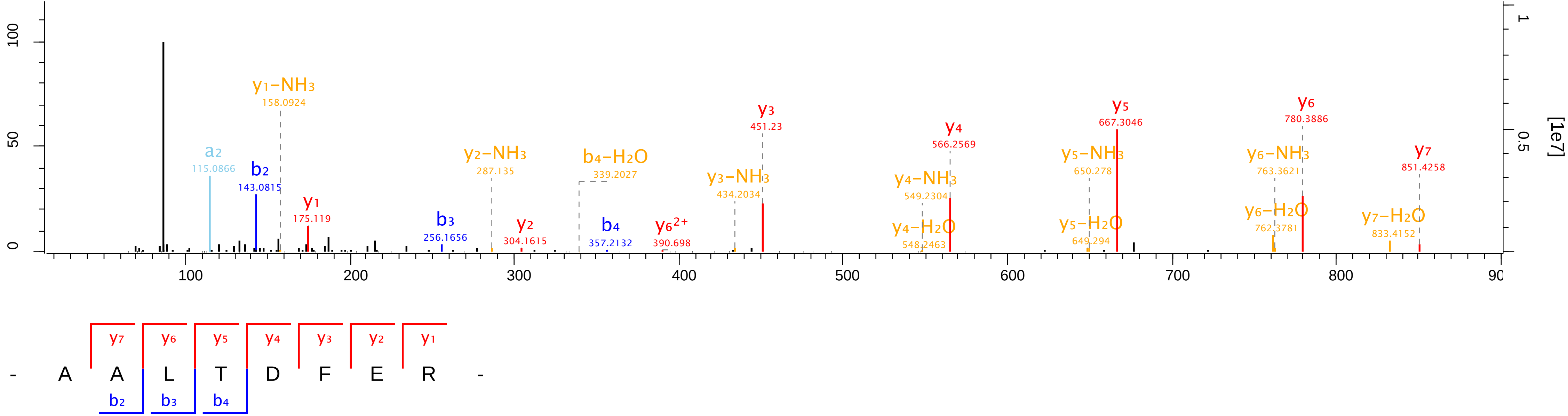

Raw file	Scan	Method	Score	m/z	Gene names
20140602_QEp4_FaHo_SA_IOC4_03	5826	FTMS; HCD	104.22	493.8	CDC19;PYK2

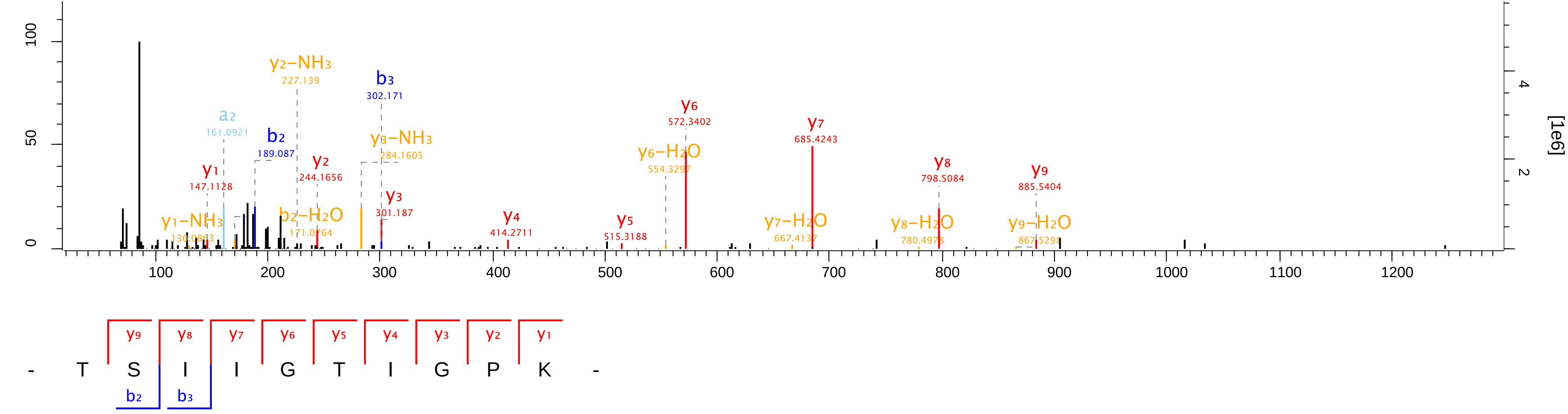

Raw file	Scan	Method	Score	m/z	Gene names
20140602_QEp4_FaHo_SA_IOC4_03	8661	FTMS; HCD	128.54	715.9	DCW1

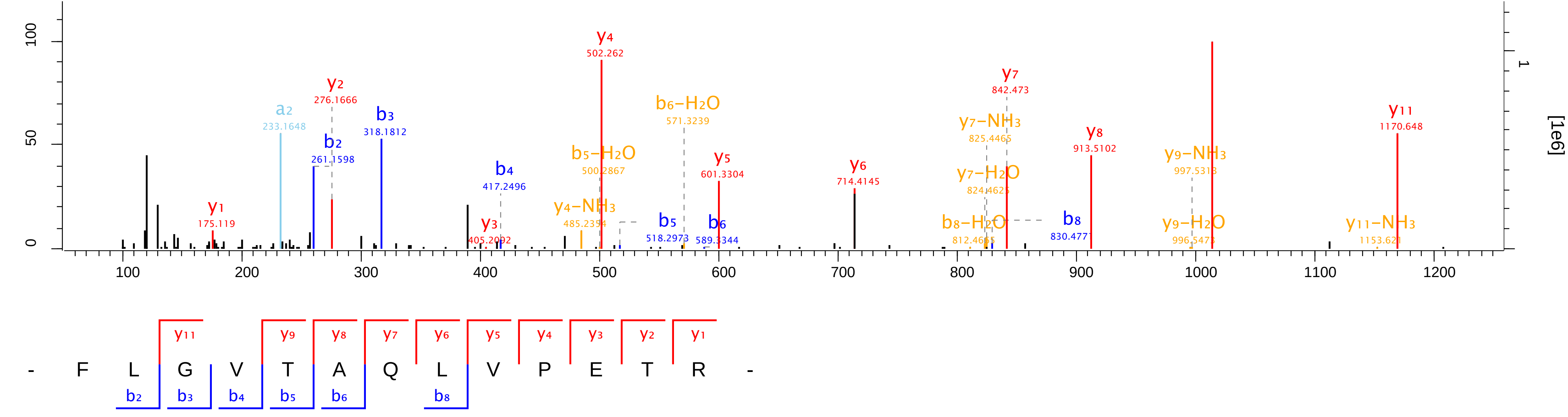

Raw file	Scan	Method	Score	m/z	Gene names
20140602_QEp4_FaHo_SA_NTO1_02	2834	FTMS; HCD	134.26	486.76	RPL36B

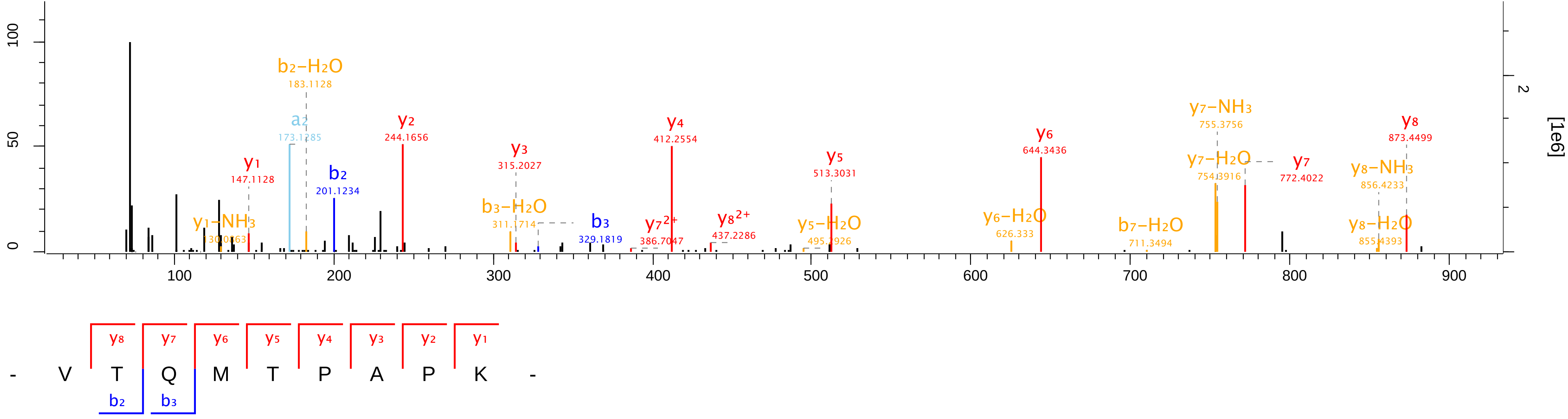

Raw file	Scan	Method	Score	m/z	Gene names
20140602_QEp4_FaHo_SA_NTO1_03	2936	FTMS; HCD	134.85	390.7	RPL17A;RPL17B

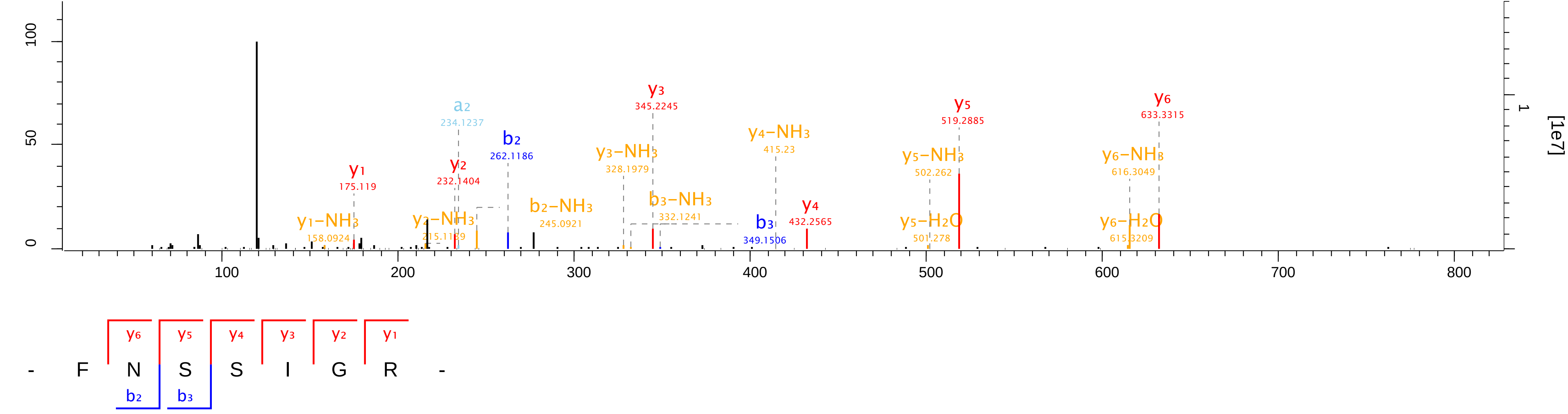

Raw file	Scan	Method	Score	m/z	Gene names
20140602_QEp4_FaHo_SA_NTO1_03	3110	FTMS; HCD	163.84	414.75	RPL9A;RPL9B

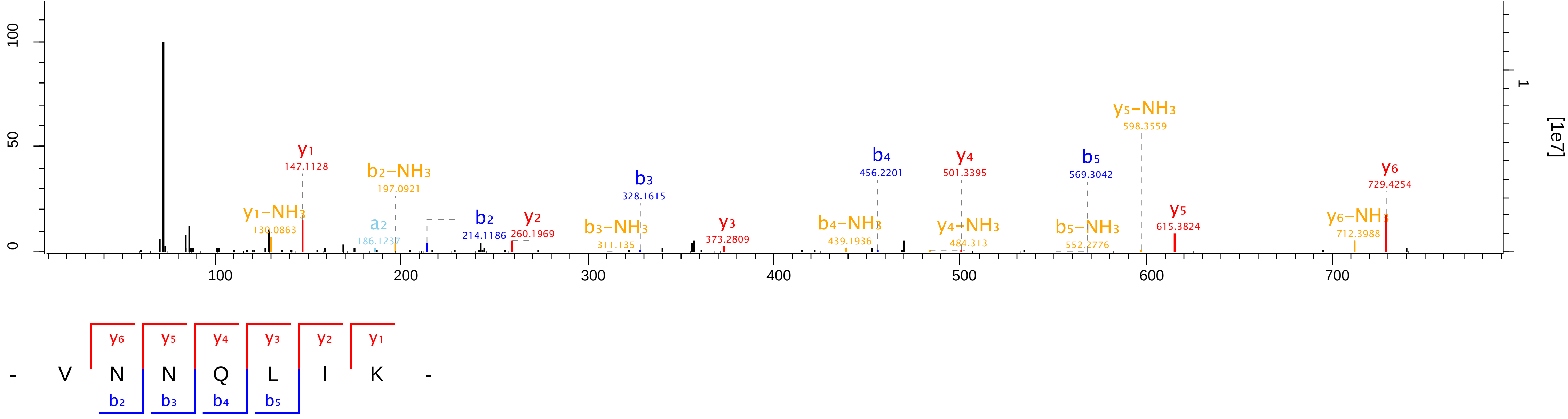

Raw file	Scan	Method	Score	m/z	Gene names
20140602_QEp4_FaHo_SA_NTO1_03	3675	FTMS; HCD	167.67	605.83	TY1B-NL1;TY1B-BL;TY1B-MR1;TY1A-MR1

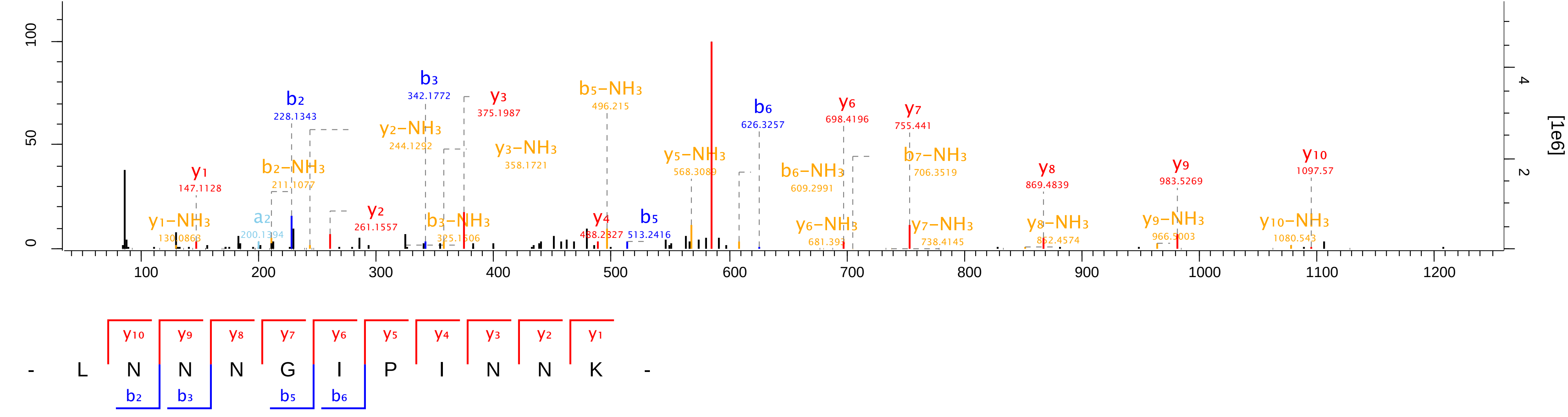

Raw file	Scan	Method	Score	m/z	Gene names
20140602_QEp4_FaHo_SA_NTO1_03	5759	FTMS; HCD	233.87	1064.51	TY1B-BR;TY1B-MR2;TY1B-OR;TY1B-NL2;TY1B-JR2;TY1B-NL1;TY1B-LR4;TY1B-PR3;TY1B-PL;TY1B-LR2;TY1B-DR6;TY1B-BL;TY1B-MR1

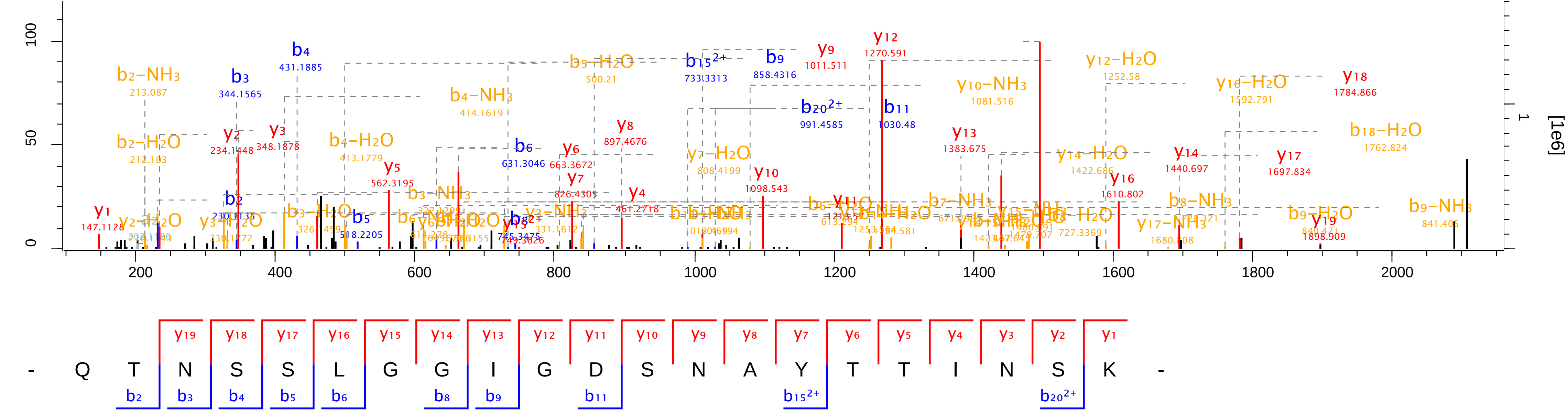

Raw file	Scan	Method	Score	m/z	Gene names
20140602_QEp4_FaHo_SA_NTO1_03	6277	FTMS; HCD	154.84	580.3	RPL31A;RPL31B

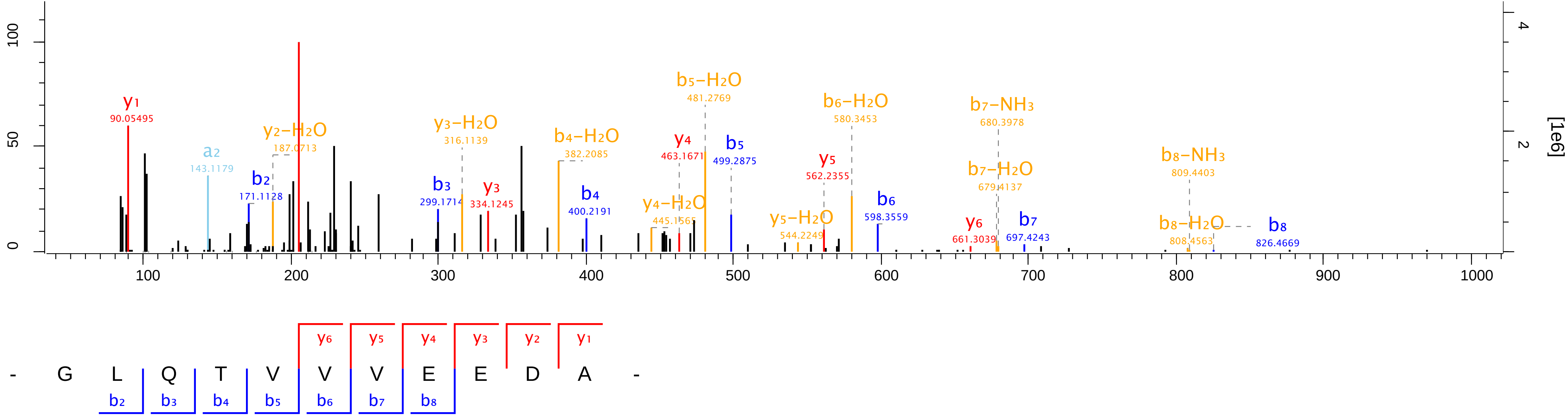

Raw file	Scan	Method	Score	m/z	Gene names
20140602_QEp4_FaHo_SA_parental_01	6375	FTMS; HCD	91.91	394.25	RPL7A;RPL7B

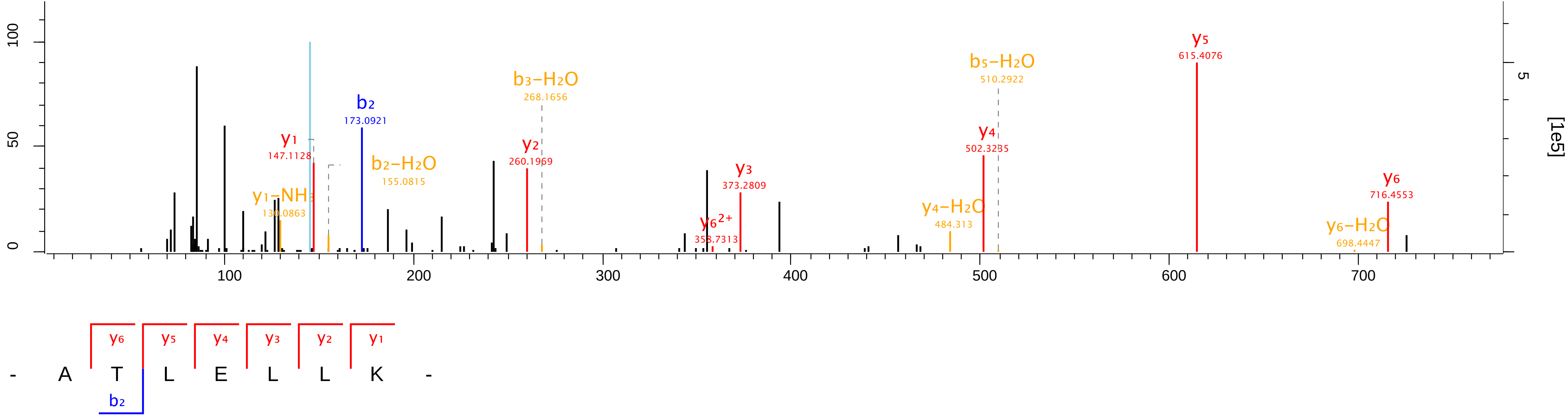

Raw file	Scan	Method	Score	m/z	Gene names
20140602_QEp4_FaHo_SA_parental_01	7691	FTMS; HCD	113.93	579.83	SSS1

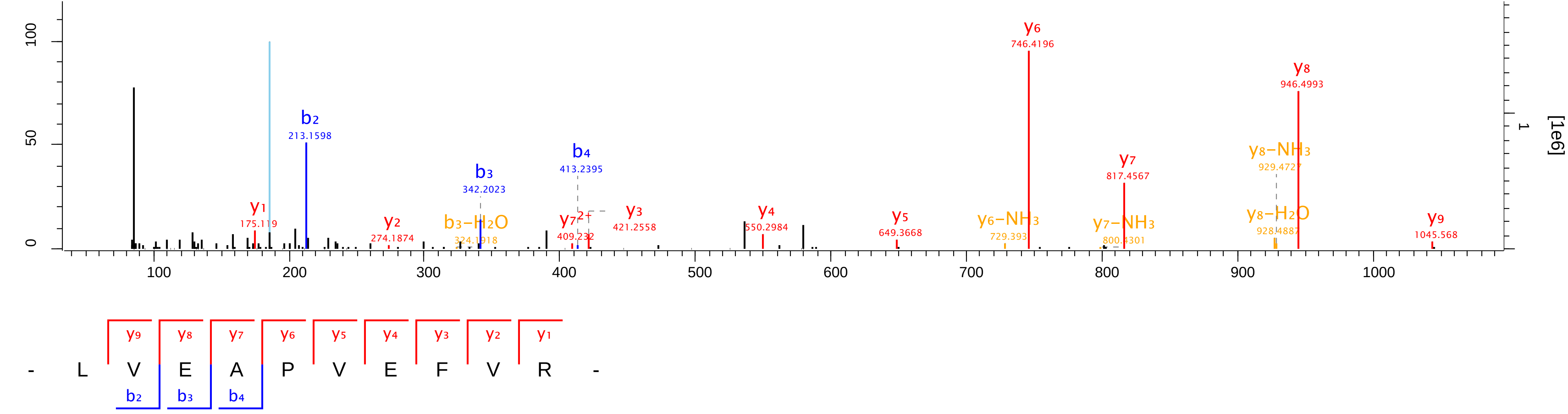

Raw file	Scan	Method	Score	m/z	Gene names
20140602_QEp4_FaHo_SA_parental_02	1683	FTMS; HCD	87.25	431.23	TY1A-PL;TY1A-LR2;TY1A-ER1;TY1A-DR6;TY1B-OL;TY1B-LR4;TY1B-ML2;TY1B-PR3;TY1B-PL;TY1B-LR2;TY1B-DR6

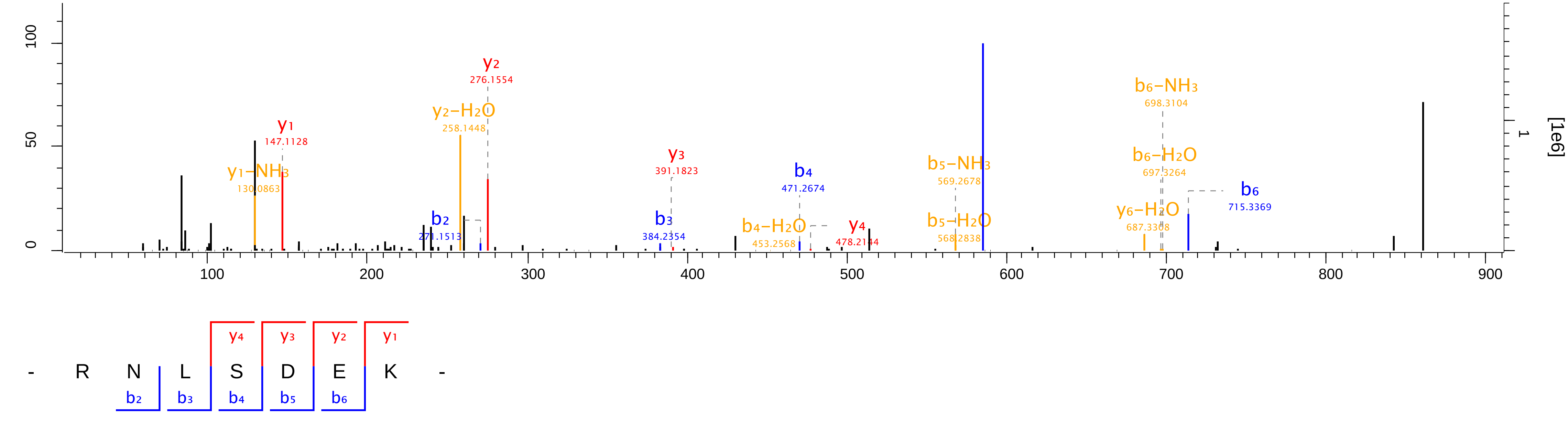

Raw file	Scan	Method	Score	m/z	Gene names
20140602_QEp4_FaHo_SA_parental_02	2081	FTMS; HCD	30.57	343.85	RPL13B;RPL13A

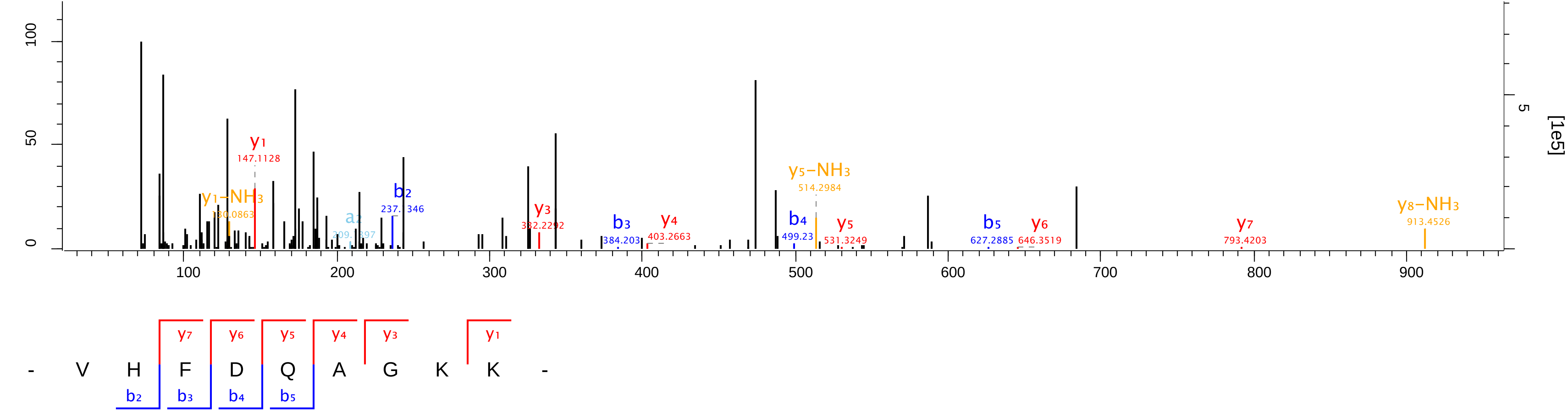

Raw file	Scan	Method	Score	m/z	Gene names
20140602_QEp4_FaHo_SA_parental_02	3361	FTMS; HCD	94.41	367.21	TY1B-ML1;TY1B-BR;TY1B-H;TY1B-MR2;TY1B-OR;TY1B-PR2;TY1B-DR5;TY1B-JR2;TY1B-NL1;TY1B-PR3;TY1B-BL;TY1B-MR1

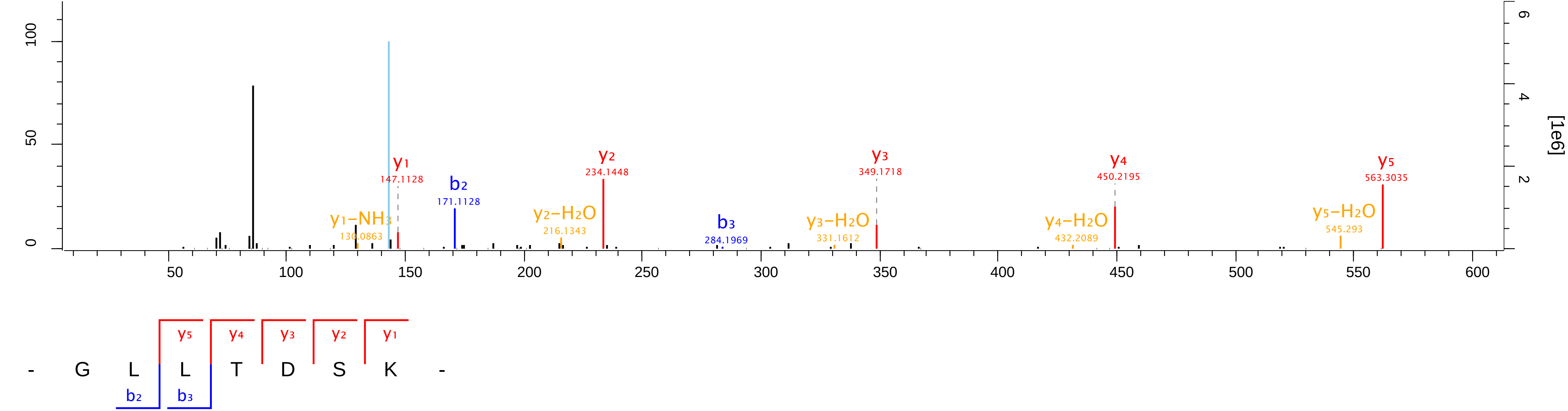

Raw file	Sca	Metho	Scor	m/z	Gene names
20140602_QEp4_FaHo	35	FTMS	147	558	TY1B-ML1;TY1B-BR;TY1B-H;TY1B-MR2;TY1B-OR;TY1B-DR1;TY1B-NL2;TY1B-PR2;TY1B-DR5;TY1B-PR1;TY1B-JR2;TY1B-NL1;TY2B-C;TY1B-OL;TY1B-LR4;TY1B-ML2;TY1B-DR3;T

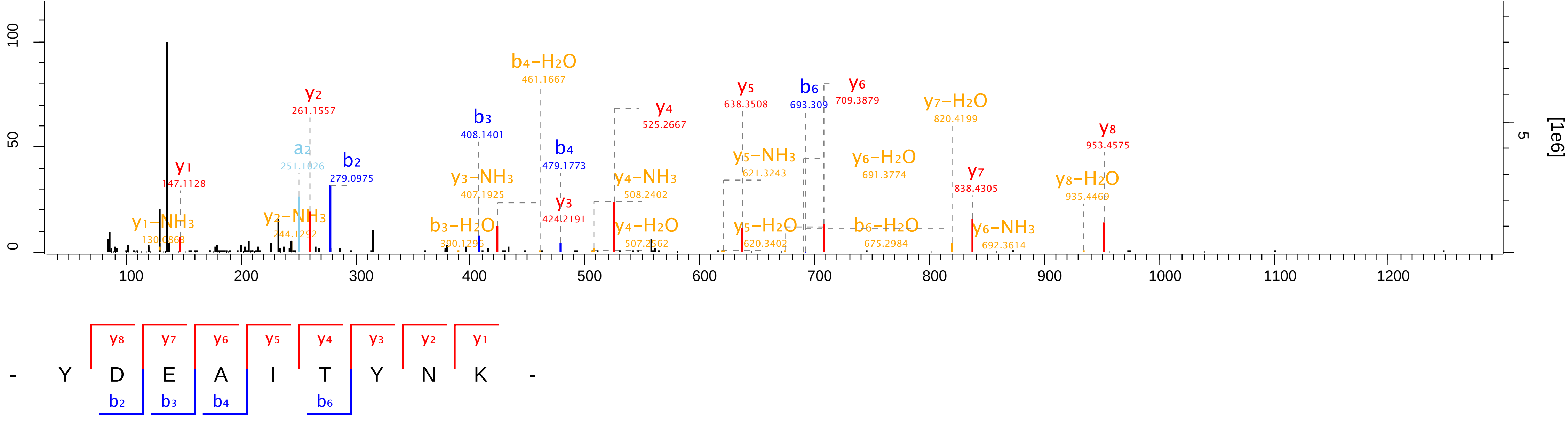

Raw file	Scan	Method	Score	m/z	Gene names
20140602_QEp4_FaHo_SA_pare	356[illegible]	FTMS;	105.2	381.2	TY1B-NL2;TY1B-PR1;TY2B-C;TY1B-OL;TY1B-LR4;TY1B-ML2;TY1B-DR3;TY1B-PL;TY1B-LR2;TY1B-DR6;TY1B-A;TY2B-B;TY2B-GR2;TY2B-F;TY2B-GR1;TY2B-OR1

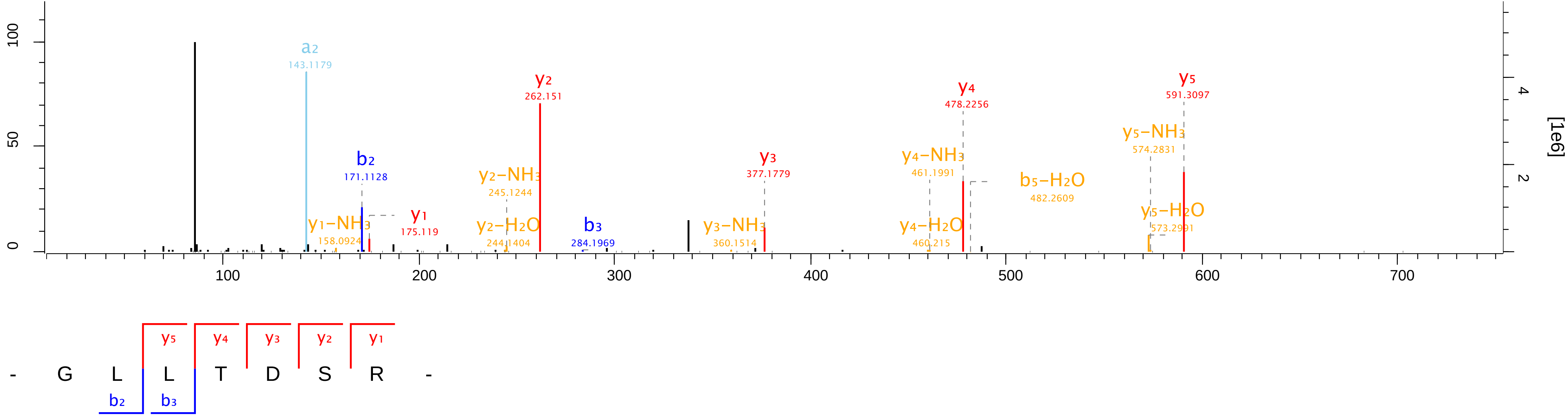

Raw file	Scan	Method	Score	m/z	Gene names
20140602_QEp4_FaHo_SA_parental_02	4199	FTMS; HCD	138.99	497.88	TY1B-JR2;TY1B-OL;TY1B-LR4;TY1B-ML2;TY1B-PL;TY1B-LR2;TY1B-DR6

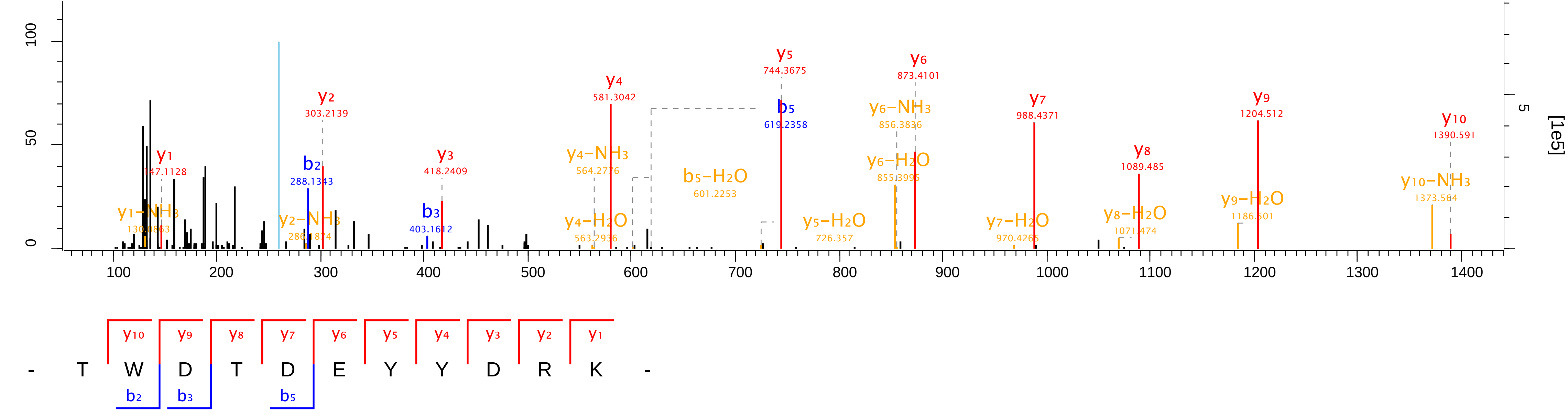

Raw file	Scan	Method	Score	m/z	Gene names
20140602_QEp4_FaHo_SA_parental_02	4997	FTMS; HCD	82.64	695.85	TY1B-PR3

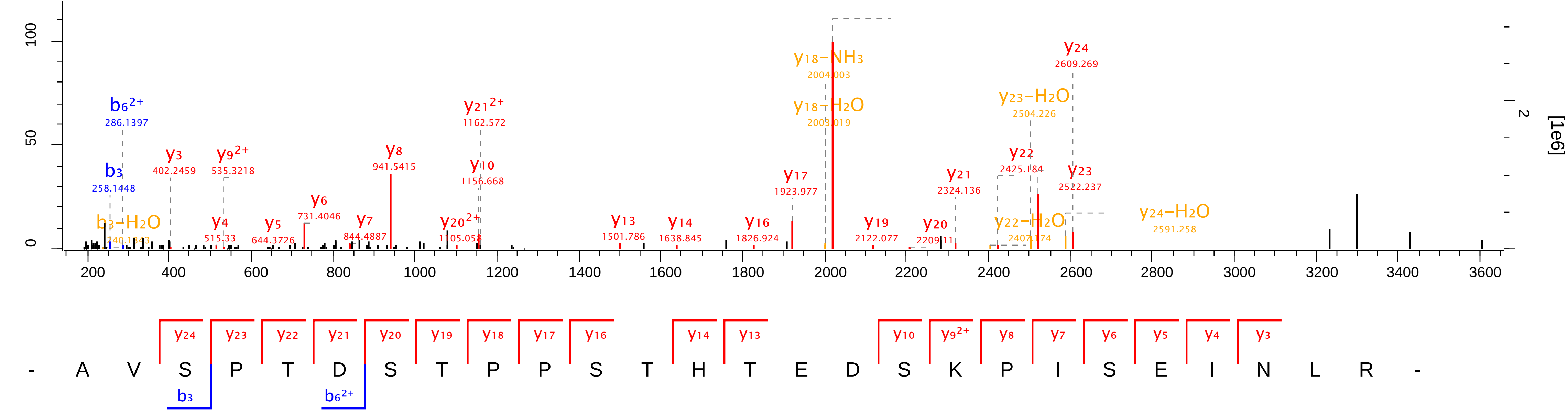

Raw file	Scan	Method	Score	m/z	Gene names
20140602_QEp4_FaHo_SA_parental_02	7727	FTMS; HCD	120.93	781.63	TY1B-ML1;TY1B-H;TY1B-NL2;TY1B-PR2;TY1B-DR5;TY1B-PR1;TY1B-OL;TY1B-LR4;TY1B-ML2;TY1B-PR3;TY1B-PL;TY1B-LR2;TY1B-DR6

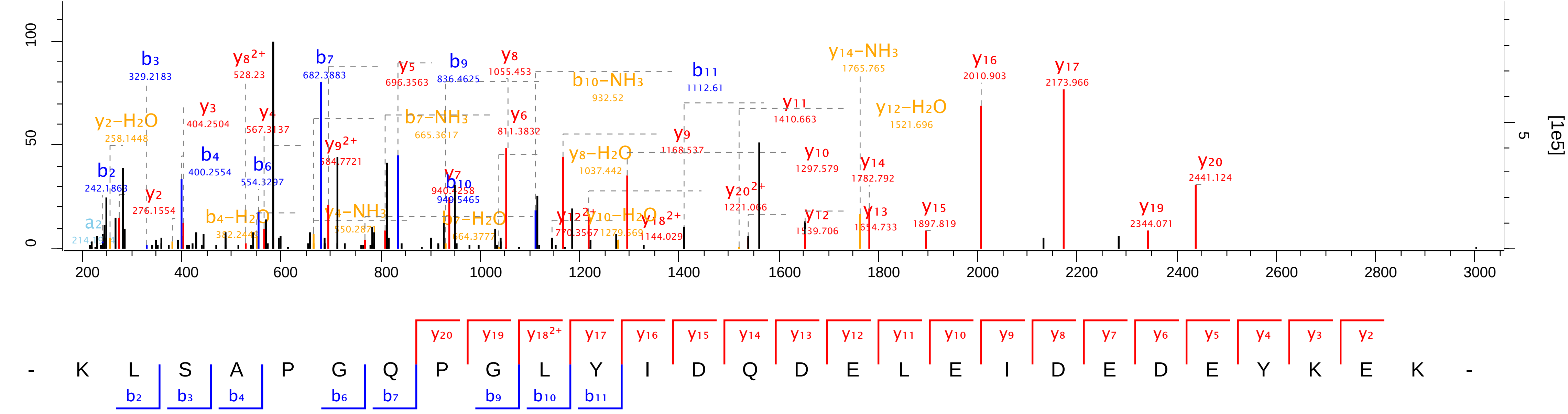

Raw file	Scan	Method	Score	m/z	Gene names
20140602_QEp4_FaHo_SA_parental_02	9615	FTMS; HCD	67.65	678.68	GCN20

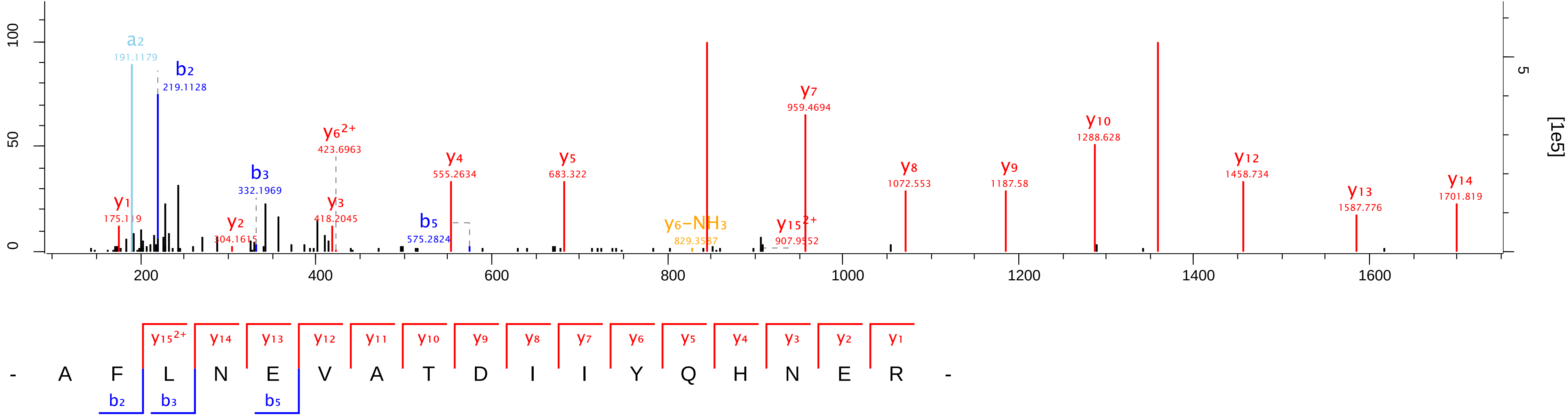

Raw file	Scan	Method	Score	m/z	Gene names
20140602_QEp4_FaHo_SA_parenta	7684	FTMS;	159.94	660.37	TY1B-ML1;TY1B-BR;TY1B-H;TY1B-MR2;TY1B-OR;TY1B-PR2;TY1B-DR5;TY1B-JR2;TY1B-NL1;TY1B-OL;TY1B-ML2;TY1B-PR3;TY1B-PL;TY1B-LR2;TY1B-BL;TY

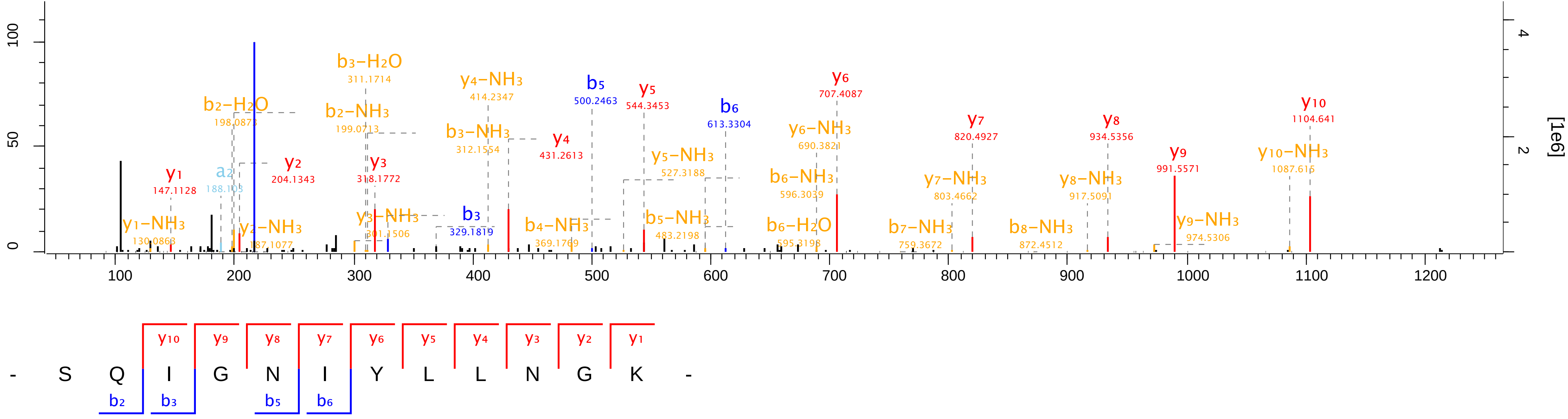

Raw file	Scan	Method	Score	m/z	Gene names
20140602_QEp4_FaHo_SA_PHO23_01	3609	FTMS; HCD	169.79	772.4	RPS14B;RPS14A

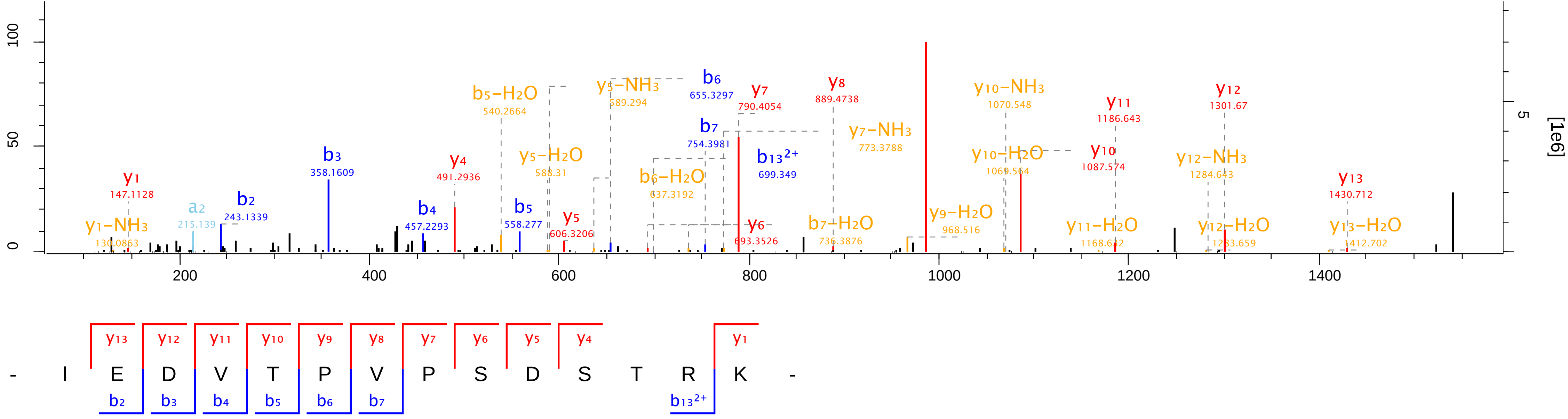

Raw file	Scan	Method	Score	m/z	Gene names
20140602_QEp4_FaHo_SA_PHO23_01	3747	FTMS; HCD	115.57	665.85	RPO41

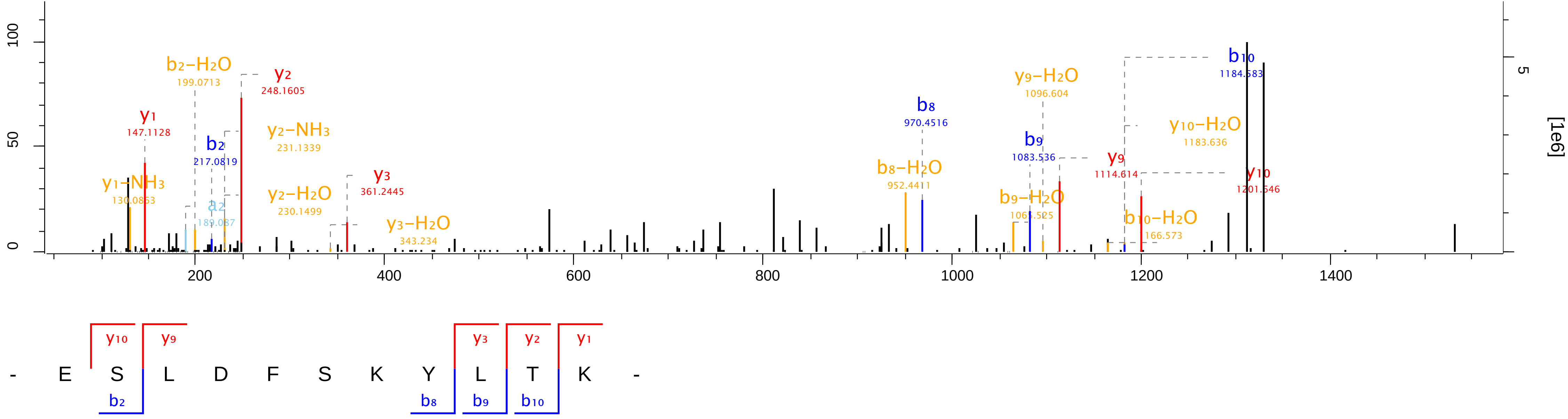

Raw file	Scan	Method	Score	m/z	Gene names
20140602_QEp4_FaHo_SA_PHO23_01	5074	FTMS; HCD	158.98	690.33	TY1B-BL;TY1B-MR1;TY1A-MR1

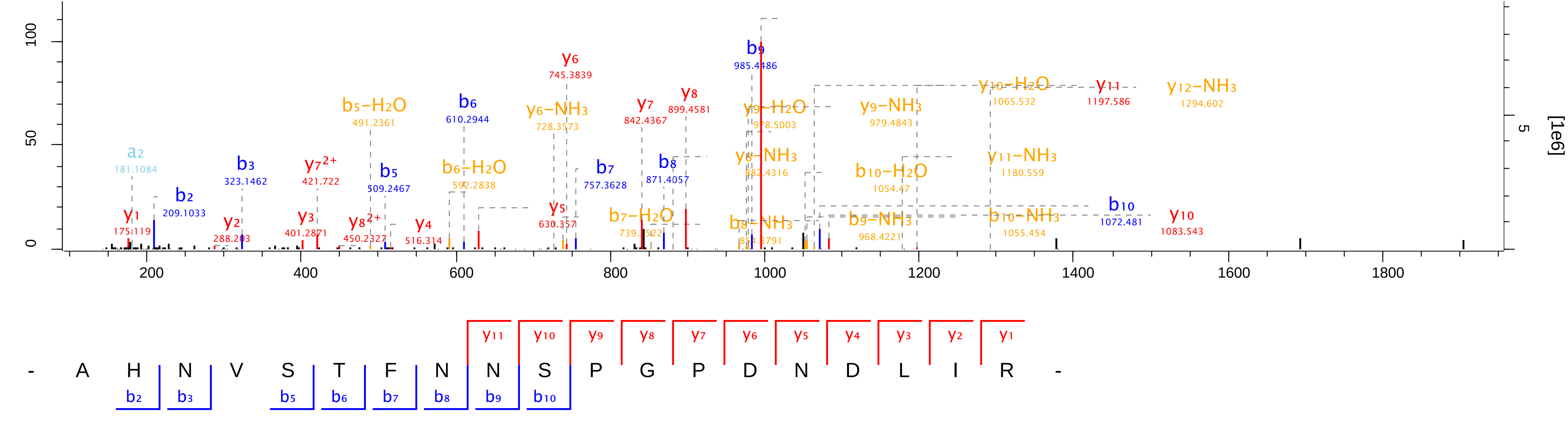

Raw file	Scan	Method	Score	m/z	Gene names
20140602_QEp4_FaHo_SA_PHO23_01	5456	FTMS; HCD	221.83	890.41	TY1B-OL

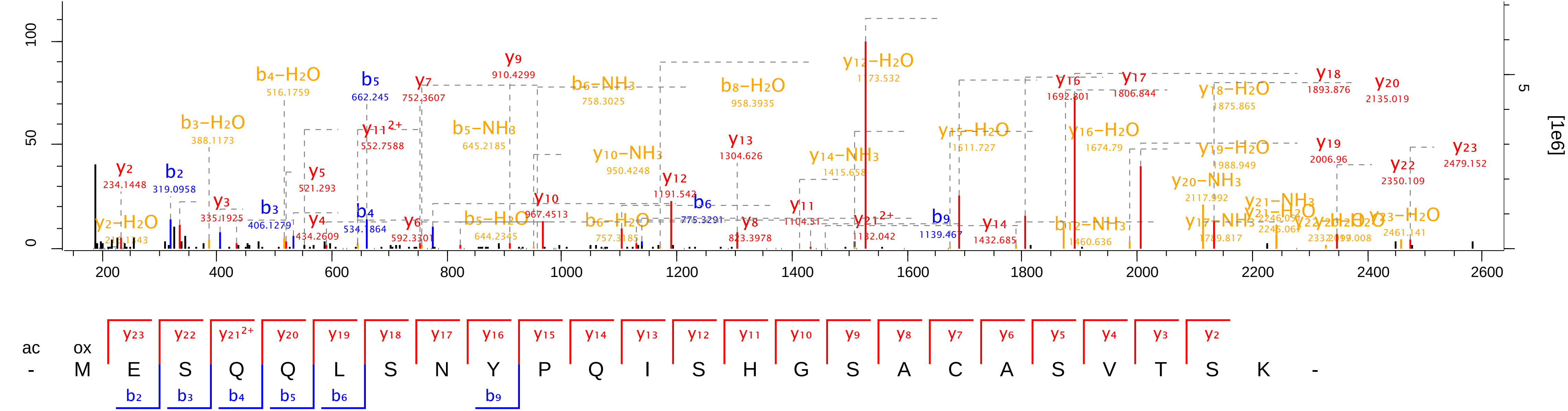

Raw file	Scan	Method	Score	m/z	Gene names
20140602_QEp4_FaHo_SA_PHO23_01	9876	FTMS; HCD	140.45	985.51	RPL13A

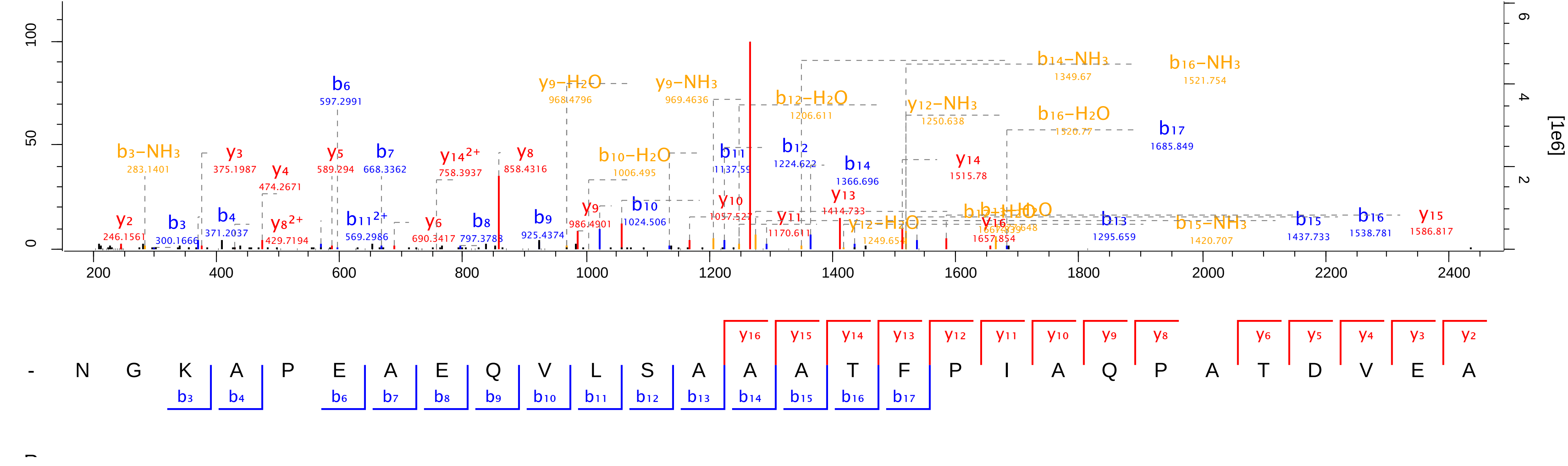

Raw file	Scan	Method	Score	m/z	Gene names
20140602_QEp4_FaHo_SA_PHO23_02	4954	FTMS; HCD	169.65	667.33	RPL13B;RPL13A

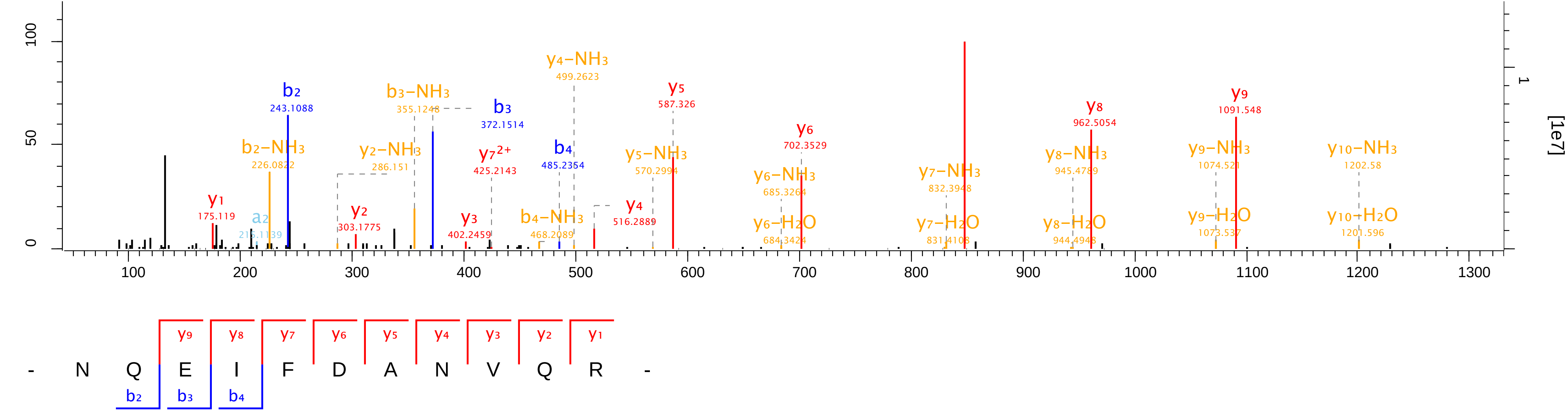

Raw file	Scan	Method	Score	m/z	Gene names
20140602_QEp4_FaHo_SA_PHO23_02	9130	FTMS; HCD	140.2	787.74	TOM22

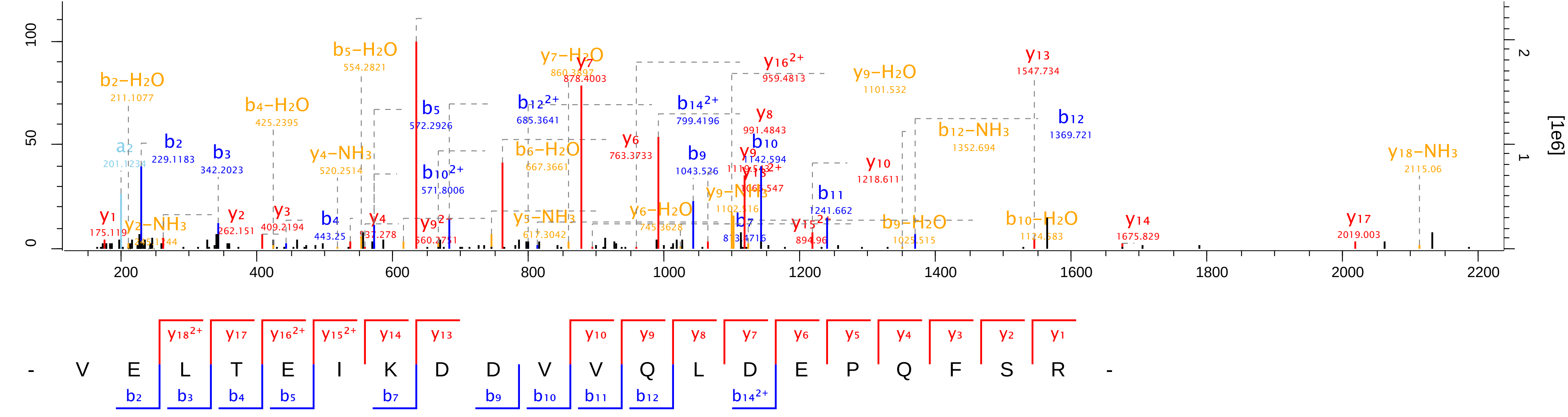

Raw file	Scan	Method	Score	m/z	Gene names
20140602_QEp4_FaHo_SA_PHO23_03	3751	FTMS; HCD	133.86	518.24	ARF1;ARF2

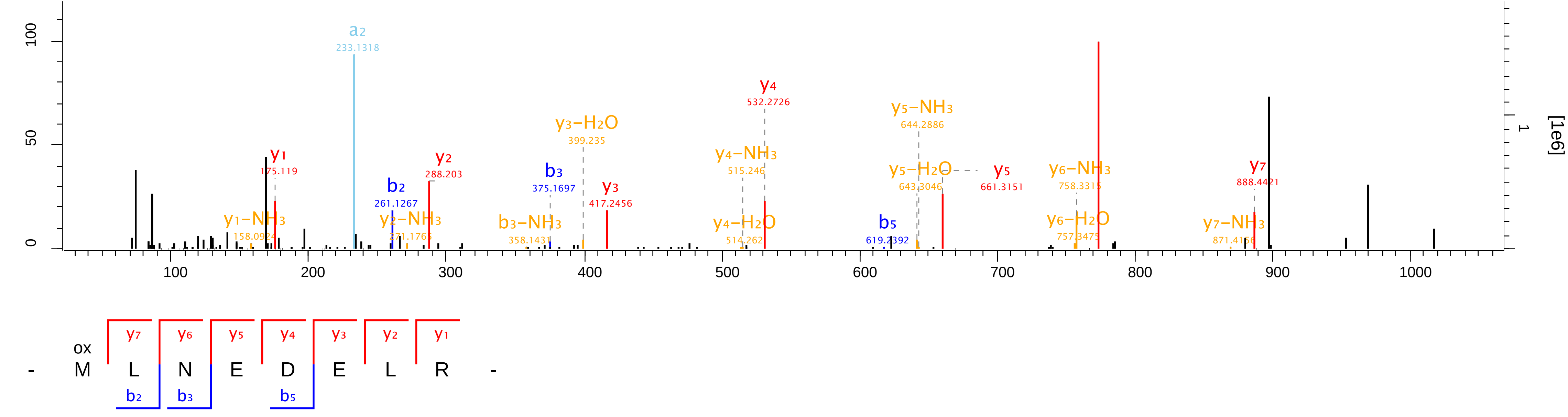

Raw file	Scan	Method	Score	m/z	Gene names
20140602_QEp4_FaHo_SA_PHO23_03	5581	FTMS; HCD	219.23	893.08	TY1B-BL

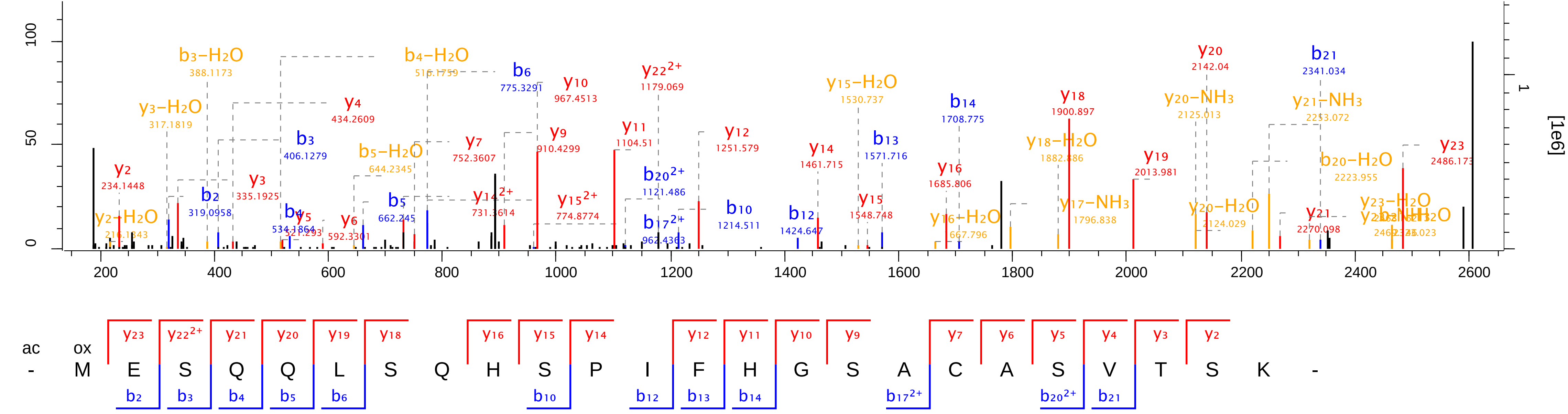

Raw file	Scan	Method	Score	m/z	Gene names
20140602_QEp4_FaHo_SA_RCO1_02	2326	FTMS; HCD	175.88	505.25	RPL17A;RPL17B

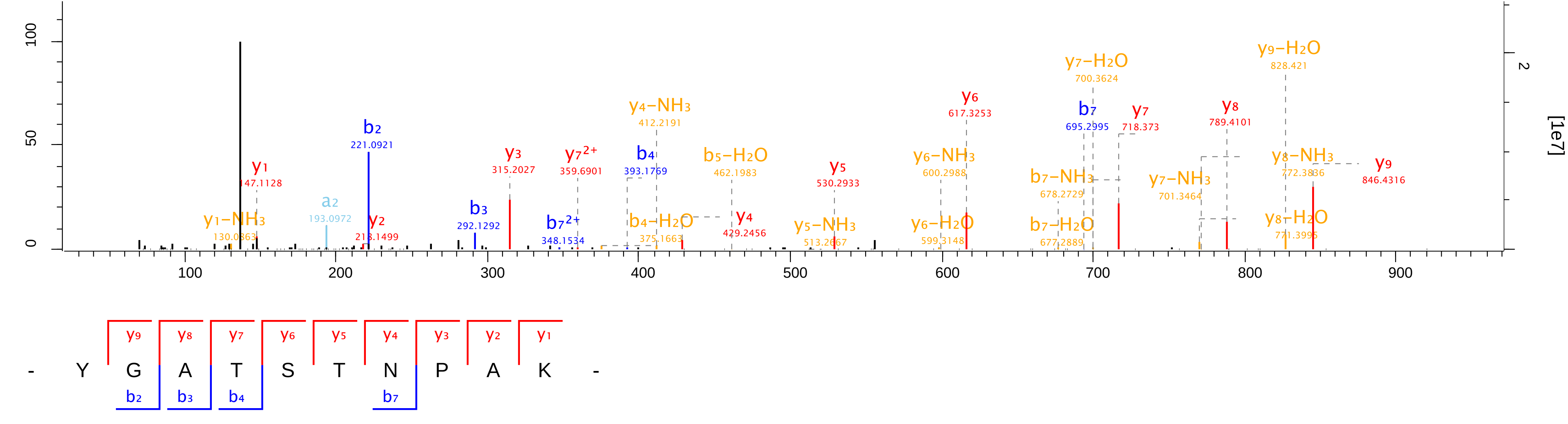

Raw file	Scan	Method	Score	m/z	Gene names
20140602_QEp4_FaHo_SA_RCO1_02	3957	FTMS; HCD	148.96	681.84	RPL36B;RPL36A

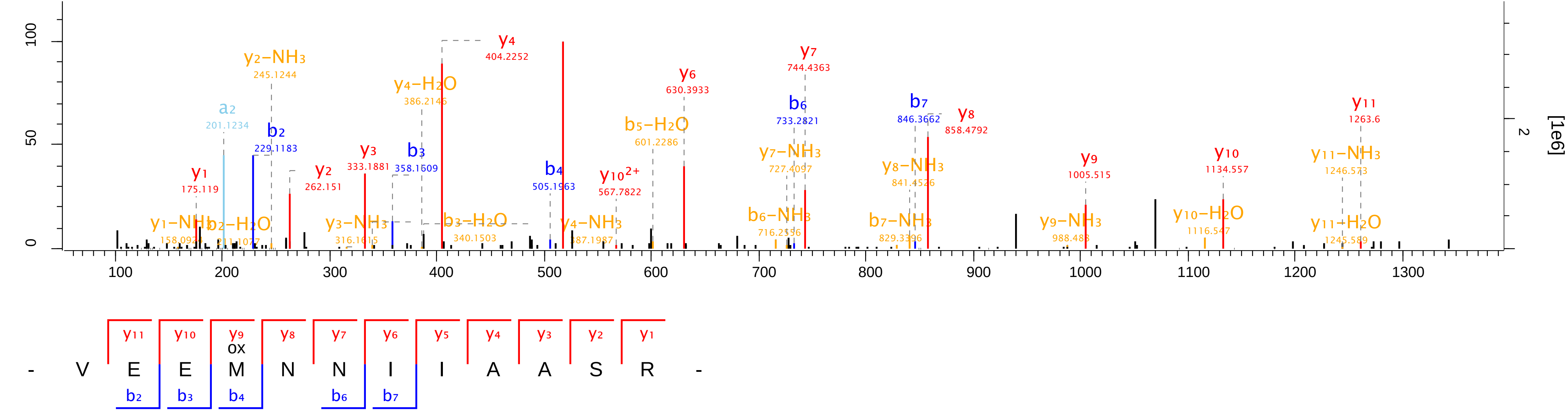

Raw file	Scan	Method	Score	m/z	Gene names
20140602_QEp4_FaHo_SA_RCO1_02	7234	FTMS; HCD	183.15	871.4	TY1B-PR3

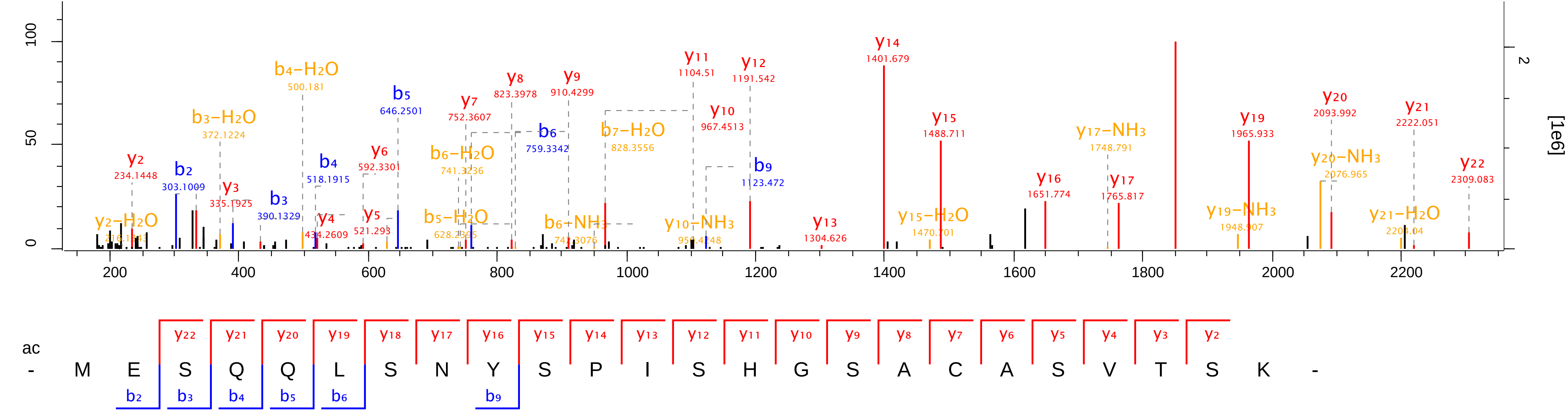

Raw file	Scan	Method	Score	m/z	Gene names
20140602_QEp4_FaHo_SA_RCO1_02	9347	FTMS; HCD	142.91	852.45	VMA2

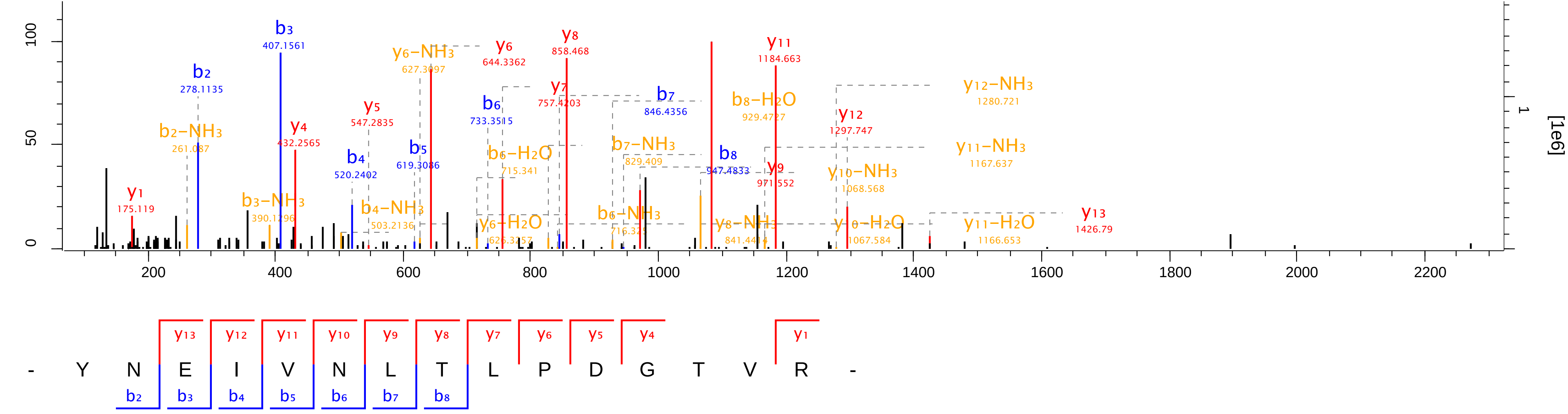

Raw file	Scan	Method	Score	m/z	Gene names
20140602_QEp4_FaHo_SA_RCO1_03	2675	FTMS; HCD	90.37	569.29	YHR212C;YAR060C

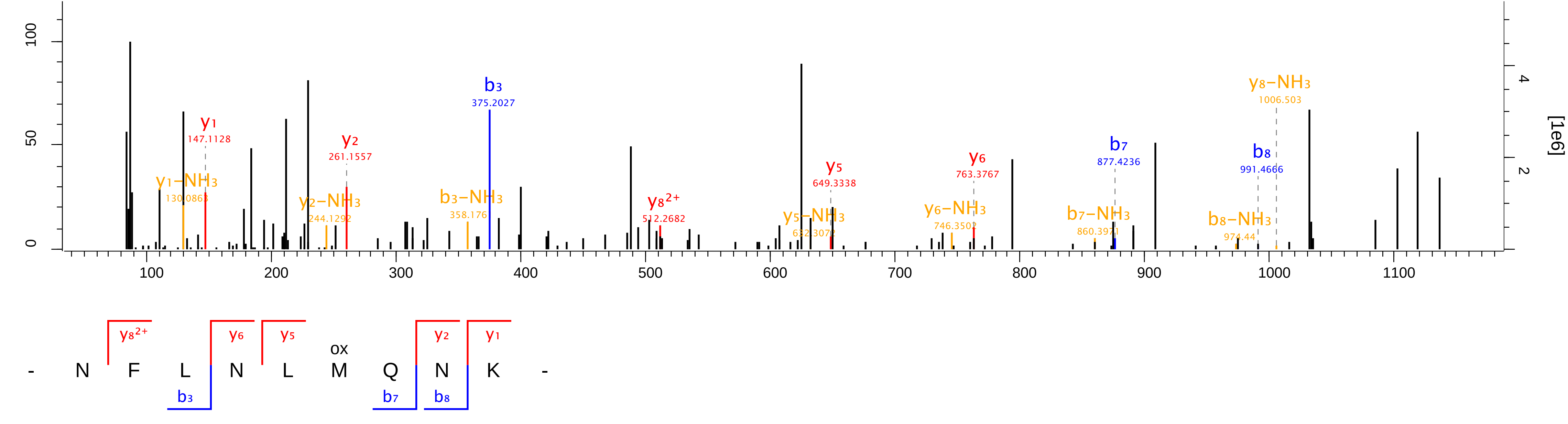

Raw file	Scan	Method	Score	m/z	Gene names
20140602_QEp4_FaHo_SA_RCO1_03	3799	FTMS; HCD	73.23	470.29	TRX2;TRX1

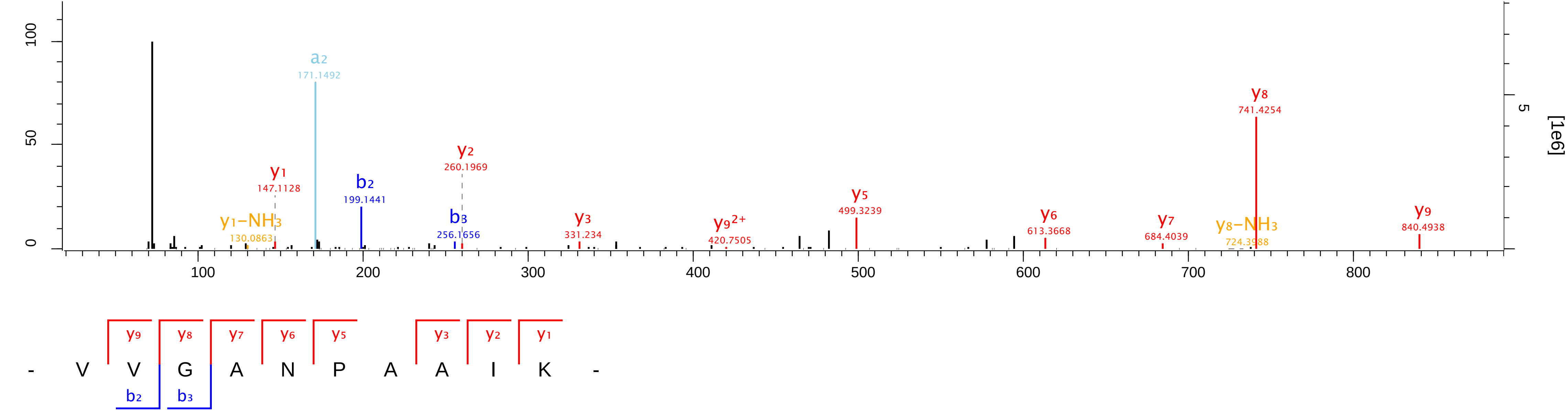

Raw file	Scar	Method	Score	m/z	Gene names
20140602_QEp4_FaHo_SA_	380	FTMS;	107.9	589.2	TY1B-ML1;TY1B-BR;TY1B-H;TY1B-MR2;TY1B-OR;TY1B-DR1;TY1B-NL2;TY1B-PR2;TY1B-DR5;TY1B-PR1;TY1B-JR2;TY1B-NL1;TY1B-OL;TY1B-LR4;TY1B-ML2;TY1B-DR3

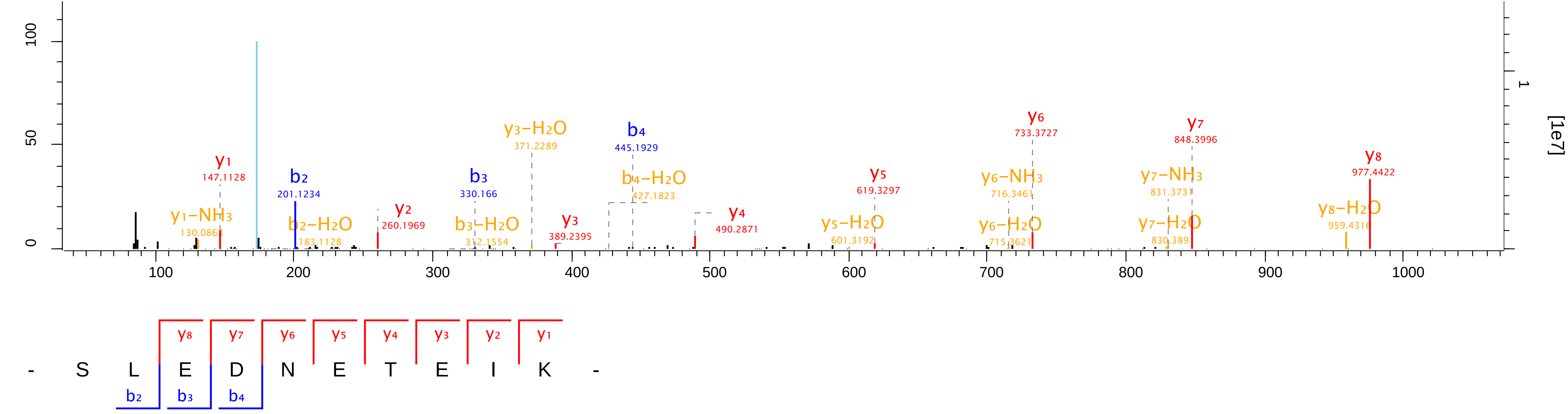

Raw file	Scan	Method	Score	m/z	Gene names
20140602_QEp4_FaHo_SA_RCO1_03	4101	FTMS; HCD	122.79	371.23	RPL14B;RPL14A

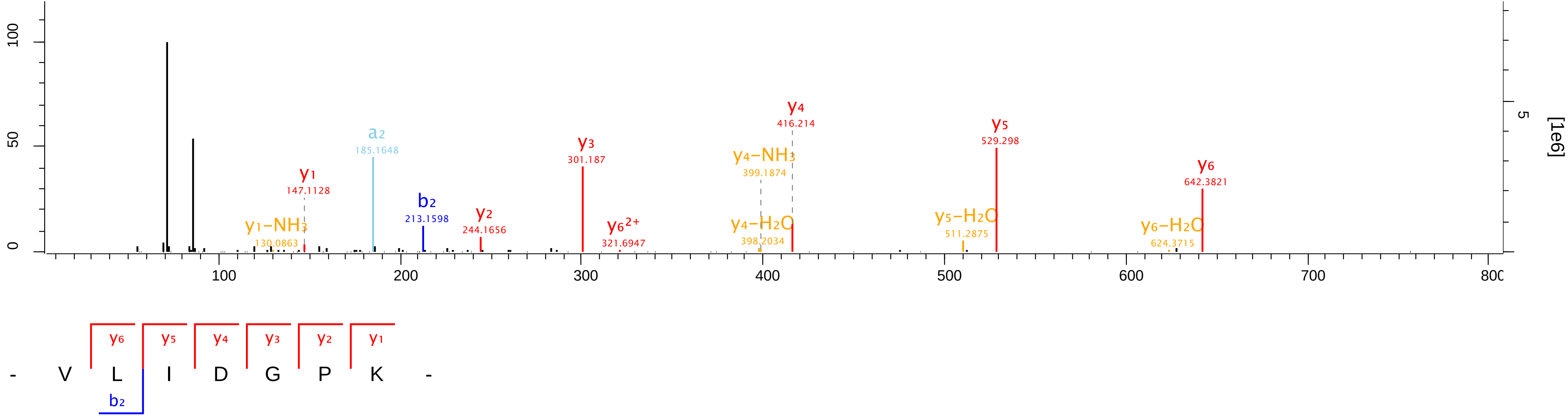

Raw file	Scan	Method	Score	m/z	Gene names
20140602_QEp4_FaHo_SA_RCO1_03	4645	FTMS; HCD	83.76	544.29	TY1B-ML1;TY1B-BR;TY1B-H;TY1B-MR2;TY1B-OR;TY1B-NL2;TY1B-PR2;TY1B-DR5;TY1B-PR1;TY1B-JR2;TY1B-DR3

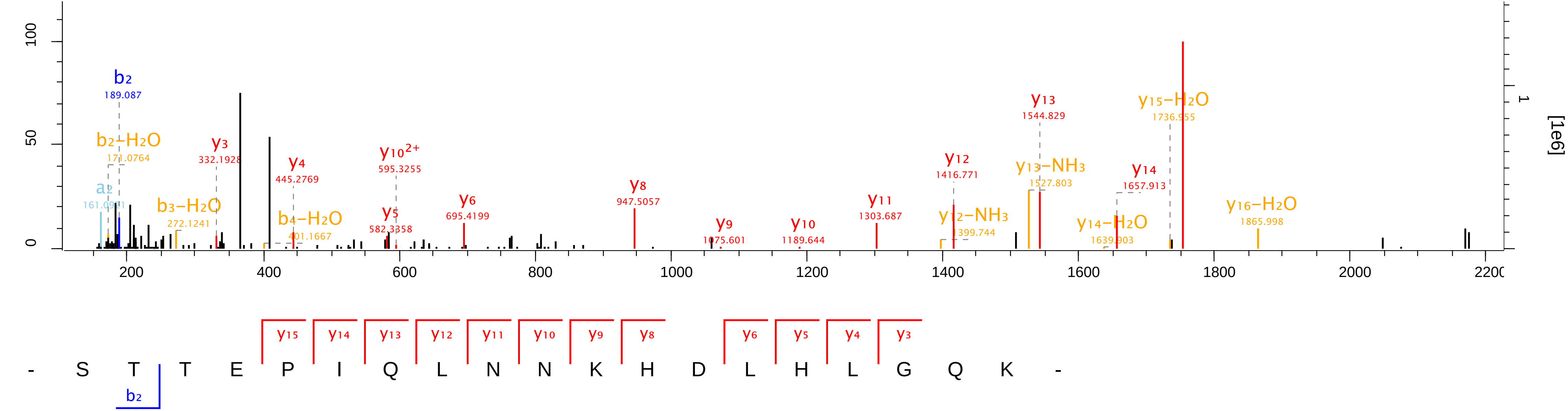

Raw file	Scan	Method	Score	m/z	Gene names
20140602_QEp4_FaHo_SA_RCO1_03	4931	FTMS; HCD	121.64	514.8	RPL7A;RPL7B

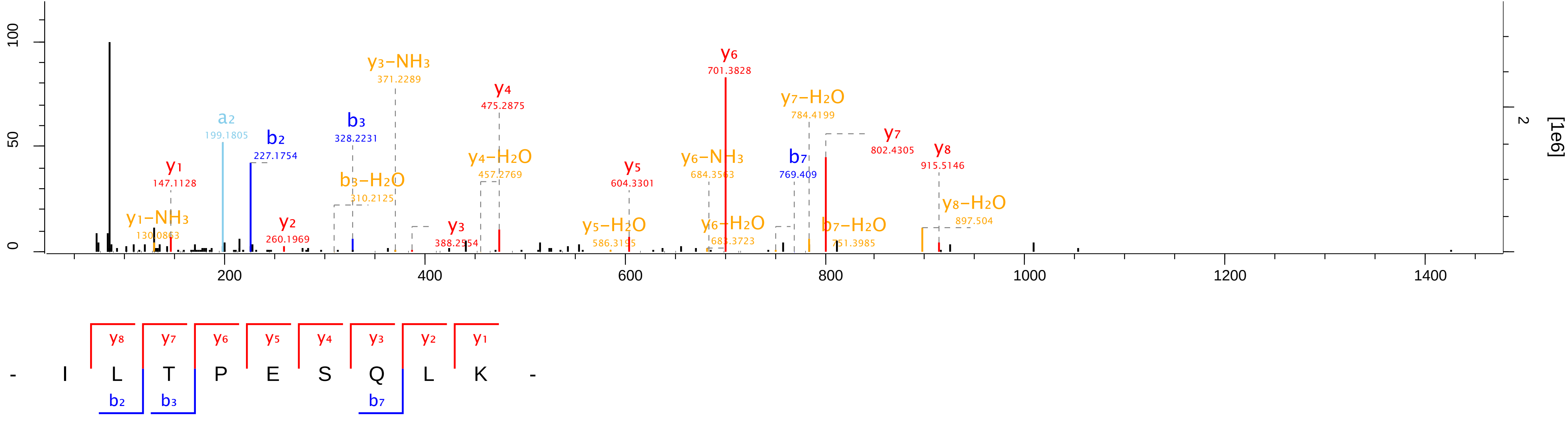

Raw file Scan Method Score m/z Gene names

20140602_QEp4_FaHo_SA_R(510(FTMS; 107.5 381.2 TY1B-DR1;TY1B-NL2;TY1B-PR1;TY2B-C;TY1B-OL;TY1B-LR4;TY1B-ML2;TY1B-DR3;TY1B-PL;TY1B-LR2;TY1B-DR6;TY1B-A;TY2B-B;TY2B-GR2;TY2B-F;TY2B-GR1;TY

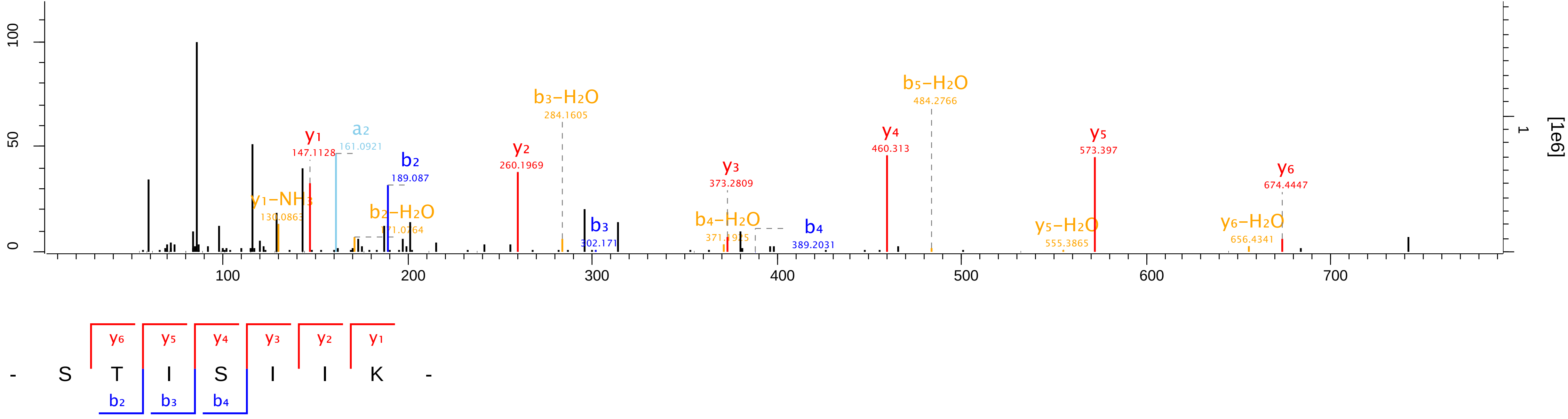

Raw file	Scan	Method	Score	m/z	Gene names
20140602_QEp4_FaHo_SA_RSC2_0	1715	FTMS; HCD	79.66	394.85	TY1A-PR1;TY1A-A;TY1A-DR4;TY1B-PR1;TY1B-JR2;TY1A-PL;TY1A-LR2;TY1A-ER1;TY1A-DR6;TY1B-OL;TY1B-LR4;TY1B-ML2;TY1B-PR3;TY1B-PL;TY1B-L

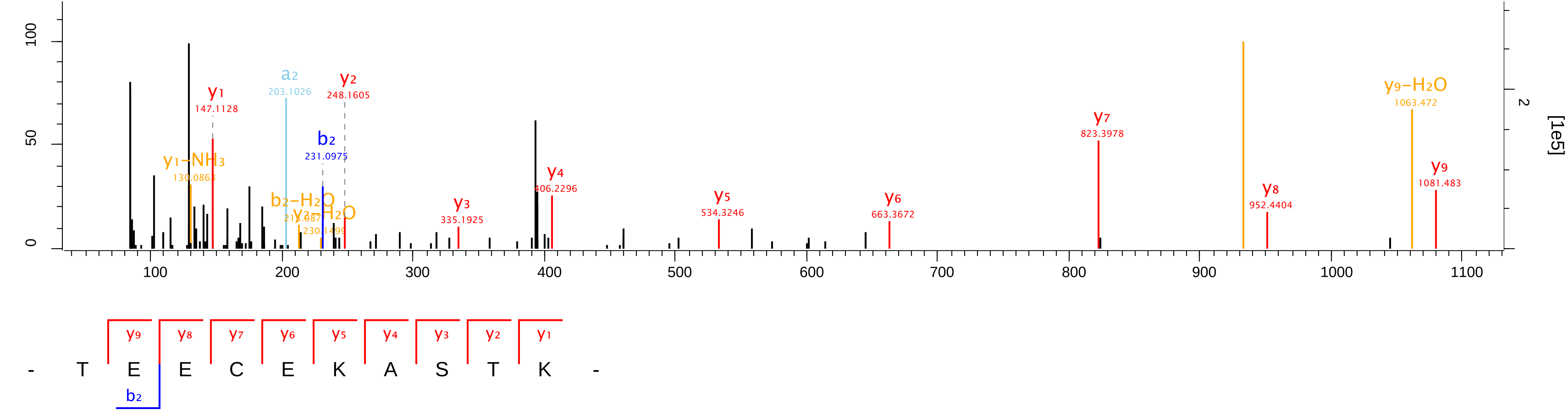

Raw file	Scan	Method	Score	m/z	Gene names
20140602_QEp4_FaHo_SA_RSC2_01	2857	FTMS; HCD	73.33	465.79	RPL4B;RPL4A

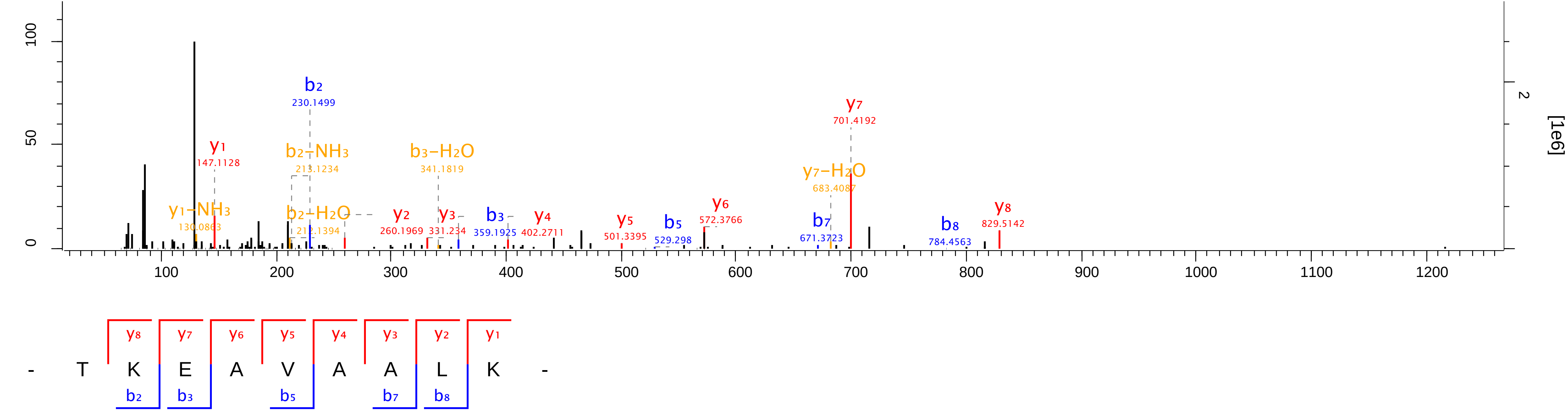

Raw file	Scan	Method	Score	m/z	Gene names
20140602_QEp4_FaHo_SA_RSC2_02	4397	FTMS; HCD	136.93	502.62	HXT6;HXT7

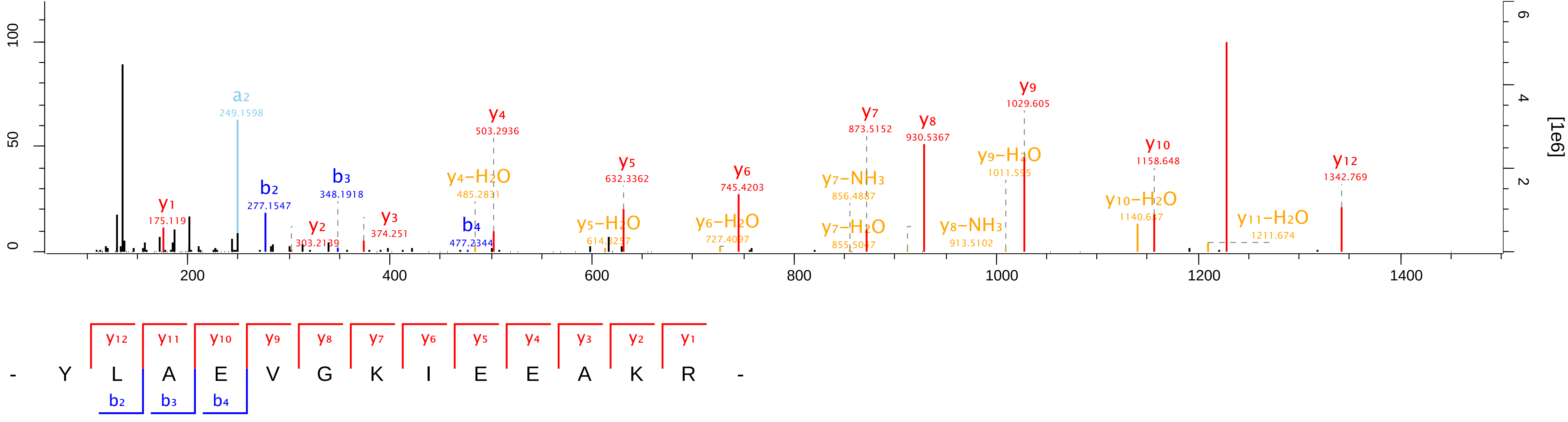

Raw file	Scan	Method	Score	m/z	Gene names
20140602_QEp4_FaHo_SA_RSC2_02	6702	FTMS; HCD	102.87	465.28	YOR1

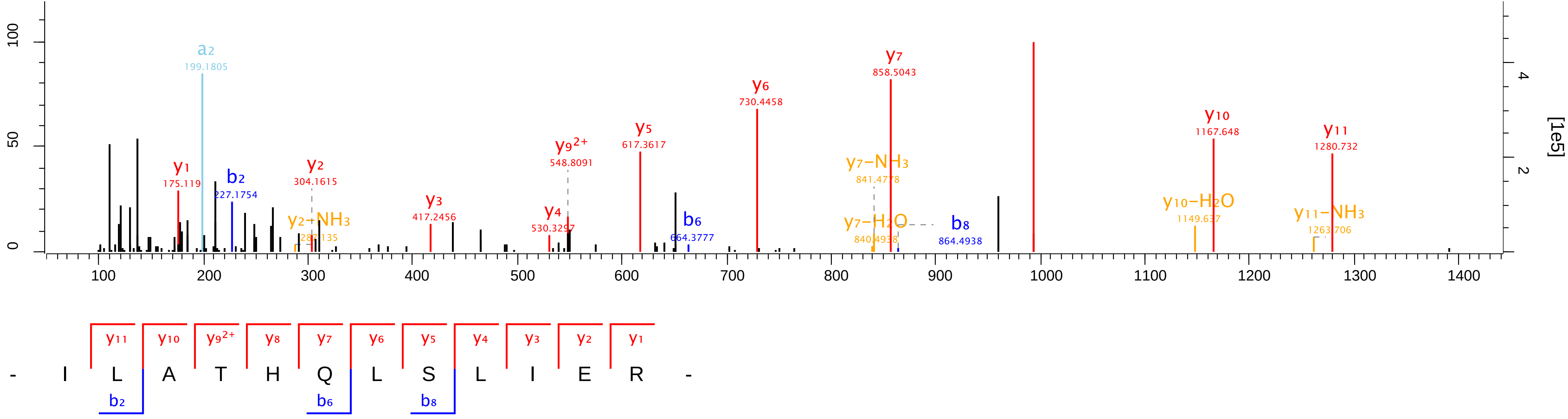

Raw file	Scan	Method	Score	m/z	Gene names
20140602_QEp4_FaHo_SA_RSC2_02	6784	FTMS; HCD	101.2	544.31	RPL4B;RPL4A

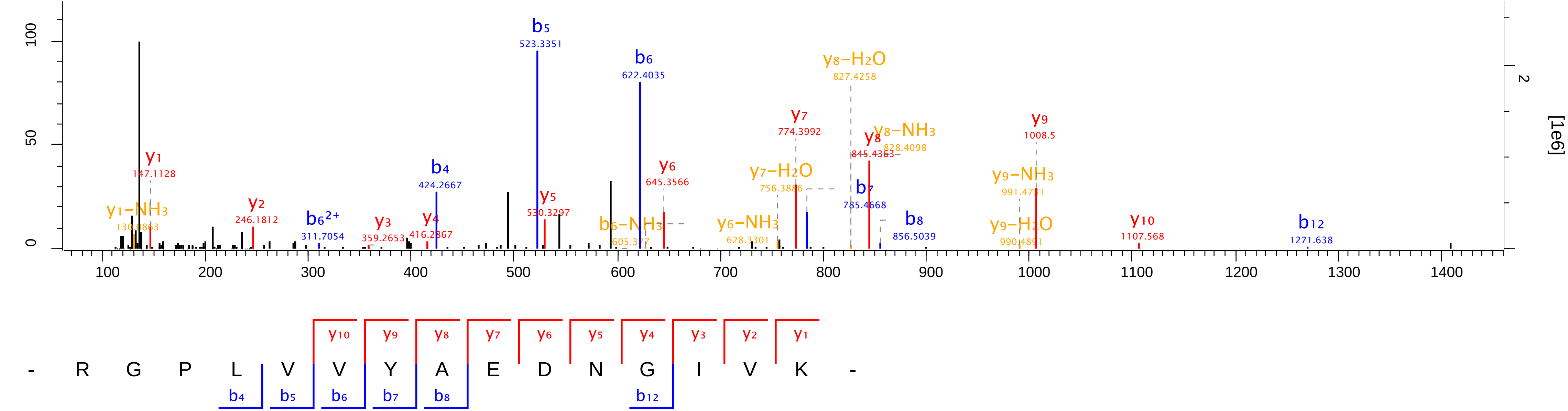

Raw file	Scan	Method	Score	m/z	Gene names
20140602_QEp4_FaHo_SA_RSC2_02	8436	FTMS; HCD	91.93	602.34	SAT4

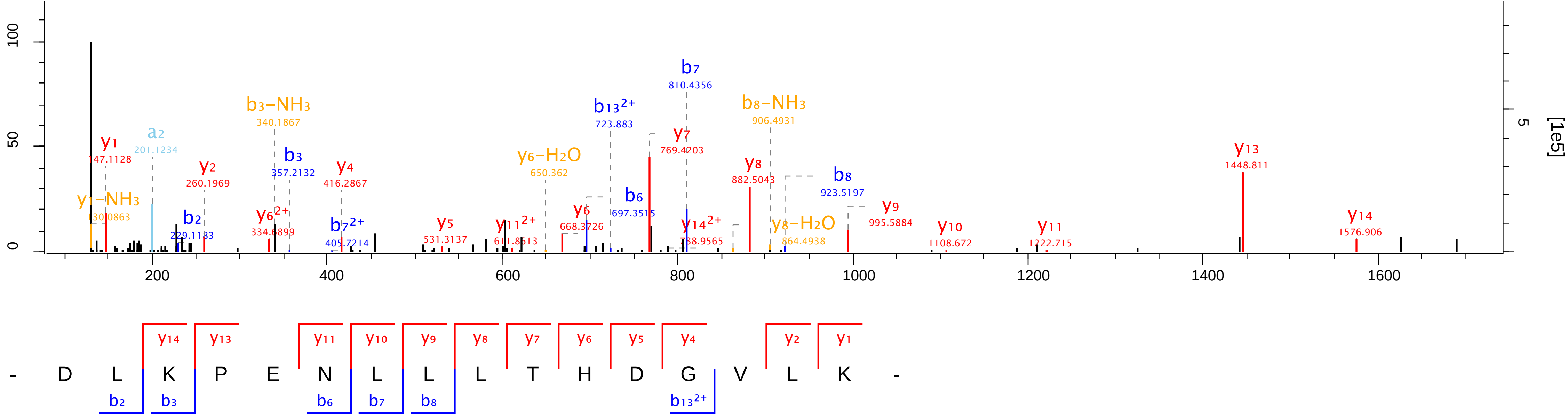

Raw file	Scan	Method	Score	m/z	Gene names
20140602_QEp4_FaHo_SA_RSC2_03	3050	FTMS; HCD	88.92	348.5	RPL31A

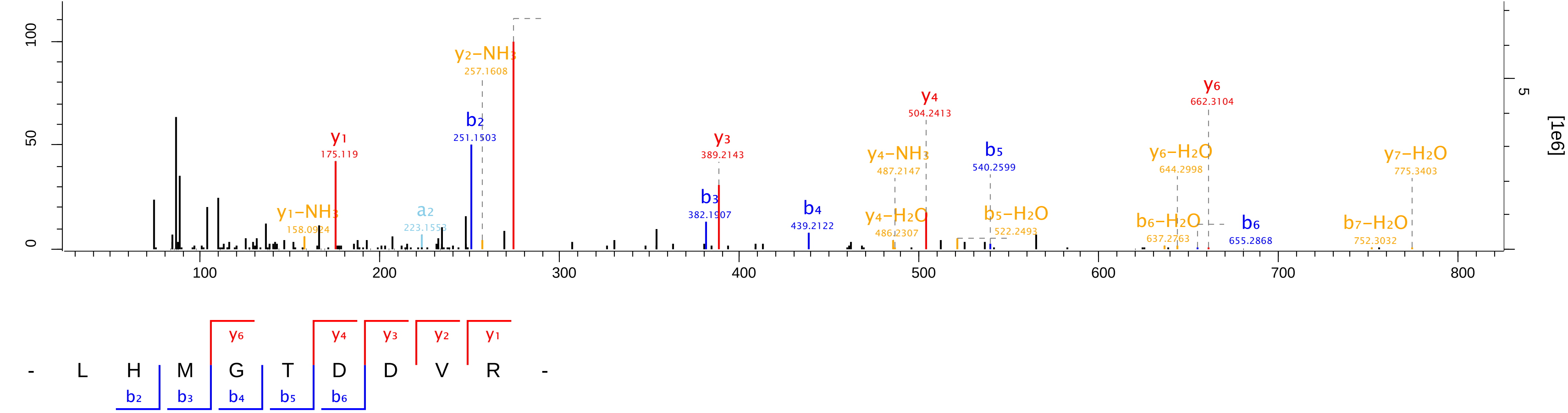

Raw file	Scan	Method	Score	m/z	Gene names
20140602_QEp4_FaHo_SA_RSC2_03	4541	FTMS; HCD	0.45	604.92	YME2

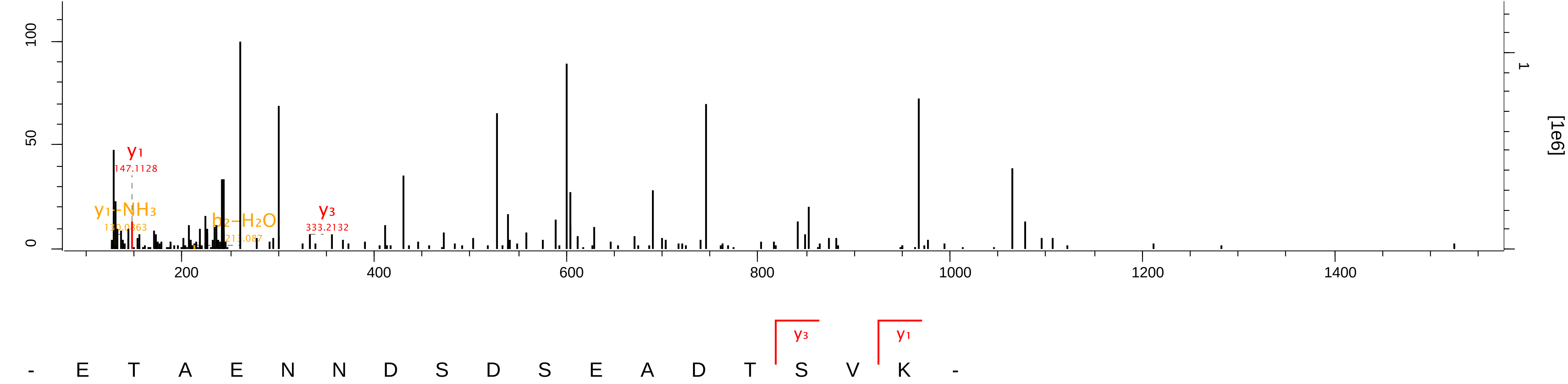

Raw file	Scan	Method	Score	m/z	Gene names
20140602_QEp4_FaHo_SA_RSC2_03	6503	FTMS; HCD	90.39	649.97	ATO3

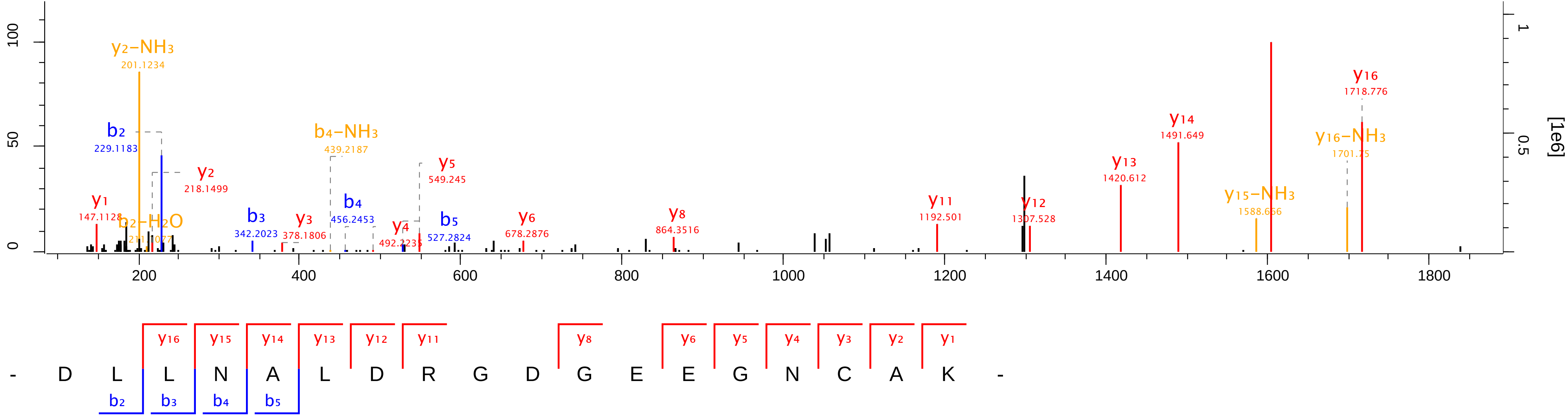

Raw file	Scan	Method	Score	m/z	Gene names
20140602_QEp4_FaHo_SA_RSC2_(	8010	FTMS;	136.57	906.44	TY2B-C;TY2B-GR2;TY2B-F;TY2B-GR1;TY2A-GR1;TY2B-OR1;TY2A-DR2;TY2B-DR1;TY2B-DR3;TY2B-LR1;TY2B-OR2;TY2A-OR1;TY2A-LR2;TY2A-OR2;TY2

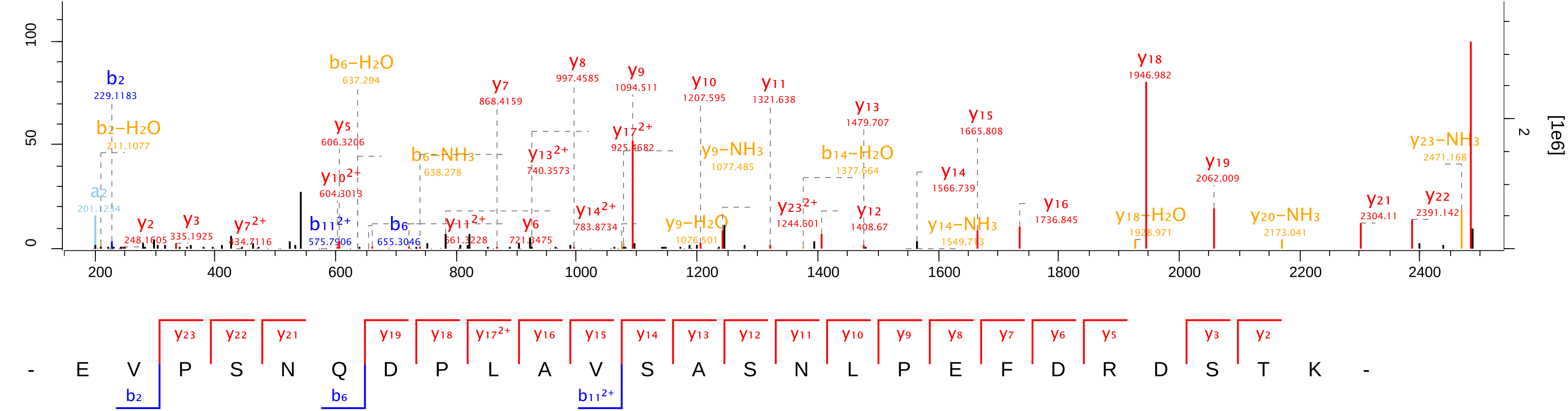

Raw file	Scan	Method	Score	m/z	Gene names
20140602_QEp4_FaHo_SA_RSC2_03	9025	FTMS; HCD	115.68	581.33	FRE1

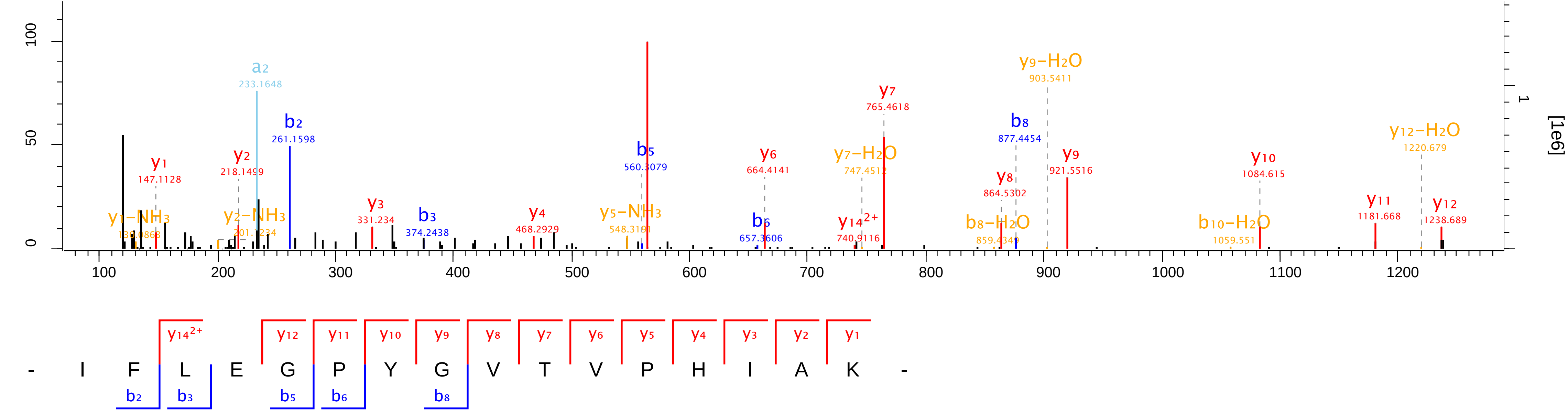

Raw file	Scan	Method	Score	m/z	Gene names
20140602_QEp4_FaHo_SA_RSC2_0	9414	FTMS; HCD	150.39	666.33	TY1B-ML1;TY1B-BR;TY1B-H;TY1B-MR2;TY1B-OR;TY1B-NL2;TY1B-PR2;TY1B-DR5;TY1B-PR1;TY1B-JR2;TY1B-NL1;TY1B-OL;TY1B-ML2;TY1B-PR3;TY1B-

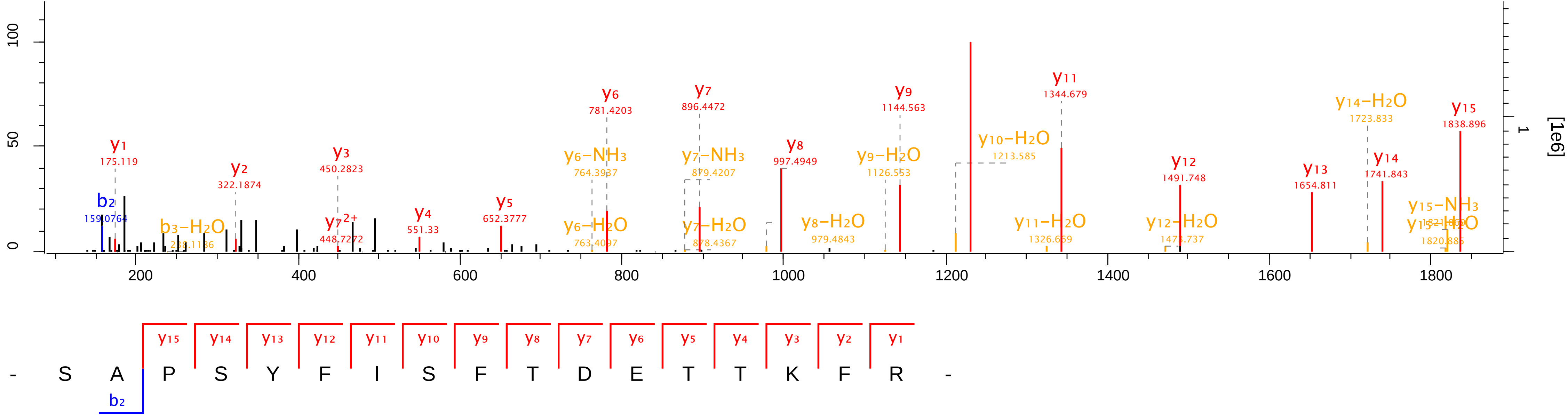

Raw file	Scan	Method	Score	m/z	Gene names
20140602_QEp4_FaHo_SA_RSC4_01	2251	FTMS; HCD	107.59	449.2	RPL7A;RPL7B

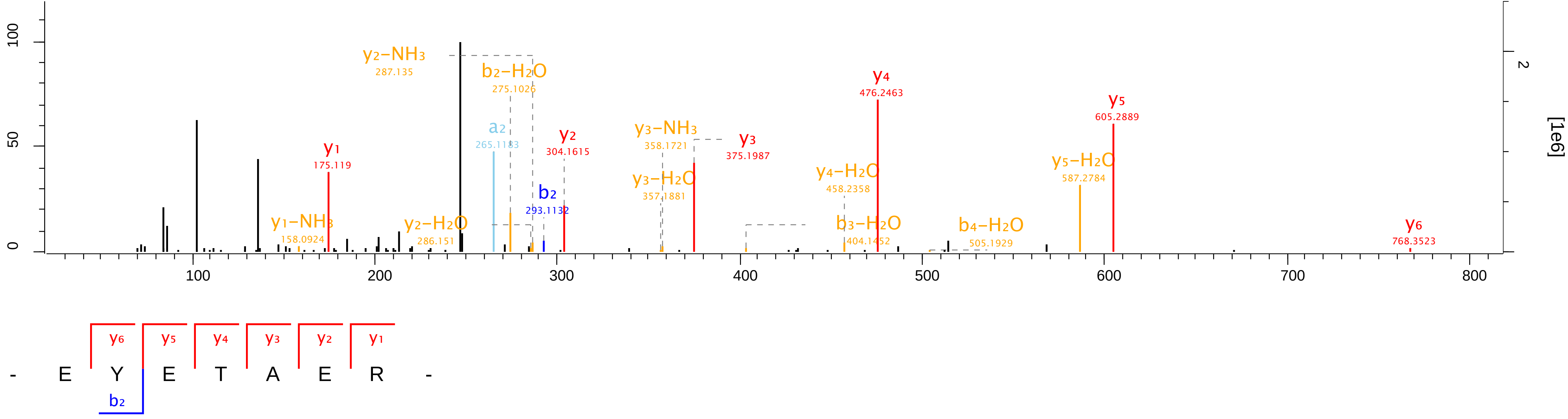

Raw file	Scan	Method	Score	m/z	Gene names
20140602_QEp4_FaHo_SA_RSC4_01	3289	FTMS; HCD	136.52	505.79	RPL4B;RPL4A

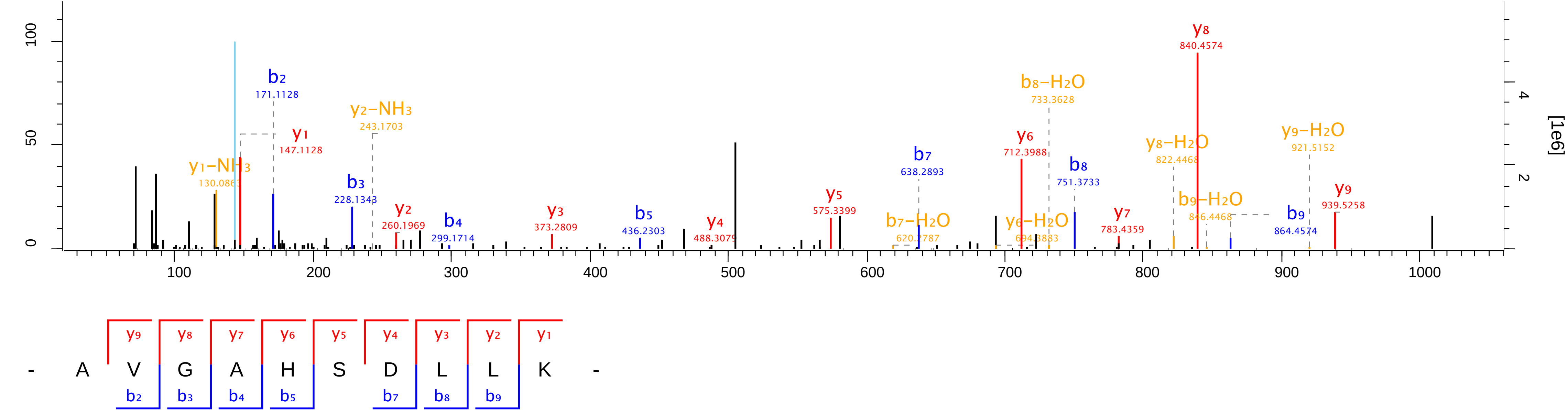

Raw file	Scan	Method	Score	m/z	Gene names
20140602_QEp4_FaHo_SA_RSC4_01	3708	FTMS; HCD	134.05	491.28	RPL13B;RPL13A

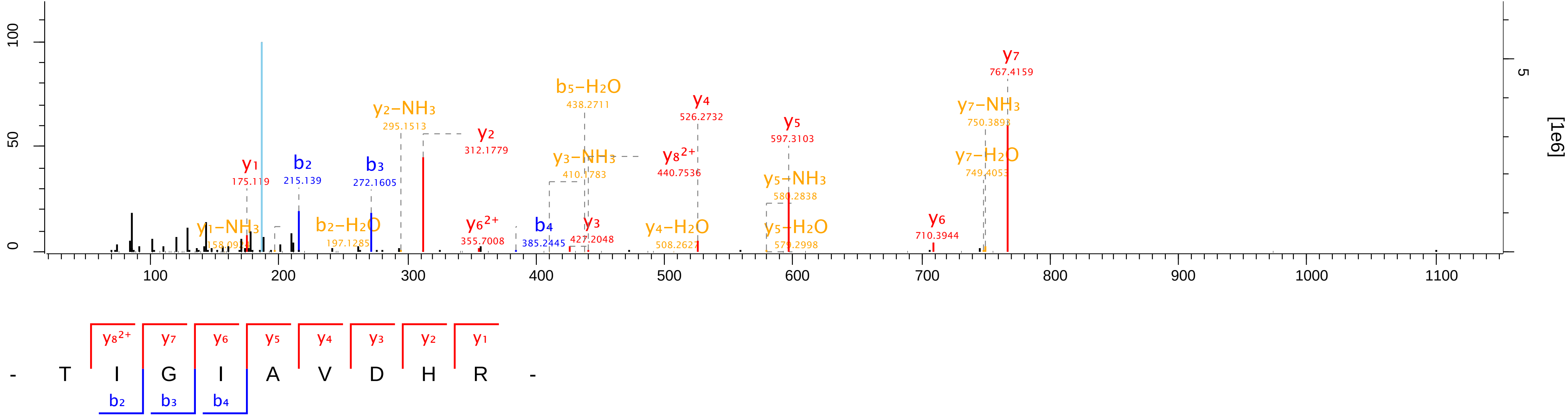

Raw file	Scai	Method	Score	m/z	Gene names
20140602_QEp4_FaHo_	40[illegible]	FTMS)[illegible]	1160.[illegible]	622.[illegible]	TY1B-ML1;TY1B-BR;TY1A-PR1;TY1A-A;TY1A-DR4;TY1B-H;TY1B-MR2;TY1B-OR;TY1B-DR1;TY1B-NL2;TY1B-PR2;TY1B-DR5;TY1B-PR1;TY1B-JR2;TY1A-PL;TY1A-LR2;TY1A-[illegible]

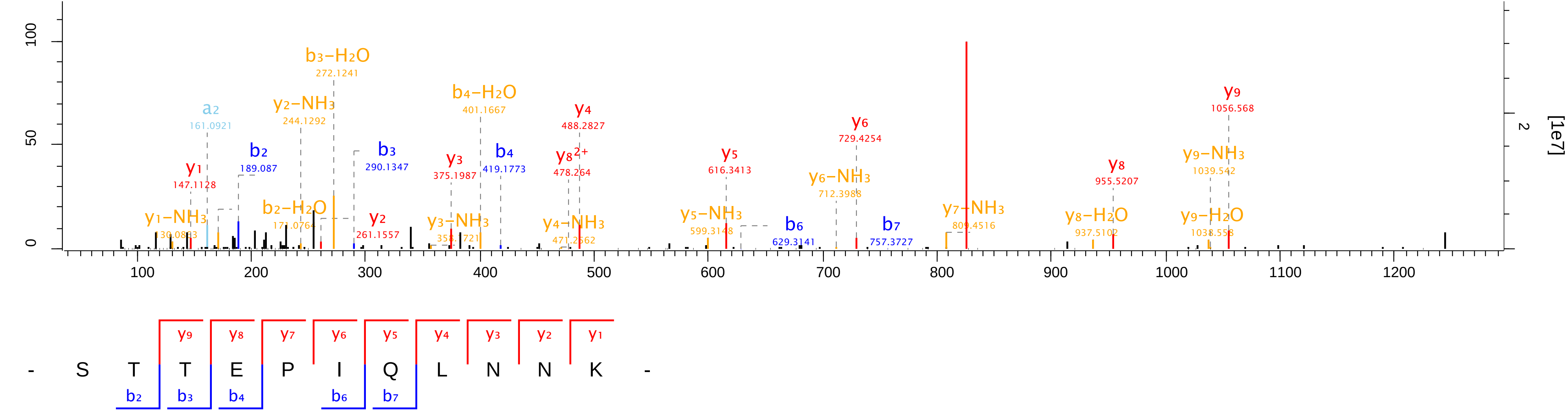

Raw file	Scan	Method	Score	m/z	Gene names
20140602_QEp4_FaHo_SA_RSC4_01	5057	FTMS; HCD	189.51	803.71	TY1A-DR2;TY1B-BL;TY1B-MR1;TY1A-MR1

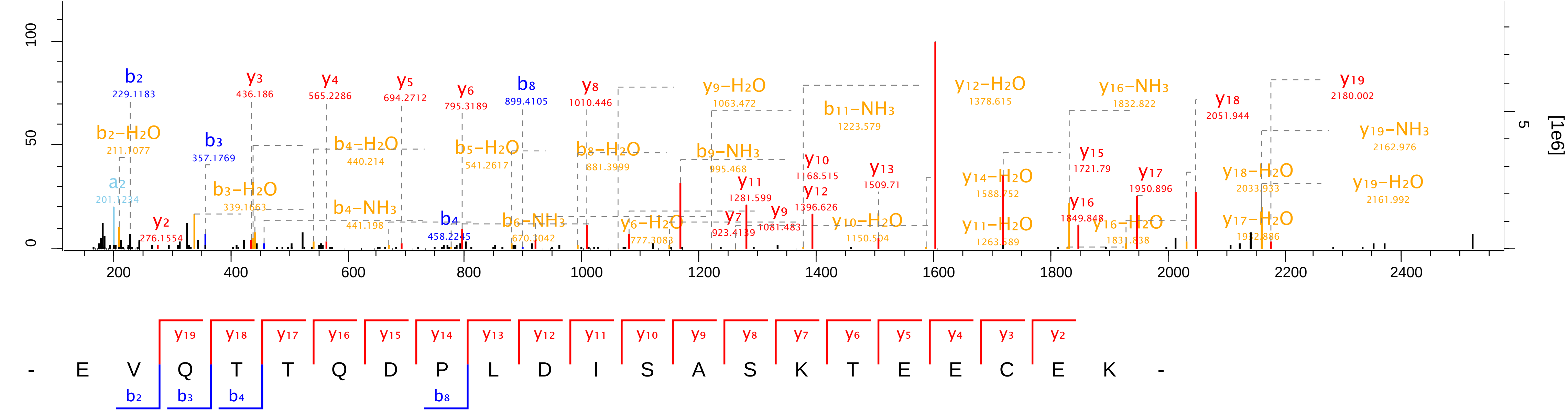

Raw file	Scan	Method	Score	m/z	Gene names
20140602_QEp4_FaHo_SA_RSC4_01	5705	FTMS; HCD	160.81	816.91	TY1A-DR2;TY1B-BL;TY1B-MR1;TY1A-MR1

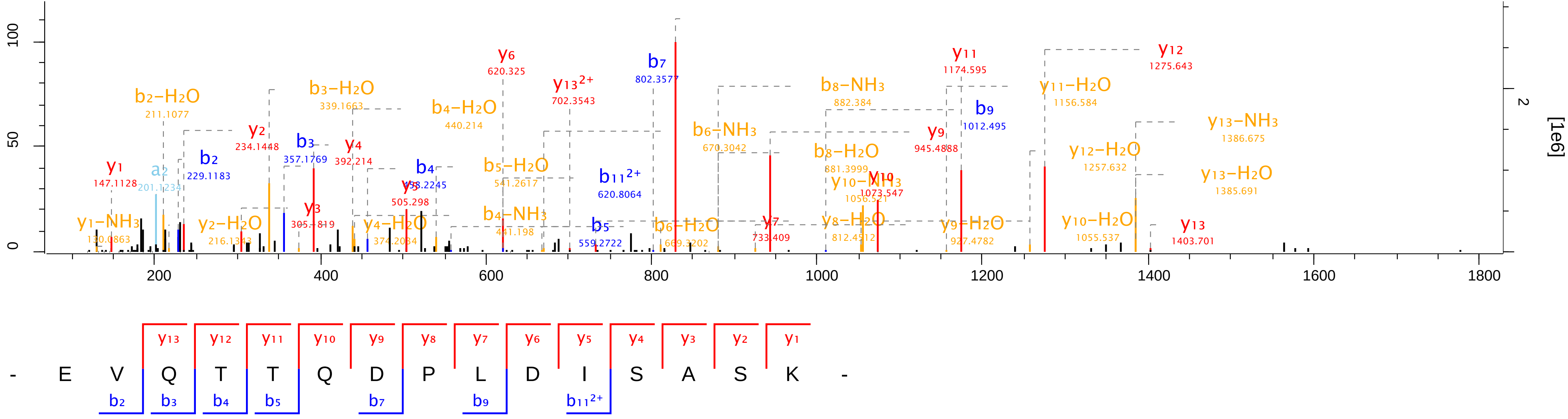

Raw file	Scan	Method	Score	m/z	Gene names
20140602_QEp4_FaHo_SA_RSC4_01	8772	FTMS; HCD	109.14	808.94	ILV1

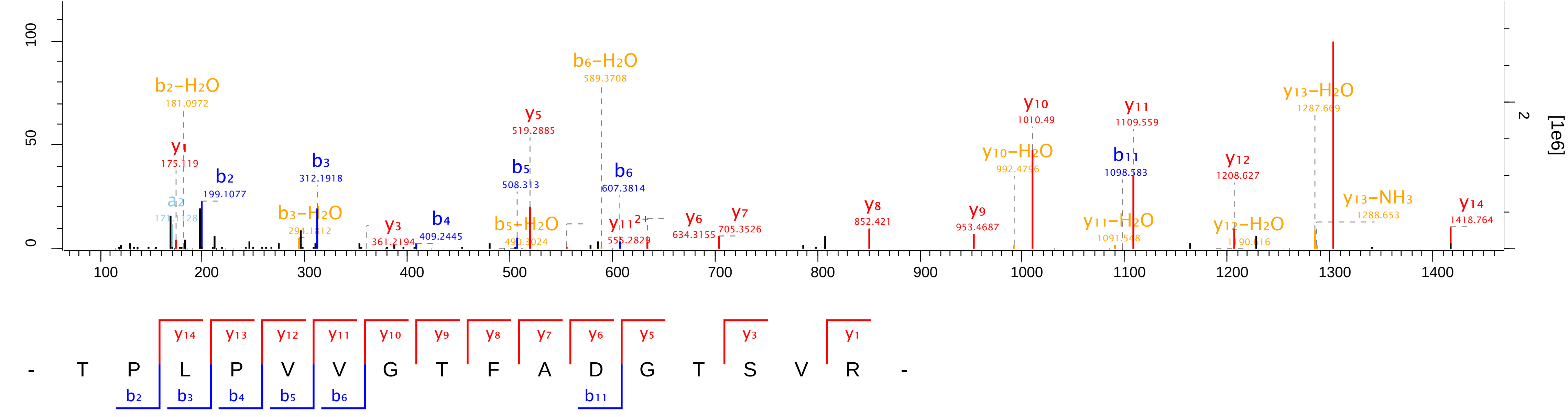

Raw file	Scan	Method	Score	m/z	Gene names
20140602_QEp4_FaHo_SA_RSC4_(	8929	FTMS;	153.2	847.4	TY1B-ML1;TY1B-BR;TY1B-H;TY1B-MR2;TY1B-OR;TY1B-NL2;TY1B-PR2;TY1B-DR5;TY1B-PR1;TY1B-JR2;TY1B-NL1;TY1B-OL;TY1B-ML2;TY1B-PR3;TY1B-BL;

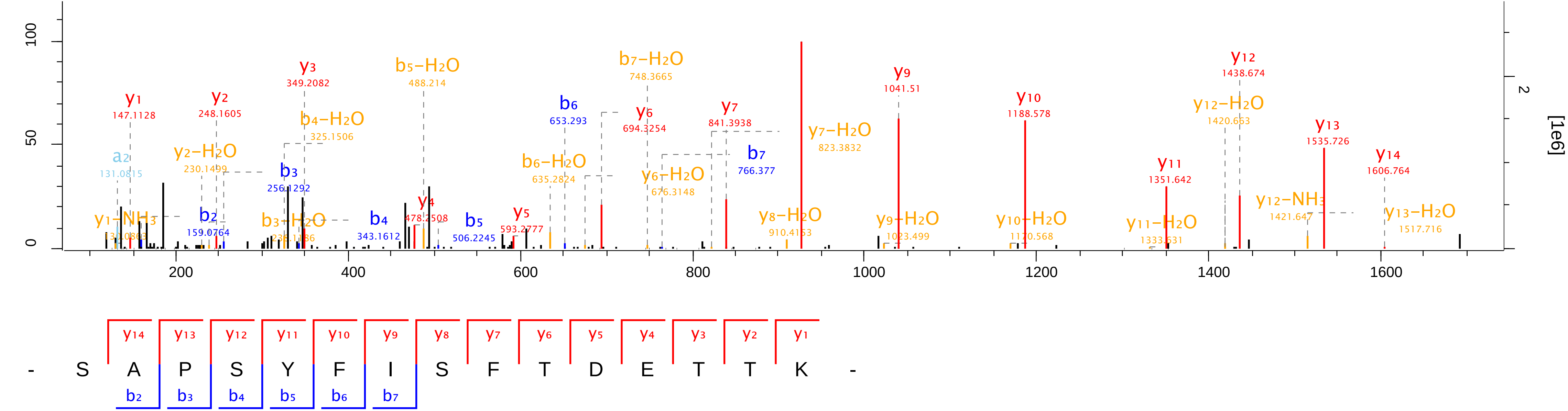

Raw file	Scan	Method	Score	m/z	Gene names
20140602_QEp4_FaHo_SA_RSC4_01	9096	FTMS; HCD	3.06	786.41	UME6

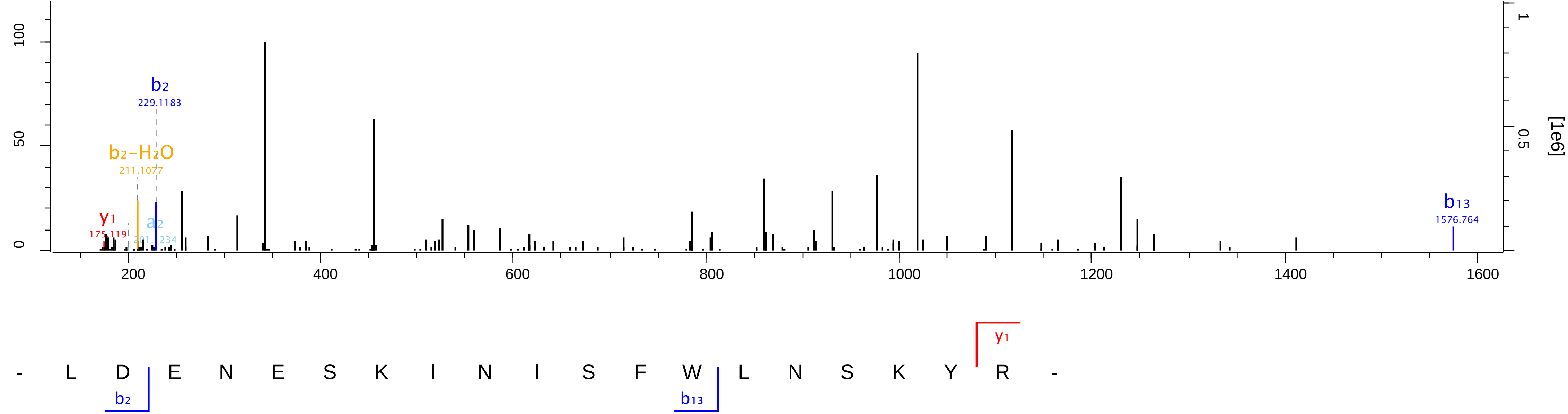

Raw file	Scan	Method	Score	m/z	Gene names
20140602_QEp4_FaHo_SA_RSC4_0	3219	FTMS; HCD	164.05	698.56	TY1B-ML1;TY1B-MR2;TY1B-OR;TY1B-DR1;TY1B-PR2;TY1B-DR5;TY1B-PR1;TY1B-JR2;TY1B-OL;TY1B-LR4;TY1B-ML2;TY1B-DR3;TY1B-PL;TY1B-LR2;TY1

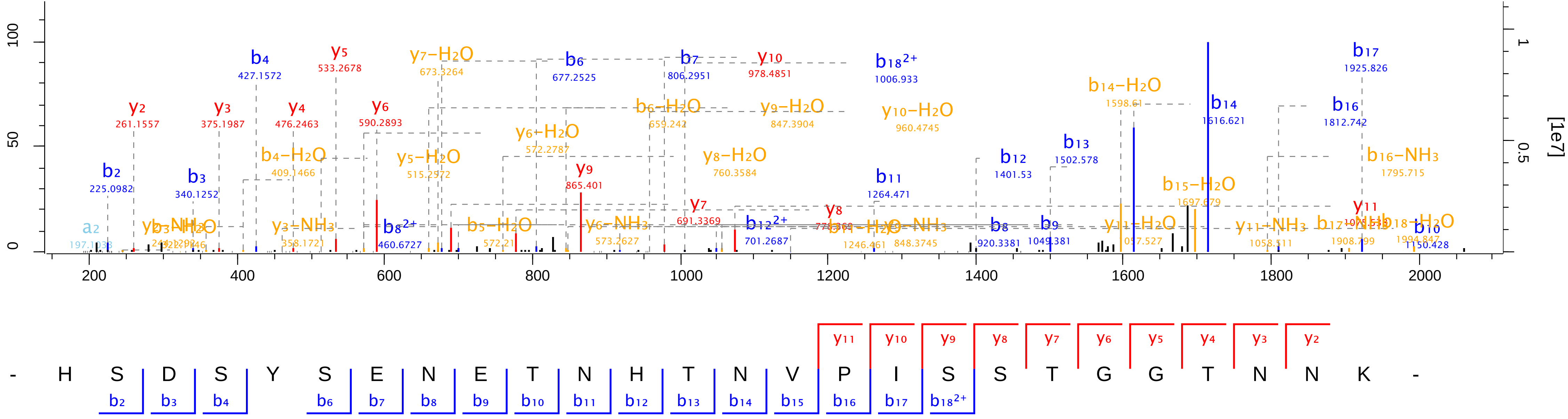

Raw file	Scan	Method	Score	m/z	Gene names
20140602_QEp4_FaHo_SA_RSC4_02	7587	FTMS; HCD	157.68	650.69	TIF6

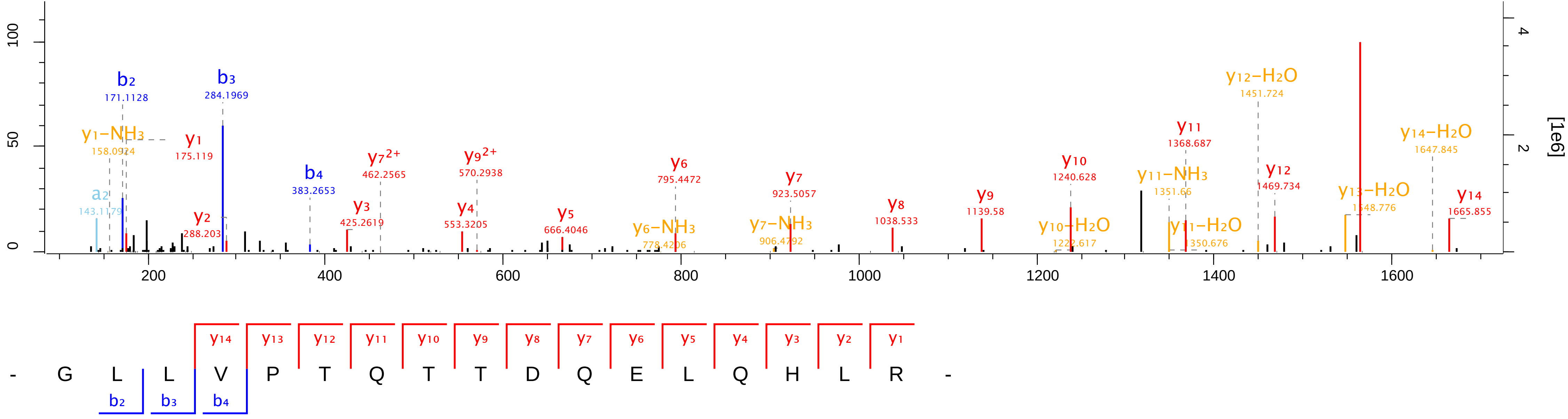

Raw file	Scan	Method	Score	m/z	Gene names
20140602_QEp4_FaHo_SA_RSC4_02	8241	FTMS; HCD	209.6	832.41	RPP1B

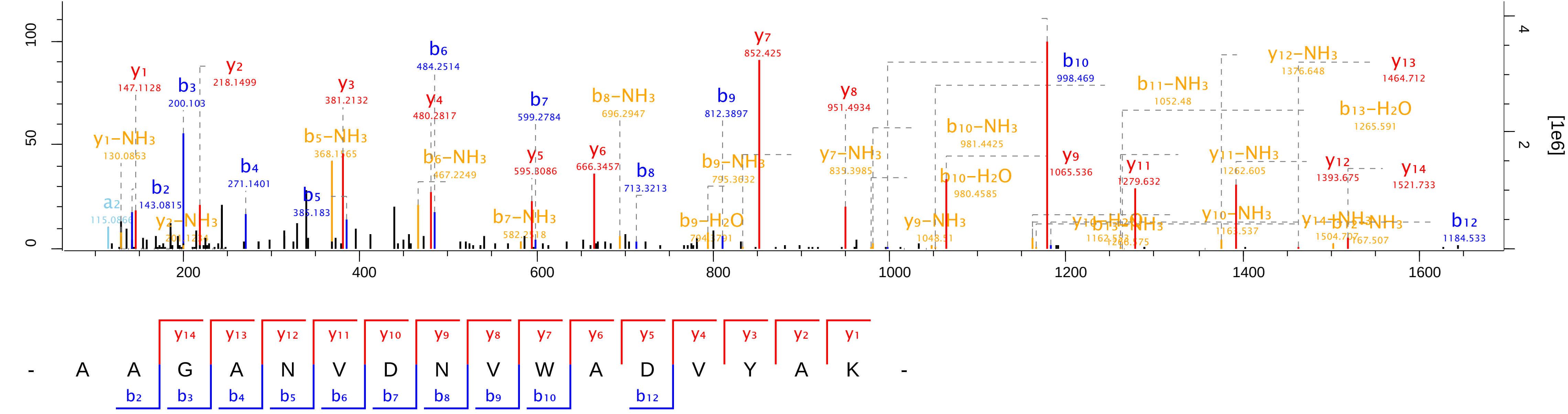

Raw file	Scan	Method	Score	m/z	Gene names
20140602_QEp4_FaHo_SA_RSC4_02	9555	FTMS; HCD	64.86	857.73	SEC13

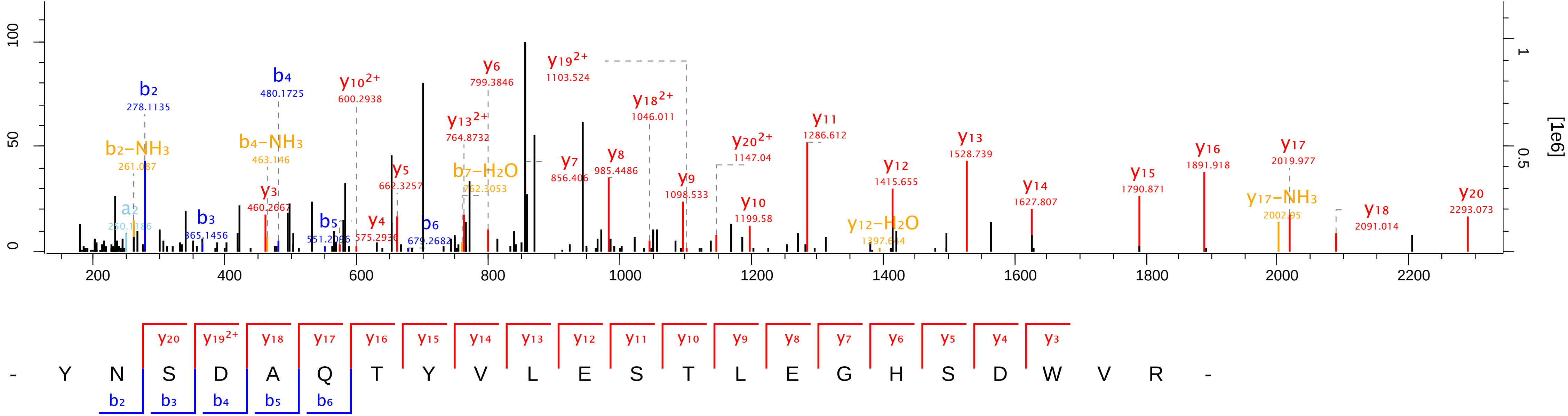

Raw file	Scan	Method	Score	m/z	Gene names
20140602_QEp4_FaHo_SA_RSC4_02	9910	FTMS; HCD	108.7	550.34	MNP1

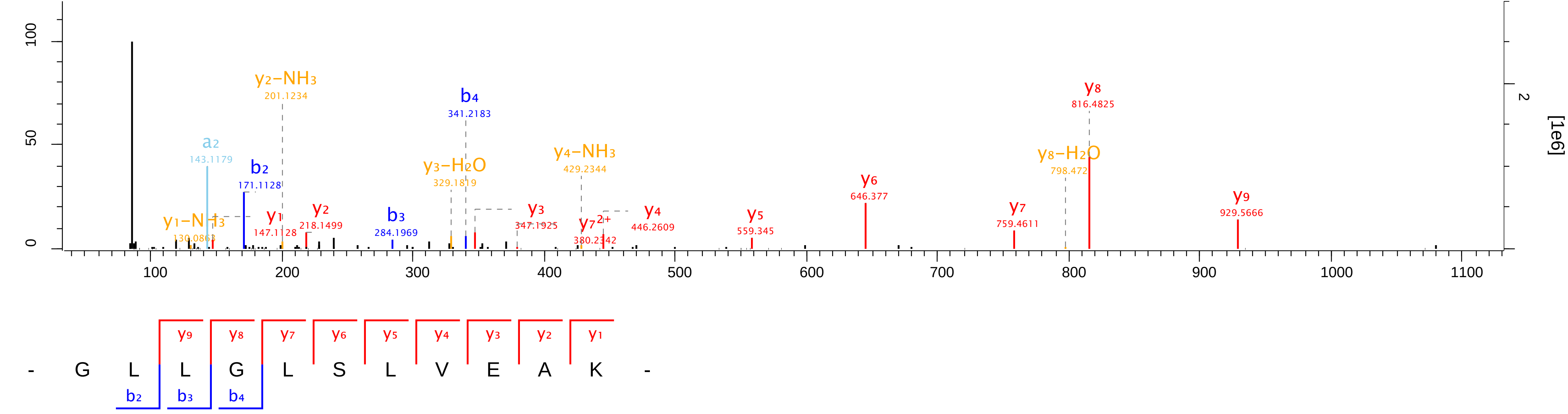

Raw file	Scan	Method	Score	m/z	Gene names
20140602_QEp4_FaHo_SA_RSC4_03	7110	FTMS; HCD	102.64	784.44	MIC26

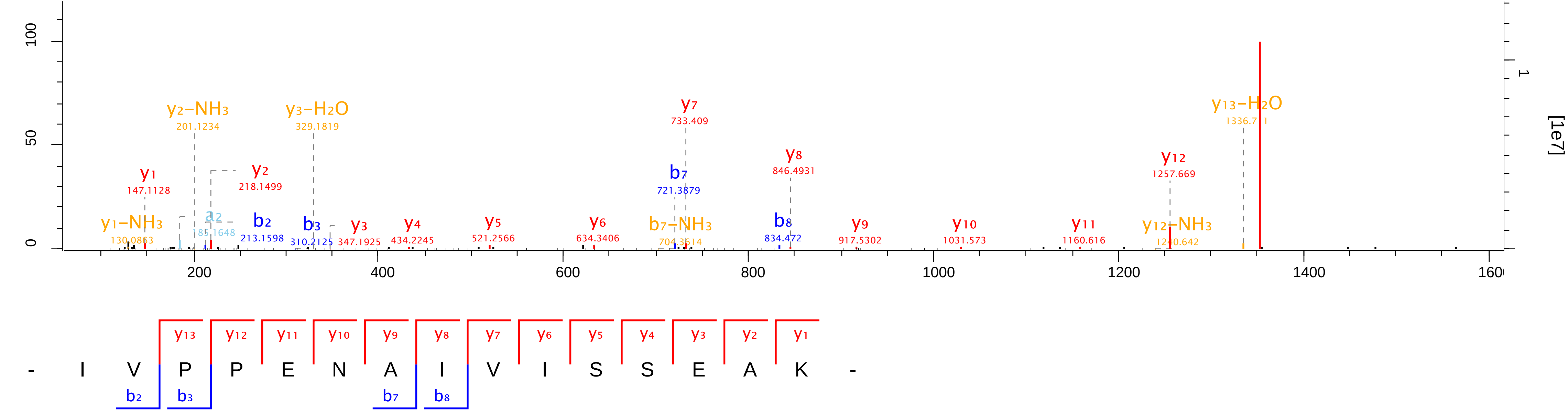

Raw file	Sca	Metho	Sco	m/z	Gene names
20140602_QEp4_Fal	79!	FTMS(	74(!	480.	TY1B-ML1;TY1B-BR;TY1B-H;TY1B-MR2;TY1B-OR;TY1B-DR1;TY1B-NL2;TY1B-PR2;TY1B-DR5;TY1B-PR1;TY1B-JR2;TY1B-NL1;TY2B-C;TY1B-OL;TY1B-LR4;TY1B-ML2;TY1B-DR3;TY1

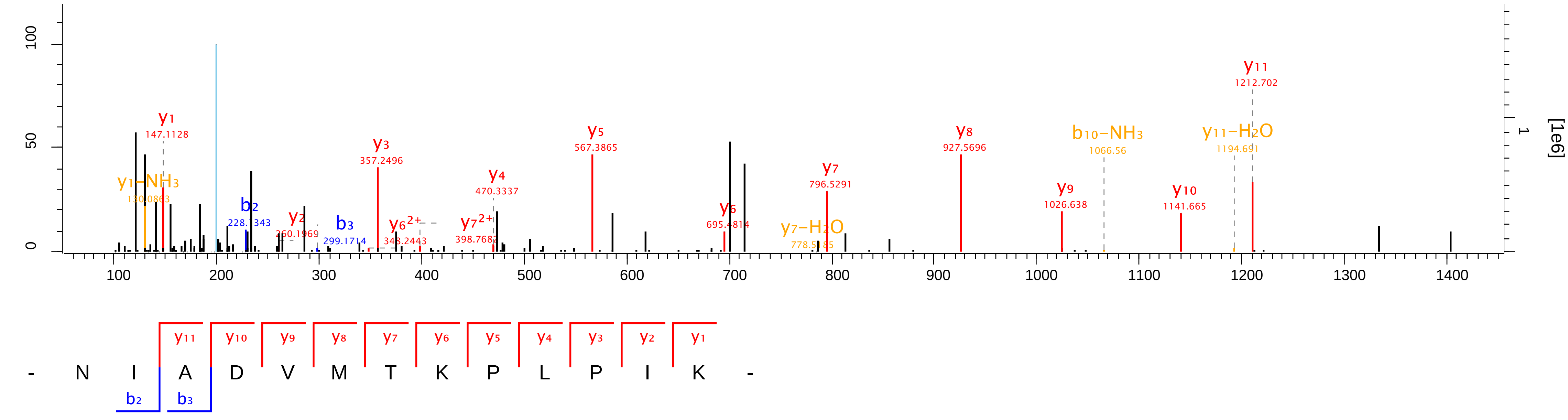

Raw file	Scan	Method	Score	m/z	Gene names
20140602_QEp4_FaHo_SA_RSC4_03	9400	FTMS; HCD	117	632.67	MRPL9

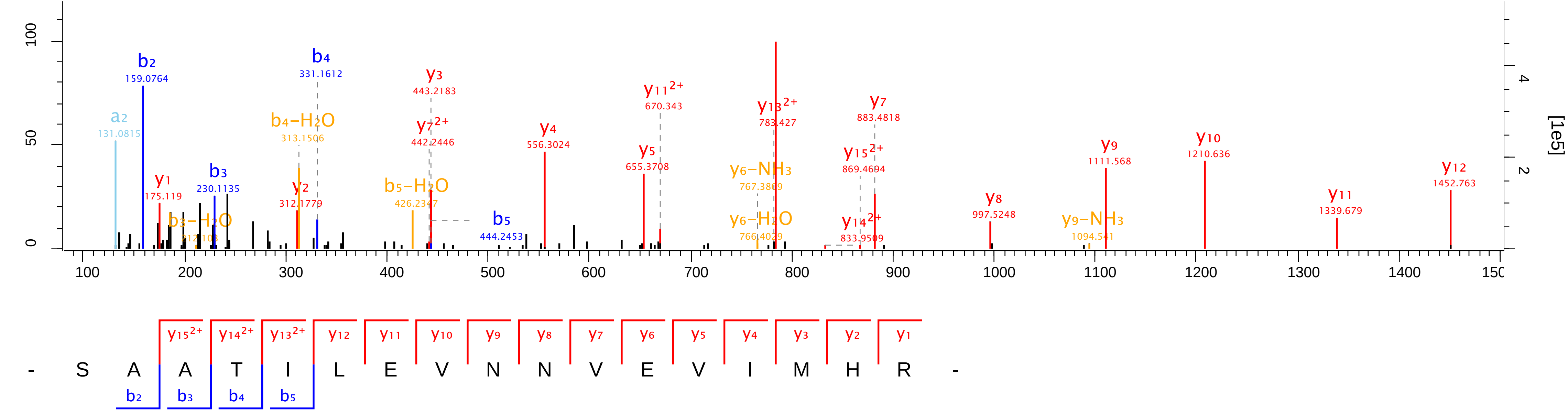

Raw file	Scar	Method	Score	m/z	Gene names
20140602_QEp4_FaHo_SA_	434	FTMS;	128.2	778.8	TY1B-ML1;TY1B-BR;TY1B-H;TY1B-MR2;TY1B-OR;TY1B-DR1;TY1B-NL2;TY1B-PR2;TY1B-DR5;TY1B-PR1;TY1B-JR2;TY1B-NL1;TY1B-OL;TY1B-LR4;TY1B-ML2;TY1B-DR3;

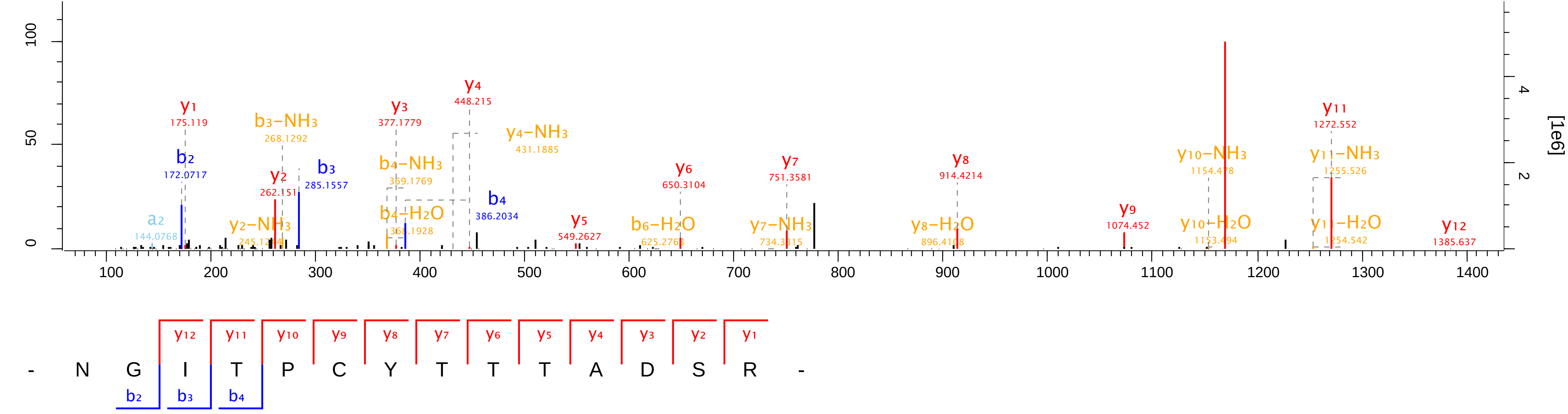

Raw file	Scan	Method	Score	m/z	Gene names
20140602_QEp4_FaHo_SA_RSC8_01	5438	FTMS; HCD	111.86	550.31	DBP2

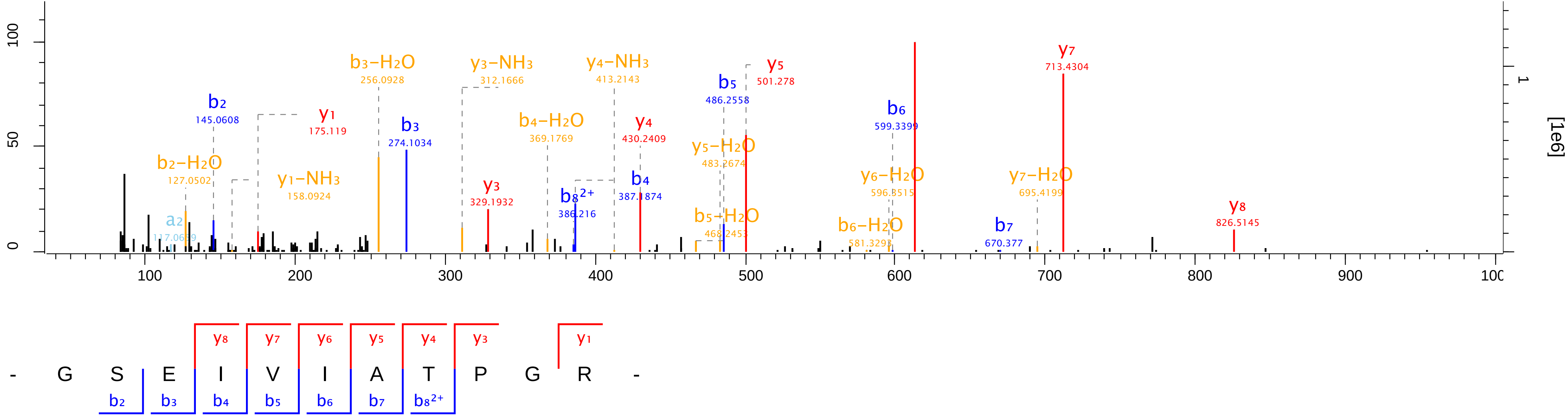

Raw file	Scan	Method	Score	m/z	Gene names
20140602_QEp4_FaHo_SA_RSC8_01	5917	FTMS; HCD	106.04	457.26	VTC1

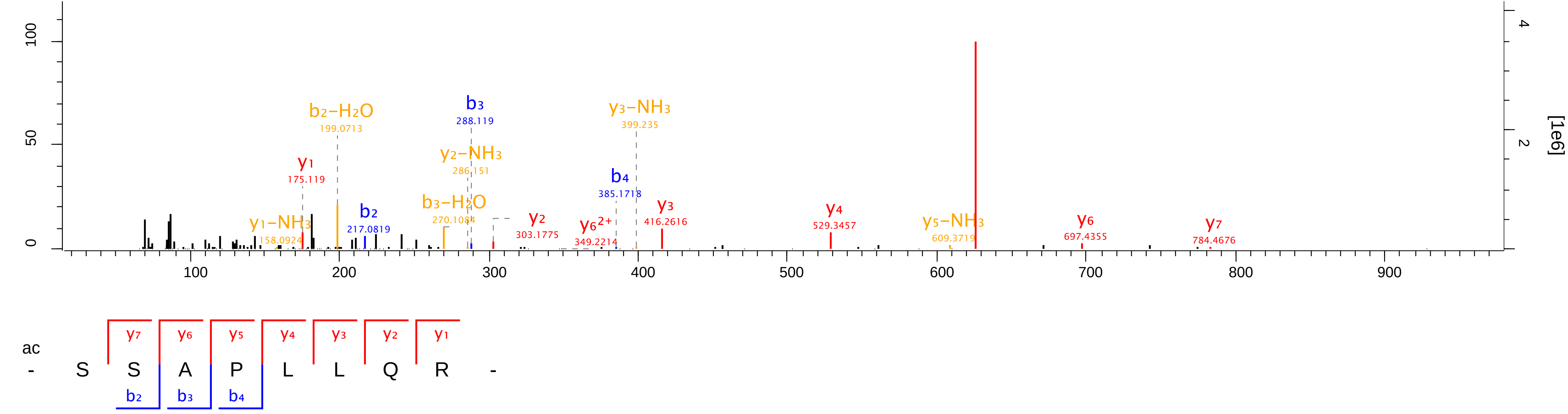

Raw file	Scan	Method	Score	m/z	Gene names
20140602_QEp4_FaHo_SA_RSC8_01	6809	FTMS; HCD	113.65	826.38	ACP1

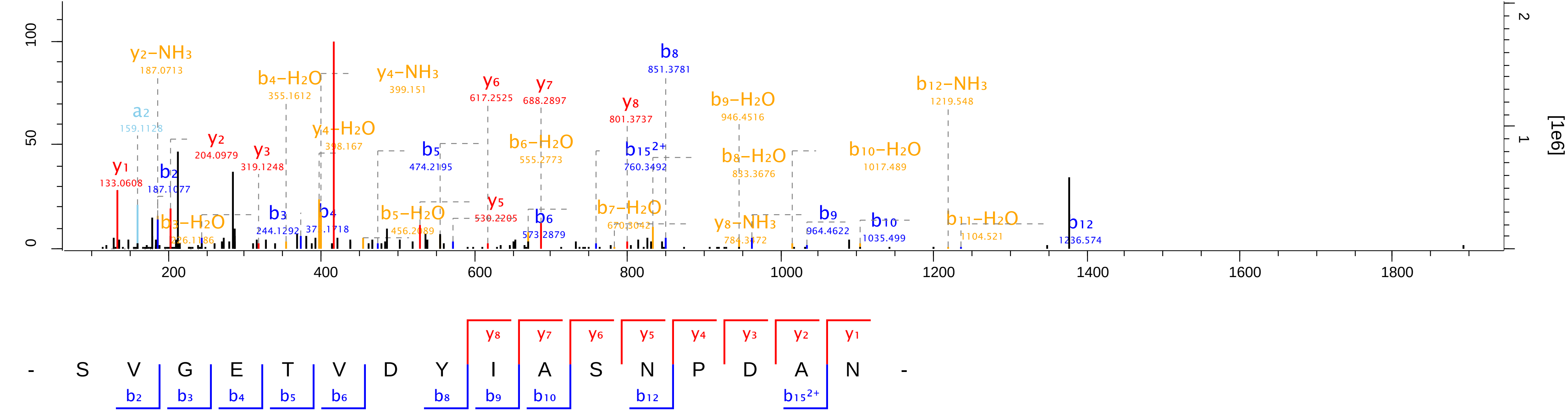

Raw file	Scan	Method	Score	m/z	Gene names
20140602_QEp4_FaHo_SA_RSC8_01	7530	FTMS; HCD	66.11	628.33	TY2B-C;TY2B-B;TY2B-GR1;TY2A-GR1;TY2B-OR1;TY2A-DR2;TY2B-DR1;TY2B-DR3;TY2B-LR1;TY2B-OR2;TY2A-OR1;TY2A-LR2;TY2A-OR2;TY2A-B

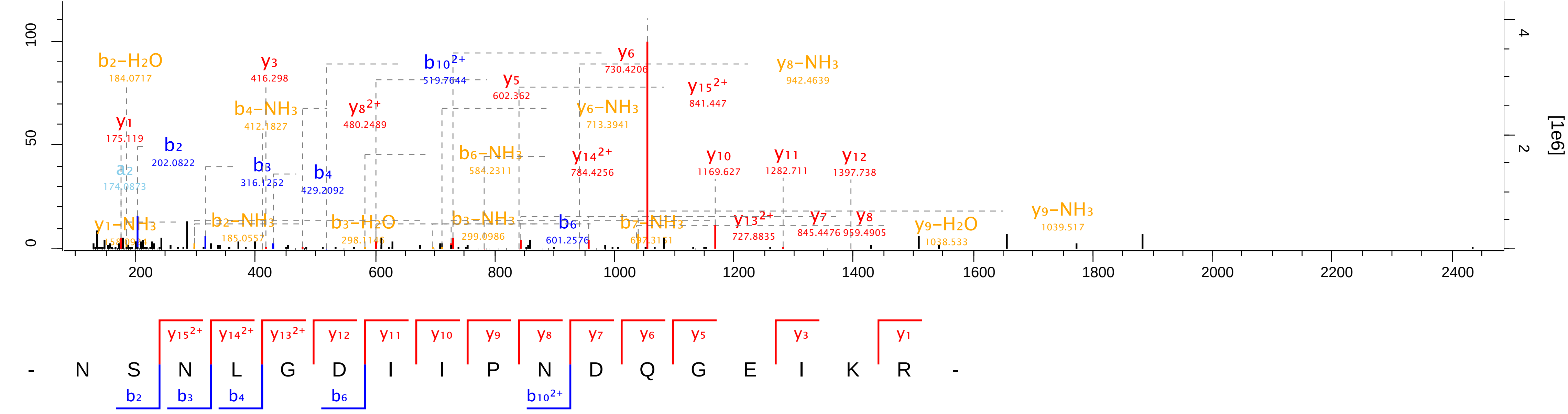

Raw file	Scan	Method	Score	m/z	Gene names
20140602_QEp4_FaHo_SA_RSC8_01	9641	FTMS; HCD	195.92	923.99	RPL4B;RPL4A

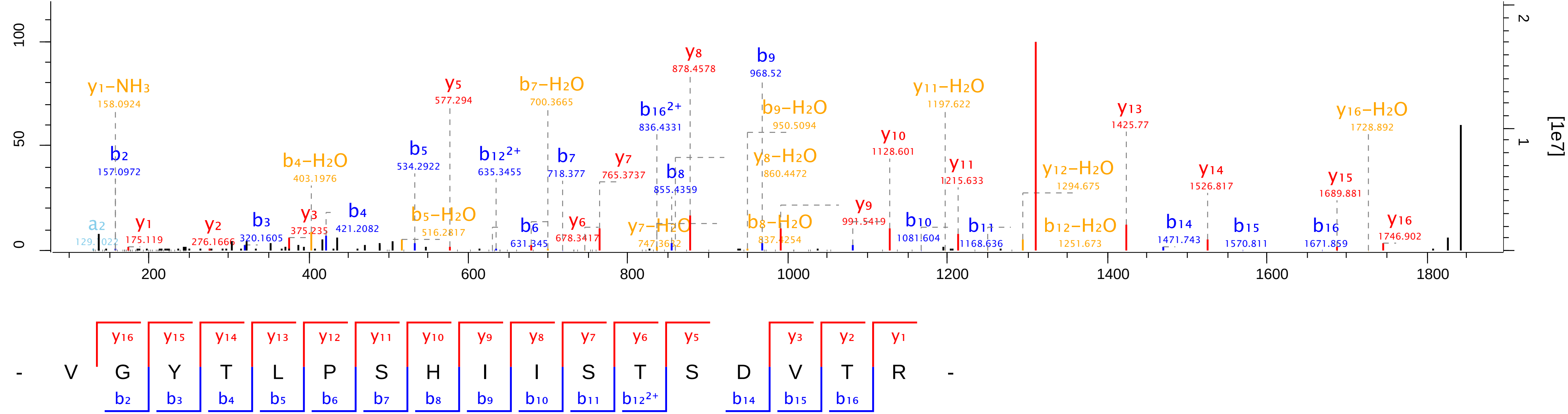

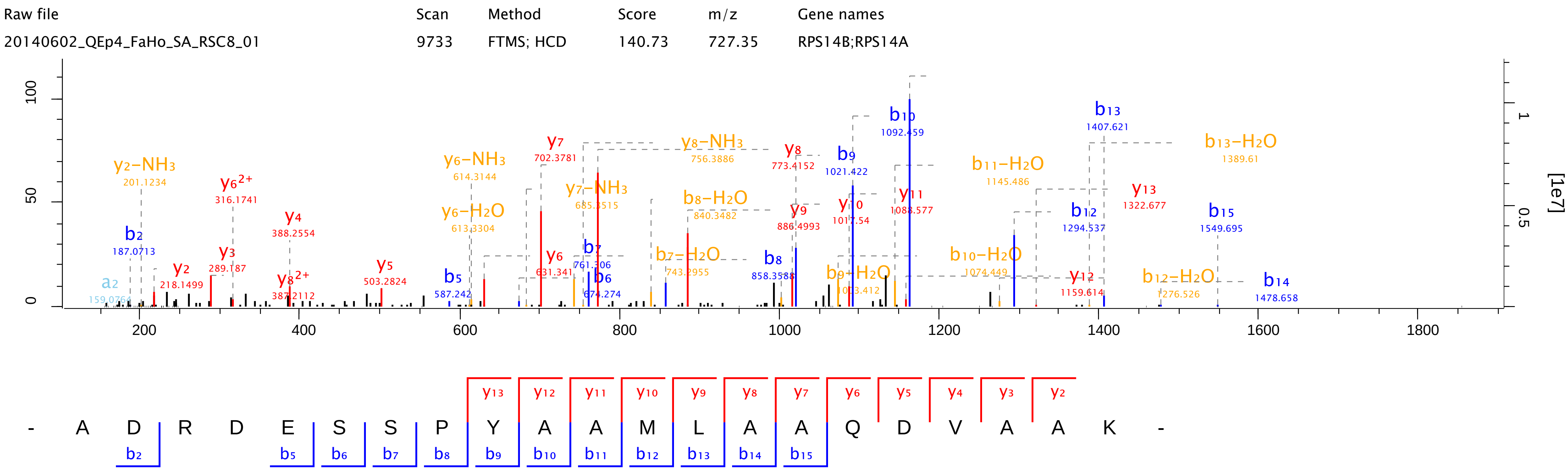

Raw file
20140602_QEp4_FaHo_SA_RSC8_01
Scan
9733
Method
FTMS; HCD
Score
140.73
m/z
727.35
Gene names
RPS14B;RPS14A
[1e7]
a2 159.0764
b2 187.0713
y2−NH3 201.1234
y2 218.1499
y3 289.187
y6 2+ 316.1741
y8 2+ 387.2112
y4 388.2554
y5 503.2824
b5 587.242
y6−H2O 613.3304
y6−NH3 614.3144
y6 631.341
b6 674.274
y7−NH3 685.3515
y7 702.3781
b7 761.306
y8−NH3 756.3886
b7−H2O 743.2955
b8−H2O 840.3482
b8 858.3588
y8 773.4152
y9 886.4993
b9 1021.422
b9−H2O 1003.412
y10 1017.54
y11 1088.577
b10 1092.459
b10−H2O 1074.449
b11−H2O 1145.486
y12 1159.614
b12 1294.537
b12−H2O 1276.526
y13 1322.677
b13 1407.621
b13−H2O 1389.61
b14 1478.658
b15 1549.695
- A D R D E S S P Y A A M L A A Q D V A A K -

Raw file	Scan	Method	Score	m/z	Gene names
20140602_QEp4_FaHo_SA_RSC8_01	10067	FTMS; HCD	299.56	901.14	RPL17A;RPL17B

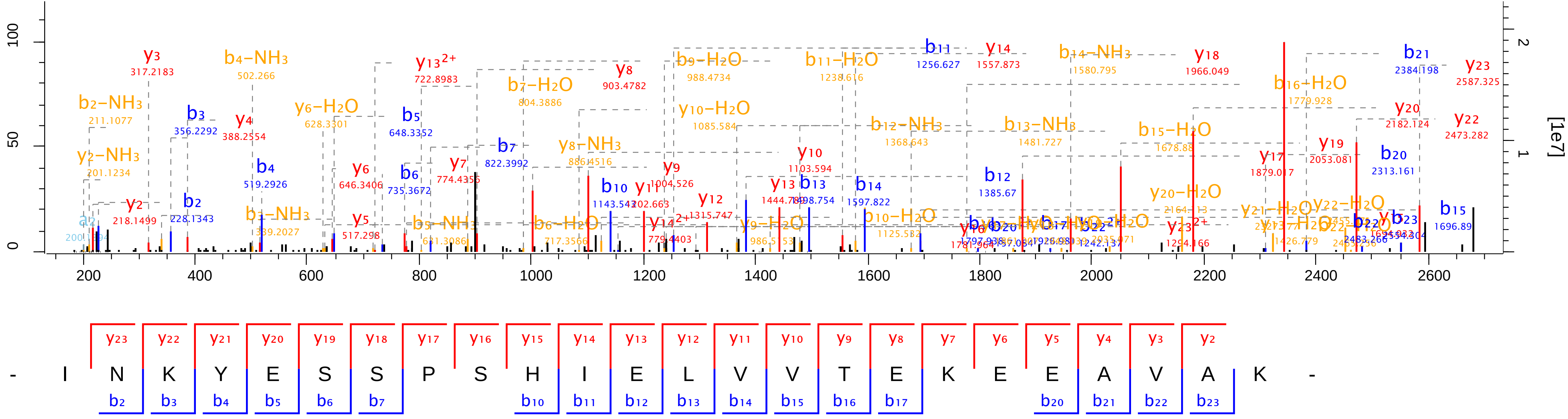

Raw file	Scan	Method	Score	m/z	Gene names
20140602_QEp4_FaHo_SA_RSC8_01	10079	FTMS; HCD	107.73	645.01	RFA3

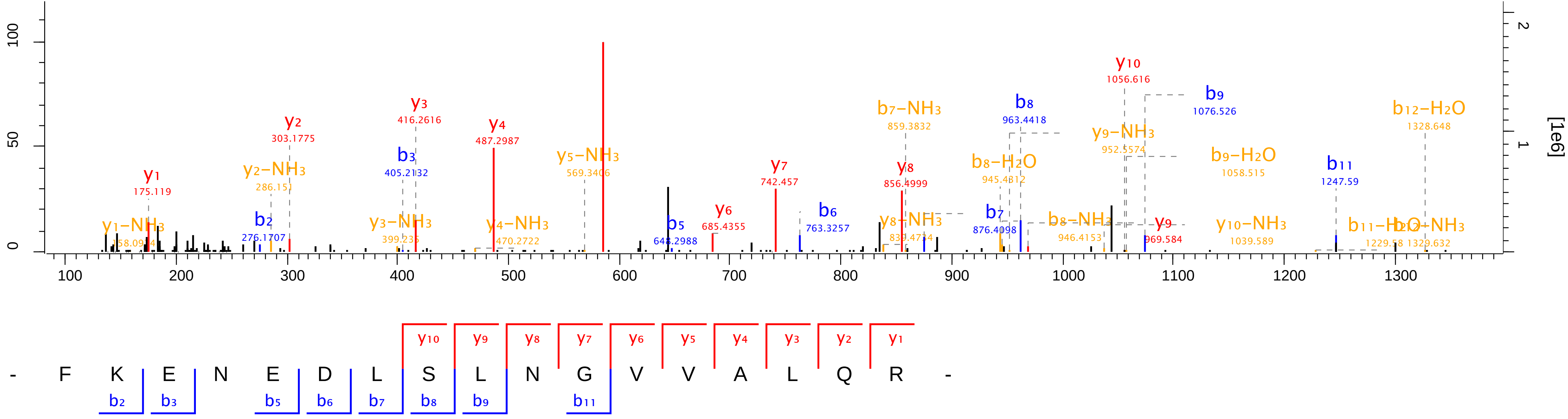

Raw file	Scan	Method	Score	m/z	Gene names
20140602_QEp4_FaHo_SA_RSC8_02	177	FTMS; HCD	135.84	423.21	TY1B-ML1;TY1B-BR;TY1A-PR1;TY1A-A;TY1A-DR4;TY1B-H;TY1B-MR2;TY1B-OR;TY1B-DR1;TY1B-NL2;TY1B-PR2;TY1B-DR5;TY1B-PR1;TY1B-JR2;TY1

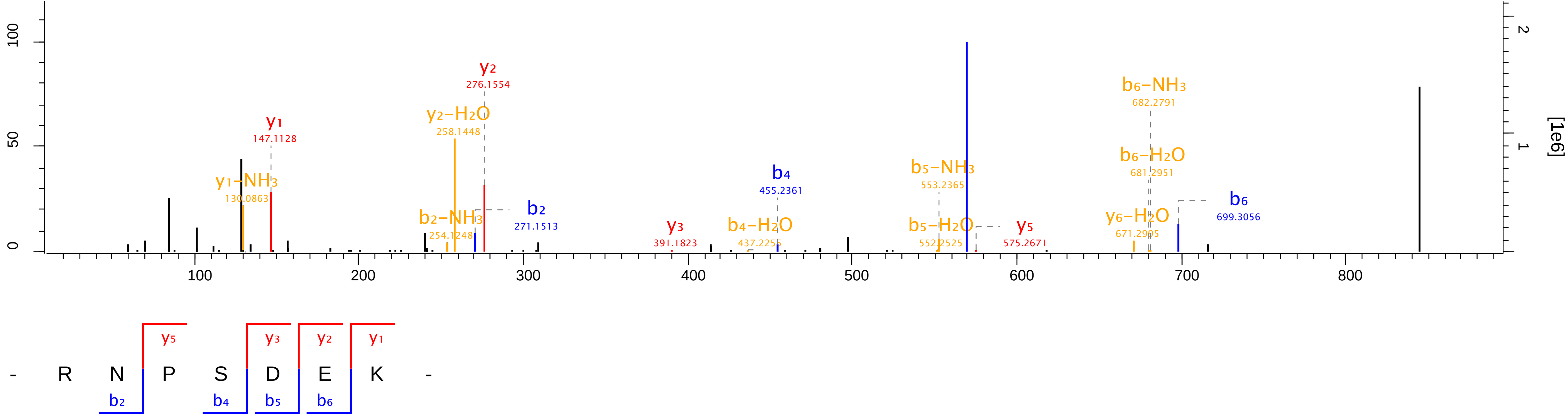

Raw file	Scan	Method	Score	m/z	Gene names
20140602_QEp4_FaHo_SA_RSC8_02	3398	FTMS; HCD	74.44	351.21	RPL4B;RPL4A

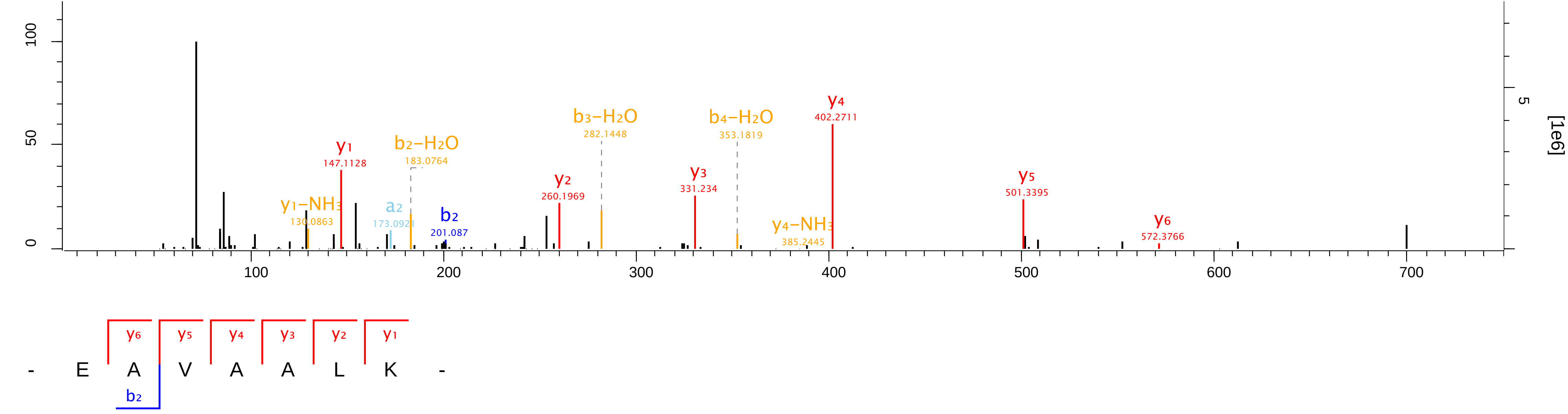

Raw file	Scan	Method	Score	m/z	Gene names
20140602_QEp4_FaHo_SA_RSC8_(	6206	FTMS;	402.03	867.4	TY1A-PR1;TY1A-A;TY1A-DR4;TY1B-NL2;TY1B-PR1;TY1B-NL1;TY1A-PL;TY1A-LR2;TY1A-ER1;TY1A-DR6;TY1B-PL;TY1B-LR2;TY1B-DR6;TY1A-DR2;TY1B-[illegible]

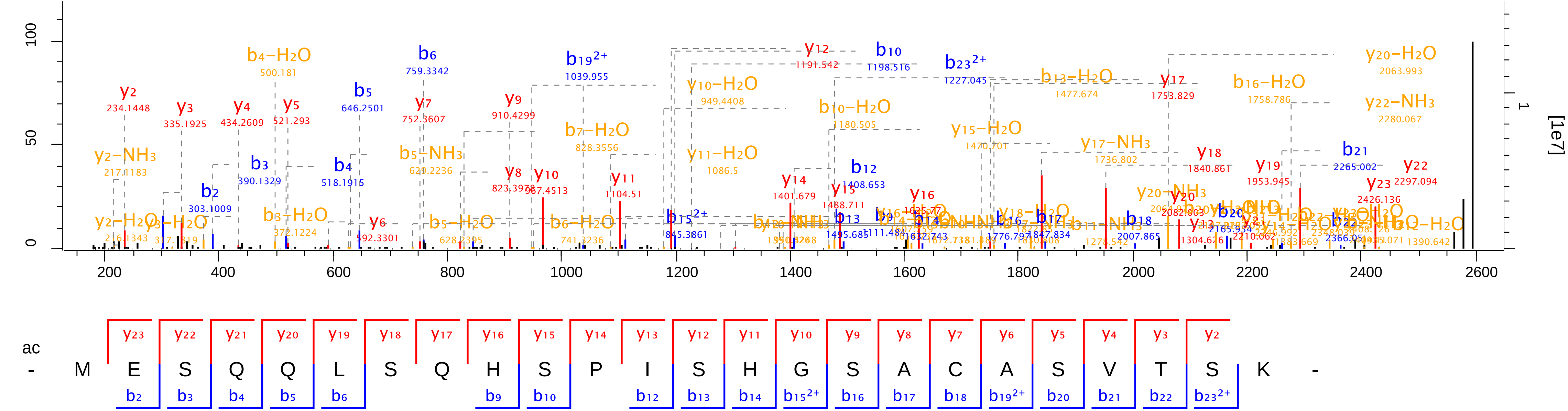

Raw file	Scan	Method	Score	m/z	Gene names
20140602_QEp4_FaHo_SA_RSC8_03	7920	FTMS; HCD	182.02	808.74	RPS14B;RPS14A

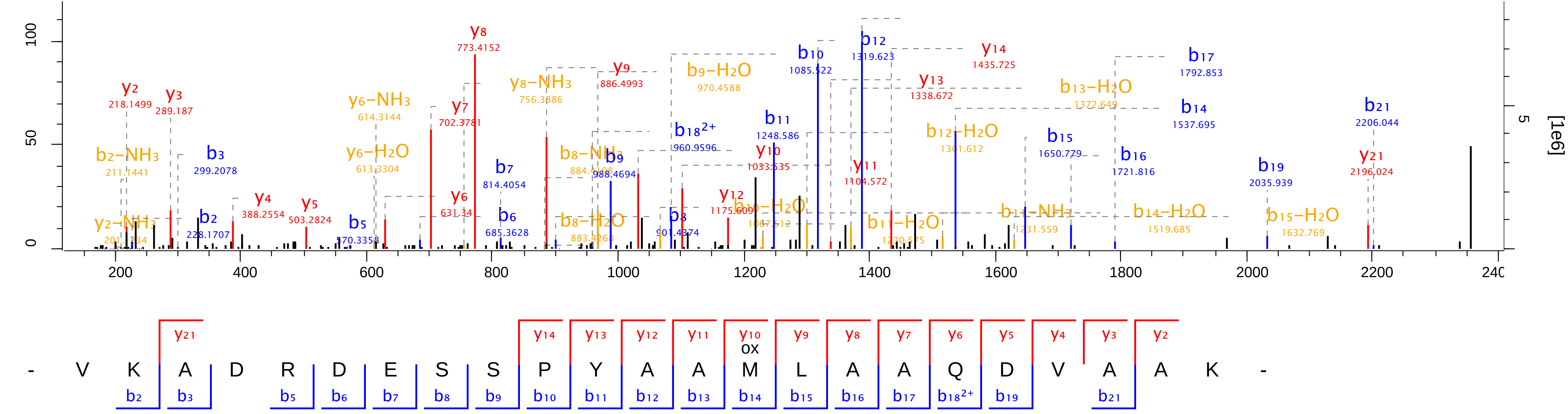

Raw file	Scan	Method	Score	m/z	Gene names
20140602_QEp4_FaHo_SA_RSC8_03	9291	FTMS; HCD	94.2	422.48	RPL4B

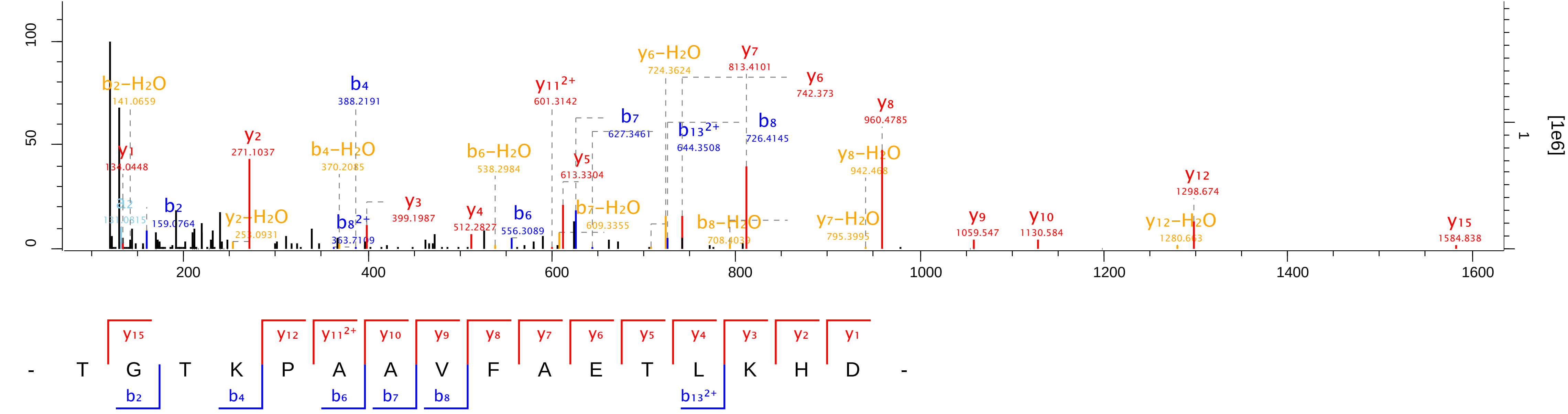

Raw file	Scan	Method	Score	m/z	Gene names
20140602_QEp4_FaHo_SA_RSC8_03	9334	FTMS; HCD	120.86	436.24	RPL9A;RPL9B

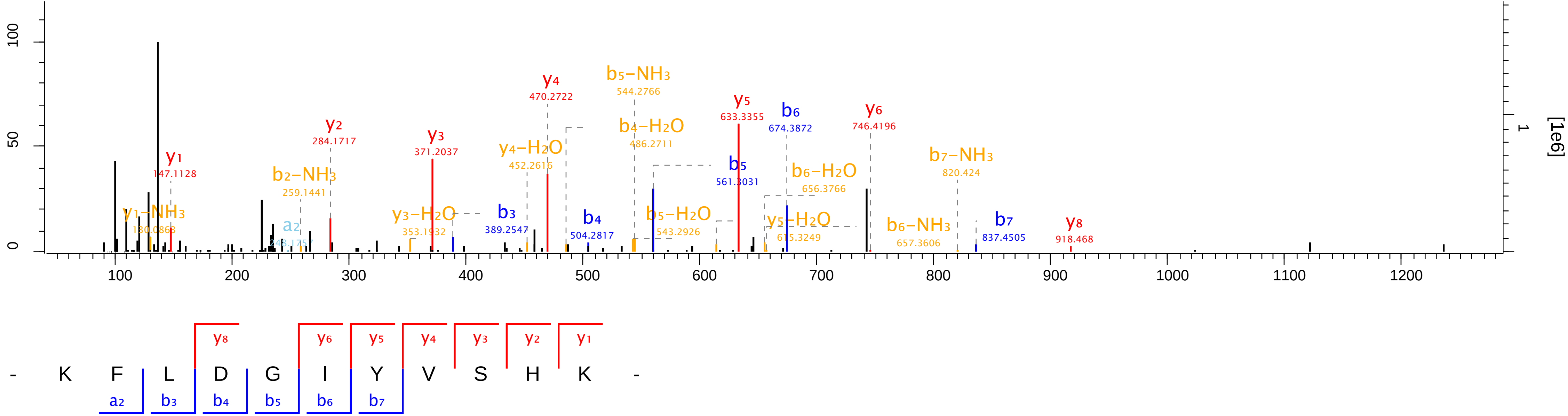

Raw file	Scan	Method	Score	m/z	Gene names
20140602_QEp4_FaHo_SA_RSC8_0	9694	FTMS; HCD	131.56	592.05	TY1B-ML1;TY1B-MR2;TY1B-OR;TY1B-DR1;TY1B-PR2;TY1B-DR5;TY1B-PR1;TY1B-JR2;TY1B-OL;TY1B-LR4;TY1B-ML2;TY1B-PR3;TY1B-PL;TY1B-LR2;TY1I

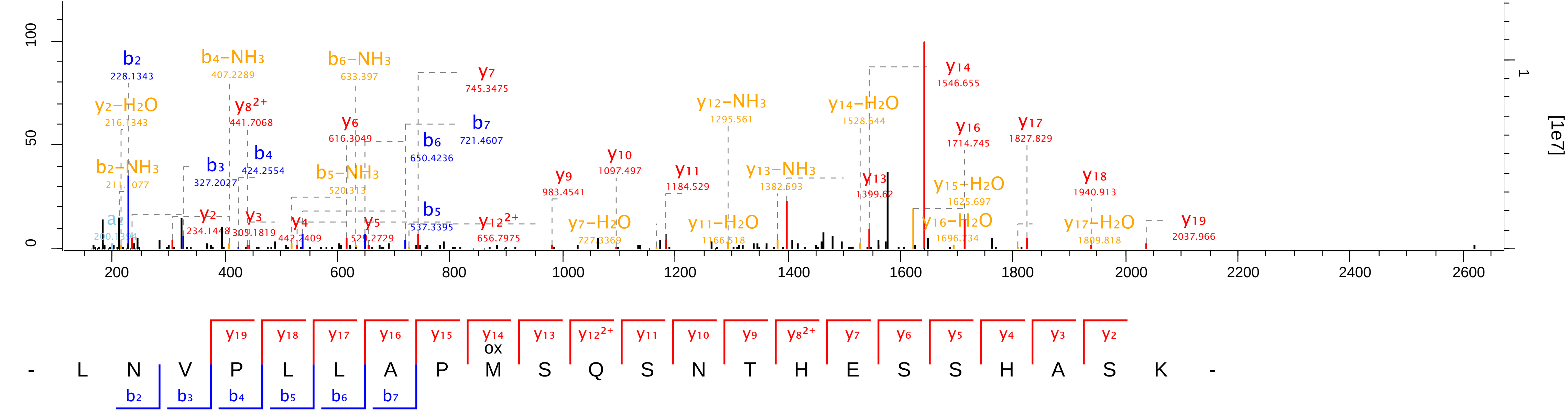

Raw file	Scan	Method	Score	m/z	Gene names
20140602_QEp4_FaHo_SA_SET1_02	1451	FTMS; HCD	166.69	581.26	TY1B-ML1;TY1B-BR;TY1A-PR1;TY1A-A;TY1A-DR4;TY1B-H;TY1B-MR2;TY1B-OR;TY1B-DR1;TY1B-NL2;TY1B-PR2;TY1B-DR5;TY1B-PR1;TY1B-JR2

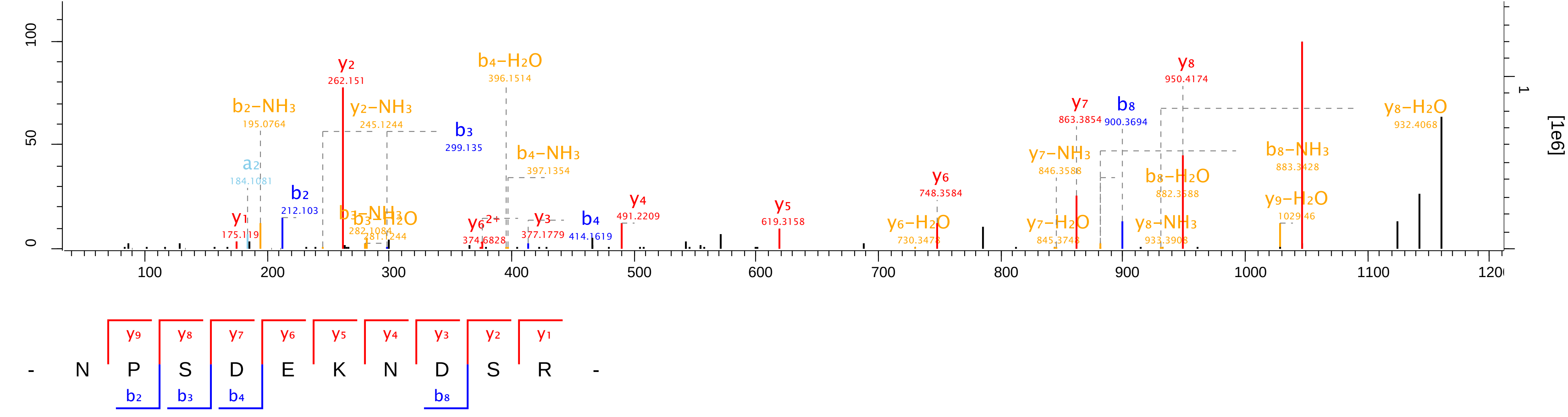

Raw file	Scan	Method	Score	m/z	Gene names
20140602_QEp4_FaHo_SA_SET1_02	3044	FTMS; HCD	182.39	443.74	RPS14B

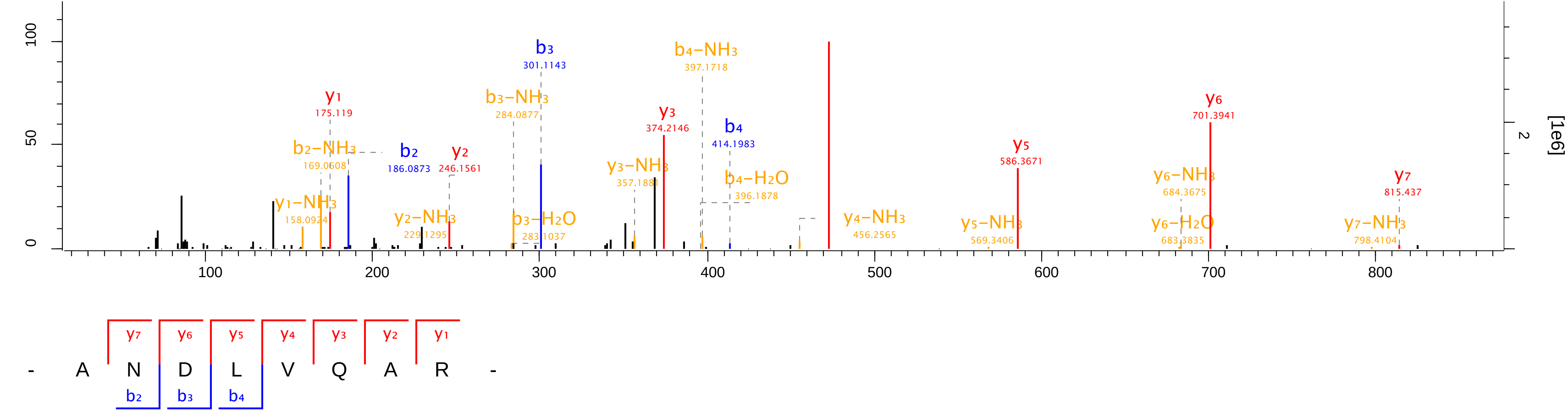

Raw file	Scan	Method	Score	m/z	Gene names
20140602_QEp4_FaHo_SA_SET1_02	3205	FTMS; HCD	130.98	512.78	RPS14B;RPS14A

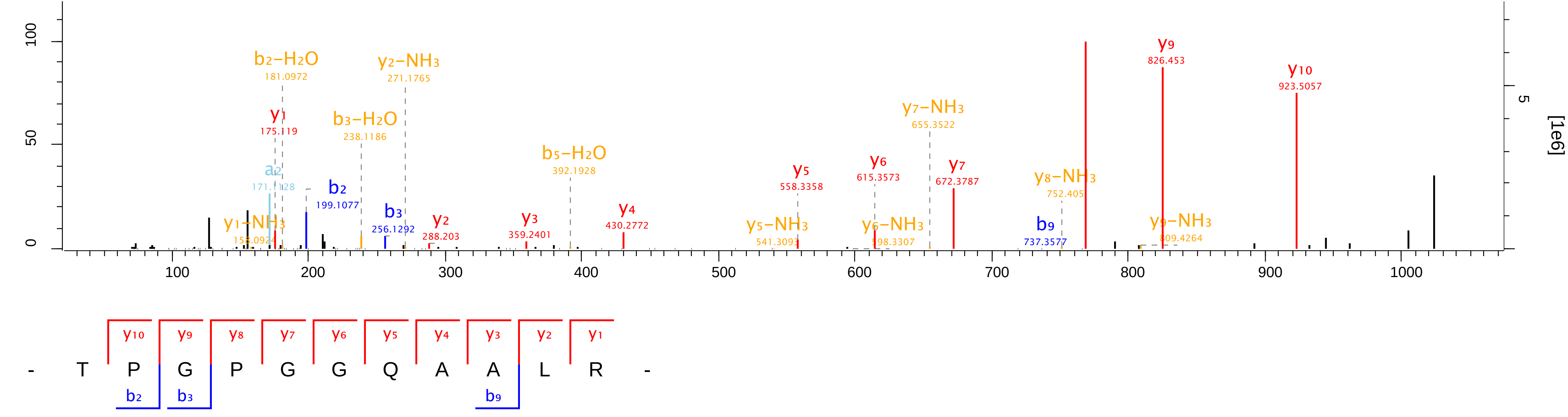

Raw file	Scan	Method	Score	m/z	Gene names
20140602_QEp4_FaHo_SA_SET1_02	3255	FTMS; HCD	124.34	379.72	RPS0B

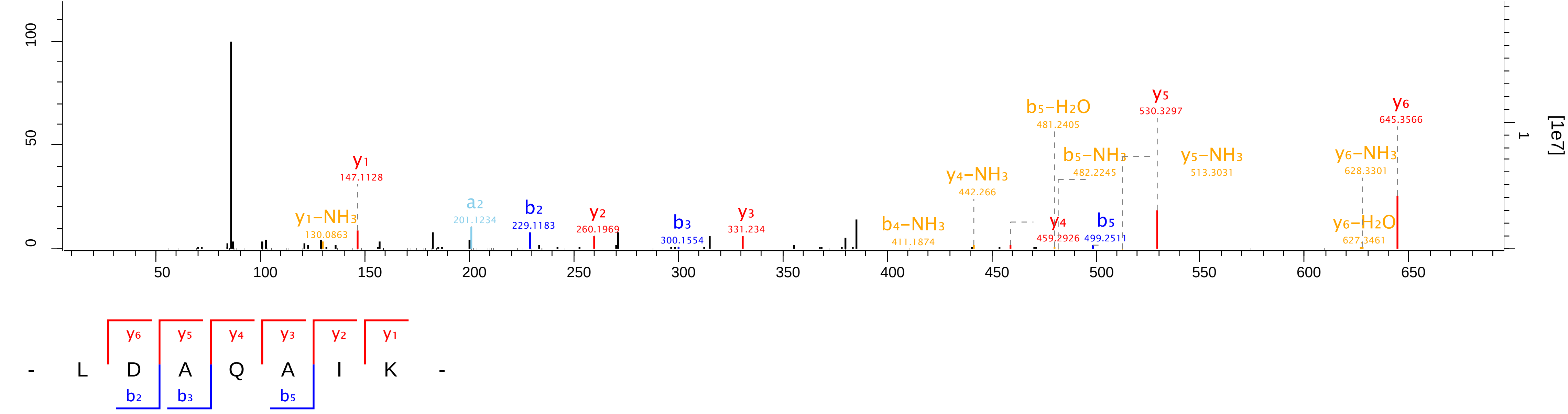

Raw file	Scan	Method	Score	m/z	Gene names
20140602_QEp4_FaHo_SA_SET1_02	3978	FTMS; HCD	60.76	396.21	RPL14B;RPL14A

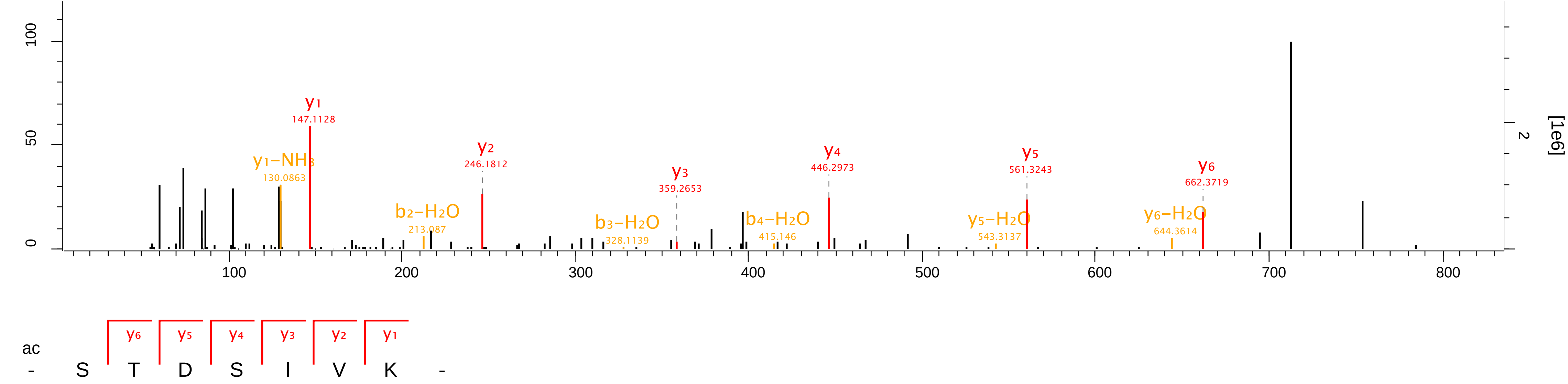

Raw file	Scan	Method	Score	m/z	Gene names
20140602_QEp4_FaHo_SA_SET1_02	7348	FTMS; HCD	258.96	773.41	RPL36B;RPL36A

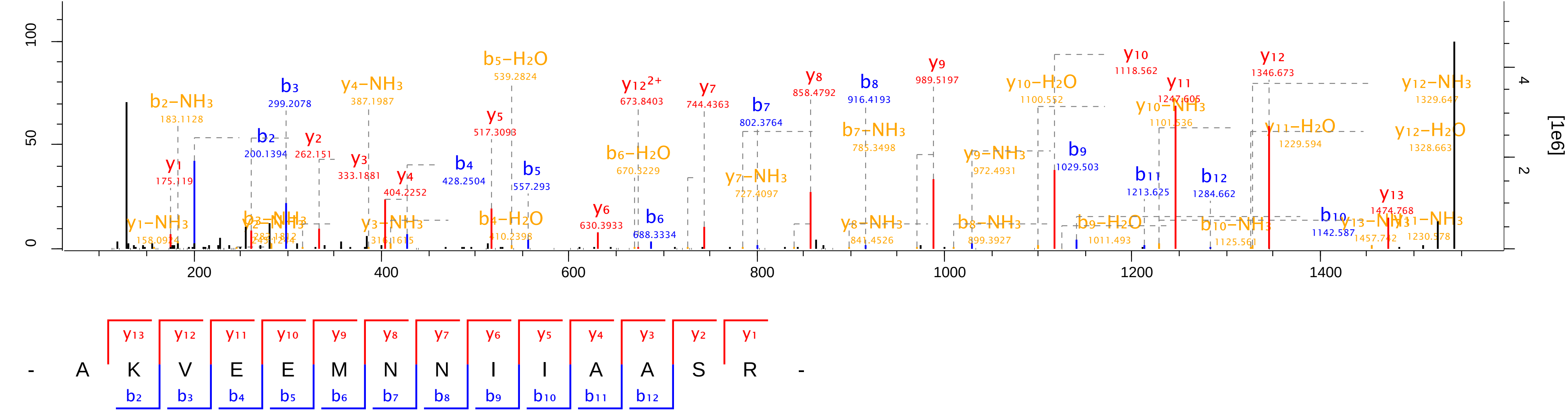

Raw file	Scan	Method	Score	m/z	Gene names
20140602_QEp4_FaHo_SA_SET1_02	7349	FTMS; HCD	189.57	650.84	RPL17A

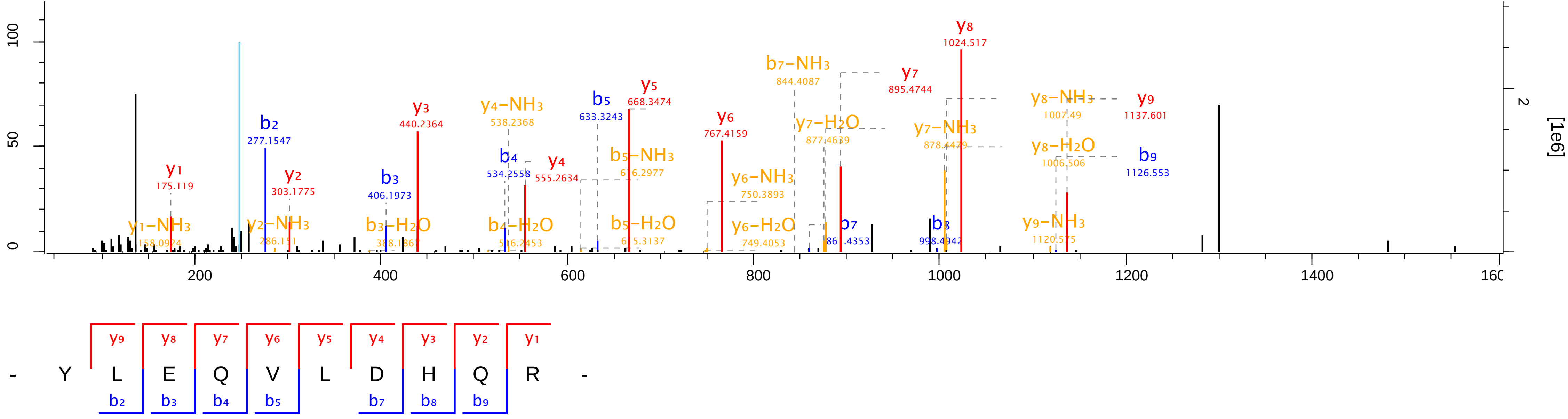

Raw file	Scan	Method	Score	m/z	Gene names
20140602_QEp4_FaHo_SA_SET3_03	1597	FTMS; HCD	123.86	465.72	IMP2'

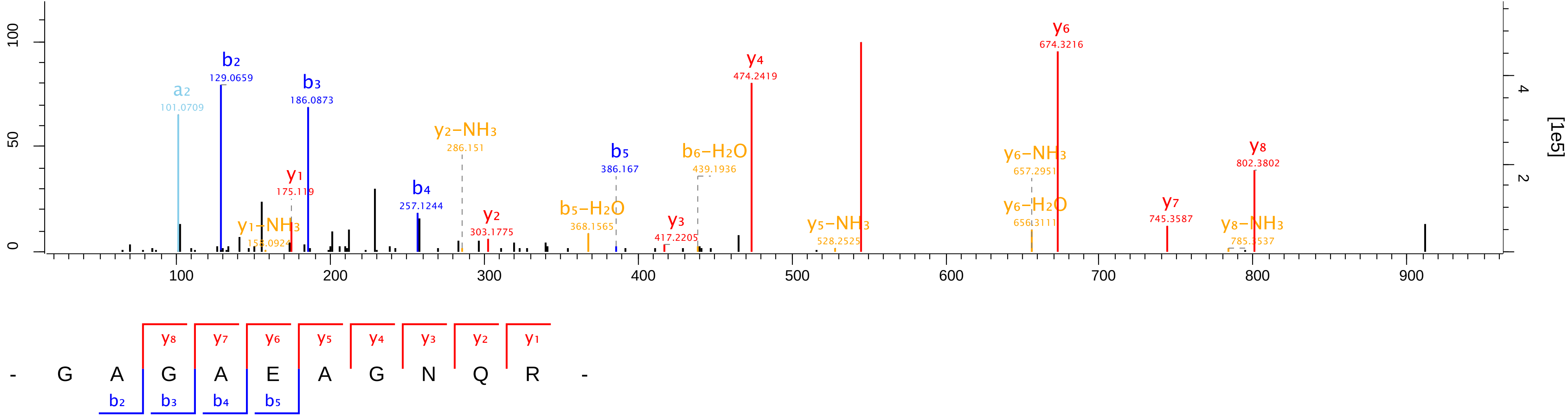

Raw file	Sca	Metho	Scoı	m/z	Gene names
20140602_QEp4_Fal	26[illegible]	FTMS[illegible]	82.[illegible]	377.	TY1B-ML1;TY1B-BR;TY1A-PR1;TY1A-A;TY1A-DR4;TY1B-H;TY1B-MR2;TY1B-OR;TY1B-DR1;TY1B-NL2;TY1B-PR2;TY1B-DR5;TY1B-PR1;TY1B-JR2;TY1B-NL1;TY1A-PL;TY1A-LR2;TY

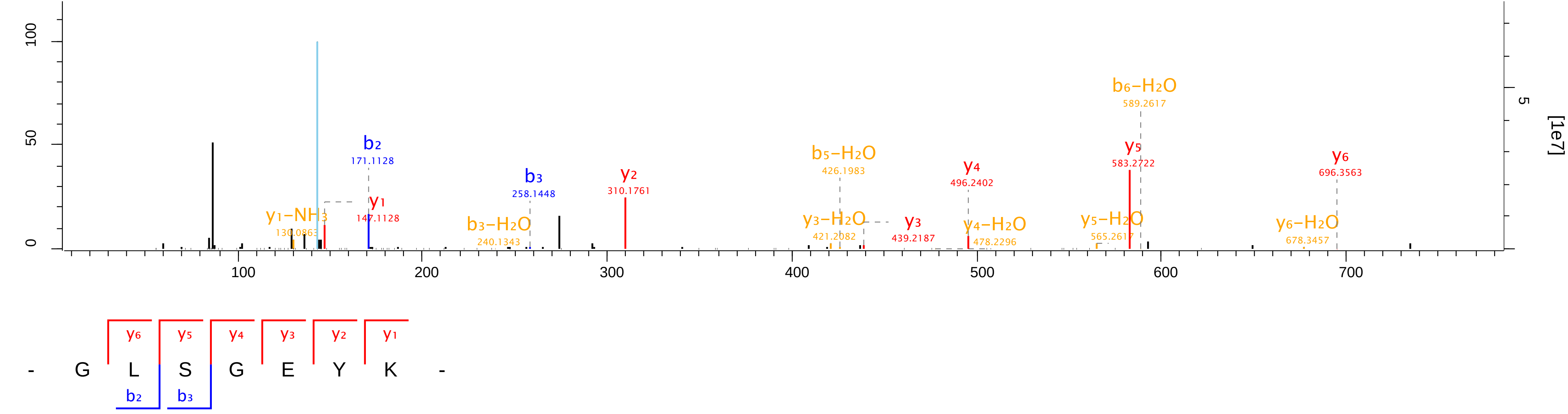

Raw file	Scan	Method	Score	m/z	Gene names
20140602_QEp4_FaHo_[illegible]	18[illegible]	FTMS[illegible]	2143.[illegible]	520.[illegible]	TY1B-ML1;TY1B-BR;TY1A-PR1;TY1A-A;TY1A-DR4;TY1B-H;TY1B-MR2;TY1B-OR;TY1B-DR1;TY1B-NL2;TY1B-PR2;TY1B-DR5;TY1B-PR1;TY1B-JR2;TY1A-PL;TY1A-LR2;TY1A-[illegible]

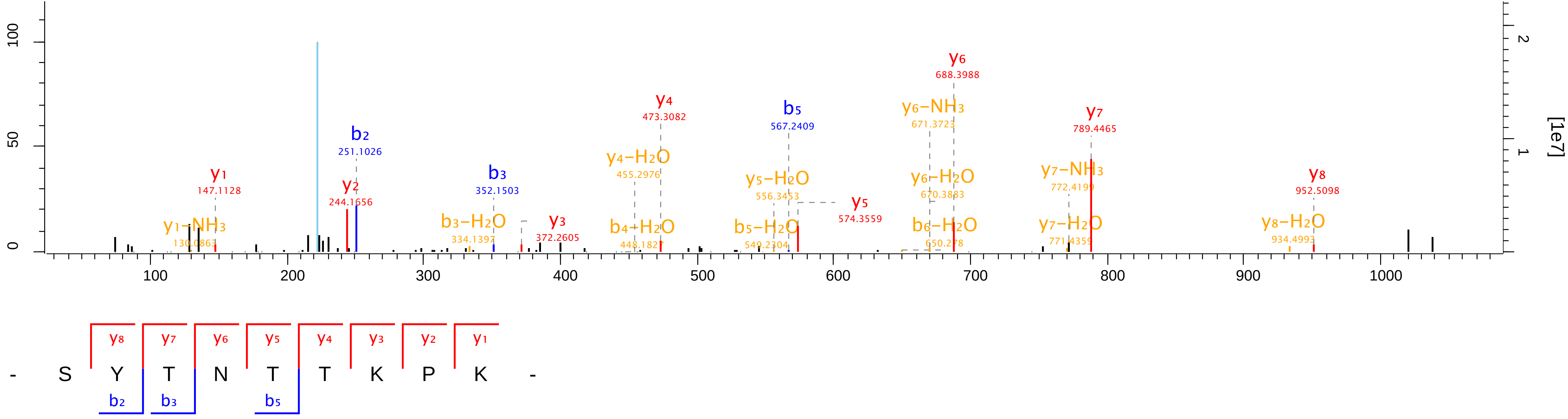

Raw file	Scan	Method	Score	m/z	Gene names
20140602_QEp4_FaHo_SA_SHG1_03	3005	FTMS; HCD	87.09	784.38	SUR4

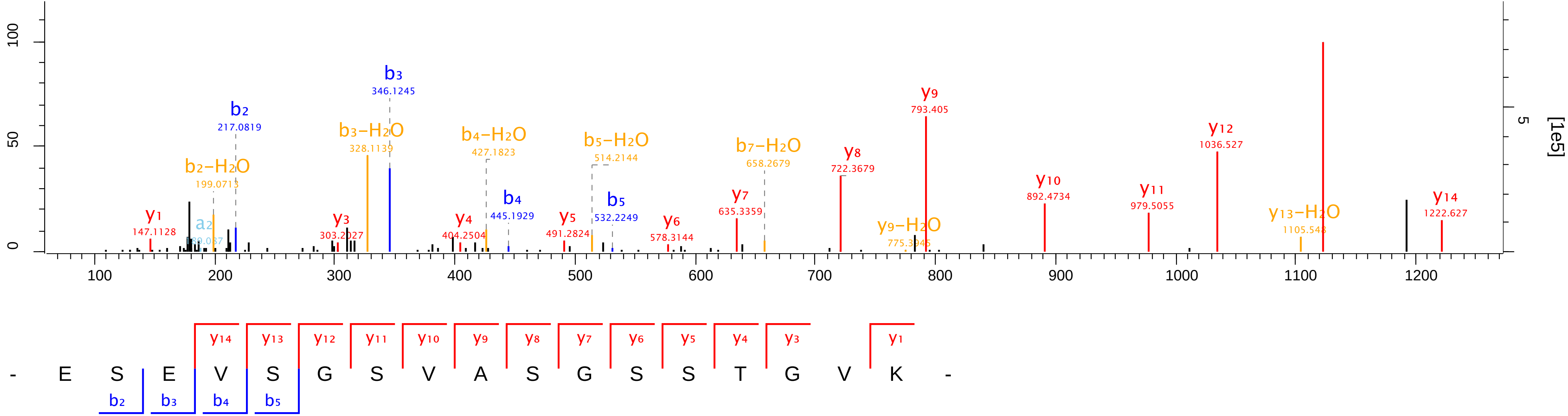

Raw file	Scan	Method	Score	m/z	Gene names
20140602_QEp4_FaHo_SA_SIR2_01	4785	FTMS; HCD	97.96	512.28	MES1

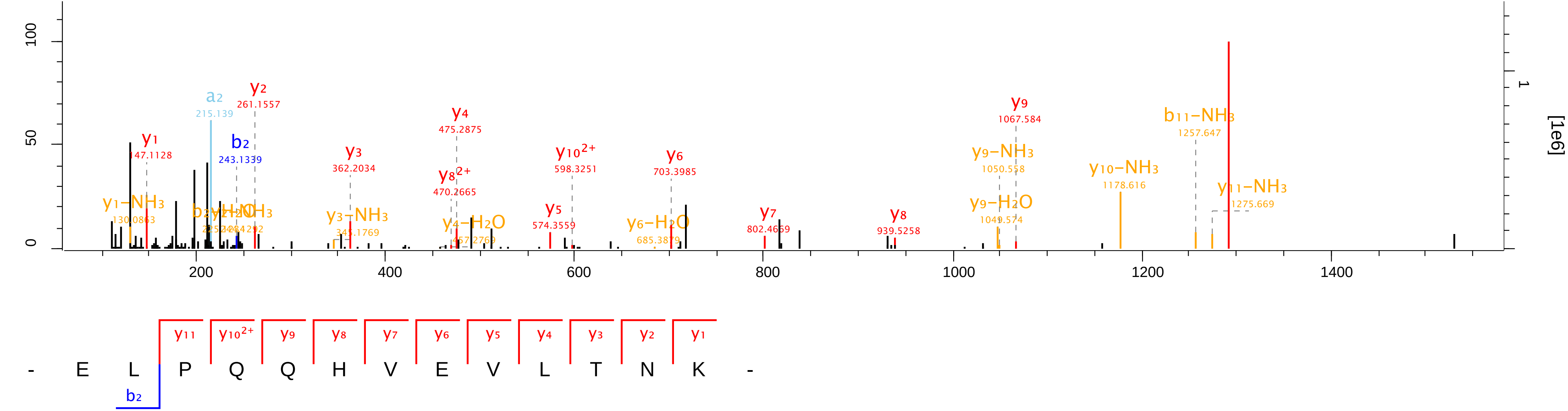

Raw file	Scan	Method	Score	m/z	Gene names
20140602_QEp4_FaHo_SA_SIR2_01	5927	FTMS; HCD	156.17	753.88	RPL4B;RPL4A

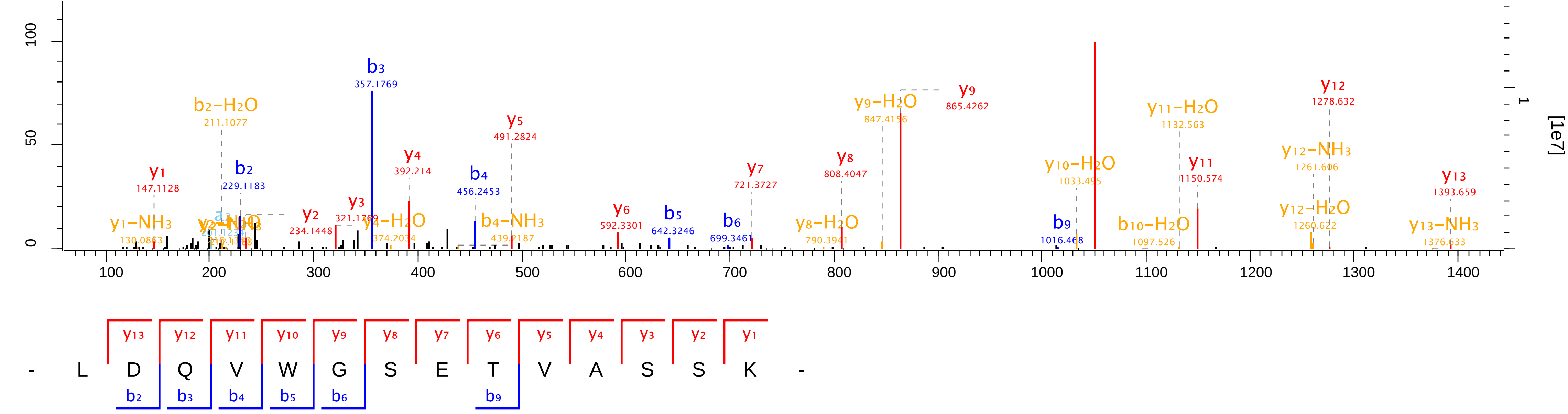

Raw file	Scan	Method	Score	m/z	Gene names
20140602_QEp4_FaHo_SA_SIR2_01	5939	FTMS; HCD	74.46	345.54	PBI2

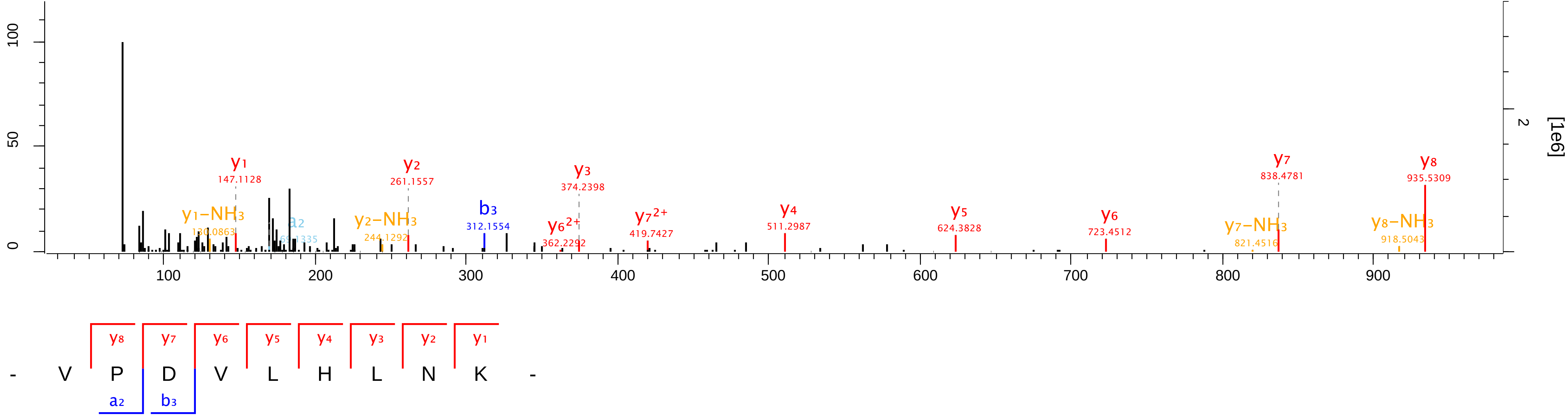

Raw file	Scan	Method	Score	m/z	Gene names
20140602_QEp4_FaHo_SA_SIR2_01	7038	FTMS; HCD	70.84	703.36	THR1

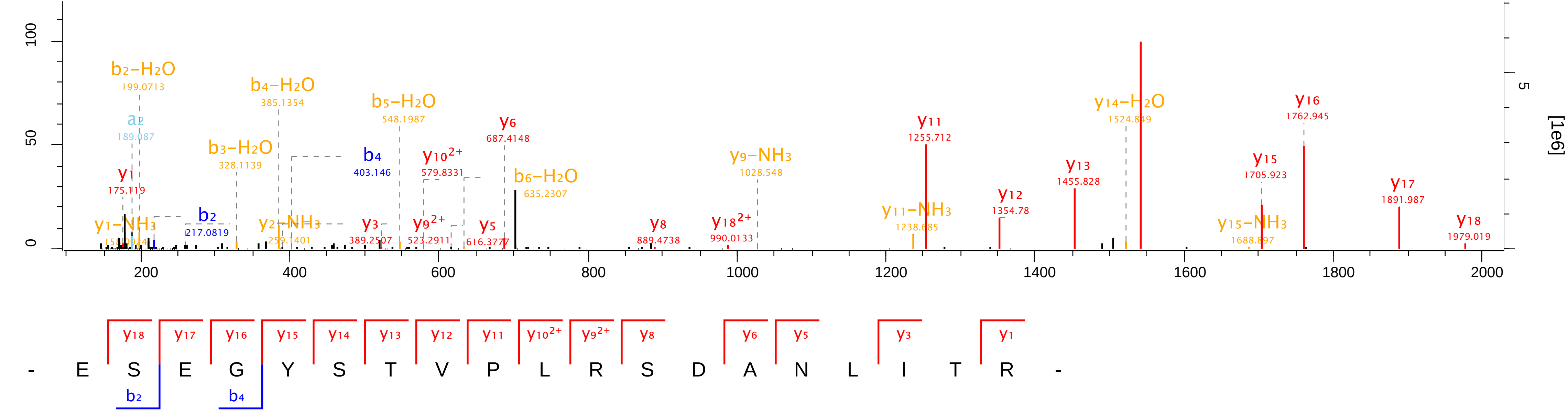

Raw file	Scan	Method	Score	m/z	Gene names
20140602_QEp4_FaHo_SA_SIR2_01	7470	FTMS; HCD	94.01	521.65	DLD3

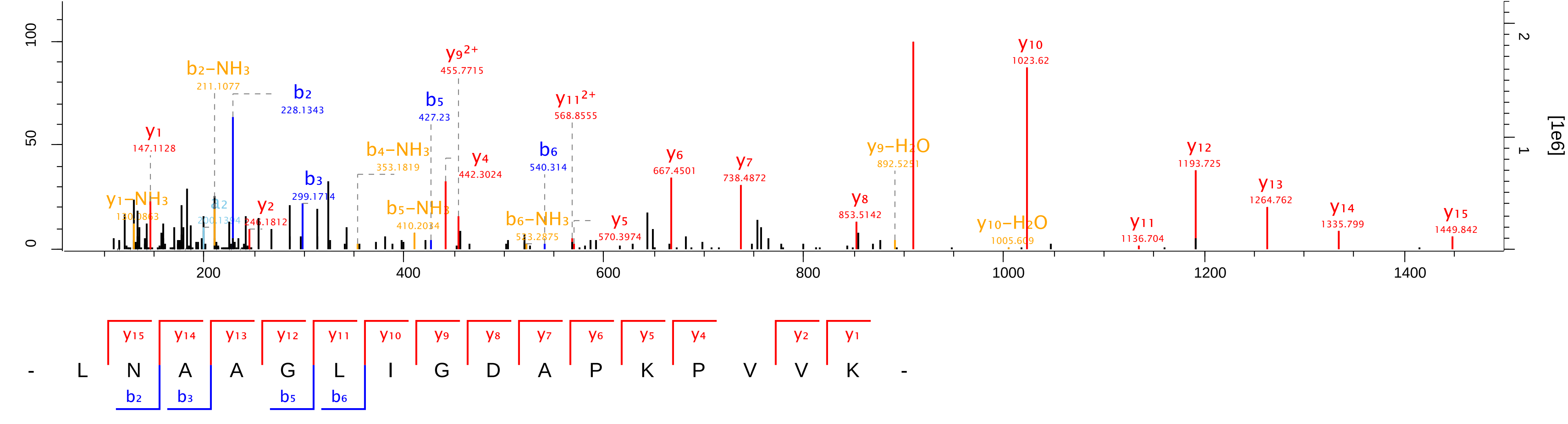

Raw file	Scan	Method	Score	m/z	Gene names
20140602_QEp4_FaHo_SA_SIR2_01	8133	FTMS; HCD	120.84	737.9	RPL4B;RPL4A

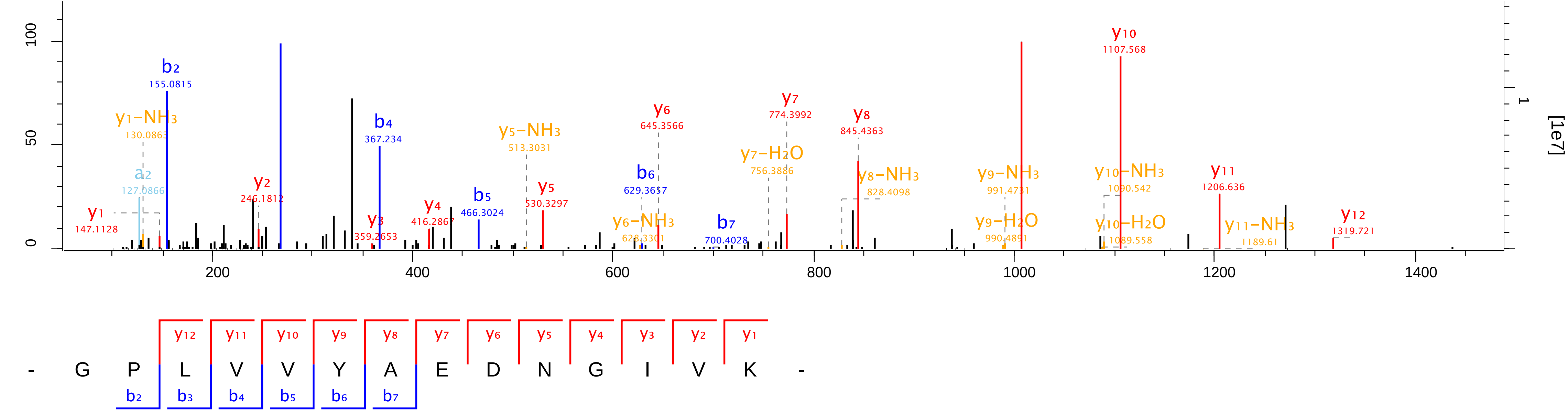

Raw file	Scan	Method	Score	m/z	Gene names
20140602_QEp4_FaHo_SA_SIR2_01	8275	FTMS; HCD	237.89	777.93	RPL7A;RPL7B

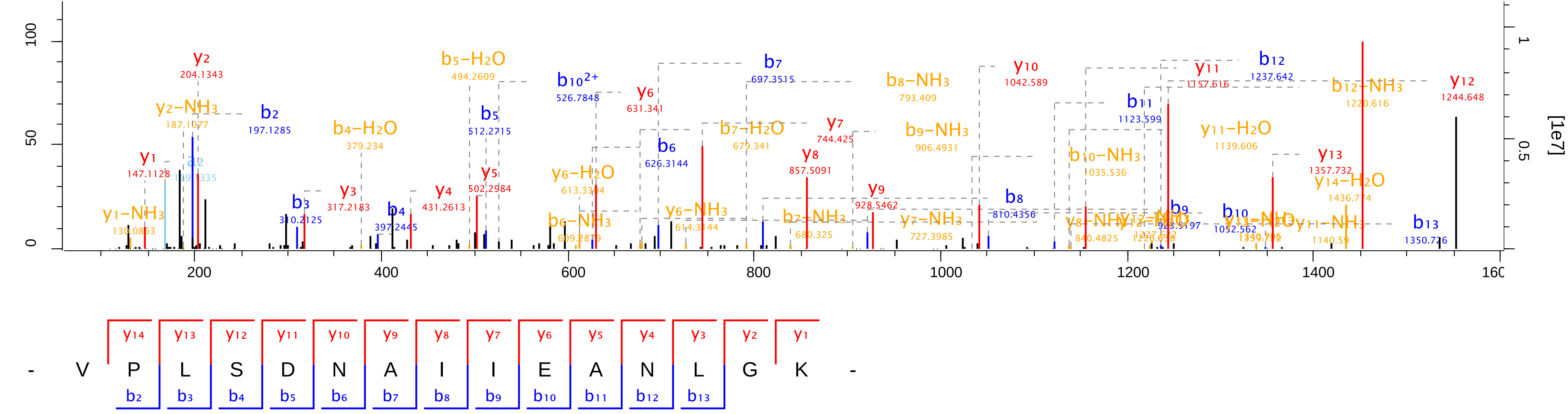

Raw file	Scan	Method	Score	m/z	Gene names
20140602_QEp4_FaHo_SA_SIR2_01	10008	FTMS; HCD	129.35	1018.83	APA1

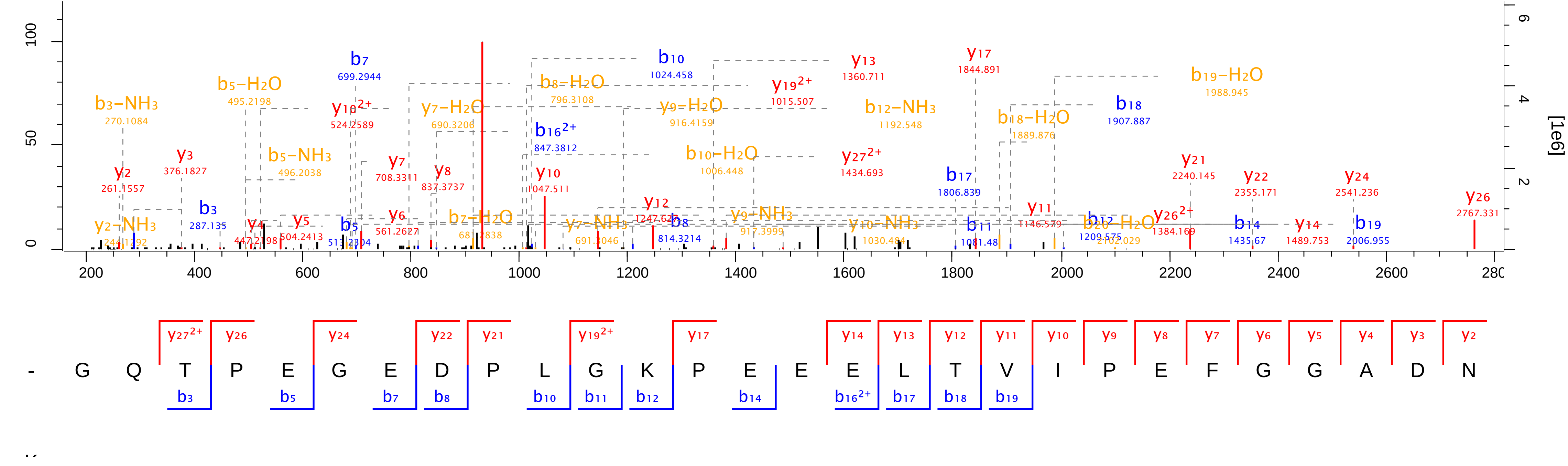

Raw file	Scan	Method	Score	m/z	Gene names
20140602_QEp4_FaHo_SA_SIR2_01	10034	FTMS; HCD	132.84	553.65	PGM2

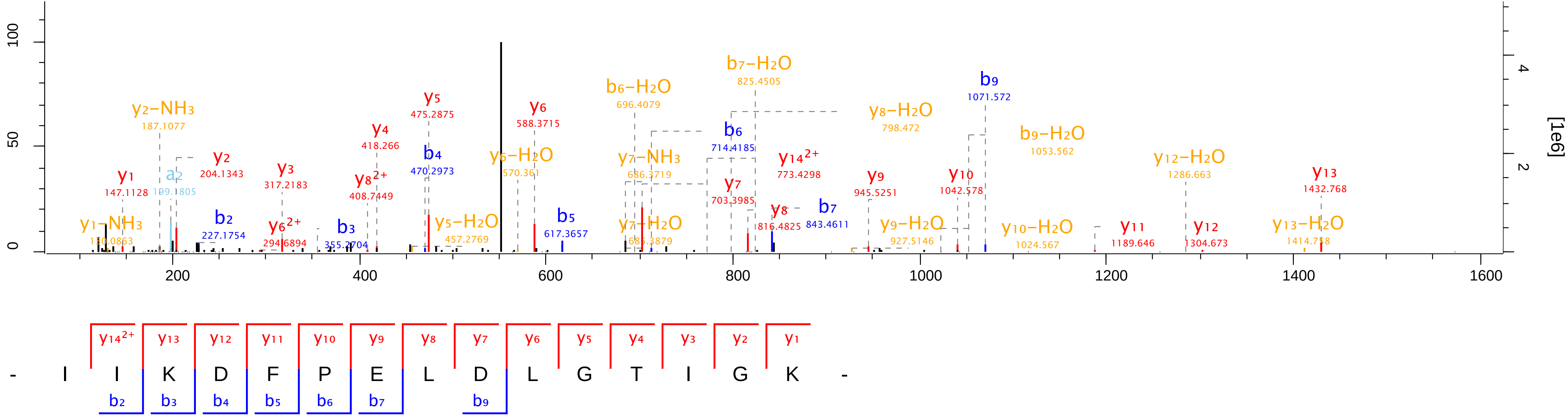

Raw file	Scan	Method	Score	m/z	Gene names
20140602_QEp4_FaHo_SA_SIR2_01	10081	FTMS; HCD	105.79	731.36	TPM2

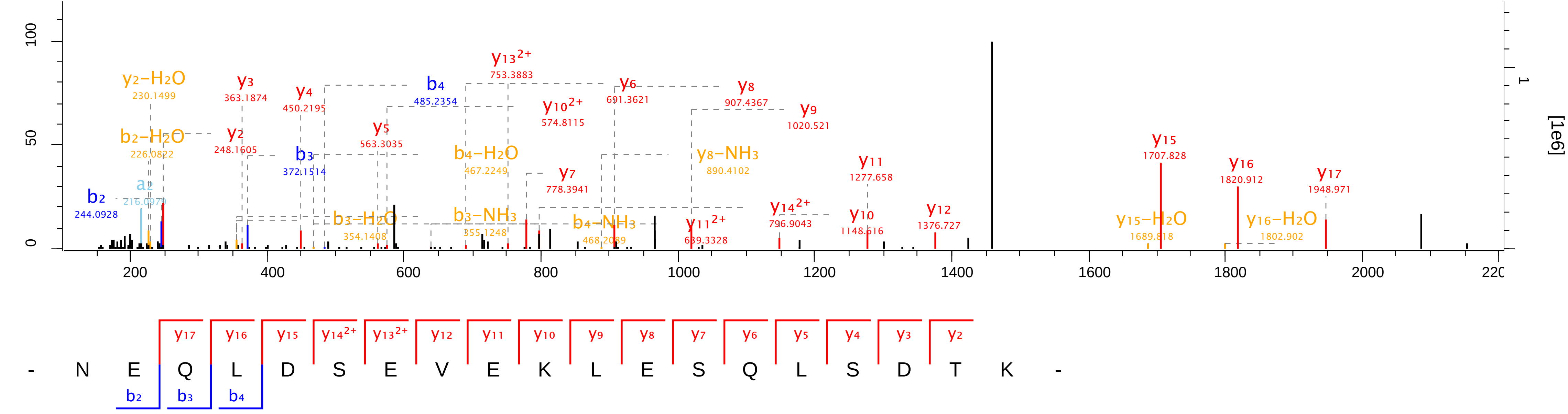

Raw file	Scan	Method	Score	m/z	Gene names
20140602_QEp4_FaHo_SA_SIR2_02	3479	FTMS; HCD	163.65	571.28	PFY1

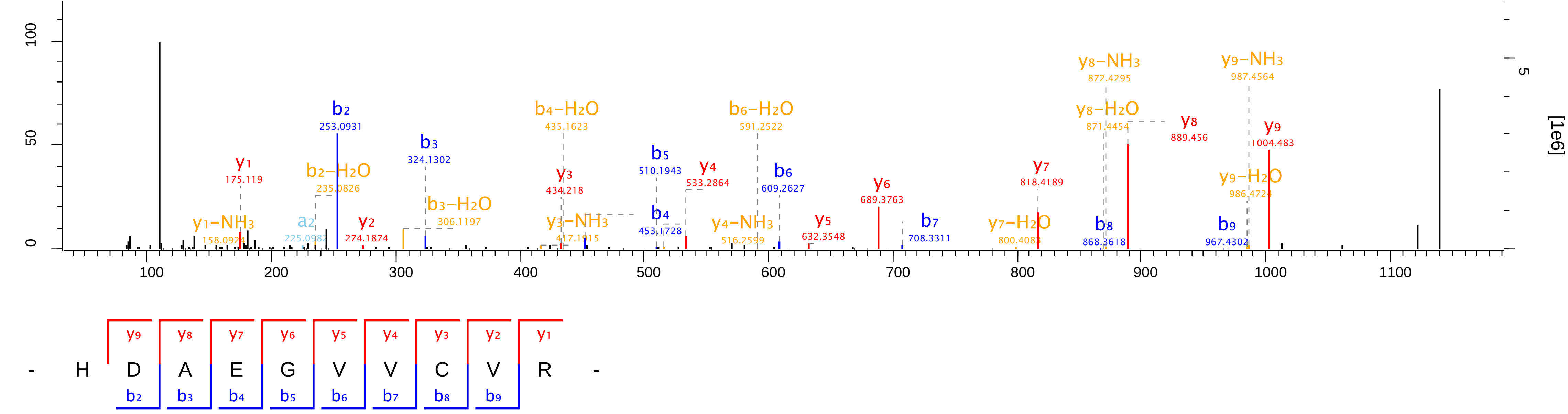

Raw file	Scan	Method	Score	m/z	Gene names
20140602_QEp4_FaHo_SA_SIR2_03	4500	FTMS; HCD	101.97	471.77	RPS26A;RPS26B

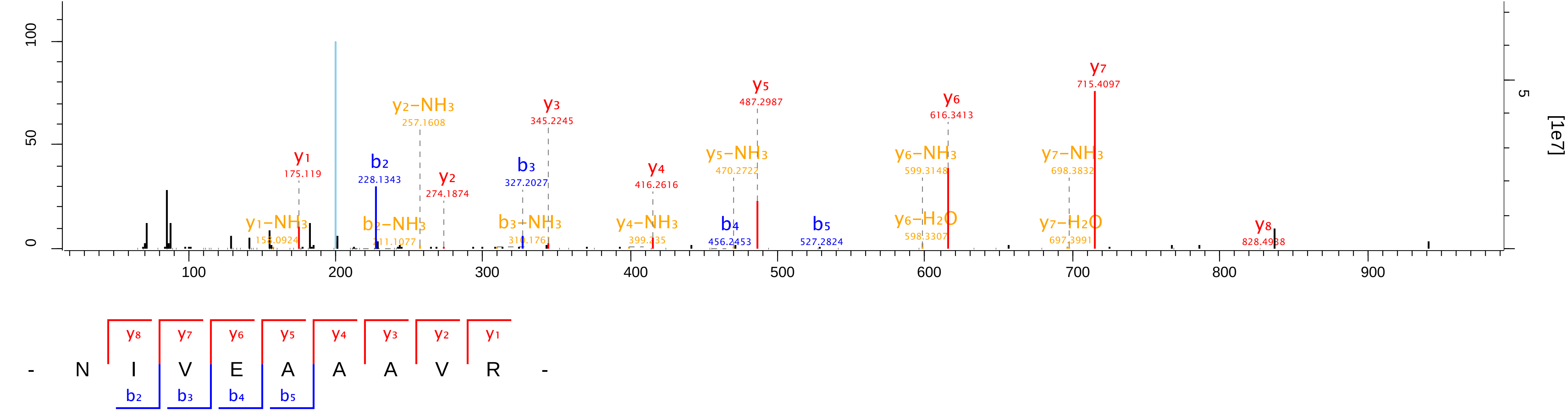

Raw file	Scan	Method	Score	m/z	Gene names
20140602_QEp4_FaHo_SA_SIR2_03	5025	FTMS; HCD	106.32	691.83	TRX1

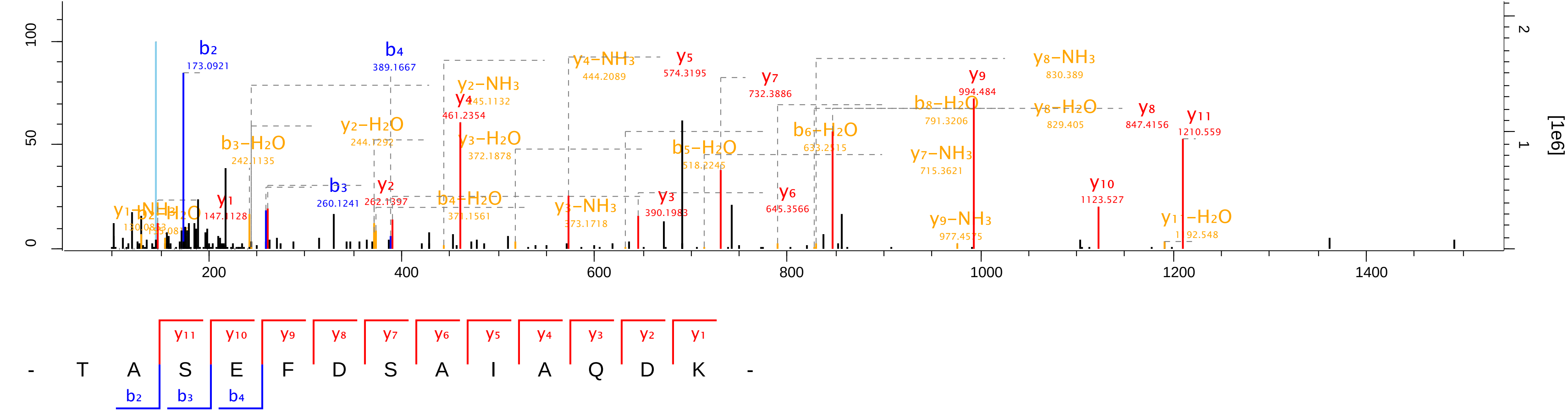

Raw file	Scan	Method	Score	m/z	Gene names
20140602_QEp4_FaHo_SA_SIR2_03	6518	FTMS; HCD	97.73	529.32	YPT31;YPT32

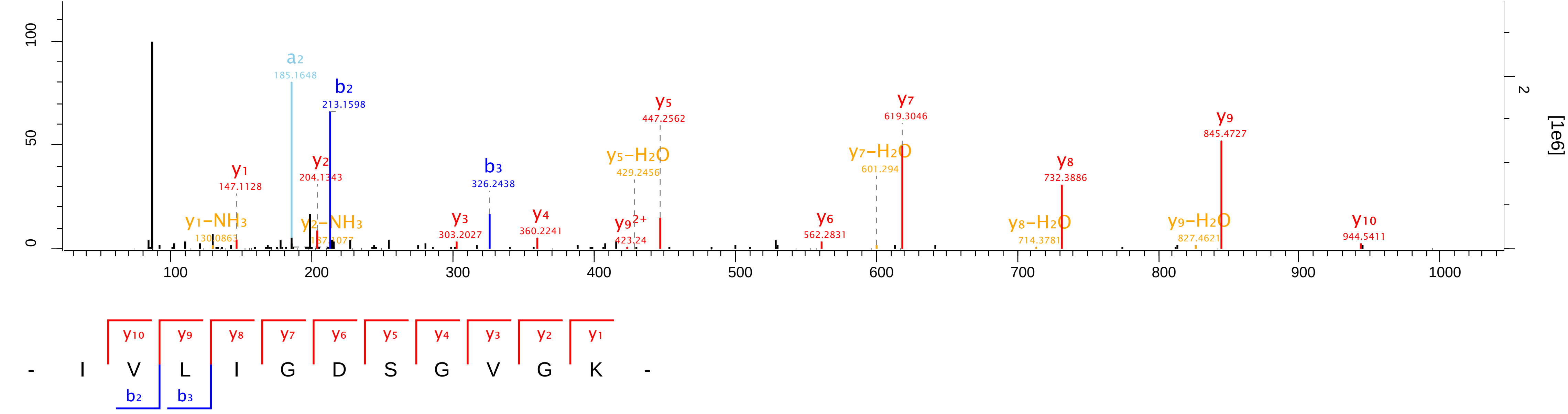

Raw file	Scan	Method	Score	m/z	Gene names
20140602_QEp4_FaHo_SA_SIR2_03	7214	FTMS; HCD	135.8	816.9	ARF1;ARF2

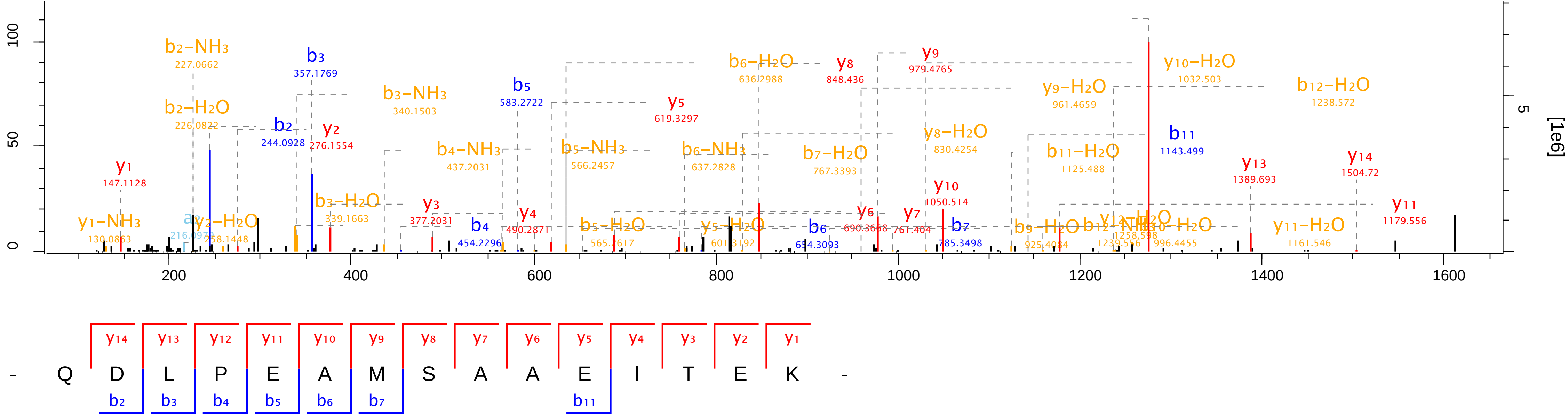

Raw file	Scan	Method	Score	m/z	Gene names
20140602_QEp4_FaHo_SA_SIR2_03	7535	FTMS; HCD	50.12	662.4	GUS1

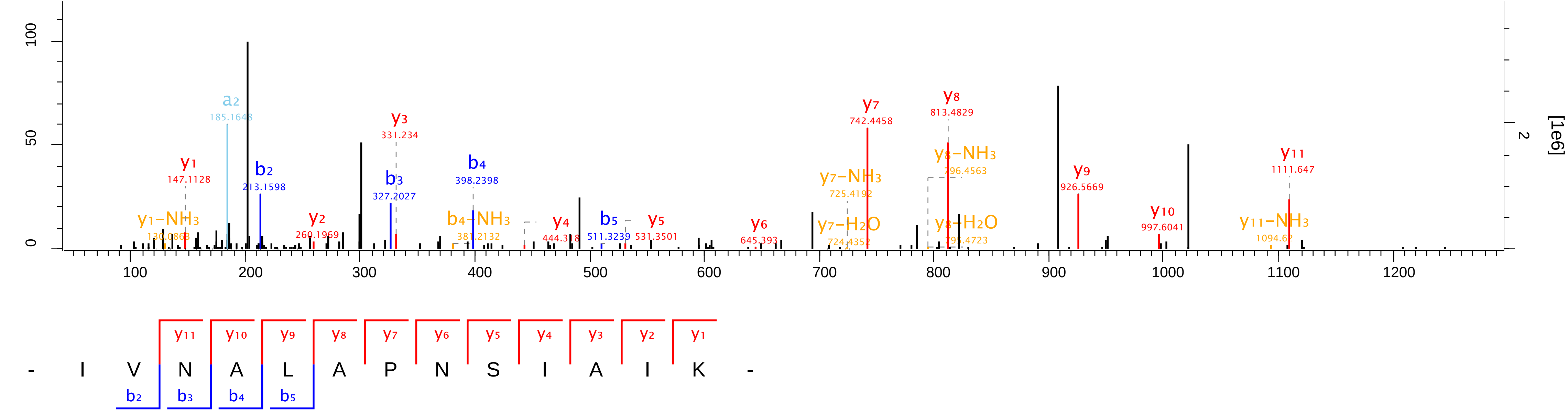

Raw file	Scan	Method	Score	m/z	Gene names
20140602_QEp4_FaHo_SA_SIR2_03	8173	FTMS; HCD	108.9	594.35	ARG4

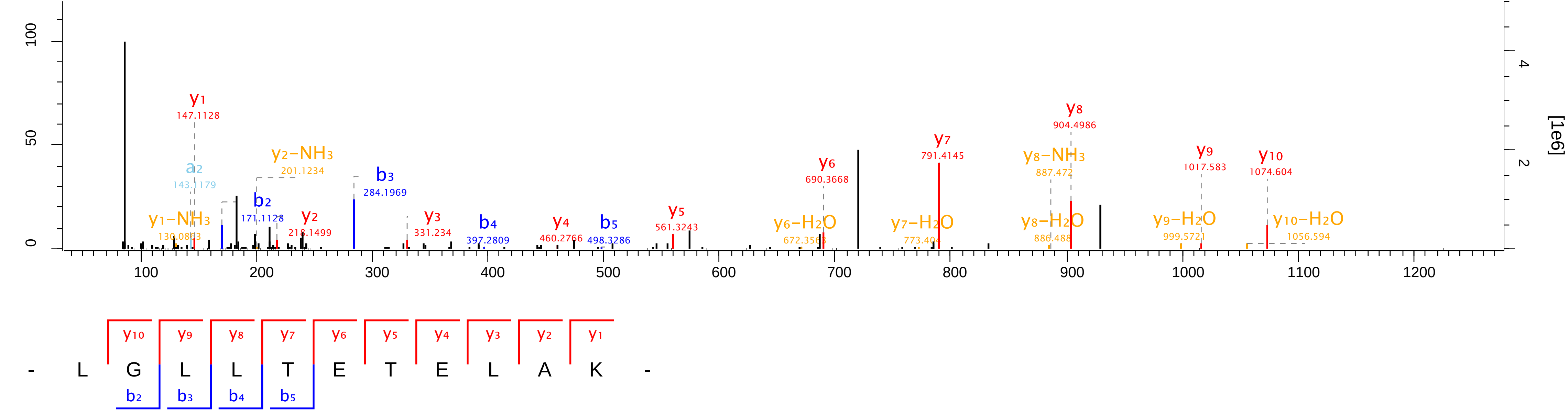

Raw file	Scan	Method	Score	m/z	Gene names
20140602_QEp4_FaHo_SA_SIR2_03	8701	FTMS; HCD	106.44	664.35	YCP4

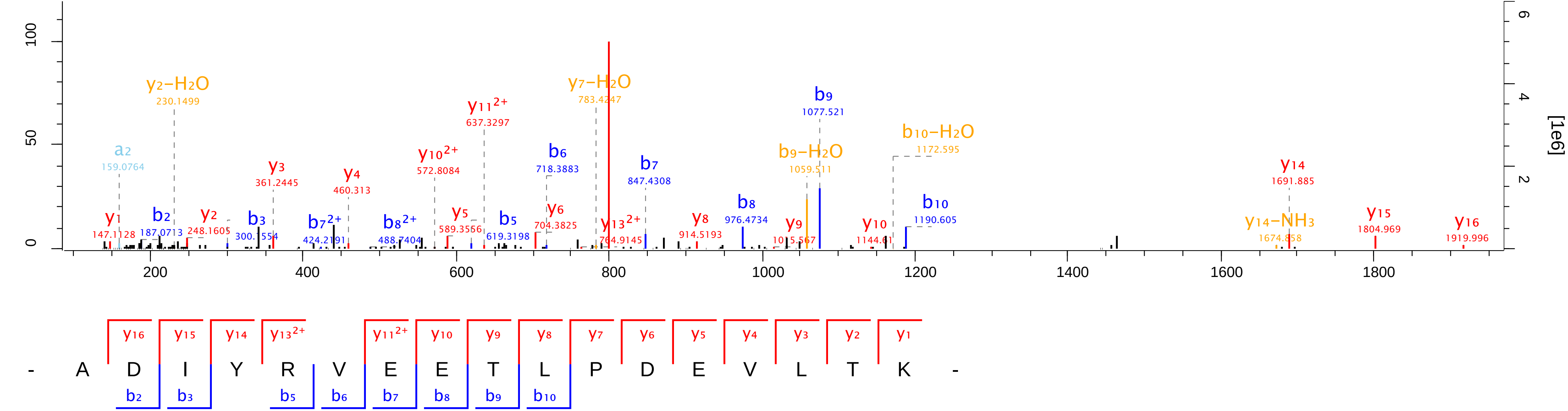

Raw file	Scan	Method	Score	m/z	Gene names
20140602_QEp4_FaHo_SA_SIR2_03	9437	FTMS; HCD	251.35	1088.58	RPL9B

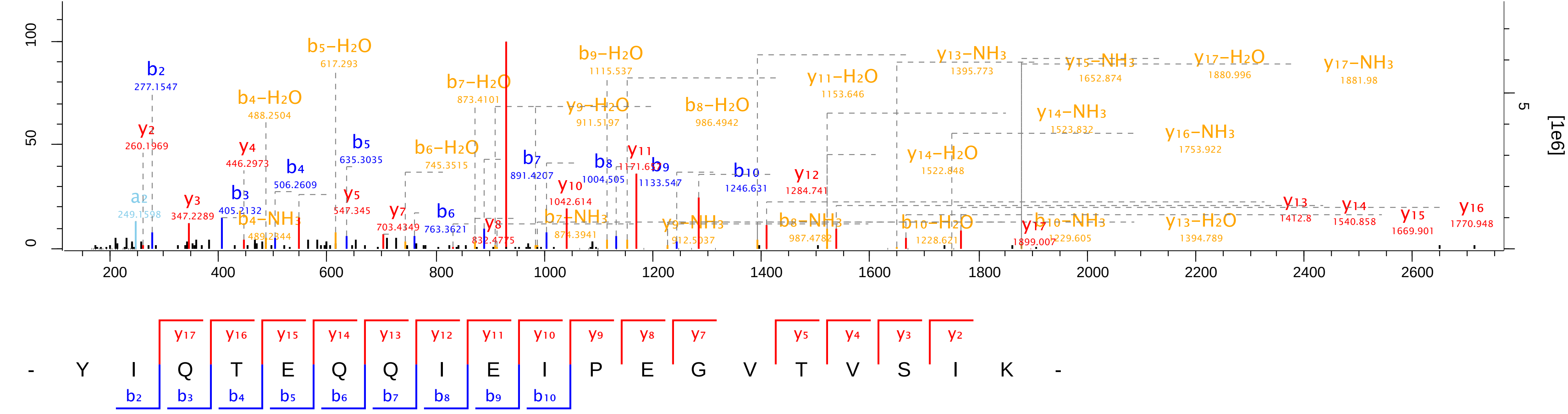

Raw file	Scan	Method	Score	m/z	Gene names
20140602_QEp4_FaHo_SA_SIR2_03	9921	FTMS; HCD	193.42	881.8	ATP16

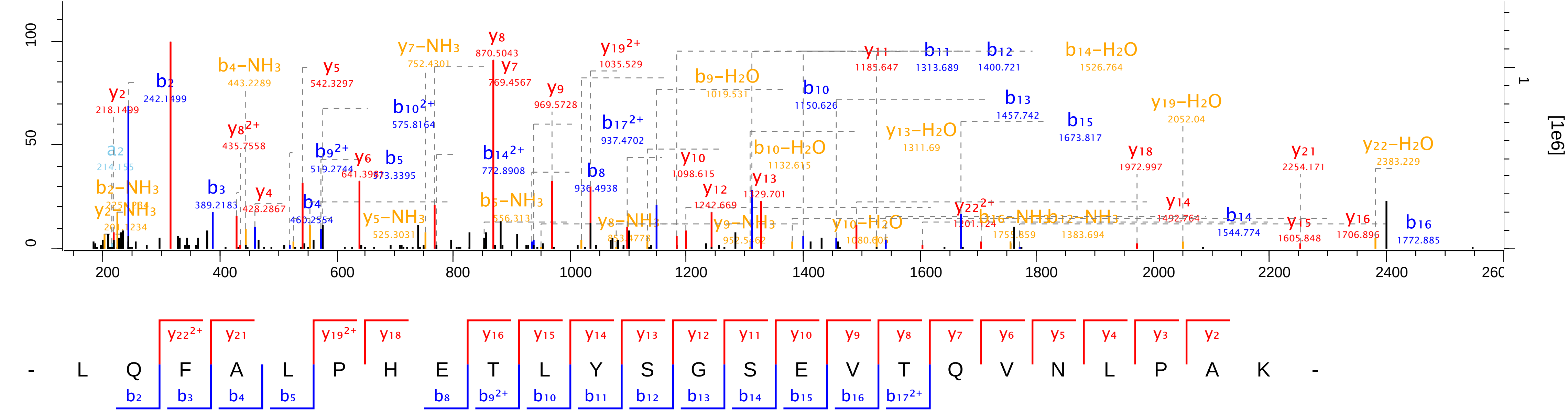

Raw file	Scan	Method	Score	m/z	Gene names
20140602_QEp4_FaHo_SA_SIR2_03	9926	FTMS; HCD	75.1	748.36	VMA5

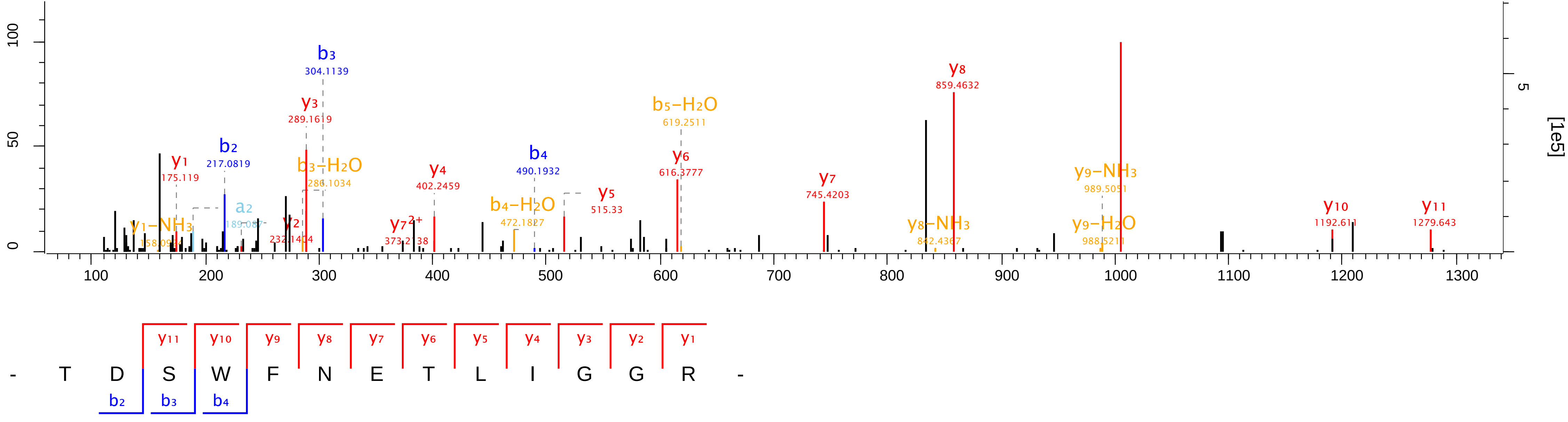

Raw file	Scan	Method	Score	m/z	Gene names
20140602_QEp4_FaHo_SA_SIR2_03	10095	FTMS; HCD	88.43	595.34	FMP52

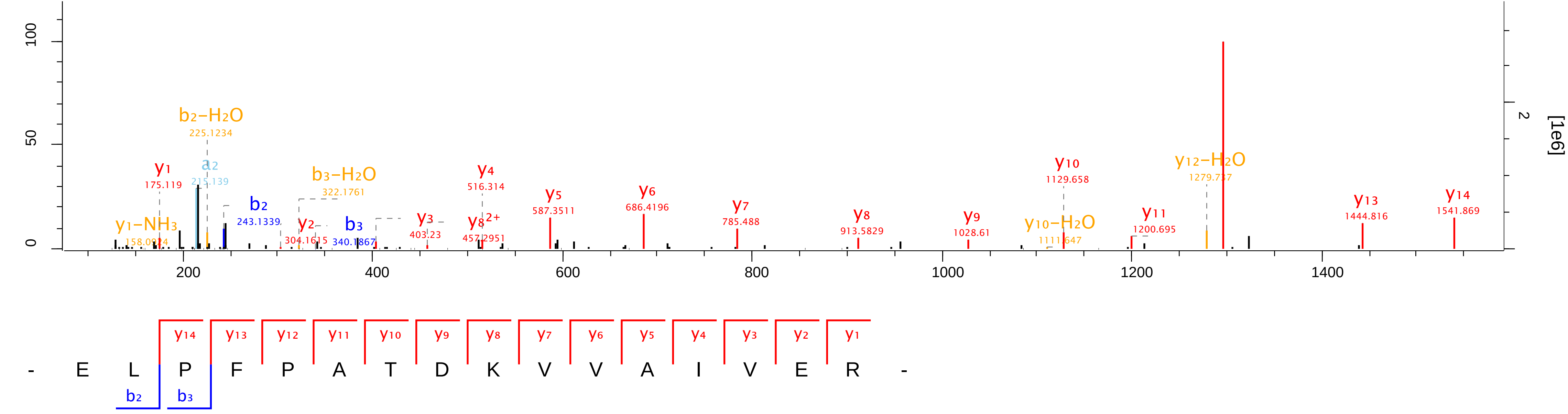

Raw file	Scan	Method	Score	m/z	Gene names
20140602_QEp4_FaHo_SA_SNF2_02	3172	FTMS; HCD	120.46	423.73	RPL24A;RPL24B

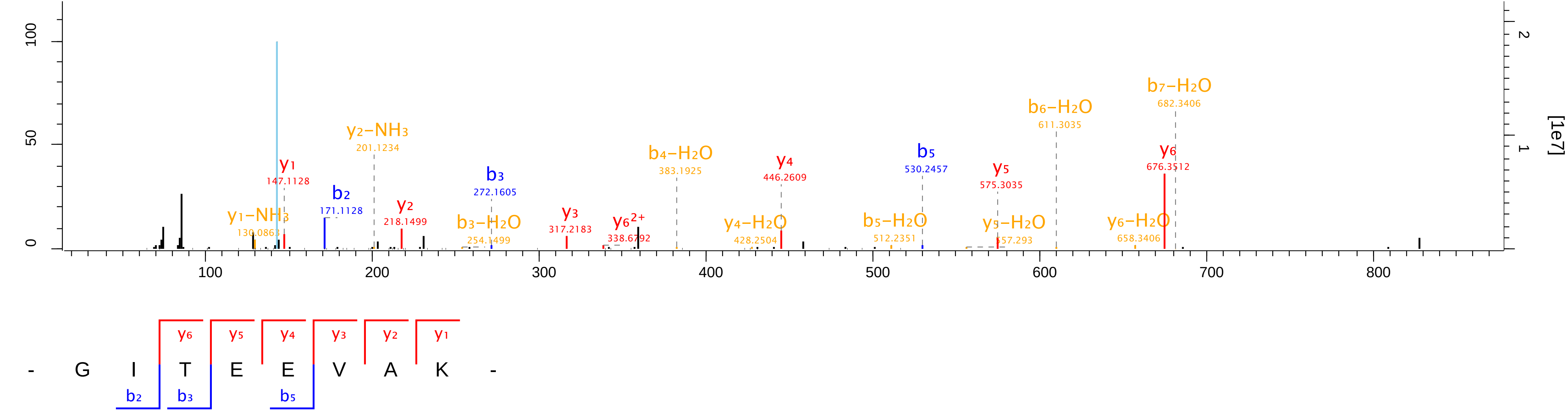

Raw file	Scan	Method	Score	m/z	Gene names
20140602_QEp4_FaHo_SA_SNF2_02	3183	FTMS; HCD	116.37	435.28	RPL14B;RPL14A

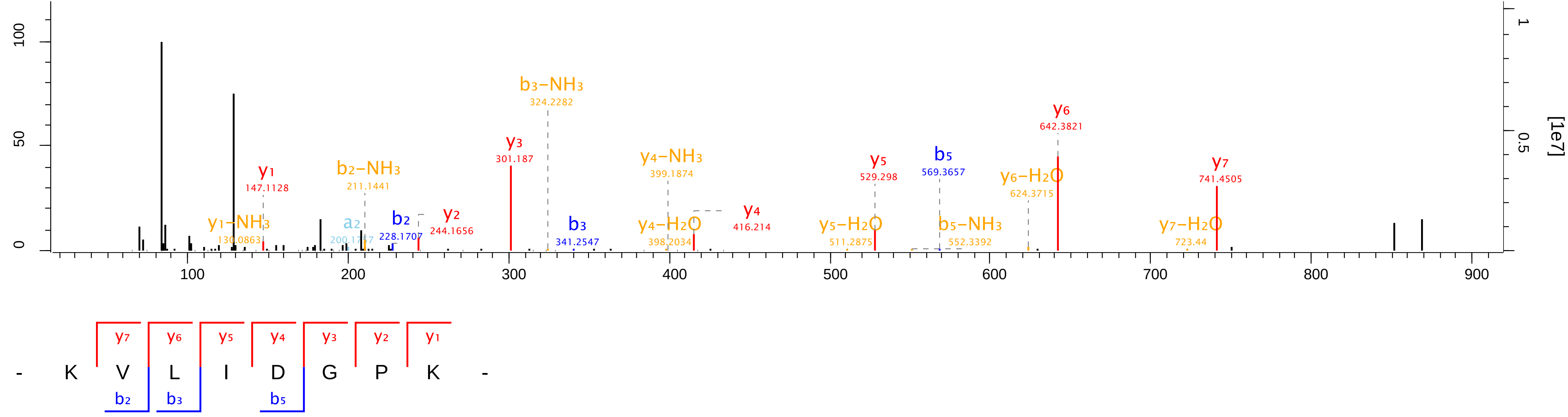

Raw file	Scan	Method	Score	m/z	Gene names
20140602_QEp4_FaHo_SA_SNF2_02	4817	FTMS; HCD	194.94	546.78	RPS14B;RPS14A

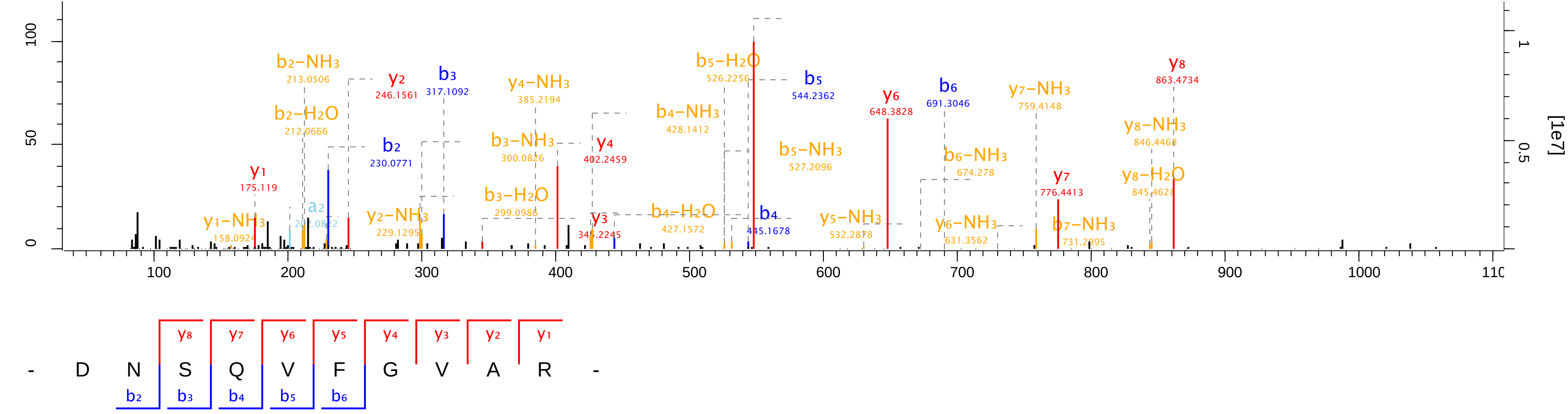

Raw file	Scan	Method	Score	m/z
20140602_QEp4_FaHo_SA_SNF2_02	8653	FTMS; HCD	7.71	649.63

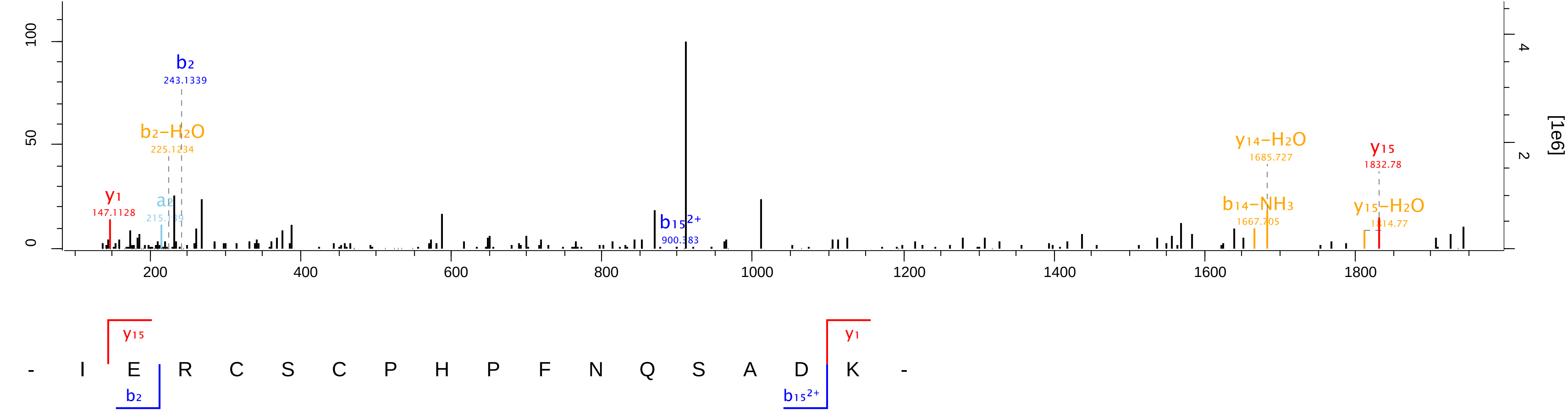

Raw file	Scan	Method	Score	m/z
20140602_QEp4_FaHo_SA_SNF2_02	9226	FTMS; HCD	160.43	821.93

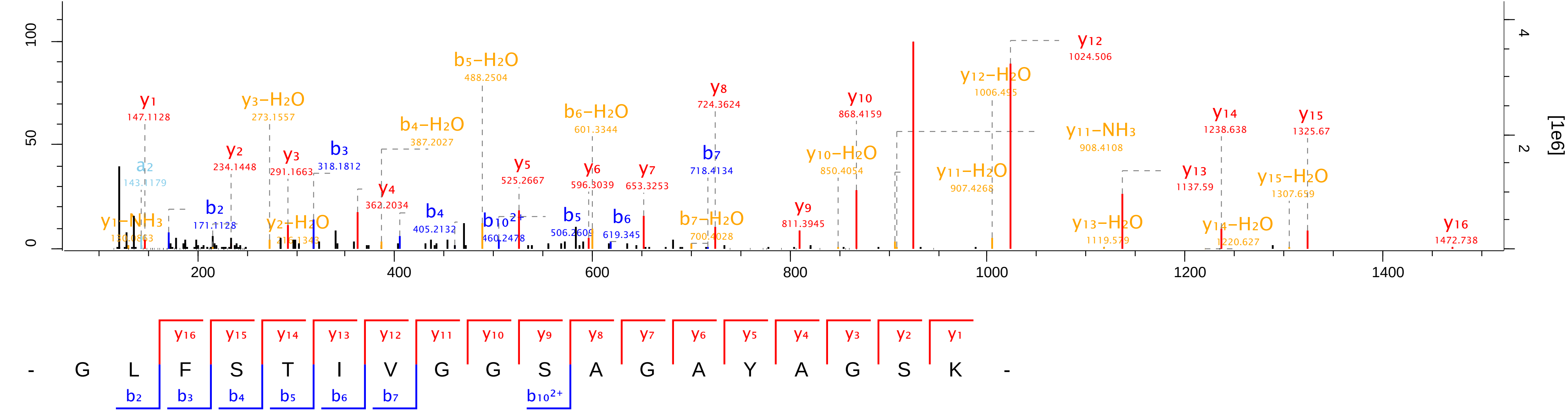

Raw file	Scan	Method	Score	m/z	Gene names
20140602_QEp4_FaHo_SA_SNT2_01	4180	FTMS; HCD	0.79	526.81	RMI1

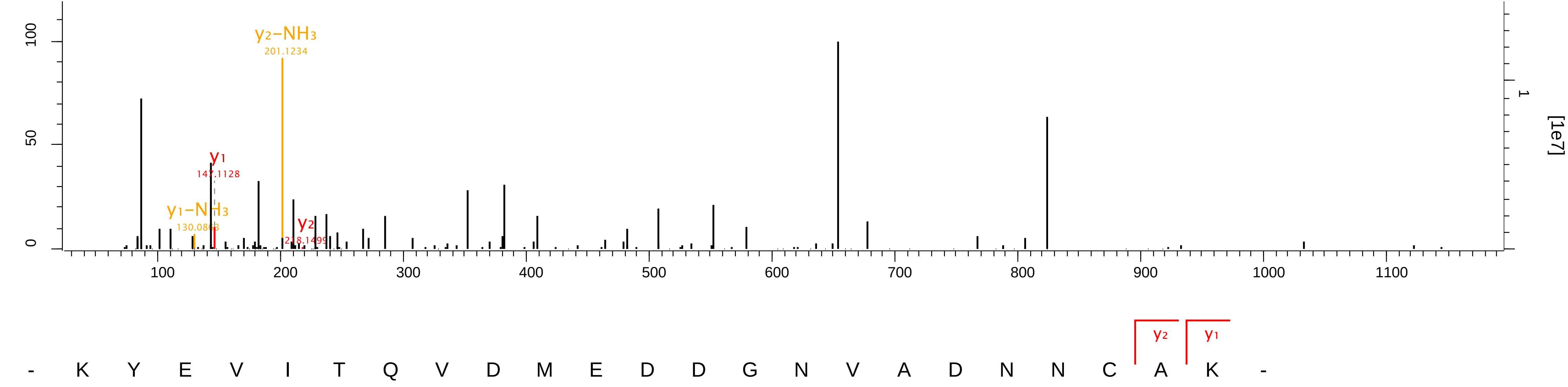

Raw file	Scai	Methoc	Scor	m/z	Gene names
20140602_QEp4_FaHo_	405	FTMS;	0	370. 820	TY1B-ML1;TY1B-BR;TY1A-PR1;TY1A-A;TY1A-DR4;TY1B-H;TY1B-MR2;TY1B-OR;TY1B-DR1;TY1B-NL2;TY1B-PR2;TY1B-DR5;TY1B-PR1;TY1B-JR2;TY1B-NL1;TY1A-PL;TY1A-LR2

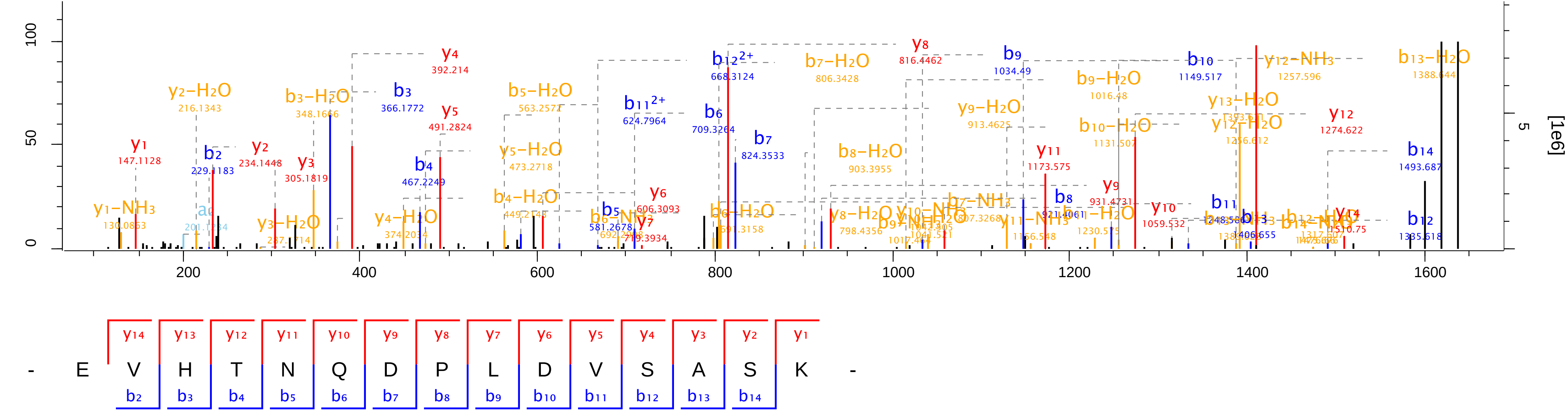

Raw file	Scan	Method	Score	m/z	Gene names
20140602_QEp4_FaHo_SA_SNT2	593	FTMS;	87.26	482.24	TY2B-C;TY2B-B;TY2B-GR2;TY2B-F;TY2B-GR1;TY2A-GR1;TY2B-OR1;TY2A-DR2;TY2B-DR1;TY2B-DR3;TY2B-LR1;TY2B-OR2;TY2A-OR1;TY2A-LR2;TY2A-OR2;

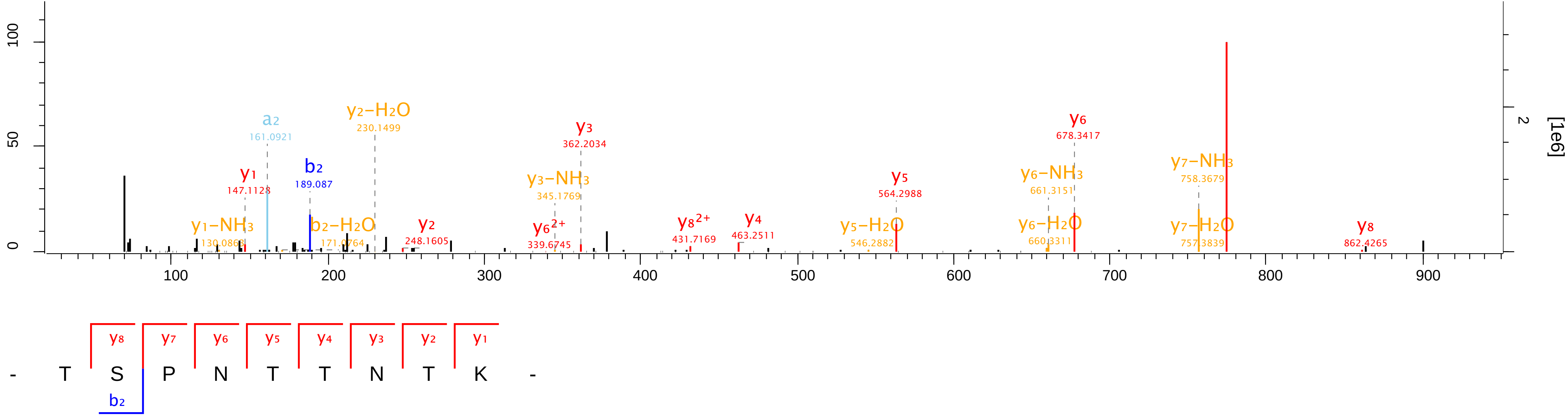

Raw file	Sca	Methc	Sco	m/	Gene names
20140602_QEp4_Fa	61[illegible]	N	86.[illegible]	37[illegible]	TY1B-ML1;TY1B-BR;TY1B-H;TY1B-MR2;TY1B-OR;TY1B-DR1;TY1B-NL2;TY1B-PR2;TY1B-DR5;TY1B-PR1;TY1B-JR2;TY1B-NL1;TY2B-C;TY1B-OL;TY1B-LR4;TY1B-ML2;TY1B-DR3;TY1B

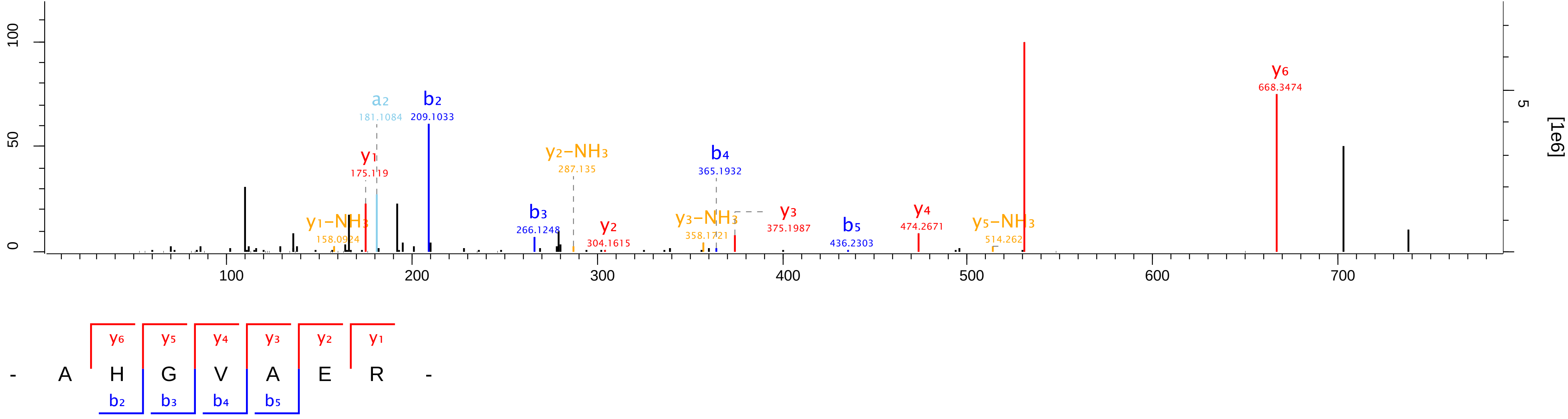

Raw file	Scar	Method	Score	m/z	Gene names
20140602_QEp4_FaHo_SA_	210	T FTMS;	138.8	413.6	TY1B-ML1;TY1B-BR;TY1B-H;TY1B-MR2;TY1B-OR;TY1B-DR1;TY1B-NL2;TY1B-PR2;TY1B-DR5;TY1B-PR1;TY1B-JR2;TY1B-NL1;TY1B-OL;TY1B-LR4;TY1B-ML2;TY1B-DR3;

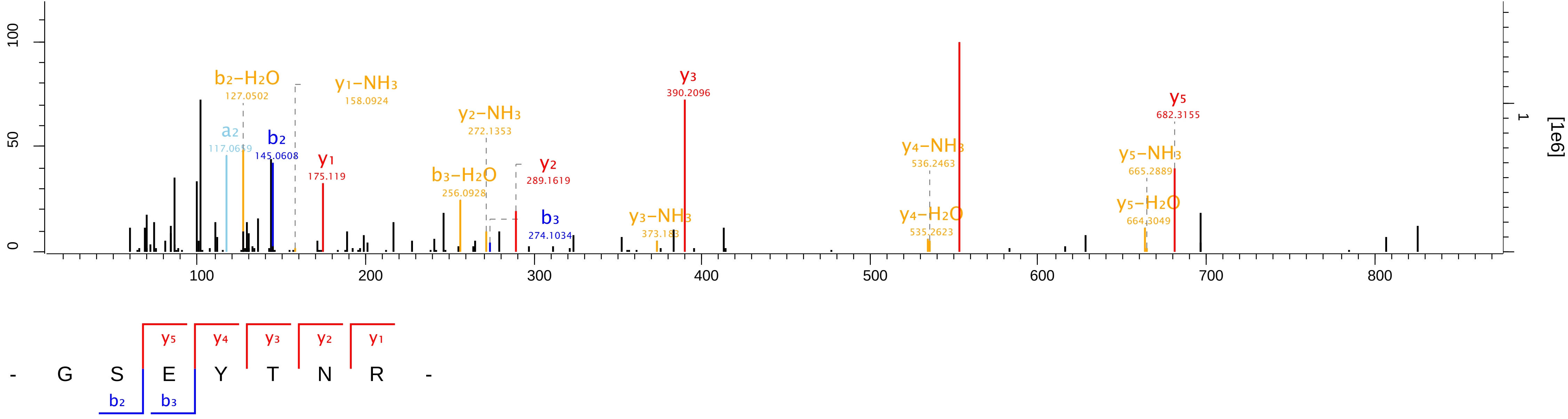

Raw file	Scan	Method	Score	m/z	Gene names
20140602_QEp4_FaHo_SA_SNT	4663	FTMS;	157.7	561.2	TY1B-ML1;TY1B-BR;TY1B-H;TY1B-MR2;TY1B-OR;TY1B-PR2;TY1B-DR5;TY1B-PR1;TY1B-JR2;TY1B-NL1;TY1B-OL;TY1B-LR4;TY1B-ML2;TY1B-PR3;TY1B-PL;TY1B

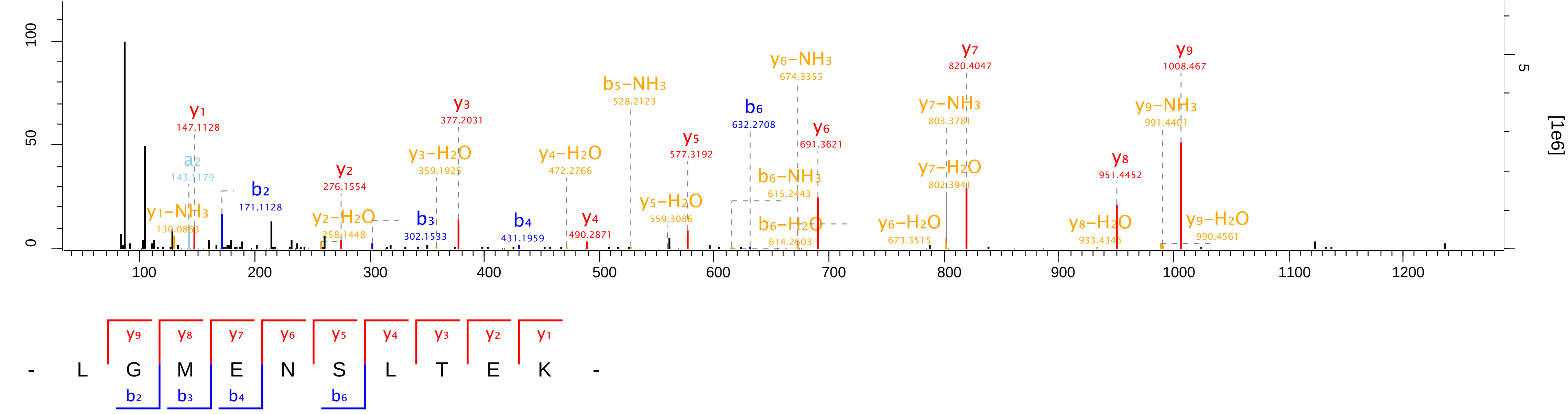

Raw file	Scan	Method	Score	m/z	Gene names
20140602_QEp4_FaHo_SA_SNT2_03	8235	FTMS; HCD	169.65	642.37	RPL31A;RPL31B

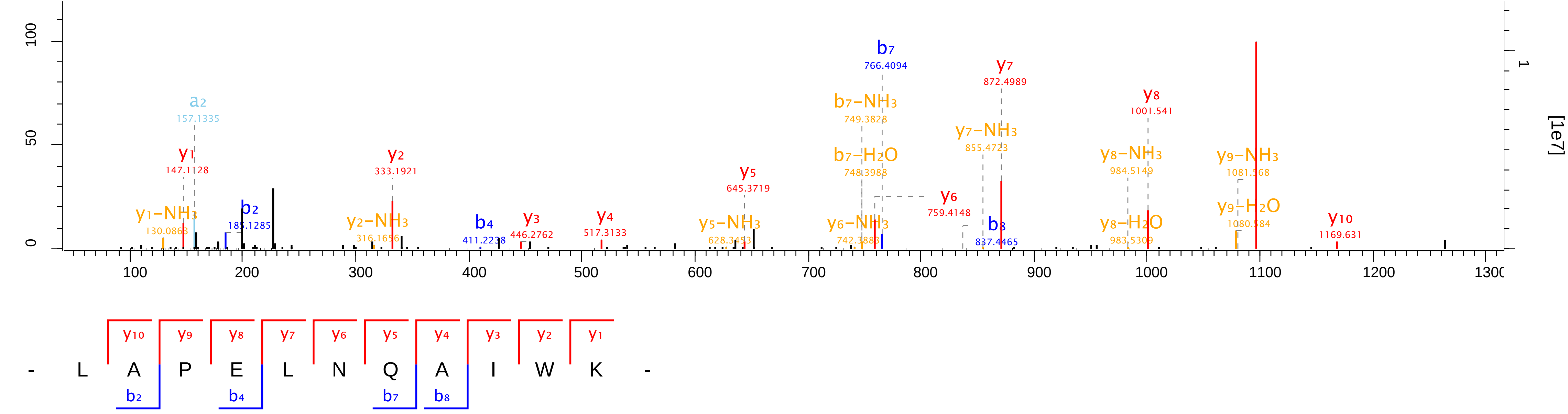

Raw file	Scan	Method	Score	m/z	Gene names
20140602_QEp4_FaHo_SA_SPP1_01	3273	FTMS; HCD	249.91	886.07	TY1B-JR2;TY1B-ML2

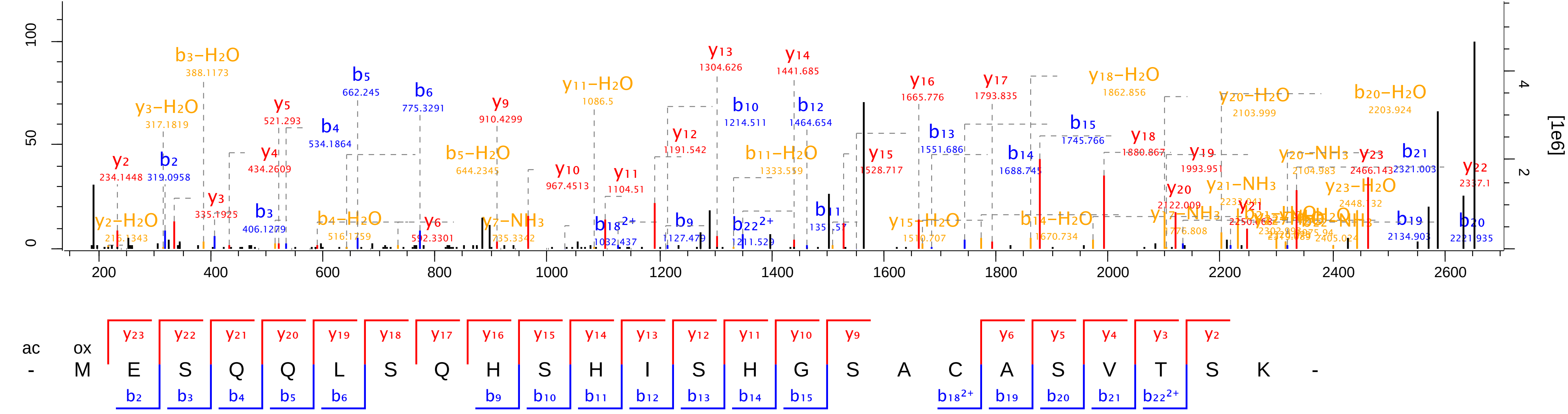

Raw file	Scan	Method	Score	m/z	Gene names
20140602_QEp4_FaHo_SA_SPP1_0	3836	FTMS; HCD	405.88	806.37	TY1A-PR1;TY1A-A;TY1A-DR4;TY1B-PR1;TY1B-JR2;TY1A-PL;TY1A-LR2;TY1A-ER1;TY1A-DR6;TY1B-OL;TY1B-LR4;TY1B-ML2;TY1B-PR3;TY1B-PL;TY1B-L

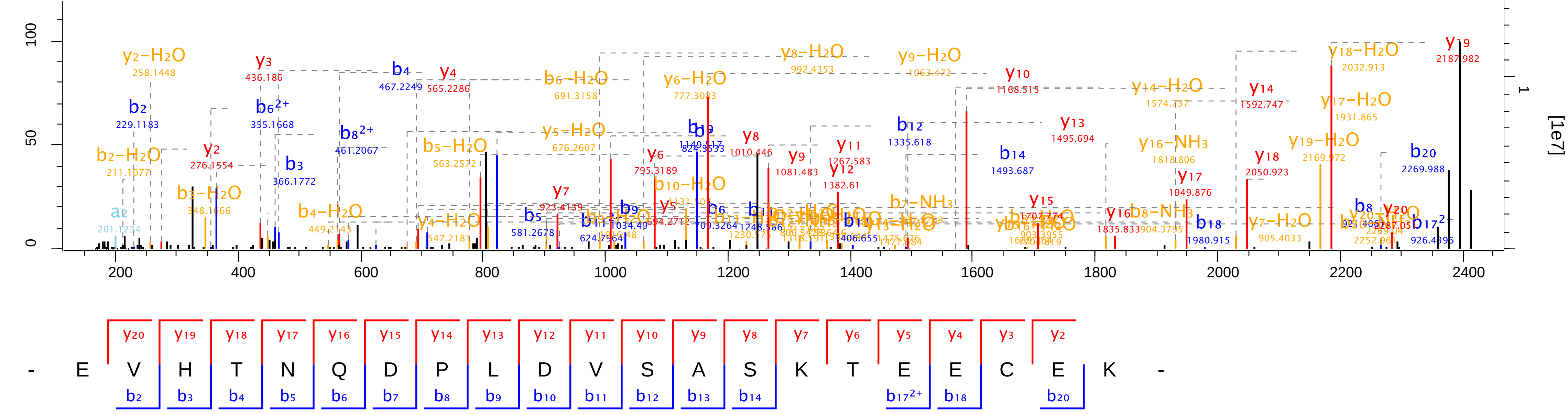

Raw file	Scan	Method	Score	m/z	Gene names
20140602_QEp4_FaHo_SA_SPP1_01	5705	FTMS; HCD	149.53	836.1	TY1A-PR1;TY1A-A;TY1A-DR4

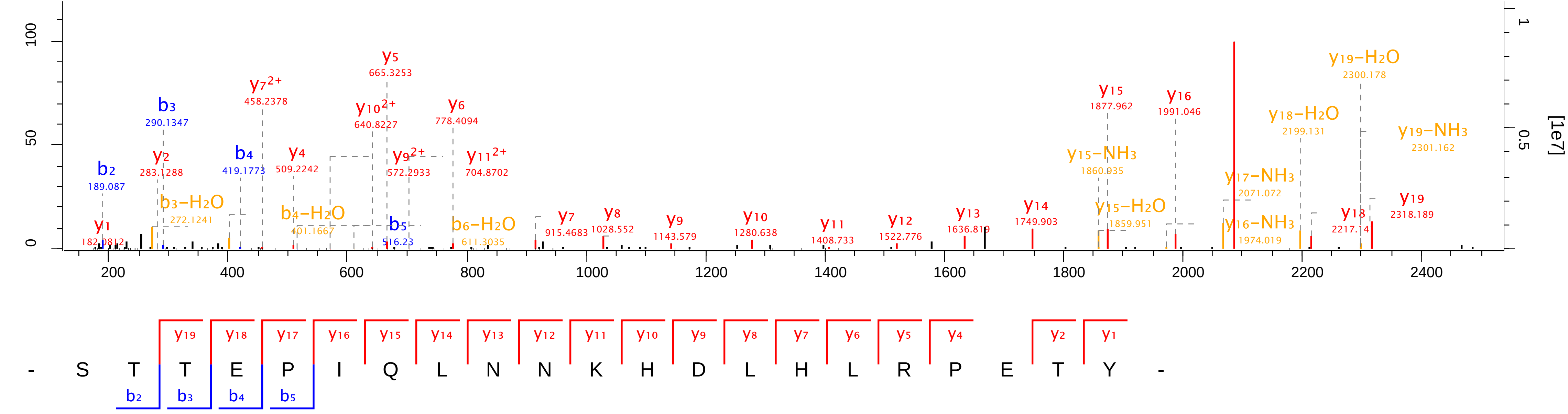

Raw file	Scan	Method	Score	m/z	Gene names
20140602_QEp4_FaHo_SA_S	581	FTMS;	77.7	583.3	TY1B-ML1;TY1B-BR;TY1B-OR;TY1B-DR1;TY1B-NL2;TY1B-PR2;TY1B-DR5;TY1B-PR1;TY1B-JR2;TY1B-NL1;TY1B-OL;TY1B-LR4;TY1B-ML2;TY1B-DR3;TY1B-PR3;TY1B-

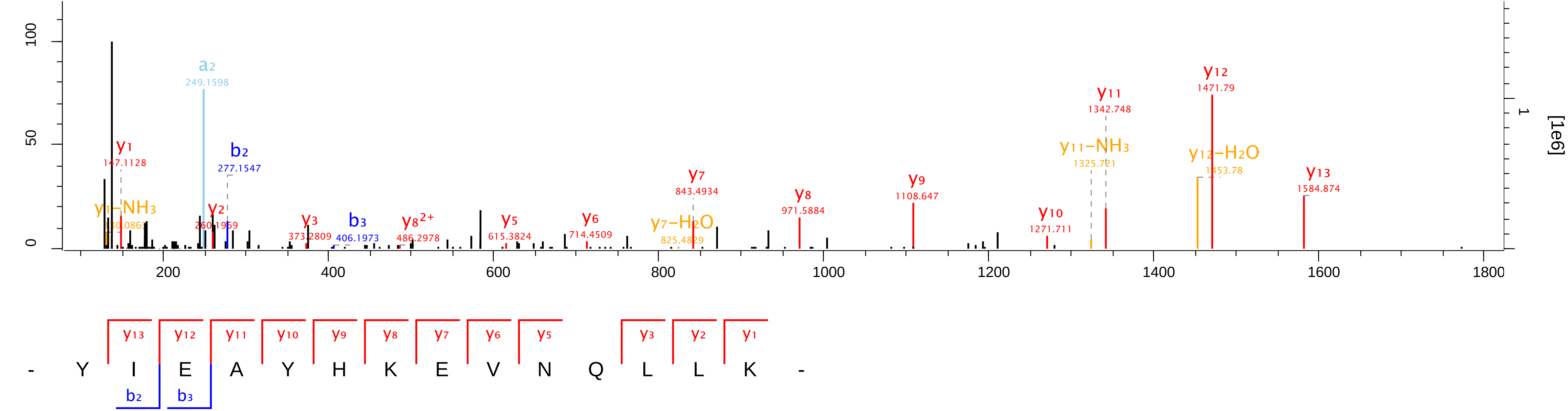

Raw file	Scan	Method	Score	m/z	Gene names
20140602_QEp4_FaHo_SA_SPP1_02	3640	FTMS; HCD	100.02	433.24	RPL7A;RPL7B

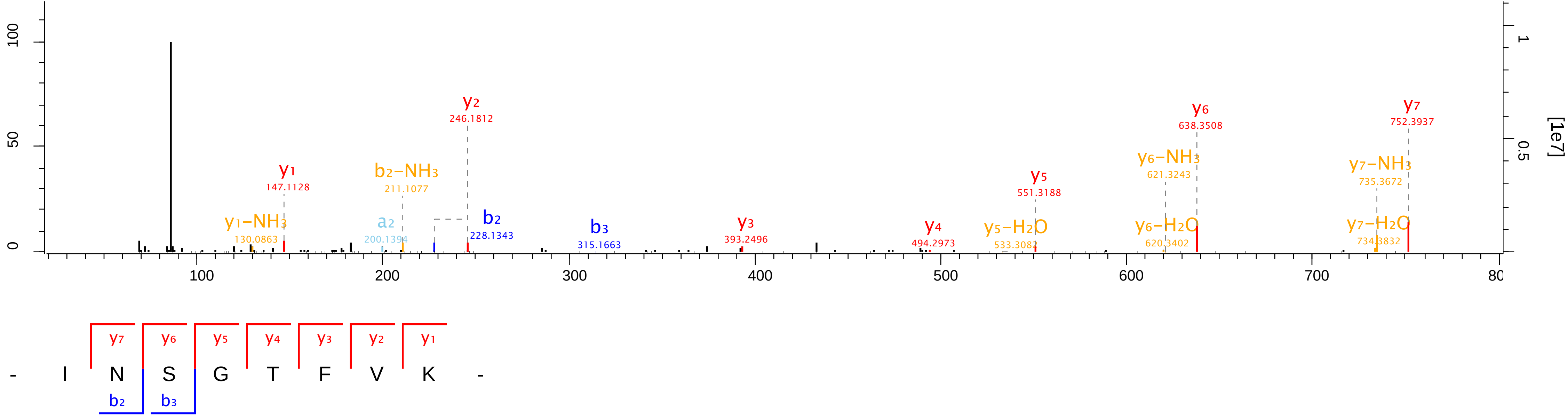

Raw file	Scan	Method	Score	m/z	Gene names
20140602_QEp4_FaHo_SA_SPP1_02	7551	FTMS; HCD	128.75	446.76	RPL14B;RPL14A

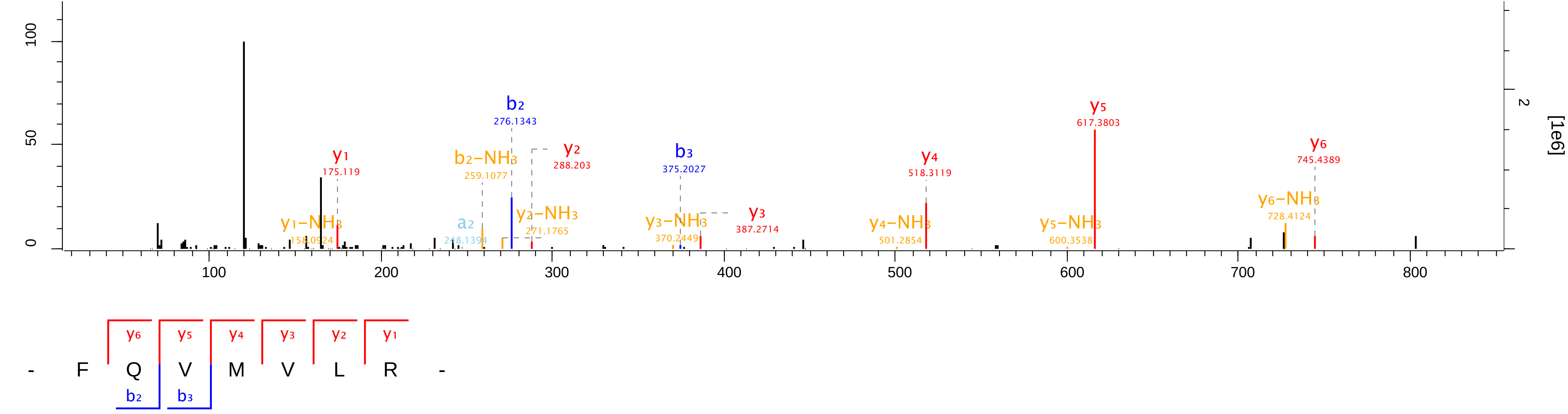

Raw file	Scan	Method	Score	m/z	Gene names
20140602_QEp4_FaHo_SA_SPP1_03	9441	FTMS; HCD	144.27	652.88	TY1B-DR1;TY1B-NL2;TY1B-PR1;TY2B-C;TY1B-LR4;TY1B-DR3;TY1B-DR6;TY2B-B;TY2B-GR2;TY2B-F;TY2B-GR1;TY2B-OR1;TY2B-DR1;TY2B-LR1;TY2[illegible]

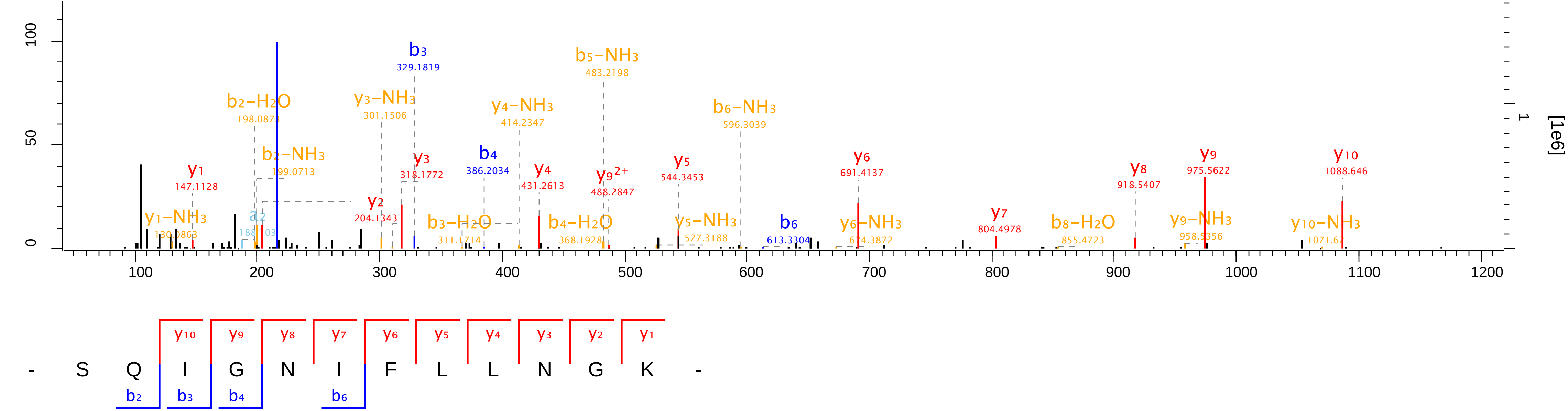

Raw file	Scan	Method	Score	m/z	Gene names
20140602_QEp4_FaHo_SA_SPT7_01	3375	FTMS; HCD	108.92	408.22	RPS14A

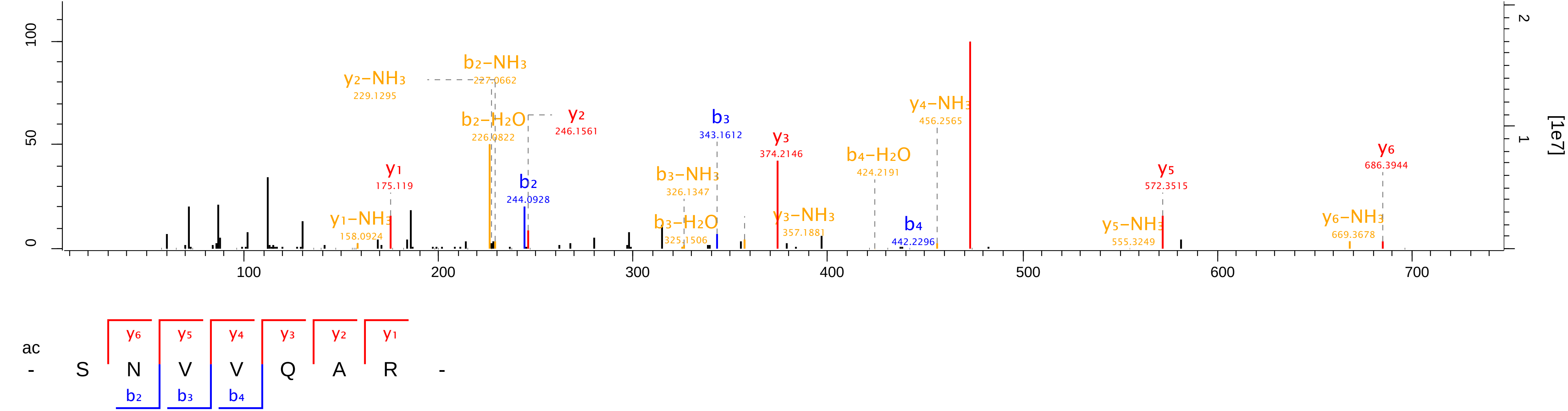

Raw file	Scan	Method	Score	m/z	Gene names
20140602_QEp4_FaHo_SA_SPT7_01	3448	FTMS; HCD	210.73	670.87	RPS14B;RPS14A

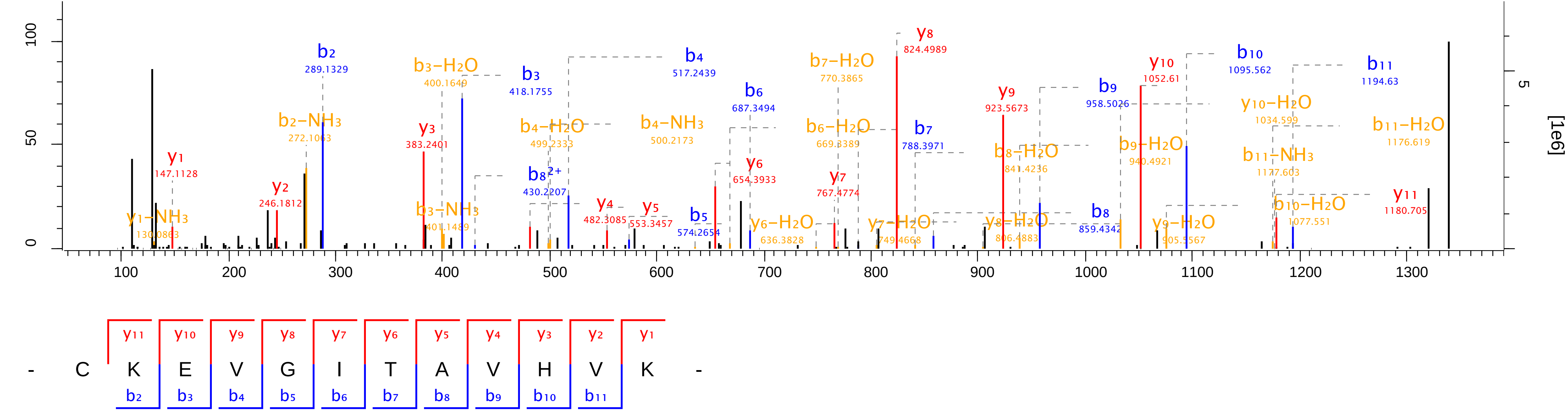

Raw file	Scan Method	Score	m/z	Gene names
20140602_QEp4_FaHo_SA_S 660[illegible]	FTMS;	201.3	821.4	TY1B-ML1;TY1B-BR;TY1B-H;TY1B-MR2;TY1B-OR;TY1B-DR1;TY1B-PR2;TY1B-DR5;TY1B-PR1;TY1B-JR2;TY1B-OL;TY1B-LR4;TY1B-ML2;TY1B-DR3;TY1B-PR3;TY1B-P

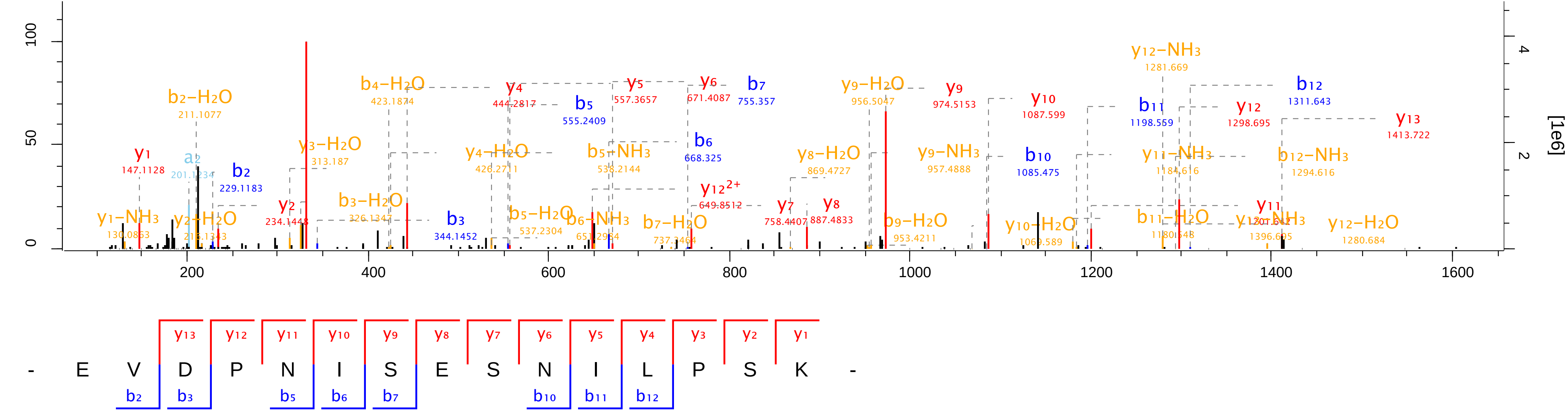

Raw file	Scan	Method	Score	m/z	Gene names
20140602_QEp4_FaHo_SA_SPT7_01	6704	FTMS; HCD	89.86	650.34	SLA1

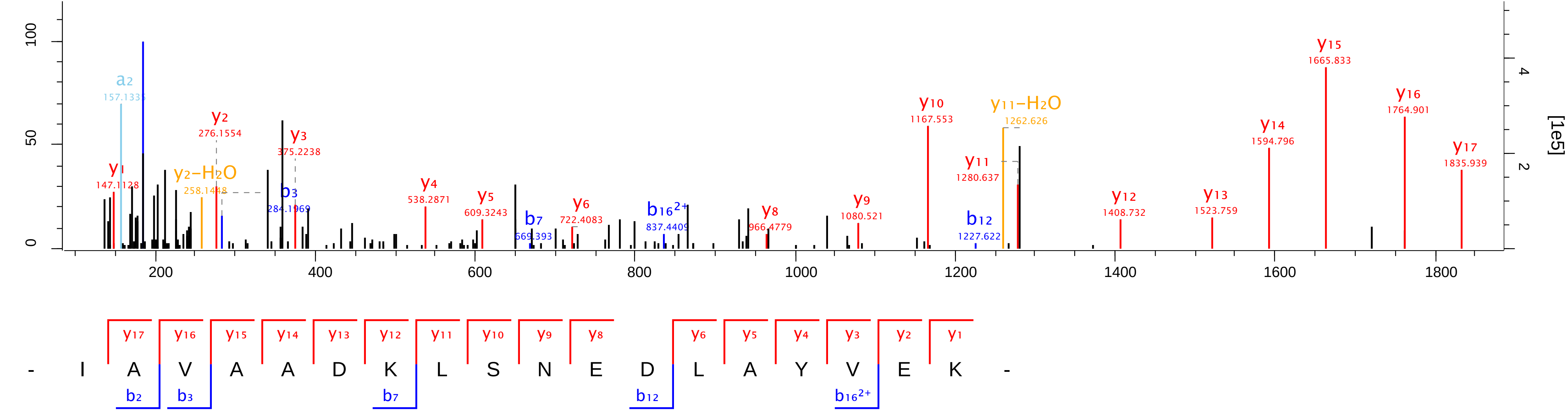

Raw file	Scan	Method	Score	m/z	Gene names
20140602_QEp4_FaHo_SA_SPT7_01	7128	FTMS; HCD	90.7	516.26	PAM16

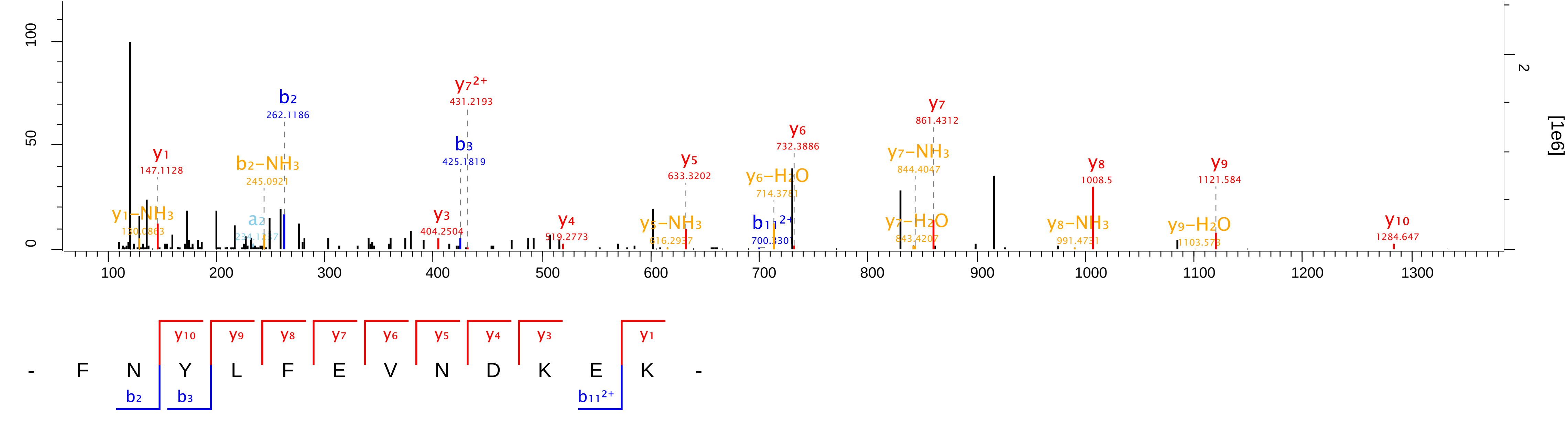

Raw file	Scan	Method	Score	m/z	Gene names
20140602_QEp4_FaHo_SA_SPT7_01	9483	FTMS; HCD	66.89	509.03	NAB6

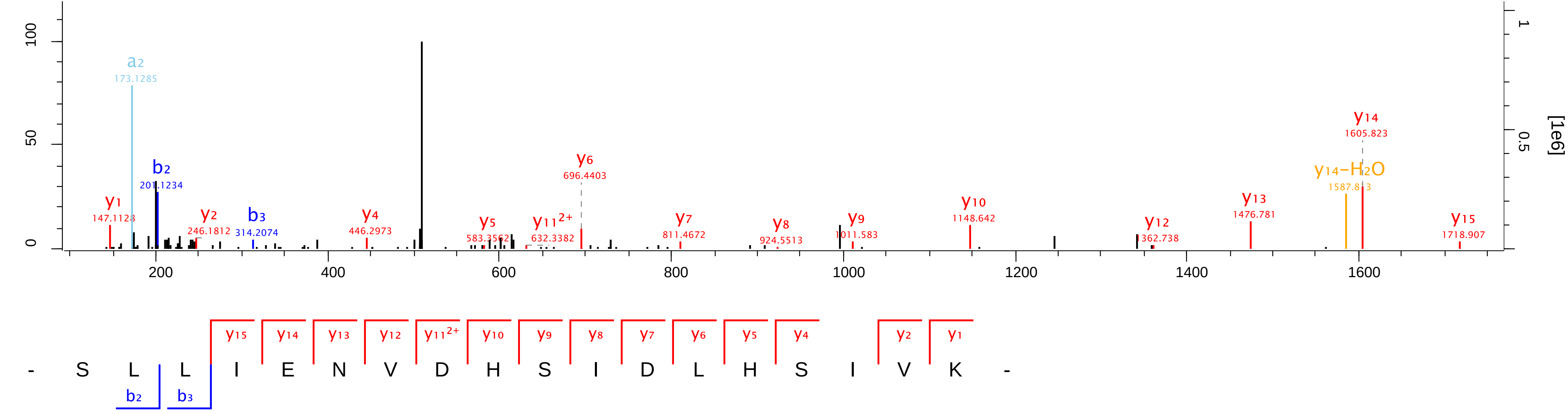

Raw file	Scan	Method	Score	m/z	Gene names
20140602_QEp4_FaHo_SA_SPT7_02	3850	FTMS; HCD	91.07	482.75	TRX2;TRX1

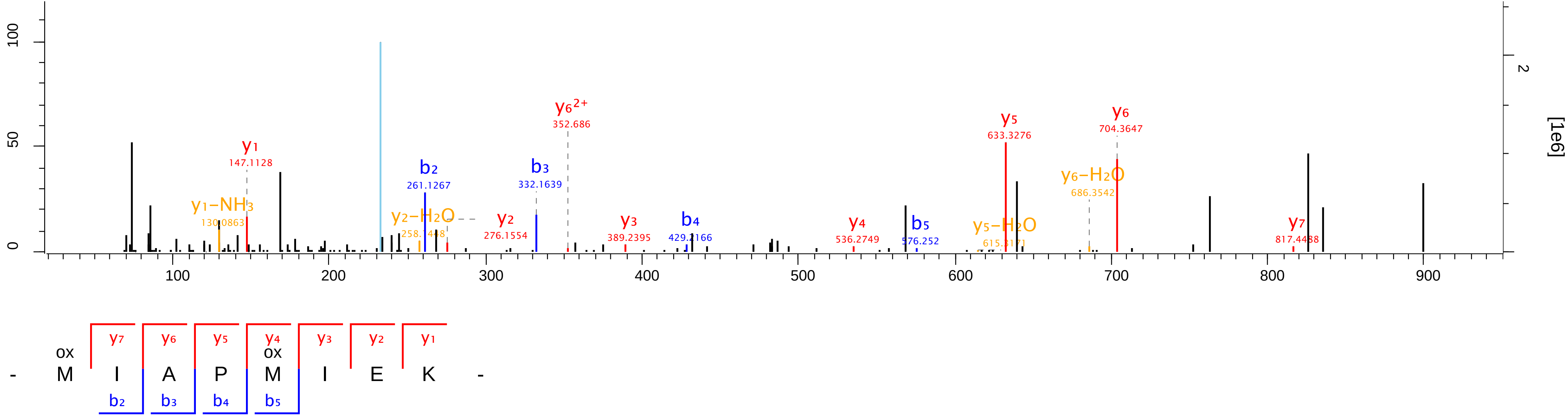

Raw file	Scan	Method	Score	m/z	Gene names
20140602_QEp4_FaHo_SA_SPT7_02	5309	FTMS; HCD	79.47	456.78	RPS0B;RPS0A

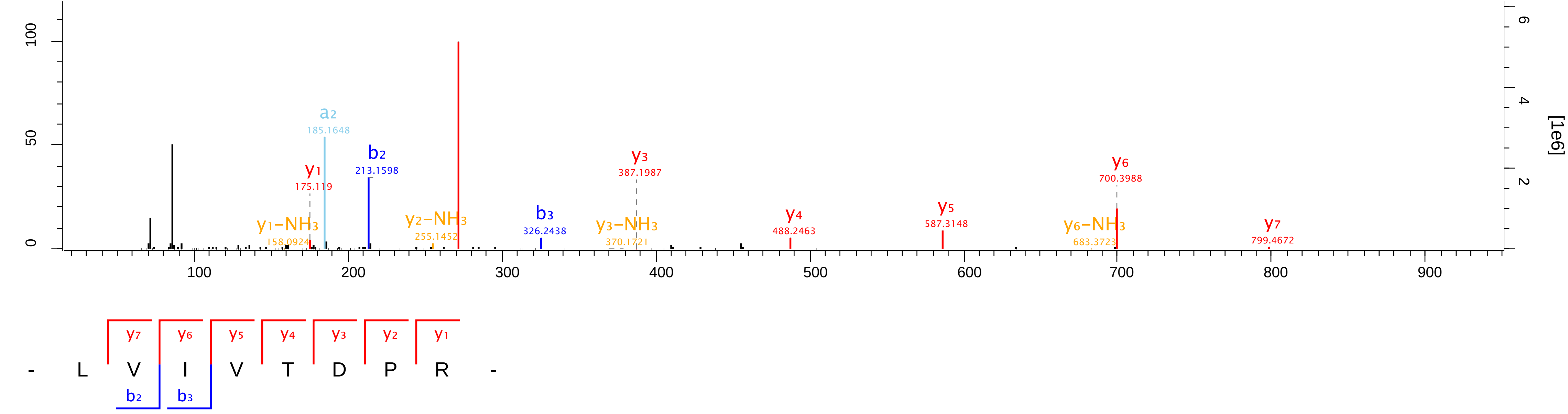

Raw file	Scar	Method	Score	m/z	Gene names
20140602_QEp4_FaHo_S/	854	F FTMS;	64.7(	401.7	TY1B-ML1;TY1B-H;TY1B-MR2;TY1B-DR1;TY1B-NL2;TY1B-PR2;TY1B-DR5;TY1B-PR1;TY2B-C;TY1B-OL;TY1B-ML2;TY1B-DR3;TY1B-PR3;TY1B-PL;TY1B-LR2;TY1B-A;TY2B-

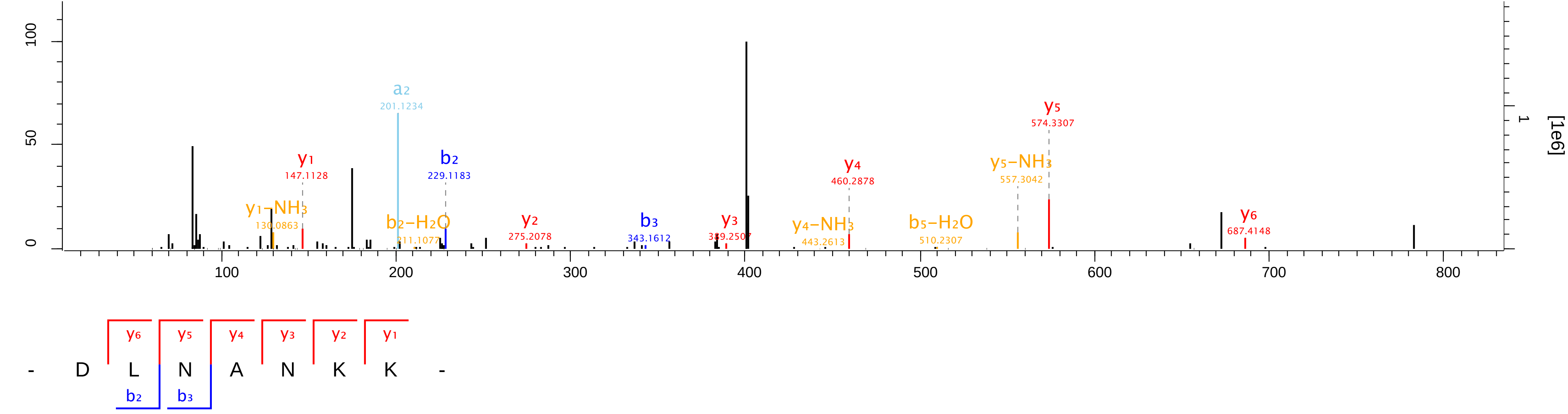

Raw file	Scan	Method	Score	m/z	Gene names
20140602_QEp4_FaHo_SA_SPT7_03	1668	FTMS; HCD	155	502.75	RPL13B;RPL13A

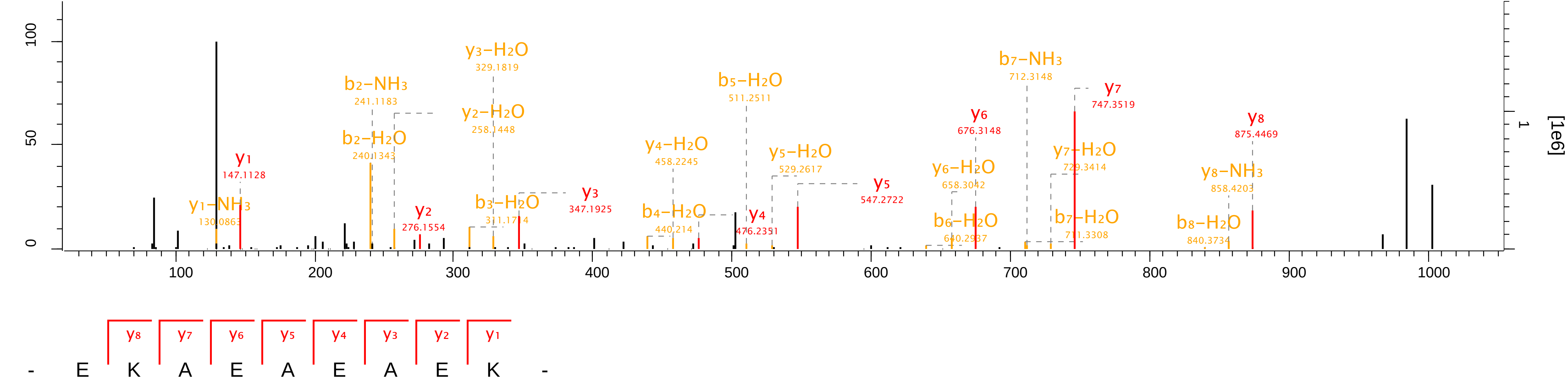

Raw file	Scan	Method	Score	m/z	Gene names
20140602_QEp4_FaHo_SA_SPT7_03	1894	FTMS; HCD	89.89	521.76	TY1B-ML1;TY1B-H;TY1B-PR2;TY1B-DR5;TY1B-PR1;TY1B-OL;TY1B-ML2;TY1B-PR3;TY1B-PL;TY1B-LR2;TY1B-A

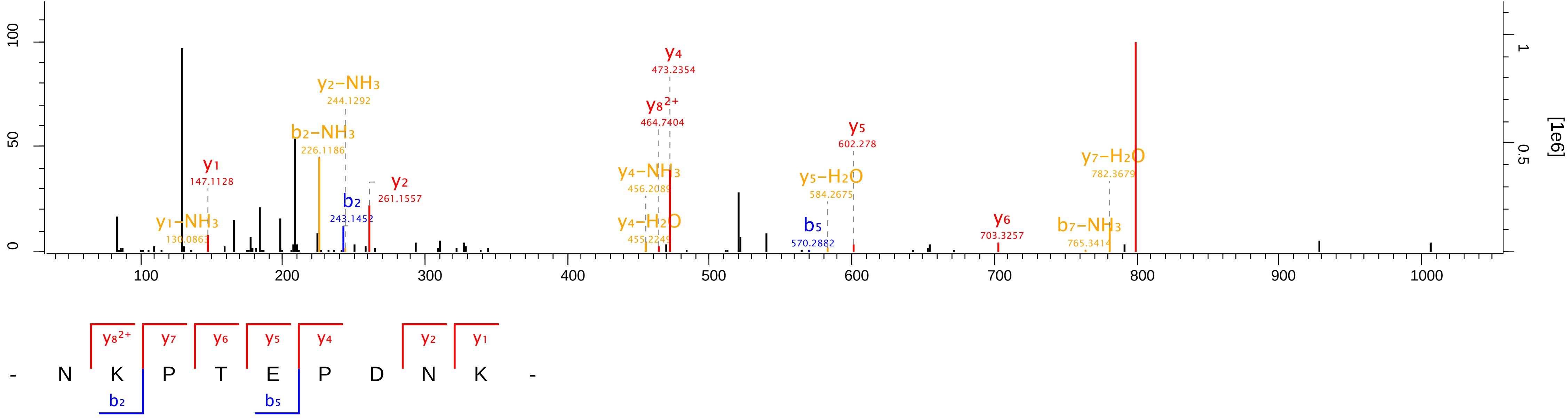

Raw file	Scan	Method	Score	m/z	Gene names
20140602_QEp4_FaHo_SA_SPT7_03	5516	FTMS; HCD	193.54	812.42	TY1A-PL;TY1A-LR2;TY1A-ER1;TY1A-DR6

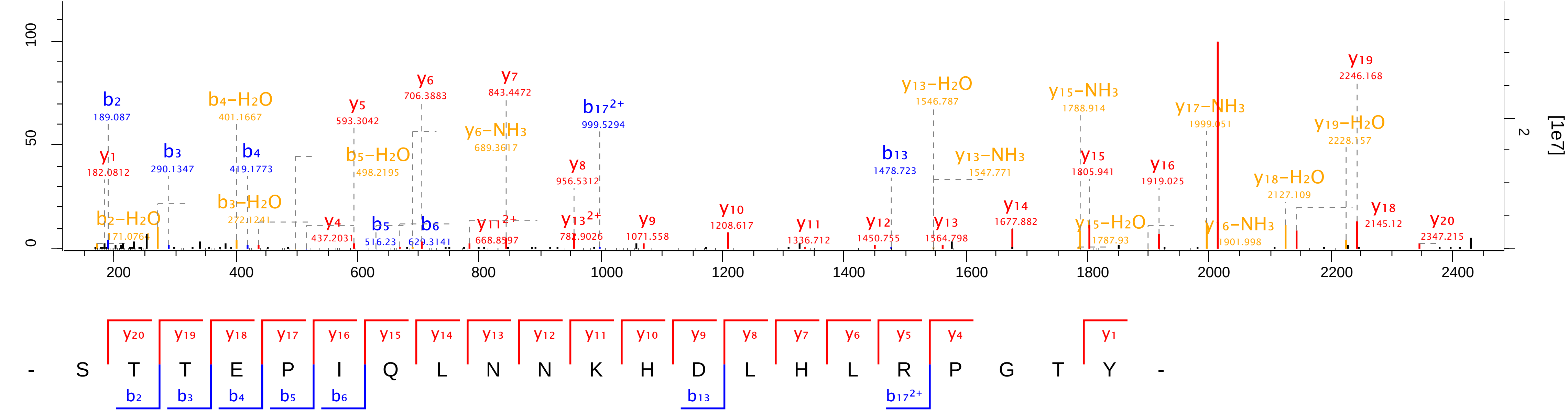

Raw file	Scan	Method	Score	m/z	Gene names
20140602_QEp4_FaHo_SA_SPT7_03	9232	FTMS; HCD	154.72	602.66	RPL9A;RPL9B

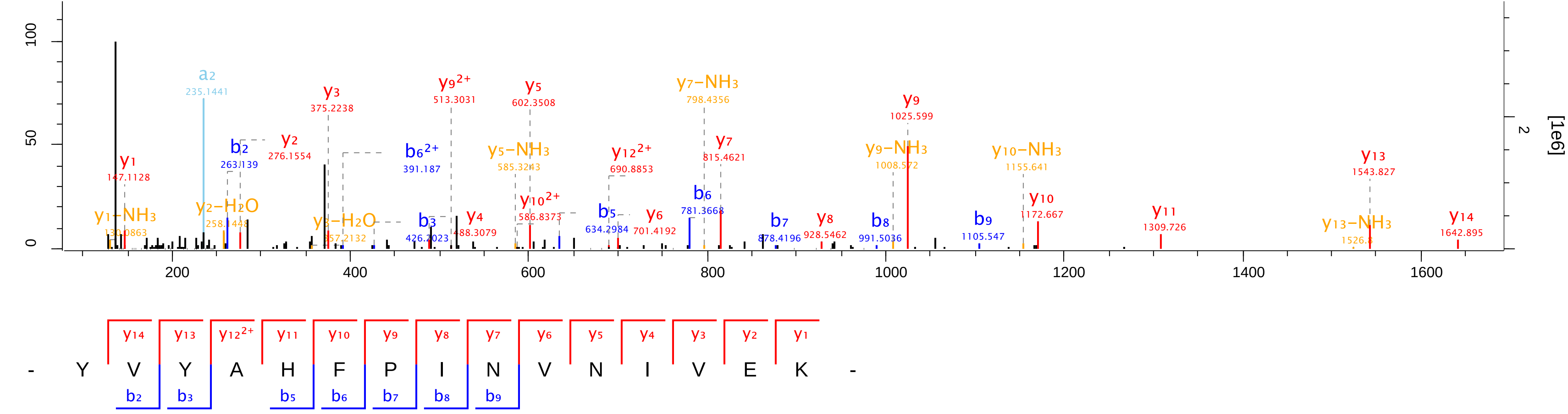

Raw file	Scan	Method	Score	m/z	Gene names
20140602_QEp4_FaHo_SA_SPT7_03	9256	FTMS; HCD	171.08	1035.51	RPC19

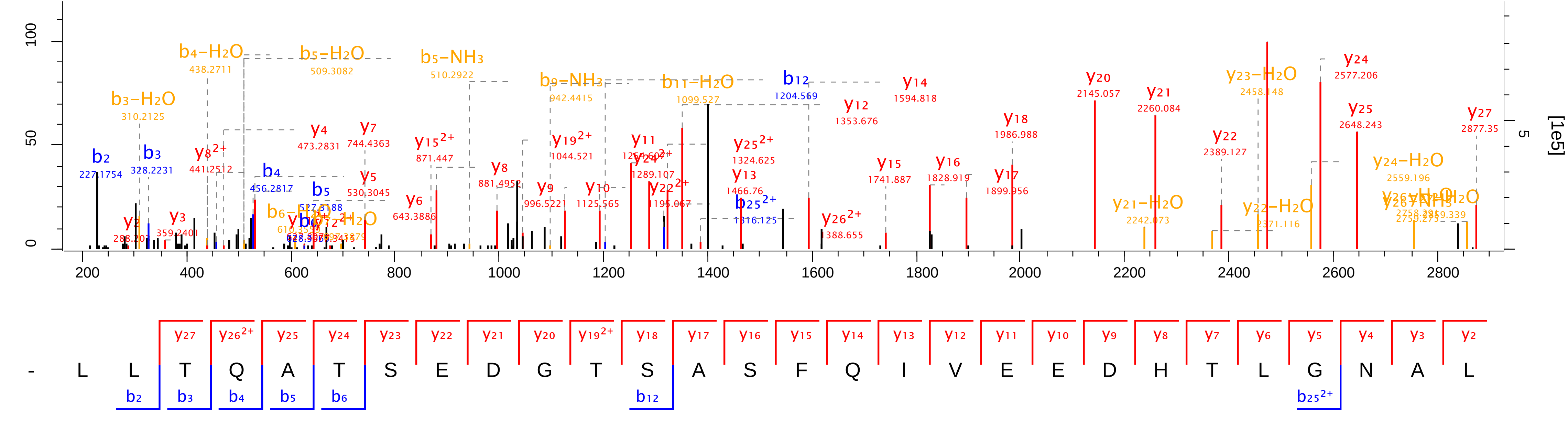

Raw file	Scan	Method	Score	m/z	Gene names
20140602_QEp4_FaHo_SA_SPT7_03	9370	FTMS; HCD	254.68	880.97	TY1B-NL1;TY1A-DR2;TY1B-BL;TY1B-MR1;TY1A-MR1

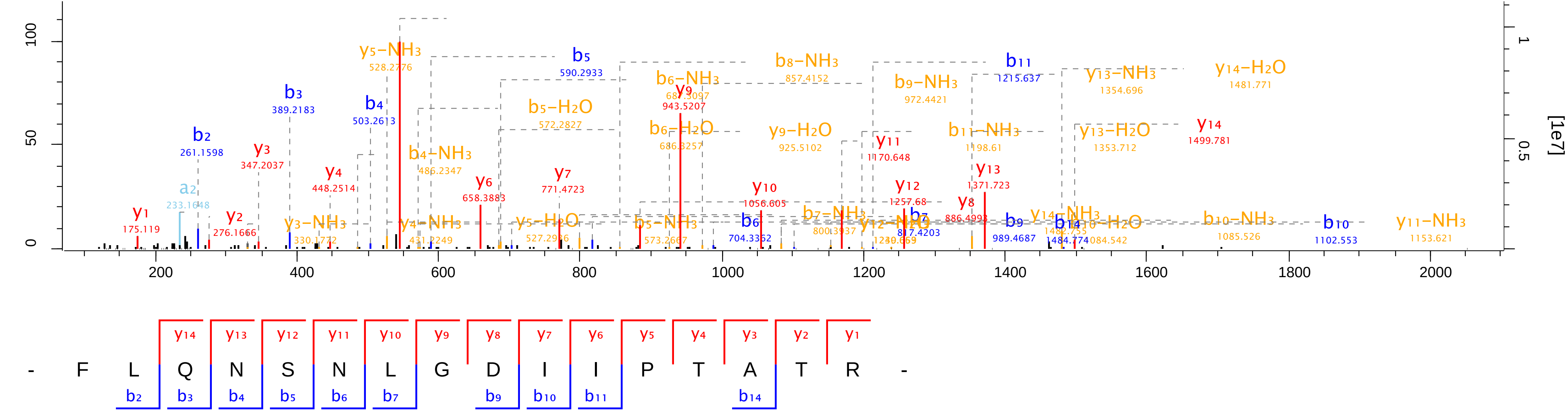

Raw file	Scan	Method	Score	m/z	Gene names
20140602_QEp4_FaHo_SA_SPT7_03	9536	FTMS; HCD	122.46	997.01	RPL7A;RPL7B

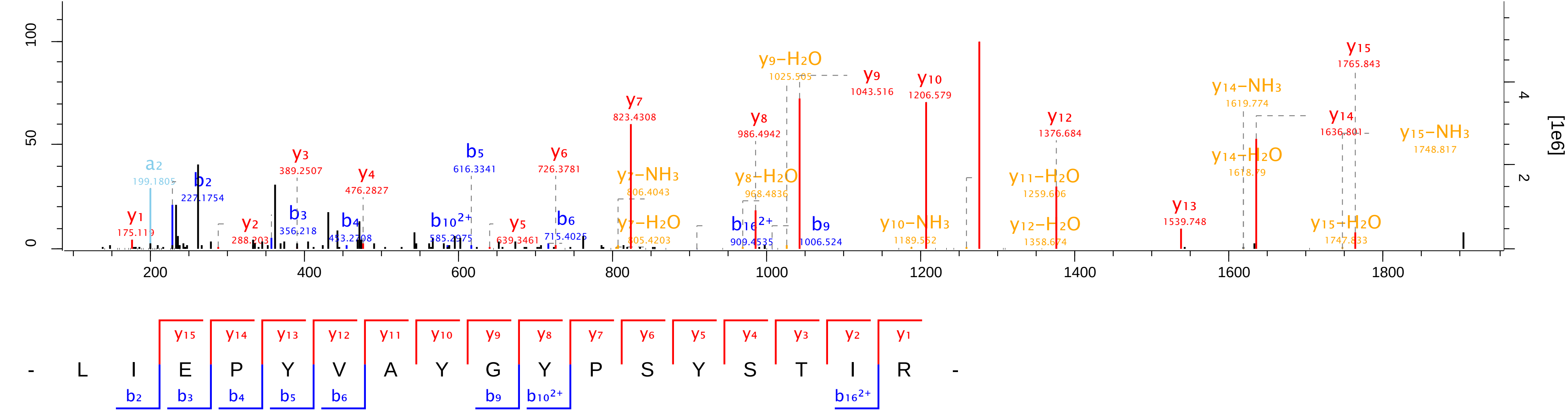

Raw file	Scan	Method	Score	m/z
20140602_QEp4_FaHo_SA_STH1_01	2343	FTMS; HCD	114.02	367.21

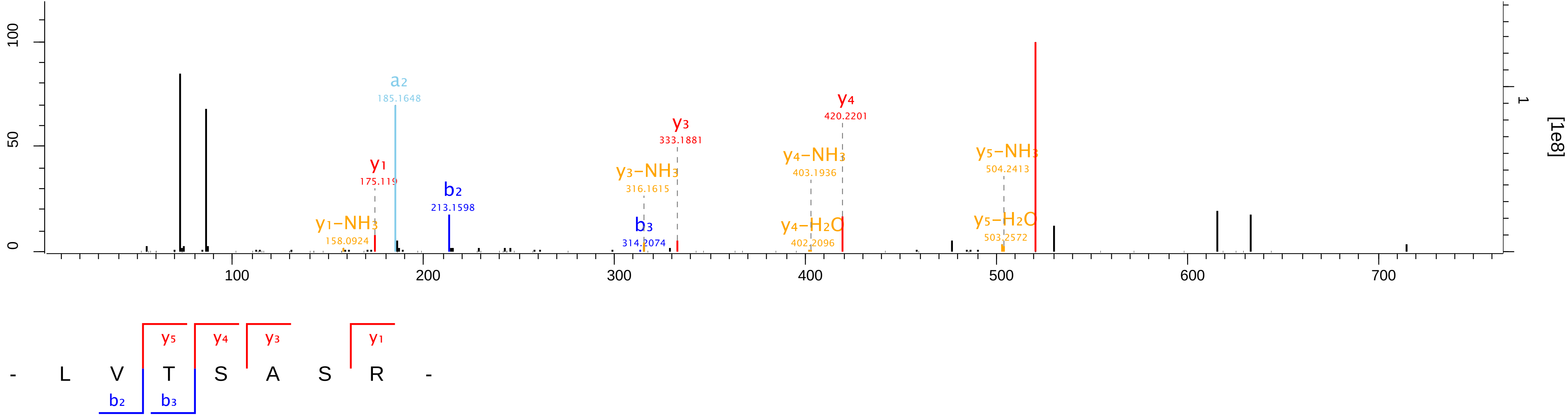

Raw file	Scan	Method	Score	m/z	Gene names
20140602_QEp4_FaHo_SA_STH1_02	5230	FTMS; HCD	169.76	567.79	RPL36B;RPL36A

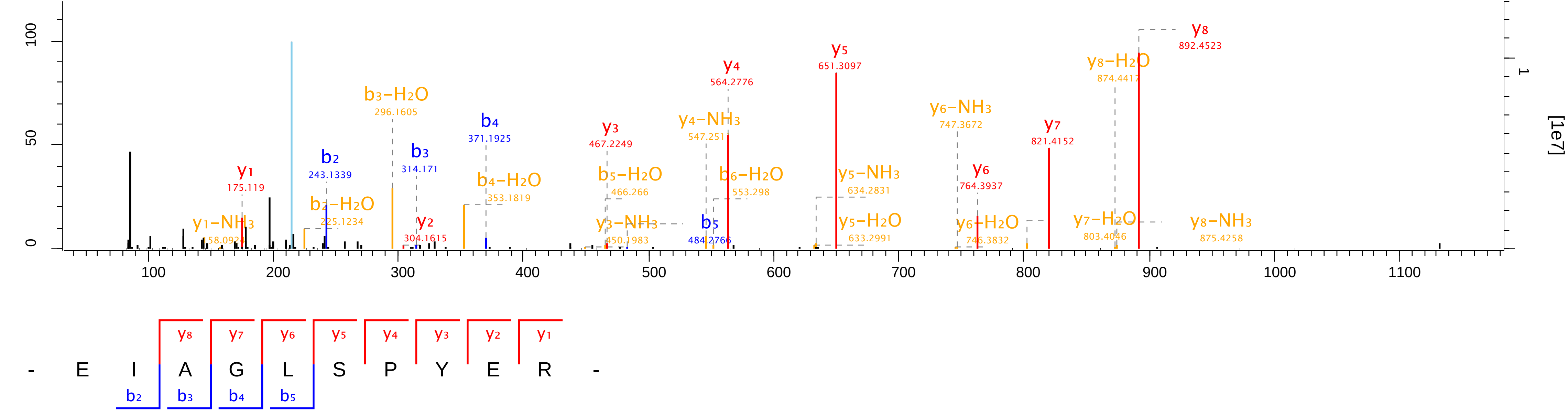

Raw file	Scan	Method	Score	m/z	Gene names
20140602_QEp4_FaHo_SA_STH1_02	8369	FTMS; HCD	164.93	841.42	RPS26A;RPS26B

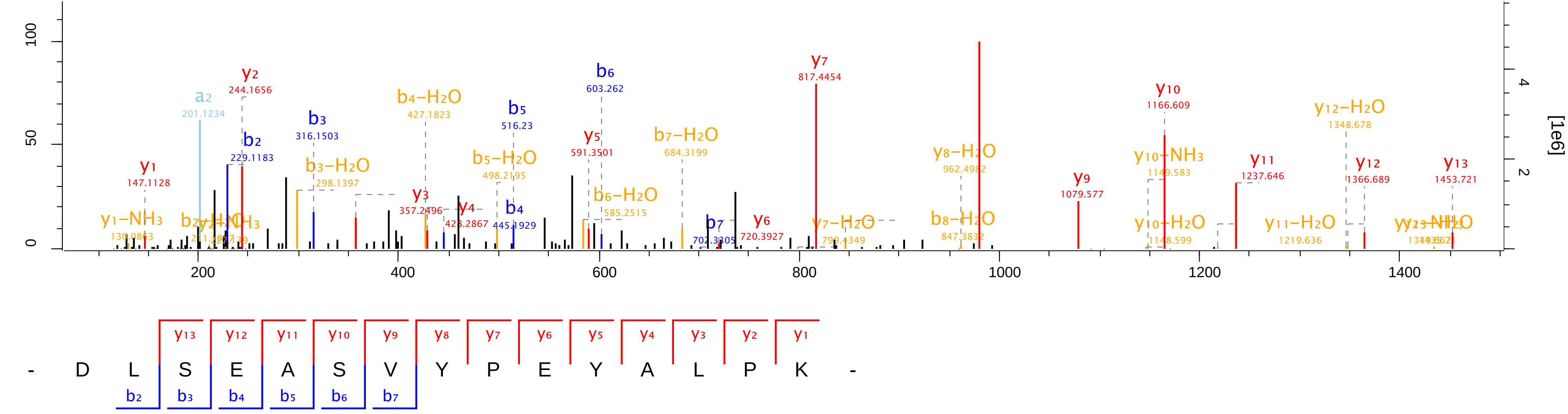

Raw file	Scan	Method	Score	m/z	Gene names
20140602_QEp4_FaHo_SA_SWD3_01	1724	FTMS; HCD	83.78	351.86	TY1B-NL1;TY1A-DR2;TY1B-BL;TY1B-MR1;TY1A-MR1

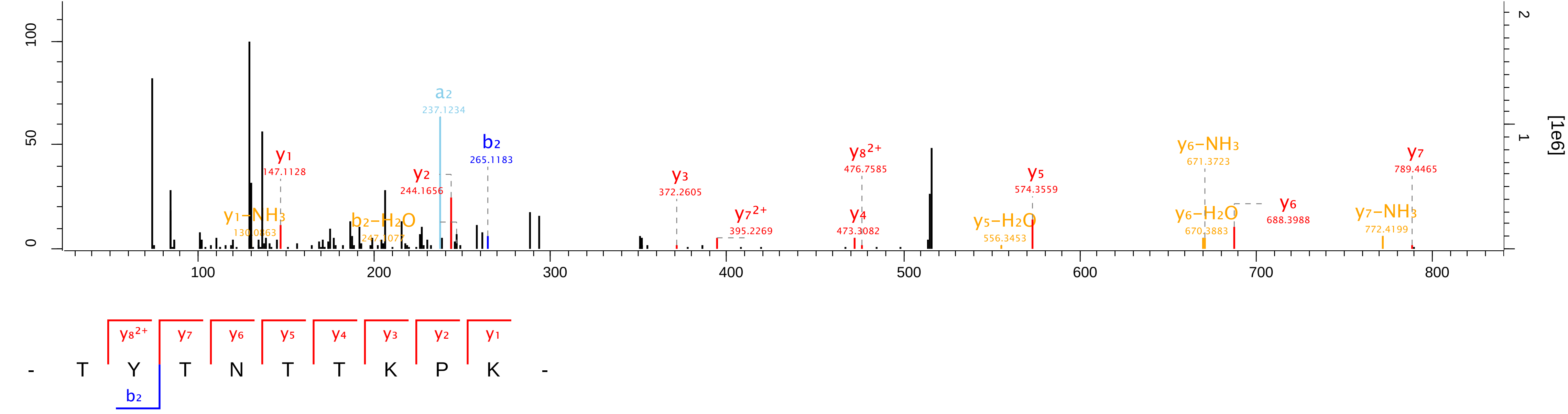

Raw file	Scan	Method	Score	m/z	Gene names
20140602_QEp4_FaHo_SA_44[illegible]	[illegible]	FTMS;	117.9	432.7	TY1B-ML1;TY1B-BR;TY1B-H;TY1B-MR2;TY1B-OR;TY1B-DR1;TY1B-NL2;TY1B-PR2;TY1B-DR5;TY1B-PR1;TY1B-JR2;TY1B-NL1;TY1B-OL;TY1B-LR4;TY1B-ML2;TY1B-DR3

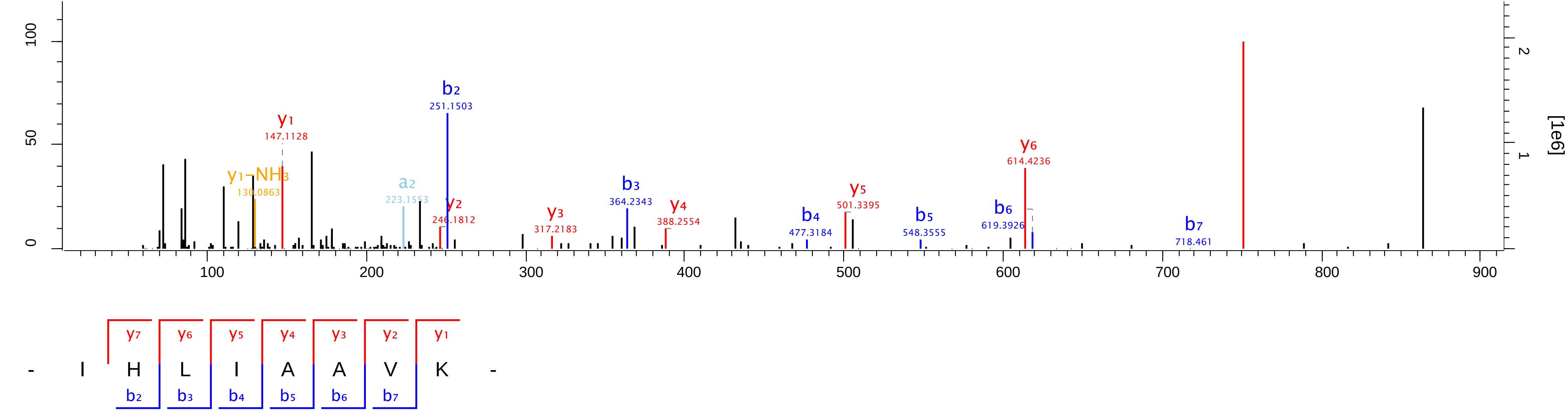

Raw file	Scan	Method	Score	m/z	Gene names
20140602_QEp4_FaHo_SA_SWD3_01	5241	FTMS; HCD	114.64	443.77	RPL36B;RPL36A

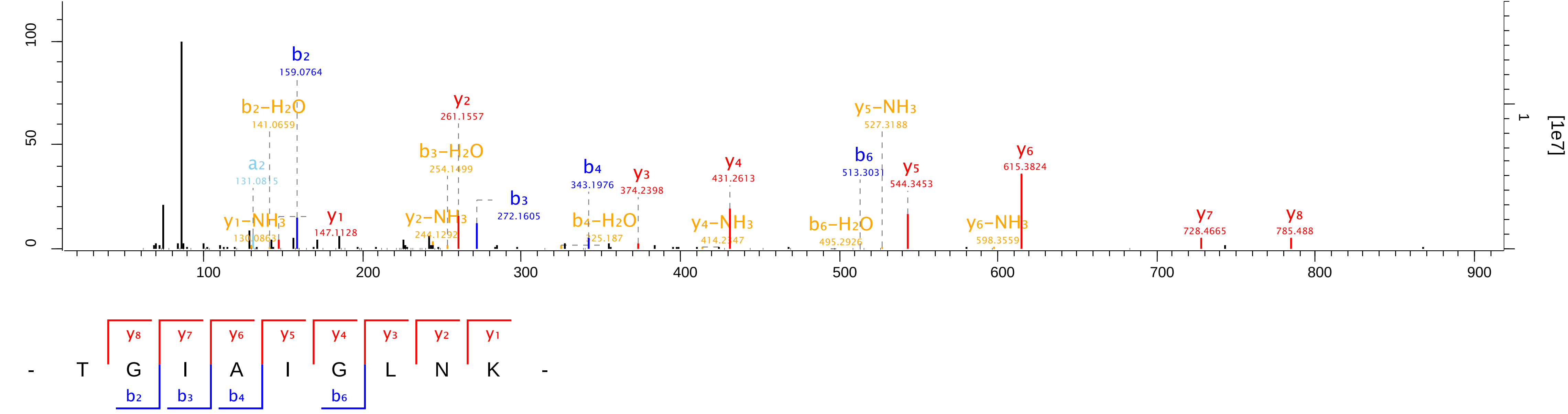

Raw file	Scan	Method	Score	m/z	Gene names
20140602_QEp4_FaHo_SA_SWD3_02	2056	FTMS; HCD	240.78	589.28	TY1A-PL;TY1A-LR2;TY1A-ER1;TY1A-DR6;TY1B-OL;TY1B-LR4;TY1B-ML2;TY1B-PR3;TY1B-PL;TY1B-LR2;TY1B-DR6

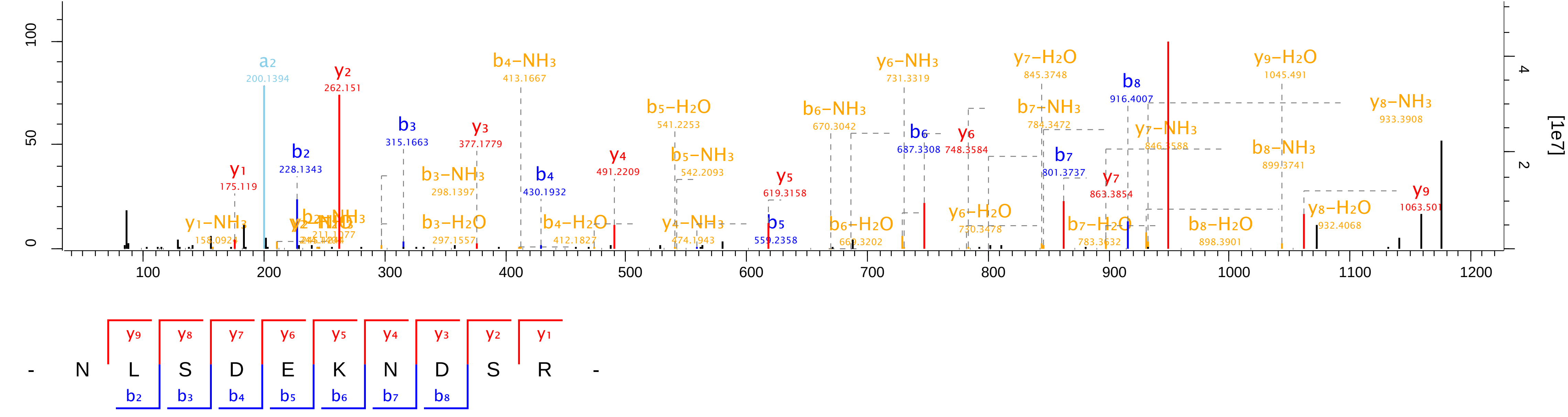

Raw file	Scan	Method	Score	m/z	Gene names
20140602_QEp4_FaHo_[illegible]	956[illegible]	FTMS;[illegible]	94.2	886.[illegible]	TY1B-ML1;TY1B-BR;TY1A-PR1;TY1A-A;TY1A-DR4;TY1B-H;TY1B-MR2;TY1B-OR;TY1B-DR1;TY1B-NL2;TY1B-PR2;TY1B-DR5;TY1B-PR1;TY1B-JR2;TY1A-PL;TY1A-LR2;TY1A-E[illegible]

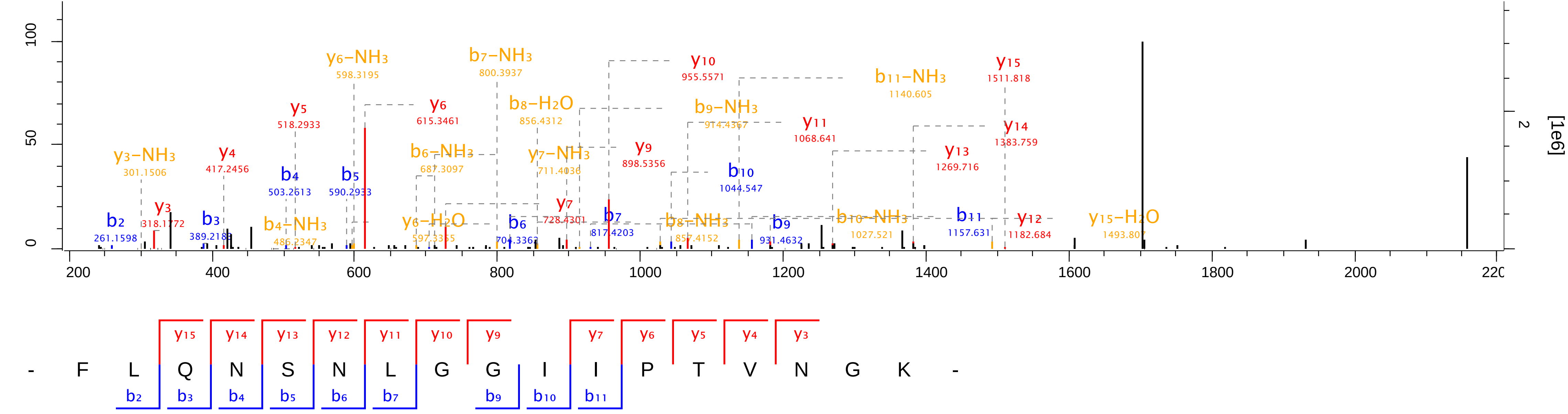

Raw file	Scan	Method	Score	m/z	Gene names
20140602_QEp4_FaHo_SA_SWD3_	3061	FTMS;	163.515	10.93	TY1B-ML1;TY1B-MR2;TY1B-OR;TY1B-DR1;TY1B-PR2;TY1B-DR5;TY1B-PR1;TY1B-JR2;TY1B-OL;TY1B-LR4;TY1B-ML2;TY1B-DR3;TY1B-PR3;TY1B-PL;TY1B-L

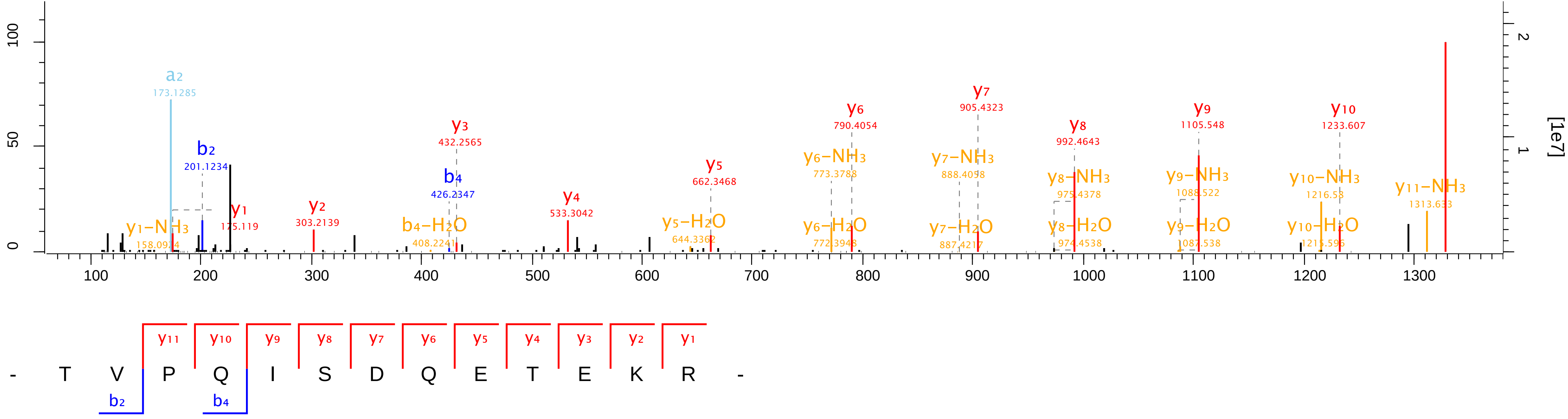

Raw file	Scan	Method	Score	m/z	Gene names
20140602_QEp4_FaHo_SA_SWD3_03	3212	FTMS; HCD	69.82	391.88	RPL31A;RPL31B

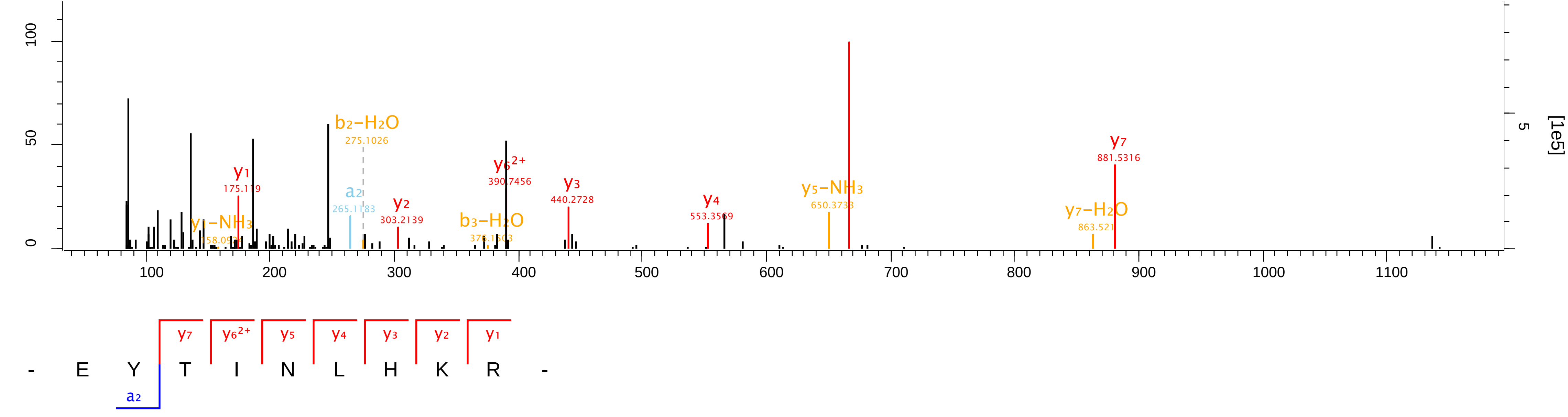

Raw file	Scan	Method	Score	m/z	Gene names
20140602_QEp4_FaHo_SA_SWD3_(	3506	FTMS; HC	225.26	701.83	TY1A-PR1;TY1A-A;TY1A-DR4;TY1B-PR1;TY1B-JR2;TY1A-PL;TY1A-LR2;TY1A-ER1;TY1A-DR6;TY1B-OL;TY1B-LR4;TY1B-ML2;TY1B-PR3;TY1B-PL;TY1B-L

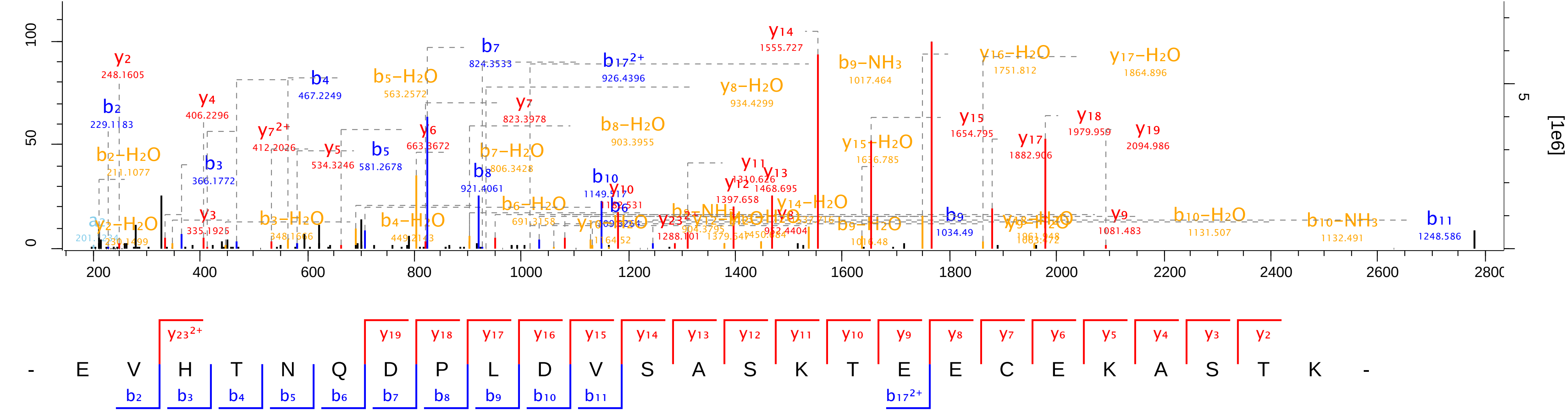

Raw file	Scar Method	Score	m/z	Gene names
20140602_QEp4_FaHo_SA_38	19 FTMS;	117.9	507.5	TY1B-ML1;TY1B-BR;TY1B-H;TY1B-MR2;TY1B-OR;TY1B-DR1;TY1B-NL2;TY1B-PR2;TY1B-DR5;TY1B-PR1;TY1B-JR2;TY1B-NL1;TY1B-OL;TY1B-LR4;TY1B-ML2;TY1B-DR3

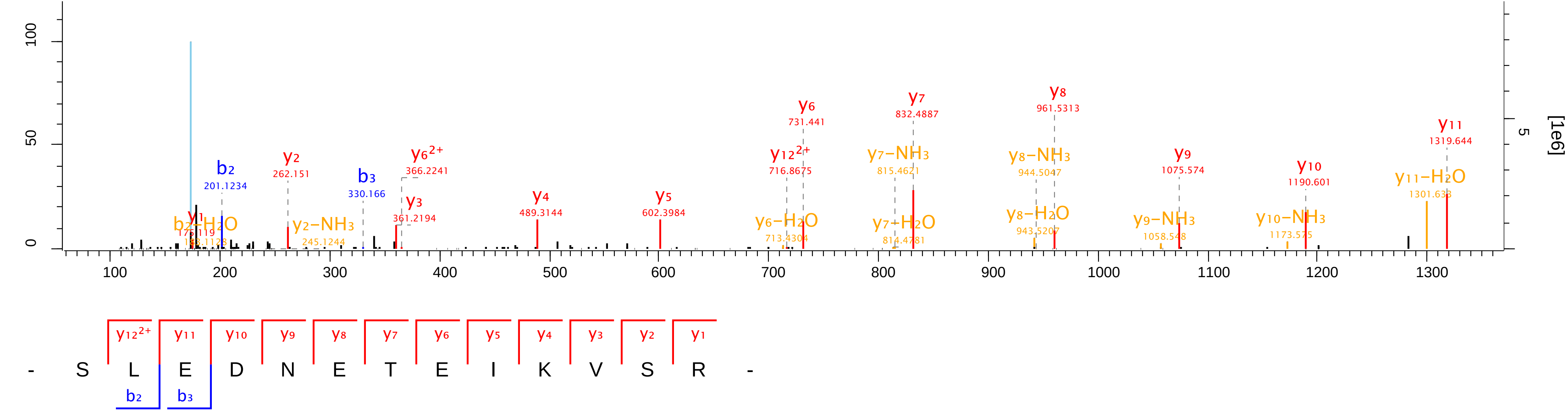

Raw file	Scan	Method	Score	m/z	Gene names
20140602_QEp4_FaHo_SA_SWD3_03	6130	FTMS; HCD	94.27	754.87	TY1B-ML1;TY1B-BR;TY1B-H;TY1B-MR2;TY1B-OR;TY1B-NL2;TY1B-PR2;TY1B-DR5;TY1B-PR1;TY1B-JR2;TY1B-DR3

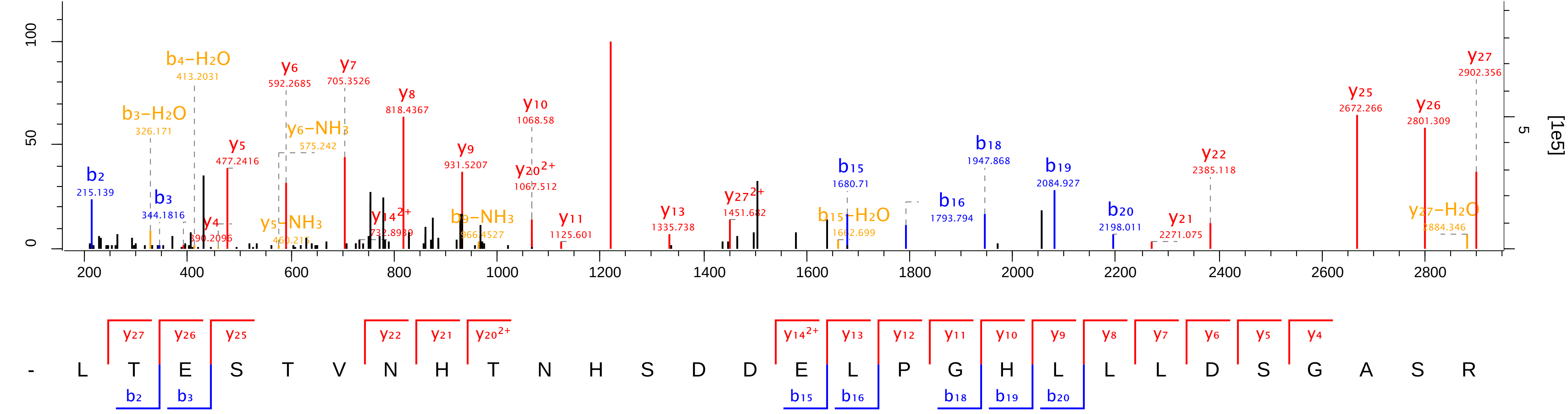

Raw file Sca Metho Scoı m/z Gene names

20140602_QEp4_FaH‹ 63⁄ FTMS 93 123 550. TY1B-ML1;TY1B-BR;TY1B-H;TY1B-MR2;TY1B-OR;TY1B-DR1;TY1B-NL2;TY1B-PR2;TY1B-DR5;TY1B-PR1;TY1B-JR2;TY1B-NL1;TY2B-C;TY1B-OL;TY1B-LR4;TY1B-ML2;TY1B-DR3;T

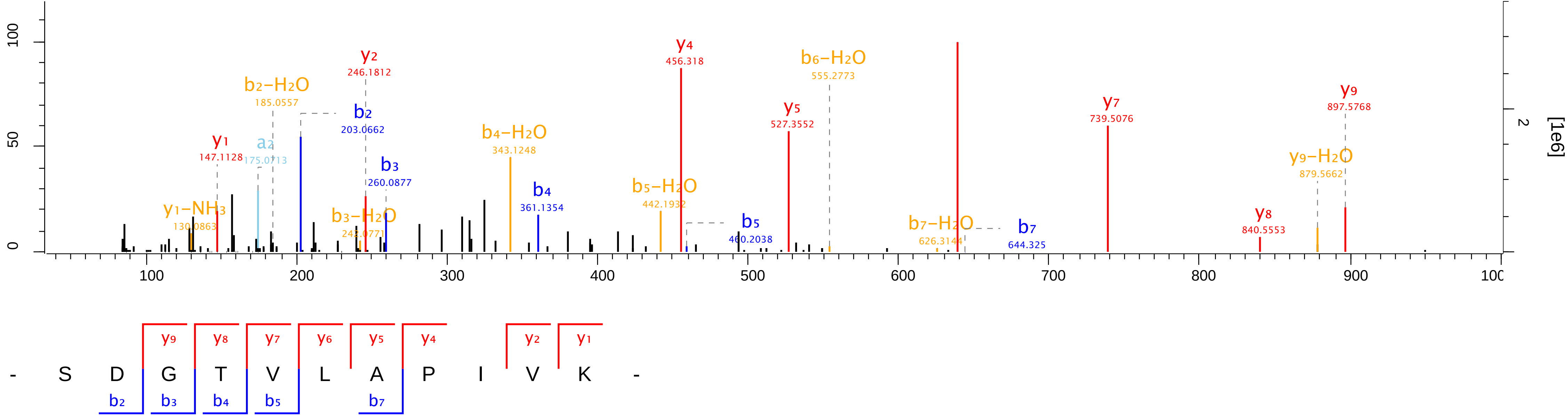

Raw file	Scan	Method	Score	m/z
20140602_QEp4_FaHo_SA_SWI3_01x	6161	FTMS; HCD	6.29	635.79

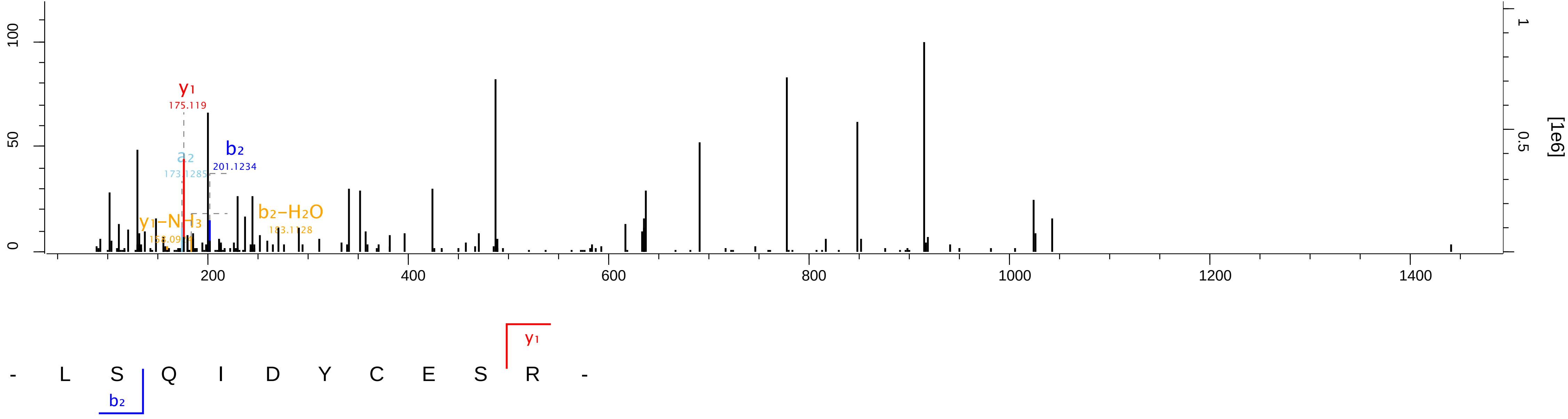

Raw file	Scan	Method	Score	m/z	Gene names
20140602_QEp4_FaHo_SA_SWI3_01x	6612	FTMS; HCD	57.48	587.3	RPL17A;RPL17B

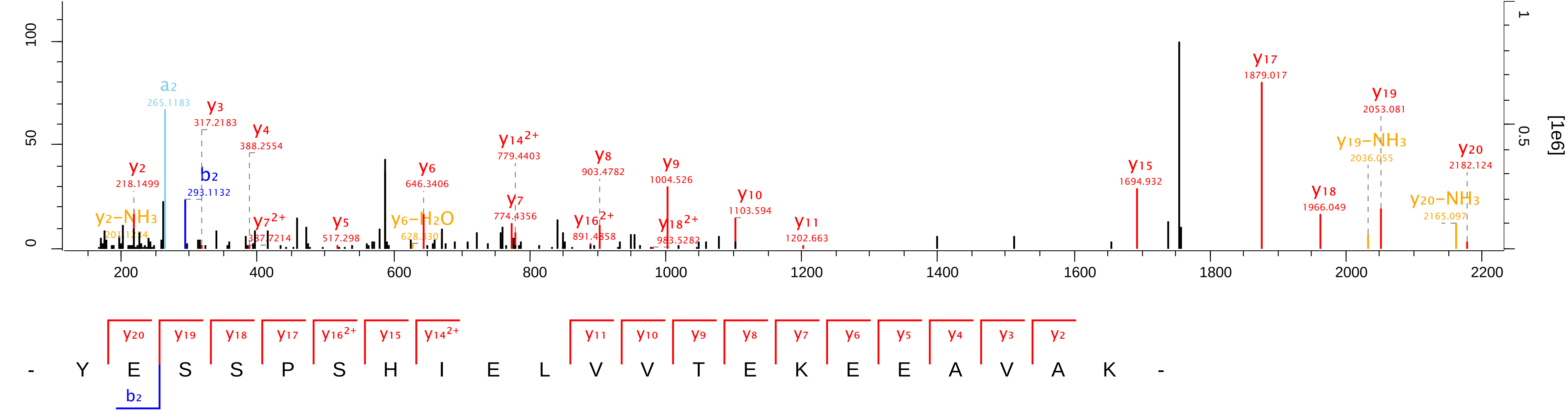

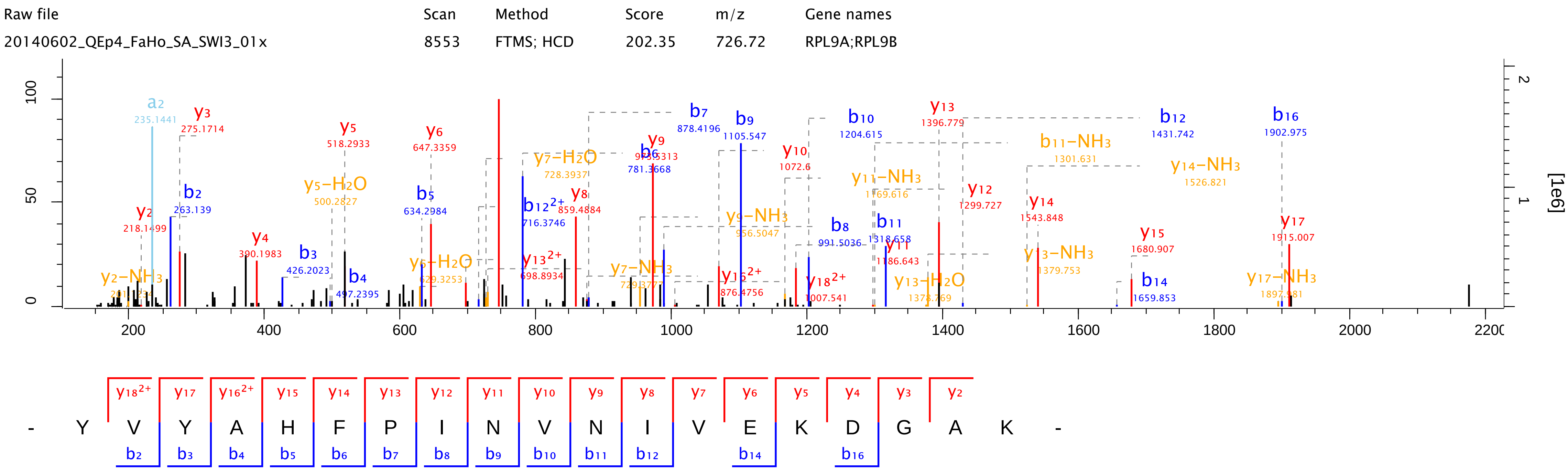
Raw file
20140602_QEp4_FaHo_SA_SWI3_01x
Scan
8553
Method
FTMS; HCD
Score
202.35
m/z
726.72
Gene names
RPL9A;RPL9B
[1e6]
- Y V Y A H F P I N V N I V E K D G A K -

Raw file	Scan	Method	Score	m/z
20140602_QEp4_FaHo_SA_SWI3_01x	8848	FTMS; HCD	62.03	703.34

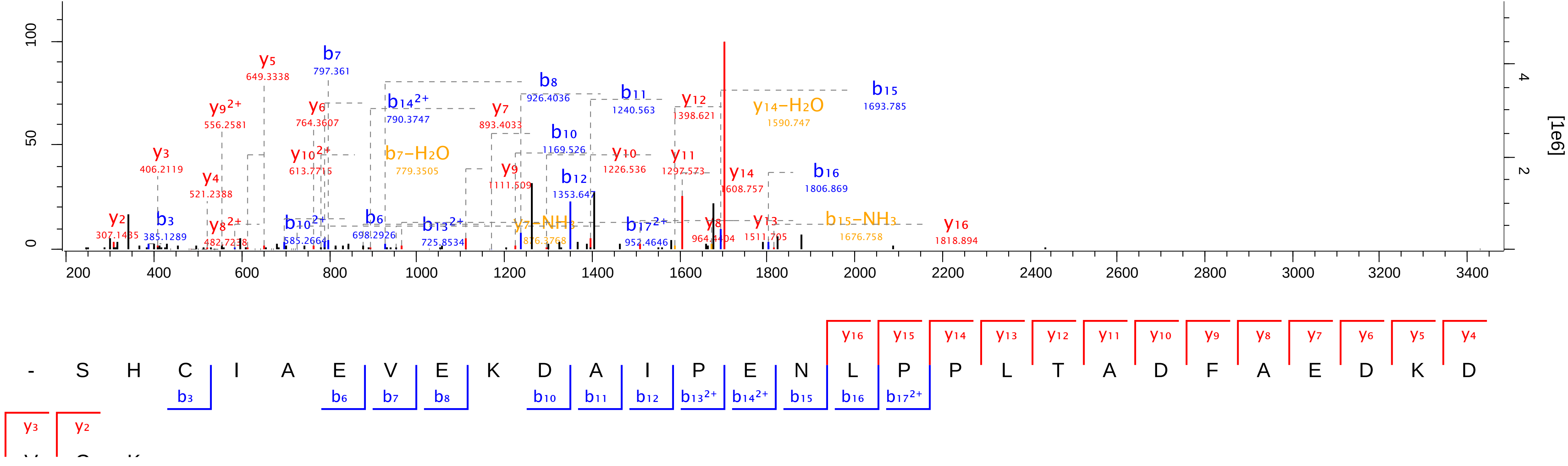

Raw file	Scan	Method	Score	m/z	Gene names
20140602_QEp4_FaHo_SA_SWI3_01x	9215	FTMS; HCD	68.66	881.1	TIM23

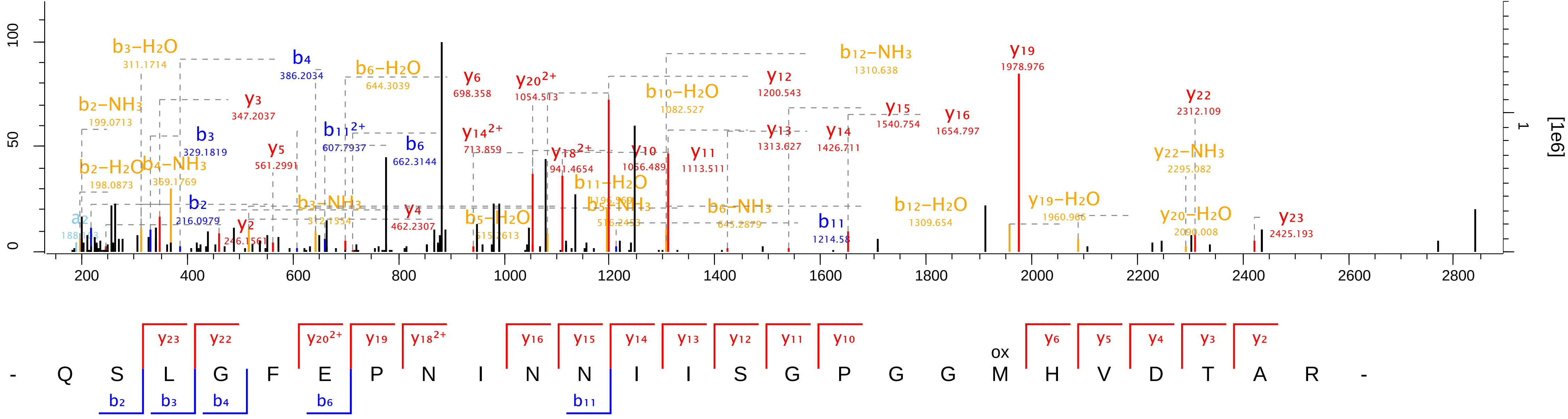

Raw file	Scan	Method	Score	m/z	Gene names
20140602_QEp4_FaHo_SA_SWI3_02	3257	FTMS; HCD	152.7	597.28	RPL13B;RPL13A

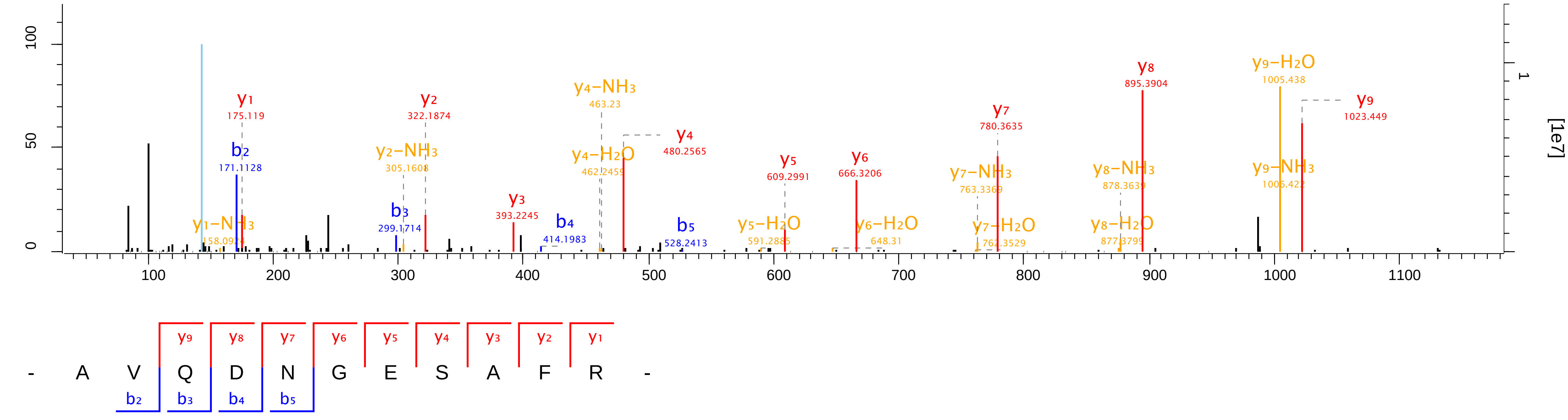

Raw file	Scan	Method	Score	m/z	Gene names
20140602_QEp4_FaHo_SA_SWI3_02	5345	FTMS; HCD	140.49	567.79	VPH1

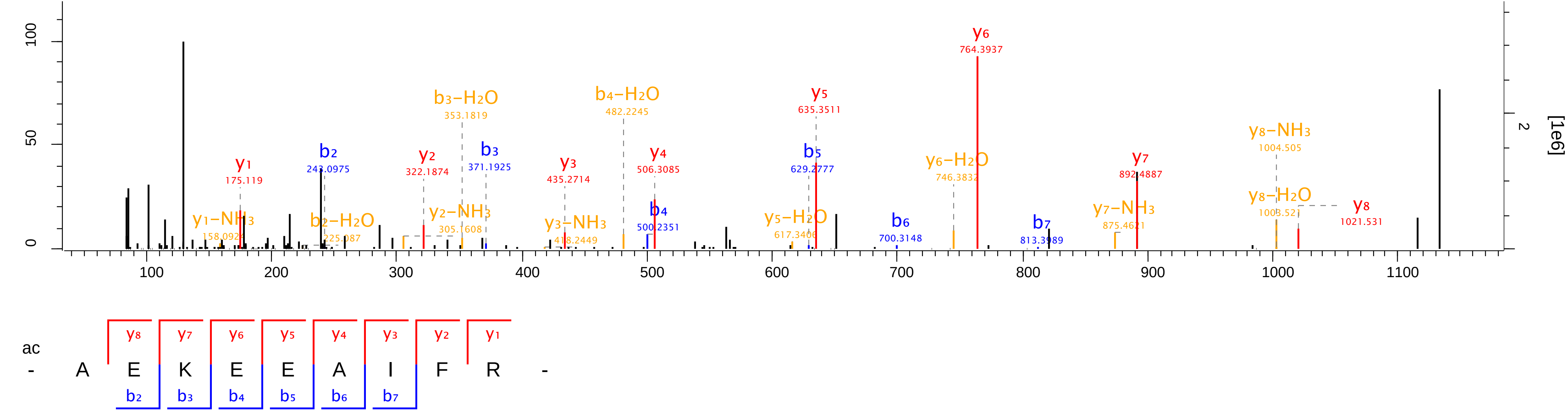

Raw file	Scan	Method	Score	m/z	Gene names
20140602_QEp4_FaHo_SA_SWI3_02	7474	FTMS; HCD	89.3	608.83	SUI2

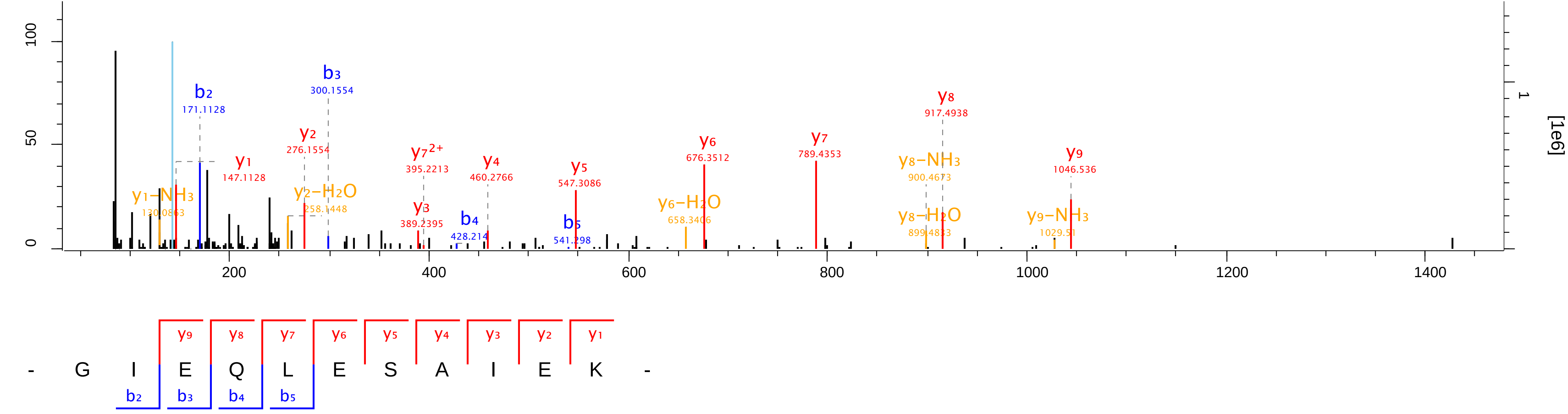

Raw file	Scan	Method	Score	m/z	Gene names
20140602_QEp4_FaHo_SA_SWI3_02	9857	FTMS; HCD	114.55	687.69	HNT1

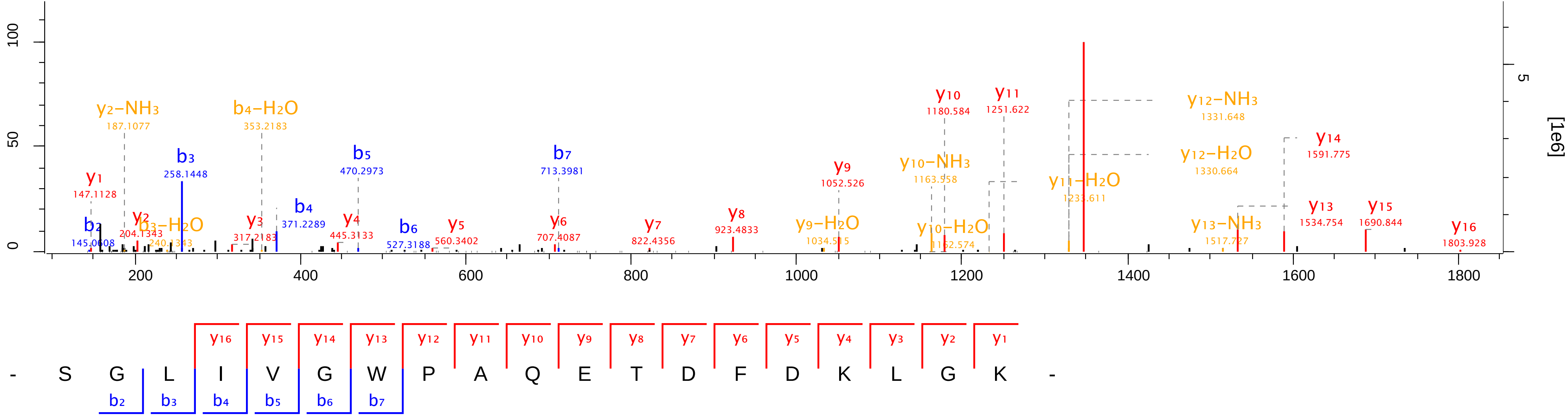

Raw file	Scan	Method	Score	m/z	Gene names
20140602_QEp4_FaHo_SA_SWI3_03	69	FTMS; HCD	62.46	367.72	RPL14B;RPL14A

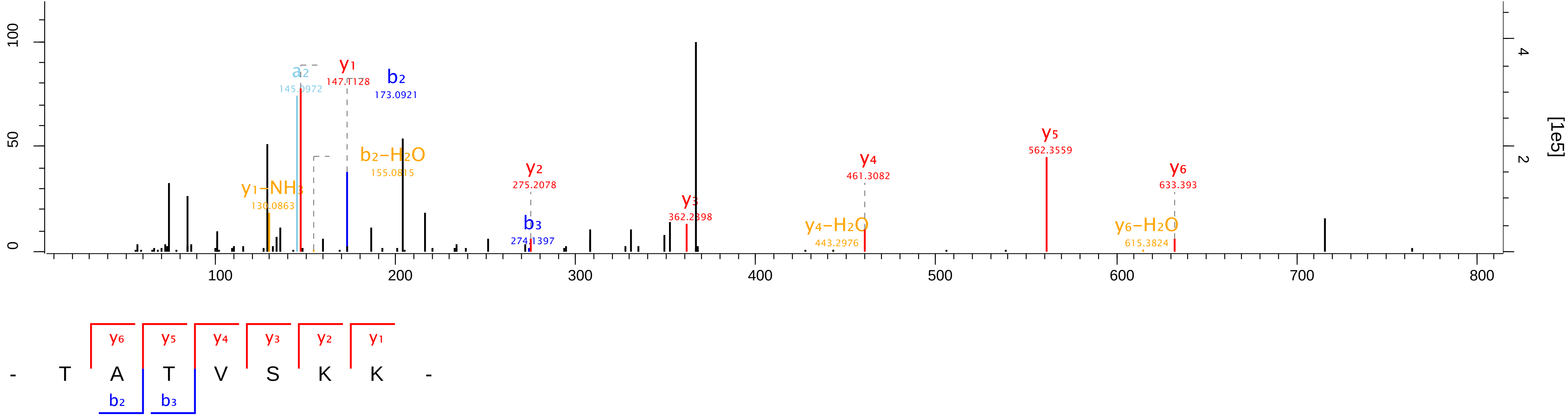

Raw file	Scan	Method	Score	m/z	Gene names
20140602_QEp4_FaHo_SA_SWI3_03	4232	FTMS; HCD	54.16	339.85	RPL31A;RPL31B

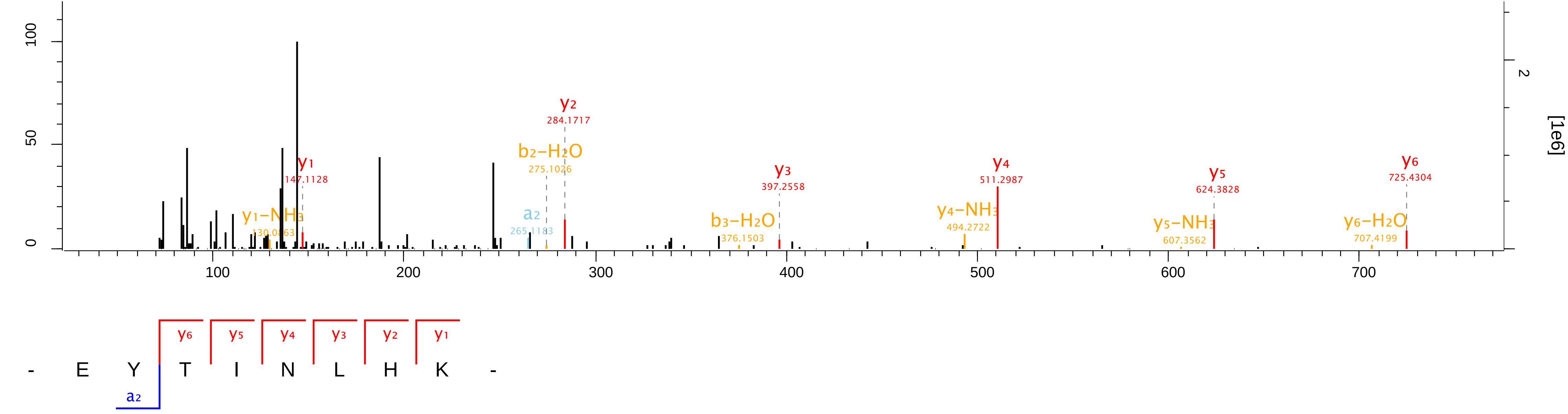

Raw file	Scan	Method	Score	m/z	Gene names
20140602_QEp4_FaHo_SA_SWI3_03	4697	FTMS; HCD	237.53	748.92	RPL17A;RPL17B

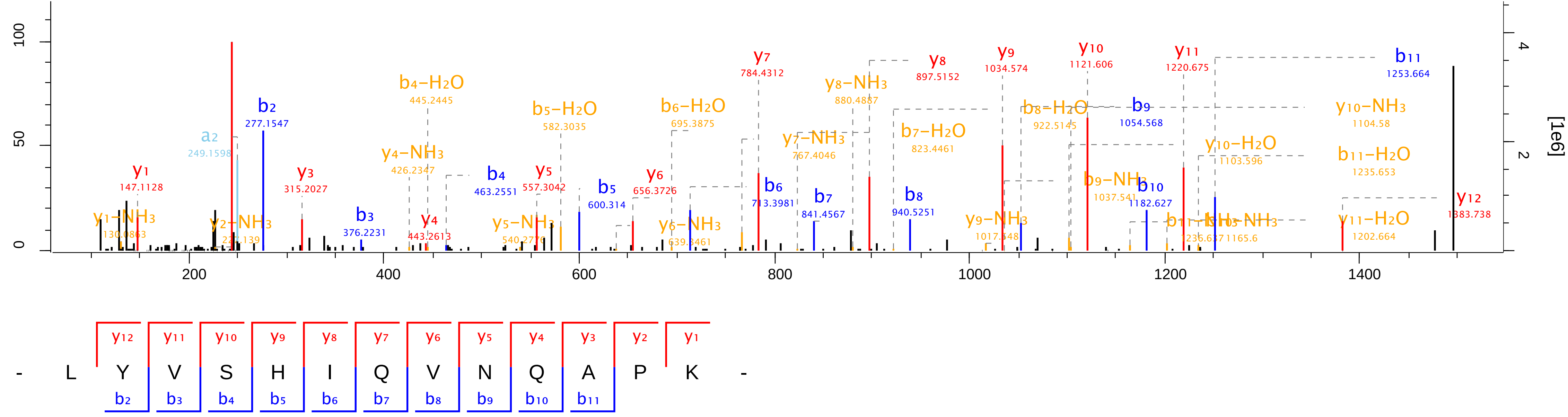

Raw file	Scan	Method	Score	m/z	Gene names
20140608_QEp4_FaHo_QC_NTO1_01	2025	FTMS; HCD	170.89	591.8	TY1B-ML1;TY1B-BR;TY1B-H;TY1B-MR2;TY1B-OR;TY1B-DR1;TY1B-NL2;TY1B-PR2;TY1B-DR5;TY1B-NL1;TY1B-DR3

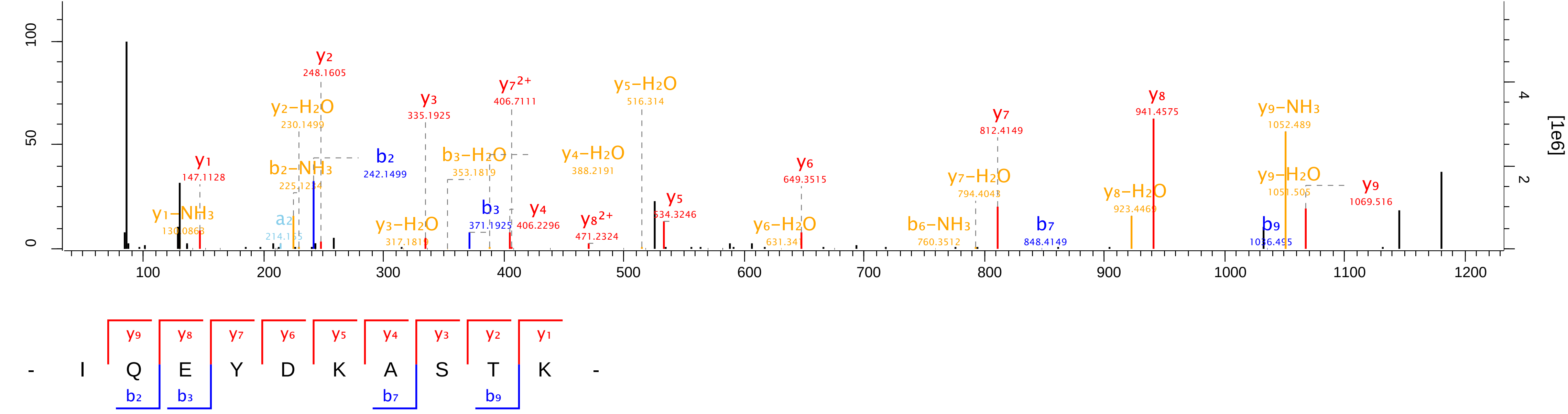

Raw file	Scar	Method	Score	m/z	Gene names
20140608_QEp4_FaHo_QC_	364	FTMS;	82.6	446.7	TY1B-ML1;TY1B-BR;TY1B-H;TY1B-MR2;TY1B-OR;TY1B-DR1;TY1B-NL2;TY1B-PR2;TY1B-DR5;TY1B-PR1;TY1B-JR2;TY1B-NL1;TY1B-OL;TY1B-LR4;TY1B-ML2;TY1B-DR3

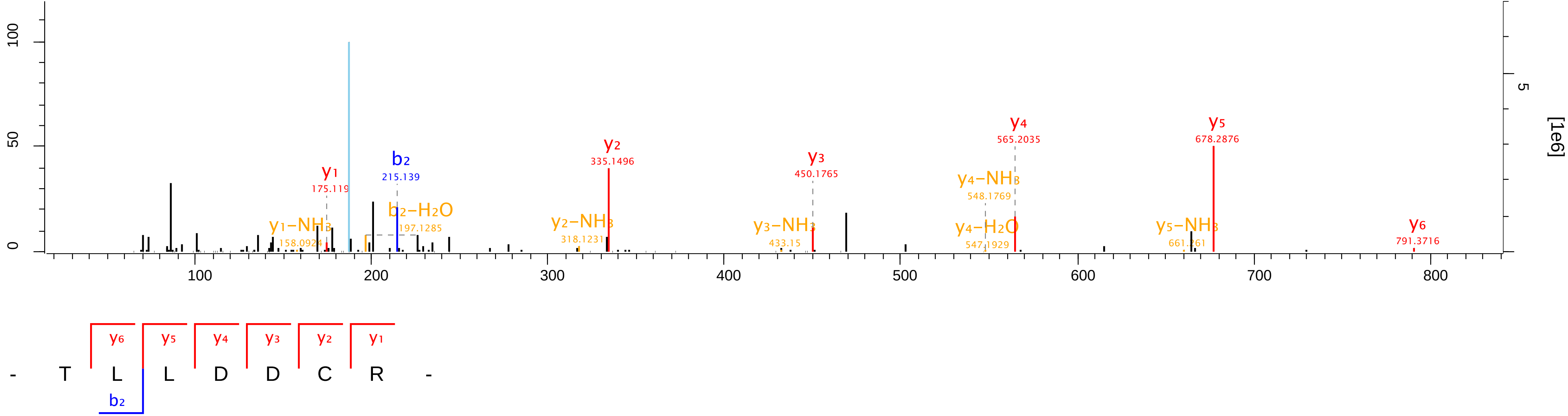

Raw file	Scan	Method	Score	m/z	Gene names
20140608_QEp4_FaHo_QC_NTO1_01	3707	FTMS; HCD	57.53	503.25	RPL14A

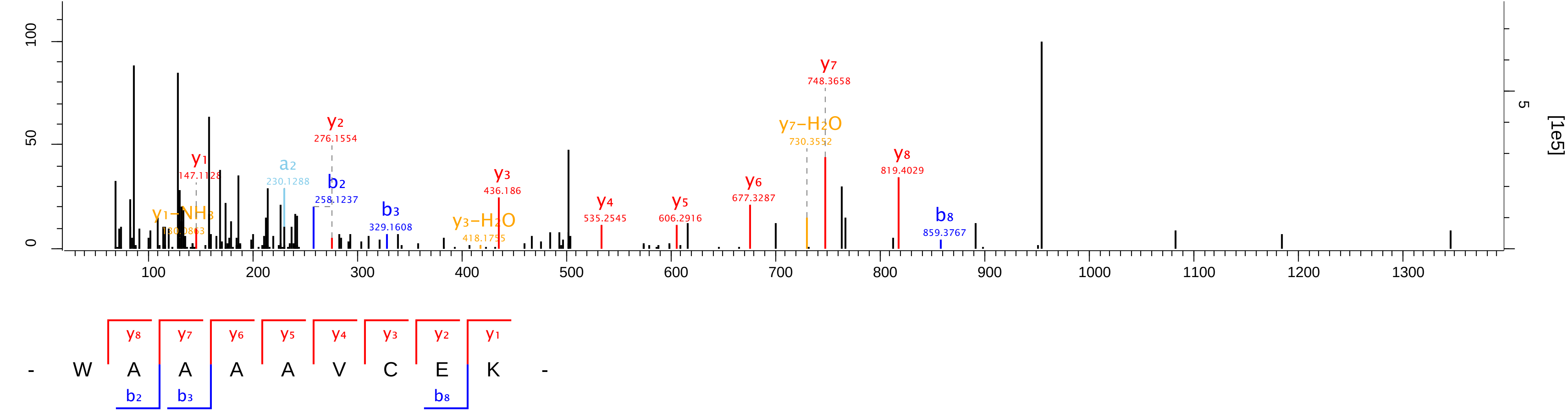

Raw file	Scan	Method	Score	m/z
20140608_QEp4_FaHo_QC_NTO1_01	7796	FTMS; HCD	63.21	582.32

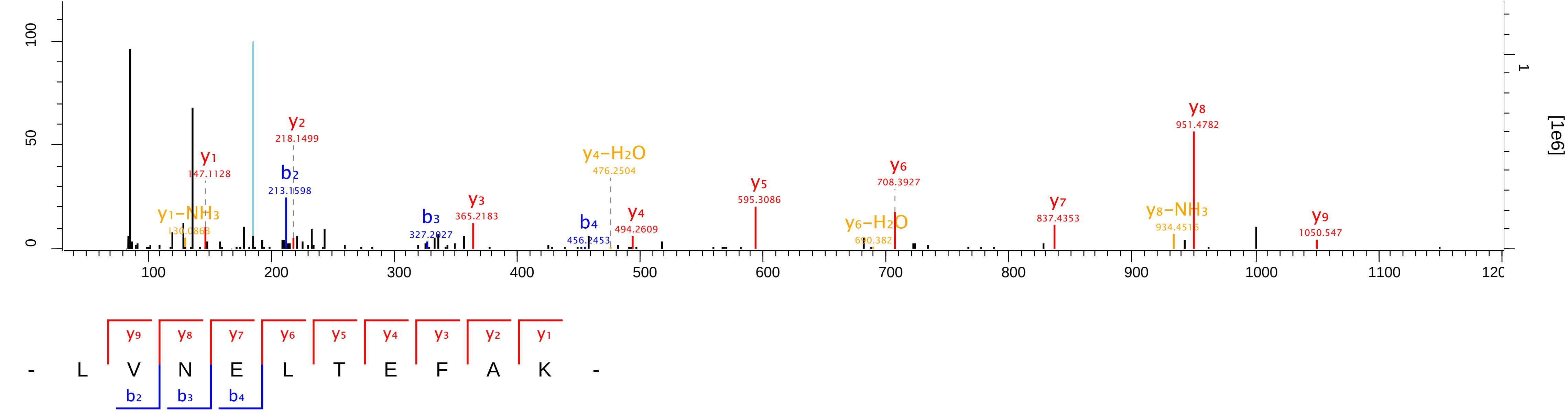

Raw file	Scan	Method	Score	m/z	Gene names
20140608_QEp4_FaHo_QC_SHG1_01	2315	FTMS; HCD	103.74	379.71	RPS26A

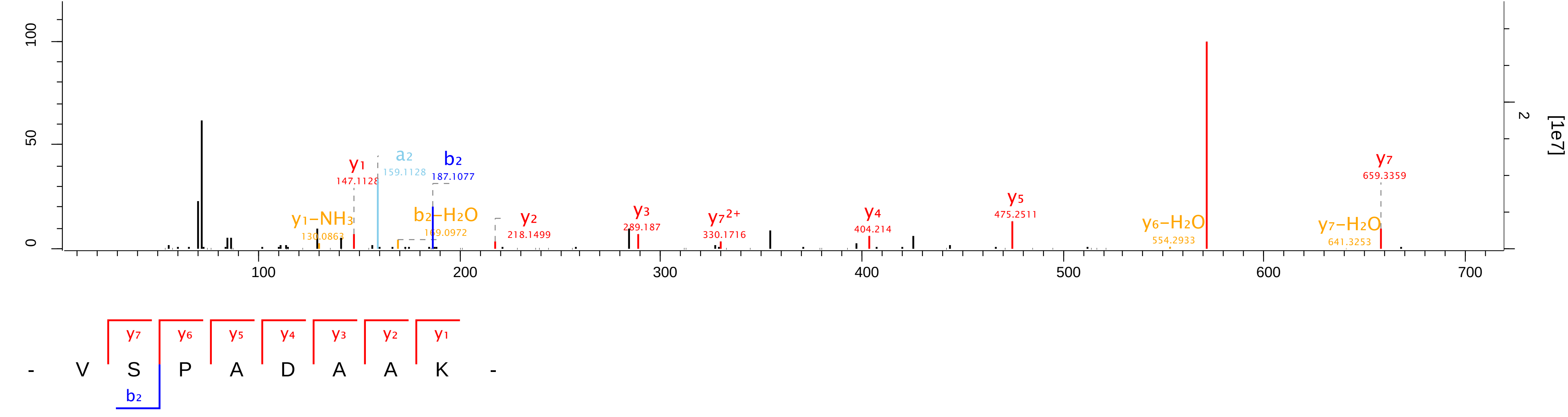

Raw file	Scan	Method	Score	m/z	Gene names
20140608_QEp4_FaHo_QC_SHG1_01	2990	FTMS; HCD	111.01	429.74	RPP1A

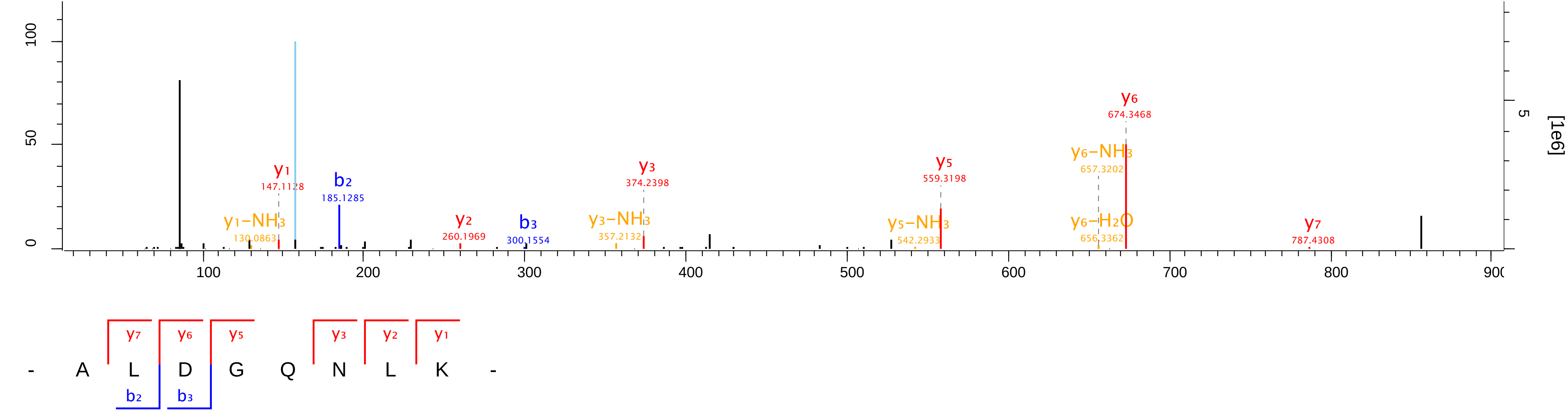

Raw file	Scan	Method	Score	m/z	Gene names
20140608_QEp4_FaHo_QC_SHG1_01	4504	FTMS; HCD	94.31	537.3	TY1B-NL1;TY1A-DR2;TY1B-BL;TY1B-MR1;TY1A-MR1

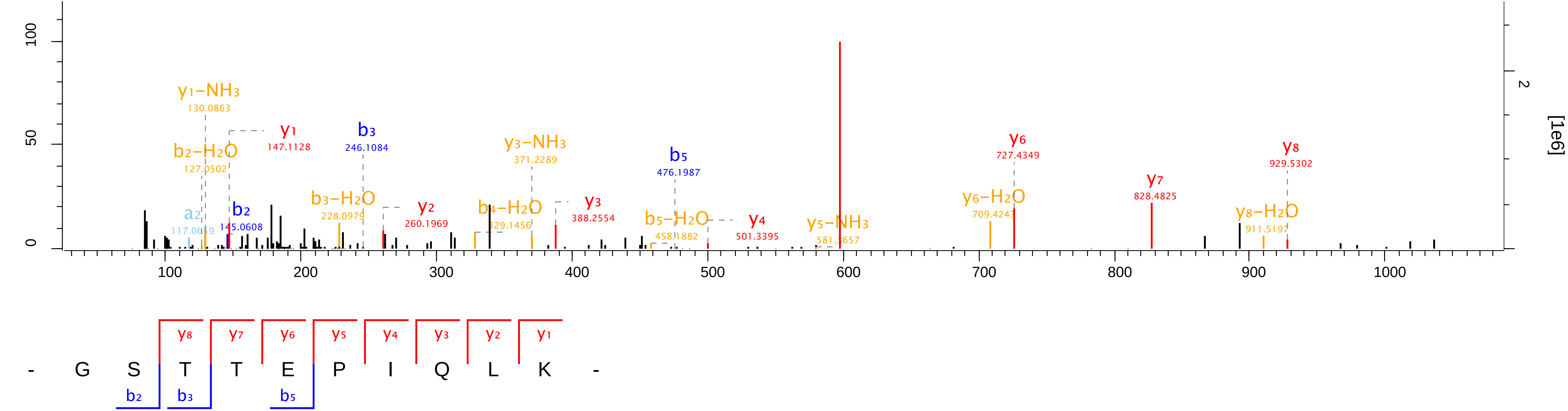

Raw file	Scan	Method	Score	m/z	Gene names
20140608_QEp4_FaHo_QC_SHG1_01	6585	FTMS; HCD	101.53	500.56	FRA1

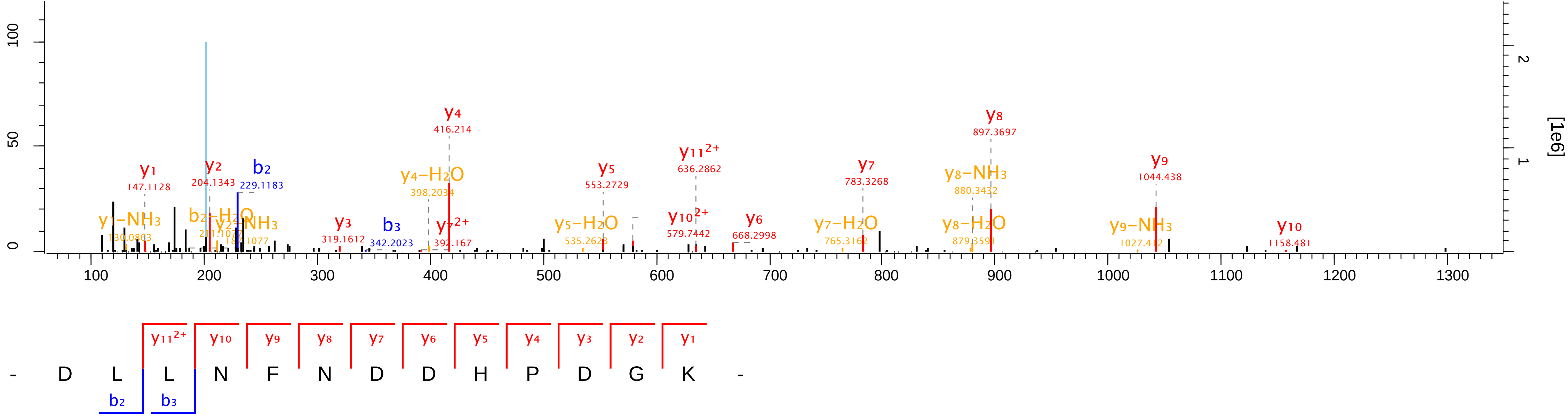

Raw file	Scan	Method	Score	m/z	Gene names
20140608_QEp4_FaHo_QC_SHG1_01	8553	FTMS; HCD	126.83	639.34	RPL9A;RPL9B

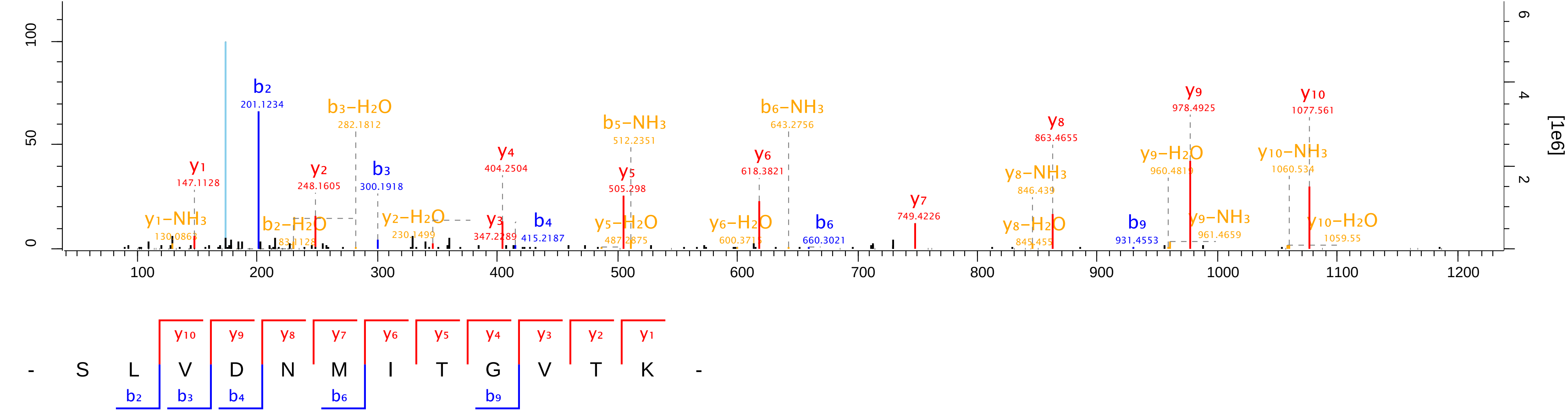

Raw file	Scan	Method	Score	m/z	Gene names
20140608_QEp4_FaHo_QC_YNG1_01	7151	FTMS; HCD	89.91	727.41	GRX5

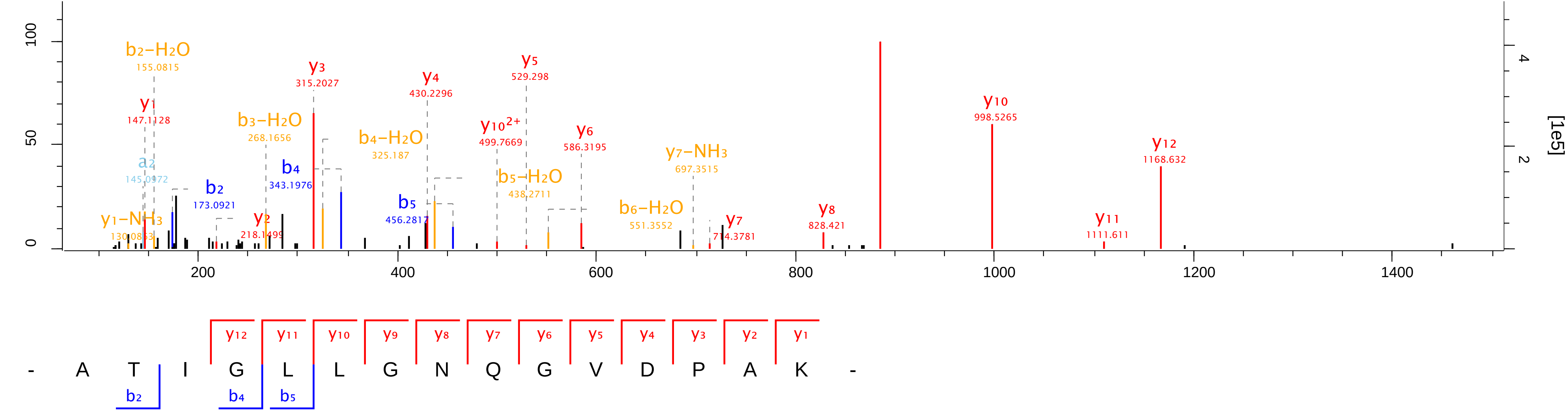

Raw file	Scan	Method	Score	m/z	Gene names
20140608_QEp4_FaHo_QC_YNG1_02	2434	FTMS; HCD	187.5	487.75	RPL7A

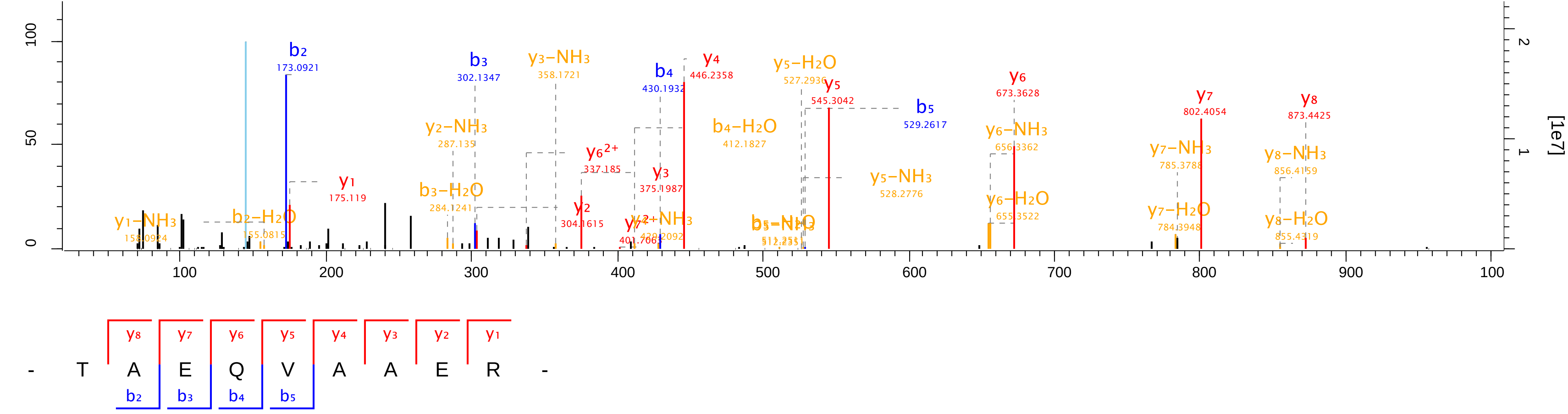

Raw file	Scan	Method	Score	m/z	Gene names
20140608_QEp4_FaHo_QC_YNG1_02	9078	FTMS; HCD	81.64	919.44	RPS14B;RPS14A

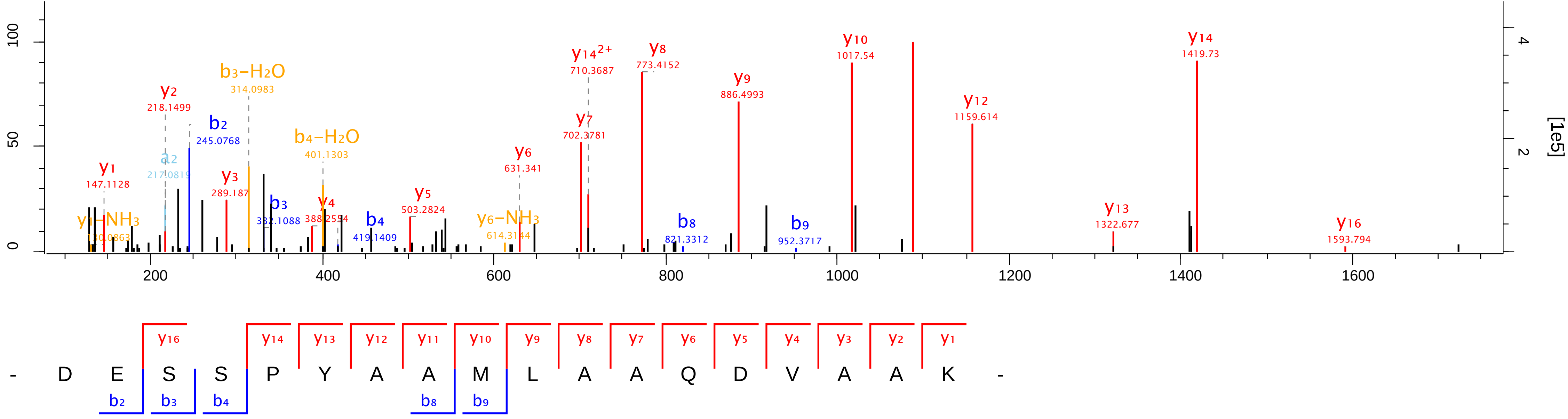

Raw file	Scan	Method	Score	m/z	Gene names
20140608_QEp4_FaHo_QC_YNG1_03	2245	FTMS; HCD	104.2	474.25	RPL36A

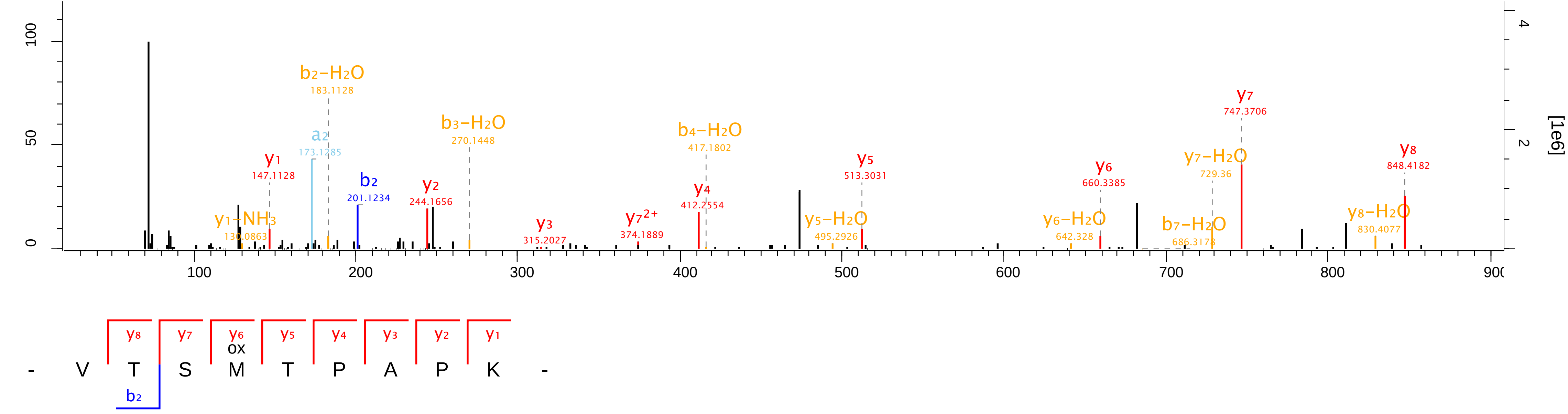

Raw file	Scan	Method	Score	m/z	Gene names
20140608_QEp4_FaHo_QC_YNG1_03	2996	FTMS; HCD	177.44	662.32	RPL7A;RPL7B

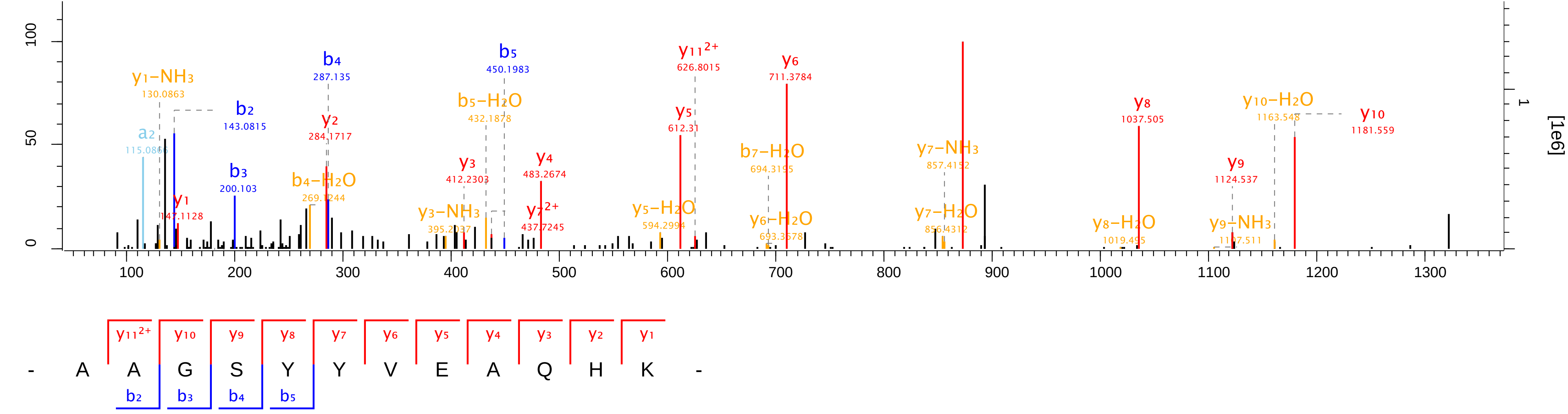

Raw file	Scan	Method	Score	m/z	Gene names
20140608_QEp4_FaHo_QC_YNG1_03	4699	FTMS; HCD	103.55	490.26	ACB1

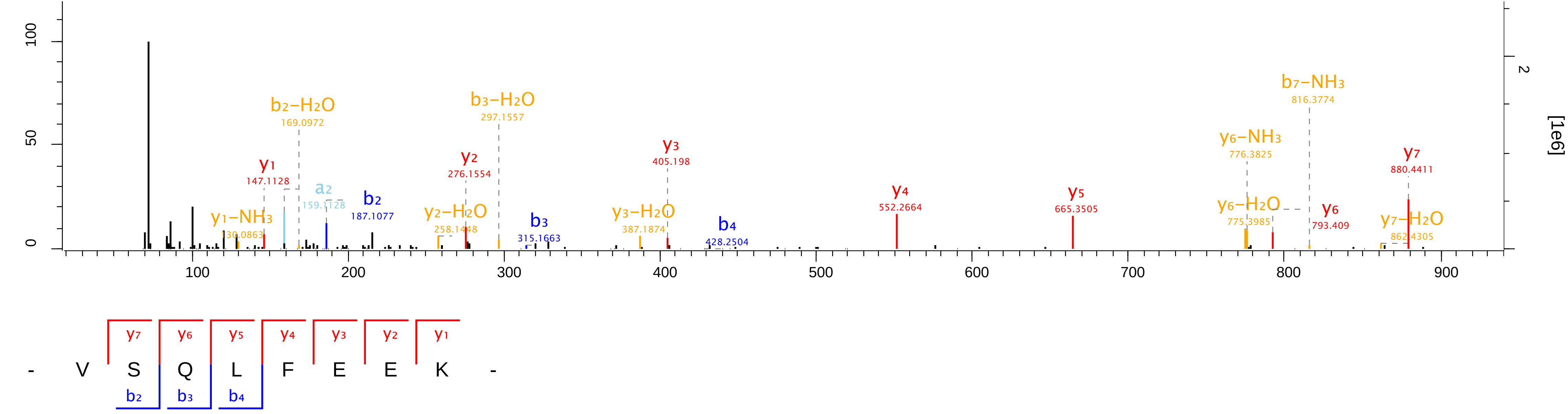

Raw file	Scan	Method	Score	m/z	Gene names
20140608_QEp4_FaHo_QC_YNG1_03	5472	FTMS; HCD	153.75	572.97	RPL4A

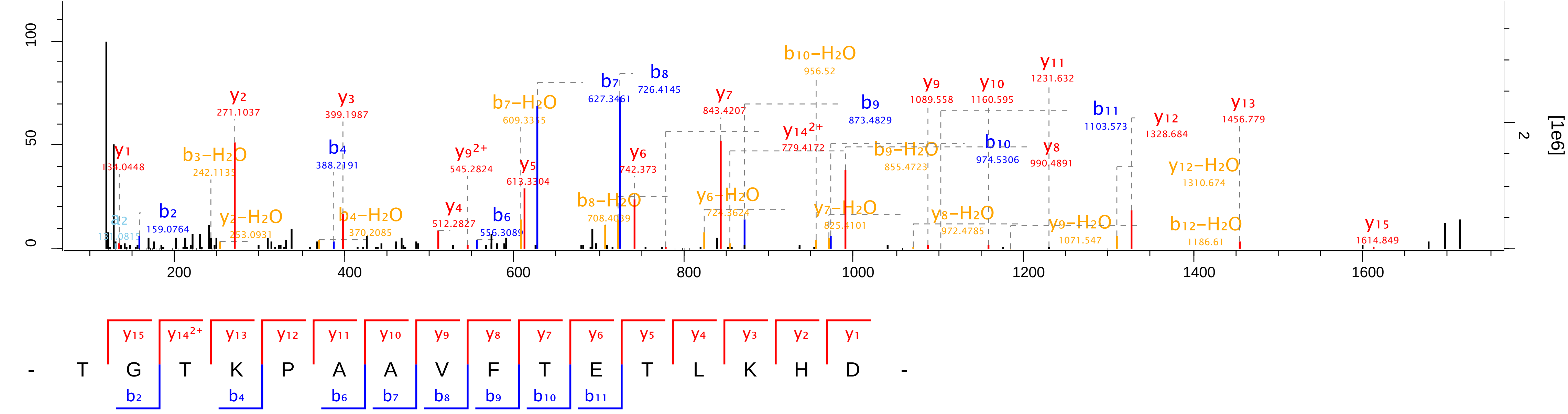

Raw file	Scan	Method	Score	m/z	Gene names
20140608_QEp4_FaHo_QC_YNG2_01	3406	FTMS; HCD	131.12	423.9	RPS0B;RPS0A

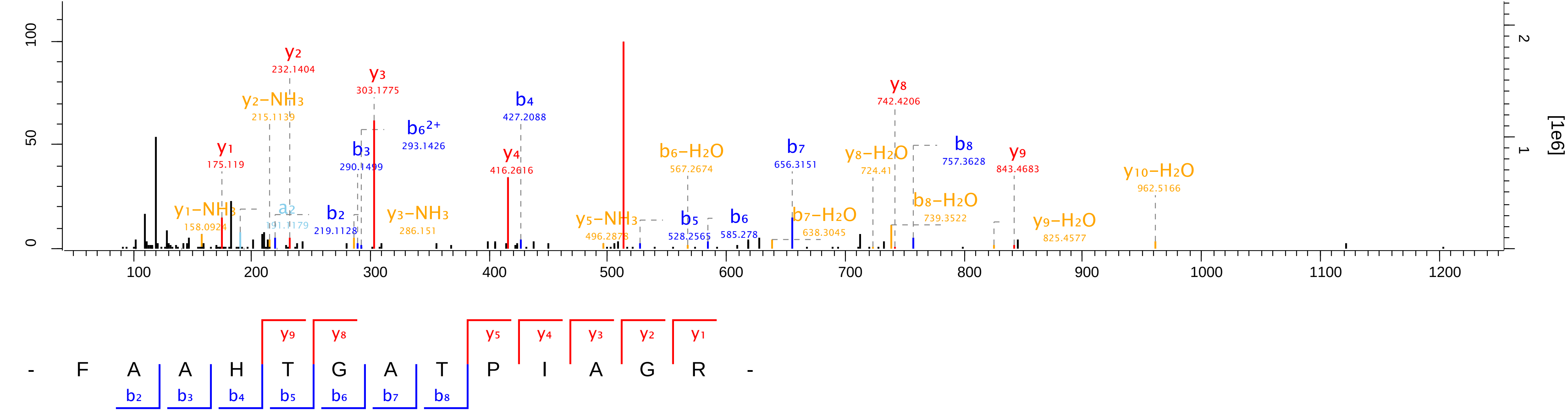

Raw file	Scan	Method	Score	m/z	Gene names
20140608_QEp4_FaHo_QC_YNG2_01	4241	FTMS; HCD	118.19	573.63	MBF1

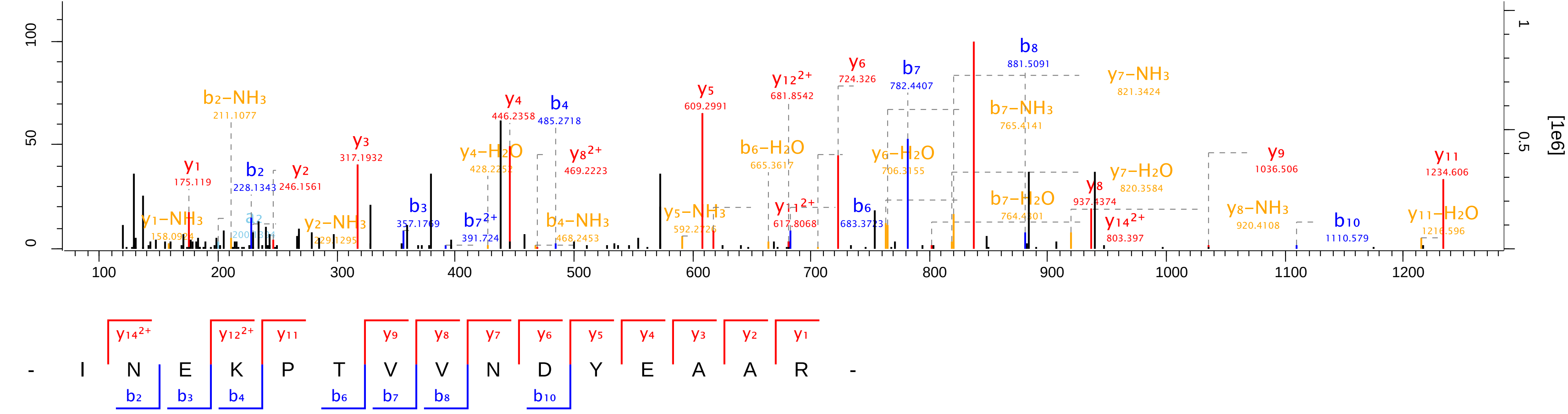

Raw file	Scan	Method	Score	m/z	Gene names
20140608_QEp4_FaHo_QC_YNG2_02	2818	FTMS; HCD	60.08	389.21	RPL17A;RPL17B

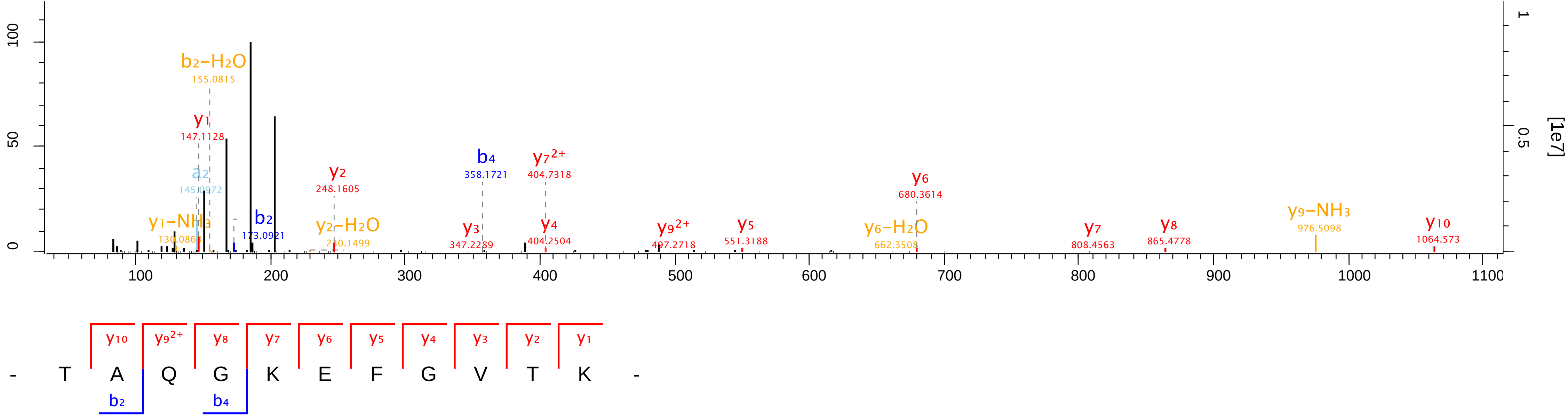

Raw file	Scan	Method	Score	m/z	Gene names
20140608_QEp4_FaHo_QC_YNG2_02	2950	FTMS; HCD	187.63	787.37	RPL7A;RPL7B

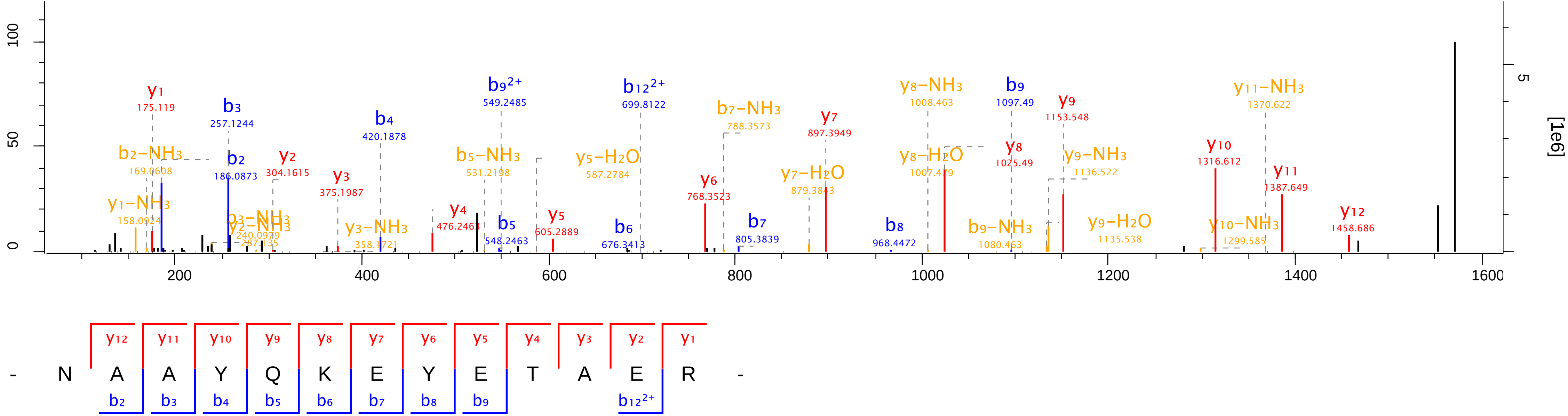

Raw file	Scan	Method	Score	m/z	Gene names
20140608_QEp4_FaHo_QC_YNG2_02	2956	FTMS; HCD	86.34	376.88	RPL13B;RPL13A

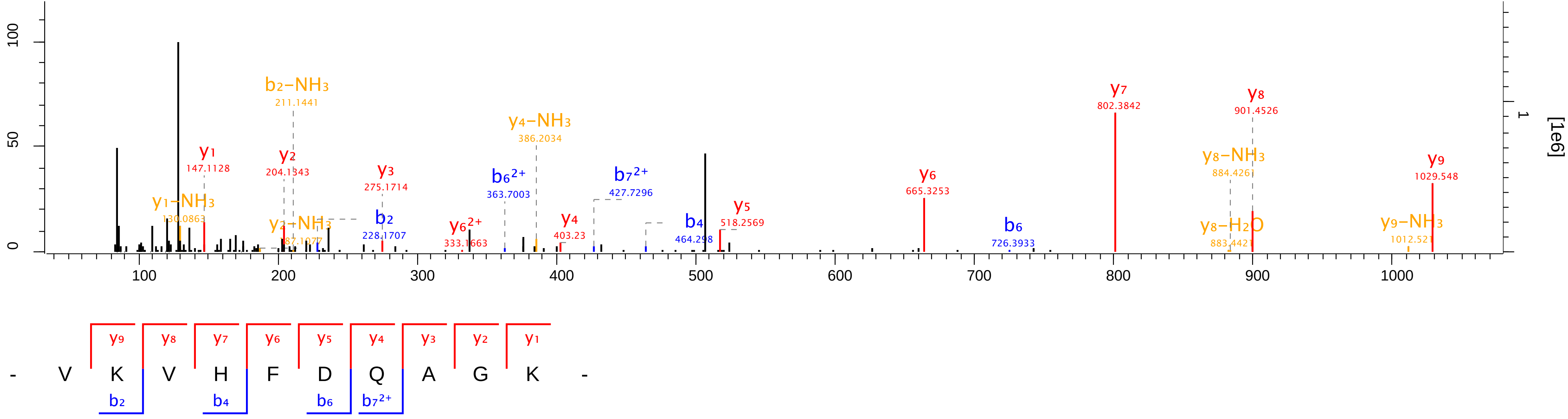

Raw file	Scar	Method	Score	m/z	Gene names
20140608_QEp4_FaHo_Q	384	FTMS;2	143.(	415.'	TY1B-ML1;TY1B-BR;TY1A-PR1;TY1A-A;TY1A-DR4;TY1B-H;TY1B-MR2;TY1B-OR;TY1B-DR1;TY1B-NL2;TY1B-PR2;TY1B-DR5;TY1B-PR1;TY1B-JR2;TY1A-PL;TY1A-LR2;TY1A

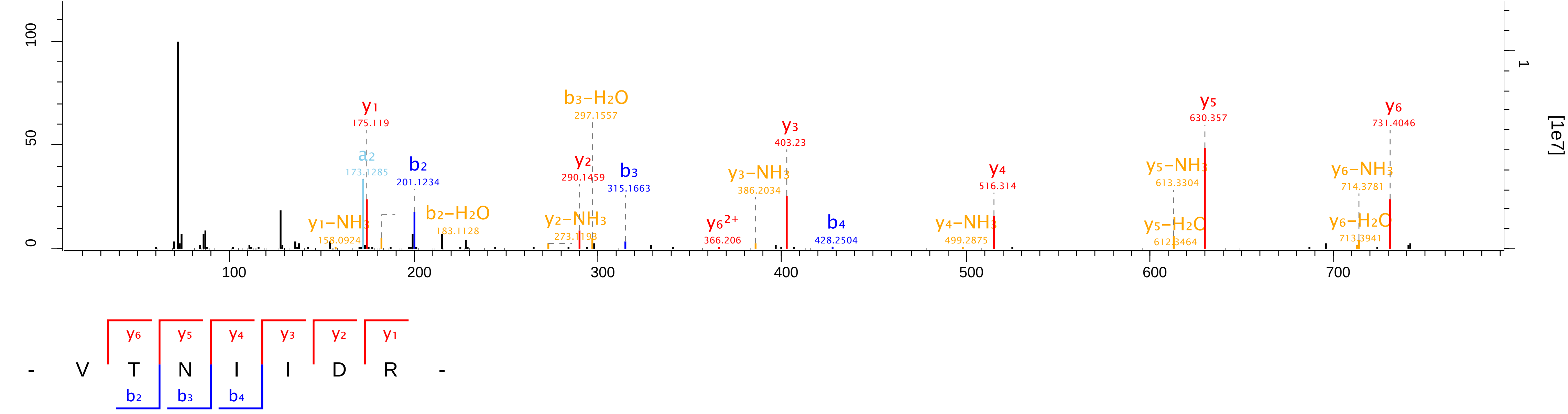

Raw file	Scan	Method	Score	m/z	Gene names
20140608_QEp4_FaHo_QC_YNG2_02	4972	FTMS; HCD	164.66	644.33	RPL17B

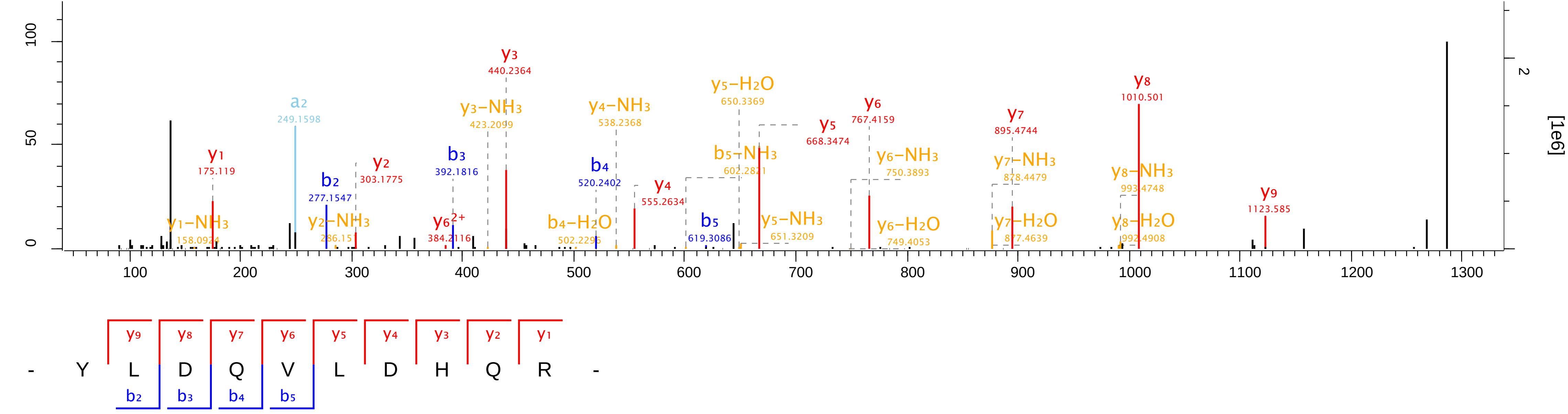

Raw file	Scan	Method	Score	m/z	Gene names
20140608_QEp4_FaHo_QC_YNG2_02	4980	FTMS; HCD	120.4	718.01	TAT1

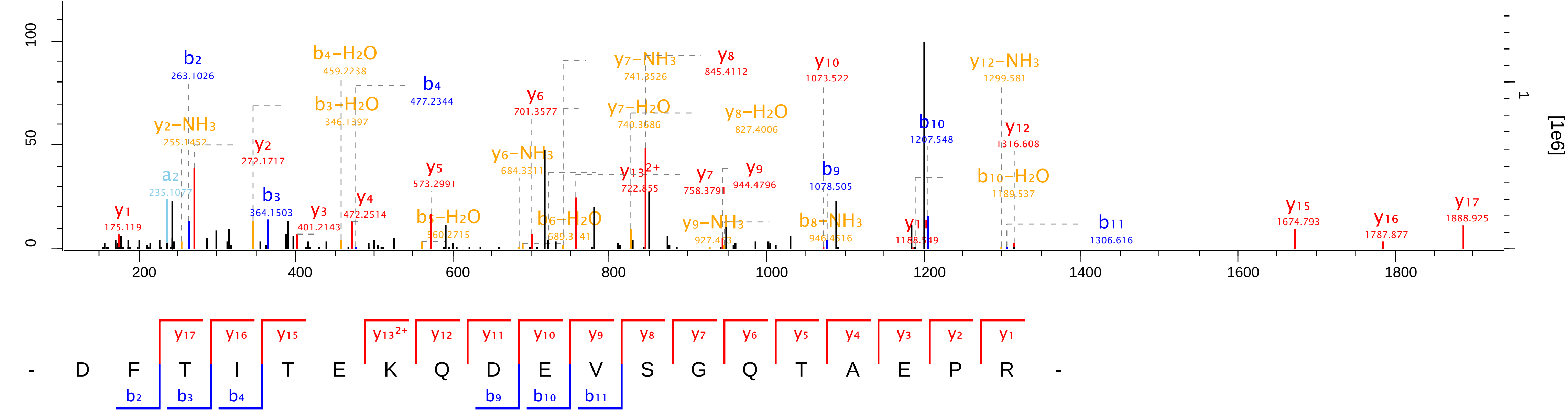

Raw file	Scan	Method	Score	m/z	Gene names
20140608_QEp4_FaHo_QC_YNG2_02	5216	FTMS; HCD	221.55	667.33	RPP2A

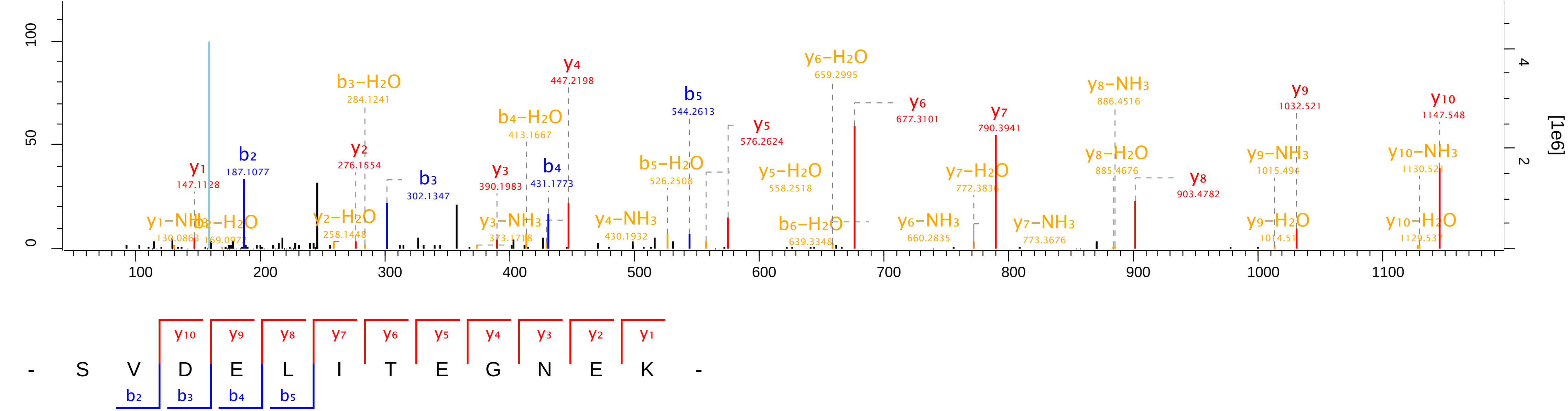

Raw file	Scan	Method	Score	m/z	Gene names
20140608_QEp4_FaHo_QC_YNG2_02	5224	FTMS; HCD	183.36	871.46	RPS14B;RPS14A

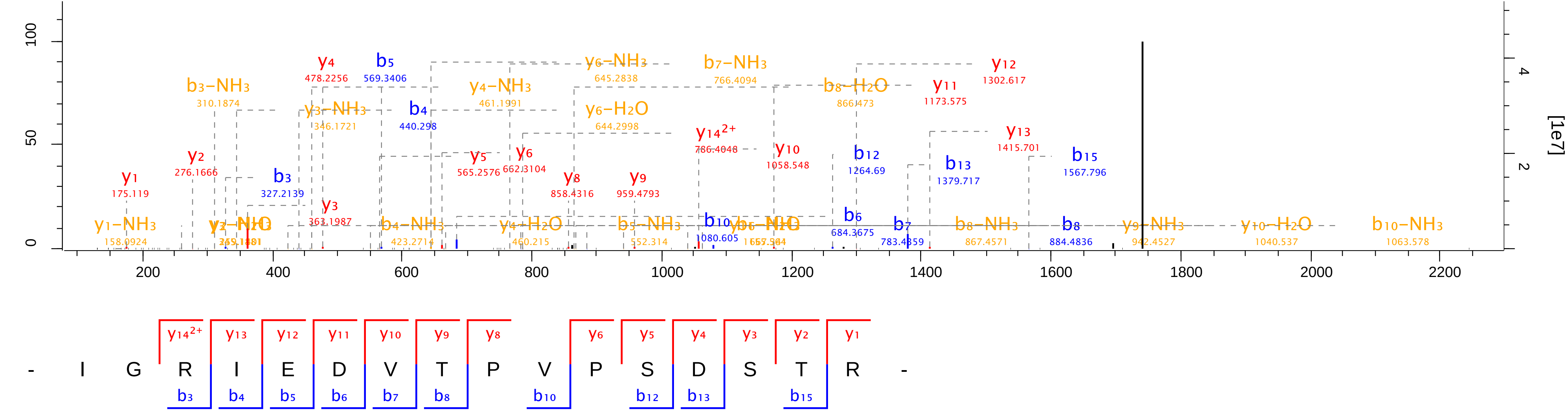

Raw file	Scan	Method	Score	m/z	Gene names
20140608_QEp4_FaHo_QC_YNG2_02	5254	FTMS; HCD	72.97	453.73	RPL14B;RPL14A

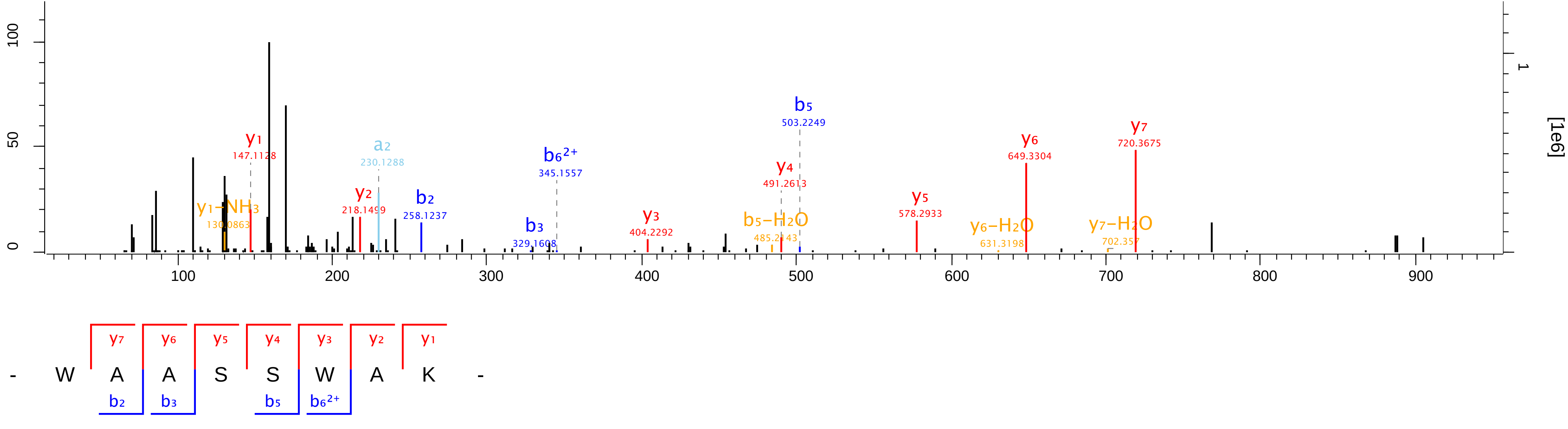

Raw file	Sca	Metho	Scor	m/z	Gene names
20140608_QEp4_FaH[illegible]	52[illegible]	FTMS[illegible]	12[illegible]	447[illegible]	TY1B-ML1;TY1B-H;TY1B-MR2;TY1B-DR1;TY1B-PR2;TY1B-DR5;TY1B-PR1;TY1B-JR2;TY1B-NL1;TY2B-C;TY1B-OL;TY1B-LR4;TY1B-ML2;TY1B-DR3;TY1B-PR3;TY1B-PL;TY1B-LR2;[illegible]

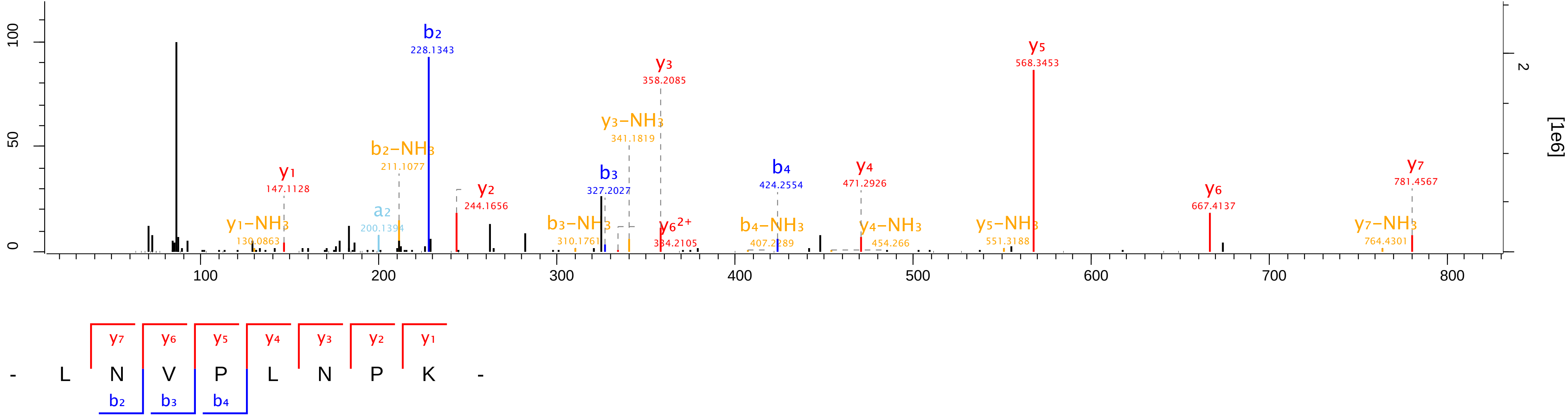

Raw file	Scan	Method	Score	m/z	Gene names
20140608_QEp4_FaHo_QC_YNG2_02	5825	FTMS; HCD	134.3	596.82	FEN1

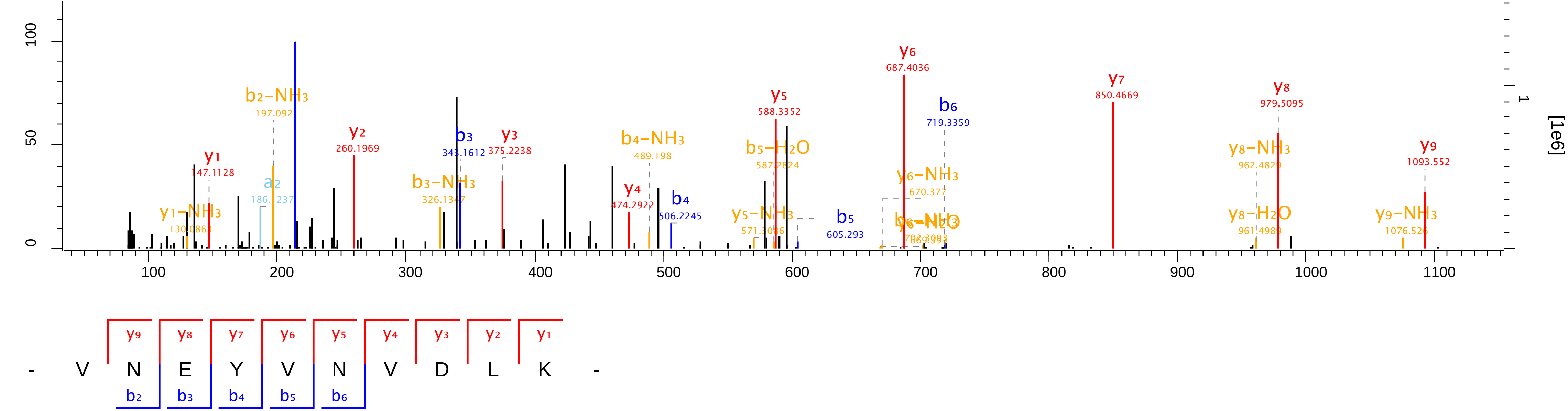

Raw file	Scan	Method	Score	m/z	Gene names
20140608_QEp4_FaHo_QC_YNG2_02	5888	FTMS; HCD	246.77	888.07	TY1B-BR;TY1B-H;TY1B-MR2;TY1B-OR;TY1B-DR1;TY1B-PR2;TY1B-DR5;TY1B-DR3

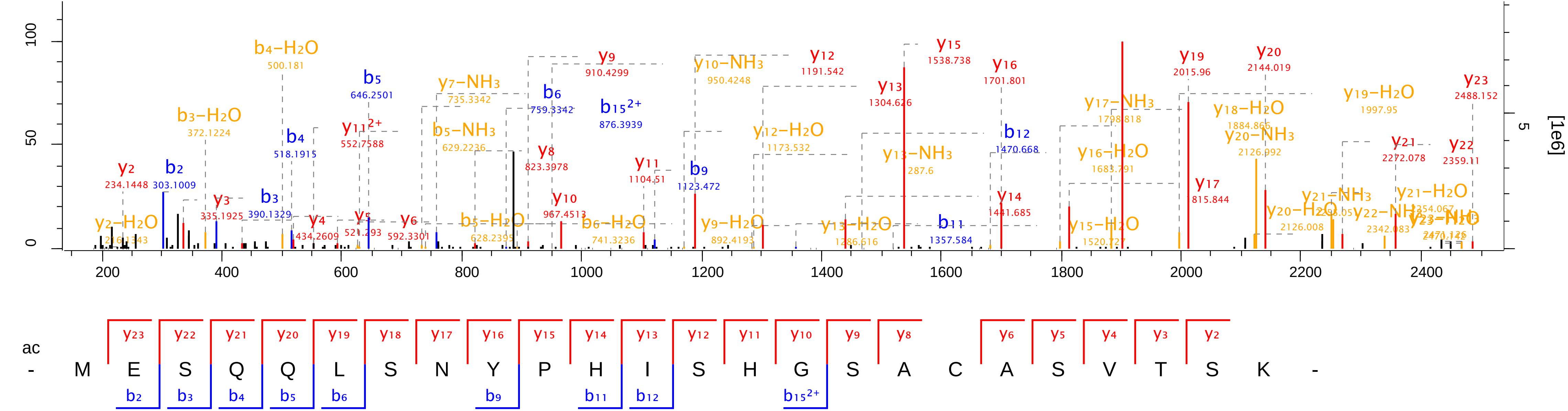

Raw file	Scan	Method	Score	m/z	Gene names
20140608_QEp4_FaHo_QC_YNG2_02	6441	FTMS; HCD	121.18	808.85	CAF20

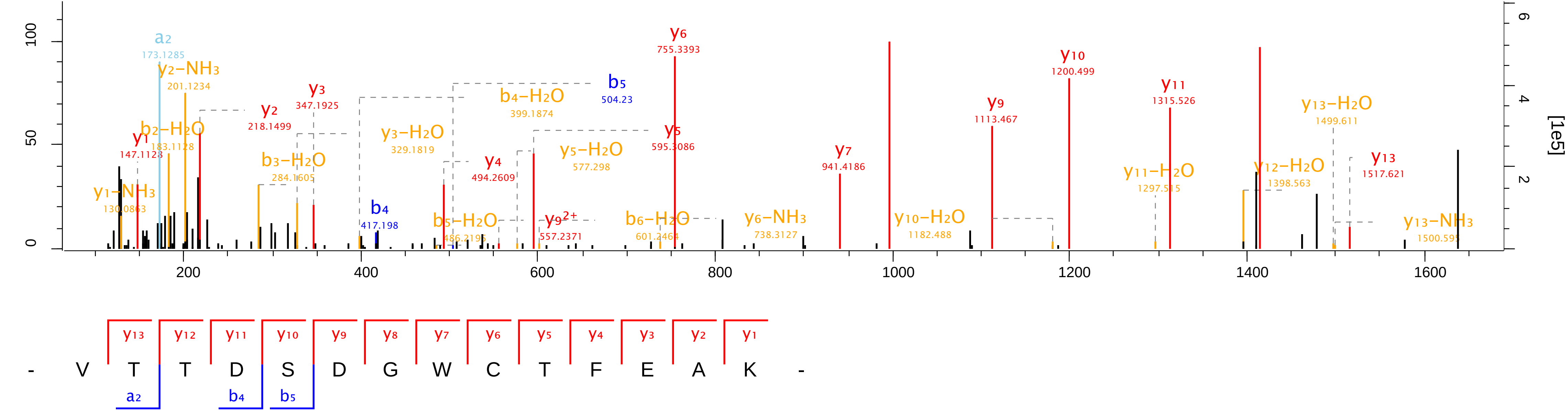

Raw file	Scan	Method	Score	m/z	Gene names
20140608_QEp4_FaHo_QC_YNG2_02	6974	FTMS; HCD	107.74	494.96	RPL24A;RPL24B

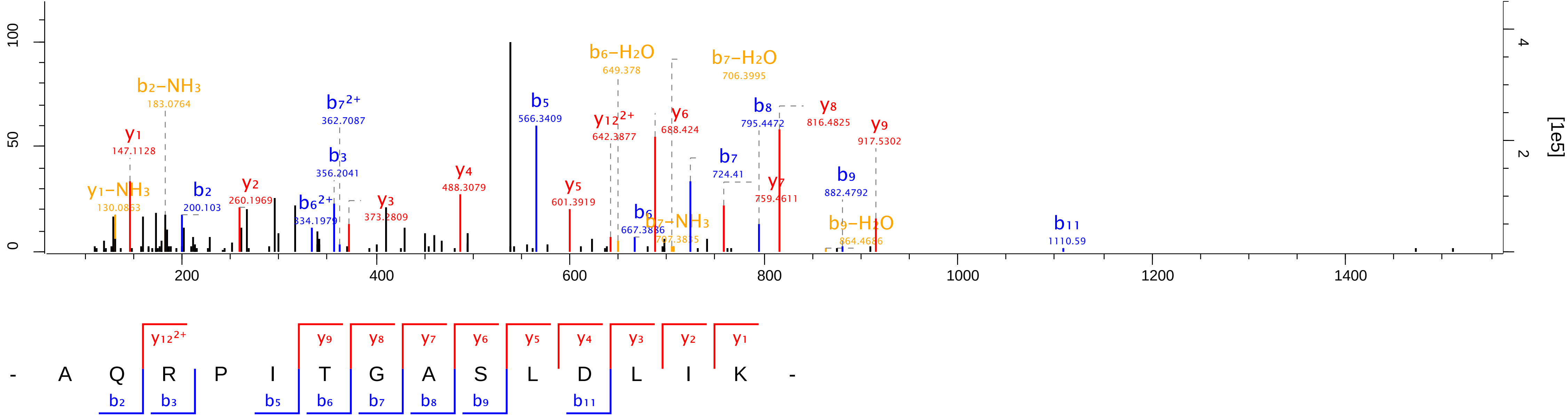

Raw file	Scan	Method	Score	m/z	Gene names
20140608_QEp4_FaHo_QC_YNG2_02	7183	FTMS; HCD	120.06	480.28	RPL31A;RPL31B

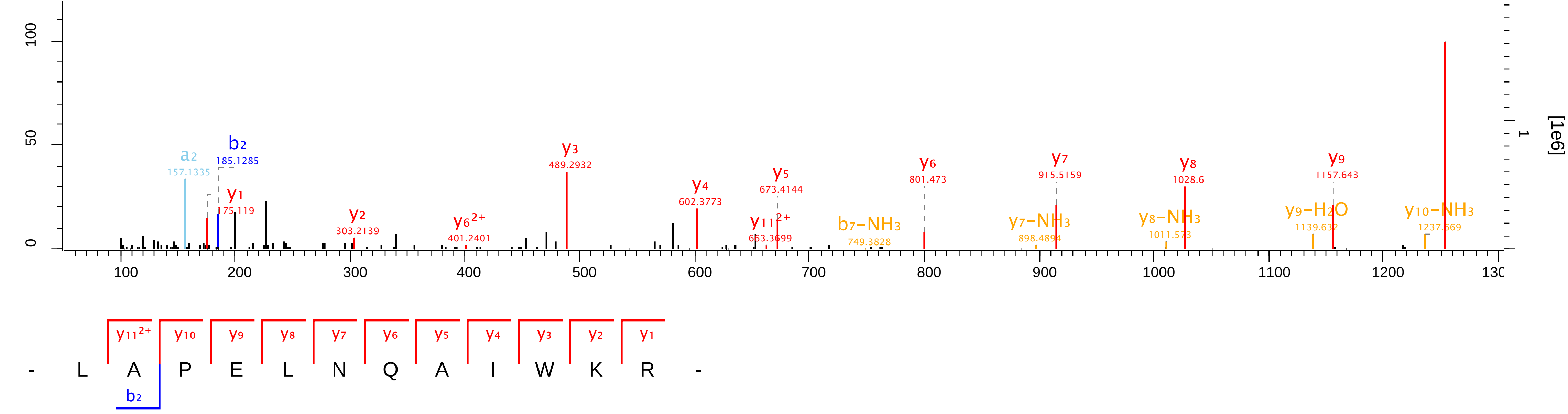

Raw file	Scan	Method	Score	m/z	Gene names
20140608_QEp4_FaHo_QC_YNG2_02	7831	FTMS; HCD	116.51	681.86	RBL2

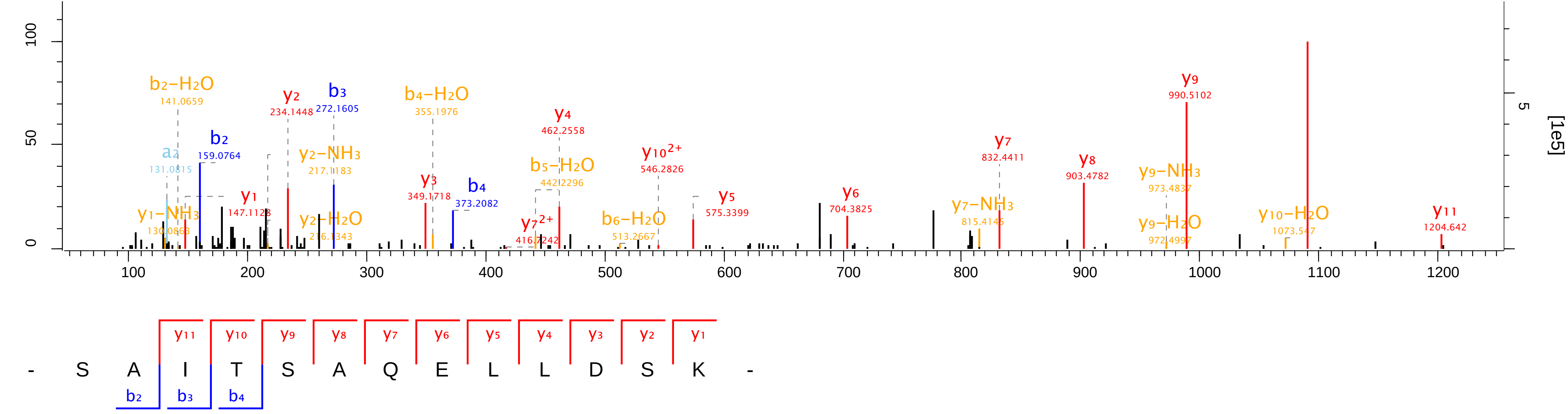

Raw file	Scan	Method	Score	m/z	Gene names
20140608_QEp4_FaHo_QC_YNG2_03	2678	FTMS; HCD	128.44	487.78	RPL24A;RPL24B

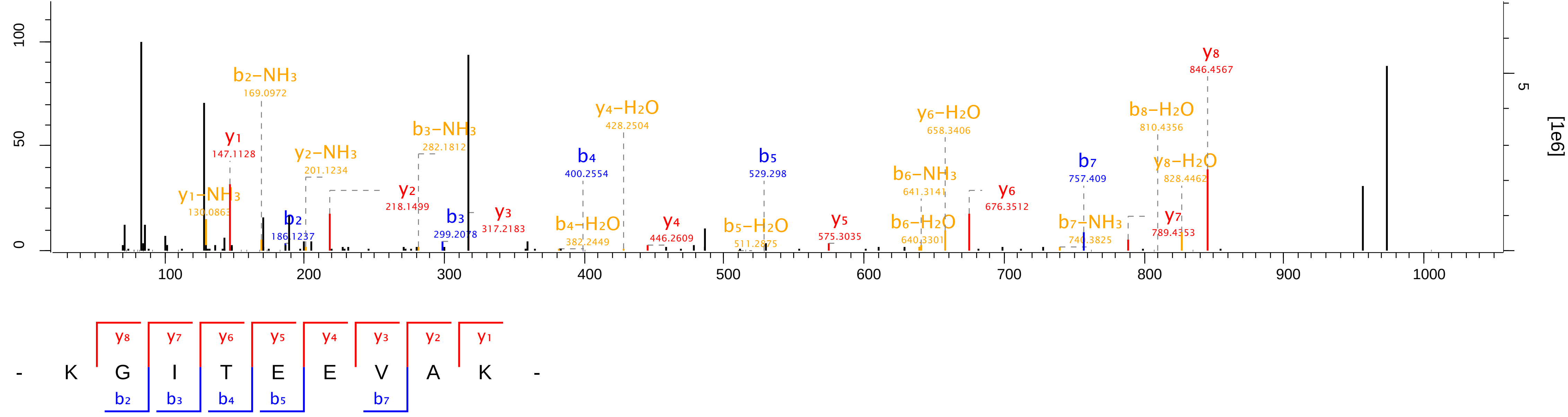

Raw file	Scan	Method	Score	m/z	Gene names
20140608_QEp4_FaHo_QC_YNG2_03	2916	FTMS; HCD	125.75	494.76	RPL7B

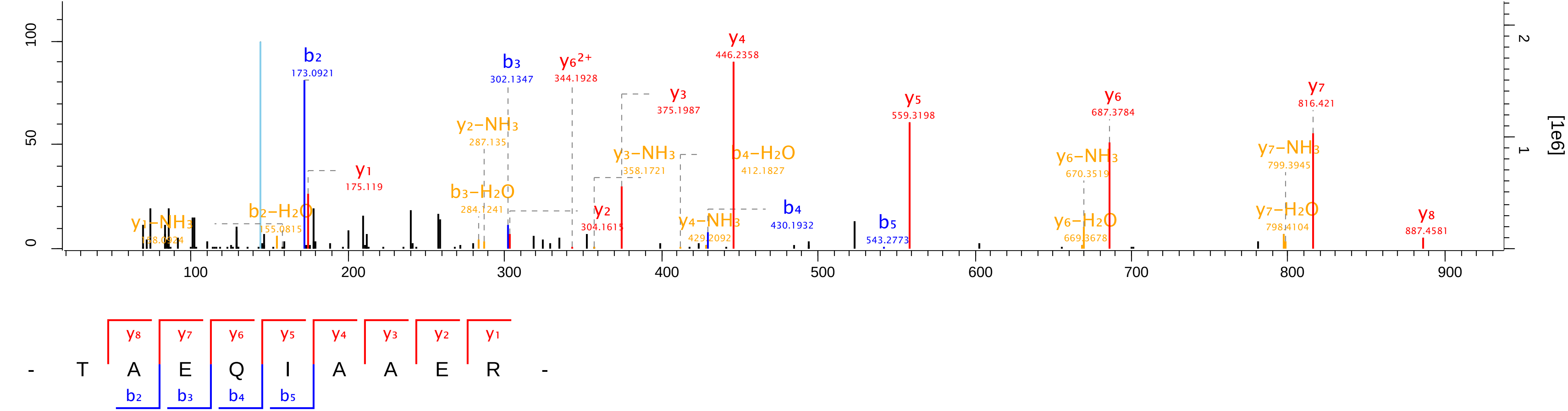

Raw file	Scan	Method	Score	m/z	Gene names
20140608_QEp4_FaHo_QC_YNG2_03	2947	FTMS; HCD	176.56	722.83	RPL4B;RPL4A

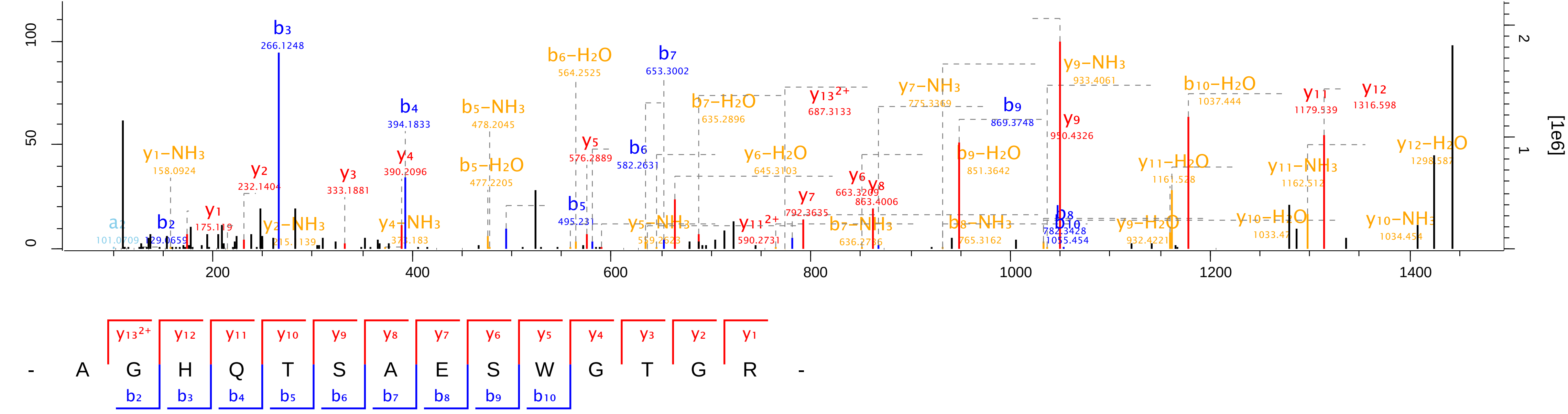

Raw file	Scan	Method	Score	m/z	Gene names
20140608_QEp4_FaHo_QC_YNG2_03	3561	FTMS; HCD	138.24	394.23	RPL31A;RPL31B

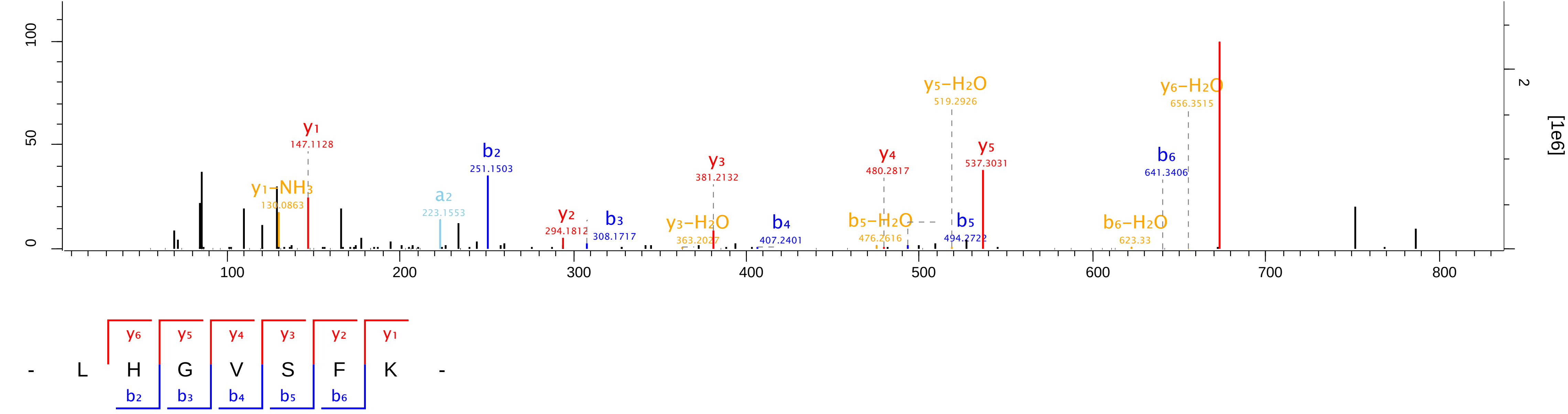

Raw file	Scan	Method	Score	m/z	Gene names
20140608_QEp4_FaHo_QC_YNG2_03	6750	FTMS; HCD	87.58	429.22	SHM2;SHM1

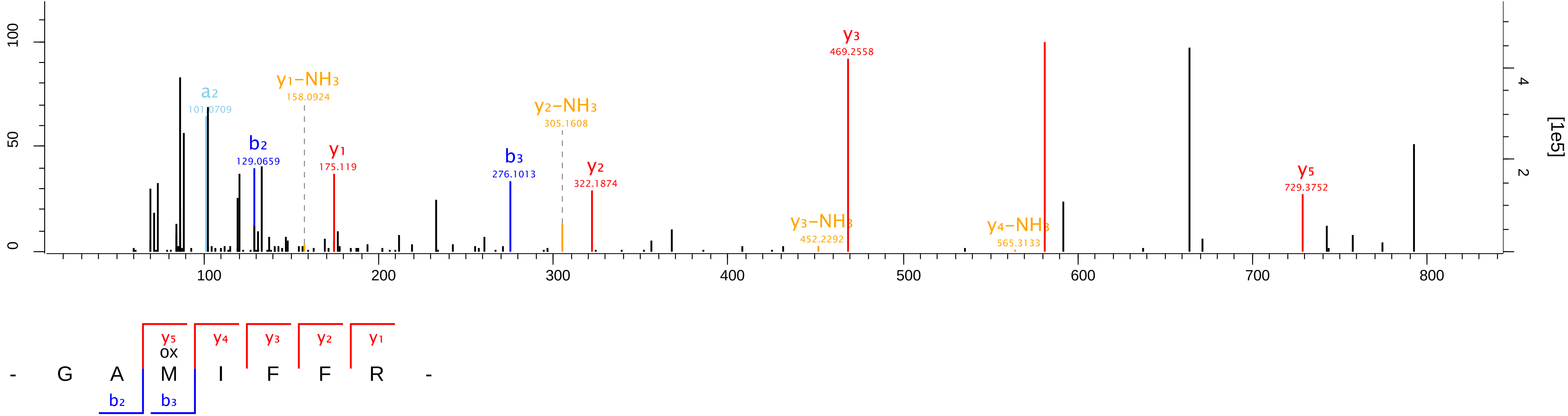

Raw file	Scan	Method	Score	m/z	Gene names
20140608_QEp4_FaHo_QC_YNG2_03	7134	FTMS; HCD	0.88	705.12	ECM38

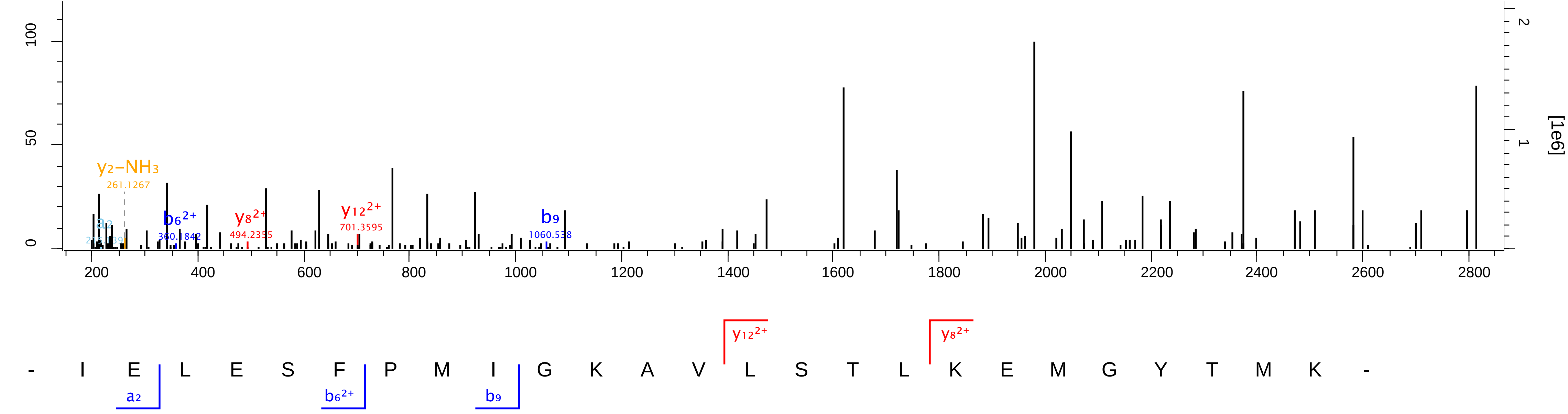

Raw file	Scan	Method	Score	m/z	Gene names
20140608_QEp4_FaHo_QC_YNG2_03	7970	FTMS; HCD	101.66	732.62	MRPS12

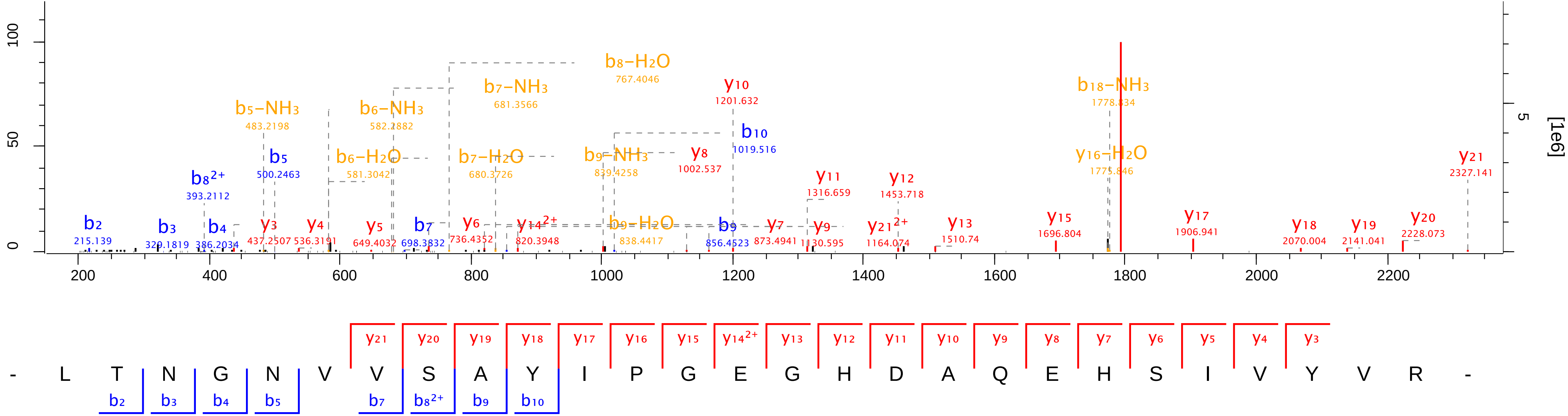

Raw file	Scan	Method	Score	m/z	Gene names
20140608_QEp4_FaHo_QC_YNG2_03	8418	FTMS; HCD	153.95	670.91	RPL14B;RPL14A

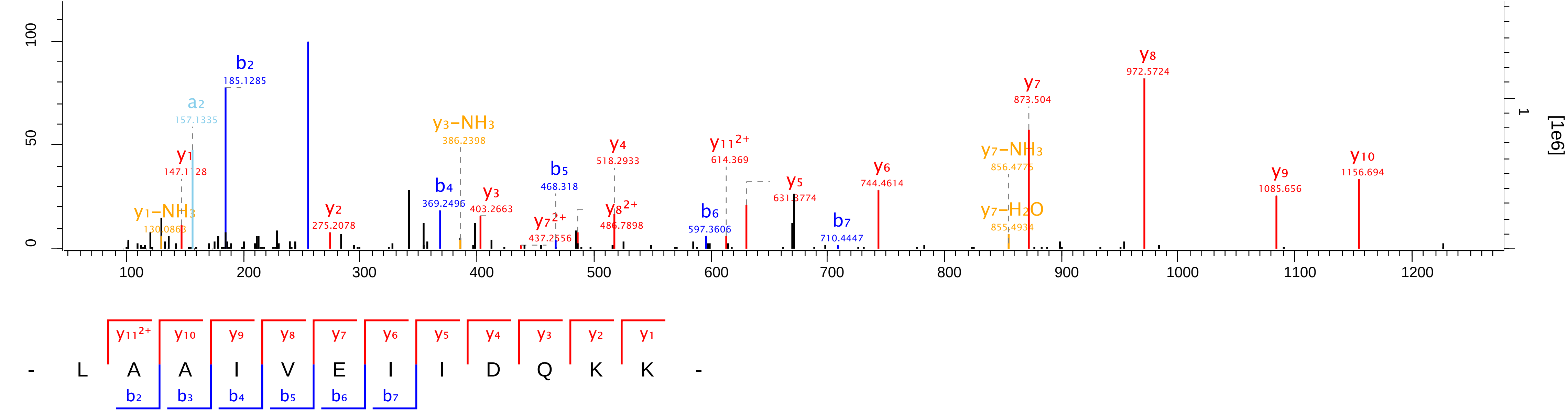

Raw file	Scan	Method	Score	m/z	Gene names
20140608_QEp4_FaHo_QC_YNG2_03	8835	FTMS; HCD	113.24	702.35	RPS0B;RPS0A

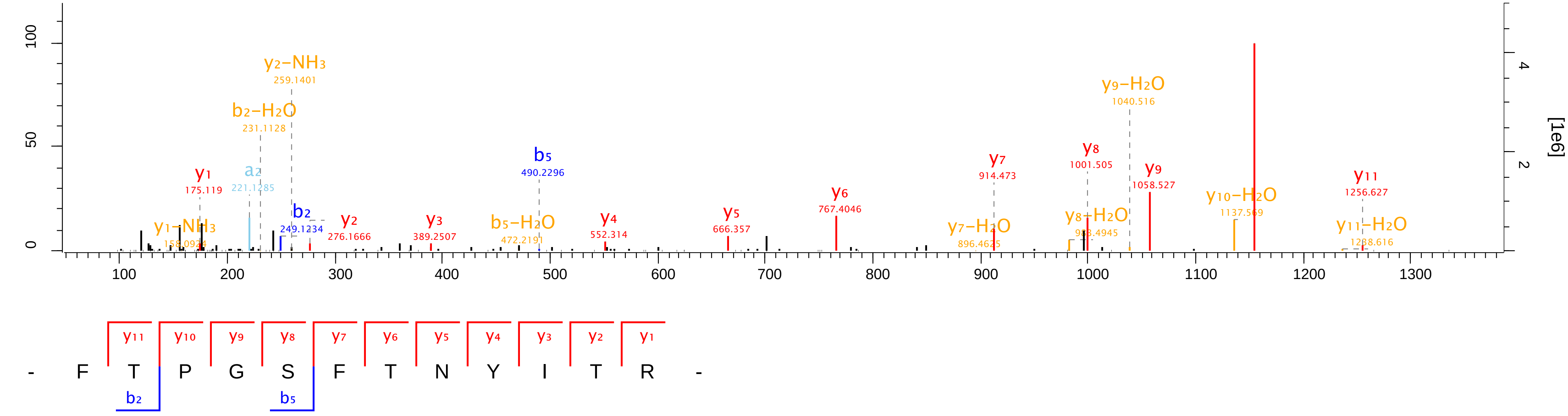

Raw file	Scan	Method	Score	m/z	Gene names
20140608_QEp4_FaHo_QC_YNG2_03	9509	FTMS; HCD	88.08	785.91	XRN1

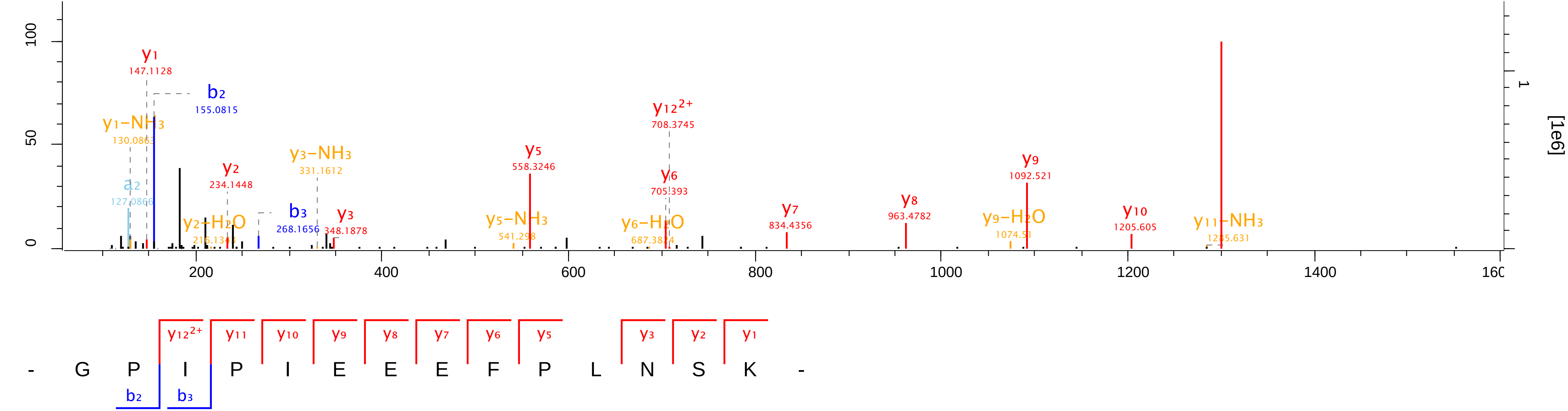

Raw file	Scan	Method	Score	m/z	Gene names
20140608_QEp4_FaHo_QC_YNG2_03	10572	FTMS; HCD	122.79	871.48	RPL4B;RPL4A

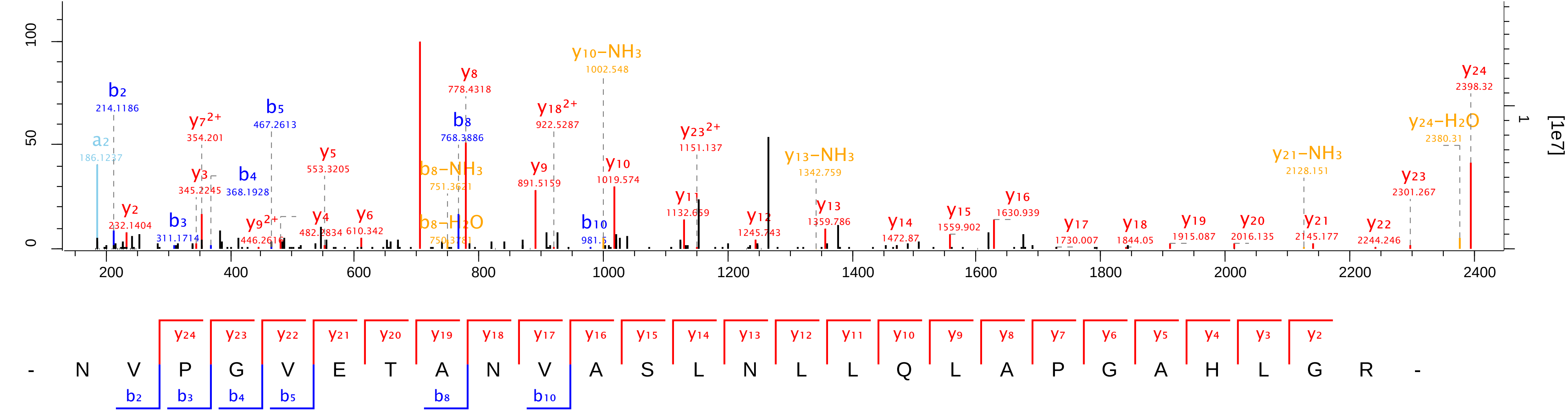

Supplement: Supplemental Data [file supp_O115.049460_mcp.O115.049460-6.pdf]
